# The trend analysis of HIV and other sexually transmitted infections among the elderly aged 50 to 69 years from 1990 to 2030

**DOI:** 10.7189/jogh.14.04105

**Published:** 2024-07-19

**Authors:** Xiaofeng Liang, Ying Deng, Hailin Xu, Zhishen Peng, Peixian Chen, Qiuyu Chen, Jun Xian, Qing Chen, Bin Yang

**Affiliations:** 1 Dermatology Hospital, Southern Medical University, Guangzhou, China; 2 Department of Epidemiology, School of Public Health, Southern Medical University, Guangzhou, China; 3 Guangdong Eye Institute, Department of Ophthalmology, Guangdong Provincial People's Hospital (Guangdong Academy of Medical Sciences), Southern Medical University, Guangzhou, China; The Second School of Clinical Medicine, Southern Medical University, Guangzhou, China; 4 Department of Urology, Sun Yat-sen Memorial Hospital, Sun Yat-sen University, Guangzhou, China

## Abstract

**Background:**

The HIV and other sexually transmitted infections (STI) excluding HIV among the elderly population urgently require more attention and in-depth study. We aimed to present and predict the worldwide of its burden from 1990 to 2030 using data from the Global Burden of Disease (GBD) study.

**Methods:**

Leveraging the 2019 GBD study, we investigated the average annual percentage change (AAPC) of HIV and other STI in incidence, prevalence, disability-adjusted life years (DALYs), and mortality rates for individuals aged 50–69 across different age groups, genders, sociodemographic index (SDI) regions, and nations. The incidence of STI in the population from 2020 to 2030 was explored by Bayesian age-period-cohort (BAPC) prediction model.

**Results:**

The HIV incidence rate experienced its fastest growth 1990–1992, peaked in 1996, and gradually declined thereafter, with the 2019 rate being lower than that of 1990. The prevalence rate didn't present a sharp turning point. After 2006, its growth rate accelerated. Both DALYs and mortality rates plateaued high between 2002 and 2005, followed by a decline. The decline was steepest from 2005–2012, yet the rate of decrease slowed noticeably from 2012–2019.When segmented by age, HIV was more prevalent among those aged 55–59 and 50–54, with the 50–54 age group witnessing the fastest decline in incidence rates. However, the fastest growth in prevalence rates was seen among the 60–64 and 65–69 age groups. The other STI incidence rate declined from 1990–1996, increased up to 2006, declined until 2015, and then saw a resurgence with accelerated growth thereafter. The prevalence rate showcased varied trends, with a notable increase in the past five years. The highest growth in incidence rate was among the 65–69 age group. We predict that the incidence rate of STI will increase in the future.

**Conclusions:**

Overall, despite the evident decline in incidence, mortality rates, and DALYs, the prevalence of HIV and other STI among the elderly is rising, and both demonstrated significant trend variations across different ages, genders, SDI regions, and nations. Comprehensive sexual health education, clinical care and adjustments in health service strategies based on the evolving trends of HIV and other STI among the elderly are paramount.

The global population is aging at a rapid pace. By 2050, the number of people aged 60 and above is expected to soar to two billion, up from 800 million in 2011, with around 80% residing in lower-income nations [1]. At the same time, the number of older individuals (those 50 and older [2,3]) living with HIV is also on the rise. From 2000 to 2016, this age group's proportion among HIV cases doubled from eight to 16%, approximately 5.7 million older adults with HIV, with a significant 80% of them living in less wealthy nations. It is projected that this will increase to 21% [4]. Due to the success and expansion of antiretroviral therapy (ART) programmes [5,6], the lifespan of older individuals with HIV is extending, making it increasingly crucial to understand their quality of life. A UK study indicated that between 2014 and 2019, 92.5% of newly diagnosed cases of sexually transmitted infections (STI) were in those under 45. Yet, while younger age groups saw a decrease in new STI diagnoses, there was a noticeable increase among those aged 45 to 64 [7]. While previous studies have examined the incidence and rate of change in STI among adolescents and young adults aged 10–24 in 204 nations from 1990 to 2019 [8], our focus shifts to those aged 50–69. Although this older demographic isn't the primary group affected by STI, numerous studies indicate a rising trend in their incidence and prevalence rates [9]. Furthermore, with improvements in medical care, younger infected individuals are aging, amplifying the global burden of STI in older populations. Therefore, using the Global Burden of Disease (GBD) database, we aim to investigate the average annual percent change (AAPC) in incidence, prevalence, disability-adjusted life years (DALYs), and mortality rates among older individuals aged 50–69 across different age groups, genders, SDI regions, and nations. This study intends to explore the development and trends of STI in older adults and to identify vulnerabilities in the global transmission of STI in order to provide data to improve the quality of life of older patients with STI, to transform prevention and care strategies for older patients, and to reduce the global burden of STI.

## METHODS

### Data source

For the GBD 2019, we retrieved data from the Global Health Data Exchange that spanned multiple time points (Global Burden of Disease 2019 Collaborative Network. Seattle, United States: Institute for Health Metrics and Evaluation, 2020. Available from https://vizhub.healthdata.org/gbd-results/). This data encompassed information from 1990 to 2019, covering 369 diseases and injuries across 204 regions, including HIV and other sexually transmitted infections excluding HIV (other STI, ‘chlamydial infection’, ‘genital herpes’, ‘gonococcal infection’, ‘syphilis’ and ‘trichomoniasis’) [10], based on the World Health Organization's 10th revision of the International Classification of Diseases. In 2019, the data from these diseases combined represented over 95% of the global DALYs burden due to STI. Sources of data for STI consisted of literature reviews, public health department disclosures, pre-pregnancy clinic reports, data from GBD partners, and case studies. All these data sets are accessible through the GBD 2019's tools on VizHub at healthdata.org [10,11].

### Study population and data collection

Initially, we selected our area of study on the website to be ‘HIV’ and ‘Sexually transmitted infections excluding HIV’. Subsequently, we downloaded the corresponding data segregated by age, gender, sociodemographic index (SDI) region, geographical area, and nation. Our target population was individuals aged 50–69. Based on the GBD's definitions and the requirements of our study, we categorised age groups as 50–54, 55–59, 60–64, and 65–69 years. Both genders were considered. The SDI is the geometric mean of the total fertility rate for individuals under 25, the average educational duration for those 15 and older, and the lag-distributed income per capita, scaled between 0 to 1. It acts as a comprehensive metric reflecting the socioeconomic conditions affecting health outcomes in each region [10]. An SDI value of 0 represents the lowest income, least education, and highest fertility rate. The SDI is divided into five quintiles: low, low-middle, middle, high-middle, and high [11]. Our study also aligned with the GBD's definition, considering 21 regions with similar geography and epidemiological characteristics, as well as 204 individual nations and territories. We extracted annual data from 1990 to 2019, encompassing the number and rates of incidence, prevalence, death and DALYs. DALYs is a measure used in public health to capture the total amount of healthy life lost to all causes. DALYs essentially combine years of life lost due to premature mortality and years lived with disability. In GBD 2019, estimates with uncertainty intervals (UI) were generated for all locations in every year, even in instances of sparse or missing data [10]. All reported rates were standardised, expressed per 100 000 individuals, ensuring comparability. Within the GBD framework, a specific algorithm was employed to estimate the uncertainty of a health statistic by iterating the estimation 1000 times. These 1000 estimates were arranged in ascending order. We selected the 25th and 975th values from these iterations to represent the 95% uncertainty interval (UI) [8].

### Statistical analysis

By fitting the data on a logarithmic scale, the software optimally connects multiple distinct segments using the points where a monotonic trend shows significant changes in its trajectory. These points are termed ‘joinpoints’ and each joinpoint was tested using the Monte Carlo permutation method. When choosing the model in joinpoint software, we combined the Weighted Bayesian Information Criterion approach with judgment of researchers. Joinpoint regression analysis is widely acknowledged as a vital tool for identifying shifts in trends, accurately determining points of trend changes, and assessing their significance [12,13]. This provides valuable insights into the dynamic changes in the epidemiology of diseases. As a result, we employed joinpoint regression analysis to calculate the Annual Average Percentage Change (AAPC). The AAPC provides an integrated measure of the trend over a particular period, representing a weighted average of the Annual Percentage Change (APC) via regression analysis. The numeric value of AAPC indicates the percentage change per year (increase, decrease, or stable). For instance, an AAPC of 0.1 suggests a 0.1% growth in the rate every year. The trend of incidence rates was delineated using the AAPC value and its 95% confidence interval (CI). Calculations for AAPC were done for the periods 1990–1999, 2000–2009, 2010–2019, and the entire span from 1990 to 2019. Bayesian age-period-cohort (BAPC) model (details available elsewhere [14]) was applied to predict the prevalence rate from 2020 to 2030 for female and male by R packages ‘BAPC’ and ‘INLA’. The BAPC model has been proven to have higher accuracy in predicting the disease burden, so we used it within an integrated nested Laplacian approximation to project the prevalence rate from 2020 to 2030 [15]. All statistical analyses were executed using R Studio software (version 4.3.1) and the Joinpoint Regression Program (version 5.0.2). In our study, both the listed ‘number’ and ‘rate’ were accompanied by UI values enclosed in parentheses. All rates are presented per 100 000 population. Alongside the AAPC, CI values are also provided within brackets. The raw data obtained from GBD download and some analysed data are presented in the Tables S1–10 in the **Online Supplementary Document**.

## RESULTS

### Global analysis

**Figure 1:**
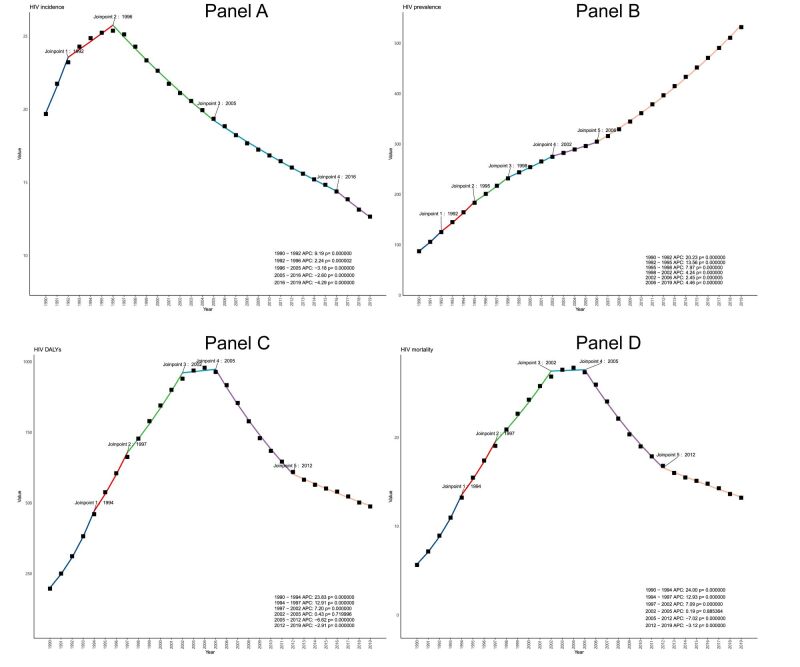
Joinpoint regression analysis of global HIV incidence (**Panel A**), prevalence (**Panel B**), DALYs (**Panel C**), and mortality (**Panel D**) in the elderly aged 50–65 years from 1990 to 2019. The joinpoints have been labeled in the figure, and the corresponding APC and *P-*values (less than 0.05 is considered statistically significant) are shown in the legend. Different colours represent different segments. All values are per 100 000 population. APC – annual percentage change, DALYs – disability-adjusted life-years

The HIV incidence rate among elder-aged individuals increased from 1990 to 1999 (AAPC 1.74, 0.09 to 3.42), but consistently declined from 2000 to 2019 (**Table 1**). Overall, the HIV incidence rate in 2019 (12.7 per 100 000 population (9.4 to 16.2); AAPC −2.2 (−2.6 to –1.9)) (Table S1 in the **Online Supplementary Document**) was lower than the incidence in 1990 (19.7 per 100 000 population (15.6 to 24.4)). As for prevalence rate, an overall upward trend was also observed from 1990 − 2019, and the growth rate was most accelerated during 1990 − 1999. The DALYs and mortality rates generally declined over 1990–2019. The joinpoint regression analysis identified a substantial change in incidence of HIV in 1992, 1996, 2005, 2018, and especially in 1992 and 1996 (**Figure 1**, panel A, Table S2 in the **Online Supplementary Document**). Prevalence rate exhibited a more linear progression, with an accelerated growth rate post-2006 (**Figure 1**, panel B). Both DALYs and mortality rates presented a high plateau phase, peaking around 2002 and 2005, and then declining (**Figure 1**, panels C–D). The decline from 2005–2012 was the most accelerated, but the pace slowed between 2012–2019, unfortunately not returning to the levels observed in 1990.

**Table 1:** Global average AAPC in incidence, prevalence, DALYs and mortality per 100 000 population of HIV and other STIs excluding HIV

HIV	1990–1999 AAPC (95% CI)	*P*-value	2000-2009 AAPC (95% CI)	*P*-value	2010–2019 AAPC (95% CI)	*P*-value	1990–2019 AAPC (95% CI)	*P*-value
DALYs	16.54 (13.81 to 19.33)	0.000	−1.74 (−3.86 to 0.42)	0.100	−3.48 (−3.91 to −3.04)	0.000	1.40 (−0.26 to 3.1)	0.096
Mortality	16.60 (13.83 to 19.43)	0.000	−2.05 (−4.2 to 0.16)	0.065	−3.71 (−4.16 to −3.25)	0.000	1.18 (−0.51 to 2.91)	0.166
Incidence	1.74 (0.09 to 3.42)	0.041	−2.94 (−3.02 to −2.86)	0.000	−3.07 (−3.4 to −2.75)	0.000	−2.21 (−2.56 to −1.86)	0.000
Prevalence	11.90 (9.91 to 13.93)	0.000	3.20 (2.94 to 3.46)	0.000	4.36 (4.26 to 4.46)	0.000	5.27 (4.69 to 5.85)	0.000
**Other STIs**								
DALYs	−1.06 (−1.42 to −0.7)	0.000	−1.78 (−1.91 to −1.65)	0.000	−0.26 (−0.44 to −0.07)	0.013	−1.32 (−1.44 to −1.19)	0.000
Mortality	−2.03 (−2.64 to −1.43)	0.000	−4.22 (−4.63 to −3.8)	0.000	−1.11 (−1.55 to −0.68)	0.000	−3.02 (−3.26 to −2.78)	0.000
Incidence	0.03 (−0.12 to 0.19)	0.636	0.20 (0 to 0.41)	0.054	0.07 (−0.06 to 0.2)	0.246	0.11 (0.05 to 0.17)	0.001
Prevalence	0.07 (−0.14 to 0.27)	0.461	−0.10 (−0.25 to 0.05)	0.151	0.28 (0.23 to 0.34)	0.000	−0.03 (−0.06 to 0.01)	0.136

AAPC – annual percentage change, CI – confidence interval, DALY – disability-adjusted life-years, STIs – sexually transmitted infections

**Figure 2:**
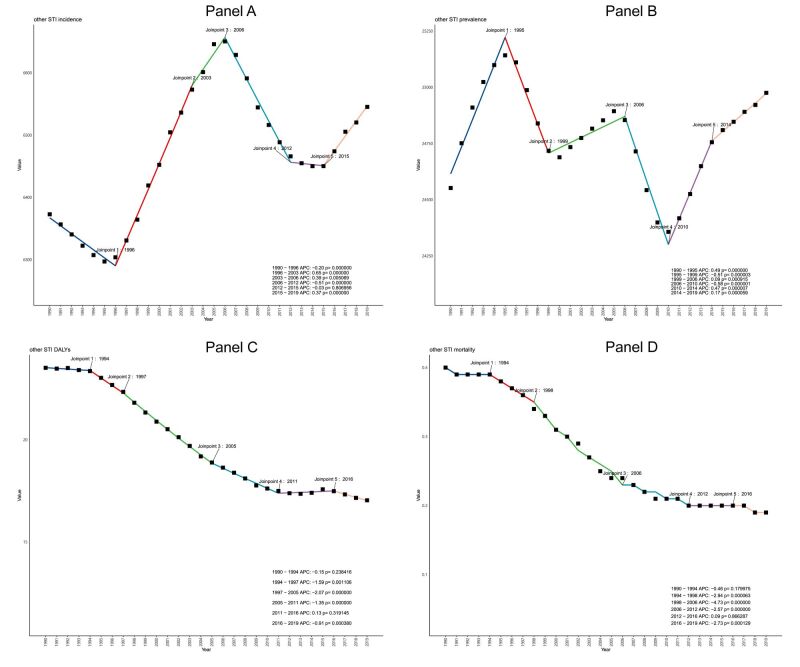
Joinpoint regression analysis of global other STI excluding HIV incidence (**Panel A**), prevalence (**Panel B**), DALYs (**Panel C**), and mortality (**Panel D**) in the elderly aged 50–65 years from 1990 to 2019. The joinpoints have been labeled in the figure, and the corresponding APC and *P-*values (less than 0.05 is considered statistically significant) are shown in the legend. Different colours represent different segments. All values are per 100 000 population. APC – annual percentage change, DALYs – disability-adjusted life years, STI – sexually transmitted infections

The incidence rate trend for other STI from 1990–2019 stands in contrast to that of HIV, predominantly showing an upward trajectory (AAPC 0.2 (0.05 to 0.17)) (**Table 1**, Table S1 in the **Online Supplementary Document**). Following a decline in prevalence rate from 2000–2009 (AAPC −0.1 (−0.25 to 0.05)), there was a marked increase from 2010–2019 (AAPC 0.28 (0.23 to 0.34)), growing from a rate of 24 552.16 (21 479.03 to 28 013.93) in 1990 to 24 975.6 per 100 000 population (21 715.39 to 28 652.43) in 2019. Both DALYs and mortality rates demonstrated a gradual decrement. Interestingly, a subsequent joinpoint regression analysis revealed a distinctive complexity in the other STI trends when compared to HIV (**Figure 2**, Table S2 in the **Online Supplementary Document**). The incidence rate showed five discernible joinpoints in 1996, 2003, 2006, 2012, and 2015 (**Figure 2**, panel A). The prevalence exhibited five joinpoints in 1996, 1999, 2006, 2010, and 2014 (**Figure 2**, panel B). Within the spectrum of other STI, only the incidence of gonococcal infections and the prevalence rate of syphilis showed a downturn. However, the incidence and prevalence rates for other STI generally increased.

In 1990 and 2019, trichomoniasis had the highest incidence rate (4603.91 per 100 000 population (3285.21 to 6329.55); 4747.31 (3380.49 to 6568.99)), while genital herpes exhibited the highest prevalence rate (22 254.35 per 100 000 population (19 105.28 to 25 931.15); 22 669.08 (19 285.70 to 26 536.32)) and DALYs (5.30 (1.62 to 13.05); 5.41 (1.65 to 13.44)) (Table S1 in the **Online Supplementary Document**). The mortality rate associated with other STI showed a considerable decline, with 2019 rate settling at relatively low values (**Figure 2**, panels C–D).

### Analysis by age group

Subsequently, we stratified the global elderly population by age. Across all four age groups, there was a pronounced decline in the HIV incidence rate (**Table 2**). The most substantial drop was among those aged 50–54, with an AAPC of −3.07 (−3.49 to −2.65), dropping from 27.8 per 100 000 population in 1990 (22.36 to 33.99) to 13.81 in 2019 (10.65 to 17.44). The slowest decline was in the 60–64 years cohort, with an AAPC of −1.24 (−1.56 to −0.92), reducing from 14.4 in 1990 (11.23 to 17.95) to 12.17 in 2019 (8.77 to 15.82). The other STI excluding HIV incidence rate growth was swiftest among those aged 65–69 years (AAPC 0.05 (0.04 to 0.06)), surging from 2776.11 in 1990 (2081.55 to 3679.01) to 2817.78 per 100 000 population in 2019 (2100.41 to 3735.47) (**Table 2**). In terms of HIV prevalence rate, there was a general uptrend (**Table 3**). The most rapid rise was observed in the 60–64 years age group, reaching an AAPC of 5.59 (5.07 to 6.12), followed closely by the 65–69 years group with an AAPC of 5.44 (4.96 to 5.94). A slight decline was observed in other STI prevalence rate across all age brackets (**Table 3**). Compared to 1990, DALYs and mortality rates across all four age groups showed a significant rise in 2019, almost doubling. Moreover, DALYs and mortality rates for other STI showed a marked reduction in all four age categories (**Table 4**, **Table 5**).

**Table 2:** Global rate and average annual percentage change (AAPC) in incidence per 100 000 population of HIV and other sexually transmitted infections (STI) excluding HIV from 1990 to 2019 globally and in different age groups, genders, sociodemographic index regions (SDI), and geographic regions

Incidence	1990	1990	2019	2019	1990–2019			1990	1990	2019	2019	1990–2019	
**HIV**	**Case (n)**	**Rate (per 100 000 population)**	**Case (n)**	**Rate (per 100 000 population)**	**AAPC (95%)**	***P*-value**	**Other STI**	**Case (n)**	**Rate (per 100 000 population)**	**Case (n)**	**Rate (per 100 000 population)**	**AAPC (95%)**	***P*-value**
**Total**	134 325 (106 555 to 166 171)	19.69 (15.62 to 24.36)	174 511 (129 425 to 223 161)	12.66 (9.39 to 16.18)	−2.21 (−2.56 to −1.86)	0.000		43 471 737 (34 051 856 to 55 727 162)	6372.86 (4991.93 to 8169.48)	90 259 068 (70 339 501 to 115 812 587)	6545.50 (5100.95 to 8398.62)	0.11 (0.05 to 0.17)	0.001
**Age**													
50–54 y	59 098 (47 544 to 72 261)	27.80 (22.36 to 33.99)	60 316 (46 528 to 76 172)	13.81 (10.65 to 17.44)	−3.07 (−3.49 to −2.65)	0.000		22 910 492 (16 054 000 to 31 012 060)	10 776.79 (7551.59 to 14 587.66)	48 523 063 (33 769 379 to 65 893 000)	11 108.33 (7730.79 to 15 084.81)	0.02 (−0.01 to 0.05)	0.213
55–59 y	36 675 (29 396 to 44 950)	19.78 (15.85 to 24.24)	51 870 (36 047 to 69 634)	13.98 (9.72 to 18.77)	−1.90 (−2.25 to −1.55)	0.000		11 461 479 (8 322 556 to 15 778 522)	6181.51 (4488.6 to 8509.82)	23 268 957 (16 920 111 to 31 989 096)	6271.74 (4560.52 to 8622.11)	0.03 (0.02 to 0.04)	0.000
60–64 y	23 140 (18 045 to 28 839)	14.4 (11.23 to 17.95)	38 023 (27 401 to 49 432)	12.17 (8.77 to 15.82)	−1.24 (−1.56 to −0.92)	0.000		5 671 584 (4 354 848 to 7 378 345)	3530.55 (2710.88 to 4593.01)	11 180 723 (8 535 806 to 14 611 766)	3577.43 (2731.15 to 4675.24)	0.04 (0.03 to 0.05)	0.000
65–69 y	15 412 (11 613 to 19 750)	12.48 (9.4 to 15.99)	24 302 (18 935 to 30 719)	9.40 (7.32 to 11.88)	−1.93 (−2.27 to −1.59)	0.000		3 428 181 (2 570 480 to 4 543 160)	2776.11 (2081.55 to 3679.01)	7 286 326 (5 431 306 to 9 659 303)	2817.78 (2100.41 to 3735.47)	0.05 (0.04 to 0.06)	0.000
**Sex**													
Female	67 934 (52 905 to 85 319)	19.66 (15.31 to 24.69)	84 218 (63 361 to 105 993)	11.98 (9.02 to 15.08)	−2.38 (−2.76 to −2)	0.000		17 652 675 (13 792 380 to 22 299 866)	5107.8 (3990.83 to 6452.47)	37 481 625 (29 060 551 to 47 589 174)	5332.97 (4134.8 to 6771.09)	0.15 (0.09 to 0.21)	0.000
Male	66 390 (51 351 to 82 840)	19.73 (15.26 to 24.62)	90 293 (64 452 to 119 489)	13.35 (9.53 to 17.67)	−2.04 (−2.36 to −1.71)	0.000		25 819 062 (19 934 089 to 33 444 418)	7672 (5923.31 to 9937.84)	52 777 444 (40 758 771 to 68 095 914)	7805.93 (6028.34 to 10 071.58)	0.1 (0.04 to 0.15)	0.002
**Sociodemographic index (SDI)**													
High SDI	5513 (4063 to 6780)	3.59 (2.65 to 4.42)	14203 (6691 to 24046)	5.64 (2.66 to 9.55)	1.71 (1.03 to 2.38)	0.000		7 057 771 (5 520 014 to 9 082 946)	4600.70 (3598.29 to 5920.83)	12 077 647 (9 406 341 to 15 553 370)	4795.24 (3734.64 to 6175.22)	0.14 (0.07 to 0.21)	0.000
High-middle SDI	3381 (2796 to 4154)	1.82 (1.5 to 2.23)	17 325 (8849 to 25 999)	5.13 (2.62 to 7.7)	3.95 (3.43 to 4.47)	0.000		11 123 765 (8 723 408 to 14 078 318)	5975.84 (4686.34 to 7563.07)	20 679 350 (16 154 690 to 26 286 126)	6123.69 (4783.82 to 7784)	0.2 (0.08 to 0.32)	0.003
Low SDI	68 558 (49 210 to 91 100)	156.06 (112.02 to 207.37)	32 694 (23 360 to 44 570)	35.19 (25.14 to 47.97)	−5.36 (−5.56 to −5.16)	0.000		3 971 195 (3 048 994 to 5 190 618)	9039.67 (6940.45 to 11 815.45)	8 434 870 (6 438 287 to 11 056 837)	9078.78 (6929.78 to 11 900.9)	0.02 (0 to 0.03)	0.087
Low-middle SDI	40 011 (29 280 to 53 476)	36.19 (26.49 to 48.37)	38 307 (26 399 to 52 572)	16.04 (11.06 to 22.02)	−3.87 (−4.24 to −3.5)	0.000		7 150 272 (5 608 426 to 9 142 827)	6467.82 (5073.13 to 8270.19)	15 543 653 (12 135 543 to 19 969 250)	6509.28 (5082.05 to 8362.61)	0.04 (0.01 to 0.08)	0.020
Middle SDI	16 702 (14 731 to 19 095)	8.89 (7.84 to 10.17)	71 830 (54 053 to 94 126)	15.72 (11.83 to 20.6)	0.47 (−0.87 to 1.83)	0.477		14 143 855 (10 978 661 to 17 997 423)	7532.53 (5846.86 to 9584.81)	33 468 290 (26 006 681 to 42 824 044)	7323.85 (5691.03 to 9371.16)	−0.10 (−0.17 to −0.03)	0.008
**Region**													
Andean Latin America	136 (76 to 465)	3.89 (2.18 to 13.31)	1106 (440 to 2021)	11.97 (4.77 to 21.88)	4.48 (3.6 to 5.38)	0.000		24 7951 (192 109 to 321 133)	7106.30 (5505.88 to 9203.72)	637 353 (491 481 to 829 853)	6901.23 (5321.73 to 8985.61)	−0.07 (−0.09 to −0.06)	0.000
Australasia	91 (59 to 124)	2.63 (1.72 to 3.61)	170 (115 to 247)	2.52 (1.71 to 3.66)	−1.68 (−2.65 to −0.71)	0.002		144 939 (113 314 to 185 652)	4211.27 (3292.41 to 5394.22)	281 888 (221 732 to 360 881)	4182.67 (3290.07 to 5354.77)	−0.06 (−0.13 to 0.02)	0.117
Caribbean	2971 (1775 to 4833)	71.94 (42.97 to 117.02)	2213 (1393 to 3302)	26.07 (16.4 to 38.9)	−2.84 (−3.41 to −2.26)	0.000		344 508 (270 442 to 437 584)	8341.01 (6547.77 to 10 594.51)	717 577 (559 318 to 917 551)	8452.65 (6588.46 to 10 808.23)	0.05 (0.03 to 0.07)	0.000
Central Asia	45 (33 to 66)	0.52 (0.37 to 0.76)	779 (252 to 1456)	5.19 (1.68 to 9.71)	10.68 (9.19 to 12.19)	0.000		656 703 (515 789 to 826 505)	7529.91 (5914.16 to 9476.9)	1 107 739 (868 360 to 1 391 590)	7384.44 (5788.68 to 9276.66)	0.52 (0.28 to 0.76)	0.000
Central Europe	92 (77 to 124)	0.37 (0.3 to 0.49)	307 (137 to 511)	1.03 (0.46 to 1.71)	2.4 (1.48 to 3.34)	0.000		1 397 445 (1 115 683 to 1 760 337)	5552.49 (4432.96 to 6994.37)	1 589 955 (1 269 936 to 1 995 215)	5318.95 (4248.38 to 6674.69)	−0.03 (−0.19 to 0.14)	0.739
Central Latin America	1549 (1332 to 1929)	10.74 (9.24 to 13.39)	5866 (2950 to 8560)	14.7 (7.39 to 21.46)	2.67 (2.13 to 3.21)	0.000		1 356 763 (1 044 069 to 1 760 291)	9413.81 (7244.21 to 12 213.66)	3 712 294 (2 848 875 to 4 814 670)	9304.58 (7140.49 to 12067.6)	0.01 (−0.02 to 0.04)	0.499
Central sub-Saharan Africa	9369 (6287 to 13164)	211.07 (141.65 to 296.56)	5632 (3597 to 8312)	54.29 (34.67 to 80.12)	−4.41 (−4.73 to −4.09)	0.000		374 833 (290 254 to 485 301)	8444.60 (6539.13 to 10 933.32)	907 426 (696 157 to 1 180 937)	8746.92 (6710.45 to 11 383.37)	0.26 (0.2 to 0.31)	0.000
East Asia	1308 (835 to 2456)	0.82 (0.52 to 1.53)	7883 (3258 to 11 395)	2.07 (0.85 to 2.99)	2.99 (0.4 to 5.65)	0.025		11 638 549 (9 036 614 to 14 785 790)	7265.66 (5641.33 to 9230.4)	27 271 361 (21 014 199 to 34 675 907)	7154.98 (5513.33 to 9097.65)	−0.16 (−0.3 to −0.02)	0.026
Eastern Europe	729 (425 to 970)	1.47 (0.86 to 1.96)	10 090 (3398 to 16 889)	18.55 (6.25 to 31.05)	11.95 (9.92 to 14.02)	0.000		2 824 037 (2 216 078 to 3 551 868)	5694.93 (4468.92 to 7162.66)	2 784 212 (2 207 069 to 3 483 186)	5119.19 (4058.03 to 6404.36)	0.16 (−0.08 to 0.4)	0.186
Eastern sub-Saharan Africa	67 175 (49 830 to 85 795)	491.56 (364.64 to 627.82)	29 030 (21 186 to 39 859)	98.28 (71.73 to 134.95)	−5.75 (−5.98 to −5.52)	0.000		1 932 993 (1 453 983 to 2 562 993)	14 144.96 (10 639.73 to 18 755.07)	4 244 222 (3 170 906 to 5 665 599)	14 369.23 (10 735.41 to 19 181.44)	0.04 (0.02 to 0.07)	0.003
High-income Asia Pacific	332 (195 to 555)	0.97 (0.57 to 1.63)	2385 (1058 to 3932)	4.81 (2.13 to 7.93)	5.25 (4.28 to 6.24)	0.000		1 665 378 (1 290 867 to 2 145 760)	4881.83 (3784 to 6290)	2 336 729 (1 837 965 to 3 001 415)	4715.29 (3708.84 to 6056.57)	−0.24 (−0.29 to −0.18)	0.000
High-income North America	3587 (2217 to 4684)	7.55 (4.67 to 9.86)	8948 (1601 to 17 892)	9.82 (1.76 to 19.64)	1 (0.28 to 1.72)	0.008		2 470 302 (1 887 817 to 3 207 918)	5198.51 (3972.73 to 6750.75)	4 789 110 (3 663 862 to 6 226 646)	5258.2 (4022.74 to 6836.55)	−0.01 (−0.12 to 0.1)	0.918
North Africa and Middle East	298 (103 to 698)	0.95 (0.33 to 2.22)	2583 (1075 to 6719)	3.24 (1.35 to 8.43)	3.47 (3.03 to 3.92)	0.000		2 233 856 (1 731 073 to 2 855 735)	7111.28 (5510.72 to 9090.98)	5 630 559 (4 349 130 to 7 222 301)	7065.33 (5457.37 to 9062.68)	0.07 (−0.01 to 0.14)	0.086
Oceania	13 (3 to 45)	2.23 (0.45 to 7.86)	605 (20 to 2072)	43.68 (1.47 to 149.6)	5.83 (2.47 to 9.3)	0.001		55 188 (42 762 to 70 842)	9729.19 (75 38.65 to 12 488.88)	138 748 (106 522 to 182 711)	10 018.2 (7691.31 to 13 192.5)	0.14 (0.1 to 0.17)	0.000
South Asia	1638 (1336 to 2185)	1.53 (1.24 to 2.03)	8466 (3201 to 16866)	3.45 (1.31 to 6.88)	2.42 (1.69 to 3.16)	0.000		5 566 693 (4 354 481 to 7 117 245)	5181.63 (4053.27 to 6624.93)	12 041 790 (9 378 082 to 15 382 000)	4911.6 (3825.13 to 6274)	−0.14 (−0.19 to −0.09)	0.000
Southeast Asia	7130 (5260 to 9266)	15.09 (11.14 to 19.61)	14 303 (8787 to 23 855)	12.42 (7.63 to 20.71)	-0.25 (-1.05 to 0.55)	0.520		3 691 980 (2 885 764 to 4 684 323)	7815.63 (6108.93 to 9916.34)	8 862 775 (6 900 238 to 11 269 199)	7695.57 (5991.49 to 9785.07)	0.09 (0.02 to 0.16)	0.013
Southern Latin America	338 (285 to 445)	4.52 (3.81 to 5.95)	758 (617 to 1030)	6.02 (4.9 to 8.18)	0.66 (0.13 to 1.19)	0.017		342 912 (266 595 to 439 873)	4579.7 (3560.46 to 5874.64)	573 429 (445 002 to 732 965)	4558.68 (3537.7 to 5826.96)	0.02 (−0.02 to 0.06)	0.275
Southern sub-Saharan Africa	15495 (7061 to 26856)	329.77 (150.27 to 571.55)	39 031 (30 250 to 50 433)	390.13 (302.36 to 504.1)	−1.55 (−2.86 to −0.22)	0.024		571 408 (440 971 to 726 528)	12 160.56 (9384.63 to 15 461.79)	1 198 383 (924 314 to 1 515 613)	11 978.23 (9238.81 to 15 149.04)	−0.03 (−0.07 to 0.01)	0.132
Tropical Latin America	1107 (1022 to 1190)	6.9 (6.37 to 7.41)	12 859 (5106 to 21 477)	31.14 (12.36 to 52.01)	5.61 (4.56 to 6.68)	0.000		1 404 150 (1 078 717 to 1 820 175)	8746.39 (6719.28 to 11 337.78)	3 571 586 (2 731 470 to 4 614 529)	8648.5 (6614.18 to 11 173.95)	−0.05 (−0.09 to 0)	0.035
Western Europe	1932 (1673 to 2384)	2.33 (2.02 to 2.88)	3171 (2294 to 4371)	2.8 (2.03 to 3.86)	0.57 (−0.07 to 1.21)	0.077		288 4091 (2 259 086 to 3 667 129)	3484.98 (2729.76 to 4431.17)	4030619 (3152761 to 5171201)	3561.91 (2786.14 to 4569.86)	0.10 (0.08 to 0.12)	0.000
Western sub-Saharan Africa	18 989 (12 590 to 27 391)	121.6 (80.63 to 175.41)	18 327 (14 694 to 23 330)	52.14 (41.8 to 66.37)	−4.29 (−4.8 to 3.78)	0.000		1 667 060 (1 285 969 to 2 163 405)	10 675.73 (8235.25 to 13 854.29)	3 831 313 (2 907 477 to 5 066 314)	10 899.29 (8271.17 to 14 412.61)	0.07 (0.04 to 0.1)	0.000

**Table 3:** Global rate and average annual percentage change in prevalence (AAPC) per 100 000 population of HIV and other sexually transmitted infections (STI) excluding HIV from 1990 to 2019 globally and in different age groups, genders, sociodemographic index (SDI) regions and geographic regions

Prevalence	1990	1990	2019	2019	1990–2019			1990	1990	2019	2019	1990–2019	
**HIV**	**Case (n)**	**Rate (per 100 000 population)**	**Case (n)**	**Rate (per 100 000 population)**	**AAPC (95%)**	***P-*value**	**Other STI**	**Case (n)**	**Rate (per 100 000 population)**	**Case (n)**	**Rate (per 100 000 population)**	**AAPC (95%)**	***P-*value**
**Total**	596 320 (516 556 to 678 971)	87.42 (75.73 to 99.54)	7 337 133 (6 701 654 to 8 025 529)	532.08 (486 to 582)	5.27 (4.69 to 5.85)	0.000		167 479 676 (146 516 684 to 191 093 692)	24 552.16 (21 479.03 to 28 013.93)	344 400 592 (299 444 023 to 395 102 144)	24 975.60 (21 715.39 to 28 652.43)	−0.03 (−0.06 to 0.01)	0.136
**Age**													
50–54 y	264 593 (230 031 to 300 919)	124.46 (108.2 to 141.55)	2 975 863 (2 767 929 to 3 194 632)	681.26 (633.66 to 731.34)	4.88 (4.24 to5.52)	0.000		56 524 257 (49 179 706 to 64 497 027)	26 588.25 (23 133.47 to 30 338.53)	118 355 962 (102 311 804 to 136 319 331)	27 095.10 (23 422.13 to 31 207.44)	−0.03 (−0.06 to 0.01)	0.156
55–59 y	165 540 (144 572 to 188 114)	89.28 (77.97 to 101.46)	212 0672 (1 923 356 to 2 326 588)	571.59 (518.41 to 627.09)	5.40 (4.82 to5.99)	0.000		45 972 919 (39 913 508 to 52 456 085)	24 794.54 (21 526.52 to 28 291.1)	94 700 878 (82 068 432 to 108 830 579)	25 524.98 (22 120.13 to 29 333.4)	−0.01 (−0.06 to 0.05)	0.796
60–64 y	103 682 (90 219 to 118 114)	64.54 (56.16 to 73.53)	1 389 249 (1 230 519 to 1 566 733)	444.51 (393.72 to 501.3)	5.59 (5.07 to6.12)	0.000		37 423 163 (32 598 452 to 43 001 995)	23 295.85 (20 292.48 to 26 768.66)	74 183 677 (64 311 224 to 85 476 886)	23 736.09 (20 577.26 to 27 349.51)	−0.05 (−0.09 to 0)	0.051
65–69 y	62 505 (53 227 to 71 697)	50.62 (43.1 to 58.06)	851 349 (738 776 to 969 807)	329.24 (285.7 to 375.05)	5.44 (4.96 to5.94)	0.000		27 559 336 (24 007 416 to 31 763 304)	22 317.32 (19 441 to 25 721.66)	57 160 075 (49 494 762 to 66 191 858)	22 105.06 (19 140.72 to 25 597.85)	−0.11 (−0.15 to −0.08)	0.000
**Sex**													
Female	274 609 (232 676 to 318 549)	79.46 (67.32 to 92.17)	3 553 751 (3 297 577 to 3 826 787)	505.64 (469.19 to 544.48)	5.12 (4.47 to 5.77)	0.000		104 455 108 (92 093 394 to 117 980 767)	30 224.09 (26 647.23 to 34 137.74)	216 215 770 (188 865 397 to 245 896 622)	30 763.65 (26 872.18 to 34 986.7)	−0.02 (−0.05 to 0.01)	0.148
Male	321 712 (271 933 to 378 679)	95.60 (80.8 to 112.52)	3 783 382 (3 340 441 to 4 266 249)	559.57 (494.06 to 630.99)	5.41 (4.89 to 5.94)	0.000		63 024 567 (54 346 897 to 73 216 436)	18 727.43 (16 148.91 to 21 755.89)	128 184 823 (110 152 966 to 148 832 503)	18 958.9 (16 291.94 to 22 012.75)	−0.05 (−0.11 to 0)	0.055
**Sociodemographic index (SDI)**													
High SDI	83 853 (57 067 to 118 359)	54.66 (37.2 to 77.15)	1 071 599 (602 259 to 1 566 747)	425.46 (239.12 to 622.05)	7.94 (7.45 to 8.44)	0.000		35 758 810 (31 260 604 to 40 791 647)	23 309.84 (20 377.62 to 26 590.56)	56 004 529 (48 413 053 to 64 914 771)	22235.7 (19 221.63 to 25 773.37)	−0.11 (−0.16 to -0.06)	0.000
High-middle SDI	26 864 (22 490 to 32 225)	14.43 (12.08 to 17.31)	460 344 (395 756 to 530 319)	136.32 (117.19 to 157.04)	7.86 (7.74 to 7.99)	0.000		42 264 996 (36 812 977 to 48 478 638)	22 705.35 (19 776.45 to 26 043.4)	78 040 825 (67 614 660 to 89 633 251)	23 109.89 (20 022.43 to 26 542.7)	−0.04 (−0.1 to 0.01)	0.121
Low SDI	321 465 (259 748 to 385 499)	731.75 (591.27 to 877.51)	1 647 063 (1 488 495 to 1 809 555)	1772.80 (1602.13 to 1947.69)	1.64 (1.13 to 2.15)	0.000		14 686 673 (13 047 569 to 16 502 699)	33 431.4 (29 700.3 to 37 565.24)	30 911 298 (27 351 191 to 34 927 572)	33 271.03 (29 439.15 to 37 593.9)	−0.16 (−0.22 to −0.1)	0.000
Low-middle SDI	117 225 (92 387 to 152 469)	106.04 (83.57 to 137.92)	1 680 710 (1 555 993 to 1 820 109)	703.84 (651.61 to 762.21)	4.66 (3.77 to 5.56)	0.000		26 033 975 (22 663 069 to 29 805 609)	23 549.17 (20 500 to 26 960.83)	59 587 451 (51 907 374 to 68 151 121)	24 953.68 (21 737.47 to 28 539.93)	0.14 (0.12 to 0.16)	0.000
Middle SDI	46 338 (39 629 to 54 188)	24.68 (21.1 to 28.86)	2 472 400 (2 300 765 to 2 666 665)	541.03 (503.48 to 583.54)	9.46 (7.98 to 10.95)	0.000		48 625 300 (42 578 486 to 55 415 479)	25 896.16 (22 675.84 to 29 512.38)	119 622 356 (104 057 681 to 136 811 564)	26 176.90 (22 770.89 to 29 938.4)	−0.11 (−0.17 to −0.05)	0.002
**Region**													
Andean Latin America	1319 (802 to 3845)	37.8 (22.99 to 110.21)	19 191 (12 424 to 27 343)	207.79 (134.53 to 296.06)	6.75 (6.46 to 7.03)	0.000		1 450 486 (1 299 154 to 1 611 386)	41571.12 (37 233.93 to 46 182.54)	3 935 212 (3 456 948 to 4 448 325)	42 610.29 (37 431.67 to 48 166.25)	0.00 (−0.06 to 0.06)	0.992
Australasia	1007 (776 to 1218)	29.25 (22.56 to 35.4)	8582 (6153 to 10 853)	127.33 (91.3 to 161.04)	5.69 (5.36 to 6.02)	0.000		772 096 (652 211 to 902 162)	22 433.67 (18 950.33 to 26 212.79)	1 303 704 (1 120 127 to 1 515 053)	19 344.4 (16 620.48 to 22 480.4)	−0.27 (−0.65 to 0.11)	0.153
Caribbean	9491 (5609 to 15 441)	229.78 (135.8 to 373.86)	67 161 (58 727 to 76 499)	791.11 (691.77 to 901.11)	2.70 (2.18 to 3.23)	0.000		1 697 817 (1 497 381 to 1 931 481)	41 106.45 (36 253.63 to 46 763.78)	3 482 267 (3 055 421 to 3 966 158)	41 019.16 (35 991.16 to 46 719.14)	−0.01 (−0.01 to 0)	0.036
Central Asia	317 (246 to 412)	3.64 (2.82 to 4.72)	9446 (6006 to 13 509)	62.97 (40.04 to 90.06)	9.91 (9.21 to 10.61)	0.000		1 857 359 (1 600 509 to 2 127 125)	21 296.92 (18 351.82 to 24 390.12)	3 150 825 (2 733 132 to 3 630 378)	21 004.12 (18 219.68 to 24 200.93)	0.08 (0.03 to 0.13)	0.002
Central Europe	1085 (874 to 1320)	4.31 (3.47 to 5.24)	10 061 (8022 to 12 015)	33.66 (26.84 to 40.19)	8.01 (7.63 to 8.4)	0.000		3 830 891 (3 313 942 to 4 414 378)	15 221.34 (13 167.34 to 17 539.72)	4 454 948 (3 831 870 to 5 173 616)	14 903.35 (12 818.94 to 17 307.54)	−0.04 (−0.07 to −0.01)	0.007
Central Latin America	6043 (4582 to 7868)	41.93 (31.79 to 54.59)	102 493 (76 022 to 126 635)	256.89 (190.54 to 317.4)	6.02 (5.82 to 6.22)	0.000		5 785 786 (5 180 436 to 6 467 213)	40 144.28 (35 944.11 to 44 872.32)	15 838 473 (13 955 090 to 17 923 104)	39 697.92 (34 977.36 to 44 922.89)	0.09 (−0.01 to 0.19)	0.085
Central sub-Saharan Africa	39 477 (27 111 to 56 170)	889.37 (610.77 to 1265.46)	164 369 (134 145 to 200 712)	1584.4 (1293.06 to 1934.72)	0.63 (0.19 to 1.08)	0.006		2 388 441 (2 149 977 to 2 628 442)	53 809.11 (48 436.76 to 59 216.07)	5 502 306 (4 922 910 to 6 071 400)	53 038.21 (47 453.26 to 58 523.86)	−0.06 (−0.08 to −0.04)	0.000
East Asia	10 034 (7597 to 12 948)	6.26 (4.74 to 8.08)	178 363 (93 634 to 311 366)	46.80 (24.57 to 81.69)	9.08 (7.91 to 10.27)	0.000		34 811 258 (29 956 728 to 40 214 662)	21731.8 (18 701.24 to 25 105.01)	83 252 408 (71 389 069 to 96 202 276)	21842.29 (18 729.8 to 25 239.85)	−0.38 (−0.56 to −0.19)	0.000
Eastern Europe	7066 (5273 to 9008)	14.25 (10.63 to 18.16)	122 540 (74 270 to 172 942)	225.31 (136.56 to 317.98)	9.46 (8.49 to 10.43)	0.000		12 500 773 (10 802 161 to 14 426 203)	25 208.94 (21 783.53 to 29 091.74)	13 484 541 (11 661 520 to 15 552 595)	24 793.37 (21 441.47 to 28 595.79)	0.02 (−0.01 to 0.06)	0.188
Eastern sub-Saharan Africa	290 447 (238 781 to 344 436)	2125.39 (1747.32 to 2520.46)	1 733 954 (1 579 112 to 1 886 117)	5870.47 (5346.24 to 6385.64)	1.99 (1.44 to 2.54)	0.000		6 729 454 (5 999 014 to 7 483 954)	49 243.76 (43 898.66 to 54 764.93)	14 157 996 (12 514 622 to 15 883 721)	47 933.28 (42 369.48 to 53 775.89)	−0.42 (−0.54 to −0.3)	0.000
High-income Asia Pacific	1597 (869 to 2543)	4.68 (2.55 to 7.45)	35 128 (20 116 to 51 685)	70.88 (40.59 to 104.3)	10.24 (9.25 to 11.25)	0.000		6 687 246 (5 946 694 to 7 513 427)	19 602.75 (17 431.92 to 22 024.58)	9 317 871 (8 018 260 to 10 833 982)	18 802.57 (16 180.08 to 21 861.93)	−0.03 (−0.13 to 0.06)	0.444
High-income North America	61 660 (40 512 to 89 770)	129.76 (85.25 to 188.91)	849 670 (448 969 to 1 279 468)	932.9 (492.95 to 1404.79)	7.45 (6.95 to 7.96)	0.000		14 459 007 (12 568 173 to 16 626 717)	30 427.58 (26 448.5 to 34 989.31)	24 947 816 (21 627 768 to 28 780 312)	27 391.46 (23 746.21 to 31 599.35)	−0.42 (−0.51 to −0.33)	0.000
North Africa and Middle East	1664 (917 to 3413)	5.30 (2.92 to 10.87)	28 046 (16 453 to 51 842)	35.19 (20.65 to 65.05)	6.01 (5.47 to 6.56)	0.000		6 916 736 (6 023 417 to 7 897 879)	22 018.82 (19 175.02 to 25 142.21)	17 264 951 (14 894 694 to 19 904 335)	21 664.39 (18 690.14 to 24 976.34)	−0.03 (−0.06 to 0)	0.035
Oceania	50 (18 to 215)	8.85 (3.26 to 37.88)	13 877 (583 to 36 125)	1001.96 (42.07 to 2608.36)	15.87 (12.54 to 19.31)	0.000		210 787 (184 944 to 240 071)	37 160.22 (32 604.37 to 42 322.79)	523 991 (461 836 to 592 707)	37 834.30 (33 346.46 to 42 795.86)	−0.01 (−0.09 to 0.07)	0.827
South Asia	7354 (5952 to 10 600)	6.85 (5.54 to 9.87)	353 233 (282 285 to 440 806)	144.08 (115.14 to 179.8)	9.96 (8.54 to 11.4)	0.000		19 025 799 (16 232 458 to 22 196 867)	17 709.74 (15 109.62 to 20 661.46)	45 287 759 (38 846 229 to 52 677 111)	18 471.94 (15 844.58 to 21 485.91)	0.13 (0.1 to 0.16)	0.000
Southeast Asia	13 309 (10 157 to 17 076)	28.17 (21.5 to 36.15)	302 352 (256 787 to 371 556)	262.53 (222.97 to 322.62)	5.82 (4.83 to 6.82)	0.000		13 858 709 (12 201 104 to 15 704 687)	29 337.79 (25 828.77 to 33 245.58)	33 511 221 (29 120 264 to 38 543 133)	29 097.87 (25 285.19 to 33 467.09)	−0.07 (−0.09 to −0.05)	0.000
Southern Latin America	3489 (2788 to 4166)	46.6 (37.24 to 55.64)	49 520 (39 379 to 60 252)	393.67 (313.06 to 478.99)	8.06 (7.81 to 8.31)	0.000		2 383 990 (2 223 012 to 2 557 579)	31 838.91 (29 689 to 34 157.25)	3 933 325 (3 436 409 to 4 498 206)	31 269.36 (27 318.94 to 35 760.08)	−0.08 (−0.1 to −0.07)	0.000
Southern sub-Saharan Africa	37 928 (20 216 to 71 965)	807.17 (430.22 to 1531.54)	2 049 014 (1 909 667 to 2 188 533)	20 480.55 (19 087.74 to 21 875.09)	9.19 (7.58 to 10.83)	0.000		2 488 189 (2 227 471 to 2 763 563)	52 953.03 (47 404.49 to 58 813.47)	5 533 013 (4 976 935 to 6 118 033)	55 304.24 (49 746.06 to 61151.7)	0.26 (0.19 to 0.33)	0.000
Tropical Latin America	8267 (5947 to 11 833)	51.49 (37.04 to 73.7)	233 641 (161 966 to 311 629)	565.76 (392.2 to 754.6)	9.31 (8.63 to 9.99)	0.000		7 693 814 (6 794 922 to 8 661 904)	47 924.39 (42 325.24 to 53 954.58)	20 272 495 (17 935 298 to 22 959 158)	49 089.28 (43 429.82 to 55 594.96)	0.04 (−0.08 to 0.17)	0.503
Western Europe	24 629 (18 205 to 30 974)	29.76 (22 to 37.43)	286 869 (219 464 to 353 904)	253.51 (193.94 to 312.75)	8.05 (7.87 to 8.24)	0.000		15 720 484 (13 518 084 to 18 171 686)	18 995.8 (16 334.54 to 21 957.7)	20 486 444 (17 544 360 to 23 885 884)	18 104.14 (15 504.18 to 21 108.27)	−0.10 (−0.17 to −0.02)	0.016
Western sub-Saharan Africa	70 089 (50 628 to 103 520)	448.85 (324.22 to 662.94)	719 625 (623 716 to 828 050)	2047.18 (1774.34 to 2355.63)	3.66 (2.79 to 4.53)	0.000		6 210 554 (5 523 124 to 6 992 976)	39 771.93 (35 369.68 to 44 782.51)	14 759 025 (13 070 916 to 16 615 998)	41 986.35 (37 184.03 to 47 269.05)	0.14 (0.11 to 0.16)	0.000

**Table 4:** Global rate and average annual percentage change (AAPC) in disability adjusted life years (DALYs) per 100 000 population of HIV and other sexually transmitted infections (STI) excluding HIV from 1990 to 2019 globally and in different age groups, genders, sociodemographic Index regions (SDI) and geographic regions

DALYs	1990	1990	2019	2019	1990–2019			1990	1990	2019	2019	1990–2019	
**HIV**	**Case (n)**	**Rate (per 100 000 population)**	**Case (n)**	**Rate (per 100 000 population)**	**AAPC (95%)**	***P-*value**	**Other STI**	**Case (n)**	**Rate (per 100 000 population)**	**Case (n)**	**Rate (per 100 000 population)**	**AAPC (95%)**	***P-*value**
**Total**	1 347 544 (993 576 to 1 780 505)	197.55 (145.66 to 261.02)	6 728 043 (5 982 448 to 7 861 113)	487.91 (433.84 to 570.08)	1.4 (−0.26 to 3.1)	0.096		160 425 (114 389 to 243 796)	23.52 (16.77 to 35.74)	235 035 (149 020 to 403 403)	17.04 (10.81 to 29.25)	−1.32 (−1.44 to −1.19)	0.000
**Age**													
50–54 y	666 401 (483 775 to 882 897)	313.47 (227.56 to 415.3)	3 320 860 (2 951 969 to 3 874 281)	760.24 (675.79 to 886.93)	1.32 (−0.36 to3.04)	0.120		56 085 (38 766 to 86 940)	26.38 (18.24 to 40.9)	87726 (54456 to 152487)	20.08 (12.47 to 34.91)	−1.12 (−1.28 to −0.96)	0.000
55–59 y	367 861 (273 226 to 481 089)	198.4 (147.36 to 259.47)	1 863 158 (1 649 345 to 2 188 416)	502.18 (444.55 to 589.85)	1.37 (−0.26 to 3.04)	0.097		49 096 (34 834 to 72 307)	26.48 (18.79 to 39)	69 177 (43 773 to 114 815)	18.65 (11.8 to 30.95)	−1.48 (−1.65 to −1.31)	0.000
60–64 y	201 555 (150 700 to 263 162)	125.47 (93.81 to 163.82)	100 5501 (884 414 to 1 184 427)	321.72 (282.98 to 378.97)	1.31 (−0.3 to2.95)	0.107		32 550 (22 685 to 49 603)	20.26 (14.12 to 30.88)	44 052 (26 842 to 77 284)	14.10 (8.59 to 24.73)	−1.54 (−1.67 to −1.4)	0.000
65–69 y	111 728 (83 422 to 146 541)	90.48 (67.55 to 118.67)	538 523 (472 789 to 632 280)	208.26 (182.84 to 244.52)	1.12 (−0.34 to2.61)	0.127		22 694 (16 136 to 34 692)	18.38 (13.07 to 28.09)	34 079 (21 114 to 58 595)	13.18 (8.17 to 22.66)	−1.34 (−1.42 to −1.26)	0.000
**Sex**													
Female	635 337 (440 444 to 862 043)	183.83 (127.44 to 249.43)	3 052 071 (2 709 078 to 3 522 125)	434.26 (385.45 to 501.14)	0.98 (−0.89 to 2.89)	0.295		106 589 (74 241 to 166 585)	30.84 (21.48 to 48.2)	171 766 (107 459 to 297 871)	24.44 (15.29 to 42.38)	−0.92 (−1.01 to −0.83)	0.000
Male	712 207 (538 676 to 921 850)	211.63 (160.06 to 273.92)	3 675 972 (3 236 144 to 4 354 434)	543.69 (478.63 to 644.03)	1.81 (0.34 to 3.3)	0.017		53 836 (33 514 to 79 299)	16.00 (9.96 to 23.56)	63 269 (38 182 to 109 656)	9.36 (5.65 to 16.22)	−2.26 (−2.45 to −2.07)	0.000
**Sociodemographic index (SDI)**													
High SDI	175 832 (170 208 to 183 598)	114.62 (110.95 to 119.68)	232 366 (191 267 to 286 447)	92.26 (75.94 to 113.73)	−1.48 (−2.12 to −0.83)	0.000		19 725 (11 484 to 35 798)	12.86 (7.49 to 23.34)	26 854 (13 934 to 52 665)	10.66 (5.53 to 20.91)	−0.60 (−0.66 to −0.54)	0.000
High-middle SDI	69 347 (66 392 to 72 464)	37.25 (35.67 to 38.93)	347 627 (315 549 to 391 924)	102.94 (93.44 to 116.06)	2.94 (1.99 to 3.89)	0.000		31 527 (20 956 to 51 575)	16.94 (11.26 to 27.71)	42 663 (23 772 to 80 873)	12.63 (7.04 to 23.95)	−1.57 (−1.78 to −1.35)	0.000
Low SDI	822 747 (549 838 to 1 129 090)	1872.83 (1251.6 to 2570.16)	1 794 163 (1 546 087 to 2 142 089)	1931.13 (1664.11 to 2305.61)	−1.53 (−3.04 to 0.01)	0.052		29 572 (17 356 to 45 589)	67.31 (39.51 to 103.77)	33 805 (22 202 to 49 233)	36.39 (23.9 to 52.99)	−2.31 (−2.41 to −2.21)	0.000
Low-middle SDI	204 942 (123 805 to 317 029)	185.38 (111.99 to 286.77)	1 988 112 (1 694 161 to 2 377 598)	832.57 (709.47 to 995.68)	2.45 (0.25 to 4.7)	0.030		39 771 (29 352 to 53 231)	35.98 (26.55 to 48.15)	59 759 (41 855 to 91 350)	25.03 (17.53 to 38.26)	−1.40 (−1.58 to −1.23)	0.000
Middle SDI	73 402 (55 180 to 94 447)	39.09 (29.39 to 50.3)	2 359 584 (2 117 270 to 2 704 341)	516.35 (463.32 to 591.79)	6.88 (3.97 to 9.86)	0.000		39 743 (26 587 to 63 809)	21.17 (14.16 to 33.98)	71 805 (41 369 to 132 601)	15.71 (9.05 to 29.02)	−1.03 (−1.15 to −0.91)	0.000
**Region**													
Andean Latin America	3585 (2270 to 8853)	102.76 (65.06 to 253.73)	20 590 (14 417 to 37 530)	222.95 (156.11 to 406.37)	2.48 (1.71 to 3.25)	0.000		804 (461 to 1489)	23.04 (13.21 to 42.68)	1770 (910 to 3481)	19.17 (9.85 to 37.69)	−0.61 (−0.64 to −0.57)	0.000
Australasia	2671 (2520 to 2831)	77.62 (73.23 to 82.26)	1685 (1411 to 2038)	25 (20.94 to 30.24)	−5.02 (−5.82 to −4.22)	0.000		374 (204 to 699)	10.87 (5.93 to 20.3)	559 (260 to 1175)	8.3 (3.86 to 17.43)	−0.72 (−0.96 to −0.49)	0.000
Caribbean	20 490 (13 831 to 32 236)	496.1 (334.88 to 780.47)	84 699 (72 783 to 98 898)	997.71 (857.34 to 1164.97)	0.04 (−1.49 to 1.58)	0.961		1329 (810 to 2293)	32.18 (19.6 to 55.52)	2344 (1444 to 3955)	27.61 (17.01 to 46.59)	−0.33 (−0.44 to −0.21)	0.000
Central Asia	969 (898 to 1031)	11.12 (10.3 to 11.82)	5698 (5155 to 6346)	37.98 (34.36 to 42.3)	4.48 (3.73 to 5.24)	0.000		2153 (1557 to 3197)	24.69 (17.85 to 36.66)	2358 (1435 to 4081)	15.72 (9.57 to 27.2)	−1.89 (−2.09 to −1.69)	0.000
Central Europe	4438 (4150 to 4676)	17.64 (16.49 to 18.58)	4875 (4415 to 5761)	16.31 (14.77 to 19.27)	−0.61 (−0.96 to −0.27)	0.001		2769 (1646 to 4856)	11 (6.54 to 19.29)	2553 (1308 to 4900)	8.54 (4.38 to 16.39)	−0.67 (−0.74 to −0.59)	0.000
Central Latin America	20 302 (19 866 to 20 783)	140.87 (137.84 to 144.2)	98 648 (92 335 to 105 611)	247.25 (231.43 to 264.71)	1.56 (0.98 to 2.15)	0.000		3825 (2237 to 7025)	26.54 (15.52 to 48.75)	9517 (5212 to 18136)	23.85 (13.06 to 45.46)	−0.11 (−0.24 to 0.03)	0.126
Central sub-Saharan Africa	103 539 (68 606 to 154 759)	2332.62 (1545.63 to 3486.56)	273 590 (221 301 to 337 663)	2637.21 (2133.18 to 3254.83)	−0.90 (−2.22 to 0.43)	0.176		4064 (1475 to 7493)	91.57 (33.22 to 168.8)	4956 (2409 to 9169)	47.77 (23.22 to 88.38)	−2.39 (−2.49 to −2.29)	0.000
East Asia	18 542 (3927 to 27 383)	11.58 (2.45 to 17.09)	376 610 (288 615 to 484 666)	98.81 (75.72 to 127.16)	6.52 (5.83 to 7.21)	0.000		30 197 (20 443 to 47 802)	18.85 (12.76 to 29.84)	47 890 (26 292 to 88 916)	12.56 (6.9 to 23.33)	−1.40 (−1.67 to −1.14)	0.000
Eastern Europe	23 377 (22 422 to 24 696)	47.14 (45.22 to 49.8)	97 186 (92 109 to 103 131)	178.69 (169.36 to 189.62)	5.21 (4.83 to 5.6)	0.000		10 955 (7910 to 16 393)	22.09 (15.95 to 33.06)	7961 (4815 to 13953)	14.64 (8.85 to 25.65)	−3.03 (−3.65 to −2.4)	0.000
Eastern sub-Saharan Africa	709 622 (468 552 to 989 177)	5192.76 (3428.69 to 7238.45)	1680 090 (1 429 214 to 2 025 823)	5688.11 (4838.74 to 6858.62)	−1.66 (−3.29 to −0.01)	0.049		18 096 (8258 to 32 268)	132.42 (60.43 to 236.13)	17 694 (10 153 to 26 961)	59.90 (34.37 to 91.28)	−2.97 (−3.1 to −2.84)	0.000
High-income Asia Pacific	1716 (1585 to 1904)	5.03 (4.65 to 5.58)	9296 (7410 to 11861)	18.76 (14.95 to 23.93)	4.75 (3.96 to 5.54)	0.000		3922 (2203 to 7149)	11.5 (6.46 to 20.96)	4770 (2427 to 9344)	9.63 (4.9 to 18.86)	−0.57 (−0.65 to −0.49)	0.000
High-income North America	126 264 (121 985 to 131 971)	265.71 (256.71 to 277.72)	175 665 (142 239 to 221 715)	192.87 (156.17 to 243.43)	−2.01 (−2.64 to −1.38)	0.000		8224 (4692 to 15150)	17.31 (9.87 to 31.88)	12 775 (6676 to 24 374)	14.03 (7.33 to 26.76)	−0.83 (−0.9 to −0.76)	0.000
North Africa and Middle East	3502 (1556 to 7622)	11.15 (4.95 to 24.27)	60 121 (35 370 to 118 512)	75.44 (44.38 to 148.71)	6.03 (4.89 to 7.18)	0.000		3679 (1969 to 6833)	11.71 (6.27 to 21.75)	8488 (4374 to 16533)	10.65 (5.49 to 20.75)	−0.35 (−0.38 to −0.33)	0.000
Oceania	69 (12 to 322)	12.18 (2.17 to 56.74)	29 527 (12 570 to 77 705)	2131.97 (907.59 to 5610.62)	17.82 (13.67 to 22.13)	0.000		146 (78 to 258)	25.69 (13.83 to 45.43)	341 (178 to 635)	24.63 (12.85 to 45.82)	−0.15 (−0.2 to −0.1)	0.000
South Asia	5125 (2594 to 13404)	4.77 (2.41 to 12.48)	333 390 (244 567 to 527 041)	135.98 (99.75 to 214.97)	10.18 (5.95 to 14.58)	0.000		39 137 (28 283 to 49 831)	36.43 (26.33 to 46.38)	59 791 (42 249 to 83 527)	24.39 (17.23 to 34.07)	−1.57 (−1.79 to −1.35)	0.000
Southeast Asia	9724 (6657 to 14 801)	20.59 (14.09 to 31.33)	338 930 (269 680 to 452 738)	294.29 (234.16 to 393.11)	5.13 (2.21 to 8.12)	0.001		8946 (5367 to 15730)	18.94 (11.36 to 33.3)	18 012 (9629 to 34539)	15.64 (8.36 to 29.99)	−0.67 (−0.73 to −0.62)	0.000
Southern Latin America	5740 (5526 to 5970)	76.66 (73.8 to 79.74)	27 050 (24 377 to 31 206)	215.05 (193.79 to 248.09)	3.40 (3 to 3.79)	0.000		1414 (885 to 2433)	18.89 (11.81 to 32.5)	1832 (1016 to 3544)	14.57 (8.08 to 28.17)	−0.78 (−0.88 to −0.67)	0.000
Southern sub-Saharan Africa	55 982 (24 899 to 117 519)	1191.39 (529.89 to 2501.01)	1 621 763 (1 417 256 to 1 902 030)	16 210.04 (14 165.93 to 19 011.4)	6.2 (2.94 to 9.56)	0.000		2911 (1894 to 4622)	61.96 (40.3 to 98.36)	4267 (2613 to 7332)	42.65 (26.12 to 73.28)	−1.30 (−1.48 to −1.13)	0.000
Tropical Latin America	25 680 (24 601 to 27 063)	159.96 (153.24 to 168.57)	172 046 (161 088 to 185 016)	416.6 (390.07 to 448.01)	2.69 (2.22 to 3.15)	0.000		4712 (2733 to 8597)	29.35 (17.02 to 53.55)	10 784 (5698 to 20 946)	26.11 (13.8 to 50.72)	−0.32 (−0.37 to −0.28)	0.000
Western Europe	52 966 (51 106 to 55 235)	64.00 (61.75 to 66.74)	65 213 (56 303 to 76 562)	57.63 (49.76 to 67.66)	-1.37 (-2.23 to -0.5)	0.003		8032 (4872 to 14 429)	9.71 (5.89 to 17.44)	8225 (4014 to 16821)	7.27 (3.55 to 14.86)	−0.83 (−0.95 to −0.71)	0.000
Western sub-Saharan Africa	153 238 (96 268 to 241 752)	981.32 (616.49 to 1548.16)	1 251 372 (1 106 090 to 1 451 087)	3559.89 (3146.59 to 4128.04)	2.36 (0.54 to 4.2)	0.013		4735 (2924 to 7820)	30.32 (18.72 to 50.08)	8147 (4115 to 15945)	23.18 (11.71 to 45.36)	−1.09 (−1.24 to −0.94)	0.000

**Table 5:** Global rate and average annual percentage change (AAPC) in mortality per 100 000 population of HIV and other sexually transmitted infections (STI) excluding HIV from 1990 to 2019 globally and in different age groups, genders, sociodemographic Index (SDI) regions and geographic regions

Mortality	1990	1990	2019	2019	1990–2019			1990	1990	2019	2019	1990–2019	
**HIV**	**Case (n)**	**Rate (per 100 000 population)**	**Case (n)**	**Rate (per 100 000 population)**	**AAPC (95%)**	***P-*value**	**Other STI**	**case (n)**	**Rate (per 100 000 population)**	**Case (n)**	**Rate (per 100 000 population)**	**AAPC (95%)**	***P-*value**
**Total**	38 533 (27 226 to 51 828)	5.65 (3.99 to 7.6)	182 403 (163 675 to 212 251)	13.23 (11.87 to 15.39)	1.18 (−0.51 to 2.91)	0.166		2702 (1985 to 3184)	0.4 (0.29 to 0.47)	2559 (1889 to 2994)	0.19 (0.14 to 0.22)	−3.02 (−3.26 to −2.78)	0.000
**Age**													
50–54 y	16 878 (11 818 to 22 876)	7.94 (5.56 to 10.76)	81 011 (72 221 to 93 999)	18.55 (16.53 to 21.52)	1.18 (−0.55 to2.93)	0.174		691 (473 to 856)	0.33 (0.22 to 0.4)	670 (469 to 834)	0.15 (0.11 to 0.19)	−2.91 (−3.25 to −2.58)	0.000
55–59 y	10 578 (7518 to 14 146)	5.71 (4.05 to 7.63)	50 790 (45 211 to 59 550)	13.69 (12.19 to 16.05)	1.17 (−0.51 to2.88)	0.165		847 (572 to 1106)	0.46 (0.31 to 0.6)	795 (535 to 1012)	0.21 (0.14 to 0.27)	−3.05 (−3.34 to −2.76)	0.000
60–64 y	6689 (4792 to 8953)	4.16 (2.98 to 5.57)	31 166 (27 565 to 36 592)	9.97 (8.82 to 11.71)	1.06 (−0.61 to2.75)	0.205		645 (469 to 791)	0.4 (0.29 to 0.49)	554 (421 to 669)	0.18 (0.13 to 0.21)	−3.29 (−3.51 to −3.08)	0.000
65–69 y	4389 (3146 to 5897)	3.55 (2.55 to 4.78)	19 435 (17 286 to 22 507)	7.52 (6.68 to 8.7)	0.82 (−0.7 to2.37)	0.278		520 (427 to 630)	0.42 (0.35 to 0.51)	540 (432 to 658)	0.21 (0.17 to 0.25)	−2.72 (−2.84 to −2.59)	0.000
**Sex**													
Female	18 099 (12 173 to 24 920)	5.24 (3.52 to 7.21)	82 349 (73 726 to 94 170)	11.72 (10.49 to 13.4)	0.77 (−1.15 to 2.71)	0.421		1694 (1239 to 1997)	0.49 (0.36 to 0.58)	1985 (1483 to 2381)	0.28 (0.21 to 0.34)	−2.12 (−2.31 to −1.93)	0.000
Male	20 434 (15 049 to 27 052)	6.07 (4.47 to 8.04)	100 054 (88 357 to 118 066)	14.80 (13.07 to 17.46)	1.57 (0.09 to 3.08)	0.038		1009 (517 to 1451)	0.3 (0.15 to 0.43)	573 (297 to 789)	0.08 (0.04 to 0.12)	−5.07 (−5.4 to −4.74)	0.000
**Sociodemographic index (SDI)**													
High SDI	5117 (5062 to 5172)	3.34 (3.3 to 3.37)	4749 (4631 to 5033)	1.89 (1.84 to 2)	−2.71 (−3.36 to −2.06)	0.000		148 (124 to 155)	0.1 (0.08 to 0.1)	96 (91 to 103)	0.04 (0.04 to 0.04)	−3.03 (−3.43 to −2.63)	0.000
High-middle SDI	1989 (1903 to 2094)	1.07 (1.02 to 1.12)	9242 (8483 to 10 336)	2.74 (2.51 to 3.06)	2.67 (1.73 to 3.62)	0.000		400 (328 to 435)	0.22 (0.18 to 0.23)	241 (215 to 267)	0.07 (0.06 to 0.08)	−5.37 (−6 to −4.74)	0.000
Low SDI	23 547 (15 141 to 32 997)	53.60 (34.47 to 75.11)	48 684 (42 071 to 58 243)	52.4 (45.28 to 62.69)	−1.74 (−3.29 to −0.16)	0.032		724 (381 to 1170)	1.65 (0.87 to 2.66)	638 (379 to 867)	0.69 (0.41 to 0.93)	−3.21 (−3.33 to −3.08)	0.000
Low-middle SDI	5783 (3292 to 9248)	5.23 (2.98 to 8.37)	54 500 (46 581 to 65 025)	22.82 (19.51 to 27.23)	2.28 (0.05 to 4.56)	0.046		884 (648 to 1111)	0.80 (0.59 to 1)	1049 (758 to 1351)	0.44 (0.32 to 0.57)	−2.29 (−2.53 to −2.05)	0.000
Middle SDI	2060 (1468 to 2746)	1.10 (0.78 to 1.46)	65 056 (58 590 to 74 087)	14.24 (12.82 to 16.21)	6.74 (3.8 to 9.76)	0.000		545 (399 to 644)	0.29 (0.21 to 0.34)	533 (393 to 628)	0.12 (0.09 to 0.14)	−3.03 (−3.3 to −2.75)	0.000
**Region**													
Andean Latin America	104 (64 to 261)	2.97 (1.84 to 7.49)	584 (398 to 1101)	6.32 (4.31 to 11.92)	2.39 (1.6 to 3.19)	0.000		8 (6 to 12)	0.24 (0.17 to 0.36)	11 (7 to 15)	0.12 (0.08 to 0.17)	−2.34 (−2.45 to −2.23)	0.000
Australasia	76 (72 to 79)	2.20 (2.10 to 2.30)	33 (31 to 34)	0.49 (0.46 to 0.51)	−6.43 (−7.19 to −5.66)	0.000		3 (3 to 4)	0.09 (0.07 to 0.1)	1 (1 to 2)	0.02 (0.02 to 0.03)	−4.57 (−5.18 to −3.95)	0.000
Caribbean	596 (398 to 937)	14.43 (9.64 to 22.69)	2376 (2028 to 2777)	27.99 (23.89 to 32.71)	−0.10 (−1.63 to 1.44)	0.890		20 (11 to 33)	0.48 (0.27 to 0.79)	28 (19 to 42)	0.33 (0.23 to 0.5)	−0.84 (−1.14 to −0.55)	0.000
Central Asia	27 (25 to 29)	0.31 (0.29 to 0.33)	144 (133 to 157)	0.96 (0.89 to 1.05)	4.06 (3.26 to 4.86)	0.000		37 (28 to 41)	0.42 (0.32 to 0.47)	23 (19 to 30)	0.15 (0.12 to 0.2)	−4.69 (−5.26 to −4.11)	0.000
Central Europe	133 (125 to 140)	0.53 (0.5 to 0.56)	128 (119 to 154)	0.43 (0.4 to 0.51)	−1.03 (−1.38 to −0.67)	0.000		21 (16 to 22)	0.08 (0.07 to 0.09)	9 (8 to 11)	0.03 (0.03 to 0.04)	−2.97 (−3.33 to −2.62)	0.000
Central Latin America	615 (605 to 627)	4.27 (4.19 to 4.35)	2799 (2639 to 2985)	7.02 (6.62 to 7.48)	1.41 (0.84 to 1.98)	0.000		32 (30 to 37)	0.22 (0.21 to 0.26)	57 (48 to 68)	0.14 (0.12 to 0.17)	−0.68 (−1.17 to −0.19)	0.008
Central sub-Saharan Africa	2985 (1915 to 4468)	67.26 (43.15 to 100.66)	7575 (6169 to 9312)	73.02 (59.47 to 89.76)	−1.09 (−2.42 to 0.27)	0.111		105 (24 to 215)	2.37 (0.54 to 4.83)	96 (30 to 217)	0.93 (0.29 to 2.09)	−3.44 (−3.60 to −3.27)	0.000
East Asia	539 (72 to 820)	0.34 (0.04 to 0.51)	11 134 (8703 to 13 784)	2.92 (2.28 to 3.62)	6.39 (5.67 to 7.11)	0.000		419 (280 to 516)	0.26 (0.17 to 0.32)	222 (156 to 274)	0.06 (0.04 to 0.07)	−4.68 (−5.35 to −4.01)	0.000
Eastern Europe	644 (633 to 656)	1.3 (1.28 to 1.32)	2531 (2464 to 2599)	4.65 (4.53 to 4.78)	5.08 (4.68 to 5.47)	0.000		183 (141 to 201)	0.37 (0.29 to 0.4)	80 (68 to 98)	0.15 (0.13 to 0.18)	−6.30 (−7.51 to −5.08)	0.000
Eastern sub-Saharan Africa	20 268 (12 837 to 28 914)	148.32 (93.94 to 211.58)	45 323 (38 710 to 54 689)	153.44 (131.06 to 185.15)	−1.89 (−3.55 to −0.19)	0.031		466 (174 to 907)	3.41 (1.28 to 6.63)	356 (174 to 555)	1.2 (0.59 to 1.88)	−3.79 (−3.96 to −3.62)	0.000
High-income Asia Pacific	49 (47 to 51)	0.14 (0.14 to 0.15)	202 (194 to 212)	0.41 (0.39 to 0.43)	3.66 (2.76 to 4.57)	0.000		24 (19 to 26)	0.07 (0.06 to 0.08)	15 (14 to 16)	0.03 (0.03 to 0.03)	−2.39 (−2.58 to −2.2)	0.000
High-income North America	3652 (3605 to 3698)	7.69 (7.59 to 7.78)	3522 (3461 to 3591)	3.87 (3.8 to 3.94)	−3.29 (−3.93 to −2.64)	0.000		46 (43 to 48)	0.1 (0.09 to 0.1)	45 (42 to 48)	0.05 (0.05 to 0.05)	−2.45 (−2.86 to −2.04)	0.000
North Africa and Middle East	98 (40 to 223)	0.31 (0.13 to 0.71)	1694 (1008 to 3334)	2.13 (1.26 to 4.18)	5.98 (4.83 to 7.15)	0.000		20 (15 to 26)	0.06 (0.05 to 0.08)	36 (20 to 47)	0.05 (0.03 to 0.06)	−1.31 (−1.67 to −0.95)	0.000
Oceania	2 (0 to 9)	0.34 (0.04 to 1.58)	823 (366 to 2147)	59.39 (26.39 to 154.99)	17.78 (13.61 to 22.1)	0.000		2 (1 to 3)	0.29 (0.13 to 0.57)	3 (1 to 7)	0.22 (0.09 to 0.48)	−0.66 (−0.81 to −0.51)	0.000
South Asia	105 (30 to 324)	0.1 (0.03 to 0.3)	8752 (6092 to 14 589)	3.57 (2.48 to 5.95)	10.49 (6.04 to 15.13)	0.000		963 (678 to 1231)	0.9 (0.63 to 1.15)	1256 (866 to 1641)	0.51 (0.35 to 0.67)	−2.18 (−2.44 to −1.92)	0.000
Southeast Asia	246 (148 to 410)	0.52 (0.31 to 0.87)	9420 (7330 to 12 883)	8.18 (6.36 to 11.19)	5.2 (2.17 to 8.32)	0.001		89 (66 to 120)	0.19 (0.14 to 0.25)	105 (65 to 141)	0.09 (0.06 to 0.12)	−2.52 (−2.74 to −2.29)	0.000
Southern Latin America	176 (171 to 180)	2.34 (2.29 to 2.41)	665 (646 to 684)	5.29 (5.14 to 5.44)	2.68 (2.23 to 3.14)	0.000		18 (15 to 21)	0.25 (0.21 to 0.28)	14 (12 to 17)	0.11 (0.1 to 0.13)	−2.33 (−2.68 to −1.97)	0.000
Southern sub-Saharan Africa	1541 (632 to 3354)	32.79 (13.45 to 71.38)	43 875 (37 974 to 52 164)	438.55 (379.56 to 521.4)	6.02 (2.7 to 9.45)	0.001		54 (33 to 83)	1.14 (0.71 to 1.77)	51 (40 to 74)	0.5 (0.4 to 0.74)	−2.79 (−3.11 to −2.48)	0.000
Tropical Latin America	751 (731 to 769)	4.68 (4.55 to 4.79)	4659 (4497 to 4829)	11.28 (10.89 to 11.69)	2.36 (1.89 to 2.82)	0.000		41 (38 to 45)	0.26 (0.24 to 0.28)	69 (61 to 75)	0.17 (0.15 to 0.18)	−1.36 (−1.49 to −1.23)	0.000
Western Europe	1565 (1539 to 1594)	1.89 (1.86 to 1.93)	1333 (1300 to 1369)	1.18 (1.15 to 1.21)	−2.58 (−3.42 to −1.73)	0.000		90 (82 to 97)	0.11 (0.1 to 0.12)	37 (34 to 41)	0.03 (0.03 to 0.04)	−3.78 (−4.22 to −3.34)	0.000
Western sub-Saharan Africa	4361 (2625 to 6965)	27.93 (16.81 to 44.61)	34831 (31097 to 39851)	99.09 (88.47 to 113.37)	2.21 (0.37 to 4.09)	0.020		64 (32 to 85)	0.41 (0.2 to 0.55)	44 (32 to 58)	0.12 (0.09 to 0.16)	−4.6 (−4.95 to −4.25)	0.000

In 2019, the HIV incidence rate was highest among those aged 55–59 years (rate 13.98 per 100 000 population (9.72 to 18.77)). The highest other STI incidence rate was observed in the 50–54 years group (rate 11 108.33 (7730.79 to 15 084.81)) (**Table 2**). The prevalence rate was highest among those aged 50–54 years (rate 681.26 per 100 000 population (633.66 to 731.34). The group aged 50–54 years recorded the highest DALYs and mortality rates, standing at 760.24 per 100 000 population (675.79 to 886.93) and 18.55 per 100 000 population (16.53 to 21.52), respectively (**Table 3**).

It's noteworthy that trichomoniasis exhibited the highest other STI incidence rate across all age groups, while genital herpes led in prevalence, followed closely by trichomoniasis. In 2019, the incidence and prevalence rates for trichomoniasis, syphilis, gonococcal infections, genital herpes, and chlamydial infections were all highest among those aged 50–54 years (Table S1 in the **Online Supplementary Document**).

### Analysis by gender group

When breaking down by gender, disparities become apparent. Both genders saw a significant decline in HIV incidence rates, while the other STI excluding HIV displayed a minor increase: for female, the AAPC was −2.38 (−2.76 to −2.00) for HIV and 0.15 (0.09 to 0.21) for other STI, and for male, it was −2.04 (−2.36 to −1.71) for HIV and 0.1 (0.04 to 0.15) for other STI. Regarding prevalence, HIV and other STI trends were the exact opposite of incidence rates (**Table 2**, **Table 3**). Female prevalence rate had an AAPC of 5.12 (4.47 to 5.77) for HIV and −0.02 (−0.05 to 0.01) for other STI. For men, it was 5.41 (4.89 to 5.94) for HIV and −0.05(−0.11 to 0) for other STI. Compared to 1990, DALYs and mortality rates for HIV have increased to varying degrees in both genders in 2019. For women, the rates went from 183.83 per 100 000 population (127.44 to 249.43) and 5.24 (3.52 to 7.21) in 1990 to 434.26 (385.45 to 501.14) and 11.72 (10.49 to 13.4) in 2019, respectively. For men, the rise went from 211.63 (160.06 to 273.92) and 6.07 (4.47 to 8.04) in 1990 to 543.69 (478.63 to 644.03) and 14.8 (13.07 to 17.46) in 2019 (**Table 3**). Both genders showed a decline in DALYs and mortality rates for other STI (**Table 4**, **Table 5**).

**Figure 3:**
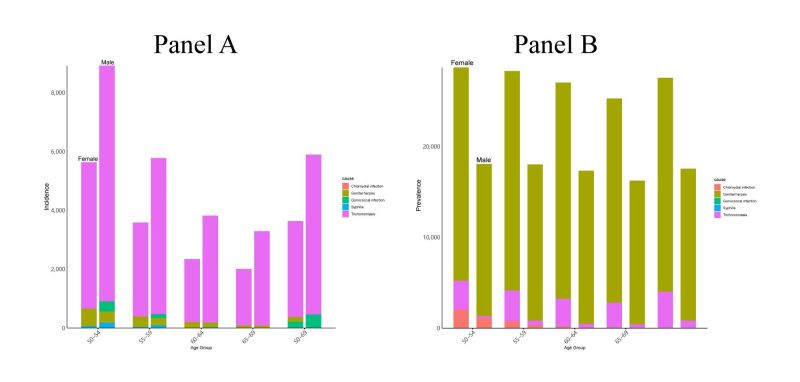
Stacked cube of incidence (**Panel A**) and prevalence (**Panel B**) rate of five other STI in 2019 based on gender and age group. In an age group, the left shows female and the right shows male. STI – sexually transmitted infections

In 2019, female displayed lower HIV (rate 11.98 (9.02 to 15.08)) and other STI (rate 5332.97 (4134.8 to 6771.09)) incidence rates than male (rate 13.35 (9.53 to 17.67); 7805.93 (6028.34 to 10 071.58)). Among other STI data female incidence rates for trichomoniasis (rate 3641.33 (2546.02 to 5019.52)), syphilis (rate 31.41 (17.73 to 52.46)), and gonococcal infections (rate 212.88 (137.77 to 329.94)) were significantly lower than for men (rate 5896.97 (4186.98 to 8229.06); 88.22 (50.84 to 147.10); 456.86 (295.78 to 720.29)). Conversely, female prevalence rates for trichomoniasis (rate 4014.85 (2616.18 to 5747.25)), genital herpes (rate 27 578.26 (23 622.00 to 32 004.49)), and chlamydial infections (rate 898.08 (556.26 to 1368.68)) were markedly higher than for men (rate 825.46 (556.90 to 1172.05); 17 565.97 (14 856.15 to 20 757.33); 467.28 (272.23 to 729.83)) (**Figure 3**, panels A–B, Table S3 in the **Online Supplementary Document**).

In 2019, the highest DALYs in women was from trichomoniasis (rate 7.49 (2.76 to 16.72)), whereas in male, it was from genital herpes (rate 4.23 (1.30 to 10.53)). Subsequent detailed joinpoint regression analyses for the different other STI from 1990–2019 revealed that among female (Table S4 in the **Online Supplementary Document**), the most rapid growth in incidence rate was observed for trichomoniasis (AAPC 0.21 (0.16 to 0.25)), while chlamydial infections led in prevalence growth (AAPC 0.19 (0.12 to 0.26)). For male, chlamydial infections took the lead for both incidence (AAPC 0.14 (0.04 to 0.23)) and prevalence growth (AAPC 0.12 (0.02 to 0.21)).

### Analysis by SDI regions

**Figure 4:**
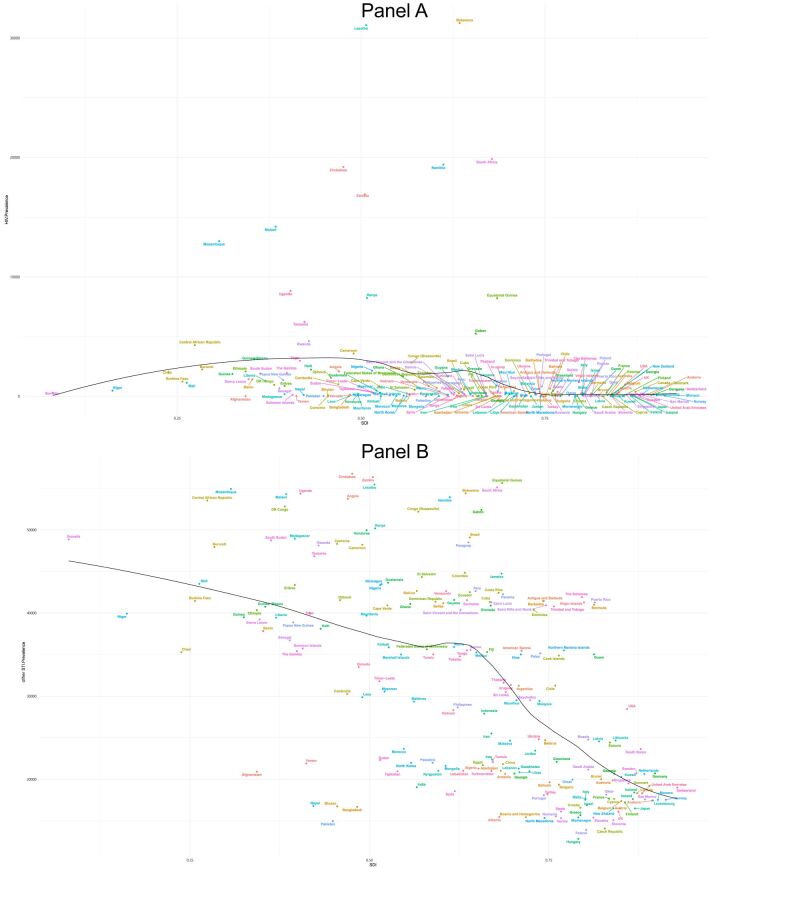
Prevalence rate for 204 countries and territories in 2019, for both sexes by SDI. **Panel A.** HIV. **Panel B.** Other STI excluding HIV. The black line represents the expected prevalence rate based solely on SDI. Locations lower than the solid black line had a lower-than-expected burden, while those below the line had a lower-than-expected burden. SDI – sociodemographic index, STI – sexually transmitted infections

When segmenting by the sociodemographic index (SDI) regions, a discernible pattern emerges for HIV incidence (Table S5 in the **Online Supplementary Document**). Generally, the lower the SDI, the higher the incidence rate (**Figure 4**, panel A). The middle SDI regions display the most accelerated growth rates for prevalence, DALYs, and mortality when compared to other SDI regions, with AAPCs of 9.46 (7.98 to 10.95), 6.88 (3.97 to 9.86), and 6.74 (3.8 to 9.76) respectively (**Table 3**, **Table 4**, **Table 5**). In terms of incidence number, the middle SDI region led in 2019, accounting for 71 830 cases (**Table 2**), making up 41.20% of the global incidence in individuals aged 50–69. For prevalence, the middle SDI region again topped in 2019, totalling 2 472 400 cases, which made up 33.72% of the global prevalence.

For the other STI in 2019, the low SDI region reported the highest incidence rate (rate 9078.78 (6929.78 to 11 900.9)), while the high SDI region reported the least (rate 4795.24 (3734.64 to 6175.22)) (**Figure 4**, panel B, **Table 2**). The most rapid increase in incidence rate was observed in the high-middle SDI (AAPC 0.2 (0.08 to 0.32)). When it comes to prevalence rates, except for the high-middle SDI region, all other areas have been witnessing a slow decline. Both DALYs and mortality rates have been dropping across all regions, most noticeably in the low SDI and high-middle SDI regions. The DALYs recorded AAPC of −2.31 (−2.41 to −2.21) and −1.57 (−1.78 to −1.35) respectively, and mortality rates showcased AAPC of −3.21 (−3.33 to −3.08) and −5.37(−6.00 to −4.74) respectively (**Table 4**, **Table 5**).

### Analysis by geographical regions

**Figure 5:**
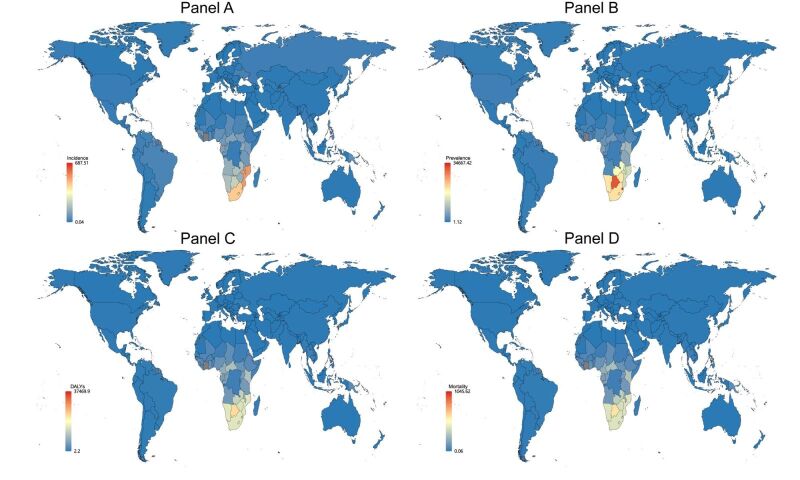
Global map of 2019 rates (per 100 000 population) of HIV. Gray areas represent missing data. **Panel A.** Incidence. **Panel B.** Prevalence. **Panel C.** DALYs. **Panel D.** Mortality. DALYs – disability-adjusted life years

**Figure 6:**
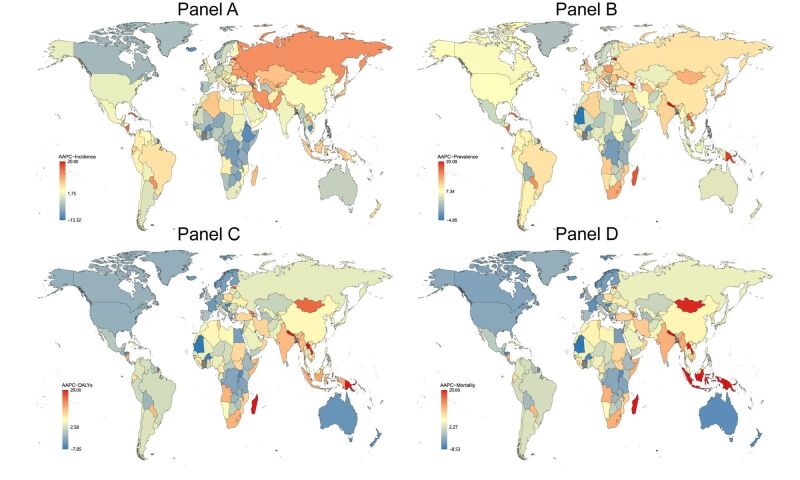
Global map of AAPC of HIV in incidence (**Panel A**), prevalence (**Panel B**), DALYs (**Panel C**), and mortality (**Panel D**) from 1990 to 2019. The gray area represents missing data or inability to calculate. AAPC – average annual percentage change, DALYs – disability-adjusted life years

**Figure 7:**
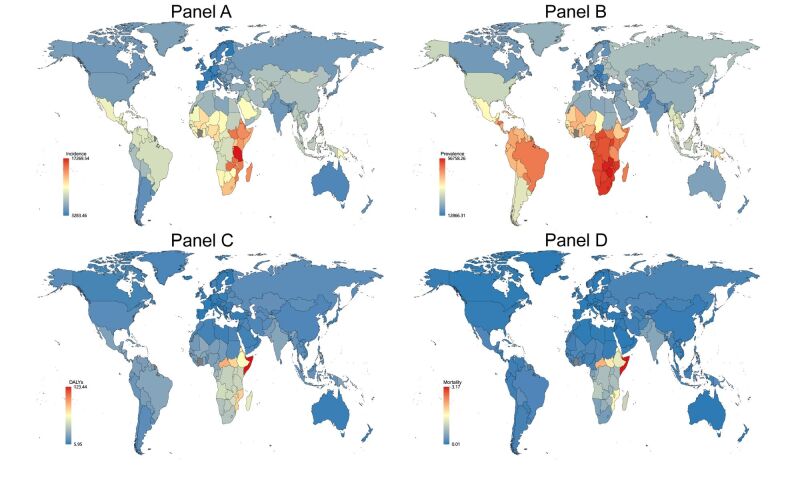
Global map of 2019 rates (per 100 000 population) of other STI excluding HIV incidence (**Panel A**), prevalence (**Panel B**), DALYs (**Panel C**), and mortality (**Panel D**). Gray areas represent missing data. DALYs – disability-adjusted life-years, STI – sexually transmitted infections

**Figure 8:**
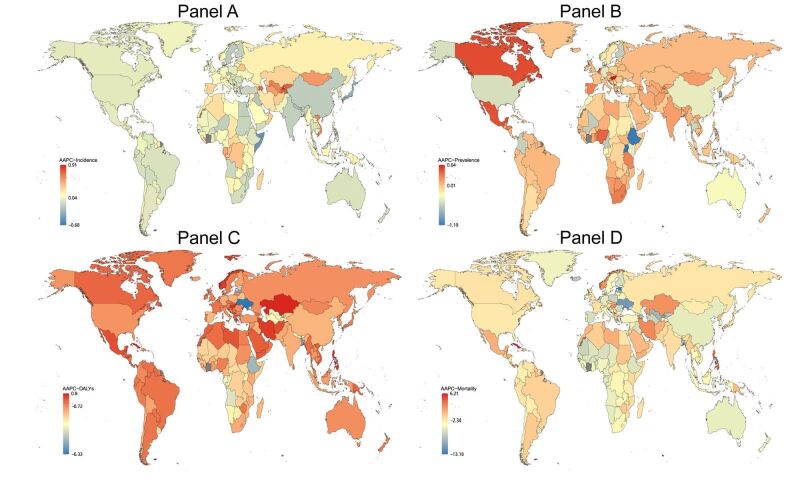
Global map of AAPC of other STI excluding HIV in incidence (**Panel A**), prevalence (**Panel B**), DALYs (**Panel C**), and mortality (**Panel D**) from 1990 to 2019. The gray area represents missing data or inability to calculate. AAPC – average annual percentage change, DALYs – disability-adjusted life years, STI – sexually transmitted infections

The regions with the fastest growing incidence rates in HIV are Eastern Europe with an AAPC of 11.95 (9.92 to 14.02), Central Asia (AAPC 10.68 (9.19 to 12.19)) (**Figure 5**, **Figure 6**). The fastest growth in other STI incidence rates is Central Asia (AAPC 0.52 (0.28 to 0.76)) (**Figure 7**, **Figure 8**). The most rapid growth in prevalence rates in HIV include Oceania (AAPC 15.87 (12.54 to 19.31)), high-income Asia Pacific (AAPC 10.24 (9.25 to 11.25)). Notably, the regions with the steepest inclines in DALYs and mortality of HIV are Oceania and South Asia, far surpassing other regions.

It's evident that in 2019, the highest rates of HIV incidence, prevalence, DALYs, and mortality were predominantly in Sub-Saharan Africa, specifically Southern sub-Saharan Africa, followed by Eastern sub-Saharan Africa (**Figure 5**, Table S6 in the **Online Supplementary Document**). Shifting focus to other STI, the global perspective shows that other STI incidence is highest in Eastern sub-Saharan Africa, Southern sub-Saharan Africa, Western sub-Saharan Africa (**Figure 7**). The highest prevalence in other STI is concentrated in Southern sub-Saharan Africa, Central sub-Saharan Africa. The majority of DALYs and mortality from other STI are in Eastern sub-Saharan Africa.

### Analysis by nations

Diving into individual nations, the highest rates of HIV incidence were observed in Lesotho, Equatorial Guinea, and Mozambique. In terms of prevalence, DALYs, and mortality rates of HIV, Lesotho topped the list, followed by Eswatini and Botswana (Table S7 in the **Online Supplementary Document**).

Pertinently, the nations with the swiftest increase in incidence of HIV is Georgia (AAPC 28.24 (23.83 to 32.81)) (**Figure 6**, Table S8 in the **Online Supplementary Document**). The fastest growth in other STI incidence rates of nation is Cabo Verde (AAPC 0.91 (0.63 to 1.19)) (**Figure 8**, Table S9 in the **Online Supplementary Document**). In terms of prevalence, the ones observing the quickest rise is Georgia (AAPC 25.30 (21.85 to 28.84)). The swiftest rise in prevalence rates is Hungary (AAPC 0.64 (0.34 to 0.94)). The sharpest rises in DALYs and mortality of include Nepal, Lao People's Democratic Republic, Madagascar, Papua New Guinea, Djibouti, Estonia, Mongolia, Mauritius, and Georgia.

### Predictions of prevalence rate at global levels

**Figure 9:**
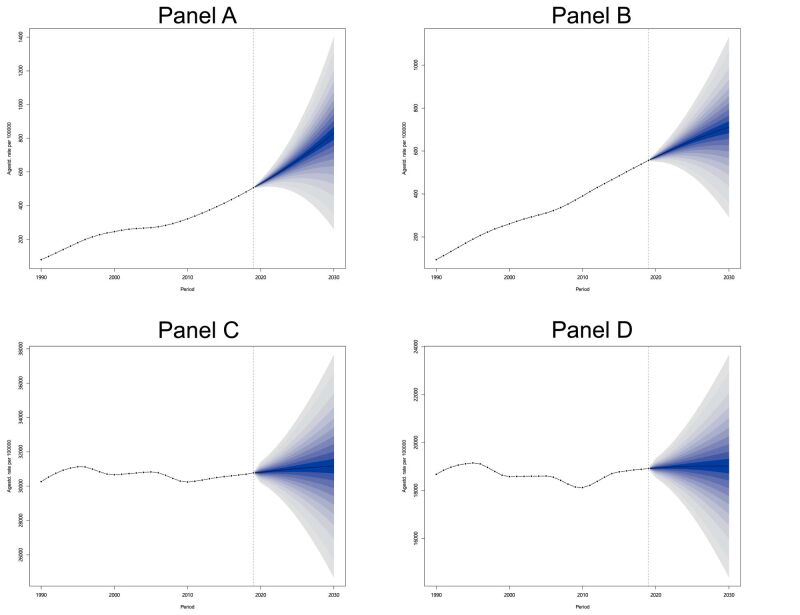
The predictions of global prevalence rates for male and female from 2020 to 2030. HIV. **Panel A.** Female. **Panel B.** Male. Other STI excluding HIV. **Panel C.** Female. **Panel D.** Male. The black line represents the predicted mean prevalence rate, and the gradient area is its 95% confidence interval. STI – sexually transmitted infections

Based on the BAPC prediction model, we obtained the prevalence rate (per 100 000) of STI from 2020 to 2030 (**Figure 9**, Table S10 in the **Online Supplementary Document**). The prevalence rate of HIV in both genders has increased, with an average increase of 63.97% for female, from 505.93 in 2019 to 829.59 in 2030. Male increased from 556.59 to 710.85, an increase of 27.72%. In terms of the other STI, female increased from 30 770.01 to 31 165.98, while male increased from 18 920.04 to 19 019.91. Overall, the prevalence of STI in the population from 2020 to 2030 showed an increasing trend, with the exception of HIV prevalence increasing more significantly compared to other STI, and the increase in female was greater than that in male.

## DISCUSSION

Our findings revealed that in 2019, the incidence and prevalence numbers for the 50–69 age bracket constituted 8.77 and 19.91% of global HIV cases and 11.72 and 26.74% of global other STI, respectively. Previous studies have also indicated a gradual rise in the incidence and prevalence of STI among the elderly worldwide [2,3,9,16], such as South Africa [17,18], the US [19,20], the UK [7,21], China [22,23], Canada [24], Australia [9], and Spain [25]. Similar to the global trend [26], we have found that sub-Saharan Africa is also the epicenter for HIV. According to our data, nations such as Lesotho, Equatorial Guinea, Mozambique, Eswatini, and Botswana are among the hardest hit. Additionally, countries including Cuba, Kazakhstan, Hungary, the Philippines, Iran, and Norway have experienced the fastest growth in the health burden of STI. These countries require our focused attention because they are vulnerable to the impact of epidemics when unprepared for increased pressure on resources. These findings also indicate that HIV infection rates and health burdens among elderly populations vary significantly across different regions, potentially influenced by factors such as regional culture, social structures, and health care resource allocation. As a result, it is crucial to develop targeted intervention measures and allocate health care resources for their health protection.

On a broad scale, our study indicated that worldwide efforts have led to a decrease in HIV incidence among individuals aged 50–69, culminating in 2019's incidence rates being lower than those in 1990. The rise in HIV incidence rates was most rapid between 1990 and 1992, after the peak in 1996, a gradual decline ensued. Coincidentally, antiretroviral therapy (ART) first became available, around the same time around the same time, in 1996 [27]. As a result, this decline may be attributed to the widespread use of ART globally in the nearly two decades. The prevalence rates didn't demonstrate a stark shift and post-2006, the growth rate of prevalence quickened – a surprising trend. This can be substantiated by widespread application of ART, societal emphasis on HIV prevention and the fact that since 2003, funding for HIV prevention and control by the US Centers for Disease Control and Prevention has steadily increased. Following a plateau between 2002 and 2005, DALYs and mortality rates due to HIV witnessed a marked decline. Perhaps, a reduction in mortality rates and subsequent increase in life expectancy are causes for the upward linear trend in the prevalence rates for the 50–69 age group – a trend incongruent with incidence rates. Overall, in contrast to declining HIV incidence rates from 1990 to 2019, other STI rates rose over the same period [28]. This may can be interpreted that although the funding from the US Centers for Disease Control and Prevention for the prevention and control of other STI has not changed since 2003, there has been a significant reduction in the funding, when adjusted for inflation.

Our research indicates that the number of HIV incidences is highest among population aged 50–54, the young elderly population, but its rate of incidence decreases most rapidly. This suggests that HIV prevention efforts may be most effective among this age segment, and continuing to intensify prevention and control measures targeting this age group could yield the greatest benefits for the overall elderly population. Regrettably, irrespective of the age group, the DALYs and mortality rates in 2019 were nearly double those of 1990. Gender-wise, women generally exhibit lower incidence and prevalence rates of HIV compared to men, with a slightly more rapid decline in their rates of incidence, which aligns with the global distribution [29]. However, a consistent body of literature also highlights that women continue to face disproportionately higher risks of HIV infection and associated mortality due to factors such as social discrimination, lack of support, and comorbidities [26]. Consequently, the efforts in its prevention and control among the elderly female population must persist with sustained attention and resources to ensure that their health and well-being are not overlooked. Delineating by age, the highest other STI incidence rate was seen in the 50–54 age bracket, whereas the fastest growth rate was among those aged 65–69. This pattern echoes the HIV trend, emphasising the need to monitor other STI prevalence among older individuals. Overall, efforts should be concentrated on enhancing the prevention of other STI among the elderly population aged 50–54 and 65–69, particularly targeting male seniors. Additionally, special attention should be given to addressing the incidence of chlamydia and the prognosis of genital herpes among male elderly individuals, along with tackling the prevalence and prognosis issues of trichomoniasis among female seniors.

In our correlation analysis, prevention and control strategies for STI should vary according to SDI levels. In middle SDI regions, both youth [30] and the elderly face heightened risks, necessitating comprehensive plans, increased investment, expanded medical services, and reinforced prevention measures to address the rapidly growing disease burden. High SDI regions should prioritise basic prevention and control measures, such as enhancing sex education, promoting social support and awareness of sexually transmitted diseases, and providing broader testing services to prevent the rise in incidence rates. Low SDI regions, despite declining HIV rates, still face substantial risks, underscoring the need for continued development of health facilities, universal medical services, and community education efforts to curb disease spread.

The rising trends of STI in the elderly can be attributed to several factors. First and foremost, due to the widespread adoption of systematic therapies, mortality rates have seen a decline. The extensive use of Highly ART has lowered the mortality rates of patients with HIV, consequently extending their life expectancy [20]. Second, societal progression in terms of perspectives, coupled with the sexual activeness of the elderly, stands in stark contrast to their often outdated views on sexual health. The societal shifts over the past decades have made it more common for older individuals to meet new partners and engage with sex workers. However, older generations are less likely to have received formal sex education in schools and are typically excluded from sexual health promotion campaigns [7]. Such factors might lead them towards unsafe sexual practices, heightening the risk of STI transmission [31]. Contrary to the misconception that elderly individuals are sexually inactive, literature consistently indicates that sexual activity among those over 50 is higher than assumed [17,32–35]. However, global overviews on HIV and aging show that very little is known about the HIV knowledge and sexual behaviours of older adults worldwide [36,37]. Older individuals are less likely to use condoms, increasing their risk of contracting or transmitting HIV [17]. The widespread use of sildenafil (commonly known as Viagra) enables older men to engage in sexual activity more frequently [38]. Moreover, older men who use Viagra are more likely to partake in unprotected intercourse [39]. Third, the allocation of sexual health resources for the elderly is inequitable, particularly in the realms of health education and clinical care. Healthcare professionals are often hesitant to broach the topic of sexual health with older patients. Due to feelings of embarrassment or other societal barriers, seniors are typically reticent to raise sexual health concerns with their health care providers. Moreover, compared to younger individuals, the elderly tend to exhibit a more extended lag between recognising symptoms and seeking clinical intervention [40]. In a sample of individuals aged 40–80 in the USA over the years, more than 75% of those with sexual health issues did not seek the counsel of health experts [41]. Such delays pose significant challenges in the effective prevention and management of STI within this demographic. Until now, global responses to HIV have predominantly focused on mothers, children, adolescent girls, and young women, often overlooking the unique needs of older individuals living with HIV [30]. The burden of STI in the elderly is increasingly concerning [7,42]. Older individuals with STI are confronting emerging challenges, such as heightened risks for cardiovascular diseases, chronic kidney ailments, and osteoporosis [43–46]. Beyond these amplified medical needs, many elderly individuals with STI also grapple with mental health and psychosocial issues like substance use, feelings of isolation, and a lack of social support [47–49].

In this context, we have also endeavoured to forecast the incidence rate of STI for the decade spanning from 2020 to 2030. This is similar to previous predictions for individual regions [7,50]. Our projections are only grounded in the age brackets and gender disparities observed in the year 2019, coupled with the World Health Organization's estimates of the forthcoming global populace, with the projected rates mirroring the global STI landscape as it was in 2019. Should there be an enhancement in global health status and economic conditions, our analysis, which is contingent upon the SDI, suggests that the actual STI incidence rate could witness a substantial decline compared to our forecasts. Furthermore, an upscale in global governmental investments dedicated to STI prevention and treatment, along with a marked improvement in the sexual health awareness among the elderly demographic, could potentially lead to a reduction in the estimated incidence rates [51–53]. We believe that these insights underscore the potential for significant progress in the fight against STI, provided that strategic investments and public health initiatives are optimised and intensified on a global scale [9,54–56]. The pandemic of COVID-19 has interestingly impacted the incidence rates of STI. Due to the containment measures implemented during the pandemic [57], there was a decline in the incidence of STI in many regions [58–60]. However, there has been a recent rebound in these rates, which calls for further analysis.

Strengths of our study include, first and foremost, our pioneering use of the GBD database to analyse the annual changes in incidence, prevalence, DALYs, and mortality rates of HIV and other STI for the 50–69 age group from 1990 to 2019. Additionally, we utilised joinpoint regression analysis, providing a comprehensive fit for HIV and other STI trends from 1990 to 2019, offering a clear and intuitive depiction of their trajectories. Moreover, by employing the joinpoint model, we calculated the AAPC for various subgroups, which directly reflects their rate of change. However, there are limitations to our study. First, our study relies on the GBD database. Given the diverse data sources across nations and regions within the GBD, there's a considerable margin of error. Furthermore, considering the potential stigma elderly individuals might associate with STI, we estimate that the data obtained might underrepresent the actual situation. Second, perhaps the most crucial limitation to highlight is that our analysis only delved into a few straightforward influencing factors. However, there are numerous elements impacting the incidence and prevalence of STI, such as economic burdens, health care policies, and more. Merely examining the correlation of rates over the years doesn't offer a holistic and comprehensive understanding of the global trends. This aspect certainly warrants more in-depth exploration in future study endeavours. In future research, exploring alternative data sources or methodologies to validate and supplement GBD data could enhance the accuracy and reliability of the data. Addressing potential data acquisition barriers stemming from underlying societal biases could involve utilising more privacy-protective data collection methods, such as anonymous surveys or community participatory research, to mitigate patient reporting biases and more accurately reflect real-world situations. Additionally, further expanding the scope of analysis to investigate other factors influencing STI incidence and prevalence, such as economic burdens and health care policies, represents a promising direction for future research.

## CONCLUSIONS

In summary, our study underscored the urgency for health care providers to turn their attention to older populations, ensuring they receive adequate sexual health education and clinical care. Our study also indicates that adjusting care strategies in line with the epidemiological trends of STI in the elderly is essential.

## Additional material


Online Supplementary Document
