# Supplementary material for: The trend analysis of HIV and other sexually transmitted infections among the elderly aged 50 to 69 years from 1990 to 2030
Source: J Glob Health. 2024 Jul 19;14:04105. doi: 10.7189/jogh.14.04105 (PMC11258536; doi:10.7189/jogh.14.04105)
Supplement: Online Supplementary Document [file jogh-14-04105-s001.pdf]

Supplementary Table Description

Table S0	Supplementary Table Description
Table S1	The number and rate of incidence, prevalence, DALYs and mortality of HIV, other STI (including Chlamydial infection, Genital herpes, Gonococcal infection, Syphilis and Trichomoniasis) in different ages.
Table S2	Global AAPC in incidence, prevalence, DALYs and mortality per 100,000 population of HIV and other STI by joinpoint regressive analysis.
Table S3	The number and rate of incidence, prevalence, DALYs and mortality of HIV, other STI in different genders.
Table S4	Global AAPC in incidence and prevalence of Chlamydial infection, Genital herpes, Gonococcal infection, Syphilis and Trichomoniasis by joinpoint regressive analysis in different genders.
Table S5	The number and rate of incidence, prevalence, DALYs and mortality of HIV, other STI in different SDI regions.
Table S6	The number and rate of incidence, prevalence, DALYs and mortality of HIV, other STI in different geographical regions.
Table S7	The number and rate of incidence, prevalence, DALYs and mortality of HIV, other STI in different nations.
Table S8	Global AAPC in incidence, prevalence, DALYs and mortality per 100,000 population of HIV in different nations.
Table S9	Global AAPC in incidence, prevalence, DALYs and mortality per 100,000 population of other STI in different nations.
Table S10	Predicted by the BAPC model, the prevalence rate for male and female from 2020 to 2030.
	DALYs = disability adjusted life year, AAPC = average annual percentage change, other STI = other sexually transmitted infections excluding HIV, SDI = Sociodemographic Index, BAPC = Bayesian age-period-cohort.

measure	location	sex	age	cause	metric	year	val	upper	lower
Deaths	Global	Both	50-54 yea	HIV/AIDS	Number	1990	16877.5	22875.8	11817.9
Deaths	Global	Both	50-54 yea	HIV/AIDS	Rate	1990	7.93895	10.7605	5.55896
Deaths	Global	Both	55-59 yea	HIV/AIDS	Number	1990	10578.5	14146	7517.95
Deaths	Global	Both	55-59 yea	HIV/AIDS	Rate	1990	5.70529	7.62934	4.05465
Deaths	Global	Both	60-64 yea	HIV/AIDS	Number	1990	6688.54	8952.92	4792.02
Deaths	Global	Both	60-64 yea	HIV/AIDS	Rate	1990	4.1636	5.57317	2.98302
Deaths	Global	Both	65-69 yea	HIV/AIDS	Number	1990	4388.78	5897.16	3145.54
Deaths	Global	Both	65-69 yea	HIV/AIDS	Rate	1990	3.554	4.77547	2.54723
Deaths	Global	Both	50-69 yea	HIV/AIDS	Number	1990	38533.3	51828.3	27226.1
Deaths	Global	Both	50-69 yea	HIV/AIDS	Rate	1990	5.6489	7.59792	3.99128
Deaths	Global	Both	50-54 yea	Sexually tr	Number	1990	691.2	855.527	472.896
Deaths	Global	Both	50-54 yea	Sexually tr	Rate	1990	0.32513	0.40243	0.22244
Deaths	Global	Both	55-59 yea	Sexually tr	Number	1990	846.807	1106.48	572.362
Deaths	Global	Both	55-59 yea	Sexually tr	Rate	1990	0.45671	0.59676	0.30869
Deaths	Global	Both	60-64 yea	Sexually tr	Number	1990	644.588	790.506	469.339
Deaths	Global	Both	60-64 yea	Sexually tr	Rate	1990	0.40125	0.49209	0.29216
Deaths	Global	Both	65-69 yea	Sexually tr	Number	1990	519.619	629.871	426.549
Deaths	Global	Both	65-69 yea	Sexually tr	Rate	1990	0.42078	0.51006	0.34542
Deaths	Global	Both	50-69 yea	Sexually tr	Number	1990	2702.21	3183.56	1985.48
Deaths	Global	Both	50-69 yea	Sexually tr	Rate	1990	0.39614	0.4667	0.29107
Deaths	Global	Both	50-54 yea	Syphilis	Number	1990	229.846	330.569	124.111
Deaths	Global	Both	50-54 yea	Syphilis	Rate	1990	0.10812	0.1555	0.05838
Deaths	Global	Both	55-59 yea	Syphilis	Number	1990	283.031	461.475	127.97
Deaths	Global	Both	55-59 yea	Syphilis	Rate	1990	0.15265	0.24889	0.06902
Deaths	Global	Both	60-64 yea	Syphilis	Number	1990	248.537	348.147	147.181
Deaths	Global	Both	60-64 yea	Syphilis	Rate	1990	0.15471	0.21672	0.09162
Deaths	Global	Both	65-69 yea	Syphilis	Number	1990	161.644	222.28	121.85
Deaths	Global	Both	65-69 yea	Syphilis	Rate	1990	0.1309	0.18	0.09867
Deaths	Global	Both	50-69 yea	Syphilis	Number	1990	923.057	1270.28	534.823
Deaths	Global	Both	50-69 yea	Syphilis	Rate	1990	0.13532	0.18622	0.0784
Deaths	Global	Both	50-54 yea	Chlamydia	Number	1990	65.8979	81.6133	48.0868
Deaths	Global	Both	50-54 yea	Chlamydia	Rate	1990	0.031	0.03839	0.02262
Deaths	Global	Both	55-59 yea	Chlamydia	Number	1990	84.5434	106.467	59.1216
Deaths	Global	Both	55-59 yea	Chlamydia	Rate	1990	0.0456	0.05742	0.03189
Deaths	Global	Both	60-64 yea	Chlamydia	Number	1990	61.6198	75.7933	44.4975
Deaths	Global	Both	60-64 yea	Chlamydia	Rate	1990	0.03836	0.04718	0.0277
Deaths	Global	Both	65-69 yea	Chlamydia	Number	1990	37.6337	45.3782	29.0174
Deaths	Global	Both	65-69 yea	Chlamydia	Rate	1990	0.03048	0.03675	0.0235
Deaths	Global	Both	50-69 yea	Chlamydia	Number	1990	249.695	295.402	181.955
Deaths	Global	Both	50-69 yea	Chlamydia	Rate	1990	0.0366	0.04331	0.02667
Deaths	Global	Both	50-54 yea	Gonococca	Number	1990	212.693	260.03	154.385
Deaths	Global	Both	50-54 yea	Gonococca	Rate	1990	0.10005	0.12231	0.07262
Deaths	Global	Both	55-59 yea	Gonococca	Number	1990	254.262	314.952	176.777
Deaths	Global	Both	55-59 yea	Gonococca	Rate	1990	0.13713	0.16986	0.09534
Deaths	Global	Both	60-64 yea	Gonococca	Number	1990	184.023	221.298	138.185
Deaths	Global	Both	60-64 yea	Gonococca	Rate	1990	0.11455	0.13776	0.08602
Deaths	Global	Both	65-69 yea	Gonococca	Number	1990	219.617	263.148	174.594
Deaths	Global	Both	65-69 yea	Gonococca	Rate	1990	0.17784	0.21309	0.14138
Deaths	Global	Both	50-69 yea	Gonococca	Number	1990	870.594	1005.48	648.614
Deaths	Global	Both	50-69 yea	Gonococca	Rate	1990	0.12763	0.1474	0.09509
Deaths	Global	Both	50-54 yea	Other sexu	Number	1990	182.764	224.625	127.449
Deaths	Global	Both	50-54 yea	Other sexu	Rate	1990	0.08597	0.10566	0.05995
Deaths	Global	Both	55-59 yea	Other sexu	Number	1990	224.971	280.835	155.765
Deaths	Global	Both	55-59 yea	Other sexu	Rate	1990	0.12133	0.15146	0.08401
Deaths	Global	Both	60-64 yea	Other sexu	Number	1990	150.408	181.645	109.447
Deaths	Global	Both	60-64 yea	Other sexu	Rate	1990	0.09363	0.11307	0.06813
Deaths	Global	Both	65-69 yea	Other sexu	Number	1990	100.724	121.635	81.4871
Deaths	Global	Both	65-69 yea	Other sexu	Rate	1990	0.08157	0.0985	0.06599
Deaths	Global	Both	50-69 yea	Other sexu	Number	1990	658.868	766.4	483.025
Deaths	Global	Both	50-69 yea	Other sexu	Rate	1990	0.09659	0.11235	0.07081
Deaths	Global	Both	50-54 yea	HIV/AIDS	Number	1991	21864.7	29350.2	15333.5
Deaths	Global	Both	50-54 yea	HIV/AIDS	Rate	1991	10.1748	13.6581	7.13545
Deaths	Global	Both	55-59 yea	HIV/AIDS	Number	1991	13679.5	18251.3	9749.35
Deaths	Global	Both	55-59 yea	HIV/AIDS	Rate	1991	7.27802	9.71039	5.18702
Deaths	Global	Both	60-64 yea	HIV/AIDS	Number	1991	8648.81	11490.8	6217.15
Deaths	Global	Both	60-64 yea	HIV/AIDS	Rate	1991	5.27409	7.00712	3.79124
Deaths	Global	Both	65-69 yea	HIV/AIDS	Number	1991	5592.31	7497.82	3997.01
Deaths	Global	Both	65-69 yea	HIV/AIDS	Rate	1991	4.39396	5.89114	3.14051
Deaths	Global	Both	50-69 yea	HIV/AIDS	Number	1991	49785.3	66624.9	35331.6
Deaths	Global	Both	50-69 yea	HIV/AIDS	Rate	1991	7.17256	9.59863	5.09021
Deaths	Global	Both	50-54 yea	Sexually tr	Number	1991	689.868	851.78	475.943
Deaths	Global	Both	50-54 yea	Sexually tr	Rate	1991	0.32103	0.39638	0.22148
Deaths	Global	Both	55-59 yea	Sexually tr	Number	1991	854.53	1121.54	575.15
Deaths	Global	Both	55-59 yea	Sexually tr	Rate	1991	0.45464	0.5967	0.306
Deaths	Global	Both	60-64 yea	Sexually tr	Number	1991	653.614	797.915	467.057
Deaths	Global	Both	60-64 yea	Sexually tr	Rate	1991	0.39858	0.48657	0.28481
Deaths	Global	Both	65-69 yea	Sexually tr	Number	1991	534.08	634.753	430.251
Deaths	Global	Both	65-69 yea	Sexually tr	Rate	1991	0.41963	0.49873	0.33805
Deaths	Global	Both	50-69 yea	Sexually tr	Number	1991	2732.09	3204.27	1989.95
Deaths	Global	Both	50-69 yea	Sexually tr	Rate	1991	0.39361	0.46164	0.28669
Deaths	Global	Both	50-54 yea	Syphilis	Number	1991	234.945	332.17	124.729
Deaths	Global	Both	50-54 yea	Syphilis	Rate	1991	0.10933	0.15458	0.05804
Deaths	Global	Both	55-59 yea	Syphilis	Number	1991	286.156	479.833	129.353
Deaths	Global	Both	55-59 yea	Syphilis	Rate	1991	0.15225	0.25529	0.06882
Deaths	Global	Both	60-64 yea	Syphilis	Number	1991	257.178	354.686	149.867
Deaths	Global	Both	60-64 yea	Syphilis	Rate	1991	0.15683	0.21629	0.09139
Deaths	Global	Both	65-69 yea	Syphilis	Number	1991	168.105	222.71	125.022
Deaths	Global	Both	65-69 yea	Syphilis	Rate	1991	0.13208	0.17499	0.09823
Deaths	Global	Both	50-69 yea	Syphilis	Number	1991	946.383	1300.35	543.699
Deaths	Global	Both	50-69 yea	Syphilis	Rate	1991	0.13635	0.18734	0.07833
Deaths	Global	Both	50-54 yea	Chlamydia	Number	1991	65.0416	79.1377	48.0986
Deaths	Global	Both	50-54 yea	Chlamydia	Rate	1991	0.03027	0.03683	0.02238
Deaths	Global	Both	55-59 yea	Chlamydia	Number	1991	85.4449	108.797	59.6768
Deaths	Global	Both	55-59 yea	Chlamydia	Rate	1991	0.04546	0.05788	0.03175
Deaths	Global	Both	60-64 yea	Chlamydia	Number	1991	61.7654	75.6221	44.7597
Deaths	Global	Both	60-64 yea	Chlamydia	Rate	1991	0.03766	0.04611	0.02729
Deaths	Global	Both	65-69 yea	Chlamydia	Number	1991	38.5915	46.7643	29.3758
Deaths	Global	Both	65-69 yea	Chlamydia	Rate	1991	0.03032	0.03674	0.02308
Deaths	Global	Both	50-69 yea	Chlamydia	Number	1991	250.843	296.01	183.073
Deaths	Global	Both	50-69 yea	Chlamydia	Rate	1991	0.03614	0.04265	0.02638
Deaths	Global	Both	50-54 yea	Gonococca	Number	1991	209.921	251.716	155.517
Deaths	Global	Both	50-54 yea	Gonococca	Rate	1991	0.09769	0.11714	0.07237
Deaths	Global	Both	55-59 yea	Gonococca	Number	1991	256.634	319.74	178.885
Deaths	Global	Both	55-59 yea	Gonococca	Rate	1991	0.13654	0.17011	0.09517
Deaths	Global	Both	60-64 yea	Gonococca	Number	1991	184.464	221.479	139.333
Deaths	Global	Both	60-64 yea	Gonococca	Rate	1991	0.11249	0.13506	0.08497
Deaths	Global	Both	65-69 yea	Gonococca	Number	1991	224.727	269.174	176.651
Deaths	Global	Both	65-69 yea	Gonococca	Rate	1991	0.17657	0.21149	0.1388
Deaths	Global	Both	50-69 yea	Gonococca	Number	1991	875.746	1009.22	649.736
Deaths	Global	Both	50-69 yea	Gonococca	Rate	1991	0.12617	0.1454	0.09361
Deaths	Global	Both	50-54 yea	Other sexu	Number	1991	179.961	220.601	128.367
Deaths	Global	Both	50-54 yea	Other sexu	Rate	1991	0.08374	0.10266	0.05974
Deaths	Global	Both	55-59 yea	Other sexu	Number	1991	226.295	286.159	156.636
Deaths	Global	Both	55-59 yea	Other sexu	Rate	1991	0.1204	0.15225	0.08334
Deaths	Global	Both	60-64 yea	Other sexu	Number	1991	150.207	183.313	108.725
Deaths	Global	Both	60-64 yea	Other sexu	Rate	1991	0.0916	0.11179	0.0663
Deaths	Global	Both	65-69 yea	Other sexu	Number	1991	102.656	123.154	81.6848
Deaths	Global	Both	65-69 yea	Other sexu	Rate	1991	0.08066	0.09676	0.06418
Deaths	Global	Both	50-69 yea	Other sexu	Number	1991	659.119	766.442	468.337
Deaths	Global	Both	50-69 yea	Other sexu	Rate	1991	0.09496	0.11042	0.06747
Deaths	Global	Both	50-54 yea	HIV/AIDS	Number	1994	42485	54769.9	30956.5
Deaths	Global	Both	50-54 yea	HIV/AIDS	Rate	1994	19.4758	25.1074	14.1909
Deaths	Global	Both	55-59 yea	HIV/AIDS	Number	1994	26355.9	33817.7	19443.8
Deaths	Global	Both	55-59 yea	HIV/AIDS	Rate	1994	13.3692	17.1542	9.86296
Deaths	Global	Both	60-64 yea	HIV/AIDS	Number	1994	16534.6	21188.3	12234.2
Deaths	Global	Both	60-64 yea	HIV/AIDS	Rate	1994	9.72419	12.4611	7.19508
Deaths	Global	Both	65-69 yea	HIV/AIDS	Number	1994	10505	13618.7	7705.72
Deaths	Global	Both	65-69 yea	HIV/AIDS	Rate	1994	7.57147	9.81566	5.55391
Deaths	Global	Both	50-69 yea	HIV/AIDS	Number	1994	95880.5	123625	70861.8
Deaths	Global	Both	50-69 yea	HIV/AIDS	Rate	1994	13.242	17.0738	9.7867
Deaths	Global	Both	50-54 yea	Sexually tr	Number	1993	685.277	835.397	465.638
Deaths	Global	Both	50-54 yea	Sexually tr	Rate	1993	0.31564	0.38479	0.21447
Deaths	Global	Both	55-59 yea	Sexually tr	Number	1993	875.88	1148.19	584.468
Deaths	Global	Both	55-59 yea	Sexually tr	Rate	1993	0.45066	0.59078	0.30072
Deaths	Global	Both	60-64 yea	Sexually tr	Number	1993	664.041	802.748	472.082
Deaths	Global	Both	60-64 yea	Sexually tr	Rate	1993	0.39441	0.47679	0.28039
Deaths	Global	Both	65-69 yea	Sexually tr	Number	1993	563.636	659.49	444.689
Deaths	Global	Both	65-69 yea	Sexually tr	Rate	1993	0.41769	0.48873	0.32954
Deaths	Global	Both	50-69 yea	Sexually tr	Number	1993	2788.83	3288.27	2002.83
Deaths	Global	Both	50-69 yea	Sexually tr	Rate	1993	0.39018	0.46005	0.28021
Deaths	Global	Both	50-54 yea	Syphilis	Number	1993	239.543	335.193	125.231
Deaths	Global	Both	50-54 yea	Syphilis	Rate	1993	0.11033	0.15439	0.05768
Deaths	Global	Both	55-59 yea	Syphilis	Number	1993	300.54	485.554	143.378
Deaths	Global	Both	55-59 yea	Syphilis	Rate	1993	0.15464	0.24983	0.07377
Deaths	Global	Both	60-64 yea	Syphilis	Number	1993	264.839	360.629	154.201
Deaths	Global	Both	60-64 yea	Syphilis	Rate	1993	0.1573	0.2142	0.09159
Deaths	Global	Both	65-69 yea	Syphilis	Number	1993	186.441	236.925	138.195
Deaths	Global	Both	65-69 yea	Syphilis	Rate	1993	0.13817	0.17558	0.10241
Deaths	Global	Both	50-69 yea	Syphilis	Number	1993	991.363	1342.88	577.053
Deaths	Global	Both	50-69 yea	Syphilis	Rate	1993	0.1387	0.18788	0.08073
Deaths	Global	Both	50-54 yea	Chlamydia	Number	1993	63.8565	76.9633	46.9638
Deaths	Global	Both	50-54 yea	Chlamydia	Rate	1993	0.02941	0.03545	0.02163
Deaths	Global	Both	55-59 yea	Chlamydia	Number	1993	86.9571	107.114	62.2433
Deaths	Global	Both	55-59 yea	Chlamydia	Rate	1993	0.04474	0.05511	0.03203
Deaths	Global	Both	60-64 yea	Chlamydia	Number	1993	62.4243	76.5783	45.1148
Deaths	Global	Both	60-64 yea	Chlamydia	Rate	1993	0.03708	0.04548	0.0268
Deaths	Global	Both	65-69 yea	Chlamydia	Number	1993	39.8688	47.5724	31.9565
Deaths	Global	Both	65-69 yea	Chlamydia	Rate	1993	0.02955	0.03525	0.02368
Deaths	Global	Both	50-69 yea	Chlamydia	Number	1993	253.107	294.019	188.816
Deaths	Global	Both	50-69 yea	Chlamydia	Rate	1993	0.03541	0.04114	0.02642
Deaths	Global	Both	50-54 yea	Gonococca	Number	1993	206.013	245.835	151.609
Deaths	Global	Both	50-54 yea	Gonococca	Rate	1993	0.09489	0.11323	0.06983
Deaths	Global	Both	55-59 yea	Gonococca	Number	1993	260.542	320.141	183.391
Deaths	Global	Both	55-59 yea	Gonococca	Rate	1993	0.13406	0.16472	0.09436
Deaths	Global	Both	60-64 yea	Gonococca	Number	1993	186.014	222.744	141.15
Deaths	Global	Both	60-64 yea	Gonococca	Rate	1993	0.11048	0.1323	0.08384
Deaths	Global	Both	65-69 yea	Gonococca	Number	1993	232.027	273.995	189.251
Deaths	Global	Both	65-69 yea	Gonococca	Rate	1993	0.17195	0.20305	0.14025
Deaths	Global	Both	50-69 yea	Gonococca	Number	1993	884.597	1011.21	662.376
Deaths	Global	Both	50-69 yea	Gonococca	Rate	1993	0.12376	0.14147	0.09267
Deaths	Global	Both	50-54 yea	Other sexu	Number	1993	175.864	213.073	123.955
Deaths	Global	Both	50-54 yea	Other sexu	Rate	1993	0.081	0.09814	0.05709
Deaths	Global	Both	55-59 yea	Other sexu	Number	1993	227.84	284.991	157.885
Deaths	Global	Both	55-59 yea	Other sexu	Rate	1993	0.11723	0.14664	0.08124
Deaths	Global	Both	60-64 yea	Other sexu	Number	1993	150.764	184.417	111.177
Deaths	Global	Both	60-64 yea	Other sexu	Rate	1993	0.08955	0.10953	0.06603
Deaths	Global	Both	65-69 yea	Other sexu	Number	1993	105.299	125.076	86.1211
Deaths	Global	Both	65-69 yea	Other sexu	Rate	1993	0.07803	0.09269	0.06382
Deaths	Global	Both	50-69 yea	Other sexu	Number	1993	659.767	759.401	474.577
Deaths	Global	Both	50-69 yea	Other sexu	Rate	1993	0.09231	0.10625	0.0664
Deaths	Global	Both	50-54 yea	HIV/AIDS	Number	1992	27746.7	36906.2	19683.9
Deaths	Global	Both	50-54 yea	HIV/AIDS	Rate	1992	12.8293	17.0643	9.10127
Deaths	Global	Both	55-59 yea	HIV/AIDS	Number	1992	17364	23085.7	12469
Deaths	Global	Both	55-59 yea	HIV/AIDS	Rate	1992	9.08111	12.0734	6.52108
Deaths	Global	Both	60-64 yea	HIV/AIDS	Number	1992	10959.8	14583.5	7934.35
Deaths	Global	Both	60-64 yea	HIV/AIDS	Rate	1992	6.58873	8.7672	4.76991
Deaths	Global	Both	65-69 yea	HIV/AIDS	Number	1992	6997.16	9341.08	5026.02
Deaths	Global	Both	65-69 yea	HIV/AIDS	Rate	1992	5.33698	7.12476	3.83352
Deaths	Global	Both	50-69 yea	HIV/AIDS	Number	1992	63067.7	83816	45124.1
Deaths	Global	Both	50-69 yea	HIV/AIDS	Rate	1992	8.94659	11.8899	6.40117
Deaths	Global	Both	50-54 yea	Sexually tr	Number	1992	693.692	848.505	463.031
Deaths	Global	Both	50-54 yea	Sexually tr	Rate	1992	0.32074	0.39232	0.21409
Deaths	Global	Both	55-59 yea	Sexually tr	Number	1992	870.599	1133.16	586.24
Deaths	Global	Both	55-59 yea	Sexually tr	Rate	1992	0.45531	0.59263	0.30659
Deaths	Global	Both	60-64 yea	Sexually tr	Number	1992	661.8	812.224	476.006
Deaths	Global	Both	60-64 yea	Sexually tr	Rate	1992	0.39786	0.48829	0.28616
Deaths	Global	Both	65-69 yea	Sexually tr	Number	1992	547.889	654.187	436.887
Deaths	Global	Both	65-69 yea	Sexually tr	Rate	1992	0.41789	0.49897	0.33323
Deaths	Global	Both	50-69 yea	Sexually tr	Number	1992	2773.98	3261.17	2006.74
Deaths	Global	Both	50-69 yea	Sexually tr	Rate	1992	0.39351	0.46262	0.28467
Deaths	Global	Both	50-54 yea	Syphilis	Number	1992	239.182	339.091	127.593
Deaths	Global	Both	50-54 yea	Syphilis	Rate	1992	0.11059	0.15679	0.059
Deaths	Global	Both	55-59 yea	Syphilis	Number	1992	291.964	475.269	136.455
Deaths	Global	Both	55-59 yea	Syphilis	Rate	1992	0.15269	0.24856	0.07136
Deaths	Global	Both	60-64 yea	Syphilis	Number	1992	262.25	359.915	153.169
Deaths	Global	Both	60-64 yea	Syphilis	Rate	1992	0.15766	0.21637	0.09208
Deaths	Global	Both	65-69 yea	Syphilis	Number	1992	176.051	230.221	130.824
Deaths	Global	Both	65-69 yea	Syphilis	Rate	1992	0.13428	0.1756	0.09978
Deaths	Global	Both	50-69 yea	Syphilis	Number	1992	969.447	1326.31	562.259
Deaths	Global	Both	50-69 yea	Syphilis	Rate	1992	0.13752	0.18815	0.07976
Deaths	Global	Both	50-54 yea	Chlamydia	Number	1992	65.0899	79.4583	47.0365
Deaths	Global	Both	50-54 yea	Chlamydia	Rate	1992	0.0301	0.03674	0.02175
Deaths	Global	Both	55-59 yea	Chlamydia	Number	1992	87.3079	110.796	59.571
Deaths	Global	Both	55-59 yea	Chlamydia	Rate	1992	0.04566	0.05794	0.03115
Deaths	Global	Both	60-64 yea	Chlamydia	Number	1992	62.3592	76.6682	44.9039
Deaths	Global	Both	60-64 yea	Chlamydia	Rate	1992	0.03749	0.04609	0.027
Deaths	Global	Both	65-69 yea	Chlamydia	Number	1992	39.2658	47.3546	30.5162
Deaths	Global	Both	65-69 yea	Chlamydia	Rate	1992	0.02995	0.03612	0.02328
Deaths	Global	Both	50-69 yea	Chlamydia	Number	1992	254.023	299.315	188.623
Deaths	Global	Both	50-69 yea	Chlamydia	Rate	1992	0.03603	0.04246	0.02676
Deaths	Global	Both	50-54 yea	Gonococca	Number	1992	209.966	252.695	150.858
Deaths	Global	Both	50-54 yea	Gonococca	Rate	1992	0.09708	0.11684	0.06975
Deaths	Global	Both	55-59 yea	Gonococca	Number	1992	261.73	323.971	181.276
Deaths	Global	Both	55-59 yea	Gonococca	Rate	1992	0.13688	0.16943	0.0948
Deaths	Global	Both	60-64 yea	Gonococca	Number	1992	185.872	222.956	139.551
Deaths	Global	Both	60-64 yea	Gonococca	Rate	1992	0.11174	0.13403	0.08389
Deaths	Global	Both	65-69 yea	Gonococca	Number	1992	228.454	274.069	182.177
Deaths	Global	Both	65-69 yea	Gonococca	Rate	1992	0.17425	0.20904	0.13895
Deaths	Global	Both	50-69 yea	Gonococca	Number	1992	886.022	1022.14	663.353
Deaths	Global	Both	50-69 yea	Gonococca	Rate	1992	0.12569	0.145	0.0941
Deaths	Global	Both	50-54 yea	Other sexu	Number	1992	179.455	218.261	126.749
Deaths	Global	Both	50-54 yea	Other sexu	Rate	1992	0.08297	0.10092	0.05861
Deaths	Global	Both	55-59 yea	Other sexu	Number	1992	229.597	286.623	160.273
Deaths	Global	Both	55-59 yea	Other sexu	Rate	1992	0.12008	0.1499	0.08382
Deaths	Global	Both	60-64 yea	Other sexu	Number	1992	151.319	184.278	111.565
Deaths	Global	Both	60-64 yea	Other sexu	Rate	1992	0.09097	0.11078	0.06707
Deaths	Global	Both	65-69 yea	Other sexu	Number	1992	104.118	125.708	83.4084
Deaths	Global	Both	65-69 yea	Other sexu	Rate	1992	0.07941	0.09588	0.06362
Deaths	Global	Both	50-69 yea	Other sexu	Number	1992	664.489	775.24	480.646
Deaths	Global	Both	50-69 yea	Other sexu	Rate	1992	0.09426	0.10997	0.06818
Deaths	Global	Both	50-54 yea	HIV/AIDS	Number	1998	73270.4	88266.4	57253
Deaths	Global	Both	50-54 yea	HIV/AIDS	Rate	1998	30.5161	36.7617	23.8451
Deaths	Global	Both	55-59 yea	HIV/AIDS	Number	1998	43870.6	52467.6	34655.8
Deaths	Global	Both	55-59 yea	HIV/AIDS	Rate	1998	21.5456	25.7677	17.02
Deaths	Global	Both	60-64 yea	HIV/AIDS	Number	1998	27413.5	32901.7	21864.5
Deaths	Global	Both	60-64 yea	HIV/AIDS	Rate	1998	15.1311	18.1604	12.0683
Deaths	Global	Both	65-69 yea	HIV/AIDS	Number	1998	17367	21055.1	13814.1
Deaths	Global	Both	65-69 yea	HIV/AIDS	Rate	1998	11.6343	14.105	9.25419
Deaths	Global	Both	50-69 yea	HIV/AIDS	Number	1998	161922	194716	127749
Deaths	Global	Both	50-69 yea	HIV/AIDS	Rate	1998	20.9155	25.1516	16.5014
Deaths	Global	Both	50-54 yea	Sexually tr	Number	1994	685.393	832.871	451.727
Deaths	Global	Both	50-54 yea	Sexually tr	Rate	1994	0.3142	0.3818	0.20708
Deaths	Global	Both	55-59 yea	Sexually tr	Number	1994	886.646	1161.51	577.599
Deaths	Global	Both	55-59 yea	Sexually tr	Rate	1994	0.44975	0.58918	0.29299
Deaths	Global	Both	60-64 yea	Sexually tr	Number	1994	663.096	802.307	468.831
Deaths	Global	Both	60-64 yea	Sexually tr	Rate	1994	0.38997	0.47185	0.27573
Deaths	Global	Both	65-69 yea	Sexually tr	Number	1994	575.943	673.964	455.016
Deaths	Global	Both	65-69 yea	Sexually tr	Rate	1994	0.41511	0.48576	0.32795
Deaths	Global	Both	50-69 yea	Sexually tr	Number	1994	2811.08	3307.58	2007.16
Deaths	Global	Both	50-69 yea	Sexually tr	Rate	1994	0.38824	0.45681	0.27721
Deaths	Global	Both	50-54 yea	Syphilis	Number	1994	239.977	345.825	121.266
Deaths	Global	Both	50-54 yea	Syphilis	Rate	1994	0.11001	0.15853	0.05559
Deaths	Global	Both	55-59 yea	Syphilis	Number	1994	312.974	497.305	153.71
Deaths	Global	Both	55-59 yea	Syphilis	Rate	1994	0.15876	0.25226	0.07797
Deaths	Global	Both	60-64 yea	Syphilis	Number	1994	264.125	360.022	155.522
Deaths	Global	Both	60-64 yea	Syphilis	Rate	1994	0.15533	0.21173	0.09146
Deaths	Global	Both	65-69 yea	Syphilis	Number	1994	195.611	245.898	142.764
Deaths	Global	Both	65-69 yea	Syphilis	Rate	1994	0.14099	0.17723	0.1029
Deaths	Global	Both	50-69 yea	Syphilis	Number	1994	1012.69	1371.71	590.537
Deaths	Global	Both	50-69 yea	Syphilis	Rate	1994	0.13986	0.18945	0.08156
Deaths	Global	Both	50-54 yea	Chlamydia	Number	1994	63.9161	76.9966	47.569
Deaths	Global	Both	50-54 yea	Chlamydia	Rate	1994	0.0293	0.0353	0.02181
Deaths	Global	Both	55-59 yea	Chlamydia	Number	1994	86.8011	107.506	62.5172
Deaths	Global	Both	55-59 yea	Chlamydia	Rate	1994	0.04403	0.05453	0.03171
Deaths	Global	Both	60-64 yea	Chlamydia	Number	1994	62.4643	76.2487	45.1428
Deaths	Global	Both	60-64 yea	Chlamydia	Rate	1994	0.03674	0.04484	0.02655
Deaths	Global	Both	65-69 yea	Chlamydia	Number	1994	40.2191	47.479	32.2533
Deaths	Global	Both	65-69 yea	Chlamydia	Rate	1994	0.02899	0.03422	0.02325
Deaths	Global	Both	50-69 yea	Chlamydia	Number	1994	253.401	292.181	189.964
Deaths	Global	Both	50-69 yea	Chlamydia	Rate	1994	0.035	0.04035	0.02624
Deaths	Global	Both	50-54 yea	Gonococca	Number	1994	206.047	243.94	151.176
Deaths	Global	Both	50-54 yea	Gonococca	Rate	1994	0.09446	0.11183	0.0693
Deaths	Global	Both	55-59 yea	Gonococca	Number	1994	260.018	317.906	184.899
Deaths	Global	Both	55-59 yea	Gonococca	Rate	1994	0.1319	0.16126	0.09379
Deaths	Global	Both	60-64 yea	Gonococca	Number	1994	185.695	223.1	139.681
Deaths	Global	Both	60-64 yea	Gonococca	Rate	1994	0.10921	0.13121	0.08215
Deaths	Global	Both	65-69 yea	Gonococca	Number	1994	234.102	276.323	191.375
Deaths	Global	Both	65-69 yea	Gonococca	Rate	1994	0.16873	0.19916	0.13793
Deaths	Global	Both	50-69 yea	Gonococca	Number	1994	885.862	1006.95	659.24
Deaths	Global	Both	50-69 yea	Gonococca	Rate	1994	0.12235	0.13907	0.09105
Deaths	Global	Both	50-54 yea	Other sexu	Number	1994	175.453	212.525	123.153
Deaths	Global	Both	50-54 yea	Other sexu	Rate	1994	0.08043	0.09742	0.05646
Deaths	Global	Both	55-59 yea	Other sexu	Number	1994	226.852	281.279	157.63
Deaths	Global	Both	55-59 yea	Other sexu	Rate	1994	0.11507	0.14268	0.07996
Deaths	Global	Both	60-64 yea	Other sexu	Number	1994	150.812	183.736	109.816
Deaths	Global	Both	60-64 yea	Other sexu	Rate	1994	0.08869	0.10806	0.06458
Deaths	Global	Both	65-69 yea	Other sexu	Number	1994	106.01	127.076	86.3431
Deaths	Global	Both	65-69 yea	Other sexu	Rate	1994	0.07641	0.09159	0.06223
Deaths	Global	Both	50-69 yea	Other sexu	Number	1994	659.128	757.521	479.674
Deaths	Global	Both	50-69 yea	Other sexu	Rate	1994	0.09103	0.10462	0.06625
Deaths	Global	Both	50-54 yea	HIV/AIDS	Number	1993	34728.6	45648.9	24975.8
Deaths	Global	Both	50-54 yea	HIV/AIDS	Rate	1993	15.9961	21.0261	11.504
Deaths	Global	Both	55-59 yea	HIV/AIDS	Number	1993	21545.4	28346.3	15574.7
Deaths	Global	Both	55-59 yea	HIV/AIDS	Rate	1993	11.0857	14.5849	8.01357
Deaths	Global	Both	60-64 yea	HIV/AIDS	Number	1993	13551.4	17725.7	9868.36
Deaths	Global	Both	60-64 yea	HIV/AIDS	Rate	1993	8.04884	10.5282	5.86132
Deaths	Global	Both	65-69 yea	HIV/AIDS	Number	1993	8687.5	11456.8	6266.52
Deaths	Global	Both	65-69 yea	HIV/AIDS	Rate	1993	6.43804	8.49024	4.64392
Deaths	Global	Both	50-69 yea	HIV/AIDS	Number	1993	78512.9	103365	56835.9
Deaths	Global	Both	50-69 yea	HIV/AIDS	Rate	1993	10.9844	14.4615	7.95169
Deaths	Global	Both	50-54 yea	HIV/AIDS	Number	1995	50428.7	63737.2	37282
Deaths	Global	Both	50-54 yea	HIV/AIDS	Rate	1995	23.017	29.0913	17.0164
Deaths	Global	Both	55-59 yea	HIV/AIDS	Number	1995	31217.8	39213.3	23481.4
Deaths	Global	Both	55-59 yea	HIV/AIDS	Rate	1995	15.6256	19.6276	11.7533
Deaths	Global	Both	60-64 yea	HIV/AIDS	Number	1995	19522.7	24571.5	14755.7
Deaths	Global	Both	60-64 yea	HIV/AIDS	Rate	1995	11.3505	14.2859	8.57901
Deaths	Global	Both	65-69 yea	HIV/AIDS	Number	1995	12400.2	15739.9	9293.68
Deaths	Global	Both	65-69 yea	HIV/AIDS	Rate	1995	8.72855	11.0794	6.54185
Deaths	Global	Both	50-69 yea	HIV/AIDS	Number	1995	113569	143538	85312.7
Deaths	Global	Both	50-69 yea	HIV/AIDS	Rate	1995	15.495	19.5837	11.6397
Deaths	Global	Both	50-54 yea	Sexually tr	Number	2000	617.847	759.405	442.612
Deaths	Global	Both	50-54 yea	Sexually tr	Rate	2000	0.23561	0.28959	0.16878
Deaths	Global	Both	55-59 yea	Sexually tr	Number	2000	780.396	1019.89	536.958
Deaths	Global	Both	55-59 yea	Sexually tr	Rate	2000	0.3791	0.49544	0.26084
Deaths	Global	Both	60-64 yea	Sexually tr	Number	2000	604.338	749.946	446.543
Deaths	Global	Both	60-64 yea	Sexually tr	Rate	2000	0.32325	0.40113	0.23885
Deaths	Global	Both	65-69 yea	Sexually tr	Number	2000	531.742	637.019	428.032
Deaths	Global	Both	65-69 yea	Sexually tr	Rate	2000	0.34714	0.41587	0.27943
Deaths	Global	Both	50-69 yea	Sexually tr	Number	2000	2534.32	3008.5	1895.35
Deaths	Global	Both	50-69 yea	Sexually tr	Rate	2000	0.31357	0.37224	0.23451
Deaths	Global	Both	50-54 yea	Syphilis	Number	2000	205.018	297.778	114.709
Deaths	Global	Both	50-54 yea	Syphilis	Rate	2000	0.07818	0.11355	0.04374
Deaths	Global	Both	55-59 yea	Syphilis	Number	2000	249.396	416.765	116.422
Deaths	Global	Both	55-59 yea	Syphilis	Rate	2000	0.12115	0.20246	0.05656
Deaths	Global	Both	60-64 yea	Syphilis	Number	2000	235.316	333.211	144.974
Deaths	Global	Both	60-64 yea	Syphilis	Rate	2000	0.12587	0.17823	0.07754
Deaths	Global	Both	65-69 yea	Syphilis	Number	2000	155.169	209.1	120.583
Deaths	Global	Both	65-69 yea	Syphilis	Rate	2000	0.1013	0.13651	0.07872
Deaths	Global	Both	50-69 yea	Syphilis	Number	2000	844.899	1164.75	516.344
Deaths	Global	Both	50-69 yea	Syphilis	Rate	2000	0.10454	0.14411	0.06389
Deaths	Global	Both	50-54 yea	Chlamydia	Number	2000	59.7339	70.7035	48.3048
Deaths	Global	Both	50-54 yea	Chlamydia	Rate	2000	0.02278	0.02696	0.01842
Deaths	Global	Both	55-59 yea	Chlamydia	Number	2000	80.6844	99.868	58.5296
Deaths	Global	Both	55-59 yea	Chlamydia	Rate	2000	0.03919	0.04851	0.02843
Deaths	Global	Both	60-64 yea	Chlamydia	Number	2000	58.2343	69.9287	44.8445
Deaths	Global	Both	60-64 yea	Chlamydia	Rate	2000	0.03115	0.0374	0.02399
Deaths	Global	Both	65-69 yea	Chlamydia	Number	2000	40.4636	48.2566	31.6856
Deaths	Global	Both	65-69 yea	Chlamydia	Rate	2000	0.02642	0.0315	0.02069
Deaths	Global	Both	50-69 yea	Chlamydia	Number	2000	239.116	273.602	185.863
Deaths	Global	Both	50-69 yea	Chlamydia	Rate	2000	0.02959	0.03385	0.023
Deaths	Global	Both	50-54 yea	Gonococca	Number	2000	191.917	226.898	155.012
Deaths	Global	Both	50-54 yea	Gonococca	Rate	2000	0.07319	0.08652	0.05911
Deaths	Global	Both	55-59 yea	Gonococca	Number	2000	240.856	296.141	175.967
Deaths	Global	Both	55-59 yea	Gonococca	Rate	2000	0.117	0.14386	0.08548
Deaths	Global	Both	60-64 yea	Gonococca	Number	2000	172.028	204.4	136.894
Deaths	Global	Both	60-64 yea	Gonococca	Rate	2000	0.09202	0.10933	0.07322
Deaths	Global	Both	65-69 yea	Gonococca	Number	2000	231.451	274.634	183.206
Deaths	Global	Both	65-69 yea	Gonococca	Rate	2000	0.1511	0.17929	0.1196
Deaths	Global	Both	50-69 yea	Gonococca	Number	2000	836.252	946.889	651.21
Deaths	Global	Both	50-69 yea	Gonococca	Rate	2000	0.10347	0.11716	0.08057
Deaths	Global	Both	50-54 yea	Other sexu	Number	2000	161.179	195.895	120.628
Deaths	Global	Both	50-54 yea	Other sexu	Rate	2000	0.06146	0.0747	0.046
Deaths	Global	Both	55-59 yea	Other sexu	Number	2000	209.46	265.707	149.428
Deaths	Global	Both	55-59 yea	Other sexu	Rate	2000	0.10175	0.12908	0.07259
Deaths	Global	Both	60-64 yea	Other sexu	Number	2000	138.76	168.884	107.629
Deaths	Global	Both	60-64 yea	Other sexu	Rate	2000	0.07422	0.09033	0.05757
Deaths	Global	Both	65-69 yea	Other sexu	Number	2000	104.658	124.684	83.6168
Deaths	Global	Both	65-69 yea	Other sexu	Rate	2000	0.06832	0.0814	0.05459
Deaths	Global	Both	50-69 yea	Other sexu	Number	2000	614.056	714.903	462.028
Deaths	Global	Both	50-69 yea	Other sexu	Rate	2000	0.07598	0.08845	0.05717
Deaths	Global	Both	50-54 yea	Sexually tr	Number	1996	660.457	810.561	445.197
Deaths	Global	Both	50-54 yea	Sexually tr	Rate	1996	0.29703	0.36453	0.20022
Deaths	Global	Both	55-59 yea	Sexually tr	Number	1996	868.928	1125.2	561.609
Deaths	Global	Both	55-59 yea	Sexually tr	Rate	1996	0.43075	0.55779	0.2784
Deaths	Global	Both	60-64 yea	Sexually tr	Number	1996	631.871	773.807	452.124
Deaths	Global	Both	60-64 yea	Sexually tr	Rate	1996	0.36195	0.44325	0.25899
Deaths	Global	Both	65-69 yea	Sexually tr	Number	1996	564.336	669.426	449.038
Deaths	Global	Both	65-69 yea	Sexually tr	Rate	1996	0.38897	0.4614	0.3095
Deaths	Global	Both	50-69 yea	Sexually tr	Number	1996	2725.59	3236.68	1963.29
Deaths	Global	Both	50-69 yea	Sexually tr	Rate	1996	0.36647	0.43519	0.26397
Deaths	Global	Both	50-54 yea	Syphilis	Number	1996	222.416	327.037	117.898
Deaths	Global	Both	50-54 yea	Syphilis	Rate	1996	0.10003	0.14708	0.05302
Deaths	Global	Both	55-59 yea	Syphilis	Number	1996	310.898	489.648	154.938
Deaths	Global	Both	55-59 yea	Syphilis	Rate	1996	0.15412	0.24273	0.07681
Deaths	Global	Both	60-64 yea	Syphilis	Number	1996	251.844	344.355	151.607
Deaths	Global	Both	60-64 yea	Syphilis	Rate	1996	0.14426	0.19725	0.08684
Deaths	Global	Both	65-69 yea	Syphilis	Number	1996	193.448	250.735	142.041
Deaths	Global	Both	65-69 yea	Syphilis	Rate	1996	0.13333	0.17282	0.0979
Deaths	Global	Both	50-69 yea	Syphilis	Number	1996	978.607	1337.46	588.188
Deaths	Global	Both	50-69 yea	Syphilis	Rate	1996	0.13158	0.17983	0.07909
Deaths	Global	Both	50-54 yea	Chlamydia	Number	1996	63.2616	76.7425	46.6416
Deaths	Global	Both	50-54 yea	Chlamydia	Rate	1996	0.02845	0.03451	0.02098
Deaths	Global	Both	55-59 yea	Chlamydia	Number	1996	84.7064	105.168	61.1292
Deaths	Global	Both	55-59 yea	Chlamydia	Rate	1996	0.04199	0.05213	0.0303
Deaths	Global	Both	60-64 yea	Chlamydia	Number	1996	59.5513	72.4427	43.3715
Deaths	Global	Both	60-64 yea	Chlamydia	Rate	1996	0.03411	0.0415	0.02484
Deaths	Global	Both	65-69 yea	Chlamydia	Number	1996	39.45	46.8455	31.9274
Deaths	Global	Both	65-69 yea	Chlamydia	Rate	1996	0.02719	0.03229	0.02201
Deaths	Global	Both	50-69 yea	Chlamydia	Number	1996	246.969	283.627	182.939
Deaths	Global	Both	50-69 yea	Chlamydia	Rate	1996	0.03321	0.03814	0.0246
Deaths	Global	Both	50-54 yea	Sexually tr	Number	2004	594.045	723.175	450.327
Deaths	Global	Both	50-54 yea	Sexually tr	Rate	2004	0.19522	0.23765	0.14799
Deaths	Global	Both	55-59 yea	Sexually tr	Number	2004	713.77	928.679	511.961
Deaths	Global	Both	55-59 yea	Sexually tr	Rate	2004	0.29802	0.38775	0.21376
Deaths	Global	Both	60-64 yea	Sexually tr	Number	2004	504.976	623.062	391.894
Deaths	Global	Both	60-64 yea	Sexually tr	Rate	2004	0.26123	0.32231	0.20273
Deaths	Global	Both	65-69 yea	Sexually tr	Number	2004	485.636	585.62	408.211
Deaths	Global	Both	65-69 yea	Sexually tr	Rate	2004	0.2931	0.35344	0.24637
Deaths	Global	Both	50-69 yea	Sexually tr	Number	2004	2298.43	2721.47	1810.71
Deaths	Global	Both	50-69 yea	Sexually tr	Rate	2004	0.25459	0.30145	0.20057
Deaths	Global	Both	50-54 yea	Syphilis	Number	2004	174.509	252.788	97.9515
Deaths	Global	Both	50-54 yea	Syphilis	Rate	2004	0.05735	0.08307	0.03219
Deaths	Global	Both	55-59 yea	Syphilis	Number	2004	201.799	337.12	86.9054
Deaths	Global	Both	55-59 yea	Syphilis	Rate	2004	0.08426	0.14076	0.03629
Deaths	Global	Both	60-64 yea	Syphilis	Number	2004	163.785	241.913	97.8709
Deaths	Global	Both	60-64 yea	Syphilis	Rate	2004	0.08473	0.12514	0.05063
Deaths	Global	Both	65-69 yea	Syphilis	Number	2004	115.487	167.486	91.2508
Deaths	Global	Both	65-69 yea	Syphilis	Rate	2004	0.0697	0.10108	0.05507
Deaths	Global	Both	50-69 yea	Syphilis	Number	2004	655.58	924.717	393.283
Deaths	Global	Both	50-69 yea	Syphilis	Rate	2004	0.07262	0.10243	0.04356
Deaths	Global	Both	50-54 yea	Chlamydia	Number	2004	61.0028	70.9795	50.127
Deaths	Global	Both	50-54 yea	Chlamydia	Rate	2004	0.02005	0.02333	0.01647
Deaths	Global	Both	55-59 yea	Chlamydia	Number	2004	78.3318	95.9609	58.3577
Deaths	Global	Both	55-59 yea	Chlamydia	Rate	2004	0.03271	0.04007	0.02437
Deaths	Global	Both	60-64 yea	Chlamydia	Number	2004	53.9497	63.9583	41.6959
Deaths	Global	Both	60-64 yea	Chlamydia	Rate	2004	0.02791	0.03309	0.02157
Deaths	Global	Both	65-69 yea	Chlamydia	Number	2004	39.8929	47.6446	30.7156
Deaths	Global	Both	65-69 yea	Chlamydia	Rate	2004	0.02408	0.02876	0.01854
Deaths	Global	Both	50-69 yea	Chlamydia	Number	2004	233.177	267.638	184.459
Deaths	Global	Both	50-69 yea	Chlamydia	Rate	2004	0.02583	0.02965	0.02043
Deaths	Global	Both	50-54 yea	Gonococca	Number	2004	195.548	225.822	158.898
Deaths	Global	Both	50-54 yea	Gonococca	Rate	2004	0.06426	0.07421	0.05222
Deaths	Global	Both	55-59 yea	Gonococca	Number	2004	232.986	282.575	173.726
Deaths	Global	Both	55-59 yea	Gonococca	Rate	2004	0.09728	0.11798	0.07254
Deaths	Global	Both	60-64 yea	Gonococca	Number	2004	157.472	185.633	126.897
Deaths	Global	Both	60-64 yea	Gonococca	Rate	2004	0.08146	0.09603	0.06564
Deaths	Global	Both	65-69 yea	Gonococca	Number	2004	227.567	267.176	183.493
Deaths	Global	Both	65-69 yea	Gonococca	Rate	2004	0.13735	0.16125	0.11074
Deaths	Global	Both	50-69 yea	Gonococca	Number	2004	813.573	932.428	646.778
Deaths	Global	Both	50-69 yea	Gonococca	Rate	2004	0.09012	0.10328	0.07164
Deaths	Global	Both	50-54 yea	Other sexu	Number	2004	162.984	194.943	125.659
Deaths	Global	Both	50-54 yea	Other sexu	Rate	2004	0.05356	0.06406	0.04129
Deaths	Global	Both	55-59 yea	Other sexu	Number	2004	200.653	248.873	145.958
Deaths	Global	Both	55-59 yea	Other sexu	Rate	2004	0.08378	0.10391	0.06094
Deaths	Global	Both	60-64 yea	Other sexu	Number	2004	129.769	156.254	101.035
Deaths	Global	Both	60-64 yea	Other sexu	Rate	2004	0.06713	0.08083	0.05227
Deaths	Global	Both	65-69 yea	Other sexu	Number	2004	102.689	122.889	84.0534
Deaths	Global	Both	65-69 yea	Other sexu	Rate	2004	0.06198	0.07417	0.05073
Deaths	Global	Both	50-69 yea	Other sexu	Number	2004	596.096	690.301	458.953
Deaths	Global	Both	50-69 yea	Other sexu	Rate	2004	0.06603	0.07646	0.05084
Deaths	Global	Both	50-54 yea	HIV/AIDS	Number	2002	109363	122670	92945.1
Deaths	Global	Both	50-54 yea	HIV/AIDS	Rate	2002	38.3919	43.0633	32.6284
Deaths	Global	Both	55-59 yea	HIV/AIDS	Number	2002	60511.1	67072.2	53058.6
Deaths	Global	Both	55-59 yea	HIV/AIDS	Rate	2002	27.7821	30.7945	24.3605
Deaths	Global	Both	60-64 yea	HIV/AIDS	Number	2002	36602.5	40572.9	32286.4
Deaths	Global	Both	60-64 yea	HIV/AIDS	Rate	2002	19.2084	21.2921	16.9434
Deaths	Global	Both	65-69 yea	HIV/AIDS	Number	2002	22725.2	25482.5	19990.9
Deaths	Global	Both	65-69 yea	HIV/AIDS	Rate	2002	14.2639	15.9945	12.5476
Deaths	Global	Both	50-69 yea	HIV/AIDS	Number	2002	229202	255507	199477
Deaths	Global	Both	50-69 yea	HIV/AIDS	Rate	2002	26.8846	29.9701	23.398
Deaths	Global	Both	50-54 yea	HIV/AIDS	Number	1996	57785	71855.2	43531.1
Deaths	Global	Both	50-54 yea	HIV/AIDS	Rate	1996	25.9876	32.3154	19.5772
Deaths	Global	Both	55-59 yea	HIV/AIDS	Number	1996	35448.9	43663.1	27090.5
Deaths	Global	Both	55-59 yea	HIV/AIDS	Rate	1996	17.5728	21.6447	13.4293
Deaths	Global	Both	60-64 yea	HIV/AIDS	Number	1996	22214.2	27501.6	17056.7
Deaths	Global	Both	60-64 yea	HIV/AIDS	Rate	1996	12.7248	15.7535	9.77042
Deaths	Global	Both	65-69 yea	HIV/AIDS	Number	1996	14143.9	17646.2	10755
Deaths	Global	Both	65-69 yea	HIV/AIDS	Rate	1996	9.74872	12.1627	7.4129
Deaths	Global	Both	50-69 yea	HIV/AIDS	Number	1996	129592	160863	98283
Deaths	Global	Both	50-69 yea	HIV/AIDS	Rate	1996	17.4243	21.6289	13.2147
Deaths	Global	Both	50-54 yea	Sexually tr	Number	1997	656.399	821.197	444.773
Deaths	Global	Both	50-54 yea	Sexually tr	Rate	1997	0.28457	0.35602	0.19282
Deaths	Global	Both	55-59 yea	Sexually tr	Number	1997	857.365	1115.75	566.834
Deaths	Global	Both	55-59 yea	Sexually tr	Rate	1997	0.42265	0.55002	0.27943
Deaths	Global	Both	60-64 yea	Sexually tr	Number	1997	634.96	786.43	450.675
Deaths	Global	Both	60-64 yea	Sexually tr	Rate	1997	0.35693	0.44208	0.25334
Deaths	Global	Both	65-69 yea	Sexually tr	Number	1997	560.865	666.638	450.757
Deaths	Global	Both	65-69 yea	Sexually tr	Rate	1997	0.3808	0.45261	0.30604
Deaths	Global	Both	50-69 yea	Sexually tr	Number	1997	2709.59	3217.8	1966.37
Deaths	Global	Both	50-69 yea	Sexually tr	Rate	1997	0.35714	0.42412	0.25918
Deaths	Global	Both	50-54 yea	Syphilis	Number	1997	215.704	322.15	116.985
Deaths	Global	Both	50-54 yea	Syphilis	Rate	1997	0.09351	0.13966	0.05072
Deaths	Global	Both	55-59 yea	Syphilis	Number	1997	296.67	482.456	147.767
Deaths	Global	Both	55-59 yea	Syphilis	Rate	1997	0.14625	0.23783	0.07284
Deaths	Global	Both	60-64 yea	Syphilis	Number	1997	248.67	345.987	149.208
Deaths	Global	Both	60-64 yea	Syphilis	Rate	1997	0.13979	0.19449	0.08387
Deaths	Global	Both	65-69 yea	Syphilis	Number	1997	183.677	240.255	135.867
Deaths	Global	Both	65-69 yea	Syphilis	Rate	1997	0.12471	0.16312	0.09225
Deaths	Global	Both	50-69 yea	Syphilis	Number	1997	944.721	1316.38	571.427
Deaths	Global	Both	50-69 yea	Syphilis	Rate	1997	0.12452	0.1735	0.07532
Deaths	Global	Both	50-54 yea	Chlamydia	Number	1997	63.8889	76.9788	47.5117
Deaths	Global	Both	50-54 yea	Chlamydia	Rate	1997	0.0277	0.03337	0.0206
Deaths	Global	Both	55-59 yea	Chlamydia	Number	1997	85.3429	105.848	60.9892
Deaths	Global	Both	55-59 yea	Chlamydia	Rate	1997	0.04207	0.05218	0.03007
Deaths	Global	Both	60-64 yea	Chlamydia	Number	1997	60.8035	74.0307	44.7267
Deaths	Global	Both	60-64 yea	Chlamydia	Rate	1997	0.03418	0.04162	0.02514
Deaths	Global	Both	65-69 yea	Chlamydia	Number	1997	40.3284	47.8911	31.8072
Deaths	Global	Both	65-69 yea	Chlamydia	Rate	1997	0.02738	0.03252	0.0216
Deaths	Global	Both	50-69 yea	Chlamydia	Number	1997	250.364	287.03	183.655
Deaths	Global	Both	50-69 yea	Chlamydia	Rate	1997	0.033	0.03783	0.02421
Deaths	Global	Both	50-54 yea	Gonococca	Number	1997	204.856	244.672	153.196
Deaths	Global	Both	50-54 yea	Gonococca	Rate	1997	0.08881	0.10607	0.06642
Deaths	Global	Both	55-59 yea	Gonococca	Number	1997	254.749	311.444	179.724
Deaths	Global	Both	55-59 yea	Gonococca	Rate	1997	0.12558	0.15353	0.0886
Deaths	Global	Both	60-64 yea	Gonococca	Number	1997	179.243	214.186	136.471
Deaths	Global	Both	60-64 yea	Gonococca	Rate	1997	0.10076	0.1204	0.07672
Deaths	Global	Both	65-69 yea	Gonococca	Number	1997	232.263	273.62	188.852
Deaths	Global	Both	65-69 yea	Gonococca	Rate	1997	0.15769	0.18577	0.12822
Deaths	Global	Both	50-69 yea	Gonococca	Number	1997	871.112	987.624	665.212
Deaths	Global	Both	50-69 yea	Gonococca	Rate	1997	0.11482	0.13017	0.08768
Deaths	Global	Both	50-54 yea	Other sexu	Number	1997	171.95	213.08	122.771
Deaths	Global	Both	50-54 yea	Other sexu	Rate	1997	0.07455	0.09238	0.05323
Deaths	Global	Both	55-59 yea	Other sexu	Number	1997	220.603	277.19	154.793
Deaths	Global	Both	55-59 yea	Other sexu	Rate	1997	0.10875	0.13664	0.07631
Deaths	Global	Both	60-64 yea	Other sexu	Number	1997	146.243	177.905	109.635
Deaths	Global	Both	60-64 yea	Other sexu	Rate	1997	0.08221	0.10001	0.06163
Deaths	Global	Both	65-69 yea	Other sexu	Number	1997	104.596	124.319	85.0158
Deaths	Global	Both	65-69 yea	Other sexu	Rate	1997	0.07102	0.08441	0.05772
Deaths	Global	Both	50-69 yea	Other sexu	Number	1997	643.392	752.241	478.691
Deaths	Global	Both	50-69 yea	Other sexu	Rate	1997	0.0848	0.09915	0.06309
Deaths	Global	Both	50-54 yea	HIV/AIDS	Number	1997	64931.2	79625.8	49594.4
Deaths	Global	Both	50-54 yea	HIV/AIDS	Rate	1997	28.1498	34.5204	21.5008
Deaths	Global	Both	55-59 yea	HIV/AIDS	Number	1997	39379.8	47921.8	30491.9
Deaths	Global	Both	55-59 yea	HIV/AIDS	Rate	1997	19.4126	23.6235	15.0313
Deaths	Global	Both	60-64 yea	HIV/AIDS	Number	1997	24753.8	30166.4	19332.4
Deaths	Global	Both	60-64 yea	HIV/AIDS	Rate	1997	13.9149	16.9575	10.8673
Deaths	Global	Both	65-69 yea	HIV/AIDS	Number	1997	15648.6	19339.5	12144.6
Deaths	Global	Both	65-69 yea	HIV/AIDS	Rate	1997	10.6246	13.1305	8.24555
Deaths	Global	Both	50-69 yea	HIV/AIDS	Number	1997	144713	176951	111458
Deaths	Global	Both	50-69 yea	HIV/AIDS	Rate	1997	19.0739	23.3229	14.6906
Deaths	Global	Both	50-54 yea	HIV/AIDS	Number	2006	121777	130640	113089
Deaths	Global	Both	50-54 yea	HIV/AIDS	Rate	2006	37.5426	40.275	34.8642
Deaths	Global	Both	55-59 yea	HIV/AIDS	Number	2006	66318.9	71389.2	61696.9
Deaths	Global	Both	55-59 yea	HIV/AIDS	Rate	2006	25.2594	27.1906	23.499
Deaths	Global	Both	60-64 yea	HIV/AIDS	Number	2006	37576.4	40638.6	34728.7
Deaths	Global	Both	60-64 yea	HIV/AIDS	Rate	2006	18.895	20.4348	17.463
Deaths	Global	Both	65-69 yea	HIV/AIDS	Number	2006	22796.8	24712.3	21083.8
Deaths	Global	Both	65-69 yea	HIV/AIDS	Rate	2006	13.333	14.4533	12.3311
Deaths	Global	Both	50-69 yea	HIV/AIDS	Number	2006	248469	266163	231824
Deaths	Global	Both	50-69 yea	HIV/AIDS	Rate	2006	25.9695	27.8189	24.2298
Deaths	Global	Both	50-54 yea	Gonococca	Number	1996	203.212	244.668	149.992
Deaths	Global	Both	50-54 yea	Gonococca	Rate	1996	0.09139	0.11003	0.06746
Deaths	Global	Both	55-59 yea	Gonococca	Number	1996	253.314	309.847	180.426
Deaths	Global	Both	55-59 yea	Gonococca	Rate	1996	0.12557	0.1536	0.08944
Deaths	Global	Both	60-64 yea	Gonococca	Number	1996	176.487	210.349	135.367
Deaths	Global	Both	60-64 yea	Gonococca	Rate	1996	0.1011	0.12049	0.07754

Deaths	Global	Both	50-54 yea	Chlamydia	Rate	1995	0.02838	0.03425	0.02105
Deaths	Global	Both	55-59 yea	Chlamydia	Number	1995	84.7254	105.165	61.845
Deaths	Global	Both	55-59 yea	Chlamydia	Rate	1995	0.04241	0.05264	0.03096
Deaths	Global	Both	60-64 yea	Chlamydia	Number	1995	60.3417	73.469	44.3745
Deaths	Global	Both	60-64 yea	Chlamydia	Rate	1995	0.03508	0.04271	0.0258
Deaths	Global	Both	65-69 yea	Chlamydia	Number	1995	39.574	47.2492	31.7978
Deaths	Global	Both	65-69 yea	Chlamydia	Rate	1995	0.02786	0.03326	0.02238
Deaths	Global	Both	50-69 yea	Chlamydia	Number	1995	246.83	284.102	188.312
Deaths	Global	Both	50-69 yea	Chlamydia	Rate	1995	0.03368	0.03876	0.02569
Deaths	Global	Both	50-54 yea	Gonococca	Number	1995	200.444	239.344	146.705
Deaths	Global	Both	50-54 yea	Gonococca	Rate	1995	0.09149	0.10924	0.06696
Deaths	Global	Both	55-59 yea	Gonococca	Number	1995	253.877	314.026	181.887
Deaths	Global	Both	55-59 yea	Gonococca	Rate	1995	0.12707	0.15718	0.09104
Deaths	Global	Both	60-64 yea	Gonococca	Number	1995	179.518	212.946	137.541
Deaths	Global	Both	60-64 yea	Gonococca	Rate	1995	0.10437	0.12381	0.07997
Deaths	Global	Both	65-69 yea	Gonococca	Number	1995	230.103	272.07	187.82
Deaths	Global	Both	65-69 yea	Gonococca	Rate	1995	0.16197	0.19151	0.13221
Deaths	Global	Both	50-69 yea	Gonococca	Number	1995	863.942	980.125	664.793
Deaths	Global	Both	50-69 yea	Gonococca	Rate	1995	0.11787	0.13372	0.0907
Deaths	Global	Both	50-54 yea	Other sexu	Number	1995	170.777	206.069	122.43
Deaths	Global	Both	50-54 yea	Other sexu	Rate	1995	0.07795	0.09406	0.05588
Deaths	Global	Both	55-59 yea	Other sexu	Number	1995	221.41	276.755	157.014
Deaths	Global	Both	55-59 yea	Other sexu	Rate	1995	0.11082	0.13853	0.07859
Deaths	Global	Both	60-64 yea	Other sexu	Number	1995	146.107	177.354	110.41
Deaths	Global	Both	60-64 yea	Other sexu	Rate	1995	0.08495	0.10311	0.06419
Deaths	Global	Both	65-69 yea	Other sexu	Number	1995	103.925	124.098	84.4085
Deaths	Global	Both	65-69 yea	Other sexu	Rate	1995	0.07315	0.08735	0.05942
Deaths	Global	Both	50-69 yea	Other sexu	Number	1995	642.219	742.115	474.385
Deaths	Global	Both	50-69 yea	Other sexu	Rate	1995	0.08762	0.10125	0.06472
Deaths	Global	Both	50-54 yea	Sexually tr	Number	2008	574.179	677.825	439.535
Deaths	Global	Both	50-54 yea	Sexually tr	Rate	2008	0.17101	0.20188	0.13091
Deaths	Global	Both	55-59 yea	Sexually tr	Number	2008	728.709	906.589	534.049
Deaths	Global	Both	55-59 yea	Sexually tr	Rate	2008	0.2584	0.32148	0.18938
Deaths	Global	Both	60-64 yea	Sexually tr	Number	2008	484.911	586.011	376.195
Deaths	Global	Both	60-64 yea	Sexually tr	Rate	2008	0.2233	0.26986	0.17324
Deaths	Global	Both	65-69 yea	Sexually tr	Number	2008	460.815	546.738	388.401
Deaths	Global	Both	65-69 yea	Sexually tr	Rate	2008	0.26427	0.31355	0.22275
Deaths	Global	Both	50-69 yea	Sexually tr	Number	2008	2248.61	2580.05	1788.6
Deaths	Global	Both	50-69 yea	Sexually tr	Rate	2008	0.22279	0.25563	0.17722
Deaths	Global	Both	50-54 yea	Syphilis	Number	2008	155.925	219.984	86.8669
Deaths	Global	Both	50-54 yea	Syphilis	Rate	2008	0.04644	0.06552	0.02587
Deaths	Global	Both	55-59 yea	Syphilis	Number	2008	186.715	294.434	85.576
Deaths	Global	Both	55-59 yea	Syphilis	Rate	2008	0.06621	0.10441	0.03035
Deaths	Global	Both	60-64 yea	Syphilis	Number	2008	144.61	207.658	87.4521
Deaths	Global	Both	60-64 yea	Syphilis	Rate	2008	0.06659	0.09563	0.04027
Deaths	Global	Both	65-69 yea	Syphilis	Number	2008	97.1268	146.461	76.2345
Deaths	Global	Both	65-69 yea	Syphilis	Rate	2008	0.0557	0.08399	0.04372
Deaths	Global	Both	50-69 yea	Syphilis	Number	2008	584.377	788.858	348.384
Deaths	Global	Both	50-69 yea	Syphilis	Rate	2008	0.0579	0.07816	0.03452
Deaths	Global	Both	50-54 yea	Chlamydia	Number	2008	61.2747	70.5942	50.6609
Deaths	Global	Both	50-54 yea	Chlamydia	Rate	2008	0.01825	0.02103	0.01509
Deaths	Global	Both	55-59 yea	Chlamydia	Number	2008	83.8346	99.8815	62.5702
Deaths	Global	Both	55-59 yea	Chlamydia	Rate	2008	0.02973	0.03542	0.02219
Deaths	Global	Both	60-64 yea	Chlamydia	Number	2008	54.1671	63.8999	41.645
Deaths	Global	Both	60-64 yea	Chlamydia	Rate	2008	0.02494	0.02943	0.01918
Deaths	Global	Both	65-69 yea	Chlamydia	Number	2008	39.425	46.6045	31.1089
Deaths	Global	Both	65-69 yea	Chlamydia	Rate	2008	0.02261	0.02673	0.01784
Deaths	Global	Both	50-69 yea	Chlamydia	Number	2008	238.702	268.247	184.68
Deaths	Global	Both	50-69 yea	Chlamydia	Rate	2008	0.02365	0.02658	0.0183
Deaths	Global	Both	50-54 yea	Gonococca	Number	2008	195.69	225.247	159.993
Deaths	Global	Both	50-54 yea	Gonococca	Rate	2008	0.05828	0.06709	0.04765
Deaths	Global	Both	55-59 yea	Gonococca	Number	2008	247.887	293.485	182.067
Deaths	Global	Both	55-59 yea	Gonococca	Rate	2008	0.0879	0.10407	0.06456
Deaths	Global	Both	60-64 yea	Gonococca	Number	2008	156.352	181.96	125.883
Deaths	Global	Both	60-64 yea	Gonococca	Rate	2008	0.072	0.08379	0.05797
Deaths	Global	Both	65-69 yea	Gonococca	Number	2008	223.354	261.654	178.744
Deaths	Global	Both	65-69 yea	Gonococca	Rate	2008	0.12809	0.15006	0.10251
Deaths	Global	Both	50-69 yea	Gonococca	Number	2008	823.283	921.863	646.62
Deaths	Global	Both	50-69 yea	Gonococca	Rate	2008	0.08157	0.09134	0.06407
Deaths	Global	Both	50-54 yea	Other sexu	Number	2008	161.289	187.157	123.47
Deaths	Global	Both	50-54 yea	Other sexu	Rate	2008	0.04804	0.05574	0.03677
Deaths	Global	Both	55-59 yea	Other sexu	Number	2008	210.272	252.309	153.334
Deaths	Global	Both	55-59 yea	Other sexu	Rate	2008	0.07456	0.08947	0.05437
Deaths	Global	Both	60-64 yea	Other sexu	Number	2008	129.782	153.956	102.075
Deaths	Global	Both	60-64 yea	Other sexu	Rate	2008	0.05976	0.0709	0.04701
Deaths	Global	Both	65-69 yea	Other sexu	Number	2008	100.909	119.225	81.6169
Deaths	Global	Both	65-69 yea	Other sexu	Rate	2008	0.05787	0.06837	0.04681
Deaths	Global	Both	50-69 yea	Other sexu	Number	2008	602.252	679.844	456.006
Deaths	Global	Both	50-69 yea	Other sexu	Rate	2008	0.05967	0.06736	0.04518
Deaths	Global	Both	50-54 yea	HIV/AIDS	Number	1999	81961.4	97172.3	65683.2
Deaths	Global	Both	50-54 yea	HIV/AIDS	Rate	1999	32.4984	38.5296	26.0439
Deaths	Global	Both	55-59 yea	HIV/AIDS	Number	1999	48597.7	56962.9	39471.5
Deaths	Global	Both	55-59 yea	HIV/AIDS	Rate	1999	23.7392	27.8255	19.2812
Deaths	Global	Both	60-64 yea	HIV/AIDS	Number	1999	30184.4	35413.7	24654.4
Deaths	Global	Both	60-64 yea	HIV/AIDS	Rate	1999	16.3925	19.2324	13.3893
Deaths	Global	Both	65-69 yea	HIV/AIDS	Number	1999	19022.2	22544.9	15491.7
Deaths	Global	Both	65-69 yea	HIV/AIDS	Rate	1999	12.5929	14.9249	10.2556
Deaths	Global	Both	50-69 yea	HIV/AIDS	Number	1999	179766	211797	145394
Deaths	Global	Both	50-69 yea	HIV/AIDS	Rate	1999	22.6946	26.7384	18.3553
Deaths	Global	Both	50-54 yea	Sexually tr	Number	1999	628.763	776.585	441.671
Deaths	Global	Both	50-54 yea	Sexually tr	Rate	1999	0.24931	0.30792	0.17513
Deaths	Global	Both	55-59 yea	Sexually tr	Number	1999	802.119	1050.33	547.645
Deaths	Global	Both	55-59 yea	Sexually tr	Rate	1999	0.39182	0.51307	0.26752
Deaths	Global	Both	60-64 yea	Sexually tr	Number	1999	622.234	773.269	455.179
Deaths	Global	Both	60-64 yea	Sexually tr	Rate	1999	0.33792	0.41995	0.2472
Deaths	Global	Both	65-69 yea	Sexually tr	Number	1999	542.243	648.492	441.058
Deaths	Global	Both	65-69 yea	Sexually tr	Rate	1999	0.35897	0.42931	0.29198
Deaths	Global	Both	50-69 yea	Sexually tr	Number	1999	2595.36	3093.09	1937.65
Deaths	Global	Both	50-69 yea	Sexually tr	Rate	1999	0.32765	0.39049	0.24462
Deaths	Global	Both	50-54 yea	Syphilis	Number	1999	209.174	305.184	116.771
Deaths	Global	Both	50-54 yea	Syphilis	Rate	1999	0.08294	0.12101	0.0463
Deaths	Global	Both	55-59 yea	Syphilis	Number	1999	264.578	443.427	126.446
Deaths	Global	Both	55-59 yea	Syphilis	Rate	1999	0.12924	0.21661	0.06177
Deaths	Global	Both	60-64 yea	Syphilis	Number	1999	244.827	339.441	151.206
Deaths	Global	Both	60-64 yea	Syphilis	Rate	1999	0.13296	0.18434	0.08212
Deaths	Global	Both	65-69 yea	Syphilis	Number	1999	164.572	219.321	125.248
Deaths	Global	Both	65-69 yea	Syphilis	Rate	1999	0.10895	0.14519	0.08292
Deaths	Global	Both	50-69 yea	Syphilis	Number	1999	883.152	1216.34	537.099
Deaths	Global	Both	50-69 yea	Syphilis	Rate	1999	0.11149	0.15356	0.06781
Deaths	Global	Both	50-54 yea	Chlamydia	Number	1999	60.6416	72.2867	46.5861
Deaths	Global	Both	50-54 yea	Chlamydia	Rate	1999	0.02404	0.02866	0.01847
Deaths	Global	Both	55-59 yea	Chlamydia	Number	1999	81.7031	101.16	59.4794
Deaths	Global	Both	55-59 yea	Chlamydia	Rate	1999	0.03991	0.04942	0.02905
Deaths	Global	Both	60-64 yea	Chlamydia	Number	1999	59.5302	71.6705	44.8569
Deaths	Global	Both	60-64 yea	Chlamydia	Rate	1999	0.03233	0.03892	0.02436
Deaths	Global	Both	65-69 yea	Chlamydia	Number	1999	40.5495	48.1023	31.7965
Deaths	Global	Both	65-69 yea	Chlamydia	Rate	1999	0.02684	0.03184	0.02105
Deaths	Global	Both	50-69 yea	Chlamydia	Number	1999	242.424	278.224	185.254
Deaths	Global	Both	50-69 yea	Chlamydia	Rate	1999	0.0306	0.03512	0.02339
Deaths	Global	Both	50-54 yea	Gonococca	Number	1999	194.833	230.622	149.548
Deaths	Global	Both	50-54 yea	Gonococca	Rate	1999	0.07725	0.09144	0.0593
Deaths	Global	Both	55-59 yea	Gonococca	Number	1999	243.933	299.87	177.897
Deaths	Global	Both	55-59 yea	Gonococca	Rate	1999	0.11916	0.14648	0.0869
Deaths	Global	Both	60-64 yea	Gonococca	Number	1999	175.777	208.873	138.244
Deaths	Global	Both	60-64 yea	Gonococca	Rate	1999	0.09546	0.11343	0.07508
Deaths	Global	Both	65-69 yea	Gonococca	Number	1999	232.256	274.634	185.053
Deaths	Global	Both	65-69 yea	Gonococca	Rate	1999	0.15376	0.18181	0.12251
Deaths	Global	Both	50-69 yea	Gonococca	Number	1999	846.799	966.979	654.748
Deaths	Global	Both	50-69 yea	Gonococca	Rate	1999	0.1069	0.12208	0.08266
Deaths	Global	Both	50-54 yea	Other sexu	Number	1999	164.114	202.087	120.209
Deaths	Global	Both	50-54 yea	Other sexu	Rate	1999	0.06507	0.08013	0.04766
Deaths	Global	Both	55-59 yea	Other sexu	Number	1999	211.905	267.379	153.077
Deaths	Global	Both	55-59 yea	Other sexu	Rate	1999	0.10351	0.13061	0.07478
Deaths	Global	Both	60-64 yea	Other sexu	Number	1999	142.099	172.985	109.226
Deaths	Global	Both	60-64 yea	Other sexu	Rate	1999	0.07717	0.09394	0.05932
Deaths	Global	Both	65-69 yea	Other sexu	Number	1999	104.864	124.506	84.135
Deaths	Global	Both	65-69 yea	Other sexu	Rate	1999	0.06942	0.08242	0.0557
Deaths	Global	Both	50-69 yea	Other sexu	Number	1999	622.982	725.714	472.535
Deaths	Global	Both	50-69 yea	Other sexu	Rate	1999	0.07865	0.09162	0.05966
Deaths	Global	Both	50-54 yea	Sexually tr	Number	2012	568.705	678.437	439.506
Deaths	Global	Both	50-54 yea	Sexually tr	Rate	2012	0.15875	0.18938	0.12268
Deaths	Global	Both	55-59 yea	Sexually tr	Number	2012	722.709	878.951	519.077
Deaths	Global	Both	55-59 yea	Sexually tr	Rate	2012	0.22755	0.27675	0.16344
Deaths	Global	Both	60-64 yea	Sexually tr	Number	2012	512.826	608.841	411.03
Deaths	Global	Both	60-64 yea	Sexually tr	Rate	2012	0.19672	0.23355	0.15767
Deaths	Global	Both	65-69 yea	Sexually tr	Number	2012	475.504	567.345	391.664
Deaths	Global	Both	65-69 yea	Sexually tr	Rate	2012	0.24943	0.2976	0.20545
Deaths	Global	Both	50-69 yea	Sexually tr	Number	2012	2279.74	2591.74	1793.95
Deaths	Global	Both	50-69 yea	Sexually tr	Rate	2012	0.20225	0.22993	0.15916
Deaths	Global	Both	50-54 yea	Syphilis	Number	2012	147.315	205.195	83.752
Deaths	Global	Both	50-54 yea	Syphilis	Rate	2012	0.04112	0.05728	0.02338
Deaths	Global	Both	55-59 yea	Syphilis	Number	2012	181.332	279.245	83.2513
Deaths	Global	Both	55-59 yea	Syphilis	Rate	2012	0.05709	0.08792	0.02621
Deaths	Global	Both	60-64 yea	Syphilis	Number	2012	144.891	205.556	88.0181
Deaths	Global	Both	60-64 yea	Syphilis	Rate	2012	0.05558	0.07885	0.03376
Deaths	Global	Both	65-69 yea	Syphilis	Number	2012	96.1513	139.872	76.0718
Deaths	Global	Both	65-69 yea	Syphilis	Rate	2012	0.05044	0.07337	0.0399
Deaths	Global	Both	50-69 yea	Syphilis	Number	2012	569.689	750.888	347.286
Deaths	Global	Both	50-69 yea	Syphilis	Rate	2012	0.05054	0.06662	0.03081
Deaths	Global	Both	50-54 yea	Chlamydia	Number	2012	62.2662	72.9486	51.0575
Deaths	Global	Both	50-54 yea	Chlamydia	Rate	2012	0.01738	0.02036	0.01425
Deaths	Global	Both	55-59 yea	Chlamydia	Number	2012	83.9116	100.592	63.8384
Deaths	Global	Both	55-59 yea	Chlamydia	Rate	2012	0.02642	0.03167	0.0201
Deaths	Global	Both	60-64 yea	Chlamydia	Number	2012	59.0905	69.8106	46.2721
Deaths	Global	Both	60-64 yea	Chlamydia	Rate	2012	0.02267	0.02678	0.01775
Deaths	Global	Both	65-69 yea	Chlamydia	Number	2012	41.2836	49.5437	31.8811
Deaths	Global	Both	65-69 yea	Chlamydia	Rate	2012	0.02166	0.02599	0.01672
Deaths	Global	Both	50-69 yea	Chlamydia	Number	2012	246.552	275.733	194.997
Deaths	Global	Both	50-69 yea	Chlamydia	Rate	2012	0.02187	0.02446	0.0173
Deaths	Global	Both	50-54 yea	Gonococca	Number	2012	198.043	231.15	162.912
Deaths	Global	Both	50-54 yea	Gonococca	Rate	2012	0.05528	0.06452	0.04548
Deaths	Global	Both	55-59 yea	Gonococca	Number	2012	247.793	294.655	186.332
Deaths	Global	Both	55-59 yea	Gonococca	Rate	2012	0.07802	0.09278	0.05867
Deaths	Global	Both	60-64 yea	Gonococca	Number	2012	169.603	197.561	137.357
Deaths	Global	Both	60-64 yea	Gonococca	Rate	2012	0.06506	0.07579	0.05269
Deaths	Global	Both	65-69 yea	Gonococca	Number	2012	232.681	277.355	182.199
Deaths	Global	Both	65-69 yea	Gonococca	Rate	2012	0.12205	0.14549	0.09557
Deaths	Global	Both	50-69 yea	Gonococca	Number	2012	848.12	946.612	669.241
Deaths	Global	Both	50-69 yea	Gonococca	Rate	2012	0.07524	0.08398	0.05937
Deaths	Global	Both	50-54 yea	Other sexu	Number	2012	161.081	190.319	126.407
Deaths	Global	Both	50-54 yea	Other sexu	Rate	2012	0.04496	0.05313	0.03529
Deaths	Global	Both	55-59 yea	Other sexu	Number	2012	209.672	251.14	151.82
Deaths	Global	Both	55-59 yea	Other sexu	Rate	2012	0.06602	0.07907	0.0478
Deaths	Global	Both	60-64 yea	Other sexu	Number	2012	139.24	164.301	110.34
Deaths	Global	Both	60-64 yea	Other sexu	Rate	2012	0.05341	0.06303	0.04233
Deaths	Global	Both	65-69 yea	Other sexu	Number	2012	105.388	126.019	83.2149
Deaths	Global	Both	65-69 yea	Other sexu	Rate	2012	0.05528	0.0661	0.04365
Deaths	Global	Both	50-69 yea	Other sexu	Number	2012	615.381	695.08	472.643
Deaths	Global	Both	50-69 yea	Other sexu	Rate	2012	0.0546	0.06167	0.04193
Deaths	Global	Both	50-54 yea	Sexually tr	Number	1998	636.984	785.993	440
Deaths	Global	Both	50-54 yea	Sexually tr	Rate	1998	0.26529	0.32735	0.18325
Deaths	Global	Both	55-59 yea	Sexually tr	Number	1998	826.678	1077.91	553.683
Deaths	Global	Both	55-59 yea	Sexually tr	Rate	1998	0.40599	0.52938	0.27192
Deaths	Global	Both	60-64 yea	Sexually tr	Number	1998	632.351	773.16	445.859
Deaths	Global	Both	60-64 yea	Sexually tr	Rate	1998	0.34903	0.42675	0.2461
Deaths	Global	Both	65-69 yea	Sexually tr	Number	1998	553.672	660.611	446.545
Deaths	Global	Both	65-69 yea	Sexually tr	Rate	1998	0.37091	0.44255	0.29914
Deaths	Global	Both	50-69 yea	Sexually tr	Number	1998	2649.68	3156.03	1922.93
Deaths	Global	Both	50-69 yea	Sexually tr	Rate	1998	0.34226	0.40767	0.24839
Deaths	Global	Both	50-54 yea	Syphilis	Number	1998	209.603	301.828	115.146
Deaths	Global	Both	50-54 yea	Syphilis	Rate	1998	0.0873	0.12571	0.04796
Deaths	Global	Both	55-59 yea	Syphilis	Number	1998	277.752	461.166	135.804
Deaths	Global	Both	55-59 yea	Syphilis	Rate	1998	0.13641	0.22649	0.0667
Deaths	Global	Both	60-64 yea	Syphilis	Number	1998	245.239	340.736	148.806
Deaths	Global	Both	60-64 yea	Syphilis	Rate	1998	0.13536	0.18807	0.08213
Deaths	Global	Both	65-69 yea	Syphilis	Number	1998	172.603	226.896	130.085
Deaths	Global	Both	65-69 yea	Syphilis	Rate	1998	0.11563	0.152	0.08715
Deaths	Global	Both	50-69 yea	Syphilis	Number	1998	905.197	1246.03	548.643
Deaths	Global	Both	50-69 yea	Syphilis	Rate	1998	0.11693	0.16095	0.07087
Deaths	Global	Both	50-54 yea	Chlamydia	Number	1998	61.8463	74.1633	46.8522
Deaths	Global	Both	50-54 yea	Chlamydia	Rate	1998	0.02576	0.03089	0.01951
Deaths	Global	Both	55-59 yea	Chlamydia	Number	1998	83.4995	104.134	60.1601
Deaths	Global	Both	55-59 yea	Chlamydia	Rate	1998	0.04101	0.05114	0.02955
Deaths	Global	Both	60-64 yea	Chlamydia	Number	1998	61.0807	73.5437	44.7893
Deaths	Global	Both	60-64 yea	Chlamydia	Rate	1998	0.03371	0.04059	0.02472
Deaths	Global	Both	65-69 yea	Chlamydia	Number	1998	40.8816	49.1239	32.0089
Deaths	Global	Both	65-69 yea	Chlamydia	Rate	1998	0.02739	0.03291	0.02144
Deaths	Global	Both	50-69 yea	Chlamydia	Number	1998	247.308	284.324	186.27
Deaths	Global	Both	50-69 yea	Chlamydia	Rate	1998	0.03194	0.03673	0.02406
Deaths	Global	Both	50-54 yea	Gonococca	Number	1998	198.493	237.192	149.767
Deaths	Global	Both	50-54 yea	Gonococca	Rate	1998	0.08267	0.09879	0.06238
Deaths	Global	Both	55-59 yea	Gonococca	Number	1998	249.243	309.702	179.005
Deaths	Global	Both	55-59 yea	Gonococca	Rate	1998	0.12241	0.1521	0.08791
Deaths	Global	Both	60-64 yea	Gonococca	Number	1998	179.626	213.545	137.124
Deaths	Global	Both	60-64 yea	Gonococca	Rate	1998	0.09915	0.11787	0.07569
Deaths	Global	Both	65-69 yea	Gonococca	Number	1998	234.507	278.949	188.977
Deaths	Global	Both	65-69 yea	Gonococca	Rate	1998	0.1571	0.18687	0.1266
Deaths	Global	Both	50-69 yea	Gonococca	Number	1998	861.869	985.232	652.425
Deaths	Global	Both	50-69 yea	Gonococca	Rate	1998	0.11133	0.12726	0.08427
Deaths	Global	Both	50-54 yea	Other sexu	Number	1998	167.043	203.885	121.98
Deaths	Global	Both	50-54 yea	Other sexu	Rate	1998	0.06957	0.08492	0.0508
Deaths	Global	Both	55-59 yea	Other sexu	Number	1998	216.182	273.255	149.657
Deaths	Global	Both	55-59 yea	Other sexu	Rate	1998	0.10617	0.1342	0.0735
Deaths	Global	Both	60-64 yea	Other sexu	Number	1998	146.405	178.37	109.216
Deaths	Global	Both	60-64 yea	Other sexu	Rate	1998	0.08081	0.09845	0.06028
Deaths	Global	Both	65-69 yea	Other sexu	Number	1998	105.681	126.254	84.8424
Deaths	Global	Both	65-69 yea	Other sexu	Rate	1998	0.0708	0.08458	0.05684
Deaths	Global	Both	50-69 yea	Other sexu	Number	1998	635.311	741.978	465.843
Deaths	Global	Both	50-69 yea	Other sexu	Rate	1998	0.08206	0.09584	0.06017
Deaths	Global	Both	50-54 yea	HIV/AIDS	Number	2010	98300.3	111031	88484
Deaths	Global	Both	50-54 yea	HIV/AIDS	Rate	2010	28.6341	32.3424	25.7747
Deaths	Global	Both	55-59 yea	HIV/AIDS	Number	2010	55905.8	64301.8	50347.4
Deaths	Global	Both	55-59 yea	HIV/AIDS	Rate	2010	18.4264	21.1937	16.5944
Deaths	Global	Both	60-64 yea	HIV/AIDS	Number	2010	30526.2	35581.6	27298.6
Deaths	Global	Both	60-64 yea	HIV/AIDS	Rate	2010	12.7767	14.8927	11.4258
Deaths	Global	Both	65-69 yea	HIV/AIDS	Number	2010	17526.5	20465.1	15631.5
Deaths	Global	Both	65-69 yea	HIV/AIDS	Rate	2010	9.82756	11.4753	8.76499
Deaths	Global	Both	50-69 yea	HIV/AIDS	Number	2010	202259	229684	183008
Deaths	Global	Both	50-69 yea	HIV/AIDS	Rate	2010	19.01	21.5877	17.2006
Deaths	Global	Both	50-54 yea	HIV/AIDS	Number	2014	87353.6	98810.1	79654.9
Deaths	Global	Both	50-54 yea	HIV/AIDS	Rate	2014	22.6759	25.6498	20.6774
Deaths	Global	Both	55-59 yea	HIV/AIDS	Number	2014	51377.6	58830.7	46764.5
Deaths	Global	Both	55-59 yea	HIV/AIDS	Rate	2014	15.7532	18.0384	14.3387
Deaths	Global	Both	60-64 yea	HIV/AIDS	Number	2014	30455.1	34740.7	27681.1
Deaths	Global	Both	60-64 yea	HIV/AIDS	Rate	2014	10.906	12.4406	9.9126
Deaths	Global	Both	65-69 yea	HIV/AIDS	Number	2014	17253.8	19507.5	15736.1
Deaths	Global	Both	65-69 yea	HIV/AIDS	Rate	2014	8.16804	9.23496	7.44957
Deaths	Global	Both	50-69 yea	HIV/AIDS	Number	2014	186440	210405	171851
Deaths	Global	Both	50-69 yea	HIV/AIDS	Rate	2014	15.5127	17.5067	14.2988
Deaths	Global	Both	50-54 yea	HIV/AIDS	Number	2018	82224.9	94862.2	73634.5
Deaths	Global	Both	50-54 yea	HIV/AIDS	Rate	2018	19.0967	22.0317	17.1016
Deaths	Global	Both	55-59 yea	HIV/AIDS	Number	2018	50940.6	59306.1	45564.5
Deaths	Global	Both	55-59 yea	HIV/AIDS	Rate	2018	14.2735	16.6175	12.7671
Deaths	Global	Both	60-64 yea	HIV/AIDS	Number	2018	31348.7	36479.7	27904.3
Deaths	Global	Both	60-64 yea	HIV/AIDS	Rate	2018	10.1435	11.8037	9.02897
Deaths	Global	Both	65-69 yea	HIV/AIDS	Number	2018	19330.7	22179.2	17336.5
Deaths	Global	Both	65-69 yea	HIV/AIDS	Rate	2018	7.72319	8.86127	6.92646
Deaths	Global	Both	50-69 yea	HIV/AIDS	Number	2018	183845	212042	165853
Deaths	Global	Both	50-69 yea	HIV/AIDS	Rate	2018	13.6504	15.744	12.3145
Deaths	Global	Both	50-54 yea	HIV/AIDS	Number	2000	90333.5	105451	73737.2
Deaths	Global	Both	50-54 yea	HIV/AIDS	Rate	2000	34.4476	40.2123	28.1188
Deaths	Global	Both	55-59 yea	HIV/AIDS	Number	2000	52789.2	60640	43926.7
Deaths	Global	Both	55-59 yea	HIV/AIDS	Rate	2000	25.644	29.4578	21.3388
Deaths	Global	Both	60-64 yea	HIV/AIDS	Number	2000	32616.4	37463.9	27357.5
Deaths	Global	Both	60-64 yea	HIV/AIDS	Rate	2000	17.446	20.0388	14.6331
Deaths	Global	Both	65-69 yea	HIV/AIDS	Number	2000	20411.8	23720.1	17042.7
Deaths	Global	Both	65-69 yea	HIV/AIDS	Rate	2000	13.3255	15.4853	11.1261
Deaths	Global	Both	50-69 yea	HIV/AIDS	Number	2000	196151	226948	162629
Deaths	Global	Both	50-69 yea	HIV/AIDS	Rate	2000	24.2694	28.0798	20.1218
Deaths	Global	Both	50-54 yea	Sexually tr	Number	2001	624.956	764.519	454.129
Deaths	Global	Both	50-54 yea	Sexually tr	Rate	2001	0.22696	0.27764	0.16492
Deaths	Global	Both	55-59 yea	Sexually tr	Number	2001	755.813	995.016	523.478
Deaths	Global	Both	55-59 yea	Sexually tr	Rate	2001	0.36107	0.47534	0.25007
Deaths	Global	Both	60-64 yea	Sexually tr	Number	2001	578.618	718.595	439.326
Deaths	Global	Both	60-64 yea	Sexually tr	Rate	2001	0.30594	0.37995	0.23229
Deaths	Global	Both	65-69 yea	Sexually tr	Number	2001	531.241	634.996	433.818
Deaths	Global	Both	65-69 yea	Sexually tr	Rate	2001	0.34074	0.40728	0.27825
Deaths	Global	Both	50-69 yea	Sexually tr	Number	2001	2490.63	2963.09	1874.2
Deaths	Global	Both	50-69 yea	Sexually tr	Rate	2001	0.30017	0.35711	0.22588
Deaths	Global	Both	50-54 yea	Syphilis	Number	2001	199.834	287.064	112.311
Deaths	Global	Both	50-54 yea	Syphilis	Rate	2001	0.07257	0.10425	0.04079
Deaths	Global	Both	55-59 yea	Syphilis	Number	2001	232.566	392.358	106.915
Deaths	Global	Both	55-59 yea	Syphilis	Rate	2001	0.1111	0.18744	0.05108
Deaths	Global	Both	60-64 yea	Syphilis	Number	2001	216.505	310.205	135.326
Deaths	Global	Both	60-64 yea	Syphilis	Rate	2001	0.11447	0.16402	0.07155
Deaths	Global	Both	65-69 yea	Syphilis	Number	2001	145.404	198.574	114.132
Deaths	Global	Both	65-69 yea	Syphilis	Rate	2001	0.09326	0.12736	0.0732
Deaths	Global	Both	50-69 yea	Syphilis	Number	2001	794.308	1114.14	480.664
Deaths	Global	Both	50-69 yea	Syphilis	Rate	2001	0.09573	0.13428	0.05793
Deaths	Global	Both	50-54 yea	Chlamydia	Number	2001	61.741	72.0645	49.4231
Deaths	Global	Both	50-54 yea	Chlamydia	Rate	2001	0.02242	0.02617	0.01795
Deaths	Global	Both	55-59 yea	Chlamydia	Number	2001	79.5807	97.9629	56.8995
Deaths	Global	Both	55-59 yea	Chlamydia	Rate	2001	0.03802	0.0468	0.02718
Deaths	Global	Both	60-64 yea	Chlamydia	Number	2001	57.224	68.6118	43.1004
Deaths	Global	Both	60-64 yea	Chlamydia	Rate	2001	0.03026	0.03628	0.02279
Deaths	Global	Both	65-69 yea	Chlamydia	Number	2001	41.5677	49.6197	32.6638
Deaths	Global	Both	65-69 yea	Chlamydia	Rate	2001	0.02666	0.03183	0.02095
Deaths	Global	Both	50-69 yea	Chlamydia	Number	2001	240.113	273.486	184.856
Deaths	Global	Both	50-69 yea	Chlamydia	Rate	2001	0.02894	0.03296	0.02228
Deaths	Global	Both	50-54 yea	Gonococca	Number	2001	198.044	229.687	156.007
Deaths	Global	Both	50-54 yea	Gonococca	Rate	2001	0.07192	0.08341	0.05665
Deaths	Global	Both	55-59 yea	Gonococca	Number	2001	237.399	290.315	168.319
Deaths	Global	Both	55-59 yea	Gonococca	Rate	2001	0.11341	0.13869	0.08041
Deaths	Global	Both	60-64 yea	Gonococca	Number	2001	168.723	199.795	133.613
Deaths	Global	Both	60-64 yea	Gonococca	Rate	2001	0.08921	0.10564	0.07065
Deaths	Global	Both	65-69 yea	Gonococca	Number	2001	237.072	281.707	189.501
Deaths	Global	Both	65-69 yea	Gonococca	Rate	2001	0.15206	0.18069	0.12155
Deaths	Global	Both	50-69 yea	Gonococca	Number	2001	841.238	951.711	647.055
Deaths	Global	Both	50-69 yea	Gonococca	Rate	2001	0.10139	0.1147	0.07798
Deaths	Global	Both	50-54 yea	Other sexu	Number	2001	165.337	198.557	123.989
Deaths	Global	Both	50-54 yea	Other sexu	Rate	2001	0.06004	0.07211	0.04503
Deaths	Global	Both	55-59 yea	Other sexu	Number	2001	206.268	257.997	143.456
Deaths	Global	Both	55-59 yea	Other sexu	Rate	2001	0.09854	0.12325	0.06853
Deaths	Global	Both	60-64 yea	Other sexu	Number	2001	136.166	164.709	105.953
Deaths	Global	Both	60-64 yea	Other sexu	Rate	2001	0.072	0.08709	0.05602
Deaths	Global	Both	65-69 yea	Other sexu	Number	2001	107.198	126.713	85.7832
Deaths	Global	Both	65-69 yea	Other sexu	Rate	2001	0.06876	0.08127	0.05502
Deaths	Global	Both	50-69 yea	Other sexu	Number	2001	614.969	712.106	461.455
Deaths	Global	Both	50-69 yea	Other sexu	Rate	2001	0.07412	0.08582	0.05561
Deaths	Global	Both	50-54 yea	HIV/AIDS	Number	2001	100664	115026	83792.6
Deaths	Global	Both	50-54 yea	HIV/AIDS	Rate	2001	36.5567	41.7722	30.4297
Deaths	Global	Both	55-59 yea	HIV/AIDS	Number	2001	56904.2	64224.8	48613.7
Deaths	Global	Both	55-59 yea	HIV/AIDS	Rate	2001	27.1842	30.6814	23.2237
Deaths	Global	Both	60-64 yea	HIV/AIDS	Number	2001	34874.9	39325.8	29994.7
Deaths	Global	Both	60-64 yea	HIV/AIDS	Rate	2001	18.4396	20.7929	15.8592
Deaths	Global	Both	65-69 yea	HIV/AIDS	Number	2001	21730.1	24857	18600.1
Deaths	Global	Both	65-69 yea	HIV/AIDS	Rate	2001	13.9376	15.9432	11.93
Deaths	Global	Both	50-69 yea	HIV/AIDS	Number	2001	214173	243370	181632
Deaths	Global	Both	50-69 yea	HIV/AIDS	Rate	2001	25.8123	29.3311	21.8904
Deaths	Global	Both	50-54 yea	HIV/AIDS	Number	2004	122960	133975	110158
Deaths	Global	Both	50-54 yea	HIV/AIDS	Rate	2004	40.4071	44.0269	36.2002
Deaths	Global	Both	55-59 yea	HIV/AIDS	Number	2004	65873.8	70935.6	61033.4
Deaths	Global	Both	55-59 yea	HIV/AIDS	Rate	2004	27.5042	29.6176	25.4832
Deaths	Global	Both	60-64 yea	HIV/AIDS	Number	2004	38926.5	42138.2	36128.2
Deaths	Global	Both	60-64 yea	HIV/AIDS	Rate	2004	20.1369	21.7984	18.6893
Deaths	Global	Both	65-69 yea	HIV/AIDS	Number	2004	23938.3	25979	22148
Deaths	Global	Both	65-69 yea	HIV/AIDS	Rate	2004	14.4477	15.6793	13.3672
Deaths	Global	Both	50-69 yea	HIV/AIDS	Number	2004	251698	271917	230635
Deaths	Global	Both	50-69 yea	HIV/AIDS	Rate	2004	27.8796	30.1192	25.5465
Deaths	Global	Both	50-54 yea	Sexually tr	Number	2016	691.534	840.212	502.348
Deaths	Global	Both	50-54 yea	Sexually tr	Rate	2016	0.16788	0.20397	0.12195
Deaths	Global	Both	55-59 yea	Sexually tr	Number	2016	800.581	993.694	556.144
Deaths	Global	Both	55-59 yea	Sexually tr	Rate	2016	0.23826	0.29574	0.16552
Deaths	Global	Both	60-64 yea	Sexually tr	Number	2016	560.346	663.115	429.896
Deaths	Global	Both	60-64 yea	Sexually tr	Rate	2016	0.18787	0.22233	0.14413
Deaths	Global	Both	65-69 yea	Sexually tr	Number	2016	512.243	608.947	417.371
Deaths	Global	Both	65-69 yea	Sexually tr	Rate	2016	0.22032	0.26191	0.17951
Deaths	Global	Both	50-69 yea	Sexually tr	Number	2016	2564.7	2956.78	1909.19
Deaths	Global	Both	50-69 yea	Sexually tr	Rate	2016	0.20057	0.23123	0.14931
Deaths	Global	Both	50-54 yea	Syphilis	Number	2016	159.749	214.196	93.6923
Deaths	Global	Both	50-54 yea	Syphilis	Rate	2016	0.03878	0.052	0.02274
Deaths	Global	Both	55-59 yea	Syphilis	Number	2016	178.623	269.048	84.8812
Deaths	Global	Both	55-59 yea	Syphilis	Rate	2016	0.05316	0.08007	0.02526
Deaths	Global	Both	60-64 yea	Syphilis	Number	2016	149.742	207.84	92.0124
Deaths	Global	Both	60-64 yea	Syphilis	Rate	2016	0.05021	0.06968	0.03085
Deaths	Global	Both	65-69 yea	Syphilis	Number	2016	100.08	142.487	81.2382
Deaths	Global	Both	65-69 yea	Syphilis	Rate	2016	0.04304	0.06128	0.03494
Deaths	Global	Both	50-69 yea	Syphilis	Number	2016	588.193	758.782	371.685
Deaths	Global	Both	50-69 yea	Syphilis	Rate	2016	0.046	0.05934	0.02907
Deaths	Global	Both	50-54 yea	Chlamydia	Number	2016	80.0569	99.494	59.0573
Deaths	Global	Both	50-54 yea	Chlamydia	Rate	2016	0.01943	0.02415	0.01434
Deaths	Global	Both	55-59 yea	Chlamydia	Number	2016	97.6955	123.593	69.6046
Deaths	Global	Both	55-59 yea	Chlamydia	Rate	2016	0.02908	0.03678	0.02072
Deaths	Global	Both	60-64 yea	Chlamydia	Number	2016	66.3918	79.677	50.9749
Deaths	Global	Both	60-64 yea	Chlamydia	Rate	2016	0.02226	0.02671	0.01709
Deaths	Global	Both	65-69 yea	Chlamydia	Number	2016	44.9768	53.7179	35.4629
Deaths	Global	Both	65-69 yea	Chlamydia	Rate	2016	0.01934	0.0231	0.01525
Deaths	Global	Both	50-69 yea	Chlamydia	Number	2016	289.121	338.085	218.975
Deaths	Global	Both	50-69 yea	Chlamydia	Rate	2016	0.02261	0.02644	0.01712
Deaths	Global	Both	50-54 yea	Gonococca	Number	2016	252.327	312.788	189.566
Deaths	Global	Both	50-54 yea	Gonococca	Rate	2016	0.06125	0.07593	0.04602
Deaths	Global	Both	55-59 yea	Gonococca	Number	2016	286.286	360.577	201.472
Deaths	Global	Both	55-59 yea	Gonococca	Rate	2016	0.0852	0.10731	0.05996
Deaths	Global	Both	60-64 yea	Gonococca	Number	2016	188.845	224.744	147.212
Deaths	Global	Both	60-64 yea	Gonococca	Rate	2016	0.06332	0.07535	0.04936
Deaths	Global	Both	65-69 yea	Gonococca	Number	2016	253.397	299.498	201.608
Deaths	Global	Both	65-69 yea	Gonococca	Rate	2016	0.10899	0.12881	0.08671
Deaths	Global	Both	50-69 yea	Gonococca	Number	2016	980.855	1128.89	749.991
Deaths	Global	Both	50-69 yea	Gonococca	Rate	2016	0.07671	0.08828	0.05865
Deaths	Global	Both	50-54 yea	Other sexu	Number	2016	199.401	243.959	147.058
Deaths	Global	Both	50-54 yea	Other sexu	Rate	2016	0.04841	0.05922	0.0357
Deaths	Global	Both	55-59 yea	Other sexu	Number	2016	237.976	295.343	164.247
Deaths	Global	Both	55-59 yea	Other sexu	Rate	2016	0.07082	0.0879	0.04888
Deaths	Global	Both	60-64 yea	Other sexu	Number	2016	155.368	186.503	117.733
Deaths	Global	Both	60-64 yea	Other sexu	Rate	2016	0.05209	0.06253	0.03947
Deaths	Global	Both	65-69 yea	Other sexu	Number	2016	113.79	135.569	89.7876
Deaths	Global	Both	65-69 yea	Other sexu	Rate	2016	0.04894	0.05831	0.03862
Deaths	Global	Both	50-69 yea	Other sexu	Number	2016	706.534	821.381	521.224
Deaths	Global	Both	50-69 yea	Other sexu	Rate	2016	0.05525	0.06424	0.04076
Deaths	Global	Both	50-54 yea	Sexually tr	Number	2002	621.987	757.934	459.17
Deaths	Global	Both	50-54 yea	Sexually tr	Rate	2002	0.21835	0.26607	0.16119
Deaths	Global	Both	55-59 yea	Sexually tr	Number	2002	740.099	970.572	517.134
Deaths	Global	Both	55-59 yea	Sexually tr	Rate	2002	0.3398	0.44561	0.23743
Deaths	Global	Both	60-64 yea	Sexually tr	Number	2002	557.598	685.847	425.044
Deaths	Global	Both	60-64 yea	Sexually tr	Rate	2002	0.29262	0.35992	0.22306
Deaths	Global	Both	65-69 yea	Sexually tr	Number	2002	529.587	631.751	431.218
Deaths	Global	Both	65-69 yea	Sexually tr	Rate	2002	0.3324	0.39653	0.27066
Deaths	Global	Both	50-69 yea	Sexually tr	Number	2002	2449.27	2870.48	1883.42
Deaths	Global	Both	50-69 yea	Sexually tr	Rate	2002	0.28729	0.3367	0.22092
Deaths	Global	Both	50-54 yea	Syphilis	Number	2002	194.239	279.425	110.66
Deaths	Global	Both	50-54 yea	Syphilis	Rate	2002	0.06819	0.09809	0.03885
Deaths	Global	Both	55-59 yea	Syphilis	Number	2002	221.894	364.604	101.483
Deaths	Global	Both	55-59 yea	Syphilis	Rate	2002	0.10188	0.1674	0.04659
Deaths	Global	Both	60-64 yea	Syphilis	Number	2002	197.928	289.071	124.098
Deaths	Global	Both	60-64 yea	Syphilis	Rate	2002	0.10387	0.1517	0.06512
Deaths	Global	Both	65-69 yea	Syphilis	Number	2002	137.459	191.885	109.321
Deaths	Global	Both	65-69 yea	Syphilis	Rate	2002	0.08628	0.12044	0.06862
Deaths	Global	Both	50-69 yea	Syphilis	Number	2002	751.52	1063.41	458.335
Deaths	Global	Both	50-69 yea	Syphilis	Rate	2002	0.08815	0.12473	0.05376
Deaths	Global	Both	50-54 yea	Chlamydia	Number	2002	62.1537	72.6249	49.9957
Deaths	Global	Both	50-54 yea	Chlamydia	Rate	2002	0.02182	0.02549	0.01755
Deaths	Global	Both	55-59 yea	Chlamydia	Number	2002	78.907	97.5448	56.9331
Deaths	Global	Both	55-59 yea	Chlamydia	Rate	2002	0.03623	0.04479	0.02614
Deaths	Global	Both	60-64 yea	Chlamydia	Number	2002	56.9109	68.0353	43.9773
Deaths	Global	Both	60-64 yea	Chlamydia	Rate	2002	0.02987	0.0357	0.02308
Deaths	Global	Both	65-69 yea	Chlamydia	Number	2002	42.3233	50.1781	33.0543
Deaths	Global	Both	65-69 yea	Chlamydia	Rate	2002	0.02656	0.0315	0.02075
Deaths	Global	Both	50-69 yea	Chlamydia	Number	2002	240.295	273.688	185.283
Deaths	Global	Both	50-69 yea	Chlamydia	Rate	2002	0.02819	0.0321	0.02173
Deaths	Global	Both	50-54 yea	Gonococca	Number	2002	199.315	230.694	157.934
Deaths	Global	Both	50-54 yea	Gonococca	Rate	2002	0.06997	0.08099	0.05544
Deaths	Global	Both	55-59 yea	Gonococca	Number	2002	235.234	287.878	170.062
Deaths	Global	Both	55-59 yea	Gonococca	Rate	2002	0.108	0.13217	0.07808
Deaths	Global	Both	60-64 yea	Gonococca	Number	2002	167.078	197.038	133.726
Deaths	Global	Both	60-64 yea	Gonococca	Rate	2002	0.08768	0.1034	0.07018
Deaths	Global	Both	65-69 yea	Gonococca	Number	2002	240.914	283.133	190.527
Deaths	Global	Both	65-69 yea	Gonococca	Rate	2002	0.15121	0.17771	0.11959
Deaths	Global	Both	50-69 yea	Gonococca	Number	2002	842.542	948.494	654.214
Deaths	Global	Both	50-69 yea	Gonococca	Rate	2002	0.09883	0.11126	0.07674
Deaths	Global	Both	50-54 yea	Other sexu	Number	2002	166.279	199.295	126.152
Deaths	Global	Both	50-54 yea	Other sexu	Rate	2002	0.05837	0.06996	0.04429
Deaths	Global	Both	55-59 yea	Other sexu	Number	2002	204.064	252.745	144.868
Deaths	Global	Both	55-59 yea	Other sexu	Rate	2002	0.09369	0.11604	0.06651
Deaths	Global	Both	60-64 yea	Other sexu	Number	2002	135.68	164.174	105.576
Deaths	Global	Both	60-64 yea	Other sexu	Rate	2002	0.0712	0.08616	0.0554
Deaths	Global	Both	65-69 yea	Other sexu	Number	2002	108.89	129.313	86.9687
Deaths	Global	Both	65-69 yea	Other sexu	Rate	2002	0.06835	0.08117	0.05459
Deaths	Global	Both	50-69 yea	Other sexu	Number	2002	614.913	704.888	466.29
Deaths	Global	Both	50-69 yea	Other sexu	Rate	2002	0.07213	0.08268	0.05469
Deaths	Global	Both	50-54 yea	Sexually tr	Number	2005	593.78	712.889	456.174
Deaths	Global	Both	50-54 yea	Sexually tr	Rate	2005	0.18747	0.22507	0.14402
Deaths	Global	Both	55-59 yea	Sexually tr	Number	2005	701.848	895.073	505.531
Deaths	Global	Both	55-59 yea	Sexually tr	Rate	2005	0.28121	0.35863	0.20255
Deaths	Global	Both	60-64 yea	Sexually tr	Number	2005	488.011	600.296	379.638
Deaths	Global	Both	60-64 yea	Sexually tr	Rate	2005	0.25025	0.30783	0.19468
Deaths	Global	Both	65-69 yea	Sexually tr	Number	2005	481.28	577.17	406.662
Deaths	Global	Both	65-69 yea	Sexually tr	Rate	2005	0.28548	0.34236	0.24122
Deaths	Global	Both	50-69 yea	Sexually tr	Number	2005	2264.92	2658.07	1800.07
Deaths	Global	Both	50-69 yea	Sexually tr	Rate	2005	0.24356	0.28584	0.19357
Deaths	Global	Both	50-54 yea	Syphilis	Number	2005	167.871	243.783	93.1835
Deaths	Global	Both	50-54 yea	Syphilis	Rate	2005	0.053	0.07697	0.02942
Deaths	Global	Both	55-59 yea	Syphilis	Number	2005	191.189	312.396	83.1442
Deaths	Global	Both	55-59 yea	Syphilis	Rate	2005	0.0766	0.12517	0.03331
Deaths	Global	Both	60-64 yea	Syphilis	Number	2005	153.321	227.227	91.4495
Deaths	Global	Both	60-64 yea	Syphilis	Rate	2005	0.07862	0.11652	0.04689
Deaths	Global	Both	65-69 yea	Syphilis	Number	2005	107.767	160.872	84.9258
Deaths	Global	Both	65-69 yea	Syphilis	Rate	2005	0.06392	0.09542	0.05038
Deaths	Global	Both	50-69 yea	Syphilis	Number	2005	620.147	866.445	367.526
Deaths	Global	Both	50-69 yea	Syphilis	Rate	2005	0.06669	0.09317	0.03952
Deaths	Global	Both	50-54 yea	Chlamydia	Number	2005	62.0479	72.4025	50.9431
Deaths	Global	Both	50-54 yea	Chlamydia	Rate	2005	0.01959	0.02286	0.01608
Deaths	Global	Both	55-59 yea	Chlamydia	Number	2005	78.465	95.0901	59.2101
Deaths	Global	Both	55-59 yea	Chlamydia	Rate	2005	0.03144	0.0381	0.02372
Deaths	Global	Both	60-64 yea	Chlamydia	Number	2005	52.9312	62.4089	41.5496
Deaths	Global	Both	60-64 yea	Chlamydia	Rate	2005	0.02714	0.032	0.02131
Deaths	Global	Both	65-69 yea	Chlamydia	Number	2005	40.3107	48.2153	31.6102
Deaths	Global	Both	65-69 yea	Chlamydia	Rate	2005	0.02391	0.0286	0.01875
Deaths	Global	Both	50-69 yea	Chlamydia	Number	2005	233.755	266.036	186.886
Deaths	Global	Both	50-69 yea	Chlamydia	Rate	2005	0.02514	0.02861	0.0201
Deaths	Global	Both	50-54 yea	Gonococca	Number	2005	198.7	231.129	162.935
Deaths	Global	Both	50-54 yea	Gonococca	Rate	2005	0.06273	0.07297	0.05144
Deaths	Global	Both	55-59 yea	Gonococca	Number	2005	232.895	280.084	175.045
Deaths	Global	Both	55-59 yea	Gonococca	Rate	2005	0.09331	0.11222	0.07013
Deaths	Global	Both	60-64 yea	Gonococca	Number	2005	153.964	179.115	125.869
Deaths	Global	Both	60-64 yea	Gonococca	Rate	2005	0.07895	0.09185	0.06454
Deaths	Global	Both	65-69 yea	Gonococca	Number	2005	229.598	272.477	185.399
Deaths	Global	Both	65-69 yea	Gonococca	Rate	2005	0.13619	0.16162	0.10997
Deaths	Global	Both	50-69 yea	Gonococca	Number	2005	815.156	925.834	651.876
Deaths	Global	Both	50-69 yea	Gonococca	Rate	2005	0.08766	0.09956	0.0701
Deaths	Global	Both	50-54 yea	Other sexu	Number	2005	165.162	194.814	127.895
Deaths	Global	Both	50-54 yea	Other sexu	Rate	2005	0.05214	0.06151	0.04038
Deaths	Global	Both	55-59 yea	Other sexu	Number	2005	199.3	243.881	145.605
Deaths	Global	Both	55-59 yea	Other sexu	Rate	2005	0.07985	0.09771	0.05834
Deaths	Global	Both	60-64 yea	Other sexu	Number	2005	127.795	152.325	101.453
Deaths	Global	Both	60-64 yea	Other sexu	Rate	2005	0.06553	0.07811	0.05202
Deaths	Global	Both	65-69 yea	Other sexu	Number	2005	103.605	123.74	84.9774
Deaths	Global	Both	65-69 yea	Other sexu	Rate	2005	0.06146	0.0734	0.05041
Deaths	Global	Both	50-69 yea	Other sexu	Number	2005	595.862	686.412	463.203
Deaths	Global	Both	50-69 yea	Other sexu	Rate	2005	0.06408	0.07381	0.04981
Deaths	Global	Both	50-54 yea	HIV/AIDS	Number	2005	125610	134488	115398
Deaths	Global	Both	50-54 yea	HIV/AIDS	Rate	2005	39.6573	42.4601	36.4333
Deaths	Global	Both	55-59 yea	HIV/AIDS	Number	2005	66471.5	70957.9	62272.7
Deaths	Global	Both	55-59 yea	HIV/AIDS	Rate	2005	26.6328	28.4304	24.9505
Deaths	Global	Both	60-64 yea	HIV/AIDS	Number	2005	38714.5	41638.7	36155.8
Deaths	Global	Both	60-64 yea	HIV/AIDS	Rate	2005	19.8525	21.352	18.5404
Deaths	Global	Both	65-69 yea	HIV/AIDS	Number	2005	23765.8	25586.8	22228.5
Deaths	Global	Both	65-69 yea	HIV/AIDS	Rate	2005	14.097	15.1772	13.1852
Deaths	Global	Both	50-69 yea	HIV/AIDS	Number	2005	254562	271776	237717
Deaths	Global	Both	50-69 yea	HIV/AIDS	Rate	2005	27.3746	29.2258	25.5631
Deaths	Global	Both	50-54 yea	HIV/AIDS	Number	2003	117078	129202	102238
Deaths	Global	Both	50-54 yea	HIV/AIDS	Rate	2003	39.6756	43.7841	34.6466
Deaths	Global	Both	55-59 yea	HIV/AIDS	Number	2003	63849.3	69794.9	57540.6
Deaths	Global	Both	55-59 yea	HIV/AIDS	Rate	2003	28.0814	30.6964	25.3068
Deaths	Global	Both	60-64 yea	HIV/AIDS	Number	2003	38055.2	41534.5	34391.3
Deaths	Global	Both	60-64 yea	HIV/AIDS	Rate	2003	19.849	21.6637	17.9379
Deaths	Global	Both	65-69 yea	HIV/AIDS	Number	2003	23497.3	25902.4	21203.2
Deaths	Global	Both	65-69 yea	HIV/AIDS	Rate	2003	14.445	15.9235	13.0347
Deaths	Global	Both	50-69 yea	HIV/AIDS	Number	2003	242480	266114	216342
Deaths	Global	Both	50-69 yea	HIV/AIDS	Rate	2003	27.6535	30.3488	24.6726
Deaths	Global	Both	50-54 yea	Sexually tr	Number	2003	615.37	745.274	455.637
Deaths	Global	Both	50-54 yea	Sexually tr	Rate	2003	0.20854	0.25256	0.15441
Deaths	Global	Both	55-59 yea	Sexually tr	Number	2003	725.437	943.508	505.81
Deaths	Global	Both	55-59 yea	Sexually tr	Rate	2003	0.31905	0.41496	0.22246
Deaths	Global	Both	60-64 yea	Sexually tr	Number	2003	534.097	652.009	411.099
Deaths	Global	Both	60-64 yea	Sexually tr	Rate	2003	0.27858	0.34008	0.21442
Deaths	Global	Both	65-69 yea	Sexually tr	Number	2003	510.863	611.005	426.442
Deaths	Global	Both	65-69 yea	Sexually tr	Rate	2003	0.31405	0.37561	0.26216
Deaths	Global	Both	50-69 yea	Sexually tr	Number	2003	2385.77	2813.55	1877.23
Deaths	Global	Both	50-69 yea	Sexually tr	Rate	2003	0.27208	0.32087	0.21409
Deaths	Global	Both	50-54 yea	Syphilis	Number	2003	185.743	269.216	105.153
Deaths	Global	Both	50-54 yea	Syphilis	Rate	2003	0.06294	0.09123	0.03563
Deaths	Global	Both	55-59 yea	Syphilis	Number	2003	212.444	355.853	95.7167
Deaths	Global	Both	55-59 yea	Syphilis	Rate	2003	0.09343	0.15651	0.0421
Deaths	Global	Both	60-64 yea	Syphilis	Number	2003	179.529	265.592	109.668
Deaths	Global	Both	60-64 yea	Syphilis	Rate	2003	0.09364	0.13853	0.0572
Deaths	Global	Both	65-69 yea	Syphilis	Number	2003	126.961	182.283	101.713
Deaths	Global	Both	65-69 yea	Syphilis	Rate	2003	0.07805	0.11206	0.06253
Deaths	Global	Both	50-69 yea	Syphilis	Number	2003	704.677	1012.61	428.442
Deaths	Global	Both	50-69 yea	Syphilis	Rate	2003	0.08036	0.11548	0.04886
Deaths	Global	Both	50-54 yea	Chlamydia	Number	2003	62.4947	73.0619	51.0482
Deaths	Global	Both	50-54 yea	Chlamydia	Rate	2003	0.02118	0.02476	0.0173
Deaths	Global	Both	55-59 yea	Chlamydia	Number	2003	78.2065	95.5919	58.3485
Deaths	Global	Both	55-59 yea	Chlamydia	Rate	2003	0.0344	0.04204	0.02566
Deaths	Global	Both	60-64 yea	Chlamydia	Number	2003	56.116	66.3301	43.9391
Deaths	Global	Both	60-64 yea	Chlamydia	Rate	2003	0.02927	0.0346	0.02292
Deaths	Global	Both	65-69 yea	Chlamydia	Number	2003	41.4109	48.9447	32.6704
Deaths	Global	Both	65-69 yea	Chlamydia	Rate	2003	0.02546	0.03009	0.02008

Deaths	Global	Both	55-59 yea	Syphilis	Rate	2006	0.07185	0.11534	0.0324
Deaths	Global	Both	60-64 yea	Syphilis	Number	2006	147.994	214.86	88.8031
Deaths	Global	Both	60-64 yea	Syphilis	Rate	2006	0.07442	0.10804	0.04465
Deaths	Global	Both	65-69 yea	Syphilis	Number	2006	103.407	153.499	81.0661
Deaths	Global	Both	65-69 yea	Syphilis	Rate	2006	0.06048	0.08978	0.04741
Deaths	Global	Both	50-69 yea	Syphilis	Number	2006	601.734	820.507	358.917
Deaths	Global	Both	50-69 yea	Syphilis	Rate	2006	0.06289	0.08576	0.03751
Deaths	Global	Both	50-54 yea	Chlamydia	Number	2006	61.5929	71.953	51.3167
Deaths	Global	Both	50-54 yea	Chlamydia	Rate	2006	0.01899	0.02218	0.01582
Deaths	Global	Both	55-59 yea	Chlamydia	Number	2006	80.6857	97.9321	60.8364
Deaths	Global	Both	55-59 yea	Chlamydia	Rate	2006	0.03073	0.0373	0.02317
Deaths	Global	Both	60-64 yea	Chlamydia	Number	2006	52.9275	62.3425	41.3108
Deaths	Global	Both	60-64 yea	Chlamydia	Rate	2006	0.02661	0.03135	0.02077
Deaths	Global	Both	65-69 yea	Chlamydia	Number	2006	40.8476	48.5921	31.5861
Deaths	Global	Both	65-69 yea	Chlamydia	Rate	2006	0.02389	0.02842	0.01847
Deaths	Global	Both	50-69 yea	Chlamydia	Number	2006	236.054	268.137	186.815
Deaths	Global	Both	50-69 yea	Chlamydia	Rate	2006	0.02467	0.02803	0.01953
Deaths	Global	Both	50-54 yea	Gonococca	Number	2006	197.022	228.369	161.958
Deaths	Global	Both	50-54 yea	Gonococca	Rate	2006	0.06074	0.0704	0.04993
Deaths	Global	Both	55-59 yea	Gonococca	Number	2006	239.07	288.148	178.731
Deaths	Global	Both	55-59 yea	Gonococca	Rate	2006	0.09106	0.10975	0.06807
Deaths	Global	Both	60-64 yea	Gonococca	Number	2006	153.251	178.72	122.265
Deaths	Global	Both	60-64 yea	Gonococca	Rate	2006	0.07706	0.08987	0.06148
Deaths	Global	Both	65-69 yea	Gonococca	Number	2006	231.928	273.583	183.153
Deaths	Global	Both	65-69 yea	Gonococca	Rate	2006	0.13565	0.16001	0.10712
Deaths	Global	Both	50-69 yea	Gonococca	Number	2006	821.272	933.2	650.094
Deaths	Global	Both	50-69 yea	Gonococca	Rate	2006	0.08584	0.09754	0.06795
Deaths	Global	Both	50-54 yea	Other sexu	Number	2006	163.312	191.065	126.951
Deaths	Global	Both	50-54 yea	Other sexu	Rate	2006	0.05035	0.0589	0.03914
Deaths	Global	Both	55-59 yea	Other sexu	Number	2006	203.737	248.222	148.15
Deaths	Global	Both	55-59 yea	Other sexu	Rate	2006	0.0776	0.09454	0.05643
Deaths	Global	Both	60-64 yea	Other sexu	Number	2006	127.879	151.744	100.5
Deaths	Global	Both	60-64 yea	Other sexu	Rate	2006	0.0643	0.0763	0.05054
Deaths	Global	Both	65-69 yea	Other sexu	Number	2006	104.586	124.125	83.6737
Deaths	Global	Both	65-69 yea	Other sexu	Rate	2006	0.06117	0.0726	0.04894
Deaths	Global	Both	50-69 yea	Other sexu	Number	2006	599.514	686.592	463.225
Deaths	Global	Both	50-69 yea	Other sexu	Rate	2006	0.06266	0.07176	0.04842
Deaths	Global	Both	50-54 yea	HIV/AIDS	Number	2007	115941	126048	106747
Deaths	Global	Both	50-54 yea	HIV/AIDS	Rate	2007	35.028	38.0816	32.2504
Deaths	Global	Both	55-59 yea	HIV/AIDS	Number	2007	63844.7	69713.5	58626.9
Deaths	Global	Both	55-59 yea	HIV/AIDS	Rate	2007	23.4784	25.6365	21.5596
Deaths	Global	Both	60-64 yea	HIV/AIDS	Number	2007	35608.9	39186.9	32410.1
Deaths	Global	Both	60-64 yea	HIV/AIDS	Rate	2007	17.1561	18.8799	15.6149
Deaths	Global	Both	65-69 yea	HIV/AIDS	Number	2007	21312.8	23466.6	19308.5
Deaths	Global	Both	65-69 yea	HIV/AIDS	Rate	2007	12.3369	13.5836	11.1767
Deaths	Global	Both	50-69 yea	HIV/AIDS	Number	2007	236707	257125	217946
Deaths	Global	Both	50-69 yea	HIV/AIDS	Rate	2007	24.0742	26.1508	22.1661
Deaths	Global	Both	50-54 yea	Sexually tr	Number	2007	578.294	689.475	441.033
Deaths	Global	Both	50-54 yea	Sexually tr	Rate	2007	0.17471	0.2083	0.13324
Deaths	Global	Both	55-59 yea	Sexually tr	Number	2007	725.766	906.874	528.955
Deaths	Global	Both	55-59 yea	Sexually tr	Rate	2007	0.26689	0.3335	0.19452
Deaths	Global	Both	60-64 yea	Sexually tr	Number	2007	484.164	583.869	377.295
Deaths	Global	Both	60-64 yea	Sexually tr	Rate	2007	0.23327	0.2813	0.18178
Deaths	Global	Both	65-69 yea	Sexually tr	Number	2007	471.25	554.689	395.89
Deaths	Global	Both	65-69 yea	Sexually tr	Rate	2007	0.27278	0.32108	0.22916
Deaths	Global	Both	50-69 yea	Sexually tr	Number	2007	2259.47	2614.1	1801.72
Deaths	Global	Both	50-69 yea	Sexually tr	Rate	2007	0.2298	0.26587	0.18324
Deaths	Global	Both	50-54 yea	Syphilis	Number	2007	158.588	223.474	89.5425
Deaths	Global	Both	50-54 yea	Syphilis	Rate	2007	0.04791	0.06752	0.02705
Deaths	Global	Both	55-59 yea	Syphilis	Number	2007	187.594	294.879	85.0361
Deaths	Global	Both	55-59 yea	Syphilis	Rate	2007	0.06899	0.10844	0.03127
Deaths	Global	Both	60-64 yea	Syphilis	Number	2007	145.55	210.633	88.1255
Deaths	Global	Both	60-64 yea	Syphilis	Rate	2007	0.07012	0.10148	0.04246
Deaths	Global	Both	65-69 yea	Syphilis	Number	2007	99.8724	148.156	79.36
Deaths	Global	Both	65-69 yea	Syphilis	Rate	2007	0.05781	0.08576	0.04594
Deaths	Global	Both	50-69 yea	Syphilis	Number	2007	591.604	797.832	352.51
Deaths	Global	Both	50-69 yea	Syphilis	Rate	2007	0.06017	0.08114	0.03585
Deaths	Global	Both	50-54 yea	Chlamydia	Number	2007	61.3679	71.3881	49.9783
Deaths	Global	Both	50-54 yea	Chlamydia	Rate	2007	0.01854	0.02157	0.0151
Deaths	Global	Both	55-59 yea	Chlamydia	Number	2007	83.1702	100.29	60.9757
Deaths	Global	Both	55-59 yea	Chlamydia	Rate	2007	0.03059	0.03688	0.02242
Deaths	Global	Both	60-64 yea	Chlamydia	Number	2007	53.8151	63.469	41.8333
Deaths	Global	Both	60-64 yea	Chlamydia	Rate	2007	0.02593	0.03058	0.02015
Deaths	Global	Both	65-69 yea	Chlamydia	Number	2007	40.2368	47.3374	31.3792
Deaths	Global	Both	65-69 yea	Chlamydia	Rate	2007	0.02329	0.0274	0.01816
Deaths	Global	Both	50-69 yea	Chlamydia	Number	2007	238.59	270.207	185.597
Deaths	Global	Both	50-69 yea	Chlamydia	Rate	2007	0.02427	0.02748	0.01888
Deaths	Global	Both	50-54 yea	Gonococca	Number	2007	196.151	227.381	159.2
Deaths	Global	Both	50-54 yea	Gonococca	Rate	2007	0.05926	0.0687	0.0481
Deaths	Global	Both	55-59 yea	Gonococca	Number	2007	246.043	294.231	180.343
Deaths	Global	Both	55-59 yea	Gonococca	Rate	2007	0.09048	0.1082	0.06632
Deaths	Global	Both	60-64 yea	Gonococca	Number	2007	155.344	181.363	123.536
Deaths	Global	Both	60-64 yea	Gonococca	Rate	2007	0.07484	0.08738	0.05952
Deaths	Global	Both	65-69 yea	Gonococca	Number	2007	228.188	265.377	183.134
Deaths	Global	Both	65-69 yea	Gonococca	Rate	2007	0.13209	0.15361	0.10601
Deaths	Global	Both	50-69 yea	Gonococca	Number	2007	825.726	929.218	650.253
Deaths	Global	Both	50-69 yea	Gonococca	Rate	2007	0.08398	0.09451	0.06613
Deaths	Global	Both	50-54 yea	Other sexu	Number	2007	162.187	189.81	123.899
Deaths	Global	Both	50-54 yea	Other sexu	Rate	2007	0.049	0.05735	0.03743
Deaths	Global	Both	55-59 yea	Other sexu	Number	2007	208.959	251.58	151.914
Deaths	Global	Both	55-59 yea	Other sexu	Rate	2007	0.07684	0.09252	0.05587
Deaths	Global	Both	60-64 yea	Other sexu	Number	2007	129.454	152.914	100.275
Deaths	Global	Both	60-64 yea	Other sexu	Rate	2007	0.06237	0.07367	0.04831
Deaths	Global	Both	65-69 yea	Other sexu	Number	2007	102.953	120.238	83.5578
Deaths	Global	Both	65-69 yea	Other sexu	Rate	2007	0.05959	0.0696	0.04837
Deaths	Global	Both	50-69 yea	Other sexu	Number	2007	603.553	687.719	460.386
Deaths	Global	Both	50-69 yea	Other sexu	Rate	2007	0.06138	0.06994	0.04682
Deaths	Global	Both	50-54 yea	HIV/AIDS	Number	2008	109364	120734	99213.6
Deaths	Global	Both	50-54 yea	HIV/AIDS	Rate	2008	32.5731	35.9595	29.5498
Deaths	Global	Both	55-59 yea	HIV/AIDS	Number	2008	60722.7	67426.7	55136
Deaths	Global	Both	55-59 yea	HIV/AIDS	Rate	2008	21.5325	23.9098	19.5515
Deaths	Global	Both	60-64 yea	HIV/AIDS	Number	2008	33593.1	37751.2	30169.6
Deaths	Global	Both	60-64 yea	HIV/AIDS	Rate	2008	15.4696	17.3844	13.8931
Deaths	Global	Both	65-69 yea	HIV/AIDS	Number	2008	19808.5	22316.9	17721.6
Deaths	Global	Both	65-69 yea	HIV/AIDS	Rate	2008	11.36	12.7986	10.1632
Deaths	Global	Both	50-69 yea	HIV/AIDS	Number	2008	223489	246496	203773
Deaths	Global	Both	50-69 yea	HIV/AIDS	Rate	2008	22.1434	24.423	20.1899
Deaths	Global	Both	50-54 yea	Sexually tr	Number	2011	575.002	680.843	441.125
Deaths	Global	Both	50-54 yea	Sexually tr	Rate	2011	0.1646	0.1949	0.12628
Deaths	Global	Both	55-59 yea	Sexually tr	Number	2011	719.9	876.872	527.042
Deaths	Global	Both	55-59 yea	Sexually tr	Rate	2011	0.23148	0.28196	0.16947
Deaths	Global	Both	60-64 yea	Sexually tr	Number	2011	495.747	590.72	395.622
Deaths	Global	Both	60-64 yea	Sexually tr	Rate	2011	0.19706	0.23481	0.15726
Deaths	Global	Both	65-69 yea	Sexually tr	Number	2011	466.637	559.601	386.802
Deaths	Global	Both	65-69 yea	Sexually tr	Rate	2011	0.25608	0.3071	0.21227
Deaths	Global	Both	50-69 yea	Sexually tr	Number	2011	2257.28	2574.24	1767.47
Deaths	Global	Both	50-69 yea	Sexually tr	Rate	2011	0.20631	0.23528	0.16154
Deaths	Global	Both	50-54 yea	Syphilis	Number	2011	149.399	210.018	84.8112
Deaths	Global	Both	50-54 yea	Syphilis	Rate	2011	0.04277	0.06012	0.02428
Deaths	Global	Both	55-59 yea	Syphilis	Number	2011	182.956	279.833	83.9395
Deaths	Global	Both	55-59 yea	Syphilis	Rate	2011	0.05883	0.08998	0.02699
Deaths	Global	Both	60-64 yea	Syphilis	Number	2011	143.085	200.651	86.7071
Deaths	Global	Both	60-64 yea	Syphilis	Rate	2011	0.05688	0.07976	0.03447
Deaths	Global	Both	65-69 yea	Syphilis	Number	2011	95.596	140.232	75.2547
Deaths	Global	Both	65-69 yea	Syphilis	Rate	2011	0.05246	0.07696	0.0413
Deaths	Global	Both	50-69 yea	Syphilis	Number	2011	571.036	743.803	347.762
Deaths	Global	Both	50-69 yea	Syphilis	Rate	2011	0.05219	0.06798	0.03178
Deaths	Global	Both	50-54 yea	Chlamydia	Number	2011	62.8603	72.9237	51.5063
Deaths	Global	Both	50-54 yea	Chlamydia	Rate	2011	0.01799	0.02088	0.01474
Deaths	Global	Both	55-59 yea	Chlamydia	Number	2011	83.0933	99.678	63.6167
Deaths	Global	Both	55-59 yea	Chlamydia	Rate	2011	0.02672	0.03205	0.02046
Deaths	Global	Both	60-64 yea	Chlamydia	Number	2011	56.4965	66.9773	45.2729
Deaths	Global	Both	60-64 yea	Chlamydia	Rate	2011	0.02246	0.02662	0.018
Deaths	Global	Both	65-69 yea	Chlamydia	Number	2011	40.3206	48.1776	31.4957
Deaths	Global	Both	65-69 yea	Chlamydia	Rate	2011	0.02213	0.02644	0.01728
Deaths	Global	Both	50-69 yea	Chlamydia	Number	2011	242.771	270.958	193.763
Deaths	Global	Both	50-69 yea	Chlamydia	Rate	2011	0.02219	0.02477	0.01771
Deaths	Global	Both	50-54 yea	Gonococca	Number	2011	199.987	231.345	165.889
Deaths	Global	Both	50-54 yea	Gonococca	Rate	2011	0.05725	0.06623	0.04749
Deaths	Global	Both	55-59 yea	Gonococca	Number	2011	245.605	293.005	185.891
Deaths	Global	Both	55-59 yea	Gonococca	Rate	2011	0.07897	0.09422	0.05977
Deaths	Global	Both	60-64 yea	Gonococca	Number	2011	162.856	190.202	133.284
Deaths	Global	Both	60-64 yea	Gonococca	Rate	2011	0.06474	0.07561	0.05298
Deaths	Global	Both	65-69 yea	Gonococca	Number	2011	227.531	268.707	180.663
Deaths	Global	Both	65-69 yea	Gonococca	Rate	2011	0.12486	0.14746	0.09914
Deaths	Global	Both	50-69 yea	Gonococca	Number	2011	835.979	930.292	661.394
Deaths	Global	Both	50-69 yea	Gonococca	Rate	2011	0.07641	0.08503	0.06045
Deaths	Global	Both	50-54 yea	Other sexu	Number	2011	162.756	191.496	127.079
Deaths	Global	Both	50-54 yea	Other sexu	Rate	2011	0.04659	0.05482	0.03638
Deaths	Global	Both	55-59 yea	Other sexu	Number	2011	208.246	248.275	152.129
Deaths	Global	Both	55-59 yea	Other sexu	Rate	2011	0.06696	0.07983	0.04892
Deaths	Global	Both	60-64 yea	Other sexu	Number	2011	133.308	157.294	106.296
Deaths	Global	Both	60-64 yea	Other sexu	Rate	2011	0.05299	0.06252	0.04225
Deaths	Global	Both	65-69 yea	Other sexu	Number	2011	103.189	122.744	82.9191
Deaths	Global	Both	65-69 yea	Other sexu	Rate	2011	0.05663	0.06736	0.0455
Deaths	Global	Both	50-69 yea	Other sexu	Number	2011	607.499	683.088	468.371
Deaths	Global	Both	50-69 yea	Other sexu	Rate	2011	0.05552	0.06243	0.04281
Deaths	Global	Both	50-54 yea	HIV/AIDS	Number	2011	94724.3	106861	85593.1
Deaths	Global	Both	50-54 yea	HIV/AIDS	Rate	2011	27.1166	30.591	24.5026
Deaths	Global	Both	55-59 yea	HIV/AIDS	Number	2011	53730.2	62023.8	48535.2
Deaths	Global	Both	55-59 yea	HIV/AIDS	Rate	2011	17.277	19.9438	15.6065
Deaths	Global	Both	60-64 yea	HIV/AIDS	Number	2011	30293.5	35377.6	27052.4
Deaths	Global	Both	60-64 yea	HIV/AIDS	Rate	2011	12.0417	14.0626	10.7533
Deaths	Global	Both	65-69 yea	HIV/AIDS	Number	2011	17009.9	19840.8	15216.4
Deaths	Global	Both	65-69 yea	HIV/AIDS	Rate	2011	9.33471	10.8883	8.35046
Deaths	Global	Both	50-69 yea	HIV/AIDS	Number	2011	195758	222943	178055
Deaths	Global	Both	50-69 yea	HIV/AIDS	Rate	2011	17.892	20.3766	16.274
Deaths	Global	Both	50-54 yea	Sexually tr	Number	2009	569.147	672.642	436.56
Deaths	Global	Both	50-54 yea	Sexually tr	Rate	2009	0.16771	0.19821	0.12864
Deaths	Global	Both	55-59 yea	Sexually tr	Number	2009	712.295	868.602	520.004
Deaths	Global	Both	55-59 yea	Sexually tr	Rate	2009	0.24468	0.29837	0.17862
Deaths	Global	Both	60-64 yea	Sexually tr	Number	2009	479.172	576.345	375.638
Deaths	Global	Both	60-64 yea	Sexually tr	Rate	2009	0.20917	0.25158	0.16397
Deaths	Global	Both	65-69 yea	Sexually tr	Number	2009	456.454	546.685	385.917
Deaths	Global	Both	65-69 yea	Sexually tr	Rate	2009	0.25884	0.31001	0.21884
Deaths	Global	Both	50-69 yea	Sexually tr	Number	2009	2217.07	2535.12	1768.84
Deaths	Global	Both	50-69 yea	Sexually tr	Rate	2009	0.21402	0.24472	0.17075
Deaths	Global	Both	50-54 yea	Syphilis	Number	2009	153.386	218.799	86.6378
Deaths	Global	Both	50-54 yea	Syphilis	Rate	2009	0.0452	0.06447	0.02553
Deaths	Global	Both	55-59 yea	Syphilis	Number	2009	185.289	283.605	83.6135
Deaths	Global	Both	55-59 yea	Syphilis	Rate	2009	0.06365	0.09742	0.02872
Deaths	Global	Both	60-64 yea	Syphilis	Number	2009	144.085	204.768	87.0594
Deaths	Global	Both	60-64 yea	Syphilis	Rate	2009	0.0629	0.08938	0.038
Deaths	Global	Both	65-69 yea	Syphilis	Number	2009	96.3116	145.724	76.3819
Deaths	Global	Both	65-69 yea	Syphilis	Rate	2009	0.05462	0.08264	0.04331
Deaths	Global	Both	50-69 yea	Syphilis	Number	2009	579.072	769.17	344.238
Deaths	Global	Both	50-69 yea	Syphilis	Rate	2009	0.0559	0.07425	0.03323
Deaths	Global	Both	50-54 yea	Chlamydia	Number	2009	60.9906	70.4059	49.597
Deaths	Global	Both	50-54 yea	Chlamydia	Rate	2009	0.01797	0.02075	0.01461
Deaths	Global	Both	55-59 yea	Chlamydia	Number	2009	81.3373	97.1376	61.5736
Deaths	Global	Both	55-59 yea	Chlamydia	Rate	2009	0.02794	0.03337	0.02115
Deaths	Global	Both	60-64 yea	Chlamydia	Number	2009	53.309	62.5698	41.2312
Deaths	Global	Both	60-64 yea	Chlamydia	Rate	2009	0.02327	0.02731	0.018
Deaths	Global	Both	65-69 yea	Chlamydia	Number	2009	39.0322	46.3858	30.3489
Deaths	Global	Both	65-69 yea	Chlamydia	Rate	2009	0.02213	0.0263	0.01721
Deaths	Global	Both	50-69 yea	Chlamydia	Number	2009	234.669	262.045	183.012
Deaths	Global	Both	50-69 yea	Chlamydia	Rate	2009	0.02265	0.0253	0.01767
Deaths	Global	Both	50-54 yea	Gonococca	Number	2009	194.653	223.236	158.258
Deaths	Global	Both	50-54 yea	Gonococca	Rate	2009	0.05736	0.06578	0.04663
Deaths	Global	Both	55-59 yea	Gonococca	Number	2009	240.803	285.78	181.076
Deaths	Global	Both	55-59 yea	Gonococca	Rate	2009	0.08272	0.09817	0.0622
Deaths	Global	Both	60-64 yea	Gonococca	Number	2009	154.406	179.337	123.015
Deaths	Global	Both	60-64 yea	Gonococca	Rate	2009	0.0674	0.07828	0.0537
Deaths	Global	Both	65-69 yea	Gonococca	Number	2009	221.039	259.228	176.006
Deaths	Global	Both	65-69 yea	Gonococca	Rate	2009	0.12535	0.147	0.09981
Deaths	Global	Both	50-69 yea	Gonococca	Number	2009	810.9	907.161	635.373
Deaths	Global	Both	50-69 yea	Gonococca	Rate	2009	0.07828	0.08757	0.06134
Deaths	Global	Both	50-54 yea	Other sexu	Number	2009	160.117	186.796	123.067
Deaths	Global	Both	50-54 yea	Other sexu	Rate	2009	0.04718	0.05504	0.03626
Deaths	Global	Both	55-59 yea	Other sexu	Number	2009	204.866	245.065	149.28
Deaths	Global	Both	55-59 yea	Other sexu	Rate	2009	0.07037	0.08418	0.05128
Deaths	Global	Both	60-64 yea	Other sexu	Number	2009	127.371	149.691	101.043
Deaths	Global	Both	60-64 yea	Other sexu	Rate	2009	0.0556	0.06534	0.04411
Deaths	Global	Both	65-69 yea	Other sexu	Number	2009	100.071	117.924	80.5552
Deaths	Global	Both	65-69 yea	Other sexu	Rate	2009	0.05675	0.06687	0.04568
Deaths	Global	Both	50-69 yea	Other sexu	Number	2009	592.426	663.598	457.035
Deaths	Global	Both	50-69 yea	Other sexu	Rate	2009	0.05719	0.06406	0.04412
Deaths	Global	Both	50-54 yea	HIV/AIDS	Number	2009	102852	115160	92421.8
Deaths	Global	Both	50-54 yea	HIV/AIDS	Rate	2009	30.3077	33.9344	27.2342
Deaths	Global	Both	55-59 yea	HIV/AIDS	Number	2009	57847.6	65583	52073.2
Deaths	Global	Both	55-59 yea	HIV/AIDS	Rate	2009	19.871	22.5281	17.8874
Deaths	Global	Both	60-64 yea	HIV/AIDS	Number	2009	31786.6	36528	28407.9
Deaths	Global	Both	60-64 yea	HIV/AIDS	Rate	2009	13.8754	15.9451	12.4005
Deaths	Global	Both	65-69 yea	HIV/AIDS	Number	2009	18484.4	21302.4	16457
Deaths	Global	Both	65-69 yea	HIV/AIDS	Rate	2009	10.4821	12.0801	9.33237
Deaths	Global	Both	50-69 yea	HIV/AIDS	Number	2009	210971	236722	190995
Deaths	Global	Both	50-69 yea	HIV/AIDS	Rate	2009	20.3658	22.8518	18.4375
Deaths	Global	Both	50-54 yea	Sexually tr	Number	2013	598.647	705.564	456.35
Deaths	Global	Both	50-54 yea	Sexually tr	Rate	2013	0.16146	0.19029	0.12308
Deaths	Global	Both	55-59 yea	Sexually tr	Number	2013	729.657	883.786	525.769
Deaths	Global	Both	55-59 yea	Sexually tr	Rate	2013	0.22631	0.27411	0.16307
Deaths	Global	Both	60-64 yea	Sexually tr	Number	2013	519.713	614.12	407.435
Deaths	Global	Both	60-64 yea	Sexually tr	Rate	2013	0.19218	0.22709	0.15066
Deaths	Global	Both	65-69 yea	Sexually tr	Number	2013	471.475	558.067	385.943
Deaths	Global	Both	65-69 yea	Sexually tr	Rate	2013	0.23591	0.27924	0.19311
Deaths	Global	Both	50-69 yea	Sexually tr	Number	2013	2319.49	2618.05	1790.24
Deaths	Global	Both	50-69 yea	Sexually tr	Rate	2013	0.19936	0.22502	0.15387
Deaths	Global	Both	50-54 yea	Syphilis	Number	2013	149.547	205.41	85.8126
Deaths	Global	Both	50-54 yea	Syphilis	Rate	2013	0.04033	0.0554	0.02314
Deaths	Global	Both	55-59 yea	Syphilis	Number	2013	179.425	274.767	82.6779
Deaths	Global	Both	55-59 yea	Syphilis	Rate	2013	0.05565	0.08522	0.02564
Deaths	Global	Both	60-64 yea	Syphilis	Number	2013	145.137	207.979	88.9255
Deaths	Global	Both	60-64 yea	Syphilis	Rate	2013	0.05367	0.07691	0.03288
Deaths	Global	Both	65-69 yea	Syphilis	Number	2013	95.0031	137.179	76.3314
Deaths	Global	Both	65-69 yea	Syphilis	Rate	2013	0.04754	0.06864	0.03819
Deaths	Global	Both	50-69 yea	Syphilis	Number	2013	569.113	739.547	347.186
Deaths	Global	Both	50-69 yea	Syphilis	Rate	2013	0.04891	0.06356	0.02984
Deaths	Global	Both	50-54 yea	Chlamydia	Number	2013	66.7799	78.3502	52.956
Deaths	Global	Both	50-54 yea	Chlamydia	Rate	2013	0.01801	0.02113	0.01428
Deaths	Global	Both	55-59 yea	Chlamydia	Number	2013	85.4932	103.775	64.9304
Deaths	Global	Both	55-59 yea	Chlamydia	Rate	2013	0.02652	0.03219	0.02014
Deaths	Global	Both	60-64 yea	Chlamydia	Number	2013	60.233	71.0971	47.4309
Deaths	Global	Both	60-64 yea	Chlamydia	Rate	2013	0.02227	0.02629	0.01754
Deaths	Global	Both	65-69 yea	Chlamydia	Number	2013	40.9671	48.5971	31.9702
Deaths	Global	Both	65-69 yea	Chlamydia	Rate	2013	0.0205	0.02432	0.016
Deaths	Global	Both	50-69 yea	Chlamydia	Number	2013	253.473	284.788	200.217
Deaths	Global	Both	50-69 yea	Chlamydia	Rate	2013	0.02179	0.02448	0.01721
Deaths	Global	Both	50-54 yea	Gonococca	Number	2013	211.755	247.358	170.036
Deaths	Global	Both	50-54 yea	Gonococca	Rate	2013	0.05711	0.06671	0.04586
Deaths	Global	Both	55-59 yea	Gonococca	Number	2013	252.115	302.717	188.737
Deaths	Global	Both	55-59 yea	Gonococca	Rate	2013	0.0782	0.09389	0.05854
Deaths	Global	Both	60-64 yea	Gonococca	Number	2013	172.663	200.905	140.961
Deaths	Global	Both	60-64 yea	Gonococca	Rate	2013	0.06385	0.07429	0.05212
Deaths	Global	Both	65-69 yea	Gonococca	Number	2013	231.069	272.11	183.051
Deaths	Global	Both	65-69 yea	Gonococca	Rate	2013	0.11562	0.13615	0.09159
Deaths	Global	Both	50-69 yea	Gonococca	Number	2013	867.603	974.615	679.198
Deaths	Global	Both	50-69 yea	Gonococca	Rate	2013	0.07457	0.08377	0.05838
Deaths	Global	Both	50-54 yea	Other sexu	Number	2013	170.565	200.634	130.718
Deaths	Global	Both	50-54 yea	Other sexu	Rate	2013	0.046	0.05411	0.03525
Deaths	Global	Both	55-59 yea	Other sexu	Number	2013	212.623	255.114	152.856
Deaths	Global	Both	55-59 yea	Other sexu	Rate	2013	0.06595	0.07913	0.04741
Deaths	Global	Both	60-64 yea	Other sexu	Number	2013	141.679	166.297	111.892
Deaths	Global	Both	60-64 yea	Other sexu	Rate	2013	0.05239	0.06149	0.04138
Deaths	Global	Both	65-69 yea	Other sexu	Number	2013	104.435	124.089	83.0361
Deaths	Global	Both	65-69 yea	Other sexu	Rate	2013	0.05226	0.06209	0.04155
Deaths	Global	Both	50-69 yea	Other sexu	Number	2013	629.302	708.463	480.224
Deaths	Global	Both	50-69 yea	Other sexu	Rate	2013	0.05409	0.06089	0.04127
Deaths	Global	Both	50-54 yea	HIV/AIDS	Number	2013	88601.7	100465	80683.9
Deaths	Global	Both	50-54 yea	HIV/AIDS	Rate	2013	23.896	27.0955	21.7605
Deaths	Global	Both	55-59 yea	HIV/AIDS	Number	2013	51331.1	59065.7	46628.5
Deaths	Global	Both	55-59 yea	HIV/AIDS	Rate	2013	15.9207	18.3197	14.4622
Deaths	Global	Both	60-64 yea	HIV/AIDS	Number	2013	29812.6	34571.7	26918.4
Deaths	Global	Both	60-64 yea	HIV/AIDS	Rate	2013	11.0242	12.784	9.95394
Deaths	Global	Both	65-69 yea	HIV/AIDS	Number	2013	16700.2	19198.7	15065.6
Deaths	Global	Both	65-69 yea	HIV/AIDS	Rate	2013	8.35624	9.60637	7.53832
Deaths	Global	Both	50-69 yea	HIV/AIDS	Number	2013	186446	212046	170936
Deaths	Global	Both	50-69 yea	HIV/AIDS	Rate	2013	16.0248	18.2251	14.6917
Deaths	Global	Both	50-54 yea	HIV/AIDS	Number	2012	91102.5	103386	82691.3
Deaths	Global	Both	50-54 yea	HIV/AIDS	Rate	2012	25.4303	28.8593	23.0824
Deaths	Global	Both	55-59 yea	HIV/AIDS	Number	2012	52103.3	60172	47198.8
Deaths	Global	Both	55-59 yea	HIV/AIDS	Rate	2012	16.4055	18.946	14.8612
Deaths	Global	Both	60-64 yea	HIV/AIDS	Number	2012	29832.1	34940.4	26731.3
Deaths	Global	Both	60-64 yea	HIV/AIDS	Rate	2012	11.4437	13.4033	10.2542
Deaths	Global	Both	65-69 yea	HIV/AIDS	Number	2012	16606.4	19286.4	14866.2
Deaths	Global	Both	65-69 yea	HIV/AIDS	Rate	2012	8.71088	10.1167	7.79806
Deaths	Global	Both	50-69 yea	HIV/AIDS	Number	2012	189644	216474	172956
Deaths	Global	Both	50-69 yea	HIV/AIDS	Rate	2012	16.8249	19.2052	15.3443
Deaths	Global	Both	50-54 yea	Sexually tr	Number	2015	686.972	832.701	502.158
Deaths	Global	Both	50-54 yea	Sexually tr	Rate	2015	0.17193	0.2084	0.12568
Deaths	Global	Both	55-59 yea	Sexually tr	Number	2015	796.787	967.52	553.963
Deaths	Global	Both	55-59 yea	Sexually tr	Rate	2015	0.24137	0.29309	0.16781
Deaths	Global	Both	60-64 yea	Sexually tr	Number	2015	557.829	659.639	432.192
Deaths	Global	Both	60-64 yea	Sexually tr	Rate	2015	0.19167	0.22666	0.1485
Deaths	Global	Both	65-69 yea	Sexually tr	Number	2015	485.937	571.736	402.753
Deaths	Global	Both	65-69 yea	Sexually tr	Rate	2015	0.22034	0.25924	0.18262
Deaths	Global	Both	50-69 yea	Sexually tr	Number	2015	2527.53	2879.86	1887.94
Deaths	Global	Both	50-69 yea	Sexually tr	Rate	2015	0.20363	0.23201	0.1521
Deaths	Global	Both	50-54 yea	Syphilis	Number	2015	158.983	216.838	94.9277
Deaths	Global	Both	50-54 yea	Syphilis	Rate	2015	0.03979	0.05427	0.02376
Deaths	Global	Both	55-59 yea	Syphilis	Number	2015	179.615	267.425	85.2616
Deaths	Global	Both	55-59 yea	Syphilis	Rate	2015	0.05441	0.08101	0.02583
Deaths	Global	Both	60-64 yea	Syphilis	Number	2015	150.333	210.22	91.8384
Deaths	Global	Both	60-64 yea	Syphilis	Rate	2015	0.05166	0.07223	0.03156
Deaths	Global	Both	65-69 yea	Syphilis	Number	2015	96.5056	139.084	77.7973
Deaths	Global	Both	65-69 yea	Syphilis	Rate	2015	0.04376	0.06307	0.03528
Deaths	Global	Both	50-69 yea	Syphilis	Number	2015	585.436	756.968	364.051
Deaths	Global	Both	50-69 yea	Syphilis	Rate	2015	0.04717	0.06098	0.02933
Deaths	Global	Both	50-54 yea	Chlamydia	Number	2015	79.4679	97.1524	59.8002
Deaths	Global	Both	50-54 yea	Chlamydia	Rate	2015	0.01989	0.02431	0.01497
Deaths	Global	Both	55-59 yea	Chlamydia	Number	2015	96.828	121.318	67.9999
Deaths	Global	Both	55-59 yea	Chlamydia	Rate	2015	0.02933	0.03675	0.0206
Deaths	Global	Both	60-64 yea	Chlamydia	Number	2015	65.806	78.6459	50.4478
Deaths	Global	Both	60-64 yea	Chlamydia	Rate	2015	0.02261	0.02702	0.01733
Deaths	Global	Both	65-69 yea	Chlamydia	Number	2015	42.4278	50.6455	33.6342
Deaths	Global	Both	65-69 yea	Chlamydia	Rate	2015	0.01924	0.02296	0.01525
Deaths	Global	Both	50-69 yea	Chlamydia	Number	2015	284.53	329.085	212.804
Deaths	Global	Both	50-69 yea	Chlamydia	Rate	2015	0.02292	0.02651	0.01714
Deaths	Global	Both	50-54 yea	Gonococca	Number	2015	250.531	306.416	191.097
Deaths	Global	Both	50-54 yea	Gonococca	Rate	2015	0.0627	0.07669	0.04783
Deaths	Global	Both	55-59 yea	Gonococca	Number	2015	283.943	352.879	197.881
Deaths	Global	Both	55-59 yea	Gonococca	Rate	2015	0.08602	0.1069	0.05994
Deaths	Global	Both	60-64 yea	Gonococca	Number	2015	187.419	220.521	148.335
Deaths	Global	Both	60-64 yea	Gonococca	Rate	2015	0.0644	0.07577	0.05097
Deaths	Global	Both	65-69 yea	Gonococca	Number	2015	239.358	284.433	192.156
Deaths	Global	Both	65-69 yea	Gonococca	Rate	2015	0.10853	0.12897	0.08713
Deaths	Global	Both	50-69 yea	Gonococca	Number	2015	961.251	1098.28	718.145
Deaths	Global	Both	50-69 yea	Gonococca	Rate	2015	0.07744	0.08848	0.05786
Deaths	Global	Both	50-54 yea	Other sexu	Number	2015	197.99	241.395	145.667
Deaths	Global	Both	50-54 yea	Other sexu	Rate	2015	0.04955	0.06041	0.03646
Deaths	Global	Both	55-59 yea	Other sexu	Number	2015	236.401	288.789	163.094
Deaths	Global	Both	55-59 yea	Other sexu	Rate	2015	0.07161	0.08748	0.04941
Deaths	Global	Both	60-64 yea	Other sexu	Number	2015	154.272	183.462	118.684
Deaths	Global	Both	60-64 yea	Other sexu	Rate	2015	0.05301	0.06304	0.04078
Deaths	Global	Both	65-69 yea	Other sexu	Number	2015	107.646	127.676	86.6131
Deaths	Global	Both	65-69 yea	Other sexu	Rate	2015	0.04881	0.05789	0.03927
Deaths	Global	Both	50-69 yea	Other sexu	Number	2015	696.309	795.966	503.701
Deaths	Global	Both	50-69 yea	Other sexu	Rate	2015	0.0561	0.06413	0.04058
Deaths	Global	Both	50-54 yea	HIV/AIDS	Number	2015	86753.2	97973.7	79004.9
Deaths	Global	Both	50-54 yea	HIV/AIDS	Rate	2015	21.7118	24.52	19.7726
Deaths	Global	Both	55-59 yea	HIV/AIDS	Number	2015	51718.3	59287.9	47117
Deaths	Global	Both	55-59 yea	HIV/AIDS	Rate	2015	15.6671	17.9601	14.2732
Deaths	Global	Both	60-64 yea	HIV/AIDS	Number	2015	31372.8	35824.9	28506.7
Deaths	Global	Both	60-64 yea	HIV/AIDS	Rate	2015	10.7798	12.3096	9.79503
Deaths	Global	Both	65-69 yea	HIV/AIDS	Number	2015	17974.8	20209.3	16459.8
Deaths	Global	Both	65-69 yea	HIV/AIDS	Rate	2015	8.15034	9.16357	7.46342
Deaths	Global	Both	50-69 yea	HIV/AIDS	Number	2015	187819	211841	173002
Deaths	Global	Both	50-69 yea	HIV/AIDS	Rate	2015	15.1315	17.0668	13.9377
Deaths	Global	Both	50-54 yea	Sexually tr	Number	2014	643.59	780.317	482.769
Deaths	Global	Both	50-54 yea	Sexually tr	Rate	2014	0.16707	0.20256	0.12532
Deaths	Global	Both	55-59 yea	Sexually tr	Number	2014	755.906	919.565	534.626
Deaths	Global	Both	55-59 yea	Sexually tr	Rate	2014	0.23177	0.28195	0.16392
Deaths	Global	Both	60-64 yea	Sexually tr	Number	2014	527.888	628.773	402.725
Deaths	Global	Both	60-64 yea	Sexually tr	Rate	2014	0.18904	0.22516	0.14422
Deaths	Global	Both	65-69 yea	Sexually tr	Number	2014	464.855	553.071	385.402
Deaths	Global	Both	65-69 yea	Sexually tr	Rate	2014	0.22007	0.26183	0.18245
Deaths	Global	Both	50-69 yea	Sexually tr	Number	2014	2392.24	2712.21	1829.11
Deaths	Global	Both	50-69 yea	Sexually tr	Rate	2014	0.19905	0.22567	0.15219
Deaths	Global	Both	50-54 yea	Syphilis	Number	2014	153.713	211.472	89.3194
Deaths	Global	Both	50-54 yea	Syphilis	Rate	2014	0.0399	0.0549	0.02319
Deaths	Global	Both	55-59 yea	Syphilis	Number	2014	178.596	273.27	82.8108
Deaths	Global	Both	55-59 yea	Syphilis	Rate	2014	0.05476	0.08379	0.02539
Deaths	Global	Both	60-64 yea	Syphilis	Number	2014	146.19	205.769	89.4687
Deaths	Global	Both	60-64 yea	Syphilis	Rate	2014	0.05235	0.07369	0.03204
Deaths	Global	Both	65-69 yea	Syphilis	Number	2014	94.3512	136.176	76.3967
Deaths	Global	Both	65-69 yea	Syphilis	Rate	2014	0.04467	0.06447	0.03617
Deaths	Global	Both	50-69 yea	Syphilis	Number	2014	572.851	748.34	349.421
Deaths	Global	Both	50-69 yea	Syphilis	Rate	2014	0.04766	0.06227	0.02907
Deaths	Global	Both	50-54 yea	Chlamydia	Number	2014	73.3618	88.9206	56.8773
Deaths	Global	Both	50-54 yea	Chlamydia	Rate	2014	0.01904	0.02308	0.01476
Deaths	Global	Both	55-59 yea	Chlamydia	Number	2014	90.1002	110.532	65.5837
Deaths	Global	Both	55-59 yea	Chlamydia	Rate	2014	0.02763	0.03389	0.02011
Deaths	Global	Both	60-64 yea	Chlamydia	Number	2014	61.4314	73.2689	48.8406
Deaths	Global	Both	60-64 yea	Chlamydia	Rate	2014	0.022	0.02624	0.01749
Deaths	Global	Both	65-69 yea	Chlamydia	Number	2014	40.2781	47.4748	32.0473
Deaths	Global	Both	65-69 yea	Chlamydia	Rate	2014	0.01907	0.02247	0.01517
Deaths	Global	Both	50-69 yea	Chlamydia	Number	2014	265.171	301.045	207.844
Deaths	Global	Both	50-69 yea	Chlamydia	Rate	2014	0.02206	0.02505	0.01729
Deaths	Global	Both	50-54 yea	Gonococca	Number	2014	231.831	279.111	181.834
Deaths	Global	Both	50-54 yea	Gonococca	Rate	2014	0.06018	0.07245	0.0472
Deaths	Global	Both	55-59 yea	Gonococca	Number	2014	265.014	324.05	190.641
Deaths	Global	Both	55-59 yea	Gonococca	Rate	2014	0.08126	0.09936	0.05845
Deaths	Global	Both	60-64 yea	Gonococca	Number	2014	175.844	207.222	139.737
Deaths	Global	Both	60-64 yea	Gonococca	Rate	2014	0.06297	0.07421	0.05004
Deaths	Global	Both	65-69 yea	Gonococca	Number	2014	227.552	266.608	183.091
Deaths	Global	Both	65-69 yea	Gonococca	Rate	2014	0.10772	0.12621	0.08668
Deaths	Global	Both	50-69 yea	Gonococca	Number	2014	900.242	1015.44	702.989
Deaths	Global	Both	50-69 yea	Gonococca	Rate	2014	0.0749	0.08449	0.05849
Deaths	Global	Both	50-54 yea	Other sexu	Number	2014	184.684	222.974	141.293
Deaths	Global	Both	50-54 yea	Other sexu	Rate	2014	0.04794	0.05788	0.03668
Deaths	Global	Both	55-59 yea	Other sexu	Number	2014	222.195	269.193	156.718
Deaths	Global	Both	55-59 yea	Other sexu	Rate	2014	0.06813	0.08254	0.04805
Deaths	Global	Both	60-64 yea	Other sexu	Number	2014	144.423	172.05	111.523
Deaths	Global	Both	60-64 yea	Other sexu	Rate	2014	0.05172	0.06161	0.03994
Deaths	Global	Both	65-69 yea	Other sexu	Number	2014	102.673	120.677	83.0686
Deaths	Global	Both	65-69 yea	Other sexu	Rate	2014	0.04861	0.05713	0.03933
Deaths	Global	Both	50-69 yea	Other sexu	Number	2014	653.975	740.81	492.389
Deaths	Global	Both	50-69 yea	Other sexu	Rate	2014	0.05441	0.06164	0.04097
Deaths	Global	Both	50-54 yea	Sexually tr	Number	2010	578.054	685.819	448.807
Deaths	Global	Both	50-54 yea	Sexually tr	Rate	2010	0.16838	0.19977	0.13073
Deaths	Global	Both	55-59 yea	Sexually tr	Number	2010	720.068	875.038	527.189
Deaths	Global	Both	55-59 yea	Sexually tr	Rate	2010	0.23733	0.28841	0.17376
Deaths	Global	Both	60-64 yea	Sexually tr	Number	2010	476.52	568.152	375.695
Deaths	Global	Both	60-64 yea	Sexually tr	Rate	2010	0.19945	0.2378	0.15725
Deaths	Global	Both	65-69 yea	Sexually tr	Number	2010	460.332	550.383	386.94
Deaths	Global	Both	65-69 yea	Sexually tr	Rate	2010	0.25812	0.30861	0.21697
Deaths	Global	Both	50-69 yea	Sexually tr	Number	2010	2234.97	2539.45	1785.47
Deaths	Global	Both	50-69 yea	Sexually tr	Rate	2010	0.21006	0.23868	0.16781
Deaths	Global	Both	50-54 yea	Syphilis	Number	2010	151.52	215.128	85.2478
Deaths	Global	Both	50-54 yea	Syphilis	Rate	2010	0.04414	0.06267	0.02483
Deaths	Global	Both	55-59 yea	Syphilis	Number	2010	185.603	283.156	85.3831
Deaths	Global	Both	55-59 yea	Syphilis	Rate	2010	0.06117	0.09333	0.02814
Deaths	Global	Both	60-64 yea	Syphilis	Number	2010	141.247	200.27	85.5841
Deaths	Global	Both	60-64 yea	Syphilis	Rate	2010	0.05912	0.08382	0.03582
Deaths	Global	Both	65-69 yea	Syphilis	Number	2010	95.4302	143.026	75.3413
Deaths	Global	Both	65-69 yea	Syphilis	Rate	2010	0.05351	0.0802	0.04225
Deaths	Global	Both	50-69 yea	Syphilis	Number	2010	573.8	761.059	340.729
Deaths	Global	Both	50-69 yea	Syphilis	Rate	2010	0.05393	0.07153	0.03202
Deaths	Global	Both	50-54 yea	Chlamydia	Number	2010	62.8932	73.4764	52.681
Deaths	Global	Both	50-54 yea	Chlamydia	Rate	2010	0.01832	0.0214	0.01535
Deaths	Global	Both	55-59 yea	Chlamydia	Number	2010	82.5616	98.6407	63.3711
Deaths	Global	Both	55-59 yea	Chlamydia	Rate	2010	0.02721	0.03251	0.02089
Deaths	Global	Both	60-64 yea	Chlamydia	Number	2010	53.4997	62.7444	42.5776
Deaths	Global	Both	60-64 yea	Chlamydia	Rate	2010	0.02239	0.02626	0.01782
Deaths	Global	Both	65-69 yea	Chlamydia	Number	2010	39.5985	47.4415	31.3922
Deaths	Global	Both	65-69 yea	Chlamydia	Rate	2010	0.0222	0.0266	0.0176
Deaths	Global	Both	50-69 yea	Chlamydia	Number	2010	238.553	266.156	191.295
Deaths	Global	Both	50-69 yea	Chlamydia	Rate	2010	0.02242	0.02502	0.01798
Deaths	Global	Both	50-54 yea	Sexually tr	Number	2018	675.234	838.103	494.574
Deaths	Global	Both	50-54 yea	Sexually tr	Rate	2018	0.15682	0.19465	0.11486
Deaths	Global	Both	55-59 yea	Sexually tr	Number	2018	794.101	997.665	528.928
Deaths	Global	Both	55-59 yea	Sexually tr	Rate	2018	0.22251	0.27954	0.14821
Deaths	Global	Both	60-64 yea	Sexually tr	Number	2018	553.896	669.985	420.782
Deaths	Global	Both	60-64 yea	Sexually tr	Rate	2018	0.17922	0.21679	0.13615
Deaths	Global	Both	65-69 yea	Sexually tr	Number	2018	529.108	637.397	429.725
Deaths	Global	Both	65-69 yea	Sexually tr	Rate	2018	0.21139	0.25466	0.17169
Deaths	Global	Both	50-69 yea	Sexually tr	Number	2018	2552.34	2987.61	1902.29
Deaths	Global	Both	50-69 yea	Sexually tr	Rate	2018	0.18951	0.22183	0.14124
Deaths	Global	Both	50-54 yea	Syphilis	Number	2018	156.284	208.057	90.6666
Deaths	Global	Both	50-54 yea	Syphilis	Rate	2018	0.0363	0.04832	0.02106
Deaths	Global	Both	55-59 yea	Syphilis	Number	2018	173.98	258.963	80.4631
Deaths	Global	Both	55-59 yea	Syphilis	Rate	2018	0.04875	0.07256	0.02255
Deaths	Global	Both	60-64 yea	Syphilis	Number	2018	145.226	203.288	86.9286
Deaths	Global	Both	60-64 yea	Syphilis	Rate	2018	0.04699	0.06578	0.02813
Deaths	Global	Both	65-69 yea	Syphilis	Number	2018	100.679	143.285	80.0792
Deaths	Global	Both	65-69 yea	Syphilis	Rate	2018	0.04022	0.05725	0.03199
Deaths	Global	Both	50-69 yea	Syphilis	Number	2018	576.169	741.728	356.446
Deaths	Global	Both	50-69 yea	Syphilis	Rate	2018	0.04278	0.05507	0.02647
Deaths	Global	Both	50-54 yea	Chlamydia	Number	2018	78.0368	98.1973	57.7502
Deaths	Global	Both	50-54 yea	Chlamydia	Rate	2018	0.01812	0.02281	0.01341
Deaths	Global	Both	55-59 yea	Chlamydia	Number	2018	97.5375	126.541	68.3357
Deaths	Global	Both	55-59 yea	Chlamydia	Rate	2018	0.02733	0.03546	0.01915
Deaths	Global	Both	60-64 yea	Chlamydia	Number	2018	66.2239	81.8768	49.0888
Deaths	Global	Both	60-64 yea	Chlamydia	Rate	2018	0.02143	0.02649	0.01588
Deaths	Global	Both	65-69 yea	Chlamydia	Number	2018	46.8327	58.325	36.749
Deaths	Global	Both	65-69 yea	Chlamydia	Rate	2018	0.01871	0.0233	0.01468
Deaths	Global	Both	50-69 yea	Chlamydia	Number	2018	288.631	341.81	221.026
Deaths	Global	Both	50-69 yea	Chlamydia	Rate	2018	0.02143	0.02538	0.01641
Deaths	Global	Both	50-54 yea	Gonococca	Number	2010	200.258	231.649	165.766
Deaths	Global	Both	50-54 yea	Gonococca	Rate	2010	0.05833	0.06748	0.04829
Deaths	Global	Both	55-59 yea	Gonococca	Number	2010	244.294	288.918	185.597
Deaths	Global	Both	55-59 yea	Gonococca	Rate	2010	0.08052	0.09523	0.06117
Deaths	Global	Both	60-64 yea	Gonococca	Number	2010	154.963	179.751	126.547
Deaths	Global	Both	60-64 yea	Gonococca	Rate	2010	0.06486	0.07523	0.05297
Deaths	Global	Both	65-69 yea	Gonococca	Number	2010	223.861	265.353	180.008
Deaths	Global	Both	65-69 yea	Gonococca	Rate	2010	0.12552	0.14879	0.10094
Deaths	Global	Both	50-69 yea	Gonococca	Number	2010	823.376	911.635	651.811
Deaths	Global	Both	50-69 yea	Gonococca	Rate	2010	0.07739	0.08568	0.06126
Deaths	Global	Both	50-54 yea	Other sexu	Number	2010	163.384	191.307	128.488
Deaths	Global	Both	50-54 yea	Other sexu	Rate	2010	0.04759	0.05573	0.03743
Deaths	Global	Both	55-59 yea	Other sexu	Number	2010	207.609	247.275	152.88
Deaths	Global	Both	55-59 yea	Other sexu	Rate	2010	0.06843	0.0815	0.05039
Deaths	Global	Both	60-64 yea	Other sexu	Number	2010	126.81	149.259	100.991
Deaths	Global	Both	60-64 yea	Other sexu	Rate	2010	0.05308	0.06247	0.04227
Deaths	Global	Both	65-69 yea	Other sexu	Number	2010	101.442	120.349	82.2004
Deaths	Global	Both	65-69 yea	Other sexu	Rate	2010	0.05688	0.06748	0.04609
Deaths	Global	Both	50-69 yea	Other sexu	Number	2010	599.246	669.511	457.172
Deaths	Global	Both	50-69 yea	Other sexu	Rate	2010	0.05632	0.06293	0.04297
Deaths	Global	Both	50-54 yea	Gonococca	Number	2018	246.025	307.047	182.727
Deaths	Global	Both	50-54 yea	Gonococca	Rate	2018	0.05714	0.07131	0.04244
Deaths	Global	Both	55-59 yea	Gonococca	Number	2018	285.587	367.055	198.379
Deaths	Global	Both	55-59 yea	Gonococca	Rate	2018	0.08002	0.10285	0.05559
Deaths	Global	Both	60-64 yea	Gonococca	Number	2018	188.079	228.173	140.253
Deaths	Global	Both	60-64 yea	Gonococca	Rate	2018	0.06086	0.07383	0.04538
Deaths	Global	Both	65-69 yea	Gonococca	Number	2018	263.387	324.226	208.581
Deaths	Global	Both	65-69 yea	Gonococca	Rate	2018	0.10523	0.12954	0.08333
Deaths	Global	Both	50-69 yea	Gonococca	Number	2018	983.078	1151.85	760.285
Deaths	Global	Both	50-69 yea	Gonococca	Rate	2018	0.07299	0.08552	0.05645
Deaths	Global	Both	50-54 yea	Other sexu	Number	2018	194.888	243.587	143.386
Deaths	Global	Both	50-54 yea	Other sexu	Rate	2018	0.04526	0.05657	0.0333
Deaths	Global	Both	55-59 yea	Other sexu	Number	2018	236.997	300.989	159.652
Deaths	Global	Both	55-59 yea	Other sexu	Rate	2018	0.06641	0.08434	0.04473
Deaths	Global	Both	60-64 yea	Other sexu	Number	2018	154.367	188.247	113.747
Deaths	Global	Both	60-64 yea	Other sexu	Rate	2018	0.04995	0.06091	0.03681
Deaths	Global	Both	65-69 yea	Other sexu	Number	2018	118.21	145.437	93.5306
Deaths	Global	Both	65-69 yea	Other sexu	Rate	2018	0.04723	0.05811	0.03737
Deaths	Global	Both	50-69 yea	Other sexu	Number	2018	704.462	834.068	523.955
Deaths	Global	Both	50-69 yea	Other sexu	Rate	2018	0.05231	0.06193	0.0389
Deaths	Global	Both	50-54 yea	HIV/AIDS	Number	2019	81011.3	93999.2	72221.1
Deaths	Global	Both	50-54 yea	HIV/AIDS	Rate	2019	18.5458	21.5191	16.5335
Deaths	Global	Both	55-59 yea	HIV/AIDS	Number	2019	50790.3	59550.2	45210.8
Deaths	Global	Both	55-59 yea	HIV/AIDS	Rate	2019	13.6896	16.0507	12.1858
Deaths	Global	Both	60-64 yea	HIV/AIDS	Number	2019	31166	36592.4	27565.1
Deaths	Global	Both	60-64 yea	HIV/AIDS	Rate	2019	9.97201	11.7082	8.81984
Deaths	Global	Both	65-69 yea	HIV/AIDS	Number	2019	19435.3	22506.6	17285.8
Deaths	Global	Both	65-69 yea	HIV/AIDS	Rate	2019	7.51604	8.70382	6.6848
Deaths	Global	Both	50-69 yea	HIV/AIDS	Number	2019	182403	212251	163675
Deaths	Global	Both	50-69 yea	HIV/AIDS	Rate	2019	13.2277	15.3922	11.8696
Deaths	Global	Both	50-54 yea	Sexually tr	Number	2019	670.298	834.498	469.23
Deaths	Global	Both	50-54 yea	Sexually tr	Rate	2019	0.15345	0.19104	0.10742
Deaths	Global	Both	55-59 yea	Sexually tr	Number	2019	794.521	1012.37	535.022
Deaths	Global	Both	55-59 yea	Sexually tr	Rate	2019	0.21415	0.27287	0.14421
Deaths	Global	Both	60-64 yea	Sexually tr	Number	2019	554.473	669.4	421.129
Deaths	Global	Both	60-64 yea	Sexually tr	Rate	2019	0.17741	0.21418	0.13475
Deaths	Global	Both	65-69 yea	Sexually tr	Number	2019	539.55	657.846	432.341
Deaths	Global	Both	65-69 yea	Sexually tr	Rate	2019	0.20866	0.2544	0.1672
Deaths	Global	Both	50-69 yea	Sexually tr	Number	2019	2558.84	2993.56	1888.77
Deaths	Global	Both	50-69 yea	Sexually tr	Rate	2019	0.18556	0.21709	0.13697
Deaths	Global	Both	50-54 yea	Syphilis	Number	2019	155.582	206.64	89.5327
Deaths	Global	Both	50-54 yea	Syphilis	Rate	2019	0.03562	0.04731	0.0205
Deaths	Global	Both	55-59 yea	Syphilis	Number	2019	173.586	253.416	81.1555
Deaths	Global	Both	55-59 yea	Syphilis	Rate	2019	0.04679	0.0683	0.02187
Deaths	Global	Both	60-64 yea	Syphilis	Number	2019	144.311	202.707	87.0353
Deaths	Global	Both	60-64 yea	Syphilis	Rate	2019	0.04617	0.06486	0.02785
Deaths	Global	Both	65-69 yea	Syphilis	Number	2019	101.544	140.985	80.8644
Deaths	Global	Both	65-69 yea	Syphilis	Rate	2019	0.03927	0.05452	0.03127
Deaths	Global	Both	50-69 yea	Syphilis	Number	2019	575.023	737.778	357.321
Deaths	Global	Both	50-69 yea	Syphilis	Rate	2019	0.0417	0.0535	0.02591
Deaths	Global	Both	50-54 yea	Chlamydia	Number	2019	77.359	99.1997	56.181
Deaths	Global	Both	50-54 yea	Chlamydia	Rate	2019	0.01771	0.02271	0.01286
Deaths	Global	Both	55-59 yea	Chlamydia	Number	2019	97.6792	129.066	69.1118
Deaths	Global	Both	55-59 yea	Chlamydia	Rate	2019	0.02633	0.03479	0.01863
Deaths	Global	Both	60-64 yea	Chlamydia	Number	2019	66.5313	82.4877	50.0277
Deaths	Global	Both	60-64 yea	Chlamydia	Rate	2019	0.02129	0.02639	0.01601
Deaths	Global	Both	65-69 yea	Chlamydia	Number	2019	47.9196	59.3602	37.2256
Deaths	Global	Both	65-69 yea	Chlamydia	Rate	2019	0.01853	0.02296	0.0144
Deaths	Global	Both	50-69 yea	Chlamydia	Number	2019	289.489	349.768	215.693
Deaths	Global	Both	50-69 yea	Chlamydia	Rate	2019	0.02099	0.02536	0.01564

Deaths	Global	Both	60-64 yea	HIV/AIDS	Rate	2016	10.6755	12.2256	9.73948
Deaths	Global	Both	65-69 yea	HIV/AIDS	Number	2016	18858.6	21162.6	17290.6
Deaths	Global	Both	65-69 yea	HIV/AIDS	Rate	2016	8.11111	9.10209	7.43671
Deaths	Global	Both	50-69 yea	HIV/AIDS	Number	2016	189400	214034	174467
Deaths	Global	Both	50-69 yea	HIV/AIDS	Rate	2016	14.8119	16.7383	13.6441
Deaths	Global	Both	50-54 yea	Sexually tr	Number	2017	687.65	846.631	501.931
Deaths	Global	Both	50-54 yea	Sexually tr	Rate	2017	0.16274	0.20036	0.11879
Deaths	Global	Both	55-59 yea	Sexually tr	Number	2017	797.057	988.817	553.948
Deaths	Global	Both	55-59 yea	Sexually tr	Rate	2017	0.23125	0.28689	0.16072
Deaths	Global	Both	60-64 yea	Sexually tr	Number	2017	556.327	655.01	420.876
Deaths	Global	Both	60-64 yea	Sexually tr	Rate	2017	0.18269	0.2151	0.13821
Deaths	Global	Both	65-69 yea	Sexually tr	Number	2017	522.16	624.84	426.865
Deaths	Global	Both	65-69 yea	Sexually tr	Rate	2017	0.21655	0.25913	0.17703
Deaths	Global	Both	50-69 yea	Sexually tr	Number	2017	2563.19	2961.07	1926.56
Deaths	Global	Both	50-69 yea	Sexually tr	Rate	2017	0.19524	0.22554	0.14674
Deaths	Global	Both	50-54 yea	Syphilis	Number	2017	158.143	213.383	92.5608
Deaths	Global	Both	50-54 yea	Syphilis	Rate	2017	0.03743	0.0505	0.02191
Deaths	Global	Both	55-59 yea	Syphilis	Number	2017	175.494	256.787	82.8348
Deaths	Global	Both	55-59 yea	Syphilis	Rate	2017	0.05092	0.0745	0.02403
Deaths	Global	Both	60-64 yea	Syphilis	Number	2017	147.236	203.517	89.5348
Deaths	Global	Both	60-64 yea	Syphilis	Rate	2017	0.04835	0.06683	0.0294
Deaths	Global	Both	65-69 yea	Syphilis	Number	2017	100.589	140.017	80.9742
Deaths	Global	Both	65-69 yea	Syphilis	Rate	2017	0.04172	0.05807	0.03358
Deaths	Global	Both	50-69 yea	Syphilis	Number	2017	581.463	744.935	362.391
Deaths	Global	Both	50-69 yea	Syphilis	Rate	2017	0.04429	0.05674	0.0276
Deaths	Global	Both	50-54 yea	Chlamydia	Number	2017	79.7102	98.5275	58.6941
Deaths	Global	Both	50-54 yea	Chlamydia	Rate	2017	0.01886	0.02332	0.01389
Deaths	Global	Both	55-59 yea	Chlamydia	Number	2017	97.7211	124.442	71.0758
Deaths	Global	Both	55-59 yea	Chlamydia	Rate	2017	0.02835	0.0361	0.02062
Deaths	Global	Both	60-64 yea	Chlamydia	Number	2017	66.2044	80.3686	50.4966
Deaths	Global	Both	60-64 yea	Chlamydia	Rate	2017	0.02174	0.02639	0.01658
Deaths	Global	Both	65-69 yea	Chlamydia	Number	2017	46.0376	56.0079	36.7795
Deaths	Global	Both	65-69 yea	Chlamydia	Rate	2017	0.01909	0.02323	0.01525
Deaths	Global	Both	50-69 yea	Chlamydia	Number	2017	289.673	339.058	219.697
Deaths	Global	Both	50-69 yea	Chlamydia	Rate	2017	0.02206	0.02583	0.01673
Deaths	Global	Both	50-54 yea	Gonococca	Number	2017	251.197	309.507	186.175
Deaths	Global	Both	50-54 yea	Gonococca	Rate	2017	0.05945	0.07325	0.04406
Deaths	Global	Both	55-59 yea	Gonococca	Number	2017	286.201	360.212	207.733
Deaths	Global	Both	55-59 yea	Gonococca	Rate	2017	0.08304	0.10451	0.06027
Deaths	Global	Both	60-64 yea	Gonococca	Number	2017	188.167	224.493	146.632
Deaths	Global	Both	60-64 yea	Gonococca	Rate	2017	0.06179	0.07372	0.04815
Deaths	Global	Both	65-69 yea	Gonococca	Number	2017	259.156	312.886	207.589
Deaths	Global	Both	65-69 yea	Gonococca	Rate	2017	0.10748	0.12976	0.08609
Deaths	Global	Both	50-69 yea	Gonococca	Number	2017	984.721	1140.86	745.937
Deaths	Global	Both	50-69 yea	Gonococca	Rate	2017	0.07501	0.0869	0.05682
Deaths	Global	Both	50-54 yea	Other sexu	Number	2017	198.599	245.288	146.329
Deaths	Global	Both	50-54 yea	Other sexu	Rate	2017	0.047	0.05805	0.03463
Deaths	Global	Both	55-59 yea	Other sexu	Number	2017	237.641	298.313	165.761
Deaths	Global	Both	55-59 yea	Other sexu	Rate	2017	0.06895	0.08655	0.04809
Deaths	Global	Both	60-64 yea	Other sexu	Number	2017	154.72	186.439	116.576
Deaths	Global	Both	60-64 yea	Other sexu	Rate	2017	0.05081	0.06122	0.03828
Deaths	Global	Both	65-69 yea	Other sexu	Number	2017	116.376	139.952	92.9584
Deaths	Global	Both	65-69 yea	Other sexu	Rate	2017	0.04826	0.05804	0.03855
Deaths	Global	Both	50-69 yea	Other sexu	Number	2017	707.336	818.974	519.863
Deaths	Global	Both	50-69 yea	Other sexu	Rate	2017	0.05388	0.06238	0.0396
DALYs (Dis	Global	Both	50-54 yea	Sexually tr	Number	1990	56085.4	86940.2	38766
DALYs (Dis	Global	Both	50-54 yea	Sexually tr	Rate	1990	26.3818	40.8955	18.235
DALYs (Dis	Global	Both	55-59 yea	Sexually tr	Number	1990	49095.7	72307.1	34834.2
DALYs (Dis	Global	Both	55-59 yea	Sexually tr	Rate	1990	26.4787	38.9973	18.7871
DALYs (Dis	Global	Both	60-64 yea	Sexually tr	Number	1990	32550.2	49602.5	22685.1
DALYs (Dis	Global	Both	60-64 yea	Sexually tr	Rate	1990	20.2624	30.8775	14.1214
DALYs (Dis	Global	Both	65-69 yea	Sexually tr	Number	1990	22694.1	34692.3	16135.9
DALYs (Dis	Global	Both	65-69 yea	Sexually tr	Rate	1990	18.3775	28.0935	13.0667
DALYs (Dis	Global	Both	50-69 yea	Sexually tr	Number	1990	160425	243796	114389
DALYs (Dis	Global	Both	50-69 yea	Sexually tr	Rate	1990	23.518	35.7399	16.7692
DALYs (Dis	Global	Both	50-54 yea	Syphilis	Number	1990	9748.53	13413.6	5742.73
DALYs (Dis	Global	Both	50-54 yea	Syphilis	Rate	1990	4.58558	6.30959	2.7013
DALYs (Dis	Global	Both	55-59 yea	Syphilis	Number	1990	10358.8	16234.6	5271.04
DALYs (Dis	Global	Both	55-59 yea	Syphilis	Rate	1990	5.58678	8.75579	2.84282
DALYs (Dis	Global	Both	60-64 yea	Syphilis	Number	1990	8067.89	10968.4	5161.12
DALYs (Dis	Global	Both	60-64 yea	Syphilis	Rate	1990	5.02225	6.82778	3.21279
DALYs (Dis	Global	Both	65-69 yea	Syphilis	Number	1990	4824.01	6274.61	3860.86
DALYs (Dis	Global	Both	65-69 yea	Syphilis	Rate	1990	3.90644	5.08113	3.12649
DALYs (Dis	Global	Both	50-69 yea	Syphilis	Number	1990	32999.2	44301.1	20516.4
DALYs (Dis	Global	Both	50-69 yea	Syphilis	Rate	1990	4.83761	6.49444	3.00766
DALYs (Dis	Global	Both	50-54 yea	Chlamydia	Number	1990	4363.75	5608.23	3313.55
DALYs (Dis	Global	Both	50-54 yea	Chlamydia	Rate	1990	2.05265	2.63804	1.55865
DALYs (Dis	Global	Both	55-59 yea	Chlamydia	Number	1990	3494.61	4311.81	2630.51
DALYs (Dis	Global	Both	55-59 yea	Chlamydia	Rate	1990	1.88475	2.32549	1.41871
DALYs (Dis	Global	Both	60-64 yea	Chlamydia	Number	1990	1772.01	2173.86	1301.73
DALYs (Dis	Global	Both	60-64 yea	Chlamydia	Rate	1990	1.10308	1.35323	0.81033
DALYs (Dis	Global	Both	65-69 yea	Chlamydia	Number	1990	893.54	1074.3	687.699
DALYs (Dis	Global	Both	65-69 yea	Chlamydia	Rate	1990	0.72358	0.86996	0.55689
DALYs (Dis	Global	Both	50-69 yea	Chlamydia	Number	1990	10523.9	12660.2	8356.37
DALYs (Dis	Global	Both	50-69 yea	Chlamydia	Rate	1990	1.54278	1.85596	1.22503
DALYs (Dis	Global	Both	50-54 yea	Gonococca	Number	1990	8708.05	10527.5	6407.51
DALYs (Dis	Global	Both	50-54 yea	Gonococca	Rate	1990	4.09615	4.95197	3.01401
DALYs (Dis	Global	Both	55-59 yea	Gonococca	Number	1990	8624.71	10632.4	6155.07
DALYs (Dis	Global	Both	55-59 yea	Gonococca	Rate	1990	4.65156	5.73439	3.31961
DALYs (Dis	Global	Both	60-64 yea	Gonococca	Number	1990	5200.58	6230.23	3918.73
DALYs (Dis	Global	Both	60-64 yea	Gonococca	Rate	1990	3.23735	3.87831	2.4394
DALYs (Dis	Global	Both	65-69 yea	Gonococca	Number	1990	5154.4	6173.05	4103.67
DALYs (Dis	Global	Both	65-69 yea	Gonococca	Rate	1990	4.17399	4.99889	3.32312
DALYs (Dis	Global	Both	50-69 yea	Gonococca	Number	1990	27687.7	32149.2	20651.1
DALYs (Dis	Global	Both	50-69 yea	Gonococca	Rate	1990	4.05896	4.713	3.02741
DALYs (Dis	Global	Both	50-54 yea	Trichomon	Number	1990	10588.8	24970.6	3376.18
DALYs (Dis	Global	Both	50-54 yea	Trichomon	Rate	1990	4.98084	11.7458	1.58811
DALYs (Dis	Global	Both	55-59 yea	Trichomon	Number	1990	7296.76	17022.6	2364.55
DALYs (Dis	Global	Both	55-59 yea	Trichomon	Rate	1990	3.93536	9.18077	1.27527
DALYs (Dis	Global	Both	60-64 yea	Trichomon	Number	1990	4982.96	11581.7	1706.22
DALYs (Dis	Global	Both	60-64 yea	Trichomon	Rate	1990	3.10188	7.20959	1.06212
DALYs (Dis	Global	Both	65-69 yea	Trichomon	Number	1990	3415.95	7907.09	1211.54
DALYs (Dis	Global	Both	65-69 yea	Trichomon	Rate	1990	2.7662	6.40309	0.98109
DALYs (Dis	Global	Both	50-69 yea	Trichomon	Number	1990	26284.5	58787.3	9664.14
DALYs (Dis	Global	Both	50-69 yea	Trichomon	Rate	1990	3.85325	8.61809	1.41674
DALYs (Dis	Global	Both	50-54 yea	Genital he	Number	1990	11878.7	29532.7	3697.4
DALYs (Dis	Global	Both	50-54 yea	Genital he	Rate	1990	5.58758	13.8918	1.73921
DALYs (Dis	Global	Both	55-59 yea	Genital he	Number	1990	9983.47	24578.7	3058.07
DALYs (Dis	Global	Both	55-59 yea	Genital he	Rate	1990	5.38438	13.256	1.64931
DALYs (Dis	Global	Both	60-64 yea	Genital he	Number	1990	8255.81	20134.9	2499.35
DALYs (Dis	Global	Both	60-64 yea	Genital he	Rate	1990	5.13923	12.5339	1.55584
DALYs (Dis	Global	Both	65-69 yea	Genital he	Number	1990	6037.7	14728.5	1828.81
DALYs (Dis	Global	Both	65-69 yea	Genital he	Rate	1990	4.88928	11.927	1.48095
DALYs (Dis	Global	Both	50-69 yea	Genital he	Number	1990	36155.7	89015	11078.4
DALYs (Dis	Global	Both	50-69 yea	Genital he	Rate	1990	5.30035	13.0494	1.62407
DALYs (Dis	Global	Both	50-54 yea	Other sexu	Number	1990	10797.5	13601.1	8176.71
DALYs (Dis	Global	Both	50-54 yea	Other sexu	Rate	1990	5.07902	6.39777	3.84622
DALYs (Dis	Global	Both	55-59 yea	Other sexu	Number	1990	9337.4	11534.3	7110.9
DALYs (Dis	Global	Both	55-59 yea	Other sexu	Rate	1990	5.03593	6.22077	3.83512
DALYs (Dis	Global	Both	60-64 yea	Other sexu	Number	1990	4270.91	5173.98	3128.86
DALYs (Dis	Global	Both	60-64 yea	Other sexu	Rate	1990	2.65863	3.22079	1.94771
DALYs (Dis	Global	Both	65-69 yea	Other sexu	Number	1990	2368.55	2853.6	1918.66
DALYs (Dis	Global	Both	65-69 yea	Other sexu	Rate	1990	1.91803	2.31082	1.55371
DALYs (Dis	Global	Both	50-69 yea	Other sexu	Number	1990	26774.4	31751.2	21360.1
DALYs (Dis	Global	Both	50-69 yea	Other sexu	Rate	1990	3.92507	4.65466	3.13134
DALYs (Dis	Global	Both	50-54 yea	HIV/AIDS	Number	1990	666401	882897	483775
DALYs (Dis	Global	Both	50-54 yea	HIV/AIDS	Rate	1990	313.466	415.303	227.561
DALYs (Dis	Global	Both	55-59 yea	HIV/AIDS	Number	1990	367861	481089	273226
DALYs (Dis	Global	Both	55-59 yea	HIV/AIDS	Rate	1990	198.398	259.465	147.359
DALYs (Dis	Global	Both	60-64 yea	HIV/AIDS	Number	1990	201555	263162	150700
DALYs (Dis	Global	Both	60-64 yea	HIV/AIDS	Rate	1990	125.467	163.818	93.8102
DALYs (Dis	Global	Both	65-69 yea	HIV/AIDS	Number	1990	111728	146541	83421.7
DALYs (Dis	Global	Both	65-69 yea	HIV/AIDS	Rate	1990	90.4766	118.667	67.5542
DALYs (Dis	Global	Both	50-69 yea	HIV/AIDS	Number	1990	1347544	1780505	993576
DALYs (Dis	Global	Both	50-69 yea	HIV/AIDS	Rate	1990	197.547	261.018	145.656
DALYs (Dis	Global	Both	50-54 yea	HIV/AIDS	Number	1992	1091434	1431402	797703
DALYs (Dis	Global	Both	50-54 yea	HIV/AIDS	Rate	1992	504.647	661.839	368.834
DALYs (Dis	Global	Both	55-59 yea	HIV/AIDS	Number	1992	601186	779710	447240
DALYs (Dis	Global	Both	55-59 yea	HIV/AIDS	Rate	1992	314.411	407.777	233.9
DALYs (Dis	Global	Both	60-64 yea	HIV/AIDS	Number	1992	328621	424513	246636
DALYs (Dis	Global	Both	60-64 yea	HIV/AIDS	Rate	1992	197.558	255.206	148.271
DALYs (Dis	Global	Both	65-69 yea	HIV/AIDS	Number	1992	177956	231585	132819
DALYs (Dis	Global	Both	65-69 yea	HIV/AIDS	Rate	1992	135.733	176.638	101.305
DALYs (Dis	Global	Both	50-69 yea	HIV/AIDS	Number	1992	2199196	2870111	1625521
DALYs (Dis	Global	Both	50-69 yea	HIV/AIDS	Rate	1992	311.971	407.145	230.591
DALYs (Dis	Global	Both	50-54 yea	HIV/AIDS	Number	1991	861666	1139256	626725
DALYs (Dis	Global	Both	50-54 yea	HIV/AIDS	Rate	1991	400.977	530.154	291.647
DALYs (Dis	Global	Both	55-59 yea	HIV/AIDS	Number	1991	474633	619585	350859
DALYs (Dis	Global	Both	55-59 yea	HIV/AIDS	Rate	1991	252.522	329.642	186.67
DALYs (Dis	Global	Both	60-64 yea	HIV/AIDS	Number	1991	259966	337125	194814
DALYs (Dis	Global	Both	60-64 yea	HIV/AIDS	Rate	1991	158.528	205.581	118.799
DALYs (Dis	Global	Both	65-69 yea	HIV/AIDS	Number	1991	142307	185737	105965
DALYs (Dis	Global	Both	65-69 yea	HIV/AIDS	Rate	1991	111.813	145.936	83.2582
DALYs (Dis	Global	Both	50-69 yea	HIV/AIDS	Number	1991	1738572	2281936	1278998
DALYs (Dis	Global	Both	50-69 yea	HIV/AIDS	Rate	1991	250.476	328.758	184.265
DALYs (Dis	Global	Both	50-54 yea	Sexually tr	Number	1992	56999.8	88429.9	39383.3
DALYs (Dis	Global	Both	50-54 yea	Sexually tr	Rate	1992	26.3551	40.8874	18.2097
DALYs (Dis	Global	Both	55-59 yea	Sexually tr	Number	1992	50854	74738.9	35518.3
DALYs (Dis	Global	Both	55-59 yea	Sexually tr	Rate	1992	26.5959	39.0873	18.5755
DALYs (Dis	Global	Both	60-64 yea	Sexually tr	Number	1992	33758.8	51976.2	23318.6
DALYs (Dis	Global	Both	60-64 yea	Sexually tr	Rate	1992	20.2949	31.2467	14.0185
DALYs (Dis	Global	Both	65-69 yea	Sexually tr	Number	1992	24103.9	36818.4	17184.4
DALYs (Dis	Global	Both	65-69 yea	Sexually tr	Rate	1992	18.3849	28.0827	13.1072
DALYs (Dis	Global	Both	50-69 yea	Sexually tr	Number	1992	165717	252445	117472
DALYs (Dis	Global	Both	50-69 yea	Sexually tr	Rate	1992	23.508	35.811	16.6642
DALYs (Dis	Global	Both	50-54 yea	Syphilis	Number	1992	10134.9	13856.3	5935.89
DALYs (Dis	Global	Both	50-54 yea	Syphilis	Rate	1992	4.68608	6.40675	2.74458
DALYs (Dis	Global	Both	55-59 yea	Syphilis	Number	1992	10690.9	16668.7	5595.27
DALYs (Dis	Global	Both	55-59 yea	Syphilis	Rate	1992	5.59116	8.7175	2.92624
DALYs (Dis	Global	Both	60-64 yea	Syphilis	Number	1992	8492.58	11251.6	5403.77
DALYs (Dis	Global	Both	60-64 yea	Syphilis	Rate	1992	5.1055	6.76418	3.2486
DALYs (Dis	Global	Both	65-69 yea	Syphilis	Number	1992	5206.07	6508.14	4109.98
DALYs (Dis	Global	Both	65-69 yea	Syphilis	Rate	1992	3.97085	4.96398	3.13482
DALYs (Dis	Global	Both	50-69 yea	Syphilis	Number	1992	34524.4	46090.5	21533.4
DALYs (Dis	Global	Both	50-69 yea	Syphilis	Rate	1992	4.89753	6.53825	3.05466
DALYs (Dis	Global	Both	50-54 yea	Chlamydia	Number	1992	4325.71	5596.66	3282.45
DALYs (Dis	Global	Both	50-54 yea	Chlamydia	Rate	1992	2.00008	2.58773	1.51771
DALYs (Dis	Global	Both	55-59 yea	Chlamydia	Number	1992	3592.92	4433.44	2726.29
DALYs (Dis	Global	Both	55-59 yea	Chlamydia	Rate	1992	1.87904	2.31862	1.42581
DALYs (Dis	Global	Both	60-64 yea	Chlamydia	Number	1992	1795.28	2194.23	1321.87
DALYs (Dis	Global	Both	60-64 yea	Chlamydia	Rate	1992	1.07927	1.31911	0.79467
DALYs (Dis	Global	Both	65-69 yea	Chlamydia	Number	1992	932.472	1122.9	727.239
DALYs (Dis	Global	Both	65-69 yea	Chlamydia	Rate	1992	0.71123	0.85647	0.55469
DALYs (Dis	Global	Both	50-69 yea	Chlamydia	Number	1992	10646.4	12684.7	8453.78
DALYs (Dis	Global	Both	50-69 yea	Chlamydia	Rate	1992	1.51026	1.79942	1.19923
DALYs (Dis	Global	Both	50-54 yea	Gonococca	Number	1992	8621.11	10226.9	6403.6
DALYs (Dis	Global	Both	50-54 yea	Gonococca	Rate	1992	3.98615	4.72862	2.96084
DALYs (Dis	Global	Both	55-59 yea	Gonococca	Number	1992	8877.86	10936.5	6318.33
DALYs (Dis	Global	Both	55-59 yea	Gonococca	Rate	1992	4.64298	5.7196	3.30439
DALYs (Dis	Global	Both	60-64 yea	Gonococca	Number	1992	5254.61	6289.74	3973.71
DALYs (Dis	Global	Both	60-64 yea	Gonococca	Rate	1992	3.15893	3.78122	2.38889
DALYs (Dis	Global	Both	65-69 yea	Gonococca	Number	1992	5360.93	6435.53	4295.91
DALYs (Dis	Global	Both	65-69 yea	Gonococca	Rate	1992	4.08897	4.9086	3.27664
DALYs (Dis	Global	Both	50-69 yea	Gonococca	Number	1992	28114.5	32271	20977.8
DALYs (Dis	Global	Both	50-69 yea	Gonococca	Rate	1992	3.98824	4.57786	2.97585
DALYs (Dis	Global	Both	50-54 yea	Trichomon	Number	1992	10949.7	25949.2	3493.48
DALYs (Dis	Global	Both	50-54 yea	Trichomon	Rate	1992	5.06282	11.9981	1.61528
DALYs (Dis	Global	Both	55-59 yea	Trichomon	Number	1992	7684.67	17884.2	2496.95
DALYs (Dis	Global	Both	55-59 yea	Trichomon	Rate	1992	4.01897	9.35316	1.30587
DALYs (Dis	Global	Both	60-64 yea	Trichomon	Number	1992	5243.78	12338.2	1800.73
DALYs (Dis	Global	Both	60-64 yea	Trichomon	Rate	1992	3.15242	7.41741	1.08255
DALYs (Dis	Global	Both	65-69 yea	Trichomon	Number	1992	3665.5	8524.2	1299.14
DALYs (Dis	Global	Both	65-69 yea	Trichomon	Rate	1992	2.7958	6.5017	0.9909
DALYs (Dis	Global	Both	50-69 yea	Trichomon	Number	1992	27543.6	61488.4	10109.7
DALYs (Dis	Global	Both	50-69 yea	Trichomon	Rate	1992	3.90726	8.72255	1.43413
DALYs (Dis	Global	Both	50-54 yea	Genital he	Number	1992	12345.3	30750	3843.15
DALYs (Dis	Global	Both	50-54 yea	Genital he	Rate	1992	5.7081	14.2179	1.77696
DALYs (Dis	Global	Both	55-59 yea	Genital he	Number	1992	10500.4	25892.6	3215.27
DALYs (Dis	Global	Both	55-59 yea	Genital he	Rate	1992	5.49155	13.5414	1.68154
DALYs (Dis	Global	Both	60-64 yea	Genital he	Number	1992	8673.66	21076	2638.21
DALYs (Dis	Global	Both	60-64 yea	Genital he	Rate	1992	5.21437	12.6703	1.58602
DALYs (Dis	Global	Both	65-69 yea	Genital he	Number	1992	6490.89	15813.1	1962.3
DALYs (Dis	Global	Both	65-69 yea	Genital he	Rate	1992	4.95083	12.0612	1.49671
DALYs (Dis	Global	Both	50-69 yea	Genital he	Number	1992	38010.2	93506.6	11627.4
DALYs (Dis	Global	Both	50-69 yea	Genital he	Rate	1992	5.39202	13.2646	1.64943
DALYs (Dis	Global	Both	50-54 yea	Other sexu	Number	1992	10623.1	13362.7	8086.75
DALYs (Dis	Global	Both	50-54 yea	Other sexu	Rate	1992	4.91183	6.17852	3.73908
DALYs (Dis	Global	Both	55-59 yea	Other sexu	Number	1992	9507.29	11677.2	7173.3
DALYs (Dis	Global	Both	55-59 yea	Other sexu	Rate	1992	4.97216	6.10702	3.75152
DALYs (Dis	Global	Both	60-64 yea	Other sexu	Number	1992	4298.9	5204.04	3178.45
DALYs (Dis	Global	Both	60-64 yea	Other sexu	Rate	1992	2.58438	3.12852	1.91079
DALYs (Dis	Global	Both	65-69 yea	Other sexu	Number	1992	2448.06	2952.96	1963.69
DALYs (Dis	Global	Both	65-69 yea	Other sexu	Rate	1992	1.86722	2.25232	1.49777
DALYs (Dis	Global	Both	50-69 yea	Other sexu	Number	1992	26877.4	31771.9	21318.7
DALYs (Dis	Global	Both	50-69 yea	Other sexu	Rate	1992	3.81274	4.50707	3.02421
DALYs (Dis	Global	Both	50-54 yea	HIV/AIDS	Number	1994	1665822	2118041	1264489
DALYs (Dis	Global	Both	50-54 yea	HIV/AIDS	Rate	1994	763.639	970.943	579.661
DALYs (Dis	Global	Both	55-59 yea	HIV/AIDS	Number	1994	909139	1140498	695456
DALYs (Dis	Global	Both	55-59 yea	HIV/AIDS	Rate	1994	461.164	578.522	352.773
DALYs (Dis	Global	Both	60-64 yea	HIV/AIDS	Number	1994	493886	616529	381312
DALYs (Dis	Global	Both	60-64 yea	HIV/AIDS	Rate	1994	290.46	362.588	224.254
DALYs (Dis	Global	Both	65-69 yea	HIV/AIDS	Number	1994	266370	335209	204699
DALYs (Dis	Global	Both	65-69 yea	HIV/AIDS	Rate	1994	191.986	241.602	147.537
DALYs (Dis	Global	Both	50-69 yea	HIV/AIDS	Number	1994	3335217	4203330	2539386
DALYs (Dis	Global	Both	50-69 yea	HIV/AIDS	Rate	1994	460.626	580.52	350.714
DALYs (Dis	Global	Both	50-54 yea	Sexually tr	Number	1994	57193.8	88348.5	39394.4
DALYs (Dis	Global	Both	50-54 yea	Sexually tr	Rate	1994	26.2185	40.5003	18.059
DALYs (Dis	Global	Both	55-59 yea	Sexually tr	Number	1994	52234.9	77536	36475.6
DALYs (Dis	Global	Both	55-59 yea	Sexually tr	Rate	1994	26.4964	39.3304	18.5024
DALYs (Dis	Global	Both	60-64 yea	Sexually tr	Number	1994	34288.9	52960.2	23435.3
DALYs (Dis	Global	Both	60-64 yea	Sexually tr	Rate	1994	20.1657	31.1465	13.7826
DALYs (Dis	Global	Both	65-69 yea	Sexually tr	Number	1994	25427.6	38933	18051.2
DALYs (Dis	Global	Both	65-69 yea	Sexually tr	Rate	1994	18.327	28.061	13.0104
DALYs (Dis	Global	Both	50-69 yea	Sexually tr	Number	1994	169145	259099	119844
DALYs (Dis	Global	Both	50-69 yea	Sexually tr	Rate	1994	23.3606	35.7841	16.5516
DALYs (Dis	Global	Both	50-54 yea	Syphilis	Number	1994	10201.3	14073.9	5792.96
DALYs (Dis	Global	Both	50-54 yea	Syphilis	Rate	1994	4.67642	6.45168	2.65559
DALYs (Dis	Global	Both	55-59 yea	Syphilis	Number	1994	11413.8	17584.6	6196.65
DALYs (Dis	Global	Both	55-59 yea	Syphilis	Rate	1994	5.78971	8.91986	3.14327
DALYs (Dis	Global	Both	60-64 yea	Syphilis	Number	1994	8566.2	11273.1	5457.62
DALYs (Dis	Global	Both	60-64 yea	Syphilis	Rate	1994	5.03788	6.62984	3.20969
DALYs (Dis	Global	Both	65-69 yea	Syphilis	Number	1994	5706.27	6978.26	4344.99
DALYs (Dis	Global	Both	65-69 yea	Syphilis	Rate	1994	4.1128	5.02959	3.13166
DALYs (Dis	Global	Both	50-69 yea	Syphilis	Number	1994	35887.6	47737.7	22465.3
DALYs (Dis	Global	Both	50-69 yea	Syphilis	Rate	1994	4.95642	6.59303	3.10268
DALYs (Dis	Global	Both	50-54 yea	Chlamydia	Number	1994	4272.98	5542.77	3234.81
DALYs (Dis	Global	Both	50-54 yea	Chlamydia	Rate	1994	1.9588	2.54089	1.48289
DALYs (Dis	Global	Both	55-59 yea	Chlamydia	Number	1994	3587.91	4366.08	2793.35
DALYs (Dis	Global	Both	55-59 yea	Chlamydia	Rate	1994	1.81998	2.21471	1.41694
DALYs (Dis	Global	Both	60-64 yea	Chlamydia	Number	1994	1800.5	2194.89	1308.03
DALYs (Dis	Global	Both	60-64 yea	Chlamydia	Rate	1994	1.05889	1.29084	0.76927
DALYs (Dis	Global	Both	65-69 yea	Chlamydia	Number	1994	955.695	1122.62	771.313
DALYs (Dis	Global	Both	65-69 yea	Chlamydia	Rate	1994	0.68882	0.80913	0.55592
DALYs (Dis	Global	Both	50-69 yea	Chlamydia	Number	1994	10617.1	12655.1	8533.86
DALYs (Dis	Global	Both	50-69 yea	Chlamydia	Rate	1994	1.46632	1.74779	1.17861
DALYs (Dis	Global	Both	50-54 yea	Gonococca	Number	1994	8484.51	9960.35	6303.24
DALYs (Dis	Global	Both	50-54 yea	Gonococca	Rate	1994	3.88943	4.56598	2.8895
DALYs (Dis	Global	Both	55-59 yea	Gonococca	Number	1994	8829.82	10791.3	6435.27
DALYs (Dis	Global	Both	55-59 yea	Gonococca	Rate	1994	4.47896	5.47392	3.26431
DALYs (Dis	Global	Both	60-64 yea	Gonococca	Number	1994	5252.02	6294.81	3971.72
DALYs (Dis	Global	Both	60-64 yea	Gonococca	Rate	1994	3.08877	3.70205	2.33581
DALYs (Dis	Global	Both	65-69 yea	Gonococca	Number	1994	5493.38	6485.55	4491.58
DALYs (Dis	Global	Both	65-69 yea	Gonococca	Rate	1994	3.95936	4.67447	3.23731
DALYs (Dis	Global	Both	50-69 yea	Gonococca	Number	1994	28059.7	31930.3	21038.5
DALYs (Dis	Global	Both	50-69 yea	Gonococca	Rate	1994	3.87532	4.40989	2.90562
DALYs (Dis	Global	Both	50-54 yea	Trichomon	Number	1994	11200.1	26602.9	3576.97
DALYs (Dis	Global	Both	50-54 yea	Trichomon	Rate	1994	5.13431	12.1952	1.63974
DALYs (Dis	Global	Both	55-59 yea	Trichomon	Number	1994	8009.4	18654.3	2635.8
DALYs (Dis	Global	Both	55-59 yea	Trichomon	Rate	1994	4.0628	9.46245	1.33702
DALYs (Dis	Global	Both	60-64 yea	Trichomon	Number	1994	5439.26	12738.1	1867.52
DALYs (Dis	Global	Both	60-64 yea	Trichomon	Rate	1994	3.19889	7.49145	1.09831
DALYs (Dis	Global	Both	65-69 yea	Trichomon	Number	1994	3881.49	8991.01	1379.4
DALYs (Dis	Global	Both	65-69 yea	Trichomon	Rate	1994	2.79759	6.48028	0.9942
DALYs (Dis	Global	Both	50-69 yea	Trichomon	Number	1994	28530.3	63914.9	10429.9
DALYs (Dis	Global	Both	50-69 yea	Trichomon	Rate	1994	3.9403	8.82726	1.44046
DALYs (Dis	Global	Both	50-54 yea	Genital he	Number	1994	12617.4	31427.4	3933.82
DALYs (Dis	Global	Both	50-54 yea	Genital he	Rate	1994	5.78402	14.4068	1.80332
DALYs (Dis	Global	Both	55-59 yea	Genital he	Number	1994	10955.9	26882.8	3363.78
DALYs (Dis	Global	Both	55-59 yea	Genital he	Rate	1994	5.55745	13.6364	1.70629
DALYs (Dis	Global	Both	60-64 yea	Genital he	Number	1994	8943.9	21673.4	2722.23
DALYs (Dis	Global	Both	60-64 yea	Genital he	Rate	1994	5.26001	12.7464	1.60098
DALYs (Dis	Global	Both	65-69 yea	Genital he	Number	1994	6898	16904.1	2091.9
DALYs (Dis	Global	Both	65-69 yea	Genital he	Rate	1994	4.97174	12.1836	1.50774
DALYs (Dis	Global	Both	50-69 yea	Genital he	Number	1994	39415.3	96578.2	12070.8
DALYs (Dis	Global	Both	50-69 yea	Genital he	Rate	1994	5.44363	13.3384	1.66709
DALYs (Dis	Global	Both	50-54 yea	Other sexu	Number	1994	10417.5	13052.5	7945.71
DALYs (Dis	Global	Both	50-54 yea	Other sexu	Rate	1994	4.77554	5.98345	3.64244
DALYs (Dis	Global	Both	55-59 yea	Other sexu	Number	1994	9438.03	11519.1	7240.74
DALYs (Dis	Global	Both	55-59 yea	Other sexu	Rate	1994	4.78748	5.8431	3.67289
DALYs (Dis	Global	Both	60-64 yea	Other sexu	Number	1994	4287.05	5218.7	3144.53
DALYs (Dis	Global	Both	60-64 yea	Other sexu	Rate	1994	2.52126	3.06918	1.84933
DALYs (Dis	Global	Both	65-69 yea	Other sexu	Number	1994	2492.78	2984.43	2031.9
DALYs (Dis	Global	Both	65-69 yea	Other sexu	Rate	1994	1.79668	2.15103	1.46449
DALYs (Dis	Global	Both	50-69 yea	Other sexu	Number	1994	26635.4	31232.5	21229.1
DALYs (Dis	Global	Both	50-69 yea	Other sexu	Rate	1994	3.6786	4.31351	2.93194
DALYs (Dis	Global	Both	50-54 yea	Sexually tr	Number	2001	62303.7	101174	41327
DALYs (Dis	Global	Both	50-54 yea	Sexually tr	Rate	2001	22.6259	36.742	15.0081
DALYs (Dis	Global	Both	55-59 yea	Sexually tr	Number	2001	49135.1	75810.1	33473.2
DALYs (Dis	Global	Both	55-59 yea	Sexually tr	Rate	2001	23.4728	36.2159	15.9907
DALYs (Dis	Global	Both	60-64 yea	Sexually tr	Number	2001	33354.5	53870	22123
DALYs (Dis	Global	Both	60-64 yea	Sexually tr	Rate	2001	17.6357	28.4829	11.6972
DALYs (Dis	Global	Both	65-69 yea	Sexually tr	Number	2001	25435.2	40441.6	17321
DALYs (Dis	Global	Both	65-69 yea	Sexually tr	Rate	2001	16.314	25.9391	11.1096
DALYs (Dis	Global	Both	50-69 yea	Sexually tr	Number	2001	170228	274505	116786
DALYs (Dis	Global	Both	50-69 yea	Sexually tr	Rate	2001	20.516	33.0835	14.0751
DALYs (Dis	Global	Both	50-54 yea	Syphilis	Number	2001	9012.63	12621.6	5703.86
DALYs (Dis	Global	Both	50-54 yea	Syphilis	Rate	2001	3.27298	4.58358	2.07138
DALYs (Dis	Global	Both	55-59 yea	Syphilis	Number	2001	8849.92	13871.7	4691.69
DALYs (Dis	Global	Both	55-59 yea	Syphilis	Rate	2001	4.22777	6.62676	2.24131
DALYs (Dis	Global	Both	60-64 yea	Syphilis	Number	2001	7265.81	10027.7	4948.64
DALYs (Dis	Global	Both	60-64 yea	Syphilis	Rate	2001	3.84169	5.30197	2.61651
DALYs (Dis	Global	Both	65-69 yea	Syphilis	Number	2001	4544.15	5889.54	3680.32
DALYs (Dis	Global	Both	65-69 yea	Syphilis	Rate	2001	2.9146	3.77753	2.36054
DALYs (Dis	Global	Both	50-69 yea	Syphilis	Number	2001	29672.5	40557.2	19599.4
DALYs (Dis	Global	Both	50-69 yea	Syphilis	Rate	2001	3.57615	4.88798	2.36213
DALYs (Dis	Global	Both	50-54 yea	Chlamydia	Number	2001	4715.67	6311.49	3514.04
DALYs (Dis	Global	Both	50-54 yea	Chlamydia	Rate	2001	1.71252	2.29205	1.27614
DALYs (Dis	Global	Both	55-59 yea	Chlamydia	Number	2001	3375.94	4127.39	2631.42
DALYs (Dis	Global	Both	55-59 yea	Chlamydia	Rate	2001	1.61275	1.97173	1.25708
DALYs (Dis	Global	Both	60-64 yea	Chlamydia	Number	2001	1661.47	1981.33	1284.51
DALYs (Dis	Global	Both	60-64 yea	Chlamydia	Rate	2001	0.87848	1.0476	0.67916
DALYs (Dis	Global	Both	65-69 yea	Chlamydia	Number	2001	989.419	1186.24	781.844
DALYs (Dis	Global	Both	65-69 yea	Chlamydia	Rate	2001	0.63461	0.76085	0.50147
DALYs (Dis	Global	Both	50-69 yea	Chlamydia	Number	2001	10742.5	12912.4	8754.45
DALYs (Dis	Global	Both	50-69 yea	Chlamydia	Rate	2001	1.29469	1.55621	1.05509
DALYs (Dis	Global	Both	50-54 yea	Gonococca	Number	2001	8347.72	9728.77	6642.49
DALYs (Dis	Global	Both	50-54 yea	Gonococca	Rate	2001	3.03151	3.53305	2.41225
DALYs (Dis	Global	Both	55-59 yea	Gonococca	Number	2001	8107.07	9839.17	5889.12
DALYs (Dis	Global	Both	55-59 yea	Gonococca	Rate	2001	3.8729	4.70035	2.81334
DALYs (Dis	Global	Both	60-64 yea	Gonococca	Number	2001	4783.69	5637.68	3801.92
DALYs (Dis	Global	Both	60-64 yea	Gonococca	Rate	2001	2.5293	2.98083	2.01021
DALYs (Dis	Global	Both	65-69 yea	Gonococca	Number	2001	5562.43	6596.66	4444.76
DALYs (Dis	Global	Both	65-69 yea	Gonococca	Rate	2001	3.56772	4.23107	2.85085
DALYs (Dis	Global	Both	50-69 yea	Gonococca	Number	2001	26800.9	30335.4	20800.7
DALYs (Dis	Global	Both	50-69 yea	Gonococca	Rate	2001	3.23006	3.65604	2.50691
DALYs (Dis	Global	Both	50-54 yea	Trichomon	Number	2001	13758.3	32650.4	4385.71
DALYs (Dis	Global	Both	50-54 yea	Trichomon	Rate	2001	4.99638	11.8572	1.59269
DALYs (Dis	Global	Both	55-59 yea	Trichomon	Number	2001	8499.06	19847	2772.6
DALYs (Dis	Global	Both	55-59 yea	Trichomon	Rate	2001	4.06016	9.48129	1.32452
DALYs (Dis	Global	Both	60-64 yea	Trichomon	Number	2001	5963.95	13965.7	2033.83
DALYs (Dis	Global	Both	60-64 yea	Trichomon	Rate	2001	3.15334	7.38412	1.07536
DALYs (Dis	Global	Both	65-69 yea	Trichomon	Number	2001	4283.85	9950.8	1543.89
DALYs (Dis	Global	Both	65-69 yea	Trichomon	Rate	2001	2.74764	6.3824	0.99025
DALYs (Dis	Global	Both	50-69 yea	Trichomon	Number	2001	32505.1	71946.1	11921.1
DALYs (Dis	Global	Both	50-69 yea	Trichomon	Rate	2001	3.91753	8.67098	1.43674
DALYs (Dis	Global	Both	50-54 yea	Genital he	Number	2001	15547.4	38608.1	4810.17
DALYs (Dis	Global	Both	50-54 yea	Genital he	Rate	2001	5.64612	14.0207	1.74683
DALYs (Dis	Global	Both	55-59 yea	Genital he	Number	2001	11523.7	28605.6	3549.18
DALYs (Dis	Global	Both	55-59 yea	Genital he	Rate	2001	5.50507	13.6654	1.69551
DALYs (Dis	Global	Both	60-64 yea	Genital he	Number	2001	9794.68	23832.9	3002.89
DALYs (Dis	Global	Both	60-64 yea	Genital he	Rate	2001	5.17879	12.6013	1.58773
DALYs (Dis	Global	Both	65-69 yea	Genital he	Number	2001	7534.31	18322	2284.33
DALYs (Dis	Global	Both	65-69 yea	Genital he	Rate	2001	4.83247	11.7516	1.46516
DALYs (Dis	Global	Both	50-69 yea	Genital he	Number	2001	44400.1	109480	13655.3
DALYs (Dis	Global	Both	50-69 yea	Genital he	Rate	2001	5.35112	13.1946	1.64574
DALYs (Dis	Global	Both	50-54 yea	Other sexu	Number	2001	10922	13877.4	8389.71
DALYs (Dis	Global	Both	50-54 yea	Other sexu	Rate	2001	3.96637	5.03964	3.04676
DALYs (Dis	Global	Both	55-59 yea	Other sexu	Number	2001	8779.46	10724.2	6673.5
DALYs (Dis	Global	Both	55-59 yea	Other sexu	Rate	2001	4.19411	5.12314	3.18805
DALYs (Dis	Global	Both	60-64 yea	Other sexu	Number	2001	3884.87	4688.43	3048.14
DALYs (Dis	Global	Both	60-64 yea	Other sexu	Rate	2001	2.05407	2.47893	1.61166
DALYs (Dis	Global	Both	65-69 yea	Other sexu	Number	2001	2521.05	2975.45	2022.12
DALYs (Dis	Global	Both	65-69 yea	Other sexu	Rate	2001	1.61699	1.90844	1.29698
DALYs (Dis	Global	Both	50-69 yea	Other sexu	Number	2001	26107.4	30794.2	21142.3
DALYs (Dis	Global	Both	50-69 yea	Other sexu	Rate	2001	3.14647	3.71133	2.54808
DALYs (Dis	Global	Both	50-54 yea	Sexually tr	Number	1991	56508.1	87448.5	38707.9
DALYs (Dis	Global	Both	50-54 yea	Sexually tr	Rate	1991	26.2961	40.6943	18.0128
DALYs (Dis	Global	Both	55-59 yea	Sexually tr	Number	1991	49813.5	73239.5	35009.5
DALYs (Dis	Global	Both	55-59 yea	Sexually tr	Rate	1991	26.5026	38.9661	18.6264
DALYs (Dis	Global	Both	60-64 yea	Sexually tr	Number	1991	33216.7	51019.4	23007
DALYs (Dis	Global	Both	60-64 yea	Sexually tr	Rate	1991	20.2557	31.1119	14.0298
DALYs (Dis	Global	Both	65-69 yea	Sexually tr	Number	1991	23418.4	35817.3	16758.5
DALYs (Dis	Global	Both	65-69 yea	Sexually tr	Rate	1991	18.4001	28.1422	13.1674
DALYs (Dis	Global	Both	50-69 yea	Sexually tr	Number	1991	162957	248650	115148
DALYs (Dis	Global	Both	50-69 yea	Sexually tr	Rate	1991	23.4771	35.8229	16.5894
DALYs (Dis	Global	Both	50-54 yea	Syphilis	Number	1991	9959.45	13626	5808.32
DALYs (Dis	Global	Both	50-54 yea	Syphilis	Rate	1991	4.63464	6.34089	2.70291
DALYs (Dis	Global	Both	55-59 yea	Syphilis	Number	1991	10480.9	16649.7	5343.11
DALYs (Dis	Global	Both	55-59 yea	Syphilis	Rate	1991	5.57622	8.85825	2.84273
DALYs (Dis	Global	Both	60-64 yea	Syphilis	Number	1991	8334.47	11106.9	5297.07
DALYs (Dis	Global	Both	60-64 yea	Syphilis	Rate	1991	5.0824	6.77306	3.23018
DALYs (Dis	Global	Both	65-69 yea	Syphilis	Number	1991	4999.23	6272.62	3970.88
DALYs (Dis	Global	Both	65-69 yea	Syphilis	Rate	1991	3.92797	4.92849	3.11998
DALYs (Dis	Global	Both	50-69 yea	Syphilis	Number	1991	33774	45654.8	20918.1
DALYs (Dis	Global	Both	50-69 yea	Syphilis	Rate	1991	4.86582	6.57747	3.01367
DALYs (Dis	Global	Both	50-54 yea	Chlamydia	Number	1991	4329	5602.1	3251.68
DALYs (Dis	Global	Both	50-54 yea	Chlamydia	Rate	1991	2.0145	2.60694	1.51318
DALYs (Dis	Global	Both	55-59 yea	Chlamydia	Number	1991	3526.5	4343.17	2646.47
DALYs (Dis	Global	Both	55-59 yea	Chlamydia	Rate	1991	1.87623	2.31073	1.40802
DALYs (Dis	Global	Both	60-64 yea	Chlamydia	Number	1991	1777.4	2157.08	1303.24
DALYs (Dis	Global	Both	60-64 yea	Chlamydia	Rate	1991	1.08386	1.3154	0.79472
DALYs (Dis	Global	Both	65-69 yea	Chlamydia	Number	1991	916.252	1104.95	708.489
DALYs (Dis	Global	Both	65-69 yea	Chlamydia	Rate	1991	0.71991	0.86817	0.55667
DALYs (Dis	Global	Both	50-69 yea	Chlamydia	Number	1991	10549.1	12579.7	8411.55
DALYs (Dis	Global	Both	50-69 yea	Chlamydia	Rate	1991	1.51981	1.81235	1.21185
DALYs (Dis	Global	Both	50-54 yea	Gonococca	Number	1991	8613.23	10311.2	6512.2
DALYs (Dis	Global	Both	50-54 yea	Gonococca	Rate	1991	4.00818	4.79834	3.03046
DALYs (Dis	Global	Both	55-59 yea	Gonococca	Number	1991	8707.65	10732.1	6197.4
DALYs (Dis	Global	Both	55-59 yea	Gonococca	Rate	1991	4.63279	5.70986	3.29725
DALYs (Dis	Global	Both	60-64 yea	Gonococca	Number	1991	5213.95	6229.13	3979.6
DALYs (Dis	Global	Both	60-64 yea	Gonococca	Rate	1991	3.17949	3.79855	2.42678
DALYs (Dis	Global	Both	65-69 yea	Gonococca	Number	1991	5273.5	6308.65	4151.97
DALYs (Dis	Global	Both	65-69 yea	Gonococca	Rate	1991	4.14347	4.95679	3.26226
DALYs (Dis	Global	Both	50-69 yea	Gonococca	Number	1991	27808.3	32130.6	20745.4
DALYs (Dis	Global	Both	50-69 yea	Gonococca	Rate	1991	4.00634	4.62906	2.98879
DALYs (Dis	Global	Both	50-54 yea	Trichomon	Number	1991	10788.3	25450.2	3434.38
DALYs (Dis	Global	Both	50-54 yea	Trichomon	Rate	1991	5.02037	11.8433	1.59819
DALYs (Dis	Global	Both	55-59 yea	Trichomon	Number	1991	7483.3	17377.8	2419.92
DALYs (Dis	Global	Both	55-59 yea	Trichomon	Rate	1991	3.98139	9.24566	1.28749
DALYs (Dis	Global	Both	60-64 yea	Trichomon	Number	1991	5129.42	12036.2	1752.47
DALYs (Dis	Global	Both	60-64 yea	Trichomon	Rate	1991	3.12794	7.33973	1.06866
DALYs (Dis	Global	Both	65-69 yea	Trichomon	Number	1991	3545.09	8292.22	1267.11
DALYs (Dis	Global	Both	65-69 yea	Trichomon	Rate	1991	2.78542	6.51532	0.99558
DALYs (Dis	Global	Both	50-69 yea	Trichomon	Number	1991	26946.1	60297.1	9862.38
DALYs (Dis	Global	Both	50-69 yea	Trichomon	Rate	1991	3.88213	8.68699	1.42087
DALYs (Dis	Global	Both	50-54 yea	Genital he	Number	1991	12147.7	30292.2	3763.46
DALYs (Dis	Global	Both	50-54 yea	Genital he	Rate	1991	5.65295	14.0965	1.75133
DALYs (Dis	Global	Both	55-59 yea	Genital he	Number	1991	10230.3	25127.1	3106.52
DALYs (Dis	Global	Both	55-59 yea	Genital he	Rate	1991	5.44288	13.3686	1.65279
DALYs (Dis	Global	Both	60-64 yea	Genital he	Number	1991	8494.99	20649.4	2590.8
DALYs (Dis	Global	Both	60-64 yea	Genital he	Rate	1991	5.18028	12.5921	1.57988
DALYs (Dis	Global	Both	65-69 yea	Genital he	Number	1991	6270.67	15327.1	1899.28
DALYs (Dis	Global	Both	65-69 yea	Genital he	Rate	1991	4.92695	12.0427	1.49229
DALYs (Dis	Global	Both	50-69 yea	Genital he	Number	1991	37143.6	91303.4	11392.4
DALYs (Dis	Global	Both	50-69 yea	Genital he	Rate	1991	5.35127	13.1541	1.6413
DALYs (Dis	Global	Both	50-54 yea	Other sexu	Number	1991	10670.4	13348.8	8139.97
DALYs (Dis	Global	Both	50-54 yea	Other sexu	Rate	1991	4.96548	6.21189	3.78794
DALYs (Dis	Global	Both	55-59 yea	Other sexu	Number	1991	9384.88	11421.7	7096.12
DALYs (Dis	Global	Both	55-59 yea	Other sexu	Rate	1991	4.99311	6.07676	3.7754
DALYs (Dis	Global	Both	60-64 yea	Other sexu	Number	1991	4266.46	5181.58	3113.72
DALYs (Dis	Global	Both	60-64 yea	Other sexu	Rate	1991	2.60171	3.15975	1.89876
DALYs (Dis	Global	Both	65-69 yea	Other sexu	Number	1991	2413.61	2892.9	1928.21
DALYs (Dis	Global	Both	65-69 yea	Other sexu	Rate	1991	1.89641	2.27299	1.51503
DALYs (Dis	Global	Both	50-69 yea	Other sexu	Number	1991	26735.4	31452.1	21403.2
DALYs (Dis	Global	Both	50-69 yea	Other sexu	Rate	1991	3.85176	4.5313	3.08355
DALYs (Dis	Global	Both	50-54 yea	Sexually tr	Number	1997	57795	91764.3	39197.1
DALYs (Dis	Global	Both	50-54 yea	Sexually tr	Rate	1997	25.056	39.7829	16.9932
DALYs (Dis	Global	Both	55-59 yea	Sexually tr	Number	1997	51890.3	77391.3	36157.5
DALYs (Dis	Global	Both	55-59 yea	Sexually tr	Rate	1997	25.5798	38.1508	17.8242
DALYs (Dis	Global	Both	60-64 yea	Sexually tr	Number	1997	34158.3	54060.9	23063.8
DALYs (Dis	Global	Both	60-64 yea	Sexually tr	Rate	1997	19.2015	30.3894	12.9649
DALYs (Dis	Global	Both	65-69 yea	Sexually tr	Number	1997	25645.3	40010.7	17958.8
DALYs (Dis	Global	Both	65-69 yea	Sexually tr	Rate	1997	17.4118	27.1652	12.1931
DALYs (Dis	Global	Both	50-69 yea	Sexually tr	Number	1997	169489	265289	118707
DALYs (Dis	Global	Both	50-69 yea	Sexually tr	Rate	1997	22.3394	34.9662	15.6462
DALYs (Dis	Global	Both	50-54 yea	Syphilis	Number	1997	9381.58	13216	5666.24
DALYs (Dis	Global	Both	50-54 yea	Syphilis	Rate	1997	4.06723	5.72956	2.45651
DALYs (Dis	Global	Both	55-59 yea	Syphilis	Number	1997	10904.8	16826.8	5923.88
DALYs (Dis	Global	Both	55-59 yea	Syphilis	Rate	1997	5.37562	8.29493	2.92023
DALYs (Dis	Global	Both	60-64 yea	Syphilis	Number	1997	8155.98	10989.2	5376.8
DALYs (Dis	Global	Both	60-64 yea	Syphilis	Rate	1997	4.58474	6.17736	3.02247
DALYs (Dis	Global	Both	65-69 yea	Syphilis	Number	1997	5453.05	6892.08	4253.15
DALYs (Dis	Global	Both	65-69 yea	Syphilis	Rate	1997	3.70233	4.67936	2.88767
DALYs (Dis	Global	Both	50-69 yea	Syphilis	Number	1997	33895.4	45538.8	21736.6
DALYs (Dis	Global	Both	50-69 yea	Syphilis	Rate	1997	4.46756	6.00221	2.86498
DALYs (Dis	Global	Both	50-54 yea	Chlamydia	Number	1997	4387.45	5772.29	3339.6
DALYs (Dis	Global	Both	50-54 yea	Chlamydia	Rate	1997	1.90211	2.50248	1.44783
DALYs (Dis	Global	Both	55-59 yea	Chlamydia	Number	1997	3552.73	4342.93	2783.17
DALYs (Dis	Global	Both	55-59 yea	Chlamydia	Rate	1997	1.75135	2.14089	1.37199
DALYs (Dis	Global	Both	60-64 yea	Chlamydia	Number	1997	1757.74	2127.04	1321.66
DALYs (Dis	Global	Both	60-64 yea	Chlamydia	Rate	1997	0.98808	1.19568	0.74295
DALYs (Dis	Global	Both	65-69 yea	Chlamydia	Number	1997	959.663	1134.14	767.065
DALYs (Dis	Global	Both	65-69 yea	Chlamydia	Rate	1997	0.65156	0.77002	0.5208
DALYs (Dis	Global	Both	50-69 yea	Chlamydia	Number	1997	10657.6	12719.5	8686.82
DALYs (Dis	Global	Both	50-69 yea	Chlamydia	Rate	1997	1.40472	1.67649	1.14496
DALYs (Dis	Global	Both	50-54 yea	Gonococca	Number	1997	8476.52	10108.1	6385.53
DALYs (Dis	Global	Both	50-54 yea	Gonococca	Rate	1997	3.67485	4.38221	2.76834
DALYs (Dis	Global	Both	55-59 yea	Gonococca	Number	1997	8664.02	10537.7	6348.01
DALYs (Dis	Global	Both	55-59 yea	Gonococca	Rate	1997	4.27101	5.19468	3.12931
DALYs (Dis	Global	Both	60-64 yea	Gonococca	Number	1997	5074.7	6046.89	3901.67
DALYs (Dis	Global	Both	60-64 yea	Gonococca	Rate	1997	2.85265	3.39915	2.19325
DALYs (Dis	Global	Both	65-69 yea	Gonococca	Number	1997	5452.28	6416.75	4432.9
DALYs (Dis	Global	Both	65-69 yea	Gonococca	Rate	1997	3.70181	4.35664	3.00971
DALYs (Dis	Global	Both	50-69 yea	Gonococca	Number	1997	27667.5	31243.9	20926.8
DALYs (Dis	Global	Both	50-69 yea	Gonococca	Rate	1997	3.6467	4.11808	2.75824
DALYs (Dis	Global	Both	50-54 yea	HIV/AIDS	Number	1998	2869129	3398730	2332203
DALYs (Dis	Global	Both	50-54 yea	HIV/AIDS	Rate	1998	1194.95	1415.52	971.329
DALYs (Dis	Global	Both	55-59 yea	HIV/AIDS	Number	1998	1510677	1770364	1245817
DALYs (Dis	Global	Both	55-59 yea	HIV/AIDS	Rate	1998	741.918	869.454	611.841
DALYs (Dis	Global	Both	60-64 yea	HIV/AIDS	Number	1998	817119	955047	682733
DALYs (Dis	Global	Both	60-64 yea	HIV/AIDS	Rate	1998	451.016	527.146	376.841
DALYs (Dis	Global	Both	65-69 yea	HIV/AIDS	Number	1998	439313	516349	367148
DALYs (Dis	Global	Both	65-69 yea	HIV/AIDS	Rate	1998	294.301	345.908	245.956
DALYs (Dis	Global	Both	50-69 yea	HIV/AIDS	Number	1998	5636238	6630522	4624096
DALYs (Dis	Global	Both	50-69 yea	HIV/AIDS	Rate	1998	728.037	856.47	597.298
DALYs (Dis	Global	Both	50-54 yea	HIV/AIDS	Number	2002	4272291	4710399	3780845
DALYs (Dis	Global	Both	50-54 yea	HIV/AIDS	Rate	2002	1499.79	1653.58	1327.27
DALYs (Dis	Global	Both	55-59 yea	HIV/AIDS	Number	2002	2080030	2268250	1884916
DALYs (Dis	Global	Both	55-59 yea	HIV/AIDS	Rate	2002	954.992	1041.41	865.41
DALYs (Dis	Global	Both	60-64 yea	HIV/AIDS	Number	2002	1089317	1192546	997887
DALYs (Dis	Global	Both	60-64 yea	HIV/AIDS	Rate	2002	571.658	625.831	523.677
DALYs (Dis	Global	Both	65-69 yea	HIV/AIDS	Number	2002	573956	628852	526224
DALYs (Dis	Global	Both	65-69 yea	HIV/AIDS	Rate	2002	360.253	394.71	330.293
DALYs (Dis	Global	Both	50-69 yea	HIV/AIDS	Number	2002	8015594	8787418	7212575
DALYs (Dis	Global	Both	50-69 yea	HIV/AIDS	Rate	2002	940.201	1030.73	846.01
DALYs (Dis	Global	Both	50-54 yea	HIV/AIDS	Number	1993	1363458	1764831	1016101
DALYs (Dis	Global	Both	50-54 yea	HIV/AIDS	Rate	1993	628.015	812.889	468.02
DALYs (Dis	Global	Both	55-59 yea	HIV/AIDS	Number	1993	744598	952146	558413
DALYs (Dis	Global	Both	55-59 yea	HIV/AIDS	Rate	1993	383.115	489.904	287.318
DALYs (Dis	Global	Both	60-64 yea	HIV/AIDS	Number	1993	405639	516577	307174
DALYs (Dis	Global	Both	60-64 yea	HIV/AIDS	Rate	1993	240.929	306.821	182.446
DALYs (Dis	Global	Both	65-69 yea	HIV/AIDS	Number	1993	220550	283208	166304
DALYs (Dis	Global	Both	65-69 yea	HIV/AIDS	Rate	1993	163.442	209.876	123.243
DALYs (Dis	Global	Both	50-69 yea	HIV/AIDS	Number	1993	2734245	3527778	2047947
DALYs (Dis	Global	Both	50-69 yea	HIV/AIDS	Rate	1993	382.538	493.558	286.521
DALYs (Dis	Global	Both	50-54 yea	HIV/AIDS	Number	1995	1975580	2457776	1519520
DALYs (Dis	Global	Both	50-54 yea	HIV/AIDS	Rate	1995	901.705	1121.79	693.547
DALYs (Dis	Global	Both	55-59 yea	HIV/AIDS	Number	1995	1075673	1320626	837903
DALYs (Dis	Global	Both	55-59 yea	HIV/AIDS	Rate	1995	538.412	661.02	419.4
DALYs (Dis	Global	Both	60-64 yea	HIV/AIDS	Number	1995	582456	713778	459019
DALYs (Dis	Global	Both	60-64 yea	HIV/AIDS	Rate	1995	338.641	414.992	266.875
DALYs (Dis	Global	Both	65-69 yea	HIV/AIDS	Number	1995	314080	387318	245714
DALYs (Dis	Global	Both	65-69 yea	HIV/AIDS	Rate	1995	221.082	272.634	172.959
DALYs (Dis	Global	Both	50-69 yea	HIV/AIDS	Number	1995	3947789	4854949	3066402
DALYs (Dis	Global	Both	50-69 yea	HIV/AIDS	Rate	1995	538.621	662.391	418.368
DALYs (Dis	Global	Both	50-54 yea	Trichomon	Number	1997	11838	27950.9	3790.15
DALYs (Dis	Global	Both	50-54 yea	Trichomon	Rate	1997	5.13216	12.1177	1.64316
DALYs (Dis	Global	Both	55-59 yea	Trichomon	Number	1997	8262.5	19161.3	2720.12
DALYs (Dis	Global	Both	55-59 yea	Trichomon	Rate	1997	4.07308	9.44574	1.34091
DALYs (Dis	Global	Both	60-64 yea	Trichomon	Number	1997	5699.41	13372.5	1943.79
DALYs (Dis	Global	Both	60-64 yea	Trichomon	Rate	1997	3.20382	7.51712	1.09267
DALYs (Dis	Global	Both	65-69 yea	Trichomon	Number	1997	4079.74	9476.76	1463.9
DALYs (Dis	Global	Both	65-69 yea	Trichomon	Rate	1997	2.76993	6.43423	0.99391
DALYs (Dis	Global	Both	50-69 yea	Trichomon	Number	1997	29879.6	67027.8	10903.2
DALYs (Dis	Global	Both	50-69 yea	Trichomon	Rate	1997	3.93827	8.83456	1.43709
DALYs (Dis	Global	Both	50-54 yea	Genital he	Number	1997	13293.5	33038.4	4135.83
DALYs (Dis	Global	Both	50-54 yea	Genital he	Rate	1997	5.76316	14.3233	1.79302
DALYs (Dis	Global	Both	55-59 yea	Genital he	Number	1997	11260.4	27629.9	3458.86
DALYs (Dis	Global	Both	55-59 yea	Genital he	Rate	1997	5.55094	13.6204	1.70508
DALYs (Dis	Global	Both	60-64 yea	Genital he	Number	1997	9306.87	22664	2835.44
DALYs (Dis	Global	Both	60-64 yea	Genital he	Rate	1997	5.23169	12.7402	1.5939
DALYs (Dis	Global	Both	65-69 yea	Genital he	Number	1997	7239.62	17672.3	2187.9
DALYs (Dis	Global	Both	65-69 yea	Genital he	Rate	1997	4.91532	11.9986	1.48547
DALYs (Dis	Global	Both	50-69 yea	Genital he	Number	1997	41100.4	100757	12590.7
DALYs (Dis	Global	Both	50-69 yea	Genital he	Rate	1997	5.41721	13.2803	1.65951
DALYs (Dis	Global	Both	50-54 yea	Other sexu	Number	1997	10418	13140.7	7991.65
DALYs (Dis	Global	Both	50-54 yea	Other sexu	Rate	1997	4.51653	5.69693	3.46465

DALYs (Dis	Global	Both	65-69 yea	Sexually tr	Rate	1993	18.3899	28.1629	13.0661
DALYs (Dis	Global	Both	50-69 yea	Sexually tr	Number	1993	167323	256349	117723
DALYs (Dis	Global	Both	50-69 yea	Sexually tr	Rate	1993	23.4096	35.8649	16.4702
DALYs (Dis	Global	Both	50-54 yea	Syphilis	Number	1993	10166.3	13849.9	5887.92
DALYs (Dis	Global	Both	50-54 yea	Syphilis	Rate	1993	4.68266	6.3793	2.712
DALYs (Dis	Global	Both	55-59 yea	Syphilis	Number	1993	10990.2	17107.1	5759.77
DALYs (Dis	Global	Both	55-59 yea	Syphilis	Rate	1993	5.65477	8.80203	2.96355
DALYs (Dis	Global	Both	60-64 yea	Syphilis	Number	1993	8575.5	11297.5	5483.07
DALYs (Dis	Global	Both	60-64 yea	Syphilis	Rate	1993	5.09342	6.71016	3.25667
DALYs (Dis	Global	Both	65-69 yea	Syphilis	Number	1993	5472.81	6709.75	4226.88
DALYs (Dis	Global	Both	65-69 yea	Syphilis	Rate	1993	4.05573	4.97239	3.13241
DALYs (Dis	Global	Both	50-69 yea	Syphilis	Number	1993	35204.9	46805.1	21911.4
DALYs (Dis	Global	Both	50-69 yea	Syphilis	Rate	1993	4.92539	6.54832	3.06554
DALYs (Dis	Global	Both	50-54 yea	Chlamydia	Number	1993	4273.65	5518.8	3231.69
DALYs (Dis	Global	Both	50-54 yea	Chlamydia	Rate	1993	1.96846	2.54198	1.48853
DALYs (Dis	Global	Both	55-59 yea	Chlamydia	Number	1993	3587.29	4366.31	2751.44
DALYs (Dis	Global	Both	55-59 yea	Chlamydia	Rate	1993	1.84575	2.24658	1.41569
DALYs (Dis	Global	Both	60-64 yea	Chlamydia	Number	1993	1798.17	2182.98	1320.37
DALYs (Dis	Global	Both	60-64 yea	Chlamydia	Rate	1993	1.06803	1.29658	0.78424
DALYs (Dis	Global	Both	65-69 yea	Chlamydia	Number	1993	946.967	1124.15	759.965
DALYs (Dis	Global	Both	65-69 yea	Chlamydia	Rate	1993	0.70177	0.83307	0.56319
DALYs (Dis	Global	Both	50-69 yea	Chlamydia	Number	1993	10606.1	12690.8	8459.34
DALYs (Dis	Global	Both	50-69 yea	Chlamydia	Rate	1993	1.48386	1.77552	1.18352
DALYs (Dis	Global	Both	50-54 yea	Gonococca	Number	1993	8478.54	10014.1	6398.64
DALYs (Dis	Global	Both	50-54 yea	Gonococca	Rate	1993	3.90525	4.61254	2.94724
DALYs (Dis	Global	Both	55-59 yea	Gonococca	Number	1993	8843.27	10824.4	6337.57
DALYs (Dis	Global	Both	55-59 yea	Gonococca	Rate	1993	4.55009	5.56941	3.26085
DALYs (Dis	Global	Both	60-64 yea	Gonococca	Number	1993	5259.52	6278.05	4019.33
DALYs (Dis	Global	Both	60-64 yea	Gonococca	Rate	1993	3.1239	3.72886	2.38728
DALYs (Dis	Global	Both	65-69 yea	Gonococca	Number	1993	5444.32	6422.07	4443.78
DALYs (Dis	Global	Both	65-69 yea	Gonococca	Rate	1993	4.03461	4.75919	3.29314
DALYs (Dis	Global	Both	50-69 yea	Gonococca	Number	1993	28025.6	31917.7	21103.2
DALYs (Dis	Global	Both	50-69 yea	Gonococca	Rate	1993	3.92096	4.46549	2.95248
DALYs (Dis	Global	Both	50-54 yea	Trichomon	Number	1993	11072.3	26205.3	3559.03
DALYs (Dis	Global	Both	50-54 yea	Trichomon	Rate	1993	5.09995	12.0703	1.6393
DALYs (Dis	Global	Both	55-59 yea	Trichomon	Number	1993	7859.44	18222.3	2563.87
DALYs (Dis	Global	Both	55-59 yea	Trichomon	Rate	1993	4.04389	9.37584	1.31918
DALYs (Dis	Global	Both	60-64 yea	Trichomon	Number	1993	5350.44	12585.2	1830.37
DALYs (Dis	Global	Both	60-64 yea	Trichomon	Rate	1993	3.1779	7.47497	1.08715
DALYs (Dis	Global	Both	65-69 yea	Trichomon	Number	1993	3774.77	8800.12	1343.75
DALYs (Dis	Global	Both	65-69 yea	Trichomon	Rate	1993	2.79736	6.52149	0.99581
DALYs (Dis	Global	Both	50-69 yea	Trichomon	Number	1993	28056.9	62627.8	10246.6
DALYs (Dis	Global	Both	50-69 yea	Trichomon	Rate	1993	3.92534	8.76202	1.43356
DALYs (Dis	Global	Both	50-54 yea	Genital her	Number	1993	12488.3	31022.2	3884.32
DALYs (Dis	Global	Both	50-54 yea	Genital her	Rate	1993	5.75215	14.2889	1.78913
DALYs (Dis	Global	Both	55-59 yea	Genital her	Number	1993	10746.6	26527.8	3335.99
DALYs (Dis	Global	Both	55-59 yea	Genital her	Rate	1993	5.52941	13.6492	1.71645
DALYs (Dis	Global	Both	60-64 yea	Genital her	Number	1993	8823.2	21461.5	2683.92
DALYs (Dis	Global	Both	60-64 yea	Genital her	Rate	1993	5.24055	12.7471	1.59412
DALYs (Dis	Global	Both	65-69 yea	Genital her	Number	1993	6700.73	16336.9	2027.38
DALYs (Dis	Global	Both	65-69 yea	Genital her	Rate	1993	4.9657	12.1068	1.50243
DALYs (Dis	Global	Both	50-69 yea	Genital her	Number	1993	38758.8	95236.6	11869.4
DALYs (Dis	Global	Both	50-69 yea	Genital her	Rate	1993	5.4226	13.3242	1.6606
DALYs (Dis	Global	Both	50-54 yea	Other sexu	Number	1993	10454	13029.2	7924.13
DALYs (Dis	Global	Both	50-54 yea	Other sexu	Rate	1993	4.81515	6.00131	3.64989
DALYs (Dis	Global	Both	55-59 yea	Other sexu	Number	1993	9457.09	11568.8	7103.23
DALYs (Dis	Global	Both	55-59 yea	Other sexu	Rate	1993	4.86592	5.95243	3.6548
DALYs (Dis	Global	Both	60-64 yea	Other sexu	Number	1993	4284.11	5217.33	3166.91
DALYs (Dis	Global	Both	60-64 yea	Other sexu	Rate	1993	2.54455	3.09884	1.88099
DALYs (Dis	Global	Both	65-69 yea	Other sexu	Number	1993	2475.73	2937.38	2025.64
DALYs (Dis	Global	Both	65-69 yea	Other sexu	Rate	1993	1.83468	2.1768	1.50114
DALYs (Dis	Global	Both	50-69 yea	Other sexu	Number	1993	26670.9	31173	21212.2
DALYs (Dis	Global	Both	50-69 yea	Other sexu	Rate	1993	3.73143	4.3613	2.96772
DALYs (Dis	Global	Both	50-54 yea	Sexually tr	Number	2009	68897.7	115758	43180.2
DALYs (Dis	Global	Both	50-54 yea	Sexually tr	Rate	2009	20.3022	34.1107	12.724
DALYs (Dis	Global	Both	55-59 yea	Sexually tr	Number	2009	56353.2	91596	36729
DALYs (Dis	Global	Both	55-59 yea	Sexually tr	Rate	2009	19.3576	31.4637	12.6166
DALYs (Dis	Global	Both	60-64 yea	Sexually tr	Number	2009	33725.3	57279.4	21205.5
DALYs (Dis	Global	Both	60-64 yea	Sexually tr	Rate	2009	14.7217	25.0034	9.25654
DALYs (Dis	Global	Both	65-69 yea	Sexually tr	Number	2009	25079.5	41516.7	16115.8
DALYs (Dis	Global	Both	65-69 yea	Sexually tr	Rate	2009	14.222	23.5432	9.1389
DALYs (Dis	Global	Both	50-69 yea	Sexually tr	Number	2009	184056	307151	117843
DALYs (Dis	Global	Both	50-69 yea	Sexually tr	Rate	2009	17.7676	29.6505	11.3759
DALYs (Dis	Global	Both	50-54 yea	Syphilis	Number	2009	7477.31	9926.11	4972.69
DALYs (Dis	Global	Both	50-54 yea	Syphilis	Rate	2009	2.20336	2.92495	1.46531
DALYs (Dis	Global	Both	55-59 yea	Syphilis	Number	2009	7658.66	10970.4	4320.73
DALYs (Dis	Global	Both	55-59 yea	Syphilis	Rate	2009	2.63079	3.76838	1.4842
DALYs (Dis	Global	Both	60-64 yea	Syphilis	Number	2009	5432.18	7214.22	3785.65
DALYs (Dis	Global	Both	60-64 yea	Syphilis	Rate	2009	2.37124	3.14913	1.6525
DALYs (Dis	Global	Both	65-69 yea	Syphilis	Number	2009	3415.2	4628.17	2784.38
DALYs (Dis	Global	Both	65-69 yea	Syphilis	Rate	2009	1.93668	2.62453	1.57896
DALYs (Dis	Global	Both	50-69 yea	Syphilis	Number	2009	23983.3	30729	16341.2
DALYs (Dis	Global	Both	50-69 yea	Syphilis	Rate	2009	2.31521	2.9664	1.57748
DALYs (Dis	Global	Both	50-54 yea	Chlamydia	Number	2009	5143.14	7011.82	3779.86
DALYs (Dis	Global	Both	50-54 yea	Chlamydia	Rate	2009	1.51554	2.06619	1.11382
DALYs (Dis	Global	Both	55-59 yea	Chlamydia	Number	2009	3691.89	4430.49	2951.59
DALYs (Dis	Global	Both	55-59 yea	Chlamydia	Rate	2009	1.26819	1.5219	1.01389
DALYs (Dis	Global	Both	60-64 yea	Chlamydia	Number	2009	1567.07	1835.51	1248.26
DALYs (Dis	Global	Both	60-64 yea	Chlamydia	Rate	2009	0.68405	0.80123	0.54489
DALYs (Dis	Global	Both	65-69 yea	Chlamydia	Number	2009	933.088	1109.31	741.198
DALYs (Dis	Global	Both	65-69 yea	Chlamydia	Rate	2009	0.52913	0.62906	0.42032
DALYs (Dis	Global	Both	50-69 yea	Chlamydia	Number	2009	11335.2	13798.7	9308.25
DALYs (Dis	Global	Both	50-69 yea	Chlamydia	Rate	2009	1.09423	1.33204	0.89856
DALYs (Dis	Global	Both	50-54 yea	Gonococca	Number	2009	8466	9844.43	6967.63
DALYs (Dis	Global	Both	50-54 yea	Gonococca	Rate	2009	2.4947	2.90088	2.05317
DALYs (Dis	Global	Both	55-59 yea	Gonococca	Number	2009	8350.98	9782.52	6369.03
DALYs (Dis	Global	Both	55-59 yea	Gonococca	Rate	2009	2.86861	3.36035	2.1878
DALYs (Dis	Global	Both	60-64 yea	Gonococca	Number	2009	4402.29	5088.92	3520.64
DALYs (Dis	Global	Both	60-64 yea	Gonococca	Rate	2009	1.92167	2.2214	1.53682
DALYs (Dis	Global	Both	65-69 yea	Gonococca	Number	2009	5194	6083.15	4146.31
DALYs (Dis	Global	Both	65-69 yea	Gonococca	Rate	2009	2.94539	3.44961	2.35128
DALYs (Dis	Global	Both	50-69 yea	Gonococca	Number	2009	26413.3	29516.7	21012.2
DALYs (Dis	Global	Both	50-69 yea	Gonococca	Rate	2009	2.54978	2.84937	2.02839
DALYs (Dis	Global	Both	50-54 yea	Trichomon	Number	2009	17114.4	40357.6	5471.71
DALYs (Dis	Global	Both	50-54 yea	Trichomon	Rate	2009	5.04313	11.8923	1.61236
DALYs (Dis	Global	Both	55-59 yea	Trichomon	Number	2009	11614.7	26896.6	3772.18
DALYs (Dis	Global	Both	55-59 yea	Trichomon	Rate	2009	3.9897	9.23914	1.29576
DALYs (Dis	Global	Both	60-64 yea	Trichomon	Number	2009	7043.67	16468.6	2447.49
DALYs (Dis	Global	Both	60-64 yea	Trichomon	Rate	2009	3.07468	7.18883	1.06837
DALYs (Dis	Global	Both	65-69 yea	Trichomon	Number	2009	4743.96	10965.4	1687.08
DALYs (Dis	Global	Both	65-69 yea	Trichomon	Rate	2009	2.69019	6.21822	0.9567
DALYs (Dis	Global	Both	50-69 yea	Trichomon	Number	2009	40516.7	89282.6	14848.4
DALYs (Dis	Global	Both	50-69 yea	Trichomon	Rate	2009	3.91123	8.6188	1.43338
DALYs (Dis	Global	Both	50-54 yea	Genital her	Number	2009	19053.8	47368.3	5931.98
DALYs (Dis	Global	Both	50-54 yea	Genital her	Rate	2009	5.61464	13.9581	1.74799
DALYs (Dis	Global	Both	55-59 yea	Genital her	Number	2009	15626.7	38482.9	4824.61
DALYs (Dis	Global	Both	55-59 yea	Genital her	Rate	2009	5.36784	13.2191	1.65728
DALYs (Dis	Global	Both	60-64 yea	Genital her	Number	2009	11619.3	28537.6	3526.77
DALYs (Dis	Global	Both	60-64 yea	Genital her	Rate	2009	5.072	12.4572	1.53949
DALYs (Dis	Global	Both	65-69 yea	Genital her	Number	2009	8435.32	20521.4	2554.93
DALYs (Dis	Global	Both	65-69 yea	Genital her	Rate	2009	4.78347	11.6372	1.44884
DALYs (Dis	Global	Both	50-69 yea	Genital her	Number	2009	54735.1	134786	16764.2
DALYs (Dis	Global	Both	50-69 yea	Genital her	Rate	2009	5.28379	13.0115	1.61831
DALYs (Dis	Global	Both	50-54 yea	Other sexu	Number	2009	11643	14906.7	8869.63
DALYs (Dis	Global	Both	50-54 yea	Other sexu	Rate	2009	3.43088	4.39259	2.61364
DALYs (Dis	Global	Both	55-59 yea	Other sexu	Number	2009	9410.36	11408.3	7462.26
DALYs (Dis	Global	Both	55-59 yea	Other sexu	Rate	2009	3.23251	3.91883	2.56333
DALYs (Dis	Global	Both	60-64 yea	Other sexu	Number	2009	3660.88	4280.76	2923.12
DALYs (Dis	Global	Both	60-64 yea	Other sexu	Rate	2009	1.59804	1.86863	1.27599
DALYs (Dis	Global	Both	65-69 yea	Other sexu	Number	2009	2357.94	2779.61	1901.49
DALYs (Dis	Global	Both	65-69 yea	Other sexu	Rate	2009	1.33713	1.57625	1.07829
DALYs (Dis	Global	Both	50-69 yea	Other sexu	Number	2009	27072.2	32132.7	22336.7
DALYs (Dis	Global	Both	50-69 yea	Other sexu	Rate	2009	2.61339	3.10189	2.15625
DALYs (Dis	Global	Both	50-54 yea	Sexually tr	Number	1995	56722.2	88939.9	39141.2
DALYs (Dis	Global	Both	50-54 yea	Sexually tr	Rate	1995	25.8895	40.5944	17.865
DALYs (Dis	Global	Both	55-59 yea	Sexually tr	Number	1995	52363.5	77614.7	36244.7
DALYs (Dis	Global	Both	55-59 yea	Sexually tr	Rate	1995	26.2098	38.8489	18.1418
DALYs (Dis	Global	Both	60-64 yea	Sexually tr	Number	1995	34056.2	53111.9	23273.9
DALYs (Dis	Global	Both	60-64 yea	Sexually tr	Rate	1995	19.8003	30.8793	13.5315
DALYs (Dis	Global	Both	65-69 yea	Sexually tr	Number	1995	25621.2	39632.3	18087.9
DALYs (Dis	Global	Both	65-69 yea	Sexually tr	Rate	1995	18.0349	27.8973	12.7322
DALYs (Dis	Global	Both	50-69 yea	Sexually tr	Number	1995	168763	259627	119595
DALYs (Dis	Global	Both	50-69 yea	Sexually tr	Rate	1995	23.0254	35.4225	16.3171
DALYs (Dis	Global	Both	50-54 yea	Syphilis	Number	1995	9968.66	13702.8	5700.16
DALYs (Dis	Global	Both	50-54 yea	Syphilis	Rate	1995	4.54995	6.2543	2.6017
DALYs (Dis	Global	Both	55-59 yea	Syphilis	Number	1995	11641.4	18059.2	6375.04
DALYs (Dis	Global	Both	55-59 yea	Syphilis	Rate	1995	5.82691	9.03929	3.19093
DALYs (Dis	Global	Both	60-64 yea	Syphilis	Number	1995	8481.7	11322	5484.89
DALYs (Dis	Global	Both	60-64 yea	Syphilis	Rate	1995	4.93128	6.58266	3.18892
DALYs (Dis	Global	Both	65-69 yea	Syphilis	Number	1995	5803.69	7139.4	4402.69
DALYs (Dis	Global	Both	65-69 yea	Syphilis	Rate	1995	4.08524	5.02545	3.09907
DALYs (Dis	Global	Both	50-69 yea	Syphilis	Number	1995	35895.4	47594.3	22586.1
DALYs (Dis	Global	Both	50-69 yea	Syphilis	Rate	1995	4.89743	6.49358	3.08156
DALYs (Dis	Global	Both	50-54 yea	Chlamydia	Number	1995	4213.48	5475.82	3187.33
DALYs (Dis	Global	Both	50-54 yea	Chlamydia	Rate	1995	1.92314	2.4993	1.45478
DALYs (Dis	Global	Both	55-59 yea	Chlamydia	Number	1995	3527.01	4322.09	2752.04
DALYs (Dis	Global	Both	55-59 yea	Chlamydia	Rate	1995	1.76539	2.16336	1.37749
DALYs (Dis	Global	Both	60-64 yea	Chlamydia	Number	1995	1742.34	2111.09	1304
DALYs (Dis	Global	Both	60-64 yea	Chlamydia	Rate	1995	1.013	1.22739	0.75815
DALYs (Dis	Global	Both	65-69 yea	Chlamydia	Number	1995	941.021	1124.19	754.883
DALYs (Dis	Global	Both	65-69 yea	Chlamydia	Rate	1995	0.66239	0.79132	0.53136
DALYs (Dis	Global	Both	50-69 yea	Chlamydia	Number	1995	10423.9	12352.8	8368.53
DALYs (Dis	Global	Both	50-69 yea	Chlamydia	Rate	1995	1.42219	1.68537	1.14177
DALYs (Dis	Global	Both	50-54 yea	Gonococca	Number	1995	8281.27	9778.38	6215.1
DALYs (Dis	Global	Both	50-54 yea	Gonococca	Rate	1995	3.77978	4.4631	2.83673
DALYs (Dis	Global	Both	55-59 yea	Gonococca	Number	1995	8634.07	10648.6	6419.66
DALYs (Dis	Global	Both	55-59 yea	Gonococca	Rate	1995	4.32166	5.33	3.21326
DALYs (Dis	Global	Both	60-64 yea	Gonococca	Number	1995	5080.63	6022.46	3912.65
DALYs (Dis	Global	Both	60-64 yea	Gonococca	Rate	1995	2.95389	3.50147	2.27482
DALYs (Dis	Global	Both	65-69 yea	Gonococca	Number	1995	5400.03	6377.23	4419.89
DALYs (Dis	Global	Both	65-69 yea	Gonococca	Rate	1995	3.8011	4.48895	3.11118
DALYs (Dis	Global	Both	50-69 yea	Gonococca	Number	1995	27396	31028.9	20889.1
DALYs (Dis	Global	Both	50-69 yea	Gonococca	Rate	1995	3.73781	4.23346	2.85003
DALYs (Dis	Global	Both	50-54 yea	Trichomon	Number	1995	11309.5	26834.1	3596.82
DALYs (Dis	Global	Both	50-54 yea	Trichomon	Rate	1995	5.16194	12.2477	1.64168
DALYs (Dis	Global	Both	55-59 yea	Trichomon	Number	1995	8148.02	18969.3	2663.48
DALYs (Dis	Global	Both	55-59 yea	Trichomon	Rate	1995	4.07837	9.49482	1.33317
DALYs (Dis	Global	Both	60-64 yea	Trichomon	Number	1995	5530.63	12977.2	1896.04
DALYs (Dis	Global	Both	60-64 yea	Trichomon	Rate	1995	3.21552	7.54496	1.10236
DALYs (Dis	Global	Both	65-69 yea	Trichomon	Number	1995	3970.8	9253.37	1412.74
DALYs (Dis	Global	Both	65-69 yea	Trichomon	Rate	1995	2.79506	6.51347	0.99443
DALYs (Dis	Global	Both	50-69 yea	Trichomon	Number	1995	28958.9	65117.6	10537.6
DALYs (Dis	Global	Both	50-69 yea	Trichomon	Rate	1995	3.95105	8.88439	1.43771
DALYs (Dis	Global	Both	50-54 yea	Genital her	Number	1995	12722.1	31639.9	3949.89
DALYs (Dis	Global	Both	50-54 yea	Genital her	Rate	1995	5.80669	14.4413	1.80283
DALYs (Dis	Global	Both	55-59 yea	Genital her	Number	1995	11138.3	27276.5	3420.32
DALYs (Dis	Global	Both	55-59 yea	Genital her	Rate	1995	5.57512	13.6529	1.71199
DALYs (Dis	Global	Both	60-64 yea	Genital her	Number	1995	9063.8	22110.9	2774.54
DALYs (Dis	Global	Both	60-64 yea	Genital her	Rate	1995	5.26971	12.8553	1.61312
DALYs (Dis	Global	Both	65-69 yea	Genital her	Number	1995	7061.44	17238.8	2134.89
DALYs (Dis	Global	Both	65-69 yea	Genital her	Rate	1995	4.97057	12.1345	1.50276
DALYs (Dis	Global	Both	50-69 yea	Genital her	Number	1995	39985.7	98071.9	12287.8
DALYs (Dis	Global	Both	50-69 yea	Genital her	Rate	1995	5.45549	13.3806	1.6765
DALYs (Dis	Global	Both	50-54 yea	Other sexu	Number	1995	10227.2	12721.2	7881.36
DALYs (Dis	Global	Both	50-54 yea	Other sexu	Rate	1995	4.66796	5.80626	3.59725
DALYs (Dis	Global	Both	55-59 yea	Other sexu	Number	1995	9274.72	11344.3	7140.86
DALYs (Dis	Global	Both	55-59 yea	Other sexu	Rate	1995	4.64232	5.6782	3.57425
DALYs (Dis	Global	Both	60-64 yea	Other sexu	Number	1995	4157.07	5024.43	3178.35
DALYs (Dis	Global	Both	60-64 yea	Other sexu	Rate	1995	2.41693	2.92121	1.8479
DALYs (Dis	Global	Both	65-69 yea	Other sexu	Number	1995	2444.24	2915.28	1992.07
DALYs (Dis	Global	Both	65-69 yea	Other sexu	Rate	1995	1.72051	2.05208	1.40222
DALYs (Dis	Global	Both	50-69 yea	Other sexu	Number	1995	26103.2	30668.4	21197.5
DALYs (Dis	Global	Both	50-69 yea	Other sexu	Rate	1995	3.56143	4.18428	2.8921
DALYs (Dis	Global	Both	50-54 yea	HIV/AIDS	Number	1999	3206906	3728384	2663019
DALYs (Dis	Global	Both	50-54 yea	HIV/AIDS	Rate	1999	1271.56	1478.33	1055.91
DALYs (Dis	Global	Both	55-59 yea	HIV/AIDS	Number	1999	1671940	1916172	1416343
DALYs (Dis	Global	Both	55-59 yea	HIV/AIDS	Rate	1999	816.716	936.019	691.861
DALYs (Dis	Global	Both	60-64 yea	HIV/AIDS	Number	1999	898862	1029399	770072
DALYs (Dis	Global	Both	60-64 yea	HIV/AIDS	Rate	1999	488.152	559.044	418.209
DALYs (Dis	Global	Both	65-69 yea	HIV/AIDS	Number	1999	480629	552591	412409
DALYs (Dis	Global	Both	65-69 yea	HIV/AIDS	Rate	1999	318.181	365.82	273.018
DALYs (Dis	Global	Both	50-69 yea	HIV/AIDS	Number	1999	6258337	7226792	5277471
DALYs (Dis	Global	Both	50-69 yea	HIV/AIDS	Rate	1999	790.087	912.35	666.257
DALYs (Dis	Global	Both	50-54 yea	HIV/AIDS	Number	2010	3917032	4482712	3497091
DALYs (Dis	Global	Both	50-54 yea	HIV/AIDS	Rate	2010	1141	1305.78	1018.68
DALYs (Dis	Global	Both	55-59 yea	HIV/AIDS	Number	2010	1970677	2317331	1753146
DALYs (Dis	Global	Both	55-59 yea	HIV/AIDS	Rate	2010	649.529	763.785	577.831
DALYs (Dis	Global	Both	60-64 yea	HIV/AIDS	Number	2010	938189	1114951	830924
DALYs (Dis	Global	Both	60-64 yea	HIV/AIDS	Rate	2010	392.678	466.662	347.782
DALYs (Dis	Global	Both	65-69 yea	HIV/AIDS	Number	2010	459270	550028	404274
DALYs (Dis	Global	Both	65-69 yea	HIV/AIDS	Rate	2010	257.524	308.415	226.687
DALYs (Dis	Global	Both	50-69 yea	HIV/AIDS	Number	2010	7285168	8446136	6535622
DALYs (Dis	Global	Both	50-69 yea	HIV/AIDS	Rate	2010	684.722	793.84	614.274
DALYs (Dis	Global	Both	50-54 yea	Sexually tr	Number	2013	74655.5	128555	46617.6
DALYs (Dis	Global	Both	50-54 yea	Sexually tr	Rate	2013	20.1347	34.6714	12.5728
DALYs (Dis	Global	Both	55-59 yea	Sexually tr	Number	2013	60940.1	101108	39239.9
DALYs (Dis	Global	Both	55-59 yea	Sexually tr	Rate	2013	18.9011	31.3594	12.1706
DALYs (Dis	Global	Both	60-64 yea	Sexually tr	Number	2013	38860.1	68060.7	23735.5
DALYs (Dis	Global	Both	60-64 yea	Sexually tr	Rate	2013	14.3697	25.1676	8.77695
DALYs (Dis	Global	Both	65-69 yea	Sexually tr	Number	2013	27480.6	46398	17116.2
DALYs (Dis	Global	Both	65-69 yea	Sexually tr	Rate	2013	13.7504	23.216	8.56437
DALYs (Dis	Global	Both	50-69 yea	Sexually tr	Number	2013	201936	343911	127504
DALYs (Dis	Global	Both	50-69 yea	Sexually tr	Rate	2013	17.3562	29.5588	10.9588
DALYs (Dis	Global	Both	50-54 yea	Syphilis	Number	2013	7450.86	9678.84	5019.36
DALYs (Dis	Global	Both	50-54 yea	Syphilis	Rate	2013	2.00951	2.61039	1.35373
DALYs (Dis	Global	Both	55-59 yea	Syphilis	Number	2013	7619.63	10855.1	4401.22
DALYs (Dis	Global	Both	55-59 yea	Syphilis	Rate	2013	2.36329	3.36679	1.36507
DALYs (Dis	Global	Both	60-64 yea	Syphilis	Number	2013	5669.1	7473.24	4011.62
DALYs (Dis	Global	Both	60-64 yea	Syphilis	Rate	2013	2.09633	2.76347	1.48342
DALYs (Dis	Global	Both	65-69 yea	Syphilis	Number	2013	3553.15	4586.4	2920.07
DALYs (Dis	Global	Both	65-69 yea	Syphilis	Rate	2013	1.77788	2.29488	1.4611
DALYs (Dis	Global	Both	50-69 yea	Syphilis	Number	2013	24292.7	30486.5	16924.6
DALYs (Dis	Global	Both	50-69 yea	Syphilis	Rate	2013	2.08794	2.62029	1.45465
DALYs (Dis	Global	Both	50-54 yea	Chlamydia	Number	2013	5619.94	7606.26	4136.12
DALYs (Dis	Global	Both	50-54 yea	Chlamydia	Rate	2013	1.5157	2.05142	1.11552
DALYs (Dis	Global	Both	55-59 yea	Chlamydia	Number	2013	3935.13	4811.55	3135.54
DALYs (Dis	Global	Both	55-59 yea	Chlamydia	Rate	2013	1.22051	1.49234	0.97251
DALYs (Dis	Global	Both	60-64 yea	Chlamydia	Number	2013	1775.76	2079.07	1415.96
DALYs (Dis	Global	Both	60-64 yea	Chlamydia	Rate	2013	0.65664	0.7688	0.5236
DALYs (Dis	Global	Both	65-69 yea	Chlamydia	Number	2013	981.332	1161.92	770.031
DALYs (Dis	Global	Both	65-69 yea	Chlamydia	Rate	2013	0.49103	0.58138	0.3853
DALYs (Dis	Global	Both	50-69 yea	Chlamydia	Number	2013	12312.2	14986.4	10064.5
DALYs (Dis	Global	Both	50-69 yea	Chlamydia	Rate	2013	1.05822	1.28807	0.86504
DALYs (Dis	Global	Both	50-54 yea	Gonococca	Number	2013	9213.36	10776.2	7358.35
DALYs (Dis	Global	Both	50-54 yea	Gonococca	Rate	2013	2.48485	2.90636	1.98455
DALYs (Dis	Global	Both	55-59 yea	Gonococca	Number	2013	8779.31	10470.6	6650.06
DALYs (Dis	Global	Both	55-59 yea	Gonococca	Rate	2013	2.72297	3.24755	2.06257
DALYs (Dis	Global	Both	60-64 yea	Gonococca	Number	2013	4928.46	5708.13	4044.74
DALYs (Dis	Global	Both	60-64 yea	Gonococca	Rate	2013	1.82246	2.11076	1.49567
DALYs (Dis	Global	Both	65-69 yea	Gonococca	Number	2013	5431.6	6388.13	4299.69
DALYs (Dis	Global	Both	65-69 yea	Gonococca	Rate	2013	2.71779	3.19641	2.15142
DALYs (Dis	Global	Both	50-69 yea	Gonococca	Number	2013	28352.7	31878.2	22586.7
DALYs (Dis	Global	Both	50-69 yea	Gonococca	Rate	2013	2.43689	2.7399	1.94131
DALYs (Dis	Global	Both	50-54 yea	HIV/AIDS	Number	2014	3527762	4044399	3201840
DALYs (Dis	Global	Both	50-54 yea	HIV/AIDS	Rate	2014	915.761	1049.87	831.156
DALYs (Dis	Global	Both	55-59 yea	HIV/AIDS	Number	2014	1843626	2136346	1659655
DALYs (Dis	Global	Both	55-59 yea	HIV/AIDS	Rate	2014	565.285	655.038	508.877
DALYs (Dis	Global	Both	60-64 yea	HIV/AIDS	Number	2014	955685	1108867	861749
DALYs (Dis	Global	Both	60-64 yea	HIV/AIDS	Rate	2014	342.23	397.084	308.592
DALYs (Dis	Global	Both	65-69 yea	HIV/AIDS	Number	2014	463963	531504	419931
DALYs (Dis	Global	Both	65-69 yea	HIV/AIDS	Rate	2014	219.643	251.618	198.798
DALYs (Dis	Global	Both	50-69 yea	HIV/AIDS	Number	2014	6791036	7781388	6194576
DALYs (Dis	Global	Both	50-69 yea	HIV/AIDS	Rate	2014	565.046	647.448	515.418
DALYs (Dis	Global	Both	50-54 yea	Trichomon	Number	2013	18746.4	44071.9	6009.48
DALYs (Dis	Global	Both	50-54 yea	Trichomon	Rate	2013	5.05591	11.8862	1.62076
DALYs (Dis	Global	Both	55-59 yea	Trichomon	Number	2013	13058.9	30495.9	4263.13
DALYs (Dis	Global	Both	55-59 yea	Trichomon	Rate	2013	4.05032	9.45854	1.32224
DALYs (Dis	Global	Both	60-64 yea	Trichomon	Number	2013	8562.6	19947.8	2956.78
DALYs (Dis	Global	Both	60-64 yea	Trichomon	Rate	2013	3.16629	7.37633	1.09336
DALYs (Dis	Global	Both	65-69 yea	Trichomon	Number	2013	5467.9	12638.4	1939.95
DALYs (Dis	Global	Both	65-69 yea	Trichomon	Rate	2013	2.73596	6.32385	0.97069
DALYs (Dis	Global	Both	50-69 yea	Trichomon	Number	2013	45835.8	101497	16850.2
DALYs (Dis	Global	Both	50-69 yea	Trichomon	Rate	2013	3.93954	8.72353	1.44826
DALYs (Dis	Global	Both	50-54 yea	Genital her	Number	2013	21134.9	52568.9	6603.59
DALYs (Dis	Global	Both	50-54 yea	Genital her	Rate	2013	5.70012	14.1779	1.781
DALYs (Dis	Global	Both	55-59 yea	Genital her	Number	2013	17634.8	43568.4	5351.19
DALYs (Dis	Global	Both	55-59 yea	Genital her	Rate	2013	5.46959	13.5131	1.65971
DALYs (Dis	Global	Both	60-64 yea	Genital her	Number	2013	13845.4	33893.4	4208.73
DALYs (Dis	Global	Both	60-64 yea	Genital her	Rate	2013	5.11979	12.5332	1.55631
DALYs (Dis	Global	Both	65-69 yea	Genital her	Number	2013	9584.37	23351.9	2864.8
DALYs (Dis	Global	Both	65-69 yea	Genital her	Rate	2013	4.7957	11.6845	1.43345
DALYs (Dis	Global	Both	50-69 yea	Genital her	Number	2013	62199.6	153247	19042.7
DALYs (Dis	Global	Both	50-69 yea	Genital her	Rate	2013	5.34599	13.1714	1.6367
DALYs (Dis	Global	Both	50-54 yea	Other sexu	Number	2013	12490.1	15952.7	9558.93
DALYs (Dis	Global	Both	50-54 yea	Other sexu	Rate	2013	3.36859	4.30246	2.57805
DALYs (Dis	Global	Both	55-59 yea	Other sexu	Number	2013	9912.32	11920	7834.3
DALYs (Dis	Global	Both	55-59 yea	Other sexu	Rate	2013	3.07438	3.69708	2.42987
DALYs (Dis	Global	Both	60-64 yea	Other sexu	Number	2013	4078.7	4766.62	3244.66
DALYs (Dis	Global	Both	60-64 yea	Other sexu	Rate	2013	1.50823	1.76261	1.19982
DALYs (Dis	Global	Both	65-69 yea	Other sexu	Number	2013	2462.24	2922.34	1975.35
DALYs (Dis	Global	Both	65-69 yea	Other sexu	Rate	2013	1.23202	1.46224	0.9884
DALYs (Dis	Global	Both	50-69 yea	Other sexu	Number	2013	28943.4	34375.3	23654.7
DALYs (Dis	Global	Both	50-69 yea	Other sexu	Rate	2013	2.48765	2.95452	2.0331
DALYs (Dis	Global	Both	50-54 yea	Sexually tr	Number	2005	67067.3	110554	43066.8
DALYs (Dis	Global	Both	50-54 yea	Sexually tr	Rate	2005	21.1743	34.904	13.5969
DALYs (Dis	Global	Both	55-59 yea	Sexually tr	Number	2005	51754.3	82359.5	34320.7
DALYs (Dis	Global	Both	55-59 yea	Sexually tr	Rate	2005	20.7362	32.9986	13.7511
DALYs (Dis	Global	Both	60-64 yea	Sexually tr	Number	2005	31487.1	52513.3	20399
DALYs (Dis	Global	Both	60-64 yea	Sexually tr	Rate	2005	16.1463	26.9284	10.4605
DALYs (Dis	Global	Both	65-69 yea	Sexually tr	Number	2005	25314	41595.9	16688.6
DALYs (Dis	Global	Both	65-69 yea	Sexually tr	Rate	2005	15.0154	24.6733	9.89915
DALYs (Dis	Global	Both	50-69 yea	Sexually tr	Number	2005	175623	290491	115935
DALYs (Dis	Global	Both	50-69 yea	Sexually tr	Rate	2005	18.8858	31.2382	12.4672
DALYs (Dis	Global	Both	50-54 yea	Syphilis	Number	2005	7969.81	10750	5151.02
DALYs (Dis	Global	Both	50-54 yea	Syphilis	Rate	2005	2.51621	3.39395	1.62627
DALYs (Dis	Global	Both	55-59 yea	Syphilis	Number	2005	7695.67	11653.3	4235.82
DALYs (Dis	Global	Both	55-59 yea	Syphilis	Rate	2005	3.08339	4.6691	1.69715
DALYs (Dis	Global	Both	60-64 yea	Syphilis	Number	2005	5520.79	7576.54	3722.91
DALYs (Dis	Global	Both	60-64 yea	Syphilis	Rate	2005	2.83102	3.88519	1.90908
DALYs (Dis	Global	Both	65-69 yea	Syphilis	Number	2005	3668.22	4921.6	2947.41
DALYs (Dis	Global	Both	65-69 yea	Syphilis	Rate	2005	2.17587	2.91933	1.74831
DALYs (Dis	Global	Both	50-69 yea	Syphilis	Number	2005	24854.5	32814.6	16712
DALYs (Dis	Global	Both	50-69 yea	Syphilis	Rate	2005	2.67275	3.52875	1.79714
DALYs (Dis	Global	Both	50-54 yea	Chlamydia	Number	2005	5121.72	6889.75	3787.64
DALYs (Dis	Global	Both	50-54 yea	Chlamydia	Rate	2005	1.61701	2.17522	1.19582
DALYs (Dis	Global	Both	55-59 yea	Chlamydia	Number	2005	3471.68	4164.93	2767.82
DALYs (Dis	Global	Both	55-59 yea	Chlamydia	Rate	2005	1.39098	1.66874	1.10897
DALYs (Dis	Global	Both	60-64 yea	Chlamydia	Number	2005	1545.52	1805.74	1244.38
DALYs (Dis	Global	Both	60-64 yea	Chlamydia	Rate	2005	0.79253	0.92597	0.63811
DALYs (Dis	Global	Both	65-69 yea	Chlamydia	Number	2005	961.895	1149	759.676
DALYs (Dis	Global	Both	65-69 yea	Chlamydia	Rate	2005	0.57056	0.68155	0.45061
DALYs (Dis	Global	Both	50-69 yea	Chlamydia	Number	2005	11100.8	13478.2	9103.19
DALYs (Dis	Global	Both	50-69 yea	Chlamydia	Rate	2005	1.19374	1.44939	0.97892
DALYs (Dis	Global	Both	50-54 yea	Gonococca	Number	2005	8535.88	9946.2	7117.8
DALYs (Dis	Global	Both	50-54 yea	Gonococca	Rate	2005	2.69492	3.14019	2.24721
DALYs (Dis	Global	Both	55-59 yea	Gonococca	Number	2005	8013.11	9574.98	6116.64
DALYs (Dis	Global	Both	55-59 yea	Gonococca	Rate	2005	3.21058	3.83637	2.45073
DALYs (Dis	Global	Both	60-64 yea	Gonococca	Number	2005	4378.71	5121.09	3579.01
DALYs (Dis	Global	Both	60-64 yea	Gonococca	Rate	2005	2.24537	2.62605	1.83529
DALYs (Dis	Global	Both	65-69 yea	Gonococca	Number	2005	5392.79	6397.54	4358.45
DALYs (Dis	Global	Both	65-69 yea	Gonococca	Rate	2005	3.19882	3.7948	2.58529
DALYs (Dis	Global	Both	50-69 yea	Gonococca	Number	2005	26320.5	29755.5	21054.2
DALYs (Dis	Global	Both	50-69 yea	Gonococca	Rate	2005	2.8304	3.19979	2.26409
DALYs (Dis	Global	Both	50-54 yea	Trichomon	Number	2005	15818.7	37523.8	5077.85
DALYs (Dis	Global	Both	50-54 yea	Trichomon	Rate	2005	4.99425	11.8469	1.60316
DALYs (Dis	Global	Both	55-59 yea	Trichomon	Number	2005	9987.88	23177.9	3304.2
DALYs (Dis	Global	Both	55-59 yea	Trichomon	Rate	2005	4.0018	9.28659	1.32388
DALYs (Dis	Global	Both	60-64 yea	Trichomon	Number	2005	6201.99	14526	2128.64
DALYs (Dis	Global	Both	60-64 yea	Trichomon	Rate	2005	3.18033	7.44883	1.09155
DALYs (Dis	Global	Both	65-69 yea	Trichomon	Number	2005	4641.93	10729.8	1633.68
DALYs (Dis	Global	Both	65-69 yea	Trichomon	Rate	2005	2.75344	6.36456	0.96904
DALYs (Dis	Global	Both	50-69 yea	Trichomon	Number	2005	36650.5	80723.5	13415.2
DALYs (Dis	Global	Both	50-69 yea	Trichomon	Rate	2005	3.94125	8.68069	1.44262
DALYs (Dis	Global	Both	50-54 yea	Genital her	Number	2005	18008.9	44768.6	5532.38
DALYs (Dis	Global	Both	50-54 yea	Genital her	Rate	2005	5.68574	14.1342	1.74667
DALYs (Dis	Global	Both	55-59 yea	Genital her	Number	2005	13693.6	33829	4180.76
DALYs (Dis	Global	Both	55-59 yea	Genital her	Rate	2005	5.48654	13.5541	1.67509
DALYs (Dis	Global	Both	60-64 yea	Genital her	Number	2005	10180.2	24689.9	3089.35
DALYs (Dis	Global	Both	60-64 yea	Genital her	Rate	2005	5.22033	12.6608	1.58419
DALYs (Dis	Global	Both	65-69 yea	Genital her	Number	2005	8209.57	20007.3	2477.32
DALYs (Dis	Global	Both	65-69 yea	Genital her	Rate	2005	4.86964	11.8676	1.46946
DALYs (Dis	Global	Both	50-69 yea	Genital her	Number	2005	50092.3	123246	15326
DALYs (Dis	Global	Both	50-69 yea	Genital her	Rate	2005	5.38673	13.2534	1.6481
DALYs (Dis	Global	Both	50-54 yea	Other sexu	Number	2005	11612.2	14828	8880.22
DALYs (Dis	Global	Both	50-54 yea	Other sexu	Rate	2005	3.66619	4.68146	2.80364
DALYs (Dis	Global	Both	55-59 yea	Other sexu	Number	2005	8892.44	10715.5	6973.86
DALYs (Dis	Global	Both	55-59 yea	Other sexu	Rate	2005	3.56289	4.29334	2.79419
DALYs (Dis	Global	Both	60-64 yea	Other sexu	Number	2005	3659.83	4346.8	2932.29
DALYs (Dis	Global	Both	60-64 yea	Other sexu	Rate	2005	1.87673	2.229	1.50366
DALYs (Dis	Global	Both	65-69 yea	Other sexu	Number	2005	2439.59	2906.25	1998.37
DALYs (Dis	Global	Both	65-69 yea	Other sexu	Rate	2005	1.44708	1.72389	1.18536
DALYs (Dis	Global	Both	50-69 yea	Other sexu	Number	2005	26604.1	31513.6	21567
DALYs (Dis	Global	Both	50-69 yea	Other sexu	Rate	2005	2.8609	3.38885	2.31923
DALYs (Dis	Global	Both	50-54 yea	HIV/AIDS	Number	1996	2262860	2765261	1763914
DALYs (Dis	Global	Both	50-54 yea	HIV/AIDS	Rate	1996	1017.67	1243.62	793.284
DALYs (Dis	Global	Both	55-59 yea	HIV/AIDS	Number	1996	1221039	1471571	967867
DALYs (Dis	Global	Both	55-59 yea	HIV/AIDS	Rate	1996	605.295	729.49	479.793
DALYs (Dis	Global	Both	60-64 yea	HIV/AIDS	Number	1996	662398	799576	531724
DALYs (Dis	Global	Both	60-64 yea	HIV/AIDS	Rate	1996	379.436	458.014	304.583
DALYs (Dis	Global	Both	65-69 yea	HIV/AIDS	Number	1996	357976	436091	286076
DALYs (Dis	Global	Both	65-69 yea	HIV/AIDS	Rate	1996	246.736	300.577	197.178
DALYs (Dis	Global	Both	50-69 yea	HIV/AIDS	Number	1996	4504272	5451823	3552072
DALYs (Dis	Global	Both	50-69 yea	HIV/AIDS	Rate	1996	605.624	733.027	477.595
DALYs (Dis	Global	Both	50-54 yea	HIV/AIDS	Number	2001	3932161	4417151	3416868
DALYs (Dis	Global	Both	50-54 yea	HIV/AIDS	Rate	2001	1427.98	1604.11	1240.85
DALYs (Dis	Global	Both	55-59 yea	HIV/AIDS	Number	2001	1955549	2164408	1736538
DALYs (Dis	Global	Both	55-59 yea	HIV/AIDS	Rate	2001	934.202	1033.98	829.576
DALYs (Dis	Global	Both	60-64 yea	HIV/AIDS	Number	2001	1037469	1145089	930251
DALYs (Dis	Global	Both	60-64 yea	HIV/AIDS	Rate	2001	548.545	605.448	491.856
DALYs (Dis	Global	Both	65-69 yea	HIV/AIDS	Number	2001	548530	608353	491449
DALYs (Dis	Global	Both	65-69 yea	HIV/AIDS	Rate	2001	351.825	390.195	315.213
DALYs (Dis	Global	Both	50-69 yea	HIV/AIDS	Number	2001	7473710	8318899	6613016
DALYs (Dis	Global	Both	50-69 yea	HIV/AIDS	Rate	2001	900.736	1002.6	797.004
DALYs (Dis	Global	Both	50-54 yea	Sexually tr	Number	1996	56962.7	90021.1	38884.2
DALYs (Dis	Global	Both	50-54 yea	Sexually tr	Rate	1996	25.6178	40.4851	17.4874
DALYs (Dis	Global	Both	55-59 yea	Sexually tr	Number	1996	52224.5	77916.1	36100.6
DALYs (Dis	Global	Both	55-59 yea	Sexually tr	Rate	1996	25.8888	38.6247	17.8958
DALYs (Dis	Global	Both	60-64 yea	Sexually tr	Number	1996	33859.7	52819.1	22930.4
DALYs (Dis	Global	Both	60-64 yea	Sexually tr	Rate	1996	19.3956	30.2559	13.1351
DALYs (Dis	Global	Both	65-69 yea	Sexually tr	Number	1996	25634.3	39814.5	17865.5
DALYs (Dis	Global	Both	65-69 yea	Sexually tr	Rate	1996	17.6685	27.4423	12.3138
DALYs (Dis	Global	Both	50-69 yea	Sexually tr	Number	1996	168681	260709	118603
DALYs (Dis	Global	Both	50-69 yea	Sexually tr	Rate	1996	22.6801	35.0537	15.9468
DALYs (Dis	Global	Both	50-54 yea	Syphilis	Number	1996	9581.12	13461.2	5676.16
DALYs (Dis	Global	Both	50-54 yea	Syphilis	Rate	1996	4.30891	6.05392	2.55274
DALYs (Dis	Global	Both	55-59 yea	Syphilis	Number	1996	11367	17145	6205.43
DALYs (Dis	Global	Both	55-59 yea	Syphilis	Rate	1996	5.63488	8.49913	3.07616
DALYs (Dis	Global	Both	60-64 yea	Syphilis	Number	1996	8240.11	10969.9	5438.42
DALYs (Dis	Global	Both	60-64 yea	Syphilis	Rate	1996	4.72012	6.28378	3.11525
DALYs (Dis	Global	Both	65-69 yea	Syphilis	Number	1996	5683.51	7069.28	4354.46
DALYs (Dis	Global	Both	65-69 yea	Syphilis	Rate	1996	3.91737	4.87252	3.00132
DALYs (Dis	Global	Both	50-69 yea	Syphilis	Number	1996	34871.8	46403.1	22037.4
DALYs (Dis	Global	Both	50-69 yea	Syphilis	Rate	1996	4.6887	6.23915	2.96305
DALYs (Dis	Global	Both	50-54 yea	Chlamydia	Number	1996	4288.78	5596.2	3239.32
DALYs (Dis	Global	Both	50-54 yea	Chlamydia	Rate	1996	1.92879	2.51677	1.45682
DALYs (Dis	Global	Both	55-59 yea	Chlamydia	Number	1996	3530.95	4330.74	2728
DALYs (Dis	Global	Both	55-59 yea	Chlamydia	Rate	1996	1.75037	2.14684	1.35233
DALYs (Dis	Global	Both	60-64 yea	Chlamydia	Number	1996	1721.56	2080.7	1271.19
DALYs (Dis	Global	Both	60-64 yea	Chlamydia	Rate	1996	0.98615	1.19187	0.72816
DALYs (Dis	Global	Both	65-69 yea	Chlamydia	Number	1996	938.622	1113.3	762.663
DALYs (Dis	Global	Both	65-69 yea	Chlamydia	Rate	1996	0.64695	0.76734	0.52567
DALYs (Dis	Global	Both	50-69 yea	Chlamydia	Number	1996	10479.9	12486	8488.82
DALYs (Dis	Global	Both	50-69 yea	Chlamydia	Rate	1996	1.40908	1.67881	1.14137
DALYs (Dis	Global	Both	50-54 yea	Gonococca	Number	1996	8395.37	9923.76	6341.52
DALYs (Dis	Global	Both	50-54 yea	Gonococca	Rate	1996	3.77565	4.46301	2.85197
DALYs (Dis	Global	Both	55-59 yea	Gonococca	Number	1996	8619.62	10466.5	6220.57
DALYs (Dis	Global	Both	55-59 yea	Gonococca	Rate	1996	4.27294	5.18846	3.08367
DALYs (Dis	Global	Both	60-64 yea	Gonococca	Number	1996	4997.24	5939.91	3860.84
DALYs (Dis	Global	Both	60-64 yea	Gonococca	Rate	1996	2.86253	3.40251	2.21157
DALYs (Dis	Global	Both	65-69 yea	Gonococca	Number	1996	5362.75	6334.25	4406.85
DALYs (Dis	Global	Both	65-69 yea	Gonococca	Rate	1996	3.69629	4.3659	3.03743
DALYs (Dis	Global	Both	50-69 yea	Gonococca	Number	1996	27375	31143.5	20605.6
DALYs (Dis	Global	Both	50-69 yea	Gonococca	Rate	1996	3.68071	4.18741	2.77053
DALYs (Dis	Global	Both	50-54 yea	Sexually tr	Number	1999	59358.5	95435.1	39749.2
DALYs (Dis	Global	Both	50-54 yea	Sexually tr	Rate	1999	23.5361	37.8408	15.7609
DALYs (Dis	Global	Both	55-59 yea	Sexually tr	Number	1999	50102.7	75556.6	34680.3
DALYs (Dis	Global	Both	55-59 yea	Sexually tr	Rate	1999	24.4744	36.9082	16.9408
DALYs (Dis	Global	Both	60-64 yea	Sexually tr	Number	1999	34137	54221.4	22763.6
DALYs (Dis	Global	Both	60-64 yea	Sexually tr	Rate	1999	18.5391	29.4464	12.3624
DALYs (Dis	Global	Both	65-69 yea	Sexually tr	Number	1999	25337.5	39727.4	17385.2
DALYs (Dis	Global	Both	65-69 yea	Sexually tr	Rate	1999	16.7736	26.2999	11.5091
DALYs (Dis	Global	Both	50-69 yea	Sexually tr	Number	1999	168936	268313	116622
DALYs (Dis	Global	Both	50-69 yea	Sexually tr	Rate	1999	21.3274	33.8733	14.723
DALYs (Dis	Global	Both	50-54 yea	Syphilis	Number	1999	9249.25	12830.7	5741.35
DALYs (Dis	Global	Both	50-54 yea	Syphilis	Rate	1999	3.6674	5.08747	2.27649
DALYs (Dis	Global	Both	55-59 yea	Syphilis	Number	1999	9868.25	15531.8	5283.74
DALYs (Dis	Global	Both	55-59 yea	Syphilis	Rate	1999	4.82048	7.58703	2.58102
DALYs (Dis	Global	Both	60-64 yea	Syphilis	Number	1999	8052.31	10934.2	5335.36
DALYs (Dis	Global	Both	60-64 yea	Syphilis	Rate	1999	4.37303	5.93812	2.89752
DALYs (Dis	Global	Both	65-69 yea	Syphilis	Number	1999	4991.78	6434.91	3946.18
DALYs (Dis	Global	Both	65-69 yea	Syphilis	Rate	1999	3.3046	4.25997	2.6124
DALYs (Dis	Global	Both	50-69 yea	Syphilis	Number	1999	32161.6	42828.6	20922.8
DALYs (Dis	Global	Both	50-69 yea	Syphilis	Rate	1999	4.06026	5.40691	2.64141
DALYs (Dis	Global	Both	50-54 yea	Chlamydia	Number	1999	4475.28	5986.67	3355.4
DALYs (Dis	Global	Both	50-54 yea	Chlamydia	Rate	1999	1.77448	2.37376	1.33044
DALYs (Dis	Global	Both	55-59 yea	Chlamydia	Number	1999	3432.71	4247.7	2680.43
DALYs (Dis	Global	Both	55-59 yea	Chlamydia	Rate	1999	1.67682	2.07493	1.30935
DALYs (Dis	Global	Both	60-64 yea	Chlamydia	Number	1999	1724.28	2069.04	1334.61
DALYs (Dis	Global	Both	60-64 yea	Chlamydia	Rate	1999	0.93642	1.12365	0.7248
DALYs (Dis	Global	Both	65-69 yea	Chlamydia	Number	1999	965.28	1148.4	762.063
DALYs (Dis	Global	Both	65-69 yea	Chlamydia	Rate	1999	0.63902	0.76025	0.50449
DALYs (Dis	Global	Both	50-69 yea	Chlamydia	Number	1999	10597.5	12664.7	8689.04
DALYs (Dis	Global	Both	50-69 yea	Chlamydia	Rate	1999	1.33789	1.59886	1.09695
DALYs (Dis	Global	Both	50-54 yea	Gonococca	Number	1999	8158.2	9682.27	6467.87
DALYs (Dis	Global	Both	50-54 yea	Gonococca	Rate	1999	3.23479	3.8391	2.56456
DALYs (Dis	Global	Both	55-59 yea	Gonococca	Number	1999	8310.22	10205.7	6118.14
DALYs (Dis	Global	Both	55-59 yea	Gonococca	Rate	1999	4.05941	4.98534	2.98862
DALYs (Dis	Global	Both	60-64 yea	Gonococca	Number	1999	4978.72	5896.56	3930.11
DALYs (Dis	Global	Both	60-64 yea	Gonococca	Rate	1999	2.70383	3.20229	2.13435
DALYs (Dis	Global	Both	65-69 yea	Gonococca	Number	1999	5451.06	6434.25	4343.11
DALYs (Dis	Global	Both	65-69 yea	Gonococca	Rate	1999	3.60865	4.25953	2.87518
DALYs (Dis	Global	Both	50-69 yea	Gonococca	Number	1999	26898.2	30592	20910.8
DALYs (Dis	Global	Both	50-69 yea	Gonococca	Rate	1999	3.39577	3.8621	2.6399
DALYs (Dis	Global	Both	50-54 yea	Trichomon	Number	1999	12699.8	30129.2	4025.02
DALYs (Dis	Global	Both	50-54 yea	Trichomon	Rate	1999	5.03557	11.9465	1.59595
DALYs (Dis	Global	Both	55-59 yea	Trichomon	Number	1999	8290.81	19358.5	2723.57
DALYs (Dis	Global	Both	55-59 yea	Trichomon	Rate	1999	4.04993	9.45632	1.33042
DALYs (Dis	Global	Both	60-64 yea	Trichomon	Number	1999	5807.82	13581	1985.01
DALYs (Dis	Global	Both	60-64 yea	Trichomon	Rate	1999	3.1541	7.37553	1.07802
DALYs (Dis	Global	Both	65-69 yea	Trichomon	Number	1999	4145.56	9627.81	1474.67
DALYs (Dis	Global	Both	65-69 yea	Trichomon	Rate	1999	2.74439	6.3737	0.97624
DALYs (Dis	Global	Both	50-69 yea	Trichomon	Number	1999	30944	68671.5	11348.1
DALYs (Dis	Global	Both	50-69 yea	Trichomon	Rate	1999	3.90654	8.66946	1.43265
DALYs (Dis	Global	Both	50-54 yea	Genital her	Number	1999	14281.2	35489.2	4432.92
DALYs (Dis	Global	Both	50-54 yea	Genital her	Rate	1999	5.6626	14.0717	1.75769
DALYs (Dis	Global	Both	55-59 yea	Genital her	Number	1999	11258.8	27870.8	3444.8
DALYs (Dis	Global	Both	55-59 yea	Genital her	Rate	1999	5.49973	13.6145	1.68273
DALYs (Dis	Global	Both	60-64 yea	Genital her	Number	1999	9524.99	23198.1	2911.68
DALYs (Dis	Global	Both	60-64 yea	Genital her	Rate	1999	5.17281	12.5984	1.58127
DALYs (Dis	Global	Both	65-69 yea	Genital her	Number	1999	7316.9	17856.8	2222.49
DALYs (Dis	Global	Both	65-69 yea	Genital her	Rate	1999	4.84385	11.8213	1.47131
DALYs (Dis	Global	Both	50-69 yea	Genital her	Number	1999	42381.8	104421	13022.9
DALYs (Dis	Global	Both	50-69 yea	Genital her	Rate	1999	5.35051	13.1827	1.64408
DALYs (Dis	Global	Both	50-54 yea	Other sexu	Number	1999	10494.8	13312	8104.96
DALYs (Dis	Global	Both	50-54 yea	Other sexu	Rate	1999	4.16126	5.2783	3.21368
DALYs (Dis	Global	Both	55-59 yea	Other sexu	Number	1999	8941.96	10894.9	6928.62
DALYs (Dis	Global	Both	55-59 yea	Other sexu	Rate	1999	4.368	5.32196	3.38452
DALYs (Dis	Global	Both	60-64 yea	Other sexu	Number	1999	4048.9	4922.31	3174.16
DALYs (Dis	Global	Both	60-64 yea	Other sexu	Rate	1999	2.19887	2.6732	1.72382
DALYs (Dis	Global	Both	65-69 yea	Other sexu	Number	1999	2466.89	2926.31	1979.98
DALYs (Dis	Global	Both	65-69 yea	Other sexu	Rate	1999	1.6331	1.93724	1.31076
DALYs (Dis	Global	Both	50-69 yea	Other sexu	Number	1999	25952.5	30372	21017.4
DALYs (Dis	Global	Both	50-69 yea	Other sexu	Rate	1999	3.27639	3.83433	2.65335
DALYs (Dis	Global	Both	50-54 yea	Trichomon	Number	1996	11503.5	27272.2	3686.94
DALYs (Dis	Global	Both	50-54 yea	Trichomon	Rate	1996	5.17345	12.2651	1.65813
DALYs (Dis	Global	Both	55-59 yea	Trichomon	Number	1996	8231.92	19147	2706.01
DALYs (Dis	Global	Both	55-59 yea	Trichomon	Rate	1996	4.08074	9.49158	1.34143
DALYs (Dis	Global	Both	60-64 yea	Trichomon	Number	1996	5619.32	13207.9	1932.59
DALYs (Dis	Global	Both	60-64 yea	Trichomon	Rate	1996	3.21887	7.56579	1.10703
DALYs (Dis	Global	Both	65-69 yea	Trichomon	Number	1996	4043.09	9402.42	1442.08
DALYs (Dis	Global	Both	65-69 yea	Trichomon	Rate	1996	2.78671	6.48064	0.99396
DALYs (Dis	Global	Both	50-69 yea	Trichomon	Number	1996	29397.8	65989.9	10684.4
DALYs (Dis	Global	Both	50-69 yea	Trichomon	Rate	1996	3.95269	8.87269	1.43657
DALYs (Dis	Global	Both	50-54 yea	Genital her	Number	1996	12908	32095.2	4012.32
DALYs (Dis	Global	Both	50-54 yea	Genital her	Rate	1996	5.80513	14.4342	1.80446
DALYs (Dis	Global	Both	55-59 yea	Genital her	Number	1996	11240.3	27650.8	3420.69
DALYs (Dis	Global	Both	55-59 yea	Genital her	Rate	1996	5.57207	13.7071	1.69571
DALYs (Dis	Global	Both	60-64 yea	Genital her	Number	1996	9181.92	22307	2801.38
DALYs (Dis	Global	Both	60-64 yea	Genital her	Rate	1996	5.2596	12.778	1.60469
DALYs (Dis	Global	Both	65-69 yea	Genital her	Number	1996	7184.1	17503.6	2184.91
DALYs (Dis	Global	Both	65-69 yea	Genital her	Rate	1996	4.95166	12.0644	1.50596
DALYs (Dis	Global	Both	50-69 yea	Genital her	Number	1996	40514.4	99339.2	12369
DALYs (Dis	Global	Both	50-69 yea	Genital her	Rate	1996	5.44738	13.3567	1.66307
DALYs (Dis	Global	Both	50-54 yea	Other sexu	Number	1996	10285.9	12927.4	7961.65
DALYs (Dis	Global	Both	50-54 yea	Other sexu	Rate	1996	4.62588	5.81381	3.58059
DALYs (Dis	Global	Both	55-59 yea	Other sexu	Number	1996	9234.69	11304.9	7035.51
DALYs (Dis	Global	Both	55-59 yea	Other sexu	Rate	1996	4.57784	5.60409	3.48766
DALYs (Dis	Global	Both	60-64 yea	Other sexu	Number	1996	4099.58	4976.83	3020.68
DALYs (Dis	Global	Both	60-64 yea	Other sexu	Rate	1996	2.34833	2.85084	1.73031
DALYs (Dis	Global	Both	65-69 yea	Other sexu	Number	1996	2422.23	2902.05	1979.63
DALYs (Dis	Global	Both	65-69 yea	Other sexu	Rate	1996	1.66953	2.00024	1.36446
DALYs (Dis	Global	Both	50-69 yea	Other sexu	Number	1996	26042.4	30443	20982.4
DALYs (Dis	Global	Both	50-69 yea	Other sexu	Rate	1996	3.50154	4.09323	2.82119
DALYs (Dis	Global	Both	50-54 yea	HIV/AIDS	Number	2018	3362456	3908672	2998095
DALYs (Dis	Global	Both	50-54 yea	HIV/AIDS	Rate	2018	780.928	907.786	696.306
DALYs (Dis	Global	Both	55-59 yea	HIV/AIDS	Number	2018	1860822	2171154	1652172
DALYs (Dis	Global	Both	55-59 yea	HIV/AIDS	Rate	2018	521.4	608.354	462.936
DALYs (Dis	Global	Both	60-64 yea	HIV/AIDS	Number	2018	1004932	1178163	886999
DALYs (Dis	Global	Both	60-64 yea	HIV/AIDS	Rate	2018	325.165	381.218	287.006
DALYs (Dis	Global	Both	65-69 yea	HIV/AIDS	Number	2018	531480	618477	470639
DALYs (Dis	Global	Both	65-69 yea	HIV/AIDS	Rate	2018	212.342	247.1	188.034
DALYs (Dis	Global	Both	50-69 yea	HIV/AIDS	Number	2018	6759690	7852626	6031507
DALYs (Dis	Global	Both	50-69 yea	HIV/AIDS	Rate	2018	501.904	583.055	447.837
DALYs (Dis	Global	Both	50-54 yea	Sexually tr	Number	2017	85990.5	149059	53042
DALYs (Dis	Global	Both	50-54 yea	Sexually tr	Rate	2017	20.3504	35.276	12.5529
DALYs (Dis	Global	Both	55-59 yea	Sexually tr	Number	2017	66213.8	110144	42888.4
DALYs (Dis	Global	Both	55-59 yea	Sexually tr	Rate	2017	19.2108	31.9562	12.4433

DALYs (Dis	Global	Both	50-69 yea	Gonococca	Rate	2017	2.45838	2.83697	1.88758
DALYs (Dis	Global	Both	50-54 yea	Trichomon	Number	2017	21705.3	50404.1	6843.63
DALYs (Dis	Global	Both	50-54 yea	Trichomon	Rate	2017	5.13676	11.9286	1.61961
DALYs (Dis	Global	Both	55-59 yea	Trichomon	Number	2017	14134	33294.9	4587.63
DALYs (Dis	Global	Both	55-59 yea	Trichomon	Rate	2017	4.10073	9.65993	1.33102
DALYs (Dis	Global	Both	60-64 yea	Trichomon	Number	2017	9630.97	22382.5	3331.62
DALYs (Dis	Global	Both	60-64 yea	Trichomon	Rate	2017	3.16269	7.35012	1.09406
DALYs (Dis	Global	Both	65-69 yea	Trichomon	Number	2017	6572.1	15231.3	2346.86
DALYs (Dis	Global	Both	65-69 yea	Trichomon	Rate	2017	2.72555	6.31665	0.97328
DALYs (Dis	Global	Both	50-69 yea	Trichomon	Number	2017	52042.4	116378	19147.7
DALYs (Dis	Global	Both	50-69 yea	Trichomon	Rate	2017	3.96403	8.86439	1.45846
DALYs (Dis	Global	Both	50-54 yea	Genital her	Number	2017	24113.5	60270.9	7491.5
DALYs (Dis	Global	Both	50-54 yea	Genital her	Rate	2017	5.70667	14.2636	1.77293
DALYs (Dis	Global	Both	55-59 yea	Genital her	Number	2017	19233.3	47534.6	5870.23
DALYs (Dis	Global	Both	55-59 yea	Genital her	Rate	2017	5.58019	13.7913	1.70314
DALYs (Dis	Global	Both	60-64 yea	Genital her	Number	2017	15875.9	39151.8	4795.94
DALYs (Dis	Global	Both	60-64 yea	Genital her	Rate	2017	5.21346	12.857	1.57493
DALYs (Dis	Global	Both	65-69 yea	Genital her	Number	2017	11652.6	28278.4	3486.25
DALYs (Dis	Global	Both	65-69 yea	Genital her	Rate	2017	4.83253	11.7275	1.4458
DALYs (Dis	Global	Both	50-69 yea	Genital her	Number	2017	70875.4	175054	21638.8
DALYs (Dis	Global	Both	50-69 yea	Genital her	Rate	2017	5.39852	13.3337	1.64821
DALYs (Dis	Global	Both	50-54 yea	Other sexu	Number	2017	14617.5	19131.7	11053.4
DALYs (Dis	Global	Both	50-54 yea	Other sexu	Rate	2017	3.45936	4.52769	2.61588
DALYs (Dis	Global	Both	55-59 yea	Other sexu	Number	2017	10917.1	13357.9	8420.15
DALYs (Dis	Global	Both	55-59 yea	Other sexu	Rate	2017	3.16741	3.87555	2.44296
DALYs (Dis	Global	Both	60-64 yea	Other sexu	Number	2017	4463.19	5330.22	3419.92
DALYs (Dis	Global	Both	60-64 yea	Other sexu	Rate	2017	1.46566	1.75038	1.12306
DALYs (Dis	Global	Both	65-69 yea	Other sexu	Number	2017	2745.14	3300.15	2188.62
DALYs (Dis	Global	Both	65-69 yea	Other sexu	Rate	2017	1.13845	1.36863	0.90765
DALYs (Dis	Global	Both	50-69 yea	Other sexu	Number	2017	32742.9	39563.7	26156.2
DALYs (Dis	Global	Both	50-69 yea	Other sexu	Rate	2017	2.494	3.01354	1.99229
DALYs (Dis	Global	Both	50-54 yea	Sexually tr	Number	2002	63521.3	103646	41945.7
DALYs (Dis	Global	Both	50-54 yea	Sexually tr	Rate	2002	22.2991	36.3848	14.725
DALYs (Dis	Global	Both	55-59 yea	Sexually tr	Number	2002	49523.9	76563.7	33681.2
DALYs (Dis	Global	Both	55-59 yea	Sexually tr	Rate	2002	22.7376	35.1522	15.4638
DALYs (Dis	Global	Both	60-64 yea	Sexually tr	Number	2002	32935.9	54083.5	21620.8
DALYs (Dis	Global	Both	60-64 yea	Sexually tr	Rate	2002	17.2843	28.3822	11.3463
DALYs (Dis	Global	Both	65-69 yea	Sexually tr	Number	2002	25677.5	40937.8	17398.6
DALYs (Dis	Global	Both	65-69 yea	Sexually tr	Rate	2002	16.1169	25.6953	10.9205
DALYs (Dis	Global	Both	50-69 yea	Sexually tr	Number	2002	171659	279487	115927
DALYs (Dis	Global	Both	50-69 yea	Sexually tr	Rate	2002	20.135	32.7828	13.5979
DALYs (Dis	Global	Both	50-54 yea	Syphilis	Number	2002	8835.68	12098.8	5589.47
DALYs (Dis	Global	Both	50-54 yea	Syphilis	Rate	2002	3.10176	4.24729	1.96218
DALYs (Dis	Global	Both	55-59 yea	Syphilis	Number	2002	8551.81	13466.8	4642.16
DALYs (Dis	Global	Both	55-59 yea	Syphilis	Rate	2002	3.92634	6.18294	2.13133
DALYs (Dis	Global	Both	60-64 yea	Syphilis	Number	2002	6745.89	9307.01	4581.79
DALYs (Dis	Global	Both	60-64 yea	Syphilis	Rate	2002	3.54015	4.88418	2.40446
DALYs (Dis	Global	Both	65-69 yea	Syphilis	Number	2002	4360.54	5663.47	3555.56
DALYs (Dis	Global	Both	65-69 yea	Syphilis	Rate	2002	2.73697	3.55477	2.23171
DALYs (Dis	Global	Both	50-69 yea	Syphilis	Number	2002	28493.9	38781.7	18920.8
DALYs (Dis	Global	Both	50-69 yea	Syphilis	Rate	2002	3.34224	4.54896	2.21934
DALYs (Dis	Global	Both	50-54 yea	Chlamydia	Number	2002	4810.21	6439.76	3594.54
DALYs (Dis	Global	Both	50-54 yea	Chlamydia	Rate	2002	1.68862	2.26068	1.26186
DALYs (Dis	Global	Both	55-59 yea	Chlamydia	Number	2002	3379.59	4098.23	2642.22
DALYs (Dis	Global	Both	55-59 yea	Chlamydia	Rate	2002	1.55165	1.8816	1.21311
DALYs (Dis	Global	Both	60-64 yea	Chlamydia	Number	2002	1653.29	1967.15	1287.12
DALYs (Dis	Global	Both	60-64 yea	Chlamydia	Rate	2002	0.86762	1.03233	0.67546
DALYs (Dis	Global	Both	65-69 yea	Chlamydia	Number	2002	1007.61	1195.15	785.855
DALYs (Dis	Global	Both	65-69 yea	Chlamydia	Rate	2002	0.63244	0.75016	0.49326
DALYs (Dis	Global	Both	50-69 yea	Chlamydia	Number	2002	10850.7	13030.2	8804.47
DALYs (Dis	Global	Both	50-69 yea	Chlamydia	Rate	2002	1.27275	1.5284	1.03273
DALYs (Dis	Global	Both	50-54 yea	Gonococca	Number	2002	8432.51	9864.87	6820.91
DALYs (Dis	Global	Both	50-54 yea	Gonococca	Rate	2002	2.96023	3.46306	2.39448
DALYs (Dis	Global	Both	55-59 yea	Gonococca	Number	2002	8046.31	9816.54	5935.75
DALYs (Dis	Global	Both	55-59 yea	Gonococca	Rate	2002	3.69426	4.50701	2.72524
DALYs (Dis	Global	Both	60-64 yea	Gonococca	Number	2002	4738.83	5585.35	3827.98
DALYs (Dis	Global	Both	60-64 yea	Gonococca	Rate	2002	2.48687	2.93111	2.00887
DALYs (Dis	Global	Both	65-69 yea	Gonococca	Number	2002	5653.57	6639.14	4474.47
DALYs (Dis	Global	Both	65-69 yea	Gonococca	Rate	2002	3.54856	4.16717	2.80848
DALYs (Dis	Global	Both	50-69 yea	Gonococca	Number	2002	26871.2	30421	20829.6
DALYs (Dis	Global	Both	50-69 yea	Gonococca	Rate	2002	3.1519	3.56828	2.44324
DALYs (Dis	Global	Both	50-54 yea	Trichomon	Number	2002	14219.4	33810.5	4555.13
DALYs (Dis	Global	Both	50-54 yea	Trichomon	Rate	2002	4.99171	11.8692	1.59908
DALYs (Dis	Global	Both	55-59 yea	Trichomon	Number	2002	8801.5	20473.1	2883.22
DALYs (Dis	Global	Both	55-59 yea	Trichomon	Rate	2002	4.04098	9.39969	1.32376
DALYs (Dis	Global	Both	60-64 yea	Trichomon	Number	2002	6030.09	14117.8	2070.27
DALYs (Dis	Global	Both	60-64 yea	Trichomon	Rate	2002	3.16451	7.40884	1.08645
DALYs (Dis	Global	Both	65-69 yea	Trichomon	Number	2002	4384.35	10163.8	1555.34
DALYs (Dis	Global	Both	65-69 yea	Trichomon	Rate	2002	2.75191	6.37949	0.97624
DALYs (Dis	Global	Both	50-69 yea	Trichomon	Number	2002	33435.3	73712.9	12250.4
DALYs (Dis	Global	Both	50-69 yea	Trichomon	Rate	2002	3.92185	8.64627	1.43692
DALYs (Dis	Global	Both	50-54 yea	Genital her	Number	2002	16113.3	39975	4966.29
DALYs (Dis	Global	Both	50-54 yea	Genital her	Rate	2002	5.65657	14.0332	1.74342
DALYs (Dis	Global	Both	55-59 yea	Genital her	Number	2002	11976.1	29643.6	3687.26
DALYs (Dis	Global	Both	55-59 yea	Genital her	Rate	2002	5.49854	13.6101	1.69291
DALYs (Dis	Global	Both	60-64 yea	Genital her	Number	2002	9895.17	24043.3	3020.68
DALYs (Dis	Global	Both	60-64 yea	Genital her	Rate	2002	5.19284	12.6176	1.58521
DALYs (Dis	Global	Both	65-69 yea	Genital her	Number	2002	7710.1	18852.4	2328.41
DALYs (Dis	Global	Both	65-69 yea	Genital her	Rate	2002	4.83937	11.833	1.46147
DALYs (Dis	Global	Both	50-69 yea	Genital her	Number	2002	45694.7	112663	14098.4
DALYs (Dis	Global	Both	50-69 yea	Genital her	Rate	2002	5.35983	13.215	1.65369
DALYs (Dis	Global	Both	50-54 yea	Other sexu	Number	2002	11110.2	14253	8471.2
DALYs (Dis	Global	Both	50-54 yea	Other sexu	Rate	2002	3.90024	5.0035	2.97381
DALYs (Dis	Global	Both	55-59 yea	Other sexu	Number	2002	8768.55	10681.4	6713.39
DALYs (Dis	Global	Both	55-59 yea	Other sexu	Rate	2002	4.02585	4.90411	3.08228
DALYs (Dis	Global	Both	60-64 yea	Other sexu	Number	2002	3872.59	4667.39	3049.2
DALYs (Dis	Global	Both	60-64 yea	Other sexu	Rate	2002	2.03228	2.44938	1.60018
DALYs (Dis	Global	Both	65-69 yea	Other sexu	Number	2002	2561.29	3044.75	2051.09
DALYs (Dis	Global	Both	65-69 yea	Other sexu	Rate	2002	1.60764	1.91109	1.2874
DALYs (Dis	Global	Both	50-69 yea	Other sexu	Number	2002	26312.7	31185.1	21355.1
DALYs (Dis	Global	Both	50-69 yea	Other sexu	Rate	2002	3.08638	3.65791	2.50487
DALYs (Dis	Global	Both	50-54 yea	Sexually tr	Number	1998	58194.6	93095.7	39341.9
DALYs (Dis	Global	Both	50-54 yea	Sexually tr	Rate	1998	24.2372	38.773	16.3853
DALYs (Dis	Global	Both	55-59 yea	Sexually tr	Number	1998	50866.4	76114.8	35268.6
DALYs (Dis	Global	Both	55-59 yea	Sexually tr	Rate	1998	24.9813	37.3812	17.321
DALYs (Dis	Global	Both	60-64 yea	Sexually tr	Number	1998	34256.1	54586.8	23231.4
DALYs (Dis	Global	Both	60-64 yea	Sexually tr	Rate	1998	18.908	30.1297	12.8228
DALYs (Dis	Global	Both	65-69 yea	Sexually tr	Number	1998	25544.8	39917.1	17794.5
DALYs (Dis	Global	Both	65-69 yea	Sexually tr	Rate	1998	17.1127	26.7409	11.9207
DALYs (Dis	Global	Both	50-69 yea	Sexually tr	Number	1998	168862	266500	117325
DALYs (Dis	Global	Both	50-69 yea	Sexually tr	Rate	1998	21.812	34.4241	15.155
DALYs (Dis	Global	Both	50-54 yea	Syphilis	Number	1998	9202.51	12730.8	5608.04
DALYs (Dis	Global	Both	50-54 yea	Syphilis	Rate	1998	3.83271	5.30219	2.33567
DALYs (Dis	Global	Both	55-59 yea	Syphilis	Number	1998	10291.8	16182.7	5533.44
DALYs (Dis	Global	Both	55-59 yea	Syphilis	Rate	1998	5.05447	7.94756	2.71756
DALYs (Dis	Global	Both	60-64 yea	Syphilis	Number	1998	8062.44	10938.4	5317.35
DALYs (Dis	Global	Both	60-64 yea	Syphilis	Rate	1998	4.45013	6.03756	2.93496
DALYs (Dis	Global	Both	65-69 yea	Syphilis	Number	1998	5186.21	6699.06	4102.94
DALYs (Dis	Global	Both	65-69 yea	Syphilis	Rate	1998	3.4743	4.48777	2.7486
DALYs (Dis	Global	Both	50-69 yea	Syphilis	Number	1998	32743	43727.2	21169.6
DALYs (Dis	Global	Both	50-69 yea	Syphilis	Rate	1998	4.22943	5.64828	2.73449
DALYs (Dis	Global	Both	50-54 yea	Chlamydia	Number	1998	4400.39	5816.65	3332.55
DALYs (Dis	Global	Both	50-54 yea	Chlamydia	Rate	1998	1.8327	2.42255	1.38796
DALYs (Dis	Global	Both	55-59 yea	Chlamydia	Number	1998	3491.02	4312.11	2700.83
DALYs (Dis	Global	Both	55-59 yea	Chlamydia	Rate	1998	1.7145	2.11775	1.32642
DALYs (Dis	Global	Both	60-64 yea	Chlamydia	Number	1998	1766.49	2123.68	1319.18
DALYs (Dis	Global	Both	60-64 yea	Chlamydia	Rate	1998	0.97503	1.17218	0.72813
DALYs (Dis	Global	Both	65-69 yea	Chlamydia	Number	1998	972.958	1166.64	770.486
DALYs (Dis	Global	Both	65-69 yea	Chlamydia	Rate	1998	0.65179	0.78154	0.51616
DALYs (Dis	Global	Both	50-69 yea	Chlamydia	Number	1998	10630.9	12799.2	8667.99
DALYs (Dis	Global	Both	50-69 yea	Chlamydia	Rate	1998	1.3732	1.65328	1.11965
DALYs (Dis	Global	Both	50-54 yea	Gonococca	Number	1998	8261.41	9862.77	6288.63
DALYs (Dis	Global	Both	50-54 yea	Gonococca	Rate	1998	3.44076	4.1077	2.61912
DALYs (Dis	Global	Both	55-59 yea	Gonococca	Number	1998	8481.69	10450.5	6161.57
DALYs (Dis	Global	Both	55-59 yea	Gonococca	Rate	1998	4.16549	5.13241	3.02605
DALYs (Dis	Global	Both	60-64 yea	Gonococca	Number	1998	5085.44	6028.4	3919.2
DALYs (Dis	Global	Both	60-64 yea	Gonococca	Rate	1998	2.80695	3.32743	2.16324
DALYs (Dis	Global	Both	65-69 yea	Gonococca	Number	1998	5504.93	6542.31	4447.85
DALYs (Dis	Global	Both	65-69 yea	Gonococca	Rate	1998	3.68781	4.38276	2.97966
DALYs (Dis	Global	Both	50-69 yea	Gonococca	Number	1998	27333.5	31205.4	20873.6
DALYs (Dis	Global	Both	50-69 yea	Gonococca	Rate	1998	3.53069	4.03082	2.69625
DALYs (Dis	Global	Both	50-54 yea	Trichomon	Number	1998	12218.8	29018	3901.06
DALYs (Dis	Global	Both	50-54 yea	Trichomon	Rate	1998	5.08897	12.0856	1.62473
DALYs (Dis	Global	Both	55-59 yea	Trichomon	Number	1998	8262.96	19241.1	2712
DALYs (Dis	Global	Both	55-59 yea	Trichomon	Rate	1998	4.05807	9.44961	1.3319
DALYs (Dis	Global	Both	60-64 yea	Trichomon	Number	1998	5757.15	13560.2	1972.71
DALYs (Dis	Global	Both	60-64 yea	Trichomon	Rate	1998	3.17771	7.48469	1.08886
DALYs (Dis	Global	Both	65-69 yea	Trichomon	Number	1998	4115.11	9554.42	1459.04
DALYs (Dis	Global	Both	65-69 yea	Trichomon	Rate	1998	2.75676	6.40061	0.97743
DALYs (Dis	Global	Both	50-69 yea	Trichomon	Number	1998	30354.1	67738.2	11123
DALYs (Dis	Global	Both	50-69 yea	Trichomon	Rate	1998	3.92086	8.7498	1.43676
DALYs (Dis	Global	Both	50-54 yea	Genital her	Number	1998	13718.4	34021.8	4245.3
DALYs (Dis	Global	Both	50-54 yea	Genital her	Rate	1998	5.71353	14.1696	1.76811
DALYs (Dis	Global	Both	55-59 yea	Genital her	Number	1998	11248	27785.7	3438.22
DALYs (Dis	Global	Both	55-59 yea	Genital her	Rate	1998	5.52407	13.646	1.68857
DALYs (Dis	Global	Both	60-64 yea	Genital her	Number	1998	9415.94	22860.6	2885.81
DALYs (Dis	Global	Both	60-64 yea	Genital her	Rate	1998	5.19721	12.6181	1.59285
DALYs (Dis	Global	Both	65-69 yea	Genital her	Number	1998	7279.08	17737.6	2205.23
DALYs (Dis	Global	Both	65-69 yea	Genital her	Rate	1998	4.87633	11.8826	1.4773
DALYs (Dis	Global	Both	50-69 yea	Genital her	Number	1998	41661.4	102399	12725.8
DALYs (Dis	Global	Both	50-69 yea	Genital her	Rate	1998	5.38144	13.227	1.6438
DALYs (Dis	Global	Both	50-54 yea	Other sexu	Number	1998	10393	13116.3	8049.31
DALYs (Dis	Global	Both	50-54 yea	Other sexu	Rate	1998	4.32854	5.46274	3.35242
DALYs (Dis	Global	Both	55-59 yea	Other sexu	Number	1998	9090.92	11087.6	6909.3
DALYs (Dis	Global	Both	55-59 yea	Other sexu	Rate	1998	4.4647	5.4453	3.39327
DALYs (Dis	Global	Both	60-64 yea	Other sexu	Number	1998	4168.65	5056.69	3129.87
DALYs (Dis	Global	Both	60-64 yea	Other sexu	Rate	1998	2.30092	2.79108	1.72756
DALYs (Dis	Global	Both	65-69 yea	Other sexu	Number	1998	2486.47	2961.52	2000.66
DALYs (Dis	Global	Both	65-69 yea	Other sexu	Rate	1998	1.66571	1.98395	1.34026
DALYs (Dis	Global	Both	50-69 yea	Other sexu	Number	1998	26139	30663.8	21210.4
DALYs (Dis	Global	Both	50-69 yea	Other sexu	Rate	1998	3.3764	3.96086	2.73976
DALYs (Dis	Global	Both	50-54 yea	HIV/AIDS	Number	2000	3530477	4051054	2998772
DALYs (Dis	Global	Both	50-54 yea	HIV/AIDS	Rate	2000	1346.3	1544.82	1143.54
DALYs (Dis	Global	Both	55-59 yea	HIV/AIDS	Number	2000	1814824	2036963	1574365
DALYs (Dis	Global	Both	55-59 yea	HIV/AIDS	Rate	2000	881.609	989.52	764.798
DALYs (Dis	Global	Both	60-64 yea	HIV/AIDS	Number	2000	970807	1090648	851098
DALYs (Dis	Global	Both	60-64 yea	HIV/AIDS	Rate	2000	519.269	583.37	455.238
DALYs (Dis	Global	Both	65-69 yea	HIV/AIDS	Number	2000	515516	582556	451288
DALYs (Dis	Global	Both	65-69 yea	HIV/AIDS	Rate	2000	336.546	380.312	294.616
DALYs (Dis	Global	Both	50-69 yea	HIV/AIDS	Number	2000	6831624	7754878	5918634
DALYs (Dis	Global	Both	50-69 yea	HIV/AIDS	Rate	2000	845.264	959.497	732.302
DALYs (Dis	Global	Both	50-54 yea	HIV/AIDS	Number	2007	4564396	4998460	4149155
DALYs (Dis	Global	Both	50-54 yea	HIV/AIDS	Rate	2007	1378.99	1510.13	1253.54
DALYs (Dis	Global	Both	55-59 yea	HIV/AIDS	Number	2007	2216130	2461408	2005600
DALYs (Dis	Global	Both	55-59 yea	HIV/AIDS	Rate	2007	814.963	905.163	737.543
DALYs (Dis	Global	Both	60-64 yea	HIV/AIDS	Number	2007	1072680	1215036	957574
DALYs (Dis	Global	Both	60-64 yea	HIV/AIDS	Rate	2007	516.809	585.395	461.352
DALYs (Dis	Global	Both	65-69 yea	HIV/AIDS	Number	2007	546070	622185	485472
DALYs (Dis	Global	Both	65-69 yea	HIV/AIDS	Rate	2007	316.092	360.151	281.015
DALYs (Dis	Global	Both	50-69 yea	HIV/AIDS	Number	2007	8399276	9253487	7627177
DALYs (Dis	Global	Both	50-69 yea	HIV/AIDS	Rate	2007	854.245	941.122	775.719
DALYs (Dis	Global	Both	50-54 yea	HIV/AIDS	Number	2004	4805900	5175562	4440785
DALYs (Dis	Global	Both	50-54 yea	HIV/AIDS	Rate	2004	1579.32	1700.8	1459.33
DALYs (Dis	Global	Both	55-59 yea	HIV/AIDS	Number	2004	2267397	2419221	2122519
DALYs (Dis	Global	Both	55-59 yea	HIV/AIDS	Rate	2004	946.703	1010.09	886.212
DALYs (Dis	Global	Both	60-64 yea	HIV/AIDS	Number	2004	1160034	1246840	1081031
DALYs (Dis	Global	Both	60-64 yea	HIV/AIDS	Rate	2004	600.093	644.999	559.224
DALYs (Dis	Global	Both	65-69 yea	HIV/AIDS	Number	2004	605650	652781	564897
DALYs (Dis	Global	Both	65-69 yea	HIV/AIDS	Rate	2004	365.533	393.978	340.937
DALYs (Dis	Global	Both	50-69 yea	HIV/AIDS	Number	2004	8838980	9458299	8258040
DALYs (Dis	Global	Both	50-69 yea	HIV/AIDS	Rate	2004	979.057	1047.66	914.709
DALYs (Dis	Global	Both	50-54 yea	Sexually tr	Number	2000	60231	97536.1	40286.7
DALYs (Dis	Global	Both	50-54 yea	Sexually tr	Rate	2000	22.9683	37.1942	15.3629
DALYs (Dis	Global	Both	55-59 yea	Sexually tr	Number	2000	49505.6	75464.8	34001.7
DALYs (Dis	Global	Both	55-59 yea	Sexually tr	Rate	2000	24.0489	36.6594	16.5174
DALYs (Dis	Global	Both	60-64 yea	Sexually tr	Number	2000	33859.3	54564.8	22502.7
DALYs (Dis	Global	Both	60-64 yea	Sexually tr	Rate	2000	18.1108	29.1858	12.0363
DALYs (Dis	Global	Both	65-69 yea	Sexually tr	Number	2000	25226.3	40034.6	17170.3
DALYs (Dis	Global	Both	65-69 yea	Sexually tr	Rate	2000	16.4686	26.1359	11.2094
DALYs (Dis	Global	Both	50-69 yea	Sexually tr	Number	2000	168822	270352	115746
DALYs (Dis	Global	Both	50-69 yea	Sexually tr	Rate	2000	20.8881	33.4501	14.321
DALYs (Dis	Global	Both	50-54 yea	Syphilis	Number	2000	9142.59	12898.9	5711.48
DALYs (Dis	Global	Both	50-54 yea	Syphilis	Rate	2000	3.48641	4.91882	2.178
DALYs (Dis	Global	Both	55-59 yea	Syphilis	Number	2000	9380.66	14715.4	5018.61
DALYs (Dis	Global	Both	55-59 yea	Syphilis	Rate	2000	4.55695	7.14848	2.43795
DALYs (Dis	Global	Both	60-64 yea	Syphilis	Number	2000	7789.5	10567.5	5213.34
DALYs (Dis	Global	Both	60-64 yea	Syphilis	Rate	2000	4.16648	5.65236	2.78853
DALYs (Dis	Global	Both	65-69 yea	Syphilis	Number	2000	4770.38	6157.55	3818.22
DALYs (Dis	Global	Both	65-69 yea	Syphilis	Rate	2000	3.11427	4.01985	2.49266
DALYs (Dis	Global	Both	50-69 yea	Syphilis	Number	2000	31083.1	42187.2	20315.2
DALYs (Dis	Global	Both	50-69 yea	Syphilis	Rate	2000	3.84586	5.21975	2.51356
DALYs (Dis	Global	Both	50-54 yea	Chlamydia	Number	2000	4523.03	6002.24	3378
DALYs (Dis	Global	Both	50-54 yea	Chlamydia	Rate	2000	1.7248	2.28888	1.28816
DALYs (Dis	Global	Both	55-59 yea	Chlamydia	Number	2000	3400.54	4167.16	2666.22
DALYs (Dis	Global	Both	55-59 yea	Chlamydia	Rate	2000	1.65192	2.02433	1.2952
DALYs (Dis	Global	Both	60-64 yea	Chlamydia	Number	2000	1689.15	2024.16	1318.18
DALYs (Dis	Global	Both	60-64 yea	Chlamydia	Rate	2000	0.9035	1.08269	0.70507
DALYs (Dis	Global	Both	65-69 yea	Chlamydia	Number	2000	963.444	1149.98	761.013
DALYs (Dis	Global	Both	65-69 yea	Chlamydia	Rate	2000	0.62897	0.75074	0.49681
DALYs (Dis	Global	Both	50-69 yea	Chlamydia	Number	2000	10576.2	12618.2	8671.24
DALYs (Dis	Global	Both	50-69 yea	Chlamydia	Rate	2000	1.30857	1.56122	1.07288
DALYs (Dis	Global	Both	50-54 yea	Gonococca	Number	2000	8075	9558.1	6582.34
DALYs (Dis	Global	Both	50-54 yea	Gonococca	Rate	2000	3.0793	3.64486	2.51009
DALYs (Dis	Global	Both	55-59 yea	Gonococca	Number	2000	8211.93	10018.1	6084.3
DALYs (Dis	Global	Both	55-59 yea	Gonococca	Rate	2000	3.98921	4.86662	2.95564
DALYs (Dis	Global	Both	60-64 yea	Gonococca	Number	2000	4875.33	5785.59	3937.78
DALYs (Dis	Global	Both	60-64 yea	Gonococca	Rate	2000	2.60773	3.09462	2.10625
DALYs (Dis	Global	Both	65-69 yea	Gonococca	Number	2000	5431.67	6435.66	4301.28
DALYs (Dis	Global	Both	65-69 yea	Gonococca	Rate	2000	3.54597	4.20142	2.80802
DALYs (Dis	Global	Both	50-69 yea	Gonococca	Number	2000	26593.9	29990	20718.6
DALYs (Dis	Global	Both	50-69 yea	Gonococca	Rate	2000	3.29042	3.71061	2.56347
DALYs (Dis	Global	Both	50-54 yea	Trichomon	Number	2000	13135.3	31197.9	4185.78
DALYs (Dis	Global	Both	50-54 yea	Trichomon	Rate	2000	5.00897	11.8969	1.5962
DALYs (Dis	Global	Both	55-59 yea	Trichomon	Number	2000	8341.36	19447.6	2726.31
DALYs (Dis	Global	Both	55-59 yea	Trichomon	Rate	2000	4.05208	9.44728	1.32439
DALYs (Dis	Global	Both	60-64 yea	Trichomon	Number	2000	5886.65	13750.7	2013.41
DALYs (Dis	Global	Both	60-64 yea	Trichomon	Rate	2000	3.14867	7.35502	1.07694
DALYs (Dis	Global	Both	65-69 yea	Trichomon	Number	2000	4201.31	9719.53	1480.07
DALYs (Dis	Global	Both	65-69 yea	Trichomon	Rate	2000	2.74276	6.34524	0.96624
DALYs (Dis	Global	Both	50-69 yea	Trichomon	Number	2000	31564.6	70132	11582.1
DALYs (Dis	Global	Both	50-69 yea	Trichomon	Rate	2000	3.90543	8.6773	1.43304
DALYs (Dis	Global	Both	50-54 yea	Genital her	Number	2000	14805.2	36748.7	4568.98
DALYs (Dis	Global	Both	50-54 yea	Genital her	Rate	2000	5.64578	14.0137	1.74232
DALYs (Dis	Global	Both	55-59 yea	Genital her	Number	2000	11311.6	27895.5	3459.67
DALYs (Dis	Global	Both	55-59 yea	Genital her	Rate	2000	5.49498	13.5512	1.68064
DALYs (Dis	Global	Both	60-64 yea	Genital her	Number	2000	9661.93	23600.6	2936.23
DALYs (Dis	Global	Both	60-64 yea	Genital her	Rate	2000	5.16801	12.6236	1.57054
DALYs (Dis	Global	Both	65-69 yea	Genital her	Number	2000	7397.64	18040.8	2242.36
DALYs (Dis	Global	Both	65-69 yea	Genital her	Rate	2000	4.82943	11.7776	1.46389
DALYs (Dis	Global	Both	50-69 yea	Genital her	Number	2000	43176.4	105824	13209.2
DALYs (Dis	Global	Both	50-69 yea	Genital her	Rate	2000	5.34214	13.0934	1.63435
DALYs (Dis	Global	Both	50-54 yea	Other sexu	Number	2000	10549.9	13501.6	8110.93
DALYs (Dis	Global	Both	50-54 yea	Other sexu	Rate	2000	4.02307	5.14867	3.093
DALYs (Dis	Global	Both	55-59 yea	Other sexu	Number	2000	8859.46	10796.1	6762.83
DALYs (Dis	Global	Both	55-59 yea	Other sexu	Rate	2000	4.30376	5.24455	3.28526
DALYs (Dis	Global	Both	60-64 yea	Other sexu	Number	2000	3956.76	4799	3121.93
DALYs (Dis	Global	Both	60-64 yea	Other sexu	Rate	2000	2.11641	2.5669	1.66987
DALYs (Dis	Global	Both	65-69 yea	Other sexu	Number	2000	2461.9	2923.39	1967.89
DALYs (Dis	Global	Both	65-69 yea	Other sexu	Rate	2000	1.60721	1.90849	1.2847
DALYs (Dis	Global	Both	50-69 yea	Other sexu	Number	2000	25828	30501.3	21129
DALYs (Dis	Global	Both	50-69 yea	Other sexu	Rate	2000	3.19565	3.77387	2.61425
DALYs (Dis	Global	Both	50-54 yea	Sexually tr	Number	2004	65284.4	107788	42202.2
DALYs (Dis	Global	Both	50-54 yea	Sexually tr	Rate	2004	21.4538	35.4215	13.8685
DALYs (Dis	Global	Both	55-59 yea	Sexually tr	Number	2004	51018.7	80299.4	33793.2
DALYs (Dis	Global	Both	55-59 yea	Sexually tr	Rate	2004	21.3018	33.5273	14.1096
DALYs (Dis	Global	Both	60-64 yea	Sexually tr	Number	2004	31792.5	52882.6	20669.2
DALYs (Dis	Global	Both	60-64 yea	Sexually tr	Rate	2004	16.4465	27.3565	10.6923
DALYs (Dis	Global	Both	65-69 yea	Sexually tr	Number	2004	25177.5	41201.8	16706.3
DALYs (Dis	Global	Both	65-69 yea	Sexually tr	Rate	2004	15.1956	24.8669	10.0829
DALYs (Dis	Global	Both	50-69 yea	Sexually tr	Number	2004	173273	285594	114940
DALYs (Dis	Global	Both	50-69 yea	Sexually tr	Rate	2004	19.1927	31.634	12.7315
DALYs (Dis	Global	Both	50-54 yea	Syphilis	Number	2004	8163.11	11162.7	5240.76
DALYs (Dis	Global	Both	50-54 yea	Syphilis	Rate	2004	2.68257	3.66828	1.72222
DALYs (Dis	Global	Both	55-59 yea	Syphilis	Number	2004	7999.34	12359	4355.92
DALYs (Dis	Global	Both	55-59 yea	Syphilis	Rate	2004	3.33995	5.16025	1.81872
DALYs (Dis	Global	Both	60-64 yea	Syphilis	Number	2004	5804.55	7950.51	3903.38
DALYs (Dis	Global	Both	60-64 yea	Syphilis	Rate	2004	3.00273	4.11285	2.01924
DALYs (Dis	Global	Both	65-69 yea	Syphilis	Number	2004	3847.17	5188.3	3116.53
DALYs (Dis	Global	Both	65-69 yea	Syphilis	Rate	2004	2.32192	3.13134	1.88095
DALYs (Dis	Global	Both	50-69 yea	Syphilis	Number	2004	25814.2	34482.7	17496.8
DALYs (Dis	Global	Both	50-69 yea	Syphilis	Rate	2004	2.85933	3.81951	1.93805
DALYs (Dis	Global	Both	50-54 yea	Chlamydia	Number	2004	4959.27	6621.11	3674.02
DALYs (Dis	Global	Both	50-54 yea	Chlamydia	Rate	2004	1.62972	2.17583	1.20736
DALYs (Dis	Global	Both	55-59 yea	Chlamydia	Number	2004	3434.35	4126.53	2712.97
DALYs (Dis	Global	Both	55-59 yea	Chlamydia	Rate	2004	1.43394	1.72294	1.13274
DALYs (Dis	Global	Both	60-64 yea	Chlamydia	Number	2004	1572.96	1853.11	1242.48
DALYs (Dis	Global	Both	60-64 yea	Chlamydia	Rate	2004	0.8137	0.95863	0.64275
DALYs (Dis	Global	Both	65-69 yea	Chlamydia	Number	2004	951.902	1128.13	745.012
DALYs (Dis	Global	Both	65-69 yea	Chlamydia	Rate	2004	0.57451	0.68087	0.44964
DALYs (Dis	Global	Both	50-69 yea	Chlamydia	Number	2004	10918.5	13232.4	8983.73
DALYs (Dis	Global	Both	50-69 yea	Chlamydia	Rate	2004	1.20939	1.4657	0.99509
DALYs (Dis	Global	Both	50-54 yea	Gonococca	Number	2004	8369.36	9807.53	6977.98
DALYs (Dis	Global	Both	50-54 yea	Gonococca	Rate	2004	2.75035	3.22296	2.29311
DALYs (Dis	Global	Both	55-59 yea	Gonococca	Number	2004	8001.87	9692.33	6031.84
DALYs (Dis	Global	Both	55-59 yea	Gonococca	Rate	2004	3.34101	4.04683	2.51847
DALYs (Dis	Global	Both	60-64 yea	Gonococca	Number	2004	4475.35	5266.86	3621.53
DALYs (Dis	Global	Both	60-64 yea	Gonococca	Rate	2004	2.31513	2.72458	1.87344
DALYs (Dis	Global	Both	65-69 yea	Gonococca	Number	2004	5345.21	6273.24	4311.18
DALYs (Dis	Global	Both	65-69 yea	Gonococca	Rate	2004	3.22604	3.78614	2.60196
DALYs (Dis	Global	Both	50-69 yea	Gonococca	Number	2004	26191.8	29818.3	20895.4
DALYs (Dis	Global	Both	50-69 yea	Gonococca	Rate	2004	2.90116	3.30285	2.31449
DALYs (Dis	Global	Both	50-54 yea	Trichomon	Number	2004	15186.8	36135.2	4876.96
DALYs (Dis	Global	Both	50-54 yea	Trichomon	Rate	2004	4.99071	11.8748	1.60267
DALYs (Dis	Global	Both	55-59 yea	Trichomon	Number	2004	9598.16	22333.8	3178.93
DALYs (Dis	Global	Both	55-59 yea	Trichomon	Rate	2004	4.00751	9.32502	1.32729
DALYs (Dis	Global	Both	60-64 yea	Trichomon	Number	2004	6144.67	14390.9	2107.79
DALYs (Dis	Global	Both	60-64 yea	Trichomon	Rate	2004	3.17868	7.44451	1.09038
DALYs (Dis	Global	Both	65-69 yea	Trichomon	Number	2004	4560.2	10634.9	1628.78
DALYs (Dis	Global	Both	65-69 yea	Trichomon	Rate	2004	2.75225	6.41855	0.98303
DALYs (Dis	Global	Both	50-69 yea	Trichomon	Number	2004	35489.9	78402.4	12986.2
DALYs (Dis	Global	Both	50-69 yea	Trichomon	Rate	2004	3.93106	8.68431	1.43843
DALYs (Dis	Global	Both	50-54 yea	Genital her	Number	2004	17284.8	42932.3	5318.03
DALYs (Dis	Global	Both	50-54 yea	Genital her	Rate	2004	5.68013	14.1084	1.74762
DALYs (Dis	Global	Both	55-59 yea	Genital her	Number	2004	13137.5	32268.1	3996.96
DALYs (Dis	Global	Both	55-59 yea	Genital her	Rate	2004	5.48527	13.4728	1.66885
DALYs (Dis	Global	Both	60-64 yea	Genital her	Number	2004	10082	24521.1	3060.66
DALYs (Dis	Global	Both	60-64 yea	Genital her	Rate	2004	5.21548	12.6849	1.5833
DALYs (Dis	Global	Both	65-69 yea	Genital her	Number	2004	8054.91	19564.8	2420.98
DALYs (Dis	Global	Both	65-69 yea	Genital her	Rate	2004	4.86145	11.8081	1.46116
DALYs (Dis	Global	Both	50-69 yea	Genital her	Number	2004	48559.1	119469	14776.5
DALYs (Dis	Global	Both	50-69 yea	Genital her	Rate	2004	5.3787	13.2331	1.63673
DALYs (Dis	Global	Both	50-54 yea	Other sexu	Number	2004	11321.1	14482.8	8642.64
DALYs (Dis	Global	Both	50-54 yea	Other sexu	Rate	2004	3.72034	4.75935	2.84015
DALYs (Dis	Global	Both	55-59 yea	Other sexu	Number	2004	8847.47	10786.7	6886.81
DALYs (Dis	Global	Both	55-59 yea	Other sexu	Rate	2004	3.69407	4.50376	2.87544
DALYs (Dis	Global	Both	60-64 yea	Other sexu	Number	2004	3713.03	4453.26	2944.43
DALYs (Dis	Global	Both	60-64 yea	Other sexu	Rate	2004	1.92078	2.3037	1.52317
DALYs (Dis	Global	Both	65-69 yea	Other sexu	Number	2004	2418.08	2886.55	1984.32
DALYs (Dis	Global	Both	65-69 yea	Other sexu	Rate	2004	1.4594	1.74214	1.19761
DALYs (Dis	Global	Both	50-69 yea	Other sexu	Number	2004	26299.7	31219.9	21474.9
DALYs (Dis	Global	Both	50-69 yea	Other sexu	Rate	2004	2.91311	3.4581	2.37869
DALYs (Dis	Global	Both	50-54 yea	Sexually tr	Number	2007	68322.2	114343	42965.2
DALYs (Dis	Global	Both	50-54 yea	Sexually tr	Rate	2007	20.6415	34.5452	12.9806
DALYs (Dis	Global	Both	55-59 yea	Sexually tr	Number	2007	54892.9	87372.7	36140.2
DALYs (Dis	Global	Both	55-59 yea	Sexually tr	Rate	2007	20.1864	32.1306	13.2903
DALYs (Dis	Global	Both	60-64 yea	Sexually tr	Number	2007	32314.3	54231.8	20605.5
DALYs (Dis	Global	Both	60-64 yea	Sexually tr	Rate	2007	15.5688	26.1284	9.92755
DALYs (Dis	Global	Both	65-69 yea	Sexually tr	Number	2007	25310.9	42136.2	16523.9
DALYs (Dis	Global	Both	65-69 yea	Sexually tr	Rate	2007	14.6512	24.3905	9.56484
DALYs (Dis	Global	Both	50-69 yea	Sexually tr	Number	2007	180840	299425	118007
DALYs (Dis	Global	Both	50-69 yea	Sexually tr	Rate	2007	18.3923	30.4529	12.0018
DALYs (Dis	Global	Both	50-54 yea	Syphilis	Number	2007	7666.26	10254.7	5044.94
DALYs (Dis	Global	Both	50-54 yea	Syphilis	Rate	2007	2.31613	3.09814	1.52417
DALYs (Dis	Global	Both	55-59 yea	Syphilis	Number	2007	7665.22	11292.3	4270.57
DALYs (Dis	Global	Both	55-59 yea	Syphilis	Rate	2007	2.81882	4.15266	1.57047
DALYs (Dis	Global	Both	60-64 yea	Syphilis	Number	2007	5370.16	7204.04	3706.09
DALYs (Dis	Global	Both	60-64 yea	Syphilis	Rate	2007	2.5873	3.47085	1.78557
DALYs (Dis	Global	Both	65-69 yea	Syphilis	Number	2007	3483.36	4715.21	2831.88
DALYs (Dis	Global	Both	65-69 yea	Syphilis	Rate	2007	2.01634	2.72939	1.63923
DALYs (Dis	Global	Both	50-69 yea	Syphilis	Number	2007	24185	31244.8	16365.8
DALYs (Dis	Global	Both	50-69 yea	Syphilis	Rate	2007	2.45972	3.17774	1.66447
DALYs (Dis	Global	Both	50-54 yea	Chlamydia	Number	2007	5171.31	6995.98	3761.68
DALYs (Dis	Global	Both	50-54 yea	Chlamydia	Rate	2007	1.56236	2.11362	1.13648
DALYs (Dis	Global	Both	55-59 yea	Chlamydia	Number	2007	3695.35	4425.01	2929.64
DALYs (Dis	Global	Both	55-59 yea	Chlamydia	Rate	2007	1.35893	1.62726	1.07735
DALYs (Dis	Global	Both	60-64 yea	Chlamydia	Number	2007	1574.78	1849.12	1243.58
DALYs (Dis	Global	Both	60-64 yea	Chlamydia	Rate	2007	0.75872	0.89089	0.59915
DALYs (Dis	Global	Both	65-69 yea	Chlamydia	Number	2007	960.856	1120.83	761.846
DALYs (Dis	Global	Both	65-69 yea	Chlamydia	Rate	2007	0.55619	0.64879	0.44099
DALYs (Dis	Global	Both	50-69 yea	Chlamydia	Number	2007	11402.3	13829	9237.69
DALYs (Dis	Global	Both	50-69 yea	Chlamydia	Rate	2007	1.15967	1.40647	0.93952
DALYs (Dis	Global	Both	50-54 yea	Gonococca	Number	2007	8492.31	9872.21	6974.28
DALYs (Dis	Global	Both	50-54 yea	Gonococca	Rate	2007	2.56569	2.98259	2.10707
DALYs (Dis	Global	Both	55-59 yea	Gonococca	Number	2007	8478.66	10035.2	6361.7
DALYs (Dis	Global	Both	55-59 yea	Gonococca	Rate	2007	3.11796	3.69035	2.33946
DALYs (Dis	Global	Both	60-64 yea	Gonococca	Number	2007	4422.28	5139.04	3525.42
DALYs (Dis	Global	Both	60-64 yea	Gonococca	Rate	2007	2.13062	2.47595	1.69852
DALYs (Dis	Global	Both	65-69 yea	Gonococca	Number	2007	5361.49	6248.94	4317.76
DALYs (Dis	Global	Both	65-69 yea	Gonococca	Rate	2007	3.1035	3.61719	2.49933
DALYs (Dis	Global	Both	50-69 yea	Gonococca	Number	2007	26754.7	30117.4	21115.3
DALYs (Dis	Global	Both	50-69 yea	Gonococca	Rate	2007	2.72108	3.06307	2.14752
DALYs (Dis	Global	Both	50-54 yea	Trichomon	Number	2007	16594.2	39301.2	5314.91
DALYs (Dis	Global	Both	50-54 yea	Trichomon	Rate	2007	5.01342	11.8736	1.60574
DALYs (Dis	Global	Both	55-59 yea	Trichomon	Number	2007	10866.9	25199.5	3549.58
DALYs (Dis	Global	Both	55-59 yea	Trichomon	Rate	2007	3.9962	9.26691	1.30533
DALYs (Dis	Global	Both	60-64 yea	Trichomon	Number	2007	6510.04	15149.8	2236.13
DALYs (Dis	Global	Both	60-64 yea	Trichomon	Rate	2007	3.13649	7.29904	1.07735
DALYs (Dis	Global	Both	65-69 yea	Trichomon	Number	2007	4715.04	10948	1677.67
DALYs (Dis	Global	Both	65-69 yea	Trichomon	Rate	2007	2.7293	6.33724	0.97112
DALYs (Dis	Global	Both	50-69 yea	Trichomon	Number	2007	38686.1	85427.2	14230.4
DALYs (Dis	Global	Both	50-69 yea	Trichomon	Rate	2007	3.93456	8.68834	1.44729
DALYs (Dis	Global	Both	50-54 yea	Genital her	Number	2007	18708.9	46614.2	5764.39
DALYs (Dis	Global	Both	50-54 yea	Genital her	Rate	2007	5.65232	14.0831	1.74154
DALYs (Dis	Global	Both	55-59 yea	Genital her	Number	2007	14790.9	36444.6	4516.48
DALYs (Dis	Global	Both	55-59 yea	Genital her	Rate	2007	5.43925	13.4022	1.6609
DALYs (Dis	Global	Both	60-64 yea	Genital her	Number	2007	10724.7	26179.6	3281.93
DALYs (Dis	Global	Both	60-64 yea	Genital her	Rate	2007	5.16709	12.6131	1.58121
DALYs (Dis	Global	Both	65-69 yea	Genital her	Number	2007	8364.94	20477.9	2527.1
DALYs (Dis	Global	Both	65-69 yea	Genital her	Rate	2007	4.84204	11.8536	1.46281
DALYs (Dis	Global	Both	50-69 yea	Genital her	Number	2007	52589.5	129570	16106.9
DALYs (Dis	Global	Both	50-69 yea	Genital her	Rate	2007	5.34859	13.1779	1.63814
DALYs (Dis	Global	Both	50-54 yea	Other sexu	Number	2007	11689.3	15040.9	8882.91
DALYs (Dis	Global	Both	50-54 yea	Other sexu	Rate	2007	3.53156	4.54414	2.6837
DALYs (Dis	Global	Both	55-59 yea	Other sexu	Number	2007	9395.8	11437.1	7450.54
DALYs (Dis	Global	Both	55-59 yea	Other sexu	Rate	2007	3.45523	4.20588	2.73988
DALYs (Dis	Global	Both	60-64 yea	Other sexu	Number	2007	3712.3	4356.55	2906.11
DALYs (Dis	Global	Both	60-64 yea	Other sexu	Rate	2007	1.78856	2.09895	1.40014
DALYs (Dis	Global	Both	65-69 yea	Other sexu	Number	2007	2425.22	2831.57	1971.35
DALYs (Dis	Global	Both	65-69 yea	Other sexu	Rate	2007	1.40383	1.63905	1.14111
DALYs (Dis	Global	Both	50-69 yea	Other sexu	Number	2007	27222.6	32316.9	22277.3
DALYs (Dis	Global	Both	50-69 yea	Other sexu	Rate	2007	2.76866	3.28678	2.2657
DALYs (Dis	Global	Both	50-54 yea	Sexually tr	Number	2006	67714.2	112685	43272.1
DALYs (Dis	Global	Both	50-54 yea	Sexually tr	Rate	2006	20.8756	34.7395	13.3403
DALYs (Dis	Global	Both	55-59 yea	Sexually tr	Number	2006	53502.2	85398.5	35349.4
DALYs (Dis	Global	Both	55-59 yea	Sexually tr	Rate	2006	20.3778	32.5264	13.4638
DALYs (Dis	Global	Both	60-64 yea	Sexually tr	Number	2006	31632.5	52776.5	20341.9
DALYs (Dis	Global	Both	60-64 yea	Sexually tr	Rate	2006	15.9061	26.5382	10.2288
DALYs (Dis	Global	Both	65-69 yea	Sexually tr	Number	2006	25461.3	41841.2	16685.6
DALYs (Dis	Global	Both	65-69 yea	Sexually tr	Rate	2006	14.8914	24.4713	9.75879
DALYs (Dis	Global	Both	50-69 yea	Sexually tr	Number	2006	178310	295062	116930
DALYs (Dis	Global	Both	50-69 yea	Sexually tr	Rate	2006	18.6366	30.8393	12.2213
DALYs (Dis	Global	Both	50-54 yea	Syphilis	Number	2006	7765.54	10406.8	5052.5
DALYs (Dis	Global	Both	50-54 yea	Syphilis	Rate	2006	2.39403	3.2083	1.55763
DALYs (Dis	Global	Both	55-59 yea	Syphilis	Number	2006	7670.14	11455.4	4269.24
DALYs (Dis	Global	Both	55-59 yea	Syphilis	Rate	2006	2.92139	4.36311	1.62606
DALYs (Dis	Global	Both	60-64 yea	Syphilis	Number	2006	5389.66	7272.58	3662.71
DALYs (Dis	Global	Both	60-64 yea	Syphilis	Rate	2006	2.71015	3.65696	1.84176
DALYs (Dis	Global	Both	65-69 yea	Syphilis	Number	2006	3567.06	4836.82	2884.37
DALYs (Dis	Global	Both	65-69 yea	Syphilis	Rate	2006	2.08624	2.82887	1.68696
DALYs (Dis	Global	Both	50-69 yea	Syphilis	Number	2006	24392.4	31655.5	16472.7
DALYs (Dis	Global	Both	50-69 yea	Syphilis	Rate	2006	2.54945	3.30858	1.7217
DALYs (Dis	Global	Both	50-54 yea	Chlamydia	Number	2006	5153.12	6938.03	3821.08
DALYs (Dis	Global	Both	50-54 yea	Chlamydia	Rate	2006	1.58865	2.13892	1.178
DALYs (Dis	Global	Both	55-59 yea	Chlamydia	Number	2006	3587.2	4332.07	2825.92
DALYs (Dis	Global	Both	55-59 yea	Chlamydia	Rate	2006	1.36629	1.64999	1.07633
DALYs (Dis	Global	Both	60-64 yea	Chlamydia	Number	2006	1547.11	1813.35	1223.4
DALYs (Dis	Global	Both	60-64 yea	Chlamydia	Rate	2006	0.77795	0.91183	0.61518
DALYs (Dis	Global	Both	65-69 yea	Chlamydia	Number	2006	974.731	1155.85	756.04
DALYs (Dis	Global	Both	65-69 yea	Chlamydia	Rate	2006	0.57008	0.67601	0.44218
DALYs (Dis	Global	Both	50-69 yea	Chlamydia	Number	2006	11262.2	13693.2	9167.47
DALYs (Dis	Global	Both	50-69 yea	Chlamydia	Rate	2006	1.1771	1.43118	0.95817
DALYs (Dis	Global	Both	50-54 yea	Gonococca	Number	2006	8501.1	10031.7	7049.85
DALYs (Dis	Global	Both	50-54 yea	Gonococca	Rate	2006	2.6208	3.09267	2.17339
DALYs (Dis	Global	Both	55-59 yea	Gonococca	Number	2006	8235.42	9856.8	6189.66
DALYs (Dis	Global	Both	55-59 yea	Gonococca	Rate	2006	3.13669	3.75424	2.3575
DALYs (Dis	Global	Both	60-64 yea	Gonococca	Number	2006	4360.5	5072.51	3499.46
DALYs (Dis	Global	Both	60-64 yea	Gonococca	Rate	2006	2.19264	2.55067	1.75968
DALYs (Dis	Global	Both	65-69 yea	Gonococca	Number	2006	5447.76	6422.13	4321.07
DALYs (Dis	Global	Both	65-69 yea	Gonococca	Rate	2006	3.18619	3.75606	2.52723
DALYs (Dis	Global	Both	50-69 yea	Gonococca	Number	2006	26544.8	30026.1	21299.6
DALYs (Dis	Global	Both	50-69 yea	Gonococca	Rate	2006	2.77441	3.13826	2.22619
DALYs (Dis	Global	Both	50-54 yea	Trichomon	Number	2006	16224.8	38381	5182.68
DALYs (Dis	Global	Both	50-54 yea	Trichomon	Rate	2006	5.00192	11.8325	1.59776
DALYs (Dis	Global	Both	55-59 yea	Trichomon	Number	2006	10495.9	24417.9	3444.46
DALYs (Dis	Global	Both	55-59 yea	Trichomon	Rate	2006	3.99764	9.30026	1.31192
DALYs (Dis	Global	Both	60-64 yea	Trichomon	Number	2006	6310.53	14773.7	2184.69
DALYs (Dis	Global	Both	60-64 yea	Trichomon	Rate	2006	3.1732	7.42886	1.09855
DALYs (Dis	Global	Both	65-69 yea	Trichomon	Number	2006	4693.19	10843.7	1665.85
DALYs (Dis	Global	Both	65-69 yea	Trichomon	Rate	2006	2.74487	6.34209	0.97429
DALYs (Dis	Global	Both	50-69 yea	Trichomon	Number	2006	37724.4	83220.8	13842.2
DALYs (Dis	Global	Both	50-69 yea	Trichomon	Rate	2006	3.94288	8.69808	1.44676
DALYs (Dis	Global	Both	50-54 yea	Genital her	Number	2006	18425	45742	5690.87
DALYs (Dis	Global	Both	50-54 yea	Genital her	Rate	2006	5.68023	14.1018	1.75443
DALYs (Dis	Global	Both	55-59 yea	Genital her	Number	2006	14362.4	35486.1	4450.26
DALYs (Dis	Global	Both	55-59 yea	Genital her	Rate	2006	5.47031	13.5159	1.69501
DALYs (Dis	Global	Both	60-64 yea	Genital her	Number	2006	10360	25227	3155.94
DALYs (Dis	Global	Both	60-64 yea	Genital her	Rate	2006	5.20945	12.6852	1.58694
DALYs (Dis	Global	Both	65-69 yea	Genital her	Number	2006	8315.8	20238.6	2516.8
DALYs (Dis	Global	Both	65-69 yea	Genital her	Rate	2006	4.86359	11.8368	1.47198
DALYs (Dis	Global	Both	50-69 yea	Genital her	Number	2006	51463.2	126907	15822
DALYs (Dis	Global	Both	50-69 yea	Genital her	Rate	2006	5.37884	13.2641	1.65368
DALYs (Dis	Global	Both	50-54 yea	Other sexu	Number	2006	11644.7	14951.8	8916.86
DALYs (Dis	Global	Both	50-54 yea	Other sexu	Rate	2006	3.58992	4.60947	2.74897
DALYs (Dis	Global	Both	55-59 yea	Other sexu	Number	2006	9151.19	11150	7186.77
DALYs (Dis	Global	Both	55-59 yea	Other sexu	Rate	2006	3.48549	4.24679	2.73728
DALYs (Dis	Global	Both	60-64 yea	Other sexu	Number	2006	3664.66	4341.37	2908.89
DALYs (Dis	Global	Both	60-64 yea	Other sexu	Rate	2006	1.84275	2.18302	1.46271
DALYs (Dis	Global	Both	65-69 yea	Other sexu	Number	2006	2462.79	2918.26	1976.19
DALYs (Dis	Global	Both	65-69 yea	Other sexu	Rate	2006	1.44039	1.70678	1.1558
DALYs (Dis	Global	Both	50-69 yea	Other sexu	Number	2006	26923.3	32106.3	21914.9
DALYs (Dis	Global	Both	50-69 yea	Other sexu	Rate	2006	2.81397	3.35569	2.2905
DALYs (Dis	Global	Both	50-54 yea	HIV/AIDS	Number	2003	4574332	4982698	4128576
DALYs (Dis	Global	Both	50-54 yea	HIV/AIDS	Rate	2003	1550.16	1688.55	1399.1
DALYs (Dis	Global	Both	55-59 yea	HIV/AIDS	Number	2003	2195581	2367538	2034235
DALYs (Dis	Global	Both	55-59 yea	HIV/AIDS	Rate	2003	965.634	1041.26	894.673
DALYs (Dis	Global	Both	60-64 yea	HIV/AIDS	Number	2003	1133086	1225229	1053937
DALYs (Dis	Global	Both	60-64 yea	HIV/AIDS	Rate	2003	590.999	639.06	549.716
DALYs (Dis	Global	Both	65-69 yea	HIV/AIDS	Number	2003	593883	642003	552639
DALYs (Dis	Global	Both	65-69 yea	HIV/AIDS	Rate	2003	365.089	394.671	339.735
DALYs (Dis	Global	Both	50-69 yea	HIV/AIDS	Number	2003	8496881	9202260	7807593
DALYs (Dis	Global	Both	50-69 yea	HIV/AIDS	Rate	2003	969.022	1049.47	890.412
DALYs (Dis	Global	Both	50-54 yea	Sexually tr	Number	2003	64722.6	105644	42378.2
DALYs (Dis	Global	Both	50-54 yea	Sexually tr	Rate	2003	21.9333	35.8009	14.3612
DALYs (Dis	Global	Both	55-59 yea	Sexually tr	Number	2003	50086.2	77556.5	33733.5
DALYs (Dis	Global	Both	55-59 yea	Sexually tr	Rate	2003	22.0283	34.11	14.8363
DALYs (Dis	Global	Both	60-64 yea	Sexually tr	Number	2003	32427.2	53738.2	21390.1
DALYs (Dis	Global	Both	60-64 yea	Sexually tr	Rate	2003	16.9135	28.029	11.1567
DALYs (Dis	Global	Both	65-69 yea	Sexually tr	Number	2003	25518.5	41266.2	17119.2
DALYs (Dis	Global	Both	65-69 yea	Sexually tr	Rate	2003	15.6875	25.3684	10.524
DALYs (Dis	Global	Both	50-69 yea	Sexually tr	Number	2003	172755	282915	116539
DALYs (Dis	Global	Both	50-69 yea	Sexually tr	Rate	2003	19.7017	32.2649	13.2906
DALYs (Dis	Global	Both	50-54 yea	Syphilis	Number	2003	8553.12	11793.6	5449.22
DALYs (Dis	Global	Both	50-54 yea	Syphilis	Rate	2003	2.8985	3.99664	1.84664
DALYs (Dis	Global	Both	55-59 yea	Syphilis	Number	2003	8287.08	13002	4465.15
DALYs (Dis	Global	Both	55-59 yea	Syphilis	Rate	2003	3.64472	5.71837	1.96381
DALYs (Dis	Global	Both	60-64 yea	Syphilis	Number	2003	6236.84	8607.5	4221.94
DALYs (Dis	Global	Both	60-64 yea	Syphilis	Rate	2003	3.25304	4.48953	2.2021
DALYs (Dis	Global	Both	65-69 yea	Syphilis	Number	2003	4116.28	5415.74	3371.02
DALYs (Dis	Global	Both	65-69 yea	Syphilis	Rate	2003	2.53048	3.32932	2.07233
DALYs (Dis	Global	Both	50-69 yea	Syphilis	Number	2003	27193.3	37461.3	18111.6
DALYs (Dis	Global	Both	50-69 yea	Syphilis	Rate	2003	3.10125	4.27225	2.06553
DALYs (Dis	Global	Both	50-54 yea	Chlamydia	Number	2003	4934.66	6657.95	3720.27
DALYs (Dis	Global	Both	50-54 yea	Chlamydia	Rate	2003	1.67227	2.25626	1.26073
DALYs (Dis	Global	Both	55-59 yea	Chlamydia	Number	2003	3386.95	4114.11	2668.62
DALYs (Dis	Global	Both	55-59 yea	Chlamydia	Rate	2003	1.48961	1.80942	1.17368
DALYs (Dis	Global	Both	60-64 yea	Chlamydia	Number	2003	1632.18	1936.58	1298.09
DALYs (Dis	Global	Both	60-64 yea	Chlamydia	Rate	2003	0.85132	1.01009	0.67706
DALYs (Dis	Global	Both	65-69 yea	Chlamydia	Number	2003	986.952	1166.58	778.366
DALYs (Dis	Global	Both	65-69 yea	Chlamydia	Rate	2003	0.60673	0.71715	0.4785
DALYs (Dis	Global	Both	50-69 yea	Chlamydia	Number	2003	10940.7	13292.8	8962.59
DALYs (Dis	Global	Both	50-69 yea	Chlamydia	Rate	2003	1.24773	1.51597	1.02213
DALYs (Dis	Global	Both	50-54 yea	Gonococca	Number	2003	8510.08	9939.26	7027.22
DALYs (Dis	Global	Both	50-54 yea	Gonococca	Rate	2003	2.88391	3.36824	2.3814
DALYs (Dis	Global	Both	55-59 yea	Gonococca	Number	2003	7985.91	9693.92	6076.23
DALYs (Dis	Global	Both	55-59 yea	Gonococca	Rate	2003	3.51227	4.26347	2.67237
DALYs (Dis	Global	Both	60-64 yea	Gonococca	Number	2003	4658.89	5432.11	3785.01
DALYs (Dis	Global	Both	60-64 yea	Gonococca	Rate	2003	2.43	2.8333	1.9742
DALYs (Dis	Global	Both	65-69 yea	Gonococca	Number	2003	5539.22	6488.15	4453.89
DALYs (Dis	Global	Both	65-69 yea	Gonococca	Rate	2003	3.40523	3.98859	2.73803
DALYs (Dis	Global	Both	50-69 yea	Gonococca	Number	2003	26694.1	30146.8	21410.9
DALYs (Dis	Global	Both	50-69 yea	Gonococca	Rate	2003	3.04431	3.43807	2.4418
DALYs (Dis	Global	Both	50-54 yea	Trichomon	Number	2003	14704.3	34780.7	4726.16
DALYs (Dis	Global	Both	50-54 yea	Trichomon	Rate	2003	4.98303	11.7866	1.60161
DALYs (Dis	Global	Both	55-59 yea	Trichomon	Number	2003	9157.54	21311.7	3010.39
DALYs (Dis	Global	Both	55-59 yea	Trichomon	Rate	2003	4.02756	9.37305	1.324
DALYs (Dis	Global	Both	60-64 yea	Trichomon	Number	2003	6078.9	14122.4	2077.45
DALYs (Dis	Global	Both	60-64 yea	Trichomon	Rate	2003	3.17066	7.36601	1.08357
DALYs (Dis	Global	Both	65-69 yea	Trichomon	Number	2003	4478.34	10417.4	1591.52
DALYs (Dis	Global	Both	65-69 yea	Trichomon	Rate	2003	2.75306	6.40407	0.97839
DALYs (Dis	Global	Both	50-69 yea	Trichomon	Number	2003	34419.1	76012.5	12595
DALYs (Dis	Global	Both	50-69 yea	Trichomon	Rate	2003	3.92531	8.6688	1.43639
DALYs (Dis	Global	Both	50-54 yea	Genital her	Number	2003	16717.3	41417.9	5157.22
DALYs (Dis	Global	Both	50-54 yea	Genital her	Rate	2003	5.66519	14.0358	1.74769
DALYs (Dis	Global	Both	55-59 yea	Genital her	Number	2003	12496.5	30883.4	3806.7
DALYs (Dis	Global	Both	55-59 yea	Genital her	Rate	2003	5.49608	13.5828	1.67422
DALYs (Dis	Global	Both	60-64 yea	Genital her	Number	2003	9983.45	24300.4	3044.69
DALYs (Dis	Global	Both	60-64 yea	Genital her	Rate	2003	5.20721	12.6747	1.58806

DALYs (Dis	Global	Both	50-54 yea	Sexually tr	Rate	2012	20.0216	34.1136	12.4742
DALYs (Dis	Global	Both	55-59 yea	Sexually tr	Number	2012	59933.1	98672.9	38585.1
DALYs (Dis	Global	Both	55-59 yea	Sexually tr	Rate	2012	18.8708	31.0685	12.1491
DALYs (Dis	Global	Both	60-64 yea	Sexually tr	Number	2012	37602	65333	23170
DALYs (Dis	Global	Both	60-64 yea	Sexually tr	Rate	2012	14.4243	25.062	8.88811
DALYs (Dis	Global	Both	65-69 yea	Sexually tr	Number	2012	26743.7	44408.1	17058.7
DALYs (Dis	Global	Both	65-69 yea	Sexually tr	Rate	2012	14.0284	23.2943	8.94812
DALYs (Dis	Global	Both	50-69 yea	Sexually tr	Number	2012	196005	334958	123941
DALYs (Dis	Global	Both	50-69 yea	Sexually tr	Rate	2012	17.3892	29.7168	10.9958
DALYs (Dis	Global	Both	50-54 yea	Syphilis	Number	2012	7312.02	9642.13	4887.58
DALYs (Dis	Global	Both	50-54 yea	Syphilis	Rate	2012	2.04108	2.6915	1.36432
DALYs (Dis	Global	Both	55-59 yea	Syphilis	Number	2012	7662.4	11051	4390.34
DALYs (Dis	Global	Both	55-59 yea	Syphilis	Rate	2012	2.41261	3.47956	1.38236
DALYs (Dis	Global	Both	60-64 yea	Syphilis	Number	2012	5612.08	7392.46	3980.88
DALYs (Dis	Global	Both	60-64 yea	Syphilis	Rate	2012	2.15282	2.83578	1.52708
DALYs (Dis	Global	Both	65-69 yea	Syphilis	Number	2012	3520.94	4595.35	2893.44
DALYs (Dis	Global	Both	65-69 yea	Syphilis	Rate	2012	1.84691	2.41049	1.51775
DALYs (Dis	Global	Both	50-69 yea	Syphilis	Number	2012	24107.4	30587.9	16627.9
DALYs (Dis	Global	Both	50-69 yea	Syphilis	Rate	2012	2.13877	2.7137	1.4752
DALYs (Dis	Global	Both	50-54 yea	Chlamydia	Number	2012	5326.48	7220.09	3915.5
DALYs (Dis	Global	Both	50-54 yea	Chlamydia	Rate	2012	1.48683	2.01541	1.09297
DALYs (Dis	Global	Both	55-59 yea	Chlamydia	Number	2012	3867.42	4688.13	3126.37
DALYs (Dis	Global	Both	55-59 yea	Chlamydia	Rate	2012	1.21771	1.47612	0.98438
DALYs (Dis	Global	Both	60-64 yea	Chlamydia	Number	2012	1739.07	2051.54	1405.73
DALYs (Dis	Global	Both	60-64 yea	Chlamydia	Rate	2012	0.66711	0.78698	0.53924
DALYs (Dis	Global	Both	65-69 yea	Chlamydia	Number	2012	987.831	1182.94	767.394
DALYs (Dis	Global	Both	65-69 yea	Chlamydia	Rate	2012	0.51817	0.62051	0.40254
DALYs (Dis	Global	Both	50-69 yea	Chlamydia	Number	2012	11920.8	14461.2	9793.65
DALYs (Dis	Global	Both	50-69 yea	Chlamydia	Rate	2012	1.05759	1.28297	0.86887
DALYs (Dis	Global	Both	50-54 yea	Gonococca	Number	2012	8661.21	10071.6	7121.58
DALYs (Dis	Global	Both	50-54 yea	Gonococca	Rate	2012	2.41769	2.81138	1.98792
DALYs (Dis	Global	Both	55-59 yea	Gonococca	Number	2012	8627.11	10199.6	6559.46
DALYs (Dis	Global	Both	55-59 yea	Gonococca	Rate	2012	2.71636	3.21149	2.06533
DALYs (Dis	Global	Both	60-64 yea	Gonococca	Number	2012	4837.66	5619.14	3936.78
DALYs (Dis	Global	Both	60-64 yea	Gonococca	Rate	2012	1.85575	2.15552	1.51017
DALYs (Dis	Global	Both	65-69 yea	Gonococca	Number	2012	5468.83	6517	4298.89
DALYs (Dis	Global	Both	65-69 yea	Gonococca	Rate	2012	2.86868	3.41849	2.25498
DALYs (Dis	Global	Both	50-69 yea	Gonococca	Number	2012	27594.8	30702.1	22139.4
DALYs (Dis	Global	Both	50-69 yea	Gonococca	Rate	2012	2.44816	2.72383	1.96417
DALYs (Dis	Global	Both	50-54 yea	Trichomon	Number	2012	18141.3	42696.8	5809.9
DALYs (Dis	Global	Both	50-54 yea	Trichomon	Rate	2012	5.06395	11.9184	1.62177
DALYs (Dis	Global	Both	55-59 yea	Trichomon	Number	2012	12785.2	29957.5	4166.81
DALYs (Dis	Global	Both	55-59 yea	Trichomon	Rate	2012	4.02558	9.43254	1.31198
DALYs (Dis	Global	Both	60-64 yea	Trichomon	Number	2012	8138.84	19017.7	2802.91
DALYs (Dis	Global	Both	60-64 yea	Trichomon	Rate	2012	3.12209	7.29528	1.07521
DALYs (Dis	Global	Both	65-69 yea	Trichomon	Number	2012	5168.03	11908.5	1844.36
DALYs (Dis	Global	Both	65-69 yea	Trichomon	Rate	2012	2.71089	6.2466	0.96746
DALYs (Dis	Global	Both	50-69 yea	Trichomon	Number	2012	44233.3	97939.5	16190.5
DALYs (Dis	Global	Both	50-69 yea	Trichomon	Rate	2012	3.92429	8.689	1.43639
DALYs (Dis	Global	Both	50-54 yea	Genital he	Number	2012	20354.3	50803.5	6300.42
DALYs (Dis	Global	Both	50-54 yea	Genital he	Rate	2012	5.6817	14.1813	1.7587
DALYs (Dis	Global	Both	55-59 yea	Genital he	Number	2012	17199.7	42580.4	5231.34
DALYs (Dis	Global	Both	55-59 yea	Genital he	Rate	2012	5.41555	13.407	1.64716
DALYs (Dis	Global	Both	60-64 yea	Genital he	Number	2012	13269.8	32527.5	4035.53
DALYs (Dis	Global	Both	60-64 yea	Genital he	Rate	2012	5.09033	12.4777	1.54805
DALYs (Dis	Global	Both	65-69 yea	Genital he	Number	2012	9113.98	22175	2754.74
DALYs (Dis	Global	Both	65-69 yea	Genital he	Rate	2012	4.78074	11.6319	1.445
DALYs (Dis	Global	Both	50-69 yea	Genital he	Number	2012	59937.7	147729	18282.7
DALYs (Dis	Global	Both	50-69 yea	Genital he	Rate	2012	5.31756	13.1063	1.62201
DALYs (Dis	Global	Both	50-54 yea	Other sexu	Number	2012	11930.9	15247.9	9162.71
DALYs (Dis	Global	Both	50-54 yea	Other sexu	Rate	2012	3.3304	4.25628	2.55768
DALYs (Dis	Global	Both	55-59 yea	Other sexu	Number	2012	9791.34	11788.6	7782.03
DALYs (Dis	Global	Both	55-59 yea	Other sexu	Rate	2012	3.08294	3.71181	2.45028
DALYs (Dis	Global	Both	60-64 yea	Other sexu	Number	2012	4004.57	4700.62	3216.55
DALYs (Dis	Global	Both	60-64 yea	Other sexu	Rate	2012	1.53617	1.80318	1.23388
DALYs (Dis	Global	Both	65-69 yea	Other sexu	Number	2012	2484.06	2967.99	1976.05
DALYs (Dis	Global	Both	65-69 yea	Other sexu	Rate	2012	1.30302	1.55686	1.03654
DALYs (Dis	Global	Both	50-69 yea	Other sexu	Number	2012	28210.9	33711.3	23079.1
DALYs (Dis	Global	Both	50-69 yea	Other sexu	Rate	2012	2.50282	2.9908	2.04754
DALYs (Dis	Global	Both	50-54 yea	HIV/AIDS	Number	2005	4915341	5259218	4564343
DALYs (Dis	Global	Both	50-54 yea	HIV/AIDS	Rate	2005	1551.86	1660.43	1441.04
DALYs (Dis	Global	Both	55-59 yea	HIV/AIDS	Number	2005	2290777	2451826	2130726
DALYs (Dis	Global	Both	55-59 yea	HIV/AIDS	Rate	2005	917.836	982.363	853.709
DALYs (Dis	Global	Both	60-64 yea	HIV/AIDS	Number	2005	1155679	1250208	1064692
DALYs (Dis	Global	Both	60-64 yea	HIV/AIDS	Rate	2005	592.623	641.096	545.965
DALYs (Dis	Global	Both	65-69 yea	HIV/AIDS	Number	2005	602602	655959	554587
DALYs (Dis	Global	Both	65-69 yea	HIV/AIDS	Rate	2005	357.443	389.093	328.962
DALYs (Dis	Global	Both	50-69 yea	HIV/AIDS	Number	2005	8964399	9578326	8378022
DALYs (Dis	Global	Both	50-69 yea	HIV/AIDS	Rate	2005	963.995	1030.01	900.939
DALYs (Dis	Global	Both	50-54 yea	Sexually tr	Number	2008	68661.6	115367	43149
DALYs (Dis	Global	Both	50-54 yea	Sexually tr	Rate	2008	20.4502	34.3611	12.8515
DALYs (Dis	Global	Both	55-59 yea	Sexually tr	Number	2008	55958.7	89334.8	36599.8
DALYs (Dis	Global	Both	55-59 yea	Sexually tr	Rate	2008	19.8432	31.6785	12.9784
DALYs (Dis	Global	Both	60-64 yea	Sexually tr	Number	2008	33014.2	55734.8	21022.9
DALYs (Dis	Global	Both	60-64 yea	Sexually tr	Rate	2008	15.203	25.6658	9.68101
DALYs (Dis	Global	Both	65-69 yea	Sexually tr	Number	2008	25100.7	41354.8	16099.8
DALYs (Dis	Global	Both	65-69 yea	Sexually tr	Rate	2008	14.3951	23.7167	9.23312
DALYs (Dis	Global	Both	50-69 yea	Sexually tr	Number	2008	182735	302484	118284
DALYs (Dis	Global	Both	50-69 yea	Sexually tr	Rate	2008	18.1055	29.9703	11.7197
DALYs (Dis	Global	Both	50-54 yea	Syphilis	Number	2008	7572.59	10079.4	5022.46
DALYs (Dis	Global	Both	50-54 yea	Syphilis	Rate	2008	2.25542	3.00204	1.49589
DALYs (Dis	Global	Both	55-59 yea	Syphilis	Number	2008	7672.99	11148.2	4323.31
DALYs (Dis	Global	Both	55-59 yea	Syphilis	Rate	2008	2.72087	3.95321	1.53306
DALYs (Dis	Global	Both	60-64 yea	Syphilis	Number	2008	5387.27	7150.13	3700.83
DALYs (Dis	Global	Both	60-64 yea	Syphilis	Rate	2008	2.48083	3.29263	1.70423
DALYs (Dis	Global	Both	65-69 yea	Syphilis	Number	2008	3424.88	4624.49	2782.3
DALYs (Dis	Global	Both	65-69 yea	Syphilis	Rate	2008	1.96415	2.65212	1.59563
DALYs (Dis	Global	Both	50-69 yea	Syphilis	Number	2008	24057.7	31036.1	16497.1
DALYs (Dis	Global	Both	50-69 yea	Syphilis	Rate	2008	2.38365	3.07507	1.63454
DALYs (Dis	Global	Both	50-54 yea	Chlamydia	Number	2008	5159.64	6986.37	3805.39
DALYs (Dis	Global	Both	50-54 yea	Chlamydia	Rate	2008	1.53675	2.08083	1.1334
DALYs (Dis	Global	Both	55-59 yea	Chlamydia	Number	2008	3746.32	4480.75	2992.7
DALYs (Dis	Global	Both	55-59 yea	Chlamydia	Rate	2008	1.32846	1.5889	1.06122
DALYs (Dis	Global	Both	60-64 yea	Chlamydia	Number	2008	1587.33	1864.01	1253.23
DALYs (Dis	Global	Both	60-64 yea	Chlamydia	Rate	2008	0.73096	0.85838	0.57711
DALYs (Dis	Global	Both	65-69 yea	Chlamydia	Number	2008	942.13	1114.37	749.616
DALYs (Dis	Global	Both	65-69 yea	Chlamydia	Rate	2008	0.54031	0.63909	0.4299
DALYs (Dis	Global	Both	50-69 yea	Chlamydia	Number	2008	11435.4	13838.2	9219.82
DALYs (Dis	Global	Both	50-69 yea	Chlamydia	Rate	2008	1.13303	1.3711	0.9135
DALYs (Dis	Global	Both	50-54 yea	Gonococca	Number	2008	8490.96	9881.83	7002.56
DALYs (Dis	Global	Both	50-54 yea	Gonococca	Rate	2008	2.52895	2.94321	2.08565
DALYs (Dis	Global	Both	55-59 yea	Gonococca	Number	2008	8558.6	10031.7	6389.46
DALYs (Dis	Global	Both	55-59 yea	Gonococca	Rate	2008	3.03492	3.55728	2.26573
DALYs (Dis	Global	Both	60-64 yea	Gonococca	Number	2008	4453.35	5164.11	3605.96
DALYs (Dis	Global	Both	60-64 yea	Gonococca	Rate	2008	2.05076	2.37807	1.66054
DALYs (Dis	Global	Both	65-69 yea	Gonococca	Number	2008	5248.66	6158.48	4202.53
DALYs (Dis	Global	Both	65-69 yea	Gonococca	Rate	2008	3.01008	3.53185	2.41013
DALYs (Dis	Global	Both	50-69 yea	Gonococca	Number	2008	26751.6	29855	20955.1
DALYs (Dis	Global	Both	50-69 yea	Gonococca	Rate	2008	2.65056	2.95805	2.07624
DALYs (Dis	Global	Both	50-54 yea	Trichomon	Number	2008	16879.7	39867.6	5392.81
DALYs (Dis	Global	Both	50-54 yea	Trichomon	Rate	2008	5.02746	11.8742	1.6062
DALYs (Dis	Global	Both	55-59 yea	Trichomon	Number	2008	11248.3	26108.4	3644.16
DALYs (Dis	Global	Both	55-59 yea	Trichomon	Rate	2008	3.98871	9.25815	1.29224
DALYs (Dis	Global	Both	60-64 yea	Trichomon	Number	2008	6741	15735.6	2324.99
DALYs (Dis	Global	Both	60-64 yea	Trichomon	Rate	2008	3.10422	7.24623	1.07065
DALYs (Dis	Global	Both	65-69 yea	Trichomon	Number	2008	4719	10916.5	1695.71
DALYs (Dis	Global	Both	65-69 yea	Trichomon	Rate	2008	2.70632	6.26053	0.97248
DALYs (Dis	Global	Both	50-69 yea	Trichomon	Number	2008	39588	87256.2	14588.4
DALYs (Dis	Global	Both	50-69 yea	Trichomon	Rate	2008	3.9224	8.64539	1.44543
DALYs (Dis	Global	Both	50-54 yea	Genital he	Number	2008	18891	46841.2	5835.03
DALYs (Dis	Global	Both	50-54 yea	Genital he	Rate	2008	5.6265	13.9512	1.73791
DALYs (Dis	Global	Both	55-59 yea	Genital he	Number	2008	15221	37459.1	4605.36
DALYs (Dis	Global	Both	55-59 yea	Genital he	Rate	2008	5.39743	13.2832	1.63308
DALYs (Dis	Global	Both	60-64 yea	Genital he	Number	2008	11120.7	27143.4	3393.49
DALYs (Dis	Global	Both	60-64 yea	Genital he	Rate	2008	5.12108	12.4995	1.5627
DALYs (Dis	Global	Both	65-69 yea	Genital he	Number	2008	8388.45	20403	2539.63
DALYs (Dis	Global	Both	65-69 yea	Genital he	Rate	2008	4.81073	11.701	1.45646
DALYs (Dis	Global	Both	50-69 yea	Genital he	Number	2008	53621.2	131739	16276.2
DALYs (Dis	Global	Both	50-69 yea	Genital he	Rate	2008	5.31281	13.0528	1.61266
DALYs (Dis	Global	Both	50-54 yea	Other sexu	Number	2008	11667.7	14939.1	8923.17
DALYs (Dis	Global	Both	50-54 yea	Other sexu	Rate	2008	3.47511	4.44948	2.65768
DALYs (Dis	Global	Both	55-59 yea	Other sexu	Number	2008	9511.43	11518.1	7467.02
DALYs (Dis	Global	Both	55-59 yea	Other sexu	Rate	2008	3.37279	4.08436	2.64784
DALYs (Dis	Global	Both	60-64 yea	Other sexu	Number	2008	3724.51	4375.73	2987.93
DALYs (Dis	Global	Both	60-64 yea	Other sexu	Rate	2008	1.71513	2.01502	1.37594
DALYs (Dis	Global	Both	65-69 yea	Other sexu	Number	2008	2377.63	2805.75	1932.48
DALYs (Dis	Global	Both	65-69 yea	Other sexu	Rate	2008	1.36356	1.60908	1.10826
DALYs (Dis	Global	Both	50-69 yea	Other sexu	Number	2008	27281.2	32479.1	22264.5
DALYs (Dis	Global	Both	50-69 yea	Other sexu	Rate	2008	2.70304	3.21805	2.20598
DALYs (Dis	Global	Both	50-54 yea	HIV/AIDS	Number	2011	3788243	4362027	3400836
DALYs (Dis	Global	Both	50-54 yea	HIV/AIDS	Rate	2011	1084.45	1248.71	973.552
DALYs (Dis	Global	Both	55-59 yea	HIV/AIDS	Number	2011	1903757	2238087	1701438
DALYs (Dis	Global	Both	55-59 yea	HIV/AIDS	Rate	2011	612.154	719.657	547.098
DALYs (Dis	Global	Both	60-64 yea	HIV/AIDS	Number	2011	936538	1111323	832707
DALYs (Dis	Global	Both	60-64 yea	HIV/AIDS	Rate	2011	372.274	441.751	331.001
DALYs (Dis	Global	Both	65-69 yea	HIV/AIDS	Number	2011	448804	535886	397490
DALYs (Dis	Global	Both	65-69 yea	HIV/AIDS	Rate	2011	246.295	294.084	218.136
DALYs (Dis	Global	Both	50-69 yea	HIV/AIDS	Number	2011	7077343	8214844	6392067
DALYs (Dis	Global	Both	50-69 yea	HIV/AIDS	Rate	2011	646.859	750.824	584.225
DALYs (Dis	Global	Both	50-54 yea	Sexually tr	Number	2014	78412.2	134290	49467.5
DALYs (Dis	Global	Both	50-54 yea	Sexually tr	Rate	2014	20.3548	34.8599	12.8411
DALYs (Dis	Global	Both	55-59 yea	Sexually tr	Number	2014	62429.5	103880	40024.9
DALYs (Dis	Global	Both	55-59 yea	Sexually tr	Rate	2014	19.1419	31.8513	12.2723
DALYs (Dis	Global	Both	60-64 yea	Sexually tr	Number	2014	40097.8	70250.2	24353.9
DALYs (Dis	Global	Both	60-64 yea	Sexually tr	Rate	2014	14.359	25.1566	8.72113
DALYs (Dis	Global	Both	65-69 yea	Sexually tr	Number	2014	28328.6	48509.2	17336
DALYs (Dis	Global	Both	65-69 yea	Sexually tr	Rate	2014	13.4109	22.9646	8.20697
DALYs (Dis	Global	Both	50-69 yea	Sexually tr	Number	2014	209268	356393	132590
DALYs (Dis	Global	Both	50-69 yea	Sexually tr	Rate	2014	17.4121	29.6535	11.0321
DALYs (Dis	Global	Both	50-54 yea	Syphilis	Number	2014	7673.23	10005.2	5215.96
DALYs (Dis	Global	Both	50-54 yea	Syphilis	Rate	2014	1.99187	2.59723	1.35399
DALYs (Dis	Global	Both	55-59 yea	Syphilis	Number	2014	7601.98	10696.2	4390.01
DALYs (Dis	Global	Both	55-59 yea	Syphilis	Rate	2014	2.33089	3.27961	1.34605
DALYs (Dis	Global	Both	60-64 yea	Syphilis	Number	2014	5743.67	7509.67	4075.48
DALYs (Dis	Global	Both	60-64 yea	Syphilis	Rate	2014	2.0568	2.68921	1.45943
DALYs (Dis	Global	Both	65-69 yea	Syphilis	Number	2014	3613.29	4682.91	2956.22
DALYs (Dis	Global	Both	65-69 yea	Syphilis	Rate	2014	1.71055	2.21692	1.3995
DALYs (Dis	Global	Both	50-69 yea	Syphilis	Number	2014	24632.2	31060.6	17255.3
DALYs (Dis	Global	Both	50-69 yea	Syphilis	Rate	2014	2.04951	2.58439	1.43572
DALYs (Dis	Global	Both	50-54 yea	Chlamydia	Number	2014	6015.27	8070.82	4408.61
DALYs (Dis	Global	Both	50-54 yea	Chlamydia	Rate	2014	1.56149	2.09508	1.14442
DALYs (Dis	Global	Both	55-59 yea	Chlamydia	Number	2014	4097.9	5008	3199.59
DALYs (Dis	Global	Both	55-59 yea	Chlamydia	Rate	2014	1.25648	1.53553	0.98104
DALYs (Dis	Global	Both	60-64 yea	Chlamydia	Number	2014	1813.7	2134.09	1449.9
DALYs (Dis	Global	Both	60-64 yea	Chlamydia	Rate	2014	0.64949	0.76422	0.51921
DALYs (Dis	Global	Both	65-69 yea	Chlamydia	Number	2014	966.862	1134.28	773.542
DALYs (Dis	Global	Both	65-69 yea	Chlamydia	Rate	2014	0.45772	0.53698	0.3662
DALYs (Dis	Global	Both	50-69 yea	Chlamydia	Number	2014	12893.7	15715.4	10473.3
DALYs (Dis	Global	Both	50-69 yea	Chlamydia	Rate	2014	1.07282	1.30759	0.87143
DALYs (Dis	Global	Both	50-54 yea	Gonococca	Number	2014	10008.9	11998.4	7864.88
DALYs (Dis	Global	Both	50-54 yea	Gonococca	Rate	2014	2.59818	3.11462	2.04162
DALYs (Dis	Global	Both	55-59 yea	Gonococca	Number	2014	9210.85	11099	6798.88
DALYs (Dis	Global	Both	55-59 yea	Gonococca	Rate	2014	2.82419	3.40312	2.08465
DALYs (Dis	Global	Both	60-64 yea	Gonococca	Number	2014	5022.98	5889.54	4071.09
DALYs (Dis	Global	Both	60-64 yea	Gonococca	Rate	2014	1.79872	2.10904	1.45785
DALYs (Dis	Global	Both	65-69 yea	Gonococca	Number	2014	5352.04	6271.55	4322.81
DALYs (Dis	Global	Both	65-69 yea	Gonococca	Rate	2014	2.53369	2.96899	2.04645
DALYs (Dis	Global	Both	50-69 yea	Gonococca	Number	2014	29594.8	33473.2	23337.5
DALYs (Dis	Global	Both	50-69 yea	Gonococca	Rate	2014	2.46242	2.78513	1.94179
DALYs (Dis	Global	Both	50-54 yea	Trichomon	Number	2014	19445.9	46009.5	6213.92
DALYs (Dis	Global	Both	50-54 yea	Trichomon	Rate	2014	5.04789	11.9435	1.61305
DALYs (Dis	Global	Both	55-59 yea	Trichomon	Number	2014	13280.6	30981.5	4312.5
DALYs (Dis	Global	Both	55-59 yea	Trichomon	Rate	2014	4.07206	9.49942	1.32228
DALYs (Dis	Global	Both	60-64 yea	Trichomon	Number	2014	8960.26	21131.5	3092.33
DALYs (Dis	Global	Both	60-64 yea	Trichomon	Rate	2014	3.20866	7.56717	1.10736
DALYs (Dis	Global	Both	65-69 yea	Trichomon	Number	2014	5823.44	13494.3	2097.42
DALYs (Dis	Global	Both	65-69 yea	Trichomon	Rate	2014	2.75685	6.38827	0.99293
DALYs (Dis	Global	Both	50-69 yea	Trichomon	Number	2014	47510.2	105706	17467
DALYs (Dis	Global	Both	50-69 yea	Trichomon	Rate	2014	3.95307	8.79522	1.45334
DALYs (Dis	Global	Both	50-54 yea	Genital he	Number	2014	22001.2	54813.4	6853.24
DALYs (Dis	Global	Both	50-54 yea	Genital he	Rate	2014	5.71122	14.2288	1.77901
DALYs (Dis	Global	Both	55-59 yea	Genital he	Number	2014	18000	44408.9	5495.6
DALYs (Dis	Global	Both	55-59 yea	Genital he	Rate	2014	5.51908	13.6165	1.68504
DALYs (Dis	Global	Both	60-64 yea	Genital he	Number	2014	14395.6	35260	4382.99
DALYs (Dis	Global	Both	60-64 yea	Genital he	Rate	2014	5.15504	12.6266	1.56954
DALYs (Dis	Global	Both	65-69 yea	Genital he	Number	2014	10150.3	24659.3	3067.58
DALYs (Dis	Global	Both	65-69 yea	Genital he	Rate	2014	4.80522	11.6739	1.45221
DALYs (Dis	Global	Both	50-69 yea	Genital he	Number	2014	64547	159519	19810.8
DALYs (Dis	Global	Both	50-69 yea	Genital he	Rate	2014	5.37061	13.2727	1.64835
DALYs (Dis	Global	Both	50-54 yea	Other sexu	Number	2014	13267.7	17127.6	10211.7
DALYs (Dis	Global	Both	50-54 yea	Other sexu	Rate	2014	3.44412	4.44611	2.65081
DALYs (Dis	Global	Both	55-59 yea	Other sexu	Number	2014	10238.2	12402.1	7984.69
DALYs (Dis	Global	Both	55-59 yea	Other sexu	Rate	2014	3.13918	3.80268	2.44823
DALYs (Dis	Global	Both	60-64 yea	Other sexu	Number	2014	4161.62	4911.95	3240.94
DALYs (Dis	Global	Both	60-64 yea	Other sexu	Rate	2014	1.49027	1.75897	1.16058
DALYs (Dis	Global	Both	65-69 yea	Other sexu	Number	2014	2422.67	2838.44	1960.09
DALYs (Dis	Global	Both	65-69 yea	Other sexu	Rate	2014	1.14691	1.34374	0.92792
DALYs (Dis	Global	Both	50-69 yea	Other sexu	Number	2014	30090.1	35944.4	24398.2
DALYs (Dis	Global	Both	50-69 yea	Other sexu	Rate	2014	2.50364	2.99074	2.03005
DALYs (Dis	Global	Both	50-54 yea	Sexually tr	Number	2011	70600.3	119384	43975.1
DALYs (Dis	Global	Both	50-54 yea	Sexually tr	Rate	2011	20.2106	34.1758	12.5887
DALYs (Dis	Global	Both	55-59 yea	Sexually tr	Number	2011	58903.3	96310.6	37819
DALYs (Dis	Global	Both	55-59 yea	Sexually tr	Rate	2011	18.9404	30.9687	12.1607
DALYs (Dis	Global	Both	60-64 yea	Sexually tr	Number	2011	36151.7	62959.3	22328.3
DALYs (Dis	Global	Both	60-64 yea	Sexually tr	Rate	2011	14.3703	25.0263	8.87548
DALYs (Dis	Global	Both	65-69 yea	Sexually tr	Number	2011	25797	42619.3	16462.4
DALYs (Dis	Global	Both	65-69 yea	Sexually tr	Rate	2011	14.157	23.3887	9.03426
DALYs (Dis	Global	Both	50-69 yea	Sexually tr	Number	2011	191452	323960	121297
DALYs (Dis	Global	Both	50-69 yea	Sexually tr	Rate	2011	17.4984	29.6094	11.0863
DALYs (Dis	Global	Both	50-54 yea	Syphilis	Number	2011	7355.2	9714.84	4961.71
DALYs (Dis	Global	Both	50-54 yea	Syphilis	Rate	2011	2.10556	2.78105	1.42038
DALYs (Dis	Global	Both	55-59 yea	Syphilis	Number	2011	7685.09	10793.3	4412.72
DALYs (Dis	Global	Both	55-59 yea	Syphilis	Rate	2011	2.47114	3.47059	1.41891
DALYs (Dis	Global	Both	60-64 yea	Syphilis	Number	2011	5518.75	7257.15	3902.17
DALYs (Dis	Global	Both	60-64 yea	Syphilis	Rate	2011	2.1937	2.88472	1.55111
DALYs (Dis	Global	Both	65-69 yea	Syphilis	Number	2011	3443.81	4582.65	2801.92
DALYs (Dis	Global	Both	65-69 yea	Syphilis	Rate	2011	1.8899	2.51487	1.53764
DALYs (Dis	Global	Both	50-69 yea	Syphilis	Number	2011	24002.8	30716.6	16484.4
DALYs (Dis	Global	Both	50-69 yea	Syphilis	Rate	2011	2.19382	2.80745	1.50665
DALYs (Dis	Global	Both	50-54 yea	Chlamydia	Number	2011	5261.84	7068.63	3916.75
DALYs (Dis	Global	Both	50-54 yea	Chlamydia	Rate	2011	1.5063	2.02353	1.12124
DALYs (Dis	Global	Both	55-59 yea	Chlamydia	Number	2011	3819.24	4621.11	3037.24
DALYs (Dis	Global	Both	55-59 yea	Chlamydia	Rate	2011	1.22808	1.48592	0.97663
DALYs (Dis	Global	Both	60-64 yea	Chlamydia	Number	2011	1662.71	1944.26	1360.56
DALYs (Dis	Global	Both	60-64 yea	Chlamydia	Rate	2011	0.66093	0.77284	0.54082
DALYs (Dis	Global	Both	65-69 yea	Chlamydia	Number	2011	964.3	1148.97	752.212
DALYs (Dis	Global	Both	65-69 yea	Chlamydia	Rate	2011	0.52919	0.63053	0.4128
DALYs (Dis	Global	Both	50-69 yea	Chlamydia	Number	2011	11708.1	14180.3	9536.12
DALYs (Dis	Global	Both	50-69 yea	Chlamydia	Rate	2011	1.0701	1.29606	0.87159
DALYs (Dis	Global	Both	50-54 yea	Gonococca	Number	2011	8702.98	10124.9	7109.1
DALYs (Dis	Global	Both	50-54 yea	Gonococca	Rate	2011	2.49139	2.89844	2.03511
DALYs (Dis	Global	Both	55-59 yea	Gonococca	Number	2011	8547.75	10174.4	6597.29
DALYs (Dis	Global	Both	55-59 yea	Gonococca	Rate	2011	2.74853	3.27159	2.12136
DALYs (Dis	Global	Both	60-64 yea	Gonococca	Number	2011	4645.04	5396.46	3786.38
DALYs (Dis	Global	Both	60-64 yea	Gonococca	Rate	2011	1.8464	2.14509	1.50508
DALYs (Dis	Global	Both	65-69 yea	Gonococca	Number	2011	5347.24	6308.09	4261.16
DALYs (Dis	Global	Both	65-69 yea	Gonococca	Rate	2011	2.93447	3.46176	2.33845
DALYs (Dis	Global	Both	50-69 yea	Gonococca	Number	2011	27243	30477	21816.9
DALYs (Dis	Global	Both	50-69 yea	Gonococca	Rate	2011	2.48997	2.78555	1.99403
DALYs (Dis	Global	Both	50-54 yea	Trichomon	Number	2011	17693.6	41644.2	5646.79
DALYs (Dis	Global	Both	50-54 yea	Trichomon	Rate	2011	5.06513	11.9214	1.6165
DALYs (Dis	Global	Both	55-59 yea	Trichomon	Number	2011	12452.5	28934.9	4056.5
DALYs (Dis	Global	Both	55-59 yea	Trichomon	Rate	2011	4.00411	9.30402	1.30437
DALYs (Dis	Global	Both	60-64 yea	Trichomon	Number	2011	7755.61	18080.8	2691.1
DALYs (Dis	Global	Both	60-64 yea	Trichomon	Rate	2011	3.08285	7.18712	1.06971
DALYs (Dis	Global	Both	65-69 yea	Trichomon	Number	2011	4913.2	11374	1747.05
DALYs (Dis	Global	Both	65-69 yea	Trichomon	Rate	2011	2.69627	6.24186	0.95875
DALYs (Dis	Global	Both	50-69 yea	Trichomon	Number	2011	42814.9	94608.2	15689.3
DALYs (Dis	Global	Both	50-69 yea	Trichomon	Rate	2011	3.91322	8.64705	1.43398
DALYs (Dis	Global	Both	50-54 yea	Genital he	Number	2011	19751.4	49250.5	6128.85
DALYs (Dis	Global	Both	50-54 yea	Genital he	Rate	2011	5.65422	14.0989	1.7545
DALYs (Dis	Global	Both	55-59 yea	Genital he	Number	2011	16710.5	41268.5	5052.05
DALYs (Dis	Global	Both	55-59 yea	Genital he	Rate	2011	5.37325	13.2699	1.62449
DALYs (Dis	Global	Both	60-64 yea	Genital he	Number	2011	12735.8	31403.5	3866.2
DALYs (Dis	Global	Both	60-64 yea	Genital he	Rate	2011	5.06247	12.4829	1.53682
DALYs (Dis	Global	Both	65-69 yea	Genital he	Number	2011	8696.66	21244.8	2634.87
DALYs (Dis	Global	Both	65-69 yea	Genital he	Rate	2011	4.77257	11.6588	1.44597
DALYs (Dis	Global	Both	50-69 yea	Genital he	Number	2011	57894.3	142373	17648.1
DALYs (Dis	Global	Both	50-69 yea	Genital he	Rate	2011	5.29146	13.0126	1.61301
DALYs (Dis	Global	Both	50-54 yea	Other sexu	Number	2011	11835.2	15112.1	9034.66
DALYs (Dis	Global	Both	50-54 yea	Other sexu	Rate	2011	3.38803	4.3261	2.58634
DALYs (Dis	Global	Both	55-59 yea	Other sexu	Number	2011	9688.22	11691	7697.67
DALYs (Dis	Global	Both	55-59 yea	Other sexu	Rate	2011	3.11525	3.75926	2.47519
DALYs (Dis	Global	Both	60-64 yea	Other sexu	Number	2011	3833.79	4501.37	3077.81
DALYs (Dis	Global	Both	60-64 yea	Other sexu	Rate	2011	1.52393	1.78929	1.22343
DALYs (Dis	Global	Both	65-69 yea	Other sexu	Number	2011	2431.83	2895.25	1960.94
DALYs (Dis	Global	Both	65-69 yea	Other sexu	Rate	2011	1.33455	1.58886	1.07613
DALYs (Dis	Global	Both	50-69 yea	Other sexu	Number	2011	27789	33184.2	22866
DALYs (Dis	Global	Both	50-69 yea	Other sexu	Rate	2011	2.53987	3.03299	2.08992
DALYs (Dis	Global	Both	50-54 yea	HIV/AIDS	Number	2013	3567044	4111355	3226262
DALYs (Dis	Global	Both	50-54 yea	HIV/AIDS	Rate	2013	962.036	1108.84	870.126
DALYs (Dis	Global	Both	55-59 yea	HIV/AIDS	Number	2013	1834815	2137065	1649970
DALYs (Dis	Global	Both	55-59 yea	HIV/AIDS	Rate	2013	569.082	662.828	511.751
DALYs (Dis	Global	Both	60-64 yea	HIV/AIDS	Number	2013	931694	1089333	836523
DALYs (Dis	Global	Both	60-64 yea	HIV/AIDS	Rate	2013	344.524	402.816	309.331
DALYs (Dis	Global	Both	65-69 yea	HIV/AIDS	Number	2013	446769	522991	400896
DALYs (Dis	Global	Both	65-69 yea	HIV/AIDS	Rate	2013	223.548	261.687	200.595
DALYs (Dis	Global	Both	50-69 yea	HIV/AIDS	Number	2013	6780322	7829777	6151327
DALYs (Dis	Global	Both	50-69 yea	HIV/AIDS	Rate	2013	582.762	672.962	528.701
DALYs (Dis	Global	Both	50-54 yea	Sexually tr	Number	2016	84326.4	144420	53000.7
DALYs (Dis	Global	Both	50-54 yea	Sexually tr	Rate	2016	20.4709	35.0591	12.8663
DALYs (Dis	Global	Both	55-59 yea	Sexually tr	Number	2016	65258	107047	42126
DALYs (Dis	Global	Both	55-59 yea	Sexually tr	Rate	2016	19.4217	31.8586	12.5373
DALYs (Dis	Global	Both	60-64 yea	Sexually tr	Number	2016	42816.8	75293.5	25958.6
DALYs (Dis	Global	Both	60-64 yea	Sexually tr	Rate	2016	14.3555	25.2442	8.70334
DALYs (Dis	Global	Both	65-69 yea	Sexually tr	Number	2016	31194.6	53493	19284.3
DALYs (Dis	Global	Both	65-69 yea	Sexually tr	Rate	2016	13.4169	23.0074	8.29423
DALYs (Dis	Global	Both	50-69 yea	Sexually tr	Number	2016	223596	374019	141921
DALYs (Dis	Global	Both	50-69 yea	Sexually tr	Rate	2016	17.4861	29.2499	11.0989
DALYs (Dis	Global	Both	50-54 yea	Syphilis	Number	2016	8028.86	10359.5	5502.41
DALYs (Dis	Global	Both	50-54 yea	Syphilis	Rate	2016	1.94907	2.51485	1.33575
DALYs (Dis	Global	Both	55-59 yea	Syphilis	Number	2016	7636.91	10623	4470.73
DALYs (Dis	Global	Both	55-59 yea	Syphilis	Rate	2016	2.27285	3.16155	1.33055
DALYs (Dis	Global	Both	60-64 yea	Syphilis	Number	2016	5951.29	7806.72	4253.96
DALYs (Dis	Global	Both	60-64 yea	Syphilis	Rate	2016	1.99534	2.61742	1.42626
DALYs (Dis	Global	Both	65-69 yea	Syphilis	Number	2016	3879.95	5035.88	3181.91
DALYs (Dis	Global	Both	65-69 yea	Syphilis	Rate	2016	1.66878	2.16594	1.36855
DALYs (Dis	Global	Both	50-69 yea	Syphilis	Number	2016	25497	31837.9	17944.6
DALYs (Dis	Global	Both	50-69 yea	Syphilis	Rate	2016	1.99398	2.48986	1.40334
DALYs (Dis	Global	Both	50-54 yea	Chlamydia	Number	2016	6562.81	8923.04	4810.84
DALYs (Dis	Global	Both	50-54 yea	Chlamydia	Rate	2016	1.59317	2.16614	1.16787
DALYs (Dis	Global	Both	55-59 yea	Chlamydia	Number	2016	4382.65	5486.49	3399.03
DALYs (Dis	Global	Both	55-59 yea	Chlamydia	Rate	2016	1.30434	1.63285	1.0116
DALYs (Dis	Global	Both	60-64 yea	Chlamydia	Number	2016	1960.51	2341.05	1526.55
DALYs (Dis	Global	Both	60-64 yea	Chlamydia	Rate	2016	0.65731	0.7849	0.51182
DALYs (Dis	Global	Both	65-69 yea	Chlamydia	Number	2016	1078.92	1288.88	844.167
DALYs (Dis	Global	Both	65-69 yea	Chlamydia	Rate	2016	0.46404	0.55435	0.36308
DALYs (Dis	Global	Both	50-69 yea	Chlamydia	Number	2016	13984.9	17316	11230
DALYs (Dis	Global	Both	50-69 yea	Chlamydia	Rate	2016	1.09368	1.35418	0.87823
DALYs (Dis	Global	Both	50-54 yea	Gonococca	Number	2016	10869.3	13268.1	8272.49
DALYs (Dis	Global	Both	50-54 yea	Gonococca	Rate	2016	2.63862	3.22093	2.00821
DALYs (Dis	Global	Both	55-59 yea	Gonococca	Number	2016	9921.02	12234.6	7148.93
DALYs (Dis	Global	Both	55-59 yea	Gonococca	Rate	2016	2.95263	3.64118	2.12762
DALYs (Dis	Global	Both	60-64 yea	Gonococca	Number	2016	5395.23	6367.11	4256.74
DALYs (Dis	Global	Both	60-64 yea	Gonococca	Rate	2016	1.8089	2.13475	1.42719
DALYs (Dis	Global	Both	65-69 yea	Gonococca	Number	2016	5958.59	7044.26	4737.92
DALYs (Dis	Global	Both	65-69 yea	Gonococca	Rate	2016	2.5628	3.02975	2.03779
DALYs (Dis	Global	Both	50-69 yea	Gonococca	Number	2016	32144.2	37040.2	24612.9
DALYs (Dis	Global	Both	50-69 yea	Gonococca	Rate	2016	2.51381	2.8967	1.92483
DALYs (Dis	Global	Both	50-54 yea	Trichomon	Number	2016	20959	48572.1	6673.78
DALYs (Dis	Global	Both	50-54 yea	Trichomon	Rate	2016	5.08795	11.7912	1.62011
DALYs (Dis	Global	Both	55-59 yea	Trichomon	Number	2016	13767.5	32312.8	4462.81
DALYs (Dis	Global	Both	55-59 yea	Trichomon	Rate	2016	4.0974	9.61672	1.32819
DALYs (Dis	Global	Both	60-64 yea	Trichomon	Number	2016	9546.67	22164.3	3308.81
DALYs (Dis	Global	Both	60-64 yea	Trichomon	Rate	2016	3.20079	7.4312	1.10937
DALYs (Dis	Global	Both	65-69 yea	Trichomon	Number	2016	6387.62	14856.8	2296.94
DALYs (Dis	Global	Both	65-69 yea	Trichomon	Rate	2016	2.74733	6.38996	0.98792
DALYs (Dis	Global	Both	50-69 yea	Trichomon	Number	2016	50660.8	113166	18612.2
DALYs (Dis	Global	Both	50-69 yea	Trichomon	Rate	2016	3.96189	8.85006	1.45555
DALYs (Dis	Global	Both	50-54 yea	Genital he	Number	2016	23520.6	58602.8	7291.41
DALYs (Dis	Global	Both	50-54 yea	Genital he	Rate	2016	5.7098	14.2263	1.77005
DALYs (Dis	Global	Both	55-59 yea	Genital he	Number	2016	18713.1	46005.7	5734.75
DALYs (Dis	Global	Both	55-59 yea	Genital he	Rate	2016	5.56928	13.6919	1.70674
DALYs (Dis	Global	Both	60-64 yea	Genital he	Number	2016	15485.2	38070.3	4702.55
DALYs (Dis	Global	Both	60-64 yea	Genital he	Rate	2016	5.19184	12.7641	1.57666
DALYs (Dis	Global	Both	65-69 yea	Genital he	Number	2016	11205.4	27257.4	3379.95
DALYs (Dis	Global	Both	65-69 yea	Genital he	Rate	2016	4.81947	11.7235	1.45372
DALYs (Dis	Global	Both	50-69 yea	Genital he	Number	2016	68924.3	170466	21141.2
DALYs (Dis	Global	Both	50-69 yea	Genital he	Rate	2016	5.39017	13.3312	1.65333
DALYs (Dis	Global	Both	50-54 yea	Other sexu	Number	2016	14385.8	18730.2	10961
DALYs (Dis	Global	Both	50-54 yea	Other sexu	Rate	2016	3.49227	4.54689	2.66087
DALYs (Dis	Global	Both	55-59 yea	Other sexu	Number	2016	10836.8	13282.8	8444.29
DALYs (Dis	Global	Both	55-59 yea	Other sexu	Rate	2016	3.22517	3.95315	2.51313
DALYs (Dis	Global	Both	60-64 yea	Other sexu	Number	2016	4477.92	5328.73	3433.8
DALYs (Dis	Global	Both	60-64 yea	Other sexu	Rate	2016	1.50135	1.7866	1.15128
DALYs (Dis	Global	Both	65-69 yea	Other sexu	Number	2016	2684.16	3185.93	2119.84
DALYs (Dis	Global	Both	65-69 yea	Other sexu	Rate	2016	1.15446	1.37027	0.91175
DALYs (Dis	Global	Both	50-69 yea	Other sexu	Number	2016	32384.7	39005.5	26169.9
DALYs (Dis	Global	Both	50-69 yea	Other sexu	Rate	2016	2.53262	3.0504	2.0466
DALYs (Dis	Global	Both	50-54 yea	Sexually tr	Number	2010	69787.3	117381	43917.6
DALYs (Dis	Global	Both	50-54 yea	Sexually tr	Rate	2010	20.3285	34.1924	12.7929
DALYs (Dis	Global	Both	55-59 yea	Sexually tr	Number	2010	57961.1	94296.9	37456.8
DALYs (Dis	Global	Both	55-59 yea	Sexually tr	Rate	2010	19.1038	31.0799	12.3456
DALYs (Dis	Global	Both	60-64 yea	Sexually tr	Number	2010	34451.2	59347.4	21455.9
DALYs (Dis	Global	Both	60-64 yea	Sexually tr	Rate	2010	14.4195	24.8398	8.98036
DALYs (Dis	Global	Both	65-69 yea	Sexually tr	Number	2010	25298.9	41738.8	16287.5
DALYs (Dis	Global	Both	65-69 yea	Sexually tr	Rate	2010	14.1857	23.404	9.13282
DALYs (Dis	Global	Both	50-69 yea	Sexually tr	Number	2010	187498	317235	120006
DALYs (Dis	Global	Both	50-69 yea	Sexually tr	Rate	2010	17.6227	29.8165	11.2791
DALYs (Dis	Global	Both	50-54 yea	Syphilis	Number	2010	7414.71	9859.42	4942.91
DALYs (Dis	Global	Both	50-54 yea	Syphilis	Rate	2010	2.15985	2.87198	1.43983
DALYs (Dis	Global	Both	55-59 yea	Syphilis	Number	2010	7732.95	11077.6	4362.72
DALYs (Dis	Global	Both	55-59 yea	Syphilis	Rate	2010	2.54876	3.65113	1.43794
DALYs (Dis	Global	Both	60-64 yea	Syphilis	Number	2010	5398.56	7092.24	3783.19
DALYs (Dis	Global	Both	60-64 yea	Syphilis	Rate	2010	2.25956	2.96845	1.58345
DALYs (Dis	Global	Both	65-69 yea	Syphilis	Number	2010	3411.03	4608.07	2786.64
DALYs (Dis	Global	Both	65-69 yea	Syphilis	Rate	2010	1.91265	2.58386	1.56254
DALYs (Dis	Global	Both	50-69 yea	Syphilis	Number	2010	23957.2	30550.2	16414.2
DALYs (Dis	Global	Both	50-69 yea	Syphilis	Rate	2010	2.25171	2.87137	1.54275
DALYs (Dis	Global	Both	50-54 yea	Chlamydia	Number	2010	5224.79	7044.81	3884.57
DALYs (Dis	Global	Both	50-54 yea	Chlamydia	Rate	2010	1.52194	2.0521	1.13155
DALYs (Dis	Global	Both	55-59 yea	Chlamydia	Number	2010	3775.9	4540.12	3043.3
DALYs (Dis	Global	Both	55-59 yea	Chlamydia	Rate	2010	1.24452	1.49641	1.00306
DALYs (Dis	Global	Both	60-64 yea	Chlamydia	Number	2010	1574.78	1834.67	1274.95
DALYs (Dis	Global	Both	60-64 yea	Chlamydia	Rate	2010	0.65912	0.7679	0.53363
DALYs (Dis	Global	Both	65-69 yea	Chlamydia	Number	2010	946.71	1128.95	750.171
DALYs (Dis	Global	Both	65-69 yea	Chlamydia	Rate	2010	0.53084	0.63303	0.42064
DALYs (Dis	Global	Both	50-69 yea	Chlamydia	Number	2010	11522.2	14009	9494.96
DALYs (Dis	Global	Both	50-69 yea	Chlamydia	Rate	2010	1.08295	1.31669	0.89242
DALYs (Dis	Global	Both	50-54 yea	Gonococca	Number	2010	8691.38	10067.4	7124.08
DALYs (Dis	Global	Both	50-54 yea	Gonococca	Rate	2010	2.53173	2.93255	2.07519
DALYs (Dis	Global	Both	55-59 yea	Gonococca	Number	2010	8493.14	9991.2	6486.44
DALYs (Dis	Global	Both	55-59 yea	Gonococca	Rate	2010	2.79931	3.29307	2.13791
DALYs (Dis	Global	Both	60-64 yea	Gonococca	Number	2010	4420.03	5090.88	3628.6
DALYs (Dis	Global	Both	60-64 yea	Gonococca	Rate	2010	1.85	2.13078	1.51875
DALYs (Dis	Global	Both	65-69 yea	Gonococca	Number	2010	5260.46	6234.59	4227.67
DALYs (Dis	Global	Both	65-69 yea	Gonococca	Rate	2010	2.94967	3.49589	2.37056
DALYs (Dis	Global	Both	50-69 yea	Gonococca	Number	2010	26865	29948.1	21414.5
DALYs (Dis	Global	Both	50-69 yea	Gonococca	Rate	2010	2.525	2.81478	2.01272
DALYs (Dis	Global	Both	50-54 yea	HIV/AIDS	Number	2016	3511389	4026709	3179547
DALYs (Dis	Global	Both	50-54 yea	HIV/AIDS	Rate	2016	852.417	977.515	771.859
DALYs (Dis	Global	Both	55-59 yea	HIV/AIDS	Number	2016	1885784	2179971	1699360
DALYs (Dis	Global	Both	55-59 yea	HIV/AIDS	Rate	2016	561.234	648.788	505.752
DALYs (Dis	Global	Both	60-64 yea	HIV/AIDS	Number	2016	1007168	1161009	907185
DALYs (Dis	Global	Both	60-64 yea	HIV/AIDS	Rate	2016	337.681	389.261	304.159
DALYs (Dis	Global	Both	65-69 yea	HIV/AIDS	Number	2016	511206	583667	463416
DALYs (Dis	Global	Both	65-69 yea	HIV/AIDS	Rate	2016	219.871	251.036	199.316
DALYs (Dis	Global	Both	50-69 yea	HIV/AIDS	Number	2016	6915547	7932602	6309281
DALYs (Dis	Global	Both	50-69 yea	HIV/AIDS	Rate	2016	540.825	620.363	493.412
DALYs (Dis	Global	Both	50-54 yea	HIV/AIDS	Number	2019	3320860	3874281	2951969
DALYs (Dis	Global	Both	50-54 yea	HIV/AIDS	Rate	2019	760.241	886.935	675.791
DALYs (Dis	Global	Both	55-59 yea	HIV/AIDS	Number	2019	1863158	2188416	1649345
DALYs (Dis	Global	Both	55-59 yea	HIV/AIDS	Rate	2019	502.182	589.85	444.553
DALYs (Dis	Global	Both	60-64 yea	HIV/AIDS	Number	2019	1005501	1184427	884414
DALYs (Dis	Global	Both	60-64 yea	HIV/AIDS	Rate	2019	321.724	378.974	282.981
DALYs (Dis	Global	Both	65-69 yea	HIV/AIDS	Number	2019	538523	632280	472789
DALYs (Dis	Global	Both	65-69 yea	HIV/AIDS	Rate	2019	208.259	244.517	182.838
DALYs (Dis	Global	Both	50-69 yea	HIV/AIDS	Number	2019	6728043	7861113	5982448
DALYs (Dis	Global	Both	50-69 yea	HIV/AIDS	Rate	2019	487.911	570.08	433.841
DALYs (Dis	Global	Both	50-54 yea	Trichomon	Number	2010	17358.6	40992.2	5521.12
DALYs (Dis	Global	Both	50-54 yea	Trichomon	Rate	2010	5.05644	11.9407	1.60826
DALYs (Dis	Global	Both	55-59 yea	Trichomon	Number	2010	12114.4	28302	3934.01
DALYs (Dis	Global	Both	55-59 yea	Trichomon	Rate	2010	3.99286	9.32826	1.29664
DALYs (Dis	Global	Both	60-64 yea	Trichomon	Number	2010	7327.3	17084.7	2557.63
DALYs (Dis	Global	Both	60-64 yea	Trichomon	Rate	2010	3.06683	7.15077	1.07049
DALYs (Dis	Global	Both	65-69 yea	Trichomon	Number	2010	4785.21	11018.6	1693.78
DALYs (Dis	Global	Both	65-69 yea	Trichomon	Rate	2010	2.68319	6.17841	0.94975
DALYs (Dis	Global	Both	50-69 yea	Trichomon	Number	2010	41585.5	91650.8	15252.1
DALYs (Dis	Global	Both	50-69 yea	Trichomon	Rate	2010	3.90856	8.61413	1.43353
DALYs (Dis	Global	Both	50-54 yea	Genital he	Number	2010	19314.3	48076.9	5977.72
DALYs (Dis	Global	Both	50-54 yea	Genital he	Rate	2010	5.6261	14.0044	1.74127
DALYs (Dis	Global	Both	55-59 yea	Genital he	Number	2010	16236.1	40067.5	5024.72
DALYs (Dis	Global	Both	55-59 yea	Genital he	Rate	2010	5.35137	13.2061	1.65613
DALYs (Dis	Global	Both	60-64 yea	Genital he	Number	2010	12083.4	29540.4	3676.33
DALYs (Dis	Global	Both	60-64 yea	Genital he	Rate	2010	5.05748	12.3641	1.53872
DALYs (Dis	Global	Both	65-69 yea	Genital he	Number	2010	8505.11	20832.6	2570.51
DALYs (Dis	Global	Both	65-69 yea	Genital he	Rate	2010	4.76903	11.6813	1.44135
DALYs (Dis	Global	Both	50-69 yea	Genital he	Number	2010	56138.9	138079	17157.7
DALYs (Dis	Global	Both	50-69 yea	Genital he	Rate	2010	5.27641	12.9779	1.61263
DALYs (Dis	Global	Both	50-54 yea	Other sexu	Number	2010	11783.5	14956.7	9017.54
DALYs (Dis	Global	Both	50-54 yea	Other sexu	Rate	2010	3.43245	4.35679	2.62674
DALYs (Dis	Global	Both	55-59 yea	Other sexu	Number	2010	9608.57	11525.9	7703.52
DALYs (Dis	Global	Both	55-59 yea	Other sexu	Rate	2010	3.16696	3.79891	2.53906
DALYs (Dis	Global	Both	60-64 yea	Other sexu	Number	2010	3647.14	4266.33	2930.91
DALYs (Dis	Global	Both	60-64 yea	Other sexu	Rate	2010	1.52651	1.78567	1.22673
DALYs (Dis	Global	Both	65-69 yea	Other sexu	Number	2010	2390.35	2832.86	1947.17
DALYs (Dis	Global	Both	65-69 yea	Other sexu	Rate	2010	1.34033	1.58845	1.09183
DALYs (Dis	Global	Both	50-69 yea	Other sexu	Number	2010	27429.6	32544.6	22583.2
DALYs (Dis	Global	Both	50-69 yea	Other sexu	Rate	2010	2.57806	3.05882	2.12256
DALYs (Dis	Global	Both	50-54 yea	HIV/AIDS	Number	2015	3512785	4023651	3193380
DALYs (Dis	Global	Both	50-54 yea	HIV/AIDS	Rate	2015	879.148	1007	799.211
DALYs (Dis	Global	Both	55-59 yea	HIV/AIDS	Number	2015	1862516	2154481	1677997
DALYs (Dis	Global	Both	55-59 yea	HIV/AIDS	Rate	2015	564.213	652.659	508.317
DALYs (Dis	Global	Both	60-64 yea	HIV/AIDS	Number	2015	988360	1138579	891166
DALYs (Dis	Global	Both	60-64 yea	HIV/AIDS	Rate	2015	339.605	391.221	306.209
DALYs (Dis	Global	Both	65-69 yea	HIV/AIDS	Number	2015	485007	551382	440357
DALYs (Dis	Global	Both	65-69 yea	HIV/AIDS	Rate	2015	219.918	250.014	199.672
DALYs (Dis	Global	Both	50-69 yea	HIV/AIDS	Number	2015	6848668	7844457	6247284
DALYs (Dis	Global	Both	50-69 yea	HIV/AIDS	Rate	2015	551.757	631.982	503.307
DALYs (Dis	Global	Both	50-54 yea	HIV/AIDS	Number	2012	3655994	4223540	3296548
DALYs (Dis	Global	Both	50-54 yea	HIV/AIDS	Rate	2012	1020.53	1178.96	920.197
DALYs (Dis	Global	Both	55-59 yea	HIV/AIDS	Number	2012	1854549	2173949	1660658
DALYs (Dis	Global	Both	55-59 yea	HIV/AIDS	Rate	2012	583.93	684.498	522.881
DALYs (Dis	Global	Both	60-64 yea	HIV/AIDS	Number	2012	927519	1098470	828617
DALYs (Dis	Global	Both	60-64 yea	HIV/AIDS	Rate	2012	355.8	421.378	317.861
DALYs (Dis	Global	Both	65-69 yea	HIV/AIDS	Number	2012	441445	523577	391959
DALYs (Dis	Global	Both	65-69 yea	HIV/AIDS	Rate	2012	231.56	274.642	205.602
DALYs (Dis	Global	Both	50-69 yea	HIV/AIDS	Number	2012	6879507	7985803	6216678
DALYs (Dis	Global	Both	50-69 yea	HIV/AIDS	Rate	2012	610.336	708.485	551.532
DALYs (Dis	Global	Both	50-54 yea	Sexually tr	Number	2019	87726.5	152487	54455.5
DALYs (Dis	Global	Both	50-54 yea	Sexually tr	Rate	2019	20.0831	34.9087	12.4664
DALYs (Dis	Global	Both	55-59 yea	Sexually tr	Number	2019	69176.9	114815	43773.4
DALYs (Dis	Global	Both	55-59 yea	Sexually tr	Rate	2019	18.6454	30.9465	11.7984
DALYs (Dis	Global	Both	60-64 yea	Sexually tr	Number	2019	44052.1	77284.5	26841.8
DALYs (Dis	Global	Both	60-64 yea	Sexually tr	Rate	2019	14.0951	24.7282	8.5884
DALYs (Dis	Global	Both	65-69 yea	Sexually tr	Number	2019	34079.3	58595.4	21113.7
DALYs (Dis	Global	Both	65-69 yea	Sexually tr	Rate	2019	13.1792	22.6601	8.16515
DALYs (Dis	Global	Both	50-69 yea	Sexually tr	Number	2019	235035	403403	149020
DALYs (Dis	Global	Both	50-69 yea	Sexually tr	Rate	2019	17.0445	29.2544	10.8068
DALYs (Dis	Global	Both	50-54 yea	Syphilis	Number	2019	8025	10316.6	5416.17
DALYs (Dis	Global	Both	50-54 yea	Syphilis	Rate	2019	1.83716	2.36178	1.23992
DALYs (Dis	Global	Both	55-59 yea	Syphilis	Number	2019	7670.54	10354.4	4533.42
DALYs (Dis	Global	Both	55-59 yea	Syphilis	Rate	2019	2.06746	2.79084	1.2219
DALYs (Dis	Global	Both	60-64 yea	Syphilis	Number	2019	5890.49	7671.71	4225.8
DALYs (Dis	Global	Both	60-64 yea	Syphilis	Rate	2019	1.88474	2.45467	1.3521
DALYs (Dis	Global	Both	65-69 yea	Syphilis	Number	2019	4101.09	5293.36	3326.2
DALYs (Dis	Global	Both	65-69 yea	Syphilis	Rate	2019	1.58598	2.04706	1.28632
DALYs (Dis	Global	Both	50-69 yea	Syphilis	Number	2019	25687.1	31867.8	18194.6
DALYs (Dis	Global	Both	50-69 yea	Syphilis	Rate	2019	1.86281	2.31103	1.31946
DALYs (Dis	Global	Both	50-54 yea	Chlamydia	Number	2019	6815.52	9579.95	4829.08
DALYs (Dis	Global	Both	50-54 yea	Chlamydia	Rate	2019	1.56027	2.19313	1.10552
DALYs (Dis	Global	Both	55-59 yea	Chlamydia	Number	2019	4530.91	5765.21	3503.86
DALYs (Dis	Global	Both	55-59 yea	Chlamydia	Rate	2019	1.22123	1.55391	0.94441
DALYs (Dis	Global	Both	60-64 yea	Chlamydia	Number	2019	1970.04	2399.67	1495.23
DALYs (Dis	Global	Both	60-64 yea	Chlamydia	Rate	2019	0.63034	0.76781	0.47842
DALYs (Dis	Global	Both	65-69 yea	Chlamydia	Number	2019	1151.64	1416.15	895.059
DALYs (Dis	Global	Both	65-69 yea	Chlamydia	Rate	2019	0.44536	0.54766	0.34614
DALYs (Dis	Global	Both	50-69 yea	Chlamydia	Number	2019	14468.1	18321.4	11412.7
DALYs (Dis	Global	Both	50-69 yea	Chlamydia	Rate	2019	1.04921	1.32865	0.82764
DALYs (Dis	Global	Both	50-54 yea	Gonococca	Number	2019	10664.9	13357.7	7997.36
DALYs (Dis	Global	Both	50-54 yea	Gonococca	Rate	2019	2.44151	3.05797	1.83083
DALYs (Dis	Global	Both	55-59 yea	Gonococca	Number	2019	9974.39	12953.5	7265.47
DALYs (Dis	Global	Both	55-59 yea	Gonococca	Rate	2019	2.68842	3.4914	1.95828
DALYs (Dis	Global	Both	60-64 yea	Gonococca	Number	2019	5399.44	6532.33	4187.38
DALYs (Dis	Global	Both	60-64 yea	Gonococca	Rate	2019	1.72763	2.09011	1.33981
DALYs (Dis	Global	Both	65-69 yea	Gonococca	Number	2019	6333.22	7782.32	4964.77
DALYs (Dis	Global	Both	65-69 yea	Gonococca	Rate	2019	2.44919	3.00959	1.91999
DALYs (Dis	Global	Both	50-69 yea	Gonococca	Number	2019	32372	38346	24612.5
DALYs (Dis	Global	Both	50-69 yea	Gonococca	Rate	2019	2.34758	2.78081	1.78487
DALYs (Dis	Global	Both	50-54 yea	Trichomon	Number	2019	22548.6	53054.9	7097.91
DALYs (Dis	Global	Both	50-54 yea	Trichomon	Rate	2019	5.16202	12.1458	1.62492
DALYs (Dis	Global	Both	55-59 yea	Trichomon	Number	2019	15173.7	35885.9	4968.2
DALYs (Dis	Global	Both	55-59 yea	Trichomon	Rate	2019	4.08982	9.67241	1.33909
DALYs (Dis	Global	Both	60-64 yea	Trichomon	Number	2019	9887.04	22984.8	3408.06
DALYs (Dis	Global	Both	60-64 yea	Trichomon	Rate	2019	3.1635	7.35429	1.09046
DALYs (Dis	Global	Both	65-69 yea	Trichomon	Number	2019	7090.75	16433	2528.57
DALYs (Dis	Global	Both	65-69 yea	Trichomon	Rate	2019	2.74215	6.355	0.97785
DALYs (Dis	Global	Both	50-69 yea	Trichomon	Number	2019	54700.1	122380	19951.2
DALYs (Dis	Global	Both	50-69 yea	Trichomon	Rate	2019	3.9668	8.87491	1.44684
DALYs (Dis	Global	Both	50-54 yea	Genital he	Number	2019	24958.9	61887.4	7751.59
DALYs (Dis	Global	Both	50-54 yea	Genital he	Rate	2019	5.71381	14.1678	1.77456
DALYs (Dis	Global	Both	55-59 yea	Genital he	Number	2019	20668.8	51100.3	6281.88
DALYs (Dis	Global	Both	55-59 yea	Genital he	Rate	2019	5.57092	13.7732	1.69317
DALYs (Dis	Global	Both	60-64 yea	Genital he	Number	2019	16436	40336.5	4957.2
DALYs (Dis	Global	Both	60-64 yea	Genital he	Rate	2019	5.25891	12.9062	1.58613
DALYs (Dis	Global	Both	65-69 yea	Genital he	Number	2019	12551.8	30746.6	3784.51
DALYs (Dis	Global	Both	65-69 yea	Genital he	Rate	2019	4.85405	11.8904	1.46355

DALYs (Dis	Global	Both	55-59 yea	Chlamydia	Rate	2015	1.31163	1.62535	1.01201
DALYs (Dis	Global	Both	60-64 yea	Chlamydia	Number	2015	1941.18	2309.29	1513.1
DALYs (Dis	Global	Both	60-64 yea	Chlamydia	Rate	2015	0.667	0.79348	0.51991
DALYs (Dis	Global	Both	65-69 yea	Chlamydia	Number	2015	1018.29	1206.6	808.876
DALYs (Dis	Global	Both	65-69 yea	Chlamydia	Rate	2015	0.46173	0.54711	0.36677
DALYs (Dis	Global	Both	50-69 yea	Chlamydia	Number	2015	13682	16679.1	10880.2
DALYs (Dis	Global	Both	50-69 yea	Chlamydia	Rate	2015	1.10228	1.34374	0.87655
DALYs (Dis	Global	Both	50-54 yea	Gonococca	Number	2015	10755	13025.7	8301.23
DALYs (Dis	Global	Both	50-54 yea	Gonococca	Rate	2015	2.69165	3.25995	2.07756
DALYs (Dis	Global	Both	55-59 yea	Gonococca	Number	2015	9835.61	12079.4	7108.93
DALYs (Dis	Global	Both	55-59 yea	Gonococca	Rate	2015	2.97951	3.65922	2.15351
DALYs (Dis	Global	Both	60-64 yea	Gonococca	Number	2015	5352.03	6299.34	4265.17
DALYs (Dis	Global	Both	60-64 yea	Gonococca	Rate	2015	1.83898	2.16449	1.46553
DALYs (Dis	Global	Both	65-69 yea	Gonococca	Number	2015	5630.39	6684.93	4514.48
DALYs (Dis	Global	Both	65-69 yea	Gonococca	Rate	2015	2.553	3.03116	2.04701
DALYs (Dis	Global	Both	50-69 yea	Gonococca	Number	2015	31573	35781.1	24050.8
DALYs (Dis	Global	Both	50-69 yea	Gonococca	Rate	2015	2.54365	2.88267	1.93763
DALYs (Dis	Global	Both	50-54 yea	Trichomon	Number	2015	20158.4	47656.5	6419.55
DALYs (Dis	Global	Both	50-54 yea	Trichomon	Rate	2015	5.04507	11.927	1.60663
DALYs (Dis	Global	Both	55-59 yea	Trichomon	Number	2015	13496.7	31269.8	4414.45
DALYs (Dis	Global	Both	55-59 yea	Trichomon	Rate	2015	4.08857	9.47257	1.33727
DALYs (Dis	Global	Both	60-64 yea	Trichomon	Number	2015	9405.64	22140.7	3193.28
DALYs (Dis	Global	Both	60-64 yea	Trichomon	Rate	2015	3.23182	7.60766	1.09723
DALYs (Dis	Global	Both	65-69 yea	Trichomon	Number	2015	6108.53	14181.3	2191.74
DALYs (Dis	Global	Both	65-69 yea	Trichomon	Rate	2015	2.76981	6.43026	0.9938
DALYs (Dis	Global	Both	50-69 yea	Trichomon	Number	2015	49169.3	109507	18114.1
DALYs (Dis	Global	Both	50-69 yea	Trichomon	Rate	2015	3.96128	8.82232	1.45935
DALYs (Dis	Global	Both	50-54 yea	Genital he	Number	2015	22821.9	56906.2	7147.16
DALYs (Dis	Global	Both	50-54 yea	Genital he	Rate	2015	5.71167	14.242	1.78873
DALYs (Dis	Global	Both	55-59 yea	Genital he	Number	2015	18318.2	44949.3	5611.72
DALYs (Dis	Global	Both	55-59 yea	Genital he	Rate	2015	5.54913	13.6165	1.69996
DALYs (Dis	Global	Both	60-64 yea	Genital he	Number	2015	15053.3	37048.6	4569.49
DALYs (Dis	Global	Both	60-64 yea	Genital he	Rate	2015	5.1724	12.7301	1.5701
DALYs (Dis	Global	Both	65-69 yea	Genital he	Number	2015	10617.4	25739.4	3201.44
DALYs (Dis	Global	Both	65-69 yea	Genital he	Rate	2015	4.81428	11.6711	1.45164
DALYs (Dis	Global	Both	50-69 yea	Genital he	Number	2015	66810.8	165385	20567.8
DALYs (Dis	Global	Both	50-69 yea	Genital he	Rate	2015	5.38256	13.3241	1.65703
DALYs (Dis	Global	Both	50-54 yea	Other sexu	Number	2015	14032.1	18263.6	10666.1
DALYs (Dis	Global	Both	50-54 yea	Other sexu	Rate	2015	3.51183	4.57086	2.6694
DALYs (Dis	Global	Both	55-59 yea	Other sexu	Number	2015	10721.3	12959.4	8345.77
DALYs (Dis	Global	Both	55-59 yea	Other sexu	Rate	2015	3.24781	3.92579	2.52819
DALYs (Dis	Global	Both	60-64 yea	Other sexu	Number	2015	4443.57	5272.91	3460.18
DALYs (Dis	Global	Both	60-64 yea	Other sexu	Rate	2015	1.52683	1.8118	1.18893
DALYs (Dis	Global	Both	65-69 yea	Other sexu	Number	2015	2540.17	3010.67	2039.9
DALYs (Dis	Global	Both	65-69 yea	Other sexu	Rate	2015	1.1518	1.36514	0.92496
DALYs (Dis	Global	Both	50-69 yea	Other sexu	Number	2015	31737.1	38252.4	25856.3
DALYs (Dis	Global	Both	50-69 yea	Other sexu	Rate	2015	2.55688	3.08177	2.08309
DALYs (Dis	Global	Both	50-54 yea	HIV/AIDS	Number	2017	3454506	3982350	3105082
DALYs (Dis	Global	Both	50-54 yea	HIV/AIDS	Rate	2017	817.54	942.459	734.846
DALYs (Dis	Global	Both	55-59 yea	HIV/AIDS	Number	2017	1882000	2182297	1688344
DALYs (Dis	Global	Both	55-59 yea	HIV/AIDS	Rate	2017	546.028	633.154	489.843
DALYs (Dis	Global	Both	60-64 yea	HIV/AIDS	Number	2017	1011849	1172861	905628
DALYs (Dis	Global	Both	60-64 yea	HIV/AIDS	Rate	2017	332.279	385.153	297.397
DALYs (Dis	Global	Both	65-69 yea	HIV/AIDS	Number	2017	525322	604602	472635
DALYs (Dis	Global	Both	65-69 yea	HIV/AIDS	Rate	2017	217.859	250.738	196.009
DALYs (Dis	Global	Both	50-69 yea	HIV/AIDS	Number	2017	6873677	7912816	6201508
DALYs (Dis	Global	Both	50-69 yea	HIV/AIDS	Rate	2017	523.562	602.713	472.364
DALYs (Dis	Global	Both	50-54 yea	Sexually tr	Number	2018	86829.6	150375	54007
DALYs (Dis	Global	Both	50-54 yea	Sexually tr	Rate	2018	20.1661	34.9245	12.5431
DALYs (Dis	Global	Both	55-59 yea	Sexually tr	Number	2018	67542.6	113847	43265.9
DALYs (Dis	Global	Both	55-59 yea	Sexually tr	Rate	2018	18.9253	31.8997	12.123
DALYs (Dis	Global	Both	60-64 yea	Sexually tr	Number	2018	43622	76348.3	26419.2
DALYs (Dis	Global	Both	60-64 yea	Sexually tr	Rate	2018	14.1148	24.704	8.54843
DALYs (Dis	Global	Both	65-69 yea	Sexually tr	Number	2018	33067	57487.7	19962.2
DALYs (Dis	Global	Both	65-69 yea	Sexually tr	Rate	2018	13.2113	22.9681	7.97549
DALYs (Dis	Global	Both	50-69 yea	Sexually tr	Number	2018	231061	396860	143251
DALYs (Dis	Global	Both	50-69 yea	Sexually tr	Rate	2018	17.1562	29.4667	10.6363
DALYs (Dis	Global	Both	50-54 yea	Syphilis	Number	2018	8004.48	10321.5	5378.64
DALYs (Dis	Global	Both	50-54 yea	Syphilis	Rate	2018	1.85903	2.39716	1.24919
DALYs (Dis	Global	Both	55-59 yea	Syphilis	Number	2018	7594.45	10404.9	4468.06
DALYs (Dis	Global	Both	55-59 yea	Syphilis	Rate	2018	2.12795	2.91545	1.25194
DALYs (Dis	Global	Both	60-64 yea	Syphilis	Number	2018	5889.76	7676.09	4212.8
DALYs (Dis	Global	Both	60-64 yea	Syphilis	Rate	2018	1.90575	2.48375	1.36313
DALYs (Dis	Global	Both	65-69 yea	Syphilis	Number	2018	4011.81	5175.44	3259.05
DALYs (Dis	Global	Both	65-69 yea	Syphilis	Rate	2018	1.60284	2.06775	1.30209
DALYs (Dis	Global	Both	50-69 yea	Syphilis	Number	2018	25500.5	31854.7	17862.5
DALYs (Dis	Global	Both	50-69 yea	Syphilis	Rate	2018	1.8934	2.3652	1.32628
DALYs (Dis	Global	Both	50-54 yea	Chlamydia	Number	2018	6711.44	9200.5	4870.71
DALYs (Dis	Global	Both	50-54 yea	Chlamydia	Rate	2018	1.55873	2.13681	1.13122
DALYs (Dis	Global	Both	55-59 yea	Chlamydia	Number	2018	4465.23	5681.86	3393.54
DALYs (Dis	Global	Both	55-59 yea	Chlamydia	Rate	2018	1.25115	1.59205	0.95087
DALYs (Dis	Global	Both	60-64 yea	Chlamydia	Number	2018	1960	2375.51	1485.61
DALYs (Dis	Global	Both	60-64 yea	Chlamydia	Rate	2018	0.6342	0.76864	0.4807
DALYs (Dis	Global	Both	65-69 yea	Chlamydia	Number	2018	1124.9	1389.13	880.897
DALYs (Dis	Global	Both	65-69 yea	Chlamydia	Rate	2018	0.44943	0.555	0.35194
DALYs (Dis	Global	Both	50-69 yea	Chlamydia	Number	2018	14261.6	17700.2	11381.2
DALYs (Dis	Global	Both	50-69 yea	Chlamydia	Rate	2018	1.05892	1.31423	0.84505
DALYs (Dis	Global	Both	50-54 yea	Gonococca	Number	2018	10712.4	13243.5	8041.15
DALYs (Dis	Global	Both	50-54 yea	Gonococca	Rate	2018	2.48795	3.07578	1.86755
DALYs (Dis	Global	Both	55-59 yea	Gonococca	Number	2018	9934.82	12636.9	7094.77
DALYs (Dis	Global	Both	55-59 yea	Gonococca	Rate	2018	2.78372	3.54083	1.98794
DALYs (Dis	Global	Both	60-64 yea	Gonococca	Number	2018	5378.48	6476.02	4141.69
DALYs (Dis	Global	Both	60-64 yea	Gonococca	Rate	2018	1.74031	2.09544	1.34013
DALYs (Dis	Global	Both	65-69 yea	Gonococca	Number	2018	6194.78	7605.7	4908.37
DALYs (Dis	Global	Both	65-69 yea	Gonococca	Rate	2018	2.475	3.03871	1.96104
DALYs (Dis	Global	Both	50-69 yea	Gonococca	Number	2018	32220.5	37762.8	25263.5
DALYs (Dis	Global	Both	50-69 yea	Gonococca	Rate	2018	2.39236	2.80387	1.87581
DALYs (Dis	Global	Both	50-54 yea	Trichomon	Number	2018	22187.3	51822.4	7049.7
DALYs (Dis	Global	Both	50-54 yea	Trichomon	Rate	2018	5.15299	12.0357	1.63729
DALYs (Dis	Global	Both	55-59 yea	Trichomon	Number	2018	14624.7	34678.5	4739.57
DALYs (Dis	Global	Both	55-59 yea	Trichomon	Rate	2018	4.09781	9.71687	1.32802
DALYs (Dis	Global	Both	60-64 yea	Trichomon	Number	2018	9765.01	22698.2	3403
DALYs (Dis	Global	Both	60-64 yea	Trichomon	Rate	2018	3.15966	7.34445	1.10111
DALYs (Dis	Global	Both	65-69 yea	Trichomon	Number	2018	6830.63	15844.6	2458.81
DALYs (Dis	Global	Both	65-69 yea	Trichomon	Rate	2018	2.72904	6.33039	0.98237
DALYs (Dis	Global	Both	50-69 yea	Trichomon	Number	2018	53407.6	119509	19542.1
DALYs (Dis	Global	Both	50-69 yea	Trichomon	Rate	2018	3.96549	8.87348	1.45099
DALYs (Dis	Global	Both	50-54 yea	Genital he	Number	2018	24569.9	61116.4	7637.81
DALYs (Dis	Global	Both	50-54 yea	Genital he	Rate	2018	5.70635	14.1943	1.77388
DALYs (Dis	Global	Both	55-59 yea	Genital he	Number	2018	19904.2	49136.5	6076.45
DALYs (Dis	Global	Both	55-59 yea	Genital he	Rate	2018	5.57714	13.768	1.70261
DALYs (Dis	Global	Both	60-64 yea	Genital he	Number	2018	16173.9	39780.3	4906.49
DALYs (Dis	Global	Both	60-64 yea	Genital he	Rate	2018	5.23337	12.8717	1.58759
DALYs (Dis	Global	Both	65-69 yea	Genital he	Number	2018	12115.5	29675.4	3654.81
DALYs (Dis	Global	Both	65-69 yea	Genital he	Rate	2018	4.84049	11.8562	1.46021
DALYs (Dis	Global	Both	50-69 yea	Genital he	Number	2018	72763.5	179695	22241.4
DALYs (Dis	Global	Both	50-69 yea	Genital he	Rate	2018	5.40266	13.3423	1.65141
DALYs (Dis	Global	Both	50-54 yea	Other sexu	Number	2018	14644.1	19142.1	11033.8
DALYs (Dis	Global	Both	50-54 yea	Other sexu	Rate	2018	3.40108	4.44575	2.56259
DALYs (Dis	Global	Both	55-59 yea	Other sexu	Number	2018	11019.2	13798.2	8470.87
DALYs (Dis	Global	Both	55-59 yea	Other sexu	Rate	2018	3.08755	3.86625	2.37353
DALYs (Dis	Global	Both	60-64 yea	Other sexu	Number	2018	4454.9	5376.55	3372.98
DALYs (Dis	Global	Both	60-64 yea	Other sexu	Rate	2018	1.44147	1.73969	1.09139
DALYs (Dis	Global	Both	65-69 yea	Other sexu	Number	2018	2789.45	3423.04	2203.24
DALYs (Dis	Global	Both	65-69 yea	Other sexu	Rate	2018	1.11447	1.36761	0.88026
DALYs (Dis	Global	Both	50-69 yea	Other sexu	Number	2018	32907.6	39783.8	26603.6
DALYs (Dis	Global	Both	50-69 yea	Other sexu	Rate	2018	2.44338	2.95393	1.9753
Prevalence	Global	Both	50-54 yea	HIV/AIDS	Number	1990	264593	300919	230031
Prevalence	Global	Both	50-54 yea	HIV/AIDS	Rate	1990	124.461	141.548	108.203
Prevalence	Global	Both	50-54 yea	HIV/AIDS	Number	1991	326178	368816	283321
Prevalence	Global	Both	50-54 yea	HIV/AIDS	Rate	1991	151.787	171.629	131.844
Prevalence	Global	Both	50-54 yea	HIV/AIDS	Number	1992	391492	439745	343534
Prevalence	Global	Both	50-54 yea	HIV/AIDS	Rate	1992	181.015	203.325	158.84
Prevalence	Global	Both	50-54 yea	HIV/AIDS	Number	1993	459320	514703	406644
Prevalence	Global	Both	50-54 yea	HIV/AIDS	Rate	1993	211.565	237.074	187.302
Prevalence	Global	Both	50-54 yea	HIV/AIDS	Number	1994	529632	590246	471984
Prevalence	Global	Both	50-54 yea	HIV/AIDS	Rate	1994	242.791	270.578	216.365
Prevalence	Global	Both	50-54 yea	HIV/AIDS	Number	1995	598421	665795	535068
Prevalence	Global	Both	50-54 yea	HIV/AIDS	Rate	1995	273.134	303.886	244.219
Prevalence	Global	Both	50-54 yea	HIV/AIDS	Number	1996	668846	742265	602263
Prevalence	Global	Both	50-54 yea	HIV/AIDS	Rate	1996	300.8	333.818	270.855
Prevalence	Global	Both	50-54 yea	HIV/AIDS	Number	1997	742924	822092	671622
Prevalence	Global	Both	50-54 yea	HIV/AIDS	Rate	1997	322.082	356.404	291.171
Prevalence	Global	Both	50-54 yea	HIV/AIDS	Number	1998	816501	905699	733201
Prevalence	Global	Both	50-54 yea	HIV/AIDS	Rate	1998	340.061	377.211	305.367
Prevalence	Global	Both	50-54 yea	HIV/AIDS	Number	1999	885348	985639	790248
Prevalence	Global	Both	50-54 yea	HIV/AIDS	Rate	1999	351.047	390.814	313.34
Prevalence	Global	Both	50-54 yea	HIV/AIDS	Number	2000	945569	1065415	837674
Prevalence	Global	Both	50-54 yea	HIV/AIDS	Rate	2000	360.581	406.283	319.436
Prevalence	Global	Both	50-54 yea	HIV/AIDS	Number	2001	1030816	1179150	901691
Prevalence	Global	Both	50-54 yea	HIV/AIDS	Rate	2001	374.346	428.214	327.453
Prevalence	Global	Both	50-54 yea	HIV/AIDS	Number	2002	1105988	1278903	956081
Prevalence	Global	Both	50-54 yea	HIV/AIDS	Rate	2002	388.257	448.959	335.632
Prevalence	Global	Both	50-54 yea	HIV/AIDS	Number	2003	1176336	1367870	1006744
Prevalence	Global	Both	50-54 yea	HIV/AIDS	Rate	2003	398.639	463.547	341.167
Prevalence	Global	Both	50-54 yea	HIV/AIDS	Number	2004	1245633	1462244	1055080
Prevalence	Global	Both	50-54 yea	HIV/AIDS	Rate	2004	409.341	480.524	346.721
Prevalence	Global	Both	50-54 yea	HIV/AIDS	Number	2005	1322129	1566950	1112396
Prevalence	Global	Both	50-54 yea	HIV/AIDS	Rate	2005	417.419	494.714	351.203
Prevalence	Global	Both	50-54 yea	HIV/AIDS	Number	2006	1387904	1643373	1162431
Prevalence	Global	Both	50-54 yea	HIV/AIDS	Rate	2006	427.876	506.634	358.365
Prevalence	Global	Both	50-54 yea	HIV/AIDS	Number	2007	1466332	1732224	1236056
Prevalence	Global	Both	50-54 yea	HIV/AIDS	Rate	2007	443.008	523.339	373.437
Prevalence	Global	Both	50-54 yea	HIV/AIDS	Number	2008	1556041	1830182	1333063
Prevalence	Global	Both	50-54 yea	HIV/AIDS	Rate	2008	463.452	545.102	397.04
Prevalence	Global	Both	50-54 yea	HIV/AIDS	Number	2009	1655265	1927844	1432188
Prevalence	Global	Both	50-54 yea	HIV/AIDS	Rate	2009	487.761	568.083	422.027
Prevalence	Global	Both	50-54 yea	HIV/AIDS	Number	2010	1764486	2037159	1542584
Prevalence	Global	Both	50-54 yea	HIV/AIDS	Rate	2010	513.982	593.409	449.343
Prevalence	Global	Both	50-54 yea	HIV/AIDS	Number	2011	1882249	2158403	1662439
Prevalence	Global	Both	50-54 yea	HIV/AIDS	Rate	2011	538.829	617.883	475.904
Prevalence	Global	Both	50-54 yea	HIV/AIDS	Number	2012	2008610	2276439	1788569
Prevalence	Global	Both	50-54 yea	HIV/AIDS	Rate	2012	560.683	635.444	499.261
Prevalence	Global	Both	50-54 yea	HIV/AIDS	Number	2013	2144081	2407482	1928037
Prevalence	Global	Both	50-54 yea	HIV/AIDS	Rate	2013	578.261	649.3	519.993
Prevalence	Global	Both	50-54 yea	HIV/AIDS	Number	2014	2286649	2543886	2067547
Prevalence	Global	Both	50-54 yea	HIV/AIDS	Rate	2014	593.584	660.359	536.708
Prevalence	Global	Both	50-54 yea	HIV/AIDS	Number	2015	2428696	2687505	2206781
Prevalence	Global	Both	50-54 yea	HIV/AIDS	Rate	2015	607.832	672.605	552.293
Prevalence	Global	Both	50-54 yea	HIV/AIDS	Number	2016	2566911	2815932	2348289
Prevalence	Global	Both	50-54 yea	HIV/AIDS	Rate	2016	623.137	683.589	570.065
Prevalence	Global	Both	50-54 yea	HIV/AIDS	Number	2017	2702635	2943350	2488629
Prevalence	Global	Both	50-54 yea	HIV/AIDS	Rate	2017	639.603	696.57	588.956
Prevalence	Global	Both	50-54 yea	HIV/AIDS	Number	2018	2837545	3070431	2623314
Prevalence	Global	Both	50-54 yea	HIV/AIDS	Rate	2018	659.018	713.106	609.263
Prevalence	Global	Both	50-54 yea	HIV/AIDS	Number	2019	2975863	3194632	2767929
Prevalence	Global	Both	50-54 yea	HIV/AIDS	Rate	2019	681.261	731.344	633.659
Prevalence	Global	Both	55-59 yea	HIV/AIDS	Number	1990	165540	188114	144572
Prevalence	Global	Both	55-59 yea	HIV/AIDS	Rate	1990	89.2804	101.456	77.972
Prevalence	Global	Both	55-59 yea	HIV/AIDS	Number	1991	203714	229745	178897
Prevalence	Global	Both	55-59 yea	HIV/AIDS	Rate	1991	108.384	122.233	95.18
Prevalence	Global	Both	55-59 yea	HIV/AIDS	Number	1992	244602	274505	215590
Prevalence	Global	Both	55-59 yea	HIV/AIDS	Rate	1992	127.923	143.562	112.75
Prevalence	Global	Both	55-59 yea	HIV/AIDS	Number	1993	285861	320263	254025
Prevalence	Global	Both	55-59 yea	HIV/AIDS	Rate	1993	147.083	164.784	130.702
Prevalence	Global	Both	55-59 yea	HIV/AIDS	Number	1994	327499	366195	293032
Prevalence	Global	Both	55-59 yea	HIV/AIDS	Rate	1994	166.125	185.754	148.641
Prevalence	Global	Both	55-59 yea	HIV/AIDS	Number	1995	368628	409824	332308
Prevalence	Global	Both	55-59 yea	HIV/AIDS	Rate	1995	184.511	205.131	166.332
Prevalence	Global	Both	55-59 yea	HIV/AIDS	Number	1996	408568	455191	368937
Prevalence	Global	Both	55-59 yea	HIV/AIDS	Rate	1996	202.536	225.648	182.89
Prevalence	Global	Both	55-59 yea	HIV/AIDS	Number	1997	447844	498556	403688
Prevalence	Global	Both	55-59 yea	HIV/AIDS	Rate	1997	220.769	245.768	199.002
Prevalence	Global	Both	55-59 yea	HIV/AIDS	Number	1998	485146	541382	434553
Prevalence	Global	Both	55-59 yea	HIV/AIDS	Rate	1998	238.263	265.881	213.416
Prevalence	Global	Both	55-59 yea	HIV/AIDS	Number	1999	520601	587368	460771
Prevalence	Global	Both	55-59 yea	HIV/AIDS	Rate	1999	254.305	286.92	225.079
Prevalence	Global	Both	55-59 yea	HIV/AIDS	Number	2000	552154	628891	483823
Prevalence	Global	Both	55-59 yea	HIV/AIDS	Rate	2000	268.226	305.504	235.032
Prevalence	Global	Both	55-59 yea	HIV/AIDS	Number	2001	585475	674601	507247
Prevalence	Global	Both	55-59 yea	HIV/AIDS	Rate	2001	279.692	322.27	242.321
Prevalence	Global	Both	55-59 yea	HIV/AIDS	Number	2002	621649	726708	533104
Prevalence	Global	Both	55-59 yea	HIV/AIDS	Rate	2002	285.414	333.649	244.761
Prevalence	Global	Both	55-59 yea	HIV/AIDS	Number	2003	659254	780118	559840
Prevalence	Global	Both	55-59 yea	HIV/AIDS	Rate	2003	289.945	343.102	246.222
Prevalence	Global	Both	55-59 yea	HIV/AIDS	Number	2004	697240	835374	586245
Prevalence	Global	Both	55-59 yea	HIV/AIDS	Rate	2004	291.118	348.792	244.774
Prevalence	Global	Both	55-59 yea	HIV/AIDS	Number	2005	734035	883576	612099
Prevalence	Global	Both	55-59 yea	HIV/AIDS	Rate	2005	294.103	354.019	245.247
Prevalence	Global	Both	55-59 yea	HIV/AIDS	Number	2006	793118	956016	658843
Prevalence	Global	Both	55-59 yea	HIV/AIDS	Rate	2006	302.081	364.126	250.939
Prevalence	Global	Both	55-59 yea	HIV/AIDS	Number	2007	853165	1023362	709569
Prevalence	Global	Both	55-59 yea	HIV/AIDS	Rate	2007	313.744	376.333	260.938
Prevalence	Global	Both	55-59 yea	HIV/AIDS	Number	2008	919593	1093150	772004
Prevalence	Global	Both	55-59 yea	HIV/AIDS	Rate	2008	326.092	387.636	273.756
Prevalence	Global	Both	55-59 yea	HIV/AIDS	Number	2009	995097	1173903	843664
Prevalence	Global	Both	55-59 yea	HIV/AIDS	Rate	2009	341.821	403.242	289.803
Prevalence	Global	Both	55-59 yea	HIV/AIDS	Number	2010	1084615	1269677	927951
Prevalence	Global	Both	55-59 yea	HIV/AIDS	Rate	2010	357.486	418.481	305.85
Prevalence	Global	Both	55-59 yea	HIV/AIDS	Number	2011	1168274	1359461	1004801
Prevalence	Global	Both	55-59 yea	HIV/AIDS	Rate	2011	375.659	437.135	323.094
Prevalence	Global	Both	55-59 yea	HIV/AIDS	Number	2012	1261372	1457772	1095645
Prevalence	Global	Both	55-59 yea	HIV/AIDS	Rate	2012	397.16	458.999	344.979
Prevalence	Global	Both	55-59 yea	HIV/AIDS	Number	2013	1363478	1565134	1189306
Prevalence	Global	Both	55-59 yea	HIV/AIDS	Rate	2013	422.894	485.439	368.873
Prevalence	Global	Both	55-59 yea	HIV/AIDS	Number	2014	1472385	1684527	1289121
Prevalence	Global	Both	55-59 yea	HIV/AIDS	Rate	2014	451.457	516.503	395.265
Prevalence	Global	Both	55-59 yea	HIV/AIDS	Number	2015	1589976	1805334	1403313
Prevalence	Global	Both	55-59 yea	HIV/AIDS	Rate	2015	481.653	546.891	425.106
Prevalence	Global	Both	55-59 yea	HIV/AIDS	Number	2016	1714952	1929141	1526147
Prevalence	Global	Both	55-59 yea	HIV/AIDS	Rate	2016	510.393	574.138	454.202
Prevalence	Global	Both	55-59 yea	HIV/AIDS	Number	2017	1845390	2055619	1654048
Prevalence	Global	Both	55-59 yea	HIV/AIDS	Rate	2017	535.407	596.401	479.892
Prevalence	Global	Both	55-59 yea	HIV/AIDS	Number	2018	1981254	2188891	1786518
Prevalence	Global	Both	55-59 yea	HIV/AIDS	Rate	2018	555.145	613.324	500.58
Prevalence	Global	Both	55-59 yea	HIV/AIDS	Number	2019	2120672	2326588	1923356
Prevalence	Global	Both	55-59 yea	HIV/AIDS	Rate	2019	571.59	627.092	518.407
Prevalence	Global	Both	60-64 yea	HIV/AIDS	Number	1990	103682	118114	90219.1
Prevalence	Global	Both	60-64 yea	HIV/AIDS	Rate	1990	64.5419	73.5255	56.1612
Prevalence	Global	Both	60-64 yea	HIV/AIDS	Number	1991	127721	143974	111792
Prevalence	Global	Both	60-64 yea	HIV/AIDS	Rate	1991	77.8849	87.7962	68.1712
Prevalence	Global	Both	60-64 yea	HIV/AIDS	Number	1992	152979	172029	134657
Prevalence	Global	Both	60-64 yea	HIV/AIDS	Rate	1992	91.9668	103.419	80.9521
Prevalence	Global	Both	60-64 yea	HIV/AIDS	Number	1993	179175	201348	158950
Prevalence	Global	Both	60-64 yea	HIV/AIDS	Rate	1993	106.421	119.591	94.4084
Prevalence	Global	Both	60-64 yea	HIV/AIDS	Number	1994	205221	229621	184043
Prevalence	Global	Both	60-64 yea	HIV/AIDS	Rate	1994	120.693	135.043	108.238
Prevalence	Global	Both	60-64 yea	HIV/AIDS	Number	1995	230983	257470	208209
Prevalence	Global	Both	60-64 yea	HIV/AIDS	Rate	1995	134.294	149.693	121.053
Prevalence	Global	Both	60-64 yea	HIV/AIDS	Number	1996	255984	285286	231324
Prevalence	Global	Both	60-64 yea	HIV/AIDS	Rate	1996	146.633	163.418	132.507
Prevalence	Global	Both	60-64 yea	HIV/AIDS	Number	1997	280361	312541	252651
Prevalence	Global	Both	60-64 yea	HIV/AIDS	Rate	1997	157.6	175.689	142.023
Prevalence	Global	Both	60-64 yea	HIV/AIDS	Number	1998	302770	338906	270918
Prevalence	Global	Both	60-64 yea	HIV/AIDS	Rate	1998	167.116	187.062	149.536
Prevalence	Global	Both	60-64 yea	HIV/AIDS	Number	1999	323785	366198	286235
Prevalence	Global	Both	60-64 yea	HIV/AIDS	Rate	1999	175.841	198.874	155.448
Prevalence	Global	Both	60-64 yea	HIV/AIDS	Number	2000	343951	392190	300849
Prevalence	Global	Both	60-64 yea	HIV/AIDS	Rate	2000	183.974	209.776	160.919
Prevalence	Global	Both	60-64 yea	HIV/AIDS	Number	2001	362758	418546	313161
Prevalence	Global	Both	60-64 yea	HIV/AIDS	Rate	2001	191.803	221.299	165.579
Prevalence	Global	Both	60-64 yea	HIV/AIDS	Number	2002	380866	445424	326226
Prevalence	Global	Both	60-64 yea	HIV/AIDS	Rate	2002	199.873	233.752	171.199
Prevalence	Global	Both	60-64 yea	HIV/AIDS	Number	2003	398435	470744	338137
Prevalence	Global	Both	60-64 yea	HIV/AIDS	Rate	2003	207.817	245.532	176.367
Prevalence	Global	Both	60-64 yea	HIV/AIDS	Number	2004	416395	499651	351284
Prevalence	Global	Both	60-64 yea	HIV/AIDS	Rate	2004	215.404	258.473	181.721
Prevalence	Global	Both	60-64 yea	HIV/AIDS	Number	2005	433991	526185	362987
Prevalence	Global	Both	60-64 yea	HIV/AIDS	Rate	2005	222.547	269.823	186.137
Prevalence	Global	Both	60-64 yea	HIV/AIDS	Number	2006	459077	558325	380785
Prevalence	Global	Both	60-64 yea	HIV/AIDS	Rate	2006	230.843	280.749	191.475
Prevalence	Global	Both	60-64 yea	HIV/AIDS	Number	2007	494605	598025	408389
Prevalence	Global	Both	60-64 yea	HIV/AIDS	Rate	2007	238.297	288.124	196.758
Prevalence	Global	Both	60-64 yea	HIV/AIDS	Number	2008	537120	642438	449464
Prevalence	Global	Both	60-64 yea	HIV/AIDS	Rate	2008	247.343	295.842	206.978
Prevalence	Global	Both	60-64 yea	HIV/AIDS	Number	2009	585975	693196	493047
Prevalence	Global	Both	60-64 yea	HIV/AIDS	Rate	2009	255.788	302.592	215.223
Prevalence	Global	Both	60-64 yea	HIV/AIDS	Number	2010	636922	748454	537062
Prevalence	Global	Both	60-64 yea	HIV/AIDS	Rate	2010	266.583	313.265	224.787
Prevalence	Global	Both	60-64 yea	HIV/AIDS	Number	2011	705136	827817	597001
Prevalence	Global	Both	60-64 yea	HIV/AIDS	Rate	2011	280.292	329.057	237.308
Prevalence	Global	Both	60-64 yea	HIV/AIDS	Number	2012	771851	899061	658101
Prevalence	Global	Both	60-64 yea	HIV/AIDS	Rate	2012	296.085	344.884	252.45
Prevalence	Global	Both	60-64 yea	HIV/AIDS	Number	2013	843514	976715	725066
Prevalence	Global	Both	60-64 yea	HIV/AIDS	Rate	2013	311.916	361.172	268.116
Prevalence	Global	Both	60-64 yea	HIV/AIDS	Number	2014	921676	1064223	796896
Prevalence	Global	Both	60-64 yea	HIV/AIDS	Rate	2014	330.051	381.097	285.368
Prevalence	Global	Both	60-64 yea	HIV/AIDS	Number	2015	1011523	1164151	877351
Prevalence	Global	Both	60-64 yea	HIV/AIDS	Rate	2015	347.564	400.008	301.462
Prevalence	Global	Both	60-64 yea	HIV/AIDS	Number	2016	1094988	1256166	956263
Prevalence	Global	Both	60-64 yea	HIV/AIDS	Rate	2016	367.125	421.165	320.614
Prevalence	Global	Both	60-64 yea	HIV/AIDS	Number	2017	1185897	1354387	1039423
Prevalence	Global	Both	60-64 yea	HIV/AIDS	Rate	2017	389.434	444.764	341.334
Prevalence	Global	Both	60-64 yea	HIV/AIDS	Number	2018	1284540	1456279	1130670
Prevalence	Global	Both	60-64 yea	HIV/AIDS	Rate	2018	415.638	471.207	365.85
Prevalence	Global	Both	60-64 yea	HIV/AIDS	Number	2019	1389249	1566733	1230519
Prevalence	Global	Both	60-64 yea	HIV/AIDS	Rate	2019	444.509	501.298	393.722
Prevalence	Global	Both	65-69 yea	HIV/AIDS	Number	1990	62505.2	71696.7	53227
Prevalence	Global	Both	65-69 yea	HIV/AIDS	Rate	1990	50.6162	58.0593	43.1028
Prevalence	Global	Both	65-69 yea	HIV/AIDS	Number	1991	78145	89206.7	67071.8
Prevalence	Global	Both	65-69 yea	HIV/AIDS	Rate	1991	61.3997	70.091	52.6993
Prevalence	Global	Both	65-69 yea	HIV/AIDS	Number	1992	94945.7	107989	82234.4
Prevalence	Global	Both	65-69 yea	HIV/AIDS	Rate	1992	72.4184	82.3672	62.7231
Prevalence	Global	Both	65-69 yea	HIV/AIDS	Number	1993	112412	127487	98317.2
Prevalence	Global	Both	65-69 yea	HIV/AIDS	Rate	1993	83.3047	94.4763	72.8598
Prevalence	Global	Both	65-69 yea	HIV/AIDS	Number	1994	129857	146224	114747
Prevalence	Global	Both	65-69 yea	HIV/AIDS	Rate	1994	93.5945	105.391	82.7043
Prevalence	Global	Both	65-69 yea	HIV/AIDS	Number	1995	146887	164563	131133
Prevalence	Global	Both	65-69 yea	HIV/AIDS	Rate	1995	103.394	115.836	92.3048
Prevalence	Global	Both	65-69 yea	HIV/AIDS	Number	1996	162745	181356	146630
Prevalence	Global	Both	65-69 yea	HIV/AIDS	Rate	1996	112.172	125	101.065
Prevalence	Global	Both	65-69 yea	HIV/AIDS	Number	1997	177586	197790	160621
Prevalence	Global	Both	65-69 yea	HIV/AIDS	Rate	1997	120.572	134.289	109.053
Prevalence	Global	Both	65-69 yea	HIV/AIDS	Number	1998	191152	213529	171802
Prevalence	Global	Both	65-69 yea	HIV/AIDS	Rate	1998	128.055	143.046	115.092
Prevalence	Global	Both	65-69 yea	HIV/AIDS	Number	1999	203148	229056	180453
Prevalence	Global	Both	65-69 yea	HIV/AIDS	Rate	1999	134.485	151.637	119.461
Prevalence	Global	Both	65-69 yea	HIV/AIDS	Number	2000	214398	244400	188021
Prevalence	Global	Both	65-69 yea	HIV/AIDS	Rate	2000	139.966	159.553	122.747
Prevalence	Global	Both	65-69 yea	HIV/AIDS	Number	2001	224958	259918	194843
Prevalence	Global	Both	65-69 yea	HIV/AIDS	Rate	2001	144.287	166.711	124.971
Prevalence	Global	Both	65-69 yea	HIV/AIDS	Number	2002	235047	274262	201547
Prevalence	Global	Both	65-69 yea	HIV/AIDS	Rate	2002	147.531	172.145	126.504
Prevalence	Global	Both	65-69 yea	HIV/AIDS	Number	2003	244590	288755	207793
Prevalence	Global	Both	65-69 yea	HIV/AIDS	Rate	2003	150.362	177.512	127.741
Prevalence	Global	Both	65-69 yea	HIV/AIDS	Number	2004	254398	305109	214331
Prevalence	Global	Both	65-69 yea	HIV/AIDS	Rate	2004	153.539	184.145	129.357
Prevalence	Global	Both	65-69 yea	HIV/AIDS	Number	2005	265296	321871	222419
Prevalence	Global	Both	65-69 yea	HIV/AIDS	Rate	2005	157.365	190.923	131.932
Prevalence	Global	Both	65-69 yea	HIV/AIDS	Number	2006	278017	339275	232659
Prevalence	Global	Both	65-69 yea	HIV/AIDS	Rate	2006	162.602	198.429	136.073
Prevalence	Global	Both	65-69 yea	HIV/AIDS	Number	2007	294061	357272	245722
Prevalence	Global	Both	65-69 yea	HIV/AIDS	Rate	2007	170.217	206.806	142.236
Prevalence	Global	Both	65-69 yea	HIV/AIDS	Number	2008	313599	378129	264188
Prevalence	Global	Both	65-69 yea	HIV/AIDS	Rate	2008	179.847	216.855	151.51
Prevalence	Global	Both	65-69 yea	HIV/AIDS	Number	2009	336363	401691	286164
Prevalence	Global	Both	65-69 yea	HIV/AIDS	Rate	2009	190.744	227.79	162.277
Prevalence	Global	Both	65-69 yea	HIV/AIDS	Number	2010	360980	425902	306837
Prevalence	Global	Both	65-69 yea	HIV/AIDS	Rate	2010	202.411	238.814	172.051
Prevalence	Global	Both	65-69 yea	HIV/AIDS	Number	2011	392411	460519	334278
Prevalence	Global	Both	65-69 yea	HIV/AIDS	Rate	2011	215.348	252.724	183.446
Prevalence	Global	Both	65-69 yea	HIV/AIDS	Number	2012	432778	502843	369447
Prevalence	Global	Both	65-69 yea	HIV/AIDS	Rate	2012	227.014	263.766	193.793
Prevalence	Global	Both	65-69 yea	HIV/AIDS	Number	2013	478278	554963	410866
Prevalence	Global	Both	65-69 yea	HIV/AIDS	Rate	2013	239.315	277.685	205.583
Prevalence	Global	Both	65-69 yea	HIV/AIDS	Number	2014	528638	611830	455459
Prevalence	Global	Both	65-69 yea	HIV/AIDS	Rate	2014	250.261	289.644	215.617
Prevalence	Global	Both	65-69 yea	HIV/AIDS	Number	2015	580091	666597	499262
Prevalence	Global	Both	65-69 yea	HIV/AIDS	Rate	2015	263.032	302.257	226.382
Prevalence	Global	Both	65-69 yea	HIV/AIDS	Number	2016	646439	740216	557691
Prevalence	Global	Both	65-69 yea	HIV/AIDS	Rate	2016	278.035	318.368	239.864
Prevalence	Global	Both	65-69 yea	HIV/AIDS	Number	2017	710070	813294	613693
Prevalence	Global	Both	65-69 yea	HIV/AIDS	Rate	2017	294.477	337.286	254.508
Prevalence	Global	Both	65-69 yea	HIV/AIDS	Number	2018	777995	887077	672437
Prevalence	Global	Both	65-69 yea	HIV/AIDS	Rate	2018	310.832	354.414	268.659
Prevalence	Global	Both	65-69 yea	HIV/AIDS	Number	2019	851349	969807	738776
Prevalence	Global	Both	65-69 yea	HIV/AIDS	Rate	2019	329.236	375.046	285.701
Prevalence	Global	Both	50-69 yea	HIV/AIDS	Number	1990	596320	678971	516556
Prevalence	Global	Both	50-69 yea	HIV/AIDS	Rate	1990	87.4193	99.5356	75.726
Prevalence	Global	Both	50-69 yea	HIV/AIDS	Number	1991	735758	831277	641070
Prevalence	Global	Both	50-69 yea	HIV/AIDS	Rate	1991	106.001	119.762	92.3588
Prevalence	Global	Both	50-69 yea	HIV/AIDS	Number	1992	884019	995595	775986
Prevalence	Global	Both	50-69 yea	HIV/AIDS	Rate	1992	125.404	141.232	110.079
Prevalence	Global	Both	50-69 yea	HIV/AIDS	Number	1993	1036768	1163858	917240
Prevalence	Global	Both	50-69 yea	HIV/AIDS	Rate	1993	145.05	162.831	128.328
Prevalence	Global	Both	50-69 yea	HIV/AIDS	Number	1994	1192209	1330803	1065333
Prevalence	Global	Both	50-69 yea	HIV/AIDS	Rate	1994	164.655	183.797	147.133
Prevalence	Global	Both	50-69 yea	HIV/AIDS	Number	1995	1344919	1495919	1211570
Prevalence	Global	Both	50-69 yea	HIV/AIDS	Rate	1995	183.496	204.098	165.302
Prevalence	Global	Both	50-69 yea	HIV/AIDS	Number	1996	1496143	1666106	1352520
Prevalence	Global	Both	50-69 yea	HIV/AIDS	Rate	1996	201.164	224.017	181.854
Prevalence	Global	Both	50-69 yea	HIV/AIDS	Number	1997	1648714	1827417	1488081
Prevalence	Global	Both	50-69 yea	HIV/AIDS	Rate	1997	217.308	240.862	196.136
Prevalence	Global	Both	50-69 yea	HIV/AIDS	Number	1998	1795568	1994388	1614226
Prevalence	Global	Both	50-69 yea	HIV/AIDS	Rate	1998	231.935	257.617	208.511
Prevalence	Global	Both	50-69 yea	HIV/AIDS	Number	1999	1932881	2163924	1720335
Prevalence	Global	Both	50-69 yea	HIV/AIDS	Rate	1999	244.017	273.186	217.184
Prevalence	Global	Both	50-69 yea	HIV/AIDS	Number	2000	2056071	2327541	1815001
Prevalence	Global	Both	50-69 yea	HIV/AIDS	Rate	2000	254.394	287.982	224.567
Prevalence	Global	Both	50-69 yea	HIV/AIDS	Number	2001	2204007	2528309	1923591
Prevalence	Global	Both	50-69 yea	HIV/AIDS	Rate	2001	265.628	304.713	231.832
Prevalence	Global	Both	50-69 yea	HIV/AIDS	Number	2002	2343550	2715357	2023748
Prevalence	Global	Both	50-69 yea	HIV/AIDS	Rate	2002	274.89	318.502	237.379
Prevalence	Global	Both	50-69 yea	HIV/AIDS	Number	2003	2478615	2903742	2117135
Prevalence	Global	Both	50-69 yea	HIV/AIDS	Rate	2003	282.672	331.155	241.447
Prevalence	Global	Both	50-69 yea	HIV/AIDS	Number	2004	2613666	3103974	2206585
Prevalence	Global	Both	50-69 yea	HIV/AIDS	Rate	2004	289.505	343.814	244.414
Prevalence	Global	Both	50-69 yea	HIV/AIDS	Number	2005	2755451	3281563	2316966
Prevalence	Global	Both	50-69 yea	HIV/AIDS	Rate	2005	296.31	352.886	249.157
Prevalence	Global	Both	50-69 yea	HIV/AIDS	Number	2006	2918116	3484652	2443918
Prevalence	Global	Both	50-69 yea	HIV/AIDS	Rate	2006	304.996	364.209	255.434
Prevalence	Global	Both	50-69 yea	HIV/AIDS	Number	2007	3108163	3698365	2619939
Prevalence	Global	Both	50-69 yea	HIV/AIDS	Rate	2007	316.114	376.141	266.46
Prevalence	Global	Both	50-69 yea	HIV/AIDS	Number	2008	3326353	3938346	2829409
Prevalence	Global	Both	50-69 yea	HIV/AIDS	Rate	2008	329.577	390.213	280.339
Prevalence	Global	Both	50-69 yea	HIV/AIDS	Number	2009	3572700	4197336	3069104
Prevalence	Global	Both	50-69 yea	HIV/AIDS	Rate	2009	344.887	405.185	296.273
Prevalence	Global	Both	50-69 yea	HIV/AIDS	Number	2010	3847003	4465452	3325563
Prevalence	Global	Both	50-69 yea	HIV/AIDS	Rate	2010	361.574	419.701	312.565
Prevalence	Global	Both	50-69 yea	HIV/AIDS	Number	2011	4148070	4779877	3605064
Prevalence	Global	Both	50-69 yea	HIV/AIDS	Rate	2011	379.127	436.874	329.497
Prevalence	Global	Both	50-69 yea	HIV/AIDS	Number	2012	4474611	5119701	3912759
Prevalence	Global	Both	50-69 yea	HIV/AIDS	Rate	2012	396.979	454.21	347.132
Prevalence	Global	Both	50-69 yea	HIV/AIDS	Number	2013	4829351	5484420	4242975
Prevalence	Global	Both	50-69 yea	HIV/AIDS	Rate	2013	415.078	471.381	364.68
Prevalence	Global	Both	50-69 yea	HIV/AIDS	Number	2014	5209348	5890404	4621684
Prevalence	Global	Both	50-69 yea	HIV/AIDS	Rate	2014	433.442	490.109	384.546
Prevalence	Global	Both	50-69 yea	HIV/AIDS	Number	2015	5610286	6299562	5003889
Prevalence	Global	Both	50-69 yea	HIV/AIDS	Rate	2015	451.988	507.519	403.134
Prevalence	Global	Both	50-69 yea	HIV/AIDS	Number	2016	6023289	6724054	5407383
Prevalence	Global	Both	50-69 yea	HIV/AIDS	Rate	2016	471.047	525.85	422.88
Prevalence	Global	Both	50-69 yea	HIV/AIDS	Number	2017	6443993	7139731	5822603
Prevalence	Global	Both	50-69 yea	HIV/AIDS	Rate	2017	490.834	543.827	443.503
Prevalence	Global	Both	50-69 yea	HIV/AIDS	Number	2018	6881333	7574303	6245049
Prevalence	Global	Both	50-69 yea	HIV/AIDS	Rate	2018	510.936	562.389	463.693
Prevalence	Global	Both	50-69 yea	HIV/AIDS	Number	2019	7337133	8025529	6701654
Prevalence	Global	Both	50-69 yea	HIV/AIDS	Rate	2019	532.082	582.004	485.997
Prevalence	Global	Both	50-54 yea	Sexually tr	Number	1990	5.7E+07	6.4E+07	4.9E+07
Prevalence	Global	Both	50-54 yea	Sexually tr	Rate	1990	26588.3	30338.5	23133.5
Prevalence	Global	Both	50-54 yea	Sexually tr	Number	1991	5.8E+07	6.6E+07	5E+07
Prevalence	Global	Both	50-54 yea	Sexually tr	Rate	1991	26835.7	30575.6	23369.6
Prevalence	Global	Both	50-54 yea	Sexually tr	Number	1992	5.9E+07	6.7E+07	5.1E+07
Prevalence	Global	Both	50-54 yea	Sexually tr	Rate	1992	27049.5	30802	23561
Prevalence	Global	Both	50-54 yea	Sexually tr	Number	1993	5.9E+07	6.7E+07	5.1E+07
Prevalence	Global	Both	50-54 yea	Sexually tr	Rate	1993	27212.6	30997	23708.9
Prevalence	Global	Both	50-54 yea	Sexually tr	Number	1994	6E+07	6.8E+07	5.2E+07
Prevalence	Global	Both	50-54 yea	Sexually tr	Rate	1994	27330.3	31119.6	23842.8
Prevalence	Global	Both	50-54 yea	Sexually tr	Number	1995	6E+07	6.8E+07	5.2E+07
Prevalence	Global	Both	50-54 yea	Sexually tr	Rate	1995	27414.1	31171.2	23914.5
Prevalence	Global	Both	50-54 yea	Sexually tr	Number	1996	6.1E+07	6.9E+07	5.3E+07
Prevalence	Global	Both	50-54 yea	Sexually tr	Rate	1996	27421.8	31192.6	23901.1
Prevalence	Global	Both	50-54 yea	Sexually tr	Number	1997	6.3E+07	7.1E+07	5.5E+07
Prevalence	Global	Both	50-54 yea	Sexually tr	Rate	1997	27245.3	30959.5	23773.7
Prevalence	Global	Both	50-54 yea	Sexually tr	Number	1998	6.5E+07	7.4E+07	5.7E+07
Prevalence	Global	Both	50-54 yea	Sexually tr	Rate	1998	27045.2	30737.8	23594
Prevalence	Global	Both	50-54 yea	Sexually tr	Number	1999	6.8E+07	7.7E+07	5.9E+07
Prevalence	Global	Both	50-54 yea	Sexually tr	Rate	1999	26844.8	30470.5	23384.5
Prevalence	Global	Both	50-54 yea	Sexually tr	Number	2000	7E+07	8E+07	6.1E+07
Prevalence	Global	Both	50-54 yea	Sexually tr	Rate	2000	26773.9	30324.9	23331.6
Prevalence	Global	Both	50-54 yea	Sexually tr	Number	2001	7.4E+07	8.4E+07	6.4E+07
Prevalence	Global	Both	50-54 yea	Sexually tr	Rate	2001	26775.6	30355	23361.2
Prevalence	Global	Both	50-54 yea	Sexually tr	Number	2002	7.6E+07	8.7E+07	6.7E+07
Prevalence	Global	Both	50-54 yea	Sexually tr	Rate	2002	26823.6	30425	23387.8
Prevalence	Global	Both	50-54 yea	Sexually tr	Number	2003	7.9E+07	9E+07	6.9E+07
Prevalence	Global	Both	50-54 yea	Sexually tr	Rate	2003	26858.5	30488.1	23428.2
Prevalence	Global	Both	50-54 yea	Sexually tr	Number	2004	8.2E+07	9.3E+07	7.1E+07
Prevalence	Global	Both	50-54 yea	Sexually tr	Rate	2004	26922.2	30574.4	23486
Prevalence	Global	Both	50-54 yea	Sexually tr	Number	2005	8.5E+07	9.7E+07	7.4E+07
Prevalence	Global	Both	50-54 yea	Sexually tr	Rate	2005	26951.6	30645.2	23509.7
Prevalence	Global	Both	50-54 yea	Sexually tr	Number	2006	8.7E+07	9.9E+07	7.6E+07
Prevalence	Global	Both	50-54 yea	Sexually tr	Rate	2006	26919.2	30638.8	23478
Prevalence	Global	Both	50-54 yea	Sexually tr	Number	2007	8.9E+07	1E+08	7.7E+07
Prevalence	Global	Both	50-54 yea	Sexually tr	Rate	2007	26783.2	30487.6	23314.7
Prevalence	Global	Both	50-54 yea	Sexually tr	Number	2008	8.9E+07	1E+08	7.8E+07
Prevalence	Global	Both	50-54 yea	Sexually tr	Rate	2008	26641.7	30335.3	23180.4
Prevalence	Global	Both	50-54 yea	Sexually tr	Number	2009	9E+07	1E+08	7.8E+07
Prevalence	Global	Both	50-54 yea	Sexually tr	Rate	2009	26559.3	30275.9	23062.5
Prevalence	Global	Both	50-54 yea	Sexually tr	Number	2010	9.1E+07	1E+08	7.9E+07
Prevalence	Global	Both	50-54 yea	Sexually tr	Rate	2010	26593.4	30329.5	23078.1
Prevalence	Global	Both	50-54 yea	Sexually tr	Number	2011	9.3E+07	1.1E+08	8.1E+07
Prevalence	Global	Both	50-54 yea	Sexually tr	Rate	2011	26704.5	30495.1	23166.8
Prevalence	Global	Both	50-54 yea	Sexually tr	Number	2012	9.6E+07	1.1E+08	8.3E+07
Prevalence	Global	Both	50-54 yea	Sexually tr	Rate	2012	26811	30662.2	23270
Prevalence	Global	Both	50-54 yea	Sexually tr	Number	2013	1E+08	1.1E+08	8.6E+07
Prevalence	Global	Both	50-54 yea	Sexually tr	Rate	2013	26886	30733.6	23316.2
Prevalence	Global	Both	50-54 yea	Sexually tr	Number	2014	1E+08	1.2E+08	9E+07
Prevalence	Global	Both	50-54 yea	Sexually tr	Rate	2014	26936.5	30825.8	23354.3
Prevalence	Global	Both	50-54 yea	Sexually tr	Number	2015	1.1E+08	1.2E+08	9.3E+07
Prevalence	Global	Both	50-54 yea	Sexually tr	Rate	2015	26939.5	30858.8	23353.1
Prevalence	Global	Both	50-54 yea	Sexually tr	Number	2016	1.1E+08	1.3E+08	9.6E+07
Prevalence	Global	Both	50-54 yea	Sexually tr	Rate	2016	26957.8	30904.4	23364.7
Prevalence	Global	Both	50-54 yea	Sexually tr	Number	2017	1.1E+08	1.3E+08	9.9E+07
Prevalence	Global	Both	50-54 yea	Sexually tr	Rate	2017	26980.4	30993.5	23331.8
Prevalence	Global	Both	50-54 yea	Sexually tr	Number	2018	1.2E+08	1.3E+08	1E+08
Prevalence	Global	Both	50-54 yea	Sexually tr	Rate	2018	27013.8	31092.7	23317.9
Prevalence	Global	Both	50-54 yea	Sexually tr	Number	2019	1.2E+08	1.4E+08	1E+08
Prevalence	Global	Both	50-54 yea	Sexually tr	Rate	2019	27095.1	31207.4	23422.1
Prevalence	Global	Both	55-59 yea	Sexually tr	Number	1990	4.6E+07	5.2E+07	4E+07
Prevalence	Global	Both	55-59 yea	Sexually tr	Rate	1990	24794.5	28291.1	21526.5
Prevalence	Global	Both	55-59 yea	Sexually tr	Number	1991	4.7E+07	5.4E+07	4.1E+07
Prevalence	Global	Both	55-59 yea	Sexually tr	Rate	1991	25037.3	28549	21762.4
Prevalence	Global	Both	55-59 yea	Sexually tr	Number	1992	4.8E+07	5.5E+07	4.2E+07
Prevalence	Global	Both	55-59 yea	Sexually tr	Rate	1992	25237.2	28772.3	21981.8
Prevalence	Global	Both	55-59 yea	Sexually tr	Number	1993	4.9E+07	5.6E+07	4.3E+07
Prevalence	Global	Both	55-59 yea	Sexually tr	Rate	1993	25389.1	28934	22138.1
Prevalence	Global	Both	55-59 yea	Sexually tr	Number	1994	5E+07	5.7E+07	4.4E+07
Prevalence	Global	Both	55-59 yea	Sexually tr	Rate	1994	25500.8	29058.3	22269.4
Prevalence	Global	Both	55-59 yea	Sexually tr	Number	1995	5.1E+07	5.8E+07	4.5E+07
Prevalence	Global	Both	55-59 yea	Sexually tr	Rate	1995	25575.2	29121.7	22351.5
Prevalence	Global	Both	55-59 yea	Sexually tr	Number	1996	5.2E+07	5.9E+07	4.5E+07
Prevalence	Global	Both	55-59 yea	Sexually tr	Rate	1996	25557.2	29113	22338.6
Prevalence	Global	Both	55-59 yea	Sexually tr	Number	1997	5.2E+07	5.9E+07	4.5E+07
Prevalence	Global	Both	55-59 yea	Sexually tr	Rate	1997	25470.2	29005.3	22263
Prevalence	Global	Both	55-59 yea	Sexually tr	Number	1998	5.2E+07	5.9E+07	4.5E+07
Prevalence	Global	Both	55-59 yea	Sexually tr	Rate	1998	25344.5	28881.1	22157.5
Prevalence	Global	Both	55-59 yea	Sexually tr	Number	1999	5.2E+07	5.9E+07	4.5E+07
Prevalence	Global	Both	55-59 yea	Sexually tr	Rate	1999	25238.6	28725.6	22055.3
Prevalence	Global	Both	55-59 yea	Sexually tr	Number	2000	5.2E+07	5.9E+07	4.5E+07
Prevalence	Global	Both	55-59 yea	Sexually tr	Rate	2000	25216.1	28693.2	22066.6
Prevalence	Global	Both	55-59 yea	Sexually tr	Number	2001	5.3E+07	6E+07	4.6E+07
Prevalence	Global	Both	55-59 yea	Sexually tr	Rate	2001	25255.4	28769.6	22119.7
Prevalence	Global	Both	55-59 yea	Sexually tr	Number	2002	5.5E+07	6.2E+07	4.8E+07
Prevalence	Global	Both	55-59 yea	Sexually tr	Rate	2002	25218.1	28685.4	22056.3
Prevalence	Global	Both	55-59 yea	Sexually tr	Number	2003	5.7E+07	6.5E+07	5E+07
Prevalence	Global	Both	55-59 yea	Sexually tr	Rate	2003	25194.4	28667.9	22013.3
Prevalence	Global	Both	55-59 yea	Sexually tr	Number	2004	6E+07	6.9E+07	5.2E+07
Prevalence	Global	Both	55-59 yea	Sexually tr	Rate	2004	25138.7	28652.7	21908.9
Prevalence	Global	Both	55-59 yea	Sexually tr	Number	2005	6.3E+07	7.2E+07	5.5E+07
Prevalence	Global	Both	55-59 yea	Sexually tr	Rate	2005	25140.4	28693.9	21934.9
Prevalence	Global	Both	55-59 yea	Sexually tr	Number	2006	6.6E+07	7.5E+07	5.7E+07
Prevalence	Global	Both	55-59 yea	Sexually tr	Rate	2006	25067.6	28580.7	21819.9
Prevalence	Global	Both	55-59 yea	Sexually tr	Number	2007	6.8E+07	7.7E+07	5.9E+07
Prevalence	Global	Both	55-59 yea	Sexually tr	Rate	2007	24940.6	28398.6	21674.6
Prevalence	Global	Both	55-59 yea	Sexually tr	Number	2008	7E+07	8E+07	6.1E+07
Prevalence	Global	Both	55-59 yea	Sexually tr	Rate	2008	24758.4	28229	21467.9
Prevalence	Global	Both	55-59 yea	Sexually tr	Number	2009	7.2E+07	8.2E+07	6.2E+07
Prevalence	Global	Both	55-59 yea	Sexually tr	Rate	2009	24632.9	28104.6	21357.8
Prevalence	Global	Both	55-59 yea	Sexually tr	Number	2010	7.5E+07	8.5E+07	6.4E+07
Prevalence	Global	Both	55-59 yea	Sexually tr	Rate	2010	24566.8	28105.4	21253
Prevalence	Global	Both	55-59 yea	Sexually tr	Number	2011	7.7E+07	8.8E+07	6.6E+07
Prevalence	Global	Both	55-59 yea	Sexually tr	Rate	2011	24660	28156.7	21331.1
Prevalence	Global	Both	55-59 yea	Sexually tr	Number	2012	7.9E+07	9E+07	6.8E+07
Prevalence	Global	Both	55-59 yea	Sexually tr	Rate	2012	24843.5	28400.9	21507.4
Prevalence	Global	Both	55-59 yea	Sexually tr	Number	2013	8.1E+07	9.3E+07	7E+07
Prevalence	Global	Both	55-59 yea	Sexually tr	Rate	2013	25069.4	28710.6	21730.5
Prevalence	Global	Both	55-59 yea	Sexually tr	Number	2014	8.2E+07	9.4E+07	7.2E+07
Prevalence	Global	Both	55-59 yea	Sexually tr	Rate	2014	25269.7	28966.4	21957
Prevalence	Global	Both	55-59 yea	Sexually tr	Number	2015	8.4E+07	9.6E+07	7.3E+07
Prevalence	Global	Both	55-59 yea	Sexually tr	Rate	2015	25401.9	29125.6	22051.3
Prevalence	Global	Both	55-59 yea	Sexually tr	Number	2016	8.6E+07	9.8E+07	7.4E+07
Prevalence	Global	Both	55-59 yea	Sexually tr	Rate	2016	25494.5	29257.2	22127.2
Prevalence	Global	Both	55-59 yea	Sexually tr	Number	2017	8.8E+07	1E+08	7.6E+07
Prevalence	Global	Both	55-59 yea	Sexually tr	Rate	2017	25548.2	29333.4	22140.8
Prevalence	Global	Both	55-59 yea	Sexually tr	Number	2018	9.1E+07	1E+08	7.9E+07
Prevalence	Global	Both	55-59 yea	Sexually tr	Rate	2018	25543.3	29351.7	22110.6
Prevalence	Global	Both	55-59 yea	Sexually tr	Number	2019	9.5E+07	1.1E+08	8.2E+07
Prevalence	Global	Both	55-59 yea	Sexually tr	Rate	2019	25525	29333.4	22120.1

Prevalence	Global	Both	60-64 yea	Sexually tr	Rate	2007	23340	26773.4	20348.5
Prevalence	Global	Both	60-64 yea	Sexually tr	Number	2008	5E+07	5.8E+07	4.4E+07
Prevalence	Global	Both	60-64 yea	Sexually tr	Rate	2008	23132.5	26560.7	20140.2
Prevalence	Global	Both	60-64 yea	Sexually tr	Number	2009	5.3E+07	6E+07	4.6E+07
Prevalence	Global	Both	60-64 yea	Sexually tr	Rate	2009	22917.3	26367	19908.7
Prevalence	Global	Both	60-64 yea	Sexually tr	Number	2010	5.5E+07	6.3E+07	4.7E+07
Prevalence	Global	Both	60-64 yea	Sexually tr	Rate	2010	22851.5	26295.8	19827.4
Prevalence	Global	Both	60-64 yea	Sexually tr	Number	2011	5.8E+07	6.6E+07	5E+07
Prevalence	Global	Both	60-64 yea	Sexually tr	Rate	2011	22882.9	26312.7	19896.7
Prevalence	Global	Both	60-64 yea	Sexually tr	Number	2012	6E+07	6.9E+07	5.2E+07
Prevalence	Global	Both	60-64 yea	Sexually tr	Rate	2012	23011.5	26475.2	20018.5
Prevalence	Global	Both	60-64 yea	Sexually tr	Number	2013	6.3E+07	7.2E+07	5.4E+07
Prevalence	Global	Both	60-64 yea	Sexually tr	Rate	2013	23154.5	26632.7	20117.3
Prevalence	Global	Both	60-64 yea	Sexually tr	Number	2014	6.5E+07	7.5E+07	5.7E+07
Prevalence	Global	Both	60-64 yea	Sexually tr	Rate	2014	23316.7	26837.9	20256.1
Prevalence	Global	Both	60-64 yea	Sexually tr	Number	2015	6.8E+07	7.8E+07	5.9E+07
Prevalence	Global	Both	60-64 yea	Sexually tr	Rate	2015	23397.8	26969.3	20314.1
Prevalence	Global	Both	60-64 yea	Sexually tr	Number	2016	7E+07	8.1E+07	6.1E+07
Prevalence	Global	Both	60-64 yea	Sexually tr	Rate	2016	23463.7	27022.3	20349
Prevalence	Global	Both	60-64 yea	Sexually tr	Number	2017	7.2E+07	8.3E+07	6.2E+07
Prevalence	Global	Both	60-64 yea	Sexually tr	Rate	2017	23538.6	27150.4	20372.4
Prevalence	Global	Both	60-64 yea	Sexually tr	Number	2018	7.3E+07	8.4E+07	6.3E+07
Prevalence	Global	Both	60-64 yea	Sexually tr	Rate	2018	23628	27260.4	20471.5
Prevalence	Global	Both	60-64 yea	Sexually tr	Number	2019	7.4E+07	8.5E+07	6.4E+07
Prevalence	Global	Both	60-64 yea	Sexually tr	Rate	2019	23736.1	27349.5	20577.3
Prevalence	Global	Both	65-69 yea	Sexually tr	Number	1990	2.8E+07	3.2E+07	2.4E+07
Prevalence	Global	Both	65-69 yea	Sexually tr	Rate	1990	22317.3	25721.7	19441
Prevalence	Global	Both	65-69 yea	Sexually tr	Number	1991	2.9E+07	3.3E+07	2.5E+07
Prevalence	Global	Both	65-69 yea	Sexually tr	Rate	1991	22471.1	25865.3	19604.4
Prevalence	Global	Both	65-69 yea	Sexually tr	Number	1992	3E+07	3.4E+07	2.6E+07
Prevalence	Global	Both	65-69 yea	Sexually tr	Rate	1992	22566.6	25904	19702
Prevalence	Global	Both	65-69 yea	Sexually tr	Number	1993	3.1E+07	3.5E+07	2.7E+07
Prevalence	Global	Both	65-69 yea	Sexually tr	Rate	1993	22620.6	25934.7	19776.8
Prevalence	Global	Both	65-69 yea	Sexually tr	Number	1994	3.1E+07	3.6E+07	2.7E+07
Prevalence	Global	Both	65-69 yea	Sexually tr	Rate	1994	22636.7	25890.8	19801.3
Prevalence	Global	Both	65-69 yea	Sexually tr	Number	1995	3.2E+07	3.7E+07	2.8E+07
Prevalence	Global	Both	65-69 yea	Sexually tr	Rate	1995	22627.7	25913.1	19790.9
Prevalence	Global	Both	65-69 yea	Sexually tr	Number	1996	3.3E+07	3.7E+07	2.9E+07
Prevalence	Global	Both	65-69 yea	Sexually tr	Rate	1996	22545.9	25825	19712.2
Prevalence	Global	Both	65-69 yea	Sexually tr	Number	1997	3.3E+07	3.8E+07	2.9E+07
Prevalence	Global	Both	65-69 yea	Sexually tr	Rate	1997	22392.9	25651.6	19587.5
Prevalence	Global	Both	65-69 yea	Sexually tr	Number	1998	3.3E+07	3.8E+07	2.9E+07
Prevalence	Global	Both	65-69 yea	Sexually tr	Rate	1998	22219.6	25494.4	19429.6
Prevalence	Global	Both	65-69 yea	Sexually tr	Number	1999	3.3E+07	3.8E+07	2.9E+07
Prevalence	Global	Both	65-69 yea	Sexually tr	Rate	1999	22073.5	25371.3	19288.3
Prevalence	Global	Both	65-69 yea	Sexually tr	Number	2000	3.4E+07	3.9E+07	3E+07
Prevalence	Global	Both	65-69 yea	Sexually tr	Rate	2000	22015.6	25334.5	19286.1
Prevalence	Global	Both	65-69 yea	Sexually tr	Number	2001	3.4E+07	3.9E+07	3E+07
Prevalence	Global	Both	65-69 yea	Sexually tr	Rate	2001	22028.8	25283.2	19277.1
Prevalence	Global	Both	65-69 yea	Sexually tr	Number	2002	3.5E+07	4E+07	3.1E+07
Prevalence	Global	Both	65-69 yea	Sexually tr	Rate	2002	22056.7	25305.8	19275.3
Prevalence	Global	Both	65-69 yea	Sexually tr	Number	2003	3.6E+07	4.1E+07	3.1E+07
Prevalence	Global	Both	65-69 yea	Sexually tr	Rate	2003	22099.6	25321.7	19319.6
Prevalence	Global	Both	65-69 yea	Sexually tr	Number	2004	3.7E+07	4.2E+07	3.2E+07
Prevalence	Global	Both	65-69 yea	Sexually tr	Rate	2004	22144.2	25387.1	19353.8
Prevalence	Global	Both	65-69 yea	Sexually tr	Number	2005	3.7E+07	4.3E+07	3.3E+07
Prevalence	Global	Both	65-69 yea	Sexually tr	Rate	2005	22182.1	25431	19381.2
Prevalence	Global	Both	65-69 yea	Sexually tr	Number	2006	3.8E+07	4.3E+07	3.3E+07
Prevalence	Global	Both	65-69 yea	Sexually tr	Rate	2006	22148.1	25394.7	19314
Prevalence	Global	Both	65-69 yea	Sexually tr	Number	2007	3.8E+07	4.4E+07	3.3E+07
Prevalence	Global	Both	65-69 yea	Sexually tr	Rate	2007	22049.4	25336.1	19201.6
Prevalence	Global	Both	65-69 yea	Sexually tr	Number	2008	3.8E+07	4.4E+07	3.3E+07
Prevalence	Global	Both	65-69 yea	Sexually tr	Rate	2008	21907.9	25186.6	19101.1
Prevalence	Global	Both	65-69 yea	Sexually tr	Number	2009	3.8E+07	4.4E+07	3.3E+07
Prevalence	Global	Both	65-69 yea	Sexually tr	Rate	2009	21778.7	25082.1	18961.6
Prevalence	Global	Both	65-69 yea	Sexually tr	Number	2010	3.9E+07	4.5E+07	3.4E+07
Prevalence	Global	Both	65-69 yea	Sexually tr	Rate	2010	21711.9	25020.2	18899
Prevalence	Global	Both	65-69 yea	Sexually tr	Number	2011	4E+07	4.6E+07	3.5E+07
Prevalence	Global	Both	65-69 yea	Sexually tr	Rate	2011	21736.5	25041.5	18941.6
Prevalence	Global	Both	65-69 yea	Sexually tr	Number	2012	4.2E+07	4.8E+07	3.6E+07
Prevalence	Global	Both	65-69 yea	Sexually tr	Rate	2012	21770	25045.6	18956.2
Prevalence	Global	Both	65-69 yea	Sexually tr	Number	2013	4.4E+07	5E+07	3.8E+07
Prevalence	Global	Both	65-69 yea	Sexually tr	Rate	2013	21845.4	25128.7	18998.6
Prevalence	Global	Both	65-69 yea	Sexually tr	Number	2014	4.6E+07	5.3E+07	4E+07
Prevalence	Global	Both	65-69 yea	Sexually tr	Rate	2014	21888.7	25181.1	19007.7
Prevalence	Global	Both	65-69 yea	Sexually tr	Number	2015	4.8E+07	5.6E+07	4.2E+07
Prevalence	Global	Both	65-69 yea	Sexually tr	Rate	2015	21930.3	25246.6	19039.2
Prevalence	Global	Both	65-69 yea	Sexually tr	Number	2016	5.1E+07	5.9E+07	4.4E+07
Prevalence	Global	Both	65-69 yea	Sexually tr	Rate	2016	21946.4	25321.1	19040.3
Prevalence	Global	Both	65-69 yea	Sexually tr	Number	2017	5.3E+07	6.1E+07	4.6E+07
Prevalence	Global	Both	65-69 yea	Sexually tr	Rate	2017	21999.8	25434.4	19027.9
Prevalence	Global	Both	65-69 yea	Sexually tr	Number	2018	5.5E+07	6.4E+07	4.8E+07
Prevalence	Global	Both	65-69 yea	Sexually tr	Rate	2018	22035.7	25524.3	19093.4
Prevalence	Global	Both	65-69 yea	Sexually tr	Number	2019	5.7E+07	6.6E+07	4.9E+07
Prevalence	Global	Both	65-69 yea	Sexually tr	Rate	2019	22105.1	25597.9	19140.7
Prevalence	Global	Both	50-69 yea	Sexually tr	Number	1990	1.7E+08	1.9E+08	1.5E+08
Prevalence	Global	Both	50-69 yea	Sexually tr	Rate	1990	24552.2	28013.9	21479
Prevalence	Global	Both	50-69 yea	Sexually tr	Number	1991	1.7E+08	2E+08	1.5E+08
Prevalence	Global	Both	50-69 yea	Sexually tr	Rate	1991	24751.7	28196	21691.9
Prevalence	Global	Both	50-69 yea	Sexually tr	Number	1992	1.8E+08	2E+08	1.5E+08
Prevalence	Global	Both	50-69 yea	Sexually tr	Rate	1992	24910.5	28349.6	21833
Prevalence	Global	Both	50-69 yea	Sexually tr	Number	1993	1.8E+08	2E+08	1.6E+08
Prevalence	Global	Both	50-69 yea	Sexually tr	Rate	1993	25023.7	28486.1	21924
Prevalence	Global	Both	50-69 yea	Sexually tr	Number	1994	1.8E+08	2.1E+08	1.6E+08
Prevalence	Global	Both	50-69 yea	Sexually tr	Rate	1994	25098.9	28540	22029.5
Prevalence	Global	Both	50-69 yea	Sexually tr	Number	1995	1.8E+08	2.1E+08	1.6E+08
Prevalence	Global	Both	50-69 yea	Sexually tr	Rate	1995	25142.3	28563.9	22043.1
Prevalence	Global	Both	50-69 yea	Sexually tr	Number	1996	1.9E+08	2.1E+08	1.6E+08
Prevalence	Global	Both	50-69 yea	Sexually tr	Rate	1996	25111	28552.6	22007.1
Prevalence	Global	Both	50-69 yea	Sexually tr	Number	1997	1.9E+08	2.2E+08	1.7E+08
Prevalence	Global	Both	50-69 yea	Sexually tr	Rate	1997	24987.7	28422	21902.7
Prevalence	Global	Both	50-69 yea	Sexually tr	Number	1998	1.9E+08	2.2E+08	1.7E+08
Prevalence	Global	Both	50-69 yea	Sexually tr	Rate	1998	24839.7	28261.8	21779.7
Prevalence	Global	Both	50-69 yea	Sexually tr	Number	1999	2E+08	2.2E+08	1.7E+08
Prevalence	Global	Both	50-69 yea	Sexually tr	Rate	1999	24718	28106.4	21691
Prevalence	Global	Both	50-69 yea	Sexually tr	Number	2000	2E+08	2.3E+08	1.8E+08
Prevalence	Global	Both	50-69 yea	Sexually tr	Rate	2000	24689.1	28089.1	21687.5
Prevalence	Global	Both	50-69 yea	Sexually tr	Number	2001	2.1E+08	2.3E+08	1.8E+08
Prevalence	Global	Both	50-69 yea	Sexually tr	Rate	2001	24734.5	28110.3	21724.3
Prevalence	Global	Both	50-69 yea	Sexually tr	Number	2002	2.1E+08	2.4E+08	1.9E+08
Prevalence	Global	Both	50-69 yea	Sexually tr	Rate	2002	24775.1	28176.1	21756.4
Prevalence	Global	Both	50-69 yea	Sexually tr	Number	2003	2.2E+08	2.5E+08	1.9E+08
Prevalence	Global	Both	50-69 yea	Sexually tr	Rate	2003	24816.5	28248.6	21784.2
Prevalence	Global	Both	50-69 yea	Sexually tr	Number	2004	2.2E+08	2.6E+08	2E+08
Prevalence	Global	Both	50-69 yea	Sexually tr	Rate	2004	24853.6	28296.7	21787.5
Prevalence	Global	Both	50-69 yea	Sexually tr	Number	2005	2.3E+08	2.6E+08	2E+08
Prevalence	Global	Both	50-69 yea	Sexually tr	Rate	2005	24894.3	28318.6	21817.8
Prevalence	Global	Both	50-69 yea	Sexually tr	Number	2006	2.4E+08	2.7E+08	2.1E+08
Prevalence	Global	Both	50-69 yea	Sexually tr	Rate	2006	24854.6	28298	21772.2
Prevalence	Global	Both	50-69 yea	Sexually tr	Number	2007	2.4E+08	2.8E+08	2.1E+08
Prevalence	Global	Both	50-69 yea	Sexually tr	Rate	2007	24715	28166.6	21637.5
Prevalence	Global	Both	50-69 yea	Sexually tr	Number	2008	2.5E+08	2.8E+08	2.2E+08
Prevalence	Global	Both	50-69 yea	Sexually tr	Rate	2008	24542.6	27984.3	21450.8
Prevalence	Global	Both	50-69 yea	Sexually tr	Number	2009	2.5E+08	2.9E+08	2.2E+08
Prevalence	Global	Both	50-69 yea	Sexually tr	Rate	2009	24398.7	27871.9	21287.2
Prevalence	Global	Both	50-69 yea	Sexually tr	Number	2010	2.6E+08	3E+08	2.3E+08
Prevalence	Global	Both	50-69 yea	Sexually tr	Rate	2010	24357	27850	21217.1
Prevalence	Global	Both	50-69 yea	Sexually tr	Number	2011	2.7E+08	3.1E+08	2.3E+08
Prevalence	Global	Both	50-69 yea	Sexually tr	Rate	2011	24417.3	27929.1	21263.2
Prevalence	Global	Both	50-69 yea	Sexually tr	Number	2012	2.8E+08	3.2E+08	2.4E+08
Prevalence	Global	Both	50-69 yea	Sexually tr	Rate	2012	24525.3	28062.3	21356.3
Prevalence	Global	Both	50-69 yea	Sexually tr	Number	2013	2.9E+08	3.3E+08	2.5E+08
Prevalence	Global	Both	50-69 yea	Sexually tr	Rate	2013	24649.4	28217.8	21463.9
Prevalence	Global	Both	50-69 yea	Sexually tr	Number	2014	3E+08	3.4E+08	2.6E+08
Prevalence	Global	Both	50-69 yea	Sexually tr	Rate	2014	24755.9	28338	21552.9
Prevalence	Global	Both	50-69 yea	Sexually tr	Number	2015	3.1E+08	3.5E+08	2.7E+08
Prevalence	Global	Both	50-69 yea	Sexually tr	Rate	2015	24810.2	28397.7	21633.9
Prevalence	Global	Both	50-69 yea	Sexually tr	Number	2016	3.2E+08	3.6E+08	2.8E+08
Prevalence	Global	Both	50-69 yea	Sexually tr	Rate	2016	24847.1	28442.5	21628.6
Prevalence	Global	Both	50-69 yea	Sexually tr	Number	2017	3.3E+08	3.8E+08	2.8E+08
Prevalence	Global	Both	50-69 yea	Sexually tr	Rate	2017	24891.3	28566	21655.4
Prevalence	Global	Both	50-69 yea	Sexually tr	Number	2018	3.4E+08	3.9E+08	2.9E+08
Prevalence	Global	Both	50-69 yea	Sexually tr	Rate	2018	24922.1	28611.6	21690.9
Prevalence	Global	Both	50-69 yea	Sexually tr	Number	2019	3.4E+08	4E+08	3E+08
Prevalence	Global	Both	50-69 yea	Sexually tr	Rate	2019	24975.6	28652.4	21715.4
Prevalence	Global	Both	50-54 yea	Genital her	Number	1990	4.9E+07	5.7E+07	4.1E+07
Prevalence	Global	Both	50-54 yea	Genital her	Rate	1990	22956.5	27008.7	19471.4
Prevalence	Global	Both	50-54 yea	Genital her	Number	1991	5E+07	5.9E+07	4.2E+07
Prevalence	Global	Both	50-54 yea	Genital her	Rate	1991	23222.8	27295.9	19737.4
Prevalence	Global	Both	50-54 yea	Genital her	Number	1992	5.1E+07	6E+07	4.3E+07
Prevalence	Global	Both	50-54 yea	Genital her	Rate	1992	23451.5	27521.7	19962.6
Prevalence	Global	Both	50-54 yea	Genital her	Number	1993	5.1E+07	6E+07	4.4E+07
Prevalence	Global	Both	50-54 yea	Genital her	Rate	1993	23629.8	27697.4	20151.6
Prevalence	Global	Both	50-54 yea	Genital her	Number	1994	5.2E+07	6.1E+07	4.4E+07
Prevalence	Global	Both	50-54 yea	Genital her	Rate	1994	23760.2	27808	20290.7
Prevalence	Global	Both	50-54 yea	Genital her	Number	1995	5.2E+07	6.1E+07	4.5E+07
Prevalence	Global	Both	50-54 yea	Genital her	Rate	1995	23847.3	27860.8	20336.1
Prevalence	Global	Both	50-54 yea	Genital her	Number	1996	5.3E+07	6.2E+07	4.5E+07
Prevalence	Global	Both	50-54 yea	Genital her	Rate	1996	23845.9	27895.2	20327
Prevalence	Global	Both	50-54 yea	Genital her	Number	1997	5.5E+07	6.4E+07	4.7E+07
Prevalence	Global	Both	50-54 yea	Genital her	Rate	1997	23666.3	27694.1	20184.8
Prevalence	Global	Both	50-54 yea	Genital her	Number	1998	5.6E+07	6.6E+07	4.8E+07
Prevalence	Global	Both	50-54 yea	Genital her	Rate	1998	23456.4	27391.9	19999.4
Prevalence	Global	Both	50-54 yea	Genital her	Number	1999	5.9E+07	6.8E+07	5E+07
Prevalence	Global	Both	50-54 yea	Genital her	Rate	1999	23242.1	27112.4	19811.1
Prevalence	Global	Both	50-54 yea	Genital her	Number	2000	6.1E+07	7.1E+07	5.2E+07
Prevalence	Global	Both	50-54 yea	Genital her	Rate	2000	23171.2	26993.3	19747.2
Prevalence	Global	Both	50-54 yea	Genital her	Number	2001	6.4E+07	7.4E+07	5.4E+07
Prevalence	Global	Both	50-54 yea	Genital her	Rate	2001	23173.9	27024.7	19760
Prevalence	Global	Both	50-54 yea	Genital her	Number	2002	6.6E+07	7.7E+07	5.6E+07
Prevalence	Global	Both	50-54 yea	Genital her	Rate	2002	23216.1	27112.9	19811.2
Prevalence	Global	Both	50-54 yea	Genital her	Number	2003	6.9E+07	8E+07	5.8E+07
Prevalence	Global	Both	50-54 yea	Genital her	Rate	2003	23242.8	27188.1	19811.9
Prevalence	Global	Both	50-54 yea	Genital her	Number	2004	7.1E+07	8.3E+07	6E+07
Prevalence	Global	Both	50-54 yea	Genital her	Rate	2004	23300.2	27275.8	19848.1
Prevalence	Global	Both	50-54 yea	Genital her	Number	2005	7.4E+07	8.7E+07	6.3E+07
Prevalence	Global	Both	50-54 yea	Genital her	Rate	2005	23320.9	27327.3	19875.5
Prevalence	Global	Both	50-54 yea	Genital her	Number	2006	7.6E+07	8.9E+07	6.4E+07
Prevalence	Global	Both	50-54 yea	Genital her	Rate	2006	23294.6	27297.4	19842.2
Prevalence	Global	Both	50-54 yea	Genital her	Number	2007	7.7E+07	9E+07	6.5E+07
Prevalence	Global	Both	50-54 yea	Genital her	Rate	2007	23180.2	27191.2	19735.8
Prevalence	Global	Both	50-54 yea	Genital her	Number	2008	7.7E+07	9.1E+07	6.6E+07
Prevalence	Global	Both	50-54 yea	Genital her	Rate	2008	23068.2	27109.4	19628.2
Prevalence	Global	Both	50-54 yea	Genital her	Number	2009	7.8E+07	9.2E+07	6.6E+07
Prevalence	Global	Both	50-54 yea	Genital her	Rate	2009	23017.2	27092.1	19536.1
Prevalence	Global	Both	50-54 yea	Genital her	Number	2010	7.9E+07	9.3E+07	6.7E+07
Prevalence	Global	Both	50-54 yea	Genital her	Rate	2010	23068.2	27176.6	19544.3
Prevalence	Global	Both	50-54 yea	Genital her	Number	2011	8.1E+07	9.5E+07	6.9E+07
Prevalence	Global	Both	50-54 yea	Genital her	Rate	2011	23187.3	27295.6	19643.3
Prevalence	Global	Both	50-54 yea	Genital her	Number	2012	8.3E+07	9.8E+07	7.1E+07
Prevalence	Global	Both	50-54 yea	Genital her	Rate	2012	23297.4	27408	19711.4
Prevalence	Global	Both	50-54 yea	Genital her	Number	2013	8.7E+07	1E+08	7.3E+07
Prevalence	Global	Both	50-54 yea	Genital her	Rate	2013	23371.1	27487.9	19763.1
Prevalence	Global	Both	50-54 yea	Genital her	Number	2014	9E+07	1.1E+08	7.6E+07
Prevalence	Global	Both	50-54 yea	Genital her	Rate	2014	23416.6	27526.9	19756.9
Prevalence	Global	Both	50-54 yea	Genital her	Number	2015	9.4E+07	1.1E+08	7.9E+07
Prevalence	Global	Both	50-54 yea	Genital her	Rate	2015	23408.7	27534.8	19752.9
Prevalence	Global	Both	50-54 yea	Genital her	Number	2016	9.6E+07	1.1E+08	8.1E+07
Prevalence	Global	Both	50-54 yea	Genital her	Rate	2016	23395.7	27547.2	19747.9
Prevalence	Global	Both	50-54 yea	Genital her	Number	2017	9.9E+07	1.2E+08	8.3E+07
Prevalence	Global	Both	50-54 yea	Genital her	Rate	2017	23389.7	27639.2	19702.2
Prevalence	Global	Both	50-54 yea	Genital her	Number	2018	1E+08	1.2E+08	8.5E+07
Prevalence	Global	Both	50-54 yea	Genital her	Rate	2018	23388.8	27599.3	19709.9
Prevalence	Global	Both	50-54 yea	Genital her	Number	2019	1E+08	1.2E+08	8.6E+07
Prevalence	Global	Both	50-54 yea	Genital her	Rate	2019	23418	27598.1	19724
Prevalence	Global	Both	55-59 yea	Genital her	Number	1990	4.2E+07	4.9E+07	3.6E+07
Prevalence	Global	Both	55-59 yea	Genital her	Rate	1990	22508.3	26255.5	19273.1
Prevalence	Global	Both	55-59 yea	Genital her	Number	1991	4.3E+07	5E+07	3.7E+07
Prevalence	Global	Both	55-59 yea	Genital her	Rate	1991	22754	26519.1	19507.5
Prevalence	Global	Both	55-59 yea	Genital her	Number	1992	4.4E+07	5.1E+07	3.8E+07
Prevalence	Global	Both	55-59 yea	Genital her	Rate	1992	22955.7	26738.5	19677.3
Prevalence	Global	Both	55-59 yea	Genital her	Number	1993	4.5E+07	5.2E+07	3.9E+07
Prevalence	Global	Both	55-59 yea	Genital her	Rate	1993	23113.3	26875.2	19814.7
Prevalence	Global	Both	55-59 yea	Genital her	Number	1994	4.6E+07	5.3E+07	3.9E+07
Prevalence	Global	Both	55-59 yea	Genital her	Rate	1994	23228.3	26965.3	19923.5
Prevalence	Global	Both	55-59 yea	Genital her	Number	1995	4.7E+07	5.4E+07	4E+07
Prevalence	Global	Both	55-59 yea	Genital her	Rate	1995	23303	27041.3	19998.6
Prevalence	Global	Both	55-59 yea	Genital her	Number	1996	4.7E+07	5.5E+07	4E+07
Prevalence	Global	Both	55-59 yea	Genital her	Rate	1996	23287.2	27022.1	19993.9
Prevalence	Global	Both	55-59 yea	Genital her	Number	1997	4.7E+07	5.5E+07	4E+07
Prevalence	Global	Both	55-59 yea	Genital her	Rate	1997	23201.8	26918.6	19931.8
Prevalence	Global	Both	55-59 yea	Genital her	Number	1998	4.7E+07	5.5E+07	4E+07
Prevalence	Global	Both	55-59 yea	Genital her	Rate	1998	23080.4	26766.5	19846.5
Prevalence	Global	Both	55-59 yea	Genital her	Number	1999	4.7E+07	5.5E+07	4E+07
Prevalence	Global	Both	55-59 yea	Genital her	Rate	1999	22976.8	26634.1	19763.8
Prevalence	Global	Both	55-59 yea	Genital her	Number	2000	4.7E+07	5.5E+07	4.1E+07
Prevalence	Global	Both	55-59 yea	Genital her	Rate	2000	22955.3	26620.9	19759.7
Prevalence	Global	Both	55-59 yea	Genital her	Number	2001	4.8E+07	5.6E+07	4.1E+07
Prevalence	Global	Both	55-59 yea	Genital her	Rate	2001	22993.7	26659	19790.5
Prevalence	Global	Both	55-59 yea	Genital her	Number	2002	5E+07	5.8E+07	4.3E+07
Prevalence	Global	Both	55-59 yea	Genital her	Rate	2002	22966.5	26646.8	19760
Prevalence	Global	Both	55-59 yea	Genital her	Number	2003	5.2E+07	6.1E+07	4.5E+07
Prevalence	Global	Both	55-59 yea	Genital her	Rate	2003	22948.8	26656.2	19733.5
Prevalence	Global	Both	55-59 yea	Genital her	Number	2004	5.5E+07	6.4E+07	4.7E+07
Prevalence	Global	Both	55-59 yea	Genital her	Rate	2004	22899.6	26630.2	19672.1
Prevalence	Global	Both	55-59 yea	Genital her	Number	2005	5.7E+07	6.7E+07	4.9E+07
Prevalence	Global	Both	55-59 yea	Genital her	Rate	2005	22905.9	26649.5	19670.4
Prevalence	Global	Both	55-59 yea	Genital her	Number	2006	6E+07	7E+07	5.1E+07
Prevalence	Global	Both	55-59 yea	Genital her	Rate	2006	22833	26557.6	19598.7
Prevalence	Global	Both	55-59 yea	Genital her	Number	2007	6.2E+07	7.2E+07	5.3E+07
Prevalence	Global	Both	55-59 yea	Genital her	Rate	2007	22701	26416.3	19466.6
Prevalence	Global	Both	55-59 yea	Genital her	Number	2008	6.3E+07	7.4E+07	5.4E+07
Prevalence	Global	Both	55-59 yea	Genital her	Rate	2008	22515	26241.9	19280.6
Prevalence	Global	Both	55-59 yea	Genital her	Number	2009	6.5E+07	7.6E+07	5.6E+07
Prevalence	Global	Both	55-59 yea	Genital her	Rate	2009	22386.2	26144	19126.6
Prevalence	Global	Both	55-59 yea	Genital her	Number	2010	6.8E+07	7.9E+07	5.8E+07
Prevalence	Global	Both	55-59 yea	Genital her	Rate	2010	22314.1	26095.4	19032.6
Prevalence	Global	Both	55-59 yea	Genital her	Number	2011	7E+07	8.1E+07	5.9E+07
Prevalence	Global	Both	55-59 yea	Genital her	Rate	2011	22404.8	26153.7	19119.2
Prevalence	Global	Both	55-59 yea	Genital her	Number	2012	7.2E+07	8.4E+07	6.1E+07
Prevalence	Global	Both	55-59 yea	Genital her	Rate	2012	22586.8	26350	19274.5
Prevalence	Global	Both	55-59 yea	Genital her	Number	2013	7.4E+07	8.6E+07	6.3E+07
Prevalence	Global	Both	55-59 yea	Genital her	Rate	2013	22814.7	26609.7	19462.2
Prevalence	Global	Both	55-59 yea	Genital her	Number	2014	7.5E+07	8.8E+07	6.4E+07
Prevalence	Global	Both	55-59 yea	Genital her	Rate	2014	23018.6	26848.2	19608.4
Prevalence	Global	Both	55-59 yea	Genital her	Number	2015	7.6E+07	8.9E+07	6.5E+07
Prevalence	Global	Both	55-59 yea	Genital her	Rate	2015	23154.2	27009.5	19713.5
Prevalence	Global	Both	55-59 yea	Genital her	Number	2016	7.8E+07	9.1E+07	6.6E+07
Prevalence	Global	Both	55-59 yea	Genital her	Rate	2016	23246.6	27136.2	19774.2
Prevalence	Global	Both	55-59 yea	Genital her	Number	2017	8E+07	9.4E+07	6.8E+07
Prevalence	Global	Both	55-59 yea	Genital her	Rate	2017	23297.8	27252.3	19773.4
Prevalence	Global	Both	55-59 yea	Genital her	Number	2018	8.3E+07	9.7E+07	7.1E+07
Prevalence	Global	Both	55-59 yea	Genital her	Rate	2018	23284.1	27236.9	19763.1
Prevalence	Global	Both	55-59 yea	Genital her	Number	2019	8.6E+07	1E+08	7.3E+07
Prevalence	Global	Both	55-59 yea	Genital her	Rate	2019	23255.9	27211.5	19779.4
Prevalence	Global	Both	60-64 yea	Genital her	Number	1990	3.5E+07	4.1E+07	3E+07
Prevalence	Global	Both	60-64 yea	Genital her	Rate	1990	21865.4	25520.1	18823.7
Prevalence	Global	Both	60-64 yea	Genital her	Number	1991	3.6E+07	4.2E+07	3.1E+07
Prevalence	Global	Both	60-64 yea	Genital her	Rate	1991	22032.3	25690.9	19004.5
Prevalence	Global	Both	60-64 yea	Genital her	Number	1992	3.7E+07	4.3E+07	3.2E+07
Prevalence	Global	Both	60-64 yea	Genital her	Rate	1992	22168.4	25799.8	19149.3
Prevalence	Global	Both	60-64 yea	Genital her	Number	1993	3.7E+07	4.4E+07	3.2E+07
Prevalence	Global	Both	60-64 yea	Genital her	Rate	1993	22270.1	25873.9	19255.4
Prevalence	Global	Both	60-64 yea	Genital her	Number	1994	3.8E+07	4.4E+07	3.3E+07
Prevalence	Global	Both	60-64 yea	Genital her	Rate	1994	22342.2	25916.9	19329.3
Prevalence	Global	Both	60-64 yea	Genital her	Number	1995	3.8E+07	4.5E+07	3.3E+07
Prevalence	Global	Both	60-64 yea	Genital her	Rate	1995	22382.2	25937.9	19368.3
Prevalence	Global	Both	60-64 yea	Genital her	Number	1996	3.9E+07	4.5E+07	3.4E+07
Prevalence	Global	Both	60-64 yea	Genital her	Rate	1996	22343.1	25898.6	19336.1
Prevalence	Global	Both	60-64 yea	Genital her	Number	1997	4E+07	4.6E+07	3.4E+07
Prevalence	Global	Both	60-64 yea	Genital her	Rate	1997	22221.4	25762.8	19232.2
Prevalence	Global	Both	60-64 yea	Genital her	Number	1998	4E+07	4.6E+07	3.5E+07
Prevalence	Global	Both	60-64 yea	Genital her	Rate	1998	22077.9	25599.2	19110.4
Prevalence	Global	Both	60-64 yea	Genital her	Number	1999	4E+07	4.7E+07	3.5E+07
Prevalence	Global	Both	60-64 yea	Genital her	Rate	1999	21973.4	25485.3	19023.4
Prevalence	Global	Both	60-64 yea	Genital her	Number	2000	4.1E+07	4.8E+07	3.6E+07
Prevalence	Global	Both	60-64 yea	Genital her	Rate	2000	21957.5	25470.6	19006
Prevalence	Global	Both	60-64 yea	Genital her	Number	2001	4.2E+07	4.8E+07	3.6E+07
Prevalence	Global	Both	60-64 yea	Genital her	Rate	2001	21999.9	25520.9	19045.3
Prevalence	Global	Both	60-64 yea	Genital her	Number	2002	4.2E+07	4.9E+07	3.6E+07
Prevalence	Global	Both	60-64 yea	Genital her	Rate	2002	22061.5	25592.7	19103
Prevalence	Global	Both	60-64 yea	Genital her	Number	2003	4.2E+07	4.9E+07	3.7E+07
Prevalence	Global	Both	60-64 yea	Genital her	Rate	2003	22114.6	25660.3	19144.2
Prevalence	Global	Both	60-64 yea	Genital her	Number	2004	4.3E+07	5E+07	3.7E+07
Prevalence	Global	Both	60-64 yea	Genital her	Rate	2004	22149.9	25713.5	19155.2
Prevalence	Global	Both	60-64 yea	Genital her	Number	2005	4.3E+07	5E+07	3.7E+07
Prevalence	Global	Both	60-64 yea	Genital her	Rate	2005	22166.7	25740.3	19161.8
Prevalence	Global	Both	60-64 yea	Genital her	Number	2006	4.4E+07	5.1E+07	3.8E+07
Prevalence	Global	Both	60-64 yea	Genital her	Rate	2006	22119.8	25686.2	19110.8
Prevalence	Global	Both	60-64 yea	Genital her	Number	2007	4.6E+07	5.3E+07	3.9E+07
Prevalence	Global	Both	60-64 yea	Genital her	Rate	2007	21935.9	25491.3	18936.3
Prevalence	Global	Both	60-64 yea	Genital her	Number	2008	4.7E+07	5.5E+07	4.1E+07
Prevalence	Global	Both	60-64 yea	Genital her	Rate	2008	21734.7	25296	18721.1
Prevalence	Global	Both	60-64 yea	Genital her	Number	2009	4.9E+07	5.7E+07	4.2E+07
Prevalence	Global	Both	60-64 yea	Genital her	Rate	2009	21525.1	25088.3	18495.8
Prevalence	Global	Both	60-64 yea	Genital her	Number	2010	5.1E+07	6E+07	4.4E+07
Prevalence	Global	Both	60-64 yea	Genital her	Rate	2010	21461.2	25033.1	18407.6
Prevalence	Global	Both	60-64 yea	Genital her	Number	2011	5.4E+07	6.3E+07	4.6E+07
Prevalence	Global	Both	60-64 yea	Genital her	Rate	2011	21483.6	25059.2	18452.2
Prevalence	Global	Both	60-64 yea	Genital her	Number	2012	5.6E+07	6.6E+07	4.8E+07
Prevalence	Global	Both	60-64 yea	Genital her	Rate	2012	21596.7	25213.6	18554.4
Prevalence	Global	Both	60-64 yea	Genital her	Number	2013	5.9E+07	6.9E+07	5.1E+07
Prevalence	Global	Both	60-64 yea	Genital her	Rate	2013	21724.3	25385.1	18677.4
Prevalence	Global	Both	60-64 yea	Genital her	Number	2014	6.1E+07	7.1E+07	5.2E+07
Prevalence	Global	Both	60-64 yea	Genital her	Rate	2014	21872.2	25567.5	18791.8
Prevalence	Global	Both	60-64 yea	Genital her	Number	2015	6.4E+07	7.5E+07	5.5E+07
Prevalence	Global	Both	60-64 yea	Genital her	Rate	2015	21946.1	25707.8	18845.2
Prevalence	Global	Both	60-64 yea	Genital her	Number	2016	6.6E+07	7.7E+07	5.6E+07
Prevalence	Global	Both	60-64 yea	Genital her	Rate	2016	22028	25812.7	18888.3
Prevalence	Global	Both	60-64 yea	Genital her	Number	2017	6.7E+07	7.9E+07	5.8E+07
Prevalence	Global	Both	60-64 yea	Genital her	Rate	2017	22121.4	25954.8	18928.5
Prevalence	Global	Both	60-64 yea	Genital her	Number	2018	6.9E+07	8.1E+07	5.9E+07
Prevalence	Global	Both	60-64 yea	Genital her	Rate	2018	22217	26063.9	19018.5
Prevalence	Global	Both	60-64 yea	Genital her	Number	2019	7E+07	8.2E+07	6E+07
Prevalence	Global	Both	60-64 yea	Genital her	Rate	2019	22327.6	26198.2	19119.3
Prevalence	Global	Both	65-69 yea	Genital her	Number	1990	2.6E+07	3E+07	2.3E+07
Prevalence	Global	Both	65-69 yea	Genital her	Rate	1990	21170.4	24636.3	18230.4
Prevalence	Global	Both	65-69 yea	Genital her	Number	1991	2.7E+07	3.2E+07	2.3E+07
Prevalence	Global	Both	65-69 yea	Genital her	Rate	1991	21324	24781.9	18403.9
Prevalence	Global	Both	65-69 yea	Genital her	Number	1992	2.8E+07	3.3E+07	2.4E+07
Prevalence	Global	Both	65-69 yea	Genital her	Rate	1992	21421.1	24862	18535.3
Prevalence	Global	Both	65-69 yea	Genital her	Number	1993	2.9E+07	3.4E+07	2.5E+07
Prevalence	Global	Both	65-69 yea	Genital her	Rate	1993	21478.1	24900.3	18595
Prevalence	Global	Both	65-69 yea	Genital her	Number	1994	3E+07	3.5E+07	2.6E+07
Prevalence	Global	Both	65-69 yea	Genital her	Rate	1994	21496.8	24898.9	18615.9
Prevalence	Global	Both	65-69 yea	Genital her	Number	1995	3.1E+07	3.5E+07	2.6E+07
Prevalence	Global	Both	65-69 yea	Genital her	Rate	1995	21488.2	24882.9	18634.3
Prevalence	Global	Both	65-69 yea	Genital her	Number	1996	3.1E+07	3.6E+07	2.7E+07
Prevalence	Global	Both	65-69 yea	Genital her	Rate	1996	21407.4	24790.2	18557.1
Prevalence	Global	Both	65-69 yea	Genital her	Number	1997	3.1E+07	3.6E+07	2.7E+07
Prevalence	Global	Both	65-69 yea	Genital her	Rate	1997	21257.4	24609.1	18424.3
Prevalence	Global	Both	65-69 yea	Genital her	Number	1998	3.1E+07	3.6E+07	2.7E+07
Prevalence	Global	Both	65-69 yea	Genital her	Rate	1998	21084.7	24409.1	18274.4
Prevalence	Global	Both	65-69 yea	Genital her	Number	1999	3.2E+07	3.7E+07	2.7E+07
Prevalence	Global	Both	65-69 yea	Genital her	Rate	1999	20939.3	24259.3	18133.4
Prevalence	Global	Both	65-69 yea	Genital her	Number	2000	3.2E+07	3.7E+07	2.8E+07
Prevalence	Global	Both	65-69 yea	Genital her	Rate	2000	20881.6	24214	18073.7
Prevalence	Global	Both	65-69 yea	Genital her	Number	2001	3.3E+07	3.8E+07	2.8E+07
Prevalence	Global	Both	65-69 yea	Genital her	Rate	2001	20893.6	24204.8	18088.8
Prevalence	Global	Both	65-69 yea	Genital her	Number	2002	3.3E+07	3.9E+07	2.9E+07
Prevalence	Global	Both	65-69 yea	Genital her	Rate	2002	20921.9	24271.5	18115
Prevalence	Global	Both	65-69 yea	Genital her	Number	2003	3.4E+07	4E+07	3E+07
Prevalence	Global	Both	65-69 yea	Genital her	Rate	2003	20965.3	24328.9	18153.2
Prevalence	Global	Both	65-69 yea	Genital her	Number	2004	3.5E+07	4E+07	3E+07
Prevalence	Global	Both	65-69 yea	Genital her	Rate	2004	21014.1	24385.3	18190.5
Prevalence	Global	Both	65-69 yea	Genital her	Number	2005	3.5E+07	4.1E+07	3.1E+07
Prevalence	Global	Both	65-69 yea	Genital her	Rate	2005	21053.5	24428	18208.5
Prevalence	Global	Both	65-69 yea	Genital her	Number	2006	3.6E+07	4.2E+07	3.1E+07
Prevalence	Global	Both	65-69 yea	Genital her	Rate	2006	21022.7	24414.4	18161.6
Prevalence	Global	Both	65-69 yea	Genital her	Number	2007	3.6E+07	4.2E+07	3.1E+07
Prevalence	Global	Both	65-69 yea	Genital her	Rate	2007	20928.6	24335.5	18049
Prevalence	Global	Both	65-69 yea	Genital her	Number	2008	3.6E+07	4.2E+07	3.1E+07
Prevalence	Global	Both	65-69 yea	Genital her	Rate	2008	20794.9	24215.3	17910.7
Prevalence	Global	Both	65-69 yea	Genital her	Number	2009	3.6E+07	4.3E+07	3.1E+07
Prevalence	Global	Both	65-69 yea	Genital her	Rate	2009	20668.6	24102.1	17777.1
Prevalence	Global	Both	65-69 yea	Genital her	Number	2010	3.7E+07	4.3E+07	3.2E+07
Prevalence	Global	Both	65-69 yea	Genital her	Rate	2010	20603.9	24049	17697.3
Prevalence	Global	Both	65-69 yea	Genital her	Number	2011	3.8E+07	4.4E+07	3.2E+07
Prevalence	Global	Both	65-69 yea	Genital her	Rate	2011	20622.3	24022.7	17716.9
Prevalence	Global	Both	65-69 yea	Genital her	Number	2012	3.9E+07	4.6E+07	3.4E+07
Prevalence	Global	Both	65-69 yea	Genital her	Rate	2012	20648.8	24026.5	17735.3
Prevalence	Global	Both	65-69 yea	Genital her	Number	2013	4.1E+07	4.8E+07	3.6E+07
Prevalence	Global	Both	65-69 yea	Genital her	Rate	2013	20714.7	24101.3	17812.8
Prevalence	Global	Both	65-69 yea	Genital her	Number	2014	4.4E+07	5.1E+07	3.8E+07
Prevalence	Global	Both	65-69 yea	Genital her	Rate	2014	20749.1	24219.5	17816
Prevalence	Global	Both	65-69 yea	Genital her	Number	2015	4.6E+07	5.4E+07	3.9E+07
Prevalence	Global	Both	65-69 yea	Genital her	Rate	2015	20787.4	24320.9	17829.9
Prevalence	Global	Both	65-69 yea	Genital her	Number	2016	4.8E+07	5.7E+07	4.1E+07
Prevalence	Global	Both	65-69 yea	Genital her	Rate	2016	20812.8	24331.7	17831.7
Prevalence	Global	Both	65-69 yea	Genital her	Number	2017	5E+07	5.9E+07	4.3E+07
Prevalence	Global	Both	65-69 yea	Genital her	Rate	2017	20875.3	24417.8	17873.1
Prevalence	Global	Both	65-69 yea	Genital her	Number	2018	5.2E+07	6.1E+07	4.5E+07
Prevalence	Global	Both	65-69 yea	Genital her	Rate	2018	20909.5	24490.4	17903.4
Prevalence	Global	Both	65-69 yea	Genital her	Number	2019	5.4E+07	6.4E+07	4.6E+07
Prevalence	Global	Both	65-69 yea	Genital her	Rate	2019	20974.8	24598	17955
Prevalence	Global	Both	50-69 yea	Genital her	Number	1990	1.5E+08	1.8E+08	1.3E+08
Prevalence	Global	Both	50-69 yea	Genital her	Rate	1990	22254.4	25931.2	19105.3
Prevalence	Global	Both	50-69 yea	Genital her	Number	1991	1.6E+08	1.8E+08	1.3E+08
Prevalence	Global	Both	50-69 yea	Genital her	Rate	1991	22466.4	26127.2	19314.9
Prevalence	Global	Both	50-69 yea	Genital her	Number	1992	1.6E+08	1.9E+08	1.4E+08
Prevalence	Global	Both	50-69 yea	Genital her	Rate	1992	22636.6	26301.5	19485.9
Prevalence	Global	Both	50-69 yea	Genital her	Number	1993	1.6E+08	1.9E+08	1.4E+08
Prevalence	Global	Both	50-69 yea	Genital her	Rate	1993	22762.8	26407.9	19603.9
Prevalence	Global	Both	50-69 yea	Genital her	Number	1994	1.7E+08	1.9E+08	1.4E+08
Prevalence	Global	Both	50-69 yea	Genital her	Rate	1994	22848.7	26478.1	19681.6
Prevalence	Global	Both	50-69 yea	Genital her	Number	1995	1.7E+08	1.9E+08	1.4E+08
Prevalence	Global	Both	50-69 yea	Genital her	Rate	1995	22897.9	26525	19732.9
Prevalence	Global	Both	50-69 yea	Genital her	Number	1996	1.7E+08	2E+08	1.5E+08
Prevalence	Global	Both	50-69 yea	Genital her	Rate	1996	22865.9	26494.8	19703.4
Prevalence	Global	Both	50-69 yea	Genital her	Number	1997	1.7E+08	2E+08	1.5E+08
Prevalence	Global	Both	50-69 yea	Genital her	Rate	1997	22735.6	26373	19600.8
Prevalence	Global	Both	50-69 yea	Genital her	Number	1998	1.7E+08	2E+08	1.5E+08
Prevalence	Global	Both	50-69 yea	Genital her	Rate	1998	22577.6	26184.4	19472.3
Prevalence	Global	Both	50-69 yea	Genital her	Number	1999	1.8E+08	2.1E+08	1.5E+08
Prevalence	Global	Both	50-69 yea	Genital her	Rate	1999	22439.5	26045	19357.6
Prevalence	Global	Both	50-69 yea	Genital her	Number	2000	1.8E+08	2.1E+08	1.6E+08
Prevalence	Global	Both	50-69 yea	Genital her	Rate	2000	22401.5	26018.2	19327.1
Prevalence	Global	Both	50-69 yea	Genital her	Number	2001	1.9E+08	2.2E+08	1.6E+08
Prevalence	Global	Both	50-69 yea	Genital her	Rate	2001	22432.4	26033.1	19345.3
Prevalence	Global	Both	50-69 yea	Genital her	Number	2002	1.9E+08	2.2E+08	1.7E+08
Prevalence	Global	Both	50-69 yea	Genital her	Rate	2002	22465.5	26057.1	19364
Prevalence	Global	Both	50-69 yea	Genital her	Number	2003	2E+08	2.3E+08	1.7E+08
Prevalence	Global	Both	50-69 yea	Genital her	Rate	2003	22497.4	26093.4	19347.9
Prevalence	Global	Both	50-69 yea	Genital her	Number	2004	2E+08	2.4E+08	1.7E+08
Prevalence	Global	Both	50-69 yea	Genital her	Rate	2004	22528.1	26146.2	19356.6
Prevalence	Global	Both	50-69 yea	Genital her	Number	2005	2.1E+08	2.4E+08	1.8E+08
Prevalence	Global	Both	50-69 yea	Genital her	Rate	2005	22556.4	26184.5	19371.1
Prevalence	Global	Both	50-69 yea	Genital her	Number	2006	2.2E+08	2.5E+08	1.8E+08
Prevalence	Global	Both	50-69 yea	Genital her	Rate	2006	22517.8	26157.2	19334.4
Prevalence	Global	Both	50-69 yea	Genital her	Number	2007	2.2E+08	2.6E+08	1.9E+08
Prevalence	Global	Both	50-69 yea	Genital her	Rate	2007	22389.4	26048.8	19212.6
Prevalence	Global	Both	50-69 yea	Genital her	Number	2008	2.2E+08	2.6E+08	1.9E+08
Prevalence	Global	Both	50-69 yea	Genital her	Rate	2008	22234	25916.1	19060
Prevalence	Global	Both	50-69 yea	Genital her	Number	2009	2.3E+08	2.7E+08	2E+08
Prevalence	Global	Both	50-69 yea	Genital her	Rate	2009	22110.1	25764.9	18931.3
Prevalence	Global	Both	50-69 yea	Genital her	Number	2010	2.3E+08	2.7E+08	2E+08
Prevalence	Global	Both	50-69 yea	Genital her	Rate	2010	22079.2	25728	18888.1
Prevalence	Global	Both	50-69 yea	Genital her	Number	2011	2.4E+08	2.8E+08	2.1E+08
Prevalence	Global	Both	50-69 yea	Genital her	Rate	2011	22145.9	25803.9	18949.9
Prevalence	Global	Both	50-69 yea	Genital her	Number	2012	2.5E+08	2.9E+08	2.1E+08
Prevalence	Global	Both	50-69 yea	Genital her	Rate	2012	22255.9	25949.2	19041
Prevalence	Global	Both	50-69 yea	Genital her	Number	2013	2.6E+08	3E+08	2.2E+08
Prevalence	Global	Both	50-69 yea	Genital her	Rate	2013	22377.9	26120.4	19132.2
Prevalence	Global	Both	50-69 yea	Genital her	Number	2014	2.7E+08	3.2E+08	2.3E+08
Prevalence	Global	Both	50-69 yea	Genital her	Rate	2014	22480.9	26265.6	19199.2
Prevalence	Global	Both	50-69 yea	Genital her	Number	2015	2.8E+08	3.3E+08	2.4E+08
Prevalence	Global	Both	50-69 yea	Genital her	Rate	2015	22532.3	26313.1	19232.5
Prevalence	Global	Both	50-69 yea	Genital her	Number	2016	2.9E+08	3.4E+08	2.5E+08
Prevalence	Global	Both	50-69 yea	Genital her	Rate	2016	22567.9	26420.3	19235.4
Prevalence	Global	Both	50-69 yea	Genital her	Number	2017	3E+08	3.5E+08	2.5E+08
Prevalence	Global	Both	50-69 yea	Genital her	Rate	2017	22609.6	26571.3	19233.4
Prevalence	Global	Both	50-69 yea	Genital her	Number	2018	3E+08	3.6E+08	2.6E+08
Prevalence	Global	Both	50-69 yea	Genital her	Rate	2018	22631.4	26521	19249.5
Prevalence	Global	Both	50-69 yea	Genital her	Number	2019	3.1E+08	3.7E+08	2.7E+08
Prevalence	Global	Both	50-69 yea	Genital her	Rate	2019	22669.1	26536.3	19285.7
Prevalence	Global	Both	50-54 yea	Gonococca	Number	1990	676968	1163732	391457
Prevalence	Global	Both	50-54 yea	Gonococca	Rate	1990	318.437	547.404	184.136
Prevalence	Global	Both	50-54 yea	Gonococca	Number	1991	686354	1176469	396133
Prevalence	Global	Both	50-54 yea	Gonococca	Rate	1991	319.396	547.471	184.341
Prevalence	Global	Both	50-54 yea	Gonococca	Number	1992	690892	1183472	397860
Prevalence	Global	Both	50-54 yea	Gonococca	Rate	1992	319.448	547.203	183.959
Prevalence	Global	Both	50-54 yea	Gonococca	Number	1993	689321	1182578	396545
Prevalence	Global	Both	50-54 yea	Gonococca	Rate	1993	317.504	544.7	182.65
Prevalence	Global	Both	50-54 yea	Gonococca	Number	1994	686576	1180491	394569
Prevalence	Global	Both	50-54 yea	Gonococca	Rate	1994	314.737	541.155	180.877
Prevalence	Global	Both	50-54 yea	Gonococca	Number	1995	682923	1179612	392764
Prevalence	Global	Both	50-54 yea	Gonococca	Rate	1995	311.704	538.405	179.267
Prevalence	Global	Both	50-54 yea	Gonococca	Number	1996	687454	1184109	396103
Prevalence	Global	Both	50-54 yea	Gonococca	Rate	1996	309.168	532.529	178.139
Prevalence	Global	Both	50-54 yea	Gonococca	Number	1997	707095	1218378	407247
Prevalence	Global	Both	50-54 yea	Gonococca	Rate	1997	306.549	528.208	176.555
Prevalence	Global	Both	50-54 yea	Gonococca	Number	1998	733272	1262485	422495
Prevalence	Global	Both	50-54 yea	Gonococca	Rate	1998	305.397	525.807	175.963
Prevalence	Global	Both	50-54 yea	Gonococca	Number	1999	769646	1328104	443761
Prevalence	Global	Both	50-54 yea	Gonococca	Rate	1999	305.171	526.604	175.955
Prevalence	Global	Both	50-54 yea	Gonococca	Number	2000	800444	1383299	460829
Prevalence	Global	Both	50-54 yea	Gonococca	Rate	2000	305.239	527.504	175.732
Prevalence	Global	Both	50-54 yea	Gonococca	Number	2001	840424	1449214	482868
Prevalence	Global	Both	50-54 yea	Gonococca	Rate	2001	305.204	526.289	175.356
Prevalence	Global	Both	50-54 yea	Gonococca	Number	2002	869961	1503598	500404
Prevalence	Global	Both	50-54 yea	Gonococca	Rate	2002	305.4	527.838	175.667
Prevalence	Global	Both	50-54 yea	Gonococca	Number	2003	901800	1561249	517713
Prevalence	Global	Both	50-54 yea	Gonococca	Rate	2003	305.604	529.079	175.444
Prevalence	Global	Both	50-54 yea	Gonococca	Number	2004	930055	1610677	533983
Prevalence	Global	Both	50-54 yea	Gonococca	Rate	2004	305.636	529.302	175.478
Prevalence	Global	Both	50-54 yea	Gonococca	Number	2005	967747	1676425	554279
Prevalence	Global	Both	50-54 yea	Gonococca	Rate	2005	305.535	529.277	174.996
Prevalence	Global	Both	50-54 yea	Gonococca	Number	2006	986098	1705155	565206
Prevalence	Global	Both	50-54 yea	Gonococca	Rate	2006	304.003	525.681	174.247
Prevalence	Global	Both	50-54 yea	Gonococca	Number	2007	994789	1714513	572274
Prevalence	Global	Both	50-54 yea	Gonococca	Rate	2007	300.545	517.988	172.895
Prevalence	Global	Both	50-54 yea	Gonococca	Number	2008	994924	1701594	578403
Prevalence	Global	Both	50-54 yea	Gonococca	Rate	2008	296.329	506.804	172.272
Prevalence	Global	Both	50-54 yea	Gonococca	Number	2009	993078	1687930	579035
Prevalence	Global	Both	50-54 yea	Gonococca	Rate	2009	292.633	497.387	170.626
Prevalence	Global	Both	50-54 yea	Gonococca	Number	2010	996954	1692378	584805
Prevalence	Global	Both	50-54 yea	Gonococca	Rate	2010	290.405	492.977	170.349
Prevalence	Global	Both	50-54 yea	Gonococca	Number	2011	1010214	1714737	592426
Prevalence	Global	Both	50-54 yea	Gonococca	Rate	2011	289.193	490.875	169.593
Prevalence	Global	Both	50-54 yea	Gonococca	Number	2012	1031823	1753466	605155
Prevalence	Global	Both	50-54 yea	Syphilis	Number	1990	1201009	2007080	724081
Prevalence	Global	Both	50-54 yea	Syphilis	Rate	1990	564.938	944.103	340.598
Prevalence	Global	Both	50-54 yea	Syphilis	Number	1991	1211670	2021355	732080
Prevalence	Global	Both	50-54 yea	Syphilis	Rate	1991	563.852	940.64	340.674
Prevalence	Global	Both	50-54 yea	Syphilis	Number	1992	1220572	2032238	738306
Prevalence	Global	Both	50-54 yea	Syphilis	Rate	1992	564.357	939.647	341.371
Prevalence	Global	Both	50-54 yea	Syphilis	Number	1993	1228382	2041316	743884
Prevalence	Global	Both	50-54 yea	Syphilis	Rate	1993	565.798	940.239	342.636
Prevalence	Global	Both	50-54 yea	Syphilis	Number	1994	1238287	2058025	751824
Prevalence	Global	Both	50-54 yea	Syphilis	Rate	1994	567.65	943.431	344.648
Prevalence	Global	Both	50-54 yea	Syphilis	Number	1995	1247347	2079884	757650
Prevalence	Global	Both	50-54 yea	Syphilis	Rate	1995	569.321	949.312	345.811
Prevalence	Global	Both	50-54 yea	Syphilis	Number	1996	1267219	2109724	770519
Prevalence	Global	Both	50-54 yea	Syphilis	Rate	1996	569.906	948.805	346.525
Prevalence	Global	Both	50-54 yea	Syphilis	Number	1997	1302679	2166560	790715
Prevalence	Global	Both	50-54 yea	Syphilis	Rate	1997	564.755	939.276	342.801
Prevalence	Global	Both	50-54 yea	Syphilis	Number	1998	1341236	2228532	811217
Prevalence	Global	Both	50-54 yea	Syphilis	Rate	1998	558.605	928.151	337.86
Prevalence	Global	Both	50-54 yea	Syphilis	Number	1999	1388591	2309065	838696
Prevalence	Global	Both	50-54 yea	Syphilis	Rate	1999	550.587	915.563	332.55
Prevalence	Global	Both	50-54 yea	Syphilis	Number	2000	1424348	2371480	859691
Prevalence	Global	Both	50-54 yea	Syphilis	Rate	2000	543.157	904.335	327.833
Prevalence	Global	Both	50-54 yea	Syphilis	Number	2001	1484691	2472975	897946
Prevalence	Global	Both	50-54 yea	Syphilis	Rate	2001	539.172	898.072	326.093
Prevalence	Global	Both	50-54 yea	Syphilis	Number	2002	1526697	2541777	925523
Prevalence	Global	Both	50-54 yea	Syphilis	Rate	2002	535.947	892.291	324.905
Prevalence	Global	Both	50-54 yea	Syphilis	Number	2003	1568234	2605428	953189
Prevalence	Global	Both	50-54 yea	Syphilis	Rate	2003	531.446	882.932	323.018
Prevalence	Global	Both	50-54 yea	Syphilis	Number	2004	1607805	2664918	977782
Prevalence	Global	Both	50-54 yea	Syphilis	Rate	2004	528.358	875.748	321.32
Prevalence	Global	Both	50-54 yea	Syphilis	Number	2005	1667940	2759325	1012917
Prevalence	Global	Both	50-54 yea	Syphilis	Rate	2005	526.598	871.167	319.795
Prevalence	Global	Both	50-54 yea	Syphilis	Number	2006	1697124	2805902	1031972
Prevalence	Global	Both	50-54 yea	Syphilis	Rate	2006	523.205	865.029	318.146
Prevalence	Global	Both	50-54 yea	Syphilis	Number	2007	1719093	2838168	1049925
Prevalence	Global	Both	50-54 yea	Syphilis	Rate	2007	519.371	857.466	317.203
Prevalence	Global	Both	50-54 yea	Syphilis	Number	2008	1734836	2856177	1061434
Prevalence	Global	Both	50-54 yea	Syphilis	Rate	2008	516.704	850.685	316.138
Prevalence	Global	Both	50-54 yea	Syphilis	Number	2009	1750891	2873974	1074682
Prevalence	Global	Both	50-54 yea	Syphilis	Rate	2009	515.94	846.881	316.679
Prevalence	Global	Both	50-54 yea	Syphilis	Number	2010	1777022	2910919	1093241
Prevalence	Global	Both	50-54 yea	Syphilis	Rate	2010	517.633	847.929	318.453
Prevalence	Global	Both	50-54 yea	Syphilis	Number	2011	1815349	2970307	1117631
Prevalence	Global	Both	50-54 yea	Syphilis	Rate	2011	519.677	850.305	319.943
Prevalence	Global	Both	50-54 yea	Syphilis	Number	2012	1862155	3044378	1145262
Prevalence	Global	Both	50-54 yea	Syphilis	Rate	2012	519.801	849.807	319.688
Prevalence	Global	Both	50-54 yea	Syphilis	Number	2013	1921422	3137277	1179669
Prevalence	Global	Both	50-54 yea	Syphilis	Rate	2013	518.209	846.127	318.158
Prevalence	Global	Both	50-54 yea	Syphilis	Number	2014	1989661	3242179	1219407
Prevalence	Global	Both	50-54 yea	Syphilis	Rate	2014	516.49	841.627	316.542
Prevalence	Global	Both	50-54 yea	Syphilis	Number	2015	2062486	3369302	1261931
Prevalence	Global	Both	50-54 yea	Syphilis	Rate	2015	516.181	843.239	315.825
Prevalence	Global	Both	50-54 yea	Syphilis	Number	2016	2163232	3551780	1313995
Prevalence	Global	Both	50-54 yea	Syphilis	Rate	2016	525.141	862.222	318.982
Prevalence	Global	Both	50-54 yea	Syphilis	Number	2017	2265097	3732833	1376603
Prevalence	Global	Both	50-54 yea	Syphilis	Rate	2017	536.055	883.408	325.785
Prevalence	Global	Both	50-54 yea	Syphilis	Number	2018	2339384	3855081	1417937

Prevalence	Global	Both	55-59 yea	Syphilis	Number	2006	1039080	1596074	657636
Prevalence	Global	Both	55-59 yea	Syphilis	Rate	2006	395.763	607.91	250.479
Prevalence	Global	Both	55-59 yea	Syphilis	Number	2007	1069976	1645665	678574
Prevalence	Global	Both	55-59 yea	Syphilis	Rate	2007	393.475	605.18	249.54
Prevalence	Global	Both	55-59 yea	Syphilis	Number	2008	1102006	1696843	697306
Prevalence	Global	Both	55-59 yea	Syphilis	Rate	2008	390.776	601.708	247.268
Prevalence	Global	Both	55-59 yea	Syphilis	Number	2009	1133181	1745578	718800
Prevalence	Global	Both	55-59 yea	Syphilis	Rate	2009	389.254	599.616	246.912
Prevalence	Global	Both	55-59 yea	Syphilis	Number	2010	1179930	1818563	750138
Prevalence	Global	Both	55-59 yea	Syphilis	Rate	2010	388.901	599.393	247.243
Prevalence	Global	Both	55-59 yea	Syphilis	Number	2011	1207835	1863569	765700
Prevalence	Global	Both	55-59 yea	Syphilis	Rate	2011	388.38	599.231	246.211
Prevalence	Global	Both	55-59 yea	Syphilis	Number	2012	1234572	1904967	783564
Prevalence	Global	Both	55-59 yea	Syphilis	Rate	2012	388.722	599.805	246.716
Prevalence	Global	Both	55-59 yea	Syphilis	Number	2013	1258125	1940522	798343
Prevalence	Global	Both	55-59 yea	Syphilis	Rate	2013	390.217	601.868	247.612
Prevalence	Global	Both	55-59 yea	Syphilis	Number	2014	1280601	1973169	812310
Prevalence	Global	Both	55-59 yea	Syphilis	Rate	2014	392.653	605.005	249.067
Prevalence	Global	Both	55-59 yea	Syphilis	Number	2015	1306438	2009853	828278
Prevalence	Global	Both	55-59 yea	Syphilis	Rate	2015	395.76	608.846	250.911
Prevalence	Global	Both	55-59 yea	Syphilis	Number	2016	1353931	2075886	853424
Prevalence	Global	Both	55-59 yea	Syphilis	Rate	2016	402.948	617.811	253.99
Prevalence	Global	Both	55-59 yea	Syphilis	Number	2017	1411716	2174543	883357
Prevalence	Global	Both	55-59 yea	Syphilis	Rate	2017	409.584	630.904	256.29
Prevalence	Global	Both	55-59 yea	Syphilis	Number	2018	1467892	2264026	919237
Prevalence	Global	Both	55-59 yea	Syphilis	Rate	2018	411.301	634.377	257.569
Prevalence	Global	Both	55-59 yea	Syphilis	Number	2019	1531652	2371407	958508
Prevalence	Global	Both	55-59 yea	Syphilis	Rate	2019	412.83	639.172	258.349
Prevalence	Global	Both	60-64 yea	Syphilis	Number	1990	411495	589436	267915
Prevalence	Global	Both	60-64 yea	Syphilis	Rate	1990	256.155	366.923	166.776
Prevalence	Global	Both	60-64 yea	Syphilis	Number	1991	420736	602244	275850
Prevalence	Global	Both	60-64 yea	Syphilis	Rate	1991	256.567	367.251	168.215
Prevalence	Global	Both	60-64 yea	Syphilis	Number	1992	427209	611352	282654
Prevalence	Global	Both	60-64 yea	Syphilis	Rate	1992	256.826	367.528	169.924
Prevalence	Global	Both	60-64 yea	Syphilis	Number	1993	432401	618879	288769
Prevalence	Global	Both	60-64 yea	Syphilis	Rate	1993	256.825	367.584	171.515
Prevalence	Global	Both	60-64 yea	Syphilis	Number	1994	436976	622814	294337
Prevalence	Global	Both	60-64 yea	Syphilis	Rate	1994	256.991	366.284	173.103
Prevalence	Global	Both	60-64 yea	Syphilis	Number	1995	442127	629976	299615
Prevalence	Global	Both	60-64 yea	Syphilis	Rate	1995	257.053	366.269	174.197
Prevalence	Global	Both	60-64 yea	Syphilis	Number	1996	447630	638152	303290
Prevalence	Global	Both	60-64 yea	Syphilis	Rate	1996	256.412	365.547	173.731
Prevalence	Global	Both	60-64 yea	Syphilis	Number	1997	453227	647281	306679
Prevalence	Global	Both	60-64 yea	Syphilis	Rate	1997	254.773	363.858	172.394
Prevalence	Global	Both	60-64 yea	Syphilis	Number	1998	458162	656868	309175
Prevalence	Global	Both	60-64 yea	Syphilis	Rate	1998	252.886	362.564	170.652
Prevalence	Global	Both	60-64 yea	Syphilis	Number	1999	462639	663697	311280
Prevalence	Global	Both	60-64 yea	Syphilis	Rate	1999	251.249	360.439	169.049
Prevalence	Global	Both	60-64 yea	Syphilis	Number	2000	467660	671763	313880
Prevalence	Global	Both	60-64 yea	Syphilis	Rate	2000	250.144	359.315	167.889
Prevalence	Global	Both	60-64 yea	Syphilis	Number	2001	471742	677767	316129
Prevalence	Global	Both	60-64 yea	Syphilis	Rate	2001	249.426	358.358	167.148
Prevalence	Global	Both	60-64 yea	Syphilis	Number	2002	474637	681108	317562
Prevalence	Global	Both	60-64 yea	Syphilis	Rate	2002	249.082	357.435	166.652
Prevalence	Global	Both	60-64 yea	Syphilis	Number	2003	477248	685532	318673
Prevalence	Global	Both	60-64 yea	Syphilis	Rate	2003	248.925	357.562	166.215
Prevalence	Global	Both	60-64 yea	Syphilis	Number	2004	480719	691038	319986
Prevalence	Global	Both	60-64 yea	Syphilis	Rate	2004	248.679	357.478	165.531
Prevalence	Global	Both	60-64 yea	Syphilis	Number	2005	484641	697441	321618
Prevalence	Global	Both	60-64 yea	Syphilis	Rate	2005	248.52	357.642	164.923
Prevalence	Global	Both	60-64 yea	Syphilis	Number	2006	492958	712418	326327
Prevalence	Global	Both	60-64 yea	Syphilis	Rate	2006	247.88	358.233	164.091
Prevalence	Global	Both	60-64 yea	Syphilis	Number	2007	508878	735928	335870
Prevalence	Global	Both	60-64 yea	Syphilis	Rate	2007	245.173	354.565	161.82
Prevalence	Global	Both	60-64 yea	Syphilis	Number	2008	525781	757492	345935
Prevalence	Global	Both	60-64 yea	Syphilis	Rate	2008	242.122	348.824	159.302
Prevalence	Global	Both	60-64 yea	Syphilis	Number	2009	547349	793221	358495
Prevalence	Global	Both	60-64 yea	Syphilis	Rate	2009	238.927	346.255	156.489
Prevalence	Global	Both	60-64 yea	Syphilis	Number	2010	564467	819988	368823
Prevalence	Global	Both	60-64 yea	Syphilis	Rate	2010	236.257	343.205	154.371
Prevalence	Global	Both	60-64 yea	Syphilis	Number	2011	592587	859929	386503
Prevalence	Global	Both	60-64 yea	Syphilis	Rate	2011	235.554	341.822	153.635
Prevalence	Global	Both	60-64 yea	Syphilis	Number	2012	614724	891105	400411
Prevalence	Global	Both	60-64 yea	Syphilis	Rate	2012	235.811	341.832	153.599
Prevalence	Global	Both	60-64 yea	Syphilis	Number	2013	638507	924652	415081
Prevalence	Global	Both	60-64 yea	Syphilis	Rate	2013	236.108	341.92	153.489
Prevalence	Global	Both	60-64 yea	Syphilis	Number	2014	660826	955914	429126
Prevalence	Global	Both	60-64 yea	Syphilis	Rate	2014	236.641	342.312	153.67
Prevalence	Global	Both	60-64 yea	Syphilis	Number	2015	690632	997823	447986
Prevalence	Global	Both	60-64 yea	Syphilis	Rate	2015	237.304	342.857	153.93
Prevalence	Global	Both	60-64 yea	Syphilis	Number	2016	707925	1026922	455724
Prevalence	Global	Both	60-64 yea	Syphilis	Rate	2016	237.351	344.304	152.794
Prevalence	Global	Both	60-64 yea	Syphilis	Number	2017	725109	1048815	462373
Prevalence	Global	Both	60-64 yea	Syphilis	Rate	2017	238.117	344.418	151.838
Prevalence	Global	Both	60-64 yea	Syphilis	Number	2018	740629	1071550	472763
Prevalence	Global	Both	60-64 yea	Syphilis	Rate	2018	239.645	346.721	152.972
Prevalence	Global	Both	60-64 yea	Syphilis	Number	2019	754286	1090679	482436
Prevalence	Global	Both	60-64 yea	Syphilis	Rate	2019	241.344	348.978	154.362
Prevalence	Global	Both	65-69 yea	Syphilis	Number	1990	169596	243745	118846
Prevalence	Global	Both	65-69 yea	Syphilis	Rate	1990	137.338	197.383	96.2408
Prevalence	Global	Both	65-69 yea	Syphilis	Number	1991	174379	250458	122674
Prevalence	Global	Both	65-69 yea	Syphilis	Rate	1991	137.012	196.789	96.3869
Prevalence	Global	Both	65-69 yea	Syphilis	Number	1992	179412	256601	126327
Prevalence	Global	Both	65-69 yea	Syphilis	Rate	1992	136.844	195.719	96.3539
Prevalence	Global	Both	65-69 yea	Syphilis	Number	1993	184634	263060	129995
Prevalence	Global	Both	65-69 yea	Syphilis	Rate	1993	136.827	194.946	96.3349
Prevalence	Global	Both	65-69 yea	Syphilis	Number	1994	189974	269674	134179
Prevalence	Global	Both	65-69 yea	Syphilis	Rate	1994	136.924	194.368	96.7098
Prevalence	Global	Both	65-69 yea	Syphilis	Number	1995	194909	275729	138372
Prevalence	Global	Both	65-69 yea	Syphilis	Rate	1995	137.197	194.086	97.4003
Prevalence	Global	Both	65-69 yea	Syphilis	Number	1996	198810	281668	141046
Prevalence	Global	Both	65-69 yea	Syphilis	Rate	1996	137.03	194.14	97.216
Prevalence	Global	Both	65-69 yea	Syphilis	Number	1997	200774	285122	142153
Prevalence	Global	Both	65-69 yea	Syphilis	Rate	1997	136.315	193.583	96.5143
Prevalence	Global	Both	65-69 yea	Syphilis	Number	1998	201827	287486	142633
Prevalence	Global	Both	65-69 yea	Syphilis	Rate	1998	135.206	192.59	95.5512
Prevalence	Global	Both	65-69 yea	Syphilis	Number	1999	202638	288974	142944
Prevalence	Global	Both	65-69 yea	Syphilis	Rate	1999	134.148	191.303	94.63
Prevalence	Global	Both	65-69 yea	Syphilis	Number	2000	204126	291585	143622
Prevalence	Global	Both	65-69 yea	Syphilis	Rate	2000	133.261	190.357	93.761
Prevalence	Global	Both	65-69 yea	Syphilis	Number	2001	206320	294233	145075
Prevalence	Global	Both	65-69 yea	Syphilis	Rate	2001	132.333	188.72	93.0506
Prevalence	Global	Both	65-69 yea	Syphilis	Number	2002	208842	298661	146727
Prevalence	Global	Both	65-69 yea	Syphilis	Rate	2002	131.083	187.46	92.0957
Prevalence	Global	Both	65-69 yea	Syphilis	Number	2003	211253	302813	148198
Prevalence	Global	Both	65-69 yea	Syphilis	Rate	2003	129.867	186.154	91.1044
Prevalence	Global	Both	65-69 yea	Syphilis	Number	2004	213485	305626	149663
Prevalence	Global	Both	65-69 yea	Syphilis	Rate	2004	128.846	184.457	90.3275
Prevalence	Global	Both	65-69 yea	Syphilis	Number	2005	215957	308728	151372
Prevalence	Global	Both	65-69 yea	Syphilis	Rate	2005	128.098	183.127	89.789
Prevalence	Global	Both	65-69 yea	Syphilis	Number	2006	218033	311988	152740
Prevalence	Global	Both	65-69 yea	Syphilis	Rate	2006	127.519	182.47	89.3316
Prevalence	Global	Both	65-69 yea	Syphilis	Number	2007	219766	314740	153717
Prevalence	Global	Both	65-69 yea	Syphilis	Rate	2007	127.212	182.187	88.9791
Prevalence	Global	Both	65-69 yea	Syphilis	Number	2008	221631	317631	154843
Prevalence	Global	Both	65-69 yea	Syphilis	Rate	2008	127.104	182.159	88.8014
Prevalence	Global	Both	65-69 yea	Syphilis	Number	2009	223987	321274	156197
Prevalence	Global	Both	65-69 yea	Syphilis	Rate	2009	127.018	182.187	88.5755
Prevalence	Global	Both	65-69 yea	Syphilis	Number	2010	226708	326368	158283
Prevalence	Global	Both	65-69 yea	Syphilis	Rate	2010	127.121	183.003	88.7535
Prevalence	Global	Both	65-69 yea	Syphilis	Number	2011	231386	332524	161697
Prevalence	Global	Both	65-69 yea	Syphilis	Rate	2011	126.981	182.483	88.7361
Prevalence	Global	Both	65-69 yea	Syphilis	Number	2012	239505	343881	167221
Prevalence	Global	Both	65-69 yea	Syphilis	Rate	2012	125.632	180.383	87.7156
Prevalence	Global	Both	65-69 yea	Syphilis	Number	2013	248055	356151	172973
Prevalence	Global	Both	65-69 yea	Syphilis	Rate	2013	124.118	178.206	86.5497
Prevalence	Global	Both	65-69 yea	Syphilis	Number	2014	258908	371684	180171
Prevalence	Global	Both	65-69 yea	Syphilis	Rate	2014	122.569	175.957	85.2944
Prevalence	Global	Both	65-69 yea	Syphilis	Number	2015	267225	383721	185508
Prevalence	Global	Both	65-69 yea	Syphilis	Rate	2015	121.168	173.991	84.1152
Prevalence	Global	Both	65-69 yea	Syphilis	Number	2016	282220	407990	195549
Prevalence	Global	Both	65-69 yea	Syphilis	Rate	2016	121.383	175.477	84.1062
Prevalence	Global	Both	65-69 yea	Syphilis	Number	2017	294919	430664	203158
Prevalence	Global	Both	65-69 yea	Syphilis	Rate	2017	122.307	178.603	84.2527
Prevalence	Global	Both	65-69 yea	Syphilis	Number	2018	307018	449454	211852
Prevalence	Global	Both	65-69 yea	Syphilis	Rate	2018	122.663	179.57	84.6414
Prevalence	Global	Both	65-69 yea	Syphilis	Number	2019	317830	466314	219035
Prevalence	Global	Both	65-69 yea	Syphilis	Rate	2019	122.912	180.334	84.7058
Prevalence	Global	Both	50-69 yea	Syphilis	Number	1990	2577325	3802850	1722579
Prevalence	Global	Both	50-69 yea	Syphilis	Rate	1990	377.83	557.49	252.526
Prevalence	Global	Both	50-69 yea	Syphilis	Number	1991	2610270	3849135	1751983
Prevalence	Global	Both	50-69 yea	Syphilis	Rate	1991	376.061	554.544	252.408
Prevalence	Global	Both	50-69 yea	Syphilis	Number	1992	2640307	3891461	1777783
Prevalence	Global	Both	50-69 yea	Syphilis	Rate	1992	374.546	552.031	252.191
Prevalence	Global	Both	50-69 yea	Syphilis	Number	1993	2667800	3930377	1798786
Prevalence	Global	Both	50-69 yea	Syphilis	Rate	1993	373.242	549.884	251.662
Prevalence	Global	Both	50-69 yea	Syphilis	Number	1994	2696749	3980554	1821909
Prevalence	Global	Both	50-69 yea	Syphilis	Rate	1994	372.447	549.753	251.623
Prevalence	Global	Both	50-69 yea	Syphilis	Number	1995	2726260	4040126	1846843
Prevalence	Global	Both	50-69 yea	Syphilis	Rate	1995	371.961	551.219	251.976
Prevalence	Global	Both	50-69 yea	Syphilis	Number	1996	2764275	4094727	1870887
Prevalence	Global	Both	50-69 yea	Syphilis	Rate	1996	371.672	550.558	251.551
Prevalence	Global	Both	50-69 yea	Syphilis	Number	1997	2814019	4166576	1900955
Prevalence	Global	Both	50-69 yea	Syphilis	Rate	1997	370.9	549.173	250.554
Prevalence	Global	Both	50-69 yea	Syphilis	Number	1998	2864071	4239938	1933164
Prevalence	Global	Both	50-69 yea	Syphilis	Rate	1998	369.954	547.676	249.708
Prevalence	Global	Both	50-69 yea	Syphilis	Number	1999	2923129	4327194	1969621
Prevalence	Global	Both	50-69 yea	Syphilis	Rate	1999	369.032	546.289	248.656
Prevalence	Global	Both	50-69 yea	Syphilis	Number	2000	2971285	4399958	2000781
Prevalence	Global	Both	50-69 yea	Syphilis	Rate	2000	367.632	544.399	247.553
Prevalence	Global	Both	50-69 yea	Syphilis	Number	2001	3050774	4524077	2049548
Prevalence	Global	Both	50-69 yea	Syphilis	Rate	2001	367.681	545.244	247.013
Prevalence	Global	Both	50-69 yea	Syphilis	Number	2002	3122150	4628317	2093738
Prevalence	Global	Both	50-69 yea	Syphilis	Rate	2002	366.217	542.886	245.588
Prevalence	Global	Both	50-69 yea	Syphilis	Number	2003	3194334	4732180	2138309
Prevalence	Global	Both	50-69 yea	Syphilis	Rate	2003	364.296	539.679	243.862
Prevalence	Global	Both	50-69 yea	Syphilis	Number	2004	3272116	4842871	2186240
Prevalence	Global	Both	50-69 yea	Syphilis	Rate	2004	362.439	536.425	242.161
Prevalence	Global	Both	50-69 yea	Syphilis	Number	2005	3364226	4981548	2242844
Prevalence	Global	Both	50-69 yea	Syphilis	Rate	2005	361.775	535.696	241.186
Prevalence	Global	Both	50-69 yea	Syphilis	Number	2006	3447195	5101696	2301207
Prevalence	Global	Both	50-69 yea	Syphilis	Rate	2006	360.294	533.219	240.518
Prevalence	Global	Both	50-69 yea	Syphilis	Number	2007	3517713	5196480	2354355
Prevalence	Global	Both	50-69 yea	Syphilis	Rate	2007	357.768	528.506	239.449
Prevalence	Global	Both	50-69 yea	Syphilis	Number	2008	3584253	5282198	2406439
Prevalence	Global	Both	50-69 yea	Syphilis	Rate	2008	355.13	523.363	238.431
Prevalence	Global	Both	50-69 yea	Syphilis	Number	2009	3655408	5374726	2461926
Prevalence	Global	Both	50-69 yea	Syphilis	Rate	2009	352.871	518.844	237.659
Prevalence	Global	Both	50-69 yea	Syphilis	Number	2010	3748127	5506812	2528196
Prevalence	Global	Both	50-69 yea	Syphilis	Rate	2010	352.281	517.577	237.621
Prevalence	Global	Both	50-69 yea	Syphilis	Number	2011	3847158	5652218	2593299
Prevalence	Global	Both	50-69 yea	Syphilis	Rate	2011	351.624	516.604	237.024
Prevalence	Global	Both	50-69 yea	Syphilis	Number	2012	3950956	5807137	2662605
Prevalence	Global	Both	50-69 yea	Syphilis	Rate	2012	350.521	515.198	236.221
Prevalence	Global	Both	50-69 yea	Syphilis	Number	2013	4066108	5980774	2741233
Prevalence	Global	Both	50-69 yea	Syphilis	Rate	2013	349.478	514.042	235.606
Prevalence	Global	Both	50-69 yea	Syphilis	Number	2014	4189997	6167562	2825076
Prevalence	Global	Both	50-69 yea	Syphilis	Rate	2014	348.627	513.17	235.06
Prevalence	Global	Both	50-69 yea	Syphilis	Number	2015	4326780	6374284	2919106
Prevalence	Global	Both	50-69 yea	Syphilis	Rate	2015	348.583	513.539	235.175
Prevalence	Global	Both	50-69 yea	Syphilis	Number	2016	4507308	6661115	3019409
Prevalence	Global	Both	50-69 yea	Syphilis	Rate	2016	352.491	520.927	236.131
Prevalence	Global	Both	50-69 yea	Syphilis	Number	2017	4696841	6965600	3129762
Prevalence	Global	Both	50-69 yea	Syphilis	Rate	2017	357.754	530.564	238.391
Prevalence	Global	Both	50-69 yea	Syphilis	Number	2018	4854923	7194012	3225163
Prevalence	Global	Both	50-69 yea	Syphilis	Rate	2018	360.476	534.153	239.467
Prevalence	Global	Both	50-69 yea	Syphilis	Number	2019	5022129	7438333	3334840
Prevalence	Global	Both	50-69 yea	Syphilis	Rate	2019	364.2	539.421	241.839
Prevalence	Global	Both	50-54 yea	Gonococca	Rate	2012	288.023	489.462	168.923
Prevalence	Global	Both	50-54 yea	Gonococca	Number	2013	1063792	1812070	622837
Prevalence	Global	Both	50-54 yea	Gonococca	Rate	2013	286.906	488.717	167.98
Prevalence	Global	Both	50-54 yea	Gonococca	Number	2014	1100910	1884258	643658
Prevalence	Global	Both	50-54 yea	Gonococca	Rate	2014	285.782	489.129	167.085
Prevalence	Global	Both	50-54 yea	Gonococca	Number	2015	1138163	1958472	664415
Prevalence	Global	Both	50-54 yea	Gonococca	Rate	2015	284.849	490.149	166.284
Prevalence	Global	Both	50-54 yea	Gonococca	Number	2016	1166924	2003064	677929
Prevalence	Global	Both	50-54 yea	Gonococca	Rate	2016	283.28	486.259	164.572
Prevalence	Global	Both	50-54 yea	Gonococca	Number	2017	1191422	2041702	693335
Prevalence	Global	Both	50-54 yea	Gonococca	Rate	2017	281.961	483.187	164.084
Prevalence	Global	Both	50-54 yea	Gonococca	Number	2018	1221968	2084911	710307
Prevalence	Global	Both	50-54 yea	Gonococca	Rate	2018	283.801	484.219	164.968
Prevalence	Global	Both	50-54 yea	Gonococca	Number	2019	1260333	2155698	729043
Prevalence	Global	Both	50-54 yea	Gonococca	Rate	2019	288.527	493.502	166.899
Prevalence	Global	Both	55-59 yea	Gonococca	Number	1990	325648	584769	175110
Prevalence	Global	Both	55-59 yea	Gonococca	Rate	1990	175.631	315.383	94.4417
Prevalence	Global	Both	55-59 yea	Gonococca	Number	1991	329030	590493	177243
Prevalence	Global	Both	55-59 yea	Gonococca	Rate	1991	175.056	314.164	94.3001
Prevalence	Global	Both	55-59 yea	Gonococca	Number	1992	334140	601110	179833
Prevalence	Global	Both	55-59 yea	Gonococca	Rate	1992	174.75	314.371	94.0501
Prevalence	Global	Both	55-59 yea	Gonococca	Number	1993	339112	612496	182634
Prevalence	Global	Both	55-59 yea	Gonococca	Rate	1993	174.482	315.145	93.9698
Prevalence	Global	Both	55-59 yea	Gonococca	Number	1994	343816	623500	185336
Prevalence	Global	Both	55-59 yea	Gonococca	Rate	1994	174.402	316.273	94.0126
Prevalence	Global	Both	55-59 yea	Gonococca	Number	1995	348241	634132	187847
Prevalence	Global	Both	55-59 yea	Gonococca	Rate	1995	174.307	317.405	94.0241
Prevalence	Global	Both	55-59 yea	Gonococca	Number	1996	350121	635677	188638
Prevalence	Global	Both	55-59 yea	Gonococca	Rate	1996	173.563	315.119	93.5119
Prevalence	Global	Both	55-59 yea	Gonococca	Number	1997	349312	632289	187987
Prevalence	Global	Both	55-59 yea	Gonococca	Rate	1997	172.197	311.693	92.6698
Prevalence	Global	Both	55-59 yea	Gonococca	Number	1998	346041	624561	186079
Prevalence	Global	Both	55-59 yea	Gonococca	Rate	1998	169.946	306.732	91.3863
Prevalence	Global	Both	55-59 yea	Gonococca	Number	1999	343297	617696	184574
Prevalence	Global	Both	55-59 yea	Gonococca	Rate	1999	167.695	301.735	90.1612
Prevalence	Global	Both	55-59 yea	Gonococca	Number	2000	341365	612350	183539
Prevalence	Global	Both	55-59 yea	Gonococca	Rate	2000	165.829	297.469	89.1599
Prevalence	Global	Both	55-59 yea	Gonococca	Number	2001	344524	619601	185152
Prevalence	Global	Both	55-59 yea	Gonococca	Rate	2001	164.585	295.995	88.4507
Prevalence	Global	Both	55-59 yea	Gonococca	Number	2002	355447	640216	190682
Prevalence	Global	Both	55-59 yea	Gonococca	Rate	2002	163.194	293.939	87.5468
Prevalence	Global	Both	55-59 yea	Gonococca	Number	2003	369612	666541	197941
Prevalence	Global	Both	55-59 yea	Gonococca	Rate	2003	162.558	293.15	87.056
Prevalence	Global	Both	55-59 yea	Gonococca	Number	2004	388615	701462	207730
Prevalence	Global	Both	55-59 yea	Gonococca	Rate	2004	162.258	292.881	86.7334
Prevalence	Global	Both	55-59 yea	Gonococca	Number	2005	404927	731285	216222
Prevalence	Global	Both	55-59 yea	Gonococca	Rate	2005	162.24	293.001	86.6327
Prevalence	Global	Both	55-59 yea	Gonococca	Number	2006	425982	767284	227100
Prevalence	Global	Both	55-59 yea	Gonococca	Rate	2006	162.247	292.241	86.4972
Prevalence	Global	Both	55-59 yea	Gonococca	Number	2007	441972	794977	235586
Prevalence	Global	Both	55-59 yea	Gonococca	Rate	2007	162.532	292.346	86.6349
Prevalence	Global	Both	55-59 yea	Gonococca	Number	2008	459294	826731	245195
Prevalence	Global	Both	55-59 yea	Gonococca	Rate	2008	162.868	293.162	86.9471
Prevalence	Global	Both	55-59 yea	Gonococca	Number	2009	475171	855756	254745
Prevalence	Global	Both	55-59 yea	Gonococca	Rate	2009	163.224	293.957	87.5063
Prevalence	Global	Both	55-59 yea	Gonococca	Number	2010	496128	890357	266183
Prevalence	Global	Both	55-59 yea	Gonococca	Rate	2010	163.522	293.459	87.7332
Prevalence	Global	Both	55-59 yea	Gonococca	Number	2011	508584	912699	273437
Prevalence	Global	Both	55-59 yea	Gonococca	Rate	2011	163.535	293.479	87.9236
Prevalence	Global	Both	55-59 yea	Gonococca	Number	2012	517700	928806	278668
Prevalence	Global	Both	55-59 yea	Gonococca	Rate	2012	163.005	292.447	87.7425
Prevalence	Global	Both	55-59 yea	Gonococca	Number	2013	522533	936909	281871
Prevalence	Global	Both	55-59 yea	Gonococca	Rate	2013	162.068	290.59	87.4245
Prevalence	Global	Both	55-59 yea	Gonococca	Number	2014	525025	940701	283631
Prevalence	Global	Both	55-59 yea	Gonococca	Rate	2014	160.981	288.434	86.9659
Prevalence	Global	Both	55-59 yea	Gonococca	Number	2015	528099	942492	285656
Prevalence	Global	Both	55-59 yea	Gonococca	Rate	2015	159.977	285.51	86.534
Prevalence	Global	Both	55-59 yea	Gonococca	Number	2016	531862	953295	289419
Prevalence	Global	Both	55-59 yea	Gonococca	Rate	2016	158.289	283.713	86.1349
Prevalence	Global	Both	55-59 yea	Gonococca	Number	2017	540432	968353	295237
Prevalence	Global	Both	55-59 yea	Gonococca	Rate	2017	156.797	280.95	85.6577
Prevalence	Global	Both	55-59 yea	Gonococca	Number	2018	560509	1007123	304356
Prevalence	Global	Both	55-59 yea	Gonococca	Rate	2018	157.054	282.194	85.2802
Prevalence	Global	Both	55-59 yea	Gonococca	Number	2019	587239	1055325	316851
Prevalence	Global	Both	55-59 yea	Gonococca	Rate	2019	158.28	284.445	85.4016
Prevalence	Global	Both	60-64 yea	Gonococca	Number	1990	87889.2	147446	50571.1
Prevalence	Global	Both	60-64 yea	Gonococca	Rate	1990	54.7109	91.785	31.4804
Prevalence	Global	Both	60-64 yea	Gonococca	Number	1991	89421.2	150380	51590.4
Prevalence	Global	Both	60-64 yea	Gonococca	Rate	1991	54.5294	91.7022	31.46
Prevalence	Global	Both	60-64 yea	Gonococca	Number	1992	90196	151531	52170.8
Prevalence	Global	Both	60-64 yea	Gonococca	Rate	1992	54.2233	91.0965	31.3637
Prevalence	Global	Both	60-64 yea	Gonococca	Number	1993	90701.6	151887	52419.2
Prevalence	Global	Both	60-64 yea	Gonococca	Rate	1993	53.8723	90.2132	31.1344
Prevalence	Global	Both	60-64 yea	Gonococca	Number	1994	90821.4	152132	52283.9
Prevalence	Global	Both	60-64 yea	Gonococca	Rate	1994	53.4131	89.4707	30.7488
Prevalence	Global	Both	60-64 yea	Gonococca	Number	1995	91201.1	152624	52373.1
Prevalence	Global	Both	60-64 yea	Gonococca	Rate	1995	53.0245	88.7361	30.4498
Prevalence	Global	Both	60-64 yea	Gonococca	Number	1996	91753.4	153642	52726.6
Prevalence	Global	Both	60-64 yea	Gonococca	Rate	1996	52.5583	88.0093	30.2029
Prevalence	Global	Both	60-64 yea	Gonococca	Number	1997	92360	154594	53114.1
Prevalence	Global	Both	60-64 yea	Gonococca	Rate	1997	51.9186	86.9025	29.8571
Prevalence	Global	Both	60-64 yea	Gonococca	Number	1998	92819.4	155358	53214
Prevalence	Global	Both	60-64 yea	Gonococca	Rate	1998	51.2325	85.7511	29.3719
Prevalence	Global	Both	60-64 yea	Gonococca	Number	1999	93356.9	156922	53491.6
Prevalence	Global	Both	60-64 yea	Gonococca	Rate	1999	50.7001	85.221	29.0501
Prevalence	Global	Both	60-64 yea	Gonococca	Number	2000	94328.6	158531	53973.4
Prevalence	Global	Both	60-64 yea	Gonococca	Rate	2000	50.4548	84.7953	28.8695
Prevalence	Global	Both	60-64 yea	Gonococca	Number	2001	95173.8	160460	54375.4
Prevalence	Global	Both	60-64 yea	Gonococca	Rate	2001	50.3216	84.8407	28.7501
Prevalence	Global	Both	60-64 yea	Gonococca	Number	2002	95555.7	161562	54590.4
Prevalence	Global	Both	60-64 yea	Gonococca	Rate	2002	50.1463	84.7856	28.6482
Prevalence	Global	Both	60-64 yea	Gonococca	Number	2003	95524.8	161897	54662.3
Prevalence	Global	Both	60-64 yea	Gonococca	Rate	2003	49.8242	84.4431	28.511
Prevalence	Global	Both	60-64 yea	Gonococca	Number	2004	95719.8	162597	54846.4
Prevalence	Global	Both	60-64 yea	Gonococca	Rate	2004	49.5165	84.1125	28.3724
Prevalence	Global	Both	60-64 yea	Gonococca	Number	2005	96030.3	163480	55096
Prevalence	Global	Both	60-64 yea	Gonococca	Rate	2005	49.2435	83.8314	28.2528
Prevalence	Global	Both	60-64 yea	Gonococca	Number	2006	97907.2	165874	55988.7
Prevalence	Global	Both	60-64 yea	Gonococca	Rate	2006	49.2318	83.4083	28.1534
Prevalence	Global	Both	60-64 yea	Gonococca	Number	2007	102539	172909	58456.4
Prevalence	Global	Both	60-64 yea	Gonococca	Rate	2007	49.4023	83.3063	28.1638
Prevalence	Global	Both	60-64 yea	Gonococca	Number	2008	108189	181454	61809.4
Prevalence	Global	Both	60-64 yea	Gonococca	Rate	2008	49.8208	83.5592	28.4631
Prevalence	Global	Both	60-64 yea	Gonococca	Number	2009	115249	192791	65721.4
Prevalence	Global	Both	60-64 yea	Gonococca	Rate	2009	50.308	84.1565	28.6885
Prevalence	Global	Both	60-64 yea	Gonococca	Number	2010	120744	202288	68709.7
Prevalence	Global	Both	60-64 yea	Gonococca	Rate	2010	50.5374	84.6674	28.7584
Prevalence	Global	Both	60-64 yea	Gonococca	Number	2011	127081	212838	72212.6
Prevalence	Global	Both	60-64 yea	Gonococca	Rate	2011	50.5146	84.603	28.7045
Prevalence	Global	Both	60-64 yea	Gonococca	Number	2012	131691	219897	75148.9
Prevalence	Global	Both	60-64 yea	Gonococca	Rate	2012	50.5172	84.3535	28.8274
Prevalence	Global	Both	60-64 yea	Gonococca	Number	2013	136690	228100	78072.7
Prevalence	Global	Both	60-64 yea	Gonococca	Rate	2013	50.5454	84.3473	28.8699
Prevalence	Global	Both	60-64 yea	Gonococca	Number	2014	141224	235087	80682
Prevalence	Global	Both	60-64 yea	Gonococca	Rate	2014	50.572	84.1843	28.8922
Prevalence	Global	Both	60-64 yea	Gonococca	Number	2015	147513	245193	84406.2
Prevalence	Global	Both	60-64 yea	Gonococca	Rate	2015	50.6862	84.2496	29.0024
Prevalence	Global	Both	60-64 yea	Gonococca	Number	2016	150770	251487	85898.3
Prevalence	Global	Both	60-64 yea	Gonococca	Rate	2016	50.5499	84.318	28.7998
Prevalence	Global	Both	60-64 yea	Gonococca	Number	2017	152585	256286	86269.9
Prevalence	Global	Both	60-64 yea	Gonococca	Rate	2017	50.1069	84.1611	28.3299
Prevalence	Global	Both	60-64 yea	Gonococca	Number	2018	153217	256815	86862
Prevalence	Global	Both	60-64 yea	Gonococca	Rate	2018	49.5765	83.0973	28.1059
Prevalence	Global	Both	60-64 yea	Gonococca	Number	2019	153427	255665	86825.2
Prevalence	Global	Both	60-64 yea	Gonococca	Rate	2019	49.0911	81.8035	27.7809
Prevalence	Global	Both	65-69 yea	Gonococca	Number	1990	26433.3	42567	14537
Prevalence	Global	Both	65-69 yea	Gonococca	Rate	1990	21.4055	34.4704	11.7719
Prevalence	Global	Both	65-69 yea	Gonococca	Number	1991	27276.5	44098.1	14992.1
Prevalence	Global	Both	65-69 yea	Gonococca	Rate	1991	21.4315	34.6485	11.7795
Prevalence	Global	Both	65-69 yea	Gonococca	Number	1992	28129.1	45696.9	15475.3
Prevalence	Global	Both	65-69 yea	Gonococca	Rate	1992	21.4551	34.8546	11.8035
Prevalence	Global	Both	65-69 yea	Gonococca	Number	1993	28930.7	47240.4	15907.5
Prevalence	Global	Both	65-69 yea	Gonococca	Rate	1993	21.4396	35.0084	11.7885
Prevalence	Global	Both	65-69 yea	Gonococca	Number	1994	29721.3	48770.3	16314
Prevalence	Global	Both	65-69 yea	Gonococca	Rate	1994	21.4216	35.1512	11.7583
Prevalence	Global	Both	65-69 yea	Gonococca	Number	1995	30370.7	49960.2	16612.8
Prevalence	Global	Both	65-69 yea	Gonococca	Rate	1995	21.378	35.1671	11.6938
Prevalence	Global	Both	65-69 yea	Gonococca	Number	1996	30823	50666.1	16844.1
Prevalence	Global	Both	65-69 yea	Gonococca	Rate	1996	21.2448	34.9218	11.6098
Prevalence	Global	Both	65-69 yea	Gonococca	Number	1997	30905.5	50713.3	16893.1
Prevalence	Global	Both	65-69 yea	Gonococca	Rate	1997	20.9832	34.4317	11.4695
Prevalence	Global	Both	65-69 yea	Gonococca	Number	1998	30885.4	50577.4	16898.9
Prevalence	Global	Both	65-69 yea	Gonococca	Rate	1998	20.6905	33.8824	11.3208
Prevalence	Global	Both	65-69 yea	Gonococca	Number	1999	30805.9	50434.7	16881.2
Prevalence	Global	Both	65-69 yea	Gonococca	Rate	1999	20.3938	33.3882	11.1755
Prevalence	Global	Both	65-69 yea	Gonococca	Number	2000	30975.9	50912.5	16992.8
Prevalence	Global	Both	65-69 yea	Gonococca	Rate	2000	20.2221	33.2374	11.0935
Prevalence	Global	Both	65-69 yea	Gonococca	Number	2001	31441.5	51516	17240.6
Prevalence	Global	Both	65-69 yea	Gonococca	Rate	2001	20.1664	33.0421	11.0581
Prevalence	Global	Both	65-69 yea	Gonococca	Number	2002	32103.7	52645.6	17586.1
Prevalence	Global	Both	65-69 yea	Gonococca	Rate	2002	20.1504	33.0439	11.0382
Prevalence	Global	Both	65-69 yea	Gonococca	Number	2003	32756.2	53863.4	17920.2
Prevalence	Global	Both	65-69 yea	Gonococca	Rate	2003	20.1369	33.1125	11.0165
Prevalence	Global	Both	65-69 yea	Gonococca	Number	2004	33350.5	54971.1	18233.3
Prevalence	Global	Both	65-69 yea	Gonococca	Rate	2004	20.1283	33.1772	11.0045
Prevalence	Global	Both	65-69 yea	Gonococca	Number	2005	33900.7	55892.9	18477.3
Prevalence	Global	Both	65-69 yea	Gonococca	Rate	2005	20.1088	33.1538	10.9601
Prevalence	Global	Both	65-69 yea	Gonococca	Number	2006	34366.1	56787.1	18716.7
Prevalence	Global	Both	65-69 yea	Gonococca	Rate	2006	20.0994	33.2126	10.9467
Prevalence	Global	Both	65-69 yea	Gonococca	Number	2007	34815	57594.2	18945.6
Prevalence	Global	Both	65-69 yea	Gonococca	Rate	2007	20.1526	33.3383	10.9667
Prevalence	Global	Both	65-69 yea	Gonococca	Number	2008	35219.1	58342.4	19357.1
Prevalence	Global	Both	65-69 yea	Gonococca	Rate	2008	20.198	33.459	11.1012
Prevalence	Global	Both	65-69 yea	Gonococca	Number	2009	35709.3	59252	19613
Prevalence	Global	Both	65-69 yea	Gonococca	Rate	2009	20.2499	33.6004	11.1221
Prevalence	Global	Both	65-69 yea	Gonococca	Number	2010	36100.1	59949.4	19797.5
Prevalence	Global	Both	65-69 yea	Gonococca	Rate	2010	20.2423	33.6152	11.101
Prevalence	Global	Both	65-69 yea	Gonococca	Number	2011	36802	61187.3	20197.1
Prevalence	Global	Both	65-69 yea	Gonococca	Rate	2011	20.1963	33.5785	11.0838
Prevalence	Global	Both	65-69 yea	Gonococca	Number	2012	38310.9	63810.7	21020.3
Prevalence	Global	Both	65-69 yea	Gonococca	Rate	2012	20.096	33.4719	11.0262
Prevalence	Global	Both	65-69 yea	Gonococca	Number	2013	40059.4	66789.9	21887.6
Prevalence	Global	Both	65-69 yea	Gonococca	Rate	2013	20.0444	33.4194	10.9518
Prevalence	Global	Both	65-69 yea	Gonococca	Number	2014	42339.1	70721	23121.3
Prevalence	Global	Both	65-69 yea	Gonococca	Rate	2014	20.0436	33.4798	10.9458
Prevalence	Global	Both	65-69 yea	Gonococca	Number	2015	44196.5	73916.5	24143.2
Prevalence	Global	Both	65-69 yea	Gonococca	Rate	2015	20.0401	33.5161	10.9473
Prevalence	Global	Both	65-69 yea	Gonococca	Number	2016	46414.6	77715.6	25117.1
Prevalence	Global	Both	65-69 yea	Gonococca	Rate	2016	19.963	33.4256	10.8029
Prevalence	Global	Both	65-69 yea	Gonococca	Number	2017	47999.7	79909.3	26035.6
Prevalence	Global	Both	65-69 yea	Gonococca	Rate	2017	19.9062	33.1396	10.7974
Prevalence	Global	Both	65-69 yea	Gonococca	Number	2018	49792.5	82870.2	26961.7
Prevalence	Global	Both	65-69 yea	Gonococca	Rate	2018	19.8936	33.1091	10.772
Prevalence	Global	Both	65-69 yea	Gonococca	Number	2019	51242.9	85363.8	27865.3
Prevalence	Global	Both	65-69 yea	Gonococca	Rate	2019	19.8168	33.0121	10.7761
Prevalence	Global	Both	50-69 yea	Gonococca	Number	1990	1116939	1761284	731390
Prevalence	Global	Both	50-69 yea	Gonococca	Rate	1990	163.741	258.2	107.22
Prevalence	Global	Both	50-69 yea	Gonococca	Number	1991	1132082	1781381	741104
Prevalence	Global	Both	50-69 yea	Gonococca	Rate	1991	163.099	256.643	106.771
Prevalence	Global	Both	50-69 yea	Gonococca	Number	1992	1143357	1797647	750012
Prevalence	Global	Both	50-69 yea	Gonococca	Rate	1992	162.193	255.009	106.394
Prevalence	Global	Both	50-69 yea	Gonococca	Number	1993	1148065	1803515	753316
Prevalence	Global	Both	50-69 yea	Gonococca	Rate	1993	160.622	252.323	105.394
Prevalence	Global	Both	50-69 yea	Gonococca	Number	1994	1150935	1803646	755101
Prevalence	Global	Both	50-69 yea	Gonococca	Rate	1994	158.955	249.101	104.287
Prevalence	Global	Both	50-69 yea	Gonococca	Number	1995	1152736	1801715	755660
Prevalence	Global	Both	50-69 yea	Gonococca	Rate	1995	157.275	245.819	103.099
Prevalence	Global	Both	50-69 yea	Gonococca	Number	1996	1160151	1812686	761173
Prevalence	Global	Both	50-69 yea	Gonococca	Rate	1996	155.989	243.725	102.344
Prevalence	Global	Both	50-69 yea	Gonococca	Number	1997	1179673	1846672	771879
Prevalence	Global	Both	50-69 yea	Gonococca	Rate	1997	155.486	243.4	101.737
Prevalence	Global	Both	50-69 yea	Gonococca	Number	1998	1203018	1884510	786180
Prevalence	Global	Both	50-69 yea	Gonococca	Rate	1998	155.395	243.424	101.552
Prevalence	Global	Both	50-69 yea	Gonococca	Number	1999	1237106	1929696	808300
Prevalence	Global	Both	50-69 yea	Gonococca	Rate	1999	156.179	243.615	102.044
Prevalence	Global	Both	50-69 yea	Gonococca	Number	2000	1267114	1984230	828462
Prevalence	Global	Both	50-69 yea	Gonococca	Rate	2000	156.778	245.505	102.504
Prevalence	Global	Both	50-69 yea	Gonococca	Number	2001	1311563	2061810	855924
Prevalence	Global	Both	50-69 yea	Gonococca	Rate	2001	158.07	248.491	103.156
Prevalence	Global	Both	50-69 yea	Gonococca	Number	2002	1353067	2133510	880552
Prevalence	Global	Both	50-69 yea	Gonococca	Rate	2002	158.71	250.253	103.286
Prevalence	Global	Both	50-69 yea	Gonococca	Number	2003	1399693	2213599	909699
Prevalence	Global	Both	50-69 yea	Gonococca	Rate	2003	159.627	252.449	103.746
Prevalence	Global	Both	50-69 yea	Gonococca	Number	2004	1447740	2289771	939624
Prevalence	Global	Both	50-69 yea	Gonococca	Rate	2004	160.36	253.629	104.078
Prevalence	Global	Both	50-69 yea	Gonococca	Number	2005	1502605	2380648	972159
Prevalence	Global	Both	50-69 yea	Gonococca	Rate	2005	161.584	256.005	104.542
Prevalence	Global	Both	50-69 yea	Gonococca	Number	2006	1544353	2431358	1001393
Prevalence	Global	Both	50-69 yea	Gonococca	Rate	2006	161.413	254.121	104.664
Prevalence	Global	Both	50-69 yea	Gonococca	Number	2007	1574115	2469137	1022734
Prevalence	Global	Both	50-69 yea	Gonococca	Rate	2007	160.095	251.123	104.017
Prevalence	Global	Both	50-69 yea	Gonococca	Number	2008	1597626	2507759	1039888
Prevalence	Global	Both	50-69 yea	Gonococca	Rate	2008	158.294	248.47	103.033
Prevalence	Global	Both	50-69 yea	Gonococca	Number	2009	1619207	2545017	1058529
Prevalence	Global	Both	50-69 yea	Gonococca	Rate	2009	156.308	245.681	102.184
Prevalence	Global	Both	50-69 yea	Gonococca	Number	2010	1649926	2585738	1080295
Prevalence	Global	Both	50-69 yea	Gonococca	Rate	2010	155.074	243.03	101.535
Prevalence	Global	Both	50-69 yea	Gonococca	Number	2011	1682681	2628152	1105136
Prevalence	Global	Both	50-69 yea	Gonococca	Rate	2011	153.795	240.209	101.008
Prevalence	Global	Both	50-69 yea	Gonococca	Number	2012	1719524	2680728	1130419
Prevalence	Global	Both	50-69 yea	Gonococca	Rate	2012	152.553	237.829	100.289
Prevalence	Global	Both	50-69 yea	Gonococca	Number	2013	1763075	2748311	1158713
Prevalence	Global	Both	50-69 yea	Gonococca	Rate	2013	151.535	236.215	99.5902
Prevalence	Global	Both	50-69 yea	Gonococca	Number	2014	1809497	2822410	1189243
Prevalence	Global	Both	50-69 yea	Gonococca	Rate	2014	150.559	234.838	98.9505
Prevalence	Global	Both	50-69 yea	Gonococca	Number	2015	1857971	2900171	1220952
Prevalence	Global	Both	50-69 yea	Gonococca	Rate	2015	149.686	233.65	98.3649
Prevalence	Global	Both	50-69 yea	Gonococca	Number	2016	1895971	2959800	1247334
Prevalence	Global	Both	50-69 yea	Gonococca	Rate	2016	148.273	231.469	97.5468
Prevalence	Global	Both	50-69 yea	Gonococca	Number	2017	1932438	3027310	1271819
Prevalence	Global	Both	50-69 yea	Gonococca	Rate	2017	147.192	230.588	96.8734
Prevalence	Global	Both	50-69 yea	Gonococca	Number	2018	1985487	3109958	1301384
Prevalence	Global	Both	50-69 yea	Gonococca	Rate	2018	147.422	230.913	96.6272
Prevalence	Global	Both	50-69 yea	Gonococca	Number	2019	2052242	3223477	1336547
Prevalence	Global	Both	50-69 yea	Gonococca	Rate	2019	148.827	233.763	96.9251
Prevalence	Global	Both	50-54 yea	Trichomon	Number	1990	6732601	1.1E+07	3315257
Prevalence	Global	Both	50-54 yea	Trichomon	Rate	1990	3166.92	5360.77	1559.45
Prevalence	Global	Both	50-54 yea	Trichomon	Number	1991	6847133	1.2E+07	3376882
Prevalence	Global	Both	50-54 yea	Trichomon	Rate	1991	3186.32	5400.19	1571.44
Prevalence	Global	Both	50-54 yea	Trichomon	Number	1992	6940156	1.2E+07	3428691
Prevalence	Global	Both	50-54 yea	Trichomon	Rate	1992	3208.92	5444.12	1585.33
Prevalence	Global	Both	50-54 yea	Trichomon	Number	1993	7011746	1.2E+07	3469815
Prevalence	Global	Both	50-54 yea	Trichomon	Rate	1993	3229.64	5469.82	1598.21
Prevalence	Global	Both	50-54 yea	Trichomon	Number	1994	7088123	1.2E+07	3511899
Prevalence	Global	Both	50-54 yea	Trichomon	Rate	1994	3249.31	5491.74	1609.91
Prevalence	Global	Both	50-54 yea	Trichomon	Number	1995	7154311	1.2E+07	3545980
Prevalence	Global	Both	50-54 yea	Trichomon	Rate	1995	3265.41	5506.64	1618.48
Prevalence	Global	Both	50-54 yea	Trichomon	Number	1996	7276951	1.2E+07	3609367
Prevalence	Global	Both	50-54 yea	Trichomon	Rate	1996	3272.66	5507.94	1623.24
Prevalence	Global	Both	50-54 yea	Trichomon	Number	1997	7500625	1.3E+07	3727044
Prevalence	Global	Both	50-54 yea	Trichomon	Rate	1997	3251.77	5471.14	1615.8
Prevalence	Global	Both	50-54 yea	Trichomon	Number	1998	7750164	1.3E+07	3859490
Prevalence	Global	Both	50-54 yea	Trichomon	Rate	1998	3227.83	5428.82	1607.42
Prevalence	Global	Both	50-54 yea	Trichomon	Number	1999	8070439	1.4E+07	4021274
Prevalence	Global	Both	50-54 yea	Trichomon	Rate	1999	3199.99	5382.34	1594.47
Prevalence	Global	Both	50-54 yea	Trichomon	Number	2000	8348777	1.4E+07	4154130
Prevalence	Global	Both	50-54 yea	Trichomon	Rate	2000	3183.7	5356.68	1584.13
Prevalence	Global	Both	50-54 yea	Trichomon	Number	2001	8743843	1.5E+07	4354982
Prevalence	Global	Both	50-54 yea	Trichomon	Rate	2001	3175.37	5342.51	1581.53
Prevalence	Global	Both	50-54 yea	Trichomon	Number	2002	9036823	1.5E+07	4506625
Prevalence	Global	Both	50-54 yea	Trichomon	Rate	2002	3172.38	5345.48	1582.05
Prevalence	Global	Both	50-54 yea	Trichomon	Number	2003	9348176	1.6E+07	4667563
Prevalence	Global	Both	50-54 yea	Trichomon	Rate	2003	3167.93	5343.42	1581.75
Prevalence	Global	Both	50-54 yea	Trichomon	Number	2004	9647274	1.6E+07	4821895
Prevalence	Global	Both	50-54 yea	Trichomon	Rate	2004	3170.3	5345.61	1584.58
Prevalence	Global	Both	50-54 yea	Trichomon	Number	2005	1E+07	1.7E+07	5026249
Prevalence	Global	Both	50-54 yea	Trichomon	Rate	2005	3173	5347.28	1586.87
Prevalence	Global	Both	50-54 yea	Trichomon	Number	2006	1E+07	1.7E+07	5149870
Prevalence	Global	Both	50-54 yea	Trichomon	Rate	2006	3177.77	5350.93	1587.65
Prevalence	Global	Both	50-54 yea	Trichomon	Number	2007	1.1E+07	1.8E+07	5256370
Prevalence	Global	Both	50-54 yea	Trichomon	Rate	2007	3183.74	5364.59	1588.05
Prevalence	Global	Both	50-54 yea	Trichomon	Number	2008	1.1E+07	1.8E+07	5334020
Prevalence	Global	Both	50-54 yea	Trichomon	Rate	2008	3190.42	5381.31	1588.69
Prevalence	Global	Both	50-54 yea	Trichomon	Number	2009	1.1E+07	1.8E+07	5395297
Prevalence	Global	Both	50-54 yea	Trichomon	Rate	2009	3197.18	5397.5	1589.85
Prevalence	Global	Both	50-54 yea	Trichomon	Number	2010	1.1E+07	1.9E+07	5465883
Prevalence	Global	Both	50-54 yea	Trichomon	Rate	2010	3204.07	5398.32	1592.17
Prevalence	Global	Both	50-54 yea	Trichomon	Number	2011	1.1E+07	1.9E+07	5579372
Prevalence	Global	Both	50-54 yea	Trichomon	Rate	2011	3207.83	5399.24	1597.2
Prevalence	Global	Both	50-54 yea	Trichomon	Number	2012	1.1E+07	1.9E+07	5728823
Prevalence	Global	Both	50-54 yea	Trichomon	Rate	2012	3206.02	5390.95	1599.14
Prevalence	Global	Both	50-54 yea	Trichomon	Number	2013	1.2E+07	2E+07	5928227
Prevalence	Global	Both	50-54 yea	Trichomon	Rate	2013	3200.95	5381.75	1598.85
Prevalence	Global	Both	50-54 yea	Trichomon	Number	2014	1.2E+07	2.1E+07	6145225
Prevalence	Global	Both	50-54 yea	Trichomon	Rate	2014	3196.93	5376.55	1595.22
Prevalence	Global	Both	50-54 yea	Trichomon	Number	2015	1.3E+07	2.1E+07	6364325
Prevalence	Global	Both	50-54 yea	Trichomon	Rate	2015	3197.11	5379.1	1592.81
Prevalence	Global	Both	50-54 yea	Trichomon	Number	2016	1.3E+07	2.2E+07	6611322
Prevalence	Global	Both	50-54 yea	Trichomon	Rate	2016	3223.29	5427.56	1604.95
Prevalence	Global	Both	50-54 yea	Trichomon	Number	2017	1.4E+07	2.3E+07	6782670
Prevalence	Global	Both	50-54 yea	Trichomon	Rate	2017	3252.78	5474.56	1605.18
Prevalence	Global	Both	50-54 yea	Trichomon	Number	2018	1.4E+07	2.4E+07	6932549
Prevalence	Global	Both	50-54 yea	Trichomon	Rate	2018	3262.35	5499	1610.08
Prevalence	Global	Both	50-54 yea	Trichomon	Number	2019	1.4E+07	2.4E+07	7042743
Prevalence	Global	Both	50-54 yea	Trichomon	Rate	2019	3268.04	5501.75	1612.29
Prevalence	Global	Both	55-59 yea	Trichomon	Number	1990	4514662	7335008	2415067
Prevalence	Global	Both	55-59 yea	Trichomon	Rate	1990	2434.89	3955.98	1302.52
Prevalence	Global	Both	55-59 yea	Trichomon	Number	1991	4621489	7509120	2468241
Prevalence	Global	Both	55-59 yea	Trichomon	Rate	1991	2458.8	3995.13	1313.2
Prevalence	Global	Both	55-59 yea	Trichomon	Number	1992	4738060	7701105	2526819
Prevalence	Global	Both	55-59 yea	Trichomon	Rate	1992	2477.93	4027.56	1321.49
Prevalence	Global	Both	55-59 yea	Trichomon	Number	1993	4840570	7869908	2578235
Prevalence	Global	Both	55-59 yea	Trichomon	Rate	1993	2490.6	4049.27	1326.57
Prevalence	Global	Both	55-59 yea	Trichomon	Number	1994	4928094	8012900	2625219
Prevalence	Global	Both	55-59 yea	Trichomon	Rate	1994	2499.79	4064.57	1331.65
Prevalence	Global	Both	55-59 yea	Trichomon	Number	1995	5010523	8146948	2671715
Prevalence	Global	Both	55-59 yea	Trichomon	Rate	1995	2507.94	4077.83	1337.29
Prevalence	Global	Both	55-59 yea	Trichomon	Number	1996	5060047	8215909	2701473
Prevalence	Global	Both	55-59 yea	Trichomon	Rate	1996	2508.38	4072.8	1339.18
Prevalence	Global	Both	55-59 yea	Trichomon	Number	1997	5080933	8238866	2716788
Prevalence	Global	Both	55-59 yea	Trichomon	Rate	1997	2504.69	4061.43	1339.27
Prevalence	Global	Both	55-59 yea	Trichomon	Number	1998	5085480	8235307	2724443
Prevalence	Global	Both	55-59 yea	Trichomon	Rate	1998	2497.56	4044.49	1338.02
Prevalence	Global	Both	55-59 yea	Trichomon	Number	1999	5104566	8258008	2739884
Prevalence	Global	Both	55-59 yea	Trichomon	Rate	1999	2493.5	4033.91	1338.39
Prevalence	Global	Both	55-59 yea	Trichomon	Number	2000	5136851	8302635	2761736
Prevalence	Global	Both	55-59 yea	Trichomon	Rate	2000	2495.39	4033.27	1341.6
Prevalence	Global	Both	55-59 yea	Trichomon	Number	2001	5234155	8449795	2810980
Prevalence	Global	Both	55-59 yea	Trichomon	Rate	2001	2500.45	4036.62	1342.86

Prevalence	Global	Both	55-59 yea	Trichomon	Rate	2019	2512.05	4076.28	1336.69
Prevalence	Global	Both	60-64 yea	Trichomon	Number	1990	2995092	4387625	1928406
Prevalence	Global	Both	60-64 yea	Trichomon	Rate	1990	1864.44	2731.29	1200.43
Prevalence	Global	Both	60-64 yea	Trichomon	Number	1991	3080323	4512011	1987835
Prevalence	Global	Both	60-64 yea	Trichomon	Rate	1991	1878.4	2751.44	1212.19
Prevalence	Global	Both	60-64 yea	Trichomon	Number	1992	3146499	4606568	2033563
Prevalence	Global	Both	60-64 yea	Trichomon	Rate	1992	1891.59	2769.34	1222.52
Prevalence	Global	Both	60-64 yea	Trichomon	Number	1993	3207659	4693613	2076340
Prevalence	Global	Both	60-64 yea	Trichomon	Rate	1993	1905.19	2787.78	1233.24
Prevalence	Global	Both	60-64 yea	Trichomon	Number	1994	3258253	4752289	2112438
Prevalence	Global	Both	60-64 yea	Trichomon	Rate	1994	1916.22	2794.88	1242.35
Prevalence	Global	Both	60-64 yea	Trichomon	Number	1995	3311570	4811417	2149720
Prevalence	Global	Both	60-64 yea	Trichomon	Rate	1995	1925.35	2797.37	1249.85
Prevalence	Global	Both	60-64 yea	Trichomon	Number	1996	3363579	4888359	2182534
Prevalence	Global	Both	60-64 yea	Trichomon	Rate	1996	1926.73	2800.16	1250.2
Prevalence	Global	Both	60-64 yea	Trichomon	Number	1997	3412638	4960341	2214176
Prevalence	Global	Both	60-64 yea	Trichomon	Rate	1997	1918.35	2788.37	1244.66
Prevalence	Global	Both	60-64 yea	Trichomon	Number	1998	3449479	5014025	2232459
Prevalence	Global	Both	60-64 yea	Trichomon	Rate	1998	1903.97	2767.54	1232.23
Prevalence	Global	Both	60-64 yea	Trichomon	Number	1999	3481290	5079101	2247965
Prevalence	Global	Both	60-64 yea	Trichomon	Rate	1999	1890.61	2758.35	1220.82
Prevalence	Global	Both	60-64 yea	Trichomon	Number	2000	3527661	5143163	2279339
Prevalence	Global	Both	60-64 yea	Trichomon	Rate	2000	1886.89	2750.99	1219.18
Prevalence	Global	Both	60-64 yea	Trichomon	Number	2001	3573059	5200309	2307326
Prevalence	Global	Both	60-64 yea	Trichomon	Rate	2001	1889.2	2749.58	1219.96
Prevalence	Global	Both	60-64 yea	Trichomon	Number	2002	3611014	5246940	2330779
Prevalence	Global	Both	60-64 yea	Trichomon	Rate	2002	1895.01	2753.52	1223.16
Prevalence	Global	Both	60-64 yea	Trichomon	Number	2003	3641789	5280915	2350699
Prevalence	Global	Both	60-64 yea	Trichomon	Rate	2003	1899.5	2754.44	1226.09
Prevalence	Global	Both	60-64 yea	Trichomon	Number	2004	3678789	5335245	2375282
Prevalence	Global	Both	60-64 yea	Trichomon	Rate	2004	1903.06	2759.96	1228.75
Prevalence	Global	Both	60-64 yea	Trichomon	Number	2005	3714249	5388148	2398780
Prevalence	Global	Both	60-64 yea	Trichomon	Rate	2005	1904.64	2763	1230.07
Prevalence	Global	Both	60-64 yea	Trichomon	Number	2006	3780110	5487446	2440334
Prevalence	Global	Both	60-64 yea	Trichomon	Rate	2006	1900.8	2759.32	1227.1
Prevalence	Global	Both	60-64 yea	Trichomon	Number	2007	3903967	5673039	2516553
Prevalence	Global	Both	60-64 yea	Trichomon	Rate	2007	1880.9	2733.23	1212.46
Prevalence	Global	Both	60-64 yea	Trichomon	Number	2008	4044970	5874449	2609695
Prevalence	Global	Both	60-64 yea	Trichomon	Rate	2008	1862.7	2705.18	1201.76
Prevalence	Global	Both	60-64 yea	Trichomon	Number	2009	4229838	6154578	2733145
Prevalence	Global	Both	60-64 yea	Trichomon	Rate	2009	1846.4	2686.58	1193.06
Prevalence	Global	Both	60-64 yea	Trichomon	Number	2010	4398957	6404212	2847523
Prevalence	Global	Both	60-64 yea	Trichomon	Rate	2010	1841.18	2680.48	1191.83
Prevalence	Global	Both	60-64 yea	Trichomon	Number	2011	4653186	6777588	3007843
Prevalence	Global	Both	60-64 yea	Trichomon	Rate	2011	1849.64	2694.09	1195.62
Prevalence	Global	Both	60-64 yea	Trichomon	Number	2012	4876912	7122126	3146341
Prevalence	Global	Both	60-64 yea	Trichomon	Rate	2012	1870.8	2732.08	1206.95
Prevalence	Global	Both	60-64 yea	Trichomon	Number	2013	5122457	7498712	3295516
Prevalence	Global	Both	60-64 yea	Trichomon	Rate	2013	1894.19	2772.89	1218.62
Prevalence	Global	Both	60-64 yea	Trichomon	Number	2014	5353066	7850372	3439231
Prevalence	Global	Both	60-64 yea	Trichomon	Rate	2014	1916.93	2811.21	1231.59
Prevalence	Global	Both	60-64 yea	Trichomon	Number	2015	5615151	8235498	3603470
Prevalence	Global	Both	60-64 yea	Trichomon	Rate	2015	1929.39	2829.76	1238.17
Prevalence	Global	Both	60-64 yea	Trichomon	Number	2016	5710179	8368930	3665205
Prevalence	Global	Both	60-64 yea	Trichomon	Rate	2016	1914.5	2805.92	1228.86
Prevalence	Global	Both	60-64 yea	Trichomon	Number	2017	5773473	8518668	3698952
Prevalence	Global	Both	60-64 yea	Trichomon	Rate	2017	1895.94	2797.42	1214.69
Prevalence	Global	Both	60-64 yea	Trichomon	Number	2018	5853078	8596699	3758398
Prevalence	Global	Both	60-64 yea	Trichomon	Rate	2018	1893.88	2781.63	1216.1
Prevalence	Global	Both	60-64 yea	Trichomon	Number	2019	5922684	8664431	3797178
Prevalence	Global	Both	60-64 yea	Trichomon	Rate	2019	1895.04	2772.3	1214.96
Prevalence	Global	Both	65-69 yea	Trichomon	Number	1990	2057549	2965620	1361338
Prevalence	Global	Both	65-69 yea	Trichomon	Rate	1990	1666.19	2401.53	1102.4
Prevalence	Global	Both	65-69 yea	Trichomon	Number	1991	2133582	3087506	1406339
Prevalence	Global	Both	65-69 yea	Trichomon	Rate	1991	1676.39	2425.9	1104.98
Prevalence	Global	Both	65-69 yea	Trichomon	Number	1992	2205007	3186530	1455736
Prevalence	Global	Both	65-69 yea	Trichomon	Rate	1992	1681.84	2430.48	1110.34
Prevalence	Global	Both	65-69 yea	Trichomon	Number	1993	2271734	3272478	1502304
Prevalence	Global	Both	65-69 yea	Trichomon	Rate	1993	1683.51	2425.13	1113.31
Prevalence	Global	Both	65-69 yea	Trichomon	Number	1994	2336159	3354131	1545963
Prevalence	Global	Both	65-69 yea	Trichomon	Rate	1994	1683.79	2417.49	1114.25
Prevalence	Global	Both	65-69 yea	Trichomon	Number	1995	2391064	3434555	1581721
Prevalence	Global	Both	65-69 yea	Trichomon	Rate	1995	1683.08	2417.59	1113.38
Prevalence	Global	Both	65-69 yea	Trichomon	Number	1996	2435316	3493785	1611854
Prevalence	Global	Both	65-69 yea	Trichomon	Rate	1996	1678.55	2408.1	1110.97
Prevalence	Global	Both	65-69 yea	Trichomon	Number	1997	2460187	3537634	1629622
Prevalence	Global	Both	65-69 yea	Trichomon	Rate	1997	1670.34	2401.87	1106.43
Prevalence	Global	Both	65-69 yea	Trichomon	Number	1998	2481819	3576028	1644239
Prevalence	Global	Both	65-69 yea	Trichomon	Rate	1998	1662.6	2395.62	1101.49
Prevalence	Global	Both	65-69 yea	Trichomon	Number	1999	2501918	3611152	1654050
Prevalence	Global	Both	65-69 yea	Trichomon	Rate	1999	1656.29	2390.61	1095
Prevalence	Global	Both	65-69 yea	Trichomon	Number	2000	2535973	3666536	1675682
Prevalence	Global	Both	65-69 yea	Trichomon	Rate	2000	1655.57	2393.64	1093.94
Prevalence	Global	Both	65-69 yea	Trichomon	Number	2001	2585595	3736407	1707918
Prevalence	Global	Both	65-69 yea	Trichomon	Rate	2001	1658.39	2396.52	1095.45
Prevalence	Global	Both	65-69 yea	Trichomon	Number	2002	2645189	3820306	1747280
Prevalence	Global	Both	65-69 yea	Trichomon	Rate	2002	1660.3	2397.88	1096.71
Prevalence	Global	Both	65-69 yea	Trichomon	Number	2003	2700954	3896771	1784142
Prevalence	Global	Both	65-69 yea	Trichomon	Rate	2003	1660.41	2395.54	1096.8
Prevalence	Global	Both	65-69 yea	Trichomon	Number	2004	2750424	3961615	1817094
Prevalence	Global	Both	65-69 yea	Trichomon	Rate	2004	1659.99	2390.99	1096.69
Prevalence	Global	Both	65-69 yea	Trichomon	Number	2005	2799511	4026241	1850647
Prevalence	Global	Both	65-69 yea	Trichomon	Rate	2005	1660.58	2388.23	1097.74
Prevalence	Global	Both	65-69 yea	Trichomon	Number	2006	2830960	4072542	1870710
Prevalence	Global	Both	65-69 yea	Trichomon	Rate	2006	1655.72	2381.88	1094.11
Prevalence	Global	Both	65-69 yea	Trichomon	Number	2007	2845567	4088043	1879211
Prevalence	Global	Both	65-69 yea	Trichomon	Rate	2007	1647.15	2366.36	1087.78
Prevalence	Global	Both	65-69 yea	Trichomon	Number	2008	2851540	4095690	1882831
Prevalence	Global	Both	65-69 yea	Trichomon	Rate	2008	1635.34	2348.85	1079.79
Prevalence	Global	Both	65-69 yea	Trichomon	Number	2009	2867481	4120261	1895373
Prevalence	Global	Both	65-69 yea	Trichomon	Rate	2009	1626.08	2336.5	1074.82
Prevalence	Global	Both	65-69 yea	Trichomon	Number	2010	2894262	4161422	1916407
Prevalence	Global	Both	65-69 yea	Trichomon	Rate	2010	1622.89	2333.42	1074.58
Prevalence	Global	Both	65-69 yea	Trichomon	Number	2011	2971154	4275338	1967124
Prevalence	Global	Both	65-69 yea	Trichomon	Rate	2011	1630.52	2346.23	1079.52
Prevalence	Global	Both	65-69 yea	Trichomon	Number	2012	3123003	4495727	2064722
Prevalence	Global	Both	65-69 yea	Trichomon	Rate	2012	1638.17	2358.23	1083.05
Prevalence	Global	Both	65-69 yea	Trichomon	Number	2013	3300802	4753137	2181656
Prevalence	Global	Both	65-69 yea	Trichomon	Rate	2013	1651.61	2378.31	1091.63
Prevalence	Global	Both	65-69 yea	Trichomon	Number	2014	3511005	5057620	2321630
Prevalence	Global	Both	65-69 yea	Trichomon	Rate	2014	1662.13	2394.31	1099.07
Prevalence	Global	Both	65-69 yea	Trichomon	Number	2015	3679645	5300928	2434057
Prevalence	Global	Both	65-69 yea	Trichomon	Rate	2015	1668.47	2403.61	1103.68
Prevalence	Global	Both	65-69 yea	Trichomon	Number	2016	3852684	5556426	2544642
Prevalence	Global	Both	65-69 yea	Trichomon	Rate	2016	1657.05	2389.83	1094.46
Prevalence	Global	Both	65-69 yea	Trichomon	Number	2017	3970090	5732683	2615807
Prevalence	Global	Both	65-69 yea	Trichomon	Rate	2017	1646.46	2377.43	1084.82
Prevalence	Global	Both	65-69 yea	Trichomon	Number	2018	4125569	5967546	2724843
Prevalence	Global	Both	65-69 yea	Trichomon	Rate	2018	1648.29	2384.21	1088.66
Prevalence	Global	Both	65-69 yea	Trichomon	Number	2019	4280602	6198254	2833363
Prevalence	Global	Both	65-69 yea	Trichomon	Rate	2019	1655.4	2397	1095.72
Prevalence	Global	Both	60-64 yea	Other sexu	Rate	2016	0	0	0
Prevalence	Global	Both	60-64 yea	Other sexu	Number	2017	0	0	0
Prevalence	Global	Both	60-64 yea	Other sexu	Rate	2017	0	0	0
Prevalence	Global	Both	60-64 yea	Other sexu	Number	2018	0	0	0
Prevalence	Global	Both	60-64 yea	Other sexu	Rate	2018	0	0	0
Prevalence	Global	Both	60-64 yea	Other sexu	Number	2019	0	0	0
Prevalence	Global	Both	60-64 yea	Other sexu	Rate	2019	0	0	0
Prevalence	Global	Both	65-69 yea	Other sexu	Number	1990	0	0	0
Prevalence	Global	Both	65-69 yea	Other sexu	Rate	1990	0	0	0
Prevalence	Global	Both	65-69 yea	Other sexu	Number	1991	0	0	0
Prevalence	Global	Both	65-69 yea	Other sexu	Rate	1991	0	0	0
Prevalence	Global	Both	65-69 yea	Other sexu	Number	1992	0	0	0
Prevalence	Global	Both	65-69 yea	Other sexu	Rate	1992	0	0	0
Prevalence	Global	Both	65-69 yea	Other sexu	Number	1993	0	0	0
Prevalence	Global	Both	65-69 yea	Other sexu	Rate	1993	0	0	0
Prevalence	Global	Both	65-69 yea	Other sexu	Number	1994	0	0	0
Prevalence	Global	Both	65-69 yea	Other sexu	Rate	1994	0	0	0
Prevalence	Global	Both	65-69 yea	Other sexu	Number	1995	0	0	0
Prevalence	Global	Both	65-69 yea	Other sexu	Rate	1995	0	0	0
Prevalence	Global	Both	65-69 yea	Other sexu	Number	1996	0	0	0
Prevalence	Global	Both	65-69 yea	Other sexu	Rate	1996	0	0	0
Prevalence	Global	Both	65-69 yea	Other sexu	Number	1997	0	0	0
Prevalence	Global	Both	65-69 yea	Other sexu	Rate	1997	0	0	0
Prevalence	Global	Both	65-69 yea	Other sexu	Number	1998	0	0	0
Prevalence	Global	Both	65-69 yea	Other sexu	Rate	1998	0	0	0
Prevalence	Global	Both	65-69 yea	Other sexu	Number	1999	0	0	0
Prevalence	Global	Both	65-69 yea	Other sexu	Rate	1999	0	0	0
Prevalence	Global	Both	65-69 yea	Other sexu	Number	2000	0	0	0
Prevalence	Global	Both	65-69 yea	Other sexu	Rate	2000	0	0	0
Prevalence	Global	Both	65-69 yea	Other sexu	Number	2001	0	0	0
Prevalence	Global	Both	65-69 yea	Other sexu	Rate	2001	0	0	0
Prevalence	Global	Both	65-69 yea	Other sexu	Number	2002	0	0	0
Prevalence	Global	Both	65-69 yea	Other sexu	Rate	2002	0	0	0
Prevalence	Global	Both	65-69 yea	Other sexu	Number	2003	0	0	0
Prevalence	Global	Both	65-69 yea	Other sexu	Rate	2003	0	0	0
Prevalence	Global	Both	65-69 yea	Other sexu	Number	2004	0	0	0
Prevalence	Global	Both	65-69 yea	Other sexu	Rate	2004	0	0	0
Prevalence	Global	Both	65-69 yea	Other sexu	Number	2005	0	0	0
Prevalence	Global	Both	65-69 yea	Other sexu	Rate	2005	0	0	0
Prevalence	Global	Both	65-69 yea	Other sexu	Number	2006	0	0	0
Prevalence	Global	Both	65-69 yea	Other sexu	Rate	2006	0	0	0
Prevalence	Global	Both	65-69 yea	Other sexu	Number	2007	0	0	0
Prevalence	Global	Both	65-69 yea	Other sexu	Rate	2007	0	0	0
Prevalence	Global	Both	65-69 yea	Other sexu	Number	2008	0	0	0
Prevalence	Global	Both	65-69 yea	Other sexu	Rate	2008	0	0	0
Prevalence	Global	Both	65-69 yea	Other sexu	Number	2009	0	0	0
Prevalence	Global	Both	65-69 yea	Other sexu	Rate	2009	0	0	0
Prevalence	Global	Both	65-69 yea	Other sexu	Number	2010	0	0	0
Prevalence	Global	Both	65-69 yea	Other sexu	Rate	2010	0	0	0
Prevalence	Global	Both	65-69 yea	Other sexu	Number	2011	0	0	0
Prevalence	Global	Both	65-69 yea	Other sexu	Rate	2011	0	0	0
Prevalence	Global	Both	65-69 yea	Other sexu	Number	2012	0	0	0
Prevalence	Global	Both	65-69 yea	Other sexu	Rate	2012	0	0	0
Prevalence	Global	Both	65-69 yea	Other sexu	Number	2013	0	0	0
Prevalence	Global	Both	65-69 yea	Other sexu	Rate	2013	0	0	0
Prevalence	Global	Both	65-69 yea	Other sexu	Number	2014	0	0	0
Prevalence	Global	Both	65-69 yea	Other sexu	Rate	2014	0	0	0
Prevalence	Global	Both	65-69 yea	Other sexu	Number	2015	0	0	0
Prevalence	Global	Both	65-69 yea	Other sexu	Rate	2015	0	0	0
Prevalence	Global	Both	65-69 yea	Other sexu	Number	2016	0	0	0
Prevalence	Global	Both	65-69 yea	Other sexu	Rate	2016	0	0	0
Prevalence	Global	Both	65-69 yea	Other sexu	Number	2017	0	0	0
Prevalence	Global	Both	65-69 yea	Other sexu	Rate	2017	0	0	0
Prevalence	Global	Both	65-69 yea	Other sexu	Number	2018	0	0	0
Prevalence	Global	Both	65-69 yea	Other sexu	Rate	2018	0	0	0
Prevalence	Global	Both	65-69 yea	Other sexu	Number	2019	0	0	0
Prevalence	Global	Both	65-69 yea	Other sexu	Rate	2019	0	0	0
Prevalence	Global	Both	50-69 yea	Other sexu	Number	1990	34051.2	52996.6	20834.2
Prevalence	Global	Both	50-69 yea	Other sexu	Rate	1990	4.99183	7.76919	3.05424
Prevalence	Global	Both	50-69 yea	Other sexu	Number	1991	33810.5	52481.4	20776.6
Prevalence	Global	Both	50-69 yea	Other sexu	Rate	1991	4.87107	7.56098	2.99328
Prevalence	Global	Both	50-69 yea	Other sexu	Number	1992	33600.3	51992.2	20750.5
Prevalence	Global	Both	50-69 yea	Other sexu	Rate	1992	4.76644	7.37545	2.9436
Prevalence	Global	Both	50-69 yea	Other sexu	Number	1993	33299.3	51368	20667
Prevalence	Global	Both	50-69 yea	Other sexu	Rate	1993	4.65878	7.18671	2.89145
Prevalence	Global	Both	50-69 yea	Other sexu	Number	1994	33089.8	50929.3	20635.9
Prevalence	Global	Both	50-69 yea	Other sexu	Rate	1994	4.57003	7.03382	2.85002
Prevalence	Global	Both	50-69 yea	Other sexu	Number	1995	32964.8	50600.9	20654.7
Prevalence	Global	Both	50-69 yea	Other sexu	Rate	1995	4.49759	6.90379	2.81805
Prevalence	Global	Both	50-69 yea	Other sexu	Number	1996	33086.7	50825.4	20707.7
Prevalence	Global	Both	50-69 yea	Other sexu	Rate	1996	4.44869	6.83375	2.78427
Prevalence	Global	Both	50-69 yea	Other sexu	Number	1997	33612	51690.1	20936.8
Prevalence	Global	Both	50-69 yea	Other sexu	Rate	1997	4.43021	6.81298	2.75956
Prevalence	Global	Both	50-69 yea	Other sexu	Number	1998	34257.5	52854.5	21207
Prevalence	Global	Both	50-69 yea	Other sexu	Rate	1998	4.42506	6.82726	2.73933
Prevalence	Global	Both	50-69 yea	Other sexu	Number	1999	35201.8	54560.6	21664.7
Prevalence	Global	Both	50-69 yea	Other sexu	Rate	1999	4.44407	6.88802	2.73507
Prevalence	Global	Both	50-69 yea	Other sexu	Number	2000	36009	55998.2	22069.3
Prevalence	Global	Both	50-69 yea	Other sexu	Rate	2000	4.45533	6.92855	2.73059
Prevalence	Global	Both	50-69 yea	Other sexu	Number	2001	37240.8	57871	22796.7
Prevalence	Global	Both	50-69 yea	Other sexu	Rate	2001	4.48828	6.97464	2.74748
Prevalence	Global	Both	50-69 yea	Other sexu	Number	2002	38363.3	59445.9	23520.9
Prevalence	Global	Both	50-69 yea	Other sexu	Rate	2002	4.49988	6.9728	2.75892
Prevalence	Global	Both	50-69 yea	Other sexu	Number	2003	39648.7	61287.5	24355.5
Prevalence	Global	Both	50-69 yea	Other sexu	Rate	2003	4.52171	6.98949	2.77761
Prevalence	Global	Both	50-69 yea	Other sexu	Number	2004	41016.6	63310.1	25304.6
Prevalence	Global	Both	50-69 yea	Other sexu	Rate	2004	4.54324	7.0126	2.80289
Prevalence	Global	Both	50-69 yea	Other sexu	Number	2005	42554.1	65598.2	26342.3
Prevalence	Global	Both	50-69 yea	Other sexu	Rate	2005	4.5761	7.05417	2.83275
Prevalence	Global	Both	50-69 yea	Other sexu	Number	2006	43771.1	67113.4	27170.8
Prevalence	Global	Both	50-69 yea	Other sexu	Rate	2006	4.57487	7.01457	2.83984
Prevalence	Global	Both	50-69 yea	Other sexu	Number	2007	44601.4	67878.6	27781.1
Prevalence	Global	Both	50-69 yea	Other sexu	Rate	2007	4.53616	6.90357	2.82547
Prevalence	Global	Both	50-69 yea	Other sexu	Number	2008	45250.2	68242.6	28368
Prevalence	Global	Both	50-69 yea	Other sexu	Rate	2008	4.48341	6.76151	2.81072
Prevalence	Global	Both	50-69 yea	Other sexu	Number	2009	45752.3	68423.1	28796.3
Prevalence	Global	Both	50-69 yea	Other sexu	Rate	2009	4.41665	6.60516	2.77982
Prevalence	Global	Both	50-69 yea	Other sexu	Number	2010	46521.4	69357.5	29407.5
Prevalence	Global	Both	50-69 yea	Other sexu	Rate	2010	4.37248	6.51881	2.76397
Prevalence	Global	Both	50-69 yea	Other sexu	Number	2011	47299.9	70516.2	29871
Prevalence	Global	Both	50-69 yea	Other sexu	Rate	2011	4.32314	6.44508	2.73016
Prevalence	Global	Both	50-69 yea	Other sexu	Number	2012	48334.4	72244.5	30514.1
Prevalence	Global	Both	50-69 yea	Other sexu	Rate	2012	4.28813	6.4094	2.70715
Prevalence	Global	Both	50-69 yea	Other sexu	Number	2013	49668.7	74474.6	31274.5
Prevalence	Global	Both	50-69 yea	Other sexu	Rate	2013	4.26898	6.40102	2.68801
Prevalence	Global	Both	50-69 yea	Other sexu	Number	2014	51190.4	77125.1	32061.7
Prevalence	Global	Both	50-69 yea	Other sexu	Rate	2014	4.25928	6.41717	2.66768
Prevalence	Global	Both	50-69 yea	Other sexu	Number	2015	52821.7	79920.8	32911
Prevalence	Global	Both	50-69 yea	Other sexu	Rate	2015	4.25554	6.43875	2.65144
Prevalence	Global	Both	50-69 yea	Other sexu	Number	2016	54988.7	84150.3	34166.5
Prevalence	Global	Both	50-69 yea	Other sexu	Rate	2016	4.30035	6.58091	2.67196
Prevalence	Global	Both	50-69 yea	Other sexu	Number	2017	57138.9	88101.9	35314.3
Prevalence	Global	Both	50-69 yea	Other sexu	Rate	2017	4.35222	6.71065	2.68986
Prevalence	Global	Both	50-69 yea	Other sexu	Number	2018	58511.6	90682.6	35797.7
Prevalence	Global	Both	50-69 yea	Other sexu	Rate	2018	4.34446	6.73315	2.65796
Prevalence	Global	Both	50-69 yea	Other sexu	Number	2019	59419.4	92568.9	36149.6
Prevalence	Global	Both	50-69 yea	Other sexu	Rate	2019	4.30904	6.71301	2.62154
Prevalence	Global	Both	50-54 yea	Other sexu	Number	1990	21595.6	35477	11930
Prevalence	Global	Both	50-54 yea	Other sexu	Rate	1990	10.1583	16.6879	5.61172
Prevalence	Global	Both	50-54 yea	Other sexu	Number	1991	21394	35045.6	11852
Prevalence	Global	Both	50-54 yea	Other sexu	Rate	1991	9.95571	16.3085	5.51532
Prevalence	Global	Both	50-54 yea	Other sexu	Number	1992	21128.9	34502	11738.1
Prevalence	Global	Both	50-54 yea	Other sexu	Rate	1992	9.76941	15.9527	5.42737
Prevalence	Global	Both	50-54 yea	Other sexu	Number	1993	20775.6	33819.2	11594.1
Prevalence	Global	Both	50-54 yea	Other sexu	Rate	1993	9.56931	15.5773	5.34027
Prevalence	Global	Both	50-54 yea	Other sexu	Number	1994	20513.3	33315.4	11462.6
Prevalence	Global	Both	50-54 yea	Other sexu	Rate	1994	9.40363	15.2723	5.25464
Prevalence	Global	Both	50-54 yea	Other sexu	Number	1995	20314.3	32982.2	11344.3
Prevalence	Global	Both	50-54 yea	Other sexu	Rate	1995	9.27197	15.0539	5.17784
Prevalence	Global	Both	50-54 yea	Other sexu	Number	1996	20428.2	33168.4	11413.5
Prevalence	Global	Both	50-54 yea	Other sexu	Rate	1996	9.18715	14.9168	5.133
Prevalence	Global	Both	50-54 yea	Other sexu	Number	1997	21001.5	34126.2	11727
Prevalence	Global	Both	50-54 yea	Other sexu	Rate	1997	9.10484	14.7948	5.08404
Prevalence	Global	Both	50-54 yea	Other sexu	Number	1998	21780	35428.9	12151.5
Prevalence	Global	Both	50-54 yea	Other sexu	Rate	1998	9.07105	14.7556	5.06093
Prevalence	Global	Both	50-54 yea	Other sexu	Number	1999	22834.6	37226.3	12735.9
Prevalence	Global	Both	50-54 yea	Other sexu	Rate	1999	9.0541	14.7605	5.04988
Prevalence	Global	Both	50-54 yea	Other sexu	Number	2000	23735.7	38651.1	13244.8
Prevalence	Global	Both	50-54 yea	Other sexu	Rate	2000	9.0513	14.7391	5.05073
Prevalence	Global	Both	50-54 yea	Other sexu	Number	2001	24879.3	40531.3	13867.3
Prevalence	Global	Both	50-54 yea	Other sexu	Rate	2001	9.03504	14.7191	5.03598
Prevalence	Global	Both	50-54 yea	Other sexu	Number	2002	25631.9	41620.1	14289.3
Prevalence	Global	Both	50-54 yea	Other sexu	Rate	2002	8.99808	14.6107	5.01624
Prevalence	Global	Both	50-54 yea	Other sexu	Number	2003	26431.9	42750.9	14769.1
Prevalence	Global	Both	50-54 yea	Other sexu	Rate	2003	8.95729	14.4875	5.00498
Prevalence	Global	Both	50-54 yea	Other sexu	Number	2004	27146.6	43793.5	15226
Prevalence	Global	Both	50-54 yea	Other sexu	Rate	2004	8.92093	14.3915	5.00359
Prevalence	Global	Both	50-54 yea	Other sexu	Number	2005	28134.1	45343	15834
Prevalence	Global	Both	50-54 yea	Other sexu	Rate	2005	8.88243	14.3156	4.99908
Prevalence	Global	Both	50-54 yea	Other sexu	Number	2006	28677	45969.5	16167.9
Prevalence	Global	Both	50-54 yea	Other sexu	Rate	2006	8.8408	14.1719	4.98438
Prevalence	Global	Both	50-54 yea	Other sexu	Number	2007	29119.3	46378.8	16581.2
Prevalence	Global	Both	50-54 yea	Other sexu	Rate	2007	8.7975	14.012	5.00951
Prevalence	Global	Both	50-54 yea	Other sexu	Number	2008	29370.2	46599.6	16954.4
Prevalence	Global	Both	50-54 yea	Other sexu	Rate	2008	8.74763	13.8793	5.04971
Prevalence	Global	Both	50-54 yea	Other sexu	Number	2009	29513.8	46664.9	17164.1
Prevalence	Global	Both	50-54 yea	Other sexu	Rate	2009	8.69689	13.7509	5.0578
Prevalence	Global	Both	50-54 yea	Other sexu	Number	2010	29702.6	46938.7	17400.1
Prevalence	Global	Both	50-54 yea	Other sexu	Rate	2010	8.65214	13.6729	5.06852
Prevalence	Global	Both	50-54 yea	Other sexu	Number	2011	30144.3	47646	17661.6
Prevalence	Global	Both	50-54 yea	Other sexu	Rate	2011	8.62935	13.6395	5.05597
Prevalence	Global	Both	50-54 yea	Other sexu	Number	2012	30918.4	48894.7	18092.1
Prevalence	Global	Both	50-54 yea	Other sexu	Rate	2012	8.63056	13.6485	5.05022
Prevalence	Global	Both	50-54 yea	Other sexu	Number	2013	32109.2	50850.4	18741.8
Prevalence	Global	Both	50-54 yea	Other sexu	Rate	2013	8.65988	13.7144	5.05469
Prevalence	Global	Both	50-54 yea	Other sexu	Number	2014	33542.6	53221	19523
Prevalence	Global	Both	50-54 yea	Other sexu	Rate	2014	8.70723	13.8155	5.0679
Prevalence	Global	Both	50-54 yea	Other sexu	Number	2015	35042.5	55718.3	20363.7
Prevalence	Global	Both	50-54 yea	Other sexu	Rate	2015	8.77011	13.9447	5.09644
Prevalence	Global	Both	50-54 yea	Other sexu	Number	2016	36794.8	58881.9	21279
Prevalence	Global	Both	50-54 yea	Other sexu	Rate	2016	8.93221	14.294	5.16564
Prevalence	Global	Both	50-54 yea	Other sexu	Number	2017	38341.1	61731.5	22101.6
Prevalence	Global	Both	50-54 yea	Other sexu	Rate	2017	9.07376	14.6093	5.23054
Prevalence	Global	Both	50-54 yea	Other sexu	Number	2018	39047.7	62919.9	22278.2
Prevalence	Global	Both	50-54 yea	Other sexu	Rate	2018	9.06879	14.6131	5.17411
Prevalence	Global	Both	50-54 yea	Other sexu	Number	2019	39272.7	63578.6	21973.1
Prevalence	Global	Both	50-54 yea	Other sexu	Rate	2019	8.99066	14.555	5.03028
Prevalence	Global	Both	55-59 yea	Other sexu	Number	1990	12455.6	18335.2	7721.8
Prevalence	Global	Both	55-59 yea	Other sexu	Rate	1990	6.71765	9.88873	4.16459
Prevalence	Global	Both	55-59 yea	Other sexu	Number	1991	12416.5	18201.9	7703.84
Prevalence	Global	Both	55-59 yea	Other sexu	Rate	1991	6.60605	9.68408	4.09873
Prevalence	Global	Both	55-59 yea	Other sexu	Number	1992	12471.4	18212	7749.51
Prevalence	Global	Both	55-59 yea	Other sexu	Rate	1992	6.52234	9.5246	4.05288
Prevalence	Global	Both	55-59 yea	Other sexu	Number	1993	12523.7	18273.6	7792.62
Prevalence	Global	Both	55-59 yea	Other sexu	Rate	1993	6.44378	9.40224	4.00951
Prevalence	Global	Both	55-59 yea	Other sexu	Number	1994	12576.5	18338.5	7834.69
Prevalence	Global	Both	55-59 yea	Other sexu	Rate	1994	6.37949	9.30228	3.97418
Prevalence	Global	Both	55-59 yea	Other sexu	Number	1995	12650.5	18423	7888.19
Prevalence	Global	Both	55-59 yea	Other sexu	Rate	1995	6.33199	9.22138	3.94831
Prevalence	Global	Both	55-59 yea	Other sexu	Number	1996	12658.6	18419.5	7896.94
Prevalence	Global	Both	55-59 yea	Other sexu	Rate	1996	6.27513	9.13093	3.91468
Prevalence	Global	Both	55-59 yea	Other sexu	Number	1997	12610.5	18310	7879.06
Prevalence	Global	Both	55-59 yea	Other sexu	Rate	1997	6.21649	9.0261	3.88406
Prevalence	Global	Both	55-59 yea	Other sexu	Number	1998	12477.5	18149.7	7825.75
Prevalence	Global	Both	55-59 yea	Other sexu	Rate	1998	6.12788	8.91359	3.84335
Prevalence	Global	Both	55-59 yea	Other sexu	Number	1999	12367.2	17980.9	7784.05
Prevalence	Global	Both	55-59 yea	Other sexu	Rate	1999	6.0412	8.78341	3.80238
Prevalence	Global	Both	55-59 yea	Other sexu	Number	2000	12273.4	17824.5	7752.62
Prevalence	Global	Both	55-59 yea	Other sexu	Rate	2000	5.96219	8.65884	3.76608
Prevalence	Global	Both	55-59 yea	Other sexu	Number	2001	12361.5	17938.3	7820.54
Prevalence	Global	Both	55-59 yea	Other sexu	Rate	2001	5.9053	8.56946	3.73601
Prevalence	Global	Both	55-59 yea	Other sexu	Number	2002	12731.4	18444.9	8069.99
Prevalence	Global	Both	55-59 yea	Other sexu	Rate	2002	5.8453	8.46852	3.70513
Prevalence	Global	Both	55-59 yea	Other sexu	Number	2003	13216.8	19110	8381.31
Prevalence	Global	Both	55-59 yea	Other sexu	Rate	2003	5.81285	8.40475	3.68617
Prevalence	Global	Both	55-59 yea	Other sexu	Number	2004	13870.1	20017.1	8800.05
Prevalence	Global	Both	55-59 yea	Other sexu	Rate	2004	5.79115	8.35771	3.67427
Prevalence	Global	Both	55-59 yea	Other sexu	Number	2005	14420	20778.7	9152.53
Prevalence	Global	Both	55-59 yea	Other sexu	Rate	2005	5.77759	8.3253	3.6671
Prevalence	Global	Both	55-59 yea	Other sexu	Number	2006	15094.1	21703.7	9582.78
Prevalence	Global	Both	55-59 yea	Other sexu	Rate	2006	5.74901	8.26645	3.64987
Prevalence	Global	Both	55-59 yea	Other sexu	Number	2007	15482.1	22136.8	9909.75
Prevalence	Global	Both	55-59 yea	Other sexu	Rate	2007	5.69341	8.14062	3.64423
Prevalence	Global	Both	55-59 yea	Other sexu	Number	2008	15880	22533.1	10191.6
Prevalence	Global	Both	55-59 yea	Other sexu	Rate	2008	5.63111	7.99033	3.61398
Prevalence	Global	Both	55-59 yea	Other sexu	Number	2009	16238.6	22916.3	10438.3
Prevalence	Global	Both	55-59 yea	Other sexu	Rate	2009	5.57803	7.87189	3.58561
Prevalence	Global	Both	55-59 yea	Other sexu	Number	2010	16818.8	23759	10842.2
Prevalence	Global	Both	55-59 yea	Other sexu	Rate	2010	5.54343	7.83091	3.57357
Prevalence	Global	Both	55-59 yea	Other sexu	Number	2011	17155.7	24284.5	11058.6
Prevalence	Global	Both	55-59 yea	Other sexu	Rate	2011	5.51642	7.80867	3.55589
Prevalence	Global	Both	55-59 yea	Other sexu	Number	2012	17416	24676.2	11214.2
Prevalence	Global	Both	55-59 yea	Other sexu	Rate	2012	5.48366	7.76964	3.53094
Prevalence	Global	Both	55-59 yea	Other sexu	Number	2013	17559.6	24910.8	11309.9
Prevalence	Global	Both	55-59 yea	Other sexu	Rate	2013	5.44624	7.72628	3.50785
Prevalence	Global	Both	55-59 yea	Other sexu	Number	2014	17647.7	25071.3	11382.5
Prevalence	Global	Both	55-59 yea	Other sexu	Rate	2014	5.41107	7.68727	3.49005
Prevalence	Global	Both	55-59 yea	Other sexu	Number	2015	17779.3	25292.7	11488.4
Prevalence	Global	Both	55-59 yea	Other sexu	Rate	2015	5.38589	7.66193	3.4802
Prevalence	Global	Both	55-59 yea	Other sexu	Number	2016	18194	26030.6	11795.1
Prevalence	Global	Both	55-59 yea	Other sexu	Rate	2016	5.41477	7.74706	3.51037
Prevalence	Global	Both	55-59 yea	Other sexu	Number	2017	18797.8	26983.6	12200
Prevalence	Global	Both	55-59 yea	Other sexu	Rate	2017	5.45384	7.82881	3.53962
Prevalence	Global	Both	55-59 yea	Other sexu	Number	2018	19463.9	28034.9	12518.2
Prevalence	Global	Both	55-59 yea	Other sexu	Rate	2018	5.45377	7.85534	3.50758
Prevalence	Global	Both	55-59 yea	Other sexu	Number	2019	20146.7	29125	12917
Prevalence	Global	Both	55-59 yea	Other sexu	Rate	2019	5.4302	7.85014	3.48157
Prevalence	Global	Both	60-64 yea	Other sexu	Number	1990	0	0	0
Prevalence	Global	Both	60-64 yea	Other sexu	Rate	1990	0	0	0
Prevalence	Global	Both	60-64 yea	Other sexu	Number	1991	0	0	0
Prevalence	Global	Both	60-64 yea	Other sexu	Rate	1991	0	0	0
Prevalence	Global	Both	60-64 yea	Other sexu	Number	1992	0	0	0
Prevalence	Global	Both	60-64 yea	Other sexu	Rate	1992	0	0	0
Prevalence	Global	Both	60-64 yea	Other sexu	Number	1993	0	0	0
Prevalence	Global	Both	60-64 yea	Other sexu	Rate	1993	0	0	0
Prevalence	Global	Both	60-64 yea	Other sexu	Number	1994	0	0	0
Prevalence	Global	Both	60-64 yea	Other sexu	Rate	1994	0	0	0
Prevalence	Global	Both	60-64 yea	Other sexu	Number	1995	0	0	0
Prevalence	Global	Both	60-64 yea	Other sexu	Rate	1995	0	0	0
Prevalence	Global	Both	60-64 yea	Other sexu	Number	1996	0	0	0
Prevalence	Global	Both	60-64 yea	Other sexu	Rate	1996	0	0	0
Prevalence	Global	Both	60-64 yea	Other sexu	Number	1997	0	0	0
Prevalence	Global	Both	60-64 yea	Other sexu	Rate	1997	0	0	0
Prevalence	Global	Both	60-64 yea	Other sexu	Number	1998	0	0	0
Prevalence	Global	Both	60-64 yea	Other sexu	Rate	1998	0	0	0
Prevalence	Global	Both	60-64 yea	Other sexu	Number	1999	0	0	0
Prevalence	Global	Both	60-64 yea	Other sexu	Rate	1999	0	0	0
Prevalence	Global	Both	60-64 yea	Other sexu	Number	2000	0	0	0
Prevalence	Global	Both	60-64 yea	Other sexu	Rate	2000	0	0	0
Prevalence	Global	Both	60-64 yea	Other sexu	Number	2001	0	0	0
Prevalence	Global	Both	60-64 yea	Other sexu	Rate	2001	0	0	0
Prevalence	Global	Both	60-64 yea	Other sexu	Number	2002	0	0	0
Prevalence	Global	Both	60-64 yea	Other sexu	Rate	2002	0	0	0
Prevalence	Global	Both	60-64 yea	Other sexu	Number	2003	0	0	0
Prevalence	Global	Both	60-64 yea	Other sexu	Rate	2003	0	0	0
Prevalence	Global	Both	60-64 yea	Other sexu	Number	2004	0	0	0
Prevalence	Global	Both	60-64 yea	Other sexu	Rate	2004	0	0	0
Prevalence	Global	Both	60-64 yea	Other sexu	Number	2005	0	0	0
Prevalence	Global	Both	60-64 yea	Other sexu	Rate	2005	0	0	0
Prevalence	Global	Both	60-64 yea	Other sexu	Number	2006	0	0	0
Prevalence	Global	Both	60-64 yea	Other sexu	Rate	2006	0	0	0
Prevalence	Global	Both	60-64 yea	Other sexu	Number	2007	0	0	0
Prevalence	Global	Both	60-64 yea	Other sexu	Rate	2007	0	0	0
Prevalence	Global	Both	60-64 yea	Other sexu	Number	2008	0	0	0
Prevalence	Global	Both	60-64 yea	Other sexu	Rate	2008	0	0	0
Prevalence	Global	Both	60-64 yea	Other sexu	Number	2009	0	0	0
Prevalence	Global	Both	60-64 yea	Other sexu	Rate	2009	0	0	0
Prevalence	Global	Both	60-64 yea	Other sexu	Number	2010	0	0	0
Prevalence	Global	Both	60-64 yea	Other sexu	Rate	2010	0	0	0
Prevalence	Global	Both	60-64 yea	Other sexu	Number	2011	0	0	0
Prevalence	Global	Both	60-64 yea	Other sexu	Rate	2011	0	0	0
Prevalence	Global	Both	60-64 yea	Other sexu	Number	2012	0	0	0
Prevalence	Global	Both	60-64 yea	Other sexu	Rate	2012	0	0	0
Prevalence	Global	Both	60-64 yea	Other sexu	Number	2013	0	0	0
Prevalence	Global	Both	60-64 yea	Other sexu	Rate	2013	0	0	0
Prevalence	Global	Both	60-64 yea	Other sexu	Number	2014	0	0	0
Prevalence	Global	Both	60-64 yea	Other sexu	Rate	2014	0	0	0
Prevalence	Global	Both	60-64 yea	Other sexu	Number	2015	0	0	0
Prevalence	Global	Both	60-64 yea	Other sexu	Rate	2015	0	0	0
Prevalence	Global	Both	60-64 yea	Other sexu	Number	2016	0	0	0
Prevalence	Global	Both	50-54 yea	Chlamydia	Number	1990	3197855	5282256	1773151
Prevalence	Global	Both	50-54 yea	Chlamydia	Rate	1990	1504.23	2484.7	834.067
Prevalence	Global	Both	50-54 yea	Chlamydia	Number	1991	3220154	5321610	1784488
Prevalence	Global	Both	50-54 yea	Chlamydia	Rate	1991	1498.5	2476.42	830.413
Prevalence	Global	Both	50-54 yea	Chlamydia	Number	1992	3228645	5336091	1788675
Prevalence	Global	Both	50-54 yea	Chlamydia	Rate	1992	1492.83	2467.25	827.031
Prevalence	Global	Both	50-54 yea	Chlamydia	Number	1993	3220685	5322075	1783732
Prevalence	Global	Both	50-54 yea	Chlamydia	Rate	1993	1483.46	2451.37	821.594
Prevalence	Global	Both	50-54 yea	Chlamydia	Number	1994	3216225	5312713	1780457
Prevalence	Global	Both	50-54 yea	Chlamydia	Rate	1994	1474.37	2435.43	816.189
Prevalence	Global	Both	50-54 yea	Chlamydia	Number	1995	3222697	5321317	1782779
Prevalence	Global	Both	50-54 yea	Chlamydia	Rate	1995	1470.92	2428.78	813.705
Prevalence	Global	Both	50-54 yea	Chlamydia	Number	1996	3293776	5445285	1822800
Prevalence	Global	Both	50-54 yea	Chlamydia	Rate	1996	1481.31	2448.9	819.767
Prevalence	Global	Both	50-54 yea	Chlamydia	Number	1997	3440158	5692848	1904598
Prevalence	Global	Both	50-54 yea	Chlamydia	Rate	1997	1491.42	2468.04	825.707
Prevalence	Global	Both	50-54 yea	Chlamydia	Number	1998	3625080	6001692	2007770
Prevalence	Global	Both	50-54 yea	Chlamydia	Rate	1998	1509.79	2499.62	836.207
Prevalence	Global	Both	50-54 yea	Chlamydia	Number	1999	3872766	6413144	2145566
Prevalence	Global	Both	50-54 yea	Chlamydia	Rate	1999	1535.58	2542.86	850.734
Prevalence	Global	Both	50-54 yea	Chlamydia	Number	2000	4054483	6714836	2243427
Prevalence	Global	Both	50-54 yea	Chlamydia	Rate	2000	1546.13	2560.62	855.503
Prevalence	Global	Both	50-54 yea	Chlamydia	Number	2001	4273732	7083043	2366003
Prevalence	Global	Both	50-54 yea	Chlamydia	Rate	2001	1552.03	2572.24	859.225
Prevalence	Global	Both	50-54 yea	Chlamydia	Number	2002	4456658	7390296	2467577
Prevalence	Global	Both	50-54 yea	Chlamydia	Rate	2002	1564.51	2594.36	866.242
Prevalence	Global	Both	50-54 yea	Chlamydia	Number	2003	4660256	7727904	2579256
Prevalence	Global	Both	50-54 yea	Chlamydia	Rate	2003	1579.28	2618.85	874.063
Prevalence	Global	Both	50-54 yea	Chlamydia	Number	2004	4835761	8013917	2675437
Prevalence	Global	Both	50-54 yea	Chlamydia	Rate	2004	1589.13	2633.54	879.205
Prevalence	Global	Both	50-54 yea	Chlamydia	Number	2005	5067693	8391329	2801930
Prevalence	Global	Both	50-54 yea	Chlamydia	Rate	2005	1599.96	2649.29	884.618
Prevalence	Global	Both	50-54 yea	Chlamydia	Number	2006	5155047	8520693	2859663
Prevalence	Global	Both	50-54 yea	Chlamydia	Rate	2006	1589.24	2626.84	881.603
Prevalence	Global	Both	50-54 yea	Chlamydia	Number	2007	5147610	8505100	2866649
Prevalence	Global	Both	50-54 yea	Chlamydia	Rate	2007	1555.19	2569.56	866.071
Prevalence	Global	Both	50-54 yea	Chlamydia	Number	2008	5069692	8377467	2834410
Prevalence	Global	Both	50-54 yea	Chlamydia	Rate	2008	1509.96	2495.15	844.202
Prevalence	Global	Both	50-54 yea	Chlamydia	Number	2009	4992936	8245980	2792011
Prevalence	Global	Both	50-54 yea	Chlamydia	Rate	2009	1471.28	2429.86	822.729
Prevalence	Global	Both	50-54 yea	Chlamydia	Number	2010	4986887	8228597	2782215
Prevalence	Global	Both	50-54 yea	Chlamydia	Rate	2010	1452.64	2396.93	810.439
Prevalence	Global	Both	50-54 yea	Chlamydia	Number	2011	5066724	8353102	2827309
Prevalence	Global	Both	50-54 yea	Chlamydia	Rate	2011	1450.44	2391.23	809.37
Prevalence	Global	Both	50-54 yea	Chlamydia	Number	2012	5209873	8584218	2906880
Prevalence	Global	Both	50-54 yea	Chlamydia	Rate	2012	1454.28	2396.2	811.426
Prevalence	Global	Both	50-54 yea	Chlamydia	Number	2013	5427249	8935910	3026640
Prevalence	Global	Both	50-54 yea	Chlamydia	Rate	2013	1463.73	2410.02	816.288
Prevalence	Global	Both	50-54 yea	Chlamydia	Number	2014	5681907	9344249	3163978
Prevalence	Global	Both	50-54 yea	Chlamydia	Rate	2014	1474.95	2425.64	821.327
Prevalence	Global	Both	50-54 yea	Chlamydia	Number	2015	5935465	9748055	3295953
Prevalence	Global	Both	50-54 yea	Chlamydia	Rate	2015	1485.48	2439.66	824.882
Prevalence	Global	Both	50-54 yea	Chlamydia	Number	2016	6150218	1E+07	3427022
Prevalence	Global	Both	50-54 yea	Chlamydia	Rate	2016	1493.01	2464.19	831.936
Prevalence	Global	Both	50-54 yea	Chlamydia	Number	2017	6327232	1E+07	3509995
Prevalence	Global	Both	50-54 yea	Chlamydia	Rate	2017	1497.4	2479.73	830.672
Prevalence	Global	Both	50-54 yea	Chlamydia	Number	2018	6565994	1.1E+07	3662040
Prevalence	Global	Both	50-54 yea	Chlamydia	Rate	2018	1524.95	2522.74	850.506
Prevalence	Global	Both	50-54 yea	Chlamydia	Number	2019	6898601	1.1E+07	3831814
Prevalence	Global	Both	50-54 yea	Chlamydia	Rate	2019	1579.29	2609.56	877.213
Prevalence	Global	Both	55-59 yea	Chlamydia	Number	1990	982549	1527982	559053
Prevalence	Global	Both	55-59 yea	Chlamydia	Rate	1990	529.917	824.086	301.514
Prevalence	Global	Both	55-59 yea	Chlamydia	Number	1991	995631	1548964	567128
Prevalence	Global	Both	55-59 yea	Chlamydia	Rate	1991	529.713	824.107	301.733
Prevalence	Global	Both	55-59 yea	Chlamydia	Number	1992	1014022	1579137	578098
Prevalence	Global	Both	55-59 yea	Chlamydia	Rate	1992	530.318	825.864	302.336
Prevalence	Global	Both	55-59 yea	Chlamydia	Number	1993	1031160	1606107	588084
Prevalence	Global	Both	55-59 yea	Chlamydia	Rate	1993	530.559	826.384	302.585
Prevalence	Global	Both	55-59 yea	Chlamydia	Number	1994	1046497	1628173	596587
Prevalence	Global	Both	55-59 yea	Chlamydia	Rate	1994	530.84	825.897	302.621
Prevalence	Global	Both	55-59 yea	Chlamydia	Number	1995	1061431	1649585	604822
Prevalence	Global	Both	55-59 yea	Chlamydia	Rate	1995	531.283	825.675	302.735
Prevalence	Global	Both	55-59 yea	Chlamydia	Number	1996	1071990	1667114	611443
Prevalence	Global	Both	55-59 yea	Chlamydia	Rate	1996	531.409	826.425	303.106
Prevalence	Global	Both	55-59 yea	Chlamydia	Number	1997	1079925	1680720	616286
Prevalence	Global	Both	55-59 yea	Chlamydia	Rate	1997	532.359	828.527	303.804
Prevalence	Global	Both	55-59 yea	Chlamydia	Number	1998	1083554	1687162	618407
Prevalence	Global	Both	55-59 yea	Chlamydia	Rate	1998	532.151	828.592	303.709
Prevalence	Global	Both	55-59 yea	Chlamydia	Number	1999	1088183	1694316	620468
Prevalence	Global	Both	55-59 yea	Chlamydia	Rate	1999	531.56	827.646	303.089
Prevalence	Global	Both	55-59 yea	Chlamydia	Number	2000	1093822	1702484	623286
Prevalence	Global	Both	55-59 yea	Chlamydia	Rate	2000	531.359	827.036	302.781
Prevalence	Global	Both	55-59 yea	Chlamydia	Number	2001	1115332	1735212	635542
Prevalence	Global	Both	55-59 yea	Chlamydia	Rate	2001	532.815	828.943	303.61
Prevalence	Global	Both	55-59 yea	Chlamydia	Number	2002	1156109	1794921	658192
Prevalence	Global	Both	55-59 yea	Chlamydia	Rate	2002	530.798	824.092	302.192
Prevalence	Global	Both	55-59 yea	Chlamydia	Number	2003	1205910	1868738	685486
Prevalence	Global	Both	55-59 yea	Chlamydia	Rate	2003	530.369	821.886	301.482
Prevalence	Global	Both	55-59 yea	Chlamydia	Number	2004	1275426	1972415	724009
Prevalence	Global	Both	55-59 yea	Chlamydia	Rate	2004	532.527	823.54	302.294
Prevalence	Global	Both	55-59 yea	Chlamydia	Number	2005	1330469	2057033	754106
Prevalence	Global	Both	55-59 yea	Chlamydia	Rate	2005	533.073	824.183	302.145
Prevalence	Global	Both	55-59 yea	Chlamydia	Number	2006	1398518	2161757	794005
Prevalence	Global	Both	55-59 yea	Chlamydia	Rate	2006	532.665	823.366	302.419
Prevalence	Global	Both	55-59 yea	Chlamydia	Number	2007	1447732	2237684	823411
Prevalence	Global	Both	55-59 yea	Chlamydia	Rate	2007	532.391	822.89	302.803
Prevalence	Global	Both	55-59 yea	Chlamydia	Number	2008	1499578	2317601	852801
Prevalence	Global	Both	55-59 yea	Chlamydia	Rate	2008	531.757	821.831	302.407
Prevalence	Global	Both	55-59 yea	Chlamydia	Number	2009	1545430	2389562	878185
Prevalence	Global	Both	55-59 yea	Chlamydia	Rate	2009	530.864	820.828	301.661
Prevalence	Global	Both	55-59 yea	Chlamydia	Number	2010	1615662	2498699	917609
Prevalence	Global	Both	55-59 yea	Chlamydia	Rate	2010	532.517	823.563	302.441
Prevalence	Global	Both	55-59 yea	Chlamydia	Number	2011	1662441	2570127	944292
Prevalence	Global	Both	55-59 yea	Chlamydia	Rate	2011	534.558	826.425	303.637
Prevalence	Global	Both	55-59 yea	Chlamydia	Number	2012	1701140	2631298	967220
Prevalence	Global	Both	55-59 yea	Chlamydia	Rate	2012	535.627	828.5	304.542
Prevalence	Global	Both	55-59 yea	Chlamydia	Number	2013	1724677	2672131	980908
Prevalence	Global	Both	55-59 yea	Chlamydia	Rate	2013	534.922	828.783	304.236
Prevalence	Global	Both	55-59 yea	Chlamydia	Number	2014	1739053	2700755	989429
Prevalence	Global	Both	55-59 yea	Chlamydia	Rate	2014	533.221	828.094	303.375
Prevalence	Global	Both	55-59 yea	Chlamydia	Number	2015	1753974	2729916	998309
Prevalence	Global	Both	55-59 yea	Chlamydia	Rate	2015	531.332	826.976	302.418
Prevalence	Global	Both	55-59 yea	Chlamydia	Number	2016	1782237	2781132	1013101
Prevalence	Global	Both	55-59 yea	Chlamydia	Rate	2016	530.417	827.702	301.512
Prevalence	Global	Both	55-59 yea	Chlamydia	Number	2017	1831819	2848155	1038975
Prevalence	Global	Both	55-59 yea	Chlamydia	Rate	2017	531.469	826.341	301.44
Prevalence	Global	Both	55-59 yea	Chlamydia	Number	2018	1915878	2985336	1089221
Prevalence	Global	Both	55-59 yea	Chlamydia	Rate	2018	536.826	836.487	305.198
Prevalence	Global	Both	55-59 yea	Chlamydia	Number	2019	2026224	3146912	1149822
Prevalence	Global	Both	55-59 yea	Chlamydia	Rate	2019	546.134	848.196	309.915
Prevalence	Global	Both	60-64 yea	Chlamydia	Number	1990	214192	318902	131828
Prevalence	Global	Both	60-64 yea	Chlamydia	Rate	1990	133.334	198.516	82.0629
Prevalence	Global	Both	60-64 yea	Chlamydia	Number	1991	218529	325119	134437
Prevalence	Global	Both	60-64 yea	Chlamydia	Rate	1991	133.26	198.259	81.9804
Prevalence	Global	Both	60-64 yea	Chlamydia	Number	1992	221488	329272	136150
Prevalence	Global	Both	60-64 yea	Chlamydia	Rate	1992	133.152	197.949	81.8496

Prevalence	Global	Both	60-64 yea	Chlamydia	Rate	2010	131.428	194.72	80.6186
Prevalence	Global	Both	60-64 yea	Chlamydia	Number	2011	331650	491759	203530
Prevalence	Global	Both	60-64 yea	Chlamydia	Rate	2011	131.831	195.474	80.9032
Prevalence	Global	Both	60-64 yea	Chlamydia	Number	2012	346298	513821	212583
Prevalence	Global	Both	60-64 yea	Chlamydia	Rate	2012	132.841	197.104	81.5476
Prevalence	Global	Both	60-64 yea	Chlamydia	Number	2013	362263	537862	222245
Prevalence	Global	Both	60-64 yea	Chlamydia	Rate	2013	133.958	198.892	82.1824
Prevalence	Global	Both	60-64 yea	Chlamydia	Number	2014	376780	559655	230803
Prevalence	Global	Both	60-64 yea	Chlamydia	Rate	2014	134.925	200.412	82.6504
Prevalence	Global	Both	60-64 yea	Chlamydia	Number	2015	395224	587340	241767
Prevalence	Global	Both	60-64 yea	Chlamydia	Rate	2015	135.801	201.813	83.0722
Prevalence	Global	Both	60-64 yea	Chlamydia	Number	2016	406905	603973	249105
Prevalence	Global	Both	60-64 yea	Chlamydia	Rate	2016	136.426	202.499	83.5194
Prevalence	Global	Both	60-64 yea	Chlamydia	Number	2017	416515	617567	255637
Prevalence	Global	Both	60-64 yea	Chlamydia	Rate	2017	136.778	202.801	83.9481
Prevalence	Global	Both	60-64 yea	Chlamydia	Number	2018	422341	626961	258842
Prevalence	Global	Both	60-64 yea	Chlamydia	Rate	2018	136.657	202.865	83.7534
Prevalence	Global	Both	60-64 yea	Chlamydia	Number	2019	426297	633504	260741
Prevalence	Global	Both	60-64 yea	Chlamydia	Rate	2019	136.4	202.698	83.4275
Prevalence	Global	Both	65-69 yea	Chlamydia	Number	1990	56214.2	92730.1	30065.1
Prevalence	Global	Both	65-69 yea	Chlamydia	Rate	1990	45.5218	75.092	24.3464
Prevalence	Global	Both	65-69 yea	Chlamydia	Number	1991	58030.5	95609.4	31045.5
Prevalence	Global	Both	65-69 yea	Chlamydia	Rate	1991	45.5954	75.1217	24.3929
Prevalence	Global	Both	65-69 yea	Chlamydia	Number	1992	59882.5	98586.3	32020.2
Prevalence	Global	Both	65-69 yea	Chlamydia	Rate	1992	45.6745	75.1952	24.4229
Prevalence	Global	Both	65-69 yea	Chlamydia	Number	1993	61633.9	101437	32999.9
Prevalence	Global	Both	65-69 yea	Chlamydia	Rate	1993	45.675	75.1717	24.4552
Prevalence	Global	Both	65-69 yea	Chlamydia	Number	1994	63406.7	104351	33936.2
Prevalence	Global	Both	65-69 yea	Chlamydia	Rate	1994	45.7005	75.2114	24.4595
Prevalence	Global	Both	65-69 yea	Chlamydia	Number	1995	64894.7	106804	34717.2
Prevalence	Global	Both	65-69 yea	Chlamydia	Rate	1995	45.6796	75.18	24.4375
Prevalence	Global	Both	65-69 yea	Chlamydia	Number	1996	66223.4	109037	35416.9
Prevalence	Global	Both	65-69 yea	Chlamydia	Rate	1996	45.6447	75.1537	24.4112
Prevalence	Global	Both	65-69 yea	Chlamydia	Number	1997	67157.6	110638	35899
Prevalence	Global	Both	65-69 yea	Chlamydia	Rate	1997	45.5965	75.1172	24.3735
Prevalence	Global	Both	65-69 yea	Chlamydia	Number	1998	68067	112235	36374.5
Prevalence	Global	Both	65-69 yea	Chlamydia	Rate	1998	45.5988	75.1872	24.3677
Prevalence	Global	Both	65-69 yea	Chlamydia	Number	1999	68805.9	113581	36760.1
Prevalence	Global	Both	65-69 yea	Chlamydia	Rate	1999	45.5501	75.1915	24.3355
Prevalence	Global	Both	65-69 yea	Chlamydia	Number	2000	69751.1	115242	37261.7
Prevalence	Global	Both	65-69 yea	Chlamydia	Rate	2000	45.5359	75.2341	24.3257
Prevalence	Global	Both	65-69 yea	Chlamydia	Number	2001	71048.6	117163	37938.7
Prevalence	Global	Both	65-69 yea	Chlamydia	Rate	2001	45.5702	75.1477	24.3337
Prevalence	Global	Both	65-69 yea	Chlamydia	Number	2002	72708.5	119638	38847.3
Prevalence	Global	Both	65-69 yea	Chlamydia	Rate	2002	45.6367	75.0929	24.3831
Prevalence	Global	Both	65-69 yea	Chlamydia	Number	2003	74265.4	121941	39697.5
Prevalence	Global	Both	65-69 yea	Chlamydia	Rate	2003	45.6546	74.9633	24.404
Prevalence	Global	Both	65-69 yea	Chlamydia	Number	2004	75639.8	124007	40475.1
Prevalence	Global	Both	65-69 yea	Chlamydia	Rate	2004	45.6515	74.8427	24.4283
Prevalence	Global	Both	65-69 yea	Chlamydia	Number	2005	76963.1	125983	41226.7
Prevalence	Global	Both	65-69 yea	Chlamydia	Rate	2005	45.6519	74.7291	24.4543
Prevalence	Global	Both	65-69 yea	Chlamydia	Number	2006	77911.3	127473	41670
Prevalence	Global	Both	65-69 yea	Chlamydia	Rate	2006	45.5674	74.5542	24.3712
Prevalence	Global	Both	65-69 yea	Chlamydia	Number	2007	78539.9	128439	41945.7
Prevalence	Global	Both	65-69 yea	Chlamydia	Rate	2007	45.4627	74.3468	24.2802
Prevalence	Global	Both	65-69 yea	Chlamydia	Number	2008	78893.1	128994	42082.7
Prevalence	Global	Both	65-69 yea	Chlamydia	Rate	2008	45.2447	73.9776	24.1342
Prevalence	Global	Both	65-69 yea	Chlamydia	Number	2009	79432.7	129862	42333.1
Prevalence	Global	Both	65-69 yea	Chlamydia	Rate	2009	45.0444	73.6415	24.0061
Prevalence	Global	Both	65-69 yea	Chlamydia	Number	2010	80148.4	131004	42734.3
Prevalence	Global	Both	65-69 yea	Chlamydia	Rate	2010	44.9413	73.4574	23.9622
Prevalence	Global	Both	65-69 yea	Chlamydia	Number	2011	82062	134016	43765.7
Prevalence	Global	Both	65-69 yea	Chlamydia	Rate	2011	45.0342	73.5456	24.0178
Prevalence	Global	Both	65-69 yea	Chlamydia	Number	2012	85658.8	139836	45666.3
Prevalence	Global	Both	65-69 yea	Chlamydia	Rate	2012	44.9323	73.3507	23.9543
Prevalence	Global	Both	65-69 yea	Chlamydia	Number	2013	89913.8	147136	47911.6
Prevalence	Global	Both	65-69 yea	Chlamydia	Rate	2013	44.9898	73.622	23.9733
Prevalence	Global	Both	65-69 yea	Chlamydia	Number	2014	95431.5	156311	50865.5
Prevalence	Global	Both	65-69 yea	Chlamydia	Rate	2014	45.1779	73.9988	24.0801
Prevalence	Global	Both	65-69 yea	Chlamydia	Number	2015	99923.2	164014	53231.8
Prevalence	Global	Both	65-69 yea	Chlamydia	Rate	2015	45.3084	74.3693	24.137
Prevalence	Global	Both	65-69 yea	Chlamydia	Number	2016	105730	172973	56503.1
Prevalence	Global	Both	65-69 yea	Chlamydia	Rate	2016	45.4747	74.396	24.3021
Prevalence	Global	Both	65-69 yea	Chlamydia	Number	2017	110481	180689	59178.6
Prevalence	Global	Both	65-69 yea	Chlamydia	Rate	2017	45.8183	74.9343	24.5423
Prevalence	Global	Both	65-69 yea	Chlamydia	Number	2018	115571	189522	61654.8
Prevalence	Global	Both	65-69 yea	Chlamydia	Rate	2018	46.1743	75.7196	24.6329
Prevalence	Global	Both	65-69 yea	Chlamydia	Number	2019	120193	197500	64164.8
Prevalence	Global	Both	65-69 yea	Chlamydia	Rate	2019	46.4811	76.3777	24.814
Prevalence	Global	Both	50-69 yea	Chlamydia	Number	1990	4450810	6841918	2754606
Prevalence	Global	Both	50-69 yea	Chlamydia	Rate	1990	652.479	1003.01	403.819
Prevalence	Global	Both	50-69 yea	Chlamydia	Number	1991	4492346	6890559	2786339
Prevalence	Global	Both	50-69 yea	Chlamydia	Rate	1991	647.211	992.721	401.427
Prevalence	Global	Both	50-69 yea	Chlamydia	Number	1992	4524038	6940796	2810939
Prevalence	Global	Both	50-69 yea	Chlamydia	Rate	1992	641.766	984.6	398.751
Prevalence	Global	Both	50-69 yea	Chlamydia	Number	1993	4537756	6967375	2826242
Prevalence	Global	Both	50-69 yea	Chlamydia	Rate	1993	634.861	974.78	395.409
Prevalence	Global	Both	50-69 yea	Chlamydia	Number	1994	4552462	6986640	2840567
Prevalence	Global	Both	50-69 yea	Chlamydia	Rate	1994	628.739	964.922	392.31
Prevalence	Global	Both	50-69 yea	Chlamydia	Number	1995	4577975	7015722	2857554
Prevalence	Global	Both	50-69 yea	Chlamydia	Rate	1995	624.602	957.199	389.874
Prevalence	Global	Both	50-69 yea	Chlamydia	Number	1996	4664670	7152388	2914492
Prevalence	Global	Both	50-69 yea	Chlamydia	Rate	1996	627.19	961.677	391.869
Prevalence	Global	Both	50-69 yea	Chlamydia	Number	1997	4824947	7397207	3008850
Prevalence	Global	Both	50-69 yea	Chlamydia	Rate	1997	635.949	974.984	396.58
Prevalence	Global	Both	50-69 yea	Chlamydia	Number	1998	5019198	7711938	3115946
Prevalence	Global	Both	50-69 yea	Chlamydia	Rate	1998	648.334	996.157	402.489
Prevalence	Global	Both	50-69 yea	Chlamydia	Number	1999	5276521	8111073	3261672
Prevalence	Global	Both	50-69 yea	Chlamydia	Rate	1999	666.137	1023.99	411.771
Prevalence	Global	Both	50-69 yea	Chlamydia	Number	2000	5468837	8419227	3364472
Prevalence	Global	Both	50-69 yea	Chlamydia	Rate	2000	676.649	1041.7	416.28
Prevalence	Global	Both	50-69 yea	Chlamydia	Number	2001	5713394	8832045	3499733
Prevalence	Global	Both	50-69 yea	Chlamydia	Rate	2001	688.581	1064.44	421.79
Prevalence	Global	Both	50-69 yea	Chlamydia	Number	2002	5939997	9198700	3633885
Prevalence	Global	Both	50-69 yea	Chlamydia	Rate	2002	696.741	1078.98	426.242
Prevalence	Global	Both	50-69 yea	Chlamydia	Number	2003	6195138	9606040	3785451
Prevalence	Global	Both	50-69 yea	Chlamydia	Rate	2003	706.521	1095.52	431.71
Prevalence	Global	Both	50-69 yea	Chlamydia	Number	2004	6442294	9981261	3941872
Prevalence	Global	Both	50-69 yea	Chlamydia	Rate	2004	713.586	1105.58	436.625
Prevalence	Global	Both	50-69 yea	Chlamydia	Number	2005	6732086	1E+07	4113340
Prevalence	Global	Both	50-69 yea	Chlamydia	Rate	2005	723.942	1122.2	442.332
Prevalence	Global	Both	50-69 yea	Chlamydia	Number	2006	6893744	1.1E+07	4235655
Prevalence	Global	Both	50-69 yea	Chlamydia	Rate	2006	720.521	1113.77	442.703
Prevalence	Global	Both	50-69 yea	Chlamydia	Number	2007	6946217	1.1E+07	4296963
Prevalence	Global	Both	50-69 yea	Chlamydia	Rate	2007	706.462	1088.9	437.021
Prevalence	Global	Both	50-69 yea	Chlamydia	Number	2008	6932581	1.1E+07	4299656
Prevalence	Global	Both	50-69 yea	Chlamydia	Rate	2008	686.884	1057.33	426.012
Prevalence	Global	Both	50-69 yea	Chlamydia	Number	2009	6918405	1.1E+07	4301975
Prevalence	Global	Both	50-69 yea	Chlamydia	Rate	2009	667.861	1027.12	415.287
Prevalence	Global	Both	50-69 yea	Chlamydia	Number	2010	6996704	1.1E+07	4367710
Prevalence	Global	Both	50-69 yea	Chlamydia	Rate	2010	657.61	1008.41	410.515
Prevalence	Global	Both	50-69 yea	Chlamydia	Number	2011	7142878	1.1E+07	4458732
Prevalence	Global	Both	50-69 yea	Chlamydia	Rate	2011	652.848	1000.64	407.521
Prevalence	Global	Both	50-69 yea	Chlamydia	Number	2012	7342969	1.1E+07	4582492
Prevalence	Global	Both	50-69 yea	Chlamydia	Rate	2012	651.454	999.732	406.55
Prevalence	Global	Both	50-69 yea	Chlamydia	Number	2013	7604103	1.2E+07	4743294
Prevalence	Global	Both	50-69 yea	Chlamydia	Rate	2013	653.565	1004.01	407.681
Prevalence	Global	Both	50-69 yea	Chlamydia	Number	2014	7893173	1.2E+07	4912769
Prevalence	Global	Both	50-69 yea	Chlamydia	Rate	2014	656.749	1007.12	408.765
Prevalence	Global	Both	50-69 yea	Chlamydia	Number	2015	8184586	1.3E+07	5080925
Prevalence	Global	Both	50-69 yea	Chlamydia	Rate	2015	659.384	1012.98	409.34
Prevalence	Global	Both	50-69 yea	Chlamydia	Number	2016	8445090	1.3E+07	5252749
Prevalence	Global	Both	50-69 yea	Chlamydia	Rate	2016	660.442	1017.04	410.787
Prevalence	Global	Both	50-69 yea	Chlamydia	Number	2017	8686047	1.3E+07	5406783
Prevalence	Global	Both	50-69 yea	Chlamydia	Rate	2017	661.609	1019.09	411.83
Prevalence	Global	Both	50-69 yea	Chlamydia	Number	2018	9019784	1.4E+07	5599125
Prevalence	Global	Both	50-69 yea	Chlamydia	Rate	2018	669.716	1031.07	415.733
Prevalence	Global	Both	50-69 yea	Chlamydia	Number	2019	9471315	1.5E+07	5849641
Prevalence	Global	Both	50-69 yea	Chlamydia	Rate	2019	686.851	1059.15	424.21
Prevalence	Global	Both	50-69 yea	Trichomon	Number	1990	1.6E+07	2.3E+07	1.1E+07
Prevalence	Global	Both	50-69 yea	Trichomon	Rate	1990	2389.53	3370.97	1594.1
Prevalence	Global	Both	50-69 yea	Trichomon	Number	1991	1.7E+07	2.4E+07	1.1E+07
Prevalence	Global	Both	50-69 yea	Trichomon	Rate	1991	2403.45	3388.71	1603.36
Prevalence	Global	Both	50-69 yea	Trichomon	Number	1992	1.7E+07	2.4E+07	1.1E+07
Prevalence	Global	Both	50-69 yea	Trichomon	Rate	1992	2415.78	3406.88	1612.12
Prevalence	Global	Both	50-69 yea	Trichomon	Number	1993	1.7E+07	2.4E+07	1.2E+07
Prevalence	Global	Both	50-69 yea	Trichomon	Rate	1993	2424.82	3410.38	1618.44
Prevalence	Global	Both	50-69 yea	Trichomon	Number	1994	1.8E+07	2.5E+07	1.2E+07
Prevalence	Global	Both	50-69 yea	Trichomon	Rate	1994	2432.2	3409.67	1623.64
Prevalence	Global	Both	50-69 yea	Trichomon	Number	1995	1.8E+07	2.5E+07	1.2E+07
Prevalence	Global	Both	50-69 yea	Trichomon	Rate	1995	2437.77	3412.27	1627.23
Prevalence	Global	Both	50-69 yea	Trichomon	Number	1996	1.8E+07	2.5E+07	1.2E+07
Prevalence	Global	Both	50-69 yea	Trichomon	Rate	1996	2438.47	3412	1630.11
Prevalence	Global	Both	50-69 yea	Trichomon	Number	1997	1.8E+07	2.6E+07	1.2E+07
Prevalence	Global	Both	50-69 yea	Trichomon	Rate	1997	2432.37	3407.9	1629.69
Prevalence	Global	Both	50-69 yea	Trichomon	Number	1998	1.9E+07	2.6E+07	1.3E+07
Prevalence	Global	Both	50-69 yea	Trichomon	Rate	1998	2424.14	3401.29	1628.2
Prevalence	Global	Both	50-69 yea	Trichomon	Number	1999	1.9E+07	2.7E+07	1.3E+07
Prevalence	Global	Both	50-69 yea	Trichomon	Rate	1999	2418.64	3392.65	1626.39
Prevalence	Global	Both	50-69 yea	Trichomon	Number	2000	2E+07	2.7E+07	1.3E+07
Prevalence	Global	Both	50-69 yea	Trichomon	Rate	2000	2418.79	3389.03	1626.39
Prevalence	Global	Both	50-69 yea	Trichomon	Number	2001	2E+07	2.8E+07	1.4E+07
Prevalence	Global	Both	50-69 yea	Trichomon	Rate	2001	2426.88	3422.11	1630.85
Prevalence	Global	Both	50-69 yea	Trichomon	Number	2002	2.1E+07	2.9E+07	1.4E+07
Prevalence	Global	Both	50-69 yea	Trichomon	Rate	2002	2429.97	3429.08	1631.03
Prevalence	Global	Both	50-69 yea	Trichomon	Number	2003	2.1E+07	3E+07	1.4E+07
Prevalence	Global	Both	50-69 yea	Trichomon	Rate	2003	2432.9	3435.25	1626.86
Prevalence	Global	Both	50-69 yea	Trichomon	Number	2004	2.2E+07	3.1E+07	1.5E+07
Prevalence	Global	Both	50-69 yea	Trichomon	Rate	2004	2435.96	3444.51	1622.31
Prevalence	Global	Both	50-69 yea	Trichomon	Number	2005	2.3E+07	3.2E+07	1.5E+07
Prevalence	Global	Both	50-69 yea	Trichomon	Rate	2005	2442.83	3458.92	1622.21
Prevalence	Global	Both	50-69 yea	Trichomon	Number	2006	2.3E+07	3.3E+07	1.5E+07
Prevalence	Global	Both	50-69 yea	Trichomon	Rate	2006	2444.04	3458.62	1618.49
Prevalence	Global	Both	50-69 yea	Trichomon	Number	2007	2.4E+07	3.4E+07	1.6E+07
Prevalence	Global	Both	50-69 yea	Trichomon	Rate	2007	2438.74	3450.2	1613.38
Prevalence	Global	Both	50-69 yea	Trichomon	Number	2008	2.5E+07	3.5E+07	1.6E+07
Prevalence	Global	Both	50-69 yea	Trichomon	Rate	2008	2430.93	3433.56	1606.83
Prevalence	Global	Both	50-69 yea	Trichomon	Number	2009	2.5E+07	3.5E+07	1.7E+07
Prevalence	Global	Both	50-69 yea	Trichomon	Rate	2009	2422.93	3405.18	1602.23
Prevalence	Global	Both	50-69 yea	Trichomon	Number	2010	2.6E+07	3.6E+07	1.7E+07
Prevalence	Global	Both	50-69 yea	Trichomon	Rate	2010	2420.38	3396.58	1600.31
Prevalence	Global	Both	50-69 yea	Trichomon	Number	2011	2.6E+07	3.7E+07	1.8E+07
Prevalence	Global	Both	50-69 yea	Trichomon	Rate	2011	2421.71	3395.76	1603.25
Prevalence	Global	Both	50-69 yea	Trichomon	Number	2012	2.7E+07	3.8E+07	1.8E+07
Prevalence	Global	Both	50-69 yea	Trichomon	Rate	2012	2426.41	3405.82	1608.94
Prevalence	Global	Both	50-69 yea	Trichomon	Number	2013	2.8E+07	4E+07	1.9E+07
Prevalence	Global	Both	50-69 yea	Trichomon	Rate	2013	2433.69	3418.84	1616.95
Prevalence	Global	Both	50-69 yea	Trichomon	Number	2014	2.9E+07	4.1E+07	2E+07
Prevalence	Global	Both	50-69 yea	Trichomon	Rate	2014	2440.58	3434.6	1624.86
Prevalence	Global	Both	50-69 yea	Trichomon	Number	2015	3E+07	4.3E+07	2E+07
Prevalence	Global	Both	50-69 yea	Trichomon	Rate	2015	2445.06	3456.4	1630.42
Prevalence	Global	Both	50-69 yea	Trichomon	Number	2016	3.1E+07	4.4E+07	2.1E+07
Prevalence	Global	Both	50-69 yea	Trichomon	Rate	2016	2446.85	3442.86	1630.66
Prevalence	Global	Both	50-69 yea	Trichomon	Number	2017	3.2E+07	4.5E+07	2.1E+07
Prevalence	Global	Both	50-69 yea	Trichomon	Rate	2017	2449.98	3456.03	1624.83
Prevalence	Global	Both	50-69 yea	Trichomon	Number	2018	3.3E+07	4.7E+07	2.2E+07
Prevalence	Global	Both	50-69 yea	Trichomon	Rate	2018	2450.76	3456.63	1625.64
Prevalence	Global	Both	50-69 yea	Trichomon	Number	2019	3.4E+07	4.8E+07	2.2E+07
Prevalence	Global	Both	50-69 yea	Trichomon	Rate	2019	2451.05	3465.69	1625.33
Incidence	Global	Both	50-54 yea	HIV/AIDS	Number	1990	59098.5	72261.3	47543.6
Incidence	Global	Both	50-54 yea	HIV/AIDS	Rate	1990	27.7991	33.9907	22.3639
Incidence	Global	Both	50-54 yea	HIV/AIDS	Number	1991	66159	81711.6	52779.6
Incidence	Global	Both	50-54 yea	HIV/AIDS	Rate	1991	30.7872	38.0246	24.5611
Incidence	Global	Both	50-54 yea	HIV/AIDS	Number	1992	71299.7	89264.2	56161.2
Incidence	Global	Both	50-54 yea	HIV/AIDS	Rate	1992	32.9669	41.2731	25.9673
Incidence	Global	Both	50-54 yea	HIV/AIDS	Number	1993	75356.6	95280	59124.8
Incidence	Global	Both	50-54 yea	HIV/AIDS	Rate	1993	34.7096	43.8863	27.2331
Incidence	Global	Both	50-54 yea	HIV/AIDS	Number	1994	77871.9	98543.9	62003.4
Incidence	Global	Both	50-54 yea	HIV/AIDS	Rate	1994	35.6977	45.1741	28.4233
Incidence	Global	Both	50-54 yea	HIV/AIDS	Number	1995	79876.6	102175	63699.4
Incidence	Global	Both	50-54 yea	HIV/AIDS	Rate	1995	36.4577	46.6353	29.074
Incidence	Global	Both	50-54 yea	HIV/AIDS	Number	1996	81641.3	103786	65883.6
Incidence	Global	Both	50-54 yea	HIV/AIDS	Rate	1996	36.7165	46.6756	29.6298
Incidence	Global	Both	50-54 yea	HIV/AIDS	Number	1997	82443.4	104315	66547.8
Incidence	Global	Both	50-54 yea	HIV/AIDS	Rate	1997	35.742	45.2242	28.8507
Incidence	Global	Both	50-54 yea	HIV/AIDS	Number	1998	81342.2	102792	65858.5
Incidence	Global	Both	50-54 yea	HIV/AIDS	Rate	1998	33.8779	42.8115	27.4291
Incidence	Global	Both	50-54 yea	HIV/AIDS	Number	1999	79901.2	101136	64999
Incidence	Global	Both	50-54 yea	HIV/AIDS	Rate	1999	31.6815	40.1014	25.7726
Incidence	Global	Both	50-54 yea	HIV/AIDS	Number	2000	79217.4	100268	64601.5
Incidence	Global	Both	50-54 yea	HIV/AIDS	Rate	2000	30.2086	38.2361	24.635
Incidence	Global	Both	50-54 yea	HIV/AIDS	Number	2001	78740.5	99235.2	64586.6
Incidence	Global	Both	50-54 yea	HIV/AIDS	Rate	2001	28.595	36.0377	23.4549
Incidence	Global	Both	50-54 yea	HIV/AIDS	Number	2002	78711.7	97924.9	64711.8
Incidence	Global	Both	50-54 yea	HIV/AIDS	Rate	2002	27.6317	34.3765	22.7171
Incidence	Global	Both	50-54 yea	HIV/AIDS	Number	2003	78784.8	97012	64543.2
Incidence	Global	Both	50-54 yea	HIV/AIDS	Rate	2003	26.6987	32.8756	21.8725
Incidence	Global	Both	50-54 yea	HIV/AIDS	Number	2004	78354.2	96051.9	64414.5
Incidence	Global	Both	50-54 yea	HIV/AIDS	Rate	2004	25.7488	31.5647	21.1679
Incidence	Global	Both	50-54 yea	HIV/AIDS	Number	2005	77596.9	94781.8	63602.3
Incidence	Global	Both	50-54 yea	HIV/AIDS	Rate	2005	24.4987	29.9243	20.0804
Incidence	Global	Both	50-54 yea	HIV/AIDS	Number	2006	76596.9	93480.6	62349.4
Incidence	Global	Both	50-54 yea	HIV/AIDS	Rate	2006	23.614	28.8191	19.2217
Incidence	Global	Both	50-54 yea	HIV/AIDS	Number	2007	75337.4	92252.8	61113.7
Incidence	Global	Both	50-54 yea	HIV/AIDS	Rate	2007	22.7609	27.8714	18.4636
Incidence	Global	Both	50-54 yea	HIV/AIDS	Number	2008	74235.7	90885.6	60372.8
Incidence	Global	Both	50-54 yea	HIV/AIDS	Rate	2008	22.1104	27.0694	17.9815
Incidence	Global	Both	50-54 yea	HIV/AIDS	Number	2009	73675.2	90485	60013.4
Incidence	Global	Both	50-54 yea	HIV/AIDS	Rate	2009	21.7101	26.6635	17.6843
Incidence	Global	Both	50-54 yea	HIV/AIDS	Number	2010	73463.6	90865.7	59761.5
Incidence	Global	Both	50-54 yea	HIV/AIDS	Rate	2010	21.3994	26.4685	17.4081
Incidence	Global	Both	50-54 yea	HIV/AIDS	Number	2011	73262.6	90994.6	59076.6
Incidence	Global	Both	50-54 yea	HIV/AIDS	Rate	2011	20.9728	26.0489	16.9118
Incidence	Global	Both	50-54 yea	HIV/AIDS	Number	2012	73115.9	90961.1	58441.1
Incidence	Global	Both	50-54 yea	HIV/AIDS	Rate	2012	20.4096	25.3909	16.3132
Incidence	Global	Both	50-54 yea	HIV/AIDS	Number	2013	72632.7	91100.9	57263.8
Incidence	Global	Both	50-54 yea	HIV/AIDS	Rate	2013	19.5891	24.57	15.4441
Incidence	Global	Both	50-54 yea	HIV/AIDS	Number	2014	71865.3	90310.1	56280.7
Incidence	Global	Both	50-54 yea	HIV/AIDS	Rate	2014	18.6553	23.4433	14.6097
Incidence	Global	Both	50-54 yea	HIV/AIDS	Number	2015	70385.3	88580.1	54678.5
Incidence	Global	Both	50-54 yea	HIV/AIDS	Rate	2015	17.6154	22.169	13.6844
Incidence	Global	Both	50-54 yea	HIV/AIDS	Number	2016	68172.7	85910.1	52996.8
Incidence	Global	Both	50-54 yea	HIV/AIDS	Rate	2016	16.5495	20.8553	12.8654
Incidence	Global	Both	50-54 yea	HIV/AIDS	Number	2017	65575.1	83054.9	50792
Incidence	Global	Both	50-54 yea	HIV/AIDS	Rate	2017	15.5189	19.6557	12.0204
Incidence	Global	Both	50-54 yea	HIV/AIDS	Number	2018	61822.2	78070.1	48030
Incidence	Global	Both	50-54 yea	HIV/AIDS	Rate	2018	14.3582	18.1317	11.1549
Incidence	Global	Both	50-54 yea	HIV/AIDS	Number	2019	60316.2	76171.7	46528.1
Incidence	Global	Both	50-54 yea	HIV/AIDS	Rate	2019	13.8081	17.4379	10.6516
Incidence	Global	Both	55-59 yea	HIV/AIDS	Number	1990	36674.5	44950	29395.7
Incidence	Global	Both	55-59 yea	HIV/AIDS	Rate	1990	19.7797	24.2428	15.854
Incidence	Global	Both	55-59 yea	HIV/AIDS	Number	1991	41049.6	51089.6	32501.5
Incidence	Global	Both	55-59 yea	HIV/AIDS	Rate	1991	21.8399	27.1816	17.292
Incidence	Global	Both	55-59 yea	HIV/AIDS	Number	1992	44302.6	56018.4	34628.7
Incidence	Global	Both	55-59 yea	HIV/AIDS	Rate	1992	23.1696	29.2967	18.1103
Incidence	Global	Both	55-59 yea	HIV/AIDS	Number	1993	46755.6	60312.1	36503.8
Incidence	Global	Both	55-59 yea	HIV/AIDS	Rate	1993	24.057	31.0321	18.7822
Incidence	Global	Both	55-59 yea	HIV/AIDS	Number	1994	48346.6	61632	37984
Incidence	Global	Both	55-59 yea	HIV/AIDS	Rate	1994	24.524	31.263	19.2675
Incidence	Global	Both	55-59 yea	HIV/AIDS	Number	1995	49639.6	64069.3	39278.8
Incidence	Global	Both	55-59 yea	HIV/AIDS	Rate	1995	24.8464	32.0689	19.6604
Incidence	Global	Both	55-59 yea	HIV/AIDS	Number	1996	50723.1	64987.2	40358
Incidence	Global	Both	55-59 yea	HIV/AIDS	Rate	1996	25.1445	32.2156	20.0063
Incidence	Global	Both	55-59 yea	HIV/AIDS	Number	1997	51177.9	65205.5	40886.7
Incidence	Global	Both	55-59 yea	HIV/AIDS	Rate	1997	25.2286	32.1437	20.1555
Incidence	Global	Both	55-59 yea	HIV/AIDS	Number	1998	50349.1	64147.9	40255.6
Incidence	Global	Both	55-59 yea	HIV/AIDS	Rate	1998	24.7272	31.504	19.7702
Incidence	Global	Both	55-59 yea	HIV/AIDS	Number	1999	49431.5	62833.4	39909.7
Incidence	Global	Both	55-59 yea	HIV/AIDS	Rate	1999	24.1465	30.6931	19.4953
Incidence	Global	Both	55-59 yea	HIV/AIDS	Number	2000	48764.1	61629.4	39790.9
Incidence	Global	Both	55-59 yea	HIV/AIDS	Rate	2000	23.6887	29.9384	19.3297
Incidence	Global	Both	55-59 yea	HIV/AIDS	Number	2001	47950.3	59980.7	39427.8
Incidence	Global	Both	55-59 yea	HIV/AIDS	Rate	2001	22.9068	28.6539	18.8354
Incidence	Global	Both	55-59 yea	HIV/AIDS	Number	2002	48174.8	59455.5	39701.2
Incidence	Global	Both	55-59 yea	HIV/AIDS	Rate	2002	22.1182	27.2975	18.2278
Incidence	Global	Both	55-59 yea	HIV/AIDS	Number	2003	48827	59634	39973.2
Incidence	Global	Both	55-59 yea	HIV/AIDS	Rate	2003	21.4745	26.2275	17.5805
Incidence	Global	Both	55-59 yea	HIV/AIDS	Number	2004	49268.1	59539.4	40240.9
Incidence	Global	Both	55-59 yea	HIV/AIDS	Rate	2004	20.5708	24.8594	16.8017
Incidence	Global	Both	55-59 yea	HIV/AIDS	Number	2005	50009.2	60424.6	40722.6
Incidence	Global	Both	55-59 yea	HIV/AIDS	Rate	2005	20.037	24.2101	16.3162
Incidence	Global	Both	55-59 yea	HIV/AIDS	Number	2006	51095.9	61752.3	41422.1
Incidence	Global	Both	55-59 yea	HIV/AIDS	Rate	2006	19.4613	23.5201	15.7767
Incidence	Global	Both	55-59 yea	HIV/AIDS	Number	2007	51065	62024.1	41190.9
Incidence	Global	Both	55-59 yea	HIV/AIDS	Rate	2007	18.7787	22.8089	15.1476
Incidence	Global	Both	55-59 yea	HIV/AIDS	Number	2008	50914.3	62230.8	41718.3
Incidence	Global	Both	55-59 yea	HIV/AIDS	Rate	2008	18.0544	22.0673	14.7935
Incidence	Global	Both	55-59 yea	HIV/AIDS	Number	2009	51184.3	63132.9	42183
Incidence	Global	Both	55-59 yea	HIV/AIDS	Rate	2009	17.5821	21.6865	14.4901
Incidence	Global	Both	55-59 yea	HIV/AIDS	Number	2010	51235.4	63834.7	41902.7
Incidence	Global	Both	55-59 yea	HIV/AIDS	Rate	2010	16.887	21.0397	13.811
Incidence	Global	Both	55-59 yea	HIV/AIDS	Number	2011	51079.2	64257.3	41060.5
Incidence	Global	Both	55-59 yea	HIV/AIDS	Rate	2011	16.4245	20.6619	13.203
Incidence	Global	Both	55-59 yea	HIV/AIDS	Number	2012	51104.4	65015.2	40201.3
Incidence	Global	Both	55-59 yea	HIV/AIDS	Rate	2012	16.0909	20.4709	12.6579
Incidence	Global	Both	55-59 yea	HIV/AIDS	Number	2013	51445.9	66210.9	39306.9
Incidence	Global	Both	55-59 yea	HIV/AIDS	Rate	2013	15.9564	20.5358	12.1914
Incidence	Global	Both	55-59 yea	HIV/AIDS	Number	2014	52216.8	67772.4	38469
Incidence	Global	Both	55-59 yea	HIV/AIDS	Rate	2014	16.0105	20.7801	11.7952
Incidence	Global	Both	55-59 yea	HIV/AIDS	Number	2015	53277.4	70026	38371.9
Incidence	Global	Both	55-59 yea	HIV/AIDS	Rate	2015	16.1393	21.213	11.624
Incidence	Global	Both	55-59 yea	HIV/AIDS	Number	2016	53772.1	70981.3	38446.7
Incidence	Global	Both	55-59 yea	HIV/AIDS	Rate	2016	16.0033	21.125	11.4423
Incidence	Global	Both	55-59 yea	HIV/AIDS	Number	2017	53533.3	70909.6	37652.1
Incidence	Global	Both	55-59 yea	HIV/AIDS	Rate	2017	15.5317	20.5732	10.9241
Incidence	Global	Both	55-59 yea	HIV/AIDS	Number	2018	52540.6	70082	36658.2
Incidence	Global	Both	55-59 yea	HIV/AIDS	Rate	2018	14.7218	19.6369	10.2716
Incidence	Global	Both	55-59 yea	HIV/AIDS	Number	2019	51870	69633.8	36046.9
Incidence	Global	Both	55-59 yea	HIV/AIDS	Rate	2019	13.9807	18.7686	9.71582
Incidence	Global	Both	60-64 yea	HIV/AIDS	Number	1990	23139.6	28839.2	18044.7
Incidence	Global	Both	60-64 yea	HIV/AIDS	Rate	1990	14.4043	17.9523	11.2328
Incidence	Global	Both	60-64 yea	HIV/AIDS	Number	1991	26044	32653	20056.2
Incidence	Global	Both	60-64 yea	HIV/AIDS	Rate	1991	15.8817	19.9119	12.2303
Incidence	Global	Both	60-64 yea	HIV/AIDS	Number	1992	28392.6	36362	21753.9
Incidence	Global	Both	60-64 yea	HIV/AIDS	Rate	1992	17.0688	21.8598	13.0778
Incidence	Global	Both	60-64 yea	HIV/AIDS	Number	1993	30236.9	39048.5	23384.3
Incidence	Global	Both	60-64 yea	HIV/AIDS	Rate	1993	17.9592	23.1929	13.8891
Incidence	Global	Both	60-64 yea	HIV/AIDS	Number	1994	31469.4	40225.7	24694.9
Incidence	Global	Both	60-64 yea	HIV/AIDS	Rate	1994	18.5075	23.6572	14.5234
Incidence	Global	Both	60-64 yea	HIV/AIDS	Number	1995	32389	41685	25551
Incidence	Global	Both	60-64 yea	HIV/AIDS	Rate	1995	18.831	24.2357	14.8554
Incidence	Global	Both	60-64 yea	HIV/AIDS	Number	1996	32966.2	42497.6	26132.4
Incidence	Global	Both	60-64 yea	HIV/AIDS	Rate	1996	18.8837	24.3436	14.9692
Incidence	Global	Both	60-64 yea	HIV/AIDS	Number	1997	33427.8	42861.8	26781.5
Incidence	Global	Both	60-64 yea	HIV/AIDS	Rate	1997	18.7909	24.094	15.0547
Incidence	Global	Both	60-64 yea	HIV/AIDS	Number	1998	33113.1	42271.4	26619.5
Incidence	Global	Both	60-64 yea	HIV/AIDS	Rate	1998	18.2771	23.3321	14.6929
Incidence	Global	Both	60-64 yea	HIV/AIDS	Number	1999	32791.5	41595.6	26396.2
Incidence	Global	Both	60-64 yea	HIV/AIDS	Rate	1999	17.8083	22.5897	14.3352
Incidence	Global	Both	60-64 yea	HIV/AIDS	Number	2000	32548.2	41083.9	26522.1
Incidence	Global	Both	60-64 yea	HIV/AIDS	Rate	2000	17.4095	21.9751	14.1862
Incidence	Global	Both	60-64 yea	HIV/AIDS	Number	2001	31893.5	39893.7	26224.8
Incidence	Global	Both	60-64 yea	HIV/AIDS	Rate	2001	16.8632	21.0932	13.866
Incidence	Global	Both	60-64 yea	HIV/AIDS	Number	2002	31644.7	38995.9	26073.3
Incidence	Global	Both	60-64 yea	HIV/AIDS	Rate	2002	16.6067	20.4645	13.6829
Incidence	Global	Both	60-64 yea	HIV/AIDS	Number	2003	31610.3	38576.4	26059.6
Incidence	Global	Both	60-64 yea	HIV/AIDS	Rate	2003	16.4874	20.1208	13.5923
Incidence	Global	Both	60-64 yea	HIV/AIDS	Number	2004	31559.3	38183.4	26172.6
Incidence	Global	Both	60-64 yea	HIV/AIDS	Rate	2004	16.3259	19.7525	13.5393
Incidence	Global	Both	60-64 yea	HIV/AIDS	Number	2005	31730.5	38451.3	26340.9
Incidence	Global	Both	60-64 yea	HIV/AIDS	Rate	2005	16.2711	19.7175	13.5074
Incidence	Global	Both	60-64 yea	HIV/AIDS	Number	2006	32303.9	39335.9	26656.3
Incidence	Global	Both	60-64 yea	HIV/AIDS	Rate	2006	16.2438	19.7797	13.4039
Incidence	Global	Both	60-64 yea	HIV/AIDS	Number	2007	32932.7	40669.8	26991.5
Incidence	Global	Both	60-64 yea	HIV/AIDS	Rate	2007	15.8667	19.5944	13.0043
Incidence	Global	Both	60-64 yea	HIV/AIDS	Number	2008	33559.5	41920.6	27655.2
Incidence	Global	Both	60-64 yea	HIV/AIDS	Rate	2008	15.4541	19.3044	12.7352
Incidence	Global	Both	60-64 yea	HIV/AIDS	Number	2009	34481.1	43508.4	28332.6
Incidence	Global	Both	60-64 yea	HIV/AIDS	Rate	2009	15.0516	18.9921	12.3677
Incidence	Global	Both	60-64 yea	HIV/AIDS	Number	2010	35425.1	44818.3	29001
Incidence	Global	Both	60-64 yea	HIV/AIDS	Rate	2010	14.8271	18.7587	12.1384
Incidence	Global	Both	60-64 yea	HIV/AIDS	Number	2011	36475	46546.9	29467.7
Incidence	Global	Both	60-64 yea	HIV/AIDS	Rate	2011	14.4988	18.5024	11.7134
Incidence	Global	Both	60-64 yea	HIV/AIDS	Number	2012	36894.7	47474.4	29388.5
Incidence	Global	Both	60-64 yea	HIV/AIDS	Rate	2012	14.153	18.2114	11.2735
Incidence	Global	Both	60-64 yea	HIV/AIDS	Number	2013	37429.1	48080.3	29233.9
Incidence	Global	Both	60-64 yea	HIV/AIDS	Rate	2013	13.8406	17.7792	10.8102
Incidence	Global	Both	60-64 yea	HIV/AIDS	Number	2014	38152.6	48880.5	29057.7
Incidence	Global	Both	60-64 yea	HIV/AIDS	Rate	2014	13.6624	17.5041	10.4055
Incidence	Global	Both	60-64 yea	HIV/AIDS	Number	2015	38689.5	49885.3	28721
Incidence	Global	Both	60-64 yea	HIV/AIDS	Rate	2015	13.2939	17.1408	9.86866
Incidence	Global	Both	60-64 yea	HIV/AIDS	Number	2016	38863.7	49901.8	28675.8
Incidence	Global	Both	60-64 yea	HIV/AIDS	Rate	2016	13.0301	16.731	9.61436
Incidence	Global	Both	60-64 yea	HIV/AIDS	Number	2017	38795.6	50067.3	28386.7
Incidence	Global	Both	60-64 yea	HIV/AIDS	Rate	2017	12.74	16.4415	9.32183
Incidence	Global	Both	60-64 yea	HIV/AIDS	Number	2018	38224	49379.3	27739.5
Incidence	Global	Both	60-64 yea	HIV/AIDS	Rate	2018	12.3681	15.9776	8.97566
Incidence	Global	Both	60-64 yea	HIV/AIDS	Number	2019	38022.8	49432	27401.4
Incidence	Global	Both	60-64 yea	HIV/AIDS	Rate	2019	12.1659	15.8164	8.76746
Incidence	Global	Both	65-69 yea	HIV/AIDS	Number	1990	15412	19749.9	11613.5
Incidence	Global	Both	65-69 yea	HIV/AIDS	Rate	1990	12.4805	15.9933	9.40448
Incidence	Global	Both	65-69 yea	HIV/AIDS	Number	1991	17729.5	22604.8	13351.3
Incidence	Global	Both	65-69 yea	HIV/AIDS	Rate	1991	13.9303	17.7609	10.4903
Incidence	Global	Both	65-69 yea	HIV/AIDS	Number	1992	19672.7	25542.3	14884.6
Incidence	Global	Both	65-69 yea	HIV/AIDS	Rate	1992	15.0051	19.482	11.353
Incidence	Global	Both	65-69 yea	HIV/AIDS	Number	1993	21264.3	27828.1	16259.8
Incidence	Global	Both	65-69 yea	HIV/AIDS	Rate	1993	15.7583	20.6225	12.0496
Incidence	Global	Both	65-69 yea	HIV/AIDS	Number	1994	22380.2	29069.9	17419.5
Incidence	Global	Both	65-69 yea	HIV/AIDS	Rate	1994	16.1305	20.9522	12.5551
Incidence	Global	Both	65-69 yea	HIV/AIDS	Number	1995	23113.1	30068.4	18263.6
Incidence	Global	Both	65-69 yea	HIV/AIDS	Rate	1995	16.2694	21.1652	12.8558
Incidence	Global	Both	65-69 yea	HIV/AIDS	Number	1996	23404.9	30515.2	18651.5
Incidence	Global	Both	65-69 yea	HIV/AIDS	Rate	1996	16.1319	21.0327	12.8556
Incidence	Global	Both	65-69 yea	HIV/AIDS	Number	1997	23606.3	30715.9	19031.4
Incidence	Global	Both	65-69 yea	HIV/AIDS	Rate	1997	16.0274	20.8545	12.9213
Incidence	Global	Both	65-69 yea	HIV/AIDS	Number	1998	23245.7	29904	18897.4
Incidence	Global	Both	65-69 yea	HIV/AIDS	Rate	1998	15.5726	20.033	12.6595
Incidence	Global	Both	65-69 yea	HIV/AIDS	Number	1999	22824.5	29252.4	18797.3
Incidence	Global	Both	65-69 yea	HIV/AIDS	Rate	1999	15.11	19.3654	12.4439
Incidence	Global	Both	65-69 yea	HIV/AIDS	Number	2000	22442.1	28364.2	18863.7
Incidence	Global	Both	65-69 yea	HIV/AIDS	Rate	2000	14.6509	18.5171	12.3149
Incidence	Global	Both	65-69 yea	HIV/AIDS	Number	2001	21919.8	27222.3	18692.8
Incidence	Global	Both	65-69 yea	HIV/AIDS	Rate	2001	14.0593	17.4603	11.9895
Incidence	Global	Both	65-69 yea	HIV/AIDS	Number	2002	21523.6	26241.9	18519.1
Incidence	Global	Both	65-69 yea	HIV/AIDS	Rate	2002	13.5096	16.4712	11.6238
Incidence	Global	Both	65-69 yea	HIV/AIDS	Number	2003	21210.1	25498.7	18388.8
Incidence	Global	Both	65-69 yea	HIV/AIDS	Rate	2003	13.0389	15.6753	11.3045
Incidence	Global	Both	65-69 yea	HIV/AIDS	Number	2004	20954.7	25055.9	18267.7
Incidence	Global	Both	65-69 yea	HIV/AIDS	Rate	2004	12.6469	15.1222	11.0253
Incidence	Global	Both	65-69 yea	HIV/AIDS	Number	2005	20663.9	24637.7	18023.3
Incidence	Global	Both	65-69 yea	HIV/AIDS	Rate	2005	12.2571	14.6142	10.6908
Incidence	Global	Both	65-69 yea	HIV/AIDS	Number	2006	20359.7	24236.7	17740.8
Incidence	Global	Both	65-69 yea	HIV/AIDS	Rate	2006	11.9076	14.1751	10.3759
Incidence	Global	Both	65-69 yea	HIV/AIDS	Number	2007	20028.2	24102.6	17403.5
Incidence	Global	Both	65-69 yea	HIV/AIDS	Rate	2007	11.5933	13.9518	10.074
Incidence	Global	Both	65-69 yea	HIV/AIDS	Number	2008	19695.1	23756.7	17022.9
Incidence	Global	Both	65-69 yea	HIV/AIDS	Rate	2008	11.295	13.6243	9.76254
Incidence	Global	Both	65-69 yea	HIV/AIDS	Number	2009	19390	23601.8	16643.1
Incidence	Global	Both	65-69 yea	HIV/AIDS	Rate	2009	10.9956	13.384	9.43789
Incidence	Global	Both	65-69 yea	HIV/AIDS	Number	2010	19205.9	23715.9	16355.1
Incidence	Global	Both	65-69 yea	HIV/AIDS	Rate	2010	10.7692	13.2981	9.17073
Incidence	Global	Both	65-69 yea	HIV/AIDS	Number	2011	19225	24101.1	16133.6
Incidence	Global	Both	65-69 yea	HIV/AIDS	Rate	2011	10.5503	13.2263	8.85383
Incidence	Global	Both	65-69 yea	HIV/AIDS	Number	2012	19468	24538.9	16100.4
Incidence	Global	Both	65-69 yea	HIV/AIDS	Rate	2012	10.2119	12.8719	8.44545
Incidence	Global	Both	65-69 yea	HIV/AIDS	Number	2013	20046.1	25779.7	16177.3
Incidence	Global	Both	65-69 yea	HIV/AIDS	Rate	2013	10.0304	12.8993	8.09458
Incidence	Global	Both	65-69 yea	HIV/AIDS	Number	2014	20543	26278	16454.2
Incidence	Global	Both	65-69 yea	HIV/AIDS	Rate	2014	9.7252	12.4402	7.78952
Incidence	Global	Both	65-69 yea	HIV/AIDS	Number	2015	21864.4	27877.5	17296.9
Incidence	Global	Both	65-69 yea	HIV/AIDS	Rate	2015	9.91402	12.6405	7.84296
Incidence	Global	Both	65-69 yea	HIV/AIDS	Number	2016	23127.7	29104.1	18239.4
Incidence	Global	Both	65-69 yea	HIV/AIDS	Rate	2016	9.94726	12.5177	7.8448
Incidence	Global	Both	65-69 yea	HIV/AIDS	Number	2017	23866.8	30050.6	18772.3
Incidence	Global	Both	65-69 yea	HIV/AIDS	Rate	2017	9.89792	12.4625	7.78518
Incidence	Global	Both	65-69 yea	HIV/AIDS	Number	2018	24412.1	30789.5	19100.6
Incidence	Global	Both	65-69 yea	HIV/AIDS	Rate	2018	9.75339	12.3013	7.63125
Incidence	Global	Both	65-69 yea	HIV/AIDS	Number	2019	24302.3	30719.2	18935.4
Incidence	Global	Both	65-69 yea	HIV/AIDS	Rate	2019	9.39823	11.8798	7.32272
Incidence	Global	Both	50-69 yea	HIV/AIDS	Number	1990	134325	166171	106555
Incidence	Global	Both	50-69 yea	HIV/AIDS	Rate	1990	19.6917	24.3603	15.6207
Incidence	Global	Both	50-69 yea	HIV/AIDS	Number	1991	150982	187595	118748
Incidence	Global	Both	50-69 yea	HIV/AIDS	Rate	1991	21.7519	27.0267	17.108
Incidence	Global	Both	50-69 yea	HIV/AIDS	Number	1992	163668	206554	127590
Incidence	Global	Both	50-69 yea	HIV/AIDS	Rate	1992	23.2174	29.3011	18.0995
Incidence	Global	Both	50-69 yea	HIV/AIDS	Number	1993	173613	222099	135420
Incidence	Global	Both	50-69 yea	HIV/AIDS	Rate	1993	24.2896	31.073	18.9461
Incidence	Global	Both	50-69 yea	HIV/AIDS	Number	1994	180068	229226	142130
Incidence	Global	Both	50-69 yea	HIV/AIDS	Rate	1994	24.8691	31.6583	19.6295
Incidence	Global	Both	50-69 yea	HIV/AIDS	Number	1995	185018	237469	146878
Incidence	Global	Both	50-69 yea	HIV/AIDS	Rate	1995	25.2432	32.3993	20.0395
Incidence	Global	Both	50-69 yea	HIV/AIDS	Number	1996	188735	241590	150919
Incidence	Global	Both	50-69 yea	HIV/AIDS	Rate	1996	25.3765	32.4831	20.2918
Incidence	Global	Both	50-69 yea	HIV/AIDS	Number	1997	190655	242832	153466
Incidence	Global	Both	50-69 yea	HIV/AIDS	Rate	1997	25.1292	32.0064	20.2275
Incidence	Global	Both	50-69 yea	HIV/AIDS	Number	1998	188050	238478	151701
Incidence	Global	Both	50-69 yea	HIV/AIDS	Rate	1998	24.2906	30.8044	19.5953
Incidence	Global	Both	50-69 yea	HIV/AIDS	Number	1999	184949	234062	150042
Incidence	Global	Both	50-69 yea	HIV/AIDS	Rate	1999	23.3489	29.5492	18.9421
Incidence	Global	Both	50-69 yea	HIV/AIDS	Number	2000	182972	231599	149729
Incidence	Global	Both	50-69 yea	HIV/AIDS	Rate	2000	22.6388	28.6553	18.5257
Incidence	Global	Both	50-69 yea	HIV/AIDS	Number	2001	180504	226349	148641
Incidence	Global	Both	50-69 yea	HIV/AIDS	Rate	2001	21.7545	27.2797	17.9143
Incidence	Global	Both	50-69 yea	HIV/AIDS	Number	2002	180055	223541	149637
Incidence	Global	Both	50-69 yea	HIV/AIDS	Rate	2002	21.1198	26.2206	17.5519
Incidence	Global	Both	50-69 yea	HIV/AIDS	Number	2003	180432	221345	149944
Incidence	Global	Both	50-69 yea	HIV/AIDS	Rate	2003	20.5773	25.2432	17.1002
Incidence	Global	Both	50-69 yea	HIV/AIDS	Number	2004	180136	218959	149875
Incidence	Global	Both	50-69 yea	HIV/AIDS	Rate	2004	19.953	24.2531	16.6011
Incidence	Global	Both	50-69 yea	HIV/AIDS	Number	2005	180000	217938	149572
Incidence	Global	Both	50-69 yea	HIV/AIDS	Rate	2005	19.3565	23.4361	16.0843
Incidence	Global	Both	50-69 yea	HIV/AIDS	Number	2006	180356	217528	148756
Incidence	Global	Both	50-69 yea	HIV/AIDS	Rate	2006	18.8505	22.7356	15.5477
Incidence	Global	Both	50-69 yea	HIV/AIDS	Number	2007	179363	218205	147808
Incidence	Global	Both	50-69 yea	HIV/AIDS	Rate	2007	18.2421	22.1925	15.0328
Incidence	Global	Both	50-69 yea	HIV/AIDS	Number	2008	178405	217815	147859
Incidence	Global	Both	50-69 yea	HIV/AIDS	Rate	2008	17.6764	21.5812	14.65
Incidence	Global	Both	50-69 yea	HIV/AIDS	Number	2009	178731	219804	148002
Incidence	Global	Both	50-69 yea	HIV/AIDS	Rate	2009	17.2536	21.2185	14.2872
Incidence	Global	Both	50-69 yea	HIV/AIDS	Number	2010	179330	222766	147637
Incidence	Global	Both	50-69 yea	HIV/AIDS	Rate	2010	16.855	20.9375	13.8762
Incidence	Global	Both	50-69 yea	HIV/AIDS	Number	2011	180042	224719	146239
Incidence	Global	Both	50-69 yea	HIV/AIDS	Rate	2011	16.4555	20.5389	13.366
Incidence	Global	Both	50-69 yea	HIV/AIDS	Number	2012	180583	226604	144908
Incidence	Global	Both	50-69 yea	HIV/AIDS	Rate	2012	16.021	20.1039	12.856
Incidence	Global	Both	50-69 yea	HIV/AIDS	Number	2013	181554	229207	142345
Incidence	Global	Both	50-69 yea	HIV/AIDS	Rate	2013	15.6044	19.7001	12.2344
Incidence	Global	Both	50-69 yea	HIV/AIDS	Number	2014	182778	231048	140376
Incidence	Global	Both	50-69 yea	HIV/AIDS	Rate	2014	15.208	19.2243	11.6799
Incidence	Global	Both	50-69 yea	HIV/AIDS	Number	2015	184217	233949	139047
Incidence	Global	Both	50-69 yea	HIV/AIDS	Rate	2015	14.8413	18.8479	11.2022
Incidence	Global	Both	50-69 yea	HIV/AIDS	Number	2016	183936	233935	138580
Incidence	Global	Both	50-69 yea	HIV/AIDS	Rate	2016	14.3846	18.2947	10.8376
Incidence	Global	Both	50-69 yea	HIV/AIDS	Number	2017	181771	231429	136519
Incidence	Global	Both	50-69 yea	HIV/AIDS	Rate	2017	13.8453	17.6277	10.3986
Incidence	Global	Both	50-69 yea	HIV/AIDS	Number	2018	176999	226263	131489
Incidence	Global	Both	50-69 yea	HIV/AIDS	Rate	2018	13.1421	16.7999	9.76303
Incidence	Global	Both	50-69 yea	HIV/AIDS	Number	2019	174511	223161	129425
Incidence	Global	Both	50-69 yea	HIV/AIDS	Rate	2019	12.6554	16.1834	9.38576
Incidence	Global	Both	50-54 yea	Chlamydia	Number	1990	4980427	8267605	2751405
Incidence	Global	Both	50-54 yea	Chlamydia	Rate	1990	2342.73	3888.97	1294.22
Incidence	Global	Both	50-54 yea	Chlamydia	Number	1991	5013363	8317822	2769463
Incidence	Global	Both	50-54 yea	Chlamydia	Rate	1991	2332.97	3870.71	1288.77
Incidence	Global	Both	50-54 yea	Chlamydia	Number	1992	5027212	8337301	2776693
Incidence	Global	Both	50-54 yea	Chlamydia	Rate	1992	2324.44	3854.92	1283.86
Incidence	Global	Both	50-54 yea	Chlamydia	Number	1993	5020887	8322942	2772175
Incidence	Global	Both	50-54 yea	Chlamydia	Rate	1993	2312.64	3833.58	1276.88
Incidence	Global	Both	50-54 yea	Chlamydia	Number	1994	5019285	8322979	2770048
Incidence	Global	Both	50-54 yea	Chlamydia	Rate	1994	2300.92	3815.38	1269.83
Incidence	Global	Both	50-54 yea	Chlamydia	Number	1995	5035568	8361093	2777402
Incidence	Global	Both	50-54 yea	Chlamydia	Rate	1995	2298.36	3816.22	1267.68
Incidence	Global	Both	50-54 yea	Chlamydia	Number	1996	5148281	8554537	2841935
Incidence	Global	Both	50-54 yea	Chlamydia	Rate	1996	2315.33	3847.23	1278.1
Incidence	Global	Both	50-54 yea	Chlamydia	Number	1997	5377285	8940195	2969598
Incidence	Global	Both	50-54 yea	Chlamydia	Rate	1997	2331.23	3875.87	1287.42
Incidence	Global	Both	50-54 yea	Chlamydia	Number	1998	5658512	9409469	3126008
Incidence	Global	Both	50-54 yea	Chlamydia	Rate	1998	2356.69	3918.91	1301.94
Incidence	Global	Both	50-54 yea	Chlamydia	Number	1999	6039933	1E+07	3337427
Incidence	Global	Both	50-54 yea	Chlamydia	Rate	1999	2394.88	3982.02	1323.32
Incidence	Global	Both	50-54 yea	Chlamydia	Number	2000	6313546	1E+07	3490867
Incidence	Global	Both	50-54 yea	Chlamydia	Rate	2000	2407.59	4002.19	1331.2
Incidence	Global	Both	50-54 yea	Chlamydia	Number	2001	6651283	1.1E+07	3685742
Incidence	Global	Both	50-54 yea	Chlamydia	Rate	2001	2415.44	4016.64	1338.49
Incidence	Global	Both	50-54 yea	Chlamydia	Number	2002	6930315	1.2E+07	3840158
Incidence	Global	Both	50-54 yea	Chlamydia	Rate	2002	2432.89	4046.02	1348.09
Incidence	Global	Both	50-54 yea	Chlamydia	Number	2003	7241712	1.2E+07	4010476
Incidence	Global	Both	50-54 yea	Chlamydia	Rate	2003	2454.08	4079.04	1359.08
Incidence	Global	Both	50-54 yea	Chlamydia	Number	2004	7505776	1.2E+07	4154636
Incidence	Global	Both	50-54 yea	Chlamydia	Rate	2004	2466.55	4094.74	1365.3
Incidence	Global	Both	50-54 yea	Chlamydia	Number	2005	7864411	1.3E+07	4349745
Incidence	Global	Both	50-54 yea	Chlamydia	Rate	2005	2482.93	4117.45	1373.29
Incidence	Global	Both	50-54 yea	Chlamydia	Number	2006	8003524	1.3E+07	4439472
Incidence	Global	Both	50-54 yea	Chlamydia	Rate	2006	2467.4	4094.12	1368.64
Incidence	Global	Both	50-54 yea	Chlamydia	Number	2007	8004964	1.3E+07	4455879
Incidence	Global	Both	50-54 yea	Chlamydia	Rate	2007	2418.46	4014.99	1346.21
Incidence	Global	Both	50-54 yea	Chlamydia	Number	2008	7900084	1.3E+07	4389495
Incidence	Global	Both	50-54 yea	Chlamydia	Rate	2008	2352.96	3898.47	1307.37
Incidence	Global	Both	50-54 yea	Chlamydia	Number	2009	7792623	1.3E+07	4321094
Incidence	Global	Both	50-54 yea	Chlamydia	Rate	2009	2296.27	3801.84	1273.31
Incidence	Global	Both	50-54 yea	Chlamydia	Number	2010	7786110	1.3E+07	4309037
Incidence	Global	Both	50-54 yea	Chlamydia	Rate	2010	2268.04	3753.61	1255.19
Incidence	Global	Both	50-54 yea	Chlamydia	Number	2011	7907135	1.3E+07	4376741
Incidence	Global	Both	50-54 yea	Chlamydia	Rate	2011	2263.56	3746.13	1252.92
Incidence	Global	Both	50-54 yea	Chlamydia	Number	2012	8127493	1.3E+07	4495326
Incidence	Global	Both	50-54 yea	Chlamydia	Rate	2012	2268.71	3749.01	1254.82
Incidence	Global	Both	50-54 yea	Chlamydia	Number	2013	8464887	1.4E+07	4674969
Incidence	Global	Both	50-54 yea	Chlamydia	Rate	2013	2282.99	3768.04	1260.84

Incidence	Global	Both	55-59 yea	Chlamydia	Rate	2001	804.435	1257.07	455.45
Incidence	Global	Both	55-59 yea	Chlamydia	Number	2002	1746824	2730042	988419
Incidence	Global	Both	55-59 yea	Chlamydia	Rate	2002	802.009	1253.43	453.807
Incidence	Global	Both	55-59 yea	Chlamydia	Number	2003	1821423	2845924	1029993
Incidence	Global	Both	55-59 yea	Chlamydia	Rate	2003	801.077	1251.66	452.999
Incidence	Global	Both	55-59 yea	Chlamydia	Number	2004	1926491	3008002	1088484
Incidence	Global	Both	55-59 yea	Chlamydia	Rate	2004	804.365	1255.93	454.473
Incidence	Global	Both	55-59 yea	Chlamydia	Number	2005	2007396	3131640	1133225
Incidence	Global	Both	55-59 yea	Chlamydia	Rate	2005	804.295	1254.74	454.044
Incidence	Global	Both	55-59 yea	Chlamydia	Number	2006	2109384	3291915	1190200
Incidence	Global	Both	55-59 yea	Chlamydia	Rate	2006	803.418	1253.82	453.321
Incidence	Global	Both	55-59 yea	Chlamydia	Number	2007	2183130	3408629	1231759
Incidence	Global	Both	55-59 yea	Chlamydia	Rate	2007	802.828	1253.5	452.969
Incidence	Global	Both	55-59 yea	Chlamydia	Number	2008	2261359	3531287	1276191
Incidence	Global	Both	55-59 yea	Chlamydia	Rate	2008	801.888	1252.21	452.543
Incidence	Global	Both	55-59 yea	Chlamydia	Number	2009	2329380	3636745	1314998
Incidence	Global	Both	55-59 yea	Chlamydia	Rate	2009	800.155	1249.24	451.709
Incidence	Global	Both	55-59 yea	Chlamydia	Number	2010	2435439	3801430	1374765
Incidence	Global	Both	55-59 yea	Chlamydia	Rate	2010	802.713	1252.94	453.118
Incidence	Global	Both	55-59 yea	Chlamydia	Number	2011	2505013	3911460	1415798
Incidence	Global	Both	55-59 yea	Chlamydia	Rate	2011	805.487	1257.73	455.25
Incidence	Global	Both	55-59 yea	Chlamydia	Number	2012	2562730	4002852	1449741
Incidence	Global	Both	55-59 yea	Chlamydia	Rate	2012	806.911	1260.35	456.471
Incidence	Global	Both	55-59 yea	Chlamydia	Number	2013	2597971	4057617	1469972
Incidence	Global	Both	55-59 yea	Chlamydia	Rate	2013	805.781	1258.5	455.924
Incidence	Global	Both	55-59 yea	Chlamydia	Number	2014	2619507	4088884	1481565
Incidence	Global	Both	55-59 yea	Chlamydia	Rate	2014	803.182	1253.72	454.271
Incidence	Global	Both	55-59 yea	Chlamydia	Number	2015	2641741	4119579	1493342
Incidence	Global	Both	55-59 yea	Chlamydia	Rate	2015	800.265	1247.95	452.379
Incidence	Global	Both	55-59 yea	Chlamydia	Number	2016	2682535	4189485	1517555
Incidence	Global	Both	55-59 yea	Chlamydia	Rate	2016	798.358	1246.85	451.644
Incidence	Global	Both	55-59 yea	Chlamydia	Number	2017	2755447	4318404	1560193
Incidence	Global	Both	55-59 yea	Chlamydia	Rate	2017	799.443	1252.91	452.662
Incidence	Global	Both	55-59 yea	Chlamydia	Number	2018	2882111	4505503	1633414
Incidence	Global	Both	55-59 yea	Chlamydia	Rate	2018	807.563	1262.44	457.68
Incidence	Global	Both	55-59 yea	Chlamydia	Number	2019	3049915	4766621	1726836
Incidence	Global	Both	55-59 yea	Chlamydia	Rate	2019	822.052	1284.76	465.439
Incidence	Global	Both	60-64 yea	Chlamydia	Number	1990	311392	467585	188626
Incidence	Global	Both	60-64 yea	Chlamydia	Rate	1990	193.841	291.071	117.419
Incidence	Global	Both	60-64 yea	Chlamydia	Number	1991	317988	477668	192914
Incidence	Global	Both	60-64 yea	Chlamydia	Rate	1991	193.911	291.284	117.64
Incidence	Global	Both	60-64 yea	Chlamydia	Number	1992	322596	484841	195950
Incidence	Global	Both	60-64 yea	Chlamydia	Rate	1992	193.936	291.473	117.8
Incidence	Global	Both	60-64 yea	Chlamydia	Number	1993	326800	491432	198705
Incidence	Global	Both	60-64 yea	Chlamydia	Rate	1993	194.103	291.886	118.021
Incidence	Global	Both	60-64 yea	Chlamydia	Number	1994	330053	496159	200484
Incidence	Global	Both	60-64 yea	Chlamydia	Rate	1994	194.108	291.797	117.907
Incidence	Global	Both	60-64 yea	Chlamydia	Number	1995	333993	501803	202600
Incidence	Global	Both	60-64 yea	Chlamydia	Rate	1995	194.184	291.749	117.792
Incidence	Global	Both	60-64 yea	Chlamydia	Number	1996	339491	510131	205869
Incidence	Global	Both	60-64 yea	Chlamydia	Rate	1996	194.468	292.214	117.926
Incidence	Global	Both	60-64 yea	Chlamydia	Number	1997	346711	520939	210130
Incidence	Global	Both	60-64 yea	Chlamydia	Rate	1997	194.898	292.836	118.121
Incidence	Global	Both	60-64 yea	Chlamydia	Number	1998	353617	531315	214177
Incidence	Global	Both	60-64 yea	Chlamydia	Rate	1998	195.182	293.264	118.217
Incidence	Global	Both	60-64 yea	Chlamydia	Number	1999	359742	540543	217715
Incidence	Global	Both	60-64 yea	Chlamydia	Rate	1999	195.368	293.557	118.236
Incidence	Global	Both	60-64 yea	Chlamydia	Number	2000	365421	549104	221090
Incidence	Global	Both	60-64 yea	Chlamydia	Rate	2000	195.458	293.707	118.257
Incidence	Global	Both	60-64 yea	Chlamydia	Number	2001	369083	554176	223506
Incidence	Global	Both	60-64 yea	Chlamydia	Rate	2001	195.147	293.012	118.175
Incidence	Global	Both	60-64 yea	Chlamydia	Number	2002	371094	556681	224856
Incidence	Global	Both	60-64 yea	Chlamydia	Rate	2002	194.745	292.138	118.001
Incidence	Global	Both	60-64 yea	Chlamydia	Number	2003	371890	557493	225648
Incidence	Global	Both	60-64 yea	Chlamydia	Rate	2003	193.972	290.78	117.694
Incidence	Global	Both	60-64 yea	Chlamydia	Number	2004	373443	559482	226884
Incidence	Global	Both	60-64 yea	Chlamydia	Rate	2004	193.185	289.424	117.368
Incidence	Global	Both	60-64 yea	Chlamydia	Number	2005	376037	563121	228662
Incidence	Global	Both	60-64 yea	Chlamydia	Rate	2005	192.829	288.764	117.256
Incidence	Global	Both	60-64 yea	Chlamydia	Number	2006	383955	575208	233457
Incidence	Global	Both	60-64 yea	Chlamydia	Rate	2006	193.069	289.238	117.392
Incidence	Global	Both	60-64 yea	Chlamydia	Number	2007	398831	597795	242334
Incidence	Global	Both	60-64 yea	Chlamydia	Rate	2007	192.154	288.013	116.754
Incidence	Global	Both	60-64 yea	Chlamydia	Number	2008	416217	623731	252709
Incidence	Global	Both	60-64 yea	Chlamydia	Rate	2008	191.667	287.227	116.372
Incidence	Global	Both	60-64 yea	Chlamydia	Number	2009	439733	658724	266759
Incidence	Global	Both	60-64 yea	Chlamydia	Rate	2009	191.951	287.544	116.445
Incidence	Global	Both	60-64 yea	Chlamydia	Number	2010	458733	686870	278183
Incidence	Global	Both	60-64 yea	Chlamydia	Rate	2010	192.002	287.489	116.433
Incidence	Global	Both	60-64 yea	Chlamydia	Number	2011	484176	725382	293479
Incidence	Global	Both	60-64 yea	Chlamydia	Rate	2011	192.46	288.339	116.658
Incidence	Global	Both	60-64 yea	Chlamydia	Number	2012	505170	757200	306252
Incidence	Global	Both	60-64 yea	Chlamydia	Rate	2012	193.785	290.465	117.48
Incidence	Global	Both	60-64 yea	Chlamydia	Number	2013	528150	791969	320165
Incidence	Global	Both	60-64 yea	Chlamydia	Rate	2013	195.3	292.856	118.391
Incidence	Global	Both	60-64 yea	Chlamydia	Number	2014	548855	823127	332712
Incidence	Global	Both	60-64 yea	Chlamydia	Rate	2014	196.545	294.761	119.144
Incidence	Global	Both	60-64 yea	Chlamydia	Number	2015	575702	863495	348850
Incidence	Global	Both	60-64 yea	Chlamydia	Rate	2015	197.814	296.701	119.867
Incidence	Global	Both	60-64 yea	Chlamydia	Number	2016	592484	889845	359388
Incidence	Global	Both	60-64 yea	Chlamydia	Rate	2016	198.647	298.345	120.495
Incidence	Global	Both	60-64 yea	Chlamydia	Number	2017	606357	912152	368050
Incidence	Global	Both	60-64 yea	Chlamydia	Rate	2017	199.12	299.539	120.863
Incidence	Global	Both	60-64 yea	Chlamydia	Number	2018	615171	924286	373770
Incidence	Global	Both	60-64 yea	Chlamydia	Rate	2018	199.05	299.071	120.941
Incidence	Global	Both	60-64 yea	Chlamydia	Number	2019	621544	932528	378034
Incidence	Global	Both	60-64 yea	Chlamydia	Rate	2019	198.872	298.375	120.957
Incidence	Global	Both	65-69 yea	Chlamydia	Number	1990	80977	134267	43250.9
Incidence	Global	Both	65-69 yea	Chlamydia	Rate	1990	65.5745	108.728	35.0242
Incidence	Global	Both	65-69 yea	Chlamydia	Number	1991	83613	138466	44647.9
Incidence	Global	Both	65-69 yea	Chlamydia	Rate	1991	65.6959	108.795	35.0805
Incidence	Global	Both	65-69 yea	Chlamydia	Number	1992	86325.1	142851	46068.8
Incidence	Global	Both	65-69 yea	Chlamydia	Rate	1992	65.8431	108.958	35.1383
Incidence	Global	Both	65-69 yea	Chlamydia	Number	1993	88952.8	147268	47455.2
Incidence	Global	Both	65-69 yea	Chlamydia	Rate	1993	65.9201	109.136	35.1676
Incidence	Global	Both	65-69 yea	Chlamydia	Number	1994	91611.8	151830	48896.7
Incidence	Global	Both	65-69 yea	Chlamydia	Rate	1994	66.0293	109.432	35.2424
Incidence	Global	Both	65-69 yea	Chlamydia	Number	1995	93879.3	155767	50092.3
Incidence	Global	Both	65-69 yea	Chlamydia	Rate	1995	66.0819	109.645	35.2602
Incidence	Global	Both	65-69 yea	Chlamydia	Number	1996	95909	158825	51215.6
Incidence	Global	Both	65-69 yea	Chlamydia	Rate	1996	66.1055	109.471	35.3005
Incidence	Global	Both	65-69 yea	Chlamydia	Number	1997	97391.2	161246	52042.7
Incidence	Global	Both	65-69 yea	Chlamydia	Rate	1997	66.1235	109.478	35.3343
Incidence	Global	Both	65-69 yea	Chlamydia	Number	1998	98799.1	163697	52801.1
Incidence	Global	Both	65-69 yea	Chlamydia	Rate	1998	66.1866	109.662	35.372
Incidence	Global	Both	65-69 yea	Chlamydia	Number	1999	99990	165827	53386.3
Incidence	Global	Both	65-69 yea	Chlamydia	Rate	1999	66.1942	109.779	35.3422
Incidence	Global	Both	65-69 yea	Chlamydia	Number	2000	101420	168318	54092.2
Incidence	Global	Both	65-69 yea	Chlamydia	Rate	2000	66.2107	109.883	35.3132
Incidence	Global	Both	65-69 yea	Chlamydia	Number	2001	103330	171185	55209.8
Incidence	Global	Both	65-69 yea	Chlamydia	Rate	2001	66.2757	109.797	35.4113
Incidence	Global	Both	65-69 yea	Chlamydia	Number	2002	105722	174772	56591.2
Incidence	Global	Both	65-69 yea	Chlamydia	Rate	2002	66.3579	109.698	35.5204
Incidence	Global	Both	65-69 yea	Chlamydia	Number	2003	107973	178128	57809.3
Incidence	Global	Both	65-69 yea	Chlamydia	Rate	2003	66.3766	109.504	35.5383
Incidence	Global	Both	65-69 yea	Chlamydia	Number	2004	109955	181361	58869.4
Incidence	Global	Both	65-69 yea	Chlamydia	Rate	2004	66.3621	109.458	35.5299
Incidence	Global	Both	65-69 yea	Chlamydia	Number	2005	111839	184599	59868.4
Incidence	Global	Both	65-69 yea	Chlamydia	Rate	2005	66.3393	109.498	35.5119
Incidence	Global	Both	65-69 yea	Chlamydia	Number	2006	113215	186445	60496.3
Incidence	Global	Both	65-69 yea	Chlamydia	Rate	2006	66.2149	109.045	35.382
Incidence	Global	Both	65-69 yea	Chlamydia	Number	2007	114149	187652	60881.5
Incidence	Global	Both	65-69 yea	Chlamydia	Rate	2007	66.0752	108.622	35.2412
Incidence	Global	Both	65-69 yea	Chlamydia	Number	2008	114764	188679	61097.8
Incidence	Global	Both	65-69 yea	Chlamydia	Rate	2008	65.8167	108.206	35.0392
Incidence	Global	Both	65-69 yea	Chlamydia	Number	2009	115629	190132	61475.2
Incidence	Global	Both	65-69 yea	Chlamydia	Rate	2009	65.5706	107.819	34.8612
Incidence	Global	Both	65-69 yea	Chlamydia	Number	2010	116774	192052	62074.4
Incidence	Global	Both	65-69 yea	Chlamydia	Rate	2010	65.4782	107.688	34.8067
Incidence	Global	Both	65-69 yea	Chlamydia	Number	2011	119618	196904	63596.1
Incidence	Global	Both	65-69 yea	Chlamydia	Rate	2011	65.6444	108.057	34.9004
Incidence	Global	Both	65-69 yea	Chlamydia	Number	2012	124914	205903	66433.6
Incidence	Global	Both	65-69 yea	Chlamydia	Rate	2012	65.5238	108.006	34.8477
Incidence	Global	Both	65-69 yea	Chlamydia	Number	2013	131047	216312	69594.8
Incidence	Global	Both	65-69 yea	Chlamydia	Rate	2013	65.5715	108.235	34.8229
Incidence	Global	Both	65-69 yea	Chlamydia	Number	2014	139072	229830	73823.1
Incidence	Global	Both	65-69 yea	Chlamydia	Rate	2014	65.8378	108.803	34.9483
Incidence	Global	Both	65-69 yea	Chlamydia	Number	2015	145452	240634	77195.9
Incidence	Global	Both	65-69 yea	Chlamydia	Rate	2015	65.9526	109.111	35.0031
Incidence	Global	Both	65-69 yea	Chlamydia	Number	2016	153758	254036	81809.4
Incidence	Global	Both	65-69 yea	Chlamydia	Rate	2016	66.1318	109.262	35.1864
Incidence	Global	Both	65-69 yea	Chlamydia	Number	2017	160516	265373	85737.2
Incidence	Global	Both	65-69 yea	Chlamydia	Rate	2017	66.5687	110.054	35.5565
Incidence	Global	Both	65-69 yea	Chlamydia	Number	2018	167923	277551	89470
Incidence	Global	Both	65-69 yea	Chlamydia	Rate	2018	67.0905	110.89	35.746
Incidence	Global	Both	65-69 yea	Chlamydia	Number	2019	174703	289302	93035.8
Incidence	Global	Both	65-69 yea	Chlamydia	Rate	2019	67.5615	111.88	35.979
Incidence	Global	Both	50-69 yea	Chlamydia	Number	1990	6854907	1.1E+07	4207443
Incidence	Global	Both	50-69 yea	Chlamydia	Rate	1990	1004.91	1549.96	616.802
Incidence	Global	Both	50-69 yea	Chlamydia	Number	1991	6916879	1.1E+07	4250847
Incidence	Global	Both	50-69 yea	Chlamydia	Rate	1991	996.513	1537.39	612.419
Incidence	Global	Both	50-69 yea	Chlamydia	Number	1992	6964744	1.1E+07	4282973
Incidence	Global	Both	50-69 yea	Chlamydia	Rate	1992	987.997	1524.28	607.569
Incidence	Global	Both	50-69 yea	Chlamydia	Number	1993	6990144	1.1E+07	4299253
Incidence	Global	Both	50-69 yea	Chlamydia	Rate	1993	977.965	1509.53	601.493
Incidence	Global	Both	50-69 yea	Chlamydia	Number	1994	7016546	1.1E+07	4315852
Incidence	Global	Both	50-69 yea	Chlamydia	Rate	1994	969.053	1495.05	596.061
Incidence	Global	Both	50-69 yea	Chlamydia	Number	1995	7060265	1.1E+07	4343310
Incidence	Global	Both	50-69 yea	Chlamydia	Rate	1995	963.276	1485.39	592.585
Incidence	Global	Both	50-69 yea	Chlamydia	Number	1996	7195995	1.1E+07	4431378
Incidence	Global	Both	50-69 yea	Chlamydia	Rate	1996	967.54	1494.36	595.823
Incidence	Global	Both	50-69 yea	Chlamydia	Number	1997	7445826	1.2E+07	4592267
Incidence	Global	Both	50-69 yea	Chlamydia	Rate	1997	981.393	1516.08	605.281
Incidence	Global	Both	50-69 yea	Chlamydia	Number	1998	7742584	1.2E+07	4773285
Incidence	Global	Both	50-69 yea	Chlamydia	Rate	1998	1000.12	1544.74	616.569
Incidence	Global	Both	50-69 yea	Chlamydia	Number	1999	8139792	1.3E+07	4991875
Incidence	Global	Both	50-69 yea	Chlamydia	Rate	1999	1027.61	1587.96	630.201
Incidence	Global	Both	50-69 yea	Chlamydia	Number	2000	8430833	1.3E+07	5166759
Incidence	Global	Both	50-69 yea	Chlamydia	Rate	2000	1043.13	1616.15	639.274
Incidence	Global	Both	50-69 yea	Chlamydia	Number	2001	8807606	1.4E+07	5392841
Incidence	Global	Both	50-69 yea	Chlamydia	Rate	2001	1061.5	1650.36	649.948
Incidence	Global	Both	50-69 yea	Chlamydia	Number	2002	9153955	1.4E+07	5598683
Incidence	Global	Both	50-69 yea	Chlamydia	Rate	2002	1073.73	1671.17	656.706
Incidence	Global	Both	50-69 yea	Chlamydia	Number	2003	9542998	1.5E+07	5830515
Incidence	Global	Both	50-69 yea	Chlamydia	Rate	2003	1088.33	1695.01	664.938
Incidence	Global	Both	50-69 yea	Chlamydia	Number	2004	9915664	1.5E+07	6049600
Incidence	Global	Both	50-69 yea	Chlamydia	Rate	2004	1098.32	1708.15	670.089
Incidence	Global	Both	50-69 yea	Chlamydia	Number	2005	1E+07	1.6E+07	6313612
Incidence	Global	Both	50-69 yea	Chlamydia	Rate	2005	1114.04	1732.99	678.94
Incidence	Global	Both	50-69 yea	Chlamydia	Number	2006	1.1E+07	1.6E+07	6468638
Incidence	Global	Both	50-69 yea	Chlamydia	Rate	2006	1108.94	1720.6	676.09
Incidence	Global	Both	50-69 yea	Chlamydia	Number	2007	1.1E+07	1.7E+07	6551830
Incidence	Global	Both	50-69 yea	Chlamydia	Rate	2007	1088.35	1685.24	666.351
Incidence	Global	Both	50-69 yea	Chlamydia	Number	2008	1.1E+07	1.7E+07	6576481
Incidence	Global	Both	50-69 yea	Chlamydia	Rate	2008	1059.41	1638.37	651.601
Incidence	Global	Both	50-69 yea	Chlamydia	Number	2009	1.1E+07	1.7E+07	6567175
Incidence	Global	Both	50-69 yea	Chlamydia	Rate	2009	1030.73	1593.99	633.955
Incidence	Global	Both	50-69 yea	Chlamydia	Number	2010	1.1E+07	1.7E+07	6645589
Incidence	Global	Both	50-69 yea	Chlamydia	Rate	2010	1014.8	1563.47	624.609
Incidence	Global	Both	50-69 yea	Chlamydia	Number	2011	1.1E+07	1.7E+07	6783893
Incidence	Global	Both	50-69 yea	Chlamydia	Rate	2011	1006.84	1551.8	620.038
Incidence	Global	Both	50-69 yea	Chlamydia	Number	2012	1.1E+07	1.7E+07	6967341
Incidence	Global	Both	50-69 yea	Chlamydia	Rate	2012	1004.32	1548.49	618.129
Incidence	Global	Both	50-69 yea	Chlamydia	Number	2013	1.2E+07	1.8E+07	7209154
Incidence	Global	Both	50-69 yea	Chlamydia	Rate	2013	1007.5	1557.45	619.62
Incidence	Global	Both	50-69 yea	Chlamydia	Number	2014	1.2E+07	1.9E+07	7485615
Incidence	Global	Both	50-69 yea	Chlamydia	Rate	2014	1012.54	1567.79	622.838
Incidence	Global	Both	50-69 yea	Chlamydia	Number	2015	1.3E+07	2E+07	7770241
Incidence	Global	Both	50-69 yea	Chlamydia	Rate	2015	1016.73	1571.3	626.003
Incidence	Global	Both	50-69 yea	Chlamydia	Number	2016	1.3E+07	2E+07	8020739
Incidence	Global	Both	50-69 yea	Chlamydia	Rate	2016	1017.91	1575.89	627.256
Incidence	Global	Both	50-69 yea	Chlamydia	Number	2017	1.3E+07	2.1E+07	8232644
Incidence	Global	Both	50-69 yea	Chlamydia	Rate	2017	1019.26	1577.25	627.074
Incidence	Global	Both	50-69 yea	Chlamydia	Number	2018	1.4E+07	2.1E+07	8533094
Incidence	Global	Both	50-69 yea	Chlamydia	Rate	2018	1031.14	1596.24	633.579
Incidence	Global	Both	50-69 yea	Chlamydia	Number	2019	1.5E+07	2.3E+07	8923359
Incidence	Global	Both	50-69 yea	Chlamydia	Rate	2019	1056.49	1634.8	647.113
Incidence	Global	Both	50-54 yea	Sexually tr	Number	1990	2.3E+07	3.1E+07	1.6E+07
Incidence	Global	Both	50-54 yea	Sexually tr	Rate	1990	10776.8	14587.7	7551.59
Incidence	Global	Both	50-54 yea	Sexually tr	Number	1991	2.3E+07	3.1E+07	1.6E+07
Incidence	Global	Both	50-54 yea	Sexually tr	Rate	1991	10787.1	14602.4	7557.46
Incidence	Global	Both	50-54 yea	Sexually tr	Number	1992	2.3E+07	3.2E+07	1.6E+07
Incidence	Global	Both	50-54 yea	Sexually tr	Rate	1992	10806.1	14629.1	7560.11
Incidence	Global	Both	50-54 yea	Sexually tr	Number	1993	2.4E+07	3.2E+07	1.6E+07
Incidence	Global	Both	50-54 yea	Sexually tr	Rate	1993	10824.7	14657.5	7560.39
Incidence	Global	Both	50-54 yea	Sexually tr	Number	1994	2.4E+07	3.2E+07	1.6E+07
Incidence	Global	Both	50-54 yea	Sexually tr	Rate	1994	10839.1	14679.7	7555.88
Incidence	Global	Both	50-54 yea	Sexually tr	Number	1995	2.4E+07	3.2E+07	1.7E+07
Incidence	Global	Both	50-54 yea	Sexually tr	Rate	1995	10863.4	14713.9	7567.17
Incidence	Global	Both	50-54 yea	Sexually tr	Number	1996	2.4E+07	3.3E+07	1.7E+07
Incidence	Global	Both	50-54 yea	Sexually tr	Rate	1996	10897.5	14757	7595.95
Incidence	Global	Both	50-54 yea	Sexually tr	Number	1997	2.5E+07	3.4E+07	1.8E+07
Incidence	Global	Both	50-54 yea	Sexually tr	Rate	1997	10887.4	14735.9	7597.8
Incidence	Global	Both	50-54 yea	Sexually tr	Number	1998	2.6E+07	3.5E+07	1.8E+07
Incidence	Global	Both	50-54 yea	Sexually tr	Rate	1998	10875.7	14731.7	7600.11
Incidence	Global	Both	50-54 yea	Sexually tr	Number	1999	2.7E+07	3.7E+07	1.9E+07
Incidence	Global	Both	50-54 yea	Sexually tr	Rate	1999	10884.1	14773.4	7614.71
Incidence	Global	Both	50-54 yea	Sexually tr	Number	2000	2.8E+07	3.9E+07	2E+07
Incidence	Global	Both	50-54 yea	Sexually tr	Rate	2000	10868.1	14771.2	7607.29
Incidence	Global	Both	50-54 yea	Sexually tr	Number	2001	3E+07	4.1E+07	2.1E+07
Incidence	Global	Both	50-54 yea	Sexually tr	Rate	2001	10867.7	14765.5	7603.08
Incidence	Global	Both	50-54 yea	Sexually tr	Number	2002	3.1E+07	4.2E+07	2.2E+07
Incidence	Global	Both	50-54 yea	Sexually tr	Rate	2002	10892.6	14792.9	7611.58
Incidence	Global	Both	50-54 yea	Sexually tr	Number	2003	3.2E+07	4.4E+07	2.3E+07
Incidence	Global	Both	50-54 yea	Sexually tr	Rate	2003	10924.8	14832	7625.28
Incidence	Global	Both	50-54 yea	Sexually tr	Number	2004	3.3E+07	4.5E+07	2.3E+07
Incidence	Global	Both	50-54 yea	Sexually tr	Rate	2004	10948.2	14855.4	7637.35
Incidence	Global	Both	50-54 yea	Sexually tr	Number	2005	3.5E+07	4.7E+07	2.4E+07
Incidence	Global	Both	50-54 yea	Sexually tr	Rate	2005	10980.8	14895.5	7655.95
Incidence	Global	Both	50-54 yea	Sexually tr	Number	2006	3.6E+07	4.8E+07	2.5E+07
Incidence	Global	Both	50-54 yea	Sexually tr	Rate	2006	10975.7	14885.7	7659.69
Incidence	Global	Both	50-54 yea	Sexually tr	Number	2007	3.6E+07	4.9E+07	2.5E+07
Incidence	Global	Both	50-54 yea	Sexually tr	Rate	2007	10935.9	14820.9	7626.85
Incidence	Global	Both	50-54 yea	Sexually tr	Number	2008	3.7E+07	4.9E+07	2.5E+07
Incidence	Global	Both	50-54 yea	Sexually tr	Rate	2008	10876	14742.8	7569.13
Incidence	Global	Both	50-54 yea	Sexually tr	Number	2009	3.7E+07	5E+07	2.6E+07
Incidence	Global	Both	50-54 yea	Sexually tr	Rate	2009	10821.7	14685	7528.58
Incidence	Global	Both	50-54 yea	Sexually tr	Number	2010	3.7E+07	5E+07	2.6E+07
Incidence	Global	Both	50-54 yea	Sexually tr	Rate	2010	10794.1	14648.5	7509.14
Incidence	Global	Both	50-54 yea	Sexually tr	Number	2011	3.8E+07	5.1E+07	2.6E+07
Incidence	Global	Both	50-54 yea	Sexually tr	Rate	2011	10780.3	14640.1	7500.76
Incidence	Global	Both	50-54 yea	Sexually tr	Number	2012	3.9E+07	5.2E+07	2.7E+07
Incidence	Global	Both	50-54 yea	Sexually tr	Rate	2012	10766.1	14624.7	7493.43
Incidence	Global	Both	50-54 yea	Sexually tr	Number	2013	4E+07	5.4E+07	2.8E+07
Incidence	Global	Both	50-54 yea	Sexually tr	Rate	2013	10759.3	14617.2	7496.64
Incidence	Global	Both	50-54 yea	Sexually tr	Number	2014	4.1E+07	5.6E+07	2.9E+07
Incidence	Global	Both	50-54 yea	Sexually tr	Rate	2014	10762.4	14625	7510.64
Incidence	Global	Both	50-54 yea	Sexually tr	Number	2015	4.3E+07	5.9E+07	3E+07
Incidence	Global	Both	50-54 yea	Sexually tr	Rate	2015	10779.5	14657.4	7533.95
Incidence	Global	Both	50-54 yea	Sexually tr	Number	2016	4.5E+07	6.1E+07	3.1E+07
Incidence	Global	Both	50-54 yea	Sexually tr	Rate	2016	10858.7	14767.2	7571.49
Incidence	Global	Both	50-54 yea	Sexually tr	Number	2017	4.6E+07	6.3E+07	3.2E+07
Incidence	Global	Both	50-54 yea	Sexually tr	Rate	2017	10938.2	14869.6	7615.96
Incidence	Global	Both	50-54 yea	Sexually tr	Number	2018	4.7E+07	6.4E+07	3.3E+07
Incidence	Global	Both	50-54 yea	Sexually tr	Rate	2018	11003.9	14963.5	7658.73
Incidence	Global	Both	50-54 yea	Sexually tr	Number	2019	4.9E+07	6.6E+07	3.4E+07
Incidence	Global	Both	50-54 yea	Sexually tr	Rate	2019	11108.3	15084.8	7730.79
Incidence	Global	Both	55-59 yea	Sexually tr	Number	1990	1.1E+07	1.6E+07	8322556
Incidence	Global	Both	55-59 yea	Sexually tr	Rate	1990	6181.51	8509.82	4488.6
Incidence	Global	Both	55-59 yea	Sexually tr	Number	1991	1.2E+07	1.6E+07	8434836
Incidence	Global	Both	55-59 yea	Sexually tr	Rate	1991	6185.23	8504.55	4487.65
Incidence	Global	Both	55-59 yea	Sexually tr	Number	1992	1.2E+07	1.6E+07	8583506
Incidence	Global	Both	55-59 yea	Sexually tr	Rate	1992	6184.39	8496.92	4489.04
Incidence	Global	Both	55-59 yea	Sexually tr	Number	1993	1.2E+07	1.7E+07	8723787
Incidence	Global	Both	55-59 yea	Sexually tr	Rate	1993	6180.46	8494.13	4488.62
Incidence	Global	Both	55-59 yea	Sexually tr	Number	1994	1.2E+07	1.7E+07	8849223
Incidence	Global	Both	55-59 yea	Sexually tr	Rate	1994	6177.75	8495.58	4488.8
Incidence	Global	Both	55-59 yea	Sexually tr	Number	1995	1.2E+07	1.7E+07	8969991
Incidence	Global	Both	55-59 yea	Sexually tr	Rate	1995	6175.98	8499.64	4489.8
Incidence	Global	Both	55-59 yea	Sexually tr	Number	1996	1.2E+07	1.7E+07	9053532
Incidence	Global	Both	55-59 yea	Sexually tr	Rate	1996	6175.37	8490.57	4488.03
Incidence	Global	Both	55-59 yea	Sexually tr	Number	1997	1.3E+07	1.7E+07	9108855
Incidence	Global	Both	55-59 yea	Sexually tr	Rate	1997	6181.38	8492.3	4490.3
Incidence	Global	Both	55-59 yea	Sexually tr	Number	1998	1.3E+07	1.7E+07	9148967
Incidence	Global	Both	55-59 yea	Sexually tr	Rate	1998	6189.75	8500.34	4493.2
Incidence	Global	Both	55-59 yea	Sexually tr	Number	1999	1.3E+07	1.7E+07	9201854
Incidence	Global	Both	55-59 yea	Sexually tr	Rate	1999	6196.62	8519.66	4494.96
Incidence	Global	Both	55-59 yea	Sexually tr	Number	2000	1.3E+07	1.8E+07	9263585
Incidence	Global	Both	55-59 yea	Sexually tr	Rate	2000	6207.85	8547.1	4500.08
Incidence	Global	Both	55-59 yea	Sexually tr	Number	2001	1.3E+07	1.8E+07	9444381
Incidence	Global	Both	55-59 yea	Sexually tr	Rate	2001	6221.23	8569.82	4511.76
Incidence	Global	Both	55-59 yea	Sexually tr	Number	2002	1.4E+07	1.9E+07	9815005
Incidence	Global	Both	55-59 yea	Sexually tr	Rate	2002	6210.86	8559.52	4506.31
Incidence	Global	Both	55-59 yea	Sexually tr	Number	2003	1.4E+07	1.9E+07	1E+07
Incidence	Global	Both	55-59 yea	Sexually tr	Rate	2003	6197.7	8546.03	4501.7
Incidence	Global	Both	55-59 yea	Sexually tr	Number	2004	1.5E+07	2E+07	1.1E+07
Incidence	Global	Both	55-59 yea	Sexually tr	Rate	2004	6191.64	8541.83	4503.42
Incidence	Global	Both	55-59 yea	Sexually tr	Number	2005	1.5E+07	2.1E+07	1.1E+07
Incidence	Global	Both	55-59 yea	Sexually tr	Rate	2005	6178.59	8528.7	4500.59
Incidence	Global	Both	55-59 yea	Sexually tr	Number	2006	1.6E+07	2.2E+07	1.2E+07
Incidence	Global	Both	55-59 yea	Sexually tr	Rate	2006	6173.46	8519.21	4489.44
Incidence	Global	Both	55-59 yea	Sexually tr	Number	2007	1.7E+07	2.3E+07	1.2E+07
Incidence	Global	Both	55-59 yea	Sexually tr	Rate	2007	6178.98	8526.8	4490.03
Incidence	Global	Both	55-59 yea	Sexually tr	Number	2008	1.7E+07	2.4E+07	1.3E+07
Incidence	Global	Both	55-59 yea	Sexually tr	Rate	2008	6186.66	8538.4	4494.78
Incidence	Global	Both	55-59 yea	Sexually tr	Number	2009	1.8E+07	2.5E+07	1.3E+07
Incidence	Global	Both	55-59 yea	Sexually tr	Rate	2009	6193.36	8549.37	4499.64
Incidence	Global	Both	55-59 yea	Sexually tr	Number	2010	1.9E+07	2.6E+07	1.4E+07
Incidence	Global	Both	55-59 yea	Sexually tr	Rate	2010	6207.06	8568.78	4510.35
Incidence	Global	Both	55-59 yea	Sexually tr	Number	2011	1.9E+07	2.7E+07	1.4E+07
Incidence	Global	Both	55-59 yea	Sexually tr	Rate	2011	6214.44	8578.98	4518.94
Incidence	Global	Both	55-59 yea	Sexually tr	Number	2012	2E+07	2.7E+07	1.4E+07
Incidence	Global	Both	55-59 yea	Sexually tr	Rate	2012	6215.27	8580.75	4522.83
Incidence	Global	Both	55-59 yea	Sexually tr	Number	2013	2E+07	2.8E+07	1.5E+07
Incidence	Global	Both	55-59 yea	Sexually tr	Rate	2013	6210.09	8576.47	4521.93
Incidence	Global	Both	55-59 yea	Sexually tr	Number	2014	2E+07	2.8E+07	1.5E+07
Incidence	Global	Both	55-59 yea	Sexually tr	Rate	2014	6203.22	8571.67	4519.39
Incidence	Global	Both	55-59 yea	Sexually tr	Number	2015	2E+07	2.8E+07	1.5E+07
Incidence	Global	Both	55-59 yea	Sexually tr	Rate	2015	6199.61	8571.61	4514.82
Incidence	Global	Both	55-59 yea	Sexually tr	Number	2016	2.1E+07	2.9E+07	1.5E+07
Incidence	Global	Both	55-59 yea	Sexually tr	Rate	2016	6216.69	8577.06	4524.59
Incidence	Global	Both	55-59 yea	Sexually tr	Number	2017	2.2E+07	3E+07	1.6E+07
Incidence	Global	Both	55-59 yea	Sexually tr	Rate	2017	6239.26	8598.17	4538.22
Incidence	Global	Both	55-59 yea	Sexually tr	Number	2018	2.2E+07	3.1E+07	1.6E+07
Incidence	Global	Both	55-59 yea	Sexually tr	Rate	2018	6253.7	8607.68	4547.35
Incidence	Global	Both	55-59 yea	Sexually tr	Number	2019	2.3E+07	3.2E+07	1.7E+07
Incidence	Global	Both	55-59 yea	Sexually tr	Rate	2019	6271.74	8622.11	4560.52
Incidence	Global	Both	60-64 yea	Sexually tr	Number	1990	5671584	7378345	4354848
Incidence	Global	Both	60-64 yea	Sexually tr	Rate	1990	3530.55	4593.01	2710.88
Incidence	Global	Both	60-64 yea	Sexually tr	Number	1991	5786983	7533225	4437767
Incidence	Global	Both	60-64 yea	Sexually tr	Rate	1991	3528.93	4593.8	2706.17
Incidence	Global	Both	60-64 yea	Sexually tr	Number	1992	5869776	7643482	4501422
Incidence	Global	Both	60-64 yea	Sexually tr	Rate	1992	3528.75	4595.05	2706.13
Incidence	Global	Both	60-64 yea	Sexually tr	Number	1993	5940945	7723438	4558278
Incidence	Global	Both	60-64 yea	Sexually tr	Rate	1993	3528.63	4587.34	2707.39
Incidence	Global	Both	60-64 yea	Sexually tr	Number	1994	6003359	7805069	4608758
Incidence	Global	Both	60-64 yea	Sexually tr	Rate	1994	3530.65	4590.25	2710.46
Incidence	Global	Both	60-64 yea	Sexually tr	Number	1995	6075643	7901168	4667349
Incidence	Global	Both	60-64 yea	Sexually tr	Rate	1995	3532.39	4593.75	2713.6
Incidence	Global	Both	60-64 yea	Sexually tr	Number	1996	6169061	8018554	4726923
Incidence	Global	Both	60-64 yea	Sexually tr	Rate	1996	3533.77	4593.2	2707.68
Incidence	Global	Both	60-64 yea	Sexually tr	Number	1997	6285060	8164845	4815286
Incidence	Global	Both	60-64 yea	Sexually tr	Rate	1997	3533.04	4589.72	2706.83
Incidence	Global	Both	60-64 yea	Sexually tr	Number	1998	6397741	8305744	4902430
Incidence	Global	Both	60-64 yea	Sexually tr	Rate	1998	3531.29	4584.43	2705.94
Incidence	Global	Both	60-64 yea	Sexually tr	Number	1999	6499729	8439321	4981989
Incidence	Global	Both	60-64 yea	Sexually tr	Rate	1999	3529.86	4583.21	2705.61
Incidence	Global	Both	60-64 yea	Sexually tr	Number	2000	6596668	8568158	5057910
Incidence	Global	Both	60-64 yea	Sexually tr	Rate	2000	3528.45	4582.97	2705.39
Incidence	Global	Both	60-64 yea	Sexually tr	Number	2001	6672029	8672587	5117475
Incidence	Global	Both	60-64 yea	Sexually tr	Rate	2001	3527.73	4585.49	2705.79
Incidence	Global	Both	60-64 yea	Sexually tr	Number	2002	6727555	8754663	5162355
Incidence	Global	Both	60-64 yea	Sexually tr	Rate	2002	3530.52	4594.32	2709.13
Incidence	Global	Both	60-64 yea	Sexually tr	Number	2003	6779019	8832709	5205128
Incidence	Global	Both	60-64 yea	Sexually tr	Rate	2003	3535.83	4607	2714.91
Incidence	Global	Both	60-64 yea	Sexually tr	Number	2004	6844064	8927893	5259347
Incidence	Global	Both	60-64 yea	Sexually tr	Rate	2004	3540.48	4618.46	2720.7
Incidence	Global	Both	60-64 yea	Sexually tr	Number	2005	6917191	9016680	5318894
Incidence	Global	Both	60-64 yea	Sexually tr	Rate	2005	3547.08	4623.68	2727.48
Incidence	Global	Both	60-64 yea	Sexually tr	Number	2006	7068460	9215649	5426644
Incidence	Global	Both	60-64 yea	Sexually tr	Rate	2006	3554.32	4634.01	2728.74
Incidence	Global	Both	60-64 yea	Sexually tr	Number	2007	7369212	9609422	5648091
Incidence	Global	Both	60-64 yea	Sexually tr	Rate	2007	3550.43	4629.74	2721.21
Incidence	Global	Both	60-64 yea	Sexually tr	Number	2008	7696137	1E+07	5890716
Incidence	Global	Both	60-64 yea	Sexually tr	Rate	2008	3544.06	4621.75	2712.67
Incidence	Global	Both	60-64 yea	Sexually tr	Number	2009	8111778	1.1E+07	6199948
Incidence	Global	Both	60-64 yea	Sexually tr	Rate	2009	3540.93	4618.84	2706.38
Incidence	Global	Both	60-64 yea	Sexually tr	Number	2010	8445253	1.1E+07	6447094
Incidence	Global	Both	60-64 yea	Sexually tr	Rate	2010	3534.75	4614.16	2698.43
Incidence	Global	Both	60-64 yea	Sexually tr	Number	2011	8888242	1.2E+07	6784548
Incidence	Global	Both	60-64 yea	Sexually tr	Rate	2011	3533.08	4611.65	2696.86
Incidence	Global	Both	60-64 yea	Sexually tr	Number	2012	9221797	1.2E+07	7046542
Incidence	Global	Both	60-64 yea	Sexually tr	Rate	2012	3537.52	4614.44	2703.08
Incidence	Global	Both	60-64 yea	Sexually tr	Number	2013	9583416	1.3E+07	7333287
Incidence	Global	Both	60-64 yea	Sexually tr	Rate	2013	3543.77	4623.06	2711.72
Incidence	Global	Both	60-64 yea	Sexually tr	Number	2014	9912411	1.3E+07	7598316
Incidence	Global	Both	60-64 yea	Sexually tr	Rate	2014	3549.63	4629.01	2720.95
Incidence	Global	Both	60-64 yea	Sexually tr	Number	2015	1E+07	1.4E+07	7932502
Incidence	Global	Both	60-64 yea	Sexually tr	Rate	2015	3558.61	4639.25	2725.65
Incidence	Global	Both	60-64 yea	Sexually tr	Number	2016	1.1E+07	1.4E+07	8146013
Incidence	Global	Both	60-64 yea	Sexually tr	Rate	2016	3570.49	4658.35	2731.18
Incidence	Global	Both	60-64 yea	Sexually tr	Number	2017	1.1E+07	1.4E+07	8337090
Incidence	Global	Both	60-64 yea	Sexually tr	Rate	2017	3580.75	4675.51	2737.8
Incidence	Global	Both	60-64 yea	Sexually tr	Number	2018	1.1E+07	1.4E+07	8457489
Incidence	Global	Both	60-64 yea	Sexually tr	Rate	2018	3581.55	4673.31	2736.58
Incidence	Global	Both	60-64 yea	Sexually tr	Number	2019	1.1E+07	1.5E+07	8535806
Incidence	Global	Both	60-64 yea	Sexually tr	Rate	2019	3577.43	4675.24	2731.15
Incidence	Global	Both	65-69 yea	Sexually tr	Number	1990	3428181	4543160	2570480
Incidence	Global	Both	65-69 yea	Sexually tr	Rate	1990	2776.11	3679.01	2081.55
Incidence	Global	Both	65-69 yea	Sexually tr	Number	1991	3528621	4680939	2646610
Incidence	Global	Both	65-69 yea	Sexually tr	Rate	1991	2772.49	3677.88	2079.48
Incidence	Global	Both	65-69 yea	Sexually tr	Number	1992	3630946	4817240	2720255
Incidence	Global	Both	65-69 yea	Sexually tr	Rate	1992	2769.45	3674.28	2074.83
Incidence	Global	Both	65-69 yea	Sexually tr	Number	1993	3736444	4952192	2793974
Incidence	Global	Both	65-69 yea	Sexually tr	Rate	1993	2768.96	3669.92	2070.53
Incidence	Global	Both	65-69 yea	Sexually tr	Number	1994	3842877	5086337	2871540
Incidence	Global	Both	65-69 yea	Sexually tr	Rate	1994	2769.76	3665.98	2069.67
Incidence	Global	Both	65-69 yea	Sexually tr	Number	1995	3939166	5207625	2938283
Incidence	Global	Both	65-69 yea	Sexually tr	Rate	1995	2772.79	3665.66	2068.27
Incidence	Global	Both	65-69 yea	Sexually tr	Number	1996	4027249	5325476	3007901
Incidence	Global	Both	65-69 yea	Sexually tr	Rate	1996	2775.79	3670.6	2073.2
Incidence	Global	Both	65-69 yea	Sexually tr	Number	1997	4094706	5415731	3060611
Incidence	Global	Both	65-69 yea	Sexually tr	Rate	1997	2780.09	3677	2077.99
Incidence	Global	Both	65-69 yea	Sexually tr	Number	1998	4155565	5497537	3106099
Incidence	Global	Both	65-69 yea	Sexually tr	Rate	1998	2783.86	3682.86	2080.81
Incidence	Global	Both	65-69 yea	Sexually tr	Number	1999	4211753	5573197	3141929
Incidence	Global	Both	65-69 yea	Sexually tr	Rate	1999	2788.22	3689.5	2079.98
Incidence	Global	Both	65-69 yea	Sexually tr	Number	2000	4274366	5653902	3189403
Incidence	Global	Both	65-69 yea	Sexually tr	Rate	2000	2790.45	3691.06	2082.15
Incidence	Global	Both	65-69 yea	Sexually tr	Number	2001	4350665	5756690	3247141
Incidence	Global	Both	65-69 yea	Sexually tr	Rate	2001	2790.5	3692.32	2082.7
Incidence	Global	Both	65-69 yea	Sexually tr	Number	2002	4441500	5877996	3316354
Incidence	Global	Both	65-69 yea	Sexually tr	Rate	2002	2787.78	3689.42	2081.56
Incidence	Global	Both	65-69 yea	Sexually tr	Number	2003	4528836	5995400	3384890
Incidence	Global	Both	65-69 yea	Sexually tr	Rate	2003	2784.1	3685.67	2080.86
Incidence	Global	Both	65-69 yea	Sexually tr	Number	2004	4607932	6103466	3449175
Incidence	Global	Both	65-69 yea	Sexually tr	Rate	2004	2781.06	3683.67	2081.71
Incidence	Global	Both	65-69 yea	Sexually tr	Number	2005	4685396	6203369	3507143
Incidence	Global	Both	65-69 yea	Sexually tr	Rate	2005	2779.22	3679.63	2080.32
Incidence	Global	Both	65-69 yea	Sexually tr	Number	2006	4752205	6300062	3555227
Incidence	Global	Both	65-69 yea	Sexually tr	Rate	2006	2779.38	3684.67	2079.32
Incidence	Global	Both	65-69 yea	Sexually tr	Number	2007	4808402	6378392	3593660
Incidence	Global	Both	65-69 yea	Sexually tr	Rate	2007	2783.34	3692.13	2080.19
Incidence	Global	Both	65-69 yea	Sexually tr	Number	2008	4864772	6457298	3632386
Incidence	Global	Both	65-69 yea	Sexually tr	Rate	2008	2789.92	3703.22	2083.15
Incidence	Global	Both	65-69 yea	Sexually tr	Number	2009	4930473	6549194	3682527
Incidence	Global	Both	65-69 yea	Sexually tr	Rate	2009	2795.96	3713.9	2088.28
Incidence	Global	Both	65-69 yea	Sexually tr	Number	2010	4999229	6644451	3729317
Incidence	Global	Both	65-69 yea	Sexually tr	Rate	2010	2803.2	3725.71	2091.12
Incidence	Global	Both	65-69 yea	Sexually tr	Number	2011	5120816	6808439	3819822
Incidence	Global	Both	65-69 yea	Sexually tr	Rate	2011	2810.21	3736.35	2096.25
Incidence	Global	Both	65-69 yea	Sexually tr	Number	2012	5353473	7107844	3993497
Incidence	Global	Both	65-69 yea	Sexually tr	Rate	2012	2808.17	3728.42	2094.79
Incidence	Global	Both	65-69 yea	Sexually tr	Number	2013	5603418	7426246	4180862
Incidence	Global	Both	65-69 yea	Sexually tr	Rate	2013	2803.76	3715.84	2091.96
Incidence	Global	Both	65-69 yea	Sexually tr	Number	2014	5918956	7835576	4417060
Incidence	Global	Both	65-69 yea	Sexually tr	Rate	2014	2802.07	3709.41	2091.07
Incidence	Global	Both	65-69 yea	Sexually tr	Number	2015	6170989	8172037	4600729
Incidence	Global	Both	65-69 yea	Sexually tr	Rate	2015	2798.13	3705.47	2086.12
Incidence	Global	Both	65-69 yea	Sexually tr	Number	2016	6516294	8630019	4862558
Incidence	Global	Both	65-69 yea	Sexually tr	Rate	2016	2802.67	3711.79	2091.4
Incidence	Global	Both	65-69 yea	Sexually tr	Number	2017	6781529	8993019	5056753
Incidence	Global	Both	65-69 yea	Sexually tr	Rate	2017	2812.41	3729.54	2097.11
Incidence	Global	Both	65-69 yea	Sexually tr	Number	2018	7051504	9353804	5256393
Incidence	Global	Both	65-69 yea	Sexually tr	Rate	2018	2817.29	3737.13	2100.09
Incidence	Global	Both	65-69 yea	Sexually tr	Number	2019	7286326	9659303	5431306
Incidence	Global	Both	65-69 yea	Sexually tr	Rate	2019	2817.78	3735.47	2100.41
Incidence	Global	Both	50-69 yea	Sexually tr	Number	1990	4.3E+07	5.6E+07	3.4E+07
Incidence	Global	Both	50-69 yea	Sexually tr	Rate	1990	6372.86	8169.48	4991.93
Incidence	Global	Both	50-69 yea	Sexually tr	Number	1991	4.4E+07	5.7E+07	3.5E+07
Incidence	Global	Both	50-69 yea	Sexually tr	Rate	1991	6356.62	8142.55	4982
Incidence	Global	Both	50-69 yea	Sexually tr	Number	1992	4.5E+07	5.7E+07	3.5E+07
Incidence	Global	Both	50-69 yea	Sexually tr	Rate	1992	6340.58	8112.56	4970.85
Incidence	Global	Both	50-69 yea	Sexually tr	Number	1993	4.5E+07	5.8E+07	3.5E+07
Incidence	Global	Both	50-69 yea	Sexually tr	Rate	1993	6322.43	8080	4958.14
Incidence	Global	Both	50-69 yea	Sexually tr	Number	1994	4.6E+07	5.8E+07	3.6E+07
Incidence	Global	Both	50-69 yea	Sexually tr	Rate	1994	6307.44	8052.18	4946.25
Incidence	Global	Both	50-69 yea	Sexually tr	Number	1995	4.6E+07	5.9E+07	3.6E+07
Incidence	Global	Both	50-69 yea	Sexually tr	Rate	1995	6297.17	8030.47	4937.73
Incidence	Global	Both	50-69 yea	Sexually tr	Number	1996	4.7E+07	6E+07	3.7E+07
Incidence	Global	Both	50-69 yea	Sexually tr	Rate	1996	6303.92	8036.64	4942.74
Incidence	Global	Both	50-69 yea	Sexually tr	Number	1997	4.8E+07	6.1E+07	3.8E+07
Incidence	Global	Both	50-69 yea	Sexually tr	Rate	1997	6330.87	8076.06	4960.18
Incidence	Global	Both	50-69 yea	Sexually tr	Number	1998	4.9E+07	6.3E+07	3.9E+07
Incidence	Global	Both	50-69 yea	Sexually tr	Rate	1998	6364.22	8116.28	4985.06
Incidence	Global	Both	50-69 yea	Sexually tr	Number	1999	5.1E+07	6.5E+07	4E+07
Incidence	Global	Both	50-69 yea	Sexually tr	Rate	1999	6419.16	8191.07	5026.36
Incidence	Global	Both	50-69 yea	Sexually tr	Number	2000	5.2E+07	6.7E+07	4.1E+07
Incidence	Global	Both	50-69 yea	Sexually tr	Rate	2000	6452.42	8247.6	5051.23
Incidence	Global	Both	50-69 yea	Sexually tr	Number	2001	5.4E+07	6.9E+07	4.2E+07
Incidence	Global	Both	50-69 yea	Sexually tr	Rate	2001	6504.64	8324.54	5085.23
Incidence	Global	Both	50-69 yea	Sexually tr	Number	2002	5.6E+07	7.1E+07	4.4E+07
Incidence	Global	Both	50-69 yea	Sexually tr	Rate	2002	6536.37	8365.78	5103.9
Incidence	Global	Both	50-69 yea	Sexually tr	Number	2003	5.8E+07	7.4E+07	4.5E+07
Incidence	Global	Both	50-69 yea	Sexually tr	Rate	2003	6573.22	8417.72	5131.48
Incidence	Global	Both	50-69 yea	Sexually tr	Number	2004	6E+07	7.6E+07	4.7E+07
Incidence	Global	Both	50-69 yea	Sexually tr	Rate	2004	6601.29	8453.4	5150.62
Incidence	Global	Both	50-69 yea	Sexually tr	Number	2005	6.2E+07	7.9E+07	4.8E+07
Incidence	Global	Both	50-69 yea	Sexually tr	Rate	2005	6646.14	8516.34	5172.47
Incidence	Global	Both	50-69 yea	Sexually tr	Number	2006	6.4E+07	8.2E+07	5E+07
Incidence	Global	Both	50-69 yea	Sexually tr	Rate	2006	6650.59	8529.46	5179.96
Incidence	Global	Both	50-69 yea	Sexually tr	Number	2007	6.5E+07	8.4E+07	5.1E+07
Incidence	Global	Both	50-69 yea	Sexually tr	Rate	2007	6628.84	8509.24	5164.57
Incidence	Global	Both	50-69 yea	Sexually tr	Number	2008	6.7E+07	8.6E+07	5.2E+07
Incidence	Global	Both	50-69 yea	Sexually tr	Rate	2008	6591.19	8475.34	5132.34
Incidence	Global	Both	50-69 yea	Sexually tr	Number	2009	6.8E+07	8.7E+07	5.3E+07
Incidence	Global	Both	50-69 yea	Sexually tr	Rate	2009	6544.66	8431.34	5095.48
Incidence	Global	Both	50-69 yea	Sexually tr	Number	2010	6.9E+07	8.9E+07	5.4E+07
Incidence	Global	Both	50-69 yea	Sexually tr	Rate	2010	6516.46	8405.43	5073.93
Incidence	Global	Both	50-69 yea	Sexually tr	Number	2011	7.1E+07	9.1E+07	5.5E+07
Incidence	Global	Both	50-69 yea	Sexually tr	Rate	2011	6488.69	8353.11	5057.14
Incidence	Global	Both	50-69 yea	Sexually tr	Number	2012	7.3E+07	9.4E+07	5.7E+07
Incidence	Global	Both	50-69 yea	Sexually tr	Rate	2012	6466.09	8307.71	5042.66
Incidence	Global	Both	50-69 yea	Sexually tr	Number	2013	7.5E+07	9.6E+07	5.9E+07
Incidence	Global	Both	50-69 yea	Sexually tr	Rate	2013	6455	8280.62	5034.77
Incidence	Global	Both	50-69 yea	Sexually tr	Number	2014	7.8E+07	9.9E+07	6E+07
Incidence	Global	Both	50-69 yea	Sexually tr	Rate	2014	6450.22	8272.44	5030.7
Incidence	Global	Both	50-69 yea	Sexually tr	Number	2015	8E+07	1E+08	6.2E+07
Incidence	Global	Both	50-69 yea	Sexually tr	Rate	2015	6450.32	8275.86	5033.73
Incidence	Global	Both	50-69 yea	Sexually tr	Number	2016	8.3E+07	1.1E+08	6.5E+07
Incidence	Global	Both	50-69 yea	Sexually tr	Rate	2016	6474.12	8303.29	5053.52
Incidence	Global	Both	50-69 yea	Sexually tr	Number	2017	8.5E+07	1.1E+08	6.7E+07
Incidence	Global	Both	50-69 yea	Sexually tr	Rate	2017	6505.6	8337.81	5070.63
Incidence	Global	Both	50-69 yea	Sexually tr	Number	2018	8.8E+07	1.1E+08	6.9E+07
Incidence	Global	Both	50-69 yea	Sexually tr	Rate	2018	6520.52	8364.37	5089.31
Incidence	Global	Both	50-69 yea	Sexually tr	Number	2019	9E+07	1.2E+08	7E+07
Incidence	Global	Both	50-69 yea	Sexually tr	Rate	2019	6545.5	8398.62	5100.95
Incidence	Global	Both	50-54 yea	Gonococca	Number	1990	1497454	2596120	862305
Incidence	Global	Both	50-54 yea	Gonococca	Rate	1990	704.382	1221.18	405.617
Incidence	Global	Both	50-54 yea	Gonococca	Number	1991	1519227	2625819	872862
Incidence	Global	Both	50-54 yea	Gonococca	Rate	1991	706.974	1221.93	406.187
Incidence	Global	Both	50-54 yea	Gonococca	Number	1992	1531147	2640085	880323
Incidence	Global	Both	50-54 yea	Gonococca	Rate	1992	707.957	1220.7	407.036
Incidence	Global	Both	50-54 yea	Gonococca	Number	1993	1531377	2645805	882775
Incidence	Global	Both	50-54 yea	Gonococca	Rate	1993	705.359	1218.67	406.61
Incidence	Global	Both	50-54 yea	Gonococca	Number	1994	1529017	2646264	883791
Incidence	Global	Both	50-54 yea	Gonococca	Rate	1994	700.925	1213.09	405.144
Incidence	Global	Both	50-54 yea	Gonococca	Number	1995	1525202	2640271	882643
Incidence	Global	Both	50-54 yea	Gonococca	Rate	1995	696.141	1205.09	402.86
Incidence	Global	Both	50-54 yea	Gonococca	Number	1996	1537667	2656159	891252
Incidence	Global	Both	50-54 yea	Gonococca	Rate	1996	691.534	1194.55	400.822
Incidence	Global	Both	50-54 yea	Gonococca	Number	1997	1580008	2728463	915477
Incidence	Global	Both	50-54 yea	Gonococca	Rate	1997	684.986	1182.88	396.89
Incidence	Global	Both	50-54 yea	Gonococca	Number	1998	1634232	2821382	946479
Incidence	Global	Both	50-54 yea	Gonococca	Rate	1998	680.634	1175.06	394.195
Incidence	Global	Both	50-54 yea	Gonococca	Number	1999	1710845	2952862	983798
Incidence	Global	Both	50-54 yea	Gonococca	Rate	1999	678.364	1170.83	390.084
Incidence	Global	Both	50-54 yea	Gonococca	Number	2000	1775286	3063535	1013947
Incidence	Global	Both	50-54 yea	Gonococca	Rate	2000	676.983	1168.24	386.656
Incidence	Global	Both	50-54 yea	Gonococca	Number	2001	1864346	3221510	1062962
Incidence	Global	Both	50-54 yea	Gonococca	Rate	2001	677.046	1169.91	386.019
Incidence	Global	Both	50-54 yea	Gonococca	Number	2002	1931803	3338625	1103140
Incidence	Global	Both	50-54 yea	Gonococca	Rate	2002	678.159	1172.02	387.257
Incidence	Global	Both	50-54 yea	Gonococca	Number	2003	2004301	3469823	1143047
Incidence	Global	Both	50-54 yea	Gonococca	Rate	2003	679.221	1175.86	387.358
Incidence	Global	Both	50-54 yea	Gonococca	Number	2004	2068712	3582534	1178358
Incidence	Global	Both	50-54 yea	Gonococca	Rate	2004	679.822	1177.3	387.233

Incidence	Global	Both	55-59 yea	Gonococca	Rate	1992	381.151	689.19	204.593
Incidence	Global	Both	55-59 yea	Gonococca	Number	1993	739421	1333734	397641
Incidence	Global	Both	55-59 yea	Gonococca	Rate	1993	380.451	686.241	204.597
Incidence	Global	Both	55-59 yea	Gonococca	Number	1994	749257	1348081	402856
Incidence	Global	Both	55-59 yea	Gonococca	Rate	1994	380.063	683.819	204.35
Incidence	Global	Both	55-59 yea	Gonococca	Number	1995	758322	1363251	408616
Incidence	Global	Both	55-59 yea	Gonococca	Rate	1995	379.567	682.355	204.526
Incidence	Global	Both	55-59 yea	Gonococca	Number	1996	762298	1370425	410036
Incidence	Global	Both	55-59 yea	Gonococca	Rate	1996	377.888	679.349	203.264
Incidence	Global	Both	55-59 yea	Gonococca	Number	1997	761058	1367171	408573
Incidence	Global	Both	55-59 yea	Gonococca	Rate	1997	375.171	673.96	201.41
Incidence	Global	Both	55-59 yea	Gonococca	Number	1998	755414	1356060	405684
Incidence	Global	Both	55-59 yea	Gonococca	Rate	1998	370.996	665.983	199.238
Incidence	Global	Both	55-59 yea	Gonococca	Number	1999	750970	1347275	403523
Incidence	Global	Both	55-59 yea	Gonococca	Rate	1999	366.837	658.122	197.115
Incidence	Global	Both	55-59 yea	Gonococca	Number	2000	748594	1342122	401896
Incidence	Global	Both	55-59 yea	Gonococca	Rate	2000	363.653	651.979	195.234
Incidence	Global	Both	55-59 yea	Gonococca	Number	2001	756930	1356624	406900
Incidence	Global	Both	55-59 yea	Gonococca	Rate	2001	361.599	648.085	194.384
Incidence	Global	Both	55-59 yea	Gonococca	Number	2002	781331	1400431	419175
Incidence	Global	Both	55-59 yea	Gonococca	Rate	2002	358.728	642.972	192.454
Incidence	Global	Both	55-59 yea	Gonococca	Number	2003	811997	1459067	435077
Incidence	Global	Both	55-59 yea	Gonococca	Rate	2003	357.123	641.709	191.351
Incidence	Global	Both	55-59 yea	Gonococca	Number	2004	853080	1535147	456629
Incidence	Global	Both	55-59 yea	Gonococca	Rate	2004	356.185	640.968	190.656
Incidence	Global	Both	55-59 yea	Gonococca	Number	2005	887646	1600802	473850
Incidence	Global	Both	55-59 yea	Gonococca	Rate	2005	355.649	641.386	189.855
Incidence	Global	Both	55-59 yea	Gonococca	Number	2006	933976	1684803	499068
Incidence	Global	Both	55-59 yea	Gonococca	Rate	2006	355.731	641.704	190.084
Incidence	Global	Both	55-59 yea	Gonococca	Number	2007	969881	1751813	517303
Incidence	Global	Both	55-59 yea	Gonococca	Rate	2007	356.666	644.215	190.234
Incidence	Global	Both	55-59 yea	Gonococca	Number	2008	1008482	1823334	537306
Incidence	Global	Both	55-59 yea	Gonococca	Rate	2008	357.612	646.562	190.531
Incidence	Global	Both	55-59 yea	Gonococca	Number	2009	1043590	1890141	557422
Incidence	Global	Both	55-59 yea	Gonococca	Rate	2009	358.479	649.274	191.477
Incidence	Global	Both	55-59 yea	Gonococca	Number	2010	1090090	1967824	582590
Incidence	Global	Both	55-59 yea	Gonococca	Rate	2010	359.29	648.589	192.02
Incidence	Global	Both	55-59 yea	Gonococca	Number	2011	1117195	2012974	598188
Incidence	Global	Both	55-59 yea	Gonococca	Rate	2011	359.234	647.272	192.347
Incidence	Global	Both	55-59 yea	Gonococca	Number	2012	1137861	2045937	610698
Incidence	Global	Both	55-59 yea	Gonococca	Rate	2012	358.271	644.191	192.287
Incidence	Global	Both	55-59 yea	Gonococca	Number	2013	1150343	2062784	618941
Incidence	Global	Both	55-59 yea	Gonococca	Rate	2013	356.788	639.789	191.969
Incidence	Global	Both	55-59 yea	Gonococca	Number	2014	1158616	2071082	625651
Incidence	Global	Both	55-59 yea	Gonococca	Rate	2014	355.25	635.027	191.835
Incidence	Global	Both	55-59 yea	Gonococca	Number	2015	1168465	2086710	632546
Incidence	Global	Both	55-59 yea	Gonococca	Rate	2015	353.964	632.129	191.618
Incidence	Global	Both	55-59 yea	Gonococca	Number	2016	1179189	2111491	639563
Incidence	Global	Both	55-59 yea	Gonococca	Rate	2016	350.942	628.408	190.342
Incidence	Global	Both	55-59 yea	Gonococca	Number	2017	1199656	2153324	650380
Incidence	Global	Both	55-59 yea	Gonococca	Rate	2017	348.059	624.748	188.696
Incidence	Global	Both	55-59 yea	Gonococca	Number	2018	1243335	2246678	670281
Incidence	Global	Both	55-59 yea	Gonococca	Rate	2018	348.381	629.516	187.812
Incidence	Global	Both	55-59 yea	Gonococca	Number	2019	1299878	2340141	697860
Incidence	Global	Both	55-59 yea	Gonococca	Rate	2019	350.36	630.745	188.096
Incidence	Global	Both	60-64 yea	Gonococca	Number	1990	184959	312212	106170
Incidence	Global	Both	60-64 yea	Gonococca	Rate	1990	115.136	194.351	66.0903
Incidence	Global	Both	60-64 yea	Gonococca	Number	1991	188315	318056	108167
Incidence	Global	Both	60-64 yea	Gonococca	Rate	1991	114.835	193.952	65.9605
Incidence	Global	Both	60-64 yea	Gonococca	Number	1992	190158	321065	109391
Incidence	Global	Both	60-64 yea	Gonococca	Rate	1992	114.318	193.015	65.7629
Incidence	Global	Both	60-64 yea	Gonococca	Number	1993	191403	322562	110091
Incidence	Global	Both	60-64 yea	Gonococca	Rate	1993	113.684	191.586	65.3889
Incidence	Global	Both	60-64 yea	Gonococca	Number	1994	191934	322430	109893
Incidence	Global	Both	60-64 yea	Gonococca	Rate	1994	112.879	189.625	64.6293
Incidence	Global	Both	60-64 yea	Gonococca	Number	1995	192945	323629	110264
Incidence	Global	Both	60-64 yea	Gonococca	Rate	1995	112.179	188.159	64.1075
Incidence	Global	Both	60-64 yea	Gonococca	Number	1996	194387	327431	110930
Incidence	Global	Both	60-64 yea	Gonococca	Rate	1996	111.349	187.56	63.5431
Incidence	Global	Both	60-64 yea	Gonococca	Number	1997	195912	330931	111719
Incidence	Global	Both	60-64 yea	Gonococca	Rate	1997	110.129	186.027	62.801
Incidence	Global	Both	60-64 yea	Gonococca	Number	1998	197163	332832	112501
Incidence	Global	Both	60-64 yea	Gonococca	Rate	1998	108.826	183.71	62.0958
Incidence	Global	Both	60-64 yea	Gonococca	Number	1999	198542	334883	113260
Incidence	Global	Both	60-64 yea	Gonococca	Rate	1999	107.824	181.868	61.5089
Incidence	Global	Both	60-64 yea	Gonococca	Number	2000	200692	338867	114545
Incidence	Global	Both	60-64 yea	Gonococca	Rate	2000	107.347	181.254	61.2684
Incidence	Global	Both	60-64 yea	Gonococca	Number	2001	202645	343571	115721
Incidence	Global	Both	60-64 yea	Gonococca	Rate	2001	107.145	181.658	61.1857
Incidence	Global	Both	60-64 yea	Gonococca	Number	2002	203839	346898	116545
Incidence	Global	Both	60-64 yea	Gonococca	Rate	2002	106.972	182.047	61.161
Incidence	Global	Both	60-64 yea	Gonococca	Number	2003	204385	347693	116992
Incidence	Global	Both	60-64 yea	Gonococca	Rate	2003	106.604	181.351	61.0214
Incidence	Global	Both	60-64 yea	Gonococca	Number	2004	205341	348883	117645
Incidence	Global	Both	60-64 yea	Gonococca	Rate	2004	106.224	180.479	60.8583
Incidence	Global	Both	60-64 yea	Gonococca	Number	2005	206533	351005	118430
Incidence	Global	Both	60-64 yea	Gonococca	Rate	2005	105.908	179.992	60.73
Incidence	Global	Both	60-64 yea	Gonococca	Number	2006	210654	357106	120608
Incidence	Global	Both	60-64 yea	Gonococca	Rate	2006	105.926	179.568	60.6465
Incidence	Global	Both	60-64 yea	Gonococca	Number	2007	220011	373072	125533
Incidence	Global	Both	60-64 yea	Gonococca	Rate	2007	105.999	179.743	60.4807
Incidence	Global	Both	60-64 yea	Gonococca	Number	2008	231080	391195	131222
Incidence	Global	Both	60-64 yea	Gonococca	Rate	2008	106.412	180.145	60.4276
Incidence	Global	Both	60-64 yea	Gonococca	Number	2009	245168	414899	139157
Incidence	Global	Both	60-64 yea	Gonococca	Rate	2009	107.02	181.111	60.7445
Incidence	Global	Both	60-64 yea	Gonococca	Number	2010	256070	433276	145381
Incidence	Global	Both	60-64 yea	Gonococca	Rate	2010	107.178	181.347	60.8491
Incidence	Global	Both	60-64 yea	Gonococca	Number	2011	269373	455789	152749
Incidence	Global	Both	60-64 yea	Gonococca	Rate	2011	107.076	181.176	60.7177
Incidence	Global	Both	60-64 yea	Gonococca	Number	2012	278991	471747	157927
Incidence	Global	Both	60-64 yea	Gonococca	Rate	2012	107.022	180.964	60.5814
Incidence	Global	Both	60-64 yea	Gonococca	Number	2013	289313	488120	164086
Incidence	Global	Both	60-64 yea	Gonococca	Rate	2013	106.983	180.498	60.6762
Incidence	Global	Both	60-64 yea	Gonococca	Number	2014	298649	501989	169779
Incidence	Global	Both	60-64 yea	Gonococca	Rate	2014	106.946	179.762	60.7976
Incidence	Global	Both	60-64 yea	Gonococca	Number	2015	311991	523223	177624
Incidence	Global	Both	60-64 yea	Gonococca	Rate	2015	107.202	179.782	61.0324
Incidence	Global	Both	60-64 yea	Gonococca	Number	2016	319258	535685	181015
Incidence	Global	Both	60-64 yea	Gonococca	Rate	2016	107.04	179.603	60.6903
Incidence	Global	Both	60-64 yea	Gonococca	Number	2017	324089	544450	183091
Incidence	Global	Both	60-64 yea	Gonococca	Rate	2017	106.427	178.791	60.1249
Incidence	Global	Both	60-64 yea	Gonococca	Number	2018	326592	549463	184902
Incidence	Global	Both	60-64 yea	Gonococca	Rate	2018	105.675	177.789	59.8287
Incidence	Global	Both	60-64 yea	Gonococca	Number	2019	327982	550033	184949
Incidence	Global	Both	60-64 yea	Gonococca	Rate	2019	104.943	175.991	59.1768
Incidence	Global	Both	65-69 yea	Gonococca	Number	1990	54357.4	88699.5	29925.3
Incidence	Global	Both	65-69 yea	Gonococca	Rate	1990	44.0182	71.8281	24.2333
Incidence	Global	Both	65-69 yea	Gonococca	Number	1991	55989.6	91456.9	30727.5
Incidence	Global	Both	65-69 yea	Gonococca	Rate	1991	43.9918	71.859	24.143
Incidence	Global	Both	65-69 yea	Gonococca	Number	1992	57689.5	94297.2	31568.4
Incidence	Global	Both	65-69 yea	Gonococca	Rate	1992	44.0018	71.9238	24.0783
Incidence	Global	Both	65-69 yea	Gonococca	Number	1993	59360.5	97036.4	32419
Incidence	Global	Both	65-69 yea	Gonococca	Rate	1993	43.9902	71.9106	24.0247
Incidence	Global	Both	65-69 yea	Gonococca	Number	1994	61048.7	99889.2	33216.1
Incidence	Global	Both	65-69 yea	Gonococca	Rate	1994	44.0009	71.9953	23.9406
Incidence	Global	Both	65-69 yea	Gonococca	Number	1995	62510	102629	33893.4
Incidence	Global	Both	65-69 yea	Gonococca	Rate	1995	44.001	72.2408	23.8577
Incidence	Global	Both	65-69 yea	Gonococca	Number	1996	63608	104460	34485.1
Incidence	Global	Both	65-69 yea	Gonococca	Rate	1996	43.842	71.9995	23.7689
Incidence	Global	Both	65-69 yea	Gonococca	Number	1997	64001.5	105158	34736.9
Incidence	Global	Both	65-69 yea	Gonococca	Rate	1997	43.4536	71.3971	23.5845
Incidence	Global	Both	65-69 yea	Gonococca	Number	1998	64165.8	105503	34889.3
Incidence	Global	Both	65-69 yea	Gonococca	Rate	1998	42.9854	70.6775	23.3727
Incidence	Global	Both	65-69 yea	Gonococca	Number	1999	64212.9	105593	35092.1
Incidence	Global	Both	65-69 yea	Gonococca	Rate	1999	42.5095	69.9033	23.2313
Incidence	Global	Both	65-69 yea	Gonococca	Number	2000	64698.8	106440	35464.5
Incidence	Global	Both	65-69 yea	Gonococca	Rate	2000	42.2376	69.4873	23.1524
Incidence	Global	Both	65-69 yea	Gonococca	Number	2001	65796.5	108404	35942.6
Incidence	Global	Both	65-69 yea	Gonococca	Rate	2001	42.2016	69.5296	23.0534
Incidence	Global	Both	65-69 yea	Gonococca	Number	2002	67313.6	111156	36665.6
Incidence	Global	Both	65-69 yea	Gonococca	Rate	2002	42.2505	69.7689	23.0138
Incidence	Global	Both	65-69 yea	Gonococca	Number	2003	68829.3	113849	37434.3
Incidence	Global	Both	65-69 yea	Gonococca	Rate	2003	42.3128	69.9884	23.0128
Incidence	Global	Both	65-69 yea	Gonococca	Number	2004	70201.2	116158	38080.6
Incidence	Global	Both	65-69 yea	Gonococca	Rate	2004	42.3691	70.106	22.9831
Incidence	Global	Both	65-69 yea	Gonococca	Number	2005	71406.1	118073	38627.9
Incidence	Global	Both	65-69 yea	Gonococca	Rate	2005	42.3557	70.0371	22.9128
Incidence	Global	Both	65-69 yea	Gonococca	Number	2006	72310.1	119606	39059.9
Incidence	Global	Both	65-69 yea	Gonococca	Rate	2006	42.2914	69.9528	22.8447
Incidence	Global	Both	65-69 yea	Gonococca	Number	2007	73060.8	120842	39506.4
Incidence	Global	Both	65-69 yea	Gonococca	Rate	2007	42.2912	69.9492	22.8683
Incidence	Global	Both	65-69 yea	Gonococca	Number	2008	73702.9	122528	39976.4
Incidence	Global	Both	65-69 yea	Gonococca	Rate	2008	42.2682	70.2692	22.9262
Incidence	Global	Both	65-69 yea	Gonococca	Number	2009	74520.5	124395	40761.6
Incidence	Global	Both	65-69 yea	Gonococca	Rate	2009	42.2588	70.5415	23.115
Incidence	Global	Both	65-69 yea	Gonococca	Number	2010	75299.5	125940	41325.7
Incidence	Global	Both	65-69 yea	Gonococca	Rate	2010	42.2224	70.6179	23.1724
Incidence	Global	Both	65-69 yea	Gonococca	Number	2011	76818.3	128539	42020.9
Incidence	Global	Both	65-69 yea	Gonococca	Rate	2011	42.1565	70.5398	23.0603
Incidence	Global	Both	65-69 yea	Gonococca	Number	2012	79964.3	133720	43735.1
Incidence	Global	Both	65-69 yea	Gonococca	Rate	2012	41.9453	70.1427	22.9413
Incidence	Global	Both	65-69 yea	Gonococca	Number	2013	83540.2	139563	45645.8
Incidence	Global	Both	65-69 yea	Gonococca	Rate	2013	41.8007	69.8329	22.8396
Incidence	Global	Both	65-69 yea	Gonococca	Number	2014	88257	147550	48037.7
Incidence	Global	Both	65-69 yea	Gonococca	Rate	2014	41.7814	69.8511	22.7414
Incidence	Global	Both	65-69 yea	Gonococca	Number	2015	92042.1	153864	49991.6
Incidence	Global	Both	65-69 yea	Gonococca	Rate	2015	41.7349	69.7668	22.6678
Incidence	Global	Both	65-69 yea	Gonococca	Number	2016	96880.4	161525	52668
Incidence	Global	Both	65-69 yea	Gonococca	Rate	2016	41.6684	69.4723	22.6526
Incidence	Global	Both	65-69 yea	Gonococca	Number	2017	100419	166997	54491.2
Incidence	Global	Both	65-69 yea	Gonococca	Rate	2017	41.6454	69.2562	22.5983
Incidence	Global	Both	65-69 yea	Gonococca	Number	2018	104148	173759	56057.1
Incidence	Global	Both	65-69 yea	Gonococca	Rate	2018	41.6103	69.422	22.3965
Incidence	Global	Both	65-69 yea	Gonococca	Number	2019	107018	177308	58007
Incidence	Global	Both	65-69 yea	Gonococca	Rate	2019	41.3864	68.5688	22.4326
Incidence	Global	Both	50-54 yea	Genital he	Number	1990	1268350	1998409	675054
Incidence	Global	Both	50-54 yea	Genital he	Rate	1990	596.615	940.025	317.536
Incidence	Global	Both	50-54 yea	Genital he	Number	1991	1287052	2036623	685158
Incidence	Global	Both	50-54 yea	Genital he	Rate	1991	598.931	947.745	318.839
Incidence	Global	Both	50-54 yea	Genital he	Number	1992	1299546	2058697	691228
Incidence	Global	Both	50-54 yea	Genital he	Rate	1992	600.872	951.881	319.603
Incidence	Global	Both	50-54 yea	Genital he	Number	1993	1308403	2074518	694797
Incidence	Global	Both	50-54 yea	Genital he	Rate	1993	602.656	955.532	320.026
Incidence	Global	Both	50-54 yea	Genital he	Number	1994	1318026	2090901	698469
Incidence	Global	Both	50-54 yea	Genital he	Rate	1994	604.204	958.502	320.189
Incidence	Global	Both	50-54 yea	Genital he	Number	1995	1326678	2105304	703452
Incidence	Global	Both	50-54 yea	Genital he	Rate	1995	605.53	960.914	321.073
Incidence	Global	Both	50-54 yea	Genital he	Number	1996	1346881	2135989	713175
Incidence	Global	Both	50-54 yea	Genital he	Rate	1996	605.732	960.617	320.736
Incidence	Global	Both	50-54 yea	Genital he	Number	1997	1392922	2206518	739243
Incidence	Global	Both	50-54 yea	Genital he	Rate	1997	603.878	956.599	320.486
Incidence	Global	Both	50-54 yea	Genital he	Number	1998	1444067	2285615	767306
Incidence	Global	Both	50-54 yea	Genital he	Rate	1998	601.433	951.926	319.572
Incidence	Global	Both	50-54 yea	Genital he	Number	1999	1511260	2388455	802748
Incidence	Global	Both	50-54 yea	Genital he	Rate	1999	599.227	947.042	318.296
Incidence	Global	Both	50-54 yea	Genital he	Number	2000	1568300	2482571	832204
Incidence	Global	Both	50-54 yea	Genital he	Rate	2000	598.052	946.698	317.351
Incidence	Global	Both	50-54 yea	Genital he	Number	2001	1647924	2606884	875238
Incidence	Global	Both	50-54 yea	Genital he	Rate	2001	598.451	946.702	317.847
Incidence	Global	Both	50-54 yea	Genital he	Number	2002	1708582	2701743	906885
Incidence	Global	Both	50-54 yea	Genital he	Rate	2002	599.797	948.447	318.362
Incidence	Global	Both	50-54 yea	Genital he	Number	2003	1774406	2805041	939655
Incidence	Global	Both	50-54 yea	Genital he	Rate	2003	601.314	950.577	318.432
Incidence	Global	Both	50-54 yea	Genital he	Number	2004	1833843	2898475	968064
Incidence	Global	Both	50-54 yea	Genital he	Rate	2004	602.639	952.499	318.126
Incidence	Global	Both	50-54 yea	Genital he	Number	2005	1910976	3018564	1006537
Incidence	Global	Both	50-54 yea	Genital he	Rate	2005	603.328	953.013	317.781
Incidence	Global	Both	50-54 yea	Genital he	Number	2006	1955685	3086408	1031952
Incidence	Global	Both	50-54 yea	Genital he	Rate	2006	602.917	951.506	318.14
Incidence	Global	Both	50-54 yea	Genital he	Number	2007	1992477	3142514	1054517
Incidence	Global	Both	50-54 yea	Genital he	Rate	2007	601.966	949.415	318.59
Incidence	Global	Both	50-54 yea	Genital he	Number	2008	2016706	3182207	1070493
Incidence	Global	Both	50-54 yea	Genital he	Rate	2008	600.657	947.79	318.836
Incidence	Global	Both	50-54 yea	Genital he	Number	2009	2034354	3213907	1082028
Incidence	Global	Both	50-54 yea	Genital he	Rate	2009	599.468	947.05	318.844
Incidence	Global	Both	50-54 yea	Genital he	Number	2010	2055386	3251193	1093962
Incidence	Global	Both	50-54 yea	Genital he	Rate	2010	598.719	947.048	318.663
Incidence	Global	Both	50-54 yea	Genital he	Number	2011	2091516	3307835	1118632
Incidence	Global	Both	50-54 yea	Genital he	Rate	2011	598.735	946.929	320.229
Incidence	Global	Both	50-54 yea	Genital he	Number	2012	2147626	3388395	1152003
Incidence	Global	Both	50-54 yea	Genital he	Rate	2012	599.488	945.836	321.57
Incidence	Global	Both	50-54 yea	Genital he	Number	2013	2226938	3504475	1196019
Incidence	Global	Both	50-54 yea	Genital he	Rate	2013	600.607	945.16	322.567
Incidence	Global	Both	50-54 yea	Genital he	Number	2014	2317693	3646701	1245296
Incidence	Global	Both	50-54 yea	Genital he	Rate	2014	601.643	946.636	323.262
Incidence	Global	Both	50-54 yea	Genital he	Number	2015	2406275	3789079	1289405
Incidence	Global	Both	50-54 yea	Genital he	Rate	2015	602.221	948.297	322.701
Incidence	Global	Both	50-54 yea	Genital he	Number	2016	2482718	3938981	1324965
Incidence	Global	Both	50-54 yea	Genital he	Rate	2016	602.699	956.218	321.646
Incidence	Global	Both	50-54 yea	Genital he	Number	2017	2548807	4063280	1347636
Incidence	Global	Both	50-54 yea	Genital he	Rate	2017	603.198	961.612	318.93
Incidence	Global	Both	50-54 yea	Genital he	Number	2018	2601243	4153663	1377777
Incidence	Global	Both	50-54 yea	Genital he	Rate	2018	604.137	964.686	319.988
Incidence	Global	Both	50-54 yea	Genital he	Number	2019	2646654	4224581	1398399
Incidence	Global	Both	50-54 yea	Genital he	Rate	2019	605.896	967.129	320.134
Incidence	Global	Both	55-59 yea	Genital he	Number	1990	674555	975728	418028
Incidence	Global	Both	55-59 yea	Genital he	Rate	1990	363.807	526.239	225.455
Incidence	Global	Both	55-59 yea	Genital he	Number	1991	684376	989087	423710
Incidence	Global	Both	55-59 yea	Genital he	Rate	1991	364.114	526.231	225.43
Incidence	Global	Both	55-59 yea	Genital he	Number	1992	696472	1006189	431529
Incidence	Global	Both	55-59 yea	Genital he	Rate	1992	364.244	526.221	225.683
Incidence	Global	Both	55-59 yea	Genital he	Number	1993	708021	1022891	439814
Incidence	Global	Both	55-59 yea	Genital he	Rate	1993	364.295	526.304	226.296
Incidence	Global	Both	55-59 yea	Genital he	Number	1994	718141	1038561	445987
Incidence	Global	Both	55-59 yea	Genital he	Rate	1994	364.28	526.814	226.229
Incidence	Global	Both	55-59 yea	Genital he	Number	1995	727946	1051929	452072
Incidence	Global	Both	55-59 yea	Genital he	Rate	1995	364.363	526.528	226.278
Incidence	Global	Both	55-59 yea	Genital he	Number	1996	734917	1062908	456559
Incidence	Global	Both	55-59 yea	Genital he	Rate	1996	364.314	526.907	226.326
Incidence	Global	Both	55-59 yea	Genital he	Number	1997	738266	1068721	458912
Incidence	Global	Both	55-59 yea	Genital he	Rate	1997	363.935	526.836	226.225
Incidence	Global	Both	55-59 yea	Genital he	Number	1998	740247	1072383	460438
Incidence	Global	Both	55-59 yea	Genital he	Rate	1998	363.547	526.665	226.128
Incidence	Global	Both	55-59 yea	Genital he	Number	1999	743699	1078083	462785
Incidence	Global	Both	55-59 yea	Genital he	Rate	1999	363.285	526.626	226.063
Incidence	Global	Both	55-59 yea	Genital he	Number	2000	747709	1084368	465304
Incidence	Global	Both	55-59 yea	Genital he	Rate	2000	363.224	526.766	226.036
Incidence	Global	Both	55-59 yea	Genital he	Number	2001	760623	1104090	473308
Incidence	Global	Both	55-59 yea	Genital he	Rate	2001	363.363	527.444	226.108
Incidence	Global	Both	55-59 yea	Genital he	Number	2002	791793	1150006	492730
Incidence	Global	Both	55-59 yea	Genital he	Rate	2002	363.531	527.996	226.224
Incidence	Global	Both	55-59 yea	Genital he	Number	2003	827030	1201828	514024
Incidence	Global	Both	55-59 yea	Genital he	Rate	2003	363.734	528.574	226.072
Incidence	Global	Both	55-59 yea	Genital he	Number	2004	871439	1266941	541127
Incidence	Global	Both	55-59 yea	Genital he	Rate	2004	363.851	528.984	225.936
Incidence	Global	Both	55-59 yea	Genital he	Number	2005	907999	1321446	563627
Incidence	Global	Both	55-59 yea	Genital he	Rate	2005	363.804	529.458	225.826
Incidence	Global	Both	55-59 yea	Genital he	Number	2006	955362	1388363	593600
Incidence	Global	Both	55-59 yea	Genital he	Rate	2006	363.876	528.797	226.089
Incidence	Global	Both	55-59 yea	Genital he	Number	2007	990293	1437046	614686
Incidence	Global	Both	55-59 yea	Genital he	Rate	2007	364.172	528.462	226.046
Incidence	Global	Both	55-59 yea	Genital he	Number	2008	1027842	1489423	638861
Incidence	Global	Both	55-59 yea	Genital he	Rate	2008	364.477	528.156	226.543
Incidence	Global	Both	55-59 yea	Genital he	Number	2009	1061803	1536620	660508
Incidence	Global	Both	55-59 yea	Genital he	Rate	2009	364.735	527.838	226.888
Incidence	Global	Both	55-59 yea	Genital he	Number	2010	1107007	1602743	689853
Incidence	Global	Both	55-59 yea	Genital he	Rate	2010	364.866	528.259	227.373
Incidence	Global	Both	55-59 yea	Genital he	Number	2011	1134147	1641251	707474
Incidence	Global	Both	55-59 yea	Genital he	Rate	2011	364.685	527.745	227.488
Incidence	Global	Both	55-59 yea	Genital he	Number	2012	1156913	1675324	722945
Incidence	Global	Both	55-59 yea	Genital he	Rate	2012	364.27	527.499	227.629
Incidence	Global	Both	55-59 yea	Genital he	Number	2013	1172694	1700860	731154
Incidence	Global	Both	55-59 yea	Genital he	Rate	2013	363.72	527.535	226.773
Incidence	Global	Both	55-59 yea	Genital he	Number	2014	1184661	1721013	737278
Incidence	Global	Both	55-59 yea	Genital he	Rate	2014	363.236	527.69	226.061
Incidence	Global	Both	55-59 yea	Genital he	Number	2015	1198055	1742898	745630
Incidence	Global	Both	55-59 yea	Genital he	Rate	2015	362.928	527.977	225.874
Incidence	Global	Both	55-59 yea	Genital he	Number	2016	1220495	1779656	758687
Incidence	Global	Both	55-59 yea	Genital he	Rate	2016	363.235	529.649	225.795
Incidence	Global	Both	55-59 yea	Genital he	Number	2017	1253628	1829961	777007
Incidence	Global	Both	55-59 yea	Genital he	Rate	2017	363.718	530.93	225.435
Incidence	Global	Both	55-59 yea	Genital he	Number	2018	1299103	1893403	805864
Incidence	Global	Both	55-59 yea	Genital he	Rate	2018	364.007	530.529	225.802
Incidence	Global	Both	55-59 yea	Genital he	Number	2019	1352487	1969208	838854
Incidence	Global	Both	55-59 yea	Genital he	Rate	2019	364.54	530.766	226.098
Incidence	Global	Both	60-64 yea	Genital he	Number	1990	312889	536880	154940
Incidence	Global	Both	60-64 yea	Genital he	Rate	1990	194.773	334.207	96.4497
Incidence	Global	Both	60-64 yea	Genital he	Number	1991	318409	545338	157802
Incidence	Global	Both	60-64 yea	Genital he	Rate	1991	194.167	332.55	96.2286
Incidence	Global	Both	60-64 yea	Genital he	Number	1992	322189	549920	159039
Incidence	Global	Both	60-64 yea	Genital he	Rate	1992	193.691	330.597	95.6098
Incidence	Global	Both	60-64 yea	Genital he	Number	1993	325520	556076	160975
Incidence	Global	Both	60-64 yea	Genital he	Rate	1993	193.343	330.282	95.6113
Incidence	Global	Both	60-64 yea	Genital he	Number	1994	328398	561521	162718
Incidence	Global	Both	60-64 yea	Genital he	Rate	1994	193.134	330.237	95.6966
Incidence	Global	Both	60-64 yea	Genital he	Number	1995	331991	568272	164850
Incidence	Global	Both	60-64 yea	Genital he	Rate	1995	193.02	330.394	95.844
Incidence	Global	Both	60-64 yea	Genital he	Number	1996	336982	576448	167147
Incidence	Global	Both	60-64 yea	Genital he	Rate	1996	193.031	330.202	95.7451
Incidence	Global	Both	60-64 yea	Genital he	Number	1997	343629	587572	170330
Incidence	Global	Both	60-64 yea	Genital he	Rate	1997	193.165	330.293	95.7478
Incidence	Global	Both	60-64 yea	Genital he	Number	1998	350306	599080	173512
Incidence	Global	Both	60-64 yea	Genital he	Rate	1998	193.354	330.668	95.7714
Incidence	Global	Both	60-64 yea	Genital he	Number	1999	356284	609762	176283
Incidence	Global	Both	60-64 yea	Genital he	Rate	1999	193.49	331.148	95.7355
Incidence	Global	Both	60-64 yea	Genital he	Number	2000	361837	621688	179819
Incidence	Global	Both	60-64 yea	Genital he	Rate	2000	193.541	332.531	96.1822
Incidence	Global	Both	60-64 yea	Genital he	Number	2001	366010	628454	181463
Incidence	Global	Both	60-64 yea	Genital he	Rate	2001	193.522	332.285	95.9458
Incidence	Global	Both	60-64 yea	Genital he	Number	2002	368541	632531	182165
Incidence	Global	Both	60-64 yea	Genital he	Rate	2002	193.405	331.943	95.5977
Incidence	Global	Both	60-64 yea	Genital he	Number	2003	370642	635922	182638
Incidence	Global	Both	60-64 yea	Genital he	Rate	2003	193.321	331.687	95.2611
Incidence	Global	Both	60-64 yea	Genital he	Number	2004	373665	640175	183996
Incidence	Global	Both	60-64 yea	Genital he	Rate	2004	193.3	331.167	95.1825
Incidence	Global	Both	60-64 yea	Genital he	Number	2005	377009	645403	185532
Incidence	Global	Both	60-64 yea	Genital he	Rate	2005	193.327	330.957	95.1393
Incidence	Global	Both	60-64 yea	Genital he	Number	2006	384922	659249	189616
Incidence	Global	Both	60-64 yea	Genital he	Rate	2006	193.555	331.498	95.3466
Incidence	Global	Both	60-64 yea	Genital he	Number	2007	403039	690342	199427
Incidence	Global	Both	60-64 yea	Genital he	Rate	2007	194.181	332.601	96.0824
Incidence	Global	Both	60-64 yea	Genital he	Number	2008	423211	725349	210348
Incidence	Global	Both	60-64 yea	Genital he	Rate	2008	194.888	334.023	96.865
Incidence	Global	Both	60-64 yea	Genital he	Number	2009	447700	768143	222485
Incidence	Global	Both	60-64 yea	Genital he	Rate	2009	195.428	335.307	97.1184
Incidence	Global	Both	60-64 yea	Genital he	Number	2010	467360	802585	232287
Incidence	Global	Both	60-64 yea	Genital he	Rate	2010	195.613	335.921	97.2236
Incidence	Global	Both	60-64 yea	Genital he	Number	2011	491452	844497	244302
Incidence	Global	Both	60-64 yea	Genital he	Rate	2011	195.352	335.688	97.1101
Incidence	Global	Both	60-64 yea	Genital he	Number	2012	507648	873258	252420
Incidence	Global	Both	60-64 yea	Genital he	Rate	2012	194.736	334.985	96.8294
Incidence	Global	Both	60-64 yea	Genital he	Number	2013	524558	901116	260308
Incidence	Global	Both	60-64 yea	Genital he	Rate	2013	193.972	333.216	96.2573
Incidence	Global	Both	60-64 yea	Genital he	Number	2014	539647	926178	266733
Incidence	Global	Both	60-64 yea	Genital he	Rate	2014	193.247	331.664	95.5169
Incidence	Global	Both	60-64 yea	Genital he	Number	2015	560970	962491	276180
Incidence	Global	Both	60-64 yea	Genital he	Rate	2015	192.752	330.717	94.8966
Incidence	Global	Both	60-64 yea	Genital he	Number	2016	575101	986827	282851
Incidence	Global	Both	60-64 yea	Genital he	Rate	2016	192.818	330.861	94.8338
Incidence	Global	Both	60-64 yea	Genital he	Number	2017	587619	1009032	288516
Incidence	Global	Both	60-64 yea	Genital he	Rate	2017	192.967	331.353	94.745
Incidence	Global	Both	60-64 yea	Genital he	Number	2018	595786	1025651	292708
Incidence	Global	Both	60-64 yea	Genital he	Rate	2018	192.778	331.869	94.7112
Incidence	Global	Both	60-64 yea	Genital he	Number	2019	601852	1038678	295446
Incidence	Global	Both	60-64 yea	Genital he	Rate	2019	192.571	332.339	94.5321
Incidence	Global	Both	65-69 yea	Genital he	Number	1990	108243	170467	61672.7
Incidence	Global	Both	65-69 yea	Genital he	Rate	1990	87.6546	138.043	49.942
Incidence	Global	Both	65-69 yea	Genital he	Number	1991	111137	174732	63570.5
Incidence	Global	Both	65-69 yea	Genital he	Rate	1991	87.3218	137.29	49.9483
Incidence	Global	Both	65-69 yea	Genital he	Number	1992	114113	179322	65501.5
Incidence	Global	Both	65-69 yea	Genital he	Rate	1992	87.0377	136.775	49.9603
Incidence	Global	Both	65-69 yea	Genital he	Number	1993	117150	184174	67433.1
Incidence	Global	Both	65-69 yea	Genital he	Rate	1993	86.8162	136.485	49.9725
Incidence	Global	Both	65-69 yea	Genital he	Number	1994	120240	189205	69377.7
Incidence	Global	Both	65-69 yea	Genital he	Rate	1994	86.6633	136.369	50.0041
Incidence	Global	Both	65-69 yea	Genital he	Number	1995	123006	193779	71132.7
Incidence	Global	Both	65-69 yea	Genital he	Rate	1995	86.5841	136.402	50.0706
Incidence	Global	Both	65-69 yea	Genital he	Number	1996	125644	198002	72525.6
Incidence	Global	Both	65-69 yea	Genital he	Rate	1996	86.6007	136.473	49.9885
Incidence	Global	Both	65-69 yea	Genital he	Number	1997	127679	201287	73561.5
Incidence	Global	Both	65-69 yea	Genital he	Rate	1997	86.6873	136.663	49.9444
Incidence	Global	Both	65-69 yea	Genital he	Number	1998	129575	203805	74568.5
Incidence	Global	Both	65-69 yea	Genital he	Rate	1998	86.8034	136.531	49.9542
Incidence	Global	Both	65-69 yea	Genital he	Number	1999	131300	205981	75596.2
Incidence	Global	Both	65-69 yea	Genital he	Rate	1999	86.9214	136.361	50.0453
Incidence	Global	Both	65-69 yea	Genital he	Number	2000	133236	208525	76782.6
Incidence	Global	Both	65-69 yea	Genital he	Rate	2000	86.9812	136.132	50.1263
Incidence	Global	Both	65-69 yea	Genital he	Number	2001	135615	212728	78206
Incidence	Global	Both	65-69 yea	Genital he	Rate	2001	86.9826	136.443	50.161
Incidence	Global	Both	65-69 yea	Genital he	Number	2002	138557	217870	79950.9
Incidence	Global	Both	65-69 yea	Genital he	Rate	2002	86.9674	136.75	50.1825
Incidence	Global	Both	65-69 yea	Genital he	Number	2003	141433	222531	81649.5
Incidence	Global	Both	65-69 yea	Genital he	Rate	2003	86.9461	136.801	50.194
Incidence	Global	Both	65-69 yea	Genital he	Number	2004	144025	226588	83168.9
Incidence	Global	Both	65-69 yea	Genital he	Rate	2004	86.9246	136.754	50.1956
Incidence	Global	Both	65-69 yea	Genital he	Number	2005	146557	230600	84639.2
Incidence	Global	Both	65-69 yea	Genital he	Rate	2005	86.9325	136.784	50.2051
Incidence	Global	Both	65-69 yea	Genital he	Number	2006	148831	234139	85851.3
Incidence	Global	Both	65-69 yea	Genital he	Rate	2006	87.0458	136.939	50.2112
Incidence	Global	Both	65-69 yea	Genital he	Number	2007	150711	237119	86795.6
Incidence	Global	Both	65-69 yea	Genital he	Rate	2007	87.2389	137.256	50.2415
Incidence	Global	Both	65-69 yea	Genital he	Number	2008	152542	240152	87688.6
Incidence	Global	Both	65-69 yea	Genital he	Rate	2008	87.4821	137.726	50.2889
Incidence	Global	Both	65-69 yea	Genital he	Number	2009	154661	243093	88746.1
Incidence	Global	Both	65-69 yea	Genital he	Rate	2009	87.7045	137.852	50.3259
Incidence	Global	Both	65-69 yea	Genital he	Number	2010	156586	245332	89730.1
Incidence	Global	Both	65-69 yea	Genital he	Rate	2010	87.8017	137.564	50.314
Incidence	Global	Both	65-69 yea	Genital he	Number	2011	159829	250503	91823.2
Incidence	Global	Both	65-69 yea	Genital he	Rate	2011	87.7115	137.471	50.3909
Incidence	Global	Both	65-69 yea	Genital he	Number	2012	166918	261808	96174.3
Incidence	Global	Both	65-69 yea	Genital he	Rate	2012	87.5571	137.332	50.4482
Incidence	Global	Both	65-69 yea	Genital he	Number	2013	174567	274065	100846
Incidence	Global	Both	65-69 yea	Genital he	Rate	2013	87.3477	137.133	50.4599
Incidence	Global	Both	65-69 yea	Genital he	Number	2014	184045	289129	106552
Incidence	Global	Both	65-69 yea	Genital he	Rate	2014	87.128	136.876	50.4426
Incidence	Global	Both	65-69 yea	Genital he	Number	2015	191803	301438	111054
Incidence	Global	Both	65-69 yea	Genital he	Rate	2015	86.9699	136.682	50.3553
Incidence	Global	Both	65-69 yea	Genital he	Number	2016	202354	319113	117009
Incidence	Global	Both	65-69 yea	Genital he	Rate	2016	87.0328	137.251	50.3256
Incidence	Global	Both	65-69 yea	Genital he	Number	2017	209969	332514	120812
Incidence	Global	Both	65-69 yea	Genital he	Rate	2017	87.0773	137.899	50.1028
Incidence	Global	Both	65-69 yea	Genital he	Number	2018	217612	343945	125072
Incidence	Global	Both	65-69 yea	Genital he	Rate	2018	86.9424	137.416	49.9701
Incidence	Global	Both	65-69 yea	Genital he	Number	2019	224342	354171	128693
Incidence	Global	Both	65-69 yea	Genital he	Rate	2019	86.7579	136.966	49.7685
Incidence	Global	Both	50-69 yea	Genital he	Number	1990	2364037	3408002	1482067
Incidence	Global	Both	50-69 yea	Genital he	Rate	1990	346.563	499.606	217.268
Incidence	Global	Both	50-69 yea	Genital he	Number	1991	2400974	3460669	1504552
Incidence	Global	Both	50-69 yea	Genital he	Rate	1991	345.908	498.578	216.761
Incidence	Global	Both	50-69 yea	Genital he	Number	1992	2432319	3497251	1525254
Incidence	Global	Both	50-69 yea	Genital he	Rate	1992	345.041	496.109	216.368
Incidence	Global	Both	50-69 yea	Genital he	Number	1993	2459094	3526268	1541423
Incidence	Global	Both	50-69 yea	Genital he	Rate	1993	344.043	493.347	215.655
Incidence	Global	Both	50-69 yea	Genital he	Number	1994	2484806	3556557	1554059
Incidence	Global	Both	50-69 yea	Genital he	Rate	1994	343.176	491.195	214.631
Incidence	Global	Both	50-69 yea	Genital he	Number	1995	2509621	3593680	1565889
Incidence	Global	Both	50-69 yea	Genital he	Rate	1995	342.403	490.308	213.644
Incidence	Global	Both	50-69 yea	Genital he	Number	1996	2544423	3641560	1597010
Incidence	Global	Both	50-69 yea	Genital he	Rate	1996	342.111	489.627	214.727
Incidence	Global	Both	50-69 yea	Genital he	Number	1997	2602496	3733971	1633000
Incidence	Global	Both	50-69 yea	Genital he	Rate	1997	343.02	492.154	215.237
Incidence	Global	Both	50-69 yea	Genital he	Number	1998	2664194	3836448	1668432
Incidence	Global	Both	50-69 yea	Genital he	Rate	1998	344.136	495.557	215.513
Incidence	Global	Both	50-69 yea	Genital he	Number	1999	2742543	3942730	1715388
Incidence	Global	Both	50-69 yea	Genital he	Rate	1999	346.234	497.752	216.56
Incidence	Global	Both	50-69 yea	Genital he	Number	2000	2811083	4042675	1753131
Incidence	Global	Both	50-69 yea	Genital he	Rate	2000	347.81	500.193	216.912
Incidence	Global	Both	50-69 yea	Genital he	Number	2001	2910171	4195392	1816890
Incidence	Global	Both	50-69 yea	Genital he	Rate	2001	350.735	505.631	218.973
Incidence	Global	Both	50-69 yea	Genital he	Number	2002	3007472	4342872	1878490
Incidence	Global	Both	50-69 yea	Genital he	Rate	2002	352.766	509.404	220.34
Incidence	Global	Both	50-69 yea	Genital he	Number	2003	3113511	4498990	1945707
Incidence	Global	Both	50-69 yea	Genital he	Rate	2003	355.078	513.085	221.897
Incidence	Global	Both	50-69 yea	Genital he	Number	2004	3222972	4661470	2015042
Incidence	Global	Both	50-69 yea	Genital he	Rate	2004	356.995	516.332	223.198
Incidence	Global	Both	50-69 yea	Genital he	Number	2005	3342541	4839136	2092034
Incidence	Global	Both	50-69 yea	Genital he	Rate	2005	359.443	520.381	224.969
Incidence	Global	Both	50-69 yea	Genital he	Number	2006	3444801	4986363	2160860
Incidence	Global	Both	50-69 yea	Genital he	Rate	2006	360.044	521.165	225.849
Incidence	Global	Both	50-69 yea	Genital he	Number	2007	3536520	5116490	2222795
Incidence	Global	Both	50-69 yea	Genital he	Rate	2007	359.68	520.371	226.068
Incidence	Global	Both	50-69 yea	Genital he	Number	2008	3620301	5247747	2278897
Incidence	Global	Both	50-69 yea	Genital he	Rate	2008	358.701	519.949	225.794
Incidence	Global	Both	50-69 yea	Genital he	Number	2009	3698517	5358641	2317423
Incidence	Global	Both	50-69 yea	Genital he	Rate	2009	357.032	517.291	223.71
Incidence	Global	Both	50-69 yea	Genital he	Number	2010	3786339	5480483	2360154
Incidence	Global	Both	50-69 yea	Genital he	Rate	2010	355.873	515.103	221.827
Incidence	Global	Both	50-69 yea	Genital he	Number	2011	3876945	5612694	2424205
Incidence	Global	Both	50-69 yea	Genital he	Rate	2011	354.347	512.992	221.569
Incidence	Global	Both	50-69 yea	Genital he	Number	2012	3979105	5752822	2484662
Incidence	Global	Both	50-69 yea	Genital he	Rate	2012	353.018	510.379	220.434
Incidence	Global	Both	50-69 yea	Genital he	Number	2013	4098757	5928330	2555996
Incidence	Global	Both	50-69 yea	Genital he	Rate	2013	352.284	509.534	219.685
Incidence	Global	Both	50-69 yea	Genital he	Number	2014	4226046	6117720	2640284
Incidence	Global	Both	50-69 yea	Genital he	Rate	2014	351.627	509.023	219.684
Incidence	Global	Both	50-69 yea	Genital he	Number	2015	4357104	6301391	2723972
Incidence	Global	Both	50-69 yea	Genital he	Rate	2015	351.026	507.666	219.454
Incidence	Global	Both	50-69 yea	Genital he	Number	2016	4480668	6501009	2792003
Incidence	Global	Both	50-69 yea	Genital he	Rate	2016	350.407	508.407	218.347
Incidence	Global	Both	50-69 yea	Genital he	Number	2017	4600023	6702953	2854632
Incidence	Global	Both	50-69 yea	Genital he	Rate	2017	350.38	510.558	217.435
Incidence	Global	Both	50-69 yea	Genital he	Number	2018	4713744	6855570	2941165
Incidence	Global	Both	50-69 yea	Genital he	Rate	2018	349.994	509.023	218.38
Incidence	Global	Both	50-69 yea	Genital he	Number	2019	4825335	7022289	3023072
Incidence	Global	Both	50-69 yea	Genital he	Rate	2019	349.929	509.25	219.23
Incidence	Global	Both	50-54 yea	Syphilis	Number	1990	253949	454752	135767
Incidence	Global	Both	50-54 yea	Syphilis	Rate	1990	119.454	213.909	63.8631
Incidence	Global	Both	50-54 yea	Syphilis	Number	1991	256788	458826	137098
Incidence	Global	Both	50-54 yea	Syphilis	Rate	1991	119.496	213.515	63.7988
Incidence	Global	Both	50-54 yea	Syphilis	Number	1992	259019	462276	138088
Incidence	Global	Both	50-54 yea	Syphilis	Rate	1992	119.763	213.743	63.848
Incidence	Global	Both	50-54 yea	Syphilis	Number	1993	260842	465444	138860
Incidence	Global	Both	50-54 yea	Syphilis	Rate	1993	120.145	214.385	63.9595
Incidence	Global	Both	50-54 yea	Syphilis	Number	1994	263034	469535	139829
Incidence	Global	Both	50-54 yea	Syphilis	Rate	1994	120.579	215.242	64.0996
Incidence	Global	Both	50-54 yea	Syphilis	Number	1995	265075	473497	140730
Incidence	Global	Both	50-54 yea	Syphilis	Rate	1995	120.987	216.116	64.2325
Incidence	Global	Both	50-54 yea	Syphilis	Number	1996	269506	481387	143848
Incidence	Global	Both	50-54 yea	Syphilis	Rate	1996	121.205	216.494	64.6926
Incidence	Global	Both	50-54 yea	Syphilis	Number	1997	277637	496426	148918
Incidence	Global	Both	50-54 yea	Syphilis	Rate	1997	120.365	215.217	64.5611
Incidence	Global	Both	50-54 yea	Syphilis	Number	1998	286468	513108	153462
Incidence	Global	Both	50-54 yea	Syphilis	Rate	1998	119.31	213.702	63.9145
Incidence	Global	Both	50-54 yea	Syphilis	Number	1999	297456	534154	159188
Incidence	Global	Both	50-54 yea	Syphilis	Rate	1999	117.944	211.796	63.1192
Incidence	Global	Both	50-54 yea	Syphilis	Number	2000	306175	550778	163668
Incidence	Global	Both	50-54 yea	Syphilis	Rate	2000	116.756	210.032	62.4128
Incidence	Global	Both	50-54 yea	Syphilis	Number	2001	319921	574873	171082
Incidence	Global	Both	50-54 yea	Syphilis	Rate	2001	116.181	208.768	62.1292
Incidence	Global	Both	50-54 yea	Syphilis	Number	2002	329846	591438	176500
Incidence	Global	Both	50-54 yea	Syphilis	Rate	2002	115.792	207.624	61.9604
Incidence	Global	Both	50-54 yea	Syphilis	Number	2003	339833	608169	181994
Incidence	Global	Both	50-54 yea	Syphilis	Rate	2003	115.163	206.098	61.6744
Incidence	Global	Both	50-54 yea	Syphilis	Number	2004	349043	623790	187043
Incidence	Global	Both	50-54 yea	Syphilis	Rate	2004	114.703	204.991	61.4661
Incidence	Global	Both	50-54 yea	Syphilis	Number	2005	362036	646913	194052
Incidence	Global	Both	50-54 yea	Syphilis	Rate	2005	114.301	204.242	61.2655
Incidence	Global	Both	50-54 yea	Syphilis	Number	2006	368330	657741	197198
Incidence	Global	Both	50-54 yea	Syphilis	Rate	2006	113.552	202.774	60.7939
Incidence	Global	Both	50-54 yea	Syphilis	Number	2007	373047	663799	199559
Incidence	Global	Both	50-54 yea	Syphilis	Rate	2007	112.705	200.547	60.2906
Incidence	Global	Both	50-54 yea	Syphilis	Number	2008	376325	667620	201600
Incidence	Global	Both	50-54 yea	Syphilis	Rate	2008	112.085	198.844	60.0446
Incidence	Global	Both	50-54 yea	Syphilis	Number	2009	379537	674279	203648
Incidence	Global	Both	50-54 yea	Syphilis	Rate	2009	111.839	198.692	60.0096
Incidence	Global	Both	50-54 yea	Syphilis	Number	2010	384841	685016	206876
Incidence	Global	Both	50-54 yea	Syphilis	Rate	2010	112.101	199.54	60.2613
Incidence	Global	Both	50-54 yea	Syphilis	Number	2011	393129	699101	210721
Incidence	Global	Both	50-54 yea	Syphilis	Rate	2011	112.54	200.131	60.3228
Incidence	Global	Both	50-54 yea	Syphilis	Number	2012	403919	718259	215930
Incidence	Global	Both	50-54 yea	Syphilis	Rate	2012	112.75	200.495	60.2746
Incidence	Global	Both	50-54 yea	Syphilis	Number	2013	417915	744425	223387
Incidence	Global	Both	50-54 yea	Syphilis	Rate	2013	112.712	200.772	60.2476
Incidence	Global	Both	50-54 yea	Syphilis	Number	2014	434034	775707	232317
Incidence	Global	Both	50-54 yea	Syphilis	Rate	2014	112.67	201.363	60.3064
Incidence	Global	Both	50-54 yea	Syphilis	Number	2015	450871	808864	241610
Incidence	Global	Both	50-54 yea	Syphilis	Rate	2015	112.84	202.435	60.4681
Incidence	Global	Both	50-54 yea	Syphilis	Number	2016	469833	839522	251040
Incidence	Global	Both	50-54 yea	Syphilis	Rate	2016	114.056	203.8	60.9419
Incidence	Global	Both	50-54 yea	Syphilis	Number	2017	488134	876434	260292
Incidence	Global	Both	50-54 yea	Syphilis	Rate	2017	115.521	207.416	61.6004
Incidence	Global	Both	50-54 yea	Syphilis	Number	2018	504204	903552	268862
Incidence	Global	Both	50-54 yea	Syphilis	Rate	2018	117.101	209.849	62.4431
Incidence	Global	Both	50-54 yea	Syphilis	Number	2019	522703	937582	279339
Incidence	Global	Both	50-54 yea	Syphilis	Rate	2019	119.662	214.64	63.9487
Incidence	Global	Both	55-59 yea	Syphilis	Number	1990	123089	216778	64185.3
Incidence	Global	Both	55-59 yea	Syphilis	Rate	1990	66.3856	116.914	34.617
Incidence	Global	Both	55-59 yea	Syphilis	Number	1991	124913	218635	65121.6
Incidence	Global	Both	55-59 yea	Syphilis	Rate	1991	66.4585	116.322	34.6471
Incidence	Global	Both	55-59 yea	Syphilis	Number	1992	127071	221794	66440.5
Incidence	Global	Both	55-59 yea	Syphilis	Rate	1992	66.4559	115.995	34.7473
Incidence	Global	Both	55-59 yea	Syphilis	Number	1993	129153	224875	67730.1
Incidence	Global	Both	55-59 yea	Syphilis	Rate	1993	66.4525	115.704	34.8489
Incidence	Global	Both	55-59 yea	Syphilis	Number	1994	131089	228110	68905.7
Incidence	Global	Both	55-59 yea	Syphilis	Rate	1994	66.4953	115.71	34.9527
Incidence	Global	Both	55-59 yea	Syphilis	Number	1995	132968	231804	69983.1
Incidence	Global	Both	55-59 yea	Syphilis	Rate	1995	66.555	116.026	35.029

Incidence	Global	Both	55-59 yea	Syphilis	Rate	2013	65.5442	113.282	34.7942
Incidence	Global	Both	55-59 yea	Syphilis	Number	2014	215701	372694	114534
Incidence	Global	Both	55-59 yea	Syphilis	Rate	2014	66.1373	114.274	35.1179
Incidence	Global	Both	55-59 yea	Syphilis	Number	2015	219746	380674	116591
Incidence	Global	Both	55-59 yea	Syphilis	Rate	2015	66.5678	115.318	35.3189
Incidence	Global	Both	55-59 yea	Syphilis	Number	2016	222477	388064	117434
Incidence	Global	Both	55-59 yea	Syphilis	Rate	2016	66.2121	115.493	34.95
Incidence	Global	Both	55-59 yea	Syphilis	Number	2017	226766	401491	118734
Incidence	Global	Both	55-59 yea	Syphilis	Rate	2017	65.7921	116.485	34.4485
Incidence	Global	Both	55-59 yea	Syphilis	Number	2018	235592	416270	123349
Incidence	Global	Both	55-59 yea	Syphilis	Rate	2018	66.0126	116.638	34.5621
Incidence	Global	Both	55-59 yea	Syphilis	Number	2019	246572	436023	128821
Incidence	Global	Both	55-59 yea	Syphilis	Rate	2019	66.4593	117.522	34.7215
Incidence	Global	Both	60-64 yea	Syphilis	Number	1990	24488.2	45908.2	10222.9
Incidence	Global	Both	60-64 yea	Syphilis	Rate	1990	15.2439	28.5778	6.36371
Incidence	Global	Both	60-64 yea	Syphilis	Number	1991	25259.9	47258.6	10607.5
Incidence	Global	Both	60-64 yea	Syphilis	Rate	1991	15.4036	28.8185	6.4685
Incidence	Global	Both	60-64 yea	Syphilis	Number	1992	25797.7	48248.9	10875
Incidence	Global	Both	60-64 yea	Syphilis	Rate	1992	15.5089	29.0059	6.53777
Incidence	Global	Both	60-64 yea	Syphilis	Number	1993	26200.6	48957.2	11069.3
Incidence	Global	Both	60-64 yea	Syphilis	Rate	1993	15.5619	29.0781	6.57462
Incidence	Global	Both	60-64 yea	Syphilis	Number	1994	26509.8	49618.2	11213.2
Incidence	Global	Both	60-64 yea	Syphilis	Rate	1994	15.5907	29.181	6.59459
Incidence	Global	Both	60-64 yea	Syphilis	Number	1995	26832.6	50332.4	11402.9
Incidence	Global	Both	60-64 yea	Syphilis	Rate	1995	15.6005	29.2633	6.62967
Incidence	Global	Both	60-64 yea	Syphilis	Number	1996	27191.4	50880.1	11542.5
Incidence	Global	Both	60-64 yea	Syphilis	Rate	1996	15.5758	29.1452	6.6118
Incidence	Global	Both	60-64 yea	Syphilis	Number	1997	27618.8	51588.6	11712.2
Incidence	Global	Both	60-64 yea	Syphilis	Rate	1997	15.5254	28.9996	6.58382
Incidence	Global	Both	60-64 yea	Syphilis	Number	1998	28046.1	52292.5	11887.4
Incidence	Global	Both	60-64 yea	Syphilis	Rate	1998	15.4803	28.8633	6.56138
Incidence	Global	Both	60-64 yea	Syphilis	Number	1999	28461.7	52950.4	12063.3
Incidence	Global	Both	60-64 yea	Syphilis	Rate	1999	15.4569	28.7562	6.5513
Incidence	Global	Both	60-64 yea	Syphilis	Number	2000	28898.7	53718.4	12255.1
Incidence	Global	Both	60-64 yea	Syphilis	Rate	2000	15.4574	28.7331	6.55505
Incidence	Global	Both	60-64 yea	Syphilis	Number	2001	29343.1	54690.4	12461
Incidence	Global	Both	60-64 yea	Syphilis	Rate	2001	15.5147	28.9167	6.58854
Incidence	Global	Both	60-64 yea	Syphilis	Number	2002	29782.3	55680.8	12680
Incidence	Global	Both	60-64 yea	Syphilis	Rate	2002	15.6293	29.2205	6.65427
Incidence	Global	Both	60-64 yea	Syphilis	Number	2003	30260.9	56767.6	12904.2
Incidence	Global	Both	60-64 yea	Syphilis	Rate	2003	15.7836	29.6091	6.73062
Incidence	Global	Both	60-64 yea	Syphilis	Number	2004	30776.1	57917.9	13129.9
Incidence	Global	Both	60-64 yea	Syphilis	Rate	2004	15.9207	29.9613	6.79218
Incidence	Global	Both	60-64 yea	Syphilis	Number	2005	31276	59009	13343.2
Incidence	Global	Both	60-64 yea	Syphilis	Rate	2005	16.0381	30.2593	6.84229
Incidence	Global	Both	60-64 yea	Syphilis	Number	2006	32019.4	60398.1	13687.4
Incidence	Global	Both	60-64 yea	Syphilis	Rate	2006	16.1007	30.3707	6.88258
Incidence	Global	Both	60-64 yea	Syphilis	Number	2007	33481.9	63072.2	14421.7
Incidence	Global	Both	60-64 yea	Syphilis	Rate	2007	16.1313	30.3877	6.94826
Incidence	Global	Both	60-64 yea	Syphilis	Number	2008	34963.9	65732.4	15115.3
Incidence	Global	Both	60-64 yea	Syphilis	Rate	2008	16.1008	30.2697	6.9606
Incidence	Global	Both	60-64 yea	Syphilis	Number	2009	36925.5	69312.3	15928.4
Incidence	Global	Both	60-64 yea	Syphilis	Rate	2009	16.1186	30.256	6.95304
Incidence	Global	Both	60-64 yea	Syphilis	Number	2010	38595.7	72263.7	16622.2
Incidence	Global	Both	60-64 yea	Syphilis	Rate	2010	16.1542	30.2459	6.95722
Incidence	Global	Both	60-64 yea	Syphilis	Number	2011	41043.5	77016.2	17677.2
Incidence	Global	Both	60-64 yea	Syphilis	Rate	2011	16.3148	30.6139	7.02669
Incidence	Global	Both	60-64 yea	Syphilis	Number	2012	43175.8	81195.8	18638
Incidence	Global	Both	60-64 yea	Syphilis	Rate	2012	16.5624	31.147	7.1496
Incidence	Global	Both	60-64 yea	Syphilis	Number	2013	45611.5	85792.1	19814.8
Incidence	Global	Both	60-64 yea	Syphilis	Rate	2013	16.8663	31.7244	7.32716
Incidence	Global	Both	60-64 yea	Syphilis	Number	2014	47735.8	90055.2	20839.7
Incidence	Global	Both	60-64 yea	Syphilis	Rate	2014	17.0942	32.2487	7.46269
Incidence	Global	Both	60-64 yea	Syphilis	Number	2015	50130.9	94950.6	21965.7
Incidence	Global	Both	60-64 yea	Syphilis	Rate	2015	17.2252	32.6255	7.54752
Incidence	Global	Both	60-64 yea	Syphilis	Number	2016	48695.9	91156	20996.9
Incidence	Global	Both	60-64 yea	Syphilis	Rate	2016	16.3267	30.5626	7.03981
Incidence	Global	Both	60-64 yea	Syphilis	Number	2017	46891.5	89549.7	19817.4
Incidence	Global	Both	60-64 yea	Syphilis	Rate	2017	15.3986	29.407	6.5078
Incidence	Global	Both	60-64 yea	Syphilis	Number	2018	47455.6	90431.7	20152.3
Incidence	Global	Both	60-64 yea	Syphilis	Rate	2018	15.3552	29.2609	6.52066
Incidence	Global	Both	60-64 yea	Syphilis	Number	2019	47931.2	91433.9	20340
Incidence	Global	Both	60-64 yea	Syphilis	Rate	2019	15.3363	29.2555	6.50807
Incidence	Global	Both	65-69 yea	Syphilis	Number	1990	0	0	0
Incidence	Global	Both	65-69 yea	Syphilis	Rate	1990	0	0	0
Incidence	Global	Both	65-69 yea	Syphilis	Number	1991	0	0	0
Incidence	Global	Both	65-69 yea	Syphilis	Rate	1991	0	0	0
Incidence	Global	Both	65-69 yea	Syphilis	Number	1992	0	0	0
Incidence	Global	Both	65-69 yea	Syphilis	Rate	1992	0	0	0
Incidence	Global	Both	65-69 yea	Syphilis	Number	1993	0	0	0
Incidence	Global	Both	65-69 yea	Syphilis	Rate	1993	0	0	0
Incidence	Global	Both	65-69 yea	Syphilis	Number	1994	0	0	0
Incidence	Global	Both	65-69 yea	Syphilis	Rate	1994	0	0	0
Incidence	Global	Both	65-69 yea	Syphilis	Number	1995	0	0	0
Incidence	Global	Both	65-69 yea	Syphilis	Rate	1995	0	0	0
Incidence	Global	Both	65-69 yea	Syphilis	Number	1996	0	0	0
Incidence	Global	Both	65-69 yea	Syphilis	Rate	1996	0	0	0
Incidence	Global	Both	65-69 yea	Syphilis	Number	1997	0	0	0
Incidence	Global	Both	65-69 yea	Syphilis	Rate	1997	0	0	0
Incidence	Global	Both	65-69 yea	Syphilis	Number	1998	0	0	0
Incidence	Global	Both	65-69 yea	Syphilis	Rate	1998	0	0	0
Incidence	Global	Both	65-69 yea	Syphilis	Number	1999	0	0	0
Incidence	Global	Both	65-69 yea	Syphilis	Rate	1999	0	0	0
Incidence	Global	Both	65-69 yea	Syphilis	Number	2000	0	0	0
Incidence	Global	Both	65-69 yea	Syphilis	Rate	2000	0	0	0
Incidence	Global	Both	65-69 yea	Syphilis	Number	2001	0	0	0
Incidence	Global	Both	65-69 yea	Syphilis	Rate	2001	0	0	0
Incidence	Global	Both	65-69 yea	Syphilis	Number	2002	0	0	0
Incidence	Global	Both	65-69 yea	Syphilis	Rate	2002	0	0	0
Incidence	Global	Both	65-69 yea	Syphilis	Number	2003	0	0	0
Incidence	Global	Both	65-69 yea	Syphilis	Rate	2003	0	0	0
Incidence	Global	Both	65-69 yea	Syphilis	Number	2004	0	0	0
Incidence	Global	Both	65-69 yea	Syphilis	Rate	2004	0	0	0
Incidence	Global	Both	65-69 yea	Syphilis	Number	2005	0	0	0
Incidence	Global	Both	65-69 yea	Syphilis	Rate	2005	0	0	0
Incidence	Global	Both	65-69 yea	Syphilis	Number	2006	0	0	0
Incidence	Global	Both	65-69 yea	Syphilis	Rate	2006	0	0	0
Incidence	Global	Both	65-69 yea	Syphilis	Number	2007	0	0	0
Incidence	Global	Both	65-69 yea	Syphilis	Rate	2007	0	0	0
Incidence	Global	Both	65-69 yea	Syphilis	Number	2008	0	0	0
Incidence	Global	Both	65-69 yea	Syphilis	Rate	2008	0	0	0
Incidence	Global	Both	65-69 yea	Syphilis	Number	2009	0	0	0
Incidence	Global	Both	65-69 yea	Syphilis	Rate	2009	0	0	0
Incidence	Global	Both	65-69 yea	Syphilis	Number	2010	0	0	0
Incidence	Global	Both	65-69 yea	Syphilis	Rate	2010	0	0	0
Incidence	Global	Both	65-69 yea	Syphilis	Number	2011	0	0	0
Incidence	Global	Both	65-69 yea	Syphilis	Rate	2011	0	0	0
Incidence	Global	Both	65-69 yea	Syphilis	Number	2012	0	0	0
Incidence	Global	Both	65-69 yea	Syphilis	Rate	2012	0	0	0
Incidence	Global	Both	65-69 yea	Syphilis	Number	2013	0	0	0
Incidence	Global	Both	65-69 yea	Syphilis	Rate	2013	0	0	0
Incidence	Global	Both	65-69 yea	Syphilis	Number	2014	0	0	0
Incidence	Global	Both	65-69 yea	Syphilis	Rate	2014	0	0	0
Incidence	Global	Both	65-69 yea	Syphilis	Number	2015	0	0	0
Incidence	Global	Both	65-69 yea	Syphilis	Rate	2015	0	0	0
Incidence	Global	Both	65-69 yea	Syphilis	Number	2016	0	0	0
Incidence	Global	Both	65-69 yea	Syphilis	Rate	2016	0	0	0
Incidence	Global	Both	65-69 yea	Syphilis	Number	2017	0	0	0
Incidence	Global	Both	65-69 yea	Syphilis	Rate	2017	0	0	0
Incidence	Global	Both	65-69 yea	Syphilis	Number	2018	0	0	0
Incidence	Global	Both	65-69 yea	Syphilis	Rate	2018	0	0	0
Incidence	Global	Both	65-69 yea	Syphilis	Number	2019	0	0	0
Incidence	Global	Both	65-69 yea	Syphilis	Rate	2019	0	0	0
Incidence	Global	Both	50-69 yea	Syphilis	Number	1990	401527	672843	231219
Incidence	Global	Both	50-69 yea	Syphilis	Rate	1990	58.8629	98.6373	33.8962
Incidence	Global	Both	50-69 yea	Syphilis	Number	1991	406961	681242	235013
Incidence	Global	Both	50-69 yea	Syphilis	Rate	1991	58.6308	98.1464	33.8583
Incidence	Global	Both	50-69 yea	Syphilis	Number	1992	411887	686679	238482
Incidence	Global	Both	50-69 yea	Syphilis	Rate	1992	58.4291	97.4101	33.8303
Incidence	Global	Both	50-69 yea	Syphilis	Number	1993	416196	689965	241559
Incidence	Global	Both	50-69 yea	Syphilis	Rate	1993	58.2284	96.5304	33.7957
Incidence	Global	Both	50-69 yea	Syphilis	Number	1994	420632	693578	244665
Incidence	Global	Both	50-69 yea	Syphilis	Rate	1994	58.0934	95.7898	33.7906
Incidence	Global	Both	50-69 yea	Syphilis	Number	1995	424876	696895	247475
Incidence	Global	Both	50-69 yea	Syphilis	Rate	1995	57.9684	95.0818	33.7646
Incidence	Global	Both	50-69 yea	Syphilis	Number	1996	431142	710092	251742
Incidence	Global	Both	50-69 yea	Syphilis	Rate	1996	57.9694	95.4757	33.8481
Incidence	Global	Both	50-69 yea	Syphilis	Number	1997	440789	729995	257804
Incidence	Global	Both	50-69 yea	Syphilis	Rate	1997	58.0979	96.2166	33.9797
Incidence	Global	Both	50-69 yea	Syphilis	Number	1998	450931	750834	263075
Incidence	Global	Both	50-69 yea	Syphilis	Rate	1998	58.2471	96.9858	33.9815
Incidence	Global	Both	50-69 yea	Syphilis	Number	1999	463349	775641	269416
Incidence	Global	Both	50-69 yea	Syphilis	Rate	1999	58.4957	97.9212	34.0125
Incidence	Global	Both	50-69 yea	Syphilis	Number	2000	473495	796120	275952
Incidence	Global	Both	50-69 yea	Syphilis	Rate	2000	58.5846	98.5025	34.143
Incidence	Global	Both	50-69 yea	Syphilis	Number	2001	490129	825449	285061
Incidence	Global	Both	50-69 yea	Syphilis	Rate	2001	59.0706	99.4836	34.3557
Incidence	Global	Both	50-69 yea	Syphilis	Number	2002	505568	850724	293494
Incidence	Global	Both	50-69 yea	Syphilis	Rate	2002	59.3014	99.787	34.4258
Incidence	Global	Both	50-69 yea	Syphilis	Number	2003	521550	876121	302240
Incidence	Global	Both	50-69 yea	Syphilis	Rate	2003	59.4798	99.9167	34.4688
Incidence	Global	Both	50-69 yea	Syphilis	Number	2004	538148	901507	312393
Incidence	Global	Both	50-69 yea	Syphilis	Rate	2004	59.6085	99.8562	34.6025
Incidence	Global	Both	50-69 yea	Syphilis	Number	2005	557074	931983	323350
Incidence	Global	Both	50-69 yea	Syphilis	Rate	2005	59.9055	100.222	34.7717
Incidence	Global	Both	50-69 yea	Syphilis	Number	2006	571656	956052	331505
Incidence	Global	Both	50-69 yea	Syphilis	Rate	2006	59.7484	99.9247	34.6483
Incidence	Global	Both	50-69 yea	Syphilis	Number	2007	582905	974056	337882
Incidence	Global	Both	50-69 yea	Syphilis	Rate	2007	59.2841	99.066	34.3641
Incidence	Global	Both	50-69 yea	Syphilis	Number	2008	593034	989454	343863
Incidence	Global	Both	50-69 yea	Syphilis	Rate	2008	58.7581	98.0356	34.0701
Incidence	Global	Both	50-69 yea	Syphilis	Number	2009	603329	1004619	350304
Incidence	Global	Both	50-69 yea	Syphilis	Rate	2009	58.2417	96.9799	33.8162
Incidence	Global	Both	50-69 yea	Syphilis	Number	2010	618186	1027425	359429
Incidence	Global	Both	50-69 yea	Syphilis	Rate	2010	58.1024	96.5662	33.7822
Incidence	Global	Both	50-69 yea	Syphilis	Number	2011	634487	1054572	369387
Incidence	Global	Both	50-69 yea	Syphilis	Rate	2011	57.9912	96.3863	33.7614
Incidence	Global	Both	50-69 yea	Syphilis	Number	2012	653252	1086821	380411
Incidence	Global	Both	50-69 yea	Syphilis	Rate	2012	57.9553	96.4206	33.7493
Incidence	Global	Both	50-69 yea	Syphilis	Number	2013	674852	1124460	393022
Incidence	Global	Both	50-69 yea	Syphilis	Rate	2013	58.0028	96.6462	33.7799
Incidence	Global	Both	50-69 yea	Syphilis	Number	2014	697471	1164455	406107
Incidence	Global	Both	50-69 yea	Syphilis	Rate	2014	58.0328	96.8881	33.79
Incidence	Global	Both	50-69 yea	Syphilis	Number	2015	720748	1205269	419354
Incidence	Global	Both	50-69 yea	Syphilis	Rate	2015	58.0664	97.1014	33.7849
Incidence	Global	Both	50-69 yea	Syphilis	Number	2016	741006	1242068	427445
Incidence	Global	Both	50-69 yea	Syphilis	Rate	2016	57.9498	97.135	33.428
Incidence	Global	Both	50-69 yea	Syphilis	Number	2017	761792	1284380	436993
Incidence	Global	Both	50-69 yea	Syphilis	Rate	2017	58.0251	97.8301	33.2854
Incidence	Global	Both	50-69 yea	Syphilis	Number	2018	787251	1329321	453350
Incidence	Global	Both	50-69 yea	Syphilis	Rate	2018	58.4531	98.7016	33.6611
Incidence	Global	Both	50-69 yea	Syphilis	Number	2019	817206	1380797	471940
Incidence	Global	Both	50-69 yea	Syphilis	Rate	2019	59.263	100.134	34.2246
Incidence	Global	Both	50-69 yea	Gonococca	Number	1990	2446241	3846333	1598323
Incidence	Global	Both	50-69 yea	Gonococca	Rate	1990	358.614	563.864	234.311
Incidence	Global	Both	50-69 yea	Gonococca	Number	1991	2481196	3895536	1620412
Incidence	Global	Both	50-69 yea	Gonococca	Rate	1991	357.465	561.229	233.452
Incidence	Global	Both	50-69 yea	Gonococca	Number	1992	2507795	3934737	1636721
Incidence	Global	Both	50-69 yea	Gonococca	Rate	1992	355.748	558.17	232.18
Incidence	Global	Both	50-69 yea	Gonococca	Number	1993	2521562	3956662	1645069
Incidence	Global	Both	50-69 yea	Gonococca	Rate	1993	352.782	553.562	230.156
Incidence	Global	Both	50-69 yea	Gonococca	Number	1994	2531256	3972657	1651378
Incidence	Global	Both	50-69 yea	Gonococca	Rate	1994	349.591	548.662	228.071
Incidence	Global	Both	50-69 yea	Gonococca	Number	1995	2538979	3976812	1659277
Incidence	Global	Both	50-69 yea	Gonococca	Rate	1995	346.409	542.581	226.385
Incidence	Global	Both	50-69 yea	Gonococca	Number	1996	2557961	4008468	1673280
Incidence	Global	Both	50-69 yea	Gonococca	Rate	1996	343.932	538.96	224.981
Incidence	Global	Both	50-69 yea	Gonococca	Number	1997	2600980	4072230	1702515
Incidence	Global	Both	50-69 yea	Gonococca	Rate	1997	342.821	536.738	224.399
Incidence	Global	Both	50-69 yea	Gonococca	Number	1998	2650975	4144527	1736131
Incidence	Global	Both	50-69 yea	Gonococca	Rate	1998	342.428	535.352	224.257
Incidence	Global	Both	50-69 yea	Gonococca	Number	1999	2724570	4258943	1784918
Incidence	Global	Both	50-69 yea	Gonococca	Rate	1999	343.965	537.672	225.338
Incidence	Global	Both	50-69 yea	Gonococca	Number	2000	2789270	4378591	1822723
Incidence	Global	Both	50-69 yea	Gonococca	Rate	2000	345.111	541.755	225.522
Incidence	Global	Both	50-69 yea	Gonococca	Number	2001	2889718	4552061	1882335
Incidence	Global	Both	50-69 yea	Gonococca	Rate	2001	348.27	548.617	226.86
Incidence	Global	Both	50-69 yea	Gonococca	Number	2002	2984288	4705282	1941068
Incidence	Global	Both	50-69 yea	Gonococca	Rate	2002	350.047	551.913	227.681
Incidence	Global	Both	50-69 yea	Gonococca	Number	2003	3089512	4882445	2005512
Incidence	Global	Both	50-69 yea	Gonococca	Rate	2003	352.342	556.815	228.717
Incidence	Global	Both	50-69 yea	Gonococca	Number	2004	3197334	5057771	2072089
Incidence	Global	Both	50-69 yea	Gonococca	Rate	2004	354.156	560.228	229.517
Incidence	Global	Both	50-69 yea	Gonococca	Number	2005	3320564	5246988	2144422
Incidence	Global	Both	50-69 yea	Gonococca	Rate	2005	357.08	564.24	230.603
Incidence	Global	Both	50-69 yea	Gonococca	Number	2006	3416479	5407738	2206391
Incidence	Global	Both	50-69 yea	Gonococca	Rate	2006	357.084	565.206	230.608
Incidence	Global	Both	50-69 yea	Gonococca	Number	2007	3490495	5499100	2257650
Incidence	Global	Both	50-69 yea	Gonococca	Rate	2007	354.999	559.284	229.613
Incidence	Global	Both	50-69 yea	Gonococca	Number	2008	3552164	5580154	2302841
Incidence	Global	Both	50-69 yea	Gonococca	Rate	2008	351.95	552.885	228.167
Incidence	Global	Both	50-69 yea	Gonococca	Number	2009	3608367	5667252	2344614
Incidence	Global	Both	50-69 yea	Gonococca	Rate	2009	348.33	547.082	226.335
Incidence	Global	Both	50-69 yea	Gonococca	Number	2010	3682138	5790390	2403696
Incidence	Global	Both	50-69 yea	Gonococca	Rate	2010	346.079	544.23	225.92
Incidence	Global	Both	50-69 yea	Gonococca	Number	2011	3757143	5900073	2460557
Incidence	Global	Both	50-69 yea	Gonococca	Rate	2011	343.397	539.258	224.891
Incidence	Global	Both	50-69 yea	Gonococca	Number	2012	3840731	6027191	2518135
Incidence	Global	Both	50-69 yea	Gonococca	Rate	2012	340.742	534.721	223.404
Incidence	Global	Both	50-69 yea	Gonococca	Number	2013	3939170	6181042	2579232
Incidence	Global	Both	50-69 yea	Gonococca	Rate	2013	338.568	531.254	221.682
Incidence	Global	Both	50-69 yea	Gonococca	Number	2014	4044737	6348593	2646327
Incidence	Global	Both	50-69 yea	Gonococca	Rate	2014	336.541	528.233	220.187
Incidence	Global	Both	50-69 yea	Gonococca	Number	2015	4155561	6511289	2721710
Incidence	Global	Both	50-69 yea	Gonococca	Rate	2015	334.789	524.576	219.272
Incidence	Global	Both	50-69 yea	Gonococca	Number	2016	4244866	6642756	2786265
Incidence	Global	Both	50-69 yea	Gonococca	Rate	2016	331.966	519.492	217.898
Incidence	Global	Both	50-69 yea	Gonococca	Number	2017	4331664	6762702	2837250
Incidence	Global	Both	50-69 yea	Gonococca	Rate	2017	329.939	515.109	216.111
Incidence	Global	Both	50-69 yea	Gonococca	Number	2018	4447501	6968819	2910517
Incidence	Global	Both	50-69 yea	Gonococca	Rate	2018	330.225	517.432	216.105
Incidence	Global	Both	50-69 yea	Gonococca	Number	2019	4585078	7213865	2991850
Incidence	Global	Both	50-69 yea	Gonococca	Rate	2019	332.505	523.142	216.966
Incidence	Global	Both	50-54 yea	Other sexu	Number	1990	0	0	0
Incidence	Global	Both	50-54 yea	Other sexu	Rate	1990	0	0	0
Incidence	Global	Both	50-54 yea	Other sexu	Number	1991	0	0	0
Incidence	Global	Both	50-54 yea	Other sexu	Rate	1991	0	0	0
Incidence	Global	Both	50-54 yea	Other sexu	Number	1992	0	0	0
Incidence	Global	Both	50-54 yea	Other sexu	Rate	1992	0	0	0
Incidence	Global	Both	50-54 yea	Other sexu	Number	1993	0	0	0
Incidence	Global	Both	50-54 yea	Other sexu	Rate	1993	0	0	0
Incidence	Global	Both	50-54 yea	Other sexu	Number	1994	0	0	0
Incidence	Global	Both	50-54 yea	Other sexu	Rate	1994	0	0	0
Incidence	Global	Both	50-54 yea	Other sexu	Number	1995	0	0	0
Incidence	Global	Both	50-54 yea	Other sexu	Rate	1995	0	0	0
Incidence	Global	Both	50-54 yea	Other sexu	Number	1996	0	0	0
Incidence	Global	Both	50-54 yea	Other sexu	Rate	1996	0	0	0
Incidence	Global	Both	50-54 yea	Other sexu	Number	1997	0	0	0
Incidence	Global	Both	50-54 yea	Other sexu	Rate	1997	0	0	0
Incidence	Global	Both	50-54 yea	Other sexu	Number	1998	0	0	0
Incidence	Global	Both	50-54 yea	Other sexu	Rate	1998	0	0	0
Incidence	Global	Both	50-54 yea	Other sexu	Number	1999	0	0	0
Incidence	Global	Both	50-54 yea	Other sexu	Rate	1999	0	0	0
Incidence	Global	Both	50-54 yea	Other sexu	Number	2000	0	0	0
Incidence	Global	Both	50-54 yea	Other sexu	Rate	2000	0	0	0
Incidence	Global	Both	50-54 yea	Other sexu	Number	2001	0	0	0
Incidence	Global	Both	50-54 yea	Other sexu	Rate	2001	0	0	0
Incidence	Global	Both	50-54 yea	Other sexu	Number	2002	0	0	0
Incidence	Global	Both	50-54 yea	Other sexu	Rate	2002	0	0	0
Incidence	Global	Both	50-54 yea	Other sexu	Number	2003	0	0	0
Incidence	Global	Both	50-54 yea	Other sexu	Rate	2003	0	0	0
Incidence	Global	Both	50-54 yea	Other sexu	Number	2004	0	0	0
Incidence	Global	Both	50-54 yea	Other sexu	Rate	2004	0	0	0
Incidence	Global	Both	50-54 yea	Other sexu	Number	2005	0	0	0
Incidence	Global	Both	50-54 yea	Other sexu	Rate	2005	0	0	0
Incidence	Global	Both	50-54 yea	Other sexu	Number	2006	0	0	0
Incidence	Global	Both	50-54 yea	Other sexu	Rate	2006	0	0	0
Incidence	Global	Both	50-54 yea	Other sexu	Number	2007	0	0	0
Incidence	Global	Both	50-54 yea	Other sexu	Rate	2007	0	0	0
Incidence	Global	Both	50-54 yea	Other sexu	Number	2008	0	0	0
Incidence	Global	Both	50-54 yea	Other sexu	Rate	2008	0	0	0
Incidence	Global	Both	50-54 yea	Other sexu	Number	2009	0	0	0
Incidence	Global	Both	50-54 yea	Other sexu	Rate	2009	0	0	0
Incidence	Global	Both	50-54 yea	Other sexu	Number	2010	0	0	0
Incidence	Global	Both	50-54 yea	Other sexu	Rate	2010	0	0	0
Incidence	Global	Both	50-54 yea	Other sexu	Number	2011	0	0	0
Incidence	Global	Both	50-54 yea	Other sexu	Rate	2011	0	0	0
Incidence	Global	Both	50-54 yea	Other sexu	Number	2012	0	0	0
Incidence	Global	Both	50-54 yea	Other sexu	Rate	2012	0	0	0
Incidence	Global	Both	50-54 yea	Other sexu	Number	2013	0	0	0
Incidence	Global	Both	50-54 yea	Other sexu	Rate	2013	0	0	0
Incidence	Global	Both	50-54 yea	Other sexu	Number	2014	0	0	0
Incidence	Global	Both	50-54 yea	Other sexu	Rate	2014	0	0	0
Incidence	Global	Both	50-54 yea	Other sexu	Number	2015	0	0	0
Incidence	Global	Both	50-54 yea	Other sexu	Rate	2015	0	0	0
Incidence	Global	Both	50-54 yea	Other sexu	Number	2016	0	0	0
Incidence	Global	Both	50-54 yea	Other sexu	Rate	2016	0	0	0
Incidence	Global	Both	50-54 yea	Other sexu	Number	2017	0	0	0
Incidence	Global	Both	50-54 yea	Other sexu	Rate	2017	0	0	0
Incidence	Global	Both	50-54 yea	Other sexu	Number	2018	0	0	0
Incidence	Global	Both	50-54 yea	Other sexu	Rate	2018	0	0	0
Incidence	Global	Both	50-54 yea	Other sexu	Number	2019	0	0	0
Incidence	Global	Both	50-54 yea	Other sexu	Rate	2019	0	0	0
Incidence	Global	Both	55-59 yea	Other sexu	Number	1990	0	0	0
Incidence	Global	Both	55-59 yea	Other sexu	Rate	1990	0	0	0
Incidence	Global	Both	55-59 yea	Other sexu	Number	1991	0	0	0
Incidence	Global	Both	55-59 yea	Other sexu	Rate	1991	0	0	0
Incidence	Global	Both	55-59 yea	Other sexu	Number	1992	0	0	0
Incidence	Global	Both	55-59 yea	Other sexu	Rate	1992	0	0	0
Incidence	Global	Both	55-59 yea	Other sexu	Number	1993	0	0	0
Incidence	Global	Both	55-59 yea	Other sexu	Rate	1993	0	0	0
Incidence	Global	Both	55-59 yea	Other sexu	Number	1994	0	0	0
Incidence	Global	Both	55-59 yea	Other sexu	Rate	1994	0	0	0
Incidence	Global	Both	55-59 yea	Other sexu	Number	1995	0	0	0
Incidence	Global	Both	55-59 yea	Other sexu	Rate	1995	0	0	0
Incidence	Global	Both	55-59 yea	Other sexu	Number	1996	0	0	0
Incidence	Global	Both	55-59 yea	Other sexu	Rate	1996	0	0	0
Incidence	Global	Both	55-59 yea	Other sexu	Number	1997	0	0	0
Incidence	Global	Both	55-59 yea	Other sexu	Rate	1997	0	0	0
Incidence	Global	Both	55-59 yea	Other sexu	Number	1998	0	0	0
Incidence	Global	Both	55-59 yea	Other sexu	Rate	1998	0	0	0
Incidence	Global	Both	55-59 yea	Other sexu	Number	1999	0	0	0
Incidence	Global	Both	55-59 yea	Other sexu	Rate	1999	0	0	0
Incidence	Global	Both	55-59 yea	Other sexu	Number	2000	0	0	0
Incidence	Global	Both	55-59 yea	Other sexu	Rate	2000	0	0	0
Incidence	Global	Both	55-59 yea	Other sexu	Number	2001	0	0	0
Incidence	Global	Both	55-59 yea	Other sexu	Rate	2001	0	0	0
Incidence	Global	Both	55-59 yea	Other sexu	Number	2002	0	0	0
Incidence	Global	Both	55-59 yea	Other sexu	Rate	2002	0	0	0
Incidence	Global	Both	55-59 yea	Other sexu	Number	2003	0	0	0
Incidence	Global	Both	55-59 yea	Other sexu	Rate	2003	0	0	0
Incidence	Global	Both	55-59 yea	Other sexu	Number	2004	0	0	0
Incidence	Global	Both	55-59 yea	Other sexu	Rate	2004	0	0	0
Incidence	Global	Both	55-59 yea	Other sexu	Number	2005	0	0	0
Incidence	Global	Both	55-59 yea	Other sexu	Rate	2005	0	0	0
Incidence	Global	Both	55-59 yea	Other sexu	Number	2006	0	0	0
Incidence	Global	Both	55-59 yea	Other sexu	Rate	2006	0	0	0
Incidence	Global	Both	55-59 yea	Other sexu	Number	2007	0	0	0
Incidence	Global	Both	55-59 yea	Other sexu	Rate	2007	0	0	0
Incidence	Global	Both	55-59 yea	Other sexu	Number	2008	0	0	0
Incidence	Global	Both	55-59 yea	Other sexu	Rate	2008	0	0	0
Incidence	Global	Both	55-59 yea	Other sexu	Number	2009	0	0	0
Incidence	Global	Both	55-59 yea	Other sexu	Rate	2009	0	0	0
Incidence	Global	Both	55-59 yea	Other sexu	Number	2010	0	0	0
Incidence	Global	Both	55-59 yea	Other sexu	Rate	2010	0	0	0
Incidence	Global	Both	55-59 yea	Other sexu	Number	2011	0	0	0
Incidence	Global	Both	55-59 yea	Other sexu	Rate	2011	0	0	0
Incidence	Global	Both	55-59 yea	Other sexu	Number	2012	0	0	0
Incidence	Global	Both	55-59 yea	Other sexu	Rate	2012	0	0	0
Incidence	Global	Both	55-59 yea	Other sexu	Number	2013	0	0	0
Incidence	Global	Both	55-59 yea	Other sexu	Rate	2013	0	0	0
Incidence	Global	Both	55-59 yea	Other sexu	Number	2014	0	0	0
Incidence	Global	Both	55-59 yea	Other sexu	Rate	2014	0	0	0
Incidence	Global	Both	55-59 yea	Other sexu	Number	2015	0	0	0
Incidence	Global	Both	55-59 yea	Other sexu	Rate	2015	0	0	0
Incidence	Global	Both	55-59 yea	Other sexu	Number	2016	0	0	0
Incidence	Global	Both	55-59 yea	Other sexu	Rate	2016	0	0	0
Incidence	Global	Both	55-59 yea	Other sexu	Number	2017	0	0	0
Incidence	Global	Both	55-59 yea	Other sexu	Rate	2017	0	0	0
Incidence	Global	Both	55-59 yea	Other sexu	Number	2018	0	0	0
Incidence	Global	Both	55-59 yea	Other sexu	Rate	2018	0	0	0
Incidence	Global	Both	55-59 yea	Other sexu	Number	2019	0	0	0
Incidence	Global	Both	55-59 yea	Other sexu	Rate	2019	0	0	0
Incidence	Global	Both	60-64 yea	Other sexu	Number	1990	0	0	0
Incidence	Global	Both	60-64 yea	Other sexu	Rate	1990	0	0	0
Incidence	Global	Both	60-64 yea	Other sexu	Number	1991	0	0	0
Incidence	Global	Both	60-64 yea	Other sexu	Rate	1991	0	0	0
Incidence	Global	Both	60-64 yea	Other sexu	Number	1992	0	0	0
Incidence	Global	Both	60-64 yea	Other sexu	Rate	1992	0	0	0
Incidence	Global	Both	60-64 yea	Other sexu	Number	1993	0	0	0
Incidence	Global	Both	60-64 yea	Other sexu	Rate	1993	0	0	0
Incidence	Global	Both	60-64 yea	Other sexu	Number	1994	0	0	0
Incidence	Global	Both	60-64 yea	Other sexu	Rate	1994	0	0	0
Incidence	Global	Both	60-64 yea	Other sexu	Number	1995	0	0	0
Incidence	Global	Both	60-64 yea	Other sexu	Rate	1995	0	0	0
Incidence	Global	Both	60-64 yea	Other sexu	Number	1996	0	0	0
Incidence	Global	Both	60-64 yea	Other sexu	Rate	1996	0	0	0
Incidence	Global	Both	60-64 yea	Other sexu	Number	1997	0	0	0
Incidence	Global	Both	60-64 yea	Other sexu	Rate	1997	0	0	0
Incidence	Global	Both	60-64 yea	Other sexu	Number	1998	0	0	0
Incidence	Global	Both	60-64 yea	Other sexu	Rate	1998	0	0	0
Incidence	Global	Both	60-64 yea	Other sexu	Number	1999	0	0	0
Incidence	Global	Both	60-64 yea	Other sexu	Rate	1999	0	0	0
Incidence	Global	Both	60-64 yea	Other sexu	Number	2000	0	0	0
Incidence	Global	Both	60-64 yea	Other sexu	Rate	2000	0	0	0
Incidence	Global	Both	60-64 yea	Other sexu	Number	2001	0	0	0
Incidence	Global	Both	60-64 yea	Other sexu	Rate	2001	0	0	0
Incidence	Global	Both	60-64 yea	Other sexu	Number	2002	0	0	0
Incidence	Global	Both	60-64 yea	Other sexu	Rate	2002	0	0	0
Incidence	Global	Both	60-64 yea	Other sexu	Number	2003	0	0	0
Incidence	Global	Both	60-64 yea	Other sexu	Rate	2003	0	0	0
Incidence	Global	Both	60-64 yea	Other sexu	Number	2004	0	0	0
Incidence	Global	Both	60-64 yea	Other sexu	Rate	2004	0	0	0
Incidence	Global	Both	60-64 yea	Other sexu	Number	2005	0	0	0
Incidence	Global	Both	60-64 yea	Other sexu	Rate	2005	0	0	0
Incidence	Global	Both	60-64 yea	Other sexu	Number	2006	0	0	0
Incidence	Global	Both	60-64 yea	Other sexu	Rate	2006	0	0	0
Incidence	Global	Both	60-64 yea	Other sexu	Number	2007	0	0	0
Incidence	Global	Both	60-64 yea	Other sexu	Rate	2007	0	0	0
Incidence	Global	Both	60-64 yea	Other sexu	Number	2008	0	0	0
Incidence	Global	Both	60-64 yea	Other sexu	Rate	2008	0	0	0
Incidence	Global	Both	60-64 yea	Other sexu	Number	2009	0	0	0
Incidence	Global	Both	60-64 yea	Other sexu	Rate	2009	0	0	0
Incidence	Global	Both	60-64 yea	Other sexu	Number	2010	0	0	0
Incidence	Global	Both	60-64 yea	Other sexu	Rate	2010	0	0	0
Incidence	Global	Both	60-64 yea	Other sexu	Number	2011	0	0	0
Incidence	Global	Both	60-64 yea	Other sexu	Rate	2011	0	0	0
Incidence	Global	Both	60-64 yea	Other sexu	Number	2012	0	0	0
Incidence	Global	Both	60-64 yea	Other sexu	Rate	2012	0	0	0
Incidence	Global	Both	60-64 yea	Other sexu	Number	2013	0	0	0
Incidence	Global	Both	60-64 yea	Other sexu	Rate	2013	0	0	0
Incidence	Global	Both	60-64 yea	Other sexu	Number	2014	0	0	0
Incidence	Global	Both	60-64 yea	Other sexu	Rate	2014	0	0	0
Incidence	Global	Both	60-64 yea	Other sexu	Number	2015	0	0	0
Incidence	Global	Both	60-64 yea	Other sexu	Rate	2015	0	0	0
Incidence	Global	Both	60-64 yea	Other sexu	Number	2016	0	0	0
Incidence	Global	Both	60-64 yea	Other sexu	Rate	2016	0	0	0
Incidence	Global	Both	60-64 yea	Other sexu	Number	2017	0	0	0
Incidence	Global	Both	60-64 yea	Other sexu	Rate	2017	0	0	0
Incidence	Global	Both	60-64 yea	Other sexu	Number	2018	0	0	0
Incidence	Global	Both	60-64 yea	Other sexu	Rate	2018	0	0	0
Incidence	Global	Both	60-64 yea	Other sexu	Number	2019	0	0	0
Incidence	Global	Both	60-64 yea	Other sexu	Rate	2019	0	0	0
Incidence	Global	Both	65-69 yea	Other sexu	Number	1990	0	0	0
Incidence	Global	Both	65-69 yea	Other sexu	Rate	1990	0	0	0
Incidence	Global	Both	65-69 yea	Other sexu	Number	1991	0	0	0
Incidence	Global	Both	65-69 yea	Other sexu	Rate	1991	0	0	0
Incidence	Global	Both	65-69 yea	Other sexu	Number	1992	0	0	0
Incidence	Global	Both	65-69 yea	Other sexu	Rate	1992	0	0	0
Incidence	Global	Both	65-69 yea	Other sexu	Number	1993	0	0	0
Incidence	Global	Both	65-69 yea	Other sexu	Rate	1993	0	0	0
Incidence	Global	Both	65-69 yea	Other sexu	Number	1994	0	0	0
Incidence	Global	Both	65-69 yea	Other sexu	Rate	1994	0	0	0
Incidence	Global	Both	65-69 yea	Other sexu	Number	1995	0	0	0
Incidence	Global	Both	65-69 yea	Other sexu	Rate	1995	0	0	0
Incidence	Global	Both	65-69 yea	Other sexu	Number	1996	0	0	0
Incidence	Global	Both	65-69 yea	Other sexu	Rate	1996	0	0	0
Incidence	Global	Both	65-69 yea	Other sexu	Number	1997	0	0	0
Incidence	Global	Both	65-69 yea	Other sexu	Rate	1997	0	0	0
Incidence	Global	Both	65-69 yea	Other sexu	Number	1998	0	0	0
Incidence	Global	Both	65-69 yea	Other sexu	Rate	1998	0	0	0
Incidence	Global	Both	65-69 yea	Other sexu	Number	1999	0	0	0
Incidence	Global	Both	65-69 yea	Other sexu	Rate	1999	0	0	0
Incidence	Global	Both	65-69 yea	Other sexu	Number	2000	0	0	0
Incidence	Global	Both	65-69 yea	Other sexu	Rate	2000	0	0	0
Incidence	Global	Both	65-69 yea	Other sexu	Number	2001	0	0	0
Incidence	Global	Both	65-69 yea	Other sexu	Rate	2001	0	0	0
Incidence	Global	Both	65-69 yea	Other sexu	Number	2002	0	0	0
Incidence	Global	Both	65-69 yea	Other sexu	Rate	2002	0	0	0
Incidence	Global	Both	65-69 yea	Other sexu	Number	2003	0	0	0
Incidence	Global	Both	65-69 yea	Other sexu	Rate	2003	0	0	0
Incidence	Global	Both	65-69 yea	Other sexu	Number	2004	0	0	0
Incidence	Global	Both	65-69 yea	Other sexu	Rate	2004	0	0	0
Incidence	Global	Both	65-69 yea	Other sexu	Number	2005	0	0	0
Incidence	Global	Both	65-69 yea	Other sexu	Rate	2005	0	0	0
Incidence	Global	Both	65-69 yea	Other sexu	Number	2006	0	0	0
Incidence	Global	Both	65-69 yea	Other sexu	Rate	2006	0	0	0
Incidence	Global	Both	65-69 yea	Other sexu	Number	2007	0	0	0
Incidence	Global	Both	65-69 yea	Other sexu	Rate	2007	0	0	0
Incidence	Global	Both	65-69 yea	Other sexu	Number	2008	0	0	0
Incidence	Global	Both	65-69 yea	Other sexu	Rate	2008	0	0	0
Incidence	Global	Both	65-69 yea	Other sexu	Number	2009	0	0	0
Incidence	Global	Both	65-69 yea	Other sexu	Rate	2009	0	0	0
Incidence	Global	Both	65-69 yea	Other sexu	Number	2010	0	0	0
Incidence	Global	Both	65-69 yea	Other sexu	Rate	2010	0	0	0
Incidence	Global	Both	65-69 yea	Other sexu	Number	2011	0	0	0
Incidence	Global	Both	65-69 yea	Other sexu	Rate	2011	0	0	0
Incidence	Global	Both	65-69 yea	Other sexu	Number	2012	0	0	0
Incidence	Global	Both	65-69 yea	Other sexu	Rate	2012	0	0	0
Incidence	Global	Both	65-69 yea	Other sexu	Number	2013	0	0	0
Incidence	Global	Both	65-69 yea	Other sexu	Rate	2013	0	0	0
Incidence	Global	Both	65-69 yea	Other sexu	Number	2014	0	0	0
Incidence	Global	Both	65-69 yea	Other sexu	Rate	2014	0	0	0
Incidence	Global	Both	65-69 yea	Other sexu	Number	2015	0	0	0
Incidence	Global	Both	65-69 yea	Other sexu	Rate	2015	0	0	0
Incidence	Global	Both	65-69 yea	Other sexu	Number	2016	0	0	0
Incidence	Global	Both	65-69 yea	Other sexu	Rate	2016	0	0	0
Incidence	Global	Both	65-69 yea	Other sexu	Number	2017	0	0	0
Incidence	Global	Both	65-69 yea	Other sexu	Rate	2017	0	0	0
Incidence	Global	Both	65-69 yea	Other sexu	Number	2018	0	0	0
Incidence	Global	Both	65-69 yea	Other sexu	Rate	2018	0	0	0
Incidence	Global	Both	65-69 yea	Other sexu	Number	2019	0	0	0
Incidence	Global	Both	65-69 yea	Other sexu	Rate	2019	0	0	0
Incidence	Global	Both	50-69 yea	Other sexu	Number	1990	0	0	0
Incidence	Global	Both	50-69 yea	Other sexu	Rate	1990	0	0	0
Incidence	Global	Both	50-69 yea	Other sexu	Number	1991	0	0	0
Incidence	Global	Both	50-69 yea	Other sexu	Rate	1991	0	0	0
Incidence	Global	Both	50-69 yea	Other sexu	Number	1992	0	0	0
Incidence	Global	Both	50-69 yea	Other sexu	Rate	1992	0	0	0
Incidence	Global	Both	50-69 yea	Other sexu	Number	1993	0	0	0
Incidence	Global	Both	50-69 yea	Other sexu	Rate	1993	0	0	0
Incidence	Global	Both	50-69 yea	Other sexu	Number	1994	0	0	0
Incidence	Global	Both	50-69 yea	Other sexu	Rate	1994	0	0	0
Incidence	Global	Both	50-69 yea	Other sexu	Number	1995	0	0	0
Incidence	Global	Both	50-69 yea	Other sexu	Rate	1995	0	0	0
Incidence	Global	Both	50-69 yea	Other sexu	Number	1996	0	0	0
Incidence	Global	Both	50-69 yea	Other sexu	Rate	1996	0	0	0
Incidence	Global	Both	50-69 yea	Other sexu	Number	1997	0	0	0
Incidence	Global	Both	50-69 yea	Other sexu	Rate	1997	0	0	0
Incidence	Global	Both	50-69 yea	Other sexu	Number	1998	0	0	0
Incidence	Global	Both	50-69 yea	Other sexu	Rate	1998	0	0	0
Incidence	Global	Both	50-69 yea	Other sexu	Number	1999	0	0	0
Incidence	Global	Both	50-69 yea	Other sexu	Rate	1999	0	0	0
Incidence	Global	Both	50-69 yea	Other sexu	Number	2000	0	0	0
Incidence	Global	Both	50-69 yea	Other sexu	Rate	2000	0	0	0
Incidence	Global	Both	50-69 yea	Other sexu	Number	2001	0	0	0
Incidence	Global	Both	50-69 yea	Other sexu	Rate	2001	0	0	0
Incidence	Global	Both	50-69 yea	Other sexu	Number	2002	0	0	0
Incidence	Global	Both	50-69 yea	Other sexu	Rate	2002	0	0	0
Incidence	Global	Both	50-69 yea	Other sexu	Number	2003	0	0	0
Incidence	Global	Both	50-69 yea	Other sexu	Rate	2003	0	0	0
Incidence	Global	Both	50-69 yea	Other sexu	Number	2004	0	0	0
Incidence	Global	Both	50-69 yea	Other sexu	Rate	2004	0	0	0
Incidence	Global	Both	50-69 yea	Other sexu	Number	2005	0	0	0
Incidence	Global	Both	50-69 yea	Other sexu	Rate	2005	0	0	0
Incidence	Global	Both	50-69 yea	Other sexu	Number	2006	0	0	0
Incidence	Global	Both	50-69 yea	Other sexu	Rate	2006	0	0	0
Incidence	Global	Both	50-69 yea	Other sexu	Number	2007	0	0	0
Incidence	Global	Both	50-69 yea	Other sexu	Rate	2007	0	0	0
Incidence	Global	Both	50-69 yea	Other sexu	Number	2008	0	0	0
Incidence	Global	Both	50-69 yea	Other sexu	Rate	2008	0	0	0
Incidence	Global	Both	50-69 yea	Other sexu	Number	2009	0	0	0
Incidence	Global	Both	50-69 yea	Other sexu	Rate	2009	0	0	0
Incidence	Global	Both	50-69 yea	Other sexu	Number	2010	0	0	0
Incidence	Global	Both	50-69 yea	Other sexu	Rate	2010	0	0	0
Incidence	Global	Both	50-69 yea	Other sexu	Number	2011	0	0	0
Incidence	Global	Both	50-69 yea	Other sexu	Rate	2011	0	0	0
Incidence	Global	Both	50-69 yea	Other sexu	Number	2012	0	0	0
Incidence	Global	Both	50-69 yea	Other sexu	Rate	2012	0	0	0
Incidence	Global	Both	50-69 yea	Other sexu	Number	2013	0	0	0
Incidence	Global	Both	50-69 yea	Other sexu	Rate	2013	0	0	0
Incidence	Global	Both	50-69 yea	Other sexu	Number	2014	0	0	0
Incidence	Global	Both	50-69 yea	Other sexu	Rate	2014	0	0	0
Incidence	Global	Both	50-69 yea	Other sexu	Number	2015	0	0	0
Incidence	Global	Both	50-69 yea	Other sexu	Rate	2015	0	0	0
Incidence	Global	Both	50-69 yea	Other sexu	Number	2016	0	0	0
Incidence	Global	Both	50-69 yea	Other sexu	Rate	2016	0	0	0

Incidence	Global	Both	50-54 yea	Trichomon	Number	2006	2.3E+07	3.5E+07	1.4E+07
Incidence	Global	Both	50-54 yea	Trichomon	Rate	2006	7113.71	10904.3	4176.9
Incidence	Global	Both	50-54 yea	Trichomon	Number	2007	2.4E+07	3.6E+07	1.4E+07
Incidence	Global	Both	50-54 yea	Trichomon	Rate	2007	7129.81	10933.8	4185.49
Incidence	Global	Both	50-54 yea	Trichomon	Number	2008	2.4E+07	3.7E+07	1.4E+07
Incidence	Global	Both	50-54 yea	Trichomon	Rate	2008	7143.42	10956	4191.08
Incidence	Global	Both	50-54 yea	Trichomon	Number	2009	2.4E+07	3.7E+07	1.4E+07
Incidence	Global	Both	50-54 yea	Trichomon	Rate	2009	7152.51	10965.7	4194.87
Incidence	Global	Both	50-54 yea	Trichomon	Number	2010	2.5E+07	3.8E+07	1.4E+07
Incidence	Global	Both	50-54 yea	Trichomon	Rate	2010	7156.69	10962.4	4196.24
Incidence	Global	Both	50-54 yea	Trichomon	Number	2011	2.5E+07	3.8E+07	1.5E+07
Incidence	Global	Both	50-54 yea	Trichomon	Rate	2011	7148.8	10957.8	4189.07
Incidence	Global	Both	50-54 yea	Trichomon	Number	2012	2.6E+07	3.9E+07	1.5E+07
Incidence	Global	Both	50-54 yea	Trichomon	Rate	2012	7130.84	10935.5	4175.31
Incidence	Global	Both	50-54 yea	Trichomon	Number	2013	2.6E+07	4E+07	1.5E+07
Incidence	Global	Both	50-54 yea	Trichomon	Rate	2013	7111.41	10907.3	4160.88
Incidence	Global	Both	50-54 yea	Trichomon	Number	2014	2.7E+07	4.2E+07	1.6E+07
Incidence	Global	Both	50-54 yea	Trichomon	Rate	2014	7098.91	10887	4151.18
Incidence	Global	Both	50-54 yea	Trichomon	Number	2015	2.8E+07	4.4E+07	1.7E+07
Incidence	Global	Both	50-54 yea	Trichomon	Rate	2015	7101.17	10891.1	4150.79
Incidence	Global	Both	50-54 yea	Trichomon	Number	2016	3E+07	4.5E+07	1.7E+07
Incidence	Global	Both	50-54 yea	Trichomon	Rate	2016	7171.38	10985.8	4184.97
Incidence	Global	Both	50-54 yea	Trichomon	Number	2017	3.1E+07	4.7E+07	1.8E+07
Incidence	Global	Both	50-54 yea	Trichomon	Rate	2017	7245.48	11077.9	4237.87
Incidence	Global	Both	50-54 yea	Trichomon	Number	2018	3.1E+07	4.8E+07	1.8E+07
Incidence	Global	Both	50-54 yea	Trichomon	Rate	2018	7264.43	11123.6	4248.44
Incidence	Global	Both	50-54 yea	Trichomon	Number	2019	3.2E+07	4.9E+07	1.9E+07
Incidence	Global	Both	50-54 yea	Trichomon	Rate	2019	7275.64	11134	4260.62
Incidence	Global	Both	55-59 yea	Trichomon	Number	1990	8472253	1.3E+07	5440046
Incidence	Global	Both	55-59 yea	Trichomon	Rate	1990	4569.33	6832.26	2933.98
Incidence	Global	Both	55-59 yea	Trichomon	Number	1991	8596690	1.3E+07	5520265
Incidence	Global	Both	55-59 yea	Trichomon	Rate	1991	4573.76	6857.4	2936.99
Incidence	Global	Both	55-59 yea	Trichomon	Number	1992	8744225	1.3E+07	5614044
Incidence	Global	Both	55-59 yea	Trichomon	Rate	1992	4573.09	6850.54	2936.06
Incidence	Global	Both	55-59 yea	Trichomon	Number	1993	8881851	1.3E+07	5701355
Incidence	Global	Both	55-59 yea	Trichomon	Rate	1993	4569.94	6838.74	2933.5
Incidence	Global	Both	55-59 yea	Trichomon	Number	1994	9004737	1.3E+07	5780925
Incidence	Global	Both	55-59 yea	Trichomon	Rate	1994	4567.69	6828.9	2932.4
Incidence	Global	Both	55-59 yea	Trichomon	Number	1995	9122685	1.4E+07	5853963
Incidence	Global	Both	55-59 yea	Trichomon	Rate	1995	4566.22	6820.75	2930.11
Incidence	Global	Both	55-59 yea	Trichomon	Number	1996	9213364	1.4E+07	5919898
Incidence	Global	Both	55-59 yea	Trichomon	Rate	1996	4567.27	6827.4	2934.62
Incidence	Global	Both	55-59 yea	Trichomon	Number	1997	9280026	1.4E+07	5960365
Incidence	Global	Both	55-59 yea	Trichomon	Rate	1997	4574.68	6842.46	2938.22
Incidence	Global	Both	55-59 yea	Trichomon	Number	1998	9339703	1.4E+07	5992151
Incidence	Global	Both	55-59 yea	Trichomon	Rate	1998	4586.88	6865.1	2942.84
Incidence	Global	Both	55-59 yea	Trichomon	Number	1999	9413181	1.4E+07	6036637
Incidence	Global	Both	55-59 yea	Trichomon	Rate	1999	4598.19	6887.88	2948.8
Incidence	Global	Both	55-59 yea	Trichomon	Number	2000	9493916	1.4E+07	6088381
Incidence	Global	Both	55-59 yea	Trichomon	Rate	2000	4611.97	6916.06	2957.62
Incidence	Global	Both	55-59 yea	Trichomon	Number	2001	9680476	1.5E+07	6207689
Incidence	Global	Both	55-59 yea	Trichomon	Rate	2001	4624.54	6936.8	2965.53
Incidence	Global	Both	55-59 yea	Trichomon	Number	2002	1E+07	1.5E+07	6451779
Incidence	Global	Both	55-59 yea	Trichomon	Rate	2002	4619.58	6931.74	2962.17
Incidence	Global	Both	55-59 yea	Trichomon	Number	2003	1E+07	1.6E+07	6721768
Incidence	Global	Both	55-59 yea	Trichomon	Rate	2003	4609.15	6911.27	2956.29
Incidence	Global	Both	55-59 yea	Trichomon	Number	2004	1.1E+07	1.7E+07	7073395
Incidence	Global	Both	55-59 yea	Trichomon	Rate	2004	4601.13	6892.6	2953.34
Incidence	Global	Both	55-59 yea	Trichomon	Number	2005	1.1E+07	1.7E+07	7358881
Incidence	Global	Both	55-59 yea	Trichomon	Rate	2005	4589.23	6866.54	2948.45
Incidence	Global	Both	55-59 yea	Trichomon	Number	2006	1.2E+07	1.8E+07	7725219
Incidence	Global	Both	55-59 yea	Trichomon	Rate	2006	4585.19	6872.38	2942.37
Incidence	Global	Both	55-59 yea	Trichomon	Number	2007	1.2E+07	1.9E+07	8008590
Incidence	Global	Both	55-59 yea	Trichomon	Rate	2007	4590.45	6890.42	2945.09
Incidence	Global	Both	55-59 yea	Trichomon	Number	2008	1.3E+07	1.9E+07	8310485
Incidence	Global	Both	55-59 yea	Trichomon	Rate	2008	4598.24	6909.44	2946.93
Incidence	Global	Both	55-59 yea	Trichomon	Number	2009	1.3E+07	2E+07	8592231
Incidence	Global	Both	55-59 yea	Trichomon	Rate	2009	4605.8	6921.82	2951.48
Incidence	Global	Both	55-59 yea	Trichomon	Number	2010	1.4E+07	2.1E+07	8975599
Incidence	Global	Both	55-59 yea	Trichomon	Rate	2010	4616	6935.32	2958.33
Incidence	Global	Both	55-59 yea	Trichomon	Number	2011	1.4E+07	2.2E+07	9213185
Incidence	Global	Both	55-59 yea	Trichomon	Rate	2011	4620.62	6942.65	2962.5
Incidence	Global	Both	55-59 yea	Trichomon	Number	2012	1.5E+07	2.2E+07	9414176
Incidence	Global	Both	55-59 yea	Trichomon	Rate	2012	4620.9	6941.06	2964.18
Incidence	Global	Both	55-59 yea	Trichomon	Number	2013	1.5E+07	2.2E+07	9558756
Incidence	Global	Both	55-59 yea	Trichomon	Rate	2013	4618.26	6930.67	2964.72
Incidence	Global	Both	55-59 yea	Trichomon	Number	2014	1.5E+07	2.3E+07	9671907
Incidence	Global	Both	55-59 yea	Trichomon	Rate	2014	4615.41	6918.7	2965.56
Incidence	Global	Both	55-59 yea	Trichomon	Number	2015	1.5E+07	2.3E+07	9791708
Incidence	Global	Both	55-59 yea	Trichomon	Rate	2015	4615.88	6910.91	2966.21
Incidence	Global	Both	55-59 yea	Trichomon	Number	2016	1.6E+07	2.3E+07	1E+07
Incidence	Global	Both	55-59 yea	Trichomon	Rate	2016	4637.94	6956.48	2979.02
Incidence	Global	Both	55-59 yea	Trichomon	Number	2017	1.6E+07	2.4E+07	1E+07
Incidence	Global	Both	55-59 yea	Trichomon	Rate	2017	4662.24	6979.54	2994.6
Incidence	Global	Both	55-59 yea	Trichomon	Number	2018	1.7E+07	2.5E+07	1.1E+07
Incidence	Global	Both	55-59 yea	Trichomon	Rate	2018	4667.74	6993.56	2994.41
Incidence	Global	Both	55-59 yea	Trichomon	Number	2019	1.7E+07	2.6E+07	1.1E+07
Incidence	Global	Both	55-59 yea	Trichomon	Rate	2019	4668.33	7006.05	2993.1
Incidence	Global	Both	60-64 yea	Trichomon	Number	1990	4837856	6530547	3539923
Incidence	Global	Both	60-64 yea	Trichomon	Rate	1990	3011.56	4065.25	2203.6
Incidence	Global	Both	60-64 yea	Trichomon	Number	1991	4937011	6659603	3614410
Incidence	Global	Both	60-64 yea	Trichomon	Rate	1991	3010.61	4061.06	2204.08
Incidence	Global	Both	60-64 yea	Trichomon	Number	1992	5009035	6748842	3665942
Incidence	Global	Both	60-64 yea	Trichomon	Rate	1992	3011.29	4057.22	2203.86
Incidence	Global	Both	60-64 yea	Trichomon	Number	1993	5071021	6823810	3710025
Incidence	Global	Both	60-64 yea	Trichomon	Rate	1993	3011.94	4053.01	2203.57
Incidence	Global	Both	60-64 yea	Trichomon	Number	1994	5126464	6889847	3743949
Incidence	Global	Both	60-64 yea	Trichomon	Rate	1994	3014.93	4052	2201.86
Incidence	Global	Both	60-64 yea	Trichomon	Number	1995	5189882	6967362	3788112
Incidence	Global	Both	60-64 yea	Trichomon	Rate	1995	3017.41	4050.84	2202.41
Incidence	Global	Both	60-64 yea	Trichomon	Number	1996	5271010	7086384	3846544
Incidence	Global	Both	60-64 yea	Trichomon	Rate	1996	3019.35	4059.23	2203.38
Incidence	Global	Both	60-64 yea	Trichomon	Number	1997	5371188	7235908	3921242
Incidence	Global	Both	60-64 yea	Trichomon	Rate	1997	3019.32	4067.54	2204.26
Incidence	Global	Both	60-64 yea	Trichomon	Number	1998	5468610	7373955	3989649
Incidence	Global	Both	60-64 yea	Trichomon	Rate	1998	3018.45	4070.12	2202.12
Incidence	Global	Both	60-64 yea	Trichomon	Number	1999	5556699	7490918	4053799
Incidence	Global	Both	60-64 yea	Trichomon	Rate	1999	3017.72	4068.15	2201.53
Incidence	Global	Both	60-64 yea	Trichomon	Number	2000	5639819	7599861	4110477
Incidence	Global	Both	60-64 yea	Trichomon	Rate	2000	3016.65	4065.04	2198.63
Incidence	Global	Both	60-64 yea	Trichomon	Number	2001	5704948	7693940	4165674
Incidence	Global	Both	60-64 yea	Trichomon	Rate	2001	3016.4	4068.05	2202.54
Incidence	Global	Both	60-64 yea	Trichomon	Number	2002	5754298	7764410	4199785
Incidence	Global	Both	60-64 yea	Trichomon	Rate	2002	3019.77	4074.65	2203.99
Incidence	Global	Both	60-64 yea	Trichomon	Number	2003	5801841	7830511	4233745
Incidence	Global	Both	60-64 yea	Trichomon	Rate	2003	3026.15	4084.27	2208.25
Incidence	Global	Both	60-64 yea	Trichomon	Number	2004	5860839	7910825	4281006
Incidence	Global	Both	60-64 yea	Trichomon	Rate	2004	3031.85	4092.32	2214.59
Incidence	Global	Both	60-64 yea	Trichomon	Number	2005	5926336	8000028	4336751
Incidence	Global	Both	60-64 yea	Trichomon	Rate	2005	3038.98	4102.35	2223.85
Incidence	Global	Both	60-64 yea	Trichomon	Number	2006	6056910	8176834	4433528
Incidence	Global	Both	60-64 yea	Trichomon	Rate	2006	3045.67	4111.65	2229.36
Incidence	Global	Both	60-64 yea	Trichomon	Number	2007	6313849	8525897	4615391
Incidence	Global	Both	60-64 yea	Trichomon	Rate	2007	3041.96	4107.71	2223.66
Incidence	Global	Both	60-64 yea	Trichomon	Number	2008	6590665	8903604	4819221
Incidence	Global	Both	60-64 yea	Trichomon	Rate	2008	3034.99	4100.1	2219.25
Incidence	Global	Both	60-64 yea	Trichomon	Number	2009	6942252	9382699	5075363
Incidence	Global	Both	60-64 yea	Trichomon	Rate	2009	3030.41	4095.71	2215.48
Incidence	Global	Both	60-64 yea	Trichomon	Number	2010	7224494	9771040	5278985
Incidence	Global	Both	60-64 yea	Trichomon	Rate	2010	3023.81	4089.66	2209.51
Incidence	Global	Both	60-64 yea	Trichomon	Number	2011	7602197	1E+07	5551742
Incidence	Global	Both	60-64 yea	Trichomon	Rate	2011	3021.87	4090.51	2206.82
Incidence	Global	Both	60-64 yea	Trichomon	Number	2012	7886812	1.1E+07	5763256
Incidence	Global	Both	60-64 yea	Trichomon	Rate	2012	3025.41	4098.87	2210.81
Incidence	Global	Both	60-64 yea	Trichomon	Number	2013	8195784	1.1E+07	5989242
Incidence	Global	Both	60-64 yea	Trichomon	Rate	2013	3030.65	4104.67	2214.71
Incidence	Global	Both	60-64 yea	Trichomon	Number	2014	8477524	1.1E+07	6191405
Incidence	Global	Both	60-64 yea	Trichomon	Rate	2014	3035.79	4102.01	2217.14
Incidence	Global	Both	60-64 yea	Trichomon	Number	2015	8857907	1.2E+07	6465047
Incidence	Global	Both	60-64 yea	Trichomon	Rate	2015	3043.62	4106.61	2221.42
Incidence	Global	Both	60-64 yea	Trichomon	Number	2016	9113809	1.2E+07	6664885
Incidence	Global	Both	60-64 yea	Trichomon	Rate	2016	3055.66	4126.78	2234.59
Incidence	Global	Both	60-64 yea	Trichomon	Number	2017	9339096	1.3E+07	6834990
Incidence	Global	Both	60-64 yea	Trichomon	Rate	2017	3066.84	4132.57	2244.53
Incidence	Global	Both	60-64 yea	Trichomon	Number	2018	9483864	1.3E+07	6923613
Incidence	Global	Both	60-64 yea	Trichomon	Rate	2018	3068.69	4128.46	2240.27
Incidence	Global	Both	60-64 yea	Trichomon	Number	2019	9581412	1.3E+07	6976975
Incidence	Global	Both	60-64 yea	Trichomon	Rate	2019	3065.7	4115.54	2232.38
Incidence	Global	Both	65-69 yea	Trichomon	Number	1990	3184604	4294290	2328345
Incidence	Global	Both	65-69 yea	Trichomon	Rate	1990	2578.87	3477.48	1885.47
Incidence	Global	Both	65-69 yea	Trichomon	Number	1991	3277881	4415218	2398167
Incidence	Global	Both	65-69 yea	Trichomon	Rate	1991	2575.48	3469.1	1884.27
Incidence	Global	Both	65-69 yea	Trichomon	Number	1992	3372819	4537118	2468068
Incidence	Global	Both	65-69 yea	Trichomon	Rate	1992	2572.57	3460.62	1882.48
Incidence	Global	Both	65-69 yea	Trichomon	Number	1993	3470981	4663082	2540369
Incidence	Global	Both	65-69 yea	Trichomon	Rate	1993	2572.24	3455.66	1882.59
Incidence	Global	Both	65-69 yea	Trichomon	Number	1994	3569977	4795155	2614130
Incidence	Global	Both	65-69 yea	Trichomon	Rate	1994	2573.06	3456.11	1884.14
Incidence	Global	Both	65-69 yea	Trichomon	Number	1995	3659771	4915224	2678642
Incidence	Global	Both	65-69 yea	Trichomon	Rate	1995	2576.12	3459.84	1885.5
Incidence	Global	Both	65-69 yea	Trichomon	Number	1996	3742087	5025643	2735531
Incidence	Global	Both	65-69 yea	Trichomon	Rate	1996	2579.24	3463.94	1885.47
Incidence	Global	Both	65-69 yea	Trichomon	Number	1997	3805634	5117936	2779179
Incidence	Global	Both	65-69 yea	Trichomon	Rate	1997	2583.83	3474.81	1886.92
Incidence	Global	Both	65-69 yea	Trichomon	Number	1998	3863026	5205709	2821126
Incidence	Global	Both	65-69 yea	Trichomon	Rate	1998	2587.88	3487.36	1889.9
Incidence	Global	Both	65-69 yea	Trichomon	Number	1999	3916251	5276617	2855618
Incidence	Global	Both	65-69 yea	Trichomon	Rate	1999	2592.59	3493.17	1890.44
Incidence	Global	Both	65-69 yea	Trichomon	Number	2000	3975010	5354162	2896673
Incidence	Global	Both	65-69 yea	Trichomon	Rate	2000	2595.02	3495.38	1891.05
Incidence	Global	Both	65-69 yea	Trichomon	Number	2001	4045923	5454002	2951672
Incidence	Global	Both	65-69 yea	Trichomon	Rate	2001	2595.04	3498.17	1893.19
Incidence	Global	Both	65-69 yea	Trichomon	Number	2002	4129908	5569946	3018532
Incidence	Global	Both	65-69 yea	Trichomon	Rate	2002	2592.2	3496.07	1894.63
Incidence	Global	Both	65-69 yea	Trichomon	Number	2003	4210600	5679822	3076362
Incidence	Global	Both	65-69 yea	Trichomon	Rate	2003	2588.47	3491.67	1891.19
Incidence	Global	Both	65-69 yea	Trichomon	Number	2004	4283751	5778406	3128476
Incidence	Global	Both	65-69 yea	Trichomon	Rate	2004	2585.41	3487.49	1888.15
Incidence	Global	Both	65-69 yea	Trichomon	Number	2005	4355594	5872552	3183765
Incidence	Global	Both	65-69 yea	Trichomon	Rate	2005	2583.59	3483.4	1888.5
Incidence	Global	Both	65-69 yea	Trichomon	Number	2006	4417849	5958928	3230066
Incidence	Global	Both	65-69 yea	Trichomon	Rate	2006	2583.83	3485.15	1889.14
Incidence	Global	Both	65-69 yea	Trichomon	Number	2007	4470481	6033544	3268972
Incidence	Global	Both	65-69 yea	Trichomon	Rate	2007	2587.73	3492.51	1892.24
Incidence	Global	Both	65-69 yea	Trichomon	Number	2008	4523762	6106819	3306184
Incidence	Global	Both	65-69 yea	Trichomon	Rate	2008	2594.35	3502.22	1896.08
Incidence	Global	Both	65-69 yea	Trichomon	Number	2009	4585662	6194369	3351181
Incidence	Global	Both	65-69 yea	Trichomon	Rate	2009	2600.42	3512.68	1900.38
Incidence	Global	Both	65-69 yea	Trichomon	Number	2010	4650570	6285491	3397693
Incidence	Global	Both	65-69 yea	Trichomon	Rate	2010	2607.69	3524.44	1905.17
Incidence	Global	Both	65-69 yea	Trichomon	Number	2011	4764550	6443756	3483034
Incidence	Global	Both	65-69 yea	Trichomon	Rate	2011	2614.7	3536.22	1911.43
Incidence	Global	Both	65-69 yea	Trichomon	Number	2012	4981676	6743411	3639576
Incidence	Global	Both	65-69 yea	Trichomon	Rate	2012	2613.14	3537.26	1909.14
Incidence	Global	Both	65-69 yea	Trichomon	Number	2013	5214264	7061963	3809292
Incidence	Global	Both	65-69 yea	Trichomon	Rate	2013	2609.04	3533.57	1906.04
Incidence	Global	Both	65-69 yea	Trichomon	Number	2014	5507582	7447675	4022730
Incidence	Global	Both	65-69 yea	Trichomon	Rate	2014	2607.33	3525.78	1904.39
Incidence	Global	Both	65-69 yea	Trichomon	Number	2015	5741692	7744993	4194371
Incidence	Global	Both	65-69 yea	Trichomon	Rate	2015	2603.47	3511.83	1901.86
Incidence	Global	Both	65-69 yea	Trichomon	Number	2016	6063302	8182941	4435764
Incidence	Global	Both	65-69 yea	Trichomon	Rate	2016	2607.84	3519.5	1907.83
Incidence	Global	Both	65-69 yea	Trichomon	Number	2017	6310624	8503240	4618801
Incidence	Global	Both	65-69 yea	Trichomon	Rate	2017	2617.11	3526.43	1915.49
Incidence	Global	Both	65-69 yea	Trichomon	Number	2018	6561821	8828152	4789614
Incidence	Global	Both	65-69 yea	Trichomon	Rate	2018	2621.65	3527.11	1913.6
Incidence	Global	Both	65-69 yea	Trichomon	Number	2019	6780263	9100435	4935688
Incidence	Global	Both	65-69 yea	Trichomon	Rate	2019	2622.08	3519.34	1908.74
Incidence	Global	Both	50-69 yea	Trichomon	Number	1990	3.1E+07	4.3E+07	2.2E+07
Incidence	Global	Both	50-69 yea	Trichomon	Rate	1990	4603.91	6329.55	3285.21
Incidence	Global	Both	50-69 yea	Trichomon	Number	1991	3.2E+07	4.4E+07	2.3E+07
Incidence	Global	Both	50-69 yea	Trichomon	Rate	1991	4598.1	6319.74	3284.91
Incidence	Global	Both	50-69 yea	Trichomon	Number	1992	3.2E+07	4.4E+07	2.3E+07
Incidence	Global	Both	50-69 yea	Trichomon	Rate	1992	4593.36	6311.51	3285.03
Incidence	Global	Both	50-69 yea	Trichomon	Number	1993	3.3E+07	4.5E+07	2.3E+07
Incidence	Global	Both	50-69 yea	Trichomon	Rate	1993	4589.41	6303.89	3286.09
Incidence	Global	Both	50-69 yea	Trichomon	Number	1994	3.3E+07	4.6E+07	2.4E+07
Incidence	Global	Both	50-69 yea	Trichomon	Rate	1994	4587.53	6299.44	3288.92
Incidence	Global	Both	50-69 yea	Trichomon	Number	1995	3.4E+07	4.6E+07	2.4E+07
Incidence	Global	Both	50-69 yea	Trichomon	Rate	1995	4587.11	6297.7	3292.63
Incidence	Global	Both	50-69 yea	Trichomon	Number	1996	3.4E+07	4.7E+07	2.5E+07
Incidence	Global	Both	50-69 yea	Trichomon	Rate	1996	4592.37	6300.63	3294.69
Incidence	Global	Both	50-69 yea	Trichomon	Number	1997	3.5E+07	4.8E+07	2.5E+07
Incidence	Global	Both	50-69 yea	Trichomon	Rate	1997	4605.54	6323.61	3302.9
Incidence	Global	Both	50-69 yea	Trichomon	Number	1998	3.6E+07	4.9E+07	2.6E+07
Incidence	Global	Both	50-69 yea	Trichomon	Rate	1998	4619.29	6344.12	3309.51
Incidence	Global	Both	50-69 yea	Trichomon	Number	1999	3.7E+07	5.1E+07	2.6E+07
Incidence	Global	Both	50-69 yea	Trichomon	Rate	1999	4642.86	6382.01	3319.04
Incidence	Global	Both	50-69 yea	Trichomon	Number	2000	3.8E+07	5.2E+07	2.7E+07
Incidence	Global	Both	50-69 yea	Trichomon	Rate	2000	4657.78	6407.89	3323.27
Incidence	Global	Both	50-69 yea	Trichomon	Number	2001	3.9E+07	5.4E+07	2.8E+07
Incidence	Global	Both	50-69 yea	Trichomon	Rate	2001	4685.07	6451.83	3342.47
Incidence	Global	Both	50-69 yea	Trichomon	Number	2002	4E+07	5.5E+07	2.9E+07
Incidence	Global	Both	50-69 yea	Trichomon	Rate	2002	4700.53	6482.13	3356.24
Incidence	Global	Both	50-69 yea	Trichomon	Number	2003	4.1E+07	5.7E+07	3E+07
Incidence	Global	Both	50-69 yea	Trichomon	Rate	2003	4717.99	6518.18	3367.79
Incidence	Global	Both	50-69 yea	Trichomon	Number	2004	4.3E+07	5.9E+07	3E+07
Incidence	Global	Both	50-69 yea	Trichomon	Rate	2004	4732.22	6556.12	3368.39
Incidence	Global	Both	50-69 yea	Trichomon	Number	2005	4.4E+07	6.1E+07	3.1E+07
Incidence	Global	Both	50-69 yea	Trichomon	Rate	2005	4755.67	6598.49	3377.23
Incidence	Global	Both	50-69 yea	Trichomon	Number	2006	4.6E+07	6.3E+07	3.2E+07
Incidence	Global	Both	50-69 yea	Trichomon	Rate	2006	4764.77	6622.19	3378.49
Incidence	Global	Both	50-69 yea	Trichomon	Number	2007	4.7E+07	6.5E+07	3.3E+07
Incidence	Global	Both	50-69 yea	Trichomon	Rate	2007	4766.53	6628.73	3378.76
Incidence	Global	Both	50-69 yea	Trichomon	Number	2008	4.8E+07	6.7E+07	3.4E+07
Incidence	Global	Both	50-69 yea	Trichomon	Rate	2008	4762.37	6626.79	3374.23
Incidence	Global	Both	50-69 yea	Trichomon	Number	2009	4.9E+07	6.8E+07	3.5E+07
Incidence	Global	Both	50-69 yea	Trichomon	Rate	2009	4750.33	6609.88	3366
Incidence	Global	Both	50-69 yea	Trichomon	Number	2010	5E+07	7E+07	3.6E+07
Incidence	Global	Both	50-69 yea	Trichomon	Rate	2010	4741.61	6580.43	3360.11
Incidence	Global	Both	50-69 yea	Trichomon	Number	2011	5.2E+07	7.2E+07	3.7E+07
Incidence	Global	Both	50-69 yea	Trichomon	Rate	2011	4726.12	6540.47	3355.79
Incidence	Global	Both	50-69 yea	Trichomon	Number	2012	5.3E+07	7.3E+07	3.8E+07
Incidence	Global	Both	50-69 yea	Trichomon	Rate	2012	4710.05	6508.63	3348.22
Incidence	Global	Both	50-69 yea	Trichomon	Number	2013	5.5E+07	7.6E+07	3.9E+07
Incidence	Global	Both	50-69 yea	Trichomon	Rate	2013	4698.64	6493.49	3346.83
Incidence	Global	Both	50-69 yea	Trichomon	Number	2014	5.6E+07	7.8E+07	4E+07
Incidence	Global	Both	50-69 yea	Trichomon	Rate	2014	4691.48	6489.61	3346.56
Incidence	Global	Both	50-69 yea	Trichomon	Number	2015	5.8E+07	8.1E+07	4.2E+07
Incidence	Global	Both	50-69 yea	Trichomon	Rate	2015	4689.71	6487.57	3346.64
Incidence	Global	Both	50-69 yea	Trichomon	Number	2016	6E+07	8.3E+07	4.3E+07
Incidence	Global	Both	50-69 yea	Trichomon	Rate	2016	4715.89	6502.85	3361.1
Incidence	Global	Both	50-69 yea	Trichomon	Number	2017	6.2E+07	8.6E+07	4.4E+07
Incidence	Global	Both	50-69 yea	Trichomon	Rate	2017	4747.99	6552.29	3379.64
Incidence	Global	Both	50-69 yea	Trichomon	Number	2018	6.4E+07	8.8E+07	4.6E+07
Incidence	Global	Both	50-69 yea	Trichomon	Rate	2018	4750.71	6566.35	3384.8
Incidence	Global	Both	50-69 yea	Trichomon	Number	2019	6.5E+07	9.1E+07	4.7E+07
Incidence	Global	Both	50-69 yea	Trichomon	Rate	2019	4747.31	6568.99	3380.49
Incidence	Global	Both	50-69 yea	Other sexu	Rate	2018	0	0	0
Incidence	Global	Both	50-69 yea	Other sexu	Number	2019	0	0	0
Incidence	Global	Both	50-69 yea	Other sexu	Rate	2019	0	0	0

HIV

measure	AAPC	AAPC C.I. l	AAPC C.I. l	P-Value
DALYs	3.13	2.72	3.54	0.000
Mortality	2.95	2.51	3.38	0.000
Incidence	-1.53	-1.67	-1.39	0.000
Prevalence	6.44	6.18	6.70	0.000

Other STI

measure	AAPC	AAPC C.I. l	AAPC C.I. l	P-Value
DALYs	-1.11	-1.22	-1.00	0.000
Mortality	-2.59	-2.85	-2.33	0.000
Incidence	0.10	0.06	0.13	0.000
Prevalence	0.05	0.01	0.08	0.005

measure	location	sex	age	cause	metric	year	val	upper	lower
Deaths	Global	Male	50-69 ye	HIV/AIDS	Number	1990	20434.1	27052	15048.6
Deaths	Global	Female	50-69 ye	HIV/AIDS	Number	1990	18099.2	24920.5	12173.4
Deaths	Global	Male	50-69 ye	HIV/AIDS	Rate	1990	6.07188	8.03837	4.47161
Deaths	Global	Female	50-69 ye	HIV/AIDS	Rate	1990	5.23702	7.21075	3.52236
Deaths	Global	Male	50-69 ye	Sexually	Number	1990	1008.58	1450.73	517.195
Deaths	Global	Female	50-69 ye	Sexually	Number	1990	1693.63	1996.6	1238.53
Deaths	Global	Male	50-69 ye	Sexually	Rate	1990	0.2997	0.43108	0.15368
Deaths	Global	Female	50-69 ye	Sexually	Rate	1990	0.49005	0.57771	0.35837
Deaths	Global	Male	50-69 ye	Syphilis	Number	1990	823.397	1169.52	435.651
Deaths	Global	Female	50-69 ye	Syphilis	Number	1990	99.6602	119.444	69.1868
Deaths	Global	Male	50-69 ye	Syphilis	Rate	1990	0.24467	0.34752	0.12945
Deaths	Global	Female	50-69 ye	Syphilis	Rate	1990	0.02884	0.03456	0.02002
Deaths	Global	Male	50-69 ye	Chlamydi	Number	1990	0	0	0
Deaths	Global	Female	50-69 ye	Chlamydi	Number	1990	249.695	295.402	181.955
Deaths	Global	Male	50-69 ye	Chlamydi	Rate	1990	0	0	0
Deaths	Global	Female	50-69 ye	Chlamydi	Rate	1990	0.07225	0.08547	0.05265
Deaths	Global	Male	50-69 ye	Gonococc	Number	1990	56.5622	84.1109	24.4775
Deaths	Global	Female	50-69 ye	Gonococc	Number	1990	814.032	954.965	606.086
Deaths	Global	Male	50-69 ye	Gonococc	Rate	1990	0.01681	0.02499	0.00727
Deaths	Global	Female	50-69 ye	Gonococc	Rate	1990	0.23554	0.27632	0.17537
Deaths	Global	Male	50-69 ye	Other se	Number	1990	128.626	201.393	49.4273
Deaths	Global	Female	50-69 ye	Other se	Number	1990	530.242	629.689	381.44
Deaths	Global	Male	50-69 ye	Other se	Rate	1990	0.03822	0.05984	0.01469
Deaths	Global	Female	50-69 ye	Other se	Rate	1990	0.15343	0.1822	0.11037
Deaths	Global	Male	50-69 ye	HIV/AIDS	Number	1991	25918.6	33873.1	19203.7
Deaths	Global	Female	50-69 ye	HIV/AIDS	Number	1991	23866.7	32525.1	16243.4
Deaths	Global	Male	50-69 ye	HIV/AIDS	Rate	1991	7.56033	9.88061	5.60161
Deaths	Global	Female	50-69 ye	HIV/AIDS	Rate	1991	6.79414	9.25892	4.62402
Deaths	Global	Male	50-69 ye	Sexually	Number	1992	1049.74	1508.31	542.134
Deaths	Global	Female	50-69 ye	Sexually	Number	1992	1724.24	2024.5	1274.38
Deaths	Global	Male	50-69 ye	Sexually	Rate	1992	0.30126	0.43286	0.15558
Deaths	Global	Female	50-69 ye	Sexually	Rate	1992	0.48368	0.56791	0.35749
Deaths	Global	Male	50-69 ye	Syphilis	Number	1992	869.087	1226.89	463.439
Deaths	Global	Female	50-69 ye	Syphilis	Number	1992	100.36	120.04	68.1719
Deaths	Global	Male	50-69 ye	Syphilis	Rate	1992	0.24941	0.3521	0.133
Deaths	Global	Female	50-69 ye	Syphilis	Rate	1992	0.02815	0.03367	0.01912
Deaths	Global	Male	50-69 ye	Chlamydi	Number	1992	0	0	0
Deaths	Global	Female	50-69 ye	Chlamydi	Number	1992	254.023	299.315	188.623
Deaths	Global	Male	50-69 ye	Chlamydi	Rate	1992	0	0	0
Deaths	Global	Female	50-69 ye	Chlamydi	Rate	1992	0.07126	0.08396	0.05291
Deaths	Global	Male	50-69 ye	Gonococc	Number	1992	55.3834	82.8792	24.4394
Deaths	Global	Female	50-69 ye	Gonococc	Number	1992	830.639	970.715	619.152
Deaths	Global	Male	50-69 ye	Gonococc	Rate	1992	0.01589	0.02378	0.00701
Deaths	Global	Female	50-69 ye	Gonococc	Rate	1992	0.23301	0.2723	0.17368
Deaths	Global	Male	50-69 ye	Other se	Number	1992	125.268	199.381	47.5509
Deaths	Global	Female	50-69 ye	Other se	Number	1992	539.221	638.24	394.536
Deaths	Global	Male	50-69 ye	Other se	Rate	1992	0.03595	0.05722	0.01365
Deaths	Global	Female	50-69 ye	Other se	Rate	1992	0.15126	0.17904	0.11067
Deaths	Global	Male	50-69 ye	HIV/AIDS	Number	1992	32362.7	41896.3	23851.4
Deaths	Global	Female	50-69 ye	HIV/AIDS	Number	1992	30704.9	41455.5	21122.7
Deaths	Global	Male	50-69 ye	HIV/AIDS	Rate	1992	9.28755	12.0235	6.84494
Deaths	Global	Female	50-69 ye	HIV/AIDS	Rate	1992	8.61331	11.629	5.92529
Deaths	Global	Male	50-69 ye	Sexually	Number	1993	1070.22	1521.68	562.225
Deaths	Global	Female	50-69 ye	Sexually	Number	1993	1718.61	1992.87	1276.82
Deaths	Global	Male	50-69 ye	Sexually	Rate	1993	0.30261	0.43027	0.15897
Deaths	Global	Female	50-69 ye	Sexually	Rate	1993	0.47593	0.55188	0.35359
Deaths	Global	Male	50-69 ye	Syphilis	Number	1993	892.368	1244.31	486.836
Deaths	Global	Female	50-69 ye	Syphilis	Number	1993	98.9958	117.159	69.375
Deaths	Global	Male	50-69 ye	Syphilis	Rate	1993	0.25232	0.35184	0.13766
Deaths	Global	Female	50-69 ye	Syphilis	Rate	1993	0.02741	0.03244	0.01921
Deaths	Global	Male	50-69 ye	Chlamydi	Number	1993	0	0	0
Deaths	Global	Female	50-69 ye	Chlamydi	Number	1993	253.107	294.019	188.816
Deaths	Global	Male	50-69 ye	Chlamydi	Rate	1993	0	0	0
Deaths	Global	Female	50-69 ye	Chlamydi	Rate	1993	0.07009	0.08142	0.05229
Deaths	Global	Male	50-69 ye	Gonococc	Number	1993	54.6437	80.8169	24.0708
Deaths	Global	Female	50-69 ye	Gonococc	Number	1993	829.954	955.079	622.202
Deaths	Global	Male	50-69 ye	Gonococc	Rate	1993	0.01545	0.02285	0.00681
Deaths	Global	Female	50-69 ye	Gonococc	Rate	1993	0.22984	0.26449	0.17231
Deaths	Global	Male	50-69 ye	Other se	Number	1993	123.208	197.593	46.9114
Deaths	Global	Female	50-69 ye	Other se	Number	1993	536.558	625.261	394.565
Deaths	Global	Male	50-69 ye	Other se	Rate	1993	0.03484	0.05587	0.01326
Deaths	Global	Female	50-69 ye	Other se	Rate	1993	0.14859	0.17315	0.10927
Deaths	Global	Male	50-69 ye	Sexually	Number	1991	1029.4	1497.17	521.877
Deaths	Global	Female	50-69 ye	Sexually	Number	1991	1702.69	2008.05	1241.23
Deaths	Global	Male	50-69 ye	Sexually	Rate	1991	0.30027	0.43672	0.15223
Deaths	Global	Female	50-69 ye	Sexually	Rate	1991	0.4847	0.57163	0.35334
Deaths	Global	Male	50-69 ye	Syphilis	Number	1991	846.871	1202.56	447.718
Deaths	Global	Female	50-69 ye	Syphilis	Number	1991	99.512	119.075	69.3506
Deaths	Global	Male	50-69 ye	Syphilis	Rate	1991	0.24703	0.35078	0.1306
Deaths	Global	Female	50-69 ye	Syphilis	Rate	1991	0.02833	0.0339	0.01974
Deaths	Global	Male	50-69 ye	Chlamydi	Number	1991	0	0	0
Deaths	Global	Female	50-69 ye	Chlamydi	Number	1991	250.843	296.01	183.073
Deaths	Global	Male	50-69 ye	Chlamydi	Rate	1991	0	0	0
Deaths	Global	Female	50-69 ye	Chlamydi	Rate	1991	0.07141	0.08427	0.05212
Deaths	Global	Male	50-69 ye	Gonococc	Number	1991	55.8502	84.0399	24.4813
Deaths	Global	Female	50-69 ye	Gonococc	Number	1991	819.896	958.044	607.628
Deaths	Global	Male	50-69 ye	Gonococc	Rate	1991	0.01629	0.02451	0.00714
Deaths	Global	Female	50-69 ye	Gonococc	Rate	1991	0.2334	0.27273	0.17297
Deaths	Global	Male	50-69 ye	Other se	Number	1991	126.68	201.422	47.7859
Deaths	Global	Female	50-69 ye	Other se	Number	1991	532.439	630.324	382.658
Deaths	Global	Male	50-69 ye	Other se	Rate	1991	0.03695	0.05875	0.01394
Deaths	Global	Female	50-69 ye	Other se	Rate	1991	0.15157	0.17943	0.10893
Deaths	Global	Male	50-69 ye	HIV/AIDS	Number	1994	47902.3	60858.3	35927
Deaths	Global	Female	50-69 ye	HIV/AIDS	Number	1994	47978.2	62632.9	34411.6
Deaths	Global	Male	50-69 ye	HIV/AIDS	Rate	1994	13.3625	16.9766	10.022
Deaths	Global	Female	50-69 ye	HIV/AIDS	Rate	1994	13.1239	17.1325	9.41287
Deaths	Global	Male	50-69 ye	Sexually	Number	1996	1044.5	1504.67	567.246
Deaths	Global	Female	50-69 ye	Sexually	Number	1996	1681.09	1932.68	1245.71
Deaths	Global	Male	50-69 ye	Sexually	Rate	1996	0.28356	0.40849	0.154
Deaths	Global	Female	50-69 ye	Sexually	Rate	1996	0.44783	0.51485	0.33184
Deaths	Global	Male	50-69 ye	Syphilis	Number	1996	880.283	1242.58	497.704
Deaths	Global	Female	50-69 ye	Syphilis	Number	1996	98.3238	115.329	68.8044
Deaths	Global	Male	50-69 ye	Syphilis	Rate	1996	0.23898	0.33733	0.13512
Deaths	Global	Female	50-69 ye	Syphilis	Rate	1996	0.02619	0.03072	0.01833
Deaths	Global	Male	50-69 ye	Chlamydi	Number	1996	0	0	0
Deaths	Global	Female	50-69 ye	Chlamydi	Number	1996	246.969	283.627	182.939
Deaths	Global	Male	50-69 ye	Chlamydi	Rate	1996	0	0	0
Deaths	Global	Female	50-69 ye	Chlamydi	Rate	1996	0.06579	0.07556	0.04873
Deaths	Global	Male	50-69 ye	Sexually	Number	1994	1089.57	1542.95	581.209
Deaths	Global	Female	50-69 ye	Sexually	Number	1994	1721.51	1988.28	1282.05
Deaths	Global	Male	50-69 ye	Sexually	Rate	1994	0.30394	0.43041	0.16213
Deaths	Global	Female	50-69 ye	Sexually	Rate	1994	0.4709	0.54387	0.35069
Deaths	Global	Male	50-69 ye	Syphilis	Number	1994	913.553	1272	498.729
Deaths	Global	Female	50-69 ye	Syphilis	Number	1994	99.1346	117.1	69.46
Deaths	Global	Male	50-69 ye	Syphilis	Rate	1994	0.25484	0.35483	0.13912
Deaths	Global	Female	50-69 ye	Syphilis	Rate	1994	0.02712	0.03203	0.019
Deaths	Global	Male	50-69 ye	Chlamydi	Number	1994	0	0	0
Deaths	Global	Female	50-69 ye	Chlamydi	Number	1994	253.401	292.181	189.964
Deaths	Global	Male	50-69 ye	Chlamydi	Rate	1994	0	0	0
Deaths	Global	Female	50-69 ye	Chlamydi	Rate	1994	0.06931	0.07992	0.05196
Deaths	Global	Male	50-69 yea	Gonococca	Number	1994	54.1453	81.0557	24.1566
Deaths	Global	Female	50-69 yea	Gonococca	Number	1994	831.717	957.414	627.207
Deaths	Global	Male	50-69 yea	Gonococca	Rate	1994	0.0151	0.02261	0.00674
Deaths	Global	Female	50-69 yea	Gonococca	Rate	1994	0.22751	0.26189	0.17157
Deaths	Global	Male	50-69 yea	Other sexu	Number	1994	121.872	197.413	46.5965
Deaths	Global	Female	50-69 yea	Other sexu	Number	1994	537.256	622.663	396.72
Deaths	Global	Male	50-69 yea	Other sexu	Rate	1994	0.034	0.05507	0.013
Deaths	Global	Female	50-69 yea	Other sexu	Rate	1994	0.14696	0.17032	0.10852
Deaths	Global	Male	50-69 yea	Gonococca	Number	1996	50.381	77.2427	23.3011
Deaths	Global	Female	50-69 yea	Gonococca	Number	1996	811.106	929.677	611.605
Deaths	Global	Male	50-69 yea	Gonococca	Rate	1996	0.01368	0.02097	0.00633
Deaths	Global	Female	50-69 yea	Gonococca	Rate	1996	0.21607	0.24766	0.16293
Deaths	Global	Male	50-69 yea	Other sexu	Number	1996	113.836	191.643	45.738
Deaths	Global	Female	50-69 yea	Other sexu	Number	1996	524.693	604.099	384.875
Deaths	Global	Male	50-69 yea	Other sexu	Rate	1996	0.0309	0.05203	0.01242
Deaths	Global	Female	50-69 yea	Other sexu	Rate	1996	0.13977	0.16093	0.10253
Deaths	Global	Male	50-69 yea	HIV/AIDS	Number	1993	39660.4	51054	29360.5
Deaths	Global	Female	50-69 yea	HIV/AIDS	Number	1993	38852.5	51648.9	27148.8
Deaths	Global	Male	50-69 yea	HIV/AIDS	Rate	1993	11.2143	14.4359	8.30192
Deaths	Global	Female	50-69 yea	HIV/AIDS	Rate	1993	10.7593	14.303	7.51828
Deaths	Global	Male	50-69 yea	HIV/AIDS	Number	1998	75715.8	91944.5	59618.8
Deaths	Global	Female	50-69 yea	HIV/AIDS	Number	1998	86205.7	104039	67136.2
Deaths	Global	Male	50-69 yea	HIV/AIDS	Rate	1998	19.7477	23.9804	15.5494
Deaths	Global	Female	50-69 yea	HIV/AIDS	Rate	1998	22.0614	26.6251	17.1812
Deaths	Global	Male	50-69 yea	Sexually tr	Number	1997	1003.5	1473.08	549.888
Deaths	Global	Female	50-69 yea	Sexually tr	Number	1997	1706.08	1965.62	1250.85
Deaths	Global	Male	50-69 yea	Sexually tr	Rate	1997	0.267	0.39193	0.1463
Deaths	Global	Female	50-69 yea	Sexually tr	Rate	1997	0.44563	0.51342	0.32672
Deaths	Global	Male	50-69 yea	Syphilis	Number	1997	844.027	1211.8	479.922
Deaths	Global	Female	50-69 yea	Syphilis	Number	1997	100.694	118.585	71.9599
Deaths	Global	Male	50-69 yea	Syphilis	Rate	1997	0.22456	0.32242	0.12769
Deaths	Global	Female	50-69 yea	Syphilis	Rate	1997	0.0263	0.03097	0.0188
Deaths	Global	Male	50-69 yea	Chlamydia	Number	1997	0	0	0
Deaths	Global	Female	50-69 yea	Chlamydia	Number	1997	250.364	287.03	183.655
Deaths	Global	Male	50-69 yea	Chlamydia	Rate	1997	0	0	0
Deaths	Global	Female	50-69 yea	Chlamydia	Rate	1997	0.06539	0.07497	0.04797
Deaths	Global	Male	50-69 yea	Gonococca	Number	1997	48.8177	76.625	23.0373
Deaths	Global	Female	50-69 yea	Gonococca	Number	1997	822.294	940.864	613.22
Deaths	Global	Male	50-69 yea	Gonococca	Rate	1997	0.01299	0.02039	0.00613
Deaths	Global	Female	50-69 yea	Gonococca	Rate	1997	0.21478	0.24575	0.16017
Deaths	Global	Male	50-69 yea	Other sexu	Number	1997	110.659	191.882	44.5098
Deaths	Global	Female	50-69 yea	Other sexu	Number	1997	532.733	613.65	386.909
Deaths	Global	Male	50-69 yea	Other sexu	Rate	1997	0.02944	0.05105	0.01184
Deaths	Global	Female	50-69 yea	Other sexu	Rate	1997	0.13915	0.16029	0.10106
Deaths	Global	Male	50-69 yea	Sexually tr	Number	1995	1087.41	1554.87	582.696
Deaths	Global	Female	50-69 yea	Sexually tr	Number	1995	1678.34	1934.71	1273.08
Deaths	Global	Male	50-69 yea	Sexually tr	Rate	1995	0.29957	0.42835	0.16053
Deaths	Global	Female	50-69 yea	Sexually tr	Rate	1995	0.45366	0.52296	0.34412
Deaths	Global	Male	50-69 yea	Syphilis	Number	1995	915.989	1280.97	510.167
Deaths	Global	Female	50-69 yea	Syphilis	Number	1995	96.7728	113.547	69.4457
Deaths	Global	Male	50-69 yea	Syphilis	Rate	1995	0.25234	0.35289	0.14055
Deaths	Global	Female	50-69 yea	Syphilis	Rate	1995	0.02616	0.03069	0.01877
Deaths	Global	Male	50-69 yea	Chlamydia	Number	1995	0	0	0
Deaths	Global	Female	50-69 yea	Chlamydia	Number	1995	246.83	284.102	188.312
Deaths	Global	Male	50-69 yea	Chlamydia	Rate	1995	0	0	0
Deaths	Global	Female	50-69 yea	Chlamydia	Rate	1995	0.06672	0.07679	0.0509
Deaths	Global	Male	50-69 yea	Gonococca	Number	1995	52.6833	79.8313	23.9176
Deaths	Global	Female	50-69 yea	Gonococca	Number	1995	811.259	930.458	627.319
Deaths	Global	Male	50-69 yea	Gonococca	Rate	1995	0.01451	0.02199	0.00659
Deaths	Global	Female	50-69 yea	Gonococca	Rate	1995	0.21929	0.25151	0.16957
Deaths	Global	Male	50-69 yea	Other sexu	Number	1995	118.738	194.028	45.7023
Deaths	Global	Female	50-69 yea	Other sexu	Number	1995	523.481	604.723	393.875
Deaths	Global	Male	50-69 yea	Other sexu	Rate	1995	0.03271	0.05345	0.01259
Deaths	Global	Female	50-69 yea	Other sexu	Rate	1995	0.1415	0.16346	0.10647
Deaths	Global	Male	50-69 yea	HIV/AIDS	Number	1997	68115.1	83933	52547.5
Deaths	Global	Female	50-69 yea	HIV/AIDS	Number	1997	76598.4	94550.8	58216.9
Deaths	Global	Male	50-69 yea	HIV/AIDS	Rate	1997	18.1229	22.3314	13.9809
Deaths	Global	Female	50-69 yea	HIV/AIDS	Rate	1997	20.0075	24.6966	15.2062
Deaths	Global	Male	50-69 yea	Sexually tr	Number	2004	702.115	1042.48	368.107
Deaths	Global	Female	50-69 yea	Sexually tr	Number	2004	1596.31	1834.62	1257.06
Deaths	Global	Male	50-69 yea	Sexually tr	Rate	2004	0.15733	0.2336	0.08248
Deaths	Global	Female	50-69 yea	Sexually tr	Rate	2004	0.34966	0.40186	0.27535
Deaths	Global	Male	50-69 yea	Syphilis	Number	2004	561.401	836.033	304.429
Deaths	Global	Female	50-69 yea	Syphilis	Number	2004	94.1791	109.921	71.9792
Deaths	Global	Male	50-69 yea	Syphilis	Rate	2004	0.1258	0.18734	0.06822
Deaths	Global	Female	50-69 yea	Syphilis	Rate	2004	0.02063	0.02408	0.01577
Deaths	Global	Male	50-69 yea	Chlamydia	Number	2004	0	0	0
Deaths	Global	Female	50-69 yea	Chlamydia	Number	2004	233.177	267.638	184.459
Deaths	Global	Male	50-69 yea	Chlamydia	Rate	2004	0	0	0
Deaths	Global	Female	50-69 yea	Chlamydia	Rate	2004	0.05108	0.05862	0.0404
Deaths	Global	Male	50-69 yea	HIV/AIDS	Number	1995	56110.2	70346.5	42604
Deaths	Global	Female	50-69 yea	HIV/AIDS	Number	1995	57459.2	73332.8	42049.4
Deaths	Global	Male	50-69 yea	HIV/AIDS	Rate	1995	15.4578	19.3797	11.7369
Deaths	Global	Female	50-69 yea	HIV/AIDS	Rate	1995	15.5315	19.8222	11.3662
Deaths	Global	Male	50-69 yea	Sexually tr	Number	2000	900.802	1315.53	498.631
Deaths	Global	Female	50-69 yea	Sexually tr	Number	2000	1633.52	1867.28	1257.8
Deaths	Global	Male	50-69 yea	Sexually tr	Rate	2000	0.22512	0.32877	0.12462
Deaths	Global	Female	50-69 yea	Sexually tr	Rate	2000	0.40029	0.45757	0.30822
Deaths	Global	Male	50-69 yea	Syphilis	Number	2000	749.053	1068.39	423.472
Deaths	Global	Female	50-69 yea	Syphilis	Number	2000	95.8462	110.962	71.6913
Deaths	Global	Male	50-69 yea	Syphilis	Rate	2000	0.1872	0.26701	0.10583
Deaths	Global	Female	50-69 yea	Syphilis	Rate	2000	0.02349	0.02719	0.01757
Deaths	Global	Male	50-69 yea	Chlamydia	Number	2000	0	0	0
Deaths	Global	Female	50-69 yea	Chlamydia	Number	2000	239.116	273.602	185.863
Deaths	Global	Male	50-69 yea	Chlamydia	Rate	2000	0	0	0
Deaths	Global	Female	50-69 yea	Chlamydia	Rate	2000	0.05859	0.06705	0.04555
Deaths	Global	Male	50-69 yea	Gonococca	Number	2000	46.3053	71.4491	22.3359
Deaths	Global	Female	50-69 yea	Gonococca	Number	2000	789.947	899.349	613.163
Deaths	Global	Male	50-69 yea	Gonococca	Rate	2000	0.01157	0.01786	0.00558
Deaths	Global	Female	50-69 yea	Gonococca	Rate	2000	0.19357	0.22038	0.15025
Deaths	Global	Male	50-69 yea	Other sexu	Number	2000	105.443	175.326	44.9039
Deaths	Global	Female	50-69 yea	Other sexu	Number	2000	508.613	584.173	391.013
Deaths	Global	Male	50-69 yea	Other sexu	Rate	2000	0.02635	0.04382	0.01122
Deaths	Global	Female	50-69 yea	Other sexu	Rate	2000	0.12463	0.14315	0.09582
Deaths	Global	Male	50-69 yea	Gonococca	Number	2004	42.4082	63.7022	21.0197
Deaths	Global	Female	50-69 yea	Gonococca	Number	2004	771.165	884.712	612.95
Deaths	Global	Male	50-69 yea	Gonococca	Rate	2004	0.0095	0.01427	0.00471
Deaths	Global	Female	50-69 yea	Gonococca	Rate	2004	0.16892	0.19379	0.13426
Deaths	Global	Male	50-69 yea	Other sexu	Number	2004	98.3063	157.106	42.8389
Deaths	Global	Female	50-69 yea	Other sexu	Number	2004	497.79	573.907	390.47
Deaths	Global	Male	50-69 yea	Other sexu	Rate	2004	0.02203	0.0352	0.0096
Deaths	Global	Female	50-69 yea	Other sexu	Rate	2004	0.10904	0.12571	0.08553
Deaths	Global	Male	50-69 yea	HIV/AIDS	Number	1996	62478	77588.7	47796.3
Deaths	Global	Female	50-69 yea	HIV/AIDS	Number	1996	67114	84334.3	50013.7
Deaths	Global	Male	50-69 yea	HIV/AIDS	Rate	1996	16.9615	21.0637	12.9757
Deaths	Global	Female	50-69 yea	HIV/AIDS	Rate	1996	17.8785	22.4659	13.3232
Deaths	Global	Male	50-69 yea	HIV/AIDS	Number	2002	108027	121593	94190.8
Deaths	Global	Female	50-69 yea	HIV/AIDS	Number	2002	121175	136717	103797
Deaths	Global	Male	50-69 yea	HIV/AIDS	Rate	2002	25.6171	28.8342	22.3362
Deaths	Global	Female	50-69 yea	HIV/AIDS	Rate	2002	28.1251	31.7323	24.0915
Deaths	Global	Male	50-69 ye	Sexually	Number	1998	961.705	1401.34	522.91
Deaths	Global	Female	50-69 ye	Sexually	Number	1998	1687.98	1937.45	1266.51
Deaths	Global	Male	50-69 ye	Sexually	Rate	1998	0.25083	0.36549	0.13638
Deaths	Global	Female	50-69 ye	Sexually	Rate	1998	0.43198	0.49583	0.32412
Deaths	Global	Male	50-69 ye	Syphilis	Number	1998	805.385	1143.23	459.481
Deaths	Global	Female	50-69 ye	Syphilis	Number	1998	99.8123	116.614	72.3791
Deaths	Global	Male	50-69 ye	Syphilis	Rate	1998	0.21006	0.29817	0.11984
Deaths	Global	Female	50-69 ye	Syphilis	Rate	1998	0.02554	0.02984	0.01852
Deaths	Global	Male	50-69 ye	Chlamydi	Number	1998	0	0	0
Deaths	Global	Female	50-69 ye	Chlamydi	Number	1998	247.308	284.324	186.27
Deaths	Global	Male	50-69 ye	Chlamydi	Rate	1998	0	0	0
Deaths	Global	Female	50-69 ye	Chlamydi	Rate	1998	0.06329	0.07276	0.04767
Deaths	Global	Male	50-69 ye	Gonococc	Number	1998	47.7474	73.7523	22.6969
Deaths	Global	Female	50-69 ye	Gonococc	Number	1998	814.121	931.872	614.83
Deaths	Global	Male	50-69 ye	Gonococc	Rate	1998	0.01245	0.01924	0.00592
Deaths	Global	Female	50-69 ye	Gonococc	Rate	1998	0.20835	0.23848	0.15734
Deaths	Global	Male	50-69 ye	Other se	Number	1998	108.573	181.96	44.5981
Deaths	Global	Female	50-69 ye	Other se	Number	1998	526.738	607.632	391.952
Deaths	Global	Male	50-69 ye	Other se	Rate	1998	0.02832	0.04746	0.01163
Deaths	Global	Female	50-69 ye	Other se	Rate	1998	0.1348	0.1555	0.10031
Deaths	Global	Male	50-69 ye	Sexually	Number	1999	940.18	1370.66	515.493
Deaths	Global	Female	50-69 ye	Sexually	Number	1999	1655.18	1899.67	1272.94
Deaths	Global	Male	50-69 ye	Sexually	Rate	1999	0.23959	0.3493	0.13137
Deaths	Global	Female	50-69 ye	Sexually	Rate	1999	0.4141	0.47527	0.31847
Deaths	Global	Male	50-69 ye	Syphilis	Number	1999	785.704	1115.78	448.505
Deaths	Global	Female	50-69 ye	Syphilis	Number	1999	97.4477	113.699	71.8822
Deaths	Global	Male	50-69 ye	Syphilis	Rate	1999	0.20023	0.28434	0.1143
Deaths	Global	Female	50-69 ye	Syphilis	Rate	1999	0.02438	0.02845	0.01798
Deaths	Global	Male	50-69 ye	Chlamydi	Number	1999	0	0	0
Deaths	Global	Female	50-69 ye	Chlamydi	Number	1999	242.424	278.224	185.254
Deaths	Global	Male	50-69 ye	Chlamydi	Rate	1999	0	0	0
Deaths	Global	Female	50-69 ye	Chlamydi	Rate	1999	0.06065	0.06961	0.04635
Deaths	Global	Male	50-69 ye	Gonococc	Number	1999	47.1425	73.1024	22.3594
Deaths	Global	Female	50-69 ye	Gonococc	Number	1999	799.656	912.805	623.318
Deaths	Global	Male	50-69 ye	Gonococc	Rate	1999	0.01201	0.01863	0.0057
Deaths	Global	Female	50-69 ye	Gonococc	Rate	1999	0.20006	0.22837	0.15595
Deaths	Global	Male	50-69 ye	Other se	Number	1999	107.334	179.16	44.1689
Deaths	Global	Female	50-69 ye	Other se	Number	1999	515.649	593.522	391.408
Deaths	Global	Male	50-69 ye	Other se	Rate	1999	0.02735	0.04566	0.01126
Deaths	Global	Female	50-69 ye	Other se	Rate	1999	0.12901	0.14849	0.09793
Deaths	Global	Male	50-69 ye	HIV/AIDS	Number	2006	119218	129647	109298
Deaths	Global	Female	50-69 ye	HIV/AIDS	Number	2006	129251	139481	119319
Deaths	Global	Male	50-69 ye	HIV/AIDS	Rate	2006	25.2149	27.4206	23.1167
Deaths	Global	Female	50-69 ye	HIV/AIDS	Rate	2006	26.7068	28.8206	24.6545
Deaths	Global	Male	50-69 ye	HIV/AIDS	Number	1999	84202.2	100351	67778.8
Deaths	Global	Female	50-69 ye	HIV/AIDS	Number	1999	95563.5	113410	76006.9
Deaths	Global	Male	50-69 ye	HIV/AIDS	Rate	1999	21.4579	25.5732	17.2726
Deaths	Global	Female	50-69 ye	HIV/AIDS	Rate	1999	23.9087	28.3738	19.0159
Deaths	Global	Male	50-69 ye	Sexually	Number	2008	618.379	884.358	307.653
Deaths	Global	Female	50-69 ye	Sexually	Number	2008	1630.24	1834.01	1265.24
Deaths	Global	Male	50-69 ye	Sexually	Rate	2008	0.12401	0.17735	0.0617
Deaths	Global	Female	50-69 ye	Sexually	Rate	2008	0.31925	0.35916	0.24777
Deaths	Global	Male	50-69 ye	Syphilis	Number	2008	487.688	691.542	248.461
Deaths	Global	Female	50-69 ye	Syphilis	Number	2008	96.6896	110.953	72.0221
Deaths	Global	Male	50-69 ye	Syphilis	Rate	2008	0.0978	0.13869	0.04983
Deaths	Global	Female	50-69 ye	Syphilis	Rate	2008	0.01893	0.02173	0.0141
Deaths	Global	Male	50-69 ye	Chlamydi	Number	2008	0	0	0
Deaths	Global	Female	50-69 ye	Chlamydi	Number	2008	238.702	268.247	184.68
Deaths	Global	Male	50-69 ye	Chlamydi	Rate	2008	0	0	0
Deaths	Global	Female	50-69 ye	Chlamydi	Rate	2008	0.04675	0.05253	0.03617
Deaths	Global	Male	50-69 ye	Gonococc	Number	2008	39.1713	55.8566	19.3997
Deaths	Global	Female	50-69 ye	Gonococc	Number	2008	784.112	880.32	611.072
Deaths	Global	Male	50-69 ye	Gonococc	Rate	2008	0.00786	0.0112	0.00389
Deaths	Global	Female	50-69 ye	Gonococc	Rate	2008	0.15355	0.17239	0.11967
Deaths	Global	Male	50-69 ye	Other se	Number	2008	91.5198	132.753	40.7091
Deaths	Global	Female	50-69 ye	Other se	Number	2008	510.732	576.029	393.204
Deaths	Global	Male	50-69 ye	Other se	Rate	2008	0.01835	0.02662	0.00816
Deaths	Global	Female	50-69 ye	Other se	Rate	2008	0.10002	0.11281	0.077
Deaths	Global	Male	50-69 ye	Sexually	Number	2012	593.793	828.709	302.348
Deaths	Global	Female	50-69 ye	Sexually	Number	2012	1685.95	1883.49	1329.28
Deaths	Global	Male	50-69 ye	Sexually	Rate	2012	0.10692	0.14922	0.05444
Deaths	Global	Female	50-69 ye	Sexually	Rate	2012	0.29485	0.3294	0.23248
Deaths	Global	Male	50-69 ye	Syphilis	Number	2012	468.167	649.469	242.983
Deaths	Global	Female	50-69 ye	Syphilis	Number	2012	101.522	115.318	77.5182
Deaths	Global	Male	50-69 ye	Syphilis	Rate	2012	0.0843	0.11694	0.04375
Deaths	Global	Female	50-69 ye	Syphilis	Rate	2012	0.01776	0.02017	0.01356
Deaths	Global	Male	50-69 ye	Chlamydi	Number	2012	0	0	0
Deaths	Global	Female	50-69 ye	Chlamydi	Number	2012	246.552	275.733	194.997
Deaths	Global	Male	50-69 ye	Chlamydi	Rate	2012	0	0	0
Deaths	Global	Female	50-69 ye	Chlamydi	Rate	2012	0.04312	0.04822	0.0341
Deaths	Global	Male	50-69 ye	HIV/AIDS	Number	2010	101580	116827	90372.1
Deaths	Global	Female	50-69 ye	HIV/AIDS	Number	2010	100679	114815	89385.1
Deaths	Global	Male	50-69 ye	HIV/AIDS	Rate	2010	19.3459	22.2497	17.2114
Deaths	Global	Female	50-69 ye	HIV/AIDS	Rate	2010	18.6827	21.306	16.587
Deaths	Global	Male	50-69 ye	Gonococc	Number	2012	37.9456	51.9806	19.1765
Deaths	Global	Female	50-69 ye	Gonococc	Number	2012	810.175	903.489	642.258
Deaths	Global	Male	50-69 ye	Gonococc	Rate	2012	0.00683	0.00936	0.00345
Deaths	Global	Female	50-69 ye	Gonococc	Rate	2012	0.14169	0.15801	0.11232
Deaths	Global	Male	50-69 ye	Other se	Number	2012	87.6808	125.71	40.1364
Deaths	Global	Female	50-69 ye	Other se	Number	2012	527.701	592.762	413.095
Deaths	Global	Male	50-69 ye	Other se	Rate	2012	0.01579	0.02264	0.00723
Deaths	Global	Female	50-69 ye	Other se	Rate	2012	0.09229	0.10367	0.07225
Deaths	Global	Male	50-69 ye	HIV/AIDS	Number	2000	92141.6	107428	76437
Deaths	Global	Female	50-69 ye	HIV/AIDS	Number	2000	104009	121378	84767.1
Deaths	Global	Male	50-69 ye	HIV/AIDS	Rate	2000	23.0275	26.8479	19.1027
Deaths	Global	Female	50-69 ye	HIV/AIDS	Rate	2000	25.4871	29.7433	20.7718
Deaths	Global	Male	50-69 ye	HIV/AIDS	Number	2014	101014	115542	92097.1
Deaths	Global	Female	50-69 ye	HIV/AIDS	Number	2014	85426	96377.2	77600.9
Deaths	Global	Male	50-69 ye	HIV/AIDS	Rate	2014	17.0887	19.5463	15.5802
Deaths	Global	Female	50-69 ye	HIV/AIDS	Rate	2014	13.9873	15.7804	12.7061
Deaths	Global	Male	50-69 ye	HIV/AIDS	Number	2018	101282	118363	89837.4
Deaths	Global	Female	50-69 ye	HIV/AIDS	Number	2018	82562.8	94025	74156.4
Deaths	Global	Male	50-69 ye	HIV/AIDS	Rate	2018	15.3322	17.918	13.5997
Deaths	Global	Female	50-69 ye	HIV/AIDS	Rate	2018	12.0315	13.7018	10.8064
Deaths	Global	Male	50-69 ye	HIV/AIDS	Number	2004	119384	130457	108765
Deaths	Global	Female	50-69 ye	HIV/AIDS	Number	2004	132314	145044	119258
Deaths	Global	Male	50-69 ye	HIV/AIDS	Rate	2004	26.7513	29.2326	24.3717
Deaths	Global	Female	50-69 ye	HIV/AIDS	Rate	2004	28.9825	31.771	26.1227
Deaths	Global	Male	50-69 ye	Sexually	Number	2001	845.895	1265.22	465.55
Deaths	Global	Female	50-69 ye	Sexually	Number	2001	1644.73	1877.24	1262.47
Deaths	Global	Male	50-69 ye	Sexually	Rate	2001	0.20601	0.30814	0.11338
Deaths	Global	Female	50-69 ye	Sexually	Rate	2001	0.39242	0.44789	0.30121
Deaths	Global	Male	50-69 ye	Syphilis	Number	2001	696.891	1023.54	396.535
Deaths	Global	Female	50-69 ye	Syphilis	Number	2001	97.4169	112.565	73.0351
Deaths	Global	Male	50-69 ye	Syphilis	Rate	2001	0.16972	0.24928	0.09657
Deaths	Global	Female	50-69 ye	Syphilis	Rate	2001	0.02324	0.02686	0.01743
Deaths	Global	Male	50-69 ye	Chlamydi	Number	2001	0	0	0
Deaths	Global	Female	50-69 ye	Chlamydi	Number	2001	240.113	273.486	184.856
Deaths	Global	Male	50-69 ye	Chlamydi	Rate	2001	0	0	0
Deaths	Global	Female	50-69 ye	Chlamydi	Rate	2001	0.05729	0.06525	0.0441
Deaths	Global	Male	50-69 yea	Gonococca	Number	2001	45.3115	70.8717	21.7791
Deaths	Global	Female	50-69 yea	Gonococca	Number	2001	795.926	903.278	616.445
Deaths	Global	Male	50-69 yea	Gonococca	Rate	2001	0.01104	0.01726	0.0053
Deaths	Global	Female	50-69 yea	Gonococca	Rate	2001	0.1899	0.21551	0.14708
Deaths	Global	Male	50-69 yea	Other sexu	Number	2001	103.693	173.704	44.0415
Deaths	Global	Female	50-69 yea	Other sexu	Number	2001	511.276	585.813	389.829
Deaths	Global	Male	50-69 yea	Other sexu	Rate	2001	0.02525	0.0423	0.01073
Deaths	Global	Female	50-69 yea	Other sexu	Rate	2001	0.12199	0.13977	0.09301
Deaths	Global	Male	50-69 yea	Sexually tr	Number	2016	590.171	813.168	306.234
Deaths	Global	Female	50-69 yea	Sexually tr	Number	2016	1974.53	2300.6	1498.45
Deaths	Global	Male	50-69 yea	Sexually tr	Rate	2016	0.09399	0.12951	0.04877
Deaths	Global	Female	50-69 yea	Sexually tr	Rate	2016	0.30339	0.35349	0.23024
Deaths	Global	Male	50-69 yea	Syphilis	Number	2016	465.814	636.119	246.861
Deaths	Global	Female	50-69 yea	Syphilis	Number	2016	122.38	144.32	90.3897
Deaths	Global	Male	50-69 yea	Syphilis	Rate	2016	0.07419	0.10131	0.03932
Deaths	Global	Female	50-69 yea	Syphilis	Rate	2016	0.0188	0.02217	0.01389
Deaths	Global	Male	50-69 yea	Chlamydia	Number	2016	0	0	0
Deaths	Global	Female	50-69 yea	Chlamydia	Number	2016	289.121	338.085	218.975
Deaths	Global	Male	50-69 yea	Chlamydia	Rate	2016	0	0	0
Deaths	Global	Female	50-69 yea	Chlamydia	Rate	2016	0.04442	0.05195	0.03365
Deaths	Global	Male	50-69 yea	Gonococca	Number	2016	37.7099	51.8129	19.3887
Deaths	Global	Female	50-69 yea	Gonococca	Number	2016	943.145	1093.64	719.358
Deaths	Global	Male	50-69 yea	Gonococca	Rate	2016	0.00601	0.00825	0.00309
Deaths	Global	Female	50-69 yea	Gonococca	Rate	2016	0.14492	0.16804	0.11053
Deaths	Global	Male	50-69 yea	Other sexu	Number	2016	86.6476	124.229	39.8068
Deaths	Global	Female	50-69 yea	Other sexu	Number	2016	619.887	725.299	466.451
Deaths	Global	Male	50-69 yea	Other sexu	Rate	2016	0.0138	0.01979	0.00634
Deaths	Global	Female	50-69 yea	Other sexu	Rate	2016	0.09525	0.11144	0.07167
Deaths	Global	Male	50-69 yea	HIV/AIDS	Number	2001	100629	115076	85554.7
Deaths	Global	Female	50-69 yea	HIV/AIDS	Number	2001	113544	129871	94939.1
Deaths	Global	Male	50-69 yea	HIV/AIDS	Rate	2001	24.5076	28.026	20.8363
Deaths	Global	Female	50-69 yea	HIV/AIDS	Rate	2001	27.0905	30.9858	22.6515
Deaths	Global	Male	50-69 yea	Sexually tr	Number	2002	800.402	1199.54	432.511
Deaths	Global	Female	50-69 yea	Sexually tr	Number	2002	1648.87	1879.93	1265.98
Deaths	Global	Male	50-69 yea	Sexually tr	Rate	2002	0.18981	0.28446	0.10256
Deaths	Global	Female	50-69 yea	Sexually tr	Rate	2002	0.38271	0.43634	0.29384
Deaths	Global	Male	50-69 yea	Syphilis	Number	2002	653.413	960.957	365.519
Deaths	Global	Female	50-69 yea	Syphilis	Number	2002	98.1079	113.337	73.1527
Deaths	Global	Male	50-69 yea	Syphilis	Rate	2002	0.15495	0.22788	0.08668
Deaths	Global	Female	50-69 yea	Syphilis	Rate	2002	0.02277	0.02631	0.01698
Deaths	Global	Male	50-69 yea	Chlamydia	Number	2002	0	0	0
Deaths	Global	Female	50-69 yea	Chlamydia	Number	2002	240.295	273.688	185.283
Deaths	Global	Male	50-69 yea	Chlamydia	Rate	2002	0	0	0
Deaths	Global	Female	50-69 yea	Chlamydia	Rate	2002	0.05577	0.06352	0.043
Deaths	Global	Male	50-69 yea	Gonococca	Number	2002	44.5472	68.3615	21.5836
Deaths	Global	Female	50-69 yea	Gonococca	Number	2002	797.995	908.971	618.485
Deaths	Global	Male	50-69 yea	Gonococca	Rate	2002	0.01056	0.01621	0.00512
Deaths	Global	Female	50-69 yea	Gonococca	Rate	2002	0.18522	0.21097	0.14355
Deaths	Global	Male	50-69 yea	Other sexu	Number	2002	102.442	168.953	43.6702
Deaths	Global	Female	50-69 yea	Other sexu	Number	2002	512.471	585.693	391.35
Deaths	Global	Male	50-69 yea	Other sexu	Rate	2002	0.02429	0.04007	0.01036
Deaths	Global	Female	50-69 yea	Other sexu	Rate	2002	0.11895	0.13594	0.09083
Deaths	Global	Male	50-69 yea	HIV/AIDS	Number	2005	121415	131277	112566
Deaths	Global	Female	50-69 yea	HIV/AIDS	Number	2005	133147	144150	123029
Deaths	Global	Male	50-69 yea	HIV/AIDS	Rate	2005	26.4192	28.5653	24.4938
Deaths	Global	Female	50-69 yea	HIV/AIDS	Rate	2005	28.308	30.6472	26.1568
Deaths	Global	Male	50-69 yea	Sexually tr	Number	2003	752.461	1149.96	401.892
Deaths	Global	Female	50-69 yea	Sexually tr	Number	2003	1633.31	1866.02	1291.44
Deaths	Global	Male	50-69 yea	Sexually tr	Rate	2003	0.17355	0.26522	0.09269
Deaths	Global	Female	50-69 yea	Sexually tr	Rate	2003	0.36847	0.42097	0.29134
Deaths	Global	Male	50-69 yea	Syphilis	Number	2003	607.602	916.217	333.798
Deaths	Global	Female	50-69 yea	Syphilis	Number	2003	97.0752	111.867	74.3189
Deaths	Global	Male	50-69 yea	Syphilis	Rate	2003	0.14014	0.21131	0.07699
Deaths	Global	Female	50-69 yea	Syphilis	Rate	2003	0.0219	0.02524	0.01677
Deaths	Global	Male	50-69 yea	Chlamydia	Number	2003	0	0	0
Deaths	Global	Female	50-69 yea	Chlamydia	Number	2003	238.228	272.752	189.106
Deaths	Global	Male	50-69 yea	Chlamydia	Rate	2003	0	0	0
Deaths	Global	Female	50-69 yea	Chlamydia	Rate	2003	0.05374	0.06153	0.04266
Deaths	Global	Male	50-69 yea	Gonococca	Number	2003	43.7644	67.58	21.5555
Deaths	Global	Female	50-69 yea	Gonococca	Number	2003	789.635	901.085	625.618
Deaths	Global	Male	50-69 yea	Gonococca	Rate	2003	0.01009	0.01559	0.00497
Deaths	Global	Female	50-69 yea	Gonococca	Rate	2003	0.17814	0.20328	0.14114
Deaths	Global	Male	50-69 yea	Other sexu	Number	2003	101.094	167.32	43.5308
Deaths	Global	Female	50-69 yea	Other sexu	Number	2003	508.367	584.666	399.961
Deaths	Global	Male	50-69 yea	Other sexu	Rate	2003	0.02332	0.03859	0.01004
Deaths	Global	Female	50-69 yea	Other sexu	Rate	2003	0.11469	0.1319	0.09023
Deaths	Global	Male	50-69 yea	HIV/AIDS	Number	2003	114630	126705	102395
Deaths	Global	Female	50-69 yea	HIV/AIDS	Number	2003	127850	142343	112058
Deaths	Global	Male	50-69 yea	HIV/AIDS	Rate	2003	26.438	29.2228	23.6162
Deaths	Global	Female	50-69 yea	HIV/AIDS	Rate	2003	28.8423	32.1119	25.2799
Deaths	Global	Male	50-69 yea	Sexually tr	Number	2005	662.806	974.452	336.284
Deaths	Global	Female	50-69 yea	Sexually tr	Number	2005	1602.11	1829.91	1272.18
Deaths	Global	Male	50-69 yea	Sexually tr	Rate	2005	0.14422	0.21204	0.07317
Deaths	Global	Female	50-69 yea	Sexually tr	Rate	2005	0.34062	0.38905	0.27047
Deaths	Global	Male	50-69 yea	Syphilis	Number	2005	525.168	767.439	274.258
Deaths	Global	Female	50-69 yea	Syphilis	Number	2005	94.9791	110.301	73.0156
Deaths	Global	Male	50-69 yea	Syphilis	Rate	2005	0.11427	0.16699	0.05968
Deaths	Global	Female	50-69 yea	Syphilis	Rate	2005	0.02019	0.02345	0.01552
Deaths	Global	Male	50-69 yea	Chlamydia	Number	2005	0	0	0
Deaths	Global	Female	50-69 yea	Chlamydia	Number	2005	233.755	266.036	186.886
Deaths	Global	Male	50-69 yea	Chlamydia	Rate	2005	0	0	0
Deaths	Global	Female	50-69 yea	Chlamydia	Rate	2005	0.0497	0.05656	0.03973
Deaths	Global	Male	50-69 yea	Gonococca	Number	2005	41.4013	61.005	20.5781
Deaths	Global	Female	50-69 yea	Gonococca	Number	2005	773.755	882.904	616.783
Deaths	Global	Male	50-69 yea	Gonococca	Rate	2005	0.00901	0.01327	0.00448
Deaths	Global	Female	50-69 yea	Gonococca	Rate	2005	0.16451	0.18771	0.13113
Deaths	Global	Male	50-69 yea	Other sexu	Number	2005	96.2362	148.387	41.7541
Deaths	Global	Female	50-69 yea	Other sexu	Number	2005	499.625	571.055	396.909
Deaths	Global	Male	50-69 yea	Other sexu	Rate	2005	0.02094	0.03229	0.00909
Deaths	Global	Female	50-69 yea	Other sexu	Rate	2005	0.10622	0.12141	0.08439
Deaths	Global	Male	50-69 yea	Sexually tr	Number	2006	639.944	925.159	325.864
Deaths	Global	Female	50-69 yea	Sexually tr	Number	2006	1618.63	1841.59	1281.64
Deaths	Global	Male	50-69 yea	Sexually tr	Rate	2006	0.13535	0.19567	0.06892
Deaths	Global	Female	50-69 yea	Sexually tr	Rate	2006	0.33445	0.38052	0.26482
Deaths	Global	Male	50-69 yea	Syphilis	Number	2006	505.469	723.9	263.715
Deaths	Global	Female	50-69 yea	Syphilis	Number	2006	96.2645	111.462	73.7017
Deaths	Global	Male	50-69 yea	Syphilis	Rate	2006	0.10691	0.15311	0.05578
Deaths	Global	Female	50-69 yea	Syphilis	Rate	2006	0.01989	0.02303	0.01523
Deaths	Global	Male	50-69 yea	Chlamydia	Number	2006	0	0	0
Deaths	Global	Female	50-69 yea	Chlamydia	Number	2006	236.054	268.137	186.815
Deaths	Global	Male	50-69 yea	Chlamydia	Rate	2006	0	0	0
Deaths	Global	Female	50-69 yea	Chlamydia	Rate	2006	0.04878	0.0554	0.0386
Deaths	Global	Male	50-69 yea	Gonococca	Number	2006	40.3707	58.2575	20.4698
Deaths	Global	Female	50-69 yea	Gonococca	Number	2006	780.901	886.008	623.777
Deaths	Global	Male	50-69 yea	Gonococca	Rate	2006	0.00854	0.01232	0.00433
Deaths	Global	Female	50-69 yea	Gonococca	Rate	2006	0.16136	0.18307	0.12889
Deaths	Global	Male	50-69 yea	Other sexu	Number	2006	94.104	139.723	41.6122
Deaths	Global	Female	50-69 yea	Other sexu	Number	2006	505.41	575.528	397.972
Deaths	Global	Male	50-69 yea	Other sexu	Rate	2006	0.0199	0.02955	0.0088
Deaths	Global	Female	50-69 yea	Other sexu	Rate	2006	0.10443	0.11892	0.08223
Deaths	Global	Male	50-69 ye	HIV/AIDS	Number	2007	114549	126435	103718
Deaths	Global	Female	50-69 ye	HIV/AIDS	Number	2007	122158	133566	111421
Deaths	Global	Male	50-69 ye	HIV/AIDS	Rate	2007	23.5763	26.0227	21.347
Deaths	Global	Female	50-69 ye	HIV/AIDS	Rate	2007	24.5607	26.8542	22.4018
Deaths	Global	Male	50-69 ye	Sexually	Number	2007	627.099	892.246	314.151
Deaths	Global	Female	50-69 ye	Sexually	Number	2007	1632.37	1844.13	1273.13
Deaths	Global	Male	50-69 ye	Sexually	Rate	2007	0.12907	0.18364	0.06466
Deaths	Global	Female	50-69 ye	Sexually	Rate	2007	0.3282	0.37077	0.25597
Deaths	Global	Male	50-69 ye	Syphilis	Number	2007	494.726	701.5	253.764
Deaths	Global	Female	50-69 ye	Syphilis	Number	2007	96.8781	111.828	73.1963
Deaths	Global	Male	50-69 ye	Syphilis	Rate	2007	0.10182	0.14438	0.05223
Deaths	Global	Female	50-69 ye	Syphilis	Rate	2007	0.01948	0.02248	0.01472
Deaths	Global	Male	50-69 ye	Chlamydi	Number	2007	0	0	0
Deaths	Global	Female	50-69 ye	Chlamydi	Number	2007	238.59	270.207	185.597
Deaths	Global	Male	50-69 ye	Chlamydi	Rate	2007	0	0	0
Deaths	Global	Female	50-69 ye	Chlamydi	Rate	2007	0.04797	0.05433	0.03732
Deaths	Global	Male	50-69 ye	Gonococc	Number	2007	39.6812	56.1402	19.6987
Deaths	Global	Female	50-69 ye	Gonococc	Number	2007	786.045	885.225	619.015
Deaths	Global	Male	50-69 ye	Gonococc	Rate	2007	0.00817	0.01155	0.00405
Deaths	Global	Female	50-69 ye	Gonococc	Rate	2007	0.15804	0.17798	0.12446
Deaths	Global	Male	50-69 ye	Other se	Number	2007	92.6916	135.652	40.7515
Deaths	Global	Female	50-69 ye	Other se	Number	2007	510.862	579.482	394.436
Deaths	Global	Male	50-69 ye	Other se	Rate	2007	0.01908	0.02792	0.00839
Deaths	Global	Female	50-69 ye	Other se	Rate	2007	0.10271	0.11651	0.0793
Deaths	Global	Male	50-69 ye	HIV/AIDS	Number	2008	109377	122434	97877.7
Deaths	Global	Female	50-69 ye	HIV/AIDS	Number	2008	114111	126639	102735
Deaths	Global	Male	50-69 ye	HIV/AIDS	Rate	2008	21.9352	24.5537	19.629
Deaths	Global	Female	50-69 ye	HIV/AIDS	Rate	2008	22.3466	24.8001	20.1189
Deaths	Global	Male	50-69 ye	HIV/AIDS	Number	2011	100345	115651	90159.6
Deaths	Global	Female	50-69 ye	HIV/AIDS	Number	2011	95413.2	109372	85127.1
Deaths	Global	Male	50-69 ye	HIV/AIDS	Rate	2011	18.5986	21.4356	16.7108
Deaths	Global	Female	50-69 ye	HIV/AIDS	Rate	2011	17.2046	19.7217	15.3498
Deaths	Global	Male	50-69 ye	Sexually	Number	2009	613.165	851.441	308.087
Deaths	Global	Female	50-69 ye	Sexually	Number	2009	1603.9	1799.76	1246.78
Deaths	Global	Male	50-69 ye	Sexually	Rate	2009	0.11987	0.16645	0.06023
Deaths	Global	Female	50-69 ye	Sexually	Rate	2009	0.30587	0.34322	0.23776
Deaths	Global	Male	50-69 ye	Syphilis	Number	2009	483.445	669.894	248.472
Deaths	Global	Female	50-69 ye	Syphilis	Number	2009	95.6265	109.151	73.1901
Deaths	Global	Male	50-69 ye	Syphilis	Rate	2009	0.09451	0.13096	0.04857
Deaths	Global	Female	50-69 ye	Syphilis	Rate	2009	0.01824	0.02082	0.01396
Deaths	Global	Male	50-69 ye	Chlamydi	Number	2009	0	0	0
Deaths	Global	Female	50-69 ye	Chlamydi	Number	2009	234.669	262.045	183.012
Deaths	Global	Male	50-69 ye	Chlamydi	Rate	2009	0	0	0
Deaths	Global	Female	50-69 ye	Chlamydi	Rate	2009	0.04475	0.04997	0.0349
Deaths	Global	Male	50-69 ye	Gonococc	Number	2009	38.9586	54.3341	19.6193
Deaths	Global	Female	50-69 ye	Gonococc	Number	2009	771.942	866.195	606.894
Deaths	Global	Male	50-69 ye	Gonococc	Rate	2009	0.00762	0.01062	0.00384
Deaths	Global	Female	50-69 ye	Gonococc	Rate	2009	0.14721	0.16518	0.11574
Deaths	Global	Male	50-69 ye	Other se	Number	2009	90.7607	132.747	40.0949
Deaths	Global	Female	50-69 ye	Other se	Number	2009	501.666	562.19	387.791
Deaths	Global	Male	50-69 ye	Other se	Rate	2009	0.01774	0.02595	0.00784
Deaths	Global	Female	50-69 ye	Other se	Rate	2009	0.09567	0.10721	0.07395
Deaths	Global	Male	50-69 ye	Sexually	Number	2011	597.678	821.623	302.102
Deaths	Global	Female	50-69 ye	Sexually	Number	2011	1659.61	1855.69	1313.99
Deaths	Global	Male	50-69 ye	Sexually	Rate	2011	0.11078	0.15229	0.05599
Deaths	Global	Female	50-69 ye	Sexually	Rate	2011	0.29925	0.33461	0.23693
Deaths	Global	Male	50-69 ye	Syphilis	Number	2011	471.114	649.861	244.448
Deaths	Global	Female	50-69 ye	Syphilis	Number	2011	99.922	113.63	76.908
Deaths	Global	Male	50-69 ye	Syphilis	Rate	2011	0.08732	0.12045	0.04531
Deaths	Global	Female	50-69 ye	Syphilis	Rate	2011	0.01802	0.02049	0.01387
Deaths	Global	Male	50-69 ye	Chlamydi	Number	2011	0	0	0
Deaths	Global	Female	50-69 ye	Chlamydi	Number	2011	242.771	270.958	193.763
Deaths	Global	Male	50-69 ye	Chlamydi	Rate	2011	0	0	0
Deaths	Global	Female	50-69 ye	Chlamydi	Rate	2011	0.04378	0.04886	0.03494
Deaths	Global	Male	50-69 ye	Gonococc	Number	2011	38.1387	52.6679	19.3852
Deaths	Global	Female	50-69 ye	Gonococc	Number	2011	797.84	890.866	631.497

Deaths	Global	Female	50-69 ye	Syphilis	Number	2013	105.214	119.721	78.7686
Deaths	Global	Male	50-69 ye	Syphilis	Rate	2013	0.081	0.11109	0.04224
Deaths	Global	Female	50-69 ye	Syphilis	Rate	2013	0.01781	0.02027	0.01333
Deaths	Global	Male	50-69 ye	Chlamydi	Number	2013	0	0	0
Deaths	Global	Female	50-69 ye	Chlamydi	Number	2013	253.473	284.788	200.217
Deaths	Global	Male	50-69 ye	Chlamydi	Rate	2013	0	0	0
Deaths	Global	Female	50-69 ye	Chlamydi	Rate	2013	0.04291	0.04821	0.03389
Deaths	Global	Male	50-69 ye	Gonococc	Number	2013	37.6495	51.876	19.1556
Deaths	Global	Female	50-69 ye	Gonococc	Number	2013	829.954	933.926	651.61
Deaths	Global	Male	50-69 ye	Gonococc	Rate	2013	0.00657	0.00906	0.00334
Deaths	Global	Female	50-69 ye	Gonococc	Rate	2013	0.14049	0.15809	0.1103
Deaths	Global	Male	50-69 ye	Other se	Number	2013	86.9097	123.183	39.7676
Deaths	Global	Female	50-69 ye	Other se	Number	2013	542.392	611.576	423.654
Deaths	Global	Male	50-69 ye	Other se	Rate	2013	0.01517	0.02151	0.00694
Deaths	Global	Female	50-69 ye	Other se	Rate	2013	0.09181	0.10352	0.07171
Deaths	Global	Male	50-69 ye	HIV/AIDS	Number	2013	99864.6	114806	90790.5
Deaths	Global	Female	50-69 ye	HIV/AIDS	Number	2013	86581	98854.6	78089.7
Deaths	Global	Male	50-69 ye	HIV/AIDS	Rate	2013	17.4369	20.0458	15.8525
Deaths	Global	Female	50-69 ye	HIV/AIDS	Rate	2013	14.6558	16.7334	13.2185
Deaths	Global	Male	50-69 ye	Sexually	Number	2010	604.014	849.195	301.179
Deaths	Global	Female	50-69 ye	Sexually	Number	2010	1630.96	1821.26	1294.92
Deaths	Global	Male	50-69 ye	Sexually	Rate	2010	0.11503	0.16173	0.05736
Deaths	Global	Female	50-69 ye	Sexually	Rate	2010	0.30265	0.33797	0.24029
Deaths	Global	Male	50-69 ye	Syphilis	Number	2010	475.984	665.151	243.079
Deaths	Global	Female	50-69 ye	Syphilis	Number	2010	97.8162	111.142	74.3852
Deaths	Global	Male	50-69 ye	Syphilis	Rate	2010	0.09065	0.12668	0.04629
Deaths	Global	Female	50-69 ye	Syphilis	Rate	2010	0.01815	0.02062	0.0138
Deaths	Global	Male	50-69 ye	Chlamydi	Number	2010	0	0	0
Deaths	Global	Female	50-69 ye	Chlamydi	Number	2010	238.553	266.156	191.295
Deaths	Global	Male	50-69 ye	Chlamydi	Rate	2010	0	0	0
Deaths	Global	Female	50-69 ye	Chlamydi	Rate	2010	0.04427	0.04939	0.0355
Deaths	Global	Male	50-69 ye	Gonococc	Number	2010	38.4566	53.0628	19.5875
Deaths	Global	Female	50-69 ye	Gonococc	Number	2010	784.919	876.974	625.363
Deaths	Global	Male	50-69 ye	Gonococc	Rate	2010	0.00732	0.01011	0.00373
Deaths	Global	Female	50-69 ye	Gonococc	Rate	2010	0.14566	0.16274	0.11605
Deaths	Global	Male	50-69 yea	Other sexu	Number	2010	89.5732	130.756	39.8268
Deaths	Global	Female	50-69 yea	Other sexu	Number	2010	509.672	570.322	404.454
Deaths	Global	Male	50-69 yea	Other sexu	Rate	2010	0.01706	0.0249	0.00759
Deaths	Global	Female	50-69 yea	Other sexu	Rate	2010	0.09458	0.10583	0.07505
Deaths	Global	Male	50-69 yea	Sexually tr	Number	2014	586.162	817.833	303.311
Deaths	Global	Female	50-69 yea	Sexually tr	Number	2014	1806.08	2049.6	1412.18
Deaths	Global	Male	50-69 yea	Sexually tr	Rate	2014	0.09916	0.13835	0.05131
Deaths	Global	Female	50-69 yea	Sexually tr	Rate	2014	0.29572	0.33559	0.23123
Deaths	Global	Male	50-69 yea	Syphilis	Number	2014	462.012	641.463	243.357
Deaths	Global	Female	50-69 yea	Syphilis	Number	2014	110.838	128.46	84.1469
Deaths	Global	Male	50-69 yea	Syphilis	Rate	2014	0.07816	0.10852	0.04117
Deaths	Global	Female	50-69 yea	Syphilis	Rate	2014	0.01815	0.02103	0.01378
Deaths	Global	Male	50-69 yea	Chlamydia	Number	2014	0	0	0
Deaths	Global	Female	50-69 yea	Chlamydia	Number	2014	265.171	301.045	207.844
Deaths	Global	Male	50-69 yea	Chlamydia	Rate	2014	0	0	0
Deaths	Global	Female	50-69 yea	Chlamydia	Rate	2014	0.04342	0.04929	0.03403
Deaths	Global	Male	50-69 yea	Gonococca	Number	2014	37.5584	51.9991	19.2887
Deaths	Global	Female	50-69 yea	Gonococca	Number	2014	862.683	976.264	678.521
Deaths	Global	Male	50-69 yea	Gonococca	Rate	2014	0.00635	0.0088	0.00326
Deaths	Global	Female	50-69 yea	Gonococca	Rate	2014	0.14125	0.15985	0.1111
Deaths	Global	Male	50-69 yea	Other sexu	Number	2014	86.5916	124.762	40.2877
Deaths	Global	Female	50-69 yea	Other sexu	Number	2014	567.383	645.719	441.995
Deaths	Global	Male	50-69 yea	Other sexu	Rate	2014	0.01465	0.02111	0.00682
Deaths	Global	Female	50-69 yea	Other sexu	Rate	2014	0.0929	0.10573	0.07237
Deaths	Global	Male	50-69 yea	HIV/AIDS	Number	2015	102460	117002	93392
Deaths	Global	Female	50-69 yea	HIV/AIDS	Number	2015	85358.9	95912.4	77929.8
Deaths	Global	Male	50-69 yea	HIV/AIDS	Rate	2015	16.7968	19.1807	15.3102
Deaths	Global	Female	50-69 yea	HIV/AIDS	Rate	2015	13.5222	15.1941	12.3453
Deaths	Global	Male	50-69 yea	Sexually tr	Number	2018	575.468	795.961	299.092
Deaths	Global	Female	50-69 yea	Sexually tr	Number	2018	1976.87	2336.89	1529.08
Deaths	Global	Male	50-69 yea	Sexually tr	Rate	2018	0.08711	0.12049	0.04528
Deaths	Global	Female	50-69 yea	Sexually tr	Rate	2018	0.28808	0.34054	0.22283
Deaths	Global	Male	50-69 yea	Syphilis	Number	2018	453.431	623.05	239.516
Deaths	Global	Female	50-69 yea	Syphilis	Number	2018	122.739	147.335	91.4973
Deaths	Global	Male	50-69 yea	Syphilis	Rate	2018	0.06864	0.09432	0.03626
Deaths	Global	Female	50-69 yea	Syphilis	Rate	2018	0.01789	0.02147	0.01333
Deaths	Global	Male	50-69 yea	Chlamydia	Number	2018	0	0	0
Deaths	Global	Female	50-69 yea	Chlamydia	Number	2018	288.631	341.81	221.026
Deaths	Global	Male	50-69 yea	Chlamydia	Rate	2018	0	0	0
Deaths	Global	Female	50-69 yea	Chlamydia	Rate	2018	0.04206	0.04981	0.03221
Deaths	Global	Male	50-69 yea	Gonococca	Number	2018	37.0075	51.1646	19.0364
Deaths	Global	Female	50-69 yea	Gonococca	Number	2018	946.07	1112.4	737.279
Deaths	Global	Male	50-69 yea	Gonococca	Rate	2018	0.0056	0.00775	0.00288
Deaths	Global	Female	50-69 yea	Gonococca	Rate	2018	0.13787	0.1621	0.10744
Deaths	Global	Male	50-69 yea	Other sexu	Number	2018	85.0301	121.831	39.3153
Deaths	Global	Female	50-69 yea	Other sexu	Number	2018	619.432	735.917	470.418
Deaths	Global	Male	50-69 yea	Other sexu	Rate	2018	0.01287	0.01844	0.00595
Deaths	Global	Female	50-69 yea	Other sexu	Rate	2018	0.09027	0.10724	0.06855
Deaths	Global	Male	50-69 yea	Sexually tr	Number	2015	590.448	816.168	301.976
Deaths	Global	Female	50-69 yea	Sexually tr	Number	2015	1937.08	2238.74	1443.99
Deaths	Global	Male	50-69 yea	Sexually tr	Rate	2015	0.09679	0.1338	0.0495
Deaths	Global	Female	50-69 yea	Sexually tr	Rate	2015	0.30686	0.35465	0.22875
Deaths	Global	Male	50-69 yea	Syphilis	Number	2015	465.823	639.458	243.424
Deaths	Global	Female	50-69 yea	Syphilis	Number	2015	119.613	139.812	87.1723
Deaths	Global	Male	50-69 yea	Syphilis	Rate	2015	0.07636	0.10483	0.03991
Deaths	Global	Female	50-69 yea	Syphilis	Rate	2015	0.01895	0.02215	0.01381
Deaths	Global	Male	50-69 yea	Chlamydia	Number	2015	0	0	0
Deaths	Global	Female	50-69 yea	Chlamydia	Number	2015	284.53	329.085	212.804
Deaths	Global	Male	50-69 yea	Chlamydia	Rate	2015	0	0	0
Deaths	Global	Female	50-69 yea	Chlamydia	Rate	2015	0.04507	0.05213	0.03371
Deaths	Global	Male	50-69 yea	Gonococca	Number	2015	37.7457	52.3851	19.0906
Deaths	Global	Female	50-69 yea	Gonococca	Number	2015	923.505	1062.21	692.014
Deaths	Global	Male	50-69 yea	Gonococca	Rate	2015	0.00619	0.00859	0.00313
Deaths	Global	Female	50-69 yea	Gonococca	Rate	2015	0.1463	0.16827	0.10963
Deaths	Global	Male	50-69 yea	Other sexu	Number	2015	86.8796	124.504	39.9775
Deaths	Global	Female	50-69 yea	Other sexu	Number	2015	609.43	705.862	451.438
Deaths	Global	Male	50-69 yea	Other sexu	Rate	2015	0.01424	0.02041	0.00655
Deaths	Global	Female	50-69 yea	Other sexu	Rate	2015	0.09654	0.11182	0.07152
Deaths	Global	Male	50-69 yea	Sexually tr	Number	2017	581.471	802.06	301.886
Deaths	Global	Female	50-69 yea	Sexually tr	Number	2017	1981.72	2320.06	1503.43
Deaths	Global	Male	50-69 yea	Sexually tr	Rate	2017	0.09027	0.12451	0.04686
Deaths	Global	Female	50-69 yea	Sexually tr	Rate	2017	0.29636	0.34696	0.22483
Deaths	Global	Male	50-69 yea	Syphilis	Number	2017	458.237	628.217	242.23
Deaths	Global	Female	50-69 yea	Syphilis	Number	2017	123.226	146.256	90.3432
Deaths	Global	Male	50-69 yea	Syphilis	Rate	2017	0.07114	0.09752	0.0376
Deaths	Global	Female	50-69 yea	Syphilis	Rate	2017	0.01843	0.02187	0.01351
Deaths	Global	Male	50-69 yea	Chlamydia	Number	2017	0	0	0
Deaths	Global	Female	50-69 yea	Chlamydia	Number	2017	289.673	339.058	219.697
Deaths	Global	Male	50-69 yea	Chlamydia	Rate	2017	0	0	0
Deaths	Global	Female	50-69 yea	Chlamydia	Rate	2017	0.04332	0.0507	0.03285
Deaths	Global	Male	50-69 yea	Gonococca	Number	2017	37.3955	51.54	19.2901
Deaths	Global	Female	50-69 yea	Gonococca	Number	2017	947.325	1104.88	719.981
Deaths	Global	Male	50-69 yea	Gonococca	Rate	2017	0.00581	0.008	0.00299
Deaths	Global	Female	50-69 yea	Gonococca	Rate	2017	0.14167	0.16523	0.10767
Deaths	Global	Male	50-69 yea	Other sexu	Number	2017	85.8382	122.888	39.7547
Deaths	Global	Female	50-69 yea	Other sexu	Number	2017	621.498	729.319	467.255
Deaths	Global	Male	50-69 yea	Other sexu	Rate	2017	0.01333	0.01908	0.00617
Deaths	Global	Female	50-69 yea	Other sexu	Rate	2017	0.09294	0.10907	0.06988
Deaths	Global	Male	50-69 yea	HIV/AIDS	Number	2019	100054	118066	88356.9
Deaths	Global	Female	50-69 yea	HIV/AIDS	Number	2019	82348.8	94169.9	73726.3
Deaths	Global	Male	50-69 yea	HIV/AIDS	Rate	2019	14.7983	17.4623	13.0682
Deaths	Global	Female	50-69 yea	HIV/AIDS	Rate	2019	11.7168	13.3987	10.4899
Deaths	Global	Male	50-69 yea	HIV/AIDS	Number	2016	103895	118529	94023
Deaths	Global	Female	50-69 yea	HIV/AIDS	Number	2016	85504.9	95965.7	78143.8
Deaths	Global	Male	50-69 yea	HIV/AIDS	Rate	2016	16.5471	18.8778	14.9747
Deaths	Global	Female	50-69 yea	HIV/AIDS	Rate	2016	13.1379	14.7452	12.0069
Deaths	Global	Male	50-69 yea	Sexually tr	Number	2019	573.438	788.793	296.642
Deaths	Global	Female	50-69 yea	Sexually tr	Number	2019	1985.4	2381.41	1483.38
Deaths	Global	Male	50-69 yea	Sexually tr	Rate	2019	0.08481	0.11666	0.04387
Deaths	Global	Female	50-69 yea	Sexually tr	Rate	2019	0.28249	0.33883	0.21106
Deaths	Global	Male	50-69 yea	Syphilis	Number	2019	451.737	618.875	234.544
Deaths	Global	Female	50-69 yea	Syphilis	Number	2019	123.286	149.173	88.5457
Deaths	Global	Male	50-69 yea	Syphilis	Rate	2019	0.06681	0.09153	0.03469
Deaths	Global	Female	50-69 yea	Syphilis	Rate	2019	0.01754	0.02122	0.0126
Deaths	Global	Male	50-69 yea	Chlamydia	Number	2019	0	0	0
Deaths	Global	Female	50-69 yea	Chlamydia	Number	2019	289.489	349.768	215.693
Deaths	Global	Male	50-69 yea	Chlamydia	Rate	2019	0	0	0
Deaths	Global	Female	50-69 yea	Chlamydia	Rate	2019	0.04119	0.04977	0.03069
Deaths	Global	Male	50-69 yea	Gonococca	Number	2019	36.8977	50.2537	18.9021
Deaths	Global	Female	50-69 yea	Gonococca	Number	2019	951.083	1135.22	720.684
Deaths	Global	Male	50-69 yea	Gonococca	Rate	2019	0.00546	0.00743	0.0028
Deaths	Global	Female	50-69 yea	Gonococca	Rate	2019	0.13532	0.16152	0.10254
Deaths	Global	Male	50-69 yea	Other sexu	Number	2019	84.804	121.256	38.9292
Deaths	Global	Female	50-69 yea	Other sexu	Number	2019	621.545	753.403	460.548
Deaths	Global	Male	50-69 yea	Other sexu	Rate	2019	0.01254	0.01793	0.00576
Deaths	Global	Female	50-69 yea	Other sexu	Rate	2019	0.08843	0.1072	0.06553
Deaths	Global	Male	50-69 yea	HIV/AIDS	Number	2017	103513	119030	92995.2
Deaths	Global	Female	50-69 yea	HIV/AIDS	Number	2017	84212	95199.5	76429.8
Deaths	Global	Male	50-69 yea	HIV/AIDS	Rate	2017	16.069	18.4778	14.4363
Deaths	Global	Female	50-69 yea	HIV/AIDS	Rate	2017	12.5936	14.2367	11.4298
DALYs (D	Global	Male	50-69 ye	HIV/AIDS	Number	1990	712207	921850	538676
DALYs (D	Global	Female	50-69 ye	HIV/AIDS	Number	1990	635337	862043	440444
DALYs (D	Global	Male	50-69 ye	HIV/AIDS	Rate	1990	211.629	273.923	160.065
DALYs (D	Global	Female	50-69 ye	HIV/AIDS	Rate	1990	183.835	249.432	127.442
DALYs (D	Global	Male	50-69 ye	Sexually	Number	1990	53836.3	79298.8	33513.8
DALYs (D	Global	Female	50-69 ye	Sexually	Number	1990	106589	166585	74241.2
DALYs (D	Global	Male	50-69 ye	Sexually	Rate	1990	15.9972	23.5633	9.95845
DALYs (D	Global	Female	50-69 ye	Sexually	Rate	1990	30.8416	48.2013	21.4817
DALYs (D	Global	Male	50-69 ye	Syphilis	Number	1990	28755.9	40002.4	16427.3
DALYs (D	Global	Female	50-69 ye	Syphilis	Number	1990	4243.28	5023.2	3155.06
DALYs (D	Global	Male	50-69 ye	Syphilis	Rate	1990	8.54467	11.8865	4.88128
DALYs (D	Global	Female	50-69 ye	Syphilis	Rate	1990	1.22779	1.45346	0.91292
DALYs (D	Global	Male	50-69 ye	Chlamydi	Number	1990	1488.81	2566.25	769.88
DALYs (D	Global	Female	50-69 ye	Chlamydi	Number	1990	9035.11	10684.3	6924.69
DALYs (D	Global	Male	50-69 ye	Chlamydi	Rate	1990	0.44239	0.76255	0.22877
DALYs (D	Global	Female	50-69 ye	Chlamydi	Rate	1990	2.61431	3.09149	2.00366
DALYs (D	Global	Male	50-69 ye	Gonococc	Number	1990	2766.19	3950.09	1556.17
DALYs (D	Global	Female	50-69 ye	Gonococc	Number	1990	24921.5	29247.9	18416.7
DALYs (D	Global	Male	50-69 ye	Gonococc	Rate	1990	0.82196	1.17375	0.46241
DALYs (D	Global	Female	50-69 ye	Gonococc	Rate	1990	7.21105	8.46287	5.32886
DALYs (D	Global	Male	50-69 ye	Trichomo	Number	1990	1007.56	2211.04	364.749
DALYs (D	Global	Female	50-69 ye	Trichomo	Number	1990	25276.9	56607.3	9311.23
DALYs (D	Global	Male	50-69 ye	Trichomo	Rate	1990	0.29939	0.657	0.10838
DALYs (D	Global	Female	50-69 ye	Trichomo	Rate	1990	7.31388	16.3793	2.69421
DALYs (D	Global	Male	50-69 ye	Genital	Number	1990	14009	35116.1	4300.6
DALYs (D	Global	Female	50-69 ye	Genital	Number	1990	22146.7	53863.7	6827.41
DALYs (D	Global	Male	50-69 ye	Genital	Rate	1990	4.16269	10.4346	1.2779
DALYs (D	Global	Female	50-69 ye	Genital	Rate	1990	6.40815	15.5855	1.97551
DALYs (D	Global	Male	50-69 ye	Other se	Number	1990	5808.83	8436.09	3062.58
DALYs (D	Global	Female	50-69 ye	Other se	Number	1990	20965.6	25072.8	16161.6
DALYs (D	Global	Male	50-69 ye	Other se	Rate	1990	1.72607	2.50674	0.91003
DALYs (D	Global	Female	50-69 ye	Other se	Rate	1990	6.06639	7.25481	4.67637
DALYs (D	Global	Male	50-69 ye	HIV/AIDS	Number	1992	1125288	1436975	860669
DALYs (D	Global	Female	50-69 ye	HIV/AIDS	Number	1992	1073908	1414976	770385
DALYs (D	Global	Male	50-69 ye	HIV/AIDS	Rate	1992	322.938	412.387	246.997
DALYs (D	Global	Female	50-69 ye	HIV/AIDS	Rate	1992	301.251	396.927	216.107
DALYs (D	Global	Male	50-69 ye	Sexually	Number	1992	56031.7	82398.2	35266.2
DALYs (D	Global	Female	50-69 ye	Sexually	Number	1992	109685	171895	76276.1
DALYs (D	Global	Male	50-69 ye	Sexually	Rate	1992	16.0801	23.6469	10.1208
DALYs (D	Global	Female	50-69 ye	Sexually	Rate	1992	30.7687	48.2196	21.3968
DALYs (D	Global	Male	50-69 ye	Syphilis	Number	1992	30237.3	41803.8	17554.6
DALYs (D	Global	Female	50-69 ye	Syphilis	Number	1992	4287.11	5060.12	3210.1
DALYs (D	Global	Male	50-69 ye	Syphilis	Rate	1992	8.67758	11.997	5.03787
DALYs (D	Global	Female	50-69 ye	Syphilis	Rate	1992	1.20261	1.41946	0.90049
DALYs (D	Global	Male	50-69 ye	Chlamydi	Number	1992	1512.86	2574.16	788.262
DALYs (D	Global	Female	50-69 ye	Chlamydi	Number	1992	9133.52	10761	7053.87
DALYs (D	Global	Male	50-69 ye	Chlamydi	Rate	1992	0.43416	0.73874	0.22622
DALYs (D	Global	Female	50-69 ye	Chlamydi	Rate	1992	2.56212	3.01865	1.97874
DALYs (D	Global	Male	50-69 ye	Gonococc	Number	1992	2762.98	3920.06	1584.33
DALYs (D	Global	Female	50-69 ye	Gonococc	Number	1992	25351.5	29588.1	19139.1
DALYs (D	Global	Male	50-69 ye	Gonococc	Rate	1992	0.79293	1.12499	0.45468
DALYs (D	Global	Female	50-69 ye	Gonococc	Rate	1992	7.11157	8.30001	5.36888
DALYs (D	Global	Male	50-69 ye	Trichomo	Number	1992	1035.81	2283.86	366.981
DALYs (D	Global	Female	50-69 ye	Trichomo	Number	1992	26507.8	59499.5	9746.48
DALYs (D	Global	Male	50-69 ye	Trichomo	Rate	1992	0.29726	0.65543	0.10532
DALYs (D	Global	Female	50-69 ye	Trichomo	Rate	1992	7.43594	16.6907	2.73407
DALYs (D	Global	Male	50-69 ye	Genital	Number	1992	14759.4	37033	4544.36
DALYs (D	Global	Female	50-69 ye	Genital	Number	1992	23250.8	56415.3	7149.48
DALYs (D	Global	Male	50-69 ye	Genital	Rate	1992	4.23571	10.6278	1.30415
DALYs (D	Global	Female	50-69 ye	Genital	Rate	1992	6.52228	15.8255	2.00556
DALYs (D	Global	Male	50-69 ye	Other se	Number	1992	5723.26	8295.81	3106.01
DALYs (D	Global	Female	50-69 ye	Other se	Number	1992	21154.1	25308.4	16353.4
DALYs (D	Global	Male	50-69 ye	Other se	Rate	1992	1.64248	2.38075	0.89137
DALYs (D	Global	Female	50-69 ye	Other se	Rate	1992	5.93413	7.09949	4.58745
DALYs (D	Global	Male	50-69 ye	HIV/AIDS	Number	1991	902397	1161699	689902
DALYs (D	Global	Female	50-69 ye	HIV/AIDS	Number	1991	836175	1114352	593023
DALYs (D	Global	Male	50-69 ye	HIV/AIDS	Rate	1991	263.224	338.862	201.241
DALYs (D	Global	Female	50-69 ye	HIV/AIDS	Rate	1991	238.034	317.223	168.816
DALYs (D	Global	Male	50-69 ye	Sexually	Number	1994	57904.4	84812.8	37020.9
DALYs (D	Global	Female	50-69 ye	Sexually	Number	1994	111241	176545	77011
DALYs (D	Global	Male	50-69 ye	Sexually	Rate	1994	16.1526	23.6588	10.3271
DALYs (D	Global	Female	50-69 ye	Sexually	Rate	1994	30.4286	48.2917	21.0655
DALYs (D	Global	Male	50-69 ye	Syphilis	Number	1994	31611.4	43485	18425.6
DALYs (D	Global	Female	50-69 ye	Syphilis	Number	1994	4276.13	5033.89	3238.15
DALYs (D	Global	Male	50-69 ye	Syphilis	Rate	1994	8.81812	12.1303	5.13989
DALYs (D	Global	Female	50-69 ye	Syphilis	Rate	1994	1.16968	1.37696	0.88576
DALYs (D	Global	Male	50-69 ye	Chlamydi	Number	1994	1538.41	2645.87	809.406
DALYs (D	Global	Female	50-69 ye	Chlamydi	Number	1994	9078.68	10546.8	7079.94
DALYs (D	Global	Male	50-69 ye	Chlamydi	Rate	1994	0.42914	0.73807	0.22579
DALYs (D	Global	Female	50-69 ye	Chlamydi	Rate	1994	2.48337	2.88494	1.93663
DALYs (D	Global	Male	50-69 ye	Gonococc	Number	1994	2746.63	3901.8	1604.71
DALYs (D	Global	Female	50-69 ye	Gonococc	Number	1994	25313.1	29023.3	19299.7
DALYs (D	Global	Male	50-69 ye	Gonococc	Rate	1994	0.76618	1.08842	0.44764
DALYs (D	Global	Female	50-69 ye	Gonococc	Rate	1994	6.9241	7.93897	5.27921
DALYs (D	Global	Male	50-69 ye	Trichomo	Number	1994	1060.81	2316.9	379.298
DALYs (D	Global	Female	50-69 ye	Trichomo	Number	1994	27469.5	61821.5	10054.3
DALYs (D	Global	Male	50-69 ye	Trichomo	Rate	1994	0.29592	0.64631	0.10581
DALYs (D	Global	Female	50-69 ye	Trichomo	Rate	1994	7.51394	16.9105	2.75025
DALYs (D	Global	Male	50-69 ye	Genital	Number	1994	15313.3	38002.3	4733.73
DALYs (D	Global	Female	50-69 ye	Genital	Number	1994	24102	58498.1	7415.55
DALYs (D	Global	Male	50-69 ye	Genital	Rate	1994	4.2717	10.6009	1.32049
DALYs (D	Global	Female	50-69 ye	Genital	Rate	1994	6.59281	16.0015	2.02844
DALYs (D	Global	Male	50-69 ye	Other se	Number	1994	5633.79	8207.86	3040.79
DALYs (D	Global	Female	50-69 ye	Other se	Number	1994	21001.6	24921.7	16454.9
DALYs (D	Global	Male	50-69 ye	Other se	Rate	1994	1.57157	2.28961	0.84824
DALYs (D	Global	Female	50-69 ye	Other se	Rate	1994	5.74473	6.81703	4.50104
DALYs (D	Global	Male	50-69 ye	Sexually	Number	1997	56186.6	84171.4	36669.8
DALYs (D	Global	Female	50-69 ye	Sexually	Number	1997	113302	180625	77402
DALYs (D	Global	Male	50-69 ye	Sexually	Rate	1997	14.9492	22.3949	9.75646
DALYs (D	Global	Female	50-69 ye	Sexually	Rate	1997	29.5945	47.1793	20.2174
DALYs (D	Global	Male	50-69 ye	Syphilis	Number	1997	29517	41175.5	17700.6
DALYs (D	Global	Female	50-69 ye	Syphilis	Number	1997	4378.45	5114.69	3394.19
DALYs (D	Global	Male	50-69 ye	Syphilis	Rate	1997	7.85336	10.9553	4.70948
DALYs (D	Global	Female	50-69 ye	Syphilis	Rate	1997	1.14365	1.33596	0.88656
DALYs (D	Global	Male	50-69 ye	Chlamydi	Number	1997	1652.91	2822.58	864.537
DALYs (D	Global	Female	50-69 ye	Chlamydi	Number	1997	9004.68	10447.5	7124.62
DALYs (D	Global	Male	50-69 ye	Chlamydi	Rate	1997	0.43978	0.75098	0.23002
DALYs (D	Global	Female	50-69 ye	Chlamydi	Rate	1997	2.35202	2.72888	1.86095
DALYs (D	Global	Male	50-69 ye	Gonococc	Number	1997	2634.61	3774.62	1572.4
DALYs (D	Global	Female	50-69 ye	Gonococc	Number	1997	25032.9	28787.7	18504.9
DALYs (D	Global	Male	50-69 ye	Gonococc	Rate	1997	0.70097	1.00429	0.41836
DALYs (D	Global	Female	50-69 ye	Gonococc	Rate	1997	6.53859	7.51933	4.83348
DALYs (D	Global	Male	50-69 ye	Trichomo	Number	1997	1119.24	2450.68	400.866
DALYs (D	Global	Female	50-69 ye	Trichomo	Number	1997	28760.4	64491.4	10502.5
DALYs (D	Global	Male	50-69 ye	Trichomo	Rate	1997	0.29779	0.65203	0.10666
DALYs (D	Global	Female	50-69 ye	Trichomo	Rate	1997	7.5122	16.8451	2.74325
DALYs (D	Global	Male	50-69 ye	Genital	Number	1997	15907.2	39519.9	4870.63
DALYs (D	Global	Female	50-69 ye	Genital	Number	1997	25193.1	61188	7779.99
DALYs (D	Global	Male	50-69 ye	Genital	Rate	1997	4.23233	10.5148	1.29589
DALYs (D	Global	Female	50-69 ye	Genital	Rate	1997	6.58044	15.9823	2.03213
DALYs (Dis	Global	Male	50-69 yea	Other sexu	Number	1997	5355.67	8006.34	3038.02
DALYs (Dis	Global	Female	50-69 yea	Other sexu	Number	1997	20932.6	24857.7	16775.2
DALYs (Dis	Global	Male	50-69 yea	Other sexu	Rate	1997	1.42494	2.13019	0.8083
DALYs (Dis	Global	Female	50-69 yea	Other sexu	Rate	1997	5.4676	6.49282	4.38168
DALYs (Dis	Global	Male	50-69 yea	Sexually tr	Number	1991	54988.9	80928.8	34628.7
DALYs (Dis	Global	Female	50-69 yea	Sexually tr	Number	1991	107968	170543	74852
DALYs (Dis	Global	Male	50-69 yea	Sexually tr	Rate	1991	16.04	23.6065	10.101
DALYs (Dis	Global	Female	50-69 yea	Sexually tr	Rate	1991	30.7352	48.5484	21.3081
DALYs (Dis	Global	Male	50-69 yea	Syphilis	Number	1991	29525.8	41384.9	16905.2
DALYs (Dis	Global	Female	50-69 yea	Syphilis	Number	1991	4248.27	5003.2	3223.67
DALYs (Dis	Global	Male	50-69 yea	Syphilis	Rate	1991	8.61252	12.0718	4.93117
DALYs (Dis	Global	Female	50-69 yea	Syphilis	Rate	1991	1.20936	1.42426	0.91768
DALYs (Dis	Global	Male	50-69 yea	Chlamydia	Number	1991	1500.68	2608.35	783.253
DALYs (Dis	Global	Female	50-69 yea	Chlamydia	Number	1991	9048.46	10565.5	6935.29
DALYs (Dis	Global	Male	50-69 yea	Chlamydia	Rate	1991	0.43774	0.76084	0.22847
DALYs (Dis	Global	Female	50-69 yea	Chlamydia	Rate	1991	2.57583	3.00769	1.97427
DALYs (Dis	Global	Male	50-69 yea	Gonococca	Number	1991	2762.78	3943.92	1572.35
DALYs (Dis	Global	Female	50-69 yea	Gonococca	Number	1991	25045.5	29246.5	18423.9
DALYs (Dis	Global	Male	50-69 yea	Gonococca	Rate	1991	0.80589	1.15042	0.45865
DALYs (Dis	Global	Female	50-69 yea	Gonococca	Rate	1991	7.12972	8.3256	5.24472
DALYs (Dis	Global	Male	50-69 yea	Trichomon	Number	1991	1023.5	2243.97	366.701
DALYs (Dis	Global	Female	50-69 yea	Trichomon	Number	1991	25922.6	58260	9506.57
DALYs (Dis	Global	Male	50-69 yea	Trichomon	Rate	1991	0.29855	0.65455	0.10696
DALYs (Dis	Global	Female	50-69 yea	Trichomon	Rate	1991	7.3794	16.5849	2.70623
DALYs (Dis	Global	Male	50-69 yea	Genital he	Number	1991	14416.9	36111	4428.21
DALYs (Dis	Global	Female	50-69 yea	Genital he	Number	1991	22726.7	55268.5	7008.25
DALYs (Dis	Global	Male	50-69 yea	Genital he	Rate	1991	4.20535	10.5334	1.29169
DALYs (Dis	Global	Female	50-69 yea	Genital he	Rate	1991	6.4696	15.7333	1.99504
DALYs (Dis	Global	Male	50-69 yea	Other sexu	Number	1991	5759.26	8340.63	3125.54
DALYs (Dis	Global	Female	50-69 yea	Other sexu	Number	1991	20976.1	25089	16215
DALYs (Dis	Global	Male	50-69 yea	Other sexu	Rate	1991	1.67995	2.43292	0.9117
DALYs (Dis	Global	Female	50-69 yea	Other sexu	Rate	1991	5.97127	7.14209	4.61592
DALYs (Dis	Global	Male	50-69 yea	HIV/AIDS	Number	1994	1664042	2077257	1288625
DALYs (Dis	Global	Female	50-69 yea	HIV/AIDS	Number	1994	1671175	2127459	1244355
DALYs (Dis	Global	Male	50-69 yea	HIV/AIDS	Rate	1994	464.19	579.458	359.467
DALYs (Dis	Global	Female	50-69 yea	HIV/AIDS	Rate	1994	457.13	581.941	340.379
DALYs (Dis	Global	Male	50-69 yea	Sexually tr	Number	1993	57002.2	82466.9	36354
DALYs (Dis	Global	Female	50-69 yea	Sexually tr	Number	1993	110321	175098	76930.9
DALYs (Dis	Global	Male	50-69 yea	Sexually tr	Rate	1993	16.1178	23.3181	10.2794
DALYs (Dis	Global	Female	50-69 yea	Sexually tr	Rate	1993	30.551	48.4897	21.3043
DALYs (Dis	Global	Male	50-69 yea	Syphilis	Number	1993	30949.8	42564.4	18001.9
DALYs (Dis	Global	Female	50-69 yea	Syphilis	Number	1993	4255.11	5007.28	3185.37
DALYs (Dis	Global	Male	50-69 yea	Syphilis	Rate	1993	8.75129	12.0354	5.09018
DALYs (Dis	Global	Female	50-69 yea	Syphilis	Rate	1993	1.17836	1.38666	0.88212
DALYs (Dis	Global	Male	50-69 yea	Chlamydia	Number	1993	1526.72	2603.86	801.661
DALYs (Dis	Global	Female	50-69 yea	Chlamydia	Number	1993	9079.36	10571.2	7024.4
DALYs (Dis	Global	Male	50-69 yea	Chlamydia	Rate	1993	0.43169	0.73626	0.22668
DALYs (Dis	Global	Female	50-69 yea	Chlamydia	Rate	1993	2.51433	2.92747	1.94526
DALYs (Dis	Global	Male	50-69 yea	Gonococca	Number	1993	2752.5	3919.11	1589.35
DALYs (Dis	Global	Female	50-69 yea	Gonococca	Number	1993	25273.1	29124.6	19024.7
DALYs (Dis	Global	Male	50-69 yea	Gonococca	Rate	1993	0.77829	1.10816	0.4494
DALYs (Dis	Global	Female	50-69 yea	Gonococca	Rate	1993	6.99885	8.06541	5.26847
DALYs (Dis	Global	Male	50-69 yea	Trichomon	Number	1993	1047.76	2310.74	372.208
DALYs (Dis	Global	Female	50-69 yea	Trichomon	Number	1993	27009.2	60541.8	9856.87
DALYs (Dis	Global	Male	50-69 yea	Trichomon	Rate	1993	0.29626	0.65338	0.10524
DALYs (Dis	Global	Female	50-69 yea	Trichomon	Rate	1993	7.47961	16.7657	2.72965
DALYs (Dis	Global	Male	50-69 yea	Genital he	Number	1993	15056.9	37359	4614.44
DALYs (Dis	Global	Female	50-69 yea	Genital he	Number	1993	23701.9	57495.6	7307.05
DALYs (Dis	Global	Male	50-69 yea	Genital he	Rate	1993	4.25746	10.5636	1.30477
DALYs (Dis	Global	Female	50-69 yea	Genital he	Rate	1993	6.56372	15.9222	2.02353
DALYs (Dis	Global	Male	50-69 yea	Other sexu	Number	1993	5668.52	8268.19	3065.75
DALYs (Dis	Global	Female	50-69 yea	Other sexu	Number	1993	21002.4	24914.4	16364.8
DALYs (Dis	Global	Male	50-69 yea	Other sexu	Rate	1993	1.60282	2.33789	0.86686
DALYs (Dis	Global	Female	50-69 yea	Other sexu	Rate	1993	5.81616	6.89949	4.53188
DALYs (Dis	Global	Male	50-69 yea	Sexually tr	Number	2001	53614.3	83627.6	34135.5
DALYs (Dis	Global	Female	50-69 yea	Sexually tr	Number	2001	116614	191580	77246.9
DALYs (Dis	Global	Male	50-69 yea	Sexually tr	Rate	2001	13.0574	20.3669	8.31347
DALYs (Dis	Global	Female	50-69 yea	Sexually tr	Rate	2001	27.823	45.7089	18.4303
DALYs (Dis	Global	Male	50-69 yea	Syphilis	Number	2001	25345	36162.4	15620.4
DALYs (Dis	Global	Female	50-69 yea	Syphilis	Number	2001	4327.53	5037.36	3454.81
DALYs (Dis	Global	Male	50-69 yea	Syphilis	Rate	2001	6.1726	8.80711	3.80425
DALYs (Dis	Global	Female	50-69 yea	Syphilis	Rate	2001	1.0325	1.20186	0.82428
DALYs (Dis	Global	Male	50-69 yea	Chlamydia	Number	2001	1945.43	3372.52	992.123
DALYs (Dis	Global	Female	50-69 yea	Chlamydia	Number	2001	8797.06	10168.1	7100.9
DALYs (Dis	Global	Male	50-69 yea	Chlamydia	Rate	2001	0.4738	0.82136	0.24162
DALYs (Dis	Global	Female	50-69 yea	Chlamydia	Rate	2001	2.09889	2.426	1.6942
DALYs (Dis	Global	Male	50-69 yea	Gonococca	Number	2001	2653.17	3808.22	1643.01
DALYs (Dis	Global	Female	50-69 yea	Gonococca	Number	2001	24147.7	27455.4	18704.3
DALYs (Dis	Global	Male	50-69 yea	Gonococca	Rate	2001	0.64616	0.92747	0.40014
DALYs (Dis	Global	Female	50-69 yea	Gonococca	Rate	2001	5.7614	6.55057	4.46266
DALYs (Dis	Global	Male	50-69 yea	Trichomon	Number	2001	1254.99	2793.2	443.117
DALYs (Dis	Global	Female	50-69 yea	Trichomon	Number	2001	31250.1	69471.1	11502.7
DALYs (Dis	Global	Male	50-69 yea	Trichomon	Rate	2001	0.30565	0.68027	0.10792
DALYs (Dis	Global	Female	50-69 yea	Trichomon	Rate	2001	7.45596	16.5751	2.74442
DALYs (Dis	Global	Male	50-69 yea	Genital he	Number	2001	17020.8	42343.6	5182.17
DALYs (Dis	Global	Female	50-69 yea	Genital he	Number	2001	27379.3	66779.8	8466.67
DALYs (Dis	Global	Male	50-69 yea	Genital he	Rate	2001	4.14531	10.3125	1.26208
DALYs (Dis	Global	Female	50-69 yea	Genital he	Rate	2001	6.53242	15.933	2.02006
DALYs (Dis	Global	Male	50-69 yea	Other sexu	Number	2001	5394.87	7862.97	3231.23
DALYs (Dis	Global	Female	50-69 yea	Other sexu	Number	2001	20712.5	24687	16684.5
DALYs (Dis	Global	Male	50-69 yea	Other sexu	Rate	2001	1.31389	1.91497	0.78695
DALYs (Dis	Global	Female	50-69 yea	Other sexu	Rate	2001	4.94179	5.89007	3.98076
DALYs (Dis	Global	Male	50-69 yea	HIV/AIDS	Number	2002	3797729	4178943	3411761
DALYs (Dis	Global	Female	50-69 yea	HIV/AIDS	Number	2002	4217865	4689686	3744621
DALYs (Dis	Global	Male	50-69 yea	HIV/AIDS	Rate	2002	900.584	990.984	809.057
DALYs (Dis	Global	Female	50-69 yea	HIV/AIDS	Rate	2002	978.977	1088.49	869.136
DALYs (Dis	Global	Male	50-69 yea	HIV/AIDS	Number	1998	2640482	3125526	2152257
DALYs (Dis	Global	Female	50-69 yea	HIV/AIDS	Number	1998	2995756	3565344	2417832
DALYs (Dis	Global	Male	50-69 yea	HIV/AIDS	Rate	1998	688.674	815.18	561.338
DALYs (Dis	Global	Female	50-69 yea	HIV/AIDS	Rate	1998	766.662	912.428	618.762
DALYs (Dis	Global	Male	50-69 yea	HIV/AIDS	Number	1995	1948671	2388653	1524766
DALYs (Dis	Global	Female	50-69 yea	HIV/AIDS	Number	1995	1999118	2510601	1510887
DALYs (Dis	Global	Male	50-69 yea	HIV/AIDS	Rate	1995	536.838	658.048	420.057
DALYs (Dis	Global	Female	50-69 yea	HIV/AIDS	Rate	1995	540.371	678.628	408.4
DALYs (Dis	Global	Male	50-69 yea	HIV/AIDS	Number	1993	1378859	1744775	1056215
DALYs (Dis	Global	Female	50-69 yea	HIV/AIDS	Number	1993	1355386	1763451	985689
DALYs (Dis	Global	Male	50-69 yea	HIV/AIDS	Rate	1993	389.883	493.349	298.653
DALYs (Dis	Global	Female	50-69 yea	HIV/AIDS	Rate	1993	375.344	488.349	272.965
DALYs (Dis	Global	Male	50-69 yea	HIV/AIDS	Number	1997	2370672	2858115	1887373
DALYs (Dis	Global	Female	50-69 yea	HIV/AIDS	Number	1997	2662970	3223739	2104033
DALYs (Dis	Global	Male	50-69 yea	HIV/AIDS	Rate	1997	630.748	760.438	502.16
DALYs (Dis	Global	Female	50-69 yea	HIV/AIDS	Rate	1997	695.567	842.039	549.573
DALYs (Dis	Global	Male	50-69 yea	Sexually tr	Number	2009	52701.2	86024.2	32477.7
DALYs (Dis	Global	Female	50-69 yea	Sexually tr	Number	2009	131355	222976	83514.5
DALYs (Dis	Global	Male	50-69 yea	Sexually tr	Rate	2009	10.3027	16.8172	6.34919
DALYs (Dis	Global	Female	50-69 yea	Sexually tr	Rate	2009	25.0495	42.5219	15.9263
DALYs (Dis	Global	Male	50-69 yea	Syphilis	Number	2009	19647.8	26107.6	12138.5
DALYs (Dis	Global	Female	50-69 yea	Syphilis	Number	2009	4335.57	5011.68	3534.47
DALYs (Dis	Global	Male	50-69 yea	Syphilis	Rate	2009	3.84102	5.10388	2.37299
DALYs (Dis	Global	Female	50-69 yea	Syphilis	Rate	2009	0.8268	0.95574	0.67403
DALYs (Dis	Global	Male	50-69 yea	Chlamydia	Number	2009	2392.25	4166.52	1224.27
DALYs (Dis	Global	Female	50-69 yea	Chlamydia	Number	2009	8942.94	10456.5	7577.92
DALYs (Dis	Global	Male	50-69 yea	Chlamydia	Rate	2009	0.46767	0.81453	0.23934
DALYs (Dis	Global	Female	50-69 yea	Chlamydia	Rate	2009	1.70543	1.99407	1.44512
DALYs (Dis	Global	Male	50-69 yea	Gonococca	Number	2009	2844.02	4196.37	1830.4
DALYs (Dis	Global	Female	50-69 yea	Gonococca	Number	2009	23569.2	26400.2	18492.5
DALYs (Dis	Global	Male	50-69 yea	Gonococca	Rate	2009	0.55599	0.82036	0.35783
DALYs (Dis	Global	Female	50-69 yea	Gonococca	Rate	2009	4.49469	5.03456	3.52655
DALYs (Dis	Global	Male	50-69 yea	Trichomon	Number	2009	1579.74	3492.87	560.172
DALYs (Dis	Global	Female	50-69 yea	Trichomon	Number	2009	38936.9	86312.6	14382.7
DALYs (Dis	Global	Male	50-69 yea	Trichomon	Rate	2009	0.30883	0.68283	0.10951
DALYs (Dis	Global	Female	50-69 yea	Trichomon	Rate	2009	7.42534	16.46	2.74281
DALYs (Dis	Global	Male	50-69 yea	Genital he	Number	2009	20767.1	51650	6348.25
DALYs (Dis	Global	Female	50-69 yea	Genital he	Number	2009	33968	83741.1	10423.5
DALYs (Dis	Global	Male	50-69 yea	Genital he	Rate	2009	4.05983	10.0972	1.24104
DALYs (Dis	Global	Female	50-69 yea	Genital he	Rate	2009	6.47776	15.9696	1.98778
DALYs (Dis	Global	Male	50-69 yea	Other sexu	Number	2009	5470.34	7493.16	3361.05
DALYs (Dis	Global	Female	50-69 yea	Other sexu	Number	2009	21601.9	25939.2	17924.1
DALYs (Dis	Global	Male	50-69 yea	Other sexu	Rate	2009	1.06942	1.46486	0.65706
DALYs (Dis	Global	Female	50-69 yea	Other sexu	Rate	2009	4.11951	4.94665	3.41816
DALYs (Dis	Global	Male	50-69 yea	HIV/AIDS	Number	2006	4241394	4658987	3835319
DALYs (Dis	Global	Female	50-69 yea	HIV/AIDS	Number	2006	4534860	4934618	4143231
DALYs (Dis	Global	Male	50-69 yea	HIV/AIDS	Rate	2006	897.064	985.386	811.178
DALYs (Dis	Global	Female	50-69 yea	HIV/AIDS	Rate	2006	937.025	1019.63	856.103
DALYs (Dis	Global	Male	50-69 yea	Sexually tr	Number	1995	58105.9	86091.4	37177.6
DALYs (Dis	Global	Female	50-69 yea	Sexually tr	Number	1995	110657	176161	76085
DALYs (Dis	Global	Male	50-69 yea	Sexually tr	Rate	1995	16.0076	23.7172	10.242
DALYs (Dis	Global	Female	50-69 yea	Sexually tr	Rate	1995	29.9112	47.6172	20.5662
DALYs (Dis	Global	Male	50-69 yea	Syphilis	Number	1995	31677.8	43445.3	18712
DALYs (Dis	Global	Female	50-69 yea	Syphilis	Number	1995	4217.57	4924.98	3235.61
DALYs (Dis	Global	Male	50-69 yea	Syphilis	Rate	1995	8.7269	11.9687	5.15496
DALYs (Dis	Global	Female	50-69 yea	Syphilis	Rate	1995	1.14003	1.33125	0.8746
DALYs (Dis	Global	Male	50-69 yea	Chlamydia	Number	1995	1556.5	2653.27	794.448
DALYs (Dis	Global	Female	50-69 yea	Chlamydia	Number	1995	8867.36	10258.3	7021.39
DALYs (Dis	Global	Male	50-69 yea	Chlamydia	Rate	1995	0.4288	0.73095	0.21886
DALYs (Dis	Global	Female	50-69 yea	Chlamydia	Rate	1995	2.39689	2.77288	1.89792
DALYs (Dis	Global	Male	50-69 yea	Gonococca	Number	1995	2714.6	3835.25	1597.56
DALYs (Dis	Global	Female	50-69 yea	Gonococca	Number	1995	24681.4	28335.6	19083.3
DALYs (Dis	Global	Male	50-69 yea	Gonococca	Rate	1995	0.74784	1.05657	0.44011
DALYs (Dis	Global	Female	50-69 yea	Gonococca	Rate	1995	6.67151	7.65925	5.15832
DALYs (Dis	Global	Male	50-69 yea	Trichomon	Number	1995	1072.43	2366.05	380.625
DALYs (Dis	Global	Female	50-69 yea	Trichomon	Number	1995	27886.5	62464.6	10190.9
DALYs (Dis	Global	Male	50-69 yea	Trichomon	Rate	1995	0.29544	0.65182	0.10486
DALYs (Dis	Global	Female	50-69 yea	Trichomon	Rate	1995	7.53786	16.8845	2.75465
DALYs (Dis	Global	Male	50-69 yea	Genital he	Number	1995	15532.6	38600.2	4755.97
DALYs (Dis	Global	Female	50-69 yea	Genital he	Number	1995	24453.1	59478.5	7534.31
DALYs (Dis	Global	Male	50-69 yea	Genital he	Rate	1995	4.27905	10.6339	1.31022
DALYs (Dis	Global	Female	50-69 yea	Genital he	Rate	1995	6.60979	16.0773	2.03656
DALYs (Dis	Global	Male	50-69 yea	Other sexu	Number	1995	5552.03	8033.17	3085.5
DALYs (Dis	Global	Female	50-69 yea	Other sexu	Number	1995	20551.2	24306.9	16325.5
DALYs (Dis	Global	Male	50-69 yea	Other sexu	Rate	1995	1.52952	2.21305	0.85002
DALYs (Dis	Global	Female	50-69 yea	Other sexu	Rate	1995	5.5551	6.57027	4.41286
DALYs (Dis	Global	Male	50-69 yea	Sexually tr	Number	2005	51659.5	82881.5	32223.1
DALYs (Dis	Global	Female	50-69 yea	Sexually tr	Number	2005	123963	209475	79942.6
DALYs (Dis	Global	Male	50-69 yea	Sexually tr	Rate	2005	11.2409	18.0346	7.0116
DALYs (Dis	Global	Female	50-69 yea	Sexually tr	Rate	2005	26.3554	44.5359	16.9963
DALYs (Dis	Global	Male	50-69 yea	Syphilis	Number	2005	20544.6	28320.1	12518.4
DALYs (Dis	Global	Female	50-69 yea	Syphilis	Number	2005	4309.84	5071.95	3530.03
DALYs (Dis	Global	Male	50-69 yea	Syphilis	Rate	2005	4.47041	6.16232	2.72395
DALYs (Dis	Global	Female	50-69 yea	Syphilis	Rate	2005	0.9163	1.07833	0.75051
DALYs (Dis	Global	Male	50-69 yea	Chlamydia	Number	2005	2297.08	3986.93	1143.13
DALYs (Dis	Global	Female	50-69 yea	Chlamydia	Number	2005	8803.73	10217.5	7360.6
DALYs (Dis	Global	Male	50-69 yea	Chlamydia	Rate	2005	0.49983	0.86754	0.24874
DALYs (Dis	Global	Female	50-69 yea	Chlamydia	Rate	2005	1.87173	2.1723	1.56491
DALYs (Dis	Global	Male	50-69 yea	Gonococca	Number	2005	2751.84	4006.16	1735.56
DALYs (Dis	Global	Female	50-69 yea	Gonococca	Number	2005	23568.6	26913.8	19095
DALYs (Dis	Global	Male	50-69 yea	Gonococca	Rate	2005	0.59879	0.87172	0.37765
DALYs (Dis	Global	Female	50-69 yea	Gonococca	Rate	2005	5.01085	5.72205	4.05972
DALYs (Dis	Global	Male	50-69 yea	Trichomon	Number	2005	1425.17	3156.51	509.416
DALYs (Dis	Global	Female	50-69 yea	Trichomon	Number	2005	35225.4	77677.9	13007.2
DALYs (Dis	Global	Male	50-69 yea	Trichomon	Rate	2005	0.31011	0.68684	0.11085
DALYs (Dis	Global	Female	50-69 yea	Trichomon	Rate	2005	7.48915	16.5148	2.76541
DALYs (Dis	Global	Male	50-69 yea	Genital he	Number	2005	19129.1	47472.2	5787.01
DALYs (Dis	Global	Female	50-69 yea	Genital he	Number	2005	30963.2	75680.3	9537.48
DALYs (Dis	Global	Male	50-69 yea	Genital he	Rate	2005	4.16241	10.3297	1.25922
DALYs (Dis	Global	Female	50-69 yea	Genital he	Rate	2005	6.58298	16.0901	2.02773
DALYs (Dis	Global	Male	50-69 yea	Other sexu	Number	2005	5511.62	7740.76	3392.19
DALYs (Dis	Global	Female	50-69 yea	Other sexu	Number	2005	21092.5	25207.3	17423.4
DALYs (Dis	Global	Male	50-69 yea	Other sexu	Rate	2005	1.1993	1.68435	0.73812
DALYs (Dis	Global	Female	50-69 yea	Other sexu	Rate	2005	4.4844	5.35924	3.70434
DALYs (Dis	Global	Male	50-69 yea	Sexually tr	Number	1996	57062.1	84567	37022.8
DALYs (Dis	Global	Female	50-69 yea	Sexually tr	Number	1996	111619	179258	75762
DALYs (Dis	Global	Male	50-69 yea	Sexually tr	Rate	1996	15.4912	22.9582	10.0509
DALYs (Dis	Global	Female	50-69 yea	Sexually tr	Rate	1996	29.7343	47.7527	20.1823
DALYs (Dis	Global	Male	50-69 yea	Syphilis	Number	1996	30582.4	42069.3	17984.4
DALYs (Dis	Global	Female	50-69 yea	Syphilis	Number	1996	4289.37	5007.22	3301.47
DALYs (Dis	Global	Male	50-69 yea	Syphilis	Rate	1996	8.30248	11.4209	4.88238
DALYs (Dis	Global	Female	50-69 yea	Syphilis	Rate	1996	1.14265	1.33388	0.87948
DALYs (Dis	Global	Male	50-69 yea	Chlamydia	Number	1996	1593.5	2713.75	821.588
DALYs (Dis	Global	Female	50-69 yea	Chlamydia	Number	1996	8886.42	10352	7006.95
DALYs (Dis	Global	Male	50-69 yea	Chlamydia	Rate	1996	0.4326	0.73673	0.22304
DALYs (Dis	Global	Female	50-69 yea	Chlamydia	Rate	1996	2.36726	2.75767	1.86659
DALYs (Dis	Global	Male	50-69 yea	Gonococca	Number	1996	2661	3772.68	1576.37
DALYs (Dis	Global	Female	50-69 yea	Gonococca	Number	1996	24714	28341.7	18469.6
DALYs (Dis	Global	Male	50-69 yea	Gonococca	Rate	1996	0.7224	1.0242	0.42795
DALYs (Dis	Global	Female	50-69 yea	Gonococca	Rate	1996	6.58357	7.54996	4.92013
DALYs (Dis	Global	Male	50-69 yea	Trichomon	Number	1996	1091.08	2405.99	392.405
DALYs (Dis	Global	Female	50-69 yea	Trichomon	Number	1996	28306.7	63471.4	10334.8
DALYs (Dis	Global	Male	50-69 yea	Trichomon	Rate	1996	0.29621	0.65318	0.10653
DALYs (Dis	Global	Female	50-69 yea	Trichomon	Rate	1996	7.54065	16.9082	2.75309
DALYs (Dis	Global	Male	50-69 yea	Genital he	Number	1996	15719.5	39047	4811.1
DALYs (Dis	Global	Female	50-69 yea	Genital he	Number	1996	24794.9	59974.1	7632.79
DALYs (Dis	Global	Male	50-69 yea	Genital he	Rate	1996	4.26752	10.6004	1.30611
DALYs (Dis	Global	Female	50-69 yea	Genital he	Rate	1996	6.60512	15.9765	2.0333
DALYs (Dis	Global	Male	50-69 yea	Other sexu	Number	1996	5414.58	8009.31	3029.98
DALYs (Dis	Global	Female	50-69 yea	Other sexu	Number	1996	20627.8	24466.1	16341.8
DALYs (Dis	Global	Male	50-69 yea	Other sexu	Rate	1996	1.46995	2.17436	0.82258
DALYs (Dis	Global	Female	50-69 yea	Other sexu	Rate	1996	5.49506	6.51755	4.35331
DALYs (Dis	Global	Male	50-69 yea	Sexually tr	Number	1999	55175.6	83539.1	35935.7
DALYs (Dis	Global	Female	50-69 yea	Sexually tr	Number	1999	113760	185229	76821.2
DALYs (Dis	Global	Male	50-69 yea	Sexually tr	Rate	1999	14.0608	21.2889	9.15777
DALYs (Dis	Global	Female	50-69 yea	Sexually tr	Rate	1999	28.4613	46.3418	19.2197
DALYs (Dis	Global	Male	50-69 yea	Syphilis	Number	1999	27865	38509.2	16876.6
DALYs (Dis	Global	Female	50-69 yea	Syphilis	Number	1999	4296.56	5026.76	3369.66
DALYs (Dis	Global	Male	50-69 yea	Syphilis	Rate	1999	7.10107	9.8136	4.3008
DALYs (Dis	Global	Female	50-69 yea	Syphilis	Rate	1999	1.07494	1.25763	0.84304
DALYs (Dis	Global	Male	50-69 yea	Chlamydia	Number	1999	1804.77	3055.47	922.598
DALYs (Dis	Global	Female	50-69 yea	Chlamydia	Number	1999	8792.77	10183.3	7145.55
DALYs (Dis	Global	Male	50-69 yea	Chlamydia	Rate	1999	0.45992	0.77865	0.23511
DALYs (Dis	Global	Female	50-69 yea	Chlamydia	Rate	1999	2.19984	2.54774	1.78772
DALYs (Dis	Global	Male	50-69 yea	Gonococca	Number	1999	2634.75	3809.54	1618.05
DALYs (Dis	Global	Female	50-69 yea	Gonococca	Number	1999	24263.4	27808.9	18820.4
DALYs (Dis	Global	Male	50-69 yea	Gonococca	Rate	1999	0.67143	0.97081	0.41234
DALYs (Dis	Global	Female	50-69 yea	Gonococca	Rate	1999	6.07039	6.95741	4.70862
DALYs (Dis	Global	Male	50-69 yea	Trichomon	Number	1999	1184.5	2591.79	421.726
DALYs (Dis	Global	Female	50-69 yea	Trichomon	Number	1999	29759.5	66262.2	10937.5
DALYs (Dis	Global	Male	50-69 yea	Trichomon	Rate	1999	0.30186	0.66049	0.10747
DALYs (Dis	Global	Female	50-69 yea	Trichomon	Rate	1999	7.44543	16.5779	2.73641
DALYs (Dis	Global	Male	50-69 yea	Genital he	Number	1999	16299.4	40566.6	4993.64
DALYs (Dis	Global	Female	50-69 yea	Genital he	Number	1999	26082.5	63758.9	8063.37
DALYs (Dis	Global	Male	50-69 yea	Genital he	Rate	1999	4.15369	10.3379	1.27257
DALYs (Dis	Global	Female	50-69 yea	Genital he	Rate	1999	6.52549	15.9516	2.01735
DALYs (Dis	Global	Male	50-69 yea	Other sexu	Number	1999	5387.18	7937.41	3122.29
DALYs (Dis	Global	Female	50-69 yea	Other sexu	Number	1999	20565.3	24232.9	16769.1
DALYs (Dis	Global	Male	50-69 yea	Other sexu	Rate	1999	1.37286	2.02275	0.79568
DALYs (Dis	Global	Female	50-69 yea	Other sexu	Rate	1999	5.14517	6.06276	4.1954
DALYs (Dis	Global	Male	50-69 yea	HIV/AIDS	Number	1996	2170314	2640867	1708091
DALYs (Dis	Global	Female	50-69 yea	HIV/AIDS	Number	1996	2333958	2884625	1813538
DALYs (Dis	Global	Male	50-69 yea	HIV/AIDS	Rate	1996	589.195	716.94	463.711
DALYs (Dis	Global	Female	50-69 yea	HIV/AIDS	Rate	1996	621.745	768.437	483.11
DALYs (Dis	Global	Male	50-69 yea	HIV/AIDS	Number	1999	2939306	3413874	2467621
DALYs (Dis	Global	Female	50-69 yea	HIV/AIDS	Number	1999	3319031	3875754	2759303
DALYs (Dis	Global	Male	50-69 yea	HIV/AIDS	Rate	1999	749.046	869.984	628.843
DALYs (Dis	Global	Female	50-69 yea	HIV/AIDS	Rate	1999	830.378	969.662	690.341
DALYs (Dis	Global	Male	50-69 yea	Sexually tr	Number	2013	56208.9	94449.2	34486.9
DALYs (Dis	Global	Female	50-69 yea	Sexually tr	Number	2013	145727	248784	91568.1
DALYs (Dis	Global	Male	50-69 yea	Sexually tr	Rate	2013	9.8144	16.4914	6.02161
DALYs (Dis	Global	Female	50-69 yea	Sexually tr	Rate	2013	24.6677	42.1125	15.5
DALYs (Dis	Global	Male	50-69 yea	Syphilis	Number	2013	19512.6	25566.7	12206.4
DALYs (Dis	Global	Female	50-69 yea	Syphilis	Number	2013	4780.18	5534.84	3818.4
DALYs (Dis	Global	Male	50-69 yea	Syphilis	Rate	2013	3.407	4.46409	2.13131
DALYs (Dis	Global	Female	50-69 yea	Syphilis	Rate	2013	0.80916	0.9369	0.64635

DALYs (Dis	Global	Male	50-69 yea	Sexually tr	Number	1998	55294.5	83659.8	35981
DALYs (Dis	Global	Female	50-69 yea	Sexually tr	Number	1998	113567	182805	77228
DALYs (Dis	Global	Male	50-69 yea	Sexually tr	Rate	1998	14.4216	21.8196	9.38433
DALYs (Dis	Global	Female	50-69 yea	Sexually tr	Rate	1998	29.0637	46.7826	19.7639
DALYs (Dis	Global	Male	50-69 yea	Syphilis	Number	1998	28387.6	39257	17198.1
DALYs (Dis	Global	Female	50-69 yea	Syphilis	Number	1998	4355.32	5049.01	3429.39
DALYs (Dis	Global	Male	50-69 yea	Syphilis	Rate	1998	7.40388	10.2388	4.48549
DALYs (Dis	Global	Female	50-69 yea	Syphilis	Rate	1998	1.1146	1.29212	0.87764
DALYs (Dis	Global	Male	50-69 yea	Chlamydia	Number	1998	1718.1	2974.57	885.858
DALYs (Dis	Global	Female	50-69 yea	Chlamydia	Number	1998	8912.75	10305.8	7077.75
DALYs (Dis	Global	Male	50-69 yea	Chlamydia	Rate	1998	0.44811	0.77581	0.23104
DALYs (Dis	Global	Female	50-69 yea	Chlamydia	Rate	1998	2.28092	2.63743	1.81131
DALYs (Dis	Global	Male	50-69 yea	Gonococca	Number	1998	2622.52	3761.11	1587.26
DALYs (Dis	Global	Female	50-69 yea	Gonococca	Number	1998	24711	28301.5	18821.1
DALYs (Dis	Global	Male	50-69 yea	Gonococca	Rate	1998	0.68399	0.98095	0.41398
DALYs (Dis	Global	Female	50-69 yea	Gonococca	Rate	1998	6.32393	7.24279	4.81661
DALYs (Dis	Global	Male	50-69 yea	Trichomon	Number	1998	1147.97	2523.62	405.767
DALYs (Dis	Global	Female	50-69 yea	Trichomon	Number	1998	29206.1	65155.8	10725
DALYs (Dis	Global	Male	50-69 yea	Trichomon	Rate	1998	0.29941	0.6582	0.10583
DALYs (Dis	Global	Female	50-69 yea	Trichomon	Rate	1998	7.4743	16.6744	2.74469
DALYs (Dis	Global	Male	50-69 yea	Genital he	Number	1998	16069	40029.5	4900.99
DALYs (Dis	Global	Female	50-69 yea	Genital he	Number	1998	25592.4	62984.3	7854.58
DALYs (Dis	Global	Male	50-69 yea	Genital he	Rate	1998	4.19102	10.4402	1.27824
DALYs (Dis	Global	Female	50-69 yea	Genital he	Rate	1998	6.5495	16.1187	2.01011
DALYs (Dis	Global	Male	50-69 yea	Other sexu	Number	1998	5349.26	7910.14	3079.55
DALYs (Dis	Global	Female	50-69 yea	Other sexu	Number	1998	20789.8	24591.4	16712.5
DALYs (Dis	Global	Male	50-69 yea	Other sexu	Rate	1998	1.39516	2.06307	0.80319
DALYs (Dis	Global	Female	50-69 yea	Other sexu	Rate	1998	5.32044	6.29333	4.27699
DALYs (Dis	Global	Male	50-69 yea	Sexually tr	Number	2017	61077.6	104776	37231.6
DALYs (Dis	Global	Female	50-69 yea	Sexually tr	Number	2017	166461	287591	103152
DALYs (Dis	Global	Male	50-69 yea	Sexually tr	Rate	2017	9.48147	16.2651	5.7797
DALYs (Dis	Global	Female	50-69 yea	Sexually tr	Rate	2017	24.8936	43.0082	15.426
DALYs (Dis	Global	Male	50-69 yea	Syphilis	Number	2017	19893.4	25834.3	12681.1
DALYs (Dis	Global	Female	50-69 yea	Syphilis	Number	2017	5567.44	6620.33	4428.07
DALYs (Dis	Global	Male	50-69 yea	Syphilis	Rate	2017	3.08817	4.01042	1.96857
DALYs (Dis	Global	Female	50-69 yea	Syphilis	Rate	2017	0.83259	0.99005	0.6622
DALYs (Dis	Global	Male	50-69 yea	Chlamydia	Number	2017	3014.1	5179.44	1537.13
DALYs (Dis	Global	Female	50-69 yea	Chlamydia	Number	2017	11127.4	13309.1	9037.04
DALYs (Dis	Global	Male	50-69 yea	Chlamydia	Rate	2017	0.4679	0.80404	0.23862
DALYs (Dis	Global	Female	50-69 yea	Chlamydia	Rate	2017	1.66406	1.99033	1.35146
DALYs (Dis	Global	Male	50-69 yea	Gonococca	Number	2017	3192.04	4866.19	2075.77
DALYs (Dis	Global	Female	50-69 yea	Gonococca	Number	2017	29083.2	34026.9	22264.4
DALYs (Dis	Global	Male	50-69 yea	Gonococca	Rate	2017	0.49552	0.75541	0.32224
DALYs (Dis	Global	Female	50-69 yea	Gonococca	Rate	2017	4.34929	5.0886	3.32957
DALYs (Dis	Global	Male	50-69 yea	Trichomon	Number	2017	1985.23	4409.81	716.962
DALYs (Dis	Global	Female	50-69 yea	Trichomon	Number	2017	50057.2	112075	18487.3
DALYs (Dis	Global	Male	50-69 yea	Trichomon	Rate	2017	0.30818	0.68456	0.1113
DALYs (Dis	Global	Female	50-69 yea	Trichomon	Rate	2017	7.48588	16.7605	2.76472
DALYs (Dis	Global	Male	50-69 yea	Genital he	Number	2017	27169.9	67970.4	8305.66
DALYs (Dis	Global	Female	50-69 yea	Genital he	Number	2017	43705.4	107805	13447.5
DALYs (Dis	Global	Male	50-69 yea	Genital he	Rate	2017	4.21776	10.5515	1.28934
DALYs (Dis	Global	Female	50-69 yea	Genital he	Rate	2017	6.53599	16.1219	2.01103
DALYs (Dis	Global	Male	50-69 yea	Other sexu	Number	2017	5822.91	8102.56	3738.01
DALYs (Dis	Global	Female	50-69 yea	Other sexu	Number	2017	26920	32961	21854.8
DALYs (Dis	Global	Male	50-69 yea	Other sexu	Rate	2017	0.90393	1.25781	0.58028
DALYs (Dis	Global	Female	50-69 yea	Other sexu	Rate	2017	4.0258	4.9292	3.26831
DALYs (Dis	Global	Male	50-69 yea	Sexually tr	Number	2002	53052.4	83097.8	33669.1
DALYs (Dis	Global	Female	50-69 yea	Sexually tr	Number	2002	118606	194622	78491.3
DALYs (Dis	Global	Male	50-69 yea	Sexually tr	Rate	2002	12.5807	19.7056	7.98421
DALYs (Dis	Global	Female	50-69 yea	Sexually tr	Rate	2002	27.5288	45.1724	18.218
DALYs (Dis	Global	Male	50-69 yea	Syphilis	Number	2002	24136	34522.8	14921.9
DALYs (Dis	Global	Female	50-69 yea	Syphilis	Number	2002	4357.94	5076.45	3468.49
DALYs (Dis	Global	Male	50-69 yea	Syphilis	Rate	2002	5.72355	8.18666	3.53854
DALYs (Dis	Global	Female	50-69 yea	Syphilis	Rate	2002	1.01149	1.17826	0.80505
DALYs (Dis	Global	Male	50-69 yea	Chlamydia	Number	2002	2013.19	3439.29	1014.81
DALYs (Dis	Global	Female	50-69 yea	Chlamydia	Number	2002	8837.5	10320	7106.86
DALYs (Dis	Global	Male	50-69 yea	Chlamydia	Rate	2002	0.4774	0.81559	0.24065
DALYs (Dis	Global	Female	50-69 yea	Chlamydia	Rate	2002	2.05121	2.39529	1.64952
DALYs (Dis	Global	Male	50-69 yea	Gonococca	Number	2002	2678.84	3861.18	1667.57
DALYs (Dis	Global	Female	50-69 yea	Gonococca	Number	2002	24192.4	27505	18802.2
DALYs (Dis	Global	Male	50-69 yea	Gonococca	Rate	2002	0.63525	0.91563	0.39544
DALYs (Dis	Global	Female	50-69 yea	Gonococca	Rate	2002	5.61512	6.38398	4.36404
DALYs (Dis	Global	Male	50-69 yea	Trichomon	Number	2002	1292.94	2864.52	463.815
DALYs (Dis	Global	Female	50-69 yea	Trichomon	Number	2002	32142.4	71289.7	11813.6
DALYs (Dis	Global	Male	50-69 yea	Trichomon	Rate	2002	0.3066	0.67929	0.10999
DALYs (Dis	Global	Female	50-69 yea	Trichomon	Rate	2002	7.46033	16.5465	2.74196
DALYs (Dis	Global	Male	50-69 yea	Genital he	Number	2002	17500.6	43568.6	5318.42
DALYs (Dis	Global	Female	50-69 yea	Genital he	Number	2002	28194.1	68687	8732.21
DALYs (Dis	Global	Male	50-69 yea	Genital he	Rate	2002	4.15004	10.3317	1.2612
DALYs (Dis	Global	Female	50-69 yea	Genital he	Rate	2002	6.54393	15.9424	2.02677
DALYs (Dis	Global	Male	50-69 yea	Other sexu	Number	2002	5430.88	7736.55	3245.44
DALYs (Dis	Global	Female	50-69 yea	Other sexu	Number	2002	20881.8	24849.5	17009.2
DALYs (Dis	Global	Male	50-69 yea	Other sexu	Rate	2002	1.28787	1.83463	0.76962
DALYs (Dis	Global	Female	50-69 yea	Other sexu	Rate	2002	4.84671	5.76764	3.94788
DALYs (Dis	Global	Male	50-69 yea	HIV/AIDS	Number	2018	3711347	4354853	3283178
DALYs (Dis	Global	Female	50-69 yea	HIV/AIDS	Number	2018	3048344	3507230	2717237
DALYs (Dis	Global	Male	50-69 yea	HIV/AIDS	Rate	2018	561.827	659.242	497.011
DALYs (Dis	Global	Female	50-69 yea	HIV/AIDS	Rate	2018	444.22	511.092	395.97
DALYs (Dis	Global	Male	50-69 yea	HIV/AIDS	Number	2001	3527387	3957577	3096532
DALYs (Dis	Global	Female	50-69 yea	HIV/AIDS	Number	2001	3946323	4447909	3432262
DALYs (Dis	Global	Male	50-69 yea	HIV/AIDS	Rate	2001	859.071	963.841	754.14
DALYs (Dis	Global	Female	50-69 yea	HIV/AIDS	Rate	2001	941.553	1061.23	818.903
DALYs (Dis	Global	Male	50-69 yea	Sexually tr	Number	2000	54472.5	83268.4	35203.6
DALYs (Dis	Global	Female	50-69 yea	Sexually tr	Number	2000	114350	186085	77078.7
DALYs (Dis	Global	Male	50-69 yea	Sexually tr	Rate	2000	13.6135	20.81	8.79789
DALYs (Dis	Global	Female	50-69 yea	Sexually tr	Rate	2000	28.0209	45.5994	18.8878
DALYs (Dis	Global	Male	50-69 yea	Syphilis	Number	2000	26824.6	37859.7	16415.4
DALYs (Dis	Global	Female	50-69 yea	Syphilis	Number	2000	4258.52	4973.04	3398.11
DALYs (Dis	Global	Male	50-69 yea	Syphilis	Rate	2000	6.70386	9.46168	4.10244
DALYs (Dis	Global	Female	50-69 yea	Syphilis	Rate	2000	1.04353	1.21862	0.83269
DALYs (Dis	Global	Male	50-69 yea	Chlamydia	Number	2000	1858.78	3194.02	938.635
DALYs (Dis	Global	Female	50-69 yea	Chlamydia	Number	2000	8717.39	10112.6	7154.68
DALYs (Dis	Global	Male	50-69 yea	Chlamydia	Rate	2000	0.46454	0.79823	0.23458
DALYs (Dis	Global	Female	50-69 yea	Chlamydia	Rate	2000	2.13616	2.47806	1.75323
DALYs (Dis	Global	Male	50-69 yea	Gonococca	Number	2000	2634.14	3789.86	1626.4
DALYs (Dis	Global	Female	50-69 yea	Gonococca	Number	2000	23959.8	27226.4	18710.2
DALYs (Dis	Global	Male	50-69 yea	Gonococca	Rate	2000	0.65831	0.94714	0.40646
DALYs (Dis	Global	Female	50-69 yea	Gonococca	Rate	2000	5.87125	6.67172	4.58486
DALYs (Dis	Global	Male	50-69 yea	Trichomon	Number	2000	1214.66	2665.75	436.049
DALYs (Dis	Global	Female	50-69 yea	Trichomon	Number	2000	30349.9	67304.7	11160.2
DALYs (Dis	Global	Male	50-69 yea	Trichomon	Rate	2000	0.30356	0.66621	0.10897
DALYs (Dis	Global	Female	50-69 yea	Trichomon	Rate	2000	7.43713	16.4928	2.73477
DALYs (Dis	Global	Male	50-69 yea	Genital he	Number	2000	16568.5	41201.6	5054.83
DALYs (Dis	Global	Female	50-69 yea	Genital he	Number	2000	26607.9	64634.1	8185.46
DALYs (Dis	Global	Male	50-69 yea	Genital he	Rate	2000	4.1407	10.2969	1.26328
DALYs (Dis	Global	Female	50-69 yea	Genital he	Rate	2000	6.52016	15.8383	2.00581
DALYs (Dis	Global	Male	50-69 yea	Other sexu	Number	2000	5371.86	7882.23	3137.46
DALYs (Dis	Global	Female	50-69 yea	Other sexu	Number	2000	20456.1	24412.9	16729.7
DALYs (Dis	Global	Male	50-69 yea	Other sexu	Rate	2000	1.3425	1.96988	0.7841
DALYs (Dis	Global	Female	50-69 yea	Other sexu	Rate	2000	5.0127	5.98229	4.09956
DALYs (Dis	Global	Male	50-69 yea	HIV/AIDS	Number	2000	3220685	3670771	2765367
DALYs (Dis	Global	Female	50-69 yea	HIV/AIDS	Number	2000	3610939	4132553	3070741
DALYs (Dis	Global	Male	50-69 yea	HIV/AIDS	Rate	2000	804.896	917.379	691.105
DALYs (Dis	Global	Female	50-69 yea	HIV/AIDS	Rate	2000	884.846	1012.67	752.473
DALYs (Dis	Global	Male	50-69 yea	Sexually tr	Number	2007	52020	83134.2	32103.2
DALYs (Dis	Global	Female	50-69 yea	Sexually tr	Number	2007	128820	216628	82153.5
DALYs (Dis	Global	Male	50-69 yea	Sexually tr	Rate	2007	10.7066	17.1105	6.60742
DALYs (Dis	Global	Female	50-69 yea	Sexually tr	Rate	2007	25.9001	43.5543	16.5175
DALYs (Dis	Global	Male	50-69 yea	Syphilis	Number	2007	19809.8	26743	12141.5
DALYs (Dis	Global	Female	50-69 yea	Syphilis	Number	2007	4375.17	5145.63	3560.86
DALYs (Dis	Global	Male	50-69 yea	Syphilis	Rate	2007	4.07722	5.50418	2.49894
DALYs (Dis	Global	Female	50-69 yea	Syphilis	Rate	2007	0.87965	1.03456	0.71593
DALYs (Dis	Global	Male	50-69 yea	Chlamydia	Number	2007	2382.84	4088.25	1200.15
DALYs (Dis	Global	Female	50-69 yea	Chlamydia	Number	2007	9019.46	10505.1	7579.4
DALYs (Dis	Global	Male	50-69 yea	Chlamydia	Rate	2007	0.49043	0.84144	0.24701
DALYs (Dis	Global	Female	50-69 yea	Chlamydia	Rate	2007	1.81342	2.11211	1.52389
DALYs (Dis	Global	Male	50-69 yea	Gonococca	Number	2007	2795.39	4119.31	1796.93
DALYs (Dis	Global	Female	50-69 yea	Gonococca	Number	2007	23959.3	27040.8	18894.8
DALYs (Dis	Global	Male	50-69 yea	Gonococca	Rate	2007	0.57534	0.84783	0.36984
DALYs (Dis	Global	Female	50-69 yea	Gonococca	Rate	2007	4.81717	5.43672	3.79891
DALYs (Dis	Global	Male	50-69 yea	Trichomon	Number	2007	1509.53	3340.73	540.113
DALYs (Dis	Global	Female	50-69 yea	Trichomon	Number	2007	37176.6	82117.4	13733.5
DALYs (Dis	Global	Male	50-69 yea	Trichomon	Rate	2007	0.31069	0.68758	0.11116
DALYs (Dis	Global	Female	50-69 yea	Trichomon	Rate	2007	7.47458	16.5102	2.76121
DALYs (Dis	Global	Male	50-69 yea	Genital he	Number	2007	20026.3	49746.1	6107.69
DALYs (Dis	Global	Female	50-69 yea	Genital he	Number	2007	32563.2	80040.3	10008.2
DALYs (Dis	Global	Male	50-69 yea	Genital he	Rate	2007	4.12177	10.2386	1.25707
DALYs (Dis	Global	Female	50-69 yea	Genital he	Rate	2007	6.54703	16.0926	2.01222
DALYs (Dis	Global	Male	50-69 yea	Other sexu	Number	2007	5496.12	7570.51	3388.33
DALYs (Dis	Global	Female	50-69 yea	Other sexu	Number	2007	21726.5	26127.5	17894
DALYs (Dis	Global	Male	50-69 yea	Other sexu	Rate	2007	1.1312	1.55815	0.69738
DALYs (Dis	Global	Female	50-69 yea	Other sexu	Rate	2007	4.36824	5.25309	3.5977
DALYs (Dis	Global	Male	50-69 yea	Sexually tr	Number	2004	51870.4	83157.9	32359.3
DALYs (Dis	Global	Female	50-69 yea	Sexually tr	Number	2004	121403	201629	79252.6
DALYs (Dis	Global	Male	50-69 yea	Sexually tr	Rate	2004	11.623	18.6338	7.25099
DALYs (Dis	Global	Female	50-69 yea	Sexually tr	Rate	2004	26.5924	44.1655	17.3597
DALYs (Dis	Global	Male	50-69 yea	Syphilis	Number	2004	21547.4	30040.2	13201.1
DALYs (Dis	Global	Female	50-69 yea	Syphilis	Number	2004	4266.76	5058.49	3476.31
DALYs (Dis	Global	Male	50-69 yea	Syphilis	Rate	2004	4.8283	6.73134	2.95807
DALYs (Dis	Global	Female	50-69 yea	Syphilis	Rate	2004	0.9346	1.10803	0.76146
DALYs (Dis	Global	Male	50-69 yea	Chlamydia	Number	2004	2191.75	3760.37	1138.83
DALYs (Dis	Global	Female	50-69 yea	Chlamydia	Number	2004	8726.72	10145.5	7222.87
DALYs (Dis	Global	Male	50-69 yea	Chlamydia	Rate	2004	0.49112	0.84261	0.25519
DALYs (Dis	Global	Female	50-69 yea	Chlamydia	Rate	2004	1.91153	2.22229	1.58212
DALYs (Dis	Global	Male	50-69 yea	Gonococca	Number	2004	2721.66	3942.86	1720
DALYs (Dis	Global	Female	50-69 yea	Gonococca	Number	2004	23470.1	26877.2	18811.8
DALYs (Dis	Global	Male	50-69 yea	Gonococca	Rate	2004	0.60986	0.88351	0.38541
DALYs (Dis	Global	Female	50-69 yea	Gonococca	Rate	2004	5.14097	5.88726	4.1206
DALYs (Dis	Global	Male	50-69 yea	Trichomon	Number	2004	1376.86	3057.79	491.139
DALYs (Dis	Global	Female	50-69 yea	Trichomon	Number	2004	34113	75551.8	12556.8
DALYs (Dis	Global	Male	50-69 yea	Trichomon	Rate	2004	0.30852	0.68518	0.11005
DALYs (Dis	Global	Female	50-69 yea	Trichomon	Rate	2004	7.47221	16.5491	2.75047
DALYs (Dis	Global	Male	50-69 yea	Genital he	Number	2004	18554.5	46131.6	5656.19
DALYs (Dis	Global	Female	50-69 yea	Genital he	Number	2004	30004.6	73237.7	9179.21
DALYs (Dis	Global	Male	50-69 yea	Genital he	Rate	2004	4.15765	10.3371	1.26743
DALYs (Dis	Global	Female	50-69 yea	Genital he	Rate	2004	6.57231	16.0422	2.01064
DALYs (Dis	Global	Male	50-69 yea	Other sexu	Number	2004	5478.2	7848.95	3361.23
DALYs (Dis	Global	Female	50-69 yea	Other sexu	Number	2004	20821.5	25145.1	17110.8
DALYs (Dis	Global	Male	50-69 yea	Other sexu	Rate	2004	1.22754	1.75878	0.75318
DALYs (Dis	Global	Female	50-69 yea	Other sexu	Rate	2004	4.5608	5.50786	3.748
DALYs (Dis	Global	Male	50-69 yea	HIV/AIDS	Number	2007	4093863	4590778	3648572
DALYs (Dis	Global	Female	50-69 yea	HIV/AIDS	Number	2007	4305413	4757093	3879949
DALYs (Dis	Global	Male	50-69 yea	HIV/AIDS	Rate	2007	842.591	944.865	750.942
DALYs (Dis	Global	Female	50-69 yea	HIV/AIDS	Rate	2007	865.63	956.443	780.088
DALYs (Dis	Global	Male	50-69 yea	HIV/AIDS	Number	2004	4219571	4566506	3905405
DALYs (Dis	Global	Female	50-69 yea	HIV/AIDS	Number	2004	4619409	5013026	4264986
DALYs (Dis	Global	Male	50-69 yea	HIV/AIDS	Rate	2004	945.512	1023.25	875.114
DALYs (Dis	Global	Female	50-69 yea	HIV/AIDS	Rate	2004	1011.85	1098.07	934.215
DALYs (Dis	Global	Male	50-69 yea	HIV/AIDS	Number	2003	4040504	4410027	3695326
DALYs (Dis	Global	Female	50-69 yea	HIV/AIDS	Number	2003	4456377	4891569	4037540
DALYs (Dis	Global	Male	50-69 yea	HIV/AIDS	Rate	2003	931.892	1017.12	852.281
DALYs (Dis	Global	Female	50-69 yea	HIV/AIDS	Rate	2003	1005.34	1103.52	910.852
DALYs (Dis	Global	Male	50-69 yea	Sexually tr	Number	2003	52500.9	83640.8	33353.4
DALYs (Dis	Global	Female	50-69 yea	Sexually tr	Number	2003	120254	199970	79436.1
DALYs (Dis	Global	Male	50-69 yea	Sexually tr	Rate	2003	12.1087	19.2907	7.69253
DALYs (Dis	Global	Female	50-69 yea	Sexually tr	Rate	2003	27.1287	45.1124	17.9204
DALYs (Dis	Global	Male	50-69 yea	Syphilis	Number	2003	22851.8	33073.5	14033.4
DALYs (Dis	Global	Female	50-69 yea	Syphilis	Number	2003	4341.51	5032.9	3498.01
DALYs (Dis	Global	Male	50-69 yea	Syphilis	Rate	2003	5.27049	7.62798	3.23664
DALYs (Dis	Global	Female	50-69 yea	Syphilis	Rate	2003	0.97943	1.1354	0.78914
DALYs (Dis	Global	Male	50-69 yea	Chlamydia	Number	2003	2113.2	3637.44	1074.59
DALYs (Dis	Global	Female	50-69 yea	Chlamydia	Number	2003	8827.55	10244.6	7358.55
DALYs (Dis	Global	Male	50-69 yea	Chlamydia	Rate	2003	0.48738	0.83893	0.24784
DALYs (Dis	Global	Female	50-69 yea	Chlamydia	Rate	2003	1.99146	2.31114	1.66006
DALYs (Dis	Global	Male	50-69 yea	Gonococca	Number	2003	2708.97	3902.49	1696.9
DALYs (Dis	Global	Female	50-69 yea	Gonococca	Number	2003	23985.1	27305.5	19163.3
DALYs (Dis	Global	Male	50-69 yea	Gonococca	Rate	2003	0.62479	0.90006	0.39137
DALYs (Dis	Global	Female	50-69 yea	Gonococca	Rate	2003	5.41095	6.16	4.32315
DALYs (Dis	Global	Male	50-69 yea	Trichomon	Number	2003	1333.37	2971.89	478.421
DALYs (Dis	Global	Female	50-69 yea	Trichomon	Number	2003	33085.7	73193.8	12219.2
DALYs (Dis	Global	Male	50-69 yea	Trichomon	Rate	2003	0.30753	0.68543	0.11034
DALYs (Dis	Global	Female	50-69 yea	Trichomon	Rate	2003	7.46401	16.5122	2.75661
DALYs (Dis	Global	Male	50-69 yea	Genital he	Number	2003	18010.2	44798	5480.06
DALYs (Dis	Global	Female	50-69 yea	Genital he	Number	2003	29076.6	71244.3	8916.66
DALYs (Dis	Global	Male	50-69 yea	Genital he	Rate	2003	4.15383	10.3321	1.26391
DALYs (Dis	Global	Female	50-69 yea	Genital he	Rate	2003	6.55956	16.0724	2.01156
DALYs (Dis	Global	Male	50-69 yea	Other sexu	Number	2003	5483.33	7922.22	3283.62
DALYs (Dis	Global	Female	50-69 yea	Other sexu	Number	2003	20937.1	24835.1	17254.3
DALYs (Dis	Global	Male	50-69 yea	Other sexu	Rate	2003	1.26466	1.82716	0.75733
DALYs (Dis	Global	Female	50-69 yea	Other sexu	Rate	2003	4.72333	5.6027	3.89251
DALYs (Dis	Global	Male	50-69 yea	Sexually tr	Number	2006	51762.7	83051.4	32426
DALYs (Dis	Global	Female	50-69 yea	Sexually tr	Number	2006	126547	213172	81494.2
DALYs (Dis	Global	Male	50-69 yea	Sexually tr	Rate	2006	10.9479	17.5656	6.85817
DALYs (Dis	Global	Female	50-69 yea	Sexually tr	Rate	2006	26.1481	44.0471	16.8389
DALYs (Dis	Global	Male	50-69 yea	Syphilis	Number	2006	20036.1	27230.5	12157.2
DALYs (Dis	Global	Female	50-69 yea	Syphilis	Number	2006	4356.29	5127.68	3583.13
DALYs (Dis	Global	Male	50-69 yea	Syphilis	Rate	2006	4.23768	5.7593	2.57127
DALYs (Dis	Global	Female	50-69 yea	Syphilis	Rate	2006	0.90013	1.05952	0.74037
DALYs (Dis	Global	Male	50-69 yea	Chlamydia	Number	2006	2350.79	4083.58	1216.42
DALYs (Dis	Global	Female	50-69 yea	Chlamydia	Number	2006	8911.37	10396.4	7457.01
DALYs (Dis	Global	Male	50-69 yea	Chlamydia	Rate	2006	0.4972	0.86369	0.25728
DALYs (Dis	Global	Female	50-69 yea	Chlamydia	Rate	2006	1.84133	2.14818	1.54082
DALYs (Dis	Global	Male	50-69 yea	Gonococca	Number	2006	2771.06	4024.25	1777.01
DALYs (Dis	Global	Female	50-69 yea	Gonococca	Number	2006	23773.7	26944.8	19024.5
DALYs (Dis	Global	Male	50-69 yea	Gonococca	Rate	2006	0.58609	0.85114	0.37584
DALYs (Dis	Global	Female	50-69 yea	Gonococca	Rate	2006	4.91229	5.56753	3.93098
DALYs (Dis	Global	Male	50-69 yea	Trichomon	Number	2006	1469.17	3259.6	518.051
DALYs (Dis	Global	Female	50-69 yea	Trichomon	Number	2006	36255.2	80048	13356
DALYs (Dis	Global	Male	50-69 yea	Trichomon	Rate	2006	0.31073	0.68941	0.10957
DALYs (Dis	Global	Female	50-69 yea	Trichomon	Rate	2006	7.4913	16.5401	2.75971
DALYs (Dis	Global	Male	50-69 yea	Genital he	Number	2006	19634.7	48955.2	6000.92
DALYs (Dis	Global	Female	50-69 yea	Genital he	Number	2006	31828.6	77944.7	9815.06
DALYs (Dis	Global	Male	50-69 yea	Genital he	Rate	2006	4.15277	10.3541	1.26921
DALYs (Dis	Global	Female	50-69 yea	Genital he	Rate	2006	6.57664	16.1055	2.02806
DALYs (Dis	Global	Male	50-69 yea	Other sexu	Number	2006	5500.92	7635.98	3416.76
DALYs (Dis	Global	Female	50-69 yea	Other sexu	Number	2006	21422.4	25812.9	17704.1
DALYs (Dis	Global	Male	50-69 yea	Other sexu	Rate	2006	1.16346	1.61502	0.72265
DALYs (Dis	Global	Female	50-69 yea	Other sexu	Rate	2006	4.42644	5.33364	3.65815
DALYs (Dis	Global	Male	50-69 yea	HIV/AIDS	Number	2009	3761061	4394680	3301177
DALYs (Dis	Global	Female	50-69 yea	HIV/AIDS	Number	2009	3798867	4359071	3352895
DALYs (Dis	Global	Male	50-69 yea	HIV/AIDS	Rate	2009	735.263	859.132	645.359
DALYs (Dis	Global	Female	50-69 yea	HIV/AIDS	Rate	2009	724.45	831.282	639.403
DALYs (Dis	Global	Male	50-69 yea	HIV/AIDS	Number	2005	4304986	4679937	3955807
DALYs (Dis	Global	Female	50-69 yea	HIV/AIDS	Number	2005	4659413	5008644	4310152
DALYs (Dis	Global	Male	50-69 yea	HIV/AIDS	Rate	2005	936.744	1018.33	860.764
DALYs (Dis	Global	Female	50-69 yea	HIV/AIDS	Rate	2005	990.622	1064.87	916.367
DALYs (Dis	Global	Male	50-69 yea	HIV/AIDS	Number	2008	3928633	4478075	3466086
DALYs (Dis	Global	Female	50-69 yea	HIV/AIDS	Number	2008	4040770	4541938	3598482
DALYs (Dis	Global	Male	50-69 yea	HIV/AIDS	Rate	2008	787.872	898.06	695.11
DALYs (Dis	Global	Female	50-69 yea	HIV/AIDS	Rate	2008	791.313	889.458	704.699
DALYs (Dis	Global	Male	50-69 yea	Sexually tr	Number	2012	55025.9	91604.9	34050.2
DALYs (Dis	Global	Female	50-69 yea	Sexually tr	Number	2012	140979	241789	89408.1
DALYs (Dis	Global	Male	50-69 yea	Sexually tr	Rate	2012	9.90788	16.4942	6.13104
DALYs (Dis	Global	Female	50-69 yea	Sexually tr	Rate	2012	24.6557	42.2863	15.6365
DALYs (Dis	Global	Male	50-69 yea	Syphilis	Number	2012	19508.5	25726	12125.8
DALYs (Dis	Global	Female	50-69 yea	Syphilis	Number	2012	4598.98	5326.18	3764.82
DALYs (Dis	Global	Male	50-69 yea	Syphilis	Rate	2012	3.51267	4.63219	2.18335
DALYs (Dis	Global	Female	50-69 yea	Syphilis	Rate	2012	0.80431	0.93149	0.65842
DALYs (Dis	Global	Male	50-69 yea	Chlamydia	Number	2012	2532.25	4296.25	1320.37
DALYs (Dis	Global	Female	50-69 yea	Chlamydia	Number	2012	9388.55	10881.5	8007.78
DALYs (Dis	Global	Male	50-69 yea	Chlamydia	Rate	2012	0.45595	0.77358	0.23774
DALYs (Dis	Global	Female	50-69 yea	Chlamydia	Rate	2012	1.64195	1.90306	1.40047
DALYs (Dis	Global	Male	50-69 yea	Gonococca	Number	2012	2934.82	4410.72	1875.9
DALYs (Dis	Global	Female	50-69 yea	Gonococca	Number	2012	24660	27524.5	19771.7
DALYs (Dis	Global	Male	50-69 yea	Gonococca	Rate	2012	0.52844	0.79419	0.33777
DALYs (Dis	Global	Female	50-69 yea	Gonococca	Rate	2012	4.31276	4.81373	3.45785
DALYs (Dis	Global	Male	50-69 yea	Trichomon	Number	2012	1697.79	3751.97	616.323
DALYs (Dis	Global	Female	50-69 yea	Trichomon	Number	2012	42535.5	94690.3	15700.4
DALYs (Dis	Global	Male	50-69 yea	Trichomon	Rate	2012	0.3057	0.67557	0.11097
DALYs (Dis	Global	Female	50-69 yea	Trichomon	Rate	2012	7.43899	16.5603	2.74582
DALYs (Dis	Global	Male	50-69 yea	Genital he	Number	2012	22851.3	56977.8	6969.02
DALYs (Dis	Global	Female	50-69 yea	Genital he	Number	2012	37086.4	91749.5	11396.8
DALYs (Dis	Global	Male	50-69 yea	Genital he	Rate	2012	4.11458	10.2593	1.25483
DALYs (Dis	Global	Female	50-69 yea	Genital he	Rate	2012	6.486	16.046	1.99317
DALYs (Dis	Global	Male	50-69 yea	Other sexu	Number	2012	5501.23	7512.94	3453.73
DALYs (Dis	Global	Female	50-69 yea	Other sexu	Number	2012	22709.7	27325.3	18884.4
DALYs (Dis	Global	Male	50-69 yea	Other sexu	Rate	2012	0.99054	1.35277	0.62187
DALYs (Dis	Global	Female	50-69 yea	Other sexu	Rate	2012	3.97167	4.77889	3.30267
DALYs (Dis	Global	Male	50-69 yea	Sexually tr	Number	2008	52299.3	84602.5	32481.8
DALYs (Dis	Global	Female	50-69 yea	Sexually tr	Number	2008	130436	219727	83036.6
DALYs (Dis	Global	Male	50-69 yea	Sexually tr	Rate	2008	10.4884	16.9667	6.5141
DALYs (Dis	Global	Female	50-69 yea	Sexually tr	Rate	2008	25.5435	43.0295	16.2612
DALYs (Dis	Global	Male	50-69 yea	Syphilis	Number	2008	19691.7	26413.6	12139.7
DALYs (Dis	Global	Female	50-69 yea	Syphilis	Number	2008	4366.06	5075.53	3493.15
DALYs (Dis	Global	Male	50-69 yea	Syphilis	Rate	2008	3.94908	5.29714	2.43458
DALYs (Dis	Global	Female	50-69 yea	Syphilis	Rate	2008	0.85501	0.99395	0.68407
DALYs (Dis	Global	Male	50-69 yea	Chlamydia	Number	2008	2387.33	4067.58	1222.4
DALYs (Dis	Global	Female	50-69 yea	Chlamydia	Number	2008	9048.08	10486.8	7501.3
DALYs (Dis	Global	Male	50-69 yea	Chlamydia	Rate	2008	0.47877	0.81574	0.24515
DALYs (Dis	Global	Female	50-69 yea	Chlamydia	Rate	2008	1.77191	2.05366	1.469
DALYs (Dis	Global	Male	50-69 yea	Gonococca	Number	2008	2818.21	4179.49	1799.43
DALYs (Dis	Global	Female	50-69 yea	Gonococca	Number	2008	23933.4	26905.9	18758.6
DALYs (Dis	Global	Male	50-69 yea	Gonococca	Rate	2008	0.56518	0.83818	0.36087
DALYs (Dis	Global	Female	50-69 yea	Gonococca	Rate	2008	4.68692	5.26904	3.67354
DALYs (Dis	Global	Male	50-69 yea	Trichomon	Number	2008	1546.5	3401	551.384
DALYs (Dis	Global	Female	50-69 yea	Trichomon	Number	2008	38041.5	84305.3	14080.2
DALYs (Dis	Global	Male	50-69 yea	Trichomon	Rate	2008	0.31014	0.68206	0.11058
DALYs (Dis	Global	Female	50-69 yea	Trichomon	Rate	2008	7.44976	16.5097	2.75736
DALYs (Dis	Global	Male	50-69 yea	Genital he	Number	2008	20378.6	50741.7	6220.26
DALYs (Dis	Global	Female	50-69 yea	Genital he	Number	2008	33242.6	81768.4	10185.1
DALYs (Dis	Global	Male	50-69 yea	Genital he	Rate	2008	4.08684	10.176	1.24745
DALYs (Dis	Global	Female	50-69 yea	Genital he	Rate	2008	6.50997	16.0129	1.99456
DALYs (Dis	Global	Male	50-69 yea	Other sexu	Number	2008	5477.03	7642.62	3381.72
DALYs (Dis	Global	Female	50-69 yea	Other sexu	Number	2008	21804.2	26247	17987.1
DALYs (Dis	Global	Male	50-69 yea	Other sexu	Rate	2008	1.0984	1.5327	0.67819
DALYs (Dis	Global	Female	50-69 yea	Other sexu	Rate	2008	4.26997	5.14	3.52245
DALYs (Dis	Global	Male	50-69 yea	Sexually tr	Number	2011	53944.8	89349.6	33488
DALYs (Dis	Global	Female	50-69 yea	Sexually tr	Number	2011	137507	235052	86400.4
DALYs (Dis	Global	Male	50-69 yea	Sexually tr	Rate	2011	9.99849	16.5607	6.20689
DALYs (Dis	Global	Female	50-69 yea	Sexually tr	Rate	2011	24.7949	42.3838	15.5794
DALYs (Dis	Global	Male	50-69 yea	Syphilis	Number	2011	19483	25906.5	12082.8
DALYs (Dis	Global	Female	50-69 yea	Syphilis	Number	2011	4519.83	5219.04	3689.55
DALYs (Dis	Global	Male	50-69 yea	Syphilis	Rate	2011	3.61111	4.80168	2.23952
DALYs (Dis	Global	Female	50-69 yea	Syphilis	Rate	2011	0.815	0.94108	0.66529
DALYs (Dis	Global	Male	50-69 yea	Chlamydia	Number	2011	2458.27	4212.63	1285.63
DALYs (Dis	Global	Female	50-69 yea	Chlamydia	Number	2011	9249.83	10687.2	7738.07
DALYs (Dis	Global	Male	50-69 yea	Chlamydia	Rate	2011	0.45563	0.7808	0.23829
DALYs (Dis	Global	Female	50-69 yea	Chlamydia	Rate	2011	1.6679	1.92708	1.3953
DALYs (Dis	Global	Male	50-69 yea	Gonococca	Number	2011	2897.32	4327.52	1844.63
DALYs (Dis	Global	Female	50-69 yea	Gonococca	Number	2011	24345.7	27112	19648.9
DALYs (Dis	Global	Male	50-69 yea	Gonococca	Rate	2011	0.53701	0.80209	0.3419
DALYs (Dis	Global	Female	50-69 yea	Gonococca	Rate	2011	4.38993	4.88874	3.54302
DALYs (Dis	Global	Male	50-69 yea	Trichomon	Number	2011	1655.82	3694.89	591.655
DALYs (Dis	Global	Female	50-69 yea	Trichomon	Number	2011	41159.1	91515.6	15094.3
DALYs (Dis	Global	Male	50-69 yea	Trichomon	Rate	2011	0.3069	0.68484	0.10966
DALYs (Dis	Global	Female	50-69 yea	Trichomon	Rate	2011	7.42167	16.5018	2.72175
DALYs (Dis	Global	Male	50-69 yea	Genital he	Number	2011	21993.6	54700.5	6699.69
DALYs (Dis	Global	Female	50-69 yea	Genital he	Number	2011	35900.7	88542.9	11025.3
DALYs (Dis	Global	Male	50-69 yea	Genital he	Rate	2011	4.07645	10.1386	1.24176
DALYs (Dis	Global	Female	50-69 yea	Genital he	Rate	2011	6.47349	15.9658	1.98804
DALYs (Dis	Global	Male	50-69 yea	Other sexu	Number	2011	5456.72	7491.31	3408.82
DALYs (Dis	Global	Female	50-69 yea	Other sexu	Number	2011	22332.3	26735.6	18515.8
DALYs (Dis	Global	Male	50-69 yea	Other sexu	Rate	2011	1.01138	1.38849	0.63181
DALYs (Dis	Global	Female	50-69 yea	Other sexu	Rate	2011	4.02688	4.82087	3.33871
DALYs (Dis	Global	Male	50-69 yea	Sexually tr	Number	2014	57545.7	97528	35354.7
DALYs (Dis	Global	Female	50-69 yea	Sexually tr	Number	2014	151722	259827	95812.2
DALYs (Dis	Global	Male	50-69 yea	Sexually tr	Rate	2014	9.73506	16.4989	5.98099
DALYs (Dis	Global	Female	50-69 yea	Sexually tr	Rate	2014	24.8425	42.5432	15.6879
DALYs (Dis	Global	Male	50-69 yea	Syphilis	Number	2014	19595.4	25863.3	12374.5
DALYs (Dis	Global	Female	50-69 yea	Syphilis	Number	2014	5036.81	5858.6	4029.19
DALYs (Dis	Global	Male	50-69 yea	Syphilis	Rate	2014	3.31497	4.37533	2.0934
DALYs (Dis	Global	Female	50-69 yea	Syphilis	Rate	2014	0.82471	0.95927	0.65972
DALYs (Dis	Global	Male	50-69 yea	Chlamydia	Number	2014	2738	4671.51	1401.79
DALYs (Dis	Global	Female	50-69 yea	Chlamydia	Number	2014	10155.7	11893.5	8325.22
DALYs (Dis	Global	Male	50-69 yea	Chlamydia	Rate	2014	0.46319	0.79028	0.23714
DALYs (Dis	Global	Female	50-69 yea	Chlamydia	Rate	2014	1.66286	1.94739	1.36314
DALYs (Dis	Global	Male	50-69 yea	Gonococca	Number	2014	3030.82	4575.52	1950.75
DALYs (Dis	Global	Female	50-69 yea	Gonococca	Number	2014	26564	30046.9	20911.4
DALYs (Dis	Global	Male	50-69 yea	Gonococca	Rate	2014	0.51273	0.77405	0.33001
DALYs (Dis	Global	Female	50-69 yea	Gonococca	Rate	2014	4.34949	4.91977	3.42395
DALYs (Dis	Global	Male	50-69 yea	Trichomon	Number	2014	1803.44	4004.03	643.532
DALYs (Dis	Global	Female	50-69 yea	Trichomon	Number	2014	45706.8	101949	16842.4
DALYs (Dis	Global	Male	50-69 yea	Trichomon	Rate	2014	0.30509	0.67737	0.10887
DALYs (Dis	Global	Female	50-69 yea	Trichomon	Rate	2014	7.48386	16.6927	2.75771
DALYs (Dis	Global	Male	50-69 yea	Genital he	Number	2014	24771.8	61693.7	7598.16
DALYs (Dis	Global	Female	50-69 yea	Genital he	Number	2014	39775.2	98049.5	12259.6
DALYs (Dis	Global	Male	50-69 yea	Genital he	Rate	2014	4.19067	10.4368	1.28539
DALYs (Dis	Global	Female	50-69 yea	Genital he	Rate	2014	6.51265	16.0543	2.00734
DALYs (Dis	Global	Male	50-69 yea	Other sexu	Number	2014	5606.23	7781.15	3518.51
DALYs (Dis	Global	Female	50-69 yea	Other sexu	Number	2014	24483.9	29548.8	20209.2
DALYs (Dis	Global	Male	50-69 yea	Other sexu	Rate	2014	0.94841	1.31634	0.59523
DALYs (Dis	Global	Female	50-69 yea	Other sexu	Rate	2014	4.00891	4.83822	3.30898
DALYs (Dis	Global	Male	50-69 yea	HIV/AIDS	Number	2011	3648669	4296913	3237946
DALYs (Dis	Global	Female	50-69 yea	HIV/AIDS	Number	2011	3428674	4021206	3044900
DALYs (Dis	Global	Male	50-69 yea	HIV/AIDS	Rate	2011	676.269	796.418	600.142
DALYs (Dis	Global	Female	50-69 yea	HIV/AIDS	Rate	2011	618.247	725.09	549.046
DALYs (Dis	Global	Male	50-69 yea	Sexually tr	Number	2010	53184.3	87881.8	32768.2
DALYs (Dis	Global	Female	50-69 yea	Sexually tr	Number	2010	134314	229614	84329.5
DALYs (Dis	Global	Male	50-69 yea	Sexually tr	Rate	2010	10.1289	16.7371	6.24071
DALYs (Dis	Global	Female	50-69 yea	Sexually tr	Rate	2010	24.9244	42.609	15.6488
DALYs (Dis	Global	Male	50-69 yea	Syphilis	Number	2010	19529.1	25972.3	12012.1
DALYs (Dis	Global	Female	50-69 yea	Syphilis	Number	2010	4428.18	5083.03	3604.18
DALYs (Dis	Global	Male	50-69 yea	Syphilis	Rate	2010	3.71931	4.94642	2.2877
DALYs (Dis	Global	Female	50-69 yea	Syphilis	Rate	2010	0.82173	0.94325	0.66882
DALYs (Dis	Global	Male	50-69 yea	Chlamydia	Number	2010	2419.69	4153.47	1245.97
DALYs (Dis	Global	Female	50-69 yea	Chlamydia	Number	2010	9102.48	10524.1	7656.54
DALYs (Dis	Global	Male	50-69 yea	Chlamydia	Rate	2010	0.46083	0.79103	0.23729
DALYs (Dis	Global	Female	50-69 yea	Chlamydia	Rate	2010	1.68913	1.95294	1.42081
DALYs (Dis	Global	Male	50-69 yea	Gonococca	Number	2010	2869.75	4271.17	1831.37
DALYs (Dis	Global	Female	50-69 yea	Gonococca	Number	2010	23995.3	26808.1	19503.7
DALYs (Dis	Global	Male	50-69 yea	Gonococca	Rate	2010	0.54654	0.81344	0.34878
DALYs (Dis	Global	Female	50-69 yea	Gonococca	Rate	2010	4.45274	4.97471	3.61925
DALYs (Dis	Global	Male	50-69 yea	Trichomon	Number	2010	1616.71	3605.5	575.944
DALYs (Dis	Global	Female	50-69 yea	Trichomon	Number	2010	39968.8	88718.2	14704.4
DALYs (Dis	Global	Male	50-69 yea	Trichomon	Rate	2010	0.3079	0.68667	0.10969
DALYs (Dis	Global	Female	50-69 yea	Trichomon	Rate	2010	7.41692	16.4632	2.72865
DALYs (Dis	Global	Male	50-69 yea	Genital he	Number	2010	21292.6	52680.2	6476.17
DALYs (Dis	Global	Female	50-69 yea	Genital he	Number	2010	34846.2	85797.1	10735.8
DALYs (Dis	Global	Male	50-69 yea	Genital he	Rate	2010	4.05518	10.0329	1.23338
DALYs (Dis	Global	Female	50-69 yea	Genital he	Rate	2010	6.46634	15.9212	1.99222
DALYs (Dis	Global	Male	50-69 yea	Other sexu	Number	2010	5456.42	7480.48	3382.5
DALYs (Dis	Global	Female	50-69 yea	Other sexu	Number	2010	21973.1	26551.8	18461.2
DALYs (Dis	Global	Male	50-69 yea	Other sexu	Rate	2010	1.03917	1.42466	0.6442
DALYs (Dis	Global	Female	50-69 yea	Other sexu	Rate	2010	4.07751	4.92715	3.4258
DALYs (Dis	Global	Male	50-69 yea	HIV/AIDS	Number	2013	3643342	4257355	3275506
DALYs (Dis	Global	Female	50-69 yea	HIV/AIDS	Number	2013	3136981	3652303	2810823
DALYs (Dis	Global	Male	50-69 yea	HIV/AIDS	Rate	2013	636.148	743.358	571.922
DALYs (Dis	Global	Female	50-69 yea	HIV/AIDS	Rate	2013	531.007	618.237	475.797
DALYs (Dis	Global	Male	50-69 yea	Sexually tr	Number	2016	60237.8	102969	37027.3
DALYs (Dis	Global	Female	50-69 yea	Sexually tr	Number	2016	163358	281739	103180
DALYs (Dis	Global	Male	50-69 yea	Sexually tr	Rate	2016	9.59388	16.3995	5.89721
DALYs (Dis	Global	Female	50-69 yea	Sexually tr	Rate	2016	25.1001	43.2895	15.8537
DALYs (Dis	Global	Male	50-69 yea	Syphilis	Number	2016	19998.2	26222.3	12675.1
DALYs (Dis	Global	Female	50-69 yea	Syphilis	Number	2016	5498.82	6456.15	4340.44
DALYs (Dis	Global	Male	50-69 yea	Syphilis	Rate	2016	3.18505	4.17634	2.01871
DALYs (Dis	Global	Female	50-69 yea	Syphilis	Rate	2016	0.8449	0.99199	0.66691
DALYs (Dis	Global	Male	50-69 yea	Chlamydia	Number	2016	2943.94	4996.95	1517.46
DALYs (Dis	Global	Female	50-69 yea	Chlamydia	Number	2016	11040.9	13050.8	8876.73
DALYs (Dis	Global	Male	50-69 yea	Chlamydia	Rate	2016	0.46887	0.79585	0.24168
DALYs (Dis	Global	Female	50-69 yea	Chlamydia	Rate	2016	1.69645	2.00528	1.36392
DALYs (Dis	Global	Male	50-69 yea	Gonococca	Number	2016	3149.8	4721.47	2023
DALYs (Dis	Global	Female	50-69 yea	Gonococca	Number	2016	28994.4	33712.2	22259.7
DALYs (Dis	Global	Male	50-69 yea	Gonococca	Rate	2016	0.50166	0.75197	0.3222
DALYs (Dis	Global	Female	50-69 yea	Gonococca	Rate	2016	4.45502	5.17992	3.42023
DALYs (Dis	Global	Male	50-69 yea	Trichomon	Number	2016	1925.64	4248.97	690.021
DALYs (Dis	Global	Female	50-69 yea	Trichomon	Number	2016	48735.2	109288	18073.1
DALYs (Dis	Global	Male	50-69 yea	Trichomon	Rate	2016	0.30669	0.67672	0.1099
DALYs (Dis	Global	Female	50-69 yea	Trichomon	Rate	2016	7.48821	16.7922	2.77695
DALYs (Dis	Global	Male	50-69 yea	Genital he	Number	2016	26444.5	65935.9	8086.05
DALYs (Dis	Global	Female	50-69 yea	Genital he	Number	2016	42479.8	104430	13052
DALYs (Dis	Global	Male	50-69 yea	Genital he	Rate	2016	4.21173	10.5014	1.28784
DALYs (Dis	Global	Female	50-69 yea	Genital he	Rate	2016	6.52707	16.0458	2.00546
DALYs (Dis	Global	Male	50-69 yea	Other sexu	Number	2016	5775.78	8022.01	3651.48
DALYs (Dis	Global	Female	50-69 yea	Other sexu	Number	2016	26608.9	32188.5	21542.1
DALYs (Dis	Global	Male	50-69 yea	Other sexu	Rate	2016	0.91989	1.27764	0.58156
DALYs (Dis	Global	Female	50-69 yea	Other sexu	Rate	2016	4.08849	4.94579	3.30997
DALYs (Dis	Global	Male	50-69 yea	HIV/AIDS	Number	2012	3631457	4262640	3241025
DALYs (Dis	Global	Female	50-69 yea	HIV/AIDS	Number	2012	3248050	3815124	2892355
DALYs (Dis	Global	Male	50-69 yea	HIV/AIDS	Rate	2012	653.875	767.525	583.574
DALYs (Dis	Global	Female	50-69 yea	HIV/AIDS	Rate	2012	568.048	667.223	505.841
DALYs (Dis	Global	Male	50-69 yea	HIV/AIDS	Number	2016	3790221	4395198	3405817
DALYs (Dis	Global	Female	50-69 yea	HIV/AIDS	Number	2016	3125326	3561231	2824572
DALYs (Dis	Global	Male	50-69 yea	HIV/AIDS	Rate	2016	603.656	700.008	542.433
DALYs (Dis	Global	Female	50-69 yea	HIV/AIDS	Rate	2016	480.21	547.187	433.999
DALYs (Dis	Global	Male	50-69 yea	HIV/AIDS	Number	2019	3675972	4354434	3236144
DALYs (Dis	Global	Female	50-69 yea	HIV/AIDS	Number	2019	3052071	3522125	2709078
DALYs (Dis	Global	Male	50-69 yea	HIV/AIDS	Rate	2019	543.687	644.033	478.635
DALYs (Dis	Global	Female	50-69 yea	HIV/AIDS	Rate	2019	434.255	501.136	385.454
DALYs (Dis	Global	Male	50-69 yea	Sexually tr	Number	2019	63268.6	109656	38181.8
DALYs (Dis	Global	Female	50-69 yea	Sexually tr	Number	2019	171766	297871	107459
DALYs (Dis	Global	Male	50-69 yea	Sexually tr	Rate	2019	9.3576	16.2185	5.6472
DALYs (Dis	Global	Female	50-69 yea	Sexually tr	Rate	2019	24.4393	42.3817	15.2894
DALYs (Dis	Global	Male	50-69 yea	Syphilis	Number	2019	19992.8	25835.8	12871.9
DALYs (Dis	Global	Female	50-69 yea	Syphilis	Number	2019	5694.33	6869.07	4439.93
DALYs (Dis	Global	Male	50-69 yea	Syphilis	Rate	2019	2.95699	3.82119	1.90378
DALYs (Dis	Global	Female	50-69 yea	Syphilis	Rate	2019	0.8102	0.97735	0.63172
DALYs (Dis	Global	Male	50-69 yea	Chlamydia	Number	2019	3289.73	5687.99	1690.47
DALYs (Dis	Global	Female	50-69 yea	Chlamydia	Number	2019	11178.4	13589.3	8892.17
DALYs (Dis	Global	Male	50-69 yea	Chlamydia	Rate	2019	0.48656	0.84127	0.25002
DALYs (Dis	Global	Female	50-69 yea	Chlamydia	Rate	2019	1.59048	1.93351	1.2652
DALYs (Dis	Global	Male	50-69 yea	Gonococca	Number	2019	3291.23	5033.65	2110.81
DALYs (Dis	Global	Female	50-69 yea	Gonococca	Number	2019	29080.7	34796.9	21820.8
DALYs (Dis	Global	Male	50-69 yea	Gonococca	Rate	2019	0.48678	0.74449	0.31219
DALYs (Dis	Global	Female	50-69 yea	Gonococca	Rate	2019	4.13767	4.95098	3.10471
DALYs (Dis	Global	Male	50-69 yea	Trichomon	Number	2019	2082.12	4621.9	741.276
DALYs (Dis	Global	Female	50-69 yea	Trichomon	Number	2019	52618	117498	19394.2
DALYs (Dis	Global	Male	50-69 yea	Trichomon	Rate	2019	0.30795	0.68359	0.10964
DALYs (Dis	Global	Female	50-69 yea	Trichomon	Rate	2019	7.4866	16.7178	2.75945
DALYs (Dis	Global	Male	50-69 yea	Genital he	Number	2019	28581.9	71166.5	8757.37
DALYs (Dis	Global	Female	50-69 yea	Genital he	Number	2019	46033.5	113808	14092.7
DALYs (Dis	Global	Male	50-69 yea	Genital he	Rate	2019	4.22735	10.5257	1.29524
DALYs (Dis	Global	Female	50-69 yea	Genital he	Rate	2019	6.54975	16.1929	2.00514
DALYs (Dis	Global	Male	50-69 yea	Other sexu	Number	2019	6030.77	8444.68	3887.9
DALYs (Dis	Global	Female	50-69 yea	Other sexu	Number	2019	27161.3	33422.3	21979.8
DALYs (Dis	Global	Male	50-69 yea	Other sexu	Rate	2019	0.89197	1.24899	0.57503
DALYs (Dis	Global	Female	50-69 yea	Other sexu	Rate	2019	3.86456	4.7554	3.12734
DALYs (Dis	Global	Male	50-69 yea	HIV/AIDS	Number	2015	3737618	4319695	3368302
DALYs (Dis	Global	Female	50-69 yea	HIV/AIDS	Number	2015	3111049	3559506	2811705
DALYs (Dis	Global	Male	50-69 yea	HIV/AIDS	Rate	2015	612.726	708.148	552.182
DALYs (Dis	Global	Female	50-69 yea	HIV/AIDS	Rate	2015	492.841	563.883	445.42
DALYs (Dis	Global	Male	50-69 yea	Sexually tr	Number	2015	59023.5	100928	36466.2
DALYs (Dis	Global	Female	50-69 yea	Sexually tr	Number	2015	159182	272360	101039
DALYs (Dis	Global	Male	50-69 yea	Sexually tr	Rate	2015	9.676	16.5455	5.97808
DALYs (Dis	Global	Female	50-69 yea	Sexually tr	Rate	2015	25.217	43.1462	16.0063
DALYs (Dis	Global	Male	50-69 yea	Syphilis	Number	2015	19860.5	26186.8	12555.4
DALYs (Dis	Global	Female	50-69 yea	Syphilis	Number	2015	5372.73	6297.46	4240.29
DALYs (Dis	Global	Male	50-69 yea	Syphilis	Rate	2015	3.25582	4.29293	2.05827
DALYs (Dis	Global	Female	50-69 yea	Syphilis	Rate	2015	0.85113	0.99762	0.67173
DALYs (Dis	Global	Male	50-69 yea	Chlamydia	Number	2015	2847.38	4831.9	1449.3
DALYs (Dis	Global	Female	50-69 yea	Chlamydia	Number	2015	10834.6	12734.2	8749.72
DALYs (Dis	Global	Male	50-69 yea	Chlamydia	Rate	2015	0.46678	0.79212	0.23759
DALYs (Dis	Global	Female	50-69 yea	Chlamydia	Rate	2015	1.71638	2.0173	1.3861
DALYs (Dis	Global	Male	50-69 yea	Gonococca	Number	2015	3097.41	4670.63	1993.47
DALYs (Dis	Global	Female	50-69 yea	Gonococca	Number	2015	28475.6	32906	21476.5
DALYs (Dis	Global	Male	50-69 yea	Gonococca	Rate	2015	0.50777	0.76568	0.3268
DALYs (Dis	Global	Female	50-69 yea	Gonococca	Rate	2015	4.51099	5.21284	3.40223
DALYs (Dis	Global	Male	50-69 yea	Trichomon	Number	2015	1866.25	4121.5	674.842
DALYs (Dis	Global	Female	50-69 yea	Trichomon	Number	2015	47303.1	105563	17468.4
DALYs (Dis	Global	Male	50-69 yea	Trichomon	Rate	2015	0.30594	0.67566	0.11063
DALYs (Dis	Global	Female	50-69 yea	Trichomon	Rate	2015	7.49357	16.7229	2.76728
DALYs (Dis	Global	Male	50-69 yea	Genital he	Number	2015	25656.6	64272.1	7861.55
DALYs (Dis	Global	Female	50-69 yea	Genital he	Number	2015	41154.3	101087	12695.6
DALYs (Dis	Global	Male	50-69 yea	Genital he	Rate	2015	4.206	10.5364	1.28878
DALYs (Dis	Global	Female	50-69 yea	Genital he	Rate	2015	6.5195	16.0139	2.01119
DALYs (Dis	Global	Male	50-69 yea	Other sexu	Number	2015	5695.4	7945.89	3560.96
DALYs (Dis	Global	Female	50-69 yea	Other sexu	Number	2015	26041.7	31363.6	21109
DALYs (Dis	Global	Male	50-69 yea	Other sexu	Rate	2015	0.93367	1.30261	0.58377
DALYs (Dis	Global	Female	50-69 yea	Other sexu	Rate	2015	4.12544	4.9685	3.34401
DALYs (Dis	Global	Male	50-69 yea	Sexually tr	Number	2018	62092	107341	37925.9
DALYs (Dis	Global	Female	50-69 yea	Sexually tr	Number	2018	168969	290122	106533
DALYs (Dis	Global	Male	50-69 yea	Sexually tr	Rate	2018	9.39955	16.2493	5.74126
DALYs (Dis	Global	Female	50-69 yea	Sexually tr	Rate	2018	24.6231	42.278	15.5246
DALYs (Dis	Global	Male	50-69 yea	Syphilis	Number	2018	19895.2	25904.3	12625.4
DALYs (Dis	Global	Female	50-69 yea	Syphilis	Number	2018	5605.33	6644.06	4435.26
DALYs (Dis	Global	Male	50-69 yea	Syphilis	Rate	2018	3.01175	3.92141	1.91124
DALYs (Dis	Global	Female	50-69 yea	Syphilis	Rate	2018	0.81684	0.96821	0.64633
DALYs (Dis	Global	Male	50-69 yea	Chlamydia	Number	2018	3132.39	5421.7	1625.87
DALYs (Dis	Global	Female	50-69 yea	Chlamydia	Number	2018	11129.2	13353.1	9043.42
DALYs (Dis	Global	Male	50-69 yea	Chlamydia	Rate	2018	0.47418	0.82074	0.24613
DALYs (Dis	Global	Female	50-69 yea	Chlamydia	Rate	2018	1.6218	1.94588	1.31785
DALYs (Dis	Global	Male	50-69 yea	Gonococca	Number	2018	3237.6	4940.66	2101.54
DALYs (Dis	Global	Female	50-69 yea	Gonococca	Number	2018	28982.9	34096.6	22581.8
DALYs (Dis	Global	Male	50-69 yea	Gonococca	Rate	2018	0.49011	0.74792	0.31813
DALYs (Dis	Global	Female	50-69 yea	Gonococca	Rate	2018	4.22354	4.96874	3.29074
DALYs (Dis	Global	Male	50-69 yea	Trichomon	Number	2018	2037.29	4529.92	735.544
DALYs (Dis	Global	Female	50-69 yea	Trichomon	Number	2018	51370.3	114550	18963
DALYs (Dis	Global	Male	50-69 yea	Trichomon	Rate	2018	0.30841	0.68574	0.11135
DALYs (Dis	Global	Female	50-69 yea	Trichomon	Rate	2018	7.48595	16.6928	2.76338
DALYs (Dis	Global	Male	50-69 yea	Genital he	Number	2018	27883	69532.8	8465.87
DALYs (Dis	Global	Female	50-69 yea	Genital he	Number	2018	44880.5	110442	13830.6
DALYs (Dis	Global	Male	50-69 yea	Genital he	Rate	2018	4.22096	10.5259	1.28157
DALYs (Dis	Global	Female	50-69 yea	Genital he	Rate	2018	6.54022	16.0942	2.01547
DALYs (Dis	Global	Male	50-69 yea	Other sexu	Number	2018	5906.54	8198.78	3797.18
DALYs (Dis	Global	Female	50-69 yea	Other sexu	Number	2018	27001.1	33160.8	21932.6
DALYs (Dis	Global	Male	50-69 yea	Other sexu	Rate	2018	0.89414	1.24114	0.57482
DALYs (Dis	Global	Female	50-69 yea	Other sexu	Rate	2018	3.93473	4.83237	3.19614
DALYs (Dis	Global	Male	50-69 yea	HIV/AIDS	Number	2017	3781978	4396738	3379609
DALYs (Dis	Global	Female	50-69 yea	HIV/AIDS	Number	2017	3091698	3532552	2780527
DALYs (Dis	Global	Male	50-69 yea	HIV/AIDS	Rate	2017	587.101	682.534	524.639
DALYs (Dis	Global	Female	50-69 yea	HIV/AIDS	Rate	2017	462.353	528.281	415.818
Prevalence	Global	Male	50-69 yea	HIV/AIDS	Number	1990	321712	378679	271933
Prevalence	Global	Female	50-69 yea	HIV/AIDS	Number	1990	274609	318549	232676
Prevalence	Global	Male	50-69 yea	HIV/AIDS	Rate	1990	95.595	112.522	80.8034
Prevalence	Global	Female	50-69 yea	HIV/AIDS	Rate	1990	79.458	92.1721	67.3249
Prevalence	Global	Male	50-69 yea	HIV/AIDS	Number	1991	390795	455883	331941
Prevalence	Global	Female	50-69 yea	HIV/AIDS	Number	1991	344963	397061	293132
Prevalence	Global	Male	50-69 yea	HIV/AIDS	Rate	1991	113.993	132.979	96.8256

Prevalenc	Global	Male	50-69 yea	HIV/AIDS	Rate	2000	263.815	303.221	224.755
Prevalenc	Global	Female	50-69 yea	HIV/AIDS	Rate	2000	245.156	280.528	213.677
Prevalenc	Global	Male	50-69 yea	HIV/AIDS	Number	2001	1138184	1321273	964218
Prevalenc	Global	Female	50-69 yea	HIV/AIDS	Number	2001	1065823	1232754	919780
Prevalenc	Global	Male	50-69 yea	HIV/AIDS	Rate	2001	277.197	321.787	234.829
Prevalenc	Global	Female	50-69 yea	HIV/AIDS	Rate	2001	254.295	294.123	219.45
Prevalenc	Global	Male	50-69 yea	HIV/AIDS	Number	2002	1218605	1427123	1023144
Prevalenc	Global	Female	50-69 yea	HIV/AIDS	Number	2002	1124945	1309802	959576
Prevalenc	Global	Male	50-69 yea	HIV/AIDS	Rate	2002	288.977	338.425	242.626
Prevalenc	Global	Female	50-69 yea	HIV/AIDS	Rate	2002	261.103	304.009	222.72
Prevalenc	Global	Male	50-69 yea	HIV/AIDS	Number	2003	1299191	1537260	1078500
Prevalenc	Global	Female	50-69 yea	HIV/AIDS	Number	2003	1179424	1385210	998704
Prevalenc	Global	Male	50-69 yea	HIV/AIDS	Rate	2003	299.642	354.55	248.742
Prevalenc	Global	Female	50-69 yea	HIV/AIDS	Rate	2003	266.073	312.498	225.303
Prevalenc	Global	Male	50-69 yea	HIV/AIDS	Number	2004	1382075	1652041	1139198
Prevalenc	Global	Female	50-69 yea	HIV/AIDS	Number	2004	1231590	1462046	1033200
Prevalenc	Global	Male	50-69 yea	HIV/AIDS	Rate	2004	309.692	370.186	255.269
Prevalenc	Global	Female	50-69 yea	HIV/AIDS	Rate	2004	269.771	320.251	226.315
Prevalenc	Global	Male	50-69 yea	HIV/AIDS	Number	2005	1469732	1773628	1204142
Prevalenc	Global	Female	50-69 yea	HIV/AIDS	Number	2005	1285719	1533978	1072691
Prevalenc	Global	Male	50-69 yea	HIV/AIDS	Rate	2005	319.806	385.933	262.015
Prevalenc	Global	Female	50-69 yea	HIV/AIDS	Rate	2005	273.353	326.134	228.061
Prevalenc	Global	Male	50-69 yea	HIV/AIDS	Number	2006	1567702	1901103	1282034
Prevalenc	Global	Female	50-69 yea	HIV/AIDS	Number	2006	1350414	1613907	1124431
Prevalenc	Global	Male	50-69 yea	HIV/AIDS	Rate	2006	331.572	402.087	271.153
Prevalenc	Global	Female	50-69 yea	HIV/AIDS	Rate	2006	279.032	333.477	232.338
Prevalenc	Global	Male	50-69 yea	HIV/AIDS	Number	2007	1679094	2033434	1380697
Prevalenc	Global	Female	50-69 yea	HIV/AIDS	Number	2007	1429069	1697311	1202870
Prevalenc	Global	Male	50-69 yea	HIV/AIDS	Rate	2007	345.588	418.517	284.172
Prevalenc	Global	Female	50-69 yea	HIV/AIDS	Rate	2007	287.323	341.255	241.844
Prevalenc	Global	Male	50-69 yea	HIV/AIDS	Number	2008	1804083	2173160	1494238
Prevalenc	Global	Female	50-69 yea	HIV/AIDS	Number	2008	1522270	1793013	1297763
Prevalenc	Global	Male	50-69 yea	HIV/AIDS	Rate	2008	361.802	435.819	299.664
Prevalenc	Global	Female	50-69 yea	HIV/AIDS	Rate	2008	298.109	351.13	254.144
Prevalenc	Global	Male	50-69 yea	HIV/AIDS	Number	2009	1942250	2324213	1618767
Prevalenc	Global	Female	50-69 yea	HIV/AIDS	Number	2009	1630450	1896491	1403818
Prevalenc	Global	Male	50-69 yea	HIV/AIDS	Rate	2009	379.697	454.369	316.458
Prevalenc	Global	Female	50-69 yea	HIV/AIDS	Rate	2009	310.93	361.664	267.71
Prevalenc	Global	Male	50-69 yea	HIV/AIDS	Number	2010	2093166	2491092	1757024
Prevalenc	Global	Female	50-69 yea	HIV/AIDS	Number	2010	1753838	2013678	1524124
Prevalenc	Global	Male	50-69 yea	HIV/AIDS	Rate	2010	398.643	474.428	334.625
Prevalenc	Global	Female	50-69 yea	HIV/AIDS	Rate	2010	325.456	373.674	282.828
Prevalenc	Global	Male	50-69 yea	HIV/AIDS	Number	2011	2254582	2669101	1900186
Prevalenc	Global	Female	50-69 yea	HIV/AIDS	Number	2011	1893488	2153610	1661094
Prevalenc	Global	Male	50-69 yea	HIV/AIDS	Rate	2011	417.879	494.709	352.193
Prevalenc	Global	Female	50-69 yea	HIV/AIDS	Rate	2011	341.427	388.331	299.523
Prevalenc	Global	Male	50-69 yea	HIV/AIDS	Number	2012	2424091	2859388	2059726
Prevalenc	Global	Female	50-69 yea	HIV/AIDS	Number	2012	2050520	2308707	1817219
Prevalenc	Global	Male	50-69 yea	HIV/AIDS	Rate	2012	436.478	514.857	370.871
Prevalenc	Global	Female	50-69 yea	HIV/AIDS	Rate	2012	358.613	403.767	317.812
Prevalenc	Global	Male	50-69 yea	HIV/AIDS	Number	2013	2602681	3056243	2224047
Prevalenc	Global	Female	50-69 yea	HIV/AIDS	Number	2013	2226671	2491781	1990556
Prevalenc	Global	Male	50-69 yea	HIV/AIDS	Rate	2013	454.443	533.637	388.331
Prevalenc	Global	Female	50-69 yea	HIV/AIDS	Rate	2013	376.916	421.792	336.948
Prevalenc	Global	Male	50-69 yea	HIV/AIDS	Number	2014	2791150	3248070	2394809
Prevalenc	Global	Female	50-69 yea	HIV/AIDS	Number	2014	2418197	2688418	2177071
Prevalenc	Global	Male	50-69 yea	HIV/AIDS	Rate	2014	472.182	549.479	405.132
Prevalenc	Global	Female	50-69 yea	HIV/AIDS	Rate	2014	395.947	440.192	356.466
Prevalenc	Global	Male	50-69 yea	HIV/AIDS	Number	2015	2986481	3455207	2577263
Prevalenc	Global	Female	50-69 yea	HIV/AIDS	Number	2015	2623805	2890453	2375125
Prevalenc	Global	Male	50-69 yea	HIV/AIDS	Rate	2015	489.588	566.429	422.503
Prevalenc	Global	Female	50-69 yea	HIV/AIDS	Rate	2015	415.653	457.895	376.258
Prevalenc	Global	Male	50-69 yea	HIV/AIDS	Number	2016	3184042	3670065	2760817
Prevalenc	Global	Female	50-69 yea	HIV/AIDS	Number	2016	2839247	3104644	2586669
Prevalenc	Global	Male	50-69 yea	HIV/AIDS	Rate	2016	507.112	584.519	439.706
Prevalenc	Global	Female	50-69 yea	HIV/AIDS	Rate	2016	436.253	477.032	397.445
Prevalenc	Global	Male	50-69 yea	HIV/AIDS	Number	2017	3379091	3863979	2951363
Prevalenc	Global	Female	50-69 yea	HIV/AIDS	Number	2017	3064901	3333044	2807324
Prevalenc	Global	Male	50-69 yea	HIV/AIDS	Rate	2017	524.558	599.83	458.159
Prevalenc	Global	Female	50-69 yea	HIV/AIDS	Rate	2017	458.345	498.445	419.825
Prevalenc	Global	Male	50-69 yea	HIV/AIDS	Number	2018	3577956	4070127	3144836
Prevalenc	Global	Female	50-69 yea	HIV/AIDS	Number	2018	3303378	3575085	3046116
Prevalenc	Global	Male	50-69 yea	HIV/AIDS	Rate	2018	541.634	616.14	476.068
Prevalenc	Global	Female	50-69 yea	HIV/AIDS	Rate	2018	481.385	520.98	443.896
Prevalenc	Global	Male	50-69 yea	HIV/AIDS	Number	2019	3783382	4266249	3340441
Prevalenc	Global	Female	50-69 yea	HIV/AIDS	Number	2019	3553751	3826787	3297577
Prevalenc	Global	Male	50-69 yea	HIV/AIDS	Rate	2019	559.573	630.99	494.061
Prevalenc	Global	Female	50-69 yea	HIV/AIDS	Rate	2019	505.635	544.484	469.186
Prevalenc	Global	Male	50-69 yea	Sexually tr	Number	1990	6.3E+07	7.3E+07	5.4E+07
Prevalenc	Global	Female	50-69 yea	Sexually tr	Number	1990	1E+08	1.2E+08	9.2E+07
Prevalenc	Global	Male	50-69 yea	Sexually tr	Rate	1990	18727.4	21755.9	16148.9
Prevalenc	Global	Female	50-69 yea	Sexually tr	Rate	1990	30224.1	34137.7	26647.2
Prevalenc	Global	Male	50-69 yea	Sexually tr	Number	1991	6.5E+07	7.5E+07	5.6E+07
Prevalenc	Global	Female	50-69 yea	Sexually tr	Number	1991	1.1E+08	1.2E+08	9.4E+07
Prevalenc	Global	Male	50-69 yea	Sexually tr	Rate	1991	18888.4	21879.8	16321.1
Prevalenc	Global	Female	50-69 yea	Sexually tr	Rate	1991	30473.9	34373.4	26899.7
Prevalenc	Global	Male	50-69 yea	Sexually tr	Number	1992	6.6E+07	7.7E+07	5.7E+07
Prevalenc	Global	Female	50-69 yea	Sexually tr	Number	1992	1.1E+08	1.2E+08	9.6E+07
Prevalenc	Global	Male	50-69 yea	Sexually tr	Rate	1992	19002.4	21993.4	16447
Prevalenc	Global	Female	50-69 yea	Sexually tr	Rate	1992	30685.5	34574.3	27064.7
Prevalenc	Global	Male	50-69 yea	Sexually tr	Number	1993	6.7E+07	7.8E+07	5.8E+07
Prevalenc	Global	Female	50-69 yea	Sexually tr	Number	1993	1.1E+08	1.3E+08	9.8E+07
Prevalenc	Global	Male	50-69 yea	Sexually tr	Rate	1993	19078.5	22065.7	16534
Prevalenc	Global	Female	50-69 yea	Sexually tr	Rate	1993	30846.3	34701.7	27244.3
Prevalenc	Global	Male	50-69 yea	Sexually tr	Number	1994	6.9E+07	7.9E+07	5.9E+07
Prevalenc	Global	Female	50-69 yea	Sexually tr	Number	1994	1.1E+08	1.3E+08	1E+08
Prevalenc	Global	Male	50-69 yea	Sexually tr	Rate	1994	19123.2	22114.2	16582.9
Prevalenc	Global	Female	50-69 yea	Sexually tr	Rate	1994	30958.6	34818.9	27333.9
Prevalenc	Global	Male	50-69 yea	Sexually tr	Number	1995	7E+07	8E+07	6E+07
Prevalenc	Global	Female	50-69 yea	Sexually tr	Number	1995	1.1E+08	1.3E+08	1E+08
Prevalenc	Global	Male	50-69 yea	Sexually tr	Rate	1995	19149.7	22131.8	16607.4
Prevalenc	Global	Female	50-69 yea	Sexually tr	Rate	1995	31022.1	34868.7	27402.9
Prevalenc	Global	Male	50-69 yea	Sexually tr	Number	1996	7E+07	8.1E+07	6.1E+07
Prevalenc	Global	Female	50-69 yea	Sexually tr	Number	1996	1.2E+08	1.3E+08	1E+08
Prevalenc	Global	Male	50-69 yea	Sexually tr	Rate	1996	19104.5	22088.8	16579.7
Prevalenc	Global	Female	50-69 yea	Sexually tr	Rate	1996	31004.9	34854.6	27399.6
Prevalenc	Global	Male	50-69 yea	Sexually tr	Number	1997	7.1E+07	8.2E+07	6.2E+07
Prevalenc	Global	Female	50-69 yea	Sexually tr	Number	1997	1.2E+08	1.3E+08	1E+08
Prevalenc	Global	Male	50-69 yea	Sexually tr	Rate	1997	18970.2	21919.4	16465.5
Prevalenc	Global	Female	50-69 yea	Sexually tr	Rate	1997	30895.3	34695.4	27335
Prevalenc	Global	Male	50-69 yea	Sexually tr	Number	1998	7.2E+07	8.3E+07	6.3E+07
Prevalenc	Global	Female	50-69 yea	Sexually tr	Number	1998	1.2E+08	1.4E+08	1.1E+08
Prevalenc	Global	Male	50-69 yea	Sexually tr	Rate	1998	18808.6	21740.2	16337
Prevalenc	Global	Female	50-69 yea	Sexually tr	Rate	1998	30757.6	34552	27210.1
Prevalenc	Global	Male	50-69 yea	Sexually tr	Number	1999	7.3E+07	8.5E+07	6.4E+07
Prevalenc	Global	Female	50-69 yea	Sexually tr	Number	1999	1.2E+08	1.4E+08	1.1E+08
Prevalenc	Global	Male	50-69 yea	Sexually tr	Rate	1999	18671.9	21602.6	16217.3
Prevalenc	Global	Female	50-69 yea	Sexually tr	Rate	1999	30653.7	34478.8	27118.3
Prevalenc	Global	Male	50-69 yea	Sexually tr	Number	2000	7.5E+07	8.6E+07	6.5E+07
Prevalenc	Global	Female	50-69 yea	Sexually tr	Number	2000	1.3E+08	1.4E+08	1.1E+08
Prevalenc	Global	Male	50-69 yea	Sexually tr	Rate	2000	18623.8	21548	16179.3
Prevalenc	Global	Female	50-69 yea	Sexually tr	Rate	2000	30636.3	34480.8	27116.2
Prevalenc	Global	Male	50-69 yea	Sexually tr	Number	2001	7.7E+07	8.9E+07	6.7E+07
Prevalenc	Global	Female	50-69 yea	Sexually tr	Number	2001	1.3E+08	1.4E+08	1.1E+08
Prevalenc	Global	Male	50-69 yea	Sexually tr	Rate	2001	18649.9	21586.5	16202.6
Prevalenc	Global	Female	50-69 yea	Sexually tr	Rate	2001	30695.3	34573.7	27149.8
Prevalenc	Global	Male	50-69 yea	Sexually tr	Number	2002	7.9E+07	9.1E+07	6.8E+07
Prevalenc	Global	Female	50-69 yea	Sexually tr	Number	2002	1.3E+08	1.5E+08	1.2E+08
Prevalenc	Global	Male	50-69 yea	Sexually tr	Rate	2002	18667.3	21578.4	16200.7
Prevalenc	Global	Female	50-69 yea	Sexually tr	Rate	2002	30753.2	34644.2	27163.4
Prevalenc	Global	Male	50-69 yea	Sexually tr	Number	2003	8.1E+07	9.4E+07	7E+07
Prevalenc	Global	Female	50-69 yea	Sexually tr	Number	2003	1.4E+08	1.5E+08	1.2E+08
Prevalenc	Global	Male	50-69 yea	Sexually tr	Rate	2003	18684.3	21614.1	16203
Prevalenc	Global	Female	50-69 yea	Sexually tr	Rate	2003	30814.7	34725.7	27220.9
Prevalenc	Global	Male	50-69 yea	Sexually tr	Number	2004	8.3E+07	9.7E+07	7.2E+07
Prevalenc	Global	Female	50-69 yea	Sexually tr	Number	2004	1.4E+08	1.6E+08	1.2E+08
Prevalenc	Global	Male	50-69 yea	Sexually tr	Rate	2004	18697.2	21626.2	16196.1
Prevalenc	Global	Female	50-69 yea	Sexually tr	Rate	2004	30871.7	34785	27257.1
Prevalenc	Global	Male	50-69 yea	Sexually tr	Number	2005	8.6E+07	9.9E+07	7.5E+07
Prevalenc	Global	Female	50-69 yea	Sexually tr	Number	2005	1.5E+08	1.6E+08	1.3E+08
Prevalenc	Global	Male	50-69 yea	Sexually tr	Rate	2005	18720.2	21637	16215.7
Prevalenc	Global	Female	50-69 yea	Sexually tr	Rate	2005	30926.8	34864.4	27316.5
Prevalenc	Global	Male	50-69 yea	Sexually tr	Number	2006	8.8E+07	1E+08	7.6E+07
Prevalenc	Global	Female	50-69 yea	Sexually tr	Number	2006	1.5E+08	1.7E+08	1.3E+08
Prevalenc	Global	Male	50-69 yea	Sexually tr	Rate	2006	18675.1	21578	16148.3
Prevalenc	Global	Female	50-69 yea	Sexually tr	Rate	2006	30891.7	34806.9	27231.3
Prevalenc	Global	Male	50-69 yea	Sexually tr	Number	2007	9E+07	1E+08	7.8E+07
Prevalenc	Global	Female	50-69 yea	Sexually tr	Number	2007	1.5E+08	1.7E+08	1.3E+08
Prevalenc	Global	Male	50-69 yea	Sexually tr	Rate	2007	18540.5	21443.1	16038.4
Prevalenc	Global	Female	50-69 yea	Sexually tr	Rate	2007	30746.6	34711.5	27072.3
Prevalenc	Global	Male	50-69 yea	Sexually tr	Number	2008	9.2E+07	1.1E+08	7.9E+07
Prevalenc	Global	Female	50-69 yea	Sexually tr	Number	2008	1.6E+08	1.8E+08	1.4E+08
Prevalenc	Global	Male	50-69 yea	Sexually tr	Rate	2008	18380.1	21255.5	15900.9
Prevalenc	Global	Female	50-69 yea	Sexually tr	Rate	2008	30560.2	34556.2	26850.2
Prevalenc	Global	Male	50-69 yea	Sexually tr	Number	2009	9.3E+07	1.1E+08	8.1E+07
Prevalenc	Global	Female	50-69 yea	Sexually tr	Number	2009	1.6E+08	1.8E+08	1.4E+08
Prevalenc	Global	Male	50-69 yea	Sexually tr	Rate	2009	18251.6	21123	15793.3
Prevalenc	Global	Female	50-69 yea	Sexually tr	Rate	2009	30395.2	34442.2	26666.8
Prevalenc	Global	Male	50-69 yea	Sexually tr	Number	2010	9.6E+07	1.1E+08	8.3E+07
Prevalenc	Global	Female	50-69 yea	Sexually tr	Number	2010	1.6E+08	1.9E+08	1.4E+08
Prevalenc	Global	Male	50-69 yea	Sexually tr	Rate	2010	18222.6	21109.4	15780.7
Prevalenc	Global	Female	50-69 yea	Sexually tr	Rate	2010	30334.2	34400	26550
Prevalenc	Global	Male	50-69 yea	Sexually tr	Number	2011	9.9E+07	1.1E+08	8.5E+07
Prevalenc	Global	Female	50-69 yea	Sexually tr	Number	2011	1.7E+08	1.9E+08	1.5E+08
Prevalenc	Global	Male	50-69 yea	Sexually tr	Rate	2011	18306.8	21184.1	15824.2
Prevalenc	Global	Female	50-69 yea	Sexually tr	Rate	2011	30361.9	34452.9	26581.4
Prevalenc	Global	Male	50-69 yea	Sexually tr	Number	2012	1E+08	1.2E+08	8.9E+07
Prevalenc	Global	Female	50-69 yea	Sexually tr	Number	2012	1.7E+08	2E+08	1.5E+08
Prevalenc	Global	Male	50-69 yea	Sexually tr	Rate	2012	18458.9	21342.3	15941.1
Prevalenc	Global	Female	50-69 yea	Sexually tr	Rate	2012	30417.6	34500.6	26644
Prevalenc	Global	Male	50-69 yea	Sexually tr	Number	2013	1.1E+08	1.2E+08	9.2E+07
Prevalenc	Global	Female	50-69 yea	Sexually tr	Number	2013	1.8E+08	2E+08	1.6E+08
Prevalenc	Global	Male	50-69 yea	Sexually tr	Rate	2013	18630.4	21549.8	16082.5
Prevalenc	Global	Female	50-69 yea	Sexually tr	Rate	2013	30484.6	34586.6	26697.2
Prevalenc	Global	Male	50-69 yea	Sexually tr	Number	2014	1.1E+08	1.3E+08	9.6E+07
Prevalenc	Global	Female	50-69 yea	Sexually tr	Number	2014	1.9E+08	2.1E+08	1.6E+08
Prevalenc	Global	Male	50-69 yea	Sexually tr	Rate	2014	18773.6	21738.2	16161.7
Prevalenc	Global	Female	50-69 yea	Sexually tr	Rate	2014	30546	34677.2	26718.3
Prevalenc	Global	Male	50-69 yea	Sexually tr	Number	2015	1.1E+08	1.3E+08	9.9E+07
Prevalenc	Global	Female	50-69 yea	Sexually tr	Number	2015	1.9E+08	2.2E+08	1.7E+08
Prevalenc	Global	Male	50-69 yea	Sexually tr	Rate	2015	18839.1	21820.1	16223.5
Prevalenc	Global	Female	50-69 yea	Sexually tr	Rate	2015	30580.3	34746.5	26751.9
Prevalenc	Global	Male	50-69 yea	Sexually tr	Number	2016	1.2E+08	1.4E+08	1E+08
Prevalenc	Global	Female	50-69 yea	Sexually tr	Number	2016	2E+08	2.3E+08	1.7E+08
Prevalenc	Global	Male	50-69 yea	Sexually tr	Rate	2016	18869.8	21861.6	16250.6
Prevalenc	Global	Female	50-69 yea	Sexually tr	Rate	2016	30613.6	34776.9	26764.5
Prevalenc	Global	Male	50-69 yea	Sexually tr	Number	2017	1.2E+08	1.4E+08	1E+08
Prevalenc	Global	Female	50-69 yea	Sexually tr	Number	2017	2E+08	2.3E+08	1.8E+08
Prevalenc	Global	Male	50-69 yea	Sexually tr	Rate	2017	18907.9	21971.1	16260.3
Prevalenc	Global	Female	50-69 yea	Sexually tr	Rate	2017	30655.4	34898.4	26762.8
Prevalenc	Global	Male	50-69 yea	Sexually tr	Number	2018	1.3E+08	1.5E+08	1.1E+08
Prevalenc	Global	Female	50-69 yea	Sexually tr	Number	2018	2.1E+08	2.4E+08	1.8E+08
Prevalenc	Global	Male	50-69 yea	Sexually tr	Rate	2018	18925.6	21969.8	16251.6
Prevalenc	Global	Female	50-69 yea	Sexually tr	Rate	2018	30694.5	34919.2	26804.3
Prevalenc	Global	Male	50-69 yea	Sexually tr	Number	2019	1.3E+08	1.5E+08	1.1E+08
Prevalenc	Global	Female	50-69 yea	Sexually tr	Number	2019	2.2E+08	2.5E+08	1.9E+08
Prevalenc	Global	Male	50-69 yea	Sexually tr	Rate	2019	18958.9	22012.8	16291.9
Prevalenc	Global	Female	50-69 yea	Sexually tr	Rate	2019	30763.6	34986.7	26872.2
Prevalenc	Global	Male	50-69 yea	Genital he	Number	1990	5.8E+07	6.9E+07	4.9E+07
Prevalenc	Global	Female	50-69 yea	Genital he	Number	1990	9.3E+07	1.1E+08	8.1E+07
Prevalenc	Global	Male	50-69 yea	Genital he	Rate	1990	17332.5	20551.4	14650.7
Prevalenc	Global	Female	50-69 yea	Genital he	Rate	1990	27047	31189.4	23431.8
Prevalenc	Global	Male	50-69 yea	Genital he	Number	1991	6E+07	7.1E+07	5.1E+07
Prevalenc	Global	Female	50-69 yea	Genital he	Number	1991	9.6E+07	1.1E+08	8.3E+07
Prevalenc	Global	Male	50-69 yea	Genital he	Rate	1991	17510.9	20694.5	14834.8
Prevalenc	Global	Female	50-69 yea	Genital he	Rate	1991	27302.6	31458.1	23696.7
Prevalenc	Global	Male	50-69 yea	Genital he	Number	1992	6.1E+07	7.2E+07	5.2E+07
Prevalenc	Global	Female	50-69 yea	Genital he	Number	1992	9.8E+07	1.1E+08	8.5E+07
Prevalenc	Global	Male	50-69 yea	Genital he	Rate	1992	17639	20746.1	14994.2
Prevalenc	Global	Female	50-69 yea	Genital he	Rate	1992	27521.7	31690.1	23899.5
Prevalenc	Global	Male	50-69 yea	Genital he	Number	1993	6.3E+07	7.4E+07	5.3E+07
Prevalenc	Global	Female	50-69 yea	Genital he	Number	1993	1E+08	1.2E+08	8.7E+07
Prevalenc	Global	Male	50-69 yea	Genital he	Rate	1993	17728.8	20784.7	15114.9
Prevalenc	Global	Female	50-69 yea	Genital he	Rate	1993	27693	31869	24040.5
Prevalenc	Global	Male	50-69 yea	Genital he	Number	1994	6.4E+07	7.5E+07	5.4E+07
Prevalenc	Global	Female	50-69 yea	Genital he	Number	1994	1E+08	1.2E+08	8.8E+07
Prevalenc	Global	Male	50-69 yea	Genital he	Rate	1994	17784.8	20831.4	15172.9
Prevalenc	Global	Female	50-69 yea	Genital he	Rate	1994	27814.2	32021.4	24156.8
Prevalenc	Global	Male	50-69 yea	Genital he	Number	1995	6.5E+07	7.6E+07	5.5E+07
Prevalenc	Global	Female	50-69 yea	Genital he	Number	1995	1E+08	1.2E+08	9E+07
Prevalenc	Global	Male	50-69 yea	Genital he	Rate	1995	17817.4	20878.3	15192.4
Prevalenc	Global	Female	50-69 yea	Genital he	Rate	1995	27882.7	32069	24209.3
Prevalenc	Global	Male	50-69 yea	Genital he	Number	1996	6.5E+07	7.7E+07	5.6E+07
Prevalenc	Global	Female	50-69 yea	Genital he	Number	1996	1E+08	1.2E+08	9.1E+07
Prevalenc	Global	Male	50-69 yea	Genital he	Rate	1996	17770.8	20808	15188.1
Prevalenc	Global	Female	50-69 yea	Genital he	Rate	1996	27865.6	32075.1	24191.3
Prevalenc	Global	Male	50-69 yea	Genital he	Number	1997	6.6E+07	7.8E+07	5.7E+07
Prevalenc	Global	Female	50-69 yea	Genital he	Number	1997	1.1E+08	1.2E+08	9.2E+07
Prevalenc	Global	Male	50-69 yea	Genital he	Rate	1997	17624.2	20659.8	15061.2
Prevalenc	Global	Female	50-69 yea	Genital he	Rate	1997	27753.7	31959.3	24089.7
Prevalenc	Global	Male	50-69 yea	Genital he	Number	1998	6.7E+07	7.9E+07	5.7E+07
Prevalenc	Global	Female	50-69 yea	Genital he	Number	1998	1.1E+08	1.2E+08	9.4E+07
Prevalenc	Global	Male	50-69 yea	Genital he	Rate	1998	17447.9	20491.9	14900.9
Prevalenc	Global	Female	50-69 yea	Genital he	Rate	1998	27611	31770	23973
Prevalenc	Global	Male	50-69 yea	Genital he	Number	1999	6.8E+07	8E+07	5.8E+07
Prevalenc	Global	Female	50-69 yea	Genital he	Number	1999	1.1E+08	1.3E+08	9.5E+07
Prevalenc	Global	Male	50-69 yea	Genital he	Rate	1999	17290	20322.1	14751
Prevalenc	Global	Female	50-69 yea	Genital he	Rate	1999	27494.9	31660.5	23883.5
Prevalenc	Global	Male	50-69 yea	Genital he	Number	2000	6.9E+07	8.1E+07	5.9E+07
Prevalenc	Global	Female	50-69 yea	Genital he	Number	2000	1.1E+08	1.3E+08	9.7E+07
Prevalenc	Global	Male	50-69 yea	Genital he	Rate	2000	17232.7	20264.2	14686
Prevalenc	Global	Female	50-69 yea	Genital he	Rate	2000	27469.6	31608.7	23870.2
Prevalenc	Global	Male	50-69 yea	Genital he	Number	2001	7.1E+07	8.3E+07	6E+07
Prevalenc	Global	Female	50-69 yea	Genital he	Number	2001	1.2E+08	1.3E+08	1E+08
Prevalenc	Global	Male	50-69 yea	Genital he	Rate	2001	17246.2	20274.2	14684.6
Prevalenc	Global	Female	50-69 yea	Genital he	Rate	2001	27513	31665.2	23907.8
Prevalenc	Global	Male	50-69 yea	Genital he	Number	2002	7.3E+07	8.6E+07	6.2E+07
Prevalenc	Global	Female	50-69 yea	Genital he	Number	2002	1.2E+08	1.4E+08	1E+08
Prevalenc	Global	Male	50-69 yea	Genital he	Rate	2002	17258.4	20285.6	14684.9
Prevalenc	Global	Female	50-69 yea	Genital he	Rate	2002	27562.1	31742.5	23928.3
Prevalenc	Global	Male	50-69 yea	Genital he	Number	2003	7.5E+07	8.8E+07	6.4E+07
Prevalenc	Global	Female	50-69 yea	Genital he	Number	2003	1.2E+08	1.4E+08	1.1E+08
Prevalenc	Global	Male	50-69 yea	Genital he	Rate	2003	17268.5	20301.1	14684
Prevalenc	Global	Female	50-69 yea	Genital he	Rate	2003	27611.9	31836.4	23942.6
Prevalenc	Global	Male	50-69 yea	Genital he	Number	2004	7.7E+07	9.1E+07	6.6E+07
Prevalenc	Global	Female	50-69 yea	Genital he	Number	2004	1.3E+08	1.5E+08	1.1E+08
Prevalenc	Global	Male	50-69 yea	Genital he	Rate	2004	17279.2	20303.2	14683.1
Prevalenc	Global	Female	50-69 yea	Genital he	Rate	2004	27659	31927.9	23969.5
Prevalenc	Global	Male	50-69 yea	Genital he	Number	2005	7.9E+07	9.3E+07	6.8E+07
Prevalenc	Global	Female	50-69 yea	Genital he	Number	2005	1.3E+08	1.5E+08	1.1E+08
Prevalenc	Global	Male	50-69 yea	Genital he	Rate	2005	17292.8	20298.1	14688
Prevalenc	Global	Female	50-69 yea	Genital he	Rate	2005	27699.4	32002.6	24012.5
Prevalenc	Global	Male	50-69 yea	Genital he	Number	2006	8.2E+07	9.6E+07	6.9E+07
Prevalenc	Global	Female	50-69 yea	Genital he	Number	2006	1.3E+08	1.5E+08	1.2E+08
Prevalenc	Global	Male	50-69 yea	Genital he	Rate	2006	17247.2	20285.4	14650.5
Prevalenc	Global	Female	50-69 yea	Genital he	Rate	2006	27666.9	32002.8	23971
Prevalenc	Global	Male	50-69 yea	Genital he	Number	2007	8.3E+07	9.8E+07	7.1E+07
Prevalenc	Global	Female	50-69 yea	Genital he	Number	2007	1.4E+08	1.6E+08	1.2E+08
Prevalenc	Global	Male	50-69 yea	Genital he	Rate	2007	17117.3	20177.3	14529.9
Prevalenc	Global	Female	50-69 yea	Genital he	Rate	2007	27539.4	31880.1	23817.3
Prevalenc	Global	Male	50-69 yea	Genital he	Number	2008	8.5E+07	1E+08	7.2E+07
Prevalenc	Global	Female	50-69 yea	Genital he	Number	2008	1.4E+08	1.6E+08	1.2E+08
Prevalenc	Global	Male	50-69 yea	Genital he	Rate	2008	16966.2	20024.9	14417.6
Prevalenc	Global	Female	50-69 yea	Genital he	Rate	2008	27377.9	31638	23658.3
Prevalenc	Global	Male	50-69 yea	Genital he	Number	2009	8.6E+07	1E+08	7.3E+07
Prevalenc	Global	Female	50-69 yea	Genital he	Number	2009	1.4E+08	1.7E+08	1.2E+08
Prevalenc	Global	Male	50-69 yea	Genital he	Rate	2009	16851.3	19915.2	14330.6
Prevalenc	Global	Female	50-69 yea	Genital he	Rate	2009	27240	31527.2	23463.1
Prevalenc	Global	Male	50-69 yea	Genital he	Number	2010	8.8E+07	1E+08	7.5E+07
Prevalenc	Global	Female	50-69 yea	Genital he	Number	2010	1.5E+08	1.7E+08	1.3E+08
Prevalenc	Global	Male	50-69 yea	Genital he	Rate	2010	16830.6	19917	14321
Prevalenc	Global	Female	50-69 yea	Genital he	Rate	2010	27193.4	31521.3	23323.6
Prevalenc	Global	Male	50-69 yea	Genital he	Number	2011	9.1E+07	1.1E+08	7.8E+07
Prevalenc	Global	Female	50-69 yea	Genital he	Number	2011	1.5E+08	1.7E+08	1.3E+08
Prevalenc	Global	Male	50-69 yea	Genital he	Rate	2011	16924	20007.4	14399.2
Prevalenc	Global	Female	50-69 yea	Genital he	Rate	2011	27226.2	31514.8	23427.3
Prevalenc	Global	Male	50-69 yea	Genital he	Number	2012	9.5E+07	1.1E+08	8.1E+07
Prevalenc	Global	Female	50-69 yea	Genital he	Number	2012	1.6E+08	1.8E+08	1.3E+08
Prevalenc	Global	Male	50-69 yea	Genital he	Rate	2012	17083.9	20158.5	14525.1
Prevalenc	Global	Female	50-69 yea	Genital he	Rate	2012	27279.3	31538.4	23494.3
Prevalenc	Global	Male	50-69 yea	Genital he	Number	2013	9.9E+07	1.2E+08	8.4E+07
Prevalenc	Global	Female	50-69 yea	Genital he	Number	2013	1.6E+08	1.9E+08	1.4E+08
Prevalenc	Global	Male	50-69 yea	Genital he	Rate	2013	17259.4	20305.4	14658.6
Prevalenc	Global	Female	50-69 yea	Genital he	Rate	2013	27340	31663.2	23509.6
Prevalenc	Global	Male	50-69 yea	Genital he	Number	2014	1E+08	1.2E+08	8.7E+07
Prevalenc	Global	Female	50-69 yea	Genital he	Number	2014	1.7E+08	1.9E+08	1.4E+08
Prevalenc	Global	Male	50-69 yea	Genital he	Rate	2014	17404.7	20459.2	14757
Prevalenc	Global	Female	50-69 yea	Genital he	Rate	2014	27394	31815.1	23546.1
Prevalenc	Global	Male	50-69 yea	Genital he	Number	2015	1.1E+08	1.3E+08	9E+07
Prevalenc	Global	Female	50-69 yea	Genital he	Number	2015	1.7E+08	2E+08	1.5E+08
Prevalenc	Global	Male	50-69 yea	Genital he	Rate	2015	17469.5	20525.5	14810.6
Prevalenc	Global	Female	50-69 yea	Genital he	Rate	2015	27424.8	31856.8	23551.8
Prevalenc	Global	Male	50-69 yea	Genital he	Number	2016	1.1E+08	1.3E+08	9.3E+07
Prevalenc	Global	Female	50-69 yea	Genital he	Number	2016	1.8E+08	2.1E+08	1.5E+08
Prevalenc	Global	Male	50-69 yea	Genital he	Rate	2016	17494.4	20631.8	14822.6
Prevalenc	Global	Female	50-69 yea	Genital he	Rate	2016	27462.4	31967.5	23541.7
Prevalenc	Global	Male	50-69 yea	Genital he	Number	2017	1.1E+08	1.3E+08	9.6E+07
Prevalenc	Global	Female	50-69 yea	Genital he	Number	2017	1.8E+08	2.1E+08	1.6E+08
Prevalenc	Global	Male	50-69 yea	Genital he	Rate	2017	17524.7	20796.7	14833.7
Prevalenc	Global	Female	50-69 yea	Genital he	Rate	2017	27508.1	32135.2	23508
Prevalenc	Global	Male	50-69 yea	Genital he	Number	2018	1.2E+08	1.4E+08	9.8E+07
Prevalenc	Global	Female	50-69 yea	Genital he	Number	2018	1.9E+08	2.2E+08	1.6E+08
Prevalenc	Global	Male	50-69 yea	Genital he	Rate	2018	17538.3	20775.6	14835.8
Prevalenc	Global	Female	50-69 yea	Genital he	Rate	2018	27534.2	32068	23551
Prevalenc	Global	Male	50-69 yea	Genital he	Number	2019	1.2E+08	1.4E+08	1E+08
Prevalenc	Global	Female	50-69 yea	Genital he	Number	2019	1.9E+08	2.2E+08	1.7E+08
Prevalenc	Global	Male	50-69 yea	Genital he	Rate	2019	17566	20757.3	14856.2
Prevalenc	Global	Female	50-69 yea	Genital he	Rate	2019	27578.3	32004.5	23623
Prevalenc	Global	Male	50-69 yea	Syphilis	Number	1990	1755890	2614219	1162519
Prevalenc	Global	Female	50-69 yea	Syphilis	Number	1990	821435	1227140	557484
Prevalenc	Global	Male	50-69 yea	Syphilis	Rate	1990	521.754	776.802	345.437
Prevalenc	Global	Female	50-69 yea	Syphilis	Rate	1990	237.682	355.073	161.308
Prevalenc	Global	Male	50-69 yea	Syphilis	Number	1991	1772815	2640658	1180730
Prevalenc	Global	Female	50-69 yea	Syphilis	Number	1991	837455	1252965	569069
Prevalenc	Global	Male	50-69 yea	Syphilis	Rate	1991	517.121	770.266	344.413
Prevalenc	Global	Female	50-69 yea	Syphilis	Rate	1991	238.398	356.682	161.997
Prevalenc	Global	Male	50-69 yea	Syphilis	Number	1992	1787778	2665954	1193532
Prevalenc	Global	Female	50-69 yea	Syphilis	Number	1992	852528	1278005	581034
Prevalenc	Global	Male	50-69 yea	Syphilis	Rate	1992	513.062	765.083	342.523
Prevalenc	Global	Female	50-69 yea	Syphilis	Rate	1992	239.15	358.504	162.991
Prevalenc	Global	Male	50-69 yea	Syphilis	Number	1993	1801525	2690850	1203323
Prevalenc	Global	Female	50-69 yea	Syphilis	Number	1993	866275	1298929	591690
Prevalenc	Global	Male	50-69 yea	Syphilis	Rate	1993	509.395	760.858	340.249
Prevalenc	Global	Female	50-69 yea	Syphilis	Rate	1993	239.896	359.71	163.856
Prevalenc	Global	Male	50-69 yea	Syphilis	Number	1994	1816678	2718930	1216344
Prevalenc	Global	Female	50-69 yea	Syphilis	Number	1994	880071	1317168	602137
Prevalenc	Global	Male	50-69 yea	Syphilis	Rate	1994	506.768	758.455	339.303
Prevalenc	Global	Female	50-69 yea	Syphilis	Rate	1994	240.733	360.296	164.707
Prevalenc	Global	Male	50-69 yea	Syphilis	Number	1995	1832880	2750079	1228512
Prevalenc	Global	Female	50-69 yea	Syphilis	Number	1995	893379	1334352	613826
Prevalenc	Global	Male	50-69 yea	Syphilis	Rate	1995	504.939	757.617	338.442
Prevalenc	Global	Female	50-69 yea	Syphilis	Rate	1995	241.485	360.682	165.92
Prevalenc	Global	Male	50-69 yea	Syphilis	Number	1996	1856495	2787236	1244512
Prevalenc	Global	Female	50-69 yea	Syphilis	Number	1996	907780	1357660	623390
Prevalenc	Global	Male	50-69 yea	Syphilis	Rate	1996	503.999	756.676	337.859
Prevalenc	Global	Female	50-69 yea	Syphilis	Rate	1996	241.824	361.668	166.065
Prevalenc	Global	Male	50-69 yea	Syphilis	Number	1997	1890824	2840391	1261903
Prevalenc	Global	Female	50-69 yea	Syphilis	Number	1997	923196	1380358	633899
Prevalenc	Global	Male	50-69 yea	Syphilis	Rate	1997	503.078	755.722	335.745
Prevalenc	Global	Female	50-69 yea	Syphilis	Rate	1997	241.138	360.549	165.574
Prevalenc	Global	Male	50-69 yea	Syphilis	Number	1998	1925641	2895143	1281769
Prevalenc	Global	Female	50-69 yea	Syphilis	Number	1998	938430	1404926	643836
Prevalenc	Global	Male	50-69 yea	Syphilis	Rate	1998	502.233	755.093	334.303
Prevalenc	Global	Female	50-69 yea	Syphilis	Rate	1998	240.159	359.543	164.768
Prevalenc	Global	Male	50-69 yea	Syphilis	Number	1999	1966973	2959978	1308392
Prevalenc	Global	Female	50-69 yea	Syphilis	Number	1999	956157	1435448	655028
Prevalenc	Global	Male	50-69 yea	Syphilis	Rate	1999	501.259	754.314	333.428
Prevalenc	Global	Female	50-69 yea	Syphilis	Rate	1999	239.218	359.13	163.879
Prevalenc	Global	Male	50-69 yea	Syphilis	Number	2000	1999985	3013263	1328447
Prevalenc	Global	Female	50-69 yea	Syphilis	Number	2000	971300	1461195	664941
Prevalenc	Global	Male	50-69 yea	Syphilis	Rate	2000	499.825	753.058	331.998
Prevalenc	Global	Female	50-69 yea	Syphilis	Rate	2000	238.013	358.06	162.941
Prevalenc	Global	Male	50-69 yea	Syphilis	Number	2001	2051289	3087392	1359563
Prevalenc	Global	Female	50-69 yea	Syphilis	Number	2001	999484	1503532	683359
Prevalenc	Global	Male	50-69 yea	Syphilis	Rate	2001	499.578	751.914	331.112
Prevalenc	Global	Female	50-69 yea	Syphilis	Rate	2001	238.467	358.727	163.043
Prevalenc	Global	Male	50-69 yea	Syphilis	Number	2002	2096242	3154384	1386430
Prevalenc	Global	Female	50-69 yea	Syphilis	Number	2002	1025908	1541755	699845
Prevalenc	Global	Male	50-69 yea	Syphilis	Rate	2002	497.098	748.023	328.775
Prevalenc	Global	Female	50-69 yea	Syphilis	Rate	2002	238.116	357.845	162.436
Prevalenc	Global	Male	50-69 yea	Syphilis	Number	2003	2141554	3222026	1416670
Prevalenc	Global	Female	50-69 yea	Syphilis	Number	2003	1052781	1585186	716289
Prevalenc	Global	Male	50-69 yea	Syphilis	Rate	2003	493.923	743.12	326.737
Prevalenc	Global	Female	50-69 yea	Syphilis	Rate	2003	237.503	357.611	161.592
Prevalenc	Global	Male	50-69 yea	Syphilis	Number	2004	2191140	3298391	1446642
Prevalenc	Global	Female	50-69 yea	Syphilis	Number	2004	1080976	1624980	734019
Prevalenc	Global	Male	50-69 yea	Syphilis	Rate	2004	490.986	739.096	324.16
Prevalenc	Global	Female	50-69 yea	Syphilis	Rate	2004	236.78	355.94	160.782
Prevalenc	Global	Male	50-69 yea	Syphilis	Number	2005	2251947	3389912	1482903
Prevalenc	Global	Female	50-69 yea	Syphilis	Number	2005	1112279	1674449	754253
Prevalenc	Global	Male	50-69 yea	Syphilis	Rate	2005	490.013	737.628	322.672
Prevalenc	Global	Female	50-69 yea	Syphilis	Rate	2005	236.478	355.999	160.359
Prevalenc	Global	Male	50-69 yea	Syphilis	Number	2006	2311342	3478746	1519935
Prevalenc	Global	Female	50-69 yea	Syphilis	Number	2006	1135853	1700264	771579
Prevalenc	Global	Male	50-69 yea	Syphilis	Rate	2006	488.854	735.762	321.47
Prevalenc	Global	Female	50-69 yea	Syphilis	Rate	2006	234.698	351.32	159.429
Prevalenc	Global	Male	50-69 yea	Syphilis	Number	2007	2366689	3560009	1556062
Prevalenc	Global	Female	50-69 yea	Syphilis	Number	2007	1151024	1713516	787445
Prevalenc	Global	Male	50-69 yea	Syphilis	Rate	2007	487.107	732.714	320.266
Prevalenc	Global	Female	50-69 yea	Syphilis	Rate	2007	231.42	344.513	158.321
Prevalenc	Global	Male	50-69 yea	Syphilis	Number	2008	2421498	3641138	1593296
Prevalenc	Global	Female	50-69 yea	Syphilis	Number	2008	1162756	1709788	801071
Prevalenc	Global	Male	50-69 yea	Syphilis	Rate	2008	485.622	730.216	319.529
Prevalenc	Global	Female	50-69 yea	Syphilis	Rate	2008	227.705	334.831	156.875
Prevalenc	Global	Male	50-69 yea	Syphilis	Number	2009	2478223	3724970	1632128
Prevalenc	Global	Female	50-69 yea	Syphilis	Number	2009	1177185	1713177	816498
Prevalenc	Global	Male	50-69 yea	Syphilis	Rate	2009	484.477	728.208	319.071
Prevalenc	Global	Female	50-69 yea	Syphilis	Rate	2009	224.491	326.706	155.707
Prevalenc	Global	Male	50-69 yea	Syphilis	Number	2010	2545108	3824681	1677227
Prevalenc	Global	Female	50-69 yea	Syphilis	Number	2010	1203019	1743364	837772
Prevalenc	Global	Male	50-69 yea	Syphilis	Rate	2010	484.716	728.41	319.428
Prevalenc	Global	Female	50-69 yea	Syphilis	Rate	2010	223.241	323.512	155.463
Prevalenc	Global	Male	50-69 yea	Syphilis	Number	2011	2612977	3926501	1721638
Prevalenc	Global	Female	50-69 yea	Syphilis	Number	2011	1234181	1785536	861486
Prevalenc	Global	Male	50-69 yea	Syphilis	Rate	2011	484.307	727.764	319.1
Prevalenc	Global	Female	50-69 yea	Syphilis	Rate	2011	222.543	321.962	155.34
Prevalenc	Global	Male	50-69 yea	Syphilis	Number	2012	2684032	4034190	1767502
Prevalenc	Global	Female	50-69 yea	Syphilis	Number	2012	1266924	1833117	883534
Prevalenc	Global	Male	50-69 yea	Syphilis	Rate	2012	483.283	726.391	318.254
Prevalenc	Global	Female	50-69 yea	Syphilis	Rate	2012	221.571	320.592	154.52
Prevalenc	Global	Male	50-69 yea	Syphilis	Number	2013	2762623	4153086	1818407
Prevalenc	Global	Female	50-69 yea	Syphilis	Number	2013	1303486	1887111	907742
Prevalenc	Global	Male	50-69 yea	Syphilis	Rate	2013	482.37	725.152	317.504
Prevalenc	Global	Female	50-69 yea	Syphilis	Rate	2013	220.645	319.437	153.656
Prevalenc	Global	Male	50-69 yea	Syphilis	Number	2014	2846180	4280105	1872973
Prevalenc	Global	Female	50-69 yea	Syphilis	Number	2014	1343817	1945899	934393
Prevalenc	Global	Male	50-69 yea	Syphilis	Rate	2014	481.491	724.07	316.853
Prevalenc	Global	Female	50-69 yea	Syphilis	Rate	2014	220.032	318.614	152.994
Prevalenc	Global	Male	50-69 yea	Syphilis	Number	2015	2937168	4421531	1933412
Prevalenc	Global	Female	50-69 yea	Syphilis	Number	2015	1389613	2014058	965030
Prevalenc	Global	Male	50-69 yea	Syphilis	Rate	2015	481.504	724.843	316.953
Prevalenc	Global	Female	50-69 yea	Syphilis	Rate	2015	220.137	319.059	152.876
Prevalenc	Global	Male	50-69 yea	Syphilis	Number	2016	3070289	4631948	2015701
Prevalenc	Global	Female	50-69 yea	Syphilis	Number	2016	1437019	2082622	992330
Prevalenc	Global	Male	50-69 yea	Syphilis	Rate	2016	488.995	737.715	321.034
Prevalenc	Global	Female	50-69 yea	Syphilis	Rate	2016	220.8	319.997	152.473
Prevalenc	Global	Male	50-69 yea	Syphilis	Number	2017	3208023	4849430	2096279
Prevalenc	Global	Female	50-69 yea	Syphilis	Number	2017	1488818	2165410	1029109
Prevalenc	Global	Male	50-69 yea	Syphilis	Rate	2017	498.002	752.808	325.419
Prevalenc	Global	Female	50-69 yea	Syphilis	Rate	2017	222.647	323.829	153.9
Prevalenc	Global	Male	50-69 yea	Syphilis	Number	2018	3301469	4998413	2160444
Prevalenc	Global	Female	50-69 yea	Syphilis	Number	2018	1553454	2257558	1071704
Prevalenc	Global	Male	50-69 yea	Syphilis	Rate	2018	499.78	756.665	327.05
Prevalenc	Global	Female	50-69 yea	Syphilis	Rate	2018	226.377	328.983	156.174
Prevalenc	Global	Male	50-69 yea	Syphilis	Number	2019	3388367	5132849	2213589
Prevalenc	Global	Female	50-69 yea	Syphilis	Number	2019	1633762	2375655	1125301
Prevalenc	Global	Male	50-69 yea	Syphilis	Rate	2019	501.149	759.163	327.396
Prevalenc	Global	Female	50-69 yea	Syphilis	Rate	2019	232.455	338.013	160.11
Prevalenc	Global	Male	50-69 yea	Other sexu	Number	1990	0	0	0
Prevalenc	Global	Female	50-69 yea	Other sexu	Number	1990	34051.2	52996.6	20834.2
Prevalenc	Global	Male	50-69 yea	Other sexu	Rate	1990	0	0	0
Prevalenc	Global	Female	50-69 yea	Other sexu	Rate	1990	9.85272	15.3346	6.02837
Prevalenc	Global	Male	50-69 yea	Other sexu	Number	1991	0	0	0
Prevalenc	Global	Female	50-69 yea	Other sexu	Number	1991	33810.5	52481.4	20776.6
Prevalenc	Global	Male	50-69 yea	Other sexu	Rate	1991	0	0	0
Prevalenc	Global	Female	50-69 yea	Other sexu	Rate	1991	9.62483	14.9399	5.91447
Prevalenc	Global	Male	50-69 yea	Other sexu	Number	1992	0	0	0
Prevalenc	Global	Female	50-69 yea	Other sexu	Number	1992	33600.3	51992.2	20750.5
Prevalenc	Global	Male	50-69 yea	Other sexu	Rate	1992	0	0	0
Prevalenc	Global	Female	50-69 yea	Other sexu	Rate	1992	9.42551	14.5848	5.8209
Prevalenc	Global	Male	50-69 yea	Other sexu	Number	1993	0	0	0
Prevalenc	Global	Female	50-69 yea	Other sexu	Number	1993	33299.3	51368	20667
Prevalenc	Global	Male	50-69 yea	Other sexu	Rate	1993	0	0	0
Prevalenc	Global	Female	50-69 yea	Other sexu	Rate	1993	9.22151	14.2253	5.72328
Prevalenc	Global	Male	50-69 yea	Other sexu	Number	1994	0	0	0
Prevalenc	Global	Female	50-69 yea	Other sexu	Number	1994	33089.8	50929.3	20635.9
Prevalenc	Global	Male	50-69 yea	Other sexu	Rate	1994	0	0	0
Prevalenc	Global	Female	50-69 yea	Other sexu	Rate	1994	9.05133	13.9311	5.64471
Prevalenc	Global	Male	50-69 yea	Other sexu	Number	1995	0	0	0
Prevalenc	Global	Female	50-69 yea	Other sexu	Number	1995	32964.8	50600.9	20654.7
Prevalenc	Global	Male	50-69 yea	Other sexu	Rate	1995	0	0	0
Prevalenc	Global	Female	50-69 yea	Other sexu	Rate	1995	8.91054	13.6777	5.58307
Prevalenc	Global	Male	50-69 yea	Other sexu	Number	1996	0	0	0
Prevalenc	Global	Female	50-69 yea	Other sexu	Number	1996	33086.7	50825.4	20707.7
Prevalenc	Global	Male	50-69 yea	Other sexu	Rate	1996	0	0	0
Prevalenc	Global	Female	50-69 yea	Other sexu	Rate	1996	8.814	13.5394	5.51635
Prevalenc	Global	Male	50-69 yea	Other sexu	Number	1997	0	0	0
Prevalenc	Global	Female	50-69 yea	Other sexu	Number	1997	33612	51690.1	20936.8
Prevalenc	Global	Male	50-69 yea	Other sexu	Rate	1997	0	0	0
Prevalenc	Global	Female	50-69 yea	Other sexu	Rate	1997	8.77945	13.5014	5.46868
Prevalenc	Global	Male	50-69 yea	Other sexu	Number	1998	0	0	0
Prevalenc	Global	Female	50-69 yea	Other sexu	Number	1998	34257.5	52854.5	21207
Prevalenc	Global	Male	50-69 yea	Other sexu	Rate	1998	0	0	0
Prevalenc	Global	Female	50-69 yea	Other sexu	Rate	1998	8.76703	13.5263	5.42722
Prevalenc	Global	Male	50-69 yea	Other sexu	Number	1999	0	0	0
Prevalenc	Global	Female	50-69 yea	Other sexu	Number	1999	35201.8	54560.6	21664.7
Prevalenc	Global	Male	50-69 yea	Other sexu	Rate	1999	0	0	0
Prevalenc	Global	Female	50-69 yea	Other sexu	Rate	1999	8.80703	13.6503	5.42022
Prevalenc	Global	Male	50-69 yea	Other sexu	Number	2000	0	0	0
Prevalenc	Global	Female	50-69 yea	Other sexu	Number	2000	36009	55998.2	22069.3
Prevalenc	Global	Male	50-69 yea	Other sexu	Rate	2000	0	0	0
Prevalenc	Global	Female	50-69 yea	Other sexu	Rate	2000	8.82387	13.7221	5.40799
Prevalenc	Global	Male	50-69 yea	Other sexu	Number	2001	0	0	0
Prevalenc	Global	Female	50-69 yea	Other sexu	Number	2001	37240.8	57871	22796.7
Prevalenc	Global	Male	50-69 yea	Other sexu	Rate	2001	0	0	0
Prevalenc	Global	Female	50-69 yea	Other sexu	Rate	2001	8.88527	13.8074	5.43907
Prevalenc	Global	Male	50-69 yea	Other sexu	Number	2002	0	0	0
Prevalenc	Global	Female	50-69 yea	Other sexu	Number	2002	38363.3	59445.9	23520.9
Prevalenc	Global	Male	50-69 yea	Other sexu	Rate	2002	0	0	0
Prevalenc	Global	Female	50-69 yea	Other sexu	Rate	2002	8.90422	13.7976	5.45926
Prevalenc	Global	Male	50-69 yea	Other sexu	Number	2003	0	0	0
Prevalenc	Global	Female	50-69 yea	Other sexu	Number	2003	39648.7	61287.5	24355.5
Prevalenc	Global	Male	50-69 yea	Other sexu	Rate	2003	0	0	0
Prevalenc	Global	Female	50-69 yea	Other sexu	Rate	2003	8.94457	13.8262	5.4945
Prevalenc	Global	Male	50-69 yea	Other sexu	Number	2004	0	0	0
Prevalenc	Global	Female	50-69 yea	Other sexu	Number	2004	41016.6	63310.1	25304.6
Prevalenc	Global	Male	50-69 yea	Other sexu	Rate	2004	0	0	0
Prevalenc	Global	Female	50-69 yea	Other sexu	Rate	2004	8.98441	13.8676	5.5428
Prevalenc	Global	Male	50-69 yea	Other sexu	Number	2005	0	0	0
Prevalenc	Global	Female	50-69 yea	Other sexu	Number	2005	42554.1	65598.2	26342.3
Prevalenc	Global	Male	50-69 yea	Other sexu	Rate	2005	0	0	0
Prevalenc	Global	Female	50-69 yea	Other sexu	Rate	2005	9.04728	13.9466	5.60055
Prevalenc	Global	Male	50-69 yea	Other sexu	Number	2006	0	0	0
Prevalenc	Global	Female	50-69 yea	Other sexu	Number	2006	43771.1	67113.4	27170.8
Prevalenc	Global	Male	50-69 yea	Other sexu	Rate	2006	0	0	0
Prevalenc	Global	Female	50-69 yea	Other sexu	Rate	2006	9.04429	13.8675	5.61422
Prevalenc	Global	Male	50-69 yea	Other sexu	Number	2007	0	0	0
Prevalenc	Global	Female	50-69 yea	Other sexu	Number	2007	44601.4	67878.6	27781.1
Prevalenc	Global	Male	50-69 yea	Other sexu	Rate	2007	0	0	0
Prevalenc	Global	Female	50-69 yea	Other sexu	Rate	2007	8.96738	13.6474	5.58557
Prevalenc	Global	Male	50-69 yea	Other sexu	Number	2008	0	0	0
Prevalenc	Global	Female	50-69 yea	Other sexu	Number	2008	45250.2	68242.6	28368
Prevalenc	Global	Male	50-69 yea	Other sexu	Rate	2008	0	0	0
Prevalenc	Global	Female	50-69 yea	Other sexu	Rate	2008	8.86144	13.3641	5.55537
Prevalenc	Global	Male	50-69 yea	Other sexu	Number	2009	0	0	0
Prevalenc	Global	Female	50-69 yea	Other sexu	Number	2009	45752.3	68423.1	28796.3
Prevalenc	Global	Male	50-69 yea	Other sexu	Rate	2009	0	0	0
Prevalenc	Global	Female	50-69 yea	Other sexu	Rate	2009	8.72504	13.0484	5.4915
Prevalenc	Global	Male	50-69 yea	Other sexu	Number	2010	0	0	0
Prevalenc	Global	Female	50-69 yea	Other sexu	Number	2010	46521.4	69357.5	29407.5
Prevalenc	Global	Male	50-69 yea	Other sexu	Rate	2010	0	0	0
Prevalenc	Global	Female	50-69 yea	Other sexu	Rate	2010	8.63287	12.8705	5.45708
Prevalenc	Global	Male	50-69 yea	Other sexu	Number	2011	0	0	0
Prevalenc	Global	Female	50-69 yea	Other sexu	Number	2011	47299.9	70516.2	29871
Prevalenc	Global	Male	50-69 yea	Other sexu	Rate	2011	0	0	0
Prevalenc	Global	Female	50-69 yea	Other sexu	Rate	2011	8.52896	12.7152	5.38623
Prevalenc	Global	Male	50-69 yea	Other sexu	Number	2012	0	0	0
Prevalenc	Global	Female	50-69 yea	Other sexu	Number	2012	48334.4	72244.5	30514.1
Prevalenc	Global	Male	50-69 yea	Other sexu	Rate	2012	0	0	0
Prevalenc	Global	Female	50-69 yea	Other sexu	Rate	2012	8.45315	12.6348	5.33657
Prevalenc	Global	Male	50-69 yea	Other sexu	Number	2013	0	0	0
Prevalenc	Global	Female	50-69 yea	Other sexu	Number	2013	49668.7	74474.6	31274.5
Prevalenc	Global	Male	50-69 yea	Other sexu	Rate	2013	0	0	0
Prevalenc	Global	Female	50-69 yea	Other sexu	Rate	2013	8.40758	12.6065	5.29393
Prevalenc	Global	Male	50-69 yea	Other sexu	Number	2014	0	0	0
Prevalenc	Global	Female	50-69 yea	Other sexu	Number	2014	51190.4	77125.1	32061.7
Prevalenc	Global	Male	50-69 yea	Other sexu	Rate	2014	0	0	0
Prevalenc	Global	Female	50-69 yea	Other sexu	Rate	2014	8.38172	12.6282	5.24966
Prevalenc	Global	Male	50-69 yea	Other sexu	Number	2015	0	0	0
Prevalenc	Global	Female	50-69 yea	Other sexu	Number	2015	52821.7	79920.8	32911
Prevalenc	Global	Male	50-69 yea	Other sexu	Rate	2015	0	0	0
Prevalenc	Global	Female	50-69 yea	Other sexu	Rate	2015	8.36782	12.6607	5.21363
Prevalenc	Global	Male	50-69 yea	Other sexu	Number	2016	0	0	0
Prevalenc	Global	Female	50-69 yea	Other sexu	Number	2016	54988.7	84150.3	34166.5
Prevalenc	Global	Male	50-69 yea	Other sexu	Rate	2016	0	0	0
Prevalenc	Global	Female	50-69 yea	Other sexu	Rate	2016	8.44908	12.9298	5.24972
Prevalenc	Global	Male	50-69 yea	Other sexu	Number	2017	0	0	0

Prevalence	Global	Male	50-69 yea	Gonococca	Number	1996	584447	920113	380033
Prevalence	Global	Female	50-69 yea	Gonococca	Number	1996	575705	893054	378878
Prevalence	Global	Male	50-69 yea	Gonococca	Rate	1996	158.665	249.791	103.171
Prevalence	Global	Female	50-69 yea	Gonococca	Rate	1996	153.362	237.901	100.929
Prevalence	Global	Male	50-69 yea	Gonococca	Number	1997	594662	936127	386830
Prevalence	Global	Female	50-69 yea	Gonococca	Number	1997	585010	908960	386137
Prevalence	Global	Male	50-69 yea	Gonococca	Rate	1997	158.217	249.069	102.921
Prevalence	Global	Female	50-69 yea	Gonococca	Rate	1997	152.805	237.42	100.859
Prevalence	Global	Male	50-69 yea	Gonococca	Number	1998	606179	956056	394819
Prevalence	Global	Female	50-69 yea	Gonococca	Number	1998	596839	926563	393253
Prevalence	Global	Male	50-69 yea	Gonococca	Rate	1998	158.1	249.352	102.974
Prevalence	Global	Female	50-69 yea	Gonococca	Rate	1998	152.741	237.122	100.64
Prevalence	Global	Male	50-69 yea	Gonococca	Number	1999	622965	987487	404489
Prevalence	Global	Female	50-69 yea	Gonococca	Number	1999	614141	954492	403104
Prevalence	Global	Male	50-69 yea	Gonococca	Rate	1999	158.755	251.649	103.079
Prevalence	Global	Female	50-69 yea	Gonococca	Rate	1999	153.65	238.801	100.851
Prevalence	Global	Male	50-69 yea	Gonococca	Number	2000	637986	1012731	413500
Prevalence	Global	Female	50-69 yea	Gonococca	Number	2000	629128	981458	411861
Prevalence	Global	Male	50-69 yea	Gonococca	Rate	2000	159.442	253.096	103.34
Prevalence	Global	Female	50-69 yea	Gonococca	Rate	2000	154.165	240.502	100.925
Prevalence	Global	Male	50-69 yea	Gonococca	Number	2001	661818	1048620	427567
Prevalence	Global	Female	50-69 yea	Gonococca	Number	2001	649744	1015512	424515
Prevalence	Global	Male	50-69 yea	Gonococca	Rate	2001	161.181	255.384	104.131
Prevalence	Global	Female	50-69 yea	Gonococca	Rate	2001	155.022	242.291	101.285
Prevalence	Global	Male	50-69 yea	Gonococca	Number	2002	683924	1085317	441012
Prevalence	Global	Female	50-69 yea	Gonococca	Number	2002	669143	1045400	436271
Prevalence	Global	Male	50-69 yea	Gonococca	Rate	2002	162.184	257.369	104.58
Prevalence	Global	Female	50-69 yea	Gonococca	Rate	2002	155.31	242.64	101.26
Prevalence	Global	Male	50-69 yea	Gonococca	Number	2003	707965	1125231	455648
Prevalence	Global	Female	50-69 yea	Gonococca	Number	2003	691728	1079717	450038
Prevalence	Global	Male	50-69 yea	Gonococca	Rate	2003	163.283	259.521	105.09
Prevalence	Global	Female	50-69 yea	Gonococca	Rate	2003	156.051	243.58	101.527
Prevalence	Global	Male	50-69 yea	Gonococca	Number	2004	732367	1160489	470272
Prevalence	Global	Female	50-69 yea	Gonococca	Number	2004	715373	1113991	463441
Prevalence	Global	Male	50-69 yea	Gonococca	Rate	2004	164.107	260.04	105.377
Prevalence	Global	Female	50-69 yea	Gonococca	Rate	2004	156.697	244.012	101.514
Prevalence	Global	Male	50-69 yea	Gonococca	Number	2005	760491	1202616	488502
Prevalence	Global	Female	50-69 yea	Gonococca	Number	2005	742114	1157351	479780
Prevalence	Global	Male	50-69 yea	Gonococca	Rate	2005	165.479	261.683	106.296
Prevalence	Global	Female	50-69 yea	Gonococca	Rate	2005	157.778	246.061	102.004
Prevalence	Global	Male	50-69 yea	Gonococca	Number	2006	783656	1240021	503726
Prevalence	Global	Female	50-69 yea	Gonococca	Number	2006	760697	1184660	490989
Prevalence	Global	Male	50-69 yea	Gonococca	Rate	2006	165.745	262.267	106.539
Prevalence	Global	Female	50-69 yea	Gonococca	Rate	2006	157.181	244.783	101.451
Prevalence	Global	Male	50-69 yea	Gonococca	Number	2007	803843	1272063	517534
Prevalence	Global	Female	50-69 yea	Gonococca	Number	2007	770273	1197612	499060
Prevalence	Global	Male	50-69 yea	Gonococca	Rate	2007	165.445	261.813	106.518
Prevalence	Global	Female	50-69 yea	Gonococca	Rate	2007	154.868	240.787	100.339
Prevalence	Global	Male	50-69 yea	Gonococca	Number	2008	822208	1304892	531234
Prevalence	Global	Female	50-69 yea	Gonococca	Number	2008	775418	1200132	504043
Prevalence	Global	Male	50-69 yea	Gonococca	Rate	2008	164.891	261.691	106.537
Prevalence	Global	Female	50-69 yea	Gonococca	Rate	2008	151.852	235.025	98.7079
Prevalence	Global	Male	50-69 yea	Gonococca	Number	2009	838776	1332669	541506
Prevalence	Global	Female	50-69 yea	Gonococca	Number	2009	780431	1197015	508740
Prevalence	Global	Male	50-69 yea	Gonococca	Rate	2009	163.975	260.528	105.861
Prevalence	Global	Female	50-69 yea	Gonococca	Rate	2009	148.829	228.273	97.0175
Prevalence	Global	Male	50-69 yea	Gonococca	Number	2010	857801	1364227	556646
Prevalence	Global	Female	50-69 yea	Gonococca	Number	2010	792125	1215047	517309
Prevalence	Global	Male	50-69 yea	Gonococca	Rate	2010	163.368	259.817	106.013
Prevalence	Global	Female	50-69 yea	Gonococca	Rate	2010	146.993	225.474	95.9959
Prevalence	Global	Male	50-69 yea	Gonococca	Number	2011	875437	1387605	569150
Prevalence	Global	Female	50-69 yea	Gonococca	Number	2011	807244	1239441	529170
Prevalence	Global	Male	50-69 yea	Gonococca	Rate	2011	162.259	257.188	105.49
Prevalence	Global	Female	50-69 yea	Gonococca	Rate	2011	145.56	223.492	95.4181
Prevalence	Global	Male	50-69 yea	Gonococca	Number	2012	894292	1412363	581826
Prevalence	Global	Female	50-69 yea	Gonococca	Number	2012	825232	1267023	540926
Prevalence	Global	Male	50-69 yea	Gonococca	Rate	2012	161.025	254.308	104.763
Prevalence	Global	Female	50-69 yea	Gonococca	Rate	2012	144.324	221.588	94.6019
Prevalence	Global	Male	50-69 yea	Gonococca	Number	2013	916121	1444631	595799
Prevalence	Global	Female	50-69 yea	Gonococca	Number	2013	846953	1302677	555439
Prevalence	Global	Male	50-69 yea	Gonococca	Rate	2013	159.96	252.241	104.03
Prevalence	Global	Female	50-69 yea	Gonococca	Rate	2013	143.366	220.508	94.0209
Prevalence	Global	Male	50-69 yea	Gonococca	Number	2014	939615	1479108	611274
Prevalence	Global	Female	50-69 yea	Gonococca	Number	2014	869882	1339432	571165
Prevalence	Global	Male	50-69 yea	Gonococca	Rate	2014	158.956	250.222	103.41
Prevalence	Global	Female	50-69 yea	Gonococca	Rate	2014	142.431	219.314	93.5204
Prevalence	Global	Male	50-69 yea	Gonococca	Number	2015	964371	1518099	625798
Prevalence	Global	Female	50-69 yea	Gonococca	Number	2015	893600	1377714	585746
Prevalence	Global	Male	50-69 yea	Gonococca	Rate	2015	158.094	248.869	102.59
Prevalence	Global	Female	50-69 yea	Gonococca	Rate	2015	141.561	218.252	92.7916
Prevalence	Global	Male	50-69 yea	Gonococca	Number	2016	984625	1550166	640256
Prevalence	Global	Female	50-69 yea	Gonococca	Number	2016	911346	1407739	600257
Prevalence	Global	Male	50-69 yea	Gonococca	Rate	2016	156.818	246.89	101.971
Prevalence	Global	Female	50-69 yea	Gonococca	Rate	2016	140.029	216.301	92.2301
Prevalence	Global	Male	50-69 yea	Gonococca	Number	2017	1004914	1582922	654064
Prevalence	Global	Female	50-69 yea	Gonococca	Number	2017	927524	1437091	611697
Prevalence	Global	Male	50-69 yea	Gonococca	Rate	2017	155.999	245.727	101.535
Prevalence	Global	Female	50-69 yea	Gonococca	Rate	2017	138.708	214.912	91.4772
Prevalence	Global	Male	50-69 yea	Gonococca	Number	2018	1028540	1633171	669366
Prevalence	Global	Female	50-69 yea	Gonococca	Number	2018	956947	1473300	624609
Prevalence	Global	Male	50-69 yea	Gonococca	Rate	2018	155.701	247.231	101.329
Prevalence	Global	Female	50-69 yea	Gonococca	Rate	2018	139.451	214.697	91.0213
Prevalence	Global	Male	50-69 yea	Gonococca	Number	2019	1053202	1657662	683054
Prevalence	Global	Female	50-69 yea	Gonococca	Number	2019	999040	1547496	648298
Prevalence	Global	Male	50-69 yea	Gonococca	Rate	2019	155.772	245.173	101.026
Prevalence	Global	Female	50-69 yea	Gonococca	Rate	2019	142.146	220.181	92.2412
Prevalence	Global	Male	50-69 yea	Chlamydia	Number	1990	1517542	2373864	885267
Prevalence	Global	Female	50-69 yea	Chlamydia	Number	1990	2933269	4464456	1832158
Prevalence	Global	Male	50-69 yea	Chlamydia	Rate	1990	450.93	705.382	263.053
Prevalence	Global	Female	50-69 yea	Chlamydia	Rate	1990	848.741	1291.79	530.135
Prevalence	Global	Male	50-69 yea	Chlamydia	Number	1991	1529315	2387705	894014
Prevalence	Global	Female	50-69 yea	Chlamydia	Number	1991	2963031	4510757	1850157
Prevalence	Global	Male	50-69 yea	Chlamydia	Rate	1991	446.093	696.481	260.779
Prevalence	Global	Female	50-69 yea	Chlamydia	Rate	1991	843.486	1284.08	526.684
Prevalence	Global	Male	50-69 yea	Chlamydia	Number	1992	1538351	2401743	901606
Prevalence	Global	Female	50-69 yea	Chlamydia	Number	1992	2985687	4546720	1866544
Prevalence	Global	Male	50-69 yea	Chlamydia	Rate	1992	441.48	689.259	258.745
Prevalence	Global	Female	50-69 yea	Chlamydia	Rate	1992	837.541	1275.44	523.6
Prevalence	Global	Male	50-69 yea	Chlamydia	Number	1993	1545652	2412992	907861
Prevalence	Global	Female	50-69 yea	Chlamydia	Number	1993	2992104	4546996	1874964
Prevalence	Global	Male	50-69 yea	Chlamydia	Rate	1993	437.045	682.292	256.705
Prevalence	Global	Female	50-69 yea	Chlamydia	Rate	1993	828.598	1259.19	519.23
Prevalence	Global	Male	50-69 yea	Chlamydia	Number	1994	1552656	2423710	913766
Prevalence	Global	Female	50-69 yea	Chlamydia	Number	1994	2999807	4558049	1888390
Prevalence	Global	Male	50-69 yea	Chlamydia	Rate	1994	433.119	676.102	254.898
Prevalence	Global	Female	50-69 yea	Chlamydia	Rate	1994	820.562	1246.8	516.547
Prevalence	Global	Male	50-69 yea	Chlamydia	Number	1995	1563629	2440659	921609
Prevalence	Global	Female	50-69 yea	Chlamydia	Number	1995	3014347	4572590	1896330
Prevalence	Global	Male	50-69 yea	Chlamydia	Rate	1995	430.763	672.375	253.893
Prevalence	Global	Female	50-69 yea	Chlamydia	Rate	1995	814.793	1235.99	512.587
Prevalence	Global	Male	50-69 yea	Chlamydia	Number	1996	1593162	2488033	937821
Prevalence	Global	Female	50-69 yea	Chlamydia	Number	1996	3071507	4662677	1935026
Prevalence	Global	Male	50-69 yea	Chlamydia	Rate	1996	432.51	675.449	254.599
Prevalence	Global	Female	50-69 yea	Chlamydia	Rate	1996	818.221	1242.09	515.473
Prevalence	Global	Male	50-69 yea	Chlamydia	Number	1997	1649875	2577867	967694
Prevalence	Global	Female	50-69 yea	Chlamydia	Number	1997	3175072	4825884	1997148
Prevalence	Global	Male	50-69 yea	Chlamydia	Rate	1997	438.97	685.875	257.467
Prevalence	Global	Female	50-69 yea	Chlamydia	Rate	1997	829.328	1260.52	521.654
Prevalence	Global	Male	50-69 yea	Chlamydia	Number	1998	1713214	2677424	1000442
Prevalence	Global	Female	50-69 yea	Chlamydia	Number	1998	3305983	5023126	2066540
Prevalence	Global	Male	50-69 yea	Chlamydia	Rate	1998	446.83	698.309	260.929
Prevalence	Global	Female	50-69 yea	Chlamydia	Rate	1998	846.054	1285.5	528.86
Prevalence	Global	Male	50-69 yea	Chlamydia	Number	1999	1800789	2828852	1045313
Prevalence	Global	Female	50-69 yea	Chlamydia	Number	1999	3475732	5281652	2161027
Prevalence	Global	Male	50-69 yea	Chlamydia	Rate	1999	458.909	720.899	266.385
Prevalence	Global	Female	50-69 yea	Chlamydia	Rate	1999	869.582	1321.4	540.66
Prevalence	Global	Male	50-69 yea	Chlamydia	Number	2000	1861648	2929295	1079011
Prevalence	Global	Female	50-69 yea	Chlamydia	Number	2000	3607189	5503714	2230669
Prevalence	Global	Male	50-69 yea	Chlamydia	Rate	2000	465.253	732.073	269.661
Prevalence	Global	Female	50-69 yea	Chlamydia	Rate	2000	883.927	1348.66	546.617
Prevalence	Global	Male	50-69 yea	Chlamydia	Number	2001	1944108	3057099	1126815
Prevalence	Global	Female	50-69 yea	Chlamydia	Number	2001	3769285	5766728	2318024
Prevalence	Global	Male	50-69 yea	Chlamydia	Rate	2001	473.475	744.536	274.428
Prevalence	Global	Female	50-69 yea	Chlamydia	Rate	2001	899.313	1375.88	553.057
Prevalence	Global	Male	50-69 yea	Chlamydia	Number	2002	2016816	3168057	1168958
Prevalence	Global	Female	50-69 yea	Chlamydia	Number	2002	3923180	6014484	2404605
Prevalence	Global	Male	50-69 yea	Chlamydia	Rate	2002	478.263	751.265	277.204
Prevalence	Global	Female	50-69 yea	Chlamydia	Rate	2002	910.58	1395.98	558.115
Prevalence	Global	Male	50-69 yea	Chlamydia	Number	2003	2097573	3297199	1215464
Prevalence	Global	Female	50-69 yea	Chlamydia	Number	2003	4097565	6299041	2505973
Prevalence	Global	Male	50-69 yea	Chlamydia	Rate	2003	483.779	760.458	280.332
Prevalence	Global	Female	50-69 yea	Chlamydia	Rate	2003	924.394	1421.04	565.337
Prevalence	Global	Male	50-69 yea	Chlamydia	Number	2004	2171810	3403239	1257711
Prevalence	Global	Female	50-69 yea	Chlamydia	Number	2004	4270484	6569072	2611958
Prevalence	Global	Male	50-69 yea	Chlamydia	Rate	2004	486.654	762.59	281.825
Prevalence	Global	Female	50-69 yea	Chlamydia	Rate	2004	935.42	1438.91	572.131
Prevalence	Global	Male	50-69 yea	Chlamydia	Number	2005	2265679	3554002	1311006
Prevalence	Global	Female	50-69 yea	Chlamydia	Number	2005	4466407	6878789	2734106
Prevalence	Global	Male	50-69 yea	Chlamydia	Rate	2005	493.001	773.334	285.268
Prevalence	Global	Female	50-69 yea	Chlamydia	Rate	2005	949.588	1462.48	581.289
Prevalence	Global	Male	50-69 yea	Chlamydia	Number	2006	2321301	3637378	1343157
Prevalence	Global	Female	50-69 yea	Chlamydia	Number	2006	4572442	7019257	2801885
Prevalence	Global	Male	50-69 yea	Chlamydia	Rate	2006	490.96	769.313	284.081
Prevalence	Global	Female	50-69 yea	Chlamydia	Rate	2006	944.79	1450.37	578.945
Prevalence	Global	Male	50-69 yea	Chlamydia	Number	2007	2349211	3682080	1360006
Prevalence	Global	Female	50-69 yea	Chlamydia	Number	2007	4597007	7032326	2833595
Prevalence	Global	Male	50-69 yea	Chlamydia	Rate	2007	483.51	757.838	279.914
Prevalence	Global	Female	50-69 yea	Chlamydia	Rate	2007	924.256	1413.89	569.712
Prevalence	Global	Male	50-69 yea	Chlamydia	Number	2008	2357734	3697965	1369535
Prevalence	Global	Female	50-69 yea	Chlamydia	Number	2008	4574847	6973798	2841328
Prevalence	Global	Male	50-69 yea	Chlamydia	Rate	2008	472.834	741.612	274.655
Prevalence	Global	Female	50-69 yea	Chlamydia	Rate	2008	895.902	1365.69	556.423
Prevalence	Global	Male	50-69 yea	Chlamydia	Number	2009	2360621	3701619	1378884
Prevalence	Global	Female	50-69 yea	Chlamydia	Number	2009	4557784	6945271	2853840
Prevalence	Global	Male	50-69 yea	Chlamydia	Rate	2009	461.486	723.643	269.563
Prevalence	Global	Female	50-69 yea	Chlamydia	Rate	2009	869.177	1324.47	544.232
Prevalence	Global	Male	50-69 yea	Chlamydia	Number	2010	2386331	3738811	1399992
Prevalence	Global	Female	50-69 yea	Chlamydia	Number	2010	4610373	7000657	2909958
Prevalence	Global	Male	50-69 yea	Chlamydia	Rate	2010	454.476	712.056	266.628
Prevalence	Global	Female	50-69 yea	Chlamydia	Rate	2010	855.536	1299.1	539.994
Prevalence	Global	Male	50-69 yea	Chlamydia	Number	2011	2429003	3797055	1425758
Prevalence	Global	Female	50-69 yea	Chlamydia	Number	2011	4713875	7141169	2974478
Prevalence	Global	Male	50-69 yea	Chlamydia	Rate	2011	450.208	703.772	264.26
Prevalence	Global	Female	50-69 yea	Chlamydia	Rate	2011	849.99	1287.67	536.348
Prevalence	Global	Male	50-69 yea	Chlamydia	Number	2012	2491370	3891837	1463055
Prevalence	Global	Female	50-69 yea	Chlamydia	Number	2012	4851599	7364709	3060440
Prevalence	Global	Male	50-69 yea	Chlamydia	Rate	2012	448.593	700.759	263.436
Prevalence	Global	Female	50-69 yea	Chlamydia	Rate	2012	848.491	1288.01	535.237
Prevalence	Global	Male	50-69 yea	Chlamydia	Number	2013	2576740	4019787	1510939
Prevalence	Global	Female	50-69 yea	Chlamydia	Number	2013	5027363	7642520	3156250
Prevalence	Global	Male	50-69 yea	Chlamydia	Rate	2013	449.913	701.877	263.818
Prevalence	Global	Female	50-69 yea	Chlamydia	Rate	2013	850.997	1293.67	534.268
Prevalence	Global	Male	50-69 yea	Chlamydia	Number	2014	2673366	4167597	1563314
Prevalence	Global	Female	50-69 yea	Chlamydia	Number	2014	5219807	7951382	3263674
Prevalence	Global	Male	50-69 yea	Chlamydia	Rate	2014	452.256	705.037	264.467
Prevalence	Global	Female	50-69 yea	Chlamydia	Rate	2014	854.672	1301.93	534.382
Prevalence	Global	Male	50-69 yea	Chlamydia	Number	2015	2770498	4316096	1623686
Prevalence	Global	Female	50-69 yea	Chlamydia	Number	2015	5414088	8239955	3374435
Prevalence	Global	Male	50-69 yea	Chlamydia	Rate	2015	454.181	707.558	266.179
Prevalence	Global	Female	50-69 yea	Chlamydia	Rate	2015	857.679	1305.34	534.565
Prevalence	Global	Male	50-69 yea	Chlamydia	Number	2016	2850642	4450269	1660988
Prevalence	Global	Female	50-69 yea	Chlamydia	Number	2016	5594448	8521823	3486403
Prevalence	Global	Male	50-69 yea	Chlamydia	Rate	2016	454.012	708.779	264.54
Prevalence	Global	Female	50-69 yea	Chlamydia	Rate	2016	859.593	1309.39	535.69
Prevalence	Global	Male	50-69 yea	Chlamydia	Number	2017	2924408	4585327	1706848
Prevalence	Global	Female	50-69 yea	Chlamydia	Number	2017	5761640	8794471	3595200
Prevalence	Global	Male	50-69 yea	Chlamydia	Rate	2017	453.975	711.81	264.965
Prevalence	Global	Female	50-69 yea	Chlamydia	Rate	2017	861.633	1315.18	537.649
Prevalence	Global	Male	50-69 yea	Chlamydia	Number	2018	3025652	4734519	1763924
Prevalence	Global	Female	50-69 yea	Chlamydia	Number	2018	5994132	9140457	3724389
Prevalence	Global	Male	50-69 yea	Chlamydia	Rate	2018	458.026	716.716	267.025
Prevalence	Global	Female	50-69 yea	Chlamydia	Rate	2018	873.496	1331.99	542.737
Prevalence	Global	Male	50-69 yea	Chlamydia	Number	2019	3159345	4934506	1840583
Prevalence	Global	Female	50-69 yea	Chlamydia	Number	2019	6311970	9619447	3909569
Prevalence	Global	Male	50-69 yea	Chlamydia	Rate	2019	467.276	729.827	272.227
Prevalence	Global	Female	50-69 yea	Chlamydia	Rate	2019	898.081	1368.68	556.262
Prevalence	Global	Male	50-69 yea	Trichomon	Number	1990	2709404	3846230	1824617
Prevalence	Global	Female	50-69 yea	Trichomon	Number	1990	1.4E+07	1.9E+07	8915850
Prevalence	Global	Male	50-69 yea	Trichomon	Rate	1990	805.086	1142.89	542.176
Prevalence	Global	Female	50-69 yea	Trichomon	Rate	1990	3932.41	5620.08	2579.8
Prevalence	Global	Male	50-69 yea	Trichomon	Number	1991	2748932	3892932	1855755
Prevalence	Global	Female	50-69 yea	Trichomon	Number	1991	1.4E+07	2E+07	9138264
Prevalence	Global	Male	50-69 yea	Trichomon	Rate	1991	801.849	1135.55	541.314
Prevalence	Global	Female	50-69 yea	Trichomon	Rate	1991	3966.48	5648.41	2601.39
Prevalence	Global	Male	50-69 yea	Trichomon	Number	1992	2784352	3936240	1883849
Prevalence	Global	Female	50-69 yea	Trichomon	Number	1992	1.4E+07	2E+07	9335623
Prevalence	Global	Male	50-69 yea	Trichomon	Rate	1992	799.061	1129.63	540.632
Prevalence	Global	Female	50-69 yea	Trichomon	Rate	1992	3996.09	5672.88	2618.82
Prevalence	Global	Male	50-69 yea	Trichomon	Number	1993	2817648	3977942	1910350
Prevalence	Global	Female	50-69 yea	Trichomon	Number	1993	1.5E+07	2.1E+07	9506344
Prevalence	Global	Male	50-69 yea	Trichomon	Rate	1993	796.712	1124.79	540.166
Prevalence	Global	Female	50-69 yea	Trichomon	Rate	1993	4019.35	5703.85	2632.57
Prevalence	Global	Male	50-69 yea	Trichomon	Number	1994	2850200	4020074	1936108
Prevalence	Global	Female	50-69 yea	Trichomon	Number	1994	1.5E+07	2.1E+07	9669082
Prevalence	Global	Male	50-69 yea	Trichomon	Rate	1994	795.073	1121.41	540.084
Prevalence	Global	Female	50-69 yea	Trichomon	Rate	1994	4037.54	5741.49	2644.86
Prevalence	Global	Male	50-69 yea	Trichomon	Number	1995	2883651	4064810	1962016
Prevalence	Global	Female	50-69 yea	Trichomon	Number	1995	1.5E+07	2.1E+07	9815479
Prevalence	Global	Male	50-69 yea	Trichomon	Rate	1995	794.415	1119.81	540.514
Prevalence	Global	Female	50-69 yea	Trichomon	Rate	1995	4050.2	5764.56	2653.17
Prevalence	Global	Male	50-69 yea	Trichomon	Number	1996	2931888	4130723	1993017
Prevalence	Global	Female	50-69 yea	Trichomon	Number	1996	1.5E+07	2.2E+07	9959079
Prevalence	Global	Male	50-69 yea	Trichomon	Rate	1996	795.946	1121.4	541.062
Prevalence	Global	Female	50-69 yea	Trichomon	Rate	1996	4050.2	5745.33	2653.01
Prevalence	Global	Male	50-69 yea	Trichomon	Number	1997	3006816	4238965	2039878
Prevalence	Global	Female	50-69 yea	Trichomon	Number	1997	1.5E+07	2.2E+07	1E+07
Prevalence	Global	Male	50-69 yea	Trichomon	Rate	1997	800.002	1127.83	542.736
Prevalence	Global	Female	50-69 yea	Trichomon	Rate	1997	4034.9	5721.37	2642.51
Prevalence	Global	Male	50-69 yea	Trichomon	Number	1998	3084872	4353299	2088443
Prevalence	Global	Female	50-69 yea	Trichomon	Number	1998	1.6E+07	2.2E+07	1E+07
Prevalence	Global	Male	50-69 yea	Trichomon	Rate	1998	804.577	1135.4	544.694
Prevalence	Global	Female	50-69 yea	Trichomon	Rate	1998	4013.29	5687.88	2626.35
Prevalence	Global	Male	50-69 yea	Trichomon	Number	1999	3183220	4513131	2152116
Prevalence	Global	Female	50-69 yea	Trichomon	Number	1999	1.6E+07	2.3E+07	1E+07
Prevalence	Global	Male	50-69 yea	Trichomon	Rate	1999	811.205	1150.12	548.441
Prevalence	Global	Female	50-69 yea	Trichomon	Rate	1999	3996.73	5667.76	2623.67
Prevalence	Global	Male	50-69 yea	Trichomon	Number	2000	3260175	4627953	2207280
Prevalence	Global	Female	50-69 yea	Trichomon	Number	2000	1.6E+07	2.3E+07	1.1E+07
Prevalence	Global	Male	50-69 yea	Trichomon	Rate	2000	814.765	1156.59	551.631
Prevalence	Global	Female	50-69 yea	Trichomon	Rate	2000	3991.58	5664.21	2623.65
Prevalence	Global	Male	50-69 yea	Trichomon	Number	2001	3367006	4790290	2275102
Prevalence	Global	Female	50-69 yea	Trichomon	Number	2001	1.7E+07	2.4E+07	1.1E+07
Prevalence	Global	Male	50-69 yea	Trichomon	Rate	2001	820.012	1166.64	554.086
Prevalence	Global	Female	50-69 yea	Trichomon	Rate	2001	4001.07	5675.97	2625.73
Prevalence	Global	Male	50-69 yea	Trichomon	Number	2002	3468921	4943389	2337536
Prevalence	Global	Female	50-69 yea	Trichomon	Number	2002	1.7E+07	2.4E+07	1.1E+07
Prevalence	Global	Male	50-69 yea	Trichomon	Rate	2002	822.611	1172.26	554.318
Prevalence	Global	Female	50-69 yea	Trichomon	Rate	2002	4003.21	5683.47	2621.35
Prevalence	Global	Male	50-69 yea	Trichomon	Number	2003	3579198	5114430	2407822
Prevalence	Global	Female	50-69 yea	Trichomon	Number	2003	1.8E+07	2.5E+07	1.2E+07
Prevalence	Global	Male	50-69 yea	Trichomon	Rate	2003	825.497	1179.58	555.334
Prevalence	Global	Female	50-69 yea	Trichomon	Rate	2003	4005.17	5692.94	2617.08
Prevalence	Global	Male	50-69 yea	Trichomon	Number	2004	3693329	5274536	2484127
Prevalence	Global	Female	50-69 yea	Trichomon	Number	2004	1.8E+07	2.6E+07	1.2E+07
Prevalence	Global	Male	50-69 yea	Trichomon	Rate	2004	827.593	1181.91	556.637
Prevalence	Global	Female	50-69 yea	Trichomon	Rate	2004	4008.19	5702.68	2611.63
Prevalence	Global	Male	50-69 yea	Trichomon	Number	2005	3823852	5465451	2570219
Prevalence	Global	Female	50-69 yea	Trichomon	Number	2005	1.9E+07	2.7E+07	1.2E+07
Prevalence	Global	Male	50-69 yea	Trichomon	Rate	2005	832.052	1189.26	559.267
Prevalence	Global	Female	50-69 yea	Trichomon	Rate	2005	4016.68	5726.68	2612.67
Prevalence	Global	Male	50-69 yea	Trichomon	Number	2006	3940921	5625114	2642440
Prevalence	Global	Female	50-69 yea	Trichomon	Number	2006	1.9E+07	2.8E+07	1.3E+07
Prevalence	Global	Male	50-69 yea	Trichomon	Rate	2006	833.513	1189.72	558.882
Prevalence	Global	Female	50-69 yea	Trichomon	Rate	2006	4017.44	5736.59	2605.94
Prevalence	Global	Male	50-69 yea	Trichomon	Number	2007	4048204	5783011	2710293
Prevalence	Global	Female	50-69 yea	Trichomon	Number	2007	2E+07	2.9E+07	1.3E+07
Prevalence	Global	Male	50-69 yea	Trichomon	Rate	2007	833.193	1190.25	557.827
Prevalence	Global	Female	50-69 yea	Trichomon	Rate	2007	4007.15	5731.8	2596.93
Prevalence	Global	Male	50-69 yea	Trichomon	Number	2008	4146358	5902427	2776851
Prevalence	Global	Female	50-69 yea	Trichomon	Number	2008	2E+07	2.9E+07	1.3E+07
Prevalence	Global	Male	50-69 yea	Trichomon	Rate	2008	831.535	1183.71	556.886
Prevalence	Global	Female	50-69 yea	Trichomon	Rate	2008	3992.73	5719.1	2583.02
Prevalence	Global	Male	50-69 yea	Trichomon	Number	2009	4236438	6009374	2839314
Prevalence	Global	Female	50-69 yea	Trichomon	Number	2009	2.1E+07	3E+07	1.4E+07
Prevalence	Global	Male	50-69 yea	Trichomon	Rate	2009	828.196	1174.79	555.068
Prevalence	Global	Female	50-69 yea	Trichomon	Rate	2009	3978.58	5702	2574.53
Prevalence	Global	Male	50-69 yea	Trichomon	Number	2010	4336441	6176511	2908124
Prevalence	Global	Female	50-69 yea	Trichomon	Number	2010	2.1E+07	3.1E+07	1.4E+07
Prevalence	Global	Male	50-69 yea	Trichomon	Rate	2010	825.875	1176.32	553.852
Prevalence	Global	Female	50-69 yea	Trichomon	Rate	2010	3974	5679.36	2570.34
Prevalence	Global	Male	50-69 yea	Trichomon	Number	2011	4438145	6323411	2977256
Prevalence	Global	Female	50-69 yea	Trichomon	Number	2011	2.2E+07	3.2E+07	1.4E+07
Prevalence	Global	Male	50-69 yea	Trichomon	Rate	2011	822.595	1172.02	551.824
Prevalence	Global	Female	50-69 yea	Trichomon	Rate	2011	3977.42	5682.78	2575.42
Prevalence	Global	Male	50-69 yea	Trichomon	Number	2012	4553822	6477773	3056010
Prevalence	Global	Female	50-69 yea	Trichomon	Number	2012	2.3E+07	3.3E+07	1.5E+07
Prevalence	Global	Male	50-69 yea	Trichomon	Rate	2012	819.955	1166.38	550.261
Prevalence	Global	Female	50-69 yea	Trichomon	Rate	2012	3986.75	5697.38	2589.52
Prevalence	Global	Male	50-69 yea	Trichomon	Number	2013	4689823	6642488	3149242
Prevalence	Global	Female	50-69 yea	Trichomon	Number	2013	2.4E+07	3.4E+07	1.5E+07
Prevalence	Global	Male	50-69 yea	Trichomon	Rate	2013	818.869	1159.82	549.875
Prevalence	Global	Female	50-69 yea	Trichomon	Rate	2013	3999.19	5728.21	2603.46
Prevalence	Global	Male	50-69 yea	Trichomon	Number	2014	4839703	6864199	3254496
Prevalence	Global	Female	50-69 yea	Trichomon	Number	2014	2.4E+07	3.5E+07	1.6E+07
Prevalence	Global	Male	50-69 yea	Trichomon	Rate	2014	818.738	1161.22	550.567
Prevalence	Global	Female	50-69 yea	Trichomon	Rate	2014	4010.32	5725.39	2616.03
Prevalence	Global	Male	50-69 yea	Trichomon	Number	2015	4999105	7112818	3365952
Prevalence	Global	Female	50-69 yea	Trichomon	Number	2015	2.5E+07	3.6E+07	1.7E+07
Prevalence	Global	Male	50-69 yea	Trichomon	Rate	2015	819.527	1166.04	551.796
Prevalence	Global	Female	50-69 yea	Trichomon	Rate	2015	4015.88	5728.35	2619.92
Prevalence	Global	Male	50-69 yea	Trichomon	Number	2016	5164560	7332154	3477448
Prevalence	Global	Female	50-69 yea	Trichomon	Number	2016	2.6E+07	3.7E+07	1.7E+07
Prevalence	Global	Male	50-69 yea	Trichomon	Rate	2016	822.542	1167.77	553.842
Prevalence	Global	Female	50-69 yea	Trichomon	Rate	2016	4013.88	5749.18	2612.17
Prevalence	Global	Male	50-69 yea	Trichomon	Number	2017	5323119	7547437	3588696
Prevalence	Global	Female	50-69 yea	Trichomon	Number	2017	2.7E+07	3.9E+07	1.7E+07
Prevalence	Global	Male	50-69 yea	Trichomon	Rate	2017	826.342	1171.64	557.096
Prevalence	Global	Female	50-69 yea	Trichomon	Rate	2017	4014.1	5784.68	2611.39
Prevalence	Global	Male	50-69 yea	Trichomon	Number	2018	5461062	7734603	3679141
Prevalence	Global	Female	50-69 yea	Trichomon	Number	2018	2.8E+07	4E+07	1.8E+07
Prevalence	Global	Male	50-69 yea	Trichomon	Rate	2018	826.701	1170.87	556.952
Prevalence	Global	Female	50-69 yea	Trichomon	Rate	2018	4014.15	5775.29	2616.32
Prevalence	Global	Male	50-69 yea	Trichomon	Number	2019	5581101	7924438	3765340
Prevalence	Global	Female	50-69 yea	Trichomon	Number	2019	2.8E+07	4E+07	1.8E+07
Prevalence	Global	Male	50-69 yea	Trichomon	Rate	2019	825.461	1172.05	556.905
Prevalence	Global	Female	50-69 yea	Trichomon	Rate	2019	4014.85	5747.25	2616.18
Incidence	Global	Male	50-69 yea	HIV/AIDS	Number	1990	66390.2	82839.8	51350.7
Incidence	Global	Female	50-69 yea	HIV/AIDS	Number	1990	67934.4	85319.4	52905.1
Incidence	Global	Male	50-69 yea	HIV/AIDS	Rate	1990	19.7275	24.6154	15.2586
Incidence	Global	Female	50-69 yea	HIV/AIDS	Rate	1990	19.6568	24.6872	15.3081
Incidence	Global	Male	50-69 yea	HIV/AIDS	Number	1991	74148.5	93126.6	56801.4
Incidence	Global	Female	50-69 yea	HIV/AIDS	Number	1991	76833.5	96883.9	59504
Incidence	Global	Male	50-69 yea	HIV/AIDS	Rate	1991	21.6287	27.1646	16.5687
Incidence	Global	Female	50-69 yea	HIV/AIDS	Rate	1991	21.8722	27.5799	16.939
Incidence	Global	Male	50-69 yea	HIV/AIDS	Number	1992	79950.9	101979	61254.3
Incidence	Global	Female	50-69 yea	HIV/AIDS	Number	1992	83716.7	106541	64404.7
Incidence	Global	Male	50-69 yea	HIV/AIDS	Rate	1992	22.9445	29.2664	17.5789
Incidence	Global	Female	50-69 yea	HIV/AIDS	Rate	1992	23.4841	29.8867	18.0667
Incidence	Global	Male	50-69 yea	HIV/AIDS	Number	1993	84573.3	108897	65586.3
Incidence	Global	Female	50-69 yea	HIV/AIDS	Number	1993	89040.1	114135	68649.6
Incidence	Global	Male	50-69 yea	HIV/AIDS	Rate	1993	23.9138	30.7915	18.545
Incidence	Global	Female	50-69 yea	HIV/AIDS	Rate	1993	24.6577	31.6073	19.011
Incidence	Global	Male	50-69 yea	HIV/AIDS	Number	1994	87338.4	113188	67810.1
Incidence	Global	Female	50-69 yea	HIV/AIDS	Number	1994	92729.8	119097	72715.9
Incidence	Global	Male	50-69 yea	HIV/AIDS	Rate	1994	24.3633	31.5742	18.9159
Incidence	Global	Female	50-69 yea	HIV/AIDS	Rate	1994	25.3651	32.5776	19.8906
Incidence	Global	Male	50-69 yea	HIV/AIDS	Number	1995	89677.2	115672	70449.7
Incidence	Global	Female	50-69 yea	HIV/AIDS	Number	1995	95341.1	121818	75314.6
Incidence	Global	Male	50-69 yea	HIV/AIDS	Rate	1995	24.7051	31.8663	19.4081
Incidence	Global	Female	50-69 yea	HIV/AIDS	Rate	1995	25.7712	32.928	20.3579
Incidence	Global	Male	50-69 yea	HIV/AIDS	Number	1996	91560.8	117868	72653.2
Incidence	Global	Female	50-69 yea	HIV/AIDS	Number	1996	97174.6	124100	77249.8
Incidence	Global	Male	50-69 yea	HIV/AIDS	Rate	1996	24.8568	31.9986	19.7238
Incidence	Global	Female	50-69 yea	HIV/AIDS	Rate	1996	25.8864	33.0592	20.5786
Incidence	Global	Male	50-69 yea	HIV/AIDS	Number	1997	92938.4	118739	74063.1
Incidence	Global	Female	50-69 yea	HIV/AIDS	Number	1997	97717	125028	77834.7
Incidence	Global	Male	50-69 yea	HIV/AIDS	Rate	1997	24.7275	31.592	19.7054
Incidence	Global	Female	50-69 yea	HIV/AIDS	Rate	1997	25.5236	32.6572	20.3304
Incidence	Global	Male	50-69 yea	HIV/AIDS	Number	1998	91703.2	117395	73426.9
Incidence	Global	Female	50-69 yea	HIV/AIDS	Number	1998	96347	123174	76831.6
Incidence	Global	Male	50-69 yea	HIV/AIDS	Rate	1998	23.9174	30.6183	19.1507
Incidence	Global	Female	50-69 yea	HIV/AIDS	Rate	1998	24.6567	31.5221	19.6624
Incidence	Global	Male	50-69 yea	HIV/AIDS	Number	1999	90491.6	115735	73348.7
Incidence	Global	Female	50-69 yea	HIV/AIDS	Number	1999	94457	120588	75750.6
Incidence	Global	Male	50-69 yea	HIV/AIDS	Rate	1999	23.0607	29.4938	18.692
Incidence	Global	Female	50-69 yea	HIV/AIDS	Rate	1999	23.6319	30.1696	18.9518
Incidence	Global	Male	50-69 yea	HIV/AIDS	Number	2000	90042.3	114015	73790.4
Incidence	Global	Female	50-69 yea	HIV/AIDS	Number	2000	92929.4	118427	74959.3
Incidence	Global	Male	50-69 yea	HIV/AIDS	Rate	2000	22.5029	28.4941	18.4413
Incidence	Global	Female	50-69 yea	HIV/AIDS	Rate	2000	22.772	29.0202	18.3685
Incidence	Global	Male	50-69 yea	HIV/AIDS	Number	2001	89120	111146	73709
Incidence	Global	Female	50-69 yea	HIV/AIDS	Number	2001	91384.2	115856	74175.9
Incidence	Global	Male	50-69 yea	HIV/AIDS	Rate	2001	21.7046	27.0689	17.9513
Incidence	Global	Female	50-69 yea	HIV/AIDS	Rate	2001	21.8033	27.6421	17.6976
Incidence	Global	Male	50-69 yea	HIV/AIDS	Number	2002	89423.2	110439	74265.7
Incidence	Global	Female	50-69 yea	HIV/AIDS	Number	2002	90631.7	113776	73467.4
Incidence	Global	Male	50-69 yea	HIV/AIDS	Rate	2002	21.2056	26.1892	17.6112
Incidence	Global	Female	50-69 yea	HIV/AIDS	Rate	2002	21.0358	26.4077	17.052
Incidence	Global	Male	50-69 yea	HIV/AIDS	Number	2003	90078.8	110155	74743
Incidence	Global	Female	50-69 yea	HIV/AIDS	Number	2003	90353.5	112544	72882.1
Incidence	Global	Male	50-69 yea	HIV/AIDS	Rate	2003	20.7755	25.4059	17.2385
Incidence	Global	Female	50-69 yea	HIV/AIDS	Rate	2003	20.3834	25.3894	16.4419
Incidence	Global	Male	50-69 yea	HIV/AIDS	Number	2004	90426.1	109433	75372.1
Incidence	Global	Female	50-69 yea	HIV/AIDS	Number	2004	89710.2	111124	72698.5
Incidence	Global	Male	50-69 yea	HIV/AIDS	Rate	2004	20.2625	24.5215	16.8892
Incidence	Global	Female	50-69 yea	HIV/AIDS	Rate	2004	19.6504	24.341	15.9241
Incidence	Global	Male	50-69 yea	HIV/AIDS	Number	2005	90496.4	109476	75198.7
Incidence	Global	Female	50-69 yea	HIV/AIDS	Number	2005	89504.1	110678	72232.2
Incidence	Global	Male	50-69 yea	HIV/AIDS	Rate	2005	19.6916	23.8215	16.3629
Incidence	Global	Female	50-69 yea	HIV/AIDS	Rate	2005	19.0292	23.5309	15.357
Incidence	Global	Male	50-69 yea	HIV/AIDS	Number	2006	90794.5	109932	74986
Incidence	Global	Female	50-69 yea	HIV/AIDS	Number	2006	89561.9	110685	72019.3
Incidence	Global	Male	50-69 yea	HIV/AIDS	Rate	2006	19.2032	23.2508	15.8597
Incidence	Global	Female	50-69 yea	HIV/AIDS	Rate	2006	18.5059	22.8706	14.8811
Incidence	Global	Male	50-69 yea	HIV/AIDS	Number	2007	90147.4	109230	74191.3
Incidence	Global	Female	50-69 yea	HIV/AIDS	Number	2007	89215.8	111201	71572.5
Incidence	Global	Male	50-69 yea	HIV/AIDS	Rate	2007	18.554	22.4814	15.2699
Incidence	Global	Female	50-69 yea	HIV/AIDS	Rate	2007	17.9374	22.3577	14.3901
Incidence	Global	Male	50-69 yea	HIV/AIDS	Number	2008	89347.2	108687	73710.8
Incidence	Global	Female	50-69 yea	HIV/AIDS	Number	2008	89057.5	111906	71641.2
Incidence	Global	Male	50-69 yea	HIV/AIDS	Rate	2008	17.9182	21.7968	14.7824
Incidence	Global	Female	50-69 yea	HIV/AIDS	Rate	2008	17.4403	21.9147	14.0297
Incidence	Global	Male	50-69 yea	HIV/AIDS	Number	2009	89252	109531	73607.2
Incidence	Global	Female	50-69 yea	HIV/AIDS	Number	2009	89478.6	112864	72778.4
Incidence	Global	Male	50-69 yea	HIV/AIDS	Rate	2009	17.4482	21.4125	14.3897
Incidence	Global	Female	50-69 yea	HIV/AIDS	Rate	2009	17.0637	21.5234	13.879
Incidence	Global	Male	50-69 yea	HIV/AIDS	Number	2010	89312.7	110693	72743.8
Incidence	Global	Female	50-69 yea	HIV/AIDS	Number	2010	90017.3	113943	73194.4
Incidence	Global	Male	50-69 yea	HIV/AIDS	Rate	2010	17.0096	21.0815	13.854
Incidence	Global	Female	50-69 yea	HIV/AIDS	Rate	2010	16.7043	21.1441	13.5825
Incidence	Global	Male	50-69 yea	HIV/AIDS	Number	2011	89509.8	112629	71532.6
Incidence	Global	Female	50-69 yea	HIV/AIDS	Number	2011	90531.9	114747	73206.6
Incidence	Global	Male	50-69 yea	HIV/AIDS	Rate	2011	16.5904	20.8754	13.2583
Incidence	Global	Female	50-69 yea	HIV/AIDS	Rate	2011	16.3244	20.6907	13.2004
Incidence	Global	Male	50-69 yea	HIV/AIDS	Number	2012	89568	113491	70068.7
Incidence	Global	Female	50-69 yea	HIV/AIDS	Number	2012	91015	115936	73101
Incidence	Global	Male	50-69 yea	HIV/AIDS	Rate	2012	16.1275	20.435	12.6165
Incidence	Global	Female	50-69 yea	HIV/AIDS	Rate	2012	15.9175	20.2759	12.7846
Incidence	Global	Male	50-69 yea	HIV/AIDS	Number	2013	90020.4	115567	68805.8
Incidence	Global	Female	50-69 yea	HIV/AIDS	Number	2013	91533.5	116437	72555.1
Incidence	Global	Male	50-69 yea	HIV/AIDS	Rate	2013	15.7181	20.1786	12.0139
Incidence	Global	Female	50-69 yea	HIV/AIDS	Rate	2013	15.4942	19.7096	12.2816
Incidence	Global	Male	50-69 yea	HIV/AIDS	Number	2014	90720.4	117596	67704.3
Incidence	Global	Female	50-69 yea	HIV/AIDS	Number	2014	92057.4	116305	71772.9
Incidence	Global	Male	50-69 yea	HIV/AIDS	Rate	2014	15.3473	19.8939	11.4536
Incidence	Global	Female	50-69 yea	HIV/AIDS	Rate	2014	15.0731	19.0433	11.7518
Incidence	Global	Male	50-69 yea	HIV/AIDS	Number	2015	92004.8	121374	66747.6
Incidence	Global	Female	50-69 yea	HIV/AIDS	Number	2015	92211.8	116211	71261.3
Incidence	Global	Male	50-69 yea	HIV/AIDS	Rate	2015	15.0828	19.8974	10.9423
Incidence	Global	Female	50-69 yea	HIV/AIDS	Rate	2015	14.6078	18.4098	11.2889
Incidence	Global	Male	50-69 yea	HIV/AIDS	Number	2016	92526.2	122301	66795.2
Incidence	Global	Female	50-69 yea	HIV/AIDS	Number	2016	91410	114574	70392.2
Incidence	Global	Male	50-69 yea	HIV/AIDS	Rate	2016	14.7363	19.4785	10.6383
Incidence	Global	Female	50-69 yea	HIV/AIDS	Rate	2016	14.0453	17.6045	10.8158
Incidence	Global	Male	50-69 yea	HIV/AIDS	Number	2017	92107.5	122059	66164
Incidence	Global	Female	50-69 yea	HIV/AIDS	Number	2017	89663.3	112163	68825
Incidence	Global	Male	50-69 yea	HIV/AIDS	Rate	2017	14.2984	18.948	10.2711
Incidence	Global	Female	50-69 yea	HIV/AIDS	Rate	2017	13.4088	16.7736	10.2925
Incidence	Global	Male	50-69 yea	HIV/AIDS	Number	2018	90862	120530	64972.4
Incidence	Global	Female	50-69 yea	HIV/AIDS	Number	2018	86136.9	107919	65311.2
Incidence	Global	Male	50-69 yea	HIV/AIDS	Rate	2018	13.7548	18.2459	9.83558
Incidence	Global	Female	50-69 yea	HIV/AIDS	Rate	2018	12.5523	15.7266	9.51748
Incidence	Global	Male	50-69 yea	HIV/AIDS	Number	2019	90293.5	119489	64452.2
Incidence	Global	Female	50-69 yea	HIV/AIDS	Number	2019	84217.9	105993	63361.1
Incidence	Global	Male	50-69 yea	HIV/AIDS	Rate	2019	13.3547	17.6728	9.53267
Incidence	Global	Female	50-69 yea	HIV/AIDS	Rate	2019	11.9827	15.0809	9.01516
Incidence	Global	Male	50-69 yea	Chlamydia	Number	1990	3345085	5233779	1950567
Incidence	Global	Female	50-69 yea	Chlamydia	Number	1990	3509822	5348507	2189077
Incidence	Global	Male	50-69 yea	Chlamydia	Rate	1990	993.975	1555.19	579.601
Incidence	Global	Female	50-69 yea	Chlamydia	Rate	1990	1015.57	1547.59	633.41
Incidence	Global	Male	50-69 yea	Chlamydia	Number	1991	3371245	5264135	1970583
Incidence	Global	Female	50-69 yea	Chlamydia	Number	1991	3545635	5404206	2210749
Incidence	Global	Male	50-69 yea	Chlamydia	Rate	1991	983.375	1535.52	574.809
Incidence	Global	Female	50-69 yea	Chlamydia	Rate	1991	1009.34	1538.42	629.334
Incidence	Global	Male	50-69 yea	Chlamydia	Number	1992	3391833	5296097	1987234
Incidence	Global	Female	50-69 yea	Chlamydia	Number	1992	3572912	5447358	2230583
Incidence	Global	Male	50-69 yea	Chlamydia	Rate	1992	973.398	1519.89	570.302
Incidence	Global	Female	50-69 yea	Chlamydia	Rate	1992	1002.27	1528.08	625.72
Incidence	Global	Male	50-69 yea	Chlamydia	Number	1993	3409414	5323204	2001862
Incidence	Global	Female	50-69 yea	Chlamydia	Number	1993	3580730	5445544	2240779
Incidence	Global	Male	50-69 yea	Chlamydia	Rate	1993	964.038	1505.18	566.042
Incidence	Global	Female	50-69 yea	Chlamydia	Rate	1993	991.605	1508.02	620.535
Incidence	Global	Male	50-69 yea	Chlamydia	Number	1994	3426488	5349248	2015784
Incidence	Global	Female	50-69 yea	Chlamydia	Number	1994	3590058	5462092	2254207
Incidence	Global	Male	50-69 yea	Chlamydia	Rate	1994	955.831	1492.19	562.31
Incidence	Global	Female	50-69 yea	Chlamydia	Rate	1994	982.018	1494.09	616.612
Incidence	Global	Male	50-69 yea	Chlamydia	Number	1995	3452701	5389831	2034164
Incidence	Global	Female	50-69 yea	Chlamydia	Number	1995	3607563	5479669	2266511
Incidence	Global	Male	50-69 yea	Chlamydia	Rate	1995	951.182	1484.84	560.39
Incidence	Global	Female	50-69 yea	Chlamydia	Rate	1995	975.142	1481.18	612.649
Incidence	Global	Male	50-69 yea	Chlamydia	Number	1996	3519853	5497285	2070971
Incidence	Global	Female	50-69 yea	Chlamydia	Number	1996	3676143	5587963	2309766
Incidence	Global	Male	50-69 yea	Chlamydia	Rate	1996	955.566	1492.4	562.225
Incidence	Global	Female	50-69 yea	Chlamydia	Rate	1996	979.29	1488.58	615.3
Incidence	Global	Male	50-69 yea	Chlamydia	Number	1997	3645545	5696421	2137198
Incidence	Global	Female	50-69 yea	Chlamydia	Number	1997	3800280	5780119	2384606
Incidence	Global	Male	50-69 yea	Chlamydia	Rate	1997	969.944	1515.61	568.629
Incidence	Global	Female	50-69 yea	Chlamydia	Rate	1997	992.632	1509.77	622.858
Incidence	Global	Male	50-69 yea	Chlamydia	Number	1998	3785385	5918864	2209538
Incidence	Global	Female	50-69 yea	Chlamydia	Number	1998	3957199	6016642	2467480
Incidence	Global	Male	50-69 yea	Chlamydia	Rate	1998	987.28	1543.72	576.278
Incidence	Global	Female	50-69 yea	Chlamydia	Rate	1998	1012.71	1539.75	631.468
Incidence	Global	Male	50-69 yea	Chlamydia	Number	1999	3979143	6251526	2309369
Incidence	Global	Female	50-69 yea	Chlamydia	Number	1999	4160649	6326561	2578873
Incidence	Global	Male	50-69 yea	Chlamydia	Rate	1999	1014.04	1593.13	588.515
Incidence	Global	Female	50-69 yea	Chlamydia	Rate	1999	1040.94	1582.82	645.2
Incidence	Global	Male	50-69 yea	Chlamydia	Number	2000	4112658	6471511	2383849
Incidence	Global	Female	50-69 yea	Chlamydia	Number	2000	4318175	6593011	2667695
Incidence	Global	Male	50-69 yea	Chlamydia	Rate	2000	1027.81	1617.32	595.758
Incidence	Global	Female	50-69 yea	Chlamydia	Rate	2000	1058.15	1615.59	653.708
Incidence	Global	Male	50-69 yea	Chlamydia	Number	2001	4295249	6757341	2489674
Incidence	Global	Female	50-69 yea	Chlamydia	Number	2001	4512356	6908246	2772219
Incidence	Global	Male	50-69 yea	Chlamydia	Rate	2001	1046.08	1645.71	606.343
Incidence	Global	Female	50-69 yea	Chlamydia	Rate	2001	1076.6	1648.24	661.423
Incidence	Global	Male	50-69 yea	Chlamydia	Number	2002	4457221	7004440	2583518
Incidence	Global	Female	50-69 yea	Chlamydia	Number	2002	4696734	7207511	2874589
Incidence	Global	Male	50-69 yea	Chlamydia	Rate	2002	1056.97	1661.02	612.649
Incidence	Global	Female	50-69 yea	Chlamydia	Rate	2002	1090.12	1672.88	667.2
Incidence	Global	Male	50-69 yea	Chlamydia	Number	2003	4637339	7295132	2687200
Incidence	Global	Female	50-69 yea	Chlamydia	Number	2003	4905660	7548676	2993241
Incidence	Global	Male	50-69 yea	Chlamydia	Rate	2003	1069.54	1682.53	619.769
Incidence	Global	Female	50-69 yea	Chlamydia	Rate	2003	1106.7	1702.95	675.263
Incidence	Global	Male	50-69 yea	Chlamydia	Number	2004	4802860	7531194	2781365
Incidence	Global	Female	50-69 yea	Chlamydia	Number	2004	5112804	7872377	3121318
Incidence	Global	Male	50-69 yea	Chlamydia	Rate	2004	1076.21	1687.57	623.242
Incidence	Global	Female	50-69 yea	Chlamydia	Rate	2004	1119.92	1724.39	683.703
Incidence	Global	Male	50-69 yea	Chlamydia	Number	2005	5012180	7865002	2900160
Incidence	Global	Female	50-69 yea	Chlamydia	Number	2005	5347504	8243674	3267436
Incidence	Global	Male	50-69 yea	Chlamydia	Rate	2005	1090.63	1711.39	631.061
Incidence	Global	Female	50-69 yea	Chlamydia	Rate	2005	1136.91	1752.66	694.679
Incidence	Global	Male	50-69 yea	Chlamydia	Number	2006	5135659	8049096	2971521
Incidence	Global	Female	50-69 yea	Chlamydia	Number	2006	5474419	8411967	3348219
Incidence	Global	Male	50-69 yea	Chlamydia	Rate	2006	1086.2	1702.4	628.483
Incidence	Global	Female	50-69 yea	Chlamydia	Rate	2006	1131.16	1738.14	691.833
Incidence	Global	Male	50-69 yea	Chlamydia	Number	2007	5197397	8147292	3008799
Incidence	Global	Female	50-69 yea	Chlamydia	Number	2007	5503678	8427488	3385045
Incidence	Global	Male	50-69 yea	Chlamydia	Rate	2007	1069.72	1676.86	619.265
Incidence	Global	Female	50-69 yea	Chlamydia	Rate	2007	1106.55	1694.4	680.584
Incidence	Global	Male	50-69 yea	Chlamydia	Number	2008	5215519	8181180	3028531
Incidence	Global	Female	50-69 yea	Chlamydia	Number	2008	5476904	8354423	3394192
Incidence	Global	Male	50-69 yea	Chlamydia	Rate	2008	1045.95	1640.7	607.36
Incidence	Global	Female	50-69 yea	Chlamydia	Rate	2008	1072.55	1636.06	664.692
Incidence	Global	Male	50-69 yea	Chlamydia	Number	2009	5221096	8188111	3048776
Incidence	Global	Female	50-69 yea	Chlamydia	Number	2009	5456269	8320074	3412115
Incidence	Global	Male	50-69 yea	Chlamydia	Rate	2009	1020.69	1600.72	596.016
Incidence	Global	Female	50-69 yea	Chlamydia	Rate	2009	1040.52	1586.65	650.696
Incidence	Global	Male	50-69 yea	Chlamydia	Number	2010	5277919	8270512	3095548
Incidence	Global	Female	50-69 yea	Chlamydia	Number	2010	5519137	8386115	3478874
Incidence	Global	Male	50-69 yea	Chlamydia	Rate	2010	1005.18	1575.12	589.547
Incidence	Global	Female	50-69 yea	Chlamydia	Rate	2010	1024.17	1556.19	645.567
Incidence	Global	Male	50-69 yea	Chlamydia	Number	2011	5372853	8400926	3152934
Incidence	Global	Female	50-69 yea	Chlamydia	Number	2011	5643089	8557651	3556109
Incidence	Global	Male	50-69 yea	Chlamydia	Rate	2011	995.84	1557.08	584.386
Incidence	Global	Female	50-69 yea	Chlamydia	Rate	2011	1017.54	1543.09	641.225
Incidence	Global	Male	50-69 yea	Chlamydia	Number	2012	5512269	8611005	3236620
Incidence	Global	Female	50-69 yea	Chlamydia	Number	2012	5808038	8826335	3651612
Incidence	Global	Male	50-69 yea	Chlamydia	Rate	2012	992.531	1550.49	582.781

Incidence	Global	Male	50-69 yea	Trichomon	Rate	1991	5774.85	8043.33	4126.77
Incidence	Global	Female	50-69 yea	Trichomon	Rate	1991	3449.69	4753.04	2457.98
Incidence	Global	Male	50-69 yea	Trichomon	Number	1992	2E+07	2.8E+07	1.4E+07
Incidence	Global	Female	50-69 yea	Trichomon	Number	1992	1.2E+07	1.7E+07	8765968
Incidence	Global	Male	50-69 yea	Trichomon	Rate	1992	5764.91	8022.68	4125.43
Incidence	Global	Female	50-69 yea	Trichomon	Rate	1992	3448.19	4751.67	2459.02
Incidence	Global	Male	50-69 yea	Trichomon	Number	1993	2E+07	2.8E+07	1.5E+07
Incidence	Global	Female	50-69 yea	Trichomon	Number	1993	1.2E+07	1.7E+07	8875792
Incidence	Global	Male	50-69 yea	Trichomon	Rate	1993	5756.34	8002.81	4117.7
Incidence	Global	Female	50-69 yea	Trichomon	Rate	1993	3446.54	4749.12	2457.96
Incidence	Global	Male	50-69 yea	Trichomon	Number	1994	2.1E+07	2.9E+07	1.5E+07
Incidence	Global	Female	50-69 yea	Trichomon	Number	1994	1.3E+07	1.7E+07	8982232
Incidence	Global	Male	50-69 yea	Trichomon	Rate	1994	5750.76	7987.19	4111.73
Incidence	Global	Female	50-69 yea	Trichomon	Rate	1994	3446.89	4749.59	2456.98
Incidence	Global	Male	50-69 yea	Trichomon	Number	1995	2.1E+07	2.9E+07	1.5E+07
Incidence	Global	Female	50-69 yea	Trichomon	Number	1995	1.3E+07	1.8E+07	9084709
Incidence	Global	Male	50-69 yea	Trichomon	Rate	1995	5749.75	7979.79	4108.67
Incidence	Global	Female	50-69 yea	Trichomon	Rate	1995	3446.36	4748.05	2455.64
Incidence	Global	Male	50-69 yea	Trichomon	Number	1996	2.1E+07	2.9E+07	1.5E+07
Incidence	Global	Female	50-69 yea	Trichomon	Number	1996	1.3E+07	1.8E+07	9231337
Incidence	Global	Male	50-69 yea	Trichomon	Rate	1996	5759.97	7998.1	4111.08
Incidence	Global	Female	50-69 yea	Trichomon	Rate	1996	3446.66	4744.57	2459.14
Incidence	Global	Male	50-69 yea	Trichomon	Number	1997	2.2E+07	3E+07	1.5E+07
Incidence	Global	Female	50-69 yea	Trichomon	Number	1997	1.3E+07	1.8E+07	9425643
Incidence	Global	Male	50-69 yea	Trichomon	Rate	1997	5782.95	8038.69	4122.97
Incidence	Global	Female	50-69 yea	Trichomon	Rate	1997	3449.65	4746.95	2461.97
Incidence	Global	Male	50-69 yea	Trichomon	Number	1998	2.2E+07	3.1E+07	1.6E+07
Incidence	Global	Female	50-69 yea	Trichomon	Number	1998	1.3E+07	1.9E+07	9631339
Incidence	Global	Male	50-69 yea	Trichomon	Rate	1998	5808.29	8086.42	4136
Incidence	Global	Female	50-69 yea	Trichomon	Rate	1998	3452.62	4743.61	2464.81
Incidence	Global	Male	50-69 yea	Trichomon	Number	1999	2.3E+07	3.2E+07	1.6E+07
Incidence	Global	Female	50-69 yea	Trichomon	Number	1999	1.4E+07	1.9E+07	9861089
Incidence	Global	Male	50-69 yea	Trichomon	Rate	1999	5847.17	8159.26	4158.55
Incidence	Global	Female	50-69 yea	Trichomon	Rate	1999	3460.52	4758.7	2467.11
Incidence	Global	Male	50-69 yea	Trichomon	Number	2000	2.3E+07	3.3E+07	1.7E+07
Incidence	Global	Female	50-69 yea	Trichomon	Number	2000	1.4E+07	1.9E+07	1E+07
Incidence	Global	Male	50-69 yea	Trichomon	Rate	2000	5867.99	8199.52	4167.67
Incidence	Global	Female	50-69 yea	Trichomon	Rate	2000	3471.15	4777.7	2469.61
Incidence	Global	Male	50-69 yea	Trichomon	Number	2001	2.4E+07	3.4E+07	1.7E+07
Incidence	Global	Female	50-69 yea	Trichomon	Number	2001	1.5E+07	2E+07	1E+07
Incidence	Global	Male	50-69 yea	Trichomon	Rate	2001	5901.82	8254.13	4190.12
Incidence	Global	Female	50-69 yea	Trichomon	Rate	2001	3493.07	4811.74	2478.49
Incidence	Global	Male	50-69 yea	Trichomon	Number	2002	2.5E+07	3.5E+07	1.8E+07
Incidence	Global	Female	50-69 yea	Trichomon	Number	2002	1.5E+07	2.1E+07	1.1E+07
Incidence	Global	Male	50-69 yea	Trichomon	Rate	2002	5919.53	8282.28	4200.17
Incidence	Global	Female	50-69 yea	Trichomon	Rate	2002	3507.41	4837.92	2485.47
Incidence	Global	Male	50-69 yea	Trichomon	Number	2003	2.6E+07	3.6E+07	1.8E+07
Incidence	Global	Female	50-69 yea	Trichomon	Number	2003	1.6E+07	2.2E+07	1.1E+07
Incidence	Global	Male	50-69 yea	Trichomon	Rate	2003	5939.12	8313.26	4209.56
Incidence	Global	Female	50-69 yea	Trichomon	Rate	2003	3523.55	4867.1	2492.97
Incidence	Global	Male	50-69 yea	Trichomon	Number	2004	2.7E+07	3.7E+07	1.9E+07
Incidence	Global	Female	50-69 yea	Trichomon	Number	2004	1.6E+07	2.2E+07	1.1E+07
Incidence	Global	Male	50-69 yea	Trichomon	Rate	2004	5953.03	8334.07	4220.41
Incidence	Global	Female	50-69 yea	Trichomon	Rate	2004	3538.83	4895.33	2498
Incidence	Global	Male	50-69 yea	Trichomon	Number	2005	2.7E+07	3.8E+07	1.9E+07
Incidence	Global	Female	50-69 yea	Trichomon	Number	2005	1.7E+07	2.3E+07	1.2E+07
Incidence	Global	Male	50-69 yea	Trichomon	Rate	2005	5980.8	8373.97	4241.9
Incidence	Global	Female	50-69 yea	Trichomon	Rate	2005	3558.63	4927.42	2508.49
Incidence	Global	Male	50-69 yea	Trichomon	Number	2006	2.8E+07	4E+07	2E+07
Incidence	Global	Female	50-69 yea	Trichomon	Number	2006	1.7E+07	2.4E+07	1.2E+07
Incidence	Global	Male	50-69 yea	Trichomon	Rate	2006	5987.06	8378.23	4235.03
Incidence	Global	Female	50-69 yea	Trichomon	Rate	2006	3570.66	4940.16	2514.43
Incidence	Global	Male	50-69 yea	Trichomon	Number	2007	2.9E+07	4.1E+07	2.1E+07
Incidence	Global	Female	50-69 yea	Trichomon	Number	2007	1.8E+07	2.5E+07	1.3E+07
Incidence	Global	Male	50-69 yea	Trichomon	Rate	2007	5979.98	8370.5	4221.45
Incidence	Global	Female	50-69 yea	Trichomon	Rate	2007	3581.16	4958.56	2521.1
Incidence	Global	Male	50-69 yea	Trichomon	Number	2008	3E+07	4.2E+07	2.1E+07
Incidence	Global	Female	50-69 yea	Trichomon	Number	2008	1.8E+07	2.5E+07	1.3E+07
Incidence	Global	Male	50-69 yea	Trichomon	Rate	2008	5963.48	8352.86	4209.85
Incidence	Global	Female	50-69 yea	Trichomon	Rate	2008	3589.5	4972.9	2526.22
Incidence	Global	Male	50-69 yea	Trichomon	Number	2009	3E+07	4.3E+07	2.1E+07
Incidence	Global	Female	50-69 yea	Trichomon	Number	2009	1.9E+07	2.6E+07	1.3E+07
Incidence	Global	Male	50-69 yea	Trichomon	Rate	2009	5937.61	8326.98	4198.28
Incidence	Global	Female	50-69 yea	Trichomon	Rate	2009	3592.15	4977.3	2532.75
Incidence	Global	Male	50-69 yea	Trichomon	Number	2010	3.1E+07	4.4E+07	2.2E+07
Incidence	Global	Female	50-69 yea	Trichomon	Number	2010	1.9E+07	2.7E+07	1.4E+07
Incidence	Global	Male	50-69 yea	Trichomon	Rate	2010	5920.4	8307.98	4190.53
Incidence	Global	Female	50-69 yea	Trichomon	Rate	2010	3593.04	4980.09	2538.51
Incidence	Global	Male	50-69 yea	Trichomon	Number	2011	3.2E+07	4.5E+07	2.3E+07
Incidence	Global	Female	50-69 yea	Trichomon	Number	2011	2E+07	2.7E+07	1.4E+07
Incidence	Global	Male	50-69 yea	Trichomon	Rate	2011	5900.9	8275.41	4181.01
Incidence	Global	Female	50-69 yea	Trichomon	Rate	2011	3583.22	4957.66	2535.58
Incidence	Global	Male	50-69 yea	Trichomon	Number	2012	3.3E+07	4.6E+07	2.3E+07
Incidence	Global	Female	50-69 yea	Trichomon	Number	2012	2E+07	2.8E+07	1.4E+07
Incidence	Global	Male	50-69 yea	Trichomon	Rate	2012	5887.05	8251.78	4176.63
Incidence	Global	Female	50-69 yea	Trichomon	Rate	2012	3566.85	4928.5	2524.35
Incidence	Global	Male	50-69 yea	Trichomon	Number	2013	3.4E+07	4.7E+07	2.4E+07
Incidence	Global	Female	50-69 yea	Trichomon	Number	2013	2.1E+07	2.9E+07	1.5E+07
Incidence	Global	Male	50-69 yea	Trichomon	Rate	2013	5883.69	8246.97	4181.94
Incidence	Global	Female	50-69 yea	Trichomon	Rate	2013	3549.79	4896.91	2511.14
Incidence	Global	Male	50-69 yea	Trichomon	Number	2014	3.5E+07	4.9E+07	2.5E+07
Incidence	Global	Female	50-69 yea	Trichomon	Number	2014	2.2E+07	3E+07	1.5E+07
Incidence	Global	Male	50-69 yea	Trichomon	Rate	2014	5885.51	8240.33	4187.86
Incidence	Global	Female	50-69 yea	Trichomon	Rate	2014	3535.8	4870	2499.03
Incidence	Global	Male	50-69 yea	Trichomon	Number	2015	3.6E+07	5E+07	2.6E+07
Incidence	Global	Female	50-69 yea	Trichomon	Number	2015	2.2E+07	3.1E+07	1.6E+07
Incidence	Global	Male	50-69 yea	Trichomon	Rate	2015	5891.14	8230.18	4186.07
Incidence	Global	Female	50-69 yea	Trichomon	Rate	2015	3528.73	4854.34	2491.36
Incidence	Global	Male	50-69 yea	Trichomon	Number	2016	3.7E+07	5.2E+07	2.6E+07
Incidence	Global	Female	50-69 yea	Trichomon	Number	2016	2.3E+07	3.2E+07	1.6E+07
Incidence	Global	Male	50-69 yea	Trichomon	Rate	2016	5895.92	8233.54	4194.87
Incidence	Global	Female	50-69 yea	Trichomon	Rate	2016	3577.46	4945.79	2510.13
Incidence	Global	Male	50-69 yea	Trichomon	Number	2017	3.8E+07	5.3E+07	2.7E+07
Incidence	Global	Female	50-69 yea	Trichomon	Number	2017	2.4E+07	3.4E+07	1.7E+07
Incidence	Global	Male	50-69 yea	Trichomon	Rate	2017	5904.87	8229.39	4191.45
Incidence	Global	Female	50-69 yea	Trichomon	Rate	2017	3633.51	5012.48	2547.41
Incidence	Global	Male	50-69 yea	Trichomon	Number	2018	3.9E+07	5.4E+07	2.8E+07
Incidence	Global	Female	50-69 yea	Trichomon	Number	2018	2.5E+07	3.4E+07	1.8E+07
Incidence	Global	Male	50-69 yea	Trichomon	Rate	2018	5904.46	8219.31	4204.07
Incidence	Global	Female	50-69 yea	Trichomon	Rate	2018	3640.07	5022.58	2550.81
Incidence	Global	Male	50-69 yea	Trichomon	Number	2019	4E+07	5.6E+07	2.8E+07
Incidence	Global	Female	50-69 yea	Trichomon	Number	2019	2.6E+07	3.5E+07	1.8E+07
Incidence	Global	Male	50-69 yea	Trichomon	Rate	2019	5896.97	8229.06	4186.98
Incidence	Global	Female	50-69 yea	Trichomon	Rate	2019	3641.34	5019.52	2546.02
Incidence	Global	Male	50-69 yea	Other sexu	Number	2019	0	0	0
Incidence	Global	Female	50-69 yea	Other sexu	Number	2019	0	0	0
Incidence	Global	Male	50-69 yea	Other sexu	Rate	2019	0	0	0
Incidence	Global	Female	50-69 yea	Other sexu	Rate	2019	0	0	0
Incidence	Global	Male	50-69 yea	Sexually tr	Number	1990	2.6E+07	3.3E+07	2E+07
Incidence	Global	Female	50-69 yea	Sexually tr	Number	1990	1.8E+07	2.2E+07	1.4E+07
Incidence	Global	Male	50-69 yea	Sexually tr	Rate	1990	7672	9937.84	5923.31
Incidence	Global	Female	50-69 yea	Sexually tr	Rate	1990	5107.8	6452.47	3990.83
Incidence	Global	Male	50-69 yea	Sexually tr	Number	1991	2.6E+07	3.4E+07	2E+07
Incidence	Global	Female	50-69 yea	Sexually tr	Number	1991	1.8E+07	2.3E+07	1.4E+07
Incidence	Global	Male	50-69 yea	Sexually tr	Rate	1991	7647.85	9893.25	5914.28
Incidence	Global	Female	50-69 yea	Sexually tr	Rate	1991	5096.48	6436.14	3981.31
Incidence	Global	Male	50-69 yea	Sexually tr	Number	1992	2.7E+07	3.4E+07	2.1E+07
Incidence	Global	Female	50-69 yea	Sexually tr	Number	1992	1.8E+07	2.3E+07	1.4E+07
Incidence	Global	Male	50-69 yea	Sexually tr	Rate	1992	7624.65	9856.64	5898.52
Incidence	Global	Female	50-69 yea	Sexually tr	Rate	1992	5085.42	6428.89	3971.5
Incidence	Global	Male	50-69 yea	Sexually tr	Number	1993	2.7E+07	3.5E+07	2.1E+07
Incidence	Global	Female	50-69 yea	Sexually tr	Number	1993	1.8E+07	2.3E+07	1.4E+07
Incidence	Global	Male	50-69 yea	Sexually tr	Rate	1993	7601.71	9826	5880.16
Incidence	Global	Female	50-69 yea	Sexually tr	Rate	1993	5069.51	6414.84	3957.78
Incidence	Global	Male	50-69 yea	Sexually tr	Number	1994	2.7E+07	3.5E+07	2.1E+07
Incidence	Global	Female	50-69 yea	Sexually tr	Number	1994	1.8E+07	2.3E+07	1.4E+07
Incidence	Global	Male	50-69 yea	Sexually tr	Rate	1994	7582.73	9803.91	5871.31
Incidence	Global	Female	50-69 yea	Sexually tr	Rate	1994	5056.91	6404.32	3950.15
Incidence	Global	Male	50-69 yea	Sexually tr	Number	1995	2.7E+07	3.6E+07	2.1E+07
Incidence	Global	Female	50-69 yea	Sexually tr	Number	1995	1.9E+07	2.4E+07	1.5E+07
Incidence	Global	Male	50-69 yea	Sexually tr	Rate	1995	7572.23	9792.91	5865.52
Incidence	Global	Female	50-69 yea	Sexually tr	Rate	1995	5046.11	6388.59	3940.93
Incidence	Global	Male	50-69 yea	Sexually tr	Number	1996	2.8E+07	3.6E+07	2.2E+07
Incidence	Global	Female	50-69 yea	Sexually tr	Number	1996	1.9E+07	2.4E+07	1.5E+07
Incidence	Global	Male	50-69 yea	Sexually tr	Rate	1996	7582.87	9809.39	5873.29
Incidence	Global	Female	50-69 yea	Sexually tr	Rate	1996	5048.95	6395.88	3943.18
Incidence	Global	Male	50-69 yea	Sexually tr	Number	1997	2.9E+07	3.7E+07	2.2E+07
Incidence	Global	Female	50-69 yea	Sexually tr	Number	1997	1.9E+07	2.5E+07	1.5E+07
Incidence	Global	Male	50-69 yea	Sexually tr	Rate	1997	7618.04	9853.17	5888.74
Incidence	Global	Female	50-69 yea	Sexually tr	Rate	1997	5067.24	6414.25	3958.95
Incidence	Global	Male	50-69 yea	Sexually tr	Number	1998	2.9E+07	3.8E+07	2.3E+07
Incidence	Global	Female	50-69 yea	Sexually tr	Number	1998	2E+07	2.5E+07	1.6E+07
Incidence	Global	Male	50-69 yea	Sexually tr	Rate	1998	7659.23	9909.1	5911.21
Incidence	Global	Female	50-69 yea	Sexually tr	Rate	1998	5093.52	6448.93	3981.41
Incidence	Global	Male	50-69 yea	Sexually tr	Number	1999	3E+07	3.9E+07	2.3E+07
Incidence	Global	Female	50-69 yea	Sexually tr	Number	1999	2.1E+07	2.6E+07	1.6E+07
Incidence	Global	Male	50-69 yea	Sexually tr	Rate	1999	7726.9	10000.1	5947.49
Incidence	Global	Female	50-69 yea	Sexually tr	Rate	1999	5135.29	6496.91	4011.21
Incidence	Global	Male	50-69 yea	Sexually tr	Number	2000	3.1E+07	4E+07	2.4E+07
Incidence	Global	Female	50-69 yea	Sexually tr	Number	2000	2.1E+07	2.7E+07	1.6E+07
Incidence	Global	Male	50-69 yea	Sexually tr	Rate	2000	7763.66	10053.6	5965.24
Incidence	Global	Female	50-69 yea	Sexually tr	Rate	2000	5166.72	6532.57	4034.53
Incidence	Global	Male	50-69 yea	Sexually tr	Number	2001	3.2E+07	4.2E+07	2.5E+07
Incidence	Global	Female	50-69 yea	Sexually tr	Number	2001	2.2E+07	2.8E+07	1.7E+07
Incidence	Global	Male	50-69 yea	Sexually tr	Rate	2001	7824.05	10122.8	6020.73
Incidence	Global	Female	50-69 yea	Sexually tr	Rate	2001	5212.07	6583.1	4072.04
Incidence	Global	Male	50-69 yea	Sexually tr	Number	2002	3.3E+07	4.3E+07	2.6E+07
Incidence	Global	Female	50-69 yea	Sexually tr	Number	2002	2.3E+07	2.9E+07	1.8E+07
Incidence	Global	Male	50-69 yea	Sexually tr	Rate	2002	7858.16	10155.2	6050.23
Incidence	Global	Female	50-69 yea	Sexually tr	Rate	2002	5242.65	6616.29	4098.48
Incidence	Global	Male	50-69 yea	Sexually tr	Number	2003	3.4E+07	4.4E+07	2.6E+07
Incidence	Global	Female	50-69 yea	Sexually tr	Number	2003	2.3E+07	3E+07	1.8E+07
Incidence	Global	Male	50-69 yea	Sexually tr	Rate	2003	7896.36	10194.7	6082.84
Incidence	Global	Female	50-69 yea	Sexually tr	Rate	2003	5279	6665.7	4122.89
Incidence	Global	Male	50-69 yea	Sexually tr	Number	2004	3.5E+07	4.6E+07	2.7E+07
Incidence	Global	Female	50-69 yea	Sexually tr	Number	2004	2.4E+07	3.1E+07	1.9E+07
Incidence	Global	Male	50-69 yea	Sexually tr	Rate	2004	7921.69	10249.7	6105.28
Incidence	Global	Female	50-69 yea	Sexually tr	Rate	2004	5310.57	6712.31	4142.42
Incidence	Global	Male	50-69 yea	Sexually tr	Number	2005	3.7E+07	4.7E+07	2.8E+07
Incidence	Global	Female	50-69 yea	Sexually tr	Number	2005	2.5E+07	3.2E+07	2E+07
Incidence	Global	Male	50-69 yea	Sexually tr	Rate	2005	7970.94	10326.3	6150.36
Incidence	Global	Female	50-69 yea	Sexually tr	Rate	2005	5351.71	6770.96	4165.9
Incidence	Global	Male	50-69 yea	Sexually tr	Number	2006	3.8E+07	4.9E+07	2.9E+07
Incidence	Global	Female	50-69 yea	Sexually tr	Number	2006	2.6E+07	3.3E+07	2E+07
Incidence	Global	Male	50-69 yea	Sexually tr	Rate	2006	7974.31	10349	6143.33
Incidence	Global	Female	50-69 yea	Sexually tr	Rate	2006	5357.39	6788.62	4174.24
Incidence	Global	Male	50-69 yea	Sexually tr	Number	2007	3.9E+07	5E+07	3E+07
Incidence	Global	Female	50-69 yea	Sexually tr	Number	2007	2.7E+07	3.4E+07	2.1E+07
Incidence	Global	Male	50-69 yea	Sexually tr	Rate	2007	7949.68	10318.8	6113.88
Incidence	Global	Female	50-69 yea	Sexually tr	Rate	2007	5338.57	6773.6	4158.63
Incidence	Global	Male	50-69 yea	Sexually tr	Number	2008	3.9E+07	5.1E+07	3E+07
Incidence	Global	Female	50-69 yea	Sexually tr	Number	2008	2.7E+07	3.4E+07	2.1E+07
Incidence	Global	Male	50-69 yea	Sexually tr	Rate	2008	7907.15	10273.6	6083.12
Incidence	Global	Female	50-69 yea	Sexually tr	Rate	2008	5306.17	6742.73	4120.72
Incidence	Global	Male	50-69 yea	Sexually tr	Number	2009	4E+07	5.2E+07	3.1E+07
Incidence	Global	Female	50-69 yea	Sexually tr	Number	2009	2.8E+07	3.5E+07	2.1E+07
Incidence	Global	Male	50-69 yea	Sexually tr	Rate	2009	7852.11	10197.9	6044.85
Incidence	Global	Female	50-69 yea	Sexually tr	Rate	2009	5269.26	6704.55	4081.7
Incidence	Global	Male	50-69 yea	Sexually tr	Number	2010	4.1E+07	5.3E+07	3.2E+07
Incidence	Global	Female	50-69 yea	Sexually tr	Number	2010	2.8E+07	3.6E+07	2.2E+07
Incidence	Global	Male	50-69 yea	Sexually tr	Rate	2010	7817.3	10137.5	6023.6
Incidence	Global	Female	50-69 yea	Sexually tr	Rate	2010	5248.96	6690.97	4060.52
Incidence	Global	Male	50-69 yea	Sexually tr	Number	2011	4.2E+07	5.4E+07	3.2E+07
Incidence	Global	Female	50-69 yea	Sexually tr	Number	2011	2.9E+07	3.7E+07	2.2E+07
Incidence	Global	Male	50-69 yea	Sexually tr	Rate	2011	7784.58	10084.5	6003.02
Incidence	Global	Female	50-69 yea	Sexually tr	Rate	2011	5227.98	6663.92	4044.42
Incidence	Global	Male	50-69 yea	Sexually tr	Number	2012	4.3E+07	5.6E+07	3.3E+07
Incidence	Global	Female	50-69 yea	Sexually tr	Number	2012	3E+07	3.8E+07	2.3E+07
Incidence	Global	Male	50-69 yea	Sexually tr	Rate	2012	7763.69	10054.7	5990.82
Incidence	Global	Female	50-69 yea	Sexually tr	Rate	2012	5205.74	6628.16	4030.17
Incidence	Global	Male	50-69 yea	Sexually tr	Number	2013	4.4E+07	5.8E+07	3.4E+07
Incidence	Global	Female	50-69 yea	Sexually tr	Number	2013	3.1E+07	3.9E+07	2.4E+07
Incidence	Global	Male	50-69 yea	Sexually tr	Rate	2013	7761.2	10052.2	5988.33
Incidence	Global	Female	50-69 yea	Sexually tr	Rate	2013	5188.69	6597.42	4019.28
Incidence	Global	Male	50-69 yea	Sexually tr	Number	2014	4.6E+07	5.9E+07	3.5E+07
Incidence	Global	Female	50-69 yea	Sexually tr	Number	2014	3.2E+07	4E+07	2.5E+07
Incidence	Global	Male	50-69 yea	Sexually tr	Rate	2014	7766.34	10064.7	5990.1
Incidence	Global	Female	50-69 yea	Sexually tr	Rate	2014	5176.38	6580.89	4011.62
Incidence	Global	Male	50-69 yea	Sexually tr	Number	2015	4.7E+07	6.1E+07	3.7E+07
Incidence	Global	Female	50-69 yea	Sexually tr	Number	2015	3.3E+07	4.1E+07	2.5E+07
Incidence	Global	Male	50-69 yea	Sexually tr	Rate	2015	7774.68	10080.8	5995.03
Incidence	Global	Female	50-69 yea	Sexually tr	Rate	2015	5170.55	6571.41	4008.08
Incidence	Global	Male	50-69 yea	Sexually tr	Number	2016	4.9E+07	6.3E+07	3.8E+07
Incidence	Global	Female	50-69 yea	Sexually tr	Number	2016	3.4E+07	4.3E+07	2.6E+07
Incidence	Global	Male	50-69 yea	Sexually tr	Rate	2016	7775.63	10046.2	5997.56
Incidence	Global	Female	50-69 yea	Sexually tr	Rate	2016	5218.51	6623.27	4047.44
Incidence	Global	Male	50-69 yea	Sexually tr	Number	2017	5E+07	6.5E+07	3.9E+07
Incidence	Global	Female	50-69 yea	Sexually tr	Number	2017	3.5E+07	4.5E+07	2.7E+07
Incidence	Global	Male	50-69 yea	Sexually tr	Rate	2017	7782.98	10059.2	6010.87
Incidence	Global	Female	50-69 yea	Sexually tr	Rate	2017	5275.04	6703.81	4078.78
Incidence	Global	Male	50-69 yea	Sexually tr	Number	2018	5.1E+07	6.7E+07	4E+07
Incidence	Global	Female	50-69 yea	Sexually tr	Number	2018	3.6E+07	4.6E+07	2.8E+07
Incidence	Global	Male	50-69 yea	Sexually tr	Rate	2018	7791.46	10076.3	6014.21
Incidence	Global	Female	50-69 yea	Sexually tr	Rate	2018	5297.06	6717.71	4100.46
Incidence	Global	Male	50-69 yea	Sexually tr	Number	2019	5.3E+07	6.8E+07	4.1E+07
Incidence	Global	Female	50-69 yea	Sexually tr	Number	2019	3.7E+07	4.8E+07	2.9E+07
Incidence	Global	Male	50-69 yea	Sexually tr	Rate	2019	7805.93	10071.6	6028.34
Incidence	Global	Female	50-69 yea	Sexually tr	Rate	2019	5332.97	6771.09	4134.8
Incidence	Global	Male	50-69 yea	Syphilis	Number	1990	298659	495544	174395
Incidence	Global	Female	50-69 yea	Syphilis	Number	1990	102868	175077	57023
Incidence	Global	Male	50-69 yea	Syphilis	Rate	1990	88.745	147.248	51.8206
Incidence	Global	Female	50-69 yea	Syphilis	Rate	1990	29.7648	50.6585	16.4996
Incidence	Global	Male	50-69 yea	Syphilis	Number	1991	301826	498337	176835
Incidence	Global	Female	50-69 yea	Syphilis	Number	1991	105135	179070	58534.4
Incidence	Global	Male	50-69 yea	Syphilis	Rate	1991	88.0412	145.362	51.5819
Incidence	Global	Female	50-69 yea	Syphilis	Rate	1991	29.9287	50.9758	16.663
Incidence	Global	Male	50-69 yea	Syphilis	Number	1992	304673	500497	179154
Incidence	Global	Female	50-69 yea	Syphilis	Number	1992	107215	182750	59885.4
Incidence	Global	Male	50-69 yea	Syphilis	Rate	1992	87.4359	143.634	51.4141
Incidence	Global	Female	50-69 yea	Syphilis	Rate	1992	30.0757	51.2648	16.7989
Incidence	Global	Male	50-69 yea	Syphilis	Number	1993	307201	504070	180731
Incidence	Global	Female	50-69 yea	Syphilis	Number	1993	108994	184842	61092.7
Incidence	Global	Male	50-69 yea	Syphilis	Rate	1993	86.8636	142.53	51.1031
Incidence	Global	Female	50-69 yea	Syphilis	Rate	1993	30.1836	51.188	16.9183
Incidence	Global	Male	50-69 yea	Syphilis	Number	1994	309891	508899	182642
Incidence	Global	Female	50-69 yea	Syphilis	Number	1994	110741	187087	62033.8
Incidence	Global	Male	50-69 yea	Syphilis	Rate	1994	86.4452	141.959	50.9487
Incidence	Global	Female	50-69 yea	Syphilis	Rate	1994	30.292	51.1754	16.9686
Incidence	Global	Male	50-69 yea	Syphilis	Number	1995	312590	513992	184140
Incidence	Global	Female	50-69 yea	Syphilis	Number	1995	112286	188936	62962.5
Incidence	Global	Male	50-69 yea	Syphilis	Rate	1995	86.1151	141.599	50.7286
Incidence	Global	Female	50-69 yea	Syphilis	Rate	1995	30.3515	51.0704	17.0191
Incidence	Global	Male	50-69 yea	Syphilis	Number	1996	317209	521339	187057
Incidence	Global	Female	50-69 yea	Syphilis	Number	1996	113933	191879	63942
Incidence	Global	Male	50-69 yea	Syphilis	Rate	1996	86.1156	141.533	50.782
Incidence	Global	Female	50-69 yea	Syphilis	Rate	1996	30.3508	51.1148	17.0336
Incidence	Global	Male	50-69 yea	Syphilis	Number	1997	325005	533834	191712
Incidence	Global	Female	50-69 yea	Syphilis	Number	1997	115784	195642	64954.7
Incidence	Global	Male	50-69 yea	Syphilis	Rate	1997	86.4718	142.033	51.0074
Incidence	Global	Female	50-69 yea	Syphilis	Rate	1997	30.2427	51.1017	16.9662
Incidence	Global	Male	50-69 yea	Syphilis	Number	1998	333305	549633	196774
Incidence	Global	Female	50-69 yea	Syphilis	Number	1998	117626	199587	65880
Incidence	Global	Male	50-69 yea	Syphilis	Rate	1998	86.9304	143.352	51.3213
Incidence	Global	Female	50-69 yea	Syphilis	Rate	1998	30.1025	51.0776	16.8597
Incidence	Global	Male	50-69 yea	Syphilis	Number	1999	343272	569549	202741
Incidence	Global	Female	50-69 yea	Syphilis	Number	1999	120077	204664	67179.2
Incidence	Global	Male	50-69 yea	Syphilis	Rate	1999	87.4788	145.143	51.6662
Incidence	Global	Female	50-69 yea	Syphilis	Rate	1999	30.0416	51.2043	16.8074
Incidence	Global	Male	50-69 yea	Syphilis	Number	2000	351128	585624	207618
Incidence	Global	Female	50-69 yea	Syphilis	Number	2000	122367	209209	68479.5
Incidence	Global	Male	50-69 yea	Syphilis	Rate	2000	87.752	146.356	51.8867
Incidence	Global	Female	50-69 yea	Syphilis	Rate	2000	29.9855	51.2658	16.7806
Incidence	Global	Male	50-69 yea	Syphilis	Number	2001	362995	606421	214427
Incidence	Global	Female	50-69 yea	Syphilis	Number	2001	127134	217706	71229.1
Incidence	Global	Male	50-69 yea	Syphilis	Rate	2001	88.4049	147.69	52.2222
Incidence	Global	Female	50-69 yea	Syphilis	Rate	2001	30.3329	51.9424	16.9945
Incidence	Global	Male	50-69 yea	Syphilis	Number	2002	373645	623634	220806
Incidence	Global	Female	50-69 yea	Syphilis	Number	2002	131924	225260	74068.2
Incidence	Global	Male	50-69 yea	Syphilis	Rate	2002	88.6052	147.887	52.3614
Incidence	Global	Female	50-69 yea	Syphilis	Rate	2002	30.6198	52.2835	17.1914
Incidence	Global	Male	50-69 yea	Syphilis	Number	2003	384502	640619	227341
Incidence	Global	Female	50-69 yea	Syphilis	Number	2003	137047	233139	77163.2
Incidence	Global	Male	50-69 yea	Syphilis	Rate	2003	88.6806	147.751	52.4334
Incidence	Global	Female	50-69 yea	Syphilis	Rate	2003	30.9173	52.5952	17.4077
Incidence	Global	Male	50-69 yea	Syphilis	Number	2004	395869	657558	233704
Incidence	Global	Female	50-69 yea	Syphilis	Number	2004	142279	241226	80256.9
Incidence	Global	Male	50-69 yea	Syphilis	Rate	2004	88.7054	147.344	52.3678
Incidence	Global	Female	50-69 yea	Syphilis	Rate	2004	31.1653	52.839	17.5797
Incidence	Global	Male	50-69 yea	Syphilis	Number	2005	409199	678571	240842
Incidence	Global	Female	50-69 yea	Syphilis	Number	2005	147875	250377	83285.8
Incidence	Global	Male	50-69 yea	Syphilis	Rate	2005	89.0397	147.654	52.4061
Incidence	Global	Female	50-69 yea	Syphilis	Rate	2005	31.4393	53.2318	17.7071
Incidence	Global	Male	50-69 yea	Syphilis	Number	2006	420045	696666	247422
Incidence	Global	Female	50-69 yea	Syphilis	Number	2006	151610	256090	85495.5
Incidence	Global	Male	50-69 yea	Syphilis	Rate	2006	88.8405	147.346	52.3303
Incidence	Global	Female	50-69 yea	Syphilis	Rate	2006	31.3268	52.9151	17.6657
Incidence	Global	Male	50-69 yea	Syphilis	Number	2007	428837	711549	252426
Incidence	Global	Female	50-69 yea	Syphilis	Number	2007	154068	258347	87109.8
Incidence	Global	Male	50-69 yea	Syphilis	Rate	2007	88.2623	146.45	51.9537
Incidence	Global	Female	50-69 yea	Syphilis	Rate	2007	30.9764	51.9423	17.514
Incidence	Global	Male	50-69 yea	Syphilis	Number	2008	437095	725283	256956
Incidence	Global	Female	50-69 yea	Syphilis	Number	2008	155939	259016	88346.9
Incidence	Global	Male	50-69 yea	Syphilis	Rate	2008	87.6577	145.453	51.5316
Incidence	Global	Female	50-69 yea	Syphilis	Rate	2008	30.5378	50.7237	17.3012
Incidence	Global	Male	50-69 yea	Syphilis	Number	2009	445382	738552	261387
Incidence	Global	Female	50-69 yea	Syphilis	Number	2009	157947	262697	89656.3
Incidence	Global	Male	50-69 yea	Syphilis	Rate	2009	87.0692	144.382	51.0995
Incidence	Global	Female	50-69 yea	Syphilis	Rate	2009	30.1208	50.0967	17.0976
Incidence	Global	Male	50-69 yea	Syphilis	Number	2010	456672	756259	267824
Incidence	Global	Female	50-69 yea	Syphilis	Number	2010	161514	268786	91703
Incidence	Global	Male	50-69 yea	Syphilis	Rate	2010	86.973	144.029	51.0071
Incidence	Global	Female	50-69 yea	Syphilis	Rate	2010	29.9718	49.878	17.0171
Incidence	Global	Male	50-69 yea	Syphilis	Number	2011	468832	776514	275345
Incidence	Global	Female	50-69 yea	Syphilis	Number	2011	165655	276188	94170.7
Incidence	Global	Male	50-69 yea	Syphilis	Rate	2011	86.8964	143.924	51.0342
Incidence	Global	Female	50-69 yea	Syphilis	Rate	2011	29.8704	49.8013	16.9805
Incidence	Global	Male	50-69 yea	Syphilis	Number	2012	482827	800406	283956
Incidence	Global	Female	50-69 yea	Syphilis	Number	2012	170425	284432	96960.9
Incidence	Global	Male	50-69 yea	Syphilis	Rate	2012	86.9372	144.12	51.1287
Incidence	Global	Female	50-69 yea	Syphilis	Rate	2012	29.8054	49.7441	16.9574
Incidence	Global	Male	50-69 yea	Syphilis	Number	2013	498876	828134	293441
Incidence	Global	Female	50-69 yea	Syphilis	Number	2013	175976	294241	100035
Incidence	Global	Male	50-69 yea	Syphilis	Rate	2013	87.1065	144.597	51.2364
Incidence	Global	Female	50-69 yea	Syphilis	Rate	2013	29.7879	49.8071	16.9333
Incidence	Global	Male	50-69 yea	Syphilis	Number	2014	515506	857473	303047
Incidence	Global	Female	50-69 yea	Syphilis	Number	2014	181965	305190	103522
Incidence	Global	Male	50-69 yea	Syphilis	Rate	2014	87.2087	145.06	51.2667
Incidence	Global	Female	50-69 yea	Syphilis	Rate	2014	29.7942	49.9707	16.9504
Incidence	Global	Male	50-69 yea	Syphilis	Number	2015	532532	887470	312707
Incidence	Global	Female	50-69 yea	Syphilis	Number	2015	188216	316678	107261
Incidence	Global	Male	50-69 yea	Syphilis	Rate	2015	87.3005	145.487	51.2635
Incidence	Global	Female	50-69 yea	Syphilis	Rate	2015	29.8164	50.1669	16.9918
Incidence	Global	Male	50-69 yea	Syphilis	Number	2016	549072	922476	319731
Incidence	Global	Female	50-69 yea	Syphilis	Number	2016	191935	319592	108720
Incidence	Global	Male	50-69 yea	Syphilis	Rate	2016	87.4488	146.92	50.9225
Incidence	Global	Female	50-69 yea	Syphilis	Rate	2016	29.491	49.1057	16.705
Incidence	Global	Male	50-69 yea	Syphilis	Number	2017	565766	958952	325007
Incidence	Global	Female	50-69 yea	Syphilis	Number	2017	196026	326747	110422
Incidence	Global	Male	50-69 yea	Syphilis	Rate	2017	87.8275	148.864	50.4529
Incidence	Global	Female	50-69 yea	Syphilis	Rate	2017	29.315	48.8638	16.5133
Incidence	Global	Male	50-69 yea	Syphilis	Number	2018	581429	980153	334272
Incidence	Global	Female	50-69 yea	Syphilis	Number	2018	205822	341067	115934
Incidence	Global	Male	50-69 yea	Syphilis	Rate	2018	88.0173	148.376	50.6024
Incidence	Global	Female	50-69 yea	Syphilis	Rate	2018	29.9935	49.7021	16.8945
Incidence	Global	Male	50-69 yea	Syphilis	Number	2019	596446	994551	343731
Incidence	Global	Female	50-69 yea	Syphilis	Number	2019	220761	368685	124581
Incidence	Global	Male	50-69 yea	Syphilis	Rate	2019	88.216	147.097	50.8387
Incidence	Global	Female	50-69 yea	Syphilis	Rate	2019	31.4103	52.4574	17.7257
Incidence	Global	Male	50-69 yea	Gonococca	Number	1990	1607070	2545824	1040419
Incidence	Global	Female	50-69 yea	Gonococca	Number	1990	839170	1293991	550698
Incidence	Global	Male	50-69 yea	Gonococca	Rate	1990	477.533	756.479	309.155
Incidence	Global	Female	50-69 yea	Gonococca	Rate	1990	242.814	374.416	159.345
Incidence	Global	Male	50-69 yea	Gonococca	Number	1991	1631984	2583383	1055751
Incidence	Global	Female	50-69 yea	Gonococca	Number	1991	849211	1311680	558036
Incidence	Global	Male	50-69 yea	Gonococca	Rate	1991	476.041	753.559	307.957
Incidence	Global	Female	50-69 yea	Gonococca	Rate	1991	241.745	373.396	158.856
Incidence	Global	Male	50-69 yea	Gonococca	Number	1992	1651343	2614303	1069702
Incidence	Global	Female	50-69 yea	Gonococca	Number	1992	856451	1325847	563401
Incidence	Global	Male	50-69 yea	Gonococca	Rate	1992	473.907	750.26	306.986
Incidence	Global	Female	50-69 yea	Gonococca	Rate	1992	240.25	371.924	158.044
Incidence	Global	Male	50-69 yea	Gonococca	Number	1993	1663619	2635203	1079663
Incidence	Global	Female	50-69 yea	Gonococca	Number	1993	857943	1329718	563749
Incidence	Global	Male	50-69 yea	Gonococca	Rate	1993	470.401	745.124	305.283
Incidence	Global	Female	50-69 yea	Gonococca	Rate	1993	237.588	368.236	156.118
Incidence	Global	Male	50-69 yea	Gonococca	Number	1994	1673045	2642496	1088104
Incidence	Global	Female	50-69 yea	Gonococca	Number	1994	858211	1330898	562449
Incidence	Global	Male	50-69 yea	Gonococca	Rate	1994	466.702	737.133	303.53
Incidence	Global	Female	50-69 yea	Gonococca	Rate	1994	234.753	364.051	153.851
Incidence	Global	Male	50-69 yea	Gonococca	Number	1995	1681375	2650494	1094258
Incidence	Global	Female	50-69 yea	Gonococca	Number	1995	857604	1331237	561591
Incidence	Global	Male	50-69 yea	Gonococca	Rate	1995	463.201	730.183	301.456
Incidence	Global	Female	50-69 yea	Gonococca	Rate	1995	231.815	359.84	151.801
Incidence	Global	Male	50-69 yea	Gonococca	Number	1996	1696029	2669439	1103951
Incidence	Global	Female	50-69 yea	Gonococca	Number	1996	861932	1337958	566077
Incidence	Global	Male	50-69 yea	Gonococca	Rate	1996	460.436	724.697	299.7
Incidence	Global	Female	50-69 yea	Gonococca	Rate	1996	229.611	356.42	150.798
Incidence	Global	Male	50-69 yea	Gonococca	Number	1997	1725098	2720881	1123269
Incidence	Global	Female	50-69 yea	Gonococca	Number	1997	875881	1362162	577862
Incidence	Global	Male	50-69 yea	Gonococca	Rate	1997	458.985	723.925	298.86
Incidence	Global	Female	50-69 yea	Gonococca	Rate	1997	228.78	355.796	150.937
Incidence	Global	Male	50-69 yea	Gonococca	Number	1998	1757366	2774246	1144963
Incidence	Global	Female	50-69 yea	Gonococca	Number	1998	893609	1388037	588532
Incidence	Global	Male	50-69 yea	Gonococca	Rate	1998	458.345	723.561	298.622
Incidence	Global	Female	50-69 yea	Gonococca	Rate	1998	228.689	355.221	150.615
Incidence	Global	Male	50-69 yea	Gonococca	Number	1999	1805034	2862901	1171553
Incidence	Global	Female	50-69 yea	Gonococca	Number	1999	919536	1430341	603069
Incidence	Global	Male	50-69 yea	Gonococca	Rate	1999	459.991	729.576	298.556
Incidence	Global	Female	50-69 yea	Gonococca	Rate	1999	230.056	357.852	150.88
Incidence	Global	Male	50-69 yea	Gonococca	Number	2000	1847277	2930783	1194925
Incidence	Global	Female	50-69 yea	Gonococca	Number	2000	941993	1471033	616173
Incidence	Global	Male	50-69 yea	Gonococca	Rate	2000	461.661	732.445	298.629
Incidence	Global	Female	50-69 yea	Gonococca	Rate	2000	230.832	360.471	150.991
Incidence	Global	Male	50-69 yea	Gonococca	Number	2001	1916837	3041044	1238344
Incidence	Global	Female	50-69 yea	Gonococca	Number	2001	972881	1521714	635120
Incidence	Global	Male	50-69 yea	Gonococca	Rate	2001	466.833	740.626	301.59
Incidence	Global	Female	50-69 yea	Gonococca	Rate	2001	232.119	363.065	151.533
Incidence	Global	Male	50-69 yea	Gonococca	Number	2002	1982329	3150515	1277766
Incidence	Global	Female	50-69 yea	Gonococca	Number	2002	1001959	1566531	652737
Incidence	Global	Male	50-69 yea	Gonococca	Rate	2002	470.085	747.105	303.006
Incidence	Global	Female	50-69 yea	Gonococca	Rate	2002	232.557	363.596	151.502
Incidence	Global	Male	50-69 yea	Gonococca	Number	2003	2053699	3266095	1321216
Incidence	Global	Female	50-69 yea	Gonococca	Number	2003	1035814	1617993	673327
Incidence	Global	Male	50-69 yea	Gonococca	Rate	2003	473.66	753.284	304.722
Incidence	Global	Female	50-69 yea	Gonococca	Rate	2003	233.675	365.012	151.9
Incidence	Global	Male	50-69 yea	Gonococca	Number	2004	2126077	3371705	1365097
Incidence	Global	Female	50-69 yea	Gonococca	Number	2004	1071256	1669483	693412
Incidence	Global	Male	50-69 yea	Gonococca	Rate	2004	476.407	755.524	305.888
Incidence	Global	Female	50-69 yea	Gonococca	Rate	2004	234.651	365.689	151.887
Incidence	Global	Male	50-69 yea	Gonococca	Number	2005	2209235	3497390	1419168
Incidence	Global	Female	50-69 yea	Gonococca	Number	2005	1111329	1734324	717846
Incidence	Global	Male	50-69 yea	Gonococca	Rate	2005	480.719	761.015	308.804
Incidence	Global	Female	50-69 yea	Gonococca	Rate	2005	236.276	368.729	152.619
Incidence	Global	Male	50-69 yea	Gonococca	Number	2006	2277308	3603877	1463867
Incidence	Global	Female	50-69 yea	Gonococca	Number	2006	1139171	1775431	734366
Incidence	Global	Male	50-69 yea	Gonococca	Rate	2006	481.655	762.228	309.611
Incidence	Global	Female	50-69 yea	Gonococca	Rate	2006	235.383	366.852	151.74
Incidence	Global	Male	50-69 yea	Gonococca	Number	2007	2336979	3697854	1504598
Incidence	Global	Female	50-69 yea	Gonococca	Number	2007	1153516	1794574	746832
Incidence	Global	Male	50-69 yea	Gonococca	Rate	2007	480.992	761.085	309.673
Incidence	Global	Female	50-69 yea	Gonococca	Rate	2007	231.922	360.81	150.155
Incidence	Global	Male	50-69 yea	Gonococca	Number	2008	2390946	3794487	1544691
Incidence	Global	Female	50-69 yea	Gonococca	Number	2008	1161218	1798603	754459
Incidence	Global	Male	50-69 yea	Gonococca	Rate	2008	479.495	760.969	309.782
Incidence	Global	Female	50-69 yea	Gonococca	Rate	2008	227.404	352.224	147.747
Incidence	Global	Male	50-69 yea	Gonococca	Number	2009	2439645	3875901	1575050
Incidence	Global	Female	50-69 yea	Gonococca	Number	2009	1168721	1793583	761313
Incidence	Global	Male	50-69 yea	Gonococca	Rate	2009	476.935	757.714	307.912
Incidence	Global	Female	50-69 yea	Gonococca	Rate	2009	222.877	342.039	145.184
Incidence	Global	Male	50-69 yea	Gonococca	Number	2010	2495903	3969374	1619424
Incidence	Global	Female	50-69 yea	Gonococca	Number	2010	1186235	1821166	774082
Incidence	Global	Male	50-69 yea	Gonococca	Rate	2010	475.344	755.967	308.419
Incidence	Global	Female	50-69 yea	Gonococca	Rate	2010	220.127	337.95	143.645
Incidence	Global	Male	50-69 yea	Gonococca	Number	2011	2548260	4041788	1654212
Incidence	Global	Female	50-69 yea	Gonococca	Number	2011	1208883	1857398	791641
Incidence	Global	Male	50-69 yea	Gonococca	Rate	2011	472.311	749.132	306.603
Incidence	Global	Female	50-69 yea	Gonococca	Rate	2011	217.982	334.92	142.746
Incidence	Global	Male	50-69 yea	Gonococca	Number	2012	2604905	4116990	1692793
Incidence	Global	Female	50-69 yea	Gonococca	Number	2012	1235826	1898654	808465
Incidence	Global	Male	50-69 yea	Gonococca	Rate	2012	469.035	741.299	304.802
Incidence	Global	Female	50-69 yea	Gonococca	Rate	2012	216.132	332.054	141.392
Incidence	Global	Male	50-69 yea	Gonococca	Number	2013	2670810	4215194	1737199
Incidence	Global	Female	50-69 yea	Gonococca	Number	2013	1268360	1952091	830159
Incidence	Global	Male	50-69 yea	Gonococca	Rate	2013	466.338	735.997	303.325
Incidence	Global	Female	50-69 yea	Gonococca	Rate	2013	214.699	330.437	140.524
Incidence	Global	Male	50-69 yea	Gonococca	Number	2014	2742039	4316258	1782977
Incidence	Global	Female	50-69 yea	Gonococca	Number	2014	1302698	2007175	854428
Incidence	Global	Male	50-69 yea	Gonococca	Rate	2014	463.874	730.186	301.628
Incidence	Global	Female	50-69 yea	Gonococca	Rate	2014	213.299	328.648	139.901
Incidence	Global	Male	50-69 yea	Gonococca	Number	2015	2817347	4440440	1826963
Incidence	Global	Female	50-69 yea	Gonococca	Number	2015	1338215	2064532	876757
Incidence	Global	Male	50-69 yea	Gonococca	Rate	2015	461.861	727.942	299.503
Incidence	Global	Female	50-69 yea	Gonococca	Rate	2015	211.995	327.055	138.893
Incidence	Global	Male	50-69 yea	Gonococca	Number	2016	2880119	4540509	1871555
Incidence	Global	Female	50-69 yea	Gonococca	Number	2016	1364747	2109470	897093
Incidence	Global	Male	50-69 yea	Gonococca	Rate	2016	458.707	723.152	298.076
Incidence	Global	Female	50-69 yea	Gonococca	Rate	2016	209.695	324.122	137.839
Incidence	Global	Male	50-69 yea	Gonococca	Number	2017	2942738	4633676	1913412
Incidence	Global	Female	50-69 yea	Gonococca	Number	2017	1388926	2153465	914838
Incidence	Global	Male	50-69 yea	Gonococca	Rate	2017	456.82	719.315	297.031
Incidence	Global	Female	50-69 yea	Gonococca	Rate	2017	207.709	322.043	136.811
Incidence	Global	Male	50-69 yea	Gonococca	Number	2018	3014480	4784291	1961313
Incidence	Global	Female	50-69 yea	Gonococca	Number	2018	1433021	2207743	935050
Incidence	Global	Male	50-69 yea	Gonococca	Rate	2018	456.335	724.251	296.905
Incidence	Global	Female	50-69 yea	Gonococca	Rate	2018	208.827	321.724	136.26
Incidence	Global	Male	50-69 yea	Gonococca	Number	2019	3088924	4870005	1999851
Incidence	Global	Female	50-69 yea	Gonococca	Number	2019	1496154	2318935	968295
Incidence	Global	Male	50-69 yea	Gonococca	Rate	2019	456.861	720.288	295.784
Incidence	Global	Female	50-69 yea	Gonococca	Rate	2019	212.876	329.943	137.771
Incidence	Global	Male	50-69 yea	Genital her	Number	1990	1095420	1577091	682568
Incidence	Global	Female	50-69 yea	Genital her	Number	1990	1268617	1835359	789338
Incidence	Global	Male	50-69 yea	Genital her	Rate	1990	325.498	468.625	202.822
Incidence	Global	Female	50-69 yea	Genital her	Rate	1990	367.075	531.061	228.395
Incidence	Global	Male	50-69 yea	Genital her	Number	1991	1116037	1604545	697543
Incidence	Global	Female	50-69 yea	Genital her	Number	1991	1284936	1859866	801566
Incidence	Global	Male	50-69 yea	Genital her	Rate	1991	325.542	468.038	203.47
Incidence	Global	Female	50-69 yea	Genital her	Rate	1991	365.783	529.448	228.182
Incidence	Global	Male	50-69 yea	Genital her	Number	1992	1132472	1625569	709017
Incidence	Global	Female	50-69 yea	Genital her	Number	1992	1299848	1880108	810874
Incidence	Global	Male	50-69 yea	Genital her	Rate	1992	325	466.51	203.476
Incidence	Global	Female	50-69 yea	Genital her	Rate	1992	364.631	527.405	227.465
Incidence	Global	Male	50-69 yea	Genital her	Number	1993	1146115	1640826	718249
Incidence	Global	Female	50-69 yea	Genital her	Number	1993	1312979	1897980	819128
Incidence	Global	Male	50-69 yea	Genital her	Rate	1993	324.073	463.956	203.09
Incidence	Global	Female	50-69 yea	Genital her	Rate	1993	363.601	525.604	226.84
Incidence	Global	Male	50-69 yea	Genital her	Number	1994	1157876	1652213	726127
Incidence	Global	Female	50-69 yea	Genital her	Number	1994	1326930	1917142	827548
Incidence	Global	Male	50-69 yea	Genital her	Rate	1994	322.993	460.891	202.556
Incidence	Global	Female	50-69 yea	Genital her	Rate	1994	362.966	524.412	226.366
Incidence	Global	Male	50-69 yea	Genital her	Number	1995	1168762	1664090	733290
Incidence	Global	Female	50-69 yea	Genital her	Number	1995	1340859	1936686	833226
Incidence	Global	Male	50-69 yea	Genital her	Rate	1995	321.981	458.439	202.014
Incidence	Global	Female	50-69 yea	Genital her	Rate	1995	362.441	523.496	225.225
Incidence	Global	Male	50-69 yea	Genital her	Number	1996	1181611	1683688	742014
Incidence	Global	Female	50-69 yea	Genital her	Number	1996	1362812	1967081	848278
Incidence	Global	Male	50-69 yea	Genital her	Rate	1996	320.783	457.086	201.441
Incidence	Global	Female	50-69 yea	Genital her	Rate	1996	363.04	524.012	225.973
Incidence	Global	Male	50-69 yea	Genital her	Number	1997	1201547	1711294	757233
Incidence	Global	Female	50-69 yea	Genital her	Number	1997	1400950	2021519	869456
Incidence	Global	Male	50-69 yea	Genital her	Rate	1997	319.687	455.312	201.472
Incidence	Global	Female	50-69 yea	Genital her	Rate	1997	365.928	528.02	227.102
Incidence	Global	Male	50-69 yea	Genital her	Number	1998	1220741	1740338	772070
Incidence	Global	Female	50-69 yea	Genital her	Number	1998	1443453	2085556	896204
Incidence	Global	Male	50-69 yea	Genital her	Rate	1998	318.386	453.904	201.366
Incidence	Global	Female	50-69 yea	Genital her	Rate	1998	369.403	533.727	229.353
Incidence	Global	Male	50-69 yea	Genital her	Number	1999	1248744	1784197	792795
Incidence	Global	Female	50-69 yea	Genital her	Number	1999	1493799	2162090	931391
Incidence	Global	Male	50-69 yea	Genital her	Rate	1999	318.227	454.681	202.034
Incidence	Global	Female	50-69 yea	Genital her	Rate	1999	373.729	540.926	233.022
Incidence	Global	Male	50-69 yea	Genital her	Number	2000	1274221	1819568	810881
Incidence	Global	Female	50-69 yea	Genital her	Number	2000	1536861	2228442	961568
Incidence	Global	Male	50-69 yea	Genital her	Rate	2000	318.446	454.736	202.651
Incidence	Global	Female	50-69 yea	Genital her	Rate	2000	376.602	546.071	235.628
Incidence	Global	Male	50-69 yea	Genital her	Number	2001	1317689	1887851	835149
Incidence	Global	Female	50-69 yea	Genital her	Number	2001	1592482	2307498	995112
Incidence	Global	Male	50-69 yea	Genital her	Rate	2001	320.914	459.774	203.395
Incidence	Global	Female	50-69 yea	Genital her	Rate	2001	379.95	550.545	237.424
Incidence	Global	Male	50-69 yea	Genital her	Number	2002	1361918	1953847	860326
Incidence	Global	Female	50-69 yea	Genital her	Number	2002	1645554	2383012	1026410
Incidence	Global	Male	50-69 yea	Genital her	Rate	2002	322.962	463.331	204.015
Incidence	Global	Female	50-69 yea	Genital her	Rate	2002	381.937	553.103	238.232
Incidence	Global	Male	50-69 yea	Genital her	Number	2003	1410642	2027558	892988
Incidence	Global	Female	50-69 yea	Genital her	Number	2003	1702869	2468253	1060191
Incidence	Global	Male	50-69 yea	Genital her	Rate	2003	325.347	467.631	205.957
Incidence	Global	Female	50-69 yea	Genital her	Rate	2003	384.16	556.828	239.175
Incidence	Global	Male	50-69 yea	Genital her	Number	2004	1460767	2102010	922181
Incidence	Global	Female	50-69 yea	Genital her	Number	2004	1762205	2558553	1096344
Incidence	Global	Male	50-69 yea	Genital her	Rate	2004	327.325	471.014	206.64
Incidence	Global	Female	50-69 yea	Genital her	Rate	2004	385.999	560.433	240.146
Incidence	Global	Male	50-69 yea	Genital her	Number	2005	1515449	2178033	956416
Incidence	Global	Female	50-69 yea	Genital her	Number	2005	1827092	2655314	1135729
Incidence	Global	Male	50-69 yea	Genital her	Rate	2005	329.754	473.929	208.111
Incidence	Global	Female	50-69 yea	Genital her	Rate	2005	388.452	564.538	241.464
Incidence	Global	Male	50-69 yea	Genital her	Number	2006	1562858	2246889	985179
Incidence	Global	Female	50-69 yea	Genital her	Number	2006	1881942	2737926	1176040
Incidence	Global	Male	50-69 yea	Genital her	Rate	2006	330.548	475.222	208.367
Incidence	Global	Female	50-69 yea	Genital her	Rate	2006	388.86	565.729	243.002
Incidence	Global	Male	50-69 yea	Genital her	Number	2007	1606923	2312502	1011542

Incidence	Global	Male	50-69 yea	Genital hei	Number	2016	2054508	2961480	1283533
Incidence	Global	Female	50-69 yea	Genital hei	Number	2016	2426159	3536768	1494856
Incidence	Global	Male	50-69 yea	Genital hei	Rate	2016	327.215	471.665	204.424
Incidence	Global	Female	50-69 yea	Genital hei	Rate	2016	372.782	543.428	229.686
Incidence	Global	Male	50-69 yea	Genital hei	Number	2017	2105738	3033504	1316610
Incidence	Global	Female	50-69 yea	Genital hei	Number	2017	2494286	3642756	1537901
Incidence	Global	Male	50-69 yea	Genital hei	Rate	2017	326.887	470.91	204.386
Incidence	Global	Female	50-69 yea	Genital hei	Rate	2017	373.012	544.761	229.988
Incidence	Global	Male	50-69 yea	Genital hei	Number	2018	2157958	3109057	1361756
Incidence	Global	Female	50-69 yea	Genital hei	Number	2018	2555786	3739721	1572127
Incidence	Global	Male	50-69 yea	Genital hei	Rate	2018	326.674	470.652	206.144
Incidence	Global	Female	50-69 yea	Genital hei	Rate	2018	372.442	544.972	229.099
Incidence	Global	Male	50-69 yea	Genital hei	Number	2019	2210247	3191341	1396893
Incidence	Global	Female	50-69 yea	Genital hei	Number	2019	2615088	3830758	1609656
Incidence	Global	Male	50-69 yea	Genital hei	Rate	2019	326.902	472.008	206.604
Incidence	Global	Female	50-69 yea	Genital hei	Rate	2019	372.08	545.049	229.025
Incidence	Global	Male	50-69 yea	Other sexu	Number	1990	0	0	0
Incidence	Global	Female	50-69 yea	Other sexu	Number	1990	0	0	0
Incidence	Global	Male	50-69 yea	Other sexu	Rate	1990	0	0	0
Incidence	Global	Female	50-69 yea	Other sexu	Rate	1990	0	0	0
Incidence	Global	Male	50-69 yea	Other sexu	Number	1991	0	0	0
Incidence	Global	Female	50-69 yea	Other sexu	Number	1991	0	0	0
Incidence	Global	Male	50-69 yea	Other sexu	Rate	1991	0	0	0
Incidence	Global	Female	50-69 yea	Other sexu	Rate	1991	0	0	0
Incidence	Global	Male	50-69 yea	Other sexu	Number	1992	0	0	0
Incidence	Global	Female	50-69 yea	Other sexu	Number	1992	0	0	0
Incidence	Global	Male	50-69 yea	Other sexu	Rate	1992	0	0	0
Incidence	Global	Female	50-69 yea	Other sexu	Rate	1992	0	0	0
Incidence	Global	Male	50-69 yea	Other sexu	Number	1993	0	0	0
Incidence	Global	Female	50-69 yea	Other sexu	Number	1993	0	0	0
Incidence	Global	Male	50-69 yea	Other sexu	Rate	1993	0	0	0
Incidence	Global	Female	50-69 yea	Other sexu	Rate	1993	0	0	0
Incidence	Global	Male	50-69 yea	Other sexu	Number	1994	0	0	0
Incidence	Global	Female	50-69 yea	Other sexu	Number	1994	0	0	0
Incidence	Global	Male	50-69 yea	Other sexu	Rate	1994	0	0	0
Incidence	Global	Female	50-69 yea	Other sexu	Rate	1994	0	0	0
Incidence	Global	Male	50-69 yea	Other sexu	Number	1995	0	0	0
Incidence	Global	Female	50-69 yea	Other sexu	Number	1995	0	0	0
Incidence	Global	Male	50-69 yea	Other sexu	Rate	1995	0	0	0
Incidence	Global	Female	50-69 yea	Other sexu	Rate	1995	0	0	0
Incidence	Global	Male	50-69 yea	Other sexu	Number	1996	0	0	0
Incidence	Global	Female	50-69 yea	Other sexu	Number	1996	0	0	0
Incidence	Global	Male	50-69 yea	Other sexu	Rate	1996	0	0	0
Incidence	Global	Female	50-69 yea	Other sexu	Rate	1996	0	0	0
Incidence	Global	Male	50-69 yea	Other sexu	Number	1997	0	0	0
Incidence	Global	Female	50-69 yea	Other sexu	Number	1997	0	0	0
Incidence	Global	Male	50-69 yea	Other sexu	Rate	1997	0	0	0
Incidence	Global	Female	50-69 yea	Other sexu	Rate	1997	0	0	0
Incidence	Global	Male	50-69 yea	Other sexu	Number	1998	0	0	0
Incidence	Global	Female	50-69 yea	Other sexu	Number	1998	0	0	0
Incidence	Global	Male	50-69 yea	Other sexu	Rate	1998	0	0	0
Incidence	Global	Female	50-69 yea	Other sexu	Rate	1998	0	0	0
Incidence	Global	Male	50-69 yea	Other sexu	Number	1999	0	0	0
Incidence	Global	Female	50-69 yea	Other sexu	Number	1999	0	0	0
Incidence	Global	Male	50-69 yea	Other sexu	Rate	1999	0	0	0
Incidence	Global	Female	50-69 yea	Other sexu	Rate	1999	0	0	0
Incidence	Global	Male	50-69 yea	Other sexu	Number	2000	0	0	0
Incidence	Global	Female	50-69 yea	Other sexu	Number	2000	0	0	0
Incidence	Global	Male	50-69 yea	Other sexu	Rate	2000	0	0	0
Incidence	Global	Female	50-69 yea	Other sexu	Rate	2000	0	0	0
Incidence	Global	Male	50-69 yea	Other sexu	Number	2001	0	0	0
Incidence	Global	Female	50-69 yea	Other sexu	Number	2001	0	0	0
Incidence	Global	Male	50-69 yea	Other sexu	Rate	2001	0	0	0
Incidence	Global	Female	50-69 yea	Other sexu	Rate	2001	0	0	0
Incidence	Global	Male	50-69 yea	Other sexu	Number	2002	0	0	0
Incidence	Global	Female	50-69 yea	Other sexu	Number	2002	0	0	0
Incidence	Global	Male	50-69 yea	Other sexu	Rate	2002	0	0	0
Incidence	Global	Female	50-69 yea	Other sexu	Rate	2002	0	0	0
Incidence	Global	Male	50-69 yea	Other sexu	Number	2003	0	0	0
Incidence	Global	Female	50-69 yea	Other sexu	Number	2003	0	0	0
Incidence	Global	Male	50-69 yea	Other sexu	Rate	2003	0	0	0
Incidence	Global	Female	50-69 yea	Other sexu	Rate	2003	0	0	0
Incidence	Global	Male	50-69 yea	Other sexu	Number	2004	0	0	0
Incidence	Global	Female	50-69 yea	Other sexu	Number	2004	0	0	0
Incidence	Global	Male	50-69 yea	Other sexu	Rate	2004	0	0	0
Incidence	Global	Female	50-69 yea	Other sexu	Rate	2004	0	0	0
Incidence	Global	Male	50-69 yea	Other sexu	Number	2005	0	0	0
Incidence	Global	Female	50-69 yea	Other sexu	Number	2005	0	0	0
Incidence	Global	Male	50-69 yea	Other sexu	Rate	2005	0	0	0
Incidence	Global	Female	50-69 yea	Other sexu	Rate	2005	0	0	0
Incidence	Global	Male	50-69 yea	Other sexu	Number	2006	0	0	0
Incidence	Global	Female	50-69 yea	Other sexu	Number	2006	0	0	0
Incidence	Global	Male	50-69 yea	Other sexu	Rate	2006	0	0	0
Incidence	Global	Female	50-69 yea	Other sexu	Rate	2006	0	0	0
Incidence	Global	Male	50-69 yea	Other sexu	Number	2007	0	0	0
Incidence	Global	Female	50-69 yea	Other sexu	Number	2007	0	0	0
Incidence	Global	Male	50-69 yea	Other sexu	Rate	2007	0	0	0
Incidence	Global	Female	50-69 yea	Other sexu	Rate	2007	0	0	0
Incidence	Global	Male	50-69 yea	Other sexu	Number	2008	0	0	0
Incidence	Global	Female	50-69 yea	Other sexu	Number	2008	0	0	0
Incidence	Global	Male	50-69 yea	Other sexu	Rate	2008	0	0	0
Incidence	Global	Female	50-69 yea	Other sexu	Rate	2008	0	0	0
Incidence	Global	Male	50-69 yea	Other sexu	Number	2009	0	0	0
Incidence	Global	Female	50-69 yea	Other sexu	Number	2009	0	0	0
Incidence	Global	Male	50-69 yea	Other sexu	Rate	2009	0	0	0
Incidence	Global	Female	50-69 yea	Other sexu	Rate	2009	0	0	0
Incidence	Global	Male	50-69 yea	Other sexu	Number	2010	0	0	0
Incidence	Global	Female	50-69 yea	Other sexu	Number	2010	0	0	0
Incidence	Global	Male	50-69 yea	Other sexu	Rate	2010	0	0	0
Incidence	Global	Female	50-69 yea	Other sexu	Rate	2010	0	0	0
Incidence	Global	Male	50-69 yea	Other sexu	Number	2011	0	0	0
Incidence	Global	Female	50-69 yea	Other sexu	Number	2011	0	0	0
Incidence	Global	Male	50-69 yea	Other sexu	Rate	2011	0	0	0
Incidence	Global	Female	50-69 yea	Other sexu	Rate	2011	0	0	0
Incidence	Global	Male	50-69 yea	Other sexu	Number	2012	0	0	0
Incidence	Global	Female	50-69 yea	Other sexu	Number	2012	0	0	0
Incidence	Global	Male	50-69 yea	Other sexu	Rate	2012	0	0	0
Incidence	Global	Female	50-69 yea	Other sexu	Rate	2012	0	0	0
Incidence	Global	Male	50-69 yea	Other sexu	Number	2013	0	0	0
Incidence	Global	Female	50-69 yea	Other sexu	Number	2013	0	0	0
Incidence	Global	Male	50-69 yea	Other sexu	Rate	2013	0	0	0
Incidence	Global	Female	50-69 yea	Other sexu	Rate	2013	0	0	0
Incidence	Global	Male	50-69 yea	Other sexu	Number	2014	0	0	0
Incidence	Global	Female	50-69 yea	Other sexu	Number	2014	0	0	0
Incidence	Global	Male	50-69 yea	Other sexu	Rate	2014	0	0	0
Incidence	Global	Female	50-69 yea	Other sexu	Rate	2014	0	0	0
Incidence	Global	Male	50-69 yea	Other sexu	Number	2015	0	0	0
Incidence	Global	Female	50-69 yea	Other sexu	Number	2015	0	0	0
Incidence	Global	Male	50-69 yea	Other sexu	Rate	2015	0	0	0
Incidence	Global	Female	50-69 yea	Other sexu	Rate	2015	0	0	0
Incidence	Global	Male	50-69 yea	Other sexu	Number	2016	0	0	0
Incidence	Global	Female	50-69 yea	Other sexu	Number	2016	0	0	0
Incidence	Global	Male	50-69 yea	Other sexu	Rate	2016	0	0	0
Incidence	Global	Female	50-69 yea	Other sexu	Rate	2016	0	0	0
Incidence	Global	Male	50-69 yea	Other sexu	Number	2017	0	0	0
Incidence	Global	Female	50-69 yea	Other sexu	Number	2017	0	0	0
Incidence	Global	Male	50-69 yea	Other sexu	Rate	2017	0	0	0
Incidence	Global	Female	50-69 yea	Other sexu	Rate	2017	0	0	0
Incidence	Global	Male	50-69 yea	Other sexu	Number	2018	0	0	0
Incidence	Global	Female	50-69 yea	Other sexu	Number	2018	0	0	0
Incidence	Global	Male	50-69 yea	Other sexu	Rate	2018	0	0	0
Incidence	Global	Female	50-69 yea	Other sexu	Rate	2018	0	0	0

measure	sex	cause	AAPC	AAPC C.I. l	AAPC C.I. l	P-Value
Incidence	Female	Chlamydia	0.1913	0.1208	0.2618	0
Incidence	Female	Genital he	0.0471	0.0189	0.0753	0.001065
Incidence	Female	Gonococca	-0.476	-0.5596	-0.3923	0
Incidence	Female	Syphilis	0.1637	0.0871	0.2403	0.000028
Incidence	Female	Trichomor	0.2084	0.1646	0.2521	0
Incidence	Male	Chlamydia	0.1372	0.0447	0.2297	0.00363
Incidence	Male	Genital he	0.008	-0.014	0.03	0.476494
Incidence	Male	Gonococca	-0.1592	-0.1976	-0.1208	0
Incidence	Male	Syphilis	-0.019	-0.0705	0.0324	0.46828
Incidence	Male	Trichomor	0.0692	0.0393	0.0991	0.000006
Prevalence	Female	Chlamydia	0.1893	0.1189	0.2597	0
Prevalence	Female	Genital he	0.0649	0.043	0.0867	0
Prevalence	Female	Gonococca	-0.4776	-0.561	-0.3942	0
Prevalence	Female	Other sexu	-0.4869	-0.5612	-0.4125	0
Prevalence	Female	Syphilis	-0.0838	-0.1522	-0.0154	0.016335
Prevalence	Female	Trichomor	0.0637	0.033	0.0945	0.000049
Prevalence	Male	Chlamydia	0.1154	0.0194	0.2116	0.018529
Prevalence	Male	Genital he	0.0356	-0.0097	0.0809	0.123031
Prevalence	Male	Gonococca	-0.216	-0.2925	-0.1395	0
Prevalence	Male	Syphilis	-0.1201	-0.1863	-0.0539	0.000379
Prevalence	Male	Trichomor	0.0957	0.0626	0.1289	0

measure	location	sex	age	cause	metric	year	val	upper	lower
Deaths	High SDI	Both	50-69 yea	HIV/AIDS	Number	1990	5117.14	5172.42	5061.88
Deaths	High SDI	Both	50-69 yea	HIV/AIDS	Rate	1990	3.33567	3.37171	3.29965
Deaths	Low-middl	Both	50-69 yea	Sexually tra	Number	1990	883.686	1110.59	648.443
Deaths	Low-middl	Both	50-69 yea	Sexually tra	Rate	1990	0.79934	1.00459	0.58655
Deaths	Low SDI	Both	50-69 yea	Sexually tra	Number	1990	723.953	1169.68	380.558
Deaths	Low SDI	Both	50-69 yea	Sexually tra	Rate	1990	1.64794	2.66254	0.86627
Deaths	Low-middl	Both	50-69 yea	HIV/AIDS	Number	1990	5783.09	9248.38	3291.84
Deaths	Low-middl	Both	50-69 yea	HIV/AIDS	Rate	1990	5.23113	8.36568	2.97765
Deaths	High SDI	Both	50-69 yea	Sexually tra	Number	1990	148.097	155.213	123.755
Deaths	High SDI	Both	50-69 yea	Sexually tra	Rate	1990	0.09654	0.10118	0.08067
Deaths	Middle SDI	Both	50-69 yea	Sexually tra	Number	1990	544.726	644.376	399.34
Deaths	Middle SDI	Both	50-69 yea	Sexually tra	Rate	1990	0.2901	0.34317	0.21267
Deaths	High SDI	Both	50-69 yea	Sexually tra	Number	1991	142.254	148.724	117.97
Deaths	High SDI	Both	50-69 yea	Sexually tra	Rate	1991	0.09173	0.0959	0.07607
Deaths	High-midc	Both	50-69 yea	Sexually tra	Number	1991	436.497	475.596	332.677
Deaths	High-midc	Both	50-69 yea	Sexually tra	Rate	1991	0.23125	0.25196	0.17625
Deaths	High-midc	Both	50-69 yea	HIV/AIDS	Number	1991	2376.99	2519.1	2268.6
Deaths	High-midc	Both	50-69 yea	HIV/AIDS	Rate	1991	1.25928	1.33457	1.20186
Deaths	Middle SDI	Both	50-69 yea	HIV/AIDS	Number	1990	2060.21	2745.89	1468.39
Deaths	Middle SDI	Both	50-69 yea	HIV/AIDS	Rate	1990	1.0972	1.46237	0.78202
Deaths	High SDI	Both	50-69 yea	Sexually tra	Number	1992	134.253	140.24	112.06
Deaths	High SDI	Both	50-69 yea	Sexually tra	Rate	1992	0.08575	0.08958	0.07158
Deaths	Low SDI	Both	50-69 yea	HIV/AIDS	Number	1990	23547.2	32996.8	15140.8
Deaths	Low SDI	Both	50-69 yea	HIV/AIDS	Rate	1990	53.6007	75.111	34.4652
Deaths	High-midc	Both	50-69 yea	HIV/AIDS	Number	1993	3371.26	3633.47	3190.01
Deaths	High-midc	Both	50-69 yea	HIV/AIDS	Rate	1993	1.76206	1.8991	1.66732
Deaths	High SDI	Both	50-69 yea	HIV/AIDS	Number	1991	6028.07	6089.87	5965.45
Deaths	High SDI	Both	50-69 yea	HIV/AIDS	Rate	1991	3.88707	3.92691	3.84668
Deaths	High-midc	Both	50-69 yea	Sexually tra	Number	1993	516.18	562.206	358.114
Deaths	High-midc	Both	50-69 yea	Sexually tra	Rate	1993	0.26979	0.29385	0.18718
Deaths	Low SDI	Both	50-69 yea	HIV/AIDS	Number	1991	29657.7	40723.3	19744.6
Deaths	Low SDI	Both	50-69 yea	HIV/AIDS	Rate	1991	66.3237	91.0698	44.1549
Deaths	Low-middl	Both	50-69 yea	Sexually tra	Number	1991	886.077	1098.21	654.409
Deaths	Low-middl	Both	50-69 yea	Sexually tra	Rate	1991	0.78362	0.97122	0.57874
Deaths	Low-middl	Both	50-69 yea	HIV/AIDS	Number	1991	8637.43	13538.1	5007.74
Deaths	Low-middl	Both	50-69 yea	HIV/AIDS	Rate	1991	7.63866	11.9726	4.42868
Deaths	High SDI	Both	50-69 yea	HIV/AIDS	Number	1992	6752.6	6828.42	6681.57
Deaths	High SDI	Both	50-69 yea	HIV/AIDS	Rate	1992	4.3131	4.36153	4.26773
Deaths	High-midc	Both	50-69 yea	HIV/AIDS	Number	1992	2783.18	2979.16	2637.46
Deaths	High-midc	Both	50-69 yea	HIV/AIDS	Rate	1992	1.45988	1.56268	1.38345
Deaths	Low SDI	Both	50-69 yea	Sexually tra	Number	1991	724.082	1169.5	378.352
Deaths	Low SDI	Both	50-69 yea	Sexually tra	Rate	1991	1.61927	2.61536	0.84611
Deaths	Low-middl	Both	50-69 yea	HIV/AIDS	Number	1993	17176.7	25284.7	10474.8
Deaths	Low-middl	Both	50-69 yea	HIV/AIDS	Rate	1993	14.5579	21.4298	8.87777
Deaths	High SDI	Both	50-69 yea	Sexually tra	Number	1993	128.97	134.763	109.048
Deaths	High SDI	Both	50-69 yea	Sexually tra	Rate	1993	0.08141	0.08507	0.06883
Deaths	Low SDI	Both	50-69 yea	HIV/AIDS	Number	1992	36506.5	49290.8	24896.3
Deaths	Low SDI	Both	50-69 yea	HIV/AIDS	Rate	1992	80.2186	108.31	54.7064
Deaths	Low-middl	Both	50-69 yea	Sexually tra	Number	1993	897.55	1112.02	657.317
Deaths	Low-middl	Both	50-69 yea	Sexually tra	Rate	1993	0.76071	0.94248	0.5571
Deaths	High-midc	Both	50-69 yea	Sexually tra	Number	1992	469.762	508.795	337.479
Deaths	High-midc	Both	50-69 yea	Sexually tra	Rate	1992	0.24641	0.26688	0.17702
Deaths	Middle SDI	Both	50-69 yea	HIV/AIDS	Number	1991	3035.45	4136.94	2141.59
Deaths	Middle SDI	Both	50-69 yea	HIV/AIDS	Rate	1991	1.57983	2.15311	1.11461
Deaths	Middle SDI	Both	50-69 yea	Sexually tra	Number	1991	541.9	630.222	395.051
Deaths	Middle SDI	Both	50-69 yea	Sexually tra	Rate	1991	0.28204	0.328	0.20561
Deaths	Low SDI	Both	50-69 yea	Sexually tra	Number	1992	726.084	1176.34	379.687
Deaths	Low SDI	Both	50-69 yea	Sexually tra	Rate	1992	1.59548	2.58486	0.83432
Deaths	Middle SDI	Both	50-69 yea	HIV/AIDS	Number	1993	6608.98	8946.03	4603.79
Deaths	Middle SDI	Both	50-69 yea	HIV/AIDS	Rate	1993	3.29738	4.46339	2.29694
Deaths	Middle SDI	Both	50-69 yea	Sexually tra	Number	1996	451.435	522.279	371.734
Deaths	Middle SDI	Both	50-69 yea	Sexually tra	Rate	1996	0.21163	0.24484	0.17427
Deaths	Low-middl	Both	50-69 yea	HIV/AIDS	Number	1992	12446.6	18908.5	7427.65
Deaths	Low-middl	Both	50-69 yea	HIV/AIDS	Rate	1992	10.7729	16.3659	6.42888
Deaths	High-midc	Both	50-69 yea	Sexually tra	Number	1994	549.908	603.474	360.872
Deaths	High-midc	Both	50-69 yea	Sexually tra	Rate	1994	0.28718	0.31516	0.18846
Deaths	Low-middl	Both	50-69 yea	Sexually tra	Number	1992	904.453	1133.27	671.013
Deaths	Low-middl	Both	50-69 yea	Sexually tra	Rate	1992	0.78283	0.98088	0.58078
Deaths	Low SDI	Both	50-69 yea	HIV/AIDS	Number	1993	43838.3	58288.2	30673.7
Deaths	Low SDI	Both	50-69 yea	HIV/AIDS	Rate	1993	94.7921	126.037	66.3259
Deaths	High-midc	Both	50-69 yea	HIV/AIDS	Number	1996	4601.95	5051.81	4288.5
Deaths	High-midc	Both	50-69 yea	HIV/AIDS	Rate	1996	2.38225	2.61513	2.21999
Deaths	High SDI	Both	50-69 yea	HIV/AIDS	Number	1993	7432.28	7507.1	7358.99
Deaths	High SDI	Both	50-69 yea	HIV/AIDS	Rate	1993	4.69153	4.73876	4.64527
Deaths	High SDI	Both	50-69 yea	HIV/AIDS	Number	1995	8835.34	8919.51	8753.48
Deaths	High SDI	Both	50-69 yea	HIV/AIDS	Rate	1995	5.47125	5.52337	5.42056
Deaths	High SDI	Both	50-69 yea	Sexually tra	Number	1997	104.199	108.77	97.9803
Deaths	High SDI	Both	50-69 yea	Sexually tra	Rate	1997	0.0626	0.06535	0.05887
Deaths	Middle SDI	Both	50-69 yea	Sexually tra	Number	1993	522.785	602.382	389.226
Deaths	Middle SDI	Both	50-69 yea	Sexually tra	Rate	1993	0.26083	0.30054	0.19419
Deaths	High-midc	Both	50-69 yea	Sexually tra	Number	1998	452.153	501.732	297.294
Deaths	High-midc	Both	50-69 yea	Sexually tra	Rate	1998	0.22789	0.25288	0.14984
Deaths	High-midc	Both	50-69 yea	Sexually tra	Number	1990	400.445	435.41	327.651
Deaths	High-midc	Both	50-69 yea	Sexually tra	Rate	1990	0.21512	0.23391	0.17602
Deaths	High-midc	Both	50-69 yea	HIV/AIDS	Number	1990	1988.91	2094.02	1902.58
Deaths	High-midc	Both	50-69 yea	HIV/AIDS	Rate	1990	1.06847	1.12494	1.02209
Deaths	Middle SDI	Both	50-69 yea	HIV/AIDS	Number	1995	12743.6	16542.8	9078.49
Deaths	Middle SDI	Both	50-69 yea	HIV/AIDS	Rate	1995	6.11087	7.93272	4.35337
Deaths	Middle SDI	Both	50-69 yea	Sexually tra	Number	1995	475.902	549.501	384.861
Deaths	Middle SDI	Both	50-69 yea	Sexually tra	Rate	1995	0.22821	0.2635	0.18455
Deaths	Low-middl	Both	50-69 yea	Sexually tra	Number	1996	916.194	1111.21	663.384
Deaths	Low-middl	Both	50-69 yea	Sexually tra	Rate	1996	0.73098	0.88657	0.52928
Deaths	Middle SDI	Both	50-69 yea	Sexually tra	Number	1992	538.163	625.257	399.24
Deaths	Middle SDI	Both	50-69 yea	Sexually tra	Rate	1992	0.2741	0.31846	0.20334
Deaths	Middle SDI	Both	50-69 yea	HIV/AIDS	Number	1992	4512.13	6135.21	3162.61
Deaths	Middle SDI	Both	50-69 yea	HIV/AIDS	Rate	1992	2.29811	3.12478	1.61078
Deaths	Low-middl	Both	50-69 yea	HIV/AIDS	Number	1994	22625.6	32276.8	14209.4
Deaths	Low-middl	Both	50-69 yea	HIV/AIDS	Rate	1994	18.7864	26.8001	11.7984
Deaths	High SDI	Both	50-69 yea	HIV/AIDS	Number	1999	4805.42	4854.68	4759.69
Deaths	High SDI	Both	50-69 yea	HIV/AIDS	Rate	1999	2.7569	2.78516	2.73066
Deaths	Low SDI	Both	50-69 yea	Sexually tra	Number	1996	729.089	1182.65	385.582
Deaths	Low SDI	Both	50-69 yea	Sexually tra	Rate	1996	1.49403	2.42345	0.79012
Deaths	Low SDI	Both	50-69 yea	HIV/AIDS	Number	1996	66014.9	82080.7	49926.3
Deaths	Low SDI	Both	50-69 yea	HIV/AIDS	Rate	1996	135.275	168.197	102.307
Deaths	Middle SDI	Both	50-69 yea	HIV/AIDS	Number	1996	16934.5	21588.1	12352.8
Deaths	Middle SDI	Both	50-69 yea	HIV/AIDS	Rate	1996	7.93879	10.1203	5.79094
Deaths	Low-middl	Both	50-69 yea	HIV/AIDS	Number	1998	47301.5	59482.7	34562.5
Deaths	Low-middl	Both	50-69 yea	HIV/AIDS	Rate	1998	36.2569	45.5938	26.4923
Deaths	High-midc	Both	50-69 yea	HIV/AIDS	Number	2000	5731.71	6315.15	5225.29
Deaths	High-midc	Both	50-69 yea	HIV/AIDS	Rate	2000	2.79375	3.07813	2.54691
Deaths	Low SDI	Both	50-69 yea	Sexually tra	Number	1993	722.095	1174.95	376.09
Deaths	Low SDI	Both	50-69 yea	Sexually tra	Rate	1993	1.56139	2.54061	0.81322
Deaths	Low SDI	Both	50-69 yea	Sexually tra	Number	1995	726.909	1175.9	385.461
Deaths	Low SDI	Both	50-69 yea	Sexually tra	Rate	1995	1.51676	2.45362	0.8043
Deaths	Low SDI	Both	50-69 yea	HIV/AIDS	Number	1997	72420.7	88743.1	56204
Deaths	Low SDI	Both	50-69 yea	HIV/AIDS	Rate	1997	145.739	178.586	113.105
Deaths	High-midc	Both	50-69 yea	HIV/AIDS	Number	1994	3920.94	4257.85	3685.32
Deaths	High-midc	Both	50-69 yea	HIV/AIDS	Rate	1994	2.04767	2.22362	1.92462
Deaths	Middle SDI	Both	50-69 yea	HIV/AIDS	Number	2002	57575.1	66506.7	48061.6
Deaths	Middle SDI	Both	50-69 yea	HIV/AIDS	Rate	2002	22.7348	26.2617	18.9782
Deaths	High SDI	Both	50-69 yea	HIV/AIDS	Number	1994	8465.26	8547.75	8378.4
Deaths	High SDI	Both	50-69 yea	HIV/AIDS	Rate	1994	5.28123	5.33269	5.22704
Deaths	High SDI	Both	50-69 yea	Sexually tra	Number	1994	122.98	128.902	104.825
Deaths	High SDI	Both	50-69 yea	Sexually tra	Rate	1994	0.07672	0.08042	0.0654
Deaths	Low-middl	Both	50-69 yea	Sexually tra	Number	1994	901.206	1112.81	671.595
Deaths	Low-middl	Both	50-69 yea	Sexually tra	Rate	1994	0.74829	0.92399	0.55764
Deaths	High SDI	Both	50-69 yea	Sexually tra	Number	2001	93.2303	98.2305	89.5593
Deaths	High SDI	Both	50-69 yea	Sexually tra	Rate	2001	0.05126	0.05401	0.04924
Deaths	Low SDI	Both	50-69 yea	Sexually tra	Number	1994	723.339	1175.52	376.534
Deaths	Low SDI	Both	50-69 yea	Sexually tra	Rate	1994	1.53672	2.49737	0.79994
Deaths	Low SDI	Both	50-69 yea	Sexually tra	Number	1999	707.693	1149.35	382.826
Deaths	Low SDI	Both	50-69 yea	Sexually tra	Rate	1999	1.37692	2.23623	0.74484
Deaths	Low SDI	Both	50-69 yea	HIV/AIDS	Number	1994	51445.6	66898.8	36906.4
Deaths	Low SDI	Both	50-69 yea	HIV/AIDS	Rate	1994	109.295	142.125	78.407
Deaths	High-midc	Both	50-69 yea	Sexually tra	Number	2002	360.182	387.445	287.133
Deaths	High-midc	Both	50-69 yea	Sexually tra	Rate	2002	0.16723	0.17989	0.13331
Deaths	Low SDI	Both	50-69 yea	Sexually tra	Number	2003	669.571	1065.49	377.078
Deaths	Low SDI	Both	50-69 yea	Sexually tra	Rate	2003	1.18607	1.88739	0.66795
Deaths	High-midc	Both	50-69 yea	HIV/AIDS	Number	1995	4340.46	4754.99	4082.51
Deaths	High-midc	Both	50-69 yea	HIV/AIDS	Rate	1995	2.26327	2.47942	2.12877
Deaths	High SDI	Both	50-69 yea	HIV/AIDS	Number	1997	4580.64	4623.37	4537.47
Deaths	High SDI	Both	50-69 yea	HIV/AIDS	Rate	1997	2.75213	2.7778	2.72619
Deaths	Middle SDI	Both	50-69 yea	HIV/AIDS	Number	1998	27985.4	34445.2	21434.8
Deaths	Middle SDI	Both	50-69 yea	HIV/AIDS	Rate	1998	12.4945	15.3785	9.56986
Deaths	Low-middl	Both	50-69 yea	Sexually tra	Number	2000	887.779	1086.2	662.492
Deaths	Low-middl	Both	50-69 yea	Sexually tra	Rate	2000	0.65624	0.80291	0.48971
Deaths	High-midc	Both	50-69 yea	Sexually tra	Number	1995	549.769	607.736	356.162
Deaths	High-midc	Both	50-69 yea	Sexually tra	Rate	1995	0.28667	0.31689	0.18572
Deaths	Middle SDI	Both	50-69 yea	HIV/AIDS	Number	1994	9316.91	12315.4	6538.17
Deaths	Middle SDI	Both	50-69 yea	HIV/AIDS	Rate	1994	4.55763	6.0244	3.19833
Deaths	High-midc	Both	50-69 yea	HIV/AIDS	Number	2004	7691.74	8367.73	7105.36
Deaths	High-midc	Both	50-69 yea	HIV/AIDS	Rate	2004	3.37987	3.67691	3.1222
Deaths	High SDI	Both	50-69 yea	HIV/AIDS	Number	2003	5437.59	5487.9	5383.85
Deaths	High SDI	Both	50-69 yea	HIV/AIDS	Rate	2003	2.86556	2.89208	2.83725
Deaths	High SDI	Both	50-69 yea	Sexually tra	Number	1995	115.822	121.158	101.08
Deaths	High SDI	Both	50-69 yea	Sexually tra	Rate	1995	0.07172	0.07503	0.06259
Deaths	Low-middl	Both	50-69 yea	HIV/AIDS	Number	1997	41106.6	53015.2	29030.9
Deaths	Low-middl	Both	50-69 yea	HIV/AIDS	Rate	1997	32.1211	41.4267	22.6851
Deaths	Middle SDI	Both	50-69 yea	Sexually tra	Number	1994	512.406	591.059	384.083
Deaths	Middle SDI	Both	50-69 yea	Sexually tra	Rate	1994	0.25066	0.28913	0.18788
Deaths	Low-middl	Both	50-69 yea	Sexually tra	Number	1995	896.111	1093.68	667.196
Deaths	Low-middl	Both	50-69 yea	Sexually tra	Rate	1995	0.72945	0.89027	0.54311
Deaths	High-midc	Both	50-69 yea	Sexually tra	Number	1996	518.961	569.296	330.447
Deaths	High-midc	Both	50-69 yea	Sexually tra	Rate	1996	0.26865	0.2947	0.17106
Deaths	Low-middl	Both	50-69 yea	Sexually tra	Number	1997	944.299	1147.05	697.19
Deaths	Low-middl	Both	50-69 yea	Sexually tra	Rate	1997	0.73789	0.89632	0.54479
Deaths	Low SDI	Both	50-69 yea	HIV/AIDS	Number	2001	88987.6	101751	76116.9
Deaths	Low SDI	Both	50-69 yea	HIV/AIDS	Rate	2001	166.054	189.871	142.037
Deaths	Low-middl	Both	50-69 yea	HIV/AIDS	Number	2002	67962.8	77112.7	57242.9
Deaths	Low-middl	Both	50-69 yea	HIV/AIDS	Rate	2002	47.6454	54.06	40.1302
Deaths	Middle SDI	Both	50-69 yea	Sexually tra	Number	2000	434.842	498.373	362.039
Deaths	Middle SDI	Both	50-69 yea	Sexually tra	Rate	2000	0.18348	0.21028	0.15276
Deaths	Low-middl	Both	50-69 yea	Sexually tra	Number	2004	824.455	995.739	647.01
Deaths	Low-middl	Both	50-69 yea	Sexually tra	Rate	2004	0.54699	0.66063	0.42926
Deaths	Low-middl	Both	50-69 yea	HIV/AIDS	Number	1995	28560.7	39509.6	18673.1
Deaths	Low-middl	Both	50-69 yea	HIV/AIDS	Rate	1995	23.2489	32.1615	15.2002
Deaths	Low SDI	Both	50-69 yea	Sexually tra	Number	1997	728.295	1199.15	390.97
Deaths	Low SDI	Both	50-69 yea	Sexually tra	Rate	1997	1.46562	2.41317	0.78679
Deaths	High SDI	Both	50-69 yea	HIV/AIDS	Number	1996	7065.24	7136.22	6997.78
Deaths	High SDI	Both	50-69 yea	HIV/AIDS	Rate	1996	4.34148	4.38509	4.30002
Deaths	Middle SDI	Both	50-69 yea	HIV/AIDS	Number	1997	22017.8	27608.3	16497
Deaths	Middle SDI	Both	50-69 yea	HIV/AIDS	Rate	1997	10.0816	12.6414	7.55374
Deaths	High-midc	Both	50-69 yea	Sexually tra	Number	2006	251.744	277.802	237.465
Deaths	High-midc	Both	50-69 yea	Sexually tra	Rate	2006	0.10458	0.1154	0.09865
Deaths	Low SDI	Both	50-69 yea	HIV/AIDS	Number	1995	58963.7	74936.2	43508.3
Deaths	Low SDI	Both	50-69 yea	HIV/AIDS	Rate	1995	123.033	156.362	90.7842
Deaths	High SDI	Both	50-69 yea	Sexually tra	Number	2005	93.0378	97.4974	89.3418
Deaths	High SDI	Both	50-69 yea	Sexually tra	Rate	2005	0.04697	0.04922	0.0451
Deaths	Middle SDI	Both	50-69 yea	Sexually tra	Number	2004	435.024	492.428	359.325
Deaths	Middle SDI	Both	50-69 yea	Sexually tra	Rate	2004	0.15977	0.18085	0.13197
Deaths	Low-middl	Both	50-69 yea	HIV/AIDS	Number	1996	34833.3	46674.5	23651.4
Deaths	Low-middl	Both	50-69 yea	HIV/AIDS	Rate	1996	27.7916	37.239	18.8702
Deaths	High-midc	Both	50-69 yea	HIV/AIDS	Number	1998	4431.66	4962.67	3990.84
Deaths	High-midc	Both	50-69 yea	HIV/AIDS	Rate	1998	2.23359	2.50123	2.01141
Deaths	Low-middl	Both	50-69 yea	HIV/AIDS	Number	2006	71606.4	78104.5	65604.7
Deaths	Low-middl	Both	50-69 yea	HIV/AIDS	Rate	2006	44.902	48.9767	41.1385
Deaths	Middle SDI	Both	50-69 yea	Sexually tra	Number	1997	444.508	523.591	373.907
Deaths	Middle SDI	Both	50-69 yea	Sexually tra	Rate	1997	0.20353	0.23974	0.17121
Deaths	High SDI	Both	50-69 yea	HIV/AIDS	Number	1998	4226.65	4266.12	4186.04
Deaths	High SDI	Both	50-69 yea	HIV/AIDS	Rate	1998	2.48091	2.50408	2.45708
Deaths	Low SDI	Both	50-69 yea	HIV/AIDS	Number	2005	92725.7	102074	84487.6
Deaths	Low SDI	Both	50-69 yea	HIV/AIDS	Rate	2005	156.009	171.737	142.148
Deaths	High SDI	Both	50-69 yea	Sexually tra	Number	1996	108.682	113.477	99.7886
Deaths	High SDI	Both	50-69 yea	Sexually tra	Rate	1996	0.06678	0.06973	0.06132
Deaths	Middle SDI	Both	50-69 yea	HIV/AIDS	Number	2006	74883.8	81399.3	68800.7
Deaths	Middle SDI	Both	50-69 yea	HIV/AIDS	Rate	2006	25.5785	27.804	23.5006
Deaths	High SDI	Both	50-69 yea	Sexually tra	Number	1999	98.2326	102.69	93.8904
Deaths	High SDI	Both	50-69 yea	Sexually tra	Rate	1999	0.05636	0.05891	0.05387
Deaths	High SDI	Both	50-69 yea	HIV/AIDS	Number	2007	5828.71	5895.87	5769.43
Deaths	High SDI	Both	50-69 yea	HIV/AIDS	Rate	2007	2.82296	2.85549	2.79425
Deaths	High SDI	Both	50-69 yea	Sexually tra	Number	1998	99.931	104.406	95.4322
Deaths	High SDI	Both	50-69 yea	Sexually tra	Rate	1998	0.05866	0.06128	0.05602
Deaths	High-midc	Both	50-69 yea	Sexually tra	Number	1999	446.331	493.526	296.048
Deaths	High-midc	Both	50-69 yea	Sexually tra	Rate	1999	0.22104	0.24441	0.14661
Deaths	Low-middl	Both	50-69 yea	Sexually tra	Number	1998	936.064	1148.88	687.202
Deaths	Low-middl	Both	50-69 yea	Sexually tra	Rate	1998	0.7175	0.88062	0.52674
Deaths	Low SDI	Both	50-69 yea	Sexually tra	Number	2007	627.138	897.31	361.028
Deaths	Low SDI	Both	50-69 yea	Sexually tra	Rate	2007	0.99842	1.42855	0.57477
Deaths	Low SDI	Both	50-69 yea	Sexually tra	Number	1998	721.209	1173.12	386.945
Deaths	Low SDI	Both	50-69 yea	Sexually tra	Rate	1998	1.42665	2.32058	0.76543
Deaths	Low SDI	Both	50-69 yea	HIV/AIDS	Number	1999	82020.9	96632.8	66969.8
Deaths	Low SDI	Both	50-69 yea	HIV/AIDS	Rate	1999	159.584	188.013	130.3
Deaths	Low SDI	Both	50-69 yea	HIV/AIDS	Number	1998	77810.2	93424.7	62016
Deaths	Low SDI	Both	50-69 yea	HIV/AIDS	Rate	1998	153.919	184.806	122.676
Deaths	High-midc	Both	50-69 yea	HIV/AIDS	Number	1999	5118	5668.94	4659.16
Deaths	High-midc	Both	50-69 yea	HIV/AIDS	Rate	1999	2.5346	2.80744	2.30736
Deaths	Middle SDI	Both	50-69 yea	Sexually tra	Number	1998	439.092	504.941	373.964
Deaths	Middle SDI	Both	50-69 yea	Sexually tra	Rate	1998	0.19604	0.22544	0.16696
Deaths	Low-middl	Both	50-69 yea	HIV/AIDS	Number	1999	52890	64436.7	40134.7
Deaths	Low-middl	Both	50-69 yea	HIV/AIDS	Rate	1999	39.7411	48.4172	30.1569
Deaths	High-midc	Both	50-69 yea	HIV/AIDS	Number	2008	8543.06	9307.06	7889.2
Deaths	High-midc	Both	50-69 yea	HIV/AIDS	Rate	2008	3.38035	3.68266	3.12163
Deaths	High-midc	Both	50-69 yea	HIV/AIDS	Number	1997	4433.93	4965.85	4058.75
Deaths	High-midc	Both	50-69 yea	HIV/AIDS	Rate	1997	2.26436	2.536	2.07276
Deaths	High-midc	Both	50-69 yea	Sexually tra	Number	1997	487.048	535.091	314.862
Deaths	High-midc	Both	50-69 yea	Sexually tra	Rate	1997	0.24873	0.27326	0.1608
Deaths	High-midc	Both	50-69 yea	Sexually tra	Number	2000	424.187	466.653	291.396
Deaths	High-midc	Both	50-69 yea	Sexually tra	Rate	2000	0.20676	0.22746	0.14203
Deaths	Low-middl	Both	50-69 yea	Sexually tra	Number	2008	851.058	1008.54	665.557
Deaths	Low-middl	Both	50-69 yea	Sexually tra	Rate	2008	0.50437	0.5977	0.39443
Deaths	Middle SDI	Both	50-69 yea	Sexually tra	Number	2008	439.797	492.466	353.874
Deaths	Middle SDI	Both	50-69 yea	Sexually tra	Rate	2008	0.14106	0.15796	0.1135
Deaths	High SDI	Both	50-69 yea	Sexually tra	Number	2009	92.6708	96.9848	88.9137
Deaths	High SDI	Both	50-69 yea	Sexually tra	Rate	2009	0.04305	0.04505	0.0413
Deaths	Low-middl	Both	50-69 yea	Sexually tra	Number	1999	906.665	1100.91	679.718
Deaths	Low-middl	Both	50-69 yea	Sexually tra	Rate	1999	0.68126	0.82721	0.51073
Deaths	Middle SDI	Both	50-69 yea	HIV/AIDS	Number	1999	34751.5	42063.2	27169.1
Deaths	Middle SDI	Both	50-69 yea	HIV/AIDS	Rate	1999	15.0448	18.2102	11.7622
Deaths	Middle SDI	Both	50-69 yea	Sexually tra	Number	2002	441.837	504.504	363.768
Deaths	Middle SDI	Both	50-69 yea	Sexually tra	Rate	2002	0.17447	0.19921	0.14364
Deaths	High SDI	Both	50-69 yea	HIV/AIDS	Number	2000	4801.78	4847.24	4754.47
Deaths	High SDI	Both	50-69 yea	HIV/AIDS	Rate	2000	2.69449	2.72	2.66794
Deaths	Low SDI	Both	50-69 yea	HIV/AIDS	Number	2009	69562.3	80755.6	61151.8
Deaths	Low SDI	Both	50-69 yea	HIV/AIDS	Rate	2009	104.348	121.139	91.732
Deaths	Low-middl	Both	50-69 yea	HIV/AIDS	Number	2000	57984.2	68954.5	45443
Deaths	Low-middl	Both	50-69 yea	HIV/AIDS	Rate	2000	42.8613	50.9704	33.5909
Deaths	Low-middl	Both	50-69 yea	HIV/AIDS	Number	2010	55524.9	64842.5	48763.6
Deaths	Low-middl	Both	50-69 yea	HIV/AIDS	Rate	2010	31.0352	36.2432	27.256
Deaths	High-midc	Both	50-69 yea	Sexually tra	Number	2010	235.231	251.316	220.548
Deaths	High-midc	Both	50-69 yea	Sexually tra	Rate	2010	0.08866	0.09472	0.08312
Deaths	Middle SDI	Both	50-69 yea	HIV/AIDS	Number	2000	42187.7	50156.3	33595.2
Deaths	Middle SDI	Both	50-69 yea	HIV/AIDS	Rate	2000	17.8005	21.1628	14.1751
Deaths	Low SDI	Both	50-69 yea	Sexually tra	Number	2000	691.249	1146.98	370.396
Deaths	Low SDI	Both	50-69 yea	Sexually tra	Rate	2000	1.32545	2.1993	0.71022
Deaths	High SDI	Both	50-69 yea	Sexually tra	Number	2000	95.0074	99.3448	91.2063
Deaths	High SDI	Both	50-69 yea	Sexually tra	Rate	2000	0.05331	0.05575	0.05118
Deaths	Middle SDI	Both	50-69 yea	HIV/AIDS	Number	2010	65125.8	75200.6	56103.6
Deaths	Middle SDI	Both	50-69 yea	HIV/AIDS	Rate	2010	19.6928	22.7392	16.9647
Deaths	Low SDI	Both	50-69 yea	HIV/AIDS	Number	2003	93307.4	103314	83681.9
Deaths	Low SDI	Both	50-69 yea	HIV/AIDS	Rate	2003	165.284	183.01	148.233
Deaths	Middle SDI	Both	50-69 yea	Sexually tra	Number	1999	435.182	502.386	364.106
Deaths	Middle SDI	Both	50-69 yea	Sexually tra	Rate	1999	0.1884	0.21749	0.15763
Deaths	High-midc	Both	50-69 yea	Sexually tra	Number	2001	389.067	423.508	287.833
Deaths	High-midc	Both	50-69 yea	Sexually tra	Rate	2001	0.18525	0.20165	0.13705
Deaths	Low-middl	Both	50-69 yea	HIV/AIDS	Number	2003	71561.1	79868	62241
Deaths	Low-middl	Both	50-69 yea	HIV/AIDS	Rate	2003	48.821	54.4882	42.4625
Deaths	High SDI	Both	50-69 yea	HIV/AIDS	Number	2011	5357.79	5454.27	5294.9
Deaths	High SDI	Both	50-69 yea	HIV/AIDS	Rate	2011	2.39074	2.43379	2.36268
Deaths	High SDI	Both	50-69 yea	HIV/AIDS	Number	2001	4974.16	5019.3	4926.85
Deaths	High SDI	Both	50-69 yea	HIV/AIDS	Rate	2001	2.73493	2.75975	2.70892
Deaths	High-midc	Both	50-69 yea	HIV/AIDS	Number	2001	6358	6964.05	5849.53
Deaths	High-midc	Both	50-69 yea	HIV/AIDS	Rate	2001	3.02729	3.31585	2.78518
Deaths	Low SDI	Both	50-69 yea	HIV/AIDS	Number	2000	85254.5	98973.7	71337.7
Deaths	Low SDI	Both	50-69 yea	HIV/AIDS	Rate	2000	163.473	189.779	136.788
Deaths	Low SDI	Both	50-69 yea	Sexually tra	Number	2011	613.583	835.465	364.76
Deaths	Low SDI	Both	50-69 yea	Sexually tra	Rate	2011	0.86489	1.17766	0.51416
Deaths	High-midc	Both	50-69 yea	Sexually tra	Number	2004	295.625	313.728	275.527
Deaths	High-midc	Both	50-69 yea	Sexually tra	Rate	2004	0.1299	0.13786	0.12107
Deaths	High SDI	Both	50-69 yea	HIV/AIDS	Number	2002	5177.61	5227.38	5128.83
Deaths	High SDI	Both	50-69 yea	HIV/AIDS	Rate	2002	2.78672	2.8135	2.76046
Deaths	High-midc	Both	50-69 yea	HIV/AIDS	Number	2002	6829.67	7469.5	6309.46
Deaths	High-midc	Both	50-69 yea	HIV/AIDS	Rate	2002	3.17092	3.46799	2.92939
Deaths	Low SDI	Both	50-69 yea	Sexually tra	Number	2001	683.742	1115.28	372.64
Deaths	Low SDI	Both	50-69 yea	Sexually tra	Rate	2001	1.27589	2.08116	0.69536
Deaths	Middle SDI	Both	50-69 yea	Sexually tra	Number	2003	440.535	501.092	364.1
Deaths	Middle SDI	Both	50-69 yea	Sexually tra	Rate	2003	0.16788	0.19096	0.13875
Deaths	High SDI	Both	50-69 yea	Sexually tra	Number	2002	90.9025	96.4462	87.3089
Deaths	High SDI	Both	50-69 yea	Sexually tra	Rate	2002	0.04893	0.05191	0.04699
Deaths	Middle SDI	Both	50-69 yea	HIV/AIDS	Number	2003	64498	73587	54978.3
Deaths	Middle SDI	Both	50-69 yea	HIV/AIDS	Rate	2003	24.5788	28.0424	20.9511
Deaths	High-midc	Both	50-69 yea	HIV/AIDS	Number	2012	9068.16	9896.84	8466.17
Deaths	High-midc	Both	50-69 yea	HIV/AIDS	Rate	2012	3.23119	3.52646	3.01668
Deaths	High SDI	Both	50-69 yea	Sexually tra	Number	2004	91.8825	96.7356	88.2594
Deaths	High SDI	Both	50-69 yea	Sexually tra	Rate	2004	0.0474	0.04991	0.04553
Deaths	Low-middl	Both	50-69 yea	HIV/AIDS	Number	2001	63393	73938.2	51659.5
Deaths	Low-middl	Both	50-69 yea	HIV/AIDS	Rate	2001	45.6377	53.2294	37.1906
Deaths	Low-middl	Both	50-69 yea	Sexually tra	Number	2001	887.069	1073.52	673.068
Deaths	Low-middl	Both	50-69 yea	Sexually tra	Rate	2001	0.63862	0.77285	0.48455
Deaths	High SDI	Both	50-69 yea	HIV/AIDS	Number	2004	5605.94	5662.9	5552.64
Deaths	High SDI	Both	50-69 yea	HIV/AIDS	Rate	2004	2.89206	2.92145	2.86456
Deaths	Low-middl	Both	50-69 yea	Sexually tra	Number	2012	864.483	1029.29	671.986
Deaths	Low-middl	Both	50-69 yea	Sexually tra	Rate	2012	0.45392	0.54045	0.35284
Deaths	Low SDI	Both	50-69 yea	Sexually tra	Number	2002	675.053	1082.56	374.567
Deaths	Low SDI	Both	50-69 yea	Sexually tra	Rate	2002	1.22676	1.96732	0.68069
Deaths	High-midc	Both	50-69 yea	HIV/AIDS	Number	2003	7458.73	8083.14	6874.68
Deaths	High-midc	Both	50-69 yea	HIV/AIDS	Rate	2003	3.37202	3.65431	3.10798
Deaths	Low-middl	Both	50-69 yea	Sexually tra	Number	2002	880.017	1061.33	671.312
Deaths	Low-middl	Both	50-69 yea	Sexually tra	Rate	2002	0.61694	0.74405	0.47062
Deaths	Low SDI	Both	50-69 yea	HIV/AIDS	Number	2002	91445.1	102762	80138.2
Deaths	Low SDI	Both	50-69 yea	HIV/AIDS	Rate	2002	166.181	186.746	145.633
Deaths	Low-middl	Both	50-69 yea	HIV/AIDS	Number	2004	73500.6	80383.9	66286.8
Deaths	Low-middl	Both	50-69 yea	HIV/AIDS	Rate	2004	48.7644	53.3311	43.9783
Deaths	Middle SDI	Both	50-69 yea	Sexually tra	Number	2012	472.189	530.115	370.132
Deaths	Middle SDI	Both	50-69 yea	Sexually tra	Rate	2012	0.13351	0.14989	0.10465
Deaths	High SDI	Both	50-69 yea	Sexually tra	Number	2013	93.2069	97.587	88.9096
Deaths	High SDI	Both	50-69 yea	Sexually tra	Rate	2013	0.04002	0.0419	0.03817
Deaths	Low SDI	Both	50-69 yea	HIV/AIDS	Number	2004	94343.4	103242	85521.8
Deaths	Low SDI	Both	50-69 yea	HIV/AIDS	Rate	2004	162.92	178.287	147.687
Deaths	Low SDI	Both	50-69 yea	HIV/AIDS	Number	2013	60468.1	69848.3	54327.4
Deaths	Low SDI	Both	50-69 yea	HIV/AIDS	Rate	2013	79.9046	92.2999	71.79
Deaths	Middle SDI	Both	50-69 yea	HIV/AIDS	Number	2001	50256.6	58842.2	40922.8
Deaths	Middle SDI	Both	50-69 yea	HIV/AIDS	Rate	2001	20.5203	24.0258	16.7092
Deaths	High SDI	Both	50-69 yea	HIV/AIDS	Number	2005	5715.46	5781.15	5659.42
Deaths	High SDI	Both	50-69 yea	HIV/AIDS	Rate	2005	2.88534	2.9185	2.85705
Deaths	High SDI	Both	50-69 yea	Sexually tra	Number	2003	91.8365	96.8356	88.2245
Deaths	High SDI	Both	50-69 yea	Sexually tra	Rate	2003	0.0484	0.05103	0.04649
Deaths	High-midc	Both	50-69 yea	Sexually tra	Number	2003	325.204	346.819	284.574
Deaths	High-midc	Both	50-69 yea	Sexually tra	Rate	2003	0.14702	0.15679	0.12865
Deaths	High-midc	Both	50-69 yea	HIV/AIDS	Number	2005	8152.74	8824.29	7524.01
Deaths	High-midc	Both	50-69 yea	HIV/AIDS	Rate	2005	3.48024	3.7669	3.21184
Deaths	Low SDI	Both	50-69 yea	Sexually tra	Number	2004	650.138	1018.17	362.665
Deaths	Low SDI	Both	50-69 yea	Sexually tra	Rate	2004	1.12271	1.75826	0.62628
Deaths	Low-middl	Both	50-69 yea	Sexually tra	Number	2003	857.319	1028.18	673.769
Deaths	Low-middl	Both	50-69 yea	Sexually tra	Rate	2003	0.58489	0.70145	0.45966
Deaths	Middle SDI	Both	50-69 yea	HIV/AIDS	Number	2004	70334	79096	61408.4
Deaths	Middle SDI	Both	50-69 yea	HIV/AIDS	Rate	2004	25.8308	29.0487	22.5528
Deaths	Middle SDI	Both	50-69 yea	Sexually tra	Number	2001	436.239	501.405	363.349
Deaths	Middle SDI	Both	50-69 yea	Sexually tra	Rate	2001	0.17812	0.20473	0.14836
Deaths	High-midc	Both	50-69 yea	HIV/AIDS	Number	2006	8180.59	8852.59	7539.94
Deaths	High-midc	Both	50-69 yea	HIV/AIDS	Rate	2006	3.39836	3.67752	3.13222
Deaths	High-midc	Both	50-69 yea	Sexually tra	Number	2005	269.413	291.594	253.573
Deaths	High-midc	Both	50-69 yea	Sexually tra	Rate	2005	0.11501	0.12448	0.10825
Deaths	High-midc	Both	50-69 yea	Sexually tra	Number	2014	236.042	254.14	219.452
Deaths	High-midc	Both	50-69 yea	Sexually tra	Rate	2014	0.07886	0.08491	0.07332
Deaths	Low SDI	Both	50-69 yea	Sexually tra	Number	2005	636.363	966.917	360.704
Deaths	Low SDI	Both	50-69 yea	Sexually tra	Rate	2005	1.07066	1.62681	0.60688
Deaths	High SDI	Both	50-69 yea	HIV/AIDS	Number	2006	5869.54	5935.48	5813.13
Deaths	High SDI	Both	50-69 yea	HIV/AIDS	Rate	2006	2.90218	2.93478	2.87429
Deaths	Low-middl	Both	50-69 yea	Sexually tra	Number	2005	829.081	1001.34	655.977
Deaths	Low-middl	Both	50-69 yea	Sexually tra	Rate	2005	0.53468	0.64577	0.42304
Deaths	High SDI	Both	50-69 yea	HIV/AIDS	Number	2015	5227.16	5401.02	5136.84
Deaths	High SDI	Both	50-69 yea	HIV/AIDS	Rate	2015	2.15942	2.23125	2.12211
Deaths	Low SDI	Both	50-69 yea	Sexually tra	Number	2015	637.663	858.956	368.421
Deaths	Low SDI	Both	50-69 yea	Sexually tra	Rate	2015	0.78878	1.06251	0.45573
Deaths	Low-middl	Both	50-69 yea	HIV/AIDS	Number	2014	53914.5	62324.9	48319.8
Deaths	Low-middl	Both	50-69 yea	HIV/AIDS	Rate	2014	26.4829	30.614	23.7347
Deaths	Low-middl	Both	50-69 yea	HIV/AIDS	Number	2005	73590.5	79771.5	67777
Deaths	Low-middl	Both	50-69 yea	HIV/AIDS	Rate	2005	47.4586	51.4447	43.7095
Deaths	Low-middl	Both	50-69 yea	Sexually tra	Number	2006	843.287	1015.63	656.545
Deaths	Low-middl	Both	50-69 yea	Sexually tra	Rate	2006	0.5288	0.63687	0.4117
Deaths	Middle SDI	Both	50-69 yea	HIV/AIDS	Number	2014	59761.7	68235.3	53714.1
Deaths	Middle SDI	Both	50-69 yea	HIV/AIDS	Rate	2014	15.6085	17.8217	14.029
Deaths	High SDI	Both	50-69 yea	Sexually tra	Number	2006	92.3548	96.7319	88.5967
Deaths	High SDI	Both	50-69 yea	Sexually tra	Rate	2006	0.04566	0.04783	0.04381
Deaths	Low SDI	Both	50-69 yea	Sexually tra	Number	2006	631.165	923.601	361.863
Deaths	Low SDI	Both	50-69 yea	Sexually tra	Rate	2006	1.03346	1.51229	0.59251
Deaths	Middle SDI	Both	50-69 yea	Sexually tra	Number	2005	435.712	496.846	359.578
Deaths	Middle SDI	Both	50-69 yea	Sexually tra	Rate	2005	0.15418	0.17582	0.12724
Deaths	Middle SDI	Both	50-69 yea	Sexually tra	Number	2006	438.706	495.001	356.714
Deaths	Middle SDI	Both	50-69 yea	Sexually tra	Rate	2006	0.14985	0.16908	0.12184
Deaths	Middle SDI	Both	50-69 yea	Sexually tra	Number	2016	532.744	611.627	407.257
Deaths	Middle SDI	Both	50-69 yea	Sexually tra	Rate	2016	0.12869	0.14774	0.09838
Deaths	Low SDI	Both	50-69 yea	HIV/AIDS	Number	2006	87717.6	97706.7	78678.6
Deaths	Low SDI	Both	50-69 yea	HIV/AIDS	Rate	2006	143.628	159.984	128.827
Deaths	High-midc	Both	50-69 yea	HIV/AIDS	Number	2016	10262.6	11042.1	9671.58
Deaths	High-midc	Both	50-69 yea	HIV/AIDS	Rate	2016	3.23844	3.4844	3.05192
Deaths	Middle SDI	Both	50-69 yea	HIV/AIDS	Number	2005	74157.7	81800.6	66719.1
Deaths	Middle SDI	Both	50-69 yea	HIV/AIDS	Rate	2005	26.2418	28.9463	23.6095
Deaths	Low-middl	Both	50-69 yea	Sexually tra	Number	2016	1049.76	1294.2	772.027
Deaths	Low-middl	Both	50-69 yea	Sexually tra	Rate	2016	0.48279	0.59521	0.35506
Deaths	High-midc	Both	50-69 yea	HIV/AIDS	Number	2007	8331.7	9031.36	7695.09
Deaths	High-midc	Both	50-69 yea	HIV/AIDS	Rate	2007	3.37533	3.65878	3.11743
Deaths	High SDI	Both	50-69 yea	Sexually tra	Number	2007	92.4026	96.6992	88.7606
Deaths	High SDI	Both	50-69 yea	Sexually tra	Rate	2007	0.04475	0.04683	0.04299
Deaths	Middle SDI	Both	50-69 yea	Sexually tra	Number	2009	443.834	498.471	357.993
Deaths	Middle SDI	Both	50-69 yea	Sexually tra	Rate	2009	0.13826	0.15528	0.11152
Deaths	Middle SDI	Both	50-69 yea	HIV/AIDS	Number	2009	67949	77121.7	59058.3
Deaths	Middle SDI	Both	50-69 yea	HIV/AIDS	Rate	2009	21.1674	24.0249	18.3978
Deaths	Low SDI	Both	50-69 yea	HIV/AIDS	Number	2007	81274.2	92061.3	71772.8
Deaths	Low SDI	Both	50-69 yea	HIV/AIDS	Rate	2007	129.391	146.565	114.265
Deaths	High-midc	Both	50-69 yea	Sexually tra	Number	2007	245.125	269.484	231.039
Deaths	High-midc	Both	50-69 yea	Sexually tra	Rate	2007	0.0993	0.10917	0.0936
Deaths	Low-middl	Both	50-69 yea	Sexually tra	Number	2007	854.451	1018.03	668.42
Deaths	Low-middl	Both	50-69 yea	Sexually tra	Rate	2007	0.52087	0.62059	0.40746
Deaths	Low SDI	Both	50-69 yea	HIV/AIDS	Number	2017	51430.6	61057.1	45117.4
Deaths	Low SDI	Both	50-69 yea	HIV/AIDS	Rate	2017	59.4287	70.5521	52.1336
Deaths	High SDI	Both	50-69 yea	Sexually tra	Number	2017	96.9804	102.554	91.7883
Deaths	High SDI	Both	50-69 yea	Sexually tra	Rate	2017	0.03908	0.04132	0.03699
Deaths	Low-middl	Both	50-69 yea	Sexually tra	Number	2010	835.942	1003.4	670.114
Deaths	Low-middl	Both	50-69 yea	Sexually tra	Rate	2010	0.46724	0.56085	0.37455
Deaths	High-midc	Both	50-69 yea	Sexually tra	Number	2018	240.974	266.342	217.377
Deaths	High-midc	Both	50-69 yea	Sexually tra	Rate	2018	0.07272	0.08038	0.0656
Deaths	Low-middl	Both	50-69 yea	HIV/AIDS	Number	2007	67690.7	74942.7	60887.7
Deaths	Low-middl	Both	50-69 yea	HIV/AIDS	Rate	2007	41.2637	45.6845	37.1167
Deaths	Low SDI	Both	50-69 yea	HIV/AIDS	Number	2010	67260.5	78079.8	59726.7
Deaths	Low SDI	Both	50-69 yea	HIV/AIDS	Rate	2010	97.8509	113.591	86.8907
Deaths	High SDI	Both	50-69 yea	HIV/AIDS	Number	2008	5862.47	5943.24	5805.47
Deaths	High SDI	Both	50-69 yea	HIV/AIDS	Rate	2008	2.78055	2.81886	2.75352
Deaths	Low-middl	Both	50-69 yea	HIV/AIDS	Number	2018	53972.1	63995.2	46565.7
Deaths	Low-middl	Both	50-69 yea	HIV/AIDS	Rate	2018	23.3098	27.6387	20.1111
Deaths	High SDI	Both	50-69 yea	Sexually tra	Number	2011	92.098	96.1748	88.0994
Deaths	High SDI	Both	50-69 yea	Sexually tra	Rate	2011	0.0411	0.04291	0.03931
Deaths	High-midc	Both	50-69 yea	Sexually tra	Number	2011	234.525	251.783	218.998
Deaths	High-midc	Both	50-69 yea	Sexually tra	Rate	2011	0.08605	0.09238	0.08035
Deaths	Middle SDI	Both	50-69 yea	HIV/AIDS	Number	2018	66173	74665.8	60080.9
Deaths	Middle SDI	Both	50-69 yea	HIV/AIDS	Rate	2018	14.9169	16.8313	13.5436
Deaths	High-midc	Both	50-69 yea	Sexually tra	Number	2008	241.618	261.631	227.279
Deaths	High-midc	Both	50-69 yea	Sexually tra	Rate	2008	0.0956	0.10352	0.08993
Deaths	Low SDI	Both	50-69 yea	Sexually tra	Number	2010	615.714	859.612	358.119
Deaths	Low SDI	Both	50-69 yea	Sexually tra	Rate	2010	0.89574	1.25057	0.52099
Deaths	Middle SDI	Both	50-69 yea	Sexually tra	Number	2007	439.024	490.921	356.642
Deaths	Middle SDI	Both	50-69 yea	Sexually tra	Rate	2007	0.14511	0.16226	0.11788
Deaths	High-midc	Both	50-69 yea	HIV/AIDS	Number	2011	8941.72	9702.73	8331.62
Deaths	High-midc	Both	50-69 yea	HIV/AIDS	Rate	2011	3.28084	3.56007	3.05699
Deaths	Middle SDI	Both	50-69 yea	Sexually tra	Number	2010	454.639	508.041	364.063
Deaths	Middle SDI	Both	50-69 yea	Sexually tra	Rate	2010	0.13747	0.15362	0.11009
Deaths	High SDI	Both	50-69 yea	HIV/AIDS	Number	2009	5763.83	5854.55	5706.32
Deaths	High SDI	Both	50-69 yea	HIV/AIDS	Rate	2009	2.67742	2.71956	2.65071
Deaths	High SDI	Both	50-69 yea	HIV/AIDS	Number	2019	4749.04	5032.67	4630.53
Deaths	High SDI	Both	50-69 yea	HIV/AIDS	Rate	2019	1.88553	1.99814	1.83848
Deaths	Low-middl	Both	50-69 yea	HIV/AIDS	Number	2008	63235.2	70986.7	56172.2
Deaths	Low-middl	Both	50-69 yea	HIV/AIDS	Rate	2008	37.4754	42.0693	33.2897
Deaths	High SDI	Both	50-69 yea	Sexually tra	Number	2008	91.7769	96.0963	88.1826
Deaths	High SDI	Both	50-69 yea	Sexually tra	Rate	2008	0.04353	0.04558	0.04182
Deaths	Middle SDI	Both	50-69 yea	HIV/AIDS	Number	2007	73385.4	80638.7	66558.5
Deaths	Middle SDI	Both	50-69 yea	HIV/AIDS	Rate	2007	24.2552	26.6525	21.9988
Deaths	Low-middl	Both	50-69 yea	HIV/AIDS	Number	2011	53316.9	62705.3	46953.6
Deaths	Low-middl	Both	50-69 yea	HIV/AIDS	Rate	2011	28.8993	33.9881	25.4502
Deaths	Low SDI	Both	50-69 yea	HIV/AIDS	Number	2011	65781.5	76040.4	59087.5
Deaths	Low SDI	Both	50-69 yea	HIV/AIDS	Rate	2011	92.7243	107.185	83.2885
Deaths	Low SDI	Both	50-69 yea	Sexually tra	Number	2019	638.282	867.335	378.578
Deaths	Low SDI	Both	50-69 yea	Sexually tra	Rate	2019	0.68701	0.93355	0.40748
Deaths	High-midc	Both	50-69 yea	Sexually tra	Number	2009	236.192	252.443	222.525
Deaths	High-midc	Both	50-69 yea	Sexually tra	Rate	2009	0.09129	0.09757	0.08601
Deaths	Low-middl	Both	50-69 yea	Sexually tra	Number	2011	851.639	1010.59	669.368
Deaths	Low-middl	Both	50-69 yea	Sexually tra	Rate	2011	0.46161	0.54777	0.36282
Deaths	Low SDI	Both	50-69 yea	Sexually tra	Number	2008	623.02	872.753	357.025
Deaths	Low SDI	Both	50-69 yea	Sexually tra	Rate	2008	0.96324	1.34935	0.55199
Deaths	Low SDI	Both	50-69 yea	Sexually tra	Number	2009	618.646	855.678	353.061
Deaths	Low SDI	Both	50-69 yea	Sexually tra	Rate	2009	0.92801	1.28358	0.52962
Deaths	High-midc	Both	50-69 yea	HIV/AIDS	Number	2009	8479.67	9256.33	7817.89
Deaths	High-midc	Both	50-69 yea	HIV/AIDS	Rate	2009	3.27738	3.57755	3.0216
Deaths	High-midc	Both	50-69 yea	Sexually tra	Number	2012	234.866	250.095	219.509
Deaths	High-midc	Both	50-69 yea	Sexually tra	Rate	2012	0.08369	0.08911	0.07822
Deaths	Middle SDI	Both	50-69 yea	HIV/AIDS	Number	2008	71234.4	79095.3	63338.8
Deaths	Middle SDI	Both	50-69 yea	HIV/AIDS	Rate	2008	22.8482	25.3696	20.3157
Deaths	Low SDI	Both	50-69 yea	HIV/AIDS	Number	2008	74426.9	85363.6	65201
Deaths	Low SDI	Both	50-69 yea	HIV/AIDS	Rate	2008	115.07	131.979	100.806
Deaths	High-midc	Both	50-69 yea	HIV/AIDS	Number	2010	8708.43	9496.65	8077.01
Deaths	High-midc	Both	50-69 yea	HIV/AIDS	Rate	2010	3.28217	3.57925	3.04419
Deaths	High SDI	Both	50-69 yea	HIV/AIDS	Number	2012	5300.21	5410	5236.42
Deaths	High SDI	Both	50-69 yea	HIV/AIDS	Rate	2012	2.31888	2.36691	2.29097
Deaths	Middle SDI	Both	50-69 yea	HIV/AIDS	Number	2011	62177.9	72499	53811.6
Deaths	Middle SDI	Both	50-69 yea	HIV/AIDS	Rate	2011	18.2096	21.2323	15.7594
Deaths	Middle SDI	Both	50-69 yea	Sexually tra	Number	2011	464.046	521.517	368.998
Deaths	Middle SDI	Both	50-69 yea	Sexually tra	Rate	2011	0.1359	0.15273	0.10807
Deaths	Low-middl	Both	50-69 yea	HIV/AIDS	Number	2009	59036.7	67591.9	51969.9
Deaths	Low-middl	Both	50-69 yea	HIV/AIDS	Rate	2009	33.9906	38.9162	29.9218
Deaths	High SDI	Both	50-69 yea	Sexually tra	Number	2012	91.4815	95.7482	87.1962
Deaths	High SDI	Both	50-69 yea	Sexually tra	Rate	2012	0.04002	0.04189	0.03815
Deaths	Low-middl	Both	50-69 yea	HIV/AIDS	Number	2012	52420.2	61574.2	46350.6
Deaths	Low-middl	Both	50-69 yea	HIV/AIDS	Rate	2012	27.5244	32.331	24.3374
Deaths	High SDI	Both	50-69 yea	HIV/AIDS	Number	2010	5458.61	5551.44	5395.98
Deaths	High SDI	Both	50-69 yea	HIV/AIDS	Rate	2010	2.4843	2.52655	2.45579
Deaths	Low-middl	Both	50-69 yea	Sexually tra	Number	2009	824.338	980.521	640.329
Deaths	Low-middl	Both	50-69 yea	Sexually tra	Rate	2009	0.47462	0.56454	0.36867
Deaths	High SDI	Both	50-69 yea	Sexually tra	Number	2010	92.0629	96.4057	88.3263
Deaths	High SDI	Both	50-69 yea	Sexually tra	Rate	2010	0.0419	0.04388	0.0402
Deaths	Low SDI	Both	50-69 yea	Sexually tra	Number	2012	615.307	834.447	361.268
Deaths	Low SDI	Both	50-69 yea	Sexually tra	Rate	2012	0.84001	1.13918	0.4932
Deaths	High-midc	Both	50-69 yea	Sexually tra	Number	2013	235.753	252.672	219.206
Deaths	High-midc	Both	50-69 yea	Sexually tra	Rate	2013	0.08135	0.08719	0.07564
Deaths	High SDI	Both	50-69 yea	HIV/AIDS	Number	2013	5345.22	5469.95	5272.04
Deaths	High SDI	Both	50-69 yea	HIV/AIDS	Rate	2013	2.29485	2.3484	2.26343
Deaths	High-midc	Both	50-69 yea	HIV/AIDS	Number	2013	9193.93	10006.6	8627.65
Deaths	High-midc	Both	50-69 yea	HIV/AIDS	Rate	2013	3.17257	3.45299	2.97717
Deaths	Low SDI	Both	50-69 yea	HIV/AIDS	Number	2012	63420.4	72838.8	57145.4
Deaths	Low SDI	Both	50-69 yea	HIV/AIDS	Rate	2012	86.5812	99.4391	78.0147
Deaths	Low SDI	Both	50-69 yea	Sexually tra	Number	2013	616.731	835.239	358.22
Deaths	Low SDI	Both	50-69 yea	Sexually tra	Rate	2013	0.81497	1.10371	0.47336
Deaths	Middle SDI	Both	50-69 yea	HIV/AIDS	Number	2012	59254.1	69589	51790
Deaths	Middle SDI	Both	50-69 yea	HIV/AIDS	Rate	2012	16.7541	19.6763	14.6436
Deaths	Low-middl	Both	50-69 yea	HIV/AIDS	Number	2013	52799	61671.2	47109.5
Deaths	Low-middl	Both	50-69 yea	HIV/AIDS	Rate	2013	26.824	31.3315	23.9336
Deaths	Low-middl	Both	50-69 yea	Sexually tra	Number	2013	890.466	1061.3	685.505
Deaths	Low-middl	Both	50-69 yea	Sexually tra	Rate	2013	0.45239	0.53918	0.34826
Deaths	High-midc	Both	50-69 yea	HIV/AIDS	Number	2014	9684.13	10426.5	9154.64
Deaths	High-midc	Both	50-69 yea	HIV/AIDS	Rate	2014	3.23552	3.48354	3.05861
Deaths	Middle SDI	Both	50-69 yea	HIV/AIDS	Number	2013	58458.4	67900.2	51880.2
Deaths	Middle SDI	Both	50-69 yea	HIV/AIDS	Rate	2013	15.9004	18.4685	14.1112
Deaths	Middle SDI	Both	50-69 yea	HIV/AIDS	Number	2016	65950.6	74017.4	60370
Deaths	Middle SDI	Both	50-69 yea	HIV/AIDS	Rate	2016	15.931	17.8796	14.583
Deaths	High SDI	Both	50-69 yea	Sexually tra	Number	2014	94.622	99.1319	89.9487
Deaths	High SDI	Both	50-69 yea	Sexually tra	Rate	2014	0.03988	0.04178	0.03791
Deaths	High SDI	Both	50-69 yea	HIV/AIDS	Number	2014	5253.77	5406.75	5170.5
Deaths	High SDI	Both	50-69 yea	HIV/AIDS	Rate	2014	2.21429	2.27877	2.17919
Deaths	Low SDI	Both	50-69 yea	HIV/AIDS	Number	2014	57644.7	66908.3	51516.9
Deaths	Low SDI	Both	50-69 yea	HIV/AIDS	Rate	2014	73.7122	85.5579	65.8763
Deaths	Low-middl	Both	50-69 yea	Sexually tra	Number	2014	940.73	1130.36	708.138
Deaths	Low-middl	Both	50-69 yea	Sexually tra	Rate	2014	0.46209	0.55524	0.34784
Deaths	Middle SDI	Both	50-69 yea	Sexually tra	Number	2013	481.874	537.819	376.74
Deaths	Middle SDI	Both	50-69 yea	Sexually tra	Rate	2013	0.13107	0.14628	0.10247
Deaths	High-midc	Both	50-69 yea	Sexually tra	Number	2015	245.391	267.714	227.095
Deaths	High-midc	Both	50-69 yea	Sexually tra	Rate	2015	0.07952	0.08675	0.07359
Deaths	Low SDI	Both	50-69 yea	Sexually tra	Number	2014	625.131	845.351	368.351
Deaths	Low SDI	Both	50-69 yea	Sexually tra	Rate	2014	0.79938	1.08098	0.47102
Deaths	Low SDI	Both	50-69 yea	Sexually tra	Number	2017	633.291	850.669	370.156
Deaths	Low SDI	Both	50-69 yea	Sexually tra	Rate	2017	0.73177	0.98296	0.42772
Deaths	Low-middl	Both	50-69 yea	Sexually tra	Number	2017	1053.62	1316.8	770.623
Deaths	Low-middl	Both	50-69 yea	Sexually tra	Rate	2017	0.46958	0.58687	0.34345
Deaths	High-midc	Both	50-69 yea	Sexually tra	Number	2016	248.414	274.715	229.097
Deaths	High-midc	Both	50-69 yea	Sexually tra	Rate	2016	0.07839	0.08669	0.07229
Deaths	Low-middl	Both	50-69 yea	HIV/AIDS	Number	2015	54396.8	62988.1	48477.5
Deaths	Low-middl	Both	50-69 yea	HIV/AIDS	Rate	2015	25.8412	29.9225	23.0293
Deaths	High-midc	Both	50-69 yea	HIV/AIDS	Number	2015	10119.9	10894.9	9581.44
Deaths	High-midc	Both	50-69 yea	HIV/AIDS	Rate	2015	3.27922	3.53035	3.10475
Deaths	High SDI	Both	50-69 yea	Sexually tra	Number	2015	95.3961	100.063	90.6612
Deaths	High SDI	Both	50-69 yea	Sexually tra	Rate	2015	0.03941	0.04134	0.03745
Deaths	Low-middl	Both	50-69 yea	HIV/AIDS	Number	2017	53883.3	63347.1	46940.8
Deaths	Low-middl	Both	50-69 yea	HIV/AIDS	Rate	2017	24.0148	28.2326	20.9206
Deaths	High-midc	Both	50-69 yea	HIV/AIDS	Number	2018	9403.48	10382.7	8663.85
Deaths	High-midc	Both	50-69 yea	HIV/AIDS	Rate	2018	2.83783	3.13335	2.61462
Deaths	Low-middl	Both	50-69 yea	Sexually tra	Number	2015	1028.87	1256.45	756.94
Deaths	Low-middl	Both	50-69 yea	Sexually tra	Rate	2015	0.48877	0.59688	0.35958
Deaths	Low-middl	Both	50-69 yea	HIV/AIDS	Number	2016	54164.7	63164.4	47851.7
Deaths	Low-middl	Both	50-69 yea	HIV/AIDS	Rate	2016	24.9106	29.0497	22.0073

Deaths	High-midc	Both	50-69 yea	Sexually tr	Rate	2019	0.07141	0.07898	0.06363
Deaths	Low SDI	Both	50-69 yea	HIV/AIDS	Number	2019	48684.3	58242.8	42070.6
Deaths	Low SDI	Both	50-69 yea	HIV/AIDS	Rate	2019	52.4008	62.689	45.2822
Deaths	High-midc	Both	50-69 yea	HIV/AIDS	Number	2019	9242.24	10335.9	8482.81
Deaths	High-midc	Both	50-69 yea	HIV/AIDS	Rate	2019	2.73687	3.06073	2.51198
Deaths	High SDI	Both	50-69 yea	HIV/AIDS	Number	2017	5018.08	5245.13	4907.73
Deaths	High SDI	Both	50-69 yea	HIV/AIDS	Rate	2017	2.02197	2.11347	1.97751
Deaths	Low SDI	Both	50-69 yea	HIV/AIDS	Number	2016	53678.7	63133.5	47601.2
Deaths	Low SDI	Both	50-69 yea	HIV/AIDS	Rate	2016	64.1925	75.4992	56.9247
Deaths	Low-middl	Both	50-69 yea	HIV/AIDS	Number	2019	54500.2	65024.8	46580.5
Deaths	Low-middl	Both	50-69 yea	HIV/AIDS	Rate	2019	22.8233	27.2307	19.5067
Deaths	High-midc	Both	50-69 yea	Sexually tr	Number	2017	242.109	262.739	222.745
Deaths	High-midc	Both	50-69 yea	Sexually tr	Rate	2017	0.07471	0.08108	0.06874
Deaths	Low-middl	Both	50-69 yea	Sexually tr	Number	2019	1048.67	1350.88	758.367
Deaths	Low-middl	Both	50-69 yea	Sexually tr	Rate	2019	0.43916	0.56571	0.31758
Deaths	Middle SDI	Both	50-69 yea	HIV/AIDS	Number	2019	65055.6	74087.4	58589.7
Deaths	Middle SDI	Both	50-69 yea	HIV/AIDS	Rate	2019	14.2361	16.2125	12.8212
Deaths	Middle SDI	Both	50-69 yea	Sexually tr	Number	2019	532.666	628.235	393.156
Deaths	Middle SDI	Both	50-69 yea	Sexually tr	Rate	2019	0.11656	0.13748	0.08603
DALYs (Dis	High-midc	Both	50-69 yea	HIV/AIDS	Number	1990	69347.2	72464.1	66392.1
DALYs (Dis	High-midc	Both	50-69 yea	HIV/AIDS	Rate	1990	37.2543	38.9287	35.6668
DALYs (Dis	Low-middl	Both	50-69 yea	HIV/AIDS	Number	1991	304318	460183	185742
DALYs (Dis	Low-middl	Both	50-69 yea	HIV/AIDS	Rate	1991	269.129	406.97	164.264
DALYs (Dis	High-midc	Both	50-69 yea	Sexually tr	Number	1993	35663.1	56127.1	24557.3
DALYs (Dis	High-midc	Both	50-69 yea	Sexually tr	Rate	1993	18.64	29.3359	12.8353
DALYs (Dis	High SDI	Both	50-69 yea	HIV/AIDS	Number	1993	260036	274713	250298
DALYs (Dis	High SDI	Both	50-69 yea	HIV/AIDS	Rate	1993	164.144	173.409	157.997
DALYs (Dis	Low SDI	Both	50-69 yea	HIV/AIDS	Number	1991	1034241	1394685	711167
DALYs (Dis	Low SDI	Both	50-69 yea	HIV/AIDS	Rate	1991	2312.88	3118.94	1590.39
DALYs (Dis	High-midc	Both	50-69 yea	Sexually tr	Number	1990	31527.3	51575.1	20955.6
DALYs (Dis	High-midc	Both	50-69 yea	Sexually tr	Rate	1990	16.9369	27.7069	11.2576
DALYs (Dis	Low-middl	Both	50-69 yea	Sexually tr	Number	1991	40154.2	53816.6	29296.7
DALYs (Dis	Low-middl	Both	50-69 yea	Sexually tr	Rate	1991	35.511	47.5936	25.909
DALYs (Dis	High SDI	Both	50-69 yea	Sexually tr	Number	1993	19473.1	36064.6	11119.7
DALYs (Dis	High SDI	Both	50-69 yea	Sexually tr	Rate	1993	12.2921	22.7653	7.01914
DALYs (Dis	Low-middl	Both	50-69 yea	HIV/AIDS	Number	1993	599724	865018	384229
DALYs (Dis	Low-middl	Both	50-69 yea	HIV/AIDS	Rate	1993	508.29	733.136	325.649
DALYs (Dis	High-midc	Both	50-69 yea	Sexually tr	Number	1992	34220.5	54318	23195.5
DALYs (Dis	High-midc	Both	50-69 yea	Sexually tr	Rate	1992	17.9499	28.4919	12.1669
DALYs (Dis	Middle SDI	Both	50-69 yea	Sexually tr	Number	1991	40387.5	65956.7	26940.1
DALYs (Dis	Middle SDI	Both	50-69 yea	Sexually tr	Rate	1991	21.0201	34.3277	14.0212
DALYs (Dis	High-midc	Both	50-69 yea	HIV/AIDS	Number	1992	96955.7	102763	92821.8
DALYs (Dis	High-midc	Both	50-69 yea	HIV/AIDS	Rate	1992	50.857	53.9034	48.6886
DALYs (Dis	High SDI	Both	50-69 yea	Sexually tr	Number	1990	19725.2	35797.8	11483.8
DALYs (Dis	High SDI	Both	50-69 yea	Sexually tr	Rate	1990	12.8581	23.3353	7.48587
DALYs (Dis	Low SDI	Both	50-69 yea	Sexually tr	Number	1991	29723.4	45509.4	17463.3
DALYs (Dis	Low SDI	Both	50-69 yea	Sexually tr	Rate	1991	66.4705	101.773	39.0532
DALYs (Dis	Low SDI	Both	50-69 yea	HIV/AIDS	Number	1993	1522609	1994087	1111017
DALYs (Dis	Low SDI	Both	50-69 yea	HIV/AIDS	Rate	1993	3292.35	4311.83	2402.36
DALYs (Dis	Low-middl	Both	50-69 yea	Sexually tr	Number	1993	41091.2	55171.9	30090.4
DALYs (Dis	Low-middl	Both	50-69 yea	Sexually tr	Rate	1993	34.8264	46.7603	25.5028
DALYs (Dis	High SDI	Both	50-69 yea	HIV/AIDS	Number	1990	175832	183598	170208
DALYs (Dis	High SDI	Both	50-69 yea	HIV/AIDS	Rate	1990	114.619	119.681	110.952
DALYs (Dis	Middle SDI	Both	50-69 yea	HIV/AIDS	Number	1994	325640	419994	239887
DALYs (Dis	Middle SDI	Both	50-69 yea	HIV/AIDS	Rate	1994	159.296	205.452	117.348
DALYs (Dis	Low SDI	Both	50-69 yea	Sexually tr	Number	1993	29946	45784.4	17652.8
DALYs (Dis	Low SDI	Both	50-69 yea	Sexually tr	Rate	1993	64.7524	99.0001	38.1708
DALYs (Dis	Low-middl	Both	50-69 yea	HIV/AIDS	Number	1990	204942	317029	123805
DALYs (Dis	Low-middl	Both	50-69 yea	HIV/AIDS	Rate	1990	185.381	286.77	111.989
DALYs (Dis	Low SDI	Both	50-69 yea	HIV/AIDS	Number	1990	822747	1129090	549838
DALYs (Dis	Low SDI	Both	50-69 yea	HIV/AIDS	Rate	1990	1872.83	2570.16	1251.6
DALYs (Dis	High SDI	Both	50-69 yea	HIV/AIDS	Number	1992	234366	246675	226036
DALYs (Dis	High SDI	Both	50-69 yea	HIV/AIDS	Rate	1992	149.697	157.559	144.376
DALYs (Dis	Low-middl	Both	50-69 yea	Sexually tr	Number	1990	39771.5	53230.8	29352.2
DALYs (Dis	Low-middl	Both	50-69 yea	Sexually tr	Rate	1990	35.9755	48.1502	26.5507
DALYs (Dis	Middle SDI	Both	50-69 yea	Sexually tr	Number	1990	39742.6	63809	26587
DALYs (Dis	Middle SDI	Both	50-69 yea	Sexually tr	Rate	1990	21.1655	33.9825	14.1593
DALYs (Dis	Middle SDI	Both	50-69 yea	HIV/AIDS	Number	1991	107634	142236	80545.7
DALYs (Dis	Middle SDI	Both	50-69 yea	HIV/AIDS	Rate	1991	56.0192	74.028	41.9207
DALYs (Dis	Middle SDI	Both	50-69 yea	Sexually tr	Number	1993	41061.2	68301.3	27044.9
DALYs (Dis	Middle SDI	Both	50-69 yea	Sexually tr	Rate	1993	20.4864	34.0771	13.4934
DALYs (Dis	High-midc	Both	50-69 yea	Sexually tr	Number	1995	36637.1	57686.9	25386.3
DALYs (Dis	High-midc	Both	50-69 yea	Sexually tr	Rate	1995	19.1039	30.0799	13.2373
DALYs (Dis	High SDI	Both	50-69 yea	Sexually tr	Number	1992	19487.1	35986.2	11152.7
DALYs (Dis	High SDI	Both	50-69 yea	Sexually tr	Rate	1992	12.447	22.9856	7.12361
DALYs (Dis	Middle SDI	Both	50-69 yea	Sexually tr	Number	1992	40941.3	66923.5	27352
DALYs (Dis	Middle SDI	Both	50-69 yea	Sexually tr	Rate	1992	20.8522	34.0854	13.9309
DALYs (Dis	Low SDI	Both	50-69 yea	HIV/AIDS	Number	1996	2288223	2797828	1797125
DALYs (Dis	Low SDI	Both	50-69 yea	HIV/AIDS	Rate	1996	4688.95	5733.22	3682.61
DALYs (Dis	Middle SDI	Both	50-69 yea	HIV/AIDS	Number	1993	231845	303975	169560
DALYs (Dis	Middle SDI	Both	50-69 yea	HIV/AIDS	Rate	1993	115.673	151.66	84.5975
DALYs (Dis	Low-middl	Both	50-69 yea	HIV/AIDS	Number	1992	436268	647627	274097
DALYs (Dis	Low-middl	Both	50-69 yea	HIV/AIDS	Rate	1992	377.604	560.543	237.239
DALYs (Dis	High SDI	Both	50-69 yea	Sexually tr	Number	1994	19456.5	36387.8	10953
DALYs (Dis	High SDI	Both	50-69 yea	Sexually tr	Rate	1994	12.1383	22.7013	6.83328
DALYs (Dis	High-midc	Both	50-69 yea	HIV/AIDS	Number	1991	82831.5	86828.8	79500.5
DALYs (Dis	High-midc	Both	50-69 yea	HIV/AIDS	Rate	1991	43.8825	46.0002	42.1178
DALYs (Dis	Low SDI	Both	50-69 yea	Sexually tr	Number	1990	29571.8	45588.6	17355.7
DALYs (Dis	Low SDI	Both	50-69 yea	Sexually tr	Rate	1990	67.3145	103.774	39.507
DALYs (Dis	High SDI	Both	50-69 yea	Sexually tr	Number	1991	19637.1	35836.8	11351.8
DALYs (Dis	High SDI	Both	50-69 yea	Sexually tr	Rate	1991	12.6625	23.1085	7.31995
DALYs (Dis	High SDI	Both	50-69 yea	HIV/AIDS	Number	1995	311010	332439	297823
DALYs (Dis	High SDI	Both	50-69 yea	HIV/AIDS	Rate	1995	192.592	205.861	184.426
DALYs (Dis	Low-middl	Both	50-69 yea	Sexually tr	Number	1992	41037.6	54712.6	29742.7
DALYs (Dis	Low-middl	Both	50-69 yea	Sexually tr	Rate	1992	35.5194	47.3555	25.7433
DALYs (Dis	High-midc	Both	50-69 yea	Sexually tr	Number	1996	35758.8	56071.7	24318.8
DALYs (Dis	High-midc	Both	50-69 yea	Sexually tr	Rate	1996	18.511	29.0261	12.5889
DALYs (Dis	Middle SDI	Both	50-69 yea	Sexually tr	Number	1994	41289.1	68843.2	26969.3
DALYs (Dis	Middle SDI	Both	50-69 yea	Sexually tr	Rate	1994	20.1977	33.6766	13.1928
DALYs (Dis	Low-middl	Both	50-69 yea	Sexually tr	Number	1994	41499.1	55295.8	30986.7
DALYs (Dis	Low-middl	Both	50-69 yea	Sexually tr	Rate	1994	34.4575	45.9131	25.7288
DALYs (Dis	High-midc	Both	50-69 yea	HIV/AIDS	Number	1997	156052	173742	143926
DALYs (Dis	High-midc	Both	50-69 yea	HIV/AIDS	Rate	1997	79.694	88.7279	73.5012
DALYs (Dis	Middle SDI	Both	50-69 yea	HIV/AIDS	Number	1999	1207850	1435727	987207
DALYs (Dis	Middle SDI	Both	50-69 yea	HIV/AIDS	Rate	1999	522.908	621.561	427.386
DALYs (Dis	Low SDI	Both	50-69 yea	HIV/AIDS	Number	1992	1270468	1690788	902445
DALYs (Dis	Low SDI	Both	50-69 yea	HIV/AIDS	Rate	1992	2791.7	3715.3	1983.01
DALYs (Dis	Middle SDI	Both	50-69 yea	HIV/AIDS	Number	1990	73401.6	94446.7	55180.4
DALYs (Dis	Middle SDI	Both	50-69 yea	HIV/AIDS	Rate	1990	39.0912	50.2991	29.3872
DALYs (Dis	High-midc	Both	50-69 yea	Sexually tr	Number	1991	32967	52758.7	22103
DALYs (Dis	High-midc	Both	50-69 yea	Sexually tr	Rate	1991	17.4653	27.9505	11.7097
DALYs (Dis	Low SDI	Both	50-69 yea	Sexually tr	Number	1992	29942	45494.2	17598.8
DALYs (Dis	Low SDI	Both	50-69 yea	Sexually tr	Rate	1992	65.7939	99.9679	38.6711
DALYs (Dis	Middle SDI	Both	50-69 yea	Sexually tr	Number	1998	41044	71065.5	25905.9
DALYs (Dis	Middle SDI	Both	50-69 yea	Sexually tr	Rate	1998	18.3247	31.7282	11.566
DALYs (Dis	High SDI	Both	50-69 yea	HIV/AIDS	Number	1991	207823	217688	200790
DALYs (Dis	High SDI	Both	50-69 yea	HIV/AIDS	Rate	1991	134.01	140.371	129.475
DALYs (Dis	High-midc	Both	50-69 yea	Sexually tr	Number	2000	33979.1	55611.9	22341.3
DALYs (Dis	High-midc	Both	50-69 yea	Sexually tr	Rate	2000	16.5621	27.1064	10.8896
DALYs (Dis	High-midc	Both	50-69 yea	HIV/AIDS	Number	1995	150483	163774	141916
DALYs (Dis	High-midc	Both	50-69 yea	HIV/AIDS	Rate	1995	78.4672	85.3974	73.9999
DALYs (Dis	Low-middl	Both	50-69 yea	HIV/AIDS	Number	1995	993769	1352145	671921
DALYs (Dis	Low-middl	Both	50-69 yea	HIV/AIDS	Rate	1995	808.946	1100.67	546.956
DALYs (Dis	High SDI	Both	50-69 yea	HIV/AIDS	Number	1996	251004	270123	239048
DALYs (Dis	High SDI	Both	50-69 yea	HIV/AIDS	Rate	1996	154.238	165.986	146.891
DALYs (Dis	Low SDI	Both	50-69 yea	Sexually tr	Number	1996	30606.4	47552.6	17964.4
DALYs (Dis	Low SDI	Both	50-69 yea	Sexually tr	Rate	1996	62.7177	97.4431	36.8121
DALYs (Dis	Middle SDI	Both	50-69 yea	HIV/AIDS	Number	1992	158825	211403	115984
DALYs (Dis	Middle SDI	Both	50-69 yea	HIV/AIDS	Rate	1992	80.8927	107.671	59.0729
DALYs (Dis	Low SDI	Both	50-69 yea	Sexually tr	Number	1994	30148	46816	17742.7
DALYs (Dis	Low SDI	Both	50-69 yea	Sexually tr	Rate	1994	64.0489	99.4598	37.6939
DALYs (Dis	High SDI	Both	50-69 yea	Sexually tr	Number	1999	20115.1	38570.2	10789
DALYs (Dis	High SDI	Both	50-69 yea	Sexually tr	Rate	1999	11.5401	22.1279	6.18968
DALYs (Dis	High-midc	Both	50-69 yea	HIV/AIDS	Number	1993	117072	125697	111450
DALYs (Dis	High-midc	Both	50-69 yea	HIV/AIDS	Rate	1993	61.1898	65.6978	58.2516
DALYs (Dis	High SDI	Both	50-69 yea	Sexually tr	Number	1995	19344.7	36443.4	10764.4
DALYs (Dis	High SDI	Both	50-69 yea	Sexually tr	Rate	1995	11.9792	22.5674	6.6658
DALYs (Dis	High SDI	Both	50-69 yea	Sexually tr	Number	2003	21595.3	42045.4	11427.7
DALYs (Dis	High SDI	Both	50-69 yea	Sexually tr	Rate	2003	11.3805	22.1576	6.02231
DALYs (Dis	High-midc	Both	50-69 yea	HIV/AIDS	Number	2001	227995	250256	208821
DALYs (Dis	High-midc	Both	50-69 yea	HIV/AIDS	Rate	2001	108.557	119.157	99.4276
DALYs (Dis	High SDI	Both	50-69 yea	HIV/AIDS	Number	2000	179040	195012	167886
DALYs (Dis	High SDI	Both	50-69 yea	HIV/AIDS	Rate	2000	100.467	109.43	94.2084
DALYs (Dis	High-midc	Both	50-69 yea	HIV/AIDS	Number	1994	135847	147412	128639
DALYs (Dis	High-midc	Both	50-69 yea	HIV/AIDS	Rate	1994	70.9447	76.9845	67.1805
DALYs (Dis	High-midc	Both	50-69 yea	Sexually tr	Number	1994	36663.1	57469.5	25342.6
DALYs (Dis	High-midc	Both	50-69 yea	Sexually tr	Rate	1994	19.1469	30.0128	13.2349
DALYs (Dis	Middle SDI	Both	50-69 yea	Sexually tr	Number	1996	40493.3	69234.4	25856.5
DALYs (Dis	Middle SDI	Both	50-69 yea	Sexually tr	Rate	1996	18.983	32.4566	12.1214
DALYs (Dis	Middle SDI	Both	50-69 yea	Sexually tr	Number	2002	44562.6	76976.4	27469
DALYs (Dis	Middle SDI	Both	50-69 yea	Sexually tr	Rate	2002	17.5965	30.3959	10.8468
DALYs (Dis	Low-middl	Both	50-69 yea	HIV/AIDS	Number	1996	1211670	1584795	856499
DALYs (Dis	Low-middl	Both	50-69 yea	HIV/AIDS	Rate	1996	966.725	1264.42	683.354
DALYs (Dis	Middle SDI	Both	50-69 yea	HIV/AIDS	Number	1996	588852	735355	453806
DALYs (Dis	Middle SDI	Both	50-69 yea	HIV/AIDS	Rate	1996	276.05	344.73	212.742
DALYs (Dis	Low SDI	Both	50-69 yea	HIV/AIDS	Number	1995	2044392	2541482	1574773
DALYs (Dis	Low SDI	Both	50-69 yea	HIV/AIDS	Rate	1995	4265.82	5303.04	3285.91
DALYs (Dis	Middle SDI	Both	50-69 yea	Sexually tr	Number	1995	40699.2	69399.4	26310.7
DALYs (Dis	Middle SDI	Both	50-69 yea	Sexually tr	Rate	1995	19.5163	33.2788	12.6167
DALYs (Dis	High SDI	Both	50-69 yea	HIV/AIDS	Number	1994	296846	314642	285531
DALYs (Dis	High SDI	Both	50-69 yea	HIV/AIDS	Rate	1994	185.193	196.296	178.135
DALYs (Dis	Low-middl	Both	50-69 yea	Sexually tr	Number	1995	41593.6	56625.2	30929.7
DALYs (Dis	Low-middl	Both	50-69 yea	Sexually tr	Rate	1995	33.8579	46.0939	25.1773
DALYs (Dis	Low-middl	Both	50-69 yea	Sexually tr	Number	1999	42679.6	59277.9	31793.9
DALYs (Dis	Low-middl	Both	50-69 yea	Sexually tr	Rate	1999	32.0691	44.5409	23.8897
DALYs (Dis	High-midc	Both	50-69 yea	Sexually tr	Number	2004	33068.7	58474.9	20172.6
DALYs (Dis	High-midc	Both	50-69 yea	Sexually tr	Rate	2004	14.5309	25.6948	8.86415
DALYs (Dis	Low-middl	Both	50-69 yea	Sexually tr	Number	2003	42588.5	60539.5	31315.1
DALYs (Dis	Low-middl	Both	50-69 yea	Sexually tr	Rate	2003	29.0551	41.3017	21.3641
DALYs (Dis	Low-middl	Both	50-69 yea	HIV/AIDS	Number	2000	2020095	2356066	1654133
DALYs (Dis	Low-middl	Both	50-69 yea	HIV/AIDS	Rate	2000	1493.23	1741.58	1222.72
DALYs (Dis	High-midc	Both	50-69 yea	HIV/AIDS	Number	2005	294909	323474	267000
DALYs (Dis	High-midc	Both	50-69 yea	HIV/AIDS	Rate	2005	125.891	138.084	113.977
DALYs (Dis	Low-middl	Both	50-69 yea	HIV/AIDS	Number	1994	788155	1098121	517453
DALYs (Dis	Low-middl	Both	50-69 yea	HIV/AIDS	Rate	1994	654.42	911.791	429.651
DALYs (Dis	High SDI	Both	50-69 yea	HIV/AIDS	Number	2004	215596	237885	199538
DALYs (Dis	High SDI	Both	50-69 yea	HIV/AIDS	Rate	2004	111.224	122.723	102.94
DALYs (Dis	High SDI	Both	50-69 yea	HIV/AIDS	Number	1998	158389	176595	146408
DALYs (Dis	High SDI	Both	50-69 yea	HIV/AIDS	Rate	1998	92.9696	103.656	85.9368
DALYs (Dis	High-midc	Both	50-69 yea	HIV/AIDS	Number	1996	159602	175202	149046
DALYs (Dis	High-midc	Both	50-69 yea	HIV/AIDS	Rate	1996	82.6199	90.695	77.1555
DALYs (Dis	Low SDI	Both	50-69 yea	Sexually tr	Number	1995	30397.9	47929.4	17800.8
DALYs (Dis	Low SDI	Both	50-69 yea	Sexually tr	Rate	1995	63.4281	100.009	37.143
DALYs (Dis	Low SDI	Both	50-69 yea	Sexually tr	Number	1999	30194.3	46336.9	17785.6
DALYs (Dis	Low SDI	Both	50-69 yea	Sexually tr	Rate	1999	58.7475	90.1554	34.6046
DALYs (Dis	High SDI	Both	50-69 yea	Sexually tr	Number	1996	19222.5	36291	10592.1
DALYs (Dis	High SDI	Both	50-69 yea	Sexually tr	Rate	1996	11.8119	22.3003	6.50869
DALYs (Dis	Low SDI	Both	50-69 yea	HIV/AIDS	Number	1994	1785051	2276408	1339362
DALYs (Dis	Low SDI	Both	50-69 yea	HIV/AIDS	Rate	1994	3792.3	4836.18	2845.45
DALYs (Dis	Low SDI	Both	50-69 yea	HIV/AIDS	Number	2000	2951874	3374879	2553931
DALYs (Dis	Low SDI	Both	50-69 yea	HIV/AIDS	Rate	2000	5660.13	6471.22	4897.08
DALYs (Dis	High SDI	Both	50-69 yea	Sexually tr	Number	1998	19756.4	37715.1	10662.6
DALYs (Dis	High SDI	Both	50-69 yea	Sexually tr	Rate	1998	11.5964	22.1376	6.25862
DALYs (Dis	Low SDI	Both	50-69 yea	Sexually tr	Number	2003	29606.1	45009.7	18201.6
DALYs (Dis	Low SDI	Both	50-69 yea	Sexually tr	Rate	2003	52.444	79.7299	32.2422
DALYs (Dis	High-midc	Both	50-69 yea	Sexually tr	Number	1997	34943.1	55259.9	23652.7
DALYs (Dis	High-midc	Both	50-69 yea	Sexually tr	Rate	1997	17.8451	28.2206	12.0792
DALYs (Dis	Middle SDI	Both	50-69 yea	Sexually tr	Number	2006	49420	86919.9	29769.6
DALYs (Dis	Middle SDI	Both	50-69 yea	Sexually tr	Rate	2006	16.8806	29.6897	10.1686
DALYs (Dis	Middle SDI	Both	50-69 yea	Sexually tr	Number	1999	41631	72613.8	26174
DALYs (Dis	Middle SDI	Both	50-69 yea	Sexually tr	Rate	1999	18.0231	31.4363	11.3313
DALYs (Dis	Low-middl	Both	50-69 yea	HIV/AIDS	Number	2004	2582262	2805313	2379854
DALYs (Dis	Low-middl	Both	50-69 yea	HIV/AIDS	Rate	2004	1713.22	1861.2	1578.93
DALYs (Dis	Low-middl	Both	50-69 yea	HIV/AIDS	Number	1998	1645987	2027013	1248801
DALYs (Dis	Low-middl	Both	50-69 yea	HIV/AIDS	Rate	1998	1261.66	1553.72	957.213
DALYs (Dis	Middle SDI	Both	50-69 yea	HIV/AIDS	Number	2003	2253922	2521438	1992633
DALYs (Dis	Middle SDI	Both	50-69 yea	HIV/AIDS	Rate	2003	858.922	960.866	759.35
DALYs (Dis	High SDI	Both	50-69 yea	Sexually tr	Number	1997	19478.3	36968.4	10648.2
DALYs (Dis	High SDI	Both	50-69 yea	Sexually tr	Rate	1997	11.7029	22.2113	6.39763
DALYs (Dis	Low-middl	Both	50-69 yea	Sexually tr	Number	1996	42508.5	57983.7	31778.7
DALYs (Dis	Low-middl	Both	50-69 yea	Sexually tr	Rate	1996	33.9152	46.2621	25.3545
DALYs (Dis	High SDI	Both	50-69 yea	HIV/AIDS	Number	1997	167613	183805	156762
DALYs (Dis	High SDI	Both	50-69 yea	HIV/AIDS	Rate	1997	100.705	110.433	94.1855
DALYs (Dis	Low SDI	Both	50-69 yea	HIV/AIDS	Number	2004	3292135	3592661	3006451
DALYs (Dis	Low SDI	Both	50-69 yea	HIV/AIDS	Rate	2004	5685.14	6204.12	5191.8
DALYs (Dis	Low-middl	Both	50-69 yea	Sexually tr	Number	1998	43388.3	60051.5	32268.7
DALYs (Dis	Low-middl	Both	50-69 yea	Sexually tr	Rate	1998	33.2573	46.0298	24.7341
DALYs (Dis	High SDI	Both	50-69 yea	Sexually tr	Number	2007	23097.7	45259.2	12037
DALYs (Dis	High SDI	Both	50-69 yea	Sexually tr	Rate	2007	11.1867	21.9199	5.82978
DALYs (Dis	Middle SDI	Both	50-69 yea	HIV/AIDS	Number	1995	443786	562328	334903
DALYs (Dis	Middle SDI	Both	50-69 yea	HIV/AIDS	Rate	1995	212.807	269.651	160.595
DALYs (Dis	High-midc	Both	50-69 yea	HIV/AIDS	Number	2000	205854	227937	187403
DALYs (Dis	High-midc	Both	50-69 yea	HIV/AIDS	Rate	2000	100.338	111.101	91.3439
DALYs (Dis	Low-middl	Both	50-69 yea	HIV/AIDS	Number	1997	1429524	1807396	1046846
DALYs (Dis	Low-middl	Both	50-69 yea	HIV/AIDS	Rate	1997	1117.05	1412.32	818.017
DALYs (Dis	High-midc	Both	50-69 yea	HIV/AIDS	Number	1999	182677	201561	166753
DALYs (Dis	High-midc	Both	50-69 yea	HIV/AIDS	Rate	1999	90.4678	99.8194	82.5815
DALYs (Dis	Low-middl	Both	50-69 yea	Sexually tr	Number	2007	44476.6	64406.5	32136.7
DALYs (Dis	Low-middl	Both	50-69 yea	Sexually tr	Rate	2007	27.1126	39.2617	19.5903
DALYs (Dis	Low SDI	Both	50-69 yea	HIV/AIDS	Number	1998	2696472	3175910	2225235
DALYs (Dis	Low SDI	Both	50-69 yea	HIV/AIDS	Rate	1998	5333.98	6282.37	4401.81
DALYs (Dis	Low SDI	Both	50-69 yea	Sexually tr	Number	1998	30538.4	47490.5	17976
DALYs (Dis	Low SDI	Both	50-69 yea	Sexually tr	Rate	1998	60.409	93.9425	35.559
DALYs (Dis	Low-middl	Both	50-69 yea	Sexually tr	Number	1997	43555.6	59762.3	32570.7
DALYs (Dis	Low-middl	Both	50-69 yea	Sexually tr	Rate	1997	34.0348	46.6989	25.4511
DALYs (Dis	High SDI	Both	50-69 yea	Sexually tr	Number	2000	20448.8	39372.1	10884.7
DALYs (Dis	High SDI	Both	50-69 yea	Sexually tr	Rate	2000	11.4747	22.0934	6.10791
DALYs (Dis	Low-middl	Both	50-69 yea	HIV/AIDS	Number	2008	2248623	2572254	1971733
DALYs (Dis	Low-middl	Both	50-69 yea	HIV/AIDS	Rate	2008	1332.61	1524.41	1168.52
DALYs (Dis	High-midc	Both	50-69 yea	Sexually tr	Number	2008	34031.6	61932.8	19842
DALYs (Dis	High-midc	Both	50-69 yea	Sexually tr	Rate	2008	13.4658	24.5059	7.85115
DALYs (Dis	High-midc	Both	50-69 yea	HIV/AIDS	Number	1998	157255	175455	142684
DALYs (Dis	High-midc	Both	50-69 yea	HIV/AIDS	Rate	1998	79.2578	88.4309	71.9138
DALYs (Dis	Low SDI	Both	50-69 yea	HIV/AIDS	Number	1997	2509862	3019936	2016025
DALYs (Dis	Low SDI	Both	50-69 yea	HIV/AIDS	Rate	1997	5050.84	6077.31	4057.04
DALYs (Dis	High-midc	Both	50-69 yea	Sexually tr	Number	1999	34218.4	55101.9	22786.3
DALYs (Dis	High-midc	Both	50-69 yea	Sexually tr	Rate	1999	16.946	27.2882	11.2845
DALYs (Dis	Low SDI	Both	50-69 yea	Sexually tr	Number	2007	29074.7	41763	18528.8
DALYs (Dis	Low SDI	Both	50-69 yea	Sexually tr	Rate	2007	46.2878	66.4881	29.4985
DALYs (Dis	Middle SDI	Both	50-69 yea	Sexually tr	Number	1997	40735.7	70259.6	25746
DALYs (Dis	Middle SDI	Both	50-69 yea	Sexually tr	Rate	1997	18.6522	32.1708	11.7887
DALYs (Dis	High-midc	Both	50-69 yea	Sexually tr	Number	1998	34039.7	54322.7	22768.1
DALYs (Dis	High-midc	Both	50-69 yea	Sexually tr	Rate	1998	17.1563	27.3791	11.4753
DALYs (Dis	Low SDI	Both	50-69 yea	HIV/AIDS	Number	2008	2631354	3088765	2282137
DALYs (Dis	Low SDI	Both	50-69 yea	HIV/AIDS	Rate	2008	4068.29	4775.48	3528.37
DALYs (Dis	Low-middl	Both	50-69 yea	Sexually tr	Number	2000	42258.7	59462.5	31465.5
DALYs (Dis	Low-middl	Both	50-69 yea	Sexually tr	Rate	2000	31.2371	43.954	23.2589
DALYs (Dis	High SDI	Both	50-69 yea	HIV/AIDS	Number	2008	237713	269000	213686
DALYs (Dis	High SDI	Both	50-69 yea	HIV/AIDS	Rate	2008	112.747	127.586	101.351
DALYs (Dis	Low SDI	Both	50-69 yea	Sexually tr	Number	1997	30682.6	47812	17979.3
DALYs (Dis	Low SDI	Both	50-69 yea	Sexually tr	Rate	1997	61.7455	96.2166	36.1814
DALYs (Dis	High SDI	Both	50-69 yea	HIV/AIDS	Number	1999	178404	196742	166761
DALYs (Dis	High SDI	Both	50-69 yea	HIV/AIDS	Rate	1999	102.351	112.872	95.6717
DALYs (Dis	Middle SDI	Both	50-69 yea	HIV/AIDS	Number	2007	2598697	2854104	2335435
DALYs (Dis	Middle SDI	Both	50-69 yea	HIV/AIDS	Rate	2007	858.916	943.332	771.903
DALYs (Dis	Middle SDI	Both	50-69 yea	HIV/AIDS	Number	1998	972367	1175218	783182
DALYs (Dis	Middle SDI	Both	50-69 yea	HIV/AIDS	Rate	1998	434.127	524.692	349.662
DALYs (Dis	Low SDI	Both	50-69 yea	Sexually tr	Number	2001	29704.3	46099.7	17969
DALYs (Dis	Low SDI	Both	50-69 yea	Sexually tr	Rate	2001	55.4295	86.0239	33.5309
DALYs (Dis	High-midc	Both	50-69 yea	HIV/AIDS	Number	2009	309772	348744	279630
DALYs (Dis	High-midc	Both	50-69 yea	HIV/AIDS	Rate	2009	119.726	134.789	108.077
DALYs (Dis	Low SDI	Both	50-69 yea	Sexually tr	Number	2000	29735.1	46042	17718.2
DALYs (Dis	Low SDI	Both	50-69 yea	Sexually tr	Rate	2000	57.0161	88.284	33.9741
DALYs (Dis	Low-middl	Both	50-69 yea	HIV/AIDS	Number	1999	1841690	2198922	1454891
DALYs (Dis	Low-middl	Both	50-69 yea	HIV/AIDS	Rate	1999	1383.83	1652.25	1093.19
DALYs (Dis	Middle SDI	Both	50-69 yea	HIV/AIDS	Number	1997	765258	941779	603593
DALYs (Dis	Middle SDI	Both	50-69 yea	HIV/AIDS	Rate	1997	350.4	431.226	276.376
DALYs (Dis	Middle SDI	Both	50-69 yea	Sexually tr	Number	2001	43361	75482.9	26826.1
DALYs (Dis	Middle SDI	Both	50-69 yea	Sexually tr	Rate	2001	17.7047	30.8204	10.9534
DALYs (Dis	Middle SDI	Both	50-69 yea	Sexually tr	Number	2000	42301.8	73196.2	26494.4
DALYs (Dis	Middle SDI	Both	50-69 yea	Sexually tr	Rate	2000	17.8487	30.8842	11.179
DALYs (Dis	Middle SDI	Both	50-69 yea	Sexually tr	Number	2010	53453.1	96456.9	31504.4
DALYs (Dis	Middle SDI	Both	50-69 yea	Sexually tr	Rate	2010	16.1632	29.1667	9.52633
DALYs (Dis	Middle SDI	Both	50-69 yea	HIV/AIDS	Number	2001	1750200	2017424	1482118
DALYs (Dis	Middle SDI	Both	50-69 yea	HIV/AIDS	Rate	2001	714.624	823.734	605.163
DALYs (Dis	High-midc	Both	50-69 yea	HIV/AIDS	Number	2002	245943	271273	224565
DALYs (Dis	High-midc	Both	50-69 yea	HIV/AIDS	Rate	2002	114.188	125.948	104.262
DALYs (Dis	High SDI	Both	50-69 yea	HIV/AIDS	Number	2003	206970	227214	192627
DALYs (Dis	High SDI	Both	50-69 yea	HIV/AIDS	Rate	2003	109.072	119.74	101.513
DALYs (Dis	High SDI	Both	50-69 yea	Sexually tr	Number	2002	21166.6	41125	11160.4
DALYs (Dis	High SDI	Both	50-69 yea	Sexually tr	Rate	2002	11.3924	22.1345	6.00681
DALYs (Dis	Low SDI	Both	50-69 yea	HIV/AIDS	Number	1999	2841470	3285119	2406222
DALYs (Dis	Low SDI	Both	50-69 yea	HIV/AIDS	Rate	1999	5528.5	6391.68	4681.66
DALYs (Dis	Middle SDI	Both	50-69 yea	HIV/AIDS	Number	2000	1468125	1713225	1217117
DALYs (Dis	Middle SDI	Both	50-69 yea	HIV/AIDS	Rate	2000	619.457	722.874	513.547
DALYs (Dis	High-midc	Both	50-69 yea	Sexually tr	Number	2002	33407.4	56618.2	21404.1
DALYs (Dis	High-midc	Both	50-69 yea	Sexually tr	Rate	2002	15.5106	26.287	9.93762
DALYs (Dis	Middle SDI	Both	50-69 yea	HIV/AIDS	Number	2011	2237800	2658192	1943662
DALYs (Dis	Middle SDI	Both	50-69 yea	HIV/AIDS	Rate	2011	655.369	778.486	569.227
DALYs (Dis	Low-middl	Both	50-69 yea	HIV/AIDS	Number	2003	2508643	2760691	2263779
DALYs (Dis	Low-middl	Both	50-69 yea	HIV/AIDS	Rate	2003	1711.47	1883.42	1544.41
DALYs (Dis	Low-middl	Both	50-69 yea	Sexually tr	Number	2002	42814.4	60978.7	31649.9
DALYs (Dis	Low-middl	Both	50-69 yea	Sexually tr	Rate	2002	30.0151	42.7492	22.1882
DALYs (Dis	High SDI	Both	50-69 yea	Sexually tr	Number	2011	24315.4	47751.8	12592.4
DALYs (Dis	High SDI	Both	50-69 yea	Sexually tr	Rate	2011	10.85	21.3077	5.61896
DALYs (Dis	Middle SDI	Both	50-69 yea	Sexually tr	Number	2003	45716.1	79592.8	28194.2
DALYs (Dis	Middle SDI	Both	50-69 yea	Sexually tr	Rate	2003	17.4215	30.3311	10.7442
DALYs (Dis	Low-middl	Both	50-69 yea	Sexually tr	Number	2011	46682.9	69174.9	33819.3
DALYs (Dis	Low-middl	Both	50-69 yea	Sexually tr	Rate	2011	25.3035	37.4948	18.3311
DALYs (Dis	High-midc	Both	50-69 yea	Sexually tr	Number	2001	33591.7	55620.2	21788.2
DALYs (Dis	High-midc	Both	50-69 yea	Sexually tr	Rate	2001	15.9943	26.4829	10.3742
DALYs (Dis	High SDI	Both	50-69 yea	HIV/AIDS	Number	2002	195833	214110	183088
DALYs (Dis	High SDI	Both	50-69 yea	HIV/AIDS	Rate	2002	105.402	115.239	98.5422
DALYs (Dis	Low SDI	Both	50-69 yea	HIV/AIDS	Number	2003	3251039	3562471	2949133
DALYs (Dis	Low SDI	Both	50-69 yea	HIV/AIDS	Rate	2003	5758.86	6310.53	5224.07
DALYs (Dis	Low SDI	Both	50-69 yea	Sexually tr	Number	2002	29604.5	45156.2	18059.6
DALYs (Dis	Low SDI	Both	50-69 yea	Sexually tr	Rate	2002	53.7994	82.0612	32.8192
DALYs (Dis	High-midc	Both	50-69 yea	Sexually tr	Number	2012	36527.7	67702.5	20616.4
DALYs (Dis	High-midc	Both	50-69 yea	Sexually tr	Rate	2012	13.0156	24.1239	7.34609
DALYs (Dis	Low SDI	Both	50-69 yea	Sexually tr	Number	2011	29732.6	42756.6	19350.2
DALYs (Dis	Low SDI	Both	50-69 yea	Sexually tr	Rate	2011	41.9105	60.2689	27.2757
DALYs (Dis	High SDI	Both	50-69 yea	Sexually tr	Number	2001	20818.5	40275.5	10966.1
DALYs (Dis	High SDI	Both	50-69 yea	Sexually tr	Rate	2001	11.4466	22.1446	6.02945
DALYs (Dis	Low-middl	Both	50-69 yea	HIV/AIDS	Number	2002	2377941	2660999	2084530
DALYs (Dis	Low-middl	Both	50-69 yea	HIV/AIDS	Rate	2002	1667.06	1865.5	1461.36
DALYs (Dis	High SDI	Both	50-69 yea	HIV/AIDS	Number	2012	235825	281616	202192
DALYs (Dis	High SDI	Both	50-69 yea	HIV/AIDS	Rate	2012	103.175	123.209	88.4604
DALYs (Dis	High-midc	Both	50-69 yea	Sexually tr	Number	2005	33161.6	59224.8	19998.6
DALYs (Dis	High-midc	Both	50-69 yea	Sexually tr	Rate	2005	14.156	25.2819	8.53698
DALYs (Dis	Middle SDI	Both	50-69 yea	HIV/AIDS	Number	2002	2008299	2277257	1733209
DALYs (Dis	Middle SDI	Both	50-69 yea	HIV/AIDS	Rate	2002	793.022	899.226	684.397
DALYs (Dis	High SDI	Both	50-69 yea	HIV/AIDS	Number	2001	186143	202955	174310
DALYs (Dis	High SDI	Both	50-69 yea	HIV/AIDS	Rate	2001	102.347	111.59	95.8408
DALYs (Dis	High-midc	Both	50-69 yea	HIV/AIDS	Number	2013	338178	379137	311569
DALYs (Dis	High-midc	Both	50-69 yea	HIV/AIDS	Rate	2013	116.696	130.83	107.514
DALYs (Dis	Low-middl	Both	50-69 yea	HIV/AIDS	Number	2012	1890783	2249956	1657655
DALYs (Dis	Low-middl	Both	50-69 yea	HIV/AIDS	Rate	2012	992.799	1181.39	870.39
DALYs (Dis	High-midc	Both	50-69 yea	HIV/AIDS	Number	2004	278154	305800	252030
DALYs (Dis	High-midc	Both	50-69 yea	HIV/AIDS	Rate	2004	122.225	134.373	110.746
DALYs (Dis	High SDI	Both	50-69 yea	HIV/AIDS	Number	2005	223461	247933	205527
DALYs (Dis	High SDI	Both	50-69 yea	HIV/AIDS	Rate	2005	112.81	125.164	103.756
DALYs (Dis	Low SDI	Both	50-69 yea	HIV/AIDS	Number	2002	3180190	3515980	2868688
DALYs (Dis	Low SDI	Both	50-69 yea	HIV/AIDS	Rate	2002	5779.28	6389.5	5213.19
DALYs (Dis	Low-middl	Both	50-69 yea	HIV/AIDS	Number	2001	2213556	2529260	1881950
DALYs (Dis	Low-middl	Both	50-69 yea	HIV/AIDS	Rate	2001	1593.58	1820.86	1354.85
DALYs (Dis	Low SDI	Both	50-69 yea	HIV/AIDS	Number	2012	2276287	2648746	2034113
DALYs (Dis	Low SDI	Both	50-69 yea	HIV/AIDS	Rate	2012	3107.58	3616.06	2776.96
DALYs (Dis	Low-middl	Both	50-69 yea	HIV/AIDS	Number	2005	2592302	2823437	2381144
DALYs (Dis	Low-middl	Both	50-69 yea	HIV/AIDS	Rate	2005	1671.78	1820.84	1535.6
DALYs (Dis	Low-middl	Both	50-69 yea	Sexually tr	Number	2001	42651.6	59163.2	31746.3
DALYs (Dis	Low-middl	Both	50-69 yea	Sexually tr	Rate	2001	30.7057	42.5926	22.8547
DALYs (Dis	High SDI	Both	50-69 yea	Sexually tr	Number	2005	22483.5	44009.8	11822.1
DALYs (Dis	High SDI	Both	50-69 yea	Sexually tr	Rate	2005	11.3503	22.2175	5.96814
DALYs (Dis	High-midc	Both	50-69 yea	Sexually tr	Number	2003	33143	57159.4	20789.5
DALYs (Dis	High-midc	Both	50-69 yea	Sexually tr	Rate	2003	14.9836	25.8412	9.39875
DALYs (Dis	Low SDI	Both	50-69 yea	HIV/AIDS	Number	2001	3088709	3462164	2738081
DALYs (Dis	Low SDI	Both	50-69 yea	HIV/AIDS	Rate	2001	5763.66	6460.54	5109.37
DALYs (Dis	High SDI	Both	50-69 yea	Sexually tr	Number	2004	22010.6	43162.5	11554.8
DALYs (Dis	High SDI	Both	50-69 yea	Sexually tr	Rate	2004	11.3551	22.2672	5.96102
DALYs (Dis	Middle SDI	Both	50-69 yea	HIV/AIDS	Number	2006	2639101	2870421	2421196
DALYs (Dis	Middle SDI	Both	50-69 yea	HIV/AIDS	Rate	2006	901.452	980.465	827.021
DALYs (Dis	Middle SDI	Both	50-69 yea	Sexually tr	Number	2014	61638.2	113047	36291.9
DALYs (Dis	Middle SDI	Both	50-69 yea	Sexually tr	Rate	2014	16.0986	29.5256	9.47871
DALYs (Dis	High SDI	Both	50-69 yea	Sexually tr	Number	2015	25876.7	50603.5	13354.3
DALYs (Dis	High SDI	Both	50-69 yea	Sexually tr	Rate	2015	10.6901	20.9051	5.51689
DALYs (Dis	Low-middl	Both	50-69 yea	Sexually tr	Number	2015	55907	83360.8	39717.8
DALYs (Dis	Low-middl	Both	50-69 yea	Sexually tr	Rate	2015	26.5586	39.6006	18.868
DALYs (Dis	Low SDI	Both	50-69 yea	Sexually tr	Number	2015	31851.7	46073.2	20604.3
DALYs (Dis	Low SDI	Both	50-69 yea	Sexually tr	Rate	2015	39.4	56.9916	25.4871
DALYs (Dis	Middle SDI	Both	50-69 yea	HIV/AIDS	Number	2015	2248840	2558330	2037141
DALYs (Dis	Middle SDI	Both	50-69 yea	HIV/AIDS	Rate	2015	564.189	641.834	511.078
DALYs (Dis	Low-middl	Both	50-69 yea	Sexually tr	Number	2005	42751	61790.8	31281.5
DALYs (Dis	Low-middl	Both	50-69 yea	Sexually tr	Rate	2005	27.5702	39.849	20.1735
DALYs (Dis	High-midc	Both	50-69 yea	HIV/AIDS	Number	2003	268715	295271	244857
DALYs (Dis	High-midc	Both	50-69 yea	HIV/AIDS	Rate	2003	121.483	133.489	110.698
DALYs (Dis	Low SDI	Both	50-69 yea	HIV/AIDS	Number	2005	3241692	3589329	2909909
DALYs (Dis	Low SDI	Both	50-69 yea	HIV/AIDS	Rate	2005	5454.07	6038.96	4895.85
DALYs (Dis	Low-middl	Both	50-69 yea	Sexually tr	Number	2004	42099.9	60663.4	30816.4
DALYs (Dis	Low-middl	Both	50-69 yea	Sexually tr	Rate	2004	27.9314	40.2475	20.4453
DALYs (Dis	Middle SDI	Both	50-69 yea	Sexually tr	Number	2005	48171.3	84453.8	29120.7
DALYs (Dis	Middle SDI	Both	50-69 yea	Sexually tr	Rate	2005	17.0461	29.8852	10.3048
DALYs (Dis	Low SDI	Both	50-69 yea	Sexually tr	Number	2005	28945.6	41813.2	18175.5
DALYs (Dis	Low SDI	Both	50-69 yea	Sexually tr	Rate	2005	48.7003	70.3497	30.5799
DALYs (Dis	Low SDI	Both	50-69 yea	Sexually tr	Number	2004	29169.9	43849	18300.4
DALYs (Dis	Low SDI	Both	50-69 yea	Sexually tr	Rate	2004	50.3731	75.7223	31.6028
DALYs (Dis	Low-middl	Both	50-69 yea	Sexually tr	Number	2008	44873.3	64894.6	32653.1
DALYs (Dis	Low-middl	Both	50-69 yea	Sexually tr	Rate	2008	26.5935	38.4589	19.3514
DALYs (Dis	High-midc	Both	50-69 yea	HIV/AIDS	Number	2007	302978	336526	273547
DALYs (Dis	High-midc	Both	50-69 yea	HIV/AIDS	Rate	2007	122.742	136.333	110.819
DALYs (Dis	High-midc	Both	50-69 yea	HIV/AIDS	Number	2006	296934	328640	267494
DALYs (Dis	High-midc	Both	50-69 yea	HIV/AIDS	Rate	2006	123.352	136.523	111.122
DALYs (Dis	High-midc	Both	50-69 yea	Sexually tr	Number	2006	33326	60251.5	19598.7
DALYs (Dis	High-midc	Both	50-69 yea	Sexually tr	Rate	2006	13.8442	25.0295	8.14163
DALYs (Dis	High-midc	Both	50-69 yea	Sexually tr	Number	2007	33674.6	61091.4	19776
DALYs (Dis	High-midc	Both	50-69 yea	Sexually tr	Rate	2007	13.6422	24.7493	8.01163
DALYs (Dis	High-midc	Both	50-69 yea	Sexually tr	Number	2016	40824.3	75926.2	23022
DALYs (Dis	High-midc	Both	50-69 yea	Sexually tr	Rate	2016	12.8823	23.959	7.26471
DALYs (Dis	Middle SDI	Both	50-69 yea	Sexually tr	Number	2004	46816.5	81830.9	28777.4
DALYs (Dis	Middle SDI	Both	50-69 yea	Sexually tr	Rate	2004	17.1938	30.0531	10.5688
DALYs (Dis	Low SDI	Both	50-69 yea	Sexually tr	Number	2008	29192.7	41645.2	18723.3
DALYs (Dis	Low SDI	Both	50-69 yea	Sexually tr	Rate	2008	45.1344	64.3869	28.9478
DALYs (Dis	Middle SDI	Both	50-69 yea	HIV/AIDS	Number	2004	2463039	2725036	2219213
DALYs (Dis	Middle SDI	Both	50-69 yea	HIV/AIDS	Rate	2004	904.574	1000.79	815.026
DALYs (Dis	High SDI	Both	50-69 yea	HIV/AIDS	Number	2016	241220	293418	202051
DALYs (Dis	High SDI	Both	50-69 yea	HIV/AIDS	Rate	2016	98.0117	119.221	82.0967
DALYs (Dis	Middle SDI	Both	50-69 yea	HIV/AIDS	Number	2005	2604310	2830992	2387639
DALYs (Dis	Middle SDI	Both	50-69 yea	HIV/AIDS	Rate	2005	921.572	1001.79	844.9
DALYs (Dis	High SDI	Both	50-69 yea	HIV/AIDS	Number	2007	233402	262845	211883
DALYs (Dis	High SDI	Both	50-69 yea	HIV/AIDS	Rate	2007	113.041	127.301	102.619
DALYs (Dis	High SDI	Both	50-69 yea	Sexually tr	Number	2006	22839.4	44776.5	11944.3
DALYs (Dis	High SDI	Both	50-69 yea	Sexually tr	Rate	2006	11.2929	22.1396	5.90584
DALYs (Dis	Low-middl	Both	50-69 yea	HIV/AIDS	Number	2016	1964644	2310980	1721417
DALYs (Dis	Low-middl	Both	50-69 yea	HIV/AIDS	Rate	2016	903.551	1062.83	791.689
DALYs (Dis	Middle SDI	Both	50-69 yea	Sexually tr	Number	2008	51207.6	91666	30570.8
DALYs (Dis	Middle SDI	Both	50-69 yea	Sexually tr	Rate	2008	16.4247	29.4016	9.80549
DALYs (Dis	High-midc	Both	50-69 yea	HIV/AIDS	Number	2017	358763	397774	328927
DALYs (Dis	High-midc	Both	50-69 yea	HIV/AIDS	Rate	2017	110.711	122.75	101.504
DALYs (Dis	High SDI	Both	50-69 yea	HIV/AIDS	Number	2006	231575	259609	211974
DALYs (Dis	High SDI	Both	50-69 yea	HIV/AIDS	Rate	2006	114.502	128.363	104.81
DALYs (Dis	High-midc	Both	50-69 yea	HIV/AIDS	Number	2010	318379	358364	289635
DALYs (Dis	High-midc	Both	50-69 yea	HIV/AIDS	Rate	2010	119.996	135.066	109.162
DALYs (Dis	Middle SDI	Both	50-69 yea	Sexually tr	Number	2007	50402.4	89010.2	30198.1
DALYs (Dis	Middle SDI	Both	50-69 yea	Sexually tr	Rate	2007	16.6589	29.4194	9.98102
DALYs (Dis	Low SDI	Both	50-69 yea	HIV/AIDS	Number	2016	1958103	2322935	1722050
DALYs (Dis	Low SDI	Both	50-69 yea	HIV/AIDS	Rate	2016	2341.63	2777.92	2059.34
DALYs (Dis	Middle SDI	Both	50-69 yea	HIV/AIDS	Number	2008	2533569	2840634	2228883
DALYs (Dis	Middle SDI	Both	50-69 yea	HIV/AIDS	Rate	2008	812.634	911.124	714.907
DALYs (Dis	Low-middl	Both	50-69 yea	Sexually tr	Number	2006	43635.1	62895.9	32321.6
DALYs (Dis	Low-middl	Both	50-69 yea	Sexually tr	Rate	2006	27.3621	39.4399	20.2678
DALYs (Dis	Low-middl	Both	50-69 yea	HIV/AIDS	Number	2007	2398081	2679537	2125658
DALYs (Dis	Low-middl	Both	50-69 yea	HIV/AIDS	Rate	2007	1461.85	1633.42	1295.78
DALYs (Dis	High-midc	Both	50-69 yea	Sexually tr	Number	2010	34874.4	64073	20006.5
DALYs (Dis	High-midc	Both	50-69 yea	Sexually tr	Rate	2010	13.144	24.1489	7.54038
DALYs (Dis	High-midc	Both	50-69 yea	Sexually tr	Number	2009	34321.2	62920	19839.6
DALYs (Dis	High-midc	Both	50-69 yea	Sexually tr	Rate	2009	13.2651	24.3185	7.66797
DALYs (Dis	Middle SDI	Both	50-69 yea	Sexually tr	Number	2018	70081.5	129958	40517.3
DALYs (Dis	Middle SDI	Both	50-69 yea	Sexually tr	Rate	2018	15.7979	29.2954	9.1335
DALYs (Dis	Low-middl	Both	50-69 yea	HIV/AIDS	Number	2006	2527822	2772883	2282927
DALYs (Dis	Low-middl	Both	50-69 yea	HIV/AIDS	Rate	2006	1585.11	1738.78	1431.55
DALYs (Dis	High SDI	Both	50-69 yea	Sexually tr	Number	2009	23589.9	46308.3	12290.5
DALYs (Dis	High SDI	Both	50-69 yea	Sexually tr	Rate	2009	10.958	21.5112	5.70922
DALYs (Dis	High-midc	Both	50-69 yea	HIV/AIDS	Number	2008	311531	347012	281568
DALYs (Dis	High-midc	Both	50-69 yea	HIV/AIDS	Rate	2008	123.268	137.307	111.412
DALYs (Dis	High SDI	Both	50-69 yea	Sexually tr	Number	2010	23911.2	47127.9	12357.6
DALYs (Dis	High SDI	Both	50-69 yea	Sexually tr	Rate	2010	10.8824	21.4486	5.62413
DALYs (Dis	High SDI	Both	50-69 yea	HIV/AIDS	Number	2010	232756	270581	204850
DALYs (Dis	High SDI	Both	50-69 yea	HIV/AIDS	Rate	2010	105.931	123.146	93.2304
DALYs (Dis	Low SDI	Both	50-69 yea	HIV/AIDS	Number	2007	2859173	3289812	2499618
DALYs (Dis	Low SDI	Both	50-69 yea	HIV/AIDS	Rate	2007	4551.9	5237.49	3979.48
DALYs (Dis	Low SDI	Both	50-69 yea	Sexually tr	Number	2006	28977.9	41484.9	18256.5
DALYs (Dis	Low SDI	Both	50-69 yea	Sexually tr	Rate	2006	47.448	67.9268	29.8929
DALYs (Dis	High SDI	Both	50-69 yea	HIV/AIDS	Number	2009	237544	271949	212360
DALYs (Dis	High SDI	Both	50-69 yea	HIV/AIDS	Rate	2009	110.344	126.326	98.6458
DALYs (Dis	Low SDI	Both	50-69 yea	HIV/AIDS	Number	2006	3073383	3465353	2719338
DALYs (Dis	Low SDI	Both	50-69 yea	HIV/AIDS	Rate	2006	5032.31	5674.12	4452.61
DALYs (Dis	Low-middl	Both	50-69 yea	Sexually tr	Number	2010	45593.6	67675.3	33077
DALYs (Dis	Low-middl	Both	50-69 yea	Sexually tr	Rate	2010	25.4842	37.8266	18.4881
DALYs (Dis	Middle SDI	Both	50-69 yea	Sexually tr	Number	2011	55016.7	99631.9	32064.4
DALYs (Dis	Middle SDI	Both	50-69 yea	Sexually tr	Rate	2011	16.1124	29.1785	9.39048
DALYs (Dis	High SDI	Both	50-69 yea	Sexually tr	Number	2019	26853.6	52665.1	13934.2
DALYs (Dis	High SDI	Both	50-69 yea	Sexually tr	Rate	2019	10.6618	20.9098	5.53237
DALYs (Dis	Low-middl	Both	50-69 yea	Sexually tr	Number	2009	44587.2	66313.3	32240
DALYs (Dis	Low-middl	Both	50-69 yea	Sexually tr	Rate	2009	25.6712	38.1801	18.5623
DALYs (Dis	High SDI	Both	50-69 yea	Sexually tr	Number	2008	23313.6	45968.8	12087.6
DALYs (Dis	High SDI	Both	50-69 yea	Sexually tr	Rate	2008	11.0576	21.8029	5.7331
DALYs (Dis	Low-middl	Both	50-69 yea	HIV/AIDS	Number	2009	2107631	2469301	1836665
DALYs (Dis	Low-middl	Both	50-69 yea	HIV/AIDS	Rate	2009	1213.47	1421.71	1057.47
DALYs (Dis	Low-middl	Both	50-69 yea	HIV/AIDS	Number	2010	1990959	2366721	1735625
DALYs (Dis	Low-middl	Both	50-69 yea	HIV/AIDS	Rate	2010	1112.83	1322.86	970.114
DALYs (Dis	Low-middl	Both	50-69 yea	Sexually tr	Number	2019	59759.2	91350.2	41854.7
DALYs (Dis	Low-middl	Both	50-69 yea	Sexually tr	Rate	2019	25.0256	38.2551	17.5277
DALYs (Dis	Low SDI	Both	50-69 yea	Sexually tr	Number	2010	29544.5	43018	19125.1
DALYs (Dis	Low SDI	Both	50-69 yea	Sexually tr	Rate	2010	42.9814	62.5828	27.8232
DALYs (Dis	Low SDI	Both	50-69 yea	HIV/AIDS	Number	2009	2471683	2918681	2155577
DALYs (Dis	Low SDI	Both	50-69 yea	HIV/AIDS	Rate	2009	3707.7	4378.22	3233.51
DALYs (Dis	Low SDI	Both	50-69 yea	Sexually tr	Number	2009	29331.5	41882.7	18671
DALYs (Dis	Low SDI	Both	50-69 yea	Sexually tr	Rate	2009	43.9992	62.827	28.0077
DALYs (Dis	Low SDI	Both	50-69 yea	HIV/AIDS	Number	2010	2399174	2825349	2111594
DALYs (Dis	Low SDI	Both	50-69 yea	HIV/AIDS	Rate	2010	3490.33	4110.33	3071.96
DALYs (Dis	Low SDI	Both	50-69 yea	Sexually tr	Number	2019	33804.7	49233	22201.8
DALYs (Dis	Low SDI	Both	50-69 yea	Sexually tr	Rate	2019	36.3853	52.9914	23.8967
DALYs (Dis	Low SDI	Both	50-69 yea	HIV/AIDS	Number	2013	2178705	2537513	1940585
DALYs (Dis	Low SDI	Both	50-69 yea	HIV/AIDS	Rate	2013	2879.01	3353.15	2564.35
DALYs (Dis	Middle SDI	Both	50-69 yea	HIV/AIDS	Number	2019	2359584	2704341	2117270
DALYs (Dis	Middle SDI	Both	50-69 yea	HIV/AIDS	Rate	2019	516.347	591.79	463.321
DALYs (Dis	Low-middl	Both	50-69 yea	Sexually tr	Number	2013	49524.8	73749.5	35266.9
DALYs (Dis	Low-middl	Both	50-69 yea	Sexually tr	Rate	2013	25.1606	37.4677	17.917
DALYs (Dis	High-midc	Both	50-69 yea	HIV/AIDS	Number	2012	332861	373473	305375
DALYs (Dis	High-midc	Both	50-69 yea	HIV/AIDS	Rate	2012	118.606	133.077	108.812
DALYs (Dis	Middle SDI	Both	50-69 yea	HIV/AIDS	Number	2010	2337458	2742980	2008329
DALYs (Dis	Middle SDI	Both	50-69 yea	HIV/AIDS	Rate	2010	706.803	829.425	607.28
DALYs (Dis	High SDI	Both	50-69 yea	Sexually tr	Number	2012	24661.7	48341.2	12811.7
DALYs (Dis	High SDI	Both	50-69 yea	Sexually tr	Rate	2012	10.7897	21.1496	5.60519
DALYs (Dis	Middle SDI	Both	50-69 yea	HIV/AIDS	Number	2009	2426928	2795023	2098727
DALYs (Dis	Middle SDI	Both	50-69 yea	HIV/AIDS	Rate	2009	756.036	870.704	653.795
DALYs (Dis	High-midc	Both	50-69 yea	Sexually tr	Number	2011	35580.6	66027.4	20391.6
DALYs (Dis	High-midc	Both	50-69 yea	Sexually tr	Rate	2011	13.055	24.2264	7.48197
DALYs (Dis	Middle SDI	Both	50-69 yea	Sexually tr	Number	2013	59164.2	108163	34833.9
DALYs (Dis	Middle SDI	Both	50-69 yea	Sexually tr	Rate	2013	16.0924	29.4199	9.47466
DALYs (Dis	Low SDI	Both	50-69 yea	Sexually tr	Number	2013	30413.1	43514.2	19572.8
DALYs (Dis	Low SDI	Both	50-69 yea	Sexually tr	Rate	2013	40.1889	57.5011	25.8641
DALYs (Dis	High-midc	Both	50-69 yea	HIV/AIDS	Number	2011	327930	367479	299240
DALYs (Dis	High-midc	Both	50-69 yea	HIV/AIDS	Rate	2011	120.322	134.833	109.795
DALYs (Dis	Middle SDI	Both	50-69 yea	Sexually tr	Number	2009	52106.4	93888.3	30867.3
DALYs (Dis	Middle SDI	Both	50-69 yea	Sexually tr	Rate	2009	16.2322	29.248	9.61577
DALYs (Dis	Low-middl	Both	50-69 yea	Sexually tr	Number	2012	47735.5	72506	34194.6
DALYs (Dis	Low-middl	Both	50-69 yea	Sexually tr	Rate	2012	25.0646	38.0709	17.9547
DALYs (Dis	High SDI	Both	50-69 yea	HIV/AIDS	Number	2011	234227	276470	203137
DALYs (Dis	High SDI	Both	50-69 yea	HIV/AIDS	Rate	2011	104.516	123.366	90.6432
DALYs (Dis	Middle SDI	Both	50-69 yea	Sexually tr	Number	2012	56913.5	103396	33219.3
DALYs (Dis	Middle SDI	Both	50-69 yea	Sexually tr	Rate	2012	16.0923	29.2353	9.39275
DALYs (Dis	Middle SDI	Both	50-69 yea	HIV/AIDS	Number	2013	2109531	2473345	1872335
DALYs (Dis	Middle SDI	Both	50-69 yea	HIV/AIDS	Rate	2013	573.783	672.739	509.267
DALYs (Dis	High-midc	Both	50-69 yea	Sexually tr	Number	2015	40032.3	74979.8	22435.4
DALYs (Dis	High-midc	Both	50-69 yea	Sexually tr	Rate	2015	12.972	24.2963	7.26993
DALYs (Dis	Low-middl	Both	50-69 yea	HIV/AIDS	Number	2011	1918005	2289695	1677616
DALYs (Dis	Low-middl	Both	50-69 yea	HIV/AIDS	Rate	2011	1039.61	1241.08	909.317
DALYs (Dis	Low SDI	Both	50-69 yea	Sexually tr	Number	2012	30039.7	43073.6	19533.7
DALYs (Dis	Low SDI	Both	50-69 yea	Sexually tr	Rate	2012	41.0101	58.8038	26.6673
DALYs (Dis	High SDI	Both	50-69 yea	HIV/AIDS	Number	2015	241201	292719	202584
DALYs (Dis	High SDI	Both	50-69 yea	HIV/AIDS	Rate	2015	99.6441	120.927	83.6908
DALYs (Dis	High-midc	Both	50-69 yea	HIV/AIDS	Number	2014	354822	393392	329532
DALYs (Dis	High-midc	Both	50-69 yea	HIV/AIDS	Rate	2014	118.548	131.434	110.098
DALYs (Dis	Low-middl	Both	50-69 yea	HIV/AIDS	Number	2015	1969740	2301234	1742926
DALYs (Dis	Low-middl	Both	50-69 yea	HIV/AIDS	Rate	2015	935.726	1093.2	827.978
DALYs (Dis	High-midc	Both	50-69 yea	Sexually tr	Number	2014	38759.4	72628.1	21732.3
DALYs (Dis	High-midc	Both	50-69 yea	Sexually tr	Rate	2014	12.9497	24.2654	7.26086
DALYs (Dis	Low SDI	Both	50-69 yea	HIV/AIDS	Number	2015	2012355	2376786	1776891
DALYs (Dis	Low SDI	Both	50-69 yea	HIV/AIDS	Rate	2015	2489.24	2940.04	2197.98
DALYs (Dis	Low SDI	Both	50-69 yea	HIV/AIDS	Number	2011	2352883	2755911	2094592
DALYs (Dis	Low SDI	Both	50-69 yea	HIV/AIDS	Rate	2011	3316.58	3884.68	2952.5
DALYs (Dis	High-midc	Both	50-69 yea	Sexually tr	Number	2013	37643.7	69993	21221.2
DALYs (Dis	High-midc	Both	50-69 yea	Sexually tr	Rate	2013	12.9898	24.1527	7.32285
DALYs (Dis	High SDI	Both	50-69 yea	Sexually tr	Number	2013	25059.8	48942.2	12911.7
DALYs (Dis	High SDI	Both	50-69 yea	Sexually tr	Rate	2013	10.7589	21.0123	5.54337
DALYs (Dis	High SDI	Both	50-69 yea	Sexually tr	Number	2014	25453.1	49758.1	13125.1
DALYs (Dis	High SDI	Both	50-69 yea	Sexually tr	Rate	2014	10.7276	20.9714	5.53181
DALYs (Dis	High SDI	Both	50-69 yea	HIV/AIDS	Number	2014	239683	289111	202612
DALYs (Dis	High SDI	Both	50-69 yea	HIV/AIDS	Rate	2014	101.018	121.851	85.3942
DALYs (Dis	Middle SDI	Both	50-69 yea	HIV/AIDS	Number	2016	2369133	2672365	2150466
DALYs (Dis	Middle SDI	Both	50-69 yea	HIV/AIDS	Rate	2016	572.287	645.536	519.466
DALYs (Dis	Middle SDI	Both	50-69 yea	HIV/AIDS	Number	2012	2137276	2534353	1869572
DALYs (Dis	Middle SDI	Both	50-69 yea	HIV/AIDS	Rate	2012	604.315	716.588	528.621
DALYs (Dis	Middle SDI	Both	50-69 yea	Sexually tr	Number	2015	64399.9	117307	37722.8
DALYs (Dis	Middle SDI	Both	50-69 yea	Sexually tr	Rate	2015	16.1567	29.4299	9.46391
DALYs (Dis	Low-middl	Both	50-69 yea	Sexually tr	Number	2014	52216.2	77573.5	37202.5
DALYs (Dis	Low-middl	Both	50-69 yea	Sexually tr	Rate	2014	25.6486	38.1042	18.2739

DALYs (Dis	Middle SDI	Both	50-69 yea	HIV/AIDS	Rate	2018	538.095	613.291	485.616
DALYs (Dis	Low SDI	Both	50-69 yea	HIV/AIDS	Number	2017	1884220	2246428	1643081
DALYs (Dis	Low SDI	Both	50-69 yea	HIV/AIDS	Rate	2017	2177.24	2595.77	1898.6
DALYs (Dis	Middle SDI	Both	50-69 yea	Sexually tra	Number	2017	68434	126087	39581.3
DALYs (Dis	Middle SDI	Both	50-69 yea	Sexually tra	Rate	2017	15.9502	29.3876	9.22539
DALYs (Dis	Low-middl	Both	50-69 yea	Sexually tra	Number	2017	58469.2	86506.1	40929.8
DALYs (Dis	Low-middl	Both	50-69 yea	Sexually tra	Rate	2017	26.0586	38.5541	18.2416
DALYs (Dis	High-midc	Both	50-69 yea	HIV/AIDS	Number	2018	350329	391111	318788
DALYs (Dis	High-midc	Both	50-69 yea	HIV/AIDS	Rate	2018	105.724	118.031	96.2054
DALYs (Dis	Low SDI	Both	50-69 yea	Sexually tra	Number	2016	32285.2	46902.7	20947.8
DALYs (Dis	Low SDI	Both	50-69 yea	Sexually tra	Rate	2016	38.6088	56.0894	25.0507
DALYs (Dis	High-midc	Both	50-69 yea	HIV/AIDS	Number	2019	347627	391924	315549
DALYs (Dis	High-midc	Both	50-69 yea	HIV/AIDS	Rate	2019	102.941	116.059	93.4422
DALYs (Dis	Low SDI	Both	50-69 yea	Sexually tra	Number	2017	32811.7	47374.3	21196.7
DALYs (Dis	Low SDI	Both	50-69 yea	Sexually tra	Rate	2017	37.9143	54.7416	24.493
DALYs (Dis	High-midc	Both	50-69 yea	Sexually tra	Number	2019	42663.1	80872.8	23772
DALYs (Dis	High-midc	Both	50-69 yea	Sexually tra	Rate	2019	12.6336	23.9485	7.03948
DALYs (Dis	High-midc	Both	50-69 yea	Sexually tra	Number	2018	41918	78850.4	23496.9
DALYs (Dis	High-midc	Both	50-69 yea	Sexually tra	Rate	2018	12.6502	23.7959	7.09101
DALYs (Dis	Middle SDI	Both	50-69 yea	Sexually tra	Number	2019	71804.7	132601	41368.9
DALYs (Dis	Middle SDI	Both	50-69 yea	Sexually tra	Rate	2019	15.713	29.017	9.05274
DALYs (Dis	Middle SDI	Both	50-69 yea	HIV/AIDS	Number	2017	2427963	2739408	2208679
DALYs (Dis	Middle SDI	Both	50-69 yea	HIV/AIDS	Rate	2017	565.896	638.486	514.787
DALYs (Dis	High SDI	Both	50-69 yea	HIV/AIDS	Number	2018	234568	288395	194222
DALYs (Dis	High SDI	Both	50-69 yea	HIV/AIDS	Rate	2018	93.8366	115.37	77.6966
DALYs (Dis	High SDI	Both	50-69 yea	HIV/AIDS	Number	2019	232366	286447	191267
DALYs (Dis	High SDI	Both	50-69 yea	HIV/AIDS	Rate	2019	92.2574	113.729	75.9393
DALYs (Dis	High SDI	Both	50-69 yea	Sexually tra	Number	2018	26635.5	52280.5	13806.3
DALYs (Dis	High SDI	Both	50-69 yea	Sexually tra	Rate	2018	10.6553	20.9143	5.5231
DALYs (Dis	Low-middl	Both	50-69 yea	HIV/AIDS	Number	2018	1967730	2357337	1680504
DALYs (Dis	Low-middl	Both	50-69 yea	HIV/AIDS	Rate	2018	849.836	1018.1	725.787
DALYs (Dis	Low-middl	Both	50-69 yea	HIV/AIDS	Number	2019	1988112	2377598	1694161
DALYs (Dis	Low-middl	Both	50-69 yea	HIV/AIDS	Rate	2019	832.57	995.677	709.471
DALYs (Dis	Low-middl	Both	50-69 yea	Sexually tra	Number	2018	58981.3	88764.6	40963.8
DALYs (Dis	Low-middl	Both	50-69 yea	Sexually tra	Rate	2018	25.4732	38.3362	17.6917
DALYs (Dis	Low SDI	Both	50-69 yea	HIV/AIDS	Number	2019	1794163	2142089	1546087
DALYs (Dis	Low SDI	Both	50-69 yea	HIV/AIDS	Rate	2019	1931.13	2305.61	1664.11
Prevalence	Middle SDI	Both	50-69 yea	Sexually tra	Number	1990	4.9E+07	5.5E+07	4.3E+07
Prevalence	Middle SDI	Both	50-69 yea	Sexually tra	Rate	1990	25896.2	29512.4	22675.8
Prevalence	Middle SDI	Both	50-69 yea	Sexually tra	Number	1991	5.1E+07	5.8E+07	4.4E+07
Prevalence	Middle SDI	Both	50-69 yea	Sexually tra	Rate	1991	26318.4	30046.1	23036.3
Prevalence	Middle SDI	Both	50-69 yea	Sexually tra	Number	1992	5.2E+07	6E+07	4.6E+07
Prevalence	Middle SDI	Both	50-69 yea	Sexually tra	Rate	1992	26646.4	30395.5	23315.3
Prevalence	Middle SDI	Both	50-69 yea	Sexually tra	Number	1993	5.4E+07	6.1E+07	4.7E+07
Prevalence	Middle SDI	Both	50-69 yea	Sexually tra	Rate	1993	26876.5	30628.4	23502.9
Prevalence	Middle SDI	Both	50-69 yea	Sexually tra	Number	1994	5.5E+07	6.3E+07	4.8E+07
Prevalence	Middle SDI	Both	50-69 yea	Sexually tra	Rate	1994	27022.2	30810.8	23623.1
Prevalence	Middle SDI	Both	50-69 yea	Sexually tra	Number	1995	5.6E+07	6.4E+07	4.9E+07
Prevalence	Middle SDI	Both	50-69 yea	Sexually tra	Rate	1995	27080.8	30851.9	23674.2
Prevalence	Middle SDI	Both	50-69 yea	Sexually tra	Number	1996	5.8E+07	6.6E+07	5E+07
Prevalence	Middle SDI	Both	50-69 yea	Sexually tra	Rate	1996	26988.3	30755.5	23607.4
Prevalence	Middle SDI	Both	50-69 yea	Sexually tra	Number	1997	5.8E+07	6.7E+07	5.1E+07
Prevalence	Middle SDI	Both	50-69 yea	Sexually tra	Rate	1997	26730.2	30456.4	23371.8
Prevalence	Middle SDI	Both	50-69 yea	Sexually tra	Number	1998	5.9E+07	6.7E+07	5.2E+07
Prevalence	Middle SDI	Both	50-69 yea	Sexually tra	Rate	1998	26418.2	30077.1	23115.1
Prevalence	Middle SDI	Both	50-69 yea	Sexually tra	Number	1999	6E+07	6.9E+07	5.3E+07
Prevalence	Middle SDI	Both	50-69 yea	Sexually tra	Rate	1999	26141.8	29735.8	22940.6
Prevalence	Middle SDI	Both	50-69 yea	Sexually tra	Number	2000	6.2E+07	7E+07	5.4E+07
Prevalence	Middle SDI	Both	50-69 yea	Sexually tra	Rate	2000	26040.3	29598.2	22834.8
Prevalence	Middle SDI	Both	50-69 yea	Sexually tra	Number	2001	6.4E+07	7.3E+07	5.6E+07
Prevalence	Middle SDI	Both	50-69 yea	Sexually tra	Rate	2001	26063.3	29682.6	22840.6
Prevalence	Middle SDI	Both	50-69 yea	Sexually tra	Number	2002	6.6E+07	7.5E+07	5.8E+07
Prevalence	Middle SDI	Both	50-69 yea	Sexually tra	Rate	2002	26097.2	29740.2	22903.4
Prevalence	Middle SDI	Both	50-69 yea	Sexually tra	Number	2003	6.9E+07	7.8E+07	6E+07
Prevalence	Middle SDI	Both	50-69 yea	Sexually tra	Rate	2003	26131.4	29761.9	22928.2
Prevalence	Middle SDI	Both	50-69 yea	Sexually tra	Number	2004	7.1E+07	8.1E+07	6.3E+07
Prevalence	Middle SDI	Both	50-69 yea	Sexually tra	Rate	2004	26153.4	29780	22961.8
Prevalence	Middle SDI	Both	50-69 yea	Sexually tra	Number	2005	7.4E+07	8.4E+07	6.5E+07
Prevalence	Middle SDI	Both	50-69 yea	Sexually tra	Rate	2005	26172.9	29771.8	22984.3
Prevalence	Middle SDI	Both	50-69 yea	Sexually tra	Number	2006	7.6E+07	8.7E+07	6.7E+07
Prevalence	Middle SDI	Both	50-69 yea	Sexually tra	Rate	2006	26073.1	29664	22841.3
Prevalence	Middle SDI	Both	50-69 yea	Sexually tra	Number	2007	7.8E+07	8.9E+07	6.8E+07
Prevalence	Middle SDI	Both	50-69 yea	Sexually tra	Rate	2007	25843.7	29479.5	22615.2
Prevalence	Middle SDI	Both	50-69 yea	Sexually tra	Number	2008	8E+07	9.1E+07	7E+07
Prevalence	Middle SDI	Both	50-69 yea	Sexually tra	Rate	2008	25579.8	29207.1	22365.6
Prevalence	Middle SDI	Both	50-69 yea	Sexually tra	Number	2009	8.1E+07	9.3E+07	7.1E+07
Prevalence	Middle SDI	Both	50-69 yea	Sexually tra	Rate	2009	25370.2	29010.8	22149.2
Prevalence	Middle SDI	Both	50-69 yea	Sexually tra	Number	2010	8.4E+07	9.6E+07	7.3E+07
Prevalence	Middle SDI	Both	50-69 yea	Sexually tra	Rate	2010	25311.2	28988.5	22048.9
Prevalence	Middle SDI	Both	50-69 yea	Sexually tra	Number	2011	8.7E+07	1E+08	7.6E+07
Prevalence	Middle SDI	Both	50-69 yea	Sexually tra	Rate	2011	25429	29144.4	22118
Prevalence	Middle SDI	Both	50-69 yea	Sexually tra	Number	2012	9.1E+07	1E+08	7.9E+07
Prevalence	Middle SDI	Both	50-69 yea	Sexually tra	Rate	2012	25658.9	29413.1	22291.8
Prevalence	Middle SDI	Both	50-69 yea	Sexually tra	Number	2013	9.5E+07	1.1E+08	8.3E+07
Prevalence	Middle SDI	Both	50-69 yea	Sexually tra	Rate	2013	25912.9	29704.7	22549.2
Prevalence	Middle SDI	Both	50-69 yea	Sexually tra	Number	2014	1E+08	1.1E+08	8.7E+07
Prevalence	Middle SDI	Both	50-69 yea	Sexually tra	Rate	2014	26112.4	29923.9	22665.8
Prevalence	Middle SDI	Both	50-69 yea	Sexually tra	Number	2015	1E+08	1.2E+08	9.1E+07
Prevalence	Middle SDI	Both	50-69 yea	Sexually tra	Rate	2015	26190.4	30007.9	22745.4
Prevalence	Middle SDI	Both	50-69 yea	Sexually tra	Number	2016	1.1E+08	1.2E+08	9.4E+07
Prevalence	Middle SDI	Both	50-69 yea	Sexually tra	Rate	2016	26154	29966.9	22739.1
Prevalence	Middle SDI	Both	50-69 yea	Sexually tra	Number	2017	1.1E+08	1.3E+08	9.8E+07
Prevalence	Middle SDI	Both	50-69 yea	Sexually tra	Rate	2017	26116.7	29855.9	22751.4
Prevalence	Middle SDI	Both	50-69 yea	Sexually tra	Number	2018	1.2E+08	1.3E+08	1E+08
Prevalence	Middle SDI	Both	50-69 yea	Sexually tra	Rate	2018	26123.6	29905.1	22750.4
Prevalence	Middle SDI	Both	50-69 yea	Sexually tra	Number	2019	1.2E+08	1.4E+08	1E+08
Prevalence	Middle SDI	Both	50-69 yea	Sexually tra	Rate	2019	26176.9	29938.4	22770.9
Prevalence	Low SDI	Both	50-69 yea	HIV/AIDS	Number	1990	321465	385499	259748
Prevalence	Low SDI	Both	50-69 yea	HIV/AIDS	Rate	1990	731.754	877.514	591.267
Prevalence	Low SDI	Both	50-69 yea	HIV/AIDS	Number	1991	384161	459570	313922
Prevalence	Low SDI	Both	50-69 yea	HIV/AIDS	Rate	1991	859.101	1027.74	702.026
Prevalence	Low SDI	Both	50-69 yea	HIV/AIDS	Number	1992	446394	529695	370938
Prevalence	Low SDI	Both	50-69 yea	HIV/AIDS	Rate	1992	980.895	1163.94	815.091
Prevalence	Low SDI	Both	50-69 yea	HIV/AIDS	Number	1993	504207	594620	422294
Prevalence	Low SDI	Both	50-69 yea	HIV/AIDS	Rate	1993	1090.25	1285.75	913.131
Prevalence	Low SDI	Both	50-69 yea	HIV/AIDS	Number	1994	558434	653270	471372
Prevalence	Low SDI	Both	50-69 yea	HIV/AIDS	Rate	1994	1186.38	1387.86	1001.42
Prevalence	Low SDI	Both	50-69 yea	HIV/AIDS	Number	1995	607043	709118	517316
Prevalence	Low SDI	Both	50-69 yea	HIV/AIDS	Rate	1995	1266.65	1479.64	1079.43
Prevalence	Low SDI	Both	50-69 yea	HIV/AIDS	Number	1996	647236	754653	558666
Prevalence	Low SDI	Both	50-69 yea	HIV/AIDS	Rate	1996	1326.29	1546.41	1144.8
Prevalence	Low SDI	Both	50-69 yea	HIV/AIDS	Number	1997	678959	787144	585285
Prevalence	Low SDI	Both	50-69 yea	HIV/AIDS	Rate	1997	1366.33	1584.05	1177.82
Prevalence	Low SDI	Both	50-69 yea	HIV/AIDS	Number	1998	701458	814540	600364
Prevalence	Low SDI	Both	50-69 yea	HIV/AIDS	Rate	1998	1387.58	1611.27	1187.6
Prevalence	Low SDI	Both	50-69 yea	HIV/AIDS	Number	1999	716631	839604	608939
Prevalence	Low SDI	Both	50-69 yea	HIV/AIDS	Rate	1999	1394.31	1633.57	1184.78
Prevalence	Low SDI	Both	50-69 yea	HIV/AIDS	Number	2000	719357	846918	605056
Prevalence	Low SDI	Both	50-69 yea	HIV/AIDS	Rate	2000	1379.34	1623.94	1160.18
Prevalence	Low SDI	Both	50-69 yea	HIV/AIDS	Number	2001	734601	872133	612818
Prevalence	Low SDI	Both	50-69 yea	HIV/AIDS	Rate	2001	1370.8	1627.44	1143.54
Prevalence	Low SDI	Both	50-69 yea	HIV/AIDS	Number	2002	744906	901461	613526
Prevalence	Low SDI	Both	50-69 yea	HIV/AIDS	Rate	2002	1353.7	1638.2	1114.95
Prevalence	Low SDI	Both	50-69 yea	HIV/AIDS	Number	2003	751033	914849	609400
Prevalence	Low SDI	Both	50-69 yea	HIV/AIDS	Rate	2003	1330.37	1620.56	1079.49
Prevalence	Low SDI	Both	50-69 yea	HIV/AIDS	Number	2004	753838	934191	606194
Prevalence	Low SDI	Both	50-69 yea	HIV/AIDS	Rate	2004	1301.79	1613.24	1046.83
Prevalence	Low SDI	Both	50-69 yea	HIV/AIDS	Number	2005	755724	947196	603446
Prevalence	Low SDI	Both	50-69 yea	HIV/AIDS	Rate	2005	1271.49	1593.63	1015.28
Prevalence	Low SDI	Both	50-69 yea	HIV/AIDS	Number	2006	764883	962279	608537
Prevalence	Low SDI	Both	50-69 yea	HIV/AIDS	Rate	2006	1252.41	1575.62	996.411
Prevalence	Low SDI	Both	50-69 yea	HIV/AIDS	Number	2007	784382	985166	630888
Prevalence	Low SDI	Both	50-69 yea	HIV/AIDS	Rate	2007	1248.76	1568.42	1004.39
Prevalence	Low SDI	Both	50-69 yea	HIV/AIDS	Number	2008	814946	1010643	665640
Prevalence	Low SDI	Both	50-69 yea	HIV/AIDS	Rate	2008	1259.97	1562.54	1029.13
Prevalence	Low SDI	Both	50-69 yea	HIV/AIDS	Number	2009	855010	1048576	706600
Prevalence	Low SDI	Both	50-69 yea	HIV/AIDS	Rate	2009	1282.57	1572.94	1059.95
Prevalence	Low SDI	Both	50-69 yea	HIV/AIDS	Number	2010	901390	1094326	754753
Prevalence	Low SDI	Both	50-69 yea	HIV/AIDS	Rate	2010	1311.35	1592.03	1098.02
Prevalence	Low SDI	Both	50-69 yea	HIV/AIDS	Number	2011	954695	1144389	805897
Prevalence	Low SDI	Both	50-69 yea	HIV/AIDS	Rate	2011	1345.72	1613.11	1135.98
Prevalence	Low SDI	Both	50-69 yea	HIV/AIDS	Number	2012	1014605	1202917	865183
Prevalence	Low SDI	Both	50-69 yea	HIV/AIDS	Rate	2012	1385.13	1642.22	1181.14
Prevalence	Low SDI	Both	50-69 yea	HIV/AIDS	Number	2013	1083097	1271377	933634
Prevalence	Low SDI	Both	50-69 yea	HIV/AIDS	Rate	2013	1431.24	1680.04	1233.74
Prevalence	Low SDI	Both	50-69 yea	HIV/AIDS	Number	2014	1158632	1341393	1010317
Prevalence	Low SDI	Both	50-69 yea	HIV/AIDS	Rate	2014	1481.58	1715.28	1291.93
Prevalence	Low SDI	Both	50-69 yea	HIV/AIDS	Number	2015	1242371	1426294	1093195
Prevalence	Low SDI	Both	50-69 yea	HIV/AIDS	Rate	2015	1536.79	1764.3	1352.26
Prevalence	Low SDI	Both	50-69 yea	HIV/AIDS	Number	2016	1333185	1513247	1180800
Prevalence	Low SDI	Both	50-69 yea	HIV/AIDS	Rate	2016	1594.31	1809.64	1412.08
Prevalence	Low SDI	Both	50-69 yea	HIV/AIDS	Number	2017	1430556	1605728	1275182
Prevalence	Low SDI	Both	50-69 yea	HIV/AIDS	Rate	2017	1653.02	1855.44	1473.49
Prevalence	Low SDI	Both	50-69 yea	HIV/AIDS	Number	2018	1535132	1708269	1380713
Prevalence	Low SDI	Both	50-69 yea	HIV/AIDS	Rate	2018	1712.82	1906	1540.53
Prevalence	Low SDI	Both	50-69 yea	HIV/AIDS	Number	2019	1647063	1809555	1488495
Prevalence	Low SDI	Both	50-69 yea	HIV/AIDS	Rate	2019	1772.8	1947.69	1602.13
Prevalence	Low SDI	Both	50-69 yea	Sexually tra	Number	1990	1.5E+07	1.7E+07	1.3E+07
Prevalence	Low SDI	Both	50-69 yea	Sexually tra	Rate	1990	33431.4	37565.2	29700.3
Prevalence	Low SDI	Both	50-69 yea	Sexually tra	Number	1991	1.5E+07	1.7E+07	1.3E+07
Prevalence	Low SDI	Both	50-69 yea	Sexually tra	Rate	1991	33666.6	37716.8	29993.2
Prevalence	Low SDI	Both	50-69 yea	Sexually tra	Number	1992	1.5E+07	1.7E+07	1.4E+07
Prevalence	Low SDI	Both	50-69 yea	Sexually tra	Rate	1992	33878.4	37914.9	30251.3
Prevalence	Low SDI	Both	50-69 yea	Sexually tra	Number	1993	1.6E+07	1.8E+07	1.4E+07
Prevalence	Low SDI	Both	50-69 yea	Sexually tra	Rate	1993	34037.5	38055.2	30434.8
Prevalence	Low SDI	Both	50-69 yea	Sexually tra	Number	1994	1.6E+07	1.8E+07	1.4E+07
Prevalence	Low SDI	Both	50-69 yea	Sexually tra	Rate	1994	34172.5	38155.9	30602.8
Prevalence	Low SDI	Both	50-69 yea	Sexually tra	Number	1995	1.6E+07	1.8E+07	1.5E+07
Prevalence	Low SDI	Both	50-69 yea	Sexually tra	Rate	1995	34235.2	38149.8	30708.7
Prevalence	Low SDI	Both	50-69 yea	Sexually tra	Number	1996	1.7E+07	1.9E+07	1.5E+07
Prevalence	Low SDI	Both	50-69 yea	Sexually tra	Rate	1996	34272.6	38222.3	30701.4
Prevalence	Low SDI	Both	50-69 yea	Sexually tra	Number	1997	1.7E+07	1.9E+07	1.5E+07
Prevalence	Low SDI	Both	50-69 yea	Sexually tra	Rate	1997	34321.4	38291.6	30712.1
Prevalence	Low SDI	Both	50-69 yea	Sexually tra	Number	1998	1.7E+07	1.9E+07	1.6E+07
Prevalence	Low SDI	Both	50-69 yea	Sexually tra	Rate	1998	34379.6	38344.6	30751.3
Prevalence	Low SDI	Both	50-69 yea	Sexually tra	Number	1999	1.8E+07	2E+07	1.6E+07
Prevalence	Low SDI	Both	50-69 yea	Sexually tra	Rate	1999	34417.2	38370.7	30761.8
Prevalence	Low SDI	Both	50-69 yea	Sexually tra	Number	2000	1.8E+07	2E+07	1.6E+07
Prevalence	Low SDI	Both	50-69 yea	Sexually tra	Rate	2000	34403.9	38356.5	30725.8
Prevalence	Low SDI	Both	50-69 yea	Sexually tra	Number	2001	1.8E+07	2E+07	1.6E+07
Prevalence	Low SDI	Both	50-69 yea	Sexually tra	Rate	2001	34235.7	38198.7	30567.2
Prevalence	Low SDI	Both	50-69 yea	Sexually tra	Number	2002	1.9E+07	2.1E+07	1.7E+07
Prevalence	Low SDI	Both	50-69 yea	Sexually tra	Rate	2002	33870	37773.8	30278.5
Prevalence	Low SDI	Both	50-69 yea	Sexually tra	Number	2003	1.9E+07	2.1E+07	1.7E+07
Prevalence	Low SDI	Both	50-69 yea	Sexually tra	Rate	2003	33447.8	37365.4	29934.6
Prevalence	Low SDI	Both	50-69 yea	Sexually tra	Number	2004	1.9E+07	2.1E+07	1.7E+07
Prevalence	Low SDI	Both	50-69 yea	Sexually tra	Rate	2004	33099.5	36977.2	29623.1
Prevalence	Low SDI	Both	50-69 yea	Sexually tra	Number	2005	2E+07	2.2E+07	1.8E+07
Prevalence	Low SDI	Both	50-69 yea	Sexually tra	Rate	2005	32954.7	36846.6	29460
Prevalence	Low SDI	Both	50-69 yea	Sexually tra	Number	2006	2E+07	2.3E+07	1.8E+07
Prevalence	Low SDI	Both	50-69 yea	Sexually tra	Rate	2006	32934	36857.8	29436
Prevalence	Low SDI	Both	50-69 yea	Sexually tra	Number	2007	2.1E+07	2.3E+07	1.8E+07
Prevalence	Low SDI	Both	50-69 yea	Sexually tra	Rate	2007	32910.4	36842.2	29381
Prevalence	Low SDI	Both	50-69 yea	Sexually tra	Number	2008	2.1E+07	2.4E+07	1.9E+07
Prevalence	Low SDI	Both	50-69 yea	Sexually tra	Rate	2008	32886.2	36852.3	29300.2
Prevalence	Low SDI	Both	50-69 yea	Sexually tra	Number	2009	2.2E+07	2.5E+07	1.9E+07
Prevalence	Low SDI	Both	50-69 yea	Sexually tra	Rate	2009	32866.1	36927.8	29248.3
Prevalence	Low SDI	Both	50-69 yea	Sexually tra	Number	2010	2.3E+07	2.5E+07	2E+07
Prevalence	Low SDI	Both	50-69 yea	Sexually tra	Rate	2010	32852.7	36950.5	29193.2
Prevalence	Low SDI	Both	50-69 yea	Sexually tra	Number	2011	2.3E+07	2.6E+07	2.1E+07
Prevalence	Low SDI	Both	50-69 yea	Sexually tra	Rate	2011	32803.9	36881.2	29106.4
Prevalence	Low SDI	Both	50-69 yea	Sexually tra	Number	2012	2.4E+07	2.7E+07	2.1E+07
Prevalence	Low SDI	Both	50-69 yea	Sexually tra	Rate	2012	32725.6	36842.5	28998.9
Prevalence	Low SDI	Both	50-69 yea	Sexually tra	Number	2013	2.5E+07	2.8E+07	2.2E+07
Prevalence	Low SDI	Both	50-69 yea	Sexually tra	Rate	2013	32643.8	36807	28886.3
Prevalence	Low SDI	Both	50-69 yea	Sexually tra	Number	2014	2.5E+07	2.9E+07	2.3E+07
Prevalence	Low SDI	Both	50-69 yea	Sexually tra	Rate	2014	32583.3	36771.9	28803.7
Prevalence	Low SDI	Both	50-69 yea	Sexually tra	Number	2015	2.6E+07	3E+07	2.3E+07
Prevalence	Low SDI	Both	50-69 yea	Sexually tra	Rate	2015	32584.7	36811.1	28811.1
Prevalence	Low SDI	Both	50-69 yea	Sexually tra	Number	2016	2.8E+07	3.1E+07	2.4E+07
Prevalence	Low SDI	Both	50-69 yea	Sexually tra	Rate	2016	32924.2	37186.7	29068.7
Prevalence	Low SDI	Both	50-69 yea	Sexually tra	Number	2017	2.9E+07	3.3E+07	2.5E+07
Prevalence	Low SDI	Both	50-69 yea	Sexually tra	Rate	2017	33262.4	37563	29363.9
Prevalence	Low SDI	Both	50-69 yea	Sexually tra	Number	2018	3E+07	3.4E+07	2.6E+07
Prevalence	Low SDI	Both	50-69 yea	Sexually tra	Rate	2018	33282.9	37574	29430.7
Prevalence	Low SDI	Both	50-69 yea	Sexually tra	Number	2019	3.1E+07	3.5E+07	2.7E+07
Prevalence	Low SDI	Both	50-69 yea	Sexually tra	Rate	2019	33271	37593.9	29439.1
Prevalence	Low-middl	Both	50-69 yea	HIV/AIDS	Number	1990	117225	152469	92387.4
Prevalence	Low-middl	Both	50-69 yea	HIV/AIDS	Rate	1990	106.036	137.917	83.5696
Prevalence	Low-middl	Both	50-69 yea	HIV/AIDS	Number	1991	161302	206979	128298
Prevalence	Low-middl	Both	50-69 yea	HIV/AIDS	Rate	1991	142.65	183.045	113.462
Prevalence	Low-middl	Both	50-69 yea	HIV/AIDS	Number	1992	211251	267593	168775
Prevalence	Low-middl	Both	50-69 yea	HIV/AIDS	Rate	1992	182.844	231.611	146.08
Prevalence	Low-middl	Both	50-69 yea	HIV/AIDS	Number	1993	263784	325598	211943
Prevalence	Low-middl	Both	50-69 yea	HIV/AIDS	Rate	1993	223.567	275.957	179.63
Prevalence	Low-middl	Both	50-69 yea	HIV/AIDS	Number	1994	316607	385382	257474
Prevalence	Low-middl	Both	50-69 yea	HIV/AIDS	Rate	1994	262.885	319.99	213.785
Prevalence	Low-middl	Both	50-69 yea	HIV/AIDS	Number	1995	367163	437351	305393
Prevalence	Low-middl	Both	50-69 yea	HIV/AIDS	Rate	1995	298.878	356.012	248.595
Prevalence	Low-middl	Both	50-69 yea	HIV/AIDS	Number	1996	414277	483674	349409
Prevalence	Low-middl	Both	50-69 yea	HIV/AIDS	Rate	1996	330.529	385.897	278.775
Prevalence	Low-middl	Both	50-69 yea	HIV/AIDS	Number	1997	456196	526075	393297
Prevalence	Low-middl	Both	50-69 yea	HIV/AIDS	Rate	1997	356.477	411.081	307.326
Prevalence	Low-middl	Both	50-69 yea	HIV/AIDS	Number	1998	493866	563780	428995
Prevalence	Low-middl	Both	50-69 yea	HIV/AIDS	Rate	1998	378.551	432.14	328.827
Prevalence	Low-middl	Both	50-69 yea	HIV/AIDS	Number	1999	525508	600876	457484
Prevalence	Low-middl	Both	50-69 yea	HIV/AIDS	Rate	1999	394.863	451.493	343.75
Prevalence	Low-middl	Both	50-69 yea	HIV/AIDS	Number	2000	549371	629524	478057
Prevalence	Low-middl	Both	50-69 yea	HIV/AIDS	Rate	2000	406.089	465.337	353.374
Prevalence	Low-middl	Both	50-69 yea	HIV/AIDS	Number	2001	578332	666005	502199
Prevalence	Low-middl	Both	50-69 yea	HIV/AIDS	Rate	2001	416.351	479.469	361.542
Prevalence	Low-middl	Both	50-69 yea	HIV/AIDS	Number	2002	602696	704821	517154
Prevalence	Low-middl	Both	50-69 yea	HIV/AIDS	Rate	2002	422.521	494.116	362.552
Prevalence	Low-middl	Both	50-69 yea	HIV/AIDS	Number	2003	623279	737469	526312
Prevalence	Low-middl	Both	50-69 yea	HIV/AIDS	Rate	2003	425.218	503.122	359.065
Prevalence	Low-middl	Both	50-69 yea	HIV/AIDS	Number	2004	642011	774891	535417
Prevalence	Low-middl	Both	50-69 yea	HIV/AIDS	Rate	2004	425.946	514.106	355.225
Prevalence	Low-middl	Both	50-69 yea	HIV/AIDS	Number	2005	662396	809214	547925
Prevalence	Low-middl	Both	50-69 yea	HIV/AIDS	Rate	2005	427.18	521.863	353.358
Prevalence	Low-middl	Both	50-69 yea	HIV/AIDS	Number	2006	686806	841617	564518
Prevalence	Low-middl	Both	50-69 yea	HIV/AIDS	Rate	2006	430.673	527.75	353.99
Prevalence	Low-middl	Both	50-69 yea	HIV/AIDS	Number	2007	718757	878467	594118
Prevalence	Low-middl	Both	50-69 yea	HIV/AIDS	Rate	2007	438.149	535.506	362.17
Prevalence	Low-middl	Both	50-69 yea	HIV/AIDS	Number	2008	759062	918558	633256
Prevalence	Low-middl	Both	50-69 yea	HIV/AIDS	Rate	2008	449.847	544.37	375.29
Prevalence	Low-middl	Both	50-69 yea	HIV/AIDS	Number	2009	808096	968257	679789
Prevalence	Low-middl	Both	50-69 yea	HIV/AIDS	Rate	2009	465.263	557.477	391.39
Prevalence	Low-middl	Both	50-69 yea	HIV/AIDS	Number	2010	865896	1025180	736811
Prevalence	Low-middl	Both	50-69 yea	HIV/AIDS	Rate	2010	483.986	573.016	411.835
Prevalence	Low-middl	Both	50-69 yea	HIV/AIDS	Number	2011	931949	1089611	802360
Prevalence	Low-middl	Both	50-69 yea	HIV/AIDS	Rate	2011	505.144	590.601	434.902
Prevalence	Low-middl	Both	50-69 yea	HIV/AIDS	Number	2012	1005805	1161776	873960
Prevalence	Low-middl	Both	50-69 yea	HIV/AIDS	Rate	2012	528.121	610.017	458.893
Prevalence	Low-middl	Both	50-69 yea	HIV/AIDS	Number	2013	1086358	1240020	952539
Prevalence	Low-middl	Both	50-69 yea	HIV/AIDS	Rate	2013	551.914	629.981	483.929
Prevalence	Low-middl	Both	50-69 yea	HIV/AIDS	Number	2014	1172511	1323237	1039289
Prevalence	Low-middl	Both	50-69 yea	HIV/AIDS	Rate	2014	575.938	649.975	510.499
Prevalence	Low-middl	Both	50-69 yea	HIV/AIDS	Number	2015	1263901	1410508	1133201
Prevalence	Low-middl	Both	50-69 yea	HIV/AIDS	Rate	2015	600.417	670.063	538.328
Prevalence	Low-middl	Both	50-69 yea	HIV/AIDS	Number	2016	1360632	1501412	1230835
Prevalence	Low-middl	Both	50-69 yea	HIV/AIDS	Rate	2016	625.762	690.508	566.068
Prevalence	Low-middl	Both	50-69 yea	HIV/AIDS	Number	2017	1462749	1600481	1336701
Prevalence	Low-middl	Both	50-69 yea	HIV/AIDS	Rate	2017	651.92	713.304	595.743
Prevalence	Low-middl	Both	50-69 yea	HIV/AIDS	Number	2018	1569735	1704663	1445791
Prevalence	Low-middl	Both	50-69 yea	HIV/AIDS	Rate	2018	677.947	736.221	624.417
Prevalence	Low-middl	Both	50-69 yea	HIV/AIDS	Number	2019	1680710	1820109	1555993
Prevalence	Low-middl	Both	50-69 yea	HIV/AIDS	Rate	2019	703.838	762.215	651.61
Prevalence	Middle SDI	Both	50-69 yea	HIV/AIDS	Number	1990	46337.8	54188.1	39628.6
Prevalence	Middle SDI	Both	50-69 yea	HIV/AIDS	Rate	1990	24.6779	28.8587	21.1048
Prevalence	Middle SDI	Both	50-69 yea	HIV/AIDS	Number	1991	65811	76035.1	57213.8
Prevalence	Middle SDI	Both	50-69 yea	HIV/AIDS	Rate	1991	34.2519	39.5732	29.7774
Prevalence	Middle SDI	Both	50-69 yea	HIV/AIDS	Number	1992	89192.9	102002	78189.4
Prevalence	Middle SDI	Both	50-69 yea	HIV/AIDS	Rate	1992	45.4277	51.9515	39.8234
Prevalence	Middle SDI	Both	50-69 yea	HIV/AIDS	Number	1993	118388	135142	104002
Prevalence	Middle SDI	Both	50-69 yea	HIV/AIDS	Rate	1993	59.0664	67.4256	51.8892
Prevalence	Middle SDI	Both	50-69 yea	HIV/AIDS	Number	1994	154217	174792	134924
Prevalence	Middle SDI	Both	50-69 yea	HIV/AIDS	Rate	1994	75.4394	85.5047	66.0016
Prevalence	Middle SDI	Both	50-69 yea	HIV/AIDS	Number	1995	195828	221124	172262
Prevalence	Middle SDI	Both	50-69 yea	HIV/AIDS	Rate	1995	93.9047	106.035	82.6042
Prevalence	Middle SDI	Both	50-69 yea	HIV/AIDS	Number	1996	245641	278904	216781
Prevalence	Middle SDI	Both	50-69 yea	HIV/AIDS	Rate	1996	115.155	130.748	101.626
Prevalence	Middle SDI	Both	50-69 yea	HIV/AIDS	Number	1997	302020	342347	267522
Prevalence	Middle SDI	Both	50-69 yea	HIV/AIDS	Rate	1997	138.29	156.755	122.494
Prevalence	Middle SDI	Both	50-69 yea	HIV/AIDS	Number	1998	361949	409243	319482
Prevalence	Middle SDI	Both	50-69 yea	HIV/AIDS	Rate	1998	161.597	182.712	142.637
Prevalence	Middle SDI	Both	50-69 yea	HIV/AIDS	Number	1999	422852	480305	371657
Prevalence	Middle SDI	Both	50-69 yea	HIV/AIDS	Rate	1999	183.063	207.936	160.899
Prevalence	Middle SDI	Both	50-69 yea	HIV/AIDS	Number	2000	483650	549934	423250
Prevalence	Middle SDI	Both	50-69 yea	HIV/AIDS	Rate	2000	204.07	232.038	178.585
Prevalence	Middle SDI	Both	50-69 yea	HIV/AIDS	Number	2001	547016	625241	474194
Prevalence	Middle SDI	Both	50-69 yea	HIV/AIDS	Rate	2001	223.352	255.292	193.618
Prevalence	Middle SDI	Both	50-69 yea	HIV/AIDS	Number	2002	608668	704608	521825
Prevalence	Middle SDI	Both	50-69 yea	HIV/AIDS	Rate	2002	240.346	278.23	206.054
Prevalence	Middle SDI	Both	50-69 yea	HIV/AIDS	Number	2003	669978	783882	570981
Prevalence	Middle SDI	Both	50-69 yea	HIV/AIDS	Rate	2003	255.314	298.721	217.589
Prevalence	Middle SDI	Both	50-69 yea	HIV/AIDS	Number	2004	731920	863157	616485
Prevalence	Middle SDI	Both	50-69 yea	HIV/AIDS	Rate	2004	268.804	317.002	226.41
Prevalence	Middle SDI	Both	50-69 yea	HIV/AIDS	Number	2005	795856	943324	665729
Prevalence	Middle SDI	Both	50-69 yea	HIV/AIDS	Rate	2005	281.625	333.808	235.578
Prevalence	Middle SDI	Both	50-69 yea	HIV/AIDS	Number	2006	866146	1022068	722162
Prevalence	Middle SDI	Both	50-69 yea	HIV/AIDS	Rate	2006	295.854	349.113	246.673
Prevalence	Middle SDI	Both	50-69 yea	HIV/AIDS	Number	2007	943944	1106360	791837
Prevalence	Middle SDI	Both	50-69 yea	HIV/AIDS	Rate	2007	311.99	365.672	261.716
Prevalence	Middle SDI	Both	50-69 yea	HIV/AIDS	Number	2008	1027691	1190392	873306
Prevalence	Middle SDI	Both	50-69 yea	HIV/AIDS	Rate	2008	329.629	381.815	280.11
Prevalence	Middle SDI	Both	50-69 yea	HIV/AIDS	Number	2009	1118560	1281532	968366
Prevalence	Middle SDI	Both	50-69 yea	HIV/AIDS	Rate	2009	348.453	399.222	301.665
Prevalence	Middle SDI	Both	50-69 yea	HIV/AIDS	Number	2010	1217634	1380063	1062475
Prevalence	Middle SDI	Both	50-69 yea	HIV/AIDS	Rate	2010	368.189	417.305	321.272
Prevalence	Middle SDI	Both	50-69 yea	HIV/AIDS	Number	2011	1325420	1493512	1161612
Prevalence	Middle SDI	Both	50-69 yea	HIV/AIDS	Rate	2011	388.167	437.394	340.193
Prevalence	Middle SDI	Both	50-69 yea	HIV/AIDS	Number	2012	1443574	1613416	1275658
Prevalence	Middle SDI	Both	50-69 yea	HIV/AIDS	Rate	2012	408.17	456.193	360.692
Prevalence	Middle SDI	Both	50-69 yea	HIV/AIDS	Number	2013	1572434	1740649	1400390
Prevalence	Middle SDI	Both	50-69 yea	HIV/AIDS	Rate	2013	427.695	473.449	380.9
Prevalence	Middle SDI	Both	50-69 yea	HIV/AIDS	Number	2014	1709896	1884377	1537700
Prevalence	Middle SDI	Both	50-69 yea	HIV/AIDS	Rate	2014	446.59	492.16	401.616
Prevalence	Middle SDI	Both	50-69 yea	HIV/AIDS	Number	2015	1853749	2032654	1681521
Prevalence	Middle SDI	Both	50-69 yea	HIV/AIDS	Rate	2015	465.069	509.953	421.86
Prevalence	Middle SDI	Both	50-69 yea	HIV/AIDS	Number	2016	1999636	2179983	1825348
Prevalence	Middle SDI	Both	50-69 yea	HIV/AIDS	Rate	2016	483.032	526.596	440.931
Prevalence	Middle SDI	Both	50-69 yea	HIV/AIDS	Number	2017	2149517	2335260	1973055
Prevalence	Middle SDI	Both	50-69 yea	HIV/AIDS	Rate	2017	500.997	544.289	459.869
Prevalence	Middle SDI	Both	50-69 yea	HIV/AIDS	Number	2018	2307119	2498435	2132268
Prevalence	Middle SDI	Both	50-69 yea	HIV/AIDS	Rate	2018	520.076	563.202	480.66
Prevalence	Middle SDI	Both	50-69 yea	HIV/AIDS	Number	2019	2472400	2666665	2300765
Prevalence	Middle SDI	Both	50-69 yea	HIV/AIDS	Rate	2019	541.034	583.545	503.475
Prevalence	High SDI	Both	50-69 yea	Sexually tra	Number	1990	3.6E+07	4.1E+07	3.1E+07
Prevalence	High SDI	Both	50-69 yea	Sexually tra	Rate	1990	23309.8	26590.6	20377.6
Prevalence	High SDI	Both	50-69 yea	Sexually tra	Number	1991	3.6E+07	4.1E+07	3.1E+07
Prevalence	High SDI	Both	50-69 yea	Sexually tra	Rate	1991	23094.9	26292.5	20237.2
Prevalence	High SDI	Both	50-69 yea	Sexually tra	Number	1992	3.6E+07	4.1E+07	3.2E+07
Prevalence	High SDI	Both	50-69 yea	Sexually tra	Rate	1992	22898.3	25969	20162.6
Prevalence	High SDI	Both	50-69 yea	Sexually tra	Number	1993	3.6E+07	4.1E+07	3.2E+07
Prevalence	High SDI	Both	50-69 yea	Sexually tra	Rate	1993	22750.7	25723.6	20078.6
Prevalence	High SDI	Both	50-69 yea	Sexually tra	Number	1994	3.6E+07	4.1E+07	3.2E+07
Prevalence	High SDI	Both	50-69 yea	Sexually tra	Rate	1994	22641	25570.9	20007.4
Prevalence	High SDI	Both	50-69 yea	Sexually tra	Number	1995	3.6E+07	4.1E+07	3.2E+07
Prevalence	High SDI	Both	50-69 yea	Sexually tra	Rate	1995	22599.9	25519.9	19991.6
Prevalence	High SDI	Both	50-69 yea	Sexually tra	Number	1996	3.7E+07	4.1E+07	3.2E+07
Prevalence	High SDI	Both	50-69 yea	Sexually tra	Rate	1996	22578.6	25483.4	19959.4
Prevalence	High SDI	Both	50-69 yea	Sexually tra	Number	1997	3.8E+07	4.2E+07	3.3E+07
Prevalence	High SDI	Both	50-69 yea	Sexually tra	Rate	1997	22557.5	25463	19955.7
Prevalence	High SDI	Both	50-69 yea	Sexually tra	Number	1998	3.8E+07	4.3E+07	3.4E+07
Prevalence	High SDI	Both	50-69 yea	Sexually tra	Rate	1998	22530.7	25417.8	19918.7
Prevalence	High SDI	Both	50-69 yea	Sexually tra	Number	1999	3.9E+07	4.4E+07	3.5E+07
Prevalence	High SDI	Both	50-69 yea	Sexually tra	Rate	1999	22516.7	25397.8	19893.9
Prevalence	High SDI	Both	50-69 yea	Sexually tra	Number	2000	4E+07	4.5E+07	3.6E+07
Prevalence	High SDI	Both	50-69 yea	Sexually tra	Rate	2000	22554.8	25470.1	19937.3
Prevalence	High SDI	Both	50-69 yea	Sexually tra	Number	2001	4.1E+07	4.7E+07	3.6E+07
Prevalence	High SDI	Both	50-69 yea	Sexually tra	Rate	2001	22652.7	25589.1	20013.1
Prevalence	High SDI	Both	50-69 yea	Sexually tra	Number	2002	4.2E+07	4.8E+07	3.7E+07
Prevalence	High SDI	Both	50-69 yea	Sexually tra	Rate	2002	22782.9	25792.1	20085.8
Prevalence	High SDI	Both	50-69 yea	Sexually tra	Number	2003	4.3E+07	4.9E+07	3.8E+07
Prevalence	High SDI	Both	50-69 yea	Sexually tra	Rate	2003	22922.9	26046.9	20102.9
Prevalence	High SDI	Both	50-69 yea	Sexually tra	Number	2004	[illegible]	[illegible]	[illegible]
Prevalence	High SDI	Both	50-69 yea	Sexually tra	Rate	2004	23050.7	26264.7	20165.9
Prevalence	High SDI	Both	50-69 yea	Sexually tra	Number	2005	4.6E+07	5.2E+07	4E+07
Prevalence	High SDI	Both	50-69 yea	Sexually tra	Rate	2005	23146.1	26428.7	20251.4
Prevalence	High SDI	Both	50-69 yea	Sexually tra	Number	2006	4.7E+07	5.3E+07	4.1E+07
Prevalence	High SDI	Both	50-69 yea	Sexually tra	Rate	2006	23139.5	26406.2	20219.2
Prevalence	High SDI	Both	50-69 yea	Sexually tra	Number	2007	4.8E+07	5.4E+07	4.1E+07
Prevalence	High SDI	Both	50-69 yea	Sexually tra	Rate	2007	23011.3	26283.1	20092
Prevalence	High SDI	Both	50-69 yea	Sexually tra	Number	2008	4.8E+07	5.5E+07	4.2E+07
Prevalence	High SDI	Both	50-69 yea	Sexually tra	Rate	2008	22826.6	26114.2	19896.4
Prevalence	High SDI	Both	50-69 yea	Sexually tra	Number	2009	4.9E+07	5.6E+07	4.2E+07
Prevalence	High SDI	Both	50-69 yea	Sexually tra	Rate	2009	22648.4	25945.7	19724.4
Prevalence	High SDI	Both	50-69 yea	Sexually tra	Number	2010	5E+07	5.7E+07	4.3E+07
Prevalence	High SDI	Both	50-69 yea	Sexually tra	Rate	2010	22568	25870.3	19667.9
Prevalence	High SDI	Both	50-69 yea	Sexually tra	Number	2011	5E+07	5.8E+07	4.4E+07
Prevalence	High SDI	Both	50-69 yea	Sexually tra	Rate	2011	22516.4	25748.6	19648.1
Prevalence	High SDI	Both	50-69 yea	Sexually tra	Number	2012	5.1E+07	5.8E+07	4.5E+07
Prevalence	High SDI	Both	50-69 yea	Sexually tra	Rate	2012	22398.7	25548.8	19615
Prevalence	High SDI	Both	50-69 yea	Sexually tra	Number	2013	5.2E+07	5.9E+07	4.6E+07
Prevalence	High SDI	Both	50-69 yea	Sexually tra	Rate	2013	22260.4	25371	19539.8
Prevalence	High SDI	Both	50-69 yea	Sexually tra	Number	2014	5.3E+07	6E+07	4.6E+07
Prevalence	High SDI	Both	50-69 yea	Sexually tra	Rate	2014	22153.2	25228.7	19472.2
Prevalence	High SDI	Both	50-69 yea	Sexually tra	Number	2015	5.3E+07	6.1E+07	4.7E+07
Prevalence	High SDI	Both	50-69 yea	Sexually tra	Rate	2015	22099.3	25172.5	19408.2
Prevalence	High SDI	Both	50-69 yea	Sexually tra	Number	2016	5.5E+07	6.3E+07	4.8E+07
Prevalence	High SDI	Both	50-69 yea	Sexually tra	Rate	2016	22157.1	25413.7	19341.9
Prevalence	High SDI	Both	50-69 yea	Sexually tra	Number	2017	5.5E+07	6.4E+07	4.8E+07
Prevalence	High SDI	Both	50-69 yea	Sexually tra	Rate	2017	22223.3	25796.3	19198.6
Prevalence	High SDI	Both	50-69 yea	Sexually tra	Number	2018	5.6E+07	6.4E+07	4.8E+07
Prevalence	High SDI	Both	50-69 yea	Sexually tra	Rate	2018	22227.8	25773.5	19220.8
Prevalence	High SDI	Both	50-69 yea	Sexually tra	Number	2019	5.6E+07	6.5E+07	4.8E+07
Prevalence	High SDI	Both	50-69 yea	Sexually tra	Rate	2019	22235.7	25773.4	19221.6
Prevalence	High SDI	Both	50-69 yea	HIV/AIDS	Number	1990	83853.1	118359	57067
Prevalence	High SDI	Both	50-69 yea	HIV/AIDS	Rate	1990	54.6607	77.154	37.1998
Prevalence	High SDI	Both	50-69 yea	HIV/AIDS	Number	1991	93082.1	131710	62021.5
Prevalence	High SDI	Both	50-69 yea	HIV/AIDS	Rate	1991	60.0219	84.93	39.9931
Prevalence	High SDI	Both	50-69 yea	HIV/AIDS	Number	1992	102065	146369	66551.1
Prevalence	High SDI	Both	50-69 yea	HIV/AIDS	Rate	1992	65.1923	93.4904	42.5083
Prevalence	High SDI	Both	50-69 yea	HIV/AIDS	Number	1993	111979	163884	71502.2
Prevalence	High SDI	Both	50-69 yea	HIV/AIDS	Rate	1993	70.6852	103.449	45.1348
Prevalence	High SDI	Both	50-69 yea	HIV/AIDS	Number	1994	121336	180382	75528.2
Prevalence	High SDI	Both	50-69 yea	HIV/AIDS	Rate	1994	75.6977	112.535	47.1198
Prevalence	High SDI	Both	50-69 yea	HIV/AIDS	Number	1995	130141	195841	79163.2
Prevalence	High SDI	Both	50-69 yea	HIV/AIDS	Rate	1995	80.5896	121.274	49.0215
Prevalence	High SDI	Both	50-69 yea	HIV/AIDS	Number	1996	140613	212853	83799.3
Prevalence	High SDI	Both	50-69 yea	HIV/AIDS	Rate	1996	86.4046	130.795	51.4933
Prevalence	High SDI	Both	50-69 yea	HIV/AIDS	Number	1997	158496	240490	93258.7
Prevalence	High SDI	Both	50-69 yea	HIV/AIDS	Rate	1997	95.2269	144.49	56.0315
Prevalence	High SDI	Both	50-69 yea	HIV/AIDS	Number	1998	179863	273353	104321
Prevalence	High SDI	Both	50-69 yea	HIV/AIDS	Rate	1998	105.574	160.45	61.2335
Prevalence	High SDI	Both	50-69 yea	HIV/AIDS	Number	1999	203299	308130	116850
Prevalence	High SDI	Both	50-69 yea	HIV/AIDS	Rate	1999	116.634	176.776	67.0372
Prevalence	High SDI	Both	50-69 yea	HIV/AIDS	Number	2000	231297	349071	131879
Prevalence	High SDI	Both	50-69 yea	HIV/AIDS	Rate	2000	129.791	195.88	74.0034
Prevalence	High SDI	Both	50-69 yea	HIV/AIDS	Number	2001	263382	396172	149622
Prevalence	High SDI	Both	50-69 yea	HIV/AIDS	Rate	2001	144.815	217.826	82.2666
Prevalence	High SDI	Both	50-69 yea	HIV/AIDS	Number	2002	297976	446033	169449
Prevalence	High SDI	Both	50-69 yea	HIV/AIDS	Rate	2002	160.378	240.066	91.2016
Prevalence	High SDI	Both	50-69 yea	HIV/AIDS	Number	2003	335815	500515	191878
Prevalence	High SDI	Both	50-69 yea	HIV/AIDS	Rate	2003	176.972	263.767	101.118
Prevalence	High SDI	Both	50-69 yea	HIV/AIDS	Number	2004	377526	560824	214006
Prevalence	High SDI	Both	50-69 yea	HIV/AIDS	Rate	2004	194.763	289.325	110.404
Prevalence	High SDI	Both	50-69 yea	HIV/AIDS	Number	2005	422438	627161	240975
Prevalence	High SDI	Both	50-69 yea	HIV/AIDS	Rate	2005	213.26	316.61	121.652
Prevalence	High SDI	Both	50-69 yea	HIV/AIDS	Number	2006	469741	695034	268157
Prevalence	High SDI	Both	50-69 yea	HIV/AIDS	Rate	2006	232.262	343.657	132.59
Prevalence	High SDI	Both	50-69 yea	HIV/AIDS	Number	2007	517966	767683	292900
Prevalence	High SDI	Both	50-69 yea	HIV/AIDS	Rate	2007	250.862	371.804	141.857
Prevalence	High SDI	Both	50-69 yea	HIV/AIDS	Number	2008	567609	844564	318769
Prevalence	High SDI	Both	50-69 yea	HIV/AIDS	Rate	2008	269.215	400.574	151.191
Prevalence	High SDI	Both	50-69 yea	HIV/AIDS	Number	2009	618543	921047	344498
Prevalence	High SDI	Both	50-69 yea	HIV/AIDS	Rate	2009	287.326	427.846	160.027
Prevalence	High SDI	Both	50-69 yea	HIV/AIDS	Number	2010	672148	996109	371916
Prevalence	High SDI	Both	50-69 yea	HIV/AIDS	Rate	2010	305.905	453.345	169.265
Prevalence	High SDI	Both	50-69 yea	HIV/AIDS	Number	2011	726397	1070165	399972
Prevalence	High SDI	Both	50-69 yea	HIV/AIDS	Rate	2011	324.131	477.526	178.474
Prevalence	High SDI	Both	50-69 yea	HIV/AIDS	Number	2012	778676	1149303	428063
Prevalence	High SDI	Both	50-69 yea	HIV/AIDS	Rate	2012	340.676	502.827	187.28
Prevalence	High SDI	Both	50-69 yea	HIV/AIDS	Number	2013	829924	1228439	457575
Prevalence	High SDI	Both	50-69 yea	HIV/AIDS	Rate	2013	356.309	527.403	196.45
Prevalence	High SDI	Both	50-69 yea	HIV/AIDS	Number	2014	881637	1304163	487689
Prevalence	High SDI	Both	50-69 yea	HIV/AIDS	Rate	2014	371.581	549.661	205.545
Prevalence	High SDI	Both	50-69 yea	HIV/AIDS	Number	2015	930910	1367376	516989
Prevalence	High SDI	Both	50-69 yea	HIV/AIDS	Rate	2015	384.574	564.885	213.577
Prevalence	High SDI	Both	50-69 yea	HIV/AIDS	Number	2016	975716	1432480	544651
Prevalence	High SDI	Both	50-69 yea	HIV/AIDS	Rate	2016	396.45	582.041	221.301
Prevalence	High SDI	Both	50-69 yea	HIV/AIDS	Number	2017	1011041	1485755	565694
Prevalence	High SDI	Both	50-69 yea	HIV/AIDS	Rate	2017	407.387	598.668	227.94
Prevalence	High SDI	Both	50-69 yea	HIV/AIDS	Number	2018	1041931	1523767	585983
Prevalence	High SDI	Both	50-69 yea	HIV/AIDS	Rate	2018	416.814	609.568	234.417
Prevalence	High SDI	Both	50-69 yea	HIV/AIDS	Number	2019	1071599	1566747	602259
Prevalence	High SDI	Both	50-69 yea	HIV/AIDS	Rate	2019	425.461	622.052	239.117
Prevalence	High-midc	Both	50-69 yea	Sexually tra	Number	1990	4.2E+07	4.8E+07	3.7E+07
Prevalence	High-midc	Both	50-69 yea	Sexually tra	Rate	1990	22705.3	26043.4	19776.4
Prevalence	High-midc	Both	50-69 yea	Sexually tra	Number	1991	4.3E+07	5E+07	3.8E+07
Prevalence	High-midc	Both	50-69 yea	Sexually tra	Rate	1991	22993	26335.4	20039.8
Prevalence	High-midc	Both	50-69 yea	Sexually tra	Number	1992	4.4E+07	5.1E+07	3.9E+07
Prevalence	High-midc	Both	50-69 yea	Sexually tra	Rate	1992	23220	26586.8	20225.7
Prevalence	High-midc	Both	50-69 yea	Sexually tra	Number	1993	4.5E+07	5.1E+07	3.9E+07
Prevalence	High-midc	Both	50-69 yea	Sexually tra	Rate	1993	23368.2	26758.2	20345
Prevalence	High-midc	Both	50-69 yea	Sexually tra	Number	1994	4.5E+07	5.1E+07	3.9E+07
Prevalence	High-midc	Both	50-69 yea	Sexually tra	Rate	1994	23451.9	26823.6	20457.8
Prevalence	High-midc	Both	50-69 yea	Sexually tra	Number	1995	4.5E+07	5.1E+07	3.9E+07
Prevalence	High-midc	Both	50-69 yea	Sexually tra	Rate	1995	23484.6	26849.6	20457.9
Prevalence	High-midc	Both	50-69 yea	Sexually tra	Number	1996	4.5E+07	5.2E+07	3.9E+07
Prevalence	High-midc	Both	50-69 yea	Sexually tra	Rate	1996	23404.5	26768.7	20413.2
Prevalence	High-midc	Both	50-69 yea	Sexually tra	Number	1997	4.5E+07	5.2E+07	4E+07
Prevalence	High-midc	Both	50-69 yea	Sexually tra	Rate	1997	23189.7	26505.9	20261.3
Prevalence	High-midc	Both	50-69 yea	Sexually tra	Number	1998	4.6E+07	5.2E+07	4E+07
Prevalence	High-midc	Both	50-69 yea	Sexually tra	Rate	1998	22935.7	26206.2	20062
Prevalence	High-midc	Both	50-69 yea	Sexually tra	Number	1999	4.6E+07	5.2E+07	4E+07
Prevalence	High-midc	Both	50-69 yea	Sexually tra	Rate	1999	22737.5	25986.5	19909.4
Prevalence	High-midc	Both	50-69 yea	Sexually tra	Number	2000	4.7E+07	5.3E+07	4.1E+07
Prevalence	High-midc	Both	50-69 yea	Sexually tra	Rate	2000	22687.3	25913.6	19859.7
Prevalence	High-midc	Both	50-69 yea	Sexually tra	Number	2001	4.8E+07	5.5E+07	4.2E+07
Prevalence	High-midc	Both	50-69 yea	Sexually tra	Rate	2001	22743.8	25964.6	19940
Prevalence	High-midc	Both	50-69 yea	Sexually tra	Number	2002	4.9E+07	5.6E+07	4.3E+07
Prevalence	High-midc	Both	50-69 yea	Sexually tra	Rate	2002	22806.9	26032.4	19986.4
Prevalence	High-midc	Both	50-69 yea	Sexually tra	Number	2003	5.1E+07	5.8E+07	4.4E+07
Prevalence	High-midc	Both	50-69 yea	Sexually tra	Rate	2003	22886.1	26111.7	20059.9
Prevalence	High-midc	Both	50-69 yea	Sexually tra	Number	2004	5.2E+07	6E+07	4.6E+07
Prevalence	High-midc	Both	50-69 yea	Sexually tra	Rate	2004	22955.8	26156.9	20150.5
Prevalence	High-midc	Both	50-69 yea	Sexually tra	Number	2005	5.4E+07	6.1E+07	4.7E+07
Prevalence	High-midc	Both	50-69 yea	Sexually tra	Rate	2005	23008.5	26229.2	20199.7
Prevalence	High-midc	Both	50-69 yea	Sexually tra	Number	2006	5.5E+07	6.3E+07	4.8E+07
Prevalence	High-midc	Both	50-69 yea	Sexually tra	Rate	2006	22955.7	26187.9	20115.3
Prevalence	High-midc	Both	50-69 yea	Sexually tra	Number	2007	5.6E+07	6.4E+07	4.9E+07
Prevalence	High-midc	Both	50-69 yea	Sexually tra	Rate	2007	22785.1	26042.1	19935.5
Prevalence	High-midc	Both	50-69 yea	Sexually tra	Number	2008	5.7E+07	6.5E+07	5E+07
Prevalence	High-midc	Both	50-69 yea	Sexually tra	Rate	2008	22574.6	25839.5	19678.8
Prevalence	High-midc	Both	50-69 yea	Sexually tra	Number	2009	5.8E+07	6.6E+07	5E+07
Prevalence	High-midc	Both	50-69 yea	Sexually tra	Rate	2009	22391.2	25694.7	19458
Prevalence	High-midc	Both	50-69 yea	Sexually tra	Number	2010	5.9E+07	6.8E+07	5.1E+07
Prevalence	High-midc	Both	50-69 yea	Sexually tra	Rate	2010	22316	25626.1	19372.1
Prevalence	High-midc	Both	50-69 yea	Sexually tra	Number	2011	6.1E+07	7E+07	5.3E+07
Prevalence	High-midc	Both	50-69 yea	Sexually tra	Rate	2011	22400.2	25717.8	19468.3
Prevalence	High-midc	Both	50-69 yea	Sexually tra	Number	2012	6.3E+07	7.3E+07	5.5E+07
Prevalence	High-midc	Both	50-69 yea	Sexually tra	Rate	2012	22597.3	25953.4	19619.9
Prevalence	High-midc	Both	50-69 yea	Sexually tra	Number	2013	6.6E+07	7.6E+07	5.7E+07
Prevalence	High-midc	Both	50-69 yea	Sexually tra	Rate	2013	22839.8	26245.3	19777.8
Prevalence	High-midc	Both	50-69 yea	Sexually tra	Number	2014	6.9E+07	7.9E+07	6E+07
Prevalence	High-midc	Both	50-69 yea	Sexually tra	Rate	2014	23042.8	26466.7	19986.6
Prevalence	High-midc	Both	50-69 yea	Sexually tra	Number	2015	7.1E+07	8.2E+07	6.2E+07
Prevalence	High-midc	Both	50-69 yea	Sexually tra	Rate	2015	23126.7	26589.1	20078
Prevalence	High-midc	Both	50-69 yea	Sexually tra	Number	2016	7.3E+07	8.4E+07	6.3E+07
Prevalence	High-midc	Both	50-69 yea	Sexually tra	Rate	2016	23093.2	26571.9	19996.4
Prevalence	High-midc	Both	50-69 yea	Sexually tra	Number	2017	7.5E+07	8.6E+07	6.5E+07
Prevalence	High-midc	Both	50-69 yea	Sexually tra	Rate	2017	23062.9	26567.6	19999.2
Prevalence	High-midc	Both	50-69 yea	Sexually tra	Number	2018	7.6E+07	8.8E+07	6.6E+07
Prevalence	High-midc	Both	50-69 yea	Sexually tra	Rate	2018	23067.9	26563.2	20012.9
Prevalence	High-midc	Both	50-69 yea	Sexually tra	Number	2019	7.8E+07	9E+07	6.8E+07
Prevalence	High-midc	Both	50-69 yea	Sexually tra	Rate	2019	23109.9	26542.7	20022.4
Prevalence	Low-middl	Both	50-69 yea	Sexually tra	Number	1990	2.6E+07	3E+07	2.3E+07
Prevalence	Low-middl	Both	50-69 yea	Sexually tra	Rate	1990	23549.2	26960.8	20500
Prevalence	Low-middl	Both	50-69 yea	Sexually tra	Number	1991	2.7E+07	3.1E+07	2.3E+07
Prevalence	Low-middl	Both	50-69 yea	Sexually tra	Rate	1991	23747.4	27123.3	20701.1
Prevalence	Low-middl	Both	50-69 yea	Sexually tra	Number	1992	2.8E+07	3.2E+07	2.4E+07
Prevalence	Low-middl	Both	50-69 yea	Sexually tra	Rate	1992	23919.3	27291.2	20832.8
Prevalence	Low-middl	Both	50-69 yea	Sexually tra	Number	1993	2.8E+07	3.2E+07	2.5E+07
Prevalence	Low-middl	Both	50-69 yea	Sexually tra	Rate	1993	24055.1	27425.4	20948.1
Prevalence	Low-middl	Both	50-69 yea	Sexually tra	Number	1994	2.9E+07	3.3E+07	2.5E+07
Prevalence	Low-middl	Both	50-69 yea	Sexually tra	Rate	1994	24153.6	27576.9	21052.7
Prevalence	Low-middl	Both	50-69 yea	Sexually tra	Number	1995	3E+07	3.4E+07	2.6E+07
Prevalence	Low-middl	Both	50-69 yea	Sexually tra	Rate	1995	24209.8	27620.4	21136.9
Prevalence	Low-middl	Both	50-69 yea	Sexually tra	Number	1996	3E+07	3.5E+07	2.6E+07
Prevalence	Low-middl	Both	50-69 yea	Sexually tra	Rate	1996	24242.4	27667	21122.3
Prevalence	Low-middl	Both	50-69 yea	Sexually tra	Number	1997	3.1E+07	3.5E+07	2.7E+07
Prevalence	Low-middl	Both	50-69 yea	Sexually tra	Rate	1997	24276.1	27713.3	21182.2
Prevalence	Low-middl	Both	50-69 yea	Sexually tra	Number	1998	3.2E+07	3.6E+07	2.8E+07
Prevalence	Low-middl	Both	50-69 yea	Sexually tra	Rate	1998	24317.6	27750.8	21277.1
Prevalence	Low-middl	Both	50-69 yea	Sexually tra	Number	1999	3.2E+07	3.7E+07	2.8E+07
Prevalence	Low-middl	Both	50-69 yea	Sexually tra	Rate	1999	24362.2	27807.9	21339.4
Prevalence	Low-middl	Both	50-69 yea	Sexually tra	Number	2000	3.3E+07	3.8E+07	2.9E+07
Prevalence	Low-middl	Both	50-69 yea	Sexually tra	Rate	2000	24397.2	27836.1	21351.2
Prevalence	Low-middl	Both	50-69 yea	Sexually tra	Number	2001	3.4E+07	3.9E+07	3E+07
Prevalence	Low-middl	Both	50-69 yea	Sexually tra	Rate	2001	24434.4	27906.3	21418.7
Prevalence	Low-middl	Both	50-69 yea	Sexually tra	Number	2002	3.5E+07	4E+07	3.1E+07
Prevalence	Low-middl	Both	50-69 yea	Sexually tra	Rate	2002	24458.9	27894.4	21438.5
Prevalence	Low-middl	Both	50-69 yea	Sexually tra	Number	2003	3.6E+07	4.1E+07	3.1E+07
Prevalence	Low-middl	Both	50-69 yea	Sexually tra	Rate	2003	24476.3	27920	21425
Prevalence	Low-middl	Both	50-69 yea	Sexually tra	Number	2004	3.7E+07	4.2E+07	3.2E+07
Prevalence	Low-middl	Both	50-69 yea	Sexually tra	Rate	2004	24495.1	27953.6	21396.7

Prevalence	High-midc	Both	50-69 yea	HIV/AIDS	Rate	1992	17.9388	21.7197	15.14
Prevalence	High-midc	Both	50-69 yea	HIV/AIDS	Number	1993	37341.7	45452.4	31512.7
Prevalence	High-midc	Both	50-69 yea	HIV/AIDS	Rate	1993	19.5173	23.7566	16.4707
Prevalence	High-midc	Both	50-69 yea	HIV/AIDS	Number	1994	40411.4	49240.8	34010.6
Prevalence	High-midc	Both	50-69 yea	HIV/AIDS	Rate	1994	21.1044	25.7155	17.7617
Prevalence	High-midc	Both	50-69 yea	HIV/AIDS	Number	1995	43424.2	52978.5	36331.6
Prevalence	High-midc	Both	50-69 yea	HIV/AIDS	Rate	1995	22.6429	27.6248	18.9446
Prevalence	High-midc	Both	50-69 yea	HIV/AIDS	Number	1996	46958	57085.3	38961.1
Prevalence	High-midc	Both	50-69 yea	HIV/AIDS	Rate	1996	24.3083	29.5508	20.1687
Prevalence	High-midc	Both	50-69 yea	HIV/AIDS	Number	1997	51531.6	62492.8	42679.8
Prevalence	High-midc	Both	50-69 yea	HIV/AIDS	Rate	1997	26.3166	31.9144	21.7961
Prevalence	High-midc	Both	50-69 yea	HIV/AIDS	Number	1998	56841.1	68755.7	47153.4
Prevalence	High-midc	Both	50-69 yea	HIV/AIDS	Rate	1998	28.6484	34.6534	23.7657
Prevalence	High-midc	Both	50-69 yea	HIV/AIDS	Number	1999	62930.9	76184.8	52304.3
Prevalence	High-midc	Both	50-69 yea	HIV/AIDS	Rate	1999	31.1654	37.7292	25.9028
Prevalence	High-midc	Both	50-69 yea	HIV/AIDS	Number	2000	70688.1	84825.4	59000.3
Prevalence	High-midc	Both	50-69 yea	HIV/AIDS	Rate	2000	34.4548	41.3456	28.7579
Prevalence	High-midc	Both	50-69 yea	HIV/AIDS	Number	2001	78887.6	94460.7	66245.7
Prevalence	High-midc	Both	50-69 yea	HIV/AIDS	Rate	2001	37.5614	44.9763	31.5421
Prevalence	High-midc	Both	50-69 yea	HIV/AIDS	Number	2002	87444.9	104371	73475.1
Prevalence	High-midc	Both	50-69 yea	HIV/AIDS	Rate	2002	40.5994	48.458	34.1135
Prevalence	High-midc	Both	50-69 yea	HIV/AIDS	Number	2003	96585.1	115674	81276.1
Prevalence	High-midc	Both	50-69 yea	HIV/AIDS	Rate	2003	43.6652	52.2951	36.7442
Prevalence	High-midc	Both	50-69 yea	HIV/AIDS	Number	2004	106386	127612	89734.4
Prevalence	High-midc	Both	50-69 yea	HIV/AIDS	Rate	2004	46.7474	56.0745	39.4307
Prevalence	High-midc	Both	50-69 yea	HIV/AIDS	Number	2005	116986	140404	99281.8
Prevalence	High-midc	Both	50-69 yea	HIV/AIDS	Rate	2005	49.939	59.9357	42.3813
Prevalence	High-midc	Both	50-69 yea	HIV/AIDS	Number	2006	128404	153861	109190
Prevalence	High-midc	Both	50-69 yea	HIV/AIDS	Rate	2006	53.3412	63.9164	45.3592
Prevalence	High-midc	Both	50-69 yea	HIV/AIDS	Number	2007	140862	167843	120665
Prevalence	High-midc	Both	50-69 yea	HIV/AIDS	Rate	2007	57.066	67.9966	48.8836
Prevalence	High-midc	Both	50-69 yea	HIV/AIDS	Number	2008	154648	182463	133884
Prevalence	High-midc	Both	50-69 yea	HIV/AIDS	Rate	2008	61.1919	72.1977	52.9758
Prevalence	High-midc	Both	50-69 yea	HIV/AIDS	Number	2009	169923	198771	148765
Prevalence	High-midc	Both	50-69 yea	HIV/AIDS	Rate	2009	65.6751	76.8248	57.4973
Prevalence	High-midc	Both	50-69 yea	HIV/AIDS	Number	2010	187189	216700	164882
Prevalence	High-midc	Both	50-69 yea	HIV/AIDS	Rate	2010	70.5506	81.6734	62.1435
Prevalence	High-midc	Both	50-69 yea	HIV/AIDS	Number	2011	206673	236540	183295
Prevalence	High-midc	Both	50-69 yea	HIV/AIDS	Rate	2011	75.8312	86.7897	67.2536
Prevalence	High-midc	Both	50-69 yea	HIV/AIDS	Number	2012	228806	259680	204239
Prevalence	High-midc	Both	50-69 yea	HIV/AIDS	Rate	2012	81.5286	92.5298	72.775
Prevalence	High-midc	Both	50-69 yea	HIV/AIDS	Number	2013	254167	284729	228419
Prevalence	High-midc	Both	50-69 yea	HIV/AIDS	Rate	2013	87.706	98.2521	78.8211
Prevalence	High-midc	Both	50-69 yea	HIV/AIDS	Number	2014	283057	314566	253260
Prevalence	High-midc	Both	50-69 yea	HIV/AIDS	Rate	2014	94.5708	105.098	84.6154
Prevalence	High-midc	Both	50-69 yea	HIV/AIDS	Number	2015	315485	351198	281673
Prevalence	High-midc	Both	50-69 yea	HIV/AIDS	Rate	2015	102.229	113.801	91.2726
Prevalence	High-midc	Both	50-69 yea	HIV/AIDS	Number	2016	349983	393433	310293
Prevalence	High-midc	Both	50-69 yea	HIV/AIDS	Rate	2016	110.439	124.15	97.9148
Prevalence	High-midc	Both	50-69 yea	HIV/AIDS	Number	2017	385715	437864	337547
Prevalence	High-midc	Both	50-69 yea	HIV/AIDS	Rate	2017	119.029	135.122	104.164
Prevalence	High-midc	Both	50-69 yea	HIV/AIDS	Number	2018	422707	483435	367397
Prevalence	High-midc	Both	50-69 yea	HIV/AIDS	Rate	2018	127.567	145.893	110.875
Prevalence	High-midc	Both	50-69 yea	HIV/AIDS	Number	2019	460344	530319	395756
Prevalence	High-midc	Both	50-69 yea	HIV/AIDS	Rate	2019	136.32	157.041	117.193
Incidence	Low SDI	Both	50-69 yea	Sexually tr	Number	1990	3971195	5190618	3048994
Incidence	Low SDI	Both	50-69 yea	Sexually tr	Rate	1990	9039.67	11815.4	6940.45
Incidence	Low SDI	Both	50-69 yea	Sexually tr	Number	1991	4040225	5277980	3102174
Incidence	Low SDI	Both	50-69 yea	Sexually tr	Rate	1991	9035.18	11803.2	6937.41
Incidence	Low SDI	Both	50-69 yea	Sexually tr	Number	1992	4107769	5362524	3155047
Incidence	Low SDI	Both	50-69 yea	Sexually tr	Rate	1992	9026.31	11783.5	6932.82
Incidence	Low SDI	Both	50-69 yea	Sexually tr	Number	1993	4166946	5439097	3196686
Incidence	Low SDI	Both	50-69 yea	Sexually tr	Rate	1993	9010.23	11761	6912.22
Incidence	Low SDI	Both	50-69 yea	Sexually tr	Number	1994	4237543	5529265	3249652
Incidence	Low SDI	Both	50-69 yea	Sexually tr	Rate	1994	9002.58	11746.8	6903.82
Incidence	Low SDI	Both	50-69 yea	Sexually tr	Number	1995	4310523	5622409	3310029
Incidence	Low SDI	Both	50-69 yea	Sexually tr	Rate	1995	8994.31	11731.7	6906.69
Incidence	Low SDI	Both	50-69 yea	Sexually tr	Number	1996	4387047	5716511	3367807
Incidence	Low SDI	Both	50-69 yea	Sexually tr	Rate	1996	8989.79	11714.1	6901.2
Incidence	Low SDI	Both	50-69 yea	Sexually tr	Number	1997	4459380	5802815	3425442
Incidence	Low SDI	Both	50-69 yea	Sexually tr	Rate	1997	8974.04	11677.6	6893.34
Incidence	Low SDI	Both	50-69 yea	Sexually tr	Number	1998	4529733	5875893	3476819
Incidence	Low SDI	Both	50-69 yea	Sexually tr	Rate	1998	8960.42	11623.3	6877.61
Incidence	Low SDI	Both	50-69 yea	Sexually tr	Number	1999	4591770	5943155	3525570
Incidence	Low SDI	Both	50-69 yea	Sexually tr	Rate	1999	8933.97	11563.3	6859.52
Incidence	Low SDI	Both	50-69 yea	Sexually tr	Number	2000	4640754	6009430	3567928
Incidence	Low SDI	Both	50-69 yea	Sexually tr	Rate	2000	8898.5	11522.9	6841.39
Incidence	Low SDI	Both	50-69 yea	Sexually tr	Number	2001	4787392	6205647	3676083
Incidence	Low SDI	Both	50-69 yea	Sexually tr	Rate	2001	8933.47	11580	6859.72
Incidence	Low SDI	Both	50-69 yea	Sexually tr	Number	2002	4928410	6407222	3782109
Incidence	Low SDI	Both	50-69 yea	Sexually tr	Rate	2002	8956.27	11643.7	6873.13
Incidence	Low SDI	Both	50-69 yea	Sexually tr	Number	2003	5067665	6606367	3888217
Incidence	Low SDI	Both	50-69 yea	Sexually tr	Rate	2003	8976.82	11702.5	6887.56
Incidence	Low SDI	Both	50-69 yea	Sexually tr	Number	2004	5212083	6790007	4001889
Incidence	Low SDI	Both	50-69 yea	Sexually tr	Rate	2004	9000.67	11725.6	6910.81
Incidence	Low SDI	Both	50-69 yea	Sexually tr	Number	2005	5370629	6995821	4124693
Incidence	Low SDI	Both	50-69 yea	Sexually tr	Rate	2005	9035.95	11770.3	6939.69
Incidence	Low SDI	Both	50-69 yea	Sexually tr	Number	2006	5506007	7184733	4229219
Incidence	Low SDI	Both	50-69 yea	Sexually tr	Rate	2006	9015.46	11764.2	6924.86
Incidence	Low SDI	Both	50-69 yea	Sexually tr	Number	2007	5652369	7392723	4338835
Incidence	Low SDI	Both	50-69 yea	Sexually tr	Rate	2007	8998.76	11769.5	6907.57
Incidence	Low SDI	Both	50-69 yea	Sexually tr	Number	2008	5815132	7621102	4461612
Incidence	Low SDI	Both	50-69 yea	Sexually tr	Rate	2008	8990.67	11782.8	6898.02
Incidence	Low SDI	Both	50-69 yea	Sexually tr	Number	2009	5994886	7872759	4590102
Incidence	Low SDI	Both	50-69 yea	Sexually tr	Rate	2009	8992.74	11809.7	6885.47
Incidence	Low SDI	Both	50-69 yea	Sexually tr	Number	2010	6192580	8145795	4732051
Incidence	Low SDI	Both	50-69 yea	Sexually tr	Rate	2010	9009	11850.5	6884.21
Incidence	Low SDI	Both	50-69 yea	Sexually tr	Number	2011	6385821	8390640	4884144
Incidence	Low SDI	Both	50-69 yea	Sexually tr	Rate	2011	9001.33	11827.3	6884.59
Incidence	Low SDI	Both	50-69 yea	Sexually tr	Number	2012	6589977	8646566	5050430
Incidence	Low SDI	Both	50-69 yea	Sexually tr	Rate	2012	8996.6	11804.2	6894.82
Incidence	Low SDI	Both	50-69 yea	Sexually tr	Number	2013	6806980	8937076	5226609
Incidence	Low SDI	Both	50-69 yea	Sexually tr	Rate	2013	8994.97	11809.8	6906.62
Incidence	Low SDI	Both	50-69 yea	Sexually tr	Number	2014	7036996	9212850	5412141
Incidence	Low SDI	Both	50-69 yea	Sexually tr	Rate	2014	8998.44	11780.8	6920.69
Incidence	Low SDI	Both	50-69 yea	Sexually tr	Number	2015	7282985	9515262	5598787
Incidence	Low SDI	Both	50-69 yea	Sexually tr	Rate	2015	9008.91	11770.2	6925.59
Incidence	Low SDI	Both	50-69 yea	Sexually tr	Number	2016	7545729	9900487	5779371
Incidence	Low SDI	Both	50-69 yea	Sexually tr	Rate	2016	9023.67	11839.6	6911.35
Incidence	Low SDI	Both	50-69 yea	Sexually tr	Number	2017	7826074	1E+07	5973691
Incidence	Low SDI	Both	50-69 yea	Sexually tr	Rate	2017	9043.12	11862.9	6902.67
Incidence	Low SDI	Both	50-69 yea	Sexually tr	Number	2018	8120569	1.1E+07	6195224
Incidence	Low SDI	Both	50-69 yea	Sexually tr	Rate	2018	9060.52	11916.4	6912.31
Incidence	Low SDI	Both	50-69 yea	Sexually tr	Number	2019	8434870	1.1E+07	6438287
Incidence	Low SDI	Both	50-69 yea	Sexually tr	Rate	2019	9078.78	11900.9	6929.78
Incidence	Low-middl	Both	50-69 yea	Sexually tr	Number	2001	8768779	1.1E+07	6880707
Incidence	Low-middl	Both	50-69 yea	Sexually tr	Rate	2001	6312.8	8058.35	4953.54
Incidence	Low-middl	Both	50-69 yea	Sexually tr	Number	2002	9024176	1.2E+07	7073040
Incidence	Low-middl	Both	50-69 yea	Sexually tr	Rate	2002	6326.41	8078.79	4958.57
Incidence	Low-middl	Both	50-69 yea	Sexually tr	Number	2003	9300074	1.2E+07	7296270
Incidence	Low-middl	Both	50-69 yea	Sexually tr	Rate	2003	6344.77	8104.69	4977.72
Incidence	Low-middl	Both	50-69 yea	Sexually tr	Number	2004	9589060	1.2E+07	7518363
Incidence	Low-middl	Both	50-69 yea	Sexually tr	Rate	2004	6361.91	8127.68	4988.1
Incidence	Low-middl	Both	50-69 yea	Sexually tr	Number	2005	9921406	1.3E+07	7746511
Incidence	Low-middl	Both	50-69 yea	Sexually tr	Rate	2005	6398.33	8177.83	4995.74
Incidence	Low-middl	Both	50-69 yea	Sexually tr	Number	2006	1E+07	1.3E+07	7988577
Incidence	Low-middl	Both	50-69 yea	Sexually tr	Rate	2006	6400.24	8194.42	5009.36
Incidence	Low-middl	Both	50-69 yea	Sexually tr	Number	2007	1E+07	1.3E+07	8230262
Incidence	Low-middl	Both	50-69 yea	Sexually tr	Rate	2007	6397.84	8205.17	5017.1
Incidence	Low-middl	Both	50-69 yea	Sexually tr	Number	2008	1.1E+07	1.4E+07	8449651
Incidence	Low-middl	Both	50-69 yea	Sexually tr	Rate	2008	6394.06	8212.33	5007.56
Incidence	Low-middl	Both	50-69 yea	Sexually tr	Number	2009	1.1E+07	1.4E+07	8683371
Incidence	Low-middl	Both	50-69 yea	Sexually tr	Rate	2009	6390.51	8216.21	4999.48
Incidence	Low-middl	Both	50-69 yea	Sexually tr	Number	2010	1.1E+07	1.5E+07	8963994
Incidence	Low-middl	Both	50-69 yea	Sexually tr	Rate	2010	6400.68	8230.31	5010.35
Incidence	Low-middl	Both	50-69 yea	Sexually tr	Number	2011	1.2E+07	1.5E+07	9233211
Incidence	Low-middl	Both	50-69 yea	Sexually tr	Rate	2011	6396.53	8221.23	5004.67
Incidence	Low-middl	Both	50-69 yea	Sexually tr	Number	2012	1.2E+07	1.6E+07	9521364
Incidence	Low-middl	Both	50-69 yea	Sexually tr	Rate	2012	6391.47	8207.46	4999.41
Incidence	Low-middl	Both	50-69 yea	Sexually tr	Number	2013	1.3E+07	1.6E+07	9842523
Incidence	Low-middl	Both	50-69 yea	Sexually tr	Rate	2013	6390.52	8198.78	5000.4
Incidence	Low-middl	Both	50-69 yea	Sexually tr	Number	2014	1.3E+07	1.7E+07	1E+07
Incidence	Low-middl	Both	50-69 yea	Sexually tr	Rate	2014	6393.79	8196.66	5004.14
Incidence	Low-middl	Both	50-69 yea	Sexually tr	Number	2015	1.3E+07	1.7E+07	1.1E+07
Incidence	Low-middl	Both	50-69 yea	Sexually tr	Rate	2015	6405.5	8208.66	5010.92
Incidence	Low-middl	Both	50-69 yea	Sexually tr	Number	2016	1.4E+07	1.8E+07	1.1E+07
Incidence	Low-middl	Both	50-69 yea	Sexually tr	Rate	2016	6434.89	8258.37	5026.42
Incidence	Low-middl	Both	50-69 yea	Sexually tr	Number	2017	1.5E+07	1.9E+07	1.1E+07
Incidence	Low-middl	Both	50-69 yea	Sexually tr	Rate	2017	6466.47	8305.23	5042.06
Incidence	Low-middl	Both	50-69 yea	Sexually tr	Number	2018	1.5E+07	1.9E+07	1.2E+07
Incidence	Low-middl	Both	50-69 yea	Sexually tr	Rate	2018	6485.89	8329.29	5060.05
Incidence	Low-middl	Both	50-69 yea	Sexually tr	Number	2019	1.6E+07	2E+07	1.2E+07
Incidence	Low-middl	Both	50-69 yea	Sexually tr	Rate	2019	6509.28	8362.61	5082.05
Incidence	Low-middl	Both	50-69 yea	Sexually tr	Number	1990	7150272	9142827	5608426
Incidence	Low-middl	Both	50-69 yea	Sexually tr	Rate	1990	6467.82	8270.19	5073.13
Incidence	Low-middl	Both	50-69 yea	Sexually tr	Number	1991	7290628	9317936	5729657
Incidence	Low-middl	Both	50-69 yea	Sexually tr	Rate	1991	6447.59	8240.47	5067.12
Incidence	Low-middl	Both	50-69 yea	Sexually tr	Number	1992	7422202	9478710	5830841
Incidence	Low-middl	Both	50-69 yea	Sexually tr	Rate	1992	6424.16	8204.13	5046.78
Incidence	Low-middl	Both	50-69 yea	Sexually tr	Number	1993	7551450	9631217	5928327
Incidence	Low-middl	Both	50-69 yea	Sexually tr	Rate	1993	6400.15	8162.83	5024.49
Incidence	Low-middl	Both	50-69 yea	Sexually tr	Number	1994	7677084	9787148	6022710
Incidence	Low-middl	Both	50-69 yea	Sexually tr	Rate	1994	6374.43	8126.46	5000.77
Incidence	Low-middl	Both	50-69 yea	Sexually tr	Number	1995	7799500	9934766	6114819
Incidence	Low-middl	Both	50-69 yea	Sexually tr	Rate	1995	6348.94	8087.08	4977.58
Incidence	Low-middl	Both	50-69 yea	Sexually tr	Number	1996	7932318	1E+07	6223815
Incidence	Low-middl	Both	50-69 yea	Sexually tr	Rate	1996	6328.76	8068.81	4965.64
Incidence	Low-middl	Both	50-69 yea	Sexually tr	Number	1997	8084414	1E+07	6347691
Incidence	Low-middl	Both	50-69 yea	Sexually tr	Rate	1997	6317.25	8051.59	4960.15
Incidence	Low-middl	Both	50-69 yea	Sexually tr	Number	1998	8228755	1E+07	6462074
Incidence	Low-middl	Both	50-69 yea	Sexually tr	Rate	1998	6307.38	8036.87	4953.21
Incidence	Low-middl	Both	50-69 yea	Sexually tr	Number	1999	8396240	1.1E+07	6594800
Incidence	Low-middl	Both	50-69 yea	Sexually tr	Rate	1999	6308.86	8039.98	4955.28
Incidence	Low-middl	Both	50-69 yea	Sexually tr	Number	2000	8512961	1.1E+07	6690176
Incidence	Low-middl	Both	50-69 yea	Sexually tr	Rate	2000	6292.68	8027.61	4945.3
Incidence	Low SDI	Both	50-69 yea	HIV/AIDS	Number	1990	68557.9	91099.8	49210.2
Incidence	Low SDI	Both	50-69 yea	HIV/AIDS	Rate	1990	156.059	207.371	112.018
Incidence	Low SDI	Both	50-69 yea	HIV/AIDS	Number	1991	73284.6	98008	52424.6
Incidence	Low SDI	Both	50-69 yea	HIV/AIDS	Rate	1991	163.887	219.176	117.237
Incidence	Low SDI	Both	50-69 yea	HIV/AIDS	Number	1992	75642.5	102978	53688
Incidence	Low SDI	Both	50-69 yea	HIV/AIDS	Rate	1992	166.215	226.281	117.973
Incidence	Low SDI	Both	50-69 yea	HIV/AIDS	Number	1993	75661.1	104766	53228.1
Incidence	Low SDI	Both	50-69 yea	HIV/AIDS	Rate	1993	163.603	226.536	115.096
Incidence	Low SDI	Both	50-69 yea	HIV/AIDS	Number	1994	73554.7	102853	51718.8
Incidence	Low SDI	Both	50-69 yea	HIV/AIDS	Rate	1994	156.265	218.509	109.876
Incidence	Low SDI	Both	50-69 yea	HIV/AIDS	Number	1995	70117.5	98644.9	49151
Incidence	Low SDI	Both	50-69 yea	HIV/AIDS	Rate	1995	146.307	205.832	102.558
Incidence	Low SDI	Both	50-69 yea	HIV/AIDS	Number	1996	66116	93369.5	46911
Incidence	Low SDI	Both	50-69 yea	HIV/AIDS	Rate	1996	135.483	191.33	96.1284
Incidence	Low SDI	Both	50-69 yea	HIV/AIDS	Number	1997	62102.3	87396.1	44591.4
Incidence	Low SDI	Both	50-69 yea	HIV/AIDS	Rate	1997	124.974	175.876	89.7356
Incidence	Low SDI	Both	50-69 yea	HIV/AIDS	Number	1998	58386.4	81315.6	42161.9
Incidence	Low SDI	Both	50-69 yea	HIV/AIDS	Rate	1998	115.496	160.853	83.4018
Incidence	Low SDI	Both	50-69 yea	HIV/AIDS	Number	1999	54984	76219.3	39981
Incidence	Low SDI	Both	50-69 yea	HIV/AIDS	Rate	1999	106.979	148.296	77.7889
Incidence	Low SDI	Both	50-69 yea	HIV/AIDS	Number	2000	52319	71550.9	38438.8
Incidence	Low SDI	Both	50-69 yea	HIV/AIDS	Rate	2000	100.32	137.197	73.7051
Incidence	Low SDI	Both	50-69 yea	HIV/AIDS	Number	2001	50297.2	68352.2	37366
Incidence	Low SDI	Both	50-69 yea	HIV/AIDS	Rate	2001	93.8566	127.548	69.7265
Incidence	Low SDI	Both	50-69 yea	HIV/AIDS	Number	2002	48452.8	64704.3	36276.4
Incidence	Low SDI	Both	50-69 yea	HIV/AIDS	Rate	2002	88.052	117.586	65.9242
Incidence	Low SDI	Both	50-69 yea	HIV/AIDS	Number	2003	46881.7	62043.8	35429.6
Incidence	Low SDI	Both	50-69 yea	HIV/AIDS	Rate	2003	83.0458	109.904	62.7598
Incidence	Low SDI	Both	50-69 yea	HIV/AIDS	Number	2004	45479.4	59419.7	34774.1
Incidence	Low SDI	Both	50-69 yea	HIV/AIDS	Rate	2004	78.5377	102.611	60.0509
Incidence	Low SDI	Both	50-69 yea	HIV/AIDS	Number	2005	44209.4	57073.4	34064.2
Incidence	Low SDI	Both	50-69 yea	HIV/AIDS	Rate	2005	74.3812	96.0247	57.3122
Incidence	Low SDI	Both	50-69 yea	HIV/AIDS	Number	2006	43140.9	55881.2	33566.2
Incidence	Low SDI	Both	50-69 yea	HIV/AIDS	Rate	2006	70.6383	91.4991	54.9608
Incidence	Low SDI	Both	50-69 yea	HIV/AIDS	Number	2007	42350.6	54503.1	33102.2
Incidence	Low SDI	Both	50-69 yea	HIV/AIDS	Rate	2007	67.4235	86.7707	52.6998
Incidence	Low SDI	Both	50-69 yea	HIV/AIDS	Number	2008	41719.4	53458.5	32696.6
Incidence	Low SDI	Both	50-69 yea	HIV/AIDS	Rate	2008	64.5017	82.6513	50.5516
Incidence	Low SDI	Both	50-69 yea	HIV/AIDS	Number	2009	41411.5	52797.6	32480.2
Incidence	Low SDI	Both	50-69 yea	HIV/AIDS	Rate	2009	62.1201	79.2001	48.7225
Incidence	Low SDI	Both	50-69 yea	HIV/AIDS	Number	2010	41116.1	52082	32200.7
Incidence	Low SDI	Both	50-69 yea	HIV/AIDS	Rate	2010	59.8159	75.7692	46.8458
Incidence	Low SDI	Both	50-69 yea	HIV/AIDS	Number	2011	40925	52130.6	31835.2
Incidence	Low SDI	Both	50-69 yea	HIV/AIDS	Rate	2011	57.687	73.4822	44.8743
Incidence	Low SDI	Both	50-69 yea	HIV/AIDS	Number	2012	40496.4	51458.6	31458.6
Incidence	Low SDI	Both	50-69 yea	HIV/AIDS	Rate	2012	55.2855	70.251	42.9471
Incidence	Low SDI	Both	50-69 yea	HIV/AIDS	Number	2013	39648	50242.5	30736.1
Incidence	Low SDI	Both	50-69 yea	HIV/AIDS	Rate	2013	52.3922	66.3921	40.6157
Incidence	Low SDI	Both	50-69 yea	HIV/AIDS	Number	2014	38555.8	49183.5	29820.4
Incidence	Low SDI	Both	50-69 yea	HIV/AIDS	Rate	2014	49.3026	62.8926	38.1324
Incidence	Low SDI	Both	50-69 yea	HIV/AIDS	Number	2015	37480.4	48298.6	28776.3
Incidence	Low SDI	Both	50-69 yea	HIV/AIDS	Rate	2015	46.3625	59.7444	35.5958
Incidence	Low SDI	Both	50-69 yea	HIV/AIDS	Number	2016	36305.5	47529.4	27621.7
Incidence	Low SDI	Both	50-69 yea	HIV/AIDS	Rate	2016	43.4165	56.8388	33.0319
Incidence	Low SDI	Both	50-69 yea	HIV/AIDS	Number	2017	34926	46208.3	25969
Incidence	Low SDI	Both	50-69 yea	HIV/AIDS	Rate	2017	40.3574	53.3942	30.0075
Incidence	Low SDI	Both	50-69 yea	HIV/AIDS	Number	2018	33463.8	45179.5	24366.7
Incidence	Low SDI	Both	50-69 yea	HIV/AIDS	Rate	2018	37.3372	50.409	27.1871
Incidence	Low SDI	Both	50-69 yea	HIV/AIDS	Number	2019	32694.3	44569.7	23359.8
Incidence	Low SDI	Both	50-69 yea	HIV/AIDS	Rate	2019	35.1901	47.9721	25.143
Incidence	Middle SDI	Both	50-69 yea	HIV/AIDS	Number	1990	16701.7	19094.5	14730.6
Incidence	Middle SDI	Both	50-69 yea	HIV/AIDS	Rate	1990	8.89476	10.1691	7.845
Incidence	Middle SDI	Both	50-69 yea	HIV/AIDS	Number	1991	20728	23302.1	18488.8
Incidence	Low-middl	Both	50-69 yea	HIV/AIDS	Number	1990	40010.5	53476.1	29279.7
Incidence	Low-middl	Both	50-69 yea	HIV/AIDS	Rate	1990	36.1917	48.3721	26.4852
Incidence	Low-middl	Both	50-69 yea	HIV/AIDS	Number	1991	48060.9	62846.4	35218
Incidence	Low-middl	Both	50-69 yea	HIV/AIDS	Rate	1991	42.5034	55.5793	31.1456
Incidence	Low-middl	Both	50-69 yea	HIV/AIDS	Number	1992	53828.4	69989.3	39856.4
Incidence	Low-middl	Both	50-69 yea	HIV/AIDS	Rate	1992	46.5902	60.578	34.497
Incidence	Low-middl	Both	50-69 yea	HIV/AIDS	Number	1993	57006.6	74101	42556.7
Incidence	Low-middl	Both	50-69 yea	HIV/AIDS	Rate	1993	48.3153	62.8034	36.0685
Incidence	Low-middl	Both	50-69 yea	HIV/AIDS	Number	1994	58170.2	76040.7	44283.5
Incidence	Low-middl	Both	50-69 yea	HIV/AIDS	Rate	1994	48.2998	63.138	36.7695
Incidence	Low-middl	Both	50-69 yea	HIV/AIDS	Number	1995	57502.6	75390.2	44123.5
Incidence	Low-middl	Both	50-69 yea	HIV/AIDS	Rate	1995	46.8082	61.369	35.9173
Incidence	Low-middl	Both	50-69 yea	HIV/AIDS	Number	1996	55441.9	72606.3	42780.2
Incidence	Low-middl	Both	50-69 yea	HIV/AIDS	Rate	1996	44.234	57.9286	34.132
Incidence	Low-middl	Both	50-69 yea	HIV/AIDS	Number	1997	53070.4	69371.4	41453.5
Incidence	Low-middl	Both	50-69 yea	HIV/AIDS	Rate	1997	41.4698	54.2076	32.3922
Incidence	Low-middl	Both	50-69 yea	HIV/AIDS	Number	1998	50420.7	65847.7	39666.3
Incidence	Low-middl	Both	50-69 yea	HIV/AIDS	Rate	1998	38.6478	50.4726	30.4044
Incidence	Low-middl	Both	50-69 yea	HIV/AIDS	Number	1999	48274.4	62709.5	37951.4
Incidence	Low-middl	Both	50-69 yea	HIV/AIDS	Rate	1999	36.273	47.1194	28.5164
Incidence	Low-middl	Both	50-69 yea	HIV/AIDS	Number	2000	46360.3	60001.9	36519.9
Incidence	Low-middl	Both	50-69 yea	HIV/AIDS	Rate	2000	34.269	44.3527	26.9951
Incidence	Low-middl	Both	50-69 yea	HIV/AIDS	Number	2001	44429.7	57107.3	35068.4
Incidence	Low-middl	Both	50-69 yea	HIV/AIDS	Rate	2001	31.9857	41.1126	25.2463
Incidence	Low-middl	Both	50-69 yea	HIV/AIDS	Number	2002	42779.1	54540.3	33899.9
Incidence	Low-middl	Both	50-69 yea	HIV/AIDS	Rate	2002	29.9904	38.2356	23.7655
Incidence	Low-middl	Both	50-69 yea	HIV/AIDS	Number	2003	41527.9	52636.5	32793
Incidence	Low-middl	Both	50-69 yea	HIV/AIDS	Rate	2003	28.3315	35.9101	22.3723
Incidence	Low-middl	Both	50-69 yea	HIV/AIDS	Number	2004	40619.5	51193.9	32084.1
Incidence	Low-middl	Both	50-69 yea	HIV/AIDS	Rate	2004	26.9493	33.9649	21.2864
Incidence	Low-middl	Both	50-69 yea	HIV/AIDS	Number	2005	39894.5	50097.5	31377.4
Incidence	Low-middl	Both	50-69 yea	HIV/AIDS	Rate	2005	25.728	32.308	20.2353
Incidence	Low-middl	Both	50-69 yea	HIV/AIDS	Number	2006	39397.5	49281.2	30840.2
Incidence	Low-middl	Both	50-69 yea	HIV/AIDS	Rate	2006	24.7048	30.9026	19.3389
Incidence	Low-middl	Both	50-69 yea	HIV/AIDS	Number	2007	39125	49161.1	30554.3
Incidence	Low-middl	Both	50-69 yea	HIV/AIDS	Rate	2007	23.8503	29.9682	18.6257
Incidence	Low-middl	Both	50-69 yea	HIV/AIDS	Number	2008	38919.3	49301.3	30228.8
Incidence	Low-middl	Both	50-69 yea	HIV/AIDS	Rate	2008	23.0649	29.2177	17.9147
Incidence	Low-middl	Both	50-69 yea	HIV/AIDS	Number	2009	38894.1	50119.6	29721.8
Incidence	Low-middl	Both	50-69 yea	HIV/AIDS	Rate	2009	22.3934	28.8565	17.1124
Incidence	Low-middl	Both	50-69 yea	HIV/AIDS	Number	2010	38686.6	50202.5	29017.4
Incidence	Low-middl	Both	50-69 yea	HIV/AIDS	Rate	2010	21.6236	28.0603	16.219
Incidence	Low-middl	Both	50-69 yea	HIV/AIDS	Number	2011	38774.6	50427.8	29083.6
Incidence	Low-middl	Both	50-69 yea	HIV/AIDS	Rate	2011	21.017	27.3333	15.7642
Incidence	Low-middl	Both	50-69 yea	HIV/AIDS	Number	2012	39055.7	51019.2	28848.1
Incidence	Low-middl	Both	50-69 yea	HIV/AIDS	Rate	2012	20.5071	26.7888	15.1473
Incidence	Low-middl	Both	50-69 yea	HIV/AIDS	Number	2013	39282.4	51706.4	28541.7
Incidence	Low-middl	Both	50-69 yea	HIV/AIDS	Rate	2013	19.957	26.2689	14.5004
Incidence	Low-middl	Both	50-69 yea	HIV/AIDS	Number	2014	39737.5	53028.4	28207.6
Incidence	Low-middl	Both	50-69 yea	HIV/AIDS	Rate	2014	19.5191	26.0476	13.8556
Incidence	Low-middl	Both	50-69 yea	HIV/AIDS	Number	2015	40094.6	54347.8	27830.5
Incidence	Low-middl	Both	50-69 yea	HIV/AIDS	Rate	2015	19.047	25.8179	13.2209
Incidence	Low-middl	Both	50-69 yea	HIV/AIDS	Number	2016	39656	54251	27285.1
Incidence	Low-middl	Both	50-69 yea	HIV/AIDS	Rate	2016	18.238	24.9503	12.5486
Incidence	Low-middl	Both	50-69 yea	HIV/AIDS	Number	2017	39134.8	53744.9	26885.5
Incidence	Low-middl	Both	50-69 yea	HIV/AIDS	Rate	2017	17.4416	23.9531	11.9824
Incidence	Low-middl	Both	50-69 yea	HIV/AIDS	Number	2018	38517.4	52717.7	26541.3
Incidence	Low-middl	Both	50-69 yea	HIV/AIDS	Rate	2018	16.6351	22.7681	11.4628
Incidence	Low-middl	Both	50-69 yea	HIV/AIDS	Number	2019	38307.1	52572.2	26399
Incidence	Low-middl	Both	50-69 yea	HIV/AIDS	Rate	2019	16.042	22.0159	11.0552
Incidence	High SDI	Both	50-69 yea	Sexually tr	Number	1990	7057771	9082946	5520014
Incidence	High SDI	Both	50-69 yea	Sexually tr	Rate	1990	4600.7	5920.83	3598.29
Incidence	High SDI	Both	50-69 yea	Sexually tr	Number	1991	7149279	9202612	5588871
Incidence	High SDI	Both	50-69 yea	Sexually tr	Rate	1991	4610.05	5934.09	3603.85
Incidence	High SDI	Both	50-69 yea	Sexually tr	Number	1992	7240975	9317207	5653698
Incidence	High SDI	Both	50-69 yea	Sexually tr	Rate	1992	4625.04	5951.2	3611.2
Incidence	High SDI	Both	50-69 yea	Sexually tr	Number	1993	7359882	9469163	5749833
Incidence	High SDI	Both	50-69 yea	Sexually tr	Rate	1993	4645.83	5977.29	3629.51
Incidence	High SDI	Both	50-69 yea	Sexually tr	Number	1994	7480994	9629582	5843699
Incidence	High SDI	Both	50-69 yea	Sexually tr	Rate	1994	4667.17	6007.61	3645.71
Incidence	High SDI	Both	50-69 yea	Sexually tr	Number	1995	7543977	9709636	5896546
Incidence	High SDI	Both	50-69 yea	Sexually tr	Rate	1995	4671.58	6012.65	3651.41
Incidence	High SDI	Both	50-69 yea	Sexually tr	Number	1996	7600788	9794134	5935900
Incidence	High SDI	Both	50-69 yea	Sexually tr	Rate	1996	4670.56	6018.34	3647.52
Incidence	High SDI	Both	50-69 yea	Sexually tr	Number	1997	7845651	1E+07	6108154
Incidence	High SDI	Both	50-69 yea	Sexually tr	Rate	1997	4713.8	6082.72	3669.88
Incidence	High SDI	Both	50-69 yea	Sexually tr	Number	1998	8100270	1E+07	6290741
Incidence	High SDI	Both	50-69 yea	Sexually tr	Rate	1998	4754.61	6144.99	3692.47
Incidence	High SDI	Both	50-69 yea	Sexually tr	Number	1999	8353541	1.1E+07	6473429
Incidence	High SDI	Both	50-69 yea	Sexually tr	Rate	1999	4792.47	6200.9	3713.84
Incidence	High SDI	Both	50-69 yea	Sexually tr	Number	2000	8618251	1.1E+07	6686240
Incidence	High SDI	Both	50-69 yea	Sexually tr	Rate	2000	4836.08	6251.84	3751.95
Incidence	High SDI	Both	50-69 yea	Sexually tr	Number	2001	8858714	1.1E+07	6866772
Incidence	High SDI	Both	50-69 yea	Sexually tr	Rate	2001	4870.77	6301.81	3775.54
Incidence	High SDI	Both	50-69 yea	Sexually tr	Number	2002	9049088	1.2E+07	7025076
Incidence	High SDI	Both	50-69 yea	Sexually tr	Rate	2002	4870.44	6304.49	3781.06
Incidence	High SDI	Both	50-69 yea	Sexually tr	Number	2003	9230303	1.2E+07	7164467
Incidence	High SDI	Both	50-69 yea	Sexually tr	Rate	2003	4864.29	6300.5	3775.61
Incidence	High SDI	Both	50-69 yea	Sexually tr	Number	2004	9427440	1.2E+07	7322776
Incidence	High SDI	Both	50-69 yea	Sexually tr	Rate	2004	4863.54	6310.44	3777.76
Incidence	High SDI	Both	50-69 yea	Sexually tr	Number	2005	9656248	1.3E+07	7488985
Incidence	High SDI	Both	50-69 yea	Sexually tr	Rate	2005	4874.77	6324.53	3780.67
Incidence	High SDI	Both	50-69 yea	Sexually tr	Number	2006	9888808	1.3E+07	7660956
Incidence	High SDI	Both	50-69 yea	Sexually tr	Rate	2006	4889.49	6350.59	3787.94
Incidence	High SDI	Both	50-69 yea	Sexually tr	Number	2007	1E+07	1.3E+07	7813120
Incidence	High SDI	Both	50-69 yea	Sexually tr	Rate	2007	4881.87	6335.19	3784.05
Incidence	High SDI	Both	50-69 yea	Sexually tr	Number	2008	1E+07	1.3E+07	7968758
Incidence	High SDI	Both	50-69 yea	Sexually tr	Rate	2008	4874.41	6319.89	3779.56
Incidence	High SDI	Both	50-69 yea	Sexually tr	Number	2009	1E+07	1.4E+07	8114095
Incidence	High SDI	Both	50-69 yea	Sexually tr	Rate	2009	4865.26	6304.44	3769.17
Incidence	High SDI	Both	50-69 yea	Sexually tr	Number	2010	1.1E+07	1.4E+07	8266410
Incidence	High SDI	Both	50-69 yea	Sexually tr	Rate	2010	4859.55	6292.25	3762.17
Incidence	High SDI	Both	50-69 yea	Sexually tr	Number	2011	1.1E+07	1.4E+07	8442191
Incidence	High SDI	Both	50-69 yea	Sexually tr	Rate	2011	4856.08	6284.18	3767.05
Incidence	High SDI	Both	50-69 yea	Sexually tr	Number	2012	1.1E+07	1.4E+07	8583684
Incidence	High SDI	Both	50-69 yea	Sexually tr	Rate	2012	4841.98	6260.15	3755.42
Incidence	High SDI	Both	50-69 yea	Sexually tr	Number	2013	1.1E+07	1.5E+07	8717054
Incidence	High SDI	Both	50-69 yea	Sexually tr	Rate	2013	4828.2	6237.67	3742.47
Incidence	High SDI	Both	50-69 yea	Sexually tr	Number	2014	1.1E+07	1.5E+07	8850612
Incidence	High SDI	Both	50-69 yea	Sexually tr	Rate	2014	4814.27	6215.1	3730.24
Incidence	High SDI	Both	50-69 yea	Sexually tr	Number	2015	1.2E+07	1.5E+07	9000529
Incidence	High SDI	Both	50-69 yea	Sexually tr	Rate	2015	4792.16	6181.35	3718.27
Incidence	High SDI	Both	50-69 yea	Sexually tr	Number	2016	1.2E+07	1.5E+07	9122239
Incidence	High SDI	Both	50-69 yea	Sexually tr	Rate	2016	4776.15	6169.13	3706.52
Incidence	High SDI	Both	50-69 yea	Sexually tr	Number	2017	1.2E+07	1.5E+07	9216895
Incidence	High SDI	Both	50-69 yea	Sexually tr	Rate	2017	4782.16	6167.91	3713.84
Incidence	High SDI	Both	50-69 yea	Sexually tr	Number	2018	1.2E+07	1.5E+07	9297050
Incidence	High SDI	Both	50-69 yea	Sexually tr	Rate	2018	4783.96	6154.61	3719.2
Incidence	High SDI	Both	50-69 yea	Sexually tr	Number	2019	1.2E+07	1.6E+07	9406341
Incidence	High SDI	Both	50-69 yea	Sexually tr	Rate	2019	4795.24	6175.22	3734.64
Incidence	High-midc	Both	50-69 yea	HIV/AIDS	Number	1990	3380.77	4154.24	2796.46
Incidence	High-midc	Both	50-69 yea	HIV/AIDS	Rate	1990	1.8162	2.23172	1.5023
Incidence	High-midc	Both	50-69 yea	HIV/AIDS	Number	1991	3567	4365.59	2876.36
Incidence	High-midc	Both	50-69 yea	HIV/AIDS	Rate	1991	1.88973	2.3128	1.52384
Incidence	High-midc	Both	50-69 yea	HIV/AIDS	Number	1992	3480.6	4337.27	2815.54
Incidence	High-midc	Both	50-69 yea	HIV/AIDS	Rate	1992	1.82571	2.27506	1.47686
Incidence	High-midc	Both	50-69 yea	HIV/AIDS	Number	1993	3448.03	4344.07	2782.91
Incidence	High-midc	Both	50-69 yea	HIV/AIDS	Rate	1993	1.80218	2.27051	1.45454
Incidence	High-midc	Both	50-69 yea	HIV/AIDS	Number	1994	3526.65	4419.32	2834.58
Incidence	High-midc	Both	50-69 yea	HIV/AIDS	Rate	1994	1.84175	2.30794	1.48033
Incidence	High-midc	Both	50-69 yea	HIV/AIDS	Number	1995	3707.22	4602.76	2983.33
Incidence	High-midc	Both	50-69 yea	HIV/AIDS	Rate	1995	1.93308	2.40004	1.55561
Incidence	High-midc	Both	50-69 yea	HIV/AIDS	Number	1996	4234.33	5410.22	3324.19
Incidence	High-midc	Both	50-69 yea	HIV/AIDS	Rate	1996	2.19195	2.80066	1.7208
Incidence	High-midc	Both	50-69 yea	HIV/AIDS	Number	1997	4722.73	6013.06	3656.86
Incidence	High-midc	Both	50-69 yea	HIV/AIDS	Rate	1997	2.41185	3.0708	1.86752
Incidence	High-midc	Both	50-69 yea	HIV/AIDS	Number	1998	5041.24	6463.42	3922.06
Incidence	High-midc	Both	50-69 yea	HIV/AIDS	Rate	1998	2.54083	3.25762	1.97675
Incidence	High-midc	Both	50-69 yea	HIV/AIDS	Number	1999	5414.14	6813.59	4256.68
Incidence	High-midc	Both	50-69 yea	HIV/AIDS	Rate	1999	2.68126	3.37431	2.10805
Incidence	High-midc	Both	50-69 yea	HIV/AIDS	Number	2000	5518.46	6708.07	4525.47
Incidence	High-midc	Both	50-69 yea	HIV/AIDS	Rate	2000	2.68981	3.26965	2.20581
Incidence	High-midc	Both	50-69 yea	HIV/AIDS	Number	2001	5682.35	6766.16	4760.55
Incidence	High-midc	Both	50-69 yea	HIV/AIDS	Rate	2001	2.70558	3.22162	2.26668
Incidence	High-midc	Both	50-69 yea	HIV/AIDS	Number	2002	5955.13	6992.44	5026.89
Incidence	High-midc	Both	50-69 yea	HIV/AIDS	Rate	2002	2.76488	3.24649	2.33391
Incidence	High-midc	Both	50-69 yea	HIV/AIDS	Number	2003	6231.91	7217.46	5304.95
Incidence	High-midc	Both	50-69 yea	HIV/AIDS	Rate	2003	2.81739	3.26295	2.39832
Incidence	High-midc	Both	50-69 yea	HIV/AIDS	Number	2004	6440.42	7412.34	5506.05
Incidence	High-midc	Both	50-69 yea	HIV/AIDS	Rate	2004	2.83002	3.2571	2.41944
Incidence	High-midc	Both	50-69 yea	HIV/AIDS	Number	2005	6583.63	7590.17	5641.35
Incidence	High-midc	Both	50-69 yea	HIV/AIDS	Rate	2005	2.81041	3.24009	2.40817
Incidence	High-midc	Both	50-69 yea	HIV/AIDS	Number	2006	6603.88	7729.17	5600.6
Incidence	High-midc	Both	50-69 yea	HIV/AIDS	Rate	2006	2.74337	3.21083	2.32659
Incidence	High-midc	Both	50-69 yea	HIV/AIDS	Number	2007	6725.58	7939.75	5651.35
Incidence	High-midc	Both	50-69 yea	HIV/AIDS	Rate	2007	2.72466	3.21655	2.28947
Incidence	High-midc	Both	50-69 yea	HIV/AIDS	Number	2008	6910.94	8226.38	5659.35
Incidence	High-midc	Both	50-69 yea	HIV/AIDS	Rate	2008	2.73455	3.25505	2.23932
Incidence	High-midc	Both	50-69 yea	HIV/AIDS	Number	2009	7299.73	8844.8	5768.68
Incidence	High-midc	Both	50-69 yea	HIV/AIDS	Rate	2009	2.82133	3.4185	2.22959
Incidence	High-midc	Both	50-69 yea	HIV/AIDS	Number	2010	7985.38	9860.25	5973.74
Incidence	High-midc	Both	50-69 yea	HIV/AIDS	Rate	2010	3.00966	3.71629	2.25148
Incidence	High-midc	Both	50-69 yea	HIV/AIDS	Number	2011	9112.95	11547.3	6370.3
Incidence	High-midc	Both	50-69 yea	HIV/AIDS	Rate	2011	3.34367	4.23688	2.33735
Incidence	High-midc	Both	50-69 yea	HIV/AIDS	Number	2012	10645.7	14162.3	6783.1
Incidence	High-midc	Both	50-69 yea	HIV/AIDS	Rate	2012	3.7933	5.04635	2.41697
Incidence	High-midc	Both	50-69 yea	HIV/AIDS	Number	2013	12644.4	18060.8	7112.89
Incidence	High-midc	Both	50-69 yea	HIV/AIDS	Rate	2013	4.36322	6.23231	2.45446
Incidence	High-midc	Both	50-69 yea	HIV/AIDS	Number	2014	14717.8	21740	7593
Incidence	High-midc	Both	50-69 yea	HIV/AIDS	Rate	2014	4.9173	7.26345	2.53686
Incidence	High-midc	Both	50-69 yea	HIV/AIDS	Number	2015	16539.8	24945.4	8117.32
Incidence	High-midc	Both	50-69 yea	HIV/AIDS	Rate	2015	5.35953	8.08325	2.63032
Incidence	High-midc	Both	50-69 yea	HIV/AIDS	Number	2016	17209.7	26174.4	8381.88
Incidence	High-midc	Both	50-69 yea	HIV/AIDS	Rate	2016	5.43063	8.25949	2.64495
Incidence	High-midc	Both	50-69 yea	HIV/AIDS	Number	2017	17561.4	26639.3	8605.23
Incidence	High-midc	Both	50-69 yea	HIV/AIDS	Rate	2017	5.41931	8.22067	2.65551
Incidence	High-midc	Both	50-69 yea	HIV/AIDS	Number	2018	17408.7	26227.1	8816.27
Incidence	High-midc	Both	50-69 yea	HIV/AIDS	Rate	2018	5.25369	7.91495	2.66062
Incidence	High-midc	Both	50-69 yea	HIV/AIDS	Number	2019	17325.1	25998.8	8849.47
Incidence	High-midc	Both	50-69 yea	HIV/AIDS	Rate	2019	5.1304	7.69892	2.62056
Incidence	Middle SDI	Both	50-69 yea	HIV/AIDS	Rate	1991	10.7881	12.1278	9.62266
Incidence	Middle SDI	Both	50-69 yea	HIV/AIDS	Number	1992	25182.5	28505.2	22348.4
Incidence	Middle SDI	Both	50-69 yea	HIV/AIDS	Rate	1992	12.8259	14.5183	11.3825
Incidence	Middle SDI	Both	50-69 yea	HIV/AIDS	Number	1993	31364.9	36154	27492.9
Incidence	Middle SDI	Both	50-69 yea	HIV/AIDS	Rate	1993	15.6487	18.0381	13.7169
Incidence	Middle SDI	Both	50-69 yea	HIV/AIDS	Number	1994	39516.3	45882.2	34428.9
Incidence	Middle SDI	Both	50-69 yea	HIV/AIDS	Rate	1994	19.3305	22.4446	16.8419
Incidence	Middle SDI	Both	50-69 yea	HIV/AIDS	Number	1995	47459.1	55534.2	40857
Incidence	Middle SDI	Both	50-69 yea	HIV/AIDS	Rate	1995	22.7579	26.6301	19.592
Incidence	Middle SDI	Both	50-69 yea	HIV/AIDS	Number	1996	55708.4	65544.1	47725.9
Incidence	Middle SDI	Both	50-69 yea	HIV/AIDS	Rate	1996	26.1157	30.7267	22.3736
Incidence	Middle SDI	Both	50-69 yea	HIV/AIDS	Number	1997	60917.5	72360.7	51599
Incidence	Middle SDI	Both	50-69 yea	HIV/AIDS	Rate	1997	27.8932	33.1328	23.6264
Incidence	Middle SDI	Both	50-69 yea	HIV/AIDS	Number	1998	64083.8	77685.2	53159.8
Incidence	Middle SDI	Both	50-69 yea	HIV/AIDS	Rate	1998	28.6111	34.6836	23.7339
Incidence	Middle SDI	Both	50-69 yea	HIV/AIDS	Number	1999	65940.3	81019.5	55072.7
Incidence	Middle SDI	Both	50-69 yea	HIV/AIDS	Rate	1999	28.5471	35.0753	23.8423
Incidence	Middle SDI	Both	50-69 yea	HIV/AIDS	Number	2000	67472.3	83419.9	56444.9
Incidence	Middle SDI	Both	50-69 yea	HIV/AIDS	Rate	2000	28.4691	35.198	23.8162
Incidence	Middle SDI	Both	50-69 yea	HIV/AIDS	Number	2001	69006.5	85081.7	57829.1
Incidence	Middle SDI	Both	50-69 yea	HIV/AIDS	Rate	2001	28.176	34.7397	23.6122
Incidence	Middle SDI	Both	50-69 yea	HIV/AIDS	Number	2002	71510.2	87418.6	59723.7
Incidence	Middle SDI	Both	50-69 yea	HIV/AIDS	Rate	2002	28.2374	34.5192	23.5832
Incidence	Middle SDI	Both	50-69 yea	HIV/AIDS	Number	2003	74390.7	90338	61693.5
Incidence	Middle SDI	Both	50-69 yea	HIV/AIDS	Rate	2003	28.3487	34.4259	23.5101
Incidence	Middle SDI	Both	50-69 yea	HIV/AIDS	Number	2004	76097.2	92315.4	62951.2
Incidence	Middle SDI	Both	50-69 yea	HIV/AIDS	Rate	2004	27.9474	33.9037	23.1194
Incidence	Middle SDI	Both	50-69 yea	HIV/AIDS	Number	2005	77981.5	94682.2	63733.1
Incidence	Middle SDI	Both	50-69 yea	HIV/AIDS	Rate	2005	27.5949	33.5046	22.5528
Incidence	Middle SDI	Both	50-69 yea	HIV/AIDS	Number	2006	79369.1	96489.3	65090.8
Incidence	Middle SDI	Both	50-69 yea	HIV/AIDS	Rate	2006	27.1105	32.9584	22.2334
Incidence	Middle SDI	Both	50-69 yea	HIV/AIDS	Number	2007	79455.3	97701.8	65012.7
Incidence	Middle SDI	Both	50-69 yea	HIV/AIDS	Rate	2007	26.2614	32.2922	21.4879
Incidence	Middle SDI	Both	50-69 yea	HIV/AIDS	Number	2008	79002.7	98118.1	65367.5
Incidence	Middle SDI	Both	50-69 yea	HIV/AIDS	Rate	2008	25.3399	31.4711	20.9664
Incidence	Middle SDI	Both	50-69 yea	HIV/AIDS	Number	2009	79033.2	98773.2	66050.7
Incidence	Middle SDI	Both	50-69 yea	HIV/AIDS	Rate	2009	24.6204	30.7698	20.5761
Incidence	Middle SDI	Both	50-69 yea	HIV/AIDS	Number	2010	78627.2	99529	65810
Incidence	Middle SDI	Both	50-69 yea	HIV/AIDS	Rate	2010	23.7754	30.0957	19.8997
Incidence	Middle SDI	Both	50-69 yea	HIV/AIDS	Number	2011	77837.1	99487.1	64488.1
Incidence	Middle SDI	Both	50-69 yea	HIV/AIDS	Rate	2011	22.7956	29.1361	18.8862
Incidence	Middle SDI	Both	50-69 yea	HIV/AIDS	Number	2012	76653.8	100022	62858.1
Incidence	Middle SDI	Both	50-69 yea	HIV/AIDS	Rate	2012	21.6739	28.2813	17.7731
Incidence	Middle SDI	Both	50-69 yea	HIV/AIDS	Number	2013	75937.3	99013.8	61350.8
Incidence	Middle SDI	Both	50-69 yea	HIV/AIDS	Rate	2013	20.6546	26.9313	16.6871
Incidence	Middle SDI	Both	50-69 yea	HIV/AIDS	Number	2014	75848.5	98767.8	60320.9
Incidence	Middle SDI	Both	50-69 yea	HIV/AIDS	Rate	2014	19.8101	25.7961	15.7546
Incidence	Middle SDI	Both	50-69 yea	HIV/AIDS	Number	2015	75642.4	98425.1	59421.9
Incidence	Middle SDI	Both	50-69 yea	HIV/AIDS	Rate	2015	18.9772	24.6929	14.9078
Incidence	Middle SDI	Both	50-69 yea	HIV/AIDS	Number	2016	75792.4	98210.4	58923
Incidence	Middle SDI	Both	50-69 yea	HIV/AIDS	Rate	2016	18.3084	23.7237	14.2334
Incidence	Middle SDI	Both	50-69 yea	HIV/AIDS	Number	2017	75491.2	97851.1	58256.3
Incidence	Middle SDI	Both	50-69 yea	HIV/AIDS	Rate	2017	17.5951	22.8066	13.5781
Incidence	Middle SDI	Both	50-69 yea	HIV/AIDS	Number	2018	73278.4	96034.3	55679.6
Incidence	Middle SDI	Both	50-69 yea	HIV/AIDS	Rate	2018	16.5186	21.6482	12.5514
Incidence	Middle SDI	Both	50-69 yea	HIV/AIDS	Number	2019	71830.2	94126.3	54052.6
Incidence	Middle SDI	Both	50-69 yea	HIV/AIDS	Rate	2019	15.7186	20.5976	11.8283
Incidence	High-midc	Both	50-69 yea	Sexually tr	Number	1990	1.1E+07	1.4E+07	8723408
Incidence	High-midc	Both	50-69 yea	Sexually tr	Rate	1990	5975.84	7563.07	4686.34
Incidence	High-midc	Both	50-69 yea	Sexually tr	Number	1991	1.1E+07	1.4E+07	8805057
Incidence	High-midc	Both	50-69 yea	Sexually tr	Rate	1991	5937.26	7509.9	4664.75
Incidence	High-midc	Both	50-69 yea	Sexually tr	Number	1992	1.1E+07	1.4E+07	8841436
Incidence	High-midc	Both	50-69 yea	Sexually tr	Rate	1992	5892.32	7452.69	4637.67
Incidence	High-midc	Both	50-69 yea	Sexually tr	Number	1993	1.1E+07	1.4E+07	8815238
Incidence	High-midc	Both	50-69 yea	Sexually tr	Rate	1993	5836.13	7381.31	4607.45
Incidence	High-midc	Both	50-69 yea	Sexually tr	Number	1994	1.1E+07	1.4E+07	8750917
Incidence	High-midc	Both	50-69 yea	Sexually tr	Rate	1994	5787.36	7319.96	4570.07
Incidence	High-midc	Both	50-69 yea	Sexually tr	Number	1995	1.1E+07	1.4E+07	8723139
Incidence	High-midc	Both	50-69 yea	Sexually tr	Rate	1995	5761.34	7291.47	4548.55
Incidence	High-midc	Both	50-69 yea	Sexually tr	Number	1996	1.1E+07	1.4E+07	8800357
Incidence	High-midc	Both	50-69 yea	Sexually tr	Rate	1996	5777.12	7302.36	4555.6
Incidence	High-midc	Both	50-69 yea	Sexually tr	Number	1997	1.1E+07	1.4E+07	9013870
Incidence	High-midc	Both	50-69 yea	Sexually tr	Rate	1997	5838.46	7377.99	4603.28
Incidence	High-midc	Both	50-69 yea	Sexually tr	Number	1998	1.2E+07	1.5E+07	9210475
Incidence	High-midc	Both	50-69 yea	Sexually tr	Rate	1998	5910.07	7475.13	4642.16
Incidence	High-midc	Both	50-69 yea	Sexually tr	Number	1999	1.2E+07	1.5E+07	9522268
Incidence	High-midc	Both	50-69 yea	Sexually tr	Rate	1999	6019.18	7608.43	4715.73
Incidence	High-midc	Both	50-69 yea	Sexually tr	Number	2000	1.3E+07	1.6E+07	9764969
Incidence	High-midc	Both	50-69 yea	Sexually tr	Rate	2000	6096.25	7721.72	4759.64
Incidence	High-midc	Both	50-69 yea	Sexually tr	Number	2001	1.3E+07	1.6E+07	1E+07
Incidence	High-midc	Both	50-69 yea	Sexually tr	Rate	2001	6188.49	7853.75	4824.1
Incidence	High-midc	Both	50-69 yea	Sexually tr	Number	2002	1.3E+07	1.7E+07	1E+07
Incidence	High-midc	Both	50-69 yea	Sexually tr	Rate	2002	6244.94	7928.55	4872.54
Incidence	High-midc	Both	50-69 yea	Sexually tr	Number	2003	1.4E+07	1.8E+07	1.1E+07
Incidence	High-midc	Both	50-69 yea	Sexually tr	Rate	2003	6316.07	8042.05	4921.22
Incidence	High-midc	Both	50-69 yea	Sexually tr	Number	2004	1.4E+07	1.8E+07	1.1E+07
Incidence	High-midc	Both	50-69 yea	Sexually tr	Rate	2004	6365.97	8118.08	4954.01
Incidence	High-midc	Both	50-69 yea	Sexually tr	Number	2005	1.5E+07	1.9E+07	1.2E+07
Incidence	High-midc	Both	50-69 yea	Sexually tr	Rate	2005	6429.94	8209.06	4996.25
Incidence	High-midc	Both	50-69 yea	Sexually tr	Number	2006	1.5E+07	2E+07	1.2E+07
Incidence	High-midc	Both	50-69 yea	Sexually tr	Rate	2006	6437.18	8233.64	4995.26
Incidence	High-midc	Both	50-69 yea	Sexually tr	Number	2007	1.6E+07	2E+07	1.2E+07
Incidence	High-midc	Both	50-69 yea	Sexually tr	Rate	2007	6409.58	8204.11	4971.13
Incidence	High-midc	Both	50-69 yea	Sexually tr	Number	2008	1.6E+07	2.1E+07	1.2E+07
Incidence	High-midc	Both	50-69 yea	Sexually tr	Rate	2008	6362.75	8150.8	4940.16
Incidence	High-midc	Both	50-69 yea	Sexually tr	Number	2009	1.6E+07	2.1E+07	1.3E+07
Incidence	High-midc	Both	50-69 yea	Sexually tr	Rate	2009	6299.71	8050.91	4904.95
Incidence	High-midc	Both	50-69 yea	Sexually tr	Number	2010	1.7E+07	2.1E+07	1.3E+07
Incidence	High-midc	Both	50-69 yea	Sexually tr	Rate	2010	6253.49	8003.91	4884.77
Incidence	High-midc	Both	50-69 yea	Sexually tr	Number	2011	1.7E+07	2.2E+07	1.3E+07
Incidence	High-midc	Both	50-69 yea	Sexually tr	Rate	2011	6205.38	7928.52	4839.52
Incidence	High-midc	Both	50-69 yea	Sexually tr	Number	2012	1.7E+07	2.2E+07	1.3E+07
Incidence	High-midc	Both	50-69 yea	Sexually tr	Rate	2012	6160.6	7860.1	4802.61
Incidence	High-midc	Both	50-69 yea	Sexually tr	Number	2013	1.8E+07	2.3E+07	1.4E+07
Incidence	High-midc	Both	50-69 yea	Sexually tr	Rate	2013	6126.15	7814.78	4780.98
Incidence	High-midc	Both	50-69 yea	Sexually tr	Number	2014	1.8E+07	2.3E+07	1.4E+07
Incidence	High-midc	Both	50-69 yea	Sexually tr	Rate	2014	6098.38	7778.34	4763.08
Incidence	High-midc	Both	50-69 yea	Sexually tr	Number	2015	1.9E+07	2.4E+07	1.5E+07
Incidence	High-midc	Both	50-69 yea	Sexually tr	Rate	2015	6077.08	7746.64	4755.08
Incidence	High-midc	Both	50-69 yea	Sexually tr	Number	2016	1.9E+07	2.5E+07	1.5E+07
Incidence	High-midc	Both	50-69 yea	Sexually tr	Rate	2016	6088.45	7745.11	4769.88
Incidence	High-midc	Both	50-69 yea	Sexually tr	Number	2017	2E+07	2.5E+07	1.5E+07
Incidence	High-midc	Both	50-69 yea	Sexually tr	Rate	2017	6107.89	7750.28	4774.37
Incidence	High-midc	Both	50-69 yea	Sexually tr	Number	2018	2E+07	2.6E+07	1.6E+07
Incidence	High-midc	Both	50-69 yea	Sexually tr	Rate	2018	6107.27	7743.03	4780.86
Incidence	High-midc	Both	50-69 yea	Sexually tr	Number	2019	2.1E+07	2.6E+07	1.6E+07
Incidence	High-midc	Both	50-69 yea	Sexually tr	Rate	2019	6123.69	7784	4783.82
Incidence	Middle SDI	Both	50-69 yea	Sexually tr	Number	1990	1.4E+07	1.8E+07	1.1E+07
Incidence	Middle SDI	Both	50-69 yea	Sexually tr	Rate	1990	7532.53	9584.81	5846.86
Incidence	Middle SDI	Both	50-69 yea	Sexually tr	Number	1991	1.4E+07	1.8E+07	1.1E+07
Incidence	Middle SDI	Both	50-69 yea	Sexually tr	Rate	1991	7499.41	9541.31	5819.5
Incidence	Middle SDI	Both	50-69 yea	Sexually tr	Number	1992	1.5E+07	1.9E+07	1.1E+07
Incidence	Middle SDI	Both	50-69 yea	Sexually tr	Rate	1992	7470.05	9502.76	5803.35
Incidence	Middle SDI	Both	50-69 yea	Sexually tr	Number	1993	1.5E+07	1.9E+07	1.2E+07
Incidence	Middle SDI	Both	50-69 yea	Sexually tr	Rate	1993	7443.8	9468.25	5780.25
Incidence	Middle SDI	Both	50-69 yea	Sexually tr	Number	1994	1.5E+07	1.9E+07	1.2E+07
Incidence	Middle SDI	Both	50-69 yea	Sexually tr	Rate	1994	7418.57	9431.32	5763.67
Incidence	Middle SDI	Both	50-69 yea	Sexually tr	Number	1995	1.5E+07	2E+07	1.2E+07
Incidence	Middle SDI	Both	50-69 yea	Sexually tr	Rate	1995	7396.22	9401.74	5756.75

Incidence	Middle SDI	Both	50-69 yea	Sexually tra	Rate	2013	7254.33	9258.2	5643.93
Incidence	Middle SDI	Both	50-69 yea	Sexually tra	Number	2014	2.8E+07	3.5E+07	2.2E+07
Incidence	Middle SDI	Both	50-69 yea	Sexually tra	Rate	2014	7246.36	9221.66	5625.72
Incidence	Middle SDI	Both	50-69 yea	Sexually tra	Number	2015	2.9E+07	3.7E+07	2.2E+07
Incidence	Middle SDI	Both	50-69 yea	Sexually tra	Rate	2015	7248.84	9238.23	5628.08
Incidence	Middle SDI	Both	50-69 yea	Sexually tra	Number	2016	3E+07	3.8E+07	2.3E+07
Incidence	Middle SDI	Both	50-69 yea	Sexually tra	Rate	2016	7282.27	9291.47	5641.84
Incidence	Middle SDI	Both	50-69 yea	Sexually tra	Number	2017	3.1E+07	4E+07	2.4E+07
Incidence	Middle SDI	Both	50-69 yea	Sexually tra	Rate	2017	7309.42	9352.21	5666.87
Incidence	Middle SDI	Both	50-69 yea	Sexually tra	Number	2018	3.2E+07	4.1E+07	2.5E+07
Incidence	Middle SDI	Both	50-69 yea	Sexually tra	Rate	2018	7310.63	9351.97	5678.42
Incidence	Middle SDI	Both	50-69 yea	Sexually tra	Number	2019	3.3E+07	4.3E+07	2.6E+07
Incidence	Middle SDI	Both	50-69 yea	Sexually tra	Rate	2019	7323.85	9371.16	5691.03
Incidence	High SDI	Both	50-69 yea	HIV/AIDS	Number	1990	5512.66	6779.83	4063.42
Incidence	High SDI	Both	50-69 yea	HIV/AIDS	Rate	1990	3.5935	4.41952	2.64879
Incidence	High SDI	Both	50-69 yea	HIV/AIDS	Number	1991	5170.01	6379.84	3815.25
Incidence	High SDI	Both	50-69 yea	HIV/AIDS	Rate	1991	3.33376	4.11389	2.46017
Incidence	High SDI	Both	50-69 yea	HIV/AIDS	Number	1992	5365.45	6793.52	4028.49
Incidence	High SDI	Both	50-69 yea	HIV/AIDS	Rate	1992	3.42708	4.33923	2.57313
Incidence	High SDI	Both	50-69 yea	HIV/AIDS	Number	1993	5972.82	7836.33	4322.01
Incidence	High SDI	Both	50-69 yea	HIV/AIDS	Rate	1993	3.77027	4.94658	2.72821
Incidence	High SDI	Both	50-69 yea	HIV/AIDS	Number	1994	5147.78	6561.19	3799.72
Incidence	High SDI	Both	50-69 yea	HIV/AIDS	Rate	1994	3.21155	4.09334	2.37053
Incidence	High SDI	Both	50-69 yea	HIV/AIDS	Number	1995	6084.76	7942.83	4341.19
Incidence	High SDI	Both	50-69 yea	HIV/AIDS	Rate	1995	3.76796	4.91857	2.68827
Incidence	High SDI	Both	50-69 yea	HIV/AIDS	Number	1996	7089.29	9255.68	4859.8
Incidence	High SDI	Both	50-69 yea	HIV/AIDS	Rate	1996	4.35626	5.68747	2.98627
Incidence	High SDI	Both	50-69 yea	HIV/AIDS	Number	1997	9697.93	12903.5	6544.79
Incidence	High SDI	Both	50-69 yea	HIV/AIDS	Rate	1997	5.82668	7.75263	3.93222
Incidence	High SDI	Both	50-69 yea	HIV/AIDS	Number	1998	9974.45	13241.4	6994.73
Incidence	High SDI	Both	50-69 yea	HIV/AIDS	Rate	1998	5.85469	7.77231	4.10569
Incidence	High SDI	Both	50-69 yea	HIV/AIDS	Number	1999	10194.1	13614.6	7346.12
Incidence	High SDI	Both	50-69 yea	HIV/AIDS	Rate	1999	5.8484	7.81076	4.21451
Incidence	High SDI	Both	50-69 yea	HIV/AIDS	Number	2000	11161.2	14651.3	8134.96
Incidence	High SDI	Both	50-69 yea	HIV/AIDS	Rate	2000	6.26305	8.2215	4.56489
Incidence	High SDI	Both	50-69 yea	HIV/AIDS	Number	2001	10948.9	14375.1	8132.04
Incidence	High SDI	Both	50-69 yea	HIV/AIDS	Rate	2001	6.02003	7.90381	4.47122
Incidence	High SDI	Both	50-69 yea	HIV/AIDS	Number	2002	11217.9	14760.8	8396.8
Incidence	High SDI	Both	50-69 yea	HIV/AIDS	Rate	2002	6.03776	7.94463	4.51936
Incidence	High SDI	Both	50-69 yea	HIV/AIDS	Number	2003	11259.5	14843.2	8394.24
Incidence	High SDI	Both	50-69 yea	HIV/AIDS	Rate	2003	5.93367	7.82222	4.4237
Incidence	High SDI	Both	50-69 yea	HIV/AIDS	Number	2004	11360.2	15204.6	8378.03
Incidence	High SDI	Both	50-69 yea	HIV/AIDS	Rate	2004	5.86063	7.84392	4.32216
Incidence	High SDI	Both	50-69 yea	HIV/AIDS	Number	2005	11191.3	15237.4	8236.5
Incidence	High SDI	Both	50-69 yea	HIV/AIDS	Rate	2005	5.6497	7.69231	4.15804
Incidence	High SDI	Both	50-69 yea	HIV/AIDS	Number	2006	11703.1	16376.2	8431.24
Incidence	High SDI	Both	50-69 yea	HIV/AIDS	Rate	2006	5.78655	8.09718	4.1688
Incidence	High SDI	Both	50-69 yea	HIV/AIDS	Number	2007	11564.2	16485.4	8209.98
Incidence	High SDI	Both	50-69 yea	HIV/AIDS	Rate	2007	5.60078	7.98419	3.97626
Incidence	High SDI	Both	50-69 yea	HIV/AIDS	Number	2008	11709.3	17119.9	8086.28
Incidence	High SDI	Both	50-69 yea	HIV/AIDS	Rate	2008	5.55368	8.11994	3.8353
Incidence	High SDI	Both	50-69 yea	HIV/AIDS	Number	2009	11947	17744.9	8083.74
Incidence	High SDI	Both	50-69 yea	HIV/AIDS	Rate	2009	5.54964	8.24288	3.75507
Incidence	High SDI	Both	50-69 yea	HIV/AIDS	Number	2010	12766.5	19359.8	8299.01
Incidence	High SDI	Both	50-69 yea	HIV/AIDS	Rate	2010	5.81026	8.81094	3.77701
Incidence	High SDI	Both	50-69 yea	HIV/AIDS	Number	2011	13242	20499.6	8148.38
Incidence	High SDI	Both	50-69 yea	HIV/AIDS	Rate	2011	5.90879	9.14726	3.63595
Incidence	High SDI	Both	50-69 yea	HIV/AIDS	Number	2012	13579	21572.9	7942.02
Incidence	High SDI	Both	50-69 yea	HIV/AIDS	Rate	2012	5.94089	9.43828	3.47468
Incidence	High SDI	Both	50-69 yea	HIV/AIDS	Number	2013	13887.7	22873.2	7679.59
Incidence	High SDI	Both	50-69 yea	HIV/AIDS	Rate	2013	5.96238	9.82009	3.29706
Incidence	High SDI	Both	50-69 yea	HIV/AIDS	Number	2014	13765.3	23024.4	7013.43
Incidence	High SDI	Both	50-69 yea	HIV/AIDS	Rate	2014	5.80164	9.70401	2.95593
Incidence	High SDI	Both	50-69 yea	HIV/AIDS	Number	2015	14308.9	24529	6721.29
Incidence	High SDI	Both	50-69 yea	HIV/AIDS	Rate	2015	5.91124	10.1333	2.77668
Incidence	High SDI	Both	50-69 yea	HIV/AIDS	Number	2016	14822.9	25257	6857.28
Incidence	High SDI	Both	50-69 yea	HIV/AIDS	Rate	2016	6.02278	10.2624	2.78623
Incidence	High SDI	Both	50-69 yea	HIV/AIDS	Number	2017	14508.2	24766.7	6786.44
Incidence	High SDI	Both	50-69 yea	HIV/AIDS	Rate	2017	5.8459	9.97946	2.73452
Incidence	High SDI	Both	50-69 yea	HIV/AIDS	Number	2018	14180.7	23956.3	6686.99
Incidence	High SDI	Both	50-69 yea	HIV/AIDS	Rate	2018	5.67285	9.5835	2.67506
Incidence	High SDI	Both	50-69 yea	HIV/AIDS	Number	2019	14203.3	24045.9	6691.22
Incidence	High SDI	Both	50-69 yea	HIV/AIDS	Rate	2019	5.63921	9.54705	2.65664

measure	location	sex	age	cause	metric	year	val	upper	lower
Deaths	East Asia	Both	50-69 yea	HIV/AIDS	Number	1990	539.374	820.438	71.6877
Deaths	East Asia	Both	50-69 yea	HIV/AIDS	Rate	1990	0.33672	0.51218	0.04475
Deaths	Southern S	Both	50-69 yea	Sexually tra	Number	1991	53.4913	81.4954	34.1966
Deaths	Southern S	Both	50-69 yea	Sexually tra	Rate	1991	1.10875	1.68921	0.70881
Deaths	Oceania	Both	50-69 yea	Sexually tra	Number	1990	1.62465	3.23294	0.72926
Deaths	Oceania	Both	50-69 yea	Sexually tra	Rate	1990	0.28641	0.56994	0.12856
Deaths	Australasia	Both	50-69 yea	Sexually tra	Number	1990	2.98559	3.56183	2.52258
Deaths	Australasia	Both	50-69 yea	Sexually tra	Rate	1990	0.08675	0.10349	0.07329
Deaths	Southern S	Both	50-69 yea	HIV/AIDS	Number	1991	2553.44	5284.3	1068.47
Deaths	Southern S	Both	50-69 yea	HIV/AIDS	Rate	1991	52.9266	109.531	22.1468
Deaths	High-incor	Both	50-69 yea	Sexually tra	Number	1991	22.568	24.1851	18.9225
Deaths	High-incor	Both	50-69 yea	Sexually tra	Rate	1991	0.06425	0.06886	0.05387
Deaths	High-incor	Both	50-69 yea	Sexually tra	Number	1990	45.701	47.8601	42.6408
Deaths	High-incor	Both	50-69 yea	Sexually tra	Rate	1990	0.09617	0.10072	0.08973
Deaths	Southeast	Both	50-69 yea	Sexually tra	Number	1990	88.5138	119.511	65.7564
Deaths	Southeast	Both	50-69 yea	Sexually tra	Rate	1990	0.18738	0.253	0.1392
Deaths	Southern L	Both	50-69 yea	Sexually tra	Number	1991	19.1914	21.7538	15.3818
Deaths	Southern L	Both	50-69 yea	Sexually tra	Rate	1991	0.25312	0.28692	0.20288
Deaths	Central Su	Both	50-69 yea	Sexually tra	Number	1990	105.207	214.502	23.9051
Deaths	Central Su	Both	50-69 yea	Sexually tra	Rate	1990	2.3702	4.83251	0.53856
Deaths	Caribbean	Both	50-69 yea	HIV/AIDS	Number	1990	595.849	937.104	398.305
Deaths	Caribbean	Both	50-69 yea	HIV/AIDS	Rate	1990	14.4263	22.6886	9.6435
Deaths	High-incor	Both	50-69 yea	HIV/AIDS	Number	1991	56.346	58.2037	54.5572
Deaths	High-incor	Both	50-69 yea	HIV/AIDS	Rate	1991	0.16042	0.16571	0.15533
Deaths	Central Lat	Both	50-69 yea	HIV/AIDS	Number	1993	971.31	986.463	954.986
Deaths	Central Lat	Both	50-69 yea	HIV/AIDS	Rate	1993	6.13177	6.22743	6.02872
Deaths	Eastern Eu	Both	50-69 yea	HIV/AIDS	Number	1990	644.123	656.399	633.237
Deaths	Eastern Eu	Both	50-69 yea	HIV/AIDS	Rate	1990	1.29893	1.32369	1.27698
Deaths	Central Lat	Both	50-69 yea	Sexually tra	Number	1993	28.7279	33.8063	26.8398
Deaths	Central Lat	Both	50-69 yea	Sexually tra	Rate	1993	0.18136	0.21342	0.16944
Deaths	Tropical La	Both	50-69 yea	Sexually tra	Number	1991	39.0664	42.9317	36.5775
Deaths	Tropical La	Both	50-69 yea	Sexually tra	Rate	1991	0.23645	0.25985	0.22139
Deaths	Tropical La	Both	50-69 yea	Sexually tra	Number	1990	41.0276	45.0949	38.2048
Deaths	Tropical La	Both	50-69 yea	Sexually tra	Rate	1990	0.25556	0.28089	0.23798
Deaths	Oceania	Both	50-69 yea	HIV/AIDS	Number	1990	1.91213	8.96639	0.23597
Deaths	Oceania	Both	50-69 yea	HIV/AIDS	Rate	1990	0.33709	1.58071	0.0416
Deaths	South Asia	Both	50-69 yea	Sexually tra	Number	1991	969.887	1234.55	687.258
Deaths	South Asia	Both	50-69 yea	Sexually tra	Rate	1991	0.88043	1.12068	0.62387
Deaths	Southern L	Both	50-69 yea	HIV/AIDS	Number	1992	229.924	236.075	224.153
Deaths	Southern L	Both	50-69 yea	HIV/AIDS	Rate	1992	2.9966	3.07676	2.92138
Deaths	South Asia	Both	50-69 yea	Sexually tra	Number	1992	997.167	1268.52	691.375
Deaths	South Asia	Both	50-69 yea	Sexually tra	Rate	1992	0.88371	1.12419	0.61271
Deaths	Central As	Both	50-69 yea	Sexually tra	Number	1992	40.7875	45.8963	30.0111
Deaths	Central As	Both	50-69 yea	Sexually tra	Rate	1992	0.45717	0.51443	0.33638
Deaths	Western Su	Both	50-69 yea	HIV/AIDS	Number	1990	4361.12	6965.31	2624.53
Deaths	Western Su	Both	50-69 yea	HIV/AIDS	Rate	1990	27.9283	44.6053	16.8073
Deaths	Australasia	Both	50-69 yea	HIV/AIDS	Number	1990	75.7527	79.3251	72.2515
Deaths	Australasia	Both	50-69 yea	HIV/AIDS	Rate	1990	2.20103	2.30483	2.09931
Deaths	Oceania	Both	50-69 yea	Sexually tra	Number	1991	1.62775	3.33484	0.71987
Deaths	Oceania	Both	50-69 yea	Sexually tra	Rate	1991	0.28542	0.58475	0.12623
Deaths	High-incor	Both	50-69 yea	Sexually tra	Number	1991	44.536	47.0204	41.8545
Deaths	High-incor	Both	50-69 yea	Sexually tra	Rate	1991	0.0933	0.09851	0.08768
Deaths	Andean La	Both	50-69 yea	Sexually tra	Number	1990	8.33817	12.4664	5.87294
Deaths	Andean La	Both	50-69 yea	Sexually tra	Rate	1990	0.23897	0.35729	0.16832
Deaths	Southern S	Both	50-69 yea	Sexually tra	Number	1992	55.7618	84.3348	36.1971
Deaths	Southern S	Both	50-69 yea	Sexually tra	Rate	1992	1.12512	1.70164	0.73036
Deaths	Andean La	Both	50-69 yea	Sexually tra	Number	1992	8.30328	12.4554	6.01321
Deaths	Andean La	Both	50-69 yea	Sexually tra	Rate	1992	0.22319	0.3348	0.16164
Deaths	Southern L	Both	50-69 yea	HIV/AIDS	Number	1991	187.118	191.889	182.61
Deaths	Southern L	Both	50-69 yea	HIV/AIDS	Rate	1991	2.46798	2.5309	2.40852
Deaths	East Asia	Both	50-69 yea	Sexually tra	Number	1990	418.577	516.382	279.712
Deaths	East Asia	Both	50-69 yea	Sexually tra	Rate	1990	0.26131	0.32236	0.17462
Deaths	Central Eu	Both	50-69 yea	HIV/AIDS	Number	1991	145.955	154.191	136.919
Deaths	Central Eu	Both	50-69 yea	HIV/AIDS	Rate	1991	0.58002	0.61275	0.54411
Deaths	South Asia	Both	50-69 yea	HIV/AIDS	Number	1992	319.055	665.887	158.032
Deaths	South Asia	Both	50-69 yea	HIV/AIDS	Rate	1992	0.28275	0.59012	0.14005
Deaths	Eastern Su	Both	50-69 yea	HIV/AIDS	Number	1992	32358.4	44105.9	21605.8
Deaths	Eastern Su	Both	50-69 yea	HIV/AIDS	Rate	1992	228.935	312.048	152.861
Deaths	Southeast	Both	50-69 yea	HIV/AIDS	Number	1990	246.405	410.054	148.276
Deaths	Southeast	Both	50-69 yea	HIV/AIDS	Rate	1990	0.52162	0.86805	0.31389
Deaths	High-incor	Both	50-69 yea	HIV/AIDS	Number	1990	3652.07	3697.97	3604.71
Deaths	High-incor	Both	50-69 yea	HIV/AIDS	Rate	1990	7.68543	7.78202	7.58575
Deaths	Eastern Su	Both	50-69 yea	Sexually tra	Number	1992	467.05	910.432	177.961
Deaths	Eastern Su	Both	50-69 yea	Sexually tra	Rate	1992	3.30436	6.44128	1.25907
Deaths	Tropical La	Both	50-69 yea	HIV/AIDS	Number	1991	968.884	992.186	944.512
Deaths	Tropical La	Both	50-69 yea	HIV/AIDS	Rate	1991	5.86426	6.0053	5.71674
Deaths	North Afric	Both	50-69 yea	Sexually tra	Number	1990	19.7768	25.9686	14.8652
Deaths	North Afric	Both	50-69 yea	Sexually tra	Rate	1990	0.06296	0.08267	0.04732
Deaths	South Asia	Both	50-69 yea	HIV/AIDS	Number	1990	104.843	324.109	30.1529
Deaths	South Asia	Both	50-69 yea	HIV/AIDS	Rate	1990	0.09759	0.30169	0.02807
Deaths	Caribbean	Both	50-69 yea	Sexually tra	Number	1990	19.9552	32.5702	11.3444
Deaths	Caribbean	Both	50-69 yea	Sexually tra	Rate	1990	0.48314	0.78857	0.27466
Deaths	Southern L	Both	50-69 yea	Sexually tra	Number	1990	18.4995	21.1967	15.4948
Deaths	Southern L	Both	50-69 yea	Sexually tra	Rate	1990	0.24707	0.28309	0.20694
Deaths	Southern S	Both	50-69 yea	HIV/AIDS	Number	1990	1540.65	3354.26	631.836
Deaths	Southern S	Both	50-69 yea	HIV/AIDS	Rate	1990	32.7877	71.3846	13.4466
Deaths	Oceania	Both	50-69 yea	HIV/AIDS	Number	1991	3.06875	12.8157	0.51541
Deaths	Oceania	Both	50-69 yea	HIV/AIDS	Rate	1991	0.5381	2.24719	0.09038
Deaths	Australasia	Both	50-69 yea	HIV/AIDS	Number	1992	94.1688	98.114	90.2701
Deaths	Australasia	Both	50-69 yea	HIV/AIDS	Rate	1992	2.6731	2.78509	2.56243
Deaths	High-incor	Both	50-69 yea	HIV/AIDS	Number	1992	60.7715	62.8901	58.8163
Deaths	High-incor	Both	50-69 yea	HIV/AIDS	Rate	1992	0.16821	0.17407	0.1628
Deaths	Eastern Eu	Both	50-69 yea	Sexually tra	Number	1990	182.829	200.62	141.345
Deaths	Eastern Eu	Both	50-69 yea	Sexually tra	Rate	1990	0.36869	0.40457	0.28503
Deaths	Western Su	Both	50-69 yea	Sexually tra	Number	1990	63.6799	85.2716	31.7069
Deaths	Western Su	Both	50-69 yea	Sexually tra	Rate	1990	0.4078	0.54607	0.20305
Deaths	High-incor	Both	50-69 yea	HIV/AIDS	Number	1991	4350.77	4406.72	4294.7
Deaths	High-incor	Both	50-69 yea	HIV/AIDS	Rate	1991	9.1148	9.232	8.99732
Deaths	Southern S	Both	50-69 yea	HIV/AIDS	Number	1992	4056.09	7878.47	1738.38
Deaths	Southern S	Both	50-69 yea	HIV/AIDS	Rate	1992	81.8406	158.966	35.0756
Deaths	Andean La	Both	50-69 yea	HIV/AIDS	Number	1990	103.548	261.184	64.3736
Deaths	Andean La	Both	50-69 yea	HIV/AIDS	Rate	1990	2.9677	7.48556	1.84496
Deaths	Southern L	Both	50-69 yea	HIV/AIDS	Number	1993	253.17	258.662	247.759
Deaths	Southern L	Both	50-69 yea	HIV/AIDS	Rate	1993	3.2613	3.33204	3.19159
Deaths	North Afric	Both	50-69 yea	HIV/AIDS	Number	1990	98.4962	223.213	40.4957
Deaths	North Afric	Both	50-69 yea	HIV/AIDS	Rate	1990	0.31355	0.71058	0.12891
Deaths	Central As	Both	50-69 yea	HIV/AIDS	Number	1992	33.3973	35.2653	31.0316
Deaths	Central As	Both	50-69 yea	HIV/AIDS	Rate	1992	0.37434	0.39527	0.34782
Deaths	East Asia	Both	50-69 yea	HIV/AIDS	Number	1991	695.162	926.184	163.405
Deaths	East Asia	Both	50-69 yea	HIV/AIDS	Rate	1991	0.42679	0.56862	0.10032
Deaths	North Afric	Both	50-69 yea	HIV/AIDS	Number	1992	155.008	327.468	72.4525
Deaths	North Afric	Both	50-69 yea	HIV/AIDS	Rate	1992	0.47121	0.99547	0.22025
Deaths	Australasia	Both	50-69 yea	Sexually tra	Number	1991	2.67464	3.22476	2.25097
Deaths	Australasia	Both	50-69 yea	Sexually tra	Rate	1991	0.07689	0.09271	0.06471
Deaths	Eastern Su	Both	50-69 yea	Sexually tra	Number	1990	466.407	906.526	174.355
Deaths	Eastern Su	Both	50-69 yea	Sexually tra	Rate	1990	3.413	6.63363	1.27587
Deaths	Central Eu	Both	50-69 yea	Sexually tra	Number	1990	20.9963	22.4146	16.4606
Deaths	Central Eu	Both	50-69 yea	Sexually tra	Rate	1990	0.08342	0.08906	0.0654
Deaths	Southeast	Both	50-69 yea	HIV/AIDS	Number	1992	1006.7	1587.55	525.63
Deaths	Southeast	Both	50-69 yea	HIV/AIDS	Rate	1992	2.03648	3.2115	1.06332
Deaths	Western Eu	Both	50-69 yea	Sexually tra	Number	1994	64.4709	71.7467	59.8833
Deaths	Western Eu	Both	50-69 yea	Sexually tra	Rate	1994	0.07705	0.08575	0.07157
Deaths	Australasia	Both	50-69 yea	Sexually tra	Number	1992	2.43178	2.94144	2.04412
Deaths	Australasia	Both	50-69 yea	Sexually tra	Rate	1992	0.06903	0.0835	0.05803
Deaths	Western Eu	Both	50-69 yea	Sexually tra	Number	1990	89.5006	97.4783	82.1563
Deaths	Western Eu	Both	50-69 yea	Sexually tra	Rate	1990	0.10815	0.11779	0.09927
Deaths	Central Lat	Both	50-69 yea	Sexually tra	Number	1991	30.9081	36.1526	28.7849
Deaths	Central Lat	Both	50-69 yea	Sexually tra	Rate	1991	0.20793	0.24321	0.19365
Deaths	Tropical La	Both	50-69 yea	HIV/AIDS	Number	1990	750.602	769.24	730.782
Deaths	Tropical La	Both	50-69 yea	HIV/AIDS	Rate	1990	4.67546	4.79156	4.55201
Deaths	Western Eu	Both	50-69 yea	Sexually tra	Number	1991	82.2475	89.4512	75.3299
Deaths	Western Eu	Both	50-69 yea	Sexually tra	Rate	1991	0.09891	0.10757	0.09059
Deaths	Central Su	Both	50-69 yea	Sexually tra	Number	1991	105.235	214.96	24.0697
Deaths	Central Su	Both	50-69 yea	Sexually tra	Rate	1991	2.33207	4.76365	0.5334
Deaths	Central Eu	Both	50-69 yea	HIV/AIDS	Number	1990	132.739	139.855	124.939
Deaths	Central Eu	Both	50-69 yea	HIV/AIDS	Rate	1990	0.52741	0.55569	0.49642
Deaths	East Asia	Both	50-69 yea	Sexually tra	Number	1991	405.574	495.729	271.197
Deaths	East Asia	Both	50-69 yea	Sexually tra	Rate	1991	0.249	0.30435	0.1665
Deaths	Southeast	Both	50-69 yea	HIV/AIDS	Number	1991	515.153	877.767	267.55
Deaths	Southeast	Both	50-69 yea	HIV/AIDS	Rate	1991	1.06444	1.8137	0.55283
Deaths	Western Eu	Both	50-69 yea	HIV/AIDS	Number	1990	1564.82	1594.08	1539.35
Deaths	Western Eu	Both	50-69 yea	HIV/AIDS	Rate	1990	1.89084	1.9262	1.86007
Deaths	Central Su	Both	50-69 yea	HIV/AIDS	Number	1995	7414.32	9571.78	5488.9
Deaths	Central Su	Both	50-69 yea	HIV/AIDS	Rate	1995	154.618	199.609	114.465
Deaths	Tropical La	Both	50-69 yea	HIV/AIDS	Number	1993	1295.37	1326.63	1267.19
Deaths	Tropical La	Both	50-69 yea	HIV/AIDS	Rate	1993	7.42709	7.60633	7.26549
Deaths	Andean La	Both	50-69 yea	Sexually tra	Number	1995	8.39002	12.307	6.17278
Deaths	Andean La	Both	50-69 yea	Sexually tra	Rate	1995	0.20526	0.30109	0.15102
Deaths	Central Su	Both	50-69 yea	HIV/AIDS	Number	1992	4735.03	6664.09	3210.6
Deaths	Central Su	Both	50-69 yea	HIV/AIDS	Rate	1992	103.246	145.308	70.0061
Deaths	Eastern Eu	Both	50-69 yea	Sexually tra	Number	1992	277.503	306.541	163.366
Deaths	Eastern Eu	Both	50-69 yea	Sexually tra	Rate	1992	0.54514	0.60219	0.32093
Deaths	Southeast	Both	50-69 yea	HIV/AIDS	Number	1995	3592.08	4279.54	2481.8
Deaths	Southeast	Both	50-69 yea	HIV/AIDS	Rate	1995	6.8353	8.14346	4.72257
Deaths	Caribbean	Both	50-69 yea	Sexually tra	Number	1995	17.3986	28.6077	10.6814
Deaths	Caribbean	Both	50-69 yea	Sexually tra	Rate	1995	0.37473	0.61615	0.23006
Deaths	Central Su	Both	50-69 yea	HIV/AIDS	Number	1990	2985.27	4467.81	1915.15
Deaths	Central Su	Both	50-69 yea	HIV/AIDS	Rate	1990	67.2551	100.655	43.1464
Deaths	Western Su	Both	50-69 yea	HIV/AIDS	Number	1991	6024.09	9404.59	3775.91
Deaths	Western Su	Both	50-69 yea	HIV/AIDS	Rate	1991	37.8788	59.135	23.7425
Deaths	Caribbean	Both	50-69 yea	Sexually tra	Number	1992	18.5364	30.9679	10.7671
Deaths	Caribbean	Both	50-69 yea	Sexually tra	Rate	1992	0.42878	0.71634	0.24906
Deaths	Western Su	Both	50-69 yea	Sexually tra	Number	1995	62.6417	83.4115	32.8924
Deaths	Western Su	Both	50-69 yea	Sexually tra	Rate	1995	0.36055	0.48009	0.18932
Deaths	High-incor	Both	50-69 yea	Sexually tra	Number	1996	37.3414	39.0925	34.9219
Deaths	High-incor	Both	50-69 yea	Sexually tra	Rate	1996	0.07406	0.07753	0.06926
Deaths	High-incor	Both	50-69 yea	Sexually tra	Number	1992	21.1683	22.7654	18.8402
Deaths	High-incor	Both	50-69 yea	Sexually tra	Rate	1992	0.05859	0.06301	0.05215
Deaths	High-incor	Both	50-69 yea	HIV/AIDS	Number	1992	4828.77	4891.72	4765.52
Deaths	High-incor	Both	50-69 yea	HIV/AIDS	Rate	1992	10.0617	10.1928	9.92988
Deaths	South Asia	Both	50-69 yea	Sexually tra	Number	1993	987.796	1237.09	694.983
Deaths	South Asia	Both	50-69 yea	Sexually tra	Rate	1993	0.85502	1.0708	0.60157
Deaths	East Asia	Both	50-69 yea	HIV/AIDS	Number	1992	852.122	1044.36	321.972
Deaths	East Asia	Both	50-69 yea	HIV/AIDS	Rate	1992	0.51482	0.63096	0.19452
Deaths	Central As	Both	50-69 yea	HIV/AIDS	Number	1993	36.3755	38.153	34.2828
Deaths	Central As	Both	50-69 yea	HIV/AIDS	Rate	1993	0.41076	0.43083	0.38713
Deaths	Central As	Both	50-69 yea	HIV/AIDS	Number	1991	31.8582	33.9151	29.5495
Deaths	Central As	Both	50-69 yea	HIV/AIDS	Rate	1991	0.35935	0.38255	0.33331
Deaths	Oceania	Both	50-69 yea	HIV/AIDS	Number	1996	25.5166	60.1995	10.8168
Deaths	Oceania	Both	50-69 yea	HIV/AIDS	Rate	1996	4.10334	9.68072	1.73945
Deaths	Southern S	Both	50-69 yea	Sexually tra	Number	1993	54.8416	82.953	35.6919
Deaths	Southern S	Both	50-69 yea	Sexually tra	Rate	1993	1.07855	1.63141	0.70194
Deaths	Eastern Eu	Both	50-69 yea	Sexually tra	Number	1995	390.933	444.642	192.279
Deaths	Eastern Eu	Both	50-69 yea	Sexually tra	Rate	1995	0.82961	0.94359	0.40804
Deaths	Southern S	Both	50-69 yea	Sexually tra	Number	1995	58.8637	85.4941	39.0378
Deaths	Southern S	Both	50-69 yea	Sexually tra	Rate	1995	1.1039	1.60332	0.7321
Deaths	Eastern Su	Both	50-69 yea	HIV/AIDS	Number	1991	25891	35809.5	16824.7
Deaths	Eastern Su	Both	50-69 yea	HIV/AIDS	Rate	1991	185.986	257.236	120.86
Deaths	Central As	Both	50-69 yea	Sexually tra	Number	1993	42.0074	47.2837	30.7345
Deaths	Central As	Both	50-69 yea	Sexually tra	Rate	1993	0.47435	0.53393	0.34706
Deaths	Central Eu	Both	50-69 yea	Sexually tra	Number	1991	19.254	20.5598	15.7374
Deaths	Central Eu	Both	50-69 yea	Sexually tra	Rate	1991	0.07651	0.0817	0.06254
Deaths	North Afric	Both	50-69 yea	HIV/AIDS	Number	1995	267.561	524.706	133.749
Deaths	North Afric	Both	50-69 yea	HIV/AIDS	Rate	1995	0.75906	1.48857	0.37944
Deaths	Southern L	Both	50-69 yea	Sexually tra	Number	1992	17.0803	19.2881	14.5965
Deaths	Southern L	Both	50-69 yea	Sexually tra	Rate	1992	0.22261	0.25138	0.19024
Deaths	Southern L	Both	50-69 yea	HIV/AIDS	Number	1995	295.746	302.685	289.288
Deaths	Southern L	Both	50-69 yea	HIV/AIDS	Rate	1995	3.72006	3.80734	3.63883
Deaths	Eastern Eu	Both	50-69 yea	HIV/AIDS	Number	1991	643.692	655.024	631.937
Deaths	Eastern Eu	Both	50-69 yea	HIV/AIDS	Rate	1991	1.27598	1.29845	1.25268
Deaths	Central Eu	Both	50-69 yea	Sexually tra	Number	1996	13.6417	14.5359	12.5857
Deaths	Central Eu	Both	50-69 yea	Sexually tra	Rate	1996	0.05571	0.05936	0.05139
Deaths	Caribbean	Both	50-69 yea	HIV/AIDS	Number	1991	821.8	1309.86	527.624
Deaths	Caribbean	Both	50-69 yea	HIV/AIDS	Rate	1991	19.458	31.014	12.4927
Deaths	Western Eu	Both	50-69 yea	HIV/AIDS	Number	1996	2515.55	2554.15	2478.41
Deaths	Western Eu	Both	50-69 yea	HIV/AIDS	Rate	1996	2.98596	3.03177	2.94187
Deaths	Eastern Su	Both	50-69 yea	HIV/AIDS	Number	1993	39454.1	52623.7	26885
Deaths	Eastern Su	Both	50-69 yea	HIV/AIDS	Rate	1993	275.701	367.729	187.87
Deaths	Australasia	Both	50-69 yea	HIV/AIDS	Number	1991	76.1947	79.6797	72.9063
Deaths	Australasia	Both	50-69 yea	HIV/AIDS	Rate	1991	2.1905	2.29069	2.09596
Deaths	Andean La	Both	50-69 yea	HIV/AIDS	Number	1991	128.799	338.785	81.2973
Deaths	Andean La	Both	50-69 yea	HIV/AIDS	Rate	1991	3.56637	9.38071	2.25107
Deaths	Eastern Su	Both	50-69 yea	Sexually tra	Number	1993	465.247	897.992	176.911
Deaths	Eastern Su	Both	50-69 yea	Sexually tra	Rate	1993	3.25109	6.27507	1.23623
Deaths	Western Eu	Both	50-69 yea	HIV/AIDS	Number	1991	1880.41	1916.45	1847.58
Deaths	Western Eu	Both	50-69 yea	HIV/AIDS	Rate	1991	2.26136	2.3047	2.22187
Deaths	High-incor	Both	50-69 yea	Sexually tra	Number	1992	42.7645	44.9599	40.0054
Deaths	High-incor	Both	50-69 yea	Sexually tra	Rate	1992	0.08911	0.09368	0.08336
Deaths	High-incor	Both	50-69 yea	HIV/AIDS	Number	1993	65.9735	68.2894	63.8026
Deaths	High-incor	Both	50-69 yea	HIV/AIDS	Rate	1993	0.17856	0.18483	0.17268
Deaths	Oceania	Both	50-69 yea	Sexually tra	Number	1992	1.64239	3.33814	0.74216
Deaths	Oceania	Both	50-69 yea	Sexually tra	Rate	1992	0.28387	0.57697	0.12828
Deaths	Tropical La	Both	50-69 yea	Sexually tra	Number	1992	40.1216	44.0095	37.7022
Deaths	Tropical La	Both	50-69 yea	Sexually tra	Rate	1992	0.2363	0.2592	0.22205
Deaths	Southern S	Both	50-69 yea	HIV/AIDS	Number	1993	6166.06	11374.2	2780.94
Deaths	Southern S	Both	50-69 yea	HIV/AIDS	Rate	1993	121.266	223.693	54.692
Deaths	Southern L	Both	50-69 yea	Sexually tra	Number	1993	16.0199	18.1306	13.9005
Deaths	Southern L	Both	50-69 yea	Sexually tra	Rate	1993	0.20637	0.23356	0.17906
Deaths	South Asia	Both	50-69 yea	Sexually tra	Number	1995	994.998	1220.02	722.474
Deaths	South Asia	Both	50-69 yea	Sexually tra	Rate	1995	0.82298	1.0091	0.59757
Deaths	Central Su	Both	50-69 yea	HIV/AIDS	Number	1991	3815.61	5560.44	2505.16
Deaths	Central Su	Both	50-69 yea	HIV/AIDS	Rate	1991	84.5565	123.223	55.516
Deaths	Central As	Both	50-69 yea	HIV/AIDS	Number	1998	54.9298	57.1993	52.7994
Deaths	Central As	Both	50-69 yea	HIV/AIDS	Rate	1998	0.68573	0.71406	0.65914
Deaths	East Asia	Both	50-69 yea	Sexually tra	Number	1992	383.647	468.527	262.017
Deaths	East Asia	Both	50-69 yea	Sexually tra	Rate	1992	0.23178	0.28306	0.1583
Deaths	Central Lat	Both	50-69 yea	HIV/AIDS	Number	1990	615.364	626.615	604.508
Deaths	Central Lat	Both	50-69 yea	HIV/AIDS	Rate	1990	4.26966	4.34772	4.19434
Deaths	High-incor	Both	50-69 yea	Sexually tra	Number	1996	19.9386	21.7937	18.5995
Deaths	High-incor	Both	50-69 yea	Sexually tra	Rate	1996	0.05209	0.05694	0.0486
Deaths	Central Lat	Both	50-69 yea	Sexually tra	Number	1990	31.7262	36.8469	29.6908
Deaths	Central Lat	Both	50-69 yea	Sexually tra	Rate	1990	0.22013	0.25566	0.20601
Deaths	Central Lat	Both	50-69 yea	HIV/AIDS	Number	1991	686.216	698.558	674.703
Deaths	Central Lat	Both	50-69 yea	HIV/AIDS	Rate	1991	4.61649	4.69952	4.53904
Deaths	Central Su	Both	50-69 yea	Sexually tra	Number	1997	104.971	222.6	25.6031
Deaths	Central Su	Both	50-69 yea	Sexually tra	Rate	1997	2.12616	4.50868	0.51858
Deaths	Oceania	Both	50-69 yea	HIV/AIDS	Number	1992	4.5024	18.7063	0.93002
Deaths	Oceania	Both	50-69 yea	HIV/AIDS	Rate	1992	0.7782	3.23322	0.16075
Deaths	Australasia	Both	50-69 yea	Sexually tra	Number	1997	1.52749	1.97397	1.28325
Deaths	Australasia	Both	50-69 yea	Sexually tra	Rate	1997	0.03905	0.05047	0.03281
Deaths	Central As	Both	50-69 yea	Sexually tra	Number	1996	45.1107	50.5646	31.7126
Deaths	Central As	Both	50-69 yea	Sexually tra	Rate	1996	0.54699	0.61313	0.38453
Deaths	North Afric	Both	50-69 yea	HIV/AIDS	Number	1991	125.815	273.435	56.2729
Deaths	North Afric	Both	50-69 yea	HIV/AIDS	Rate	1991	0.39111	0.85002	0.17493
Deaths	Western Su	Both	50-69 yea	HIV/AIDS	Number	1992	8097.41	12045.1	5160.93
Deaths	Western Su	Both	50-69 yea	HIV/AIDS	Rate	1992	49.7429	73.9937	31.7039
Deaths	Caribbean	Both	50-69 yea	Sexually tra	Number	1991	19.1028	31.0936	10.9938
Deaths	Caribbean	Both	50-69 yea	Sexually tra	Rate	1991	0.4523	0.73621	0.2603
Deaths	Andean La	Both	50-69 yea	Sexually tra	Number	1991	8.09994	12.2742	5.79883
Deaths	Andean La	Both	50-69 yea	Sexually tra	Rate	1991	0.22428	0.33987	0.16057
Deaths	High-incor	Both	50-69 yea	Sexually tra	Number	1993	19.8219	21.3592	18.3901
Deaths	High-incor	Both	50-69 yea	Sexually tra	Rate	1993	0.05365	0.05781	0.04977
Deaths	South Asia	Both	50-69 yea	Sexually tra	Number	1999	1026.27	1241.65	752.542
Deaths	South Asia	Both	50-69 yea	Sexually tra	Rate	1999	0.77993	0.94362	0.57191
Deaths	Southern L	Both	50-69 yea	HIV/AIDS	Number	1999	265.11	270.638	259.844
Deaths	Southern L	Both	50-69 yea	HIV/AIDS	Rate	1999	3.16207	3.228	3.09926
Deaths	Tropical La	Both	50-69 yea	Sexually tra	Number	1998	45.9099	49.4688	43.2645
Deaths	Tropical La	Both	50-69 yea	Sexually tra	Rate	1998	0.22774	0.24539	0.21462
Deaths	Southeast	Both	50-69 yea	Sexually tra	Number	1991	88.7036	122.126	65.23
Deaths	Southeast	Both	50-69 yea	Sexually tra	Rate	1991	0.18329	0.25235	0.13478
Deaths	Eastern Su	Both	50-69 yea	HIV/AIDS	Number	1990	20268.2	28913.8	12837.4
Deaths	Eastern Su	Both	50-69 yea	HIV/AIDS	Rate	1990	148.315	211.581	93.9399
Deaths	Eastern Eu	Both	50-69 yea	Sexually tra	Number	1993	332.435	368.156	184.221
Deaths	Eastern Eu	Both	50-69 yea	Sexually tra	Rate	1993	0.66318	0.73444	0.36751
Deaths	Caribbean	Both	50-69 yea	Sexually tra	Number	1993	18.1399	29.6419	10.9641
Deaths	Caribbean	Both	50-69 yea	Sexually tra	Rate	1993	0.41011	0.67016	0.24788
Deaths	Southern S	Both	50-69 yea	Sexually tra	Number	1999	67.2886	98.6542	49.9526
Deaths	Southern S	Both	50-69 yea	Sexually tra	Rate	1999	1.14824	1.68348	0.85241
Deaths	High-incor	Both	50-69 yea	HIV/AIDS	Number	1993	5345.07	5412.97	5280.56
Deaths	High-incor	Both	50-69 yea	HIV/AIDS	Rate	1993	10.9866	11.1262	10.854
Deaths	Central As	Both	50-69 yea	HIV/AIDS	Number	1990	27.1941	29.0638	24.9845
Deaths	Central As	Both	50-69 yea	HIV/AIDS	Rate	1990	0.31181	0.33325	0.28648
Deaths	Australasia	Both	50-69 yea	HIV/AIDS	Number	1993	104.785	109.427	100.546
Deaths	Australasia	Both	50-69 yea	HIV/AIDS	Rate	1993	2.9381	3.06824	2.81924
Deaths	East Asia	Both	50-69 yea	Sexually tra	Number	1993	363.468	439.5	256.664
Deaths	East Asia	Both	50-69 yea	Sexually tra	Rate	1993	0.21618	0.2614	0.15266
Deaths	South Asia	Both	50-69 yea	HIV/AIDS	Number	1997	2727.76	3450.77	2153.01
Deaths	South Asia	Both	50-69 yea	HIV/AIDS	Rate	1997	2.1594	2.73176	1.7044
Deaths	East Asia	Both	50-69 yea	Sexually tra	Number	1994	343.192	413.404	233.749
Deaths	East Asia	Both	50-69 yea	Sexually tra	Rate	1994	0.20107	0.2422	0.13695
Deaths	Andean La	Both	50-69 yea	Sexually tra	Number	1999	8.92315	12.0321	6.80649
Deaths	Andean La	Both	50-69 yea	Sexually tra	Rate	1999	0.1911	0.25768	0.14577
Deaths	North Afric	Both	50-69 yea	Sexually tra	Number	1997	20.4973	27.2476	15.1023
Deaths	North Afric	Both	50-69 yea	Sexually tra	Rate	1997	0.05549	0.07377	0.04089
Deaths	Oceania	Both	50-69 yea	Sexually tra	Number	1993	1.65319	3.36624	0.73773
Deaths	Oceania	Both	50-69 yea	Sexually tra	Rate	1993	0.28144	0.57307	0.12559
Deaths	Central Eu	Both	50-69 yea	HIV/AIDS	Number	1998	163.288	171.213	153.99
Deaths	Central Eu	Both	50-69 yea	HIV/AIDS	Rate	1998	0.66025	0.6923	0.62266
Deaths	High-incor	Both	50-69 yea	HIV/AIDS	Number	1994	6121.39	6198.34	6042.05
Deaths	High-incor	Both	50-69 yea	HIV/AIDS	Rate	1994	12.428	12.5842	12.2669
Deaths	Eastern Eu	Both	50-69 yea	HIV/AIDS	Number	1992	728.006	741.719	715.124
Deaths	Eastern Eu	Both	50-69 yea	HIV/AIDS	Rate	1992	1.43013	1.45707	1.40483
Deaths	Eastern Su	Both	50-69 yea	Sexually tra	Number	1997	469.16	952.964	181.63
Deaths	Eastern Su	Both	50-69 yea	Sexually tra	Rate	1997	3.02171	6.13773	1.16982
Deaths	Oceania	Both	50-69 yea	HIV/AIDS	Number	2000	140.168	296.821	59.1317
Deaths	Oceania	Both	50-69 yea	HIV/AIDS	Rate	2000	20.4576	43.3212	8.63029
Deaths	Tropical La	Both	50-69 yea	Sexually tra	Number	1993	41.1768	44.5063	38.5679
Deaths	Tropical La	Both	50-69 yea	Sexually tra	Rate	1993	0.23609	0.25518	0.22113
Deaths	Eastern Su	Both	50-69 yea	Sexually tra	Number	1991	466.774	894.943	176.575
Deaths	Eastern Su	Both	50-69 yea	Sexually tra	Rate	1991	3.35304	6.42878	1.26842
Deaths	Western Su	Both	50-69 yea	Sexually tra	Number	1991	63.4101	84.1836	31.0994
Deaths	Western Su	Both	50-69 yea	Sexually tra	Rate	1991	0.39872	0.52934	0.19555
Deaths	Central Su	Both	50-69 yea	HIV/AIDS	Number	1999	9469.19	11440.8	7827.25
Deaths	Central Su	Both	50-69 yea	HIV/AIDS	Rate	1999	185.937	224.651	153.696
Deaths	Eastern Eu	Both	50-69 yea	Sexually tra	Number	1991	231.653	256.104	152.992
Deaths	Eastern Eu	Both	50-69 yea	Sexually tra	Rate	1991	0.4592	0.50767	0.30327
Deaths	Oceania	Both	50-69 yea	HIV/AIDS	Number	1993	6.18138	23.7046	1.48313
Deaths	Oceania	Both	50-69 yea	HIV/AIDS	Rate	1993	1.05233	4.03551	0.25249
Deaths	Andean La	Both	50-69 yea	HIV/AIDS	Number	1992	149.185	387.997	91.9754
Deaths	Andean La	Both	50-69 yea	HIV/AIDS	Rate	1992	4.01012	10.4294	2.47231
Deaths	Australasia	Both	50-69 yea	HIV/AIDS	Number	1995	113.116	118.451	108.556
Deaths	Australasia	Both	50-69 yea	HIV/AIDS	Rate	1995	3.05762	3.2018	2.93435
Deaths	Central Eu	Both	50-69 yea	Sexually tra	Number	1992	17.5155	18.6983	15.0105
Deaths	Central Eu	Both	50-69 yea	Sexually tra	Rate	1992	0.0699	0.07462	0.05991
Deaths	Tropical La	Both	50-69 yea	HIV/AIDS	Number	1992	1057.93	1082.65	1035.07
Deaths	Tropical La	Both	50-69 yea	HIV/AIDS	Rate	1992	6.23078	6.37641	6.09614
Deaths	Caribbean	Both	50-69 yea	Sexually tra	Number	1999	17.9491	28.0084	12.1404
Deaths	Caribbean	Both	50-69 yea	Sexually tra	Rate	1999	0.34731	0.54195	0.23491
Deaths	Southeast	Both	50-69 yea	Sexually tra	Number	1992	88.9605	120.525	65.3128
Deaths	Southeast	Both	50-69 yea	Sexually tra	Rate	1992	0.17996	0.24381	0.13212
Deaths	South Asia	Both	50-69 yea	HIV/AIDS	Number	1993	550.396	986.095	330.898
Deaths	South Asia	Both	50-69 yea	HIV/AIDS	Rate	1993	0.47641	0.85355	0.28642
Deaths	Western Su	Both	50-69 yea	Sexually tra	Number	1999	57.1832	76.9974	32.1529
Deaths	Western Su	Both	50-69 yea	Sexually tra	Rate	1999	0.30533	0.41112	0.17168
Deaths	Southern L	Both	50-69 yea	HIV/AIDS	Number	1990	175.56	180.092	171.266
Deaths	Southern L	Both	50-69 yea	HIV/AIDS	Rate	1990	2.34465	2.40518	2.28731
Deaths	North Afric	Both	50-69 yea	Sexually tra	Number	1992	20.3237	26.7636	15.0178
Deaths	North Afric	Both	50-69 yea	Sexually tra	Rate	1992	0.06178	0.08136	0.04565
Deaths	Western Eu	Both	50-69 yea	Sexually tra	Number	2002	42.6301	52.5669	40.1477
Deaths	Western Eu	Both	50-69 yea	Sexually tra	Rate	2002	0.04712	0.0581	0.04438
Deaths	North Afric	Both	50-69 yea	Sexually tra	Number	1991	20.0665	26.1645	14.9224
Deaths	North Afric	Both	50-69 yea	Sexually tra	Rate	1991	0.06238	0.08134	0.04639
Deaths	Central Eu	Both	50-69 yea	HIV/AIDS	Number	2002	155.861	164.528	148.593
Deaths	Central Eu	Both	50-69 yea	HIV/AIDS	Rate	2002	0.60219	0.63568	0.57411
Deaths	Southeast	Both	50-69 yea	HIV/AIDS	Number	1993	1736.54	2455.97	981.018
Deaths	Southeast	Both	50-69 yea	HIV/AIDS	Rate	1993	3.44212	4.86815	1.94454
Deaths	Western Eu	Both	50-69 yea	HIV/AIDS	Number	1992	2194.75	2234.57	2158.93
Deaths	Western Eu	Both	50-69 yea	HIV/AIDS	Rate	1992	2.63947	2.68736	2.59639
Deaths	Caribbean	Both	50-69 yea	HIV/AIDS	Number	1992	1137.46	1750.64	732.469
Deaths	Caribbean	Both	50-69 yea	HIV/AIDS	Rate	1992	26.3115	40.4956	16.9434
Deaths	Central Su	Both	50-69 yea	Sexually tra	Number	1992	105.175	214.729	24.6451
Deaths	Central Su	Both	50-69 yea	Sexually tra	Rate	1992	2.29331	4.68209	0.53738
Deaths	High-incor	Both	50-69 yea	Sexually tra	Number	1990	24.0102	25.6597	18.8392
Deaths	High-incor	Both	50-69 yea	Sexually tra	Rate	1990	0.07038	0.07522	0.05522
Deaths	High-incor	Both	50-69 yea	HIV/AIDS	Number	1990	49.4429	51.4537	47.4625
Deaths	High-incor	Both	50-69 yea	HIV/AIDS	Rate	1990	0.14494	0.15083	0.13913
Deaths	Central Eu	Both	50-69 yea	Sexually tra	Number	1994	15.2716	16.2359	13.8762
Deaths	Central Eu	Both	50-69 yea	Sexually tra	Rate	1994	0.06169	0.06559	0.05605
Deaths	South Asia	Both	50-69 yea	Sexually tra	Number	1990	962.729	1230.67	677.963
Deaths	South Asia	Both	50-69 yea	Sexually tra	Rate	1990	0.89614	1.14554	0.63107
Deaths	Caribbean	Both	50-69 yea	HIV/AIDS	Number	1993	1478.1	2205.59	959.658
Deaths	Caribbean	Both	50-69 yea	HIV/AIDS	Rate	1993	33.4176	49.865	21.6964
Deaths	Andean La	Both	50-69 yea	HIV/AIDS	Number	1993	165.923	435.333	100.002
Deaths	Andean La	Both	50-69 yea	HIV/AIDS	Rate	1993	4.32316	11.3427	2.60556
Deaths	North Afric	Both	50-69 yea	Sexually tra	Number	1993	20.5085	27.1902	15.1693
Deaths	North Afric	Both	50-69 yea	Sexually tra	Rate	1993	0.06093	0.08078	0.04507
Deaths	North Afric	Both	50-69 yea	Sexually tra	Number	1994	20.6968	27.772	15.4438
Deaths	North Afric	Both	50-69 yea	Sexually tra	Rate	1994	0.0601	0.08065	0.04485
Deaths	High-incor	Both	50-69 yea	HIV/AIDS	Number	2002	4019.5	4064.51	3974.51
Deaths	High-incor	Both	50-69 yea	HIV/AIDS	Rate	2002	6.67334	6.74806	6.59864
Deaths	Andean La	Both	50-69 yea	Sexually tra	Number	2003	9.59023	12.5981	7.01865
Deaths	Andean La	Both	50-69 yea	Sexually tra	Rate	2003	0.17994	0.23638	0.13169
Deaths	Central Eu	Both	50-69 yea	HIV/AIDS	Number	1992	137.074	144.702	128.204
Deaths	Central Eu	Both	50-69 yea	HIV/AIDS	Rate	1992	0.54705	0.57749	0.51165
Deaths	Southern S	Both	50-69 yea	Sexually tra	Number	1990	53.5704	83.3192	33.2589
Deaths	Southern S	Both	50-69 yea	Sexually tra	Rate	1990	1.14007	1.77318	0.70781
Deaths	Western Eu	Both	50-69 yea	HIV/AIDS	Number	1994	2810.24	2852.89	2769.29
Deaths	Western Eu	Both	50-69 yea	HIV/AIDS	Rate	1994	3.3587	3.40968	3.30976
Deaths	Western Eu	Both	50-69 yea	Sexually tra	Number	1992	74.867	81.2014	69.4205
Deaths	Western Eu	Both	50-69 yea	Sexually tra	Rate	1992	0.09004	0.09766	0.08349
Deaths	Central As	Both	50-69 yea	Sexually tra	Number	1990	36.5582	40.8289	27.5015
Deaths	Central As	Both	50-69 yea	Sexually tra	Rate	1990	0.41918	0.46815	0.31534
Deaths	Central As	Both	50-69 yea	Sexually tra	Number	1991	38.0219	42.5976	28.334
Deaths	Central As	Both	50-69 yea	Sexually tra	Rate	1991	0.42887	0.48049	0.3196
Deaths	Southern S	Both	50-69 yea	Sexually tra	Number	2003	61.1696	98.0217	47.6043
Deaths	Southern S	Both	50-69 yea	Sexually tra	Rate	2003	0.94314	1.51134	0.73398
Deaths	Andean La	Both	50-69 yea	Sexually tra	Number	1993	8.34528	12.4976	6.09318
Deaths	Andean La	Both	50-69 yea	Sexually tra	Rate	1993	0.21744	0.32563	0.15876
Deaths	Australasia	Both	50-69 yea	Sexually tra	Number	1993	2.17686	2.63845	1.83668
Deaths	Australasia	Both	50-69 yea	Sexually tra	Rate	1993	0.06104	0.07398	0.0515
Deaths	Western Eu	Both	50-69 yea	Sexually tra	Number	1993	69.2766	75.9885	64.4473
Deaths	Western Eu	Both	50-69 yea	Sexually tra	Rate	1993	0.08313	0.09118	0.07733
Deaths	Central Lat	Both	50-69 yea	Sexually tra	Number	1992	29.7119	35.2235	27.5929
Deaths	Central Lat	Both	50-69 yea	Sexually tra	Rate	1992	0.19366	0.22959	0.17985
Deaths	Western Su	Both	50-69 yea	HIV/AIDS	Number	1993	10576	15367.7	6937.39
Deaths	Western Su	Both	50-69 yea	HIV/AIDS	Rate	1993	63.4794	92.24	41.6395
Deaths	Western Su	Both	50-69 yea	Sexually tra	Number	1993	63.3567	84.0583	31.775
Deaths	Western Su	Both	50-69 yea	Sexually tra	Rate	1993	0.38028	0.50453	0.19072
Deaths	Western Eu	Both	50-69 yea	Sexually tra	Number	1995	61.55	68.5	57.0666
Deaths	Western Eu	Both	50-69 yea	Sexually tra	Rate	1995	0.07341	0.0817	0.06806
Deaths	Western Eu	Both	50-69 yea	HIV/AIDS	Number	2004	1412.45	1437.55	1390.9
Deaths	Western Eu	Both	50-69 yea	HIV/AIDS	Rate	2004	1.5255	1.55261	1.50222
Deaths	North Afric	Both	50-69 yea	HIV/AIDS	Number	1993	189.261	387.737	90.7729
Deaths	North Afric	Both	50-69 yea	HIV/AIDS	Rate	1993	0.56231	1.15199	0.26969
Deaths	High-incor	Both	50-69 yea	Sexually tra	Number	2004	34.5499	36.6036	33.0603
Deaths	High-incor	Both	50-69 yea	Sexually tra	Rate	2004	0.05359	0.05678	0.05128
Deaths	Southeast	Both	50-69 yea	Sexually tra	Number	1995	90.8335	122.459	66.6033
Deaths	Southeast	Both	50-69 yea	Sexually tra	Rate	1995	0.17285	0.23303	0.12674
Deaths	Andean La	Both	50-69 yea	HIV/AIDS	Number	1995	192.36	526.591	116.458
Deaths	Andean La	Both	50-69 yea	HIV/AIDS	Rate	1995	4.70604	12.883	2.84912
Deaths	High-incor	Both	50-69 yea	Sexually tra	Number	1993	41.911	43.9199	39.0902
Deaths	High-incor	Both	50-69 yea	Sexually tra	Rate	1993	0.08615	0.09028	0.08035
Deaths	South Asia	Both	50-69 yea	HIV/AIDS	Number	1991	183.969	473.015	72.6439
Deaths	South Asia	Both	50-69 yea	HIV/AIDS	Rate	1991	0.167	0.42939	0.06594
Deaths	Western Su	Both	50-69 yea	Sexually tra	Number	1992	63.4616	85.551	31.4884
Deaths	Western Su	Both	50-69 yea	Sexually tra	Rate	1992	0.38985	0.52555	0.19344
Deaths	Central Lat	Both	50-69 yea	HIV/AIDS	Number	2004	1920.53	1956.84	1883.08
Deaths	Central Lat	Both	50-69 yea	HIV/AIDS	Rate	2004	8.2001	8.35516	8.04023
Deaths	Oceania	Both	50-69 yea	Sexually tra	Number	1996	1.676	3.57217	0.73586
Deaths	Oceania	Both	50-69 yea	Sexually tra	Rate	1996	0.26952	0.57444	0.11833
Deaths	Central Su	Both	50-69 yea	HIV/AIDS	Number	1993	5678.53	7793.38	3958.17
Deaths	Central Su	Both	50-69 yea	HIV/AIDS	Rate	1993	121.908	167.31	84.9751
Deaths	Eastern Eu	Both	50-69 yea	HIV/AIDS	Number	1993	791.542	804.958	779.145
Deaths	Eastern Eu	Both	50-69 yea	HIV/AIDS	Rate	1993	1.57907	1.60583	1.55434
Deaths	North Afric	Both	50-69 yea	HIV/AIDS	Number	1994	225.71	448.408	110.217
Deaths	North Afric	Both	50-69 yea	HIV/AIDS	Rate	1994	0.65544	1.30213	0.32006
Deaths	Tropical La	Both	50-69 yea	Sexually tra	Number	1995	42.2721	45.4207	39.7494
Deaths	Tropical La	Both	50-69 yea	Sexually tra	Rate	1995	0.22975	0.24686	0.21604
Deaths	Andean La	Both	50-69 yea	Sexually tra	Number	1996	8.1948	11.7163	6.07792
Deaths	Andean La	Both	50-69 yea	Sexually tra	Rate	1996	0.19423	0.2777	0.14406
Deaths	Central Eu	Both	50-69 yea	HIV/AIDS	Number	1994	149.332	157.227	139.756
Deaths	Central Eu	Both	50-69 yea	HIV/AIDS	Rate	1994	0.60325	0.63514	0.56456
Deaths	Australasia	Both	50-69 yea	Sexually tra	Number	1996	1.56102	1.99012	1.31609
Deaths	Australasia	Both	50-69 yea	Sexually tra	Rate	1996	0.0413	0.05265	0.03482
Deaths	Eastern Eu	Both	50-69 yea	Sexually tra	Number	1996	371.079	420.383	173.717
Deaths	Eastern Eu	Both	50-69 yea	Sexually tra	Rate	1996	0.80865	0.9161	0.37856
Deaths	Western Su	Both	50-69 yea	Sexually tra	Number	1996	61.0886	81.8194	32.1831
Deaths	Western Su	Both	50-69 yea	Sexually tra	Rate	1996	0.34548	0.46272	0.18201
Deaths	Australasia	Both	50-69 yea	HIV/AIDS	Number	1996	96.8957	100.974	92.5741
Deaths	Australasia	Both	50-69 yea	HIV/AIDS	Rate	1996	2.56368	2.67159	2.44934
Deaths	Southern S	Both	50-69 yea	Sexually tra	Number	1994	57.8513	85.7465	39.1678
Deaths	Southern S	Both	50-69 yea	Sexually tra	Rate	1994	1.11003	1.64527	0.75154
Deaths	Tropical La	Both	50-69 yea	HIV/AIDS	Number	1995	1677.6	1711.15	1644.42
Deaths	Tropical La	Both	50-69 yea	HIV/AIDS	Rate	1995	9.11783	9.30016	8.93751
Deaths	South Asia	Both	50-69 yea	HIV/AIDS	Number	1995	1272.83	1799.24	913.354
Deaths	South Asia	Both	50-69 yea	HIV/AIDS	Rate	1995	1.05278	1.48818	0.75545
Deaths	Eastern Eu	Both	50-69 yea	HIV/AIDS	Number	1995	865.795	879.848	851.949
Deaths	Eastern Eu	Both	50-69 yea	HIV/AIDS	Rate	1995	1.83733	1.86715	1.80795
Deaths	Central Su	Both	50-69 yea	Sexually tra	Number	1995	106.28	220.106	24.6446
Deaths	Central Su	Both	50-69 yea	Sexually tra	Rate	1995	2.21635	4.59008	0.51394
Deaths	Central As	Both	50-69 yea	HIV/AIDS	Number	1995	46.539	48.42	44.7597
Deaths	Central As	Both	50-69 yea	HIV/AIDS	Rate	1995	0.55093	0.5732	0.52987
Deaths	Western Eu	Both	50-69 yea	HIV/AIDS	Number	1995	2930.3	2974.9	2886.63
Deaths	Western Eu	Both	50-69 yea	HIV/AIDS	Rate	1995	3.49505	3.54824	3.44296
Deaths	Central Lat	Both	50-69 yea	HIV/AIDS	Number	1992	831.929	845.14	819.046
Deaths	Central Lat	Both	50-69 yea	HIV/AIDS	Rate	1992	5.42248	5.50859	5.33851
Deaths	Southeast	Both	50-69 yea	Sexually tra	Number	1996	92.1417	120.987	66.5034
Deaths	Southeast	Both	50-69 yea	Sexually tra	Rate	1996	0.17142	0.22508	0.12372
Deaths	East Asia	Both	50-69 yea	Sexually tra	Number	1997	203.194	290.466	169.913
Deaths	East Asia	Both	50-69 yea	Sexually tra	Rate	1997	0.11144	0.1593	0.09319
Deaths	Central Eu	Both	50-69 yea	Sexually tra	Number	1995	14.0882	15.0027	13.0076
Deaths	Central Eu	Both	50-69 yea	Sexually tra	Rate	1995	0.05731	0.06103	0.05292
Deaths	Western Su	Both	50-69 yea	HIV/AIDS	Number	1995	16526.3	22725.8	11455.3
Deaths	Western Su	Both	50-69 yea	HIV/AIDS	Rate	1995	95.1202	130.802	65.9331
Deaths	Southeast	Both	50-69 yea	HIV/AIDS	Number	1997	5563.46	6140.37	4600.3
Deaths	Southeast	Both	50-69 yea	HIV/AIDS	Rate	1997	10.1443	11.1962	8.38809
Deaths	East Asia	Both	50-69 yea	HIV/AIDS	Number	1993	998.032	1153.08	558.434
Deaths	East Asia	Both	50-69 yea	HIV/AIDS	Rate	1993	0.5936	0.68582	0.33214
Deaths	Tropical La	Both	50-69 yea	Sexually tra	Number	2002	50.3796	53.7962	47.4688
Deaths	Tropical La	Both	50-69 yea	Sexually tra	Rate	2002	0.21814	0.23294	0.20554
Deaths	Central Eu	Both	50-69 yea	HIV/AIDS	Number	1993	174.665	183.749	164.522
Deaths	Central Eu	Both	50-69 yea	HIV/AIDS	Rate	1993	0.70132	0.73779	0.66059
Deaths	North Afric	Both	50-69 yea	Sexually tra	Number	2005	21.1393	28.5354	15.707
Deaths	North Afric	Both	50-69 yea	Sexually tra	Rate	2005	0.04554	0.06148	0.03384
Deaths	Eastern Su	Both	50-69 yea	Sexually tra	Number	2005	397.954	711.396	167.736
Deaths	Eastern Su	Both	50-69 yea	Sexually tra	Rate	2005	2.14412	3.83291	0.90374
Deaths	Caribbean	Both	50-69 yea	HIV/AIDS	Number	1995	2202.1	3070.5	1528.79
Deaths	Caribbean	Both	50-69 yea	HIV/AIDS	Rate	1995	47.4288	66.1324	32.927
Deaths	Southeast	Both	50-69 yea	Sexually tra	Number	1993	89.6287	121.732	66.1259
Deaths	Southeast	Both	50-69 yea	Sexually tra	Rate	1993	0.17766	0.24129	0.13107
Deaths	East Asia	Both	50-69 yea	HIV/AIDS	Number	1994	1105.1	1271.16	809.214
Deaths	East Asia	Both	50-69 yea	HIV/AIDS	Rate	1994	0.64744	0.74474	0.47409
Deaths	South Asia	Both	50-69 yea	Sexually tra	Number	1994	993.907	1229.19	710.552
Deaths	South Asia	Both	50-69 yea	Sexually tra	Rate	1994	0.84064	1.03965	0.60098
Deaths	Central Eu	Both	50-69 yea	HIV/AIDS	Number	1996	149.676	157.326	139.846
Deaths	Central Eu	Both	50-69 yea	HIV/AIDS	Rate	1996	0.61121	0.64244	0.57106
Deaths	Tropical La	Both	50-69 yea	HIV/AIDS	Number	2004	2363.02	2403.05	2319.69
Deaths	Tropical La	Both	50-69 yea	HIV/AIDS	Rate	2004	9.51155	9.67268	9.33714
Deaths	Western Eu	Both	50-69 yea	Sexually tra	Number	1996	56.9693	65.2252	53.1185
Deaths	Western Eu	Both	50-69 yea	Sexually tra	Rate	1996	0.06762	0.07742	0.06305
Deaths	Southern S	Both	50-69 yea	HIV/AIDS	Number	1994	9016.5	15427.8	4344.72
Deaths	Southern S	Both	50-69 yea	HIV/AIDS	Rate	1994	173.005	296.023	83.365
Deaths	Tropical La	Both	50-69 yea	HIV/AIDS	Number	1996	1733.33	1768.28	1697.14
Deaths	Tropical La	Both	50-69 yea	HIV/AIDS	Rate	1996	9.17079	9.35567	8.97933
Deaths	Andean La	Both	50-69 yea	HIV/AIDS	Number	1996	207.862	572.804	129.894
Deaths	Andean La	Both	50-69 yea	HIV/AIDS	Rate	1996	4.92667	13.5764	3.07869
Deaths	Southern L	Both	50-69 yea	HIV/AIDS	Number	1994	290.071	296.801	283.219
Deaths	Southern L	Both	50-69 yea	HIV/AIDS	Rate	1994	3.69314	3.77883	3.60591
Deaths	Andean La	Both	50-69 yea	Sexually tra	Number	1997	8.32726	11.6625	6.36813
Deaths	Andean La	Both	50-69 yea	Sexually tra	Rate	1997	0.19079	0.2672	0.1459
Deaths	High-incor	Both	50-69 yea	HIV/AIDS	Number	1998	3038.9	3072.97	3006.21
Deaths	High-incor	Both	50-69 yea	HIV/AIDS	Rate	1998	5.68051	5.7442	5.61939
Deaths	Caribbean	Both	50-69 yea	Sexually tra	Number	2003	19.6463	30.7292	13.6408
Deaths	Caribbean	Both	50-69 yea	Sexually tra	Rate	2003	0.34268	0.53599	0.23793
Deaths	Southern S	Both	50-69 yea	Sexually tra	Number	1996	56.8324	81.0406	40.9922
Deaths	Southern S	Both	50-69 yea	Sexually tra	Rate	1996	1.03808	1.48026	0.74875
Deaths	Central As	Both	50-69 yea	Sexually tra	Number	1995	44.9234	50.5435	31.3118
Deaths	Central As	Both	50-69 yea	Sexually tra	Rate	1995	0.5318	0.59834	0.37067
Deaths	Central Lat	Both	50-69 yea	HIV/AIDS	Number	1994	1066.95	1085.2	1050.73
Deaths	Central Lat	Both	50-69 yea	HIV/AIDS	Rate	1994	6.51899	6.6305	6.4199
Deaths	North Afric	Both	50-69 yea	Sexually tra	Number	1995	20.6571	27.8349	15.3322
Deaths	North Afric	Both	50-69 yea	Sexually tra	Rate	1995	0.0586	0.07897	0.0435
Deaths	High-incor	Both	50-69 yea	Sexually tra	Number	1994	40.4829	42.4331	37.9296
Deaths	High-incor	Both	50-69 yea	Sexually tra	Rate	1994	0.08219	0.08615	0.07701
Deaths	East Asia	Both	50-69 yea	Sexually tra	Number	1996	234.132	315.587	194.909
Deaths	East Asia	Both	50-69 yea	Sexually tra	Rate	1996	0.13172	0.17755	0.10966
Deaths	Central Eu	Both	50-69 yea	HIV/AIDS	Number	1995	158.381	166.338	148.631
Deaths	Central Eu	Both	50-69 yea	HIV/AIDS	Rate	1995	0.64431	0.67668	0.60464
Deaths	South Asia	Both	50-69 yea	HIV/AIDS	Number	1994	867.015	1343.4	586.083
Deaths	South Asia	Both	50-69 yea	HIV/AIDS	Rate	1994	0.73332	1.13625	0.49571
Deaths	Caribbean	Both	50-69 yea	HIV/AIDS	Number	1996	2465.71	3359.44	1758.78
Deaths	Caribbean	Both	50-69 yea	HIV/AIDS	Rate	1996	51.7525	70.5108	36.9147
Deaths	High-incor	Both	50-69 yea	HIV/AIDS	Number	1996	5028.75	5091.92	4966.23
Deaths	High-incor	Both	50-69 yea	HIV/AIDS	Rate	1996	9.9735	10.0988	9.84949
Deaths	Central Lat	Both	50-69 yea	HIV/AIDS	Number	1995	1094.31	1111	1078.76
Deaths	Central Lat	Both	50-69 yea	HIV/AIDS	Rate	1995	6.47285	6.57157	6.38088
Deaths	Eastern Eu	Both	50-69 yea	HIV/AIDS	Number	1996	864.005	878.839	848.831
Deaths	Eastern Eu	Both	50-69 yea	HIV/AIDS	Rate	1996	1.88284	1.91516	1.84977
Deaths	Southern L	Both	50-69 yea	Sexually tra	Number	1998	14.2118	15.9918	12.6016
Deaths	Southern L	Both	50-69 yea	Sexually tra	Rate	1998	0.17205	0.1936	0.15255
Deaths	Caribbean	Both	50-69 yea	Sexually tra	Number	1996	17.1429	28.6146	10.6102
Deaths	Caribbean	Both	50-69 yea	Sexually tra	Rate	1996	0.35981	0.60059	0.22269
Deaths	Central Lat	Both	50-69 yea	HIV/AIDS	Number	1997	1253.09	1271.57	1232.63
Deaths	Central Lat	Both	50-69 yea	HIV/AIDS	Rate	1997	6.89951	7.00127	6.78684
Deaths	Southern L	Both	50-69 yea	Sexually tra	Number	1995	14.7864	16.6417	13.1071
Deaths	Southern L	Both	50-69 yea	Sexually tra	Rate	1995	0.18599	0.20933	0.16487
Deaths	Central As	Both	50-69 yea	HIV/AIDS	Number	1996	51.9868	54.2341	50.0118
Deaths	Central As	Both	50-69 yea	HIV/AIDS	Rate	1996	0.63037	0.65762	0.60642
Deaths	Western Eu	Both	50-69 yea	HIV/AIDS	Number	1997	1623.75	1647.91	1598.91
Deaths	Western Eu	Both	50-69 yea	HIV/AIDS	Rate	1997	1.90578	1.93414	1.87662
Deaths	Southern S	Both	50-69 yea	Sexually tra	Number	1998	67.3356	96.4464	49.8719
Deaths	Southern S	Both	50-69 yea	Sexually tra	Rate	1998	1.17438	1.68209	0.8698
Deaths	Australasia	Both	50-69 yea	Sexually tra	Number	1994	2.06656	2.47924	1.74157
Deaths	Australasia	Both	50-69 yea	Sexually tra	Rate	1994	0.05693	0.0683	0.04798
Deaths	Eastern Su	Both	50-69 yea	Sexually tra	Number	1999	457.452	915.363	177.84
Deaths	Eastern Su	Both	50-69 yea	Sexually tra	Rate	1999	2.839	5.68085	1.1037

Deaths	Tropical La	Both	50-69 yea	Sexually tr	Rate	1996	0.23082	0.24878	0.21711
Deaths	Eastern Su	Both	50-69 yea	HIV/AIDS	Number	1994	46923.6	61084	32918.1
Deaths	Eastern Su	Both	50-69 yea	HIV/AIDS	Rate	1994	322.221	419.46	226.046
Deaths	High-incor	Both	50-69 yea	HIV/AIDS	Number	1994	70.2897	72.602	68.1626
Deaths	High-incor	Both	50-69 yea	HIV/AIDS	Rate	1994	0.18623	0.19236	0.18059
Deaths	Central As	Both	50-69 yea	HIV/AIDS	Number	1994	40.5289	42.2417	38.7557
Deaths	Central As	Both	50-69 yea	HIV/AIDS	Rate	1994	0.46751	0.48727	0.44705
Deaths	Western S	Both	50-69 yea	HIV/AIDS	Number	1997	23036.6	29752.1	16939.4
Deaths	Western S	Both	50-69 yea	HIV/AIDS	Rate	1997	127.796	165.051	93.9715
Deaths	East Asia	Both	50-69 yea	Sexually tr	Number	1998	188.527	272.587	157.28
Deaths	East Asia	Both	50-69 yea	Sexually tr	Rate	1998	0.10087	0.14585	0.08416
Deaths	Central Lat	Both	50-69 yea	Sexually tr	Number	1994	27.4268	32.6479	25.7699
Deaths	Central Lat	Both	50-69 yea	Sexually tr	Rate	1994	0.16758	0.19948	0.15745
Deaths	Andean La	Both	50-69 yea	Sexually tr	Number	1998	8.44913	11.4681	6.47439
Deaths	Andean La	Both	50-69 yea	Sexually tr	Rate	1998	0.18717	0.25405	0.14343
Deaths	High-incor	Both	50-69 yea	Sexually tr	Number	1994	18.9468	20.3273	17.7567
Deaths	High-incor	Both	50-69 yea	Sexually tr	Rate	1994	0.0502	0.05386	0.04705
Deaths	Eastern Eu	Both	50-69 yea	Sexually tr	Number	1998	316.277	363.602	143.8
Deaths	Eastern Eu	Both	50-69 yea	Sexually tr	Rate	1998	0.70435	0.80974	0.32024
Deaths	Caribbean	Both	50-69 yea	HIV/AIDS	Number	1998	2763.67	3597.26	2062.81
Deaths	Caribbean	Both	50-69 yea	HIV/AIDS	Rate	1998	54.9604	71.5379	41.0226
Deaths	High-incor	Both	50-69 yea	Sexually tr	Number	1997	35.6346	37.3555	33.7359
Deaths	High-incor	Both	50-69 yea	Sexually tr	Rate	1997	0.06861	0.07193	0.06496
Deaths	Australasia	Both	50-69 yea	Sexually tr	Number	1999	1.45527	1.89212	1.21509
Deaths	Australasia	Both	50-69 yea	Sexually tr	Rate	1999	0.03506	0.04559	0.02927
Deaths	Southern L	Both	50-69 yea	Sexually tr	Number	2005	12.5981	14.1307	11.1701
Deaths	Southern L	Both	50-69 yea	Sexually tr	Rate	2005	0.13422	0.15055	0.119
Deaths	Central Lat	Both	50-69 yea	HIV/AIDS	Number	1996	1149.47	1166.16	1133.37
Deaths	Central Lat	Both	50-69 yea	HIV/AIDS	Rate	1996	6.56503	6.66034	6.47305
Deaths	East Asia	Both	50-69 yea	Sexually tr	Number	1999	187.05	263.937	158.339
Deaths	East Asia	Both	50-69 yea	Sexually tr	Rate	1999	0.09661	0.13633	0.08179
Deaths	Southern S	Both	50-69 yea	Sexually tr	Number	2000	67.2811	99.7457	50.983
Deaths	Southern S	Both	50-69 yea	Sexually tr	Rate	2000	1.12572	1.6689	0.85303
Deaths	Southern L	Both	50-69 yea	HIV/AIDS	Number	1998	244.103	249.192	238.669
Deaths	Southern L	Both	50-69 yea	HIV/AIDS	Rate	1998	2.9551	3.01671	2.88932
Deaths	Central Su	Both	50-69 yea	Sexually tr	Number	1996	107.181	223.425	25.4021
Deaths	Central Su	Both	50-69 yea	Sexually tr	Rate	1996	2.20266	4.59159	0.52204
Deaths	East Asia	Both	50-69 yea	HIV/AIDS	Number	1997	1489.31	1795.21	1341.16
Deaths	East Asia	Both	50-69 yea	HIV/AIDS	Rate	1997	0.81678	0.98454	0.73553
Deaths	Oceania	Both	50-69 yea	HIV/AIDS	Number	1997	41.2284	92.1306	17.3723
Deaths	Oceania	Both	50-69 yea	HIV/AIDS	Rate	1997	6.43239	14.3741	2.71039
Deaths	Tropical La	Both	50-69 yea	Sexually tr	Number	1997	45.1477	48.3441	42.5251
Deaths	Tropical La	Both	50-69 yea	Sexually tr	Rate	1997	0.23152	0.24791	0.21807
Deaths	Eastern Su	Both	50-69 yea	HIV/AIDS	Number	1997	67984.6	82658	52028
Deaths	Eastern Su	Both	50-69 yea	HIV/AIDS	Rate	1997	437.867	532.373	335.095
Deaths	High-incor	Both	50-69 yea	HIV/AIDS	Number	1997	3255.88	3292.87	3220.25
Deaths	High-incor	Both	50-69 yea	HIV/AIDS	Rate	1997	6.26918	6.3404	6.20057
Deaths	Eastern Eu	Both	50-69 yea	Sexually tr	Number	1994	373.441	419.382	194.958
Deaths	Eastern Eu	Both	50-69 yea	Sexually tr	Rate	1994	0.76676	0.86109	0.40029
Deaths	South Asia	Both	50-69 yea	Sexually tr	Number	1996	1026.4	1253.84	727.557
Deaths	South Asia	Both	50-69 yea	Sexually tr	Rate	1996	0.83097	1.01511	0.58903
Deaths	Eastern Su	Both	50-69 yea	HIV/AIDS	Number	1995	54416.7	68909.1	39207.9
Deaths	Eastern Su	Both	50-69 yea	HIV/AIDS	Rate	1995	365.731	463.134	263.514
Deaths	Oceania	Both	50-69 yea	HIV/AIDS	Number	1994	9.59187	30.0548	3.28658
Deaths	Oceania	Both	50-69 yea	HIV/AIDS	Rate	1994	1.60564	5.03106	0.55016
Deaths	Eastern Eu	Both	50-69 yea	HIV/AIDS	Number	1999	992.233	1010.4	973.216
Deaths	Eastern Eu	Both	50-69 yea	HIV/AIDS	Rate	1999	2.22144	2.26211	2.17886
Deaths	Western E	Both	50-69 yea	HIV/AIDS	Number	2001	1266.91	1286.16	1246.67
Deaths	Western E	Both	50-69 yea	HIV/AIDS	Rate	2001	1.4167	1.43823	1.39407
Deaths	Australasia	Both	50-69 yea	HIV/AIDS	Number	1997	63.4376	65.9363	60.8104
Deaths	Australasia	Both	50-69 yea	HIV/AIDS	Rate	1997	1.62197	1.68586	1.5548
Deaths	Central Su	Both	50-69 yea	HIV/AIDS	Number	1997	8681.32	10776	6798.51
Deaths	Central Su	Both	50-69 yea	HIV/AIDS	Rate	1997	175.837	218.264	137.701
Deaths	High-incor	Both	50-69 yea	HIV/AIDS	Number	1995	113.775	116.391	111.265
Deaths	High-incor	Both	50-69 yea	HIV/AIDS	Rate	1995	0.29881	0.30568	0.29222
Deaths	Central Lat	Both	50-69 yea	Sexually tr	Number	1996	26.0223	31.4681	24.39
Deaths	Central Lat	Both	50-69 yea	Sexually tr	Rate	1996	0.14862	0.17973	0.1393
Deaths	Andean La	Both	50-69 yea	Sexually tr	Number	2000	9.20391	12.4305	7.01089
Deaths	Andean La	Both	50-69 yea	Sexually tr	Rate	2000	0.19089	0.25781	0.14541
Deaths	Oceania	Both	50-69 yea	Sexually tr	Number	2000	1.80668	3.83169	0.77519
Deaths	Oceania	Both	50-69 yea	Sexually tr	Rate	2000	0.26369	0.55924	0.11314
Deaths	South Asia	Both	50-69 yea	HIV/AIDS	Number	2005	15815.2	17999.7	14049.5
Deaths	South Asia	Both	50-69 yea	HIV/AIDS	Rate	2005	10.1807	11.587	9.04407
Deaths	Australasia	Both	50-69 yea	Sexually tr	Number	2005	1.4096	1.84585	1.19402
Deaths	Australasia	Both	50-69 yea	Sexually tr	Rate	2005	0.02822	0.03696	0.02391
Deaths	Eastern Eu	Both	50-69 yea	HIV/AIDS	Number	2005	1528.89	1561.49	1499.42
Deaths	Eastern Eu	Both	50-69 yea	HIV/AIDS	Rate	2005	3.24468	3.31386	3.18213
Deaths	Southern L	Both	50-69 yea	Sexually tr	Number	1997	14.5241	16.3907	12.8517
Deaths	Southern L	Both	50-69 yea	Sexually tr	Rate	1997	0.17827	0.20118	0.15774
Deaths	East Asia	Both	50-69 yea	HIV/AIDS	Number	1996	1354.3	1623.18	1212.02
Deaths	East Asia	Both	50-69 yea	HIV/AIDS	Rate	1996	0.76192	0.91319	0.68188
Deaths	Oceania	Both	50-69 yea	Sexually tr	Number	2010	2.56855	5.88409	1.07703
Deaths	Oceania	Both	50-69 yea	Sexually tr	Rate	2010	0.2628	0.60203	0.1102
Deaths	Tropical La	Both	50-69 yea	HIV/AIDS	Number	2002	2080.56	2118.17	2043.44
Deaths	Tropical La	Both	50-69 yea	HIV/AIDS	Rate	2002	9.00874	9.17157	8.848
Deaths	High-incor	Both	50-69 yea	HIV/AIDS	Number	1995	6399.46	6481.03	6327.28
Deaths	High-incor	Both	50-69 yea	HIV/AIDS	Rate	1995	12.8369	13.0005	12.6921
Deaths	Tropical La	Both	50-69 yea	HIV/AIDS	Number	1997	1575.77	1607.31	1545.46
Deaths	Tropical La	Both	50-69 yea	HIV/AIDS	Rate	1997	8.08052	8.24225	7.92507
Deaths	Central Eu	Both	50-69 yea	HIV/AIDS	Number	2010	157.057	194.579	142.843
Deaths	Central Eu	Both	50-69 yea	HIV/AIDS	Rate	2010	0.54234	0.67191	0.49326
Deaths	Central As	Both	50-69 yea	HIV/AIDS	Number	2010	108.325	117.264	101.165
Deaths	Central As	Both	50-69 yea	HIV/AIDS	Rate	2010	1.05137	1.13812	0.98187
Deaths	Southeast	Both	50-69 yea	HIV/AIDS	Number	1999	7381.77	7976.49	6689.25
Deaths	Southeast	Both	50-69 yea	HIV/AIDS	Rate	1999	12.8745	13.9117	11.6667
Deaths	Central Lat	Both	50-69 yea	HIV/AIDS	Number	2000	1448.18	1469.19	1426.55
Deaths	Central Lat	Both	50-69 yea	HIV/AIDS	Rate	2000	7.18499	7.28924	7.0777
Deaths	Oceania	Both	50-69 yea	Sexually tr	Number	2006	2.2722	4.80759	0.95158
Deaths	Oceania	Both	50-69 yea	Sexually tr	Rate	2006	0.26988	0.57101	0.11302
Deaths	Tropical La	Both	50-69 yea	Sexually tr	Number	2006	51.1803	54.121	48.1633
Deaths	Tropical La	Both	50-69 yea	Sexually tr	Rate	2006	0.19131	0.2023	0.18003
Deaths	Central Lat	Both	50-69 yea	Sexually tr	Number	2010	42.4426	44.9929	39.7234
Deaths	Central Lat	Both	50-69 yea	Sexually tr	Rate	2010	0.14408	0.15274	0.13485
Deaths	North Afric	Both	50-69 yea	HIV/AIDS	Number	1996	312.47	606.658	157.432
Deaths	North Afric	Both	50-69 yea	HIV/AIDS	Rate	1996	0.86606	1.68146	0.43635
Deaths	Southern L	Both	50-69 yea	HIV/AIDS	Number	2001	351.452	359.047	343.807
Deaths	Southern L	Both	50-69 yea	HIV/AIDS	Rate	2001	4.05732	4.14499	3.96906
Deaths	Western E	Both	50-69 yea	Sexually tr	Number	1997	53.5179	62.251	50.0958
Deaths	Western E	Both	50-69 yea	Sexually tr	Rate	1997	0.06281	0.07306	0.0588
Deaths	Western S	Both	50-69 yea	Sexually tr	Number	1994	63.4434	84.0943	32.2734
Deaths	Western S	Both	50-69 yea	Sexually tr	Rate	1994	0.37152	0.49245	0.18899
Deaths	North Afric	Both	50-69 yea	Sexually tr	Number	1996	20.563	27.5809	15.1945
Deaths	North Afric	Both	50-69 yea	Sexually tr	Rate	1996	0.05699	0.07645	0.04211
Deaths	Central Eu	Both	50-69 yea	HIV/AIDS	Number	1997	160.004	167.508	150.308
Deaths	Central Eu	Both	50-69 yea	HIV/AIDS	Rate	1997	0.65119	0.68173	0.61172
Deaths	High-incor	Both	50-69 yea	Sexually tr	Number	2000	20.1894	22.0643	18.9749
Deaths	High-incor	Both	50-69 yea	Sexually tr	Rate	2000	0.04749	0.0519	0.04463
Deaths	High-incor	Both	50-69 yea	Sexually tr	Number	2008	17.7522	18.9121	16.6397
Deaths	High-incor	Both	50-69 yea	Sexually tr	Rate	2008	0.03873	0.04126	0.03631
Deaths	High-incor	Both	50-69 yea	HIV/AIDS	Number	2006	4576.78	4622.8	4528.86
Deaths	High-incor	Both	50-69 yea	HIV/AIDS	Rate	2006	6.62039	6.68696	6.55107
Deaths	Oceania	Both	50-69 yea	Sexually tr	Number	1995	1.67464	3.57429	0.72534
Deaths	Oceania	Both	50-69 yea	Sexually tr	Rate	1995	0.27507	0.58709	0.11914
Deaths	Australasia	Both	50-69 yea	HIV/AIDS	Number	1994	104.864	109.465	100.532
Deaths	Australasia	Both	50-69 yea	HIV/AIDS	Rate	1994	2.8887	3.01544	2.76935
Deaths	Western E	Both	50-69 yea	HIV/AIDS	Number	2002	1322.75	1344.09	1301.76
Deaths	Western E	Both	50-69 yea	HIV/AIDS	Rate	2002	1.46207	1.48565	1.43887
Deaths	Central Eu	Both	50-69 yea	Sexually tr	Number	2002	10.7629	11.5576	9.89112
Deaths	Central Eu	Both	50-69 yea	Sexually tr	Rate	2002	0.04158	0.04465	0.03822
Deaths	High-incor	Both	50-69 yea	Sexually tr	Number	1995	18.5974	19.9912	17.4224
Deaths	High-incor	Both	50-69 yea	Sexually tr	Rate	1995	0.04884	0.0525	0.04576
Deaths	Southern S	Both	50-69 yea	Sexually tr	Number	2001	63.6289	97.0953	49.171
Deaths	Southern S	Both	50-69 yea	Sexually tr	Rate	2001	1.03522	1.5797	0.79999
Deaths	Western E	Both	50-69 yea	HIV/AIDS	Number	2008	1442.26	1464.89	1418.73
Deaths	Western E	Both	50-69 yea	HIV/AIDS	Rate	2008	1.48566	1.50896	1.46141
Deaths	High-incor	Both	50-69 yea	Sexually tr	Number	1995	38.8856	40.7465	36.5839
Deaths	High-incor	Both	50-69 yea	Sexually tr	Rate	1995	0.078	0.08173	0.07338
Deaths	Andean La	Both	50-69 yea	Sexually tr	Number	2002	9.3965	12.2008	7.05146
Deaths	Andean La	Both	50-69 yea	Sexually tr	Rate	2002	0.18231	0.23672	0.13682
Deaths	Eastern Eu	Both	50-69 yea	HIV/AIDS	Number	1994	906.578	921.313	892.218
Deaths	Eastern Eu	Both	50-69 yea	HIV/AIDS	Rate	1994	1.86142	1.89167	1.83193
Deaths	Central Eu	Both	50-69 yea	Sexually tr	Number	1997	13.4024	14.2921	12.4044
Deaths	Central Eu	Both	50-69 yea	Sexually tr	Rate	1997	0.05455	0.05817	0.05048
Deaths	Andean La	Both	50-69 yea	HIV/AIDS	Number	1994	186.456	484.696	114.252
Deaths	Andean La	Both	50-69 yea	HIV/AIDS	Rate	1994	4.71002	12.2438	2.88608
Deaths	North Afric	Both	50-69 yea	Sexually tr	Number	1998	20.3686	27.1595	15.0827
Deaths	North Afric	Both	50-69 yea	Sexually tr	Rate	1998	0.05376	0.07168	0.03981
Deaths	Eastern Eu	Both	50-69 yea	Sexually tr	Number	1997	347.042	393.389	157.077
Deaths	Eastern Eu	Both	50-69 yea	Sexually tr	Rate	1997	0.76529	0.86749	0.34638
Deaths	Central As	Both	50-69 yea	Sexually tr	Number	1998	41.8401	47.1022	29.3984
Deaths	Central As	Both	50-69 yea	Sexually tr	Rate	1998	0.52232	0.58801	0.367
Deaths	High-incor	Both	50-69 yea	HIV/AIDS	Number	1996	117.882	120.698	115.232
Deaths	High-incor	Both	50-69 yea	HIV/AIDS	Rate	1996	0.30799	0.31535	0.30107
Deaths	East Asia	Both	50-69 yea	Sexually tr	Number	2003	225.706	277.989	191.138
Deaths	East Asia	Both	50-69 yea	Sexually tr	Rate	2003	0.10161	0.12515	0.08605
Deaths	Andean La	Both	50-69 yea	Sexually tr	Number	2011	10.1236	13.1906	7.13194
Deaths	Andean La	Both	50-69 yea	Sexually tr	Rate	2011	0.14414	0.18781	0.10155
Deaths	Caribbean	Both	50-69 yea	Sexually tr	Number	1997	17.3836	28.7757	11.0646
Deaths	Caribbean	Both	50-69 yea	Sexually tr	Rate	1997	0.35524	0.58804	0.22611
Deaths	Caribbean	Both	50-69 yea	Sexually tr	Number	1998	17.3259	28.2565	11.2691
Deaths	Caribbean	Both	50-69 yea	Sexually tr	Rate	1998	0.34455	0.56193	0.22411
Deaths	North Afric	Both	50-69 yea	HIV/AIDS	Number	1997	361.019	683.569	183.895
Deaths	North Afric	Both	50-69 yea	HIV/AIDS	Rate	1997	0.9774	1.85064	0.49786
Deaths	High-incor	Both	50-69 yea	Sexually tr	Number	1998	20.1711	21.9658	18.9977
Deaths	High-incor	Both	50-69 yea	Sexually tr	Rate	1998	0.04977	0.05419	0.04687
Deaths	High-incor	Both	50-69 yea	Sexually tr	Number	1998	34.3935	36.0413	32.8083
Deaths	High-incor	Both	50-69 yea	Sexually tr	Rate	1998	0.06429	0.06737	0.06133
Deaths	Eastern Su	Both	50-69 yea	Sexually tr	Number	1995	471.321	921.222	182.411
Deaths	Eastern Su	Both	50-69 yea	Sexually tr	Rate	1995	3.16772	6.19147	1.22597
Deaths	Western S	Both	50-69 yea	Sexually tr	Number	1997	59.9924	79.864	32.1452
Deaths	Western S	Both	50-69 yea	Sexually tr	Rate	1997	0.33281	0.44305	0.17833
Deaths	North Afric	Both	50-69 yea	Sexually tr	Number	1999	20.0233	26.5538	14.7957
Deaths	North Afric	Both	50-69 yea	Sexually tr	Rate	1999	0.05159	0.06841	0.03812
Deaths	Southern S	Both	50-69 yea	HIV/AIDS	Number	1995	12710.7	20941	6546.37
Deaths	Southern S	Both	50-69 yea	HIV/AIDS	Rate	1995	238.371	392.717	122.768
Deaths	Southern S	Both	50-69 yea	Sexually tr	Number	2011	58.021	91.8004	46.3351
Deaths	Southern S	Both	50-69 yea	Sexually tr	Rate	2011	0.72129	1.14122	0.57602
Deaths	Central As	Both	50-69 yea	HIV/AIDS	Number	2001	64.2297	66.6631	61.7748
Deaths	Central As	Both	50-69 yea	HIV/AIDS	Rate	2001	0.80774	0.83834	0.77687
Deaths	Central Eu	Both	50-69 yea	HIV/AIDS	Number	2001	150.536	157.343	142.25
Deaths	Central Eu	Both	50-69 yea	HIV/AIDS	Rate	2001	0.58956	0.61622	0.55711
Deaths	Oceania	Both	50-69 yea	HIV/AIDS	Number	1995	16.0587	43.1648	6.5556
Deaths	Oceania	Both	50-69 yea	HIV/AIDS	Rate	1995	2.63772	7.09003	1.07679
Deaths	Eastern Eu	Both	50-69 yea	HIV/AIDS	Number	2000	1062.63	1081.89	1044.53
Deaths	Eastern Eu	Both	50-69 yea	HIV/AIDS	Rate	2000	2.37124	2.41423	2.33085
Deaths	High-incor	Both	50-69 yea	Sexually tr	Number	2002	18.5419	20.5716	17.4205
Deaths	High-incor	Both	50-69 yea	Sexually tr	Rate	2002	0.04239	0.04703	0.03983
Deaths	Southeast	Both	50-69 yea	Sexually tr	Number	1994	90.2444	120.881	66.8428
Deaths	Southeast	Both	50-69 yea	Sexually tr	Rate	1994	0.17529	0.2348	0.12983
Deaths	Southern S	Both	50-69 yea	HIV/AIDS	Number	1996	17298.8	26374	9533.74
Deaths	Southern S	Both	50-69 yea	HIV/AIDS	Rate	1996	315.974	481.739	174.14
Deaths	Andean La	Both	50-69 yea	Sexually tr	Number	2001	9.10455	11.9418	6.87325
Deaths	Andean La	Both	50-69 yea	Sexually tr	Rate	2001	0.1828	0.23976	0.138
Deaths	Central Su	Both	50-69 yea	Sexually tr	Number	2002	97.1178	217.988	24.369
Deaths	Central Su	Both	50-69 yea	Sexually tr	Rate	2002	1.78223	4.00036	0.4472
Deaths	South Asia	Both	50-69 yea	Sexually tr	Number	2002	999.993	1205.84	733.236
Deaths	South Asia	Both	50-69 yea	Sexually tr	Rate	2002	0.70363	0.84848	0.51593
Deaths	Southern L	Both	50-69 yea	HIV/AIDS	Number	2002	351.151	358.843	343.579
Deaths	Southern L	Both	50-69 yea	HIV/AIDS	Rate	2002	3.98286	4.0701	3.89697
Deaths	Central Lat	Both	50-69 yea	Sexually tr	Number	1998	26.1111	30.9161	24.4271
Deaths	Central Lat	Both	50-69 yea	Sexually tr	Rate	1998	0.13863	0.16414	0.12969
Deaths	North Afric	Both	50-69 yea	HIV/AIDS	Number	1999	467.157	861.125	248.079
Deaths	North Afric	Both	50-69 yea	HIV/AIDS	Rate	1999	1.20354	2.21853	0.63913
Deaths	East Asia	Both	50-69 yea	HIV/AIDS	Number	2000	2064.43	2571.54	1768.05
Deaths	East Asia	Both	50-69 yea	HIV/AIDS	Rate	2000	1.04013	1.29563	0.8908
Deaths	Australasia	Both	50-69 yea	Sexually tr	Number	2009	1.28181	1.64147	1.0792
Deaths	Australasia	Both	50-69 yea	Sexually tr	Rate	2009	0.02297	0.02941	0.01934
Deaths	Caribbean	Both	50-69 yea	Sexually tr	Number	2001	19.1095	30.3208	13.3637
Deaths	Caribbean	Both	50-69 yea	Sexually tr	Rate	2001	0.35104	0.55699	0.24549
Deaths	Eastern Eu	Both	50-69 yea	HIV/AIDS	Number	1997	904.167	921.528	887.832
Deaths	Eastern Eu	Both	50-69 yea	HIV/AIDS	Rate	1997	1.99384	2.03213	1.95782
Deaths	Andean La	Both	50-69 yea	HIV/AIDS	Number	2001	354.804	662.763	259.215
Deaths	Andean La	Both	50-69 yea	HIV/AIDS	Rate	2001	7.12357	13.3066	5.20437
Deaths	North Afric	Both	50-69 yea	Sexually tr	Number	2001	20.4633	27.1295	15.1701
Deaths	North Afric	Both	50-69 yea	Sexually tr	Rate	2001	0.05016	0.0665	0.03719
Deaths	Western E	Both	50-69 yea	Sexually tr	Number	2001	43.6601	53.999	41.2278
Deaths	Western E	Both	50-69 yea	Sexually tr	Rate	2001	0.04882	0.06038	0.0461
Deaths	Southern S	Both	50-69 yea	Sexually tr	Number	2002	62.068	97.4324	47.8165
Deaths	Southern S	Both	50-69 yea	Sexually tr	Rate	2002	0.98275	1.5427	0.7571
Deaths	Central As	Both	50-69 yea	HIV/AIDS	Number	2002	64.9205	67.4957	62.4164
Deaths	Central As	Both	50-69 yea	HIV/AIDS	Rate	2002	0.8015	0.83329	0.77058
Deaths	Andean La	Both	50-69 yea	HIV/AIDS	Number	1997	238.659	623.065	158.088
Deaths	Andean La	Both	50-69 yea	HIV/AIDS	Rate	1997	5.46799	14.2753	3.62201
Deaths	Western S	Both	50-69 yea	HIV/AIDS	Number	2002	36413.4	42553.2	30576.8
Deaths	Western S	Both	50-69 yea	HIV/AIDS	Rate	2002	179.99	210.338	151.14
Deaths	Australasia	Both	50-69 yea	HIV/AIDS	Number	2001	48.8863	50.9707	46.9745
Deaths	Australasia	Both	50-69 yea	HIV/AIDS	Rate	2001	1.1088	1.15608	1.06544
Deaths	Central Lat	Both	50-69 yea	Sexually tr	Number	1997	25.8176	30.5687	24.1916
Deaths	Central Lat	Both	50-69 yea	Sexually tr	Rate	1997	0.14215	0.16831	0.1332
Deaths	High-incor	Both	50-69 yea	HIV/AIDS	Number	1997	133.162	136.041	130.455
Deaths	High-incor	Both	50-69 yea	HIV/AIDS	Rate	1997	0.33822	0.34553	0.33134
Deaths	Southern L	Both	50-69 yea	HIV/AIDS	Number	1997	264.398	270.363	257.981
Deaths	Southern L	Both	50-69 yea	HIV/AIDS	Rate	1997	3.24519	3.31841	3.16644
Deaths	Western E	Both	50-69 yea	HIV/AIDS	Number	2003	1416.93	1439.53	1396.47
Deaths	Western E	Both	50-69 yea	HIV/AIDS	Rate	2003	1.54725	1.57193	1.5249
Deaths	High-incor	Both	50-69 yea	Sexually tr	Number	2012	38.6473	40.7568	36.9967
Deaths	High-incor	Both	50-69 yea	Sexually tr	Rate	2012	0.04639	0.04892	0.04441
Deaths	Tropical La	Both	50-69 yea	Sexually tr	Number	1999	47.7239	51.1572	44.9934
Deaths	Tropical La	Both	50-69 yea	Sexually tr	Rate	1999	0.22873	0.24519	0.21565
Deaths	Central Su	Both	50-69 yea	HIV/AIDS	Number	2002	10428.6	12173.7	8878.11
Deaths	Central Su	Both	50-69 yea	HIV/AIDS	Rate	2002	191.378	223.402	162.924
Deaths	High-incor	Both	50-69 yea	HIV/AIDS	Number	1999	3668.51	3712.61	3628.19
Deaths	High-incor	Both	50-69 yea	HIV/AIDS	Rate	1999	6.67085	6.75104	6.59753
Deaths	Eastern Su	Both	50-69 yea	HIV/AIDS	Number	1996	61530.8	76319.7	45619.9
Deaths	Eastern Su	Both	50-69 yea	HIV/AIDS	Rate	1996	404.408	501.608	299.835
Deaths	High-incor	Both	50-69 yea	Sexually tr	Number	1999	20.4227	22.1564	19.225
Deaths	High-incor	Both	50-69 yea	Sexually tr	Rate	1999	0.04904	0.05321	0.04617
Deaths	Australasia	Both	50-69 yea	Sexually tr	Number	1995	1.84914	2.28195	1.55483
Deaths	Australasia	Both	50-69 yea	Sexually tr	Rate	1995	0.04998	0.06168	0.04203
Deaths	East Asia	Both	50-69 yea	HIV/AIDS	Number	1995	1263.41	1492.9	1093.62
Deaths	East Asia	Both	50-69 yea	HIV/AIDS	Rate	1995	0.72758	0.85974	0.6298
Deaths	Andean La	Both	50-69 yea	Sexually tr	Number	2004	9.49544	12.494	6.90984
Deaths	Andean La	Both	50-69 yea	Sexually tr	Rate	2004	0.1723	0.22671	0.12539
Deaths	High-incor	Both	50-69 yea	HIV/AIDS	Number	1998	132.048	135.279	128.863
Deaths	High-incor	Both	50-69 yea	HIV/AIDS	Rate	1998	0.32578	0.33375	0.31793
Deaths	Central As	Both	50-69 yea	Sexually tr	Number	2000	35.0239	39.2223	27.1806
Deaths	Central As	Both	50-69 yea	Sexually tr	Rate	2000	0.44547	0.49887	0.34571
Deaths	Central Eu	Both	50-69 yea	Sexually tr	Number	1993	16.9404	18.0781	14.7829
Deaths	Central Eu	Both	50-69 yea	Sexually tr	Rate	1993	0.06802	0.07259	0.05936
Deaths	Western E	Both	50-69 yea	HIV/AIDS	Number	1993	2475.49	2516.43	2435.99
Deaths	Western E	Both	50-69 yea	HIV/AIDS	Rate	1993	2.97051	3.01964	2.92312
Deaths	Oceania	Both	50-69 yea	Sexually tr	Number	1994	1.66274	3.4555	0.74043
Deaths	Oceania	Both	50-69 yea	Sexually tr	Rate	1994	0.27834	0.57844	0.12395
Deaths	Oceania	Both	50-69 yea	Sexually tr	Number	2002	1.90764	4.05985	0.80495
Deaths	Oceania	Both	50-69 yea	Sexually tr	Rate	2002	0.26079	0.55501	0.11004
Deaths	Southeast	Both	50-69 yea	Sexually tr	Number	1997	92.1196	122.302	64.8212
Deaths	Southeast	Both	50-69 yea	Sexually tr	Rate	1997	0.16797	0.223	0.11819
Deaths	Oceania	Both	50-69 yea	Sexually tr	Number	1998	1.72901	3.69748	0.75085
Deaths	Oceania	Both	50-69 yea	Sexually tr	Rate	1998	0.26249	0.56134	0.11399
Deaths	Caribbean	Both	50-69 yea	HIV/AIDS	Number	2005	3289.46	3893.87	2773.36
Deaths	Caribbean	Both	50-69 yea	HIV/AIDS	Rate	2005	54.6847	64.7325	46.1048
Deaths	High-incor	Both	50-69 yea	Sexually tr	Number	2008	35.8169	38.1089	34.2259
Deaths	High-incor	Both	50-69 yea	Sexually tr	Rate	2008	0.04839	0.05148	0.04624
Deaths	Tropical La	Both	50-69 yea	HIV/AIDS	Number	1998	1603.93	1635.19	1573.73
Deaths	Tropical La	Both	50-69 yea	HIV/AIDS	Rate	1998	7.95643	8.11148	7.8066
Deaths	Australasia	Both	50-69 yea	Sexually tr	Number	2003	1.50525	1.96472	1.27639
Deaths	Australasia	Both	50-69 yea	Sexually tr	Rate	2003	0.03205	0.04184	0.02718
Deaths	Oceania	Both	50-69 yea	Sexually tr	Number	1999	1.76288	3.83538	0.7599
Deaths	Oceania	Both	50-69 yea	Sexually tr	Rate	1999	0.26167	0.5693	0.11279
Deaths	Southern L	Both	50-69 yea	HIV/AIDS	Number	2005	389.444	397.249	382.074
Deaths	Southern L	Both	50-69 yea	HIV/AIDS	Rate	2005	4.14906	4.2322	4.07054
Deaths	Southeast	Both	50-69 yea	HIV/AIDS	Number	1994	2623.87	3366.6	1632.12
Deaths	Southeast	Both	50-69 yea	HIV/AIDS	Rate	1994	5.09659	6.53927	3.17022
Deaths	Central As	Both	50-69 yea	HIV/AIDS	Number	1997	54.2599	56.5785	52.231
Deaths	Central As	Both	50-69 yea	HIV/AIDS	Rate	1997	0.66858	0.69715	0.64358
Deaths	South Asia	Both	50-69 yea	HIV/AIDS	Number	1996	1902.17	2521.45	1459.3
Deaths	South Asia	Both	50-69 yea	HIV/AIDS	Rate	1996	1.53999	2.04137	1.18145
Deaths	Australasia	Both	50-69 yea	HIV/AIDS	Number	2004	56.9136	59.3199	54.5253
Deaths	Australasia	Both	50-69 yea	HIV/AIDS	Rate	2004	1.17508	1.22477	1.12577
Deaths	High-incor	Both	50-69 yea	Sexually tr	Number	2005	19.0113	20.4908	17.8965
Deaths	High-incor	Both	50-69 yea	Sexually tr	Rate	2005	0.04232	0.04561	0.03983
Deaths	East Asia	Both	50-69 yea	Sexually tr	Number	2000	190.587	257.903	162.248
Deaths	East Asia	Both	50-69 yea	Sexually tr	Rate	2000	0.09602	0.12994	0.08175
Deaths	Australasia	Both	50-69 yea	HIV/AIDS	Number	1998	59.6567	62.2376	57.0839
Deaths	Australasia	Both	50-69 yea	HIV/AIDS	Rate	1998	1.48011	1.54414	1.41628
Deaths	Southern L	Both	50-69 yea	Sexually tr	Number	1999	13.9065	15.7446	12.339
Deaths	Southern L	Both	50-69 yea	Sexually tr	Rate	1999	0.16587	0.18779	0.14717
Deaths	Southern S	Both	50-69 yea	Sexually tr	Number	2005	59.0302	95.176	45.6761
Deaths	Southern S	Both	50-69 yea	Sexually tr	Rate	2005	0.86393	1.39293	0.66849
Deaths	East Asia	Both	50-69 yea	HIV/AIDS	Number	1998	1663.56	2028.77	1484.37
Deaths	East Asia	Both	50-69 yea	HIV/AIDS	Rate	1998	0.89012	1.08553	0.79424
Deaths	Central As	Both	50-69 yea	Sexually tr	Number	1999	37.5758	42.0649	27.2379
Deaths	Central As	Both	50-69 yea	Sexually tr	Rate	1999	0.47519	0.53196	0.34445
Deaths	Central As	Both	50-69 yea	HIV/AIDS	Number	2005	82.6673	86.6249	78.9775
Deaths	Central As	Both	50-69 yea	HIV/AIDS	Rate	2005	0.95116	0.9967	0.90871
Deaths	South Asia	Both	50-69 yea	Sexually tr	Number	1998	1061.22	1290	762.574
Deaths	South Asia	Both	50-69 yea	Sexually tr	Rate	1998	0.82287	1.00027	0.5913
Deaths	Southeast	Both	50-69 yea	HIV/AIDS	Number	1998	6506.38	7079.66	5731.33
Deaths	Southeast	Both	50-69 yea	HIV/AIDS	Rate	1998	11.6087	12.6315	10.2258
Deaths	Eastern Eu	Both	50-69 yea	HIV/AIDS	Number	2001	1171.76	1193.13	1148.57
Deaths	Eastern Eu	Both	50-69 yea	HIV/AIDS	Rate	2001	2.60582	2.65334	2.55425
Deaths	East Asia	Both	50-69 yea	HIV/AIDS	Number	2004	3084.88	3707.76	2782.97
Deaths	East Asia	Both	50-69 yea	HIV/AIDS	Rate	2004	1.33307	1.60224	1.20261
Deaths	Central Su	Both	50-69 yea	Sexually tr	Number	2005	92.8693	204.615	24.2602
Deaths	Central Su	Both	50-69 yea	Sexually tr	Rate	2005	1.5519	3.41923	0.4054
Deaths	Western E	Both	50-69 yea	Sexually tr	Number	2010	38.6111	43.8241	36.3508
Deaths	Western E	Both	50-69 yea	Sexually tr	Rate	2010	0.03877	0.044	0.0365
Deaths	South Asia	Both	50-69 yea	HIV/AIDS	Number	2000	6608.27	7761.14	5617.72
Deaths	South Asia	Both	50-69 yea	HIV/AIDS	Rate	2000	4.93088	5.79111	4.19176
Deaths	Southern L	Both	50-69 yea	Sexually tr	Number	2000	13.7771	15.5658	12.2885
Deaths	Southern L	Both	50-69 yea	Sexually tr	Rate	2000	0.16172	0.18271	0.14424
Deaths	South Asia	Both	50-69 yea	HIV/AIDS	Number	1998	3820.15	4645.44	3101.18
Deaths	South Asia	Both	50-69 yea	HIV/AIDS	Rate	1998	2.96217	3.6021	2.40468
Deaths	Andean La	Both	50-69 yea	HIV/AIDS	Number	2002	393.073	707.079	292.204
Deaths	Andean La	Both	50-69 yea	HIV/AIDS	Rate	2002	7.62655	13.719	5.66945
Deaths	East Asia	Both	50-69 yea	Sexually tr	Number	2010	206.063	239.993	159.002
Deaths	East Asia	Both	50-69 yea	Sexually tr	Rate	2010	0.07485	0.08718	0.05776
Deaths	High-incor	Both	50-69 yea	Sexually tr	Number	2005	35.5868	37.6246	34.124
Deaths	High-incor	Both	50-69 yea	Sexually tr	Rate	2005	0.05327	0.05632	0.05108
Deaths	High-incor	Both	50-69 yea	Sexually tr	Number	1997	20.2095	21.9431	18.9023
Deaths	High-incor	Both	50-69 yea	Sexually tr	Rate	1997	0.05133	0.05573	0.04801
Deaths	Southern S	Both	50-69 yea	HIV/AIDS	Number	1998	28886.7	40591.3	18397
Deaths	Southern S	Both	50-69 yea	HIV/AIDS	Rate	1998	503.806	707.942	320.857
Deaths	Eastern Su	Both	50-69 yea	HIV/AIDS	Number	1999	77581.8	91641.9	62782.6
Deaths	Eastern Su	Both	50-69 yea	HIV/AIDS	Rate	1999	481.482	568.74	389.636
Deaths	Eastern Eu	Both	50-69 yea	HIV/AIDS	Number	1998	775.497	789.995	762.156
Deaths	Eastern Eu	Both	50-69 yea	HIV/AIDS	Rate	1998	1.72704	1.75932	1.69733
Deaths	High-incor	Both	50-69 yea	HIV/AIDS	Number	2005	187.253	191.745	183.116
Deaths	High-incor	Both	50-69 yea	HIV/AIDS	Rate	2005	0.41679	0.42679	0.40758
Deaths	Australasia	Both	50-69 yea	HIV/AIDS	Number	2003	43.2823	45.0966	41.5622
Deaths	Australasia	Both	50-69 yea	HIV/AIDS	Rate	2003	0.92171	0.96034	0.88508
Deaths	Tropical La	Both	50-69 yea	HIV/AIDS	Number	1994	1462	1492.22	1429.28
Deaths	Tropical La	Both	50-69 yea	HIV/AIDS	Rate	1994	8.16232	8.33103	7.9796
Deaths	Eastern Su	Both	50-69 yea	Sexually tr	Number	2002	429.879	838.225	169.051
Deaths	Eastern Su	Both	50-69 yea	Sexually tr	Rate	2002	2.501	4.87673	0.98353
Deaths	Oceania	Both	50-69 yea	HIV/AIDS	Number	2004	368.044	777.811	149.132
Deaths	Oceania	Both	50-69 yea	HIV/AIDS	Rate	2004	47.063	99.4612	19.07
Deaths	Southeast	Both	50-69 yea	Sexually tr	Number	2001	95.7732	126.843	62.3656
Deaths	Southeast	Both	50-69 yea	Sexually tr	Rate	2001	0.15865	0.21011	0.10331
Deaths	Southern S	Both	50-69 yea	Sexually tr	Number	2004	60.2197	98.5731	46.2578
Deaths	Southern S	Both	50-69 yea	Sexually tr	Rate	2004	0.90455	1.48065	0.69483
Deaths	Caribbean	Both	50-69 yea	Sexually tr	Number	2005	20.1605	30.9442	14.036
Deaths	Caribbean	Both	50-69 yea	Sexually tr	Rate	2005	0.33515	0.51442	0.23334
Deaths	Oceania	Both	50-69 yea	Sexually tr	Number	2005	2.18351	4.76237	0.90092
Deaths	Oceania	Both	50-69 yea	Sexually tr	Rate	2005	0.26953	0.58787	0.11121
Deaths	High-incor	Both	50-69 yea	Sexually tr	Number	2006	18.6902	19.9801	17.5668
Deaths	High-incor	Both	50-69 yea	Sexually tr	Rate	2006	0.04136	0.04421	0.03887
Deaths	Andean La	Both	50-69 yea	Sexually tr	Number	2005	9.74197	12.73	7.08786
Deaths	Andean La	Both	50-69 yea	Sexually tr	Rate	2005	0.17074	0.22311	0.12423
Deaths	Western E	Both	50-69 yea	HIV/AIDS	Number	2005	1369.28	1391.24	1349.1
Deaths	Western E	Both	50-69 yea	HIV/AIDS	Rate	2005	1.46245	1.4859	1.4409
Deaths	Caribbean	Both	50-69 yea	Sexually tr	Number	1994	17.6315	29.0284	10.767
Deaths	Caribbean	Both	50-69 yea	Sexually tr	Rate	1994	0.38923	0.64082	0.23769
Deaths	Western E	Both	50-69 yea	Sexually tr	Number	2005	39.9654	48.8984	37.619
Deaths	Western E	Both	50-69 yea	Sexually tr	Rate	2005	0.04268	0.05223	0.04018
Deaths	Eastern Su	Both	50-69 yea	Sexually tr	Number	2000	446.997	881.552	172.253
Deaths	Eastern Su	Both	50-69 yea	Sexually tr	Rate	2000	2.74588	5.41534	1.05814
Deaths	High-incor	Both	50-69 yea	Sexually tr	Number	2003	34.5309	36.4172	33.03
Deaths	High-incor	Both	50-69 yea	Sexually tr	Rate	2003	0.05546	0.05849	0.05305
Deaths	Tropical La	Both	50-69 yea	Sexually tr	Number	2004	51.1448	54.3247	48.2308
Deaths	Tropical La	Both	50-69 yea	Sexually tr	Rate	2004	0.20587	0.21867	0.19414
Deaths	Central Lat	Both	50-69 yea	HIV/AIDS	Number	1998	1315.98	1335.99	1296.19
Deaths	Central Lat	Both	50-69 yea	HIV/AIDS	Rate	1998	6.98685	7.09313	6.88179
Deaths	Central Eu	Both	50-69 yea	HIV/AIDS	Number	2004	149.288	160.915	142.243
Deaths	Central Eu	Both	50-69 yea	HIV/AIDS	Rate	2004	0.55918	0.60273	0.53279
Deaths	South Asia	Both	50-69 yea	Sexually tr	Number	1997	1068.21	1312.26	763.32
Deaths	South Asia	Both	50-69 yea	Sexually tr	Rate	1997	0.84563	1.03883	0.60427
Deaths	South Asia	Both	50-69 yea	HIV/AIDS	Number	2001	8498.48	9846.11	7383.24
Deaths	South Asia	Both	50-69 yea	HIV/AIDS	Rate	2001	6.15738	7.13378	5.34936
Deaths	Australasia	Both	50-69 yea	HIV/AIDS	Number	1999	50.8546	53.0605	48.7695
Deaths	Australasia	Both	50-69 yea	HIV/AIDS	Rate	1999	1.22524	1.27838	1.175
Deaths	High-incor	Both	50-69 yea	HIV/AIDS	Number	2010	4131.05	4173.98	4088.88
Deaths	High-incor	Both	50-69 yea	HIV/AIDS	Rate	2010	5.23848	5.29291	5.18501
Deaths	Tropical La	Both	50-69 yea	Sexually tr	Number	2005	50.7141	53.8339	47.8708
Deaths	Tropical La	Both	50-69 yea	Sexually tr	Rate	2005	0.19672	0.20882	0.18569
Deaths	Central Eu	Both	50-69 yea	HIV/AIDS	Number	2005	158.402	171.018	150.823
Deaths	Central Eu	Both	50-69 yea	HIV/AIDS	Rate	2005	0.58307	0.62951	0.55517
Deaths	Southern S	Both	50-69 yea	Sexually tr	Number	2006	59.6623	95.9526	46.6407
Deaths	Southern S	Both	50-69 yea	Sexually tr	Rate	2006	0.8502	1.36734	0.66464
Deaths	Southern S	Both	50-69 yea	HIV/AIDS	Number	1999	35568.2	47789.5	23576.7
Deaths	Southern S	Both	50-69 yea	HIV/AIDS	Rate	1999	606.951	815.502	402.324
Deaths	High-incor	Both	50-69 yea	HIV/AIDS	Number	2000	127.376	130.25	124.535
Deaths	High-incor	Both	50-69 yea	HIV/AIDS	Rate	2000	0.29959	0.30635	0.29291
Deaths	Central Lat	Both	50-69 yea	Sexually tr	Number	2000	27.8161	31.5475	25.9561
Deaths	Central Lat	Both	50-69 yea	Sexually tr	Rate	2000	0.13801	0.15652	0.12878
Deaths	South Asia	Both	50-69 yea	Sexually tr	Number	2005	942.128	1135.16	716.11
Deaths	South Asia	Both	50-69 yea	Sexually tr	Rate	2005	0.60648	0.73074	0.46098
Deaths	North Afric	Both	50-69 yea	HIV/AIDS	Number	2001	585.26	1075.53	321.16
Deaths	North Afric	Both	50-69 yea	HIV/AIDS	Rate	2001	1.43462	2.63638	0.78724
Deaths	Caribbean	Both	50-69 yea	HIV/AIDS	Number	1997	2605.25	3469.92	1884.99
Deaths	Caribbean	Both	50-69 yea	HIV/AIDS	Rate	1997	53.2386	70.9083	38.5199
Deaths	Eastern Eu	Both	50-69 yea	Sexually tr	Number	2003	179.109	195.615	133.75
Deaths	Eastern Eu	Both	50-69 yea	Sexually tr	Rate	2003	0.39106	0.4271	0.29203
Deaths	South Asia	Both	50-69 yea	HIV/AIDS	Number	1999	5105.24	6060.12	4230.11
Deaths	South Asia	Both	50-69 yea	HIV/AIDS	Rate	1999	3.87984	4.60552	3.21476
Deaths	High-incor	Both	50-69 yea	HIV/AIDS	Number	2010	240.004	246.436	234.093
Deaths	High-incor	Both	50-69 yea	HIV/AIDS	Rate	2010	0.51301	0.52676	0.50038
Deaths	Eastern Su	Both	50-69 yea	HIV/AIDS	Number	2000	80676.5	93958.2	67306.8
Deaths	Eastern Su	Both	50-69 yea	HIV/AIDS	Rate	2000	495.592	577.181	413.463
Deaths	Andean La	Both	50-69 yea	Sexually tr	Number	2015	10.305	14.3476	7.26581
Deaths	Andean La	Both	50-69 yea	Sexually tr	Rate	2015	0.12653	0.17617	0.08921
Deaths	Western E	Both	50-69 yea	HIV/AIDS	Number	2006	1411.79	1434.56	1388.11
Deaths	Western E	Both	50-69 yea	HIV/AIDS	Rate	2006	1.49032	1.51436	1.46533
Deaths	Western E	Both	50-69 yea	Sexually tr	Number	2003	41.7892	52.2296	39.4222
Deaths	Western E	Both	50-69 yea	Sexually tr	Rate	2003	0.04563	0.05703	0.04305
Deaths	North Afric	Both	50-69 yea	HIV/AIDS	Number	2015	1461.61	2894.7	870.588
Deaths	North Afric	Both	50-69 yea	HIV/AIDS	Rate	2015	2.14361	4.24541	1.27681
Deaths	Eastern Eu	Both	50-69 yea	Sexually tr	Number	2005	123.268	133.166	112.775
Deaths	Eastern Eu	Both	50-69 yea	Sexually tr	Rate	2005	0.2616	0.28261	0.23934
Deaths	Central As	Both	50-69 yea	Sexually tr	Number	2005	25.2133	30.6903	22.2078
Deaths	Central As	Both	50-69 yea	Sexually tr	Rate	2005	0.2901	0.35312	0.25552
Deaths	Central Su	Both	50-69 yea	HIV/AIDS	Number	2015	8854.91	10856.8	7185.89
Deaths	Central Su	Both	50-69 yea	HIV/AIDS	Rate	2015	100.41	123.111	81.4844
Deaths	South Asia	Both	50-69 yea	Sexually tr	Number	2015	1230.53	1516.24	867.445
Deaths	South Asia	Both	50-69 yea	Sexually tr	Rate	2015	0.56875	0.70081	0.40093
Deaths	Southern L	Both	50-69 yea	HIV/AIDS	Number	1996	345.621	353.219	338.385
Deaths	Southern L	Both	50-69 yea	HIV/AIDS	Rate	1996	4.29567	4.39012	4.20574
Deaths	Central As	Both	50-69 yea	HIV/AIDS	Number	1999	59.4571	61.7402	57.221
Deaths	Central As	Both	50-69 yea	HIV/AIDS	Rate	1999	0.7519	0.78077	0.72362
Deaths	Caribbean	Both	50-69 yea	HIV/AIDS	Number	1994	1863.77	2657.58	1246.77
Deaths	Caribbean	Both	50-69 yea	HIV/AIDS	Rate	1994	41.1438	58.6678	27.5233
Deaths	Southern S	Both	50-69 yea	Sexually tr	Number	2015	57	84.5649	47.0219
Deaths	Southern S	Both	50-69 yea	Sexually tr	Rate	2015	0.6317	0.93719	0.52112
Deaths	Andean La	Both	50-69 yea	HIV/AIDS	Number	2000	312.779	648.206	224.063
Deaths	Andean La	Both	50-69 yea	HIV/AIDS	Rate	2000	6.4871	13.4439	4.64711
Deaths	Australasia	Both	50-69 yea	Sexually tr	Number	1998	1.5442	1.95837	1.31763
Deaths	Australasia	Both	50-69 yea	Sexually tr	Rate	1998	0.03831	0.04859	0.03269
Deaths	Western S	Both	50-69 yea	HIV/AIDS	Number	2004	38340.4	43208.5	33871.5
Deaths	Western S	Both	50-69 yea	HIV/AIDS	Rate	2004	180.247	203.133	159.238
Deaths	Andean La	Both	50-69 yea	Sexually tr	Number	2008	9.45477	12.5016	6.96455
Deaths	Andean La	Both	50-69 yea	Sexually tr	Rate	2008	0.14937	0.1975	0.11003
Deaths	High-incor	Both	50-69 yea	HIV/AIDS	Number	1999	136.551	139.294	133.688
Deaths	High-incor	Both	50-69 yea	HIV/AIDS	Rate	1999	0.32791	0.33449	0.32103
Deaths	Southeast	Both	50-69 yea	Sexually tr	Number	1998	93.0186	120.624	65.1934
Deaths	Southeast	Both	50-69 yea	Sexually tr	Rate	1998	0.16596	0.21522	0.11632
Deaths	Andean La	Both	50-69 yea	HIV/AIDS	Number	2004	419.464	681.464	322.546
Deaths	Andean La	Both	50-69 yea	HIV/AIDS	Rate	2004	7.61154	12.3658	5.85289
Deaths	Western S	Both	50-69 yea	HIV/AIDS	Number	1994	13449.6	18953.5	9029.99
Deaths	Western S	Both	50-69 yea	HIV/AIDS	Rate	1994	78.7603	110.99	52.8791
Deaths	Western S	Both	50-69 yea	Sexually tr	Number	1998	58.7559	78.2614	31.8141
Deaths	Western S	Both	50-69 yea	Sexually tr	Rate	1998	0.31974	0.42589	0.17313
Deaths	Western E	Both	50-69 yea	Sexually tr	Number	2008	39.4864	47.2245	37.1792
Deaths	Western E	Both	50-69 yea	Sexually tr	Rate	2008	0.04067	0.04865	0.0383
Deaths	Central Lat	Both	50-69 yea	Sexually tr	Number	2005	34.3206	36.9704	32.1166
Deaths	Central Lat	Both	50-69 yea	Sexually tr	Rate	2005	0.1411	0.15199	0.13204
Deaths	Oceania	Both	50-69 yea	HIV/AIDS	Number	1999	97.8567	213.16	41.3313
Deaths	Oceania	Both	50-69 yea	HIV/AIDS	Rate	1999	14.5252	31.6399	6.13494
Deaths	Southern L	Both	50-69 yea	Sexually tr	Number	2004	13.0175	14.6152	11.5731
Deaths	Southern L	Both	50-69 yea	Sexually tr	Rate	2004	0.14181	0.15922	0.12608
Deaths	Australasia	Both	50-69 yea	Sexually tr	Number	2006	1.3853	1.81885	1.16771
Deaths	Australasia	Both	50-69 yea	Sexually tr	Rate	2006	0.02693	0.03535	0.0227
Deaths	Central Eu	Both	50-69 yea	Sexually tr	Number	2016	9.9588	11.0938	9.04308
Deaths	Central Eu	Both	50-69 yea	Sexually tr	Rate	2016	0.03328	0.03707	0.03022
Deaths	High-incor	Both	50-69 yea	Sexually tr	Number	2016	16.2155	17.6864	14.9608
Deaths	High-incor	Both	50-69 yea	Sexually tr	Rate	2016	0.03289	0.03588	0.03035
Deaths	Andean La	Both	50-69 yea	Sexually tr	Number	2006	9.4671	12.2981	6.95992
Deaths	Andean La	Both	50-69 yea	Sexually tr	Rate	2006	0.16038	0.20835	0.11791
Deaths	High-incor	Both	50-69 yea	HIV/AIDS	Number	2000	3677.39	3721.56	3635.51
Deaths	High-incor	Both	50-69 yea	HIV/AIDS	Rate	2000	6.49052	6.56847	6.4166
Deaths	Western S	Both	50-69 yea	Sexually tr	Number	2005	41.4078	53.9171	29.7041
Deaths	Western S	Both	50-69 yea	Sexually tr	Rate	2005	0.18939	0.24661	0.13586
Deaths	High-incor	Both	50-69 yea	HIV/AIDS	Number	2002	156.403	160.109	152.652
Deaths	High-incor	Both	50-69 yea	HIV/AIDS	Rate	2002	0.35758	0.36605	0.349
Deaths	Southeast	Both	50-69 yea	Sexually tr	Number	2005	103.163	141.691	61.712
Deaths	Southeast	Both	50-69 yea	Sexually tr	Rate	2005	0.15021	0.20631	0.08986
Deaths	Southern S	Both	50-69 yea	HIV/AIDS	Number	2001	50036.3	62769.2	36070
Deaths	Southern S	Both	50-69 yea	HIV/AIDS	Rate	2001	814.07	1021.23	586.845
Deaths	High-incor	Both	50-69 yea	Sexually tr	Number	2000	34.1984	35.9483	32.6209
Deaths	High-incor	Both	50-69 yea	Sexually tr	Rate	2000	0.06036	0.06345	0.05758
Deaths	Western S	Both	50-69 yea	Sexually tr	Number	2011	38.4875	49.8739	28.108
Deaths	Western S	Both	50-69 yea	Sexually tr	Rate	2011	0.14629	0.18957	0.10684
Deaths	Southeast	Both	50-69 yea	Sexually tr	Number	1999	94.8192	124.564	64.7974
Deaths	Southeast	Both	50-69 yea	Sexually tr	Rate	1999	0.16537	0.21725	0.11301
Deaths	Central As	Both	50-69 yea	HIV/AIDS	Number	2000	57.0634	59.1829	54.9738
Deaths	Central As	Both	50-69 yea	HIV/AIDS	Rate	2000	0.7258	0.75275	0.69922
Deaths	North Afric	Both	50-69 yea	HIV/AIDS	Number	2005	839.401	1481.69	488.314
Deaths	North Afric	Both	50-69 yea	HIV/AIDS	Rate	2005	1.80849	3.19232	1.05207
Deaths	Southeast	Both	50-69 yea	HIV/AIDS	Number	2001	8896.74	9689.36	7986.53
Deaths	Southeast	Both	50-69 yea	HIV/AIDS	Rate	2001	14.7372	16.0502	13.2295
Deaths	Tropical La	Both	50-69 yea	HIV/AIDS	Number	2005	2664.75	2711.01	2620.38
Deaths	Tropical La	Both	50-69 yea	HIV/AIDS	Rate	2005	10.3364	10.5159	10.1643
Deaths	Southern L	Both	50-69 yea	HIV/AIDS	Number	2000	303.757	310.414	297
Deaths	Southern L	Both	50-69 yea	HIV/AIDS	Rate	2000	3.56557	3.64371	3.48626
Deaths	Oceania	Both	50-69 yea	HIV/AIDS	Number	2005	411.334	888.586	155.516
Deaths	Oceania	Both	50-69 yea	HIV/AIDS	Rate	2005	50.7749	109.687	19.1968
Deaths	Western E	Both	50-69 yea	HIV/AIDS	Number	2009	1428.94	1452.32	1407.91
Deaths	Western E	Both	50-69 yea	HIV/AIDS	Rate	2009	1.45402	1.47781	1.43262
Deaths	Southern S	Both	50-69 yea	Sexually tr	Number	2008	58.9079	94.723	46.3671
Deaths	Southern S	Both	50-69 yea	Sexually tr	Rate	2008	0.79496	1.27828	0.62572
Deaths	Eastern Eu	Both	50-69 yea	HIV/AIDS	Number	2017	2798.49	2859.26	2733.08
Deaths	Eastern Eu	Both	50-69 yea	HIV/AIDS	Rate	2017	5.1048	5.21565	4.98548
Deaths	Australasia	Both	50-69 yea	Sexually tr	Number	2017	1.34717	1.67991	1.11795
Deaths	Australasia	Both	50-69 yea	Sexually tr	Rate	2017	0.02047	0.02552	0.01699
Deaths	Eastern Su	Both	50-69 yea	HIV/AIDS	Number	1998	73468.2	87839.7	57971
Deaths	Eastern Su	Both	50-69 yea	HIV/AIDS	Rate	1998	464.001	554.766	366.125
Deaths	East Asia	Both	50-69 yea	Sexually tr	Number	2002	221.199	280.299	185.665
Deaths	East Asia	Both	50-69 yea	Sexually tr	Rate	2002	0.10361	0.1313	0.08697
Deaths	Central Lat	Both	50-69 yea	HIV/AIDS	Number	2005	1970.72	2008.59	1934.39
Deaths	Central Lat	Both	50-69 yea	HIV/AIDS	Rate	2005	8.10189	8.2576	7.95255
Deaths	Central Lat	Both	50-69 yea	Sexually tr	Number	1999	26.8923	31.3664	25.1002
Deaths	Central Lat	Both	50-69 yea	Sexually tr	Rate	1999	0.13771	0.16062	0.12854
Deaths	South Asia	Both	50-69 yea	Sexually tr	Number	2000	1004.54	1213.32	736.233
Deaths	South Asia	Both	50-69 yea	Sexually tr	Rate	2000	0.74955	0.90534	0.54935
Deaths	Andean La	Both	50-69 yea	Sexually tr	Number	2009	10.1272	13.3312	7.39655
Deaths	Andean La	Both	50-69 yea	Sexually tr	Rate	2009	0.15459	0.2035	0.11291
Deaths	Andean La	Both	50-69 yea	HIV/AIDS	Number	1998	258.729	646.232	176.275
Deaths	Andean La	Both	50-69 yea	HIV/AIDS	Rate	1998	5.73161	14.3159	3.90501
Deaths	Southern S	Both	50-69 yea	HIV/AIDS	Number	2006	70831.9	78906.2	63068.6
Deaths	Southern S	Both	50-69 yea	HIV/AIDS	Rate	2006	1009.37	1124.43	898.738
Deaths	North Afric	Both	50-69 yea	HIV/AIDS	Number	1998	413.425	783.912	215.043
Deaths	North Afric	Both	50-69 yea	HIV/AIDS	Rate	1998	1.09116	2.06899	0.56757
Deaths	East Asia	Both	50-69 yea	HIV/AIDS	Number	1999	1859.03	2267.32	1630.08
Deaths	East Asia	Both	50-69 yea	HIV/AIDS	Rate	1999	0.96022	1.17111	0.84196
Deaths	Central Lat	Both	50-69 yea	Sexually tr	Number	2007	35.7246	38.0894	33.7003
Deaths	Central Lat	Both	50-69 yea	Sexually tr	Rate	2007	0.13607	0.14508	0.12836
Deaths	Eastern Su	Both	50-69 yea	Sexually tr	Number	2001	437.201	865.563	169.844
Deaths	Eastern Su	Both	50-69 yea	Sexually tr	Rate	2001	2.61289	5.17296	1.01506
Deaths	Southern S	Both	50-69 yea	HIV/AIDS	Number	1997	22696.2	33283.3	13444.6
Deaths	Southern S	Both	50-69 yea	HIV/AIDS	Rate	1997	404.777	593.591	239.778
Deaths	Eastern Eu	Both	50-69 yea	Sexually tr	Number	1999	309.897	355.817	144.803
Deaths	Eastern Eu	Both	50-69 yea	Sexually tr	Rate	1999	0.69381	0.79661	0.32419
Deaths	Western E	Both	50-69 yea	Sexually tr	Number	2009	39.9093	46.6108	37.5323
Deaths	Western E	Both	50-69 yea	Sexually tr	Rate	2009	0.04061	0.04743	0.03819
Deaths	East Asia	Both	50-69 yea	Sexually tr	Number	2018	221.799	273.888	157.688
Deaths	East Asia	Both	50-69 yea	Sexually tr	Rate	2018	0.05981	0.07385	0.04252
Deaths	Western S	Both	50-69 yea	HIV/AIDS	Number	2003	37783.9	43342.9	32663.9
Deaths	Western S	Both	50-69 yea	HIV/AIDS	Rate	2003	182.224	209.033	157.531
Deaths	Central As	Both	50-69 yea	Sexually tr	Number	2002	28.5243	31.7494	25.5299
Deaths	Central As	Both	50-69 yea	Sexually tr	Rate	2002	0.35216	0.39197	0.31519
Deaths	Central Lat	Both	50-69 yea	Sexually tr	Number	2008	37.3312	39.7007	35.2528
Deaths	Central Lat	Both	50-69 yea	Sexually tr	Rate	2008	0.13684	0.14552	0.12922
Deaths	Andean La	Both	50-69 yea	HIV/AIDS	Number	1999	281.858	643.861	196.749
Deaths	Andean La	Both	50-69 yea	HIV/AIDS	Rate	1999	6.03625	13.7888	4.21354
Deaths	Central Su	Both	50-69 yea	Sexually tr	Number	2003	96.3132	216.952	24.4748
Deaths	Central Su	Both	50-69 yea	Sexually tr	Rate	2003	1.71466	3.86238	0.43572
Deaths	Oceania	Both	50-69 yea	Sexually tr	Number	2018	3.00419	6.36745	1.29086
Deaths	Oceania	Both	50-69 yea	Sexually tr	Rate	2018	0.22514	0.47719	0.09674

Deaths	North Afric	Both	50-69 yea	HIV/AIDS	Rate	2002	1.54931	2.75028	0.86471
Deaths	Central La	Both	50-69 yea	HIV/AIDS	Number	1999	1384	1403.82	1364.82
Deaths	Central La	Both	50-69 yea	HIV/AIDS	Rate	1999	7.08727	7.18877	6.98908
Deaths	Central La	Both	50-69 yea	Sexually tr	Number	2001	28.2496	31.7296	26.3595
Deaths	Central La	Both	50-69 yea	Sexually tr	Rate	2001	0.13493	0.15155	0.1259
Deaths	High-incor	Both	50-69 yea	Sexually tr	Number	2009	36.5413	38.8905	34.956
Deaths	High-incor	Both	50-69 yea	Sexually tr	Rate	2009	0.04783	0.05091	0.04576
Deaths	Oceania	Both	50-69 yea	HIV/AIDS	Number	2010	512.794	1243.89	179.612
Deaths	Oceania	Both	50-69 yea	HIV/AIDS	Rate	2010	52.4668	127.269	18.3771
Deaths	Caribbean	Both	50-69 yea	HIV/AIDS	Number	2008	2749.09	3323.19	2271.53
Deaths	Caribbean	Both	50-69 yea	HIV/AIDS	Rate	2008	42.6959	51.6122	35.2789
Deaths	Central As	Both	50-69 yea	Sexually tr	Number	2007	24.736	30.4333	21.6704
Deaths	Central As	Both	50-69 yea	Sexually tr	Rate	2007	0.26891	0.33085	0.23558
Deaths	Central La	Both	50-69 yea	HIV/AIDS	Number	2007	2223.26	2293.54	2163.79
Deaths	Central La	Both	50-69 yea	HIV/AIDS	Rate	2007	8.46835	8.73608	8.24184
Deaths	High-incor	Both	50-69 yea	Sexually tr	Number	2010	36.7961	39.2074	35.1813
Deaths	High-incor	Both	50-69 yea	Sexually tr	Rate	2010	0.04666	0.04972	0.04461
Deaths	East Asia	Both	50-69 yea	Sexually tr	Number	2001	205.082	268.729	172.6
Deaths	East Asia	Both	50-69 yea	Sexually tr	Rate	2001	0.09964	0.13056	0.08386
Deaths	Southern S	Both	50-69 yea	Sexually tr	Number	2009	59.3742	94.8183	46.8197
Deaths	Southern S	Both	50-69 yea	Sexually tr	Rate	2009	0.77978	1.24527	0.6149
Deaths	Australasi	Both	50-69 yea	HIV/AIDS	Number	2000	46.7739	48.5796	44.968
Deaths	Australasi	Both	50-69 yea	HIV/AIDS	Rate	2000	1.09332	1.13552	1.05111
Deaths	Central Eu	Both	50-69 yea	Sexually tr	Number	2010	10.1536	11.0367	9.30531
Deaths	Central Eu	Both	50-69 yea	Sexually tr	Rate	2010	0.03506	0.03811	0.03213
Deaths	High-incor	Both	50-69 yea	Sexually tr	Number	2001	20.0548	21.6025	18.8526
Deaths	High-incor	Both	50-69 yea	Sexually tr	Rate	2001	0.04648	0.05007	0.04369
Deaths	East Asia	Both	50-69 yea	HIV/AIDS	Number	2001	2385.74	2996.86	2024.17
Deaths	East Asia	Both	50-69 yea	HIV/AIDS	Rate	2001	1.1591	1.45601	0.98343
Deaths	Eastern Eu	Both	50-69 yea	HIV/AIDS	Number	2002	1207.26	1230.19	1182.8
Deaths	Eastern Eu	Both	50-69 yea	HIV/AIDS	Rate	2002	2.66044	2.71098	2.60654
Deaths	Central Eu	Both	50-69 yea	Sexually tr	Number	2005	10.8043	11.6849	9.99721
Deaths	Central Eu	Both	50-69 yea	Sexually tr	Rate	2005	0.03977	0.04301	0.0368
Deaths	Oceania	Both	50-69 yea	Sexually tr	Number	2011	2.68308	6.05527	1.1342
Deaths	Oceania	Both	50-69 yea	Sexually tr	Rate	2011	0.26346	0.59458	0.11137
Deaths	Australasi	Both	50-69 yea	Sexually tr	Number	2000	1.43515	1.91466	1.19912
Deaths	Australasi	Both	50-69 yea	Sexually tr	Rate	2000	0.03355	0.04475	0.02803
Deaths	Southern S	Both	50-69 yea	HIV/AIDS	Number	2005	70882	80195.2	60298.6
Deaths	Southern S	Both	50-69 yea	HIV/AIDS	Rate	2005	1037.38	1173.68	882.491
Deaths	Eastern Su	Both	50-69 yea	HIV/AIDS	Number	2002	87076	98280.9	76427.9
Deaths	Eastern Su	Both	50-69 yea	HIV/AIDS	Rate	2002	506.602	571.791	444.652
Deaths	Australasi	Both	50-69 yea	Sexually tr	Number	2001	1.47722	1.93166	1.24963
Deaths	Australasi	Both	50-69 yea	Sexually tr	Rate	2001	0.03351	0.04381	0.02834
Deaths	Central Su	Both	50-69 yea	HIV/AIDS	Number	2003	10653.7	12429.4	9131.86
Deaths	Central Su	Both	50-69 yea	HIV/AIDS	Rate	2003	189.667	221.28	162.574
Deaths	Andean La	Both	50-69 yea	Sexually tr	Number	2010	10.2826	13.4439	7.3813
Deaths	Andean La	Both	50-69 yea	Sexually tr	Rate	2010	0.15161	0.19822	0.10883
Deaths	High-incor	Both	50-69 yea	Sexually tr	Number	2002	33.5972	35.5013	32.0512
Deaths	High-incor	Both	50-69 yea	Sexually tr	Rate	2002	0.05578	0.05894	0.05321
Deaths	Oceania	Both	50-69 yea	Sexually tr	Number	2007	2.35642	5.08271	0.98732
Deaths	Oceania	Both	50-69 yea	Sexually tr	Rate	2007	0.26976	0.58186	0.11303
Deaths	Southern L	Both	50-69 yea	HIV/AIDS	Number	2008	469.995	479.531	460.69
Deaths	Southern L	Both	50-69 yea	HIV/AIDS	Rate	2008	4.66171	4.7563	4.56942
Deaths	Central Su	Both	50-69 yea	HIV/AIDS	Number	2010	9619.71	11542.4	7928.38
Deaths	Central Su	Both	50-69 yea	HIV/AIDS	Rate	2010	134.092	160.893	110.516
Deaths	Eastern Eu	Both	50-69 yea	Sexually tr	Number	2008	96.9879	111.849	91.5561
Deaths	Eastern Eu	Both	50-69 yea	Sexually tr	Rate	2008	0.20194	0.23288	0.19063
Deaths	Eastern Eu	Both	50-69 yea	HIV/AIDS	Number	2006	1551.01	1580.37	1519.93
Deaths	Eastern Eu	Both	50-69 yea	HIV/AIDS	Rate	2006	3.26484	3.32665	3.19943
Deaths	Central La	Both	50-69 yea	HIV/AIDS	Number	2001	1636.5	1665.25	1607.22
Deaths	Central La	Both	50-69 yea	HIV/AIDS	Rate	2001	7.81663	7.95395	7.67675
Deaths	North Afric	Both	50-69 yea	HIV/AIDS	Number	2000	522.212	951.302	282.795
Deaths	North Afric	Both	50-69 yea	HIV/AIDS	Rate	2000	1.31755	2.40016	0.7135
Deaths	Central Eu	Both	50-69 yea	Sexually tr	Number	2009	10.8308	11.7167	9.93406
Deaths	Central Eu	Both	50-69 yea	Sexually tr	Rate	2009	0.03773	0.04082	0.03461
Deaths	Central La	Both	50-69 yea	HIV/AIDS	Number	2009	2447.76	2538.01	2370.41
Deaths	Central La	Both	50-69 yea	HIV/AIDS	Rate	2009	8.63326	8.95158	8.36045
Deaths	Southeast	Both	50-69 yea	Sexually tr	Number	2002	97.0494	126.629	62.4926
Deaths	Southeast	Both	50-69 yea	Sexually tr	Rate	2002	0.15595	0.20348	0.10042
Deaths	Australasi	Both	50-69 yea	HIV/AIDS	Number	2008	42.6533	44.396	40.9203
Deaths	Australasi	Both	50-69 yea	HIV/AIDS	Rate	2008	0.78463	0.81669	0.75276
Deaths	Southeast	Both	50-69 yea	HIV/AIDS	Number	2003	9960.22	11069	8730.3
Deaths	Southeast	Both	50-69 yea	HIV/AIDS	Rate	2003	15.5093	17.2358	13.5942
Deaths	Southeast	Both	50-69 yea	HIV/AIDS	Number	2015	11497.1	15796.3	9209.39
Deaths	Southeast	Both	50-69 yea	HIV/AIDS	Rate	2015	11.4051	15.6698	9.13563
Deaths	Southern S	Both	50-69 yea	HIV/AIDS	Number	2008	64076	72668.2	55559.1
Deaths	Southern S	Both	50-69 yea	HIV/AIDS	Rate	2008	864.702	980.653	749.767
Deaths	Central La	Both	50-69 yea	HIV/AIDS	Number	2006	2150.25	2202.29	2102.43
Deaths	Central La	Both	50-69 yea	HIV/AIDS	Rate	2006	8.51014	8.71608	8.32089
Deaths	Oceania	Both	50-69 yea	Sexually tr	Number	2004	2.07508	4.39666	0.8478
Deaths	Oceania	Both	50-69 yea	Sexually tr	Rate	2004	0.26535	0.56222	0.10841
Deaths	South Asia	Both	50-69 yea	HIV/AIDS	Number	2010	14872.6	18998.8	11761.4
Deaths	South Asia	Both	50-69 yea	HIV/AIDS	Rate	2010	8.12955	10.385	6.42896
Deaths	Central Su	Both	50-69 yea	HIV/AIDS	Number	2009	9785.87	11813	8084.41
Deaths	Central Su	Both	50-69 yea	HIV/AIDS	Rate	2009	141.816	171.193	117.159
Deaths	Central Su	Both	50-69 yea	HIV/AIDS	Number	2005	10665.9	12555	9060.75
Deaths	Central Su	Both	50-69 yea	HIV/AIDS	Rate	2005	178.233	209.801	151.41
Deaths	Central As	Both	50-69 yea	Sexually tr	Number	2010	24.0811	29.0788	20.8426
Deaths	Central As	Both	50-69 yea	Sexually tr	Rate	2010	0.23372	0.28223	0.20229
Deaths	Western S	Both	50-69 yea	Sexually tr	Number	2010	38.0862	48.685	27.6941
Deaths	Western S	Both	50-69 yea	Sexually tr	Rate	2010	0.14968	0.19133	0.10884
Deaths	Andean La	Both	50-69 yea	Sexually tr	Number	2019	10.8119	15.4457	7.21944
Deaths	Andean La	Both	50-69 yea	Sexually tr	Rate	2019	0.11707	0.16724	0.07817
Deaths	Western S	Both	50-69 yea	Sexually tr	Number	2006	39.9905	51.7674	28.7547
Deaths	Western S	Both	50-69 yea	Sexually tr	Rate	2006	0.17766	0.22998	0.12775
Deaths	Australasi	Both	50-69 yea	Sexually tr	Number	2011	1.21092	1.53947	1.01639
Deaths	Australasi	Both	50-69 yea	Sexually tr	Rate	2011	0.02059	0.02618	0.01728
Deaths	South Asia	Both	50-69 yea	Sexually tr	Number	2019	1256.45	1641.41	866.295
Deaths	South Asia	Both	50-69 yea	Sexually tr	Rate	2019	0.51248	0.6695	0.35334
Deaths	Southern S	Both	50-69 yea	Sexually tr	Number	2019	50.5012	74.2764	40.4294
Deaths	Southern S	Both	50-69 yea	Sexually tr	Rate	2019	0.50478	0.74242	0.4041
Deaths	Central Su	Both	50-69 yea	HIV/AIDS	Number	2019	7575.28	9312.3	6169.11
Deaths	Central Su	Both	50-69 yea	HIV/AIDS	Rate	2019	73.0201	89.7638	59.4657
Deaths	Western S	Both	50-69 yea	Sexually tr	Number	2019	43.6968	57.8784	32.1157
Deaths	Western S	Both	50-69 yea	Sexually tr	Rate	2019	0.12431	0.16465	0.09136
Deaths	Eastern Su	Both	50-69 yea	HIV/AIDS	Number	2001	84530.3	96939.7	72476.4
Deaths	Eastern Su	Both	50-69 yea	HIV/AIDS	Rate	2001	505.188	579.352	433.149
Deaths	Southern L	Both	50-69 yea	Sexually tr	Number	2008	12.6996	14.3432	11.2902
Deaths	Southern L	Both	50-69 yea	Sexually tr	Rate	2008	0.12596	0.14226	0.11198
Deaths	Southern S	Both	50-69 yea	Sexually tr	Number	2010	59.6556	94.7545	47.656
Deaths	Southern S	Both	50-69 yea	Sexually tr	Rate	2010	0.76245	1.21104	0.60908
Deaths	Oceania	Both	50-69 yea	HIV/AIDS	Number	2001	196.129	406.1	83.5558
Deaths	Oceania	Both	50-69 yea	HIV/AIDS	Rate	2001	27.678	57.3096	11.7915
Deaths	Central Eu	Both	50-69 yea	Sexually tr	Number	2004	10.9864	11.8031	10.1654
Deaths	Central Eu	Both	50-69 yea	Sexually tr	Rate	2004	0.04115	0.04421	0.03808
Deaths	Southern L	Both	50-69 yea	HIV/AIDS	Number	2003	390.429	398.399	382.306
Deaths	Southern L	Both	50-69 yea	HIV/AIDS	Rate	2003	4.34306	4.43171	4.2527
Deaths	North Afric	Both	50-69 yea	HIV/AIDS	Number	2003	720.916	1282.98	407.407
Deaths	North Afric	Both	50-69 yea	HIV/AIDS	Rate	2003	1.66106	2.95611	0.9387
Deaths	Eastern Eu	Both	50-69 yea	Sexually tr	Number	2015	89.2389	109.288	83.6361
Deaths	Eastern Eu	Both	50-69 yea	Sexually tr	Rate	2015	0.16416	0.20104	0.15385
Deaths	Caribbean	Both	50-69 yea	HIV/AIDS	Number	2003	3346.15	4012.36	2801.03
Deaths	Caribbean	Both	50-69 yea	HIV/AIDS	Rate	2003	58.3647	69.9851	48.8567
Deaths	South Asia	Both	50-69 yea	Sexually tr	Number	2007	984.345	1165.41	742.307
Deaths	South Asia	Both	50-69 yea	Sexually tr	Rate	2007	0.59484	0.70426	0.44858
Deaths	Central Eu	Both	50-69 yea	Sexually tr	Number	2008	10.7903	11.6496	9.96322
Deaths	Central Eu	Both	50-69 yea	Sexually tr	Rate	2008	0.03801	0.04103	0.03509
Deaths	Eastern Su	Both	50-69 yea	HIV/AIDS	Number	2006	82200.3	92266.4	72977.8
Deaths	Eastern Su	Both	50-69 yea	HIV/AIDS	Rate	2006	431.225	484.031	382.843
Deaths	East Asia	Both	50-69 yea	HIV/AIDS	Number	2016	9713.49	10813.1	8771.04
Deaths	East Asia	Both	50-69 yea	HIV/AIDS	Rate	2016	2.81101	3.12922	2.53828
Deaths	Andean La	Both	50-69 yea	HIV/AIDS	Number	2005	458.241	704.781	354.79
Deaths	Andean La	Both	50-69 yea	HIV/AIDS	Rate	2005	8.03135	12.3523	6.21822
Deaths	Western S	Both	50-69 yea	Sexually tr	Number	2015	41.1254	54.3236	29.8095
Deaths	Western S	Both	50-69 yea	Sexually tr	Rate	2015	0.13643	0.18022	0.09889
Deaths	Tropical La	Both	50-69 yea	Sexually tr	Number	2007	52.4911	55.5118	49.4259
Deaths	Tropical La	Both	50-69 yea	Sexually tr	Rate	2007	0.18916	0.20005	0.17811
Deaths	Central Su	Both	50-69 yea	Sexually tr	Number	2011	92.6631	204.422	26.4502
Deaths	Central Su	Both	50-69 yea	Sexually tr	Rate	2011	1.24061	2.73688	0.35413
Deaths	Tropical La	Both	50-69 yea	Sexually tr	Number	2008	52.697	55.6695	49.6376
Deaths	Tropical La	Both	50-69 yea	Sexually tr	Rate	2008	0.18309	0.19342	0.17246
Deaths	Central Eu	Both	50-69 yea	Sexually tr	Number	1998	12.6268	13.4537	11.6338
Deaths	Central Eu	Both	50-69 yea	Sexually tr	Rate	1998	0.05106	0.0544	0.04704
Deaths	Central As	Both	50-69 yea	HIV/AIDS	Number	2011	122.176	131.98	114.659
Deaths	Central As	Both	50-69 yea	HIV/AIDS	Rate	2011	1.13182	1.22264	1.06218
Deaths	East Asia	Both	50-69 yea	HIV/AIDS	Number	2002	2606	3318.11	2194.18
Deaths	East Asia	Both	50-69 yea	HIV/AIDS	Rate	2002	1.22069	1.55425	1.02779
Deaths	Oceania	Both	50-69 yea	HIV/AIDS	Number	2002	256.93	529.943	109.993
Deaths	Oceania	Both	50-69 yea	HIV/AIDS	Rate	2002	35.1241	72.447	15.0369
Deaths	Southern S	Both	50-69 yea	Sexually tr	Number	2012	56.9614	89.3684	45.8431
Deaths	Southern S	Both	50-69 yea	Sexually tr	Rate	2012	0.68822	1.07977	0.55389
Deaths	Caribbean	Both	50-69 yea	Sexually tr	Number	2002	19.0499	29.9429	13.2045
Deaths	Caribbean	Both	50-69 yea	Sexually tr	Rate	2002	0.34095	0.53591	0.23633
Deaths	High-incor	Both	50-69 yea	Sexually tr	Number	2003	18.7907	20.442	17.6503
Deaths	High-incor	Both	50-69 yea	Sexually tr	Rate	2003	0.04257	0.04631	0.03999
Deaths	Eastern Eu	Both	50-69 yea	Sexually tr	Number	2002	215.527	238.961	137.326
Deaths	Eastern Eu	Both	50-69 yea	Sexually tr	Rate	2002	0.47496	0.5266	0.30263
Deaths	High-incor	Both	50-69 yea	HIV/AIDS	Number	2001	3782.94	3824.38	3739.2
Deaths	High-incor	Both	50-69 yea	HIV/AIDS	Rate	2001	6.48293	6.55394	6.40798
Deaths	Central La	Both	50-69 yea	HIV/AIDS	Number	2003	1833.74	1867.8	1797.6
Deaths	Central La	Both	50-69 yea	HIV/AIDS	Rate	2003	8.13047	8.28153	7.97026
Deaths	Eastern Su	Both	50-69 yea	Sexually tr	Number	2011	370.627	579.067	165.868
Deaths	Eastern Su	Both	50-69 yea	Sexually tr	Rate	2011	1.66677	2.60415	0.74593
Deaths	Oceania	Both	50-69 yea	Sexually tr	Number	2009	2.47339	5.80372	1.05236
Deaths	Oceania	Both	50-69 yea	Sexually tr	Rate	2009	0.26282	0.61671	0.11182
Deaths	Central Su	Both	50-69 yea	Sexually tr	Number	2004	94.6189	213.222	24.5316
Deaths	Central Su	Both	50-69 yea	Sexually tr	Rate	2004	1.63298	3.6799	0.42338
Deaths	Australasi	Both	50-69 yea	HIV/AIDS	Number	2010	36.3678	37.7834	35.007
Deaths	Australasi	Both	50-69 yea	HIV/AIDS	Rate	2010	0.63489	0.6596	0.61113
Deaths	Oceania	Both	50-69 yea	HIV/AIDS	Number	2003	315.894	664.032	132.123
Deaths	Oceania	Both	50-69 yea	HIV/AIDS	Rate	2003	41.7902	87.8462	17.4788
Deaths	Andean La	Both	50-69 yea	Sexually tr	Number	2012	10.2194	13.7234	7.32391
Deaths	Andean La	Both	50-69 yea	Sexually tr	Rate	2012	0.1402	0.18827	0.10047
Deaths	North Afric	Both	50-69 yea	Sexually tr	Number	2000	20.0302	26.7283	14.844
Deaths	North Afric	Both	50-69 yea	Sexually tr	Rate	2000	0.05054	0.06744	0.03745
Deaths	Tropical La	Both	50-69 yea	HIV/AIDS	Number	2003	2345.73	2387.56	2305.46
Deaths	Tropical La	Both	50-69 yea	HIV/AIDS	Rate	2003	9.79483	9.96948	9.62668
Deaths	Southeast	Both	50-69 yea	HIV/AIDS	Number	2007	9061.97	11451.8	7570.32
Deaths	Southeast	Both	50-69 yea	HIV/AIDS	Rate	2007	12.2547	15.4866	10.2375
Deaths	North Afric	Both	50-69 yea	HIV/AIDS	Number	2007	942.337	1707.99	544.635
Deaths	North Afric	Both	50-69 yea	HIV/AIDS	Rate	2007	1.8917	3.42872	1.09333
Deaths	Central As	Both	50-69 yea	Sexually tr	Number	2016	21.7056	27.9427	18.1187
Deaths	Central As	Both	50-69 yea	Sexually tr	Rate	2016	0.15916	0.20489	0.13286
Deaths	Western E	Both	50-69 yea	HIV/AIDS	Number	2016	1463.46	1498.19	1432.08
Deaths	Western E	Both	50-69 yea	HIV/AIDS	Rate	2016	1.32941	1.36096	1.30091
Deaths	High-incor	Both	50-69 yea	HIV/AIDS	Number	2006	194.519	198.882	190.073
Deaths	High-incor	Both	50-69 yea	HIV/AIDS	Rate	2006	0.43041	0.44006	0.42057
Deaths	Central As	Both	50-69 yea	Sexually tr	Number	2008	24.2338	29.5987	21.1133
Deaths	Central As	Both	50-69 yea	Sexually tr	Rate	2008	0.25501	0.31146	0.22217
Deaths	High-incor	Both	50-69 yea	Sexually tr	Number	2004	19.0316	20.5273	17.9111
Deaths	High-incor	Both	50-69 yea	Sexually tr	Rate	2004	0.04274	0.0461	0.04023
Deaths	South Asia	Both	50-69 yea	Sexually tr	Number	2003	974.385	1173.09	750.649
Deaths	South Asia	Both	50-69 yea	Sexually tr	Rate	2003	0.66598	0.80179	0.51306
Deaths	Oceania	Both	50-69 yea	HIV/AIDS	Number	2016	703.736	1840.78	295.989
Deaths	Oceania	Both	50-69 yea	HIV/AIDS	Rate	2016	56.7011	148.314	23.8483
Deaths	Southern L	Both	50-69 yea	Sexually tr	Number	2001	13.6243	15.2763	12.1531
Deaths	Southern L	Both	50-69 yea	Sexually tr	Rate	2001	0.15728	0.17636	0.1403
Deaths	Southern L	Both	50-69 yea	HIV/AIDS	Number	2015	689.627	705.732	674.05
Deaths	Southern L	Both	50-69 yea	HIV/AIDS	Rate	2015	5.86122	5.9981	5.72883
Deaths	High-incor	Both	50-69 yea	Sexually tr	Number	2016	43.7368	46.4309	41.6573
Deaths	High-incor	Both	50-69 yea	Sexually tr	Rate	2016	0.04866	0.05166	0.04635
Deaths	Central As	Both	50-69 yea	Sexually tr	Number	1997	43.4553	48.812	30.338
Deaths	Central As	Both	50-69 yea	Sexually tr	Rate	1997	0.53545	0.60145	0.37382
Deaths	Eastern Su	Both	50-69 yea	HIV/AIDS	Number	2003	88773.2	98425.5	79921.7
Deaths	Eastern Su	Both	50-69 yea	HIV/AIDS	Rate	2003	503.357	558.088	453.168
Deaths	Southern L	Both	50-69 yea	Sexually tr	Number	2002	13.262	14.9052	11.8283
Deaths	Southern L	Both	50-69 yea	Sexually tr	Rate	2002	0.15042	0.16906	0.13416
Deaths	Caribbean	Both	50-69 yea	HIV/AIDS	Number	2002	3345.64	4043.76	2765.63
Deaths	Caribbean	Both	50-69 yea	HIV/AIDS	Rate	2002	59.8791	72.3739	49.4983
Deaths	Eastern Eu	Both	50-69 yea	HIV/AIDS	Number	2010	1878.25	1914.56	1840.8
Deaths	Eastern Eu	Both	50-69 yea	HIV/AIDS	Rate	2010	3.82117	3.89503	3.74497
Deaths	High-incor	Both	50-69 yea	HIV/AIDS	Number	2011	228.914	234.85	223.436
Deaths	High-incor	Both	50-69 yea	HIV/AIDS	Rate	2011	0.4868	0.49942	0.47515
Deaths	Caribbean	Both	50-69 yea	HIV/AIDS	Number	2010	2654.14	3162.65	2233.71
Deaths	Caribbean	Both	50-69 yea	HIV/AIDS	Rate	2010	39.3317	46.8674	33.1013
Deaths	Australasi	Both	50-69 yea	Sexually tr	Number	2010	1.26449	1.60131	1.06591
Deaths	Australasi	Both	50-69 yea	Sexually tr	Rate	2010	0.02207	0.02795	0.01861
Deaths	North Afric	Both	50-69 yea	Sexually tr	Number	2006	21.2544	29.0965	15.4647
Deaths	North Afric	Both	50-69 yea	Sexually tr	Rate	2006	0.04422	0.06053	0.03217
Deaths	Oceania	Both	50-69 yea	Sexually tr	Number	2012	2.74596	6.12188	1.14911
Deaths	Oceania	Both	50-69 yea	Sexually tr	Rate	2012	0.25922	0.5779	0.10848
Deaths	Oceania	Both	50-69 yea	Sexually tr	Number	2003	2.00011	4.12716	0.82174
Deaths	Oceania	Both	50-69 yea	Sexually tr	Rate	2003	0.2646	0.54599	0.10871
Deaths	Western S	Both	50-69 yea	HIV/AIDS	Number	2006	36735.2	40711.7	33103.2
Deaths	Western S	Both	50-69 yea	HIV/AIDS	Rate	2006	163.201	180.867	147.065
Deaths	High-incor	Both	50-69 yea	HIV/AIDS	Number	2003	4255.14	4300.48	4208.04
Deaths	High-incor	Both	50-69 yea	HIV/AIDS	Rate	2003	6.83459	6.90743	6.75895
Deaths	North Afric	Both	50-69 yea	Sexually tr	Number	2002	20.7481	27.4864	15.3563
Deaths	North Afric	Both	50-69 yea	Sexually tr	Rate	2002	0.04934	0.06536	0.03652
Deaths	Southeast	Both	50-69 yea	HIV/AIDS	Number	2010	9147.85	11606.2	7548.25
Deaths	Southeast	Both	50-69 yea	HIV/AIDS	Rate	2010	10.9976	13.953	9.07457
Deaths	Caribbean	Both	50-69 yea	Sexually tr	Number	2010	22.1878	33.3998	14.7698
Deaths	Caribbean	Both	50-69 yea	Sexually tr	Rate	2010	0.3288	0.49495	0.21887
Deaths	Central As	Both	50-69 yea	HIV/AIDS	Number	2009	98.3336	107.584	91.1056
Deaths	Central As	Both	50-69 yea	HIV/AIDS	Rate	2009	0.99609	1.08979	0.92287
Deaths	Eastern Eu	Both	50-69 yea	Sexually tr	Number	2010	90.6152	104.883	86.4069
Deaths	Eastern Eu	Both	50-69 yea	Sexually tr	Rate	2010	0.18435	0.21338	0.17579
Deaths	North Afric	Both	50-69 yea	HIV/AIDS	Number	2012	1272.36	2495.69	740.728
Deaths	North Afric	Both	50-69 yea	HIV/AIDS	Rate	2012	2.10857	4.1359	1.22755
Deaths	High-incor	Both	50-69 yea	HIV/AIDS	Number	2001	152.013	155.708	148.693
Deaths	High-incor	Both	50-69 yea	HIV/AIDS	Rate	2001	0.3523	0.36086	0.3446
Deaths	Tropical La	Both	50-69 yea	HIV/AIDS	Number	2007	2770.4	2818.35	2722.58
Deaths	Tropical La	Both	50-69 yea	HIV/AIDS	Rate	2007	9.9836	10.1564	9.81126
Deaths	East Asia	Both	50-69 yea	Sexually tr	Number	2009	207.371	239.996	170.817
Deaths	East Asia	Both	50-69 yea	Sexually tr	Rate	2009	0.07702	0.08914	0.06345
Deaths	Oceania	Both	50-69 yea	HIV/AIDS	Number	2011	551.515	1331.54	197.989
Deaths	Oceania	Both	50-69 yea	HIV/AIDS	Rate	2011	54.1542	130.746	19.4409
Deaths	High-incor	Both	50-69 yea	Sexually tr	Number	2011	17.123	18.5266	16.0275
Deaths	High-incor	Both	50-69 yea	Sexually tr	Rate	2011	0.03641	0.0394	0.03408
Deaths	Caribbean	Both	50-69 yea	HIV/AIDS	Number	2017	2419.04	2843.33	2058.61
Deaths	Caribbean	Both	50-69 yea	HIV/AIDS	Rate	2017	29.7049	34.9151	25.2789
Deaths	South Asia	Both	50-69 yea	HIV/AIDS	Number	2017	8876.7	14649	6121.3
Deaths	South Asia	Both	50-69 yea	HIV/AIDS	Rate	2017	3.85463	6.36121	2.65812
Deaths	Central Su	Both	50-69 yea	Sexually tr	Number	2017	94.5721	217.428	28.0202
Deaths	Central Su	Both	50-69 yea	Sexually tr	Rate	2017	0.98774	2.27089	0.29265
Deaths	Western S	Both	50-69 yea	Sexually tr	Number	2007	39.1706	50.6004	28.1422
Deaths	Western S	Both	50-69 yea	Sexually tr	Rate	2007	0.16896	0.21826	0.12139
Deaths	Southeast	Both	50-69 yea	HIV/AIDS	Number	2002	9517.21	10450.7	8472.32
Deaths	Southeast	Both	50-69 yea	HIV/AIDS	Rate	2002	15.2928	16.7929	13.6139
Deaths	Southeast	Both	50-69 yea	Sexually tr	Number	2000	95.582	127.263	62.813
Deaths	Southeast	Both	50-69 yea	Sexually tr	Rate	2000	0.16319	0.21728	0.10724
Deaths	Eastern Eu	Both	50-69 yea	Sexually tr	Number	2000	287.181	327.055	142.875
Deaths	Eastern Eu	Both	50-69 yea	Sexually tr	Rate	2000	0.64084	0.72982	0.31882
Deaths	North Afric	Both	50-69 yea	Sexually tr	Number	2010	21.9916	30.1124	15.853
Deaths	North Afric	Both	50-69 yea	Sexually tr	Rate	2010	0.03948	0.05406	0.02846
Deaths	Southern S	Both	50-69 yea	HIV/AIDS	Number	2017	46588.8	54194.5	40472.8
Deaths	Southern S	Both	50-69 yea	HIV/AIDS	Rate	2017	490.728	570.841	426.308
Deaths	North Afric	Both	50-69 yea	Sexually tr	Number	2017	34.9702	46.1278	19.0749
Deaths	North Afric	Both	50-69 yea	Sexually tr	Rate	2017	0.04737	0.06248	0.02584
Deaths	Central Eu	Both	50-69 yea	HIV/AIDS	Number	2018	133.508	163.409	122.902
Deaths	Central Eu	Both	50-69 yea	HIV/AIDS	Rate	2018	0.44613	0.54605	0.41069
Deaths	Tropical La	Both	50-69 yea	Sexually tr	Number	2018	68.7088	73.8971	61.1405
Deaths	Tropical La	Both	50-69 yea	Sexually tr	Rate	2018	0.17062	0.1835	0.15182
Deaths	Southern S	Both	50-69 yea	Sexually tr	Number	2013	56.4778	86.3545	45.9868
Deaths	Southern S	Both	50-69 yea	Sexually tr	Rate	2013	0.66286	1.01351	0.53973
Deaths	East Asia	Both	50-69 yea	HIV/AIDS	Number	2003	2708.35	3362.25	2338.69
Deaths	East Asia	Both	50-69 yea	HIV/AIDS	Rate	2003	1.21927	1.51365	1.05285
Deaths	South Asia	Both	50-69 yea	Sexually tr	Number	2001	1008.34	1215.19	747.996
Deaths	South Asia	Both	50-69 yea	Sexually tr	Rate	2001	0.73057	0.88044	0.54194
Deaths	Central La	Both	50-69 yea	HIV/AIDS	Number	2002	1670.91	1702.81	1635.54
Deaths	Central La	Both	50-69 yea	HIV/AIDS	Rate	2002	7.68879	7.83561	7.52606
Deaths	Western E	Both	50-69 yea	Sexually tr	Number	2004	40.4962	50.1693	38.1779
Deaths	Western E	Both	50-69 yea	Sexually tr	Rate	2004	0.04374	0.05418	0.04123
Deaths	Western E	Both	50-69 yea	HIV/AIDS	Number	2010	1510.69	1533.72	1487.56
Deaths	Western E	Both	50-69 yea	HIV/AIDS	Rate	2010	1.5168	1.53992	1.49358
Deaths	South Asia	Both	50-69 yea	HIV/AIDS	Number	2003	12486.7	14137	11159.2
Deaths	South Asia	Both	50-69 yea	HIV/AIDS	Rate	2003	8.53448	9.66248	7.6272
Deaths	Central As	Both	50-69 yea	Sexually tr	Number	2001	31.4345	35.4085	26.4933
Deaths	Central As	Both	50-69 yea	Sexually tr	Rate	2001	0.39532	0.44529	0.33318
Deaths	Andean La	Both	50-69 yea	Sexually tr	Number	2013	10.2107	13.6913	7.19426
Deaths	Andean La	Both	50-69 yea	Sexually tr	Rate	2013	0.13483	0.18079	0.095
Deaths	Central Eu	Both	50-69 yea	HIV/AIDS	Number	2011	159.31	202.816	141.47
Deaths	Central Eu	Both	50-69 yea	HIV/AIDS	Rate	2011	0.54564	0.69465	0.48454
Deaths	Central Eu	Both	50-69 yea	HIV/AIDS	Number	2003	138.148	149.264	131.759
Deaths	Central Eu	Both	50-69 yea	HIV/AIDS	Rate	2003	0.52569	0.56799	0.50138
Deaths	High-incor	Both	50-69 yea	HIV/AIDS	Number	2013	3993.13	4033.47	3952.21
Deaths	High-incor	Both	50-69 yea	HIV/AIDS	Rate	2013	4.69329	4.7407	4.64519
Deaths	North Afric	Both	50-69 yea	HIV/AIDS	Number	2013	1343.32	2669.41	791.226
Deaths	North Afric	Both	50-69 yea	HIV/AIDS	Rate	2013	2.13696	4.24652	1.25869
Deaths	North Afric	Both	50-69 yea	HIV/AIDS	Number	2010	1082.77	2121.47	608.835
Deaths	North Afric	Both	50-69 yea	HIV/AIDS	Rate	2010	1.94391	3.80873	1.09305
Deaths	High-incor	Both	50-69 yea	HIV/AIDS	Number	2004	4373.14	4421.88	4321.93
Deaths	High-incor	Both	50-69 yea	HIV/AIDS	Rate	2004	6.78347	6.85907	6.70404
Deaths	Southern L	Both	50-69 yea	Sexually tr	Number	2003	13.2731	14.9137	11.8602
Deaths	Southern L	Both	50-69 yea	Sexually tr	Rate	2003	0.14765	0.1659	0.13193
Deaths	Andean La	Both	50-69 yea	HIV/AIDS	Number	2003	412.109	702.388	310.019
Deaths	Andean La	Both	50-69 yea	HIV/AIDS	Rate	2003	7.73236	13.1788	5.81686
Deaths	Western S	Both	50-69 yea	Sexually tr	Number	2001	49.4598	64.7278	32.5797
Deaths	Western S	Both	50-69 yea	Sexually tr	Rate	2001	0.25045	0.32776	0.16497
Deaths	Central As	Both	50-69 yea	Sexually tr	Number	2014	21.0584	26.7916	17.8274
Deaths	Central As	Both	50-69 yea	Sexually tr	Rate	2014	0.16833	0.21415	0.1425
Deaths	Southern L	Both	50-69 yea	HIV/AIDS	Number	2010	530.053	540.603	519.651
Deaths	Southern L	Both	50-69 yea	HIV/AIDS	Rate	2010	5.01691	5.11677	4.91846
Deaths	Caribbean	Both	50-69 yea	HIV/AIDS	Number	2000	3132.82	3908.76	2476.38
Deaths	Caribbean	Both	50-69 yea	HIV/AIDS	Rate	2000	59.0657	73.6953	46.6893
Deaths	Central La	Both	50-69 yea	HIV/AIDS	Number	2011	2588.63	2702.62	2494.79
Deaths	Central La	Both	50-69 yea	HIV/AIDS	Rate	2011	8.46281	8.83546	8.15601
Deaths	Southern S	Both	50-69 yea	HIV/AIDS	Number	2002	56973.9	69721.7	42980.7
Deaths	Southern S	Both	50-69 yea	HIV/AIDS	Rate	2002	902.098	1103.94	680.536
Deaths	Southeast	Both	50-69 yea	HIV/AIDS	Number	2004	10276.8	11571	8994.78
Deaths	Southeast	Both	50-69 yea	HIV/AIDS	Rate	2004	15.4883	17.4389	13.5562
Deaths	Eastern Eu	Both	50-69 yea	Sexually tr	Number	2012	83.4877	96.0165	79.3691
Deaths	Eastern Eu	Both	50-69 yea	Sexually tr	Rate	2012	0.16379	0.18837	0.15571
Deaths	Eastern Eu	Both	50-69 yea	Sexually tr	Number	2004	148.423	159.665	129.534
Deaths	Eastern Eu	Both	50-69 yea	Sexually tr	Rate	2004	0.31932	0.34351	0.27868
Deaths	Southern S	Both	50-69 yea	HIV/AIDS	Number	2010	53822.8	64446.4	44046.9
Deaths	Southern S	Both	50-69 yea	HIV/AIDS	Rate	2010	687.899	823.677	562.955
Deaths	Southeast	Both	50-69 yea	Sexually tr	Number	2003	98.6614	130.314	61.9849
Deaths	Southeast	Both	50-69 yea	Sexually tr	Rate	2003	0.15363	0.20292	0.09652
Deaths	Eastern Eu	Both	50-69 yea	HIV/AIDS	Number	2009	1676.82	1708.38	1644.59
Deaths	Eastern Eu	Both	50-69 yea	HIV/AIDS	Rate	2009	3.46164	3.52678	3.3951
Deaths	Central Eu	Both	50-69 yea	Sexually tr	Number	2007	10.9105	11.7574	10.0968
Deaths	Central Eu	Both	50-69 yea	Sexually tr	Rate	2007	0.03892	0.04195	0.03602
Deaths	Andean La	Both	50-69 yea	HIV/AIDS	Number	2009	454.236	713.303	344.659
Deaths	Andean La	Both	50-69 yea	HIV/AIDS	Rate	2009	6.93382	10.8884	5.26115
Deaths	Eastern Eu	Both	50-69 yea	Sexually tr	Number	2001	248.287	278.937	139.497
Deaths	Eastern Eu	Both	50-69 yea	Sexually tr	Rate	2001	0.55215	0.62031	0.31022
Deaths	Central Eu	Both	50-69 yea	Sexually tr	Number	2003	10.9133	11.731	10.0749
Deaths	Central Eu	Both	50-69 yea	Sexually tr	Rate	2003	0.04153	0.04464	0.03834
Deaths	Western S	Both	50-69 yea	HIV/AIDS	Number	2008	33709.4	37345	30029
Deaths	Western S	Both	50-69 yea	HIV/AIDS	Rate	2008	141.075	156.29	125.673
Deaths	Central La	Both	50-69 yea	Sexually tr	Number	2011	45.0293	47.5466	42.3054
Deaths	Central La	Both	50-69 yea	Sexually tr	Rate	2011	0.14721	0.15544	0.13831
Deaths	Central As	Both	50-69 yea	Sexually tr	Number	2013	21.8835	27.3792	18.7235
Deaths	Central As	Both	50-69 yea	Sexually tr	Rate	2013	0.18397	0.23017	0.15741
Deaths	Central Eu	Both	50-69 yea	Sexually tr	Number	2014	9.74583	10.8684	8.86013
Deaths	Central Eu	Both	50-69 yea	Sexually tr	Rate	2014	0.03275	0.03652	0.02977
Deaths	East Asia	Both	50-69 yea	HIV/AIDS	Number	2011	4214.37	4828.66	3773.86
Deaths	East Asia	Both	50-69 yea	HIV/AIDS	Rate	2011	1.49032	1.70755	1.33454
Deaths	High-incor	Both	50-69 yea	Sexually tr	Number	2010	17.5823	18.8669	16.4875
Deaths	High-incor	Both	50-69 yea	Sexually tr	Rate	2010	0.03758	0.04033	0.03524
Deaths	Western S	Both	50-69 yea	Sexually tr	Number	2002	46.4401	59.4797	31.4803
Deaths	Western S	Both	50-69 yea	Sexually tr	Rate	2002	0.22955	0.29401	0.15561
Deaths	Andean La	Both	50-69 yea	Sexually tr	Number	2016	10.3725	14.3762	7.2486
Deaths	Andean La	Both	50-69 yea	Sexually tr	Rate	2016	0.12329	0.17088	0.08616
Deaths	East Asia	Both	50-69 yea	Sexually tr	Number	2006	215.471	260.954	182.101
Deaths	East Asia	Both	50-69 yea	Sexually tr	Rate	2006	0.08629	0.10451	0.07293
Deaths	Central As	Both	50-69 yea	HIV/AIDS	Number	2006	88.9613	93.4972	84.5859
Deaths	Central As	Both	50-69 yea	HIV/AIDS	Rate	2006	0.99563	1.04639	0.94666
Deaths	Tropical La	Both	50-69 yea	Sexually tr	Number	1994	41.5882	45.4252	39.0611
Deaths	Tropical La	Both	50-69 yea	Sexually tr	Rate	1994	0.23219	0.25361	0.21808
Deaths	Southern S	Both	50-69 yea	Sexually tr	Number	2014	56.9641	86.6551	46.9391
Deaths	Southern S	Both	50-69 yea	Sexually tr	Rate	2014	0.64952	0.98807	0.53521
Deaths	Australasi	Both	50-69 yea	HIV/AIDS	Number	2015	30.087	31.3587	28.8799
Deaths	Australasi	Both	50-69 yea	HIV/AIDS	Rate	2015	0.46804	0.48782	0.44926
Deaths	Australasi	Both	50-69 yea	HIV/AIDS	Number	2005	46.4488	48.3664	44.473
Deaths	Australasi	Both	50-69 yea	HIV/AIDS	Rate	2005	0.92998	0.96837	0.89042
Deaths	Australasi	Both	50-69 yea	Sexually tr	Number	2004	1.43175	1.86936	1.21443
Deaths	Australasi	Both	50-69 yea	Sexually tr	Rate	2004	0.02956	0.0386	0.02507
Deaths	Southern L	Both	50-69 yea	Sexually tr	Number	2012	14.6254	16.5046	12.9868
Deaths	Southern L	Both	50-69 yea	Sexually tr	Rate	2012	0.1323	0.1493	0.11748
Deaths	East Asia	Both	50-69 yea	Sexually tr	Number	2005	222.252	266.677	188.453
Deaths	East Asia	Both	50-69 yea	Sexually tr	Rate	2005	0.09226	0.1107	0.07823
Deaths	Eastern Eu	Both	50-69 yea	HIV/AIDS	Number	2004	1368.65	1395.38	1344.59
Deaths	Eastern Eu	Both	50-69 yea	HIV/AIDS	Rate	2004	2.94453	3.00204	2.89278
Deaths	Western E	Both	50-69 yea	Sexually tr	Number	2016	38.0217	42.6546	35.3748
Deaths	Western E	Both	50-69 yea	Sexually tr	Rate	2016	0.03454	0.03875	0.03213
Deaths	Southern S	Both	50-69 yea	Sexually tr	Number	2016	56.0618	83.2744	45.8795
Deaths	Southern S	Both	50-69 yea	Sexually tr	Rate	2016	0.60574	0.89976	0.49572
Deaths	Western E	Both	50-69 yea	HIV/AIDS	Number	1998	1324.44	1346.58	1303.97
Deaths	Western E	Both	50-69 yea	HIV/AIDS	Rate	1998	1.53544	1.5611	1.5117
Deaths	Eastern Eu	Both	50-69 yea	HIV/AIDS	Number	2003	1358.78	1385.96	1334.51
Deaths	Eastern Eu	Both	50-69 yea	HIV/AIDS	Rate	2003	2.96675	3.02609	2.91375
Deaths	Eastern Eu	Both	50-69 yea	HIV/AIDS	Number	2012	2167.65	2211.35	2126.33
Deaths	Eastern Eu	Both	50-69 yea	HIV/AIDS	Rate	2012	4.2527	4.33843	4.17163
Deaths	Southeast	Both	50-69 yea	Sexually tr	Number	2012	101.13	138.058	61.8895
Deaths	Southeast	Both	50-69 yea	Sexually tr	Rate	2012	0.11235	0.15338	0.06876
Deaths	Eastern Eu	Both	50-69 yea	Sexually tr	Number	2006	106.526	120.636	99.9744
Deaths	Eastern Eu	Both	50-69 yea	Sexually tr	Rate	2006	0.22423	0.25394	0.21044
Deaths	Western E	Both	50-69 yea	HIV/AIDS	Number	1999	1287.96	1308.12	1265.92
Deaths	Western E	Both	50-69 yea	HIV/AIDS	Rate	1999	1.47378	1.49685	1.44856
Deaths	South Asia	Both	50-69 yea	HIV/AIDS	Number	2012	13196.8	17985.1	10162.8
Deaths	South Asia	Both	50-69 yea	HIV/AIDS	Rate	2012	6.7399	9.1854	5.19036
Deaths	Central La	Both	50-69 yea	HIV/AIDS	Number	2010	2531.74	2635.56	2446.76
Deaths	Central La	Both	50-69 yea	HIV/AIDS	Rate	2010	8.59456	8.947	8.30608
Deaths	Central La	Both	50-69 yea	HIV/AIDS	Number	2008	2368.42	2449.97	2297.37
Deaths	Central La	Both	50-69 yea	HIV/AIDS	Rate	2008	8.68153	8.98048	8.42108
Deaths	Southeast	Both	50-69 yea	Sexually tr	Number	2004	100.799	133.647	62.1401
Deaths	Southeast	Both	50-69 yea	Sexually tr	Rate	2004	0.15192	0.20142	0.09365
Deaths	Andean La	Both	50-69 yea	Sexually tr	Number	2014	10.3342	14.1658	7.30106
Deaths	Andean La	Both	50-69 yea	Sexually tr	Rate	2014	0.13145	0.18018	0.09287
Deaths	Oceania	Both	50-69 yea	HIV/AIDS	Number	2013	601.517	1492.9	231.406
Deaths	Oceania	Both	50-69 yea	HIV/AIDS	Rate	2013	54.5682	135.432	20.9926
Deaths	Australasi	Both	50-69 yea	HIV/AIDS	Number	2012	37.962	39.5065	36.4936
Deaths	Australasi	Both	50-69 yea	HIV/AIDS	Rate	2012	0.62953	0.65514	0.60518
Deaths	North Afric	Both	50-69 yea	Sexually tr	Number	2014	29.8006	39.0751	17.6564
Deaths	North Afric	Both	50-69 yea	Sexually tr	Rate	2014	0.04551	0.05967	0.02696
Deaths	Western E	Both	50-69 yea	Sexually tr	Number	2011	37.7449	42.8372	35.4051
Deaths	Western E	Both	50-69 yea	Sexually tr	Rate	2011	0.03739	0.04243	0.03507
Deaths	Western S	Both	50-69 yea	Sexually tr	Number	2012	39.0971	50.8948	28.9634
Deaths	Western S	Both	50-69 yea	Sexually tr	Rate	2012	0.14387	0.18729	0.10658
Deaths	High-incor	Both	50-69 yea	HIV/AIDS	Number	2016	220.356	228.075	213.622
Deaths	High-incor	Both	50-69 yea	HIV/AIDS	Rate	2016	0.44697	0.46263	0.43331
Deaths	Eastern Su	Both	50-69 yea	Sexually tr	Number	2017	355.803	554.937	169.122
Deaths	Eastern Su	Both	50-69 yea	Sexually tr	Rate	2017	1.29888	2.02584	0.61739
Deaths	Central Su	Both	50-69 yea	HIV/AIDS	Number	2004	10749	12547.4	9210.52
Deaths	Central Su	Both	50-69 yea	HIV/AIDS	Rate	2004	185.512	216.549	158.96
Deaths	East Asia	Both	50-69 yea	HIV/AIDS	Number	2013	4639.03	5349.11	4118.04
Deaths	East Asia	Both	50-69 yea	HIV/AIDS	Rate	2013	1.52492	1.75834	1.35367
Deaths	Southern L	Both	50-69 yea	HIV/AIDS	Number	2004	404.485	412.917	395.718
Deaths	Southern L	Both	50-69 yea	HIV/AIDS	Rate	2004	4.40646	4.49833	4.31095
Deaths	High-incor	Both	50-69 yea	HIV/AIDS	Number	2016	3839.42	3897.95	3783.95
Deaths	High-incor	Both	50-69 yea	HIV/AIDS	Rate	2016	4.27152	4.33665	4.20982
Deaths	East Asia	Both	50-69 yea	HIV/AIDS	Number	2015	8070.37	8972.44	7187.3
Deaths	East Asia	Both	50-69 yea	HIV/AIDS	Rate	2015	2.43019	2.70183	2.16428
Deaths	North Afric	Both	50-69 yea	HIV/AIDS	Number	2004	782.587	1365.7	446.463
Deaths	North Afric	Both	50-69 yea	HIV/AIDS	Rate	2004	1.74469	3.04467	0.99534
Deaths	Southeast	Both	50-69 yea	HIV/AIDS	Number	2017	10449.5	14295.6	8498.23
Deaths	Southeast	Both	50-69 yea	HIV/AIDS	Rate	2017	9.67758	13.2395	7.87041
Deaths	Tropical La	Both	50-69 yea	Sexually tr	Number	2003	50.0217	53.4708	46.9492
Deaths	Tropical La	Both	50-69 yea	Sexually tr	Rate	2003	0.20887	0.22327	0.19604
Deaths	Central La	Both	50-69 yea	Sexually tr	Number	2003	30.7815	33.9507	28.7104
Deaths	Central La	Both	50-69 yea	Sexually tr	Rate	2003	0.13648	0.15053	0.1273
Deaths	Andean La	Both	50-69 yea	Sexually tr	Number	2017	10.5947	14.8719	7.32116
Deaths	Andean La	Both	50-69 yea	Sexually tr	Rate	2017	0.12197	0.17121	0.08428
Deaths	Western S	Both	50-69 yea	Sexually tr	Number	2017	42.6913	56.1576	31.739
Deaths	Western S	Both	50-69 yea	Sexually tr	Rate	2017	0.13127	0.17267	0.09759
Deaths	Eastern Su	Both	50-69 yea	HIV/AIDS	Number	2013	52336.2	61891.9	46349.4
Deaths	Eastern Su	Both	50-69 yea	HIV/AIDS	Rate	2013	220.008	260.178	194.841
Deaths	Central As	Both	50-69 yea	Sexually tr	Number	2011	23.8256	28.9574	20.653
Deaths	Central As	Both	50-69 yea	Sexually tr	Rate	2011	0.22072	0.26826	0.19133
Deaths	Western E	Both	50-69 yea	HIV/AIDS	Number	2011	1519.75	1544.76	1495.64
Deaths	Western E	Both	50-69 yea	HIV/AIDS	Rate	2011	1.50531	1.53009	1.48143
Deaths	High-incor	Both	50-69 yea	HIV/AIDS	Number	2005	4485.82	4535.16	4435.25
Deaths	High-incor	Both	50-69 yea	HIV/AIDS	Rate	2005	6.71531	6.78918	6.63961
Deaths	Central As	Both	50-69 yea	HIV/AIDS	Number	2013	129.397	139.36	121.817
Deaths	Central As	Both	50-69 yea	HIV/AIDS	Rate	2013	1.08782	1.17158	1.02409
Deaths	East Asia	Both	50-69 yea	Sexually tr	Number	2013	202.106	231.526	156.571
Deaths	East Asia	Both	50-69 yea	Sexually tr	Rate	2013	0.06644	0.07611	0.05147
Deaths	South Asia	Both	50-69 yea	Sexually tr	Number	2006	965.682	1148.62	743.253
Deaths	South Asia	Both	50-69 yea	Sexually tr	Rate	2006	0.60261	0.71676	0.46381
Deaths	Australasi	Both	50-69 yea	HIV/AIDS	Number	2006	53.6868	55.8342	51.4689
Deaths	Australasi	Both	50-69 yea	HIV/AIDS	Rate	2006	1.04353	1.08527	1.00042
Deaths	High-incor	Both	50-69 yea	HIV/AIDS	Number	2013	246.483	252.476	240.459
Deaths	High-incor	Both	50-69 yea	HIV/AIDS	Rate	2013	0.51802	0.53061	0.50536
Deaths	Oceania	Both	50-69 yea	HIV/AIDS	Number	2018	794.308	2072.3	340.85
Deaths	Oceania	Both	50-69 yea	HIV/AIDS	Rate	2018	59.5267	155.302	25.5438
Deaths	Southern S	Both	50-69 yea	Sexually tr	Number	2017	55.6141	84.1046	45.5888
Deaths	Southern S	Both	50-69 yea	Sexually tr	Rate	2017	0.58579	0.88589	0.4802
Deaths	Central As	Both	50-69 yea	HIV/AIDS	Number	2019	143.964	157.014	133.2
Deaths	Central As	Both	50-69 yea	HIV/AIDS	Rate	2019	0.9597	1.04669	0.88794
Deaths	Central As	Both	50-69 yea	Sexually tr	Number	2018	22.5708	29.2348	18.352
Deaths	Central As	Both	50-69 yea	Sexually tr	Rate	2018	0.15506	0.20084	0.12608
Deaths	Central Eu	Both	50-69 yea	HIV/AIDS	Number	2019	127.935	153.892	119.296
Deaths	Central Eu	Both	50-69 yea	HIV/AIDS	Rate	2019	0.42799	0.51482	0.39909
Deaths	High-incor	Both	50-69 yea	HIV/AIDS	Number	2019	3522.21	3591.24	3461.13
Deaths	High-incor	Both	50-69 yea	HIV/AIDS	Rate	2019	3.86721	3.943	3.80015
Deaths	East Asia	Both	50-69 yea	HIV/AIDS	Number	2005	3679.62	4327.44	3362.17
Deaths	East Asia	Both	50-69 yea	HIV/AIDS	Rate	2005	1.52749	1.79642	1.39571
Deaths	Central La	Both	50-69 yea	HIV/AIDS	Number	2018	2799.46	2989.78	2635.86
Deaths	Central La	Both	50-69 yea	HIV/AIDS	Rate	2018	7.229	7.72045	6.80653
Deaths	North Afric	Both	50-69 yea	HIV/AIDS	Number	2014	1403.11	2797.7	830.438
Deaths	North Afric	Both	50-69 yea	HIV/AIDS	Rate	2014	2.14268	4.27234	1.26815
Deaths	South Asia	Both	50-69 yea	HIV/AIDS	Number	2004	14340.5	16243	12772.8
Deaths	South Asia	Both	50-69 yea	HIV/AIDS	Rate	2004	9.51636	10.7789	8.47607
Deaths	North Afric	Both	50-69 yea	Sexually tr	Number	2003	21.0761	28.1042	15.4129
Deaths	North Afric	Both	50-69 yea	Sexually tr	Rate	2003	0.04856	0.06475	0.03551
Deaths	Southern L	Both	50-69 yea	Sexually tr	Number	2019	14.2434	16.6752	12.1355
Deaths	Southern L	Both	50-69 yea	Sexually tr	Rate	2019	0.11323	0.13257	0.09648
Deaths	East Asia	Both	50-69 yea	Sexually tr	Number	2007	210.029	246.16	179.239
Deaths	East Asia	Both	50-69 yea	Sexually tr	Rate	2007	0.08163	0.09567	0.06966
Deaths	Eastern Su	Both	50-69 yea	HIV/AIDS	Number	2007	74977.1	86112.4	65361.2
Deaths	Eastern Su	Both	50-69 yea	HIV/AIDS	Rate	2007	382.402	439.194	333.358
Deaths	Eastern Su	Both	50-69 yea	HIV/AIDS	Number	2010	59852.8	71489.2	52308.7
Deaths	Eastern Su	Both	50-69 yea	HIV/AIDS	Rate	2010	278.063	332.123	243.015
Deaths	East Asia	Both	50-69 yea	HIV/AIDS	Number	2008	3916.23	4489.98	3534.9
Deaths	East Asia	Both	50-69 yea	HIV/AIDS	Rate	2008	1.48638	1.70415	1.34165
Deaths	Southeast	Both	50-69 yea	HIV/AIDS	Number	2011	9372.93	12358.5	7685.33
Deaths	Southeast	Both	50-69 yea	HIV/AIDS	Rate	2011	10.833	14.2836	8.88248
Deaths	Central La	Both	50-69 yea	Sexually tr	Number	2006	34.3685	36.7055	32.3584
Deaths	Central La	Both	50-69 yea	Sexually tr	Rate	2006	0.13602	0.14527	0.12807
Deaths	Eastern Eu	Both	50-69 yea	Sexually tr	Number	2007	99.6402	114.586	94.6451
Deaths	Eastern Eu	Both	50-69 yea	Sexually tr	Rate	2007	0.20867	0.23997	0.19821
Deaths	Australasi	Both	50-69 yea	HIV/AIDS	Number	2007	51.731	53.7701	49.738
Deaths	Australasi	Both	50-69 yea	HIV/AIDS	Rate	2007	0.97789	1.01644	0.94022
Deaths	Oceania	Both	50-69 yea	HIV/AIDS	Number	2008	451.323	1089.97	143.55
Deaths	Oceania	Both	50-69 yea	HIV/AIDS	Rate	2008	49.787	120.238	15.8355
Deaths	Southern L	Both	50-69 yea	Sexually tr	Number	2009	13.1475	14.8279	11.7533
Deaths	Southern L	Both	50-69 yea	Sexually tr	Rate	2009	0.12737	0.14365	0.11386
Deaths	South Asia	Both	50-69 yea	HIV/AIDS	Number	2019	8752.32	14588.7	6092.19
Deaths	South Asia	Both	50-69 yea	HIV/AIDS	Rate	2019	3.56989	5.95043	2.48488
Deaths	Eastern Su	Both	50-69 yea	Sexually tr	Number	2019	355.806	554.944	174.413
Deaths	Eastern Su	Both	50-69 yea	Sexually tr	Rate	2019	1.20462	1.87882	0.59049
Deaths	Caribbean	Both	50-69 yea	HIV/AIDS	Number	2019	2376.27	2777.18	2027.96
Deaths	Caribbean	Both	50-69 yea	HIV/AIDS	Rate	2019	27.9912	32.7137	23.8882
Deaths	Tropical La	Both	50-69 yea	Sexually tr	Number	2009	54.2132	57.018	50.7799
Deaths	Tropical La	Both	50-69 yea	Sexually tr	Rate	2009	0.18162	0.19101	0.17012
Deaths	Central Su	Both	50-69 yea	Sexually tr	Number	2019	96.1026	216.775	29.6179
Deaths	Central Su	Both	50-69 yea	Sexually tr	Rate	2019	0.92636	2.08956	0.28549
Deaths	Southern S	Both	50-69 yea	HIV/AIDS	Number	2003	63301.9	75398.3	49542.1
Deaths	Southern S	Both	50-69 yea	HIV/AIDS	Rate	2003	976.016	1162.52	763.861
Deaths	Southeast	Both	50-69 yea	HIV/AIDS	Number	2005	10270.9	11828	8865.33
Deaths	Southeast	Both	50-69 yea	HIV/AIDS	Rate	2005	14.9552	17.2224	12.9086
Deaths	Southern L	Both	50-69 yea	Sexually tr	Number	2007	12.6956	14.2999	11.3311
Deaths	Southern L	Both	50-69 yea	Sexually tr	Rate	2007	0.12896	0.14526	0.1151
Deaths	High-incor	Both	50-69 yea	HIV/AIDS	Number	2004	163.028	167.009	159.433
Deaths	High-incor	Both	50-69 yea	HIV/AIDS	Rate	2004	0.36613	0.37507	0.35806
Deaths	High-incor	Both	50-69 yea	Sexually tr	Number	2014	41.4562	43.8008	39.508
Deaths	High-incor	Both	50-69 yea	Sexually tr	Rate	2014	0.04773	0.05043	0.04549
Deaths	Caribbean	Both	50-69 yea	HIV/AIDS	Number	2015	2701.54	3114.27	2343.59
Deaths	Caribbean	Both	50-69 yea	HIV/AIDS	Rate	2015	34.8485	40.1726	30.2312
Deaths	High-incor	Both	50-69 yea	Sexually tr	Number	2014	16.3618	17.6951	15.1932
Deaths	High-incor	Both	50-69 yea	Sexually tr	Rate	2014	0.03414	0.03692	0.0317
Deaths	Southeast	Both	50-69 yea	Sexually tr	Number	2014	102.315	136.672	64.6411
Deaths	Southeast	Both	50-69 yea	Sexually tr	Rate	2014	0.10527	0.14062	0.06651
Deaths	Central Su	Both	50-69 yea	Sexually tr	Number	1998	104.403	221.165	25.2671
Deaths	Central Su	Both	50-69 yea	Sexually tr	Rate	1998	2.0824	4.41131	0.50397
Deaths	Southern L	Both	50-69 yea	HIV/AIDS	Number	2011	531.266	542.07	520.289
Deaths	Southern L	Both	50-69 yea	HIV/AIDS	Rate	2011	4.91501	5.01496	4.81345
Deaths	Australasi	Both	50-69 yea	HIV/AIDS	Number	2011	46.898	48.8941	44.9854
Deaths	Australasi	Both	50-69 yea	HIV/AIDS	Rate	2011	0.79744	0.83138	0.76491
Deaths	Southern L	Both	50-69 yea	HIV/AIDS	Number	2007	454.082	463.385	444.523
Deaths	Southern L	Both	50-69 yea	HIV/AIDS	Rate	2007	4.61259	4.70709	4.51549
Deaths	Tropical La	Both	50-69 yea	HIV/AIDS	Number	2000	1845.24	1883.24	1809.78
Deaths	Tropical La	Both	50-69 yea	HIV/AIDS	Rate	2000	8.56754	8.74394	8.40285
Deaths	Western E	Both	50-69 yea	HIV/AIDS	Number	2000	1282.51	1304.32	1261.44
Deaths	Western E	Both	50-69 yea	HIV/AIDS	Rate	2000	1.4497	1.47434	1.42587
Deaths	South Asia	Both	50-69 yea	HIV/AIDS	Number	2002	10466.1	11919	9272.66
Deaths	South Asia	Both	50-69 yea	HIV/AIDS	Rate	2002	7.36434	8.38665	6.52459
Deaths	Australasi	Both	50-69 yea	Sexually tr	Number	2014	1.38135	1.69095	1.15877
Deaths	Australasi	Both	50-69 yea	Sexually tr	Rate	2014	0.02187	0.02678	0.01835
Deaths	Andean La	Both	50-69 yea	Sexually tr	Number	2018	10.6912	15.2533	7.26429
Deaths	Andean La	Both	50-69 yea	Sexually tr	Rate	2018	0.11932	0.17023	0.08107
Deaths	South Asia	Both	50-69 yea	HIV/AIDS	Number	2006	16745.2	19234.9	14528.7
Deaths	South Asia	Both	50-69 yea	HIV/AIDS	Rate	2006	10.4494	12.003	9.06626
Deaths	High-incor	Both	50-69 yea	HIV/AIDS	Number	2003	167.581	171.376	163.608
Deaths	High-incor	Both	50-69 yea	HIV/AIDS	Rate	2003	0.37964	0.38824	0.37065

Deaths	Central Eu	Both	50-69 yea	Sexually tr	Rate	1999	0.04958	0.05297	0.04539
Deaths	Central Su	Both	50-69 yea	Sexually tr	Number	2000	100.998	217.329	25.4382
Deaths	Central Su	Both	50-69 yea	Sexually tr	Rate	2000	1.96612	4.23073	0.4952
Deaths	Southeast	Both	50-69 yea	HIV/AIDS	Number	2006	9718	11829.8	8189.76
Deaths	Southeast	Both	50-69 yea	HIV/AIDS	Rate	2006	13.6531	16.62	11.506
Deaths	North Afric	Both	50-69 yea	Sexually tr	Number	2004	21.0676	28.0595	15.5846
Deaths	North Afric	Both	50-69 yea	Sexually tr	Rate	2004	0.04697	0.06256	0.03474
Deaths	High-incor	Both	50-69 yea	HIV/AIDS	Number	2009	4491.37	4537.19	4446.01
Deaths	High-incor	Both	50-69 yea	HIV/AIDS	Rate	2009	5.87907	5.93905	5.8197
Deaths	Eastern Su	Both	50-69 yea	HIV/AIDS	Number	2009	62488.9	74347.3	54027.9
Deaths	Eastern Su	Both	50-69 yea	HIV/AIDS	Rate	2009	299.719	356.595	259.137
Deaths	Central Su	Both	50-69 yea	HIV/AIDS	Number	1998	9145.89	11198.2	7352.49
Deaths	Central Su	Both	50-69 yea	HIV/AIDS	Rate	1998	182.422	223.358	146.651
Deaths	Oceania	Both	50-69 yea	Sexually tr	Number	2013	2.78365	5.99748	1.14864
Deaths	Oceania	Both	50-69 yea	Sexually tr	Rate	2013	0.25253	0.54408	0.1042
Deaths	Australasia	Both	50-69 yea	Sexually tr	Number	2013	1.38027	1.6896	1.15828
Deaths	Australasia	Both	50-69 yea	Sexually tr	Rate	2013	0.02232	0.02732	0.01873
Deaths	High-incor	Both	50-69 yea	Sexually tr	Number	2007	35.915	38.0011	34.3074
Deaths	High-incor	Both	50-69 yea	Sexually tr	Rate	2007	0.05019	0.0531	0.04794
Deaths	Andean La	Both	50-69 yea	HIV/AIDS	Number	2008	471.505	723.382	365.774
Deaths	Andean La	Both	50-69 yea	HIV/AIDS	Rate	2008	7.44901	11.4282	5.77863
Deaths	Western S	Both	50-69 yea	Sexually tr	Number	2014	41.0919	54.0669	30.152
Deaths	Western S	Both	50-69 yea	Sexually tr	Rate	2014	0.14145	0.18611	0.10379
Deaths	East Asia	Both	50-69 yea	HIV/AIDS	Number	2007	3503.6	4110.47	3121.58
Deaths	East Asia	Both	50-69 yea	HIV/AIDS	Rate	2007	1.3617	1.59756	1.21322
Deaths	Australasia	Both	50-69 yea	Sexually tr	Number	2016	1.34061	1.66604	1.1176
Deaths	Australasia	Both	50-69 yea	Sexually tr	Rate	2016	0.02054	0.02553	0.01712
Deaths	Central As	Both	50-69 yea	Sexually tr	Number	2019	23.0874	29.7835	18.5605
Deaths	Central As	Both	50-69 yea	Sexually tr	Rate	2019	0.15391	0.19854	0.12373
Deaths	Andean La	Both	50-69 yea	HIV/AIDS	Number	2015	689.752	1197.69	431.289
Deaths	Andean La	Both	50-69 yea	HIV/AIDS	Rate	2015	8.46925	14.7061	5.29566
Deaths	Central As	Both	50-69 yea	Sexually tr	Number	2006	25.2647	30.9493	22.1911
Deaths	Central As	Both	50-69 yea	Sexually tr	Rate	2006	0.28275	0.34638	0.24836
Deaths	Central Su	Both	50-69 yea	Sexually tr	Number	2015	94.5421	216.255	26.9765
Deaths	Central Su	Both	50-69 yea	Sexually tr	Rate	2015	1.07206	2.45222	0.3059
Deaths	North Afric	Both	50-69 yea	Sexually tr	Number	2015	32.7704	42.8984	18.1651
Deaths	North Afric	Both	50-69 yea	Sexually tr	Rate	2015	0.04806	0.06292	0.02664
Deaths	Oceania	Both	50-69 yea	HIV/AIDS	Number	2019	822.597	2146.58	365.516
Deaths	Oceania	Both	50-69 yea	HIV/AIDS	Rate	2019	59.3948	154.991	26.3917
Deaths	Eastern Su	Both	50-69 yea	HIV/AIDS	Number	2004	89389.5	98088.6	81431.6
Deaths	Eastern Su	Both	50-69 yea	HIV/AIDS	Rate	2004	494.032	542.11	450.051
Deaths	North Afric	Both	50-69 yea	Sexually tr	Number	2007	21.3753	29.1515	15.5056
Deaths	North Afric	Both	50-69 yea	Sexually tr	Rate	2007	0.04291	0.05852	0.03113
Deaths	Caribbean	Both	50-69 yea	HIV/AIDS	Number	2012	2672.22	3128.48	2285.54
Deaths	Caribbean	Both	50-69 yea	HIV/AIDS	Rate	2012	37.5966	44.016	32.1563
Deaths	Central Su	Both	50-69 yea	Sexually tr	Number	2012	93.4537	202.803	26.8655
Deaths	Central Su	Both	50-69 yea	Sexually tr	Rate	2012	1.20099	2.60626	0.34525
Deaths	Eastern Su	Both	50-69 yea	Sexually tr	Number	2006	389.688	661.695	166.669
Deaths	Eastern Su	Both	50-69 yea	Sexually tr	Rate	2006	2.04431	3.47126	0.87435
Deaths	Southern L	Both	50-69 yea	Sexually tr	Number	2006	12.6519	14.2643	11.2656
Deaths	Southern L	Both	50-69 yea	Sexually tr	Rate	2006	0.13166	0.14844	0.11724
Deaths	East Asia	Both	50-69 yea	HIV/AIDS	Number	2006	3553.73	4222.27	3187.26
Deaths	East Asia	Both	50-69 yea	HIV/AIDS	Rate	2006	1.42318	1.69091	1.27641
Deaths	Central Eu	Both	50-69 yea	Sexually tr	Number	2019	9.16084	11.1133	7.59841
Deaths	Central Eu	Both	50-69 yea	Sexually tr	Rate	2019	0.03065	0.03718	0.02542
Deaths	South Asia	Both	50-69 yea	HIV/AIDS	Number	2009	15894.1	19566	12894.4
Deaths	South Asia	Both	50-69 yea	HIV/AIDS	Rate	2009	8.9879	11.0643	7.2916
Deaths	High-incor	Both	50-69 yea	Sexually tr	Number	2007	18.0149	19.3256	16.8886
Deaths	High-incor	Both	50-69 yea	Sexually tr	Rate	2007	0.03964	0.04253	0.03717
Deaths	Southern S	Both	50-69 yea	HIV/AIDS	Number	2004	68264.5	79649.3	55567
Deaths	Southern S	Both	50-69 yea	HIV/AIDS	Rate	2004	1025.39	1196.39	834.66
Deaths	Southeast	Both	50-69 yea	Sexually tr	Number	2008	103.111	143.566	62.7456
Deaths	Southeast	Both	50-69 yea	Sexually tr	Rate	2008	0.13412	0.18674	0.08162
Deaths	Eastern Su	Both	50-69 yea	HIV/AIDS	Number	2005	87470	96840.6	78692.3
Deaths	Eastern Su	Both	50-69 yea	HIV/AIDS	Rate	2005	471.277	521.764	423.984
Deaths	Western S	Both	50-69 yea	HIV/AIDS	Number	2000	32211.2	39000.5	25840.7
Deaths	Western S	Both	50-69 yea	HIV/AIDS	Rate	2000	166.961	202.152	133.941
Deaths	North Afric	Both	50-69 yea	HIV/AIDS	Number	2006	891.901	1587.32	516.652
Deaths	North Afric	Both	50-69 yea	HIV/AIDS	Rate	2006	1.85554	3.30231	1.07486
Deaths	Southern L	Both	50-69 yea	HIV/AIDS	Number	2006	416.026	425.428	407.433
Deaths	Southern L	Both	50-69 yea	HIV/AIDS	Rate	2006	4.32938	4.42722	4.23996
Deaths	Western S	Both	50-69 yea	HIV/AIDS	Number	2009	32063.6	35782.2	28433
Deaths	Western S	Both	50-69 yea	HIV/AIDS	Rate	2009	130.066	145.151	115.339
Deaths	Caribbean	Both	50-69 yea	Sexually tr	Number	2009	22.1558	33.4058	14.6387
Deaths	Caribbean	Both	50-69 yea	Sexually tr	Rate	2009	0.33601	0.50663	0.22201
Deaths	Oceania	Both	50-69 yea	HIV/AIDS	Number	2006	438.544	988.811	149.053
Deaths	Oceania	Both	50-69 yea	HIV/AIDS	Rate	2006	52.0872	117.444	17.7034
Deaths	Caribbean	Both	50-69 yea	HIV/AIDS	Number	1999	2980.26	3786.74	2297.9
Deaths	Caribbean	Both	50-69 yea	HIV/AIDS	Rate	1999	57.6673	73.2724	44.4638
Deaths	High-incor	Both	50-69 yea	HIV/AIDS	Number	2008	4594.1	4641.29	4547.81
Deaths	High-incor	Both	50-69 yea	HIV/AIDS	Rate	2008	6.20644	6.27019	6.14391
Deaths	Southern S	Both	50-69 yea	HIV/AIDS	Number	2015	43212.7	51728.3	36913.8
Deaths	Southern S	Both	50-69 yea	HIV/AIDS	Rate	2015	478.905	573.279	409.097
Deaths	Tropical La	Both	50-69 yea	Sexually tr	Number	2016	68.1582	72.4857	60.432
Deaths	Tropical La	Both	50-69 yea	Sexually tr	Rate	2016	0.17898	0.19035	0.15869
Deaths	Andean La	Both	50-69 yea	HIV/AIDS	Number	2006	499.181	749.487	390.648
Deaths	Andean La	Both	50-69 yea	HIV/AIDS	Rate	2006	8.45678	12.6973	6.61808
Deaths	Southeast	Both	50-69 yea	HIV/AIDS	Number	2008	8877.88	11134.5	7406.66
Deaths	Southeast	Both	50-69 yea	HIV/AIDS	Rate	2008	11.5479	14.4832	9.63421
Deaths	Central As	Both	50-69 yea	HIV/AIDS	Number	2007	92.2423	98.3094	86.5317
Deaths	Central As	Both	50-69 yea	HIV/AIDS	Rate	2007	1.00279	1.06874	0.94071
Deaths	High-incor	Both	50-69 yea	HIV/AIDS	Number	2007	209.67	214.57	204.906
Deaths	High-incor	Both	50-69 yea	HIV/AIDS	Rate	2007	0.46141	0.4722	0.45093
Deaths	East Asia	Both	50-69 yea	Sexually tr	Number	2014	201.623	230.761	152.991
Deaths	East Asia	Both	50-69 yea	Sexually tr	Rate	2014	0.06343	0.07259	0.04813
Deaths	High-incor	Both	50-69 yea	HIV/AIDS	Number	2014	232.183	238.548	225.67
Deaths	High-incor	Both	50-69 yea	HIV/AIDS	Rate	2014	0.48444	0.49772	0.47085
Deaths	Central Eu	Both	50-69 yea	HIV/AIDS	Number	2009	140.953	173.326	129.548
Deaths	Central Eu	Both	50-69 yea	HIV/AIDS	Rate	2009	0.49107	0.60386	0.45134
Deaths	Central La	Both	50-69 yea	Sexually tr	Number	2009	40.7389	43.1879	38.262
Deaths	Central La	Both	50-69 yea	Sexually tr	Rate	2009	0.14369	0.15232	0.13495
Deaths	Eastern Eu	Both	50-69 yea	HIV/AIDS	Number	2008	1662.62	1696.04	1628.87
Deaths	Eastern Eu	Both	50-69 yea	HIV/AIDS	Rate	2008	3.46176	3.53134	3.3915
Deaths	Central Eu	Both	50-69 yea	HIV/AIDS	Number	2013	141.22	181.485	121.152
Deaths	Central Eu	Both	50-69 yea	HIV/AIDS	Rate	2013	0.4772	0.61326	0.40939
Deaths	Western E	Both	50-69 yea	Sexually tr	Number	2013	37.1403	42.5816	34.68
Deaths	Western E	Both	50-69 yea	Sexually tr	Rate	2013	0.03552	0.04072	0.03316
Deaths	North Afric	Both	50-69 yea	Sexually tr	Number	2013	27.3132	36.2573	17.0786
Deaths	North Afric	Both	50-69 yea	Sexually tr	Rate	2013	0.04345	0.05768	0.02717
Deaths	South Asia	Both	50-69 yea	HIV/AIDS	Number	2015	10950.7	16229.8	8066.36
Deaths	South Asia	Both	50-69 yea	HIV/AIDS	Rate	2015	5.06142	7.50142	3.72828
Deaths	Central As	Both	50-69 yea	HIV/AIDS	Number	2016	148.381	159.569	139.243
Deaths	Central As	Both	50-69 yea	HIV/AIDS	Rate	2016	1.08801	1.17005	1.02101
Deaths	North Afric	Both	50-69 yea	HIV/AIDS	Number	2017	1569.79	3118.06	936.672
Deaths	North Afric	Both	50-69 yea	HIV/AIDS	Rate	2017	2.12635	4.22356	1.26876
Deaths	High-incor	Both	50-69 yea	HIV/AIDS	Number	2014	3967.69	4010.95	3922.94
Deaths	High-incor	Both	50-69 yea	HIV/AIDS	Rate	2014	4.56825	4.61805	4.51672
Deaths	Western E	Both	50-69 yea	Sexually tr	Number	1998	50.9712	59.4715	47.681
Deaths	Western E	Both	50-69 yea	Sexually tr	Rate	1998	0.05909	0.06895	0.05528
Deaths	Caribbean	Both	50-69 yea	Sexually tr	Number	2008	20.9285	31.4299	14.5426
Deaths	Caribbean	Both	50-69 yea	Sexually tr	Rate	2008	0.32504	0.48813	0.22586
Deaths	Western S	Both	50-69 yea	HIV/AIDS	Number	1998	26216.9	33062.5	19903.2
Deaths	Western S	Both	50-69 yea	HIV/AIDS	Rate	1998	142.669	179.922	108.311
Deaths	Central As	Both	50-69 yea	Sexually tr	Number	2004	25.7948	30.4469	23.1177
Deaths	Central As	Both	50-69 yea	Sexually tr	Rate	2004	0.30492	0.35992	0.27328
Deaths	Southeast	Both	50-69 yea	Sexually tr	Number	2010	101.12	139.574	62.9788
Deaths	Southeast	Both	50-69 yea	Sexually tr	Rate	2010	0.12157	0.1678	0.07571
Deaths	Central Su	Both	50-69 yea	Sexually tr	Number	2009	92.9937	201.748	25.0869
Deaths	Central Su	Both	50-69 yea	Sexually tr	Rate	2009	1.34766	2.92372	0.36356
Deaths	Central Su	Both	50-69 yea	Sexually tr	Number	2001	99.3814	216.631	24.6702
Deaths	Central Su	Both	50-69 yea	Sexually tr	Rate	2001	1.87877	4.09533	0.46638
Deaths	Eastern Eu	Both	50-69 yea	Sexually tr	Number	2011	85.8838	99.928	81.648
Deaths	Eastern Eu	Both	50-69 yea	Sexually tr	Rate	2011	0.17162	0.19968	0.16316
Deaths	Caribbean	Both	50-69 yea	Sexually tr	Number	2011	22.461	34.0545	15.1268
Deaths	Caribbean	Both	50-69 yea	Sexually tr	Rate	2011	0.32493	0.49264	0.21883
Deaths	Tropical La	Both	50-69 yea	Sexually tr	Number	2010	55.7614	58.7953	52.0085
Deaths	Tropical La	Both	50-69 yea	Sexually tr	Rate	2010	0.18014	0.18994	0.16801
Deaths	Central Su	Both	50-69 yea	HIV/AIDS	Number	2011	9667.44	11544.9	8025.76
Deaths	Central Su	Both	50-69 yea	HIV/AIDS	Rate	2011	129.432	154.567	107.452
Deaths	North Afric	Both	50-69 yea	HIV/AIDS	Number	2011	1180.45	2303.02	678.675
Deaths	North Afric	Both	50-69 yea	HIV/AIDS	Rate	2011	2.03756	3.97522	1.17145
Deaths	South Asia	Both	50-69 yea	HIV/AIDS	Number	2018	8549.25	14412.9	5814.5
Deaths	South Asia	Both	50-69 yea	HIV/AIDS	Rate	2018	3.59845	6.06653	2.44737
Deaths	Central Su	Both	50-69 yea	HIV/AIDS	Number	2018	7760.03	9543.91	6317.55
Deaths	Central Su	Both	50-69 yea	HIV/AIDS	Rate	2018	77.8442	95.739	63.3741
Deaths	Eastern Eu	Both	50-69 yea	Sexually tr	Number	2017	82.7508	97.7007	76.9143
Deaths	Eastern Eu	Both	50-69 yea	Sexually tr	Rate	2017	0.15095	0.17822	0.1403
Deaths	Eastern Su	Both	50-69 yea	Sexually tr	Number	2012	368.214	573.966	167.841
Deaths	Eastern Su	Both	50-69 yea	Sexually tr	Rate	2012	1.6017	2.4967	0.73009
Deaths	Central La	Both	50-69 yea	Sexually tr	Number	2016	56.5179	60.3104	52.552
Deaths	Central La	Both	50-69 yea	Sexually tr	Rate	2016	0.15519	0.16561	0.1443
Deaths	South Asia	Both	50-69 yea	Sexually tr	Number	2017	1258.77	1597.34	890.615
Deaths	South Asia	Both	50-69 yea	Sexually tr	Rate	2017	0.54661	0.69363	0.38674
Deaths	Caribbean	Both	50-69 yea	Sexually tr	Number	2000	18.2929	28.9326	12.6187
Deaths	Caribbean	Both	50-69 yea	Sexually tr	Rate	2000	0.34489	0.54549	0.23791
Deaths	North Afric	Both	50-69 yea	HIV/AIDS	Number	2008	981.276	1833.9	551.976
Deaths	North Afric	Both	50-69 yea	HIV/AIDS	Rate	2008	1.89959	3.55013	1.06853
Deaths	North Afric	Both	50-69 yea	Sexually tr	Number	2008	21.7808	29.98	15.7288
Deaths	North Afric	Both	50-69 yea	Sexually tr	Rate	2008	0.04216	0.05804	0.03045
Deaths	Eastern Su	Both	50-69 yea	HIV/AIDS	Number	2011	57986.2	68803	51326
Deaths	Eastern Su	Both	50-69 yea	HIV/AIDS	Rate	2011	260.773	309.417	230.821
Deaths	Eastern Su	Both	50-69 yea	Sexually tr	Number	2008	381.832	619.448	167.758
Deaths	Eastern Su	Both	50-69 yea	Sexually tr	Rate	2008	1.88957	3.06545	0.83018
Deaths	Eastern Su	Both	50-69 yea	Sexually tr	Number	2004	410.593	758.655	167.158
Deaths	Eastern Su	Both	50-69 yea	Sexually tr	Rate	2004	2.26924	4.19289	0.92384
Deaths	South Asia	Both	50-69 yea	HIV/AIDS	Number	2008	16614.9	19728.3	13756.8
Deaths	South Asia	Both	50-69 yea	HIV/AIDS	Rate	2008	9.71615	11.5368	8.0448
Deaths	South Asia	Both	50-69 yea	Sexually tr	Number	2010	974.551	1160.53	752.377
Deaths	South Asia	Both	50-69 yea	Sexually tr	Rate	2010	0.5327	0.63436	0.41126
Deaths	High-incor	Both	50-69 yea	Sexually tr	Number	2006	35.2833	37.3915	33.7681
Deaths	High-incor	Both	50-69 yea	Sexually tr	Rate	2006	0.05104	0.05409	0.04885
Deaths	Western E	Both	50-69 yea	Sexually tr	Number	2000	45.2485	55.131	42.5767
Deaths	Western E	Both	50-69 yea	Sexually tr	Rate	2000	0.05115	0.06232	0.04813
Deaths	Southern S	Both	50-69 yea	HIV/AIDS	Number	2012	44806.4	55323	36816.5
Deaths	Southern S	Both	50-69 yea	HIV/AIDS	Rate	2012	541.363	668.427	444.826
Deaths	Southern L	Both	50-69 yea	HIV/AIDS	Number	2017	678.9	697.899	662.217
Deaths	Southern L	Both	50-69 yea	HIV/AIDS	Rate	2017	5.58169	5.73789	5.44453
Deaths	Australasia	Both	50-69 yea	Sexually tr	Number	2008	1.22103	1.59517	1.03058
Deaths	Australasia	Both	50-69 yea	Sexually tr	Rate	2008	0.02246	0.02934	0.01896
Deaths	Central Su	Both	50-69 yea	HIV/AIDS	Number	2017	8233.03	10234.2	6736.86
Deaths	Central Su	Both	50-69 yea	HIV/AIDS	Rate	2017	85.9886	106.889	70.3621
Deaths	Tropical La	Both	50-69 yea	Sexually tr	Number	2001	49.4314	53.0945	46.6301
Deaths	Tropical La	Both	50-69 yea	Sexually tr	Rate	2001	0.22183	0.23827	0.20926
Deaths	High-incor	Both	50-69 yea	HIV/AIDS	Number	2009	211.874	217.246	206.683
Deaths	High-incor	Both	50-69 yea	HIV/AIDS	Rate	2009	0.45676	0.46834	0.44556
Deaths	East Asia	Both	50-69 yea	HIV/AIDS	Number	2019	11133.8	13784.3	8702.5
Deaths	East Asia	Both	50-69 yea	HIV/AIDS	Rate	2019	2.92108	3.61647	2.28321
Deaths	Central Su	Both	50-69 yea	Sexually tr	Number	1999	103.28	222.765	25.7587
Deaths	Central Su	Both	50-69 yea	Sexually tr	Rate	1999	2.028	4.37421	0.5058
Deaths	Andean La	Both	50-69 yea	HIV/AIDS	Number	2007	478.007	718.716	365.14
Deaths	Andean La	Both	50-69 yea	HIV/AIDS	Rate	2007	7.81705	11.7534	5.97127
Deaths	East Asia	Both	50-69 yea	Sexually tr	Number	2008	208.595	242.157	170.487
Deaths	East Asia	Both	50-69 yea	Sexually tr	Rate	2008	0.07917	0.09191	0.06471
Deaths	Southern S	Both	50-69 yea	HIV/AIDS	Number	2007	68292.1	76241.4	61096.6
Deaths	Southern S	Both	50-69 yea	HIV/AIDS	Rate	2007	947.095	1057.34	847.305
Deaths	High-incor	Both	50-69 yea	HIV/AIDS	Number	2008	211.797	216.821	207.063
Deaths	High-incor	Both	50-69 yea	HIV/AIDS	Rate	2008	0.46211	0.47307	0.45178
Deaths	Australasia	Both	50-69 yea	Sexually tr	Number	2007	1.33988	1.77863	1.1227
Deaths	Australasia	Both	50-69 yea	Sexually tr	Rate	2007	0.02533	0.03362	0.02122
Deaths	Central La	Both	50-69 yea	Sexually tr	Number	2004	32.2513	34.9155	30.1166
Deaths	Central La	Both	50-69 yea	Sexually tr	Rate	2004	0.1377	0.14908	0.12859
Deaths	Central La	Both	50-69 yea	HIV/AIDS	Number	2012	2602.18	2734.37	2493.94
Deaths	Central La	Both	50-69 yea	HIV/AIDS	Rate	2012	8.20099	8.61758	7.85984
Deaths	Tropical La	Both	50-69 yea	HIV/AIDS	Number	2016	4630.37	4767.92	4499.82
Deaths	Tropical La	Both	50-69 yea	HIV/AIDS	Rate	2016	12.1593	12.5205	11.8165
Deaths	Western S	Both	50-69 yea	HIV/AIDS	Number	2017	34952.9	39408	31592.3
Deaths	Western S	Both	50-69 yea	HIV/AIDS	Rate	2017	107.473	121.172	97.1404
Deaths	South Asia	Both	50-69 yea	Sexually tr	Number	2011	997.919	1188.76	767.574
Deaths	South Asia	Both	50-69 yea	Sexually tr	Rate	2011	0.52725	0.62808	0.40555
Deaths	Central As	Both	50-69 yea	Sexually tr	Number	2009	24.0006	29.0186	20.8126
Deaths	Central As	Both	50-69 yea	Sexually tr	Rate	2009	0.24312	0.29395	0.21082
Deaths	High-incor	Both	50-69 yea	Sexually tr	Number	2009	17.6835	18.9601	16.5408
Deaths	High-incor	Both	50-69 yea	Sexually tr	Rate	2009	0.03812	0.04087	0.03566
Deaths	Central Su	Both	50-69 yea	Sexually tr	Number	2010	92.8961	201.885	25.7339
Deaths	Central Su	Both	50-69 yea	Sexually tr	Rate	2010	1.29491	2.81414	0.35871
Deaths	East Asia	Both	50-69 yea	Sexually tr	Number	2011	208.232	243.139	160.562
Deaths	East Asia	Both	50-69 yea	Sexually tr	Rate	2011	0.07364	0.08598	0.05678
Deaths	Oceania	Both	50-69 yea	HIV/AIDS	Number	2009	475.646	1165.22	158.347
Deaths	Oceania	Both	50-69 yea	HIV/AIDS	Rate	2009	50.5424	123.817	16.826
Deaths	East Asia	Both	50-69 yea	Sexually tr	Number	2019	221.925	273.785	156.355
Deaths	East Asia	Both	50-69 yea	Sexually tr	Rate	2019	0.05822	0.07183	0.04102
Deaths	Central Su	Both	50-69 yea	HIV/AIDS	Number	2000	9706.68	11583.6	8115.38
Deaths	Central Su	Both	50-69 yea	HIV/AIDS	Rate	2000	188.959	225.496	157.982
Deaths	Southeast	Both	50-69 yea	HIV/AIDS	Number	2012	9897.81	13444.5	8064.93
Deaths	Southeast	Both	50-69 yea	HIV/AIDS	Rate	2012	10.996	14.9362	8.95977
Deaths	Central Eu	Both	50-69 yea	HIV/AIDS	Number	1999	162.745	169.971	152.982
Deaths	Central Eu	Both	50-69 yea	HIV/AIDS	Rate	1999	0.65272	0.6817	0.61356
Deaths	Central Su	Both	50-69 yea	Sexually tr	Number	2014	94.4582	213.4	27.2462
Deaths	Central Su	Both	50-69 yea	Sexually tr	Rate	2014	1.11699	2.5235	0.32219
Deaths	Southeast	Both	50-69 yea	Sexually tr	Number	2013	101.089	134.983	62.7836
Deaths	Southeast	Both	50-69 yea	Sexually tr	Rate	2013	0.10802	0.14424	0.06709
Deaths	Andean La	Both	50-69 yea	HIV/AIDS	Number	2013	561.335	981.194	359.513
Deaths	Andean La	Both	50-69 yea	HIV/AIDS	Rate	2013	7.41232	12.9565	4.7473
Deaths	Tropical La	Both	50-69 yea	HIV/AIDS	Number	2012	3718.99	3809.22	3629.66
Deaths	Tropical La	Both	50-69 yea	HIV/AIDS	Rate	2012	11.1784	11.4497	10.9099
Deaths	South Asia	Both	50-69 yea	HIV/AIDS	Number	2013	12755.2	17855.4	9776.27
Deaths	South Asia	Both	50-69 yea	HIV/AIDS	Rate	2013	6.29772	8.81584	4.8269
Deaths	Southern L	Both	50-69 yea	Sexually tr	Number	2013	15.1683	17.1095	13.4028
Deaths	Southern L	Both	50-69 yea	Sexually tr	Rate	2013	0.13422	0.1514	0.1186
Deaths	Eastern Su	Both	50-69 yea	Sexually tr	Number	2013	364.837	565.652	167.724
Deaths	Eastern Su	Both	50-69 yea	Sexually tr	Rate	2013	1.53368	2.37786	0.70507
Deaths	East Asia	Both	50-69 yea	HIV/AIDS	Number	2012	4117.85	4885.56	3588.07
Deaths	East Asia	Both	50-69 yea	HIV/AIDS	Rate	2012	1.40898	1.67166	1.22771
Deaths	Southern S	Both	50-69 yea	HIV/AIDS	Number	2013	42293.5	52426.9	35056.6
Deaths	Southern S	Both	50-69 yea	HIV/AIDS	Rate	2013	496.381	615.313	411.445
Deaths	High-incor	Both	50-69 yea	Sexually tr	Number	2012	16.5585	17.9534	15.4907
Deaths	High-incor	Both	50-69 yea	Sexually tr	Rate	2012	0.03507	0.03802	0.03281
Deaths	Oceania	Both	50-69 yea	Sexually tr	Number	2014	2.82484	5.98875	1.17217
Deaths	Oceania	Both	50-69 yea	Sexually tr	Rate	2014	0.24627	0.52211	0.10219
Deaths	Central As	Both	50-69 yea	HIV/AIDS	Number	2014	164.789	175.187	155.795
Deaths	Central As	Both	50-69 yea	HIV/AIDS	Rate	2014	1.31722	1.40033	1.24532
Deaths	Central La	Both	50-69 yea	Sexually tr	Number	2014	52.4104	55.583	48.9982
Deaths	Central La	Both	50-69 yea	Sexually tr	Rate	2014	0.15388	0.1632	0.14386
Deaths	Western S	Both	50-69 yea	HIV/AIDS	Number	1999	29246.8	35945.6	22818.4
Deaths	Western S	Both	50-69 yea	HIV/AIDS	Rate	1999	156.162	191.93	121.838
Deaths	Western E	Both	50-69 yea	HIV/AIDS	Number	2014	1461.33	1488.17	1437.09
Deaths	Western E	Both	50-69 yea	HIV/AIDS	Rate	2014	1.37403	1.39927	1.35124
Deaths	Eastern Su	Both	50-69 yea	HIV/AIDS	Number	2014	50040.2	59260.7	44132
Deaths	Eastern Su	Both	50-69 yea	HIV/AIDS	Rate	2014	203.226	240.673	179.232
Deaths	Central Eu	Both	50-69 yea	Sexually tr	Number	2011	10.737	11.6819	9.80996
Deaths	Central Eu	Both	50-69 yea	Sexually tr	Rate	2011	0.03677	0.04001	0.0336
Deaths	Tropical La	Both	50-69 yea	HIV/AIDS	Number	2011	3504.6	3576.79	3435.04
Deaths	Tropical La	Both	50-69 yea	HIV/AIDS	Rate	2011	10.9182	11.1431	10.7015
Deaths	Eastern Eu	Both	50-69 yea	Sexually tr	Number	2019	80.4775	98.2211	68.3187
Deaths	Eastern Eu	Both	50-69 yea	Sexually tr	Rate	2019	0.14797	0.18059	0.12561
Deaths	Caribbean	Both	50-69 yea	Sexually tr	Number	2015	26.6441	38.1064	18.4469
Deaths	Caribbean	Both	50-69 yea	Sexually tr	Rate	2015	0.3437	0.49155	0.23796
Deaths	High-incor	Both	50-69 yea	Sexually tr	Number	2011	38.1597	40.3751	36.5615
Deaths	High-incor	Both	50-69 yea	Sexually tr	Rate	2011	0.047	0.04973	0.04503
Deaths	Central La	Both	50-69 yea	HIV/AIDS	Number	2016	2814.25	3010.85	2653.93
Deaths	Central La	Both	50-69 yea	HIV/AIDS	Rate	2016	7.72776	8.26763	7.28754
Deaths	Southeast	Both	50-69 yea	Sexually tr	Number	2017	104.102	137.866	66.4875
Deaths	Southeast	Both	50-69 yea	Sexually tr	Rate	2017	0.09641	0.12768	0.06158
Deaths	Southern L	Both	50-69 yea	Sexually tr	Number	2017	14.4435	16.8431	12.3886
Deaths	Southern L	Both	50-69 yea	Sexually tr	Rate	2017	0.11875	0.13848	0.10185
Deaths	Andean La	Both	50-69 yea	HIV/AIDS	Number	2017	656.611	1174.33	440.922
Deaths	Andean La	Both	50-69 yea	HIV/AIDS	Rate	2017	7.5589	13.5189	5.07589
Deaths	High-incor	Both	50-69 yea	HIV/AIDS	Number	2018	3622.24	3685.6	3556.86
Deaths	High-incor	Both	50-69 yea	HIV/AIDS	Rate	2018	3.99852	4.06847	3.92635
Deaths	Central La	Both	50-69 yea	Sexually tr	Number	2018	56.5885	64.0743	49.4019
Deaths	Central La	Both	50-69 yea	Sexually tr	Rate	2018	0.14613	0.16546	0.12757
Deaths	Australasia	Both	50-69 yea	HIV/AIDS	Number	2019	32.6876	34.2183	31.3143
Deaths	Australasia	Both	50-69 yea	HIV/AIDS	Rate	2019	0.48502	0.50773	0.46464
Deaths	Eastern Eu	Both	50-69 yea	HIV/AIDS	Number	2007	1571.74	1607.39	1539.89
Deaths	Eastern Eu	Both	50-69 yea	HIV/AIDS	Rate	2007	3.2916	3.36626	3.2249
Deaths	South Asia	Both	50-69 yea	HIV/AIDS	Number	2007	16923.1	19753.9	14312.1
Deaths	South Asia	Both	50-69 yea	HIV/AIDS	Rate	2007	10.2267	11.9373	8.64879
Deaths	Oceania	Both	50-69 yea	Sexually tr	Number	2008	2.41295	5.40052	1.01543
Deaths	Oceania	Both	50-69 yea	Sexually tr	Rate	2008	0.26618	0.59575	0.11202
Deaths	Western S	Both	50-69 yea	Sexually tr	Number	2009	37.6164	48.3927	26.5511
Deaths	Western S	Both	50-69 yea	Sexually tr	Rate	2009	0.15259	0.19631	0.1077
Deaths	Tropical La	Both	50-69 yea	Sexually tr	Number	2000	49.1225	52.5149	46.323
Deaths	Tropical La	Both	50-69 yea	Sexually tr	Rate	2000	0.22808	0.24383	0.21508
Deaths	Central Eu	Both	50-69 yea	HIV/AIDS	Number	2008	158.699	184.608	148.275
Deaths	Central Eu	Both	50-69 yea	HIV/AIDS	Rate	2008	0.559	0.65026	0.52228
Deaths	Australasia	Both	50-69 yea	HIV/AIDS	Number	2009	45.8382	47.9071	43.9807
Deaths	Australasia	Both	50-69 yea	HIV/AIDS	Rate	2009	0.82129	0.85836	0.78801
Deaths	Eastern Su	Both	50-69 yea	Sexually tr	Number	2015	362.943	564.506	169.519
Deaths	Eastern Su	Both	50-69 yea	Sexually tr	Rate	2015	1.42358	2.21418	0.66491
Deaths	Western E	Both	50-69 yea	Sexually tr	Number	2006	39.9957	48.0345	37.5959
Deaths	Western E	Both	50-69 yea	Sexually tr	Rate	2006	0.04222	0.05071	0.03969
Deaths	Central Su	Both	50-69 yea	HIV/AIDS	Number	2007	10183.3	12088.6	8495.02
Deaths	Central Su	Both	50-69 yea	HIV/AIDS	Rate	2007	158.91	188.642	132.565
Deaths	Oceania	Both	50-69 yea	HIV/AIDS	Number	2007	444.6	1036.65	138.791
Deaths	Oceania	Both	50-69 yea	HIV/AIDS	Rate	2007	50.8973	118.675	15.8887
Deaths	Eastern Su	Both	50-69 yea	Sexually tr	Number	2003	424.99	830.587	169.339
Deaths	Eastern Su	Both	50-69 yea	Sexually tr	Rate	2003	2.40976	4.70956	0.96018
Deaths	Southeast	Both	50-69 yea	HIV/AIDS	Number	2009	8958.35	11147.5	7450.01
Deaths	Southeast	Both	50-69 yea	HIV/AIDS	Rate	2009	11.2057	13.944	9.319
Deaths	High-incor	Both	50-69 yea	HIV/AIDS	Number	2007	4555.08	4601.78	4507.32
Deaths	High-incor	Both	50-69 yea	HIV/AIDS	Rate	2007	6.36514	6.4304	6.29841
Deaths	Western S	Both	50-69 yea	Sexually tr	Number	2004	43.3413	56.088	30.8754
Deaths	Western S	Both	50-69 yea	Sexually tr	Rate	2004	0.20376	0.26368	0.14515
Deaths	East Asia	Both	50-69 yea	HIV/AIDS	Number	2009	3910.27	4512.62	3546.62
Deaths	East Asia	Both	50-69 yea	HIV/AIDS	Rate	2009	1.45241	1.67614	1.31734
Deaths	Western S	Both	50-69 yea	Sexually tr	Number	2008	37.9227	48.9431	27.2084
Deaths	Western S	Both	50-69 yea	Sexually tr	Rate	2008	0.15871	0.20483	0.11387
Deaths	Western E	Both	50-69 yea	Sexually tr	Number	1999	48.9996	57.8344	45.9281
Deaths	Western E	Both	50-69 yea	Sexually tr	Rate	1999	0.05607	0.06618	0.05255
Deaths	Southeast	Both	50-69 yea	HIV/AIDS	Number	2019	9420	12883.4	7329.77
Deaths	Southeast	Both	50-69 yea	HIV/AIDS	Rate	2019	8.17941	11.1867	6.36445
Deaths	Andean La	Both	50-69 yea	HIV/AIDS	Number	2012	469.769	815.761	321.64
Deaths	Andean La	Both	50-69 yea	HIV/AIDS	Rate	2012	6.44458	11.1911	4.41246
Deaths	Central La	Both	50-69 yea	Sexually tr	Number	2013	50.6158	53.4068	47.2244
Deaths	Central La	Both	50-69 yea	Sexually tr	Rate	2013	0.15391	0.1624	0.1436
Deaths	Western S	Both	50-69 yea	HIV/AIDS	Number	2001	34559.3	41032.1	28380.7
Deaths	Western S	Both	50-69 yea	HIV/AIDS	Rate	2001	174.997	207.773	143.711
Deaths	Western S	Both	50-69 yea	HIV/AIDS	Number	2005	37896.1	42120.1	34264
Deaths	Western S	Both	50-69 yea	HIV/AIDS	Rate	2005	173.331	192.651	156.718
Deaths	Eastern Su	Both	50-69 yea	HIV/AIDS	Number	2018	45204.8	54721.1	38863.5
Deaths	Eastern Su	Both	50-69 yea	HIV/AIDS	Rate	2018	159.065	192.55	136.751
Deaths	North Afric	Both	50-69 yea	HIV/AIDS	Number	2019	1693.93	3334.38	1008.03
Deaths	North Afric	Both	50-69 yea	HIV/AIDS	Rate	2019	2.12557	4.18405	1.2649
Deaths	Southern L	Both	50-69 yea	HIV/AIDS	Number	2019	665.393	684.438	645.95
Deaths	Southern L	Both	50-69 yea	HIV/AIDS	Rate	2019	5.28977	5.44118	5.13521
Deaths	Eastern Eu	Both	50-69 yea	HIV/AIDS	Number	2013	2197.62	2232.17	2160.98
Deaths	Eastern Eu	Both	50-69 yea	HIV/AIDS	Rate	2013	4.20799	4.27414	4.13784
Deaths	Tropical La	Both	50-69 yea	HIV/AIDS	Number	2008	3017.55	3070.25	2965.4
Deaths	Tropical La	Both	50-69 yea	HIV/AIDS	Rate	2008	10.4843	10.6674	10.3031
Deaths	Eastern Eu	Both	50-69 yea	Sexually tr	Number	2009	91.2377	105.89	86.9571
Deaths	Eastern Eu	Both	50-69 yea	Sexually tr	Rate	2009	0.18835	0.2186	0.17951
Deaths	Southeast	Both	50-69 yea	Sexually tr	Number	2007	105.079	146.532	63.4751
Deaths	Southeast	Both	50-69 yea	Sexually tr	Rate	2007	0.1421	0.19816	0.08584
Deaths	Central As	Both	50-69 yea	HIV/AIDS	Number	2008	94.1452	102.171	87.4247
Deaths	Central As	Both	50-69 yea	HIV/AIDS	Rate	2008	0.99067	1.07512	0.91995
Deaths	North Afric	Both	50-69 yea	Sexually tr	Number	2011	22.8977	31.3132	16.0713
Deaths	North Afric	Both	50-69 yea	Sexually tr	Rate	2011	0.03952	0.05405	0.02774
Deaths	North Afric	Both	50-69 yea	HIV/AIDS	Number	2009	1016.28	1957.55	568.448
Deaths	North Afric	Both	50-69 yea	HIV/AIDS	Rate	2009	1.89553	3.65116	1.06025
Deaths	Southern L	Both	50-69 yea	Sexually tr	Number	2011	13.345	14.8722	11.8977
Deaths	Southern L	Both	50-69 yea	Sexually tr	Rate	2011	0.12346	0.13759	0.11007
Deaths	Central Eu	Both	50-69 yea	HIV/AIDS	Number	2000	163.333	170.297	153.752
Deaths	Central Eu	Both	50-69 yea	HIV/AIDS	Rate	2000	0.64768	0.67529	0.60968
Deaths	Eastern Su	Both	50-69 yea	HIV/AIDS	Number	2008	67576.7	79354.3	58204.2
Deaths	Eastern Su	Both	50-69 yea	HIV/AIDS	Rate	2008	334.416	392.699	288.034
Deaths	High-incor	Both	50-69 yea	HIV/AIDS	Number	2012	4003.82	4044.02	3962.35
Deaths	High-incor	Both	50-69 yea	HIV/AIDS	Rate	2012	4.80594	4.8542	4.75617
Deaths	South Asia	Both	50-69 yea	HIV/AIDS	Number	2011	13939.3	18567.8	10848.4
Deaths	South Asia	Both	50-69 yea	HIV/AIDS	Rate	2011	7.36483	9.81029	5.73177
Deaths	Tropical La	Both	50-69 yea	Sexually tr	Number	2011	59.8778	63.2511	55.4112
Deaths	Tropical La	Both	50-69 yea	Sexually tr	Rate	2011	0.18654	0.19705	0.17263
Deaths	Central Eu	Both	50-69 yea	Sexually tr	Number	2012	10.6928	11.7116	9.74657
Deaths	Central Eu	Both	50-69 yea	Sexually tr	Rate	2012	0.03636	0.03983	0.03315
Deaths	Australasia	Both	50-69 yea	Sexually tr	Number	2012	1.24232	1.55287	1.04266
Deaths	Australasia	Both	50-69 yea	Sexually tr	Rate	2012	0.0206	0.02575	0.01729
Deaths	Oceania	Both	50-69 yea	HIV/AIDS	Number	2015	655.375	1705.15	269.904
Deaths	Oceania	Both	50-69 yea	HIV/AIDS	Rate	2015	54.9258	142.905	22.6202
Deaths	Australasia	Both	50-69 yea	HIV/AIDS	Number	2002	44.2627	46.1146	42.4913
Deaths	Australasia	Both	50-69 yea	HIV/AIDS	Rate	2002	0.9732	1.01391	0.93425
Deaths	Caribbean	Both	50-69 yea	HIV/AIDS	Number	2001	3306.23	4062.07	2680.42
Deaths	Caribbean	Both	50-69 yea	HIV/AIDS	Rate	2001	60.7349	74.6195	49.2389
Deaths	Caribbean	Both	50-69 yea	Sexually tr	Number	2007	20.6465	31.3352	14.232
Deaths	Caribbean	Both	50-69 yea	Sexually tr	Rate	2007	0.32781	0.49752	0.22597
Deaths	Western E	Both	50-69 yea	Sexually tr	Number	2014	37.252	42.8733	34.6602
Deaths	Western E	Both	50-69 yea	Sexually tr	Rate	2014	0.03503	0.04031	0.03259
Deaths	Central Eu	Both	50-69 yea	HIV/AIDS	Number	2006	155.971	171.279	147.931
Deaths	Central Eu	Both	50-69 yea	HIV/AIDS	Rate	2006	0.56467	0.62009	0.53556
Deaths	Eastern Su	Both	50-69 yea	Sexually tr	Number	2009	379.831	612.659	166.04
Deaths	Eastern Su	Both	50-69 yea	Sexually tr	Rate	2009	1.8218	2.93853	0.79639
Deaths	Caribbean	Both	50-69 yea	HIV/AIDS	Number	2013	2680.8	3124.78	2302.81
Deaths	Caribbean	Both	50-69 yea	HIV/AIDS	Rate	2013	36.6349	42.7023	31.4695
Deaths	North Afric	Both	50-69 yea	Sexually tr	Number	2012	24.6757	33.2389	16.5658
Deaths	North Afric	Both	50-69 yea	Sexually tr	Rate	2012	0.04089	0.05508	0.02745
Deaths	Southern L	Both	50-69 yea	HIV/AIDS	Number	2014	656.155	669.164	644.066
Deaths	Southern L	Both	50-69 yea	HIV/AIDS	Rate	2014	5.68581	5.79854	5.58106
Deaths	Southeast	Both	50-69 yea	HIV/AIDS	Number	2013	10879.1	15406.2	8649.89
Deaths	Southeast	Both	50-69 yea	HIV/AIDS	Rate	2013	11.6255	16.4632	9.24334
Deaths	Western S	Both	50-69 yea	HIV/AIDS	Number	2015	34705.1	38301.8	31621.8
Deaths	Western S	Both	50-69 yea	HIV/AIDS	Rate	2015	115.133	127.065	104.904
Deaths	Central As	Both	50-69 yea	HIV/AIDS	Number	2004	74.1678	77.2228	71.1415
Deaths	Central As	Both	50-69 yea	HIV/AIDS	Rate	2004	0.87675	0.91286	0.84097
Deaths	Eastern Su	Both	50-69 yea	HIV/AIDS	Number	2012	55284.1	65362.5	49091.3
Deaths	Eastern Su	Both	50-69 yea	HIV/AIDS	Rate	2012	240.481	284.321	213.543
Deaths	Central Su	Both	50-69 yea	Sexually tr	Number	2013	94.1212	204.891	27.3113
Deaths	Central Su	Both	50-69 yea	Sexually tr	Rate	2013	1.16046	2.52618	0.33673
Deaths	Western E	Both	50-69 yea	HIV/AIDS	Number	2012	1548.37	1572.82	1522.33
Deaths	Western E	Both	50-69 yea	HIV/AIDS	Rate	2012	1.50666	1.53046	1.48132
Deaths	Central Eu	Both	50-69 yea	HIV/AIDS	Number	2014	165.517	203.113	146.441
Deaths	Central Eu	Both	50-69 yea	HIV/AIDS	Rate	2014	0.55618	0.68251	0.49208
Deaths	Andean La	Both	50-69 yea	HIV/AIDS	Number	2010	457.407	728.459	345.101
Deaths	Andean La	Both	50-69 yea	HIV/AIDS	Rate	2010	6.74404	10.7405	5.0882
Deaths	Tropical La	Both	50-69 yea	Sexually tr	Number	2014	64.5811	68.3455	58.4999
Deaths	Tropical La	Both	50-69 yea	Sexually tr	Rate	2014	0.18103	0.19158	0.16398
Deaths	Western S	Both	50-69 yea	HIV/AIDS	Number	2013	33233.6	36588.1	30160.4
Deaths	Western S	Both	50-69 yea	HIV/AIDS	Rate	2013	118.308	130.249	107.367
Deaths	High-incor	Both	50-69 yea	Sexually tr	Number	2013	16.5125	17.9234	15.3712
Deaths	High-incor	Both	50-69 yea	Sexually tr	Rate	2013	0.0347	0.03767	0.0323
Deaths	Western E	Both	50-69 yea	Sexually tr	Number	2015	37.6824	42.6891	35.1039
Deaths	Western E	Both	50-69 yea	Sexually tr	Rate	2015	0.03479	0.03941	0.03241
Deaths	Tropical La	Both	50-69 yea	HIV/AIDS	Number	2009	3220.66	3278.27	3166.51
Deaths	Tropical La	Both	50-69 yea	HIV/AIDS	Rate	2009	10.7894	10.9824	10.608
Deaths	Andean La	Both	50-69 yea	HIV/AIDS	Number	2014	640.477	1122.19	396.421
Deaths	Andean La	Both	50-69 yea	HIV/AIDS	Rate	2014	8.1466	14.2737	5.0423
Deaths	High-incor	Both	50-69 yea	HIV/AIDS	Number	2015	217.28	223.039	211.105
Deaths	High-incor	Both	50-69 yea	HIV/AIDS	Rate	2015	0.44583	0.45765	0.43316
Deaths	Western S	Both	50-69 yea	HIV/AIDS	Number	2010	31710.8	35267.5	28387.3
Deaths	Western S	Both	50-69 yea	HIV/AIDS	Rate	2010	124.623	138.601	111.561
Deaths	South Asia	Both	50-69 yea	Sexually tr	Number	2012	1017.22	1216.61	770.505
Deaths	South Asia	Both	50-69 yea	Sexually tr	Rate	2012	0.51952	0.62135	0.39351
Deaths	Southern L	Both	50-69 yea	Sexually tr	Number	2010	13.2175	14.8362	11.8376
Deaths	Southern L	Both	50-69 yea	Sexually tr	Rate	2010	0.1251	0.14042	0.11204
Deaths	Caribbean	Both	50-69 yea	Sexually tr	Number	2019	28.3639	42.3907	19.1254
Deaths	Caribbean	Both	50-69 yea	Sexually tr	Rate	2019	0.33411	0.49934	0.22529
Deaths	Tropical La	Both	50-69 yea	HIV/AIDS	Number	2006	2712.33	2759.3	2664.94
Deaths	Tropical La	Both	50-69 yea	HIV/AIDS	Rate	2006	10.1387	10.3143	9.96155
Deaths	Western E	Both	50-69 yea	Sexually tr	Number	2017	37.4556	42.1533	34.809
Deaths	Western E	Both	50-69 yea	Sexually tr	Rate	2017	0.03366	0.03789	0.03129
Deaths	High-incor	Both	50-69 yea	HIV/AIDS	Number	2017	215.918	223.855	208.308
Deaths	High-incor	Both	50-69 yea	HIV/AIDS	Rate	2017	0.43724	0.45331	0.42183
Deaths	South Asia	Both	50-69 yea	Sexually tr	Number	2009	951.827	1124.52	726.103
Deaths	South Asia	Both	50-69 yea	Sexually tr	Rate	2009	0.53825	0.6359	0.4106
Deaths	East Asia	Both	50-69 yea	HIV/AIDS	Number	2010	3975.67	4622.97	3529.65
Deaths	East Asia	Both	50-69 yea	HIV/AIDS	Rate	2010	1.44415	1.67928	1.28213
Deaths	South Asia	Both	50-69 yea	Sexually tr	Number	2014	1119.08	1349.27	824.927
Deaths	South Asia	Both	50-69 yea	Sexually tr	Rate	2014	0.53433	0.64423	0.39388
Deaths	Central Eu	Both	50-69 yea	Sexually tr	Number	2015	10.3711	11.6191	9.39731
Deaths	Central Eu	Both	50-69 yea	Sexually tr	Rate	2015	0.0347	0.03888	0.03144
Deaths	High-incor	Both	50-69 yea	HIV/AIDS	Number	2017	3736.61	3799.34	3676.2
Deaths	High-incor	Both	50-69 yea	HIV/AIDS	Rate	2017	4.13788	4.20734	4.07097
Deaths	High-incor	Both	50-69 yea	Sexually tr	Number	2015	16.1236	17.5031	14.8844
Deaths	High-incor	Both	50-69 yea	Sexually tr	Rate	2015	0.03308	0.03591	0.03054
Deaths	Eastern Eu	Both	50-69 yea	Sexually tr	Number	2014	84.5584	98.022	80.1966
Deaths	Eastern Eu	Both	50-69 yea	Sexually tr	Rate	2014	0.15831	0.18351	0.15014
Deaths	Central La	Both	50-69 yea	HIV/AIDS	Number	2015	2819.81	3010.58	2669.62
Deaths	Central La	Both	50-69 yea	HIV/AIDS	Rate	2015	7.99854	8.53967	7.57253
Deaths	Southeast	Both	50-69 yea	HIV/AIDS	Number	2014	11448.4	16259	9034.54
Deaths	Southeast	Both	50-69 yea	HIV/AIDS	Rate	2014	11.7794	16.7291	9.29574
Deaths	Southeast	Both	50-69 yea	Sexually tr	Number	2016	103.451	137.355	65.6637
Deaths	Southeast	Both	50-69 yea	Sexually tr	Rate	2016	0.09914	0.13163	0.06292
Deaths	South Asia	Both	50-69 yea	Sexually tr	Number	2018	1252.54	1605.75	852.269
Deaths	South Asia	Both	50-69 yea	Sexually tr	Rate	2018	0.52721	0.67587	0.35873
Deaths	Central As	Both	50-69 yea	Sexually tr	Number	2003	26.9768	30.5034	24.3904
Deaths	Central As	Both	50-69 yea	Sexually tr	Rate	2003	0.32686	0.36958	0.29552
Deaths	East Asia	Both	50-69 yea	Sexually tr	Number	2017	221.685	263.929	156.878
Deaths	East Asia	Both	50-69 yea	Sexually tr	Rate	2017	0.06185	0.07364	0.04377
Deaths	Caribbean	Both	50-69 yea	HIV/AIDS	Number	2016	2572.47	2994.07	2219.39
Deaths	Caribbean	Both	50-69 yea	HIV/AIDS	Rate	2016	32.3236	37.6211	27.887
Deaths	Southern L	Both	50-69 yea	HIV/AIDS	Number	2009	501.697	512.898	492.153
Deaths	Southern L	Both	50-69 yea	HIV/AIDS	Rate	2009	4.86024	4.96875	4.76778
Deaths	Caribbean	Both	50-69 yea	HIV/AIDS	Number	2011	2681.93	3158.42	2292.89
Deaths	Caribbean	Both	50-69 yea	HIV/AIDS	Rate	2011	38.7978	45.6908	33.1698
Deaths	Central La	Both	50-69 yea	HIV/AIDS	Number	2014	2769.79	2949.88	2630.81
Deaths	Central La	Both	50-69 yea	HIV/AIDS	Rate	2014	8.13235	8.66112	7.7243
Deaths	Central As	Both	50-69 yea	HIV/AIDS	Number	2012	136.521	146.686	128.703
Deaths	Central As	Both	50-69 yea	HIV/AIDS	Rate	2012	1.20609	1.29588	1.13702
Deaths	Tropical La	Both	50-69 yea	HIV/AIDS	Number	2010	3426.09	3488.04	3365.88
Deaths	Tropical La	Both	50-69 yea	HIV/AIDS	Rate	2010	11.068	11.2682	10.8735
Deaths	Caribbean	Both	50-69 yea	HIV/AIDS	Number	2018	2361.95	2772.23	2003.79
Deaths	Caribbean	Both	50-69 yea	HIV/AIDS	Rate	2018	28.4047	33.3387	24.0975
Deaths	Central Su	Both	50-69 yea	Sexually tr	Number	2018	95.1454	219.182	29.0337
Deaths	Central Su	Both	50-69 yea	Sexually tr	Rate	2018	0.95444	2.19871	0.29125
Deaths	Andean La	Both	50-69 yea	HIV/AIDS	Number	2018	621.022	1129.65	407.981
Deaths	Andean La	Both	50-69 yea	HIV/AIDS	Rate	2018	6.93083	12.6073	4.55322
Deaths	High-incor	Both	50-69 yea	Sexually tr	Number	2019	14.9247	16.4203	13.6939
Deaths	High-incor	Both	50-69 yea	Sexually tr	Rate	2019	0.03012	0.03313	0.02763
Deaths	Central Su	Both	50-69 yea	Sexually tr	Number	2016	94.5315	217.167	27.3836
Deaths	Central Su	Both	50-69 yea	Sexually tr	Rate	2016	1.02856	2.3629	0.29795
Deaths	Australasia	Both	50-69 yea	HIV/AIDS	Number	2014	33.2816	34.5675	31.8545
Deaths	Australasia	Both	50-69 yea	HIV/AIDS	Rate	2014	0.527	0.54736	0.5044
Deaths	Central As	Both	50-69 yea	Sexually tr	Number	2015	21.2774	27.0538	17.9039
Deaths	Central As	Both	50-69 yea	Sexually tr	Rate	2015	0.1625	0.20662	0.13674
Deaths	High-incor	Both	50-69 yea	Sexually tr	Number	2015	42.097	44.6577	40.1112
Deaths	High-incor	Both	50-69 yea	Sexually tr	Rate	2015	0.04756	0.05045	0.04532
Deaths	Andean La	Both	50-69 yea	HIV/AIDS	Number	2011	435.826	743.099	315.706
Deaths	Andean La	Both	50-69 yea	HIV/AIDS	Rate	2011	6.20532	10.5803	4.49505
Deaths	Eastern Eu	Both	50-69 yea	HIV/AIDS	Number	2015	2815.94	2863.38	2764.07
Deaths	Eastern Eu	Both	50-69 yea	HIV/AIDS	Rate	2015	5.18005	5.26732	5.08465
Deaths	Oceania	Both	50-69 yea	HIV/AIDS	Number	2014	621.52	1585.38	252.872
Deaths	Oceania	Both	50-69 yea	HIV/AIDS	Rate	2014	54.1851	138.216	22.0458
Deaths	Australasia	Both	50-69 yea	HIV/AIDS	Number	2013	32.2417	33.6852	30.9326
Deaths	Australasia	Both	50-69 yea	HIV/AIDS	Rate	2013	0.52137	0.54471	0.5002
Deaths	Southern L	Both	50-69 yea	Sexually tr	Number	2016	15.5656	17.8466	13.651
Deaths	Southern L	Both	50-69 yea	Sexually tr	Rate	2016	0.12997	0.14901	0.11398
Deaths	Andean La	Both	50-69 yea	HIV/AIDS	Number	2016	688.521	1204.55	443.317
Deaths	Andean La	Both	50-69 yea	HIV/AIDS	Rate	2016	8.18392	14.3175	5.26937
Deaths	North Afric	Both	50-69 yea	Sexually tr	Number	2016	34.5769	45.2928	18.5252
Deaths	North Afric	Both	50-69 yea	Sexually tr	Rate	2016	0.04872	0.06381	0.0261
Deaths	South Asia	Both	50-69 yea	HIV/AIDS	Number	2016	9799.89	15373.9	7001.3
Deaths	South Asia	Both	50-69 yea	HIV/AIDS	Rate	2016	4.38839	6.88442	3.13518
Deaths	East Asia	Both	50-69 yea	Sexually tr	Number	2012	207.683	238.527	160.691
Deaths	East Asia	Both	50-69 yea	Sexually tr	Rate	2012	0.07106	0.08162	0.05498
Deaths	Tropical La	Both	50-69 yea	HIV/AIDS	Number	2018	4670.03	4831.37	4524.86
Deaths	Tropical La	Both	50-69 yea	HIV/AIDS	Rate	2018	11.5965	11.9971	11.236
Deaths	South Asia	Both	50-69 yea	Sexually tr	Number	2013	1054.59	1276.43	791.996
Deaths	South Asia	Both	50-69 yea	Sexually tr	Rate	2013	0.52069	0.63022	0.39104
Deaths	East Asia	Both	50-69 yea	HIV/AIDS	Number	2018	11068.6	13182.5	9018.88
Deaths	East Asia	Both	50-69 yea	HIV/AIDS	Rate	2018	2.98451	3.5545	2.43184
Deaths	High-incor	Both	50-69 yea	Sexually tr	Number	2018	44.0451	47.0406	41.7353
Deaths	High-incor	Both	50-69 yea	Sexually tr	Rate	2018	0.04862	0.05193	0.04607
Deaths	Western S	Both	50-69 yea	HIV/AIDS	Number	2011	32003.5	35375.9	28759.6
Deaths	Western S	Both	50-69 yea	HIV/AIDS	Rate	2011	121.647	134.466	109.317
Deaths	Central Eu	Both	50-69 yea	HIV/AIDS	Number	2016	144.086	178.002	128.528
Deaths	Central Eu	Both	50-69 yea	HIV/AIDS	Rate	2016	0.48152	0.59487	0.42953
Deaths	Southern L	Both	50-69 yea	HIV/AIDS	Number	2013	629.793	641.971	617.169
Deaths	Southern L	Both	50-69 yea	HIV/AIDS	Rate	2013	5.57289	5.68064	5.46117
Deaths	Eastern Su	Both	50-69 yea	HIV/AIDS	Number	2016	47041.8	56294.3	41106.5
Deaths	Eastern Su	Both	50-69 yea	HIV/AIDS	Rate	2016	178.082	213.108	155.613
Deaths	Western S	Both	50-69 yea	Sexually tr	Number	2013	40.0571	52.4673	29.2385
Deaths	Western S	Both	50-69 yea	Sexually tr	Rate	2013	0.1426	0.18678	0.10409
Deaths	Southeast	Both	50-69 yea	Sexually tr	Number	2011	100.234	135.845	61.3629
Deaths	Southeast	Both	50-69 yea	Sexually tr	Rate	2011	0.11585	0.15701	0.07092
Deaths	High-incor	Both	50-69 yea	Sexually tr	Number	2018	15.4475	16.8575	14.1682
Deaths	High-incor	Both	50-69 yea	Sexually tr	Rate	2018	0.03124	0.03409	0.02865
Deaths	Eastern Eu	Both	50-69 yea	Sexually tr	Number	2013	84.645	97.7693	80.2919
Deaths	Eastern Eu	Both	50-69 yea	Sexually tr	Rate	2013	0.16208	0.18721	0.15374
Deaths	Southern S	Both	50-69 yea	HIV/AIDS	Number	2016	45592.4	53729.6	39274.3
Deaths	Southern S	Both	50-69 yea	HIV/AIDS	Rate	2016	492.617	580.537	424.351
Deaths	Western S	Both	50-69 yea	HIV/AIDS	Number	2016	35014.6	39006.1	31836.4
Deaths	Western S	Both	50-69 yea	HIV/AIDS	Rate	2016	111.853	124.604	101.7
Deaths	Southern S	Both	50-69 yea	HIV/AIDS	Number	2018	44892.1	52799.7	38890.6
Deaths	Southern S	Both	50-69 yea	HIV/AIDS	Rate	2018	460.831	542.005	399.224
Deaths	High-incor	Both	50-69 yea	HIV/AIDS	Number	2011	4040.46	4083.26	4001.17
Deaths	High-incor	Both	50-69 yea	HIV/AIDS	Rate	2011	4.97629	5.029	4.92789
Deaths	Southern S	Both	50-69 yea	HIV/AIDS	Number	2014	41878.5	51186.4	35364.2
Deaths	Southern S	Both	50-69 yea	HIV/AIDS	Rate	2014	477.511	583.642	403.233
Deaths	Eastern Su	Both	50-69 yea	Sexually tr	Number	2014	363.701	574.251	167.757
Deaths	Eastern Su	Both	50-69 yea	Sexually tr	Rate	2014	1.47709	2.33218	0.68131
Deaths	Southern L	Both	50-69 yea	HIV/AIDS	Number	2012	582.942	595.111	571.338
Deaths	Southern L	Both	50-69 yea	HIV/AIDS	Rate	2012	5.27316	5.38324	5.16819
Deaths	Tropical La	Both	50-69 yea	HIV/AIDS	Number	2001	2070.39	2108.83	2034.34
Deaths	Tropical La	Both	50-69 yea	HIV/AIDS	Rate	2001	9.29114	9.46366	9.12935
Deaths	Central La	Both	50-69 yea	HIV/AIDS	Number	2013	2741.43	2900.63	2618.36
Deaths	Central La	Both	50-69 yea	HIV/AIDS	Rate	2013	8.33625	8.82033	7.96201
Deaths	Central Eu	Both	50-69 yea	HIV/AIDS	Number	2017	139.657	170.133	125.885
Deaths	Central Eu	Both	50-69 yea	HIV/AIDS	Rate	2017	0.46725	0.56922	0.42118
Deaths	Eastern Su	Both	50-69 yea	HIV/AIDS	Number	2019	45322.6	54688.9	38709.8
Deaths	Eastern Su	Both	50-69 yea	HIV/AIDS	Rate	2019	153.444	185.155	131.056

Deaths	Central Eu	Both	50-69 yea	Sexually tra	Rate	2018	0.03201	0.03763	0.02723
Deaths	Western E	Both	50-69 yea	HIV/AIDS	Number	2015	1560.94	1587.99	1532.41
Deaths	Western E	Both	50-69 yea	HIV/AIDS	Rate	2015	1.44108	1.46606	1.41475
Deaths	Eastern Eu	Both	50-69 yea	HIV/AIDS	Number	2016	3084.41	3139.04	3033.72
Deaths	Eastern Eu	Both	50-69 yea	HIV/AIDS	Rate	2016	5.61833	5.71785	5.526
Deaths	Southeast	Both	50-69 yea	Sexually tra	Number	2019	105.076	140.834	64.7825
Deaths	Southeast	Both	50-69 yea	Sexually tra	Rate	2019	0.09124	0.12229	0.05625
Deaths	Eastern Eu	Both	50-69 yea	HIV/AIDS	Number	2019	2530.9	2598.85	2463.76
Deaths	Eastern Eu	Both	50-69 yea	HIV/AIDS	Rate	2019	4.65345	4.77838	4.52999
Deaths	Australasia	Both	50-69 yea	Sexually tra	Number	2019	1.39683	1.78416	1.14313
Deaths	Australasia	Both	50-69 yea	Sexually tra	Rate	2019	0.02073	0.02647	0.01696
Deaths	Oceania	Both	50-69 yea	Sexually tra	Number	2017	2.95597	6.12754	1.28412
Deaths	Oceania	Both	50-69 yea	Sexually tra	Rate	2017	0.2297	0.47615	0.09979
Deaths	Andean La	Both	50-69 yea	HIV/AIDS	Number	2019	583.76	1100.68	397.778
Deaths	Andean La	Both	50-69 yea	HIV/AIDS	Rate	2019	6.32092	11.9181	4.30712
Deaths	Australasia	Both	50-69 yea	HIV/AIDS	Number	2018	33.0806	34.5072	31.6615
Deaths	Australasia	Both	50-69 yea	HIV/AIDS	Rate	2018	0.49716	0.5186	0.47583
Deaths	Oceania	Both	50-69 yea	Sexually tra	Number	2019	3.05197	6.5793	1.30297
Deaths	Oceania	Both	50-69 yea	Sexually tra	Rate	2019	0.22036	0.47505	0.09408
Deaths	Eastern Eu	Both	50-69 yea	HIV/AIDS	Number	2014	2489.42	2532.63	2445.38
Deaths	Eastern Eu	Both	50-69 yea	HIV/AIDS	Rate	2014	4.66062	4.74152	4.57817
Deaths	Central As	Both	50-69 yea	HIV/AIDS	Number	2015	158.629	169.881	149.086
Deaths	Central As	Both	50-69 yea	HIV/AIDS	Rate	2015	1.21148	1.29742	1.1386
Deaths	High-incor	Both	50-69 yea	Sexually tra	Number	2013	40.0502	42.3603	38.2601
Deaths	High-incor	Both	50-69 yea	Sexually tra	Rate	2013	0.04707	0.04979	0.04497
Deaths	North Afric	Both	50-69 yea	HIV/AIDS	Number	2016	1519.95	2994.75	907.919
Deaths	North Afric	Both	50-69 yea	HIV/AIDS	Rate	2016	2.14145	4.21928	1.27916
Deaths	Central Lat	Both	50-69 yea	HIV/AIDS	Number	2019	2798.95	2984.61	2639.26
Deaths	Central Lat	Both	50-69 yea	HIV/AIDS	Rate	2019	7.01536	7.48069	6.61509
Deaths	Caribbean	Both	50-69 yea	Sexually tra	Number	2016	27.1584	39.6909	18.7684
Deaths	Caribbean	Both	50-69 yea	Sexually tra	Rate	2016	0.34125	0.49872	0.23583
Deaths	Central Lat	Both	50-69 yea	Sexually tra	Number	2015	54.3755	57.9375	50.8175
Deaths	Central Lat	Both	50-69 yea	Sexually tra	Rate	2015	0.15424	0.16434	0.14415
Deaths	Oceania	Both	50-69 yea	HIV/AIDS	Number	2017	752.641	1973.36	318.08
Deaths	Oceania	Both	50-69 yea	HIV/AIDS	Rate	2017	58.4854	153.343	24.717
Deaths	High-incor	Both	50-69 yea	Sexually tra	Number	2017	16.1966	17.5666	14.8877
Deaths	High-incor	Both	50-69 yea	Sexually tra	Rate	2017	0.0328	0.03557	0.03015
Deaths	Southern L	Both	50-69 yea	Sexually tra	Number	2014	15.5609	17.5785	13.7213
Deaths	Southern L	Both	50-69 yea	Sexually tra	Rate	2014	0.13484	0.15232	0.1189
Deaths	Central Eu	Both	50-69 yea	HIV/AIDS	Number	2015	150.904	186.455	133.328
Deaths	Central Eu	Both	50-69 yea	HIV/AIDS	Rate	2015	0.5049	0.62384	0.44609
Deaths	Tropical La	Both	50-69 yea	HIV/AIDS	Number	2019	4658.69	4829.19	4497.32
Deaths	Tropical La	Both	50-69 yea	HIV/AIDS	Rate	2019	11.2809	11.6937	10.8901
Deaths	Central Su	Both	50-69 yea	HIV/AIDS	Number	2016	8618.05	10592.9	7039.13
Deaths	Central Su	Both	50-69 yea	HIV/AIDS	Rate	2016	93.7695	115.257	76.59
Deaths	High-incor	Both	50-69 yea	Sexually tra	Number	2017	43.656	46.4819	41.4305
Deaths	High-incor	Both	50-69 yea	Sexually tra	Rate	2017	0.04834	0.05147	0.04588
Deaths	Western E	Both	50-69 yea	HIV/AIDS	Number	2007	1481.66	1507.67	1456.91
Deaths	Western E	Both	50-69 yea	HIV/AIDS	Rate	2007	1.54524	1.57237	1.51943
Deaths	Southern S	Both	50-69 yea	HIV/AIDS	Number	2011	48904.5	59675.7	40008.4
Deaths	Southern S	Both	50-69 yea	HIV/AIDS	Rate	2011	607.959	741.862	497.366
Deaths	South Asia	Both	50-69 yea	Sexually tra	Number	2016	1254.17	1565.3	887.445
Deaths	South Asia	Both	50-69 yea	Sexually tra	Rate	2016	0.56162	0.70094	0.3974
Deaths	Tropical La	Both	50-69 yea	Sexually tra	Number	2012	61.2787	64.7836	56.2306
Deaths	Tropical La	Both	50-69 yea	Sexually tra	Rate	2012	0.18419	0.19473	0.16902
Deaths	Central Eu	Both	50-69 yea	Sexually tra	Number	2017	9.90428	11.0565	8.87148
Deaths	Central Eu	Both	50-69 yea	Sexually tra	Rate	2017	0.03314	0.03699	0.02968
Deaths	East Asia	Both	50-69 yea	HIV/AIDS	Number	2017	10609.9	12146.7	9057.58
Deaths	East Asia	Both	50-69 yea	HIV/AIDS	Rate	2017	2.96037	3.38917	2.52724
Deaths	Southern L	Both	50-69 yea	Sexually tra	Number	2015	15.5907	17.7444	13.758
Deaths	Southern L	Both	50-69 yea	Sexually tra	Rate	2015	0.13251	0.15081	0.11693
Deaths	Australasia	Both	50-69 yea	HIV/AIDS	Number	2017	33.3087	34.969	31.8707
Deaths	Australasia	Both	50-69 yea	HIV/AIDS	Rate	2017	0.5061	0.53132	0.48425
Deaths	Southeast	Both	50-69 yea	Sexually tra	Number	2018	104.426	139.909	66.8341
Deaths	Southeast	Both	50-69 yea	Sexually tra	Rate	2018	0.09359	0.12539	0.0599
Deaths	Caribbean	Both	50-69 yea	Sexually tra	Number	2017	27.6697	41.1139	19.0114
Deaths	Caribbean	Both	50-69 yea	Sexually tra	Rate	2017	0.33977	0.50486	0.23345
Deaths	Western S	Both	50-69 yea	Sexually tra	Number	2018	43.0349	56.2524	32.3076
Deaths	Western S	Both	50-69 yea	Sexually tra	Rate	2018	0.1273	0.1664	0.09557
Deaths	Central Su	Both	50-69 yea	HIV/AIDS	Number	2001	10128.6	11963.4	8533.54
Deaths	Central Su	Both	50-69 yea	HIV/AIDS	Rate	2001	191.477	226.163	161.323
Deaths	Caribbean	Both	50-69 yea	HIV/AIDS	Number	2007	2879.48	3447.46	2375.55
Deaths	Caribbean	Both	50-69 yea	HIV/AIDS	Rate	2007	45.7187	54.7367	37.7175
Deaths	Caribbean	Both	50-69 yea	Sexually tra	Number	2006	20.1803	30.5417	13.9387
Deaths	Caribbean	Both	50-69 yea	Sexually tra	Rate	2006	0.32769	0.49594	0.22634
Deaths	Australasia	Both	50-69 yea	HIV/AIDS	Number	2016	33.3981	34.934	32.0253
Deaths	Australasia	Both	50-69 yea	HIV/AIDS	Rate	2016	0.51171	0.53524	0.49067
Deaths	Central Su	Both	50-69 yea	Sexually tra	Number	2008	93.0112	202.765	25.1605
Deaths	Central Su	Both	50-69 yea	Sexually tra	Rate	2008	1.3997	3.05136	0.37863
Deaths	Central Lat	Both	50-69 yea	HIV/AIDS	Number	2017	2807.33	2997.59	2651.13
Deaths	Central Lat	Both	50-69 yea	HIV/AIDS	Rate	2017	7.47351	7.98001	7.05768
Deaths	North Afric	Both	50-69 yea	Sexually tra	Number	2019	36.014	47.4145	20.054
Deaths	North Afric	Both	50-69 yea	Sexually tra	Rate	2019	0.04519	0.0595	0.02516
Deaths	Tropical La	Both	50-69 yea	Sexually tra	Number	2013	63.6212	67.4862	57.9643
Deaths	Tropical La	Both	50-69 yea	Sexually tra	Rate	2013	0.1846	0.19581	0.16818
Deaths	Eastern Eu	Both	50-69 yea	Sexually tra	Number	2016	88.7399	111.138	82.1764
Deaths	Eastern Eu	Both	50-69 yea	Sexually tra	Rate	2016	0.16164	0.20244	0.14969
Deaths	Caribbean	Both	50-69 yea	Sexually tra	Number	2014	25.6749	37.2388	17.7433
Deaths	Caribbean	Both	50-69 yea	Sexually tra	Rate	2014	0.34077	0.49425	0.2355
Deaths	Southern S	Both	50-69 yea	HIV/AIDS	Number	2019	43875.5	52164	37974
Deaths	Southern S	Both	50-69 yea	HIV/AIDS	Rate	2019	438.549	521.396	379.563
Deaths	Central Su	Both	50-69 yea	HIV/AIDS	Number	2008	10002.1	11979.5	8313.23
Deaths	Central Su	Both	50-69 yea	HIV/AIDS	Rate	2008	150.519	180.276	125.104
Deaths	Central Su	Both	50-69 yea	HIV/AIDS	Number	2014	9123.72	11127.6	7404.45
Deaths	Central Su	Both	50-69 yea	HIV/AIDS	Rate	2014	107.89	131.586	87.5592
Deaths	Eastern Su	Both	50-69 yea	Sexually tra	Number	2010	375.047	606.129	165.704
Deaths	Eastern Su	Both	50-69 yea	Sexually tra	Rate	2010	1.74239	2.81595	0.76983
Deaths	High-incor	Both	50-69 yea	HIV/AIDS	Number	2015	3900.75	3950.53	3852.66
Deaths	High-incor	Both	50-69 yea	HIV/AIDS	Rate	2015	4.40698	4.46322	4.35265
Deaths	Eastern Su	Both	50-69 yea	HIV/AIDS	Number	2015	48216.8	57541.3	42234.3
Deaths	Eastern Su	Both	50-69 yea	HIV/AIDS	Rate	2015	189.122	225.696	165.657
Deaths	Southeast	Both	50-69 yea	HIV/AIDS	Number	2016	11070	15183.9	9004.33
Deaths	Southeast	Both	50-69 yea	HIV/AIDS	Rate	2016	10.6082	14.5505	8.62872
Deaths	Western E	Both	50-69 yea	HIV/AIDS	Number	2019	1332.63	1369.45	1299.78
Deaths	Western E	Both	50-69 yea	HIV/AIDS	Rate	2019	1.17766	1.2102	1.14864
Deaths	Southern L	Both	50-69 yea	HIV/AIDS	Number	2016	687.24	705.326	670.662
Deaths	Southern L	Both	50-69 yea	HIV/AIDS	Rate	2016	5.73824	5.88925	5.59982
Deaths	Western E	Both	50-69 yea	HIV/AIDS	Number	2017	1410.35	1444.41	1377.09
Deaths	Western E	Both	50-69 yea	HIV/AIDS	Rate	2017	1.26759	1.29819	1.23768
Deaths	East Asia	Both	50-69 yea	HIV/AIDS	Number	2014	6199.87	7025.07	5494.12
Deaths	East Asia	Both	50-69 yea	HIV/AIDS	Rate	2014	1.95031	2.20989	1.7283
Deaths	Tropical La	Both	50-69 yea	HIV/AIDS	Number	2017	4671.94	4822.92	4534.07
Deaths	Tropical La	Both	50-69 yea	HIV/AIDS	Rate	2017	11.9181	12.3032	11.5663
Deaths	Eastern Eu	Both	50-69 yea	HIV/AIDS	Number	2018	2656.82	2727.24	2588.17
Deaths	Eastern Eu	Both	50-69 yea	HIV/AIDS	Rate	2018	4.85645	4.98517	4.73096
Deaths	Australasia	Both	50-69 yea	Sexually tra	Number	2018	1.38297	1.72995	1.14126
Deaths	Australasia	Both	50-69 yea	Sexually tra	Rate	2018	0.02078	0.026	0.01715
Deaths	Tropical La	Both	50-69 yea	HIV/AIDS	Number	2015	4459.19	4583.42	4340.71
Deaths	Tropical La	Both	50-69 yea	HIV/AIDS	Rate	2015	12.0895	12.4264	11.7683
Deaths	Eastern Su	Both	50-69 yea	Sexually tra	Number	2016	359.061	560.941	169.31
Deaths	Eastern Su	Both	50-69 yea	Sexually tra	Rate	2016	1.35926	2.1235	0.64094
Deaths	Southern L	Both	50-69 yea	Sexually tra	Number	2018	14.2688	16.7332	12.2289
Deaths	Southern L	Both	50-69 yea	Sexually tra	Rate	2018	0.11549	0.13544	0.09898
Deaths	East Asia	Both	50-69 yea	Sexually tra	Number	2016	220.379	261.917	163.69
Deaths	East Asia	Both	50-69 yea	Sexually tra	Rate	2016	0.06378	0.0758	0.04737
Deaths	North Afric	Both	50-69 yea	Sexually tra	Number	2018	35.4256	46.9176	19.4105
Deaths	North Afric	Both	50-69 yea	Sexually tra	Rate	2018	0.04619	0.06117	0.02531
Deaths	Western S	Both	50-69 yea	HIV/AIDS	Number	2018	34774.5	39332.8	30871.4
Deaths	Western S	Both	50-69 yea	HIV/AIDS	Rate	2018	102.869	116.353	91.3231
Deaths	Western S	Both	50-69 yea	HIV/AIDS	Number	2007	35459.3	39329.2	31728.5
Deaths	Western S	Both	50-69 yea	HIV/AIDS	Rate	2007	152.949	169.641	136.857
Deaths	Central As	Both	50-69 yea	HIV/AIDS	Number	2017	145.407	158.525	135.243
Deaths	Central As	Both	50-69 yea	HIV/AIDS	Rate	2017	1.03032	1.12327	0.9583
Deaths	High-incor	Both	50-69 yea	HIV/AIDS	Number	2019	202.118	211.955	194.233
Deaths	High-incor	Both	50-69 yea	HIV/AIDS	Rate	2019	0.40785	0.42771	0.39194
Deaths	Australasia	Both	50-69 yea	Sexually tra	Number	2015	1.37377	1.69785	1.15386
Deaths	Australasia	Both	50-69 yea	Sexually tra	Rate	2015	0.02137	0.02641	0.01795
Deaths	Central Lat	Both	50-69 yea	Sexually tra	Number	2019	57.3286	67.6501	48.2997
Deaths	Central Lat	Both	50-69 yea	Sexually tra	Rate	2019	0.14369	0.16956	0.12106
Deaths	Tropical La	Both	50-69 yea	Sexually tra	Number	2019	68.9909	74.8717	61.4396
Deaths	Tropical La	Both	50-69 yea	Sexually tra	Rate	2019	0.16706	0.1813	0.14877
Deaths	Central Eu	Both	50-69 yea	Sexually tra	Number	2006	10.7955	11.5925	9.98021
Deaths	Central Eu	Both	50-69 yea	Sexually tra	Rate	2006	0.03908	0.04197	0.03613
Deaths	Eastern Su	Both	50-69 yea	Sexually tra	Number	1998	464.501	909.69	178.057
Deaths	Eastern Su	Both	50-69 yea	Sexually tra	Rate	1998	2.93363	5.7453	1.12455
Deaths	Western E	Both	50-69 yea	HIV/AIDS	Number	2018	1370.76	1405.69	1339.02
Deaths	Western E	Both	50-69 yea	HIV/AIDS	Rate	2018	1.22058	1.25169	1.19232
Deaths	Caribbean	Both	50-69 yea	HIV/AIDS	Number	2006	3133.88	3694.47	2631.06
Deaths	Caribbean	Both	50-69 yea	HIV/AIDS	Rate	2006	50.8885	59.9914	42.7235
Deaths	Central Eu	Both	50-69 yea	HIV/AIDS	Number	2012	164.645	207.974	144.056
Deaths	Central Eu	Both	50-69 yea	HIV/AIDS	Rate	2012	0.55992	0.70728	0.4899
Deaths	Central Su	Both	50-69 yea	Sexually tra	Number	2006	92.9944	204.975	24.3101
Deaths	Central Su	Both	50-69 yea	Sexually tra	Rate	2006	1.50269	3.31219	0.39283
Deaths	Central Eu	Both	50-69 yea	HIV/AIDS	Number	2007	152.409	172.356	143.318
Deaths	Central Eu	Both	50-69 yea	HIV/AIDS	Rate	2007	0.54373	0.61489	0.5113
Deaths	Western E	Both	50-69 yea	Sexually tra	Number	2007	39.5302	47.8801	37.2057
Deaths	Western E	Both	50-69 yea	Sexually tra	Rate	2007	0.04123	0.04993	0.0388
Deaths	Tropical La	Both	50-69 yea	HIV/AIDS	Number	2014	4276.46	4383.36	4165.84
Deaths	Tropical La	Both	50-69 yea	HIV/AIDS	Rate	2014	11.9875	12.2872	11.6775
Deaths	Central Su	Both	50-69 yea	Sexually tra	Number	2007	92.7925	201.339	24.6087
Deaths	Central Su	Both	50-69 yea	Sexually tra	Rate	2007	1.44802	3.14189	0.38402
Deaths	Western E	Both	50-69 yea	Sexually tra	Number	2012	37.3057	42.2459	34.9447
Deaths	Western E	Both	50-69 yea	Sexually tra	Rate	2012	0.0363	0.04111	0.034
Deaths	Oceania	Both	50-69 yea	Sexually tra	Number	2015	2.86244	5.98731	1.21743
Deaths	Oceania	Both	50-69 yea	Sexually tra	Rate	2015	0.2399	0.50179	0.10203
Deaths	Caribbean	Both	50-69 yea	Sexually tra	Number	2013	24.556	36.3197	17.1137
Deaths	Caribbean	Both	50-69 yea	Sexually tra	Rate	2013	0.33557	0.49633	0.23387
Deaths	Central Su	Both	50-69 yea	HIV/AIDS	Number	2013	9465.11	11411.1	7780.55
Deaths	Central Su	Both	50-69 yea	HIV/AIDS	Rate	2013	116.699	140.692	95.9295
Deaths	Western S	Both	50-69 yea	HIV/AIDS	Number	2019	34831.3	39850.8	31097.5
Deaths	Western S	Both	50-69 yea	HIV/AIDS	Rate	2019	99.0879	113.367	88.4658
Deaths	Western S	Both	50-69 yea	Sexually tra	Number	2016	42.0706	55.5587	31.1834
Deaths	Western S	Both	50-69 yea	Sexually tra	Rate	2016	0.13439	0.17748	0.09961
Deaths	Eastern Su	Both	50-69 yea	Sexually tra	Number	2018	355.05	551.97	172.495
Deaths	Eastern Su	Both	50-69 yea	Sexually tra	Rate	2018	1.24933	1.94225	0.60697
Deaths	Caribbean	Both	50-69 yea	Sexually tra	Number	2012	23.1691	34.646	15.8018
Deaths	Caribbean	Both	50-69 yea	Sexually tra	Rate	2012	0.32598	0.48745	0.22232
Deaths	Central Su	Both	50-69 yea	HIV/AIDS	Number	2012	9695.42	11546.5	8025.33
Deaths	Central Su	Both	50-69 yea	HIV/AIDS	Rate	2012	124.597	148.386	103.135
Deaths	Western E	Both	50-69 yea	HIV/AIDS	Number	2013	1574.57	1600.38	1548.74
Deaths	Western E	Both	50-69 yea	HIV/AIDS	Rate	2013	1.50571	1.5304	1.48101
Deaths	Tropical La	Both	50-69 yea	HIV/AIDS	Number	2013	3970.98	4072.09	3872.36
Deaths	Tropical La	Both	50-69 yea	HIV/AIDS	Rate	2013	11.5219	11.8153	11.2358
Deaths	Caribbean	Both	50-69 yea	HIV/AIDS	Number	2014	2715.01	3142.57	2343.9
Deaths	Caribbean	Both	50-69 yea	HIV/AIDS	Rate	2014	36.0346	41.7094	31.1091
Deaths	Western S	Both	50-69 yea	HIV/AIDS	Number	2014	34091.4	37543.5	31216
Deaths	Western S	Both	50-69 yea	HIV/AIDS	Rate	2014	117.35	129.232	107.452
Deaths	Tropical La	Both	50-69 yea	Sexually tra	Number	2015	66.3416	70.5087	59.602
Deaths	Tropical La	Both	50-69 yea	Sexually tra	Rate	2015	0.17986	0.19116	0.16159
Deaths	Central As	Both	50-69 yea	Sexually tra	Number	2017	22.0439	28.3321	18.1185
Deaths	Central As	Both	50-69 yea	Sexually tra	Rate	2017	0.1562	0.20075	0.12838
Deaths	Western E	Both	50-69 yea	Sexually tra	Number	2019	36.8658	41.4053	34.0928
Deaths	Western E	Both	50-69 yea	Sexually tra	Rate	2019	0.03258	0.03659	0.03013
DALYs (Dis	East Asia	Both	50-69 yea	HIV/AIDS	Number	1990	18542.2	27382.7	3926.79
DALYs (Dis	East Asia	Both	50-69 yea	HIV/AIDS	Rate	1990	11.5754	17.0943	2.4514
DALYs (Dis	High-incor	Both	50-69 yea	Sexually tra	Number	1992	3971.05	7360.8	2188.15
DALYs (Dis	High-incor	Both	50-69 yea	Sexually tra	Rate	1992	10.9914	20.3738	6.05654
DALYs (Dis	Central Eu	Both	50-69 yea	Sexually tra	Number	1991	2714.64	4805.94	1592.26
DALYs (Dis	Central Eu	Both	50-69 yea	Sexually tra	Rate	1991	10.7878	19.0985	6.32756
DALYs (Dis	Eastern Su	Both	50-69 yea	HIV/AIDS	Number	1990	709622	989177	468552
DALYs (Dis	Eastern Su	Both	50-69 yea	HIV/AIDS	Rate	1990	5192.76	7238.45	3428.69
DALYs (Dis	Eastern Su	Both	50-69 yea	Sexually tra	Number	1990	18096.4	32268.1	8257.76
DALYs (Dis	Eastern Su	Both	50-69 yea	Sexually tra	Rate	1990	132.423	236.127	60.4274
DALYs (Dis	High-incor	Both	50-69 yea	HIV/AIDS	Number	1990	126264	131971	121985
DALYs (Dis	High-incor	Both	50-69 yea	HIV/AIDS	Rate	1990	265.71	277.719	256.706
DALYs (Dis	High-incor	Both	50-69 yea	Sexually tra	Number	1990	8224.21	15150	4692.4
DALYs (Dis	High-incor	Both	50-69 yea	Sexually tra	Rate	1990	17.307	31.8817	9.87471
DALYs (Dis	Eastern Eu	Both	50-69 yea	Sexually tra	Number	1992	13868	19539.2	9701.69
DALYs (Dis	Eastern Eu	Both	50-69 yea	Sexually tra	Rate	1992	27.243	38.3838	19.0585
DALYs (Dis	Tropical La	Both	50-69 yea	Sexually tra	Number	1990	4712.05	8597.01	2732.68
DALYs (Dis	Tropical La	Both	50-69 yea	Sexually tra	Rate	1990	29.3511	53.5504	17.0217
DALYs (Dis	South Asia	Both	50-69 yea	HIV/AIDS	Number	1991	7993.12	18018.7	4265.94
DALYs (Dis	South Asia	Both	50-69 yea	HIV/AIDS	Rate	1991	7.25586	16.3567	3.87247
DALYs (Dis	South Asia	Both	50-69 yea	Sexually tra	Number	1991	39545	50909	28631.6
DALYs (Dis	South Asia	Both	50-69 yea	Sexually tra	Rate	1991	35.8975	46.2133	25.9907
DALYs (Dis	Western E	Both	50-69 yea	Sexually tra	Number	1991	7816.38	14220.8	4651.93
DALYs (Dis	Western E	Both	50-69 yea	Sexually tra	Rate	1991	9.39986	17.1017	5.59434
DALYs (Dis	Caribbean	Both	50-69 yea	Sexually tra	Number	1991	1318.87	2243.38	800.946
DALYs (Dis	Caribbean	Both	50-69 yea	Sexually tra	Rate	1991	31.2274	53.1172	18.9643
DALYs (Dis	East Asia	Both	50-69 yea	Sexually tra	Number	1990	30197	47802	20443.5
DALYs (Dis	East Asia	Both	50-69 yea	Sexually tra	Rate	1990	18.8512	29.8416	12.7623
DALYs (Dis	Southeast	Both	50-69 yea	Sexually tra	Number	1991	9145.19	16231.1	5431.48
DALYs (Dis	Southeast	Both	50-69 yea	Sexually tra	Rate	1991	18.8964	33.5378	11.2229
DALYs (Dis	Southern L	Both	50-69 yea	HIV/AIDS	Number	1991	6154.46	6420.02	5917.63
DALYs (Dis	Southern L	Both	50-69 yea	HIV/AIDS	Rate	1991	81.1739	84.6764	78.0502
DALYs (Dis	Australasia	Both	50-69 yea	Sexually tra	Number	1990	374.094	698.623	204.118
DALYs (Dis	Australasia	Both	50-69 yea	Sexually tra	Rate	1990	10.8695	20.2989	5.93077
DALYs (Dis	Oceania	Both	50-69 yea	HIV/AIDS	Number	1991	109.17	471.957	22.6598
DALYs (Dis	Oceania	Both	50-69 yea	HIV/AIDS	Rate	1991	19.1427	82.7563	3.97332
DALYs (Dis	Southern S	Both	50-69 yea	Sexually tra	Number	1991	2946.11	4614.73	1921.08
DALYs (Dis	Southern S	Both	50-69 yea	Sexually tra	Rate	1991	61.0657	95.6524	39.8195
DALYs (Dis	Southern L	Both	50-69 yea	Sexually tra	Number	1990	1414.22	2433.3	884.552
DALYs (Dis	Southern L	Both	50-69 yea	Sexually tra	Rate	1990	18.8873	32.4975	11.8135
DALYs (Dis	Southern S	Both	50-69 yea	Sexually tra	Number	1990	2911.38	4621.96	1893.81
DALYs (Dis	Southern S	Both	50-69 yea	Sexually tra	Rate	1990	61.9592	98.3634	40.3035
DALYs (Dis	High-incor	Both	50-69 yea	HIV/AIDS	Number	1992	168730	178453	162604
DALYs (Dis	High-incor	Both	50-69 yea	HIV/AIDS	Rate	1992	351.581	371.842	338.817
DALYs (Dis	Central As	Both	50-69 yea	HIV/AIDS	Number	1991	1137.36	1203.5	1061.05
DALYs (Dis	Central As	Both	50-69 yea	HIV/AIDS	Rate	1991	12.8291	13.5751	11.9682
DALYs (Dis	Central Su	Both	50-69 yea	HIV/AIDS	Number	1990	103539	154759	68606.2
DALYs (Dis	Central Su	Both	50-69 yea	HIV/AIDS	Rate	1990	2332.62	3486.56	1545.63
DALYs (Dis	Central Su	Both	50-69 yea	Sexually tra	Number	1990	4064.49	7492.56	1474.6
DALYs (Dis	Central Su	Both	50-69 yea	Sexually tra	Rate	1990	91.5688	168.8	33.2212
DALYs (Dis	Central As	Both	50-69 yea	Sexually tra	Number	1993	2270.43	3320.72	1673.96
DALYs (Dis	Central As	Both	50-69 yea	Sexually tra	Rate	1993	25.638	37.4981	18.9026
DALYs (Dis	High-incor	Both	50-69 yea	Sexually tra	Number	1992	8166.83	15226.1	4623.86
DALYs (Dis	High-incor	Both	50-69 yea	Sexually tra	Rate	1992	17.0171	31.7266	9.6347
DALYs (Dis	Southeast	Both	50-69 yea	HIV/AIDS	Number	1993	61699.8	83721.5	39579.4
DALYs (Dis	Southeast	Both	50-69 yea	HIV/AIDS	Rate	1993	122.3	165.95	78.4531
DALYs (Dis	North Afric	Both	50-69 yea	HIV/AIDS	Number	1991	4426.63	9192.15	2082.98
DALYs (Dis	North Afric	Both	50-69 yea	HIV/AIDS	Rate	1991	13.7609	28.5752	6.47525
DALYs (Dis	North Afric	Both	50-69 yea	Sexually tra	Number	1991	3761.24	7073.47	2040.97
DALYs (Dis	North Afric	Both	50-69 yea	Sexually tra	Rate	1991	11.6924	21.989	6.34468
DALYs (Dis	Eastern Eu	Both	50-69 yea	HIV/AIDS	Number	1992	26622.6	28331.2	25413.9
DALYs (Dis	Eastern Eu	Both	50-69 yea	HIV/AIDS	Rate	1992	52.2989	55.6554	49.9245
DALYs (Dis	Australasia	Both	50-69 yea	Sexually tra	Number	1992	353.716	679.226	190.486
DALYs (Dis	Australasia	Both	50-69 yea	Sexually tra	Rate	1992	10.0407	19.2807	5.40719
DALYs (Dis	Oceania	Both	50-69 yea	Sexually tra	Number	1993	151.867	276.19	82.5471
DALYs (Dis	Oceania	Both	50-69 yea	Sexually tra	Rate	1993	25.8541	47.019	14.0529
DALYs (Dis	Australasia	Both	50-69 yea	HIV/AIDS	Number	1990	2671.5	2831.19	2520.39
DALYs (Dis	Australasia	Both	50-69 yea	HIV/AIDS	Rate	1990	77.6218	82.2618	73.2312
DALYs (Dis	Oceania	Both	50-69 yea	Sexually tra	Number	1991	146.869	261.071	79.8615
DALYs (Dis	Oceania	Both	50-69 yea	Sexually tra	Rate	1991	25.753	45.7781	14.0035
DALYs (Dis	Tropical La	Both	50-69 yea	HIV/AIDS	Number	1990	25680	27063	24601.3
DALYs (Dis	Tropical La	Both	50-69 yea	HIV/AIDS	Rate	1990	159.959	168.574	153.24
DALYs (Dis	Central Eu	Both	50-69 yea	HIV/AIDS	Number	1991	4875.23	5180.65	4550.31
DALYs (Dis	Central Eu	Both	50-69 yea	HIV/AIDS	Rate	1991	19.3739	20.5876	18.0827
DALYs (Dis	Western S	Both	50-69 yea	Sexually tra	Number	1990	4735.07	7819.99	2923.81
DALYs (Dis	Western S	Both	50-69 yea	Sexually tra	Rate	1990	30.3231	50.0786	18.7239
DALYs (Dis	Caribbean	Both	50-69 yea	HIV/AIDS	Number	1991	28351.5	44603.3	18472.6
DALYs (Dis	Caribbean	Both	50-69 yea	HIV/AIDS	Rate	1991	671.287	1056.09	437.383
DALYs (Dis	Eastern Su	Both	50-69 yea	HIV/AIDS	Number	1992	1128599	1506768	780702
DALYs (Dis	Eastern Su	Both	50-69 yea	HIV/AIDS	Rate	1992	7984.8	10660.3	5523.44
DALYs (Dis	Eastern Su	Both	50-69 yea	Sexually tra	Number	1992	18306.3	33166	8499.12
DALYs (Dis	Eastern Su	Both	50-69 yea	Sexually tra	Rate	1992	129.517	234.649	60.131
DALYs (Dis	Western E	Both	50-69 yea	HIV/AIDS	Number	1991	63741	66473.9	61496.3
DALYs (Dis	Western E	Both	50-69 yea	HIV/AIDS	Rate	1991	76.654	79.9405	73.9546
DALYs (Dis	Southern L	Both	50-69 yea	Sexually tra	Number	1991	1446.38	2467.97	910.421
DALYs (Dis	Southern L	Both	50-69 yea	Sexually tra	Rate	1991	19.0769	32.5511	12.0079
DALYs (Dis	Andean La	Both	50-69 yea	Sexually tra	Number	1992	842.476	1548.36	467.108
DALYs (Dis	Andean La	Both	50-69 yea	Sexually tra	Rate	1992	22.6459	41.6202	12.5559
DALYs (Dis	Australasia	Both	50-69 yea	HIV/AIDS	Number	1993	3643.12	3872.84	3447.63
DALYs (Dis	Australasia	Both	50-69 yea	HIV/AIDS	Rate	1993	102.15	108.591	96.6687
DALYs (Dis	High-incor	Both	50-69 yea	HIV/AIDS	Number	1990	1716.42	1904.44	1585.3
DALYs (Dis	High-incor	Both	50-69 yea	HIV/AIDS	Rate	1990	5.03146	5.5826	4.64708
DALYs (Dis	Southeast	Both	50-69 yea	Sexually tra	Number	1993	9484.37	16934.5	5715.02
DALYs (Dis	Southeast	Both	50-69 yea	Sexually tra	Rate	1993	18.7996	33.5671	11.3281
DALYs (Dis	Western S	Both	50-69 yea	HIV/AIDS	Number	1992	282960	419728	186904
DALYs (Dis	Western S	Both	50-69 yea	HIV/AIDS	Rate	1992	1738.24	2578.42	1148.16
DALYs (Dis	Central Su	Both	50-69 yea	HIV/AIDS	Number	1992	163737	227844	113492
DALYs (Dis	Central Su	Both	50-69 yea	HIV/AIDS	Rate	1992	3570.22	4968.05	2474.65
DALYs (Dis	Western S	Both	50-69 yea	HIV/AIDS	Number	1990	153238	241752	96267.8
DALYs (Dis	Western S	Both	50-69 yea	HIV/AIDS	Rate	1990	981.322	1548.16	616.492
DALYs (Dis	Andean La	Both	50-69 yea	HIV/AIDS	Number	1991	4416.09	11331.2	2818.77
DALYs (Dis	Andean La	Both	50-69 yea	HIV/AIDS	Rate	1991	122.278	313.753	78.0498
DALYs (Dis	Southern L	Both	50-69 yea	HIV/AIDS	Number	1992	7471.24	7779.72	7213.62
DALYs (Dis	Southern L	Both	50-69 yea	HIV/AIDS	Rate	1992	97.3726	101.393	94.0151
DALYs (Dis	Southeast	Both	50-69 yea	HIV/AIDS	Number	1991	19539.1	30718	11833.5
DALYs (Dis	Southeast	Both	50-69 yea	HIV/AIDS	Rate	1991	40.373	63.4717	24.4511
DALYs (Dis	High-incor	Both	50-69 yea	HIV/AIDS	Number	1991	1943.04	2166.32	1806.3
DALYs (Dis	High-incor	Both	50-69 yea	HIV/AIDS	Rate	1991	5.53197	6.16768	5.14268
DALYs (Dis	Southern S	Both	50-69 yea	HIV/AIDS	Number	1991	91212.6	182611	40800.5
DALYs (Dis	Southern S	Both	50-69 yea	HIV/AIDS	Rate	1991	1890.62	3785.09	845.697
DALYs (Dis	Central As	Both	50-69 yea	Sexually tra	Number	1991	2197.99	3273.83	1594.46
DALYs (Dis	Central As	Both	50-69 yea	Sexually tra	Rate	1991	24.7926	36.9276	17.9849
DALYs (Dis	Western E	Both	50-69 yea	HIV/AIDS	Number	1992	74647.4	77851.7	72028.6
DALYs (Dis	Western E	Both	50-69 yea	HIV/AIDS	Rate	1992	89.7733	93.627	86.6239
DALYs (Dis	Western E	Both	50-69 yea	HIV/AIDS	Number	1990	52965.6	55235	51105.9
DALYs (Dis	Western E	Both	50-69 yea	HIV/AIDS	Rate	1990	64.0009	66.7431	61.7537
DALYs (Dis	Central As	Both	50-69 yea	HIV/AIDS	Number	1993	1291.42	1348.74	1233.14
DALYs (Dis	Central As	Both	50-69 yea	HIV/AIDS	Rate	1993	14.5829	15.2301	13.9249
DALYs (Dis	Caribbean	Both	50-69 yea	Sexually tra	Number	1990	1329.22	2293.31	809.571
DALYs (Dis	Caribbean	Both	50-69 yea	Sexually tra	Rate	1990	32.1821	55.5241	19.6008
DALYs (Dis	Eastern Eu	Both	50-69 yea	Sexually tra	Number	1994	16352.7	21906.7	10444.9
DALYs (Dis	Eastern Eu	Both	50-69 yea	Sexually tra	Rate	1994	33.5759	44.9795	21.4458
DALYs (Dis	Caribbean	Both	50-69 yea	Sexually tra	Number	1993	1325.62	2246.98	793.647
DALYs (Dis	Caribbean	Both	50-69 yea	Sexually tra	Rate	1993	29.9702	50.8007	17.9431
DALYs (Dis	Southern S	Both	50-69 yea	HIV/AIDS	Number	1992	143286	272588	64600.5
DALYs (Dis	Southern S	Both	50-69 yea	HIV/AIDS	Rate	1992	2891.11	5500.06	1303.46
DALYs (Dis	High-incor	Both	50-69 yea	Sexually tra	Number	1995	8304.83	15818.9	4646.49
DALYs (Dis	High-incor	Both	50-69 yea	Sexually tra	Rate	1995	16.6589	31.7318	9.32056
DALYs (Dis	East Asia	Both	50-69 yea	HIV/AIDS	Number	1992	28503.4	34556.9	12066.6
DALYs (Dis	East Asia	Both	50-69 yea	HIV/AIDS	Rate	1992	17.2206	20.8778	7.29012
DALYs (Dis	East Asia	Both	50-69 yea	Sexually tra	Number	1992	30228.8	48433.3	20109.2
DALYs (Dis	East Asia	Both	50-69 yea	Sexually tra	Rate	1992	18.263	29.2614	12.1491
DALYs (Dis	Oceania	Both	50-69 yea	Sexually tra	Number	1990	145.719	257.705	78.4432
DALYs (Dis	Oceania	Both	50-69 yea	Sexually tra	Rate	1990	25.6892	45.4316	13.829
DALYs (Dis	Central Lat	Both	50-69 yea	HIV/AIDS	Number	1995	37226.7	39350	35800.8
DALYs (Dis	Central Lat	Both	50-69 yea	HIV/AIDS	Rate	1995	220.196	232.755	211.762
DALYs (Dis	Central Lat	Both	50-69 yea	Sexually tra	Number	1995	4107.37	7709.12	2291.14
DALYs (Dis	Central Lat	Both	50-69 yea	Sexually tra	Rate	1995	24.2951	45.5994	13.5521
DALYs (Dis	Tropical La	Both	50-69 yea	Sexually tra	Number	1995	5322.55	9964.89	2980.19
DALYs (Dis	Tropical La	Both	50-69 yea	Sexually tra	Rate	1995	28.9283	54.1597	16.1975
DALYs (Dis	Central Lat	Both	50-69 yea	HIV/AIDS	Number	1990	20302.5	20783	19865.5
DALYs (Dis	Central Lat	Both	50-69 yea	HIV/AIDS	Rate	1990	140.867	144.201	137.836
DALYs (Dis	Central Lat	Both	50-69 yea	Sexually tra	Number	1990	3824.69	7025.37	2237.46
DALYs (Dis	Central Lat	Both	50-69 yea	Sexually tra	Rate	1990	26.5374	48.745	15.5245
DALYs (Dis	Southeast	Both	50-69 yea	Sexually tra	Number	1990	8946.14	15729.8	5366.86
DALYs (Dis	Southeast	Both	50-69 yea	Sexually tra	Rate	1990	18.9383	33.2988	11.3612
DALYs (Dis	North Afric	Both	50-69 yea	HIV/AIDS	Number	1990	3502.19	7622.34	1555.73
DALYs (Dis	North Afric	Both	50-69 yea	HIV/AIDS	Rate	1990	11.1489	24.2651	4.95252
DALYs (Dis	High-incor	Both	50-69 yea	Sexually tra	Number	1991	3946.46	7285.45	2193.59
DALYs (Dis	High-incor	Both	50-69 yea	Sexually tra	Rate	1991	11.2359	20.7422	6.24531
DALYs (Dis	Tropical La	Both	50-69 yea	HIV/AIDS	Number	1992	36457	38706.4	34936.9
DALYs (Dis	Tropical La	Both	50-69 yea	HIV/AIDS	Rate	1992	214.718	227.966	205.765
DALYs (Dis	Central Eu	Both	50-69 yea	Sexually tra	Number	1993	2615.48	4735.69	1494.74
DALYs (Dis	Central Eu	Both	50-69 yea	Sexually tra	Rate	1993	10.5018	19.0149	6.00175
DALYs (Dis	Southern S	Both	50-69 yea	HIV/AIDS	Number	1990	55981.5	117519	24898.9
DALYs (Dis	Southern S	Both	50-69 yea	HIV/AIDS	Rate	1990	1191.39	2501.01	529.892
DALYs (Dis	Eastern Eu	Both	50-69 yea	HIV/AIDS	Number	1991	23521	25188.4	22407.2
DALYs (Dis	Eastern Eu	Both	50-69 yea	HIV/AIDS	Rate	1991	46.6253	49.9306	44.4175
DALYs (Dis	Central As	Both	50-69 yea	HIV/AIDS	Number	1995	1629.02	1720.69	1551.83
DALYs (Dis	Central As	Both	50-69 yea	HIV/AIDS	Rate	1995	19.2844	20.3696	18.3706
DALYs (Dis	Central Eu	Both	50-69 yea	HIV/AIDS	Number	1995	5408.59	5786.65	5024.02
DALYs (Dis	Central Eu	Both	50-69 yea	HIV/AIDS	Rate	1995	22.0026	23.5406	20.4382
DALYs (Dis	Caribbean	Both	50-69 yea	HIV/AIDS	Number	1992	39286.2	59850	25658.7
DALYs (Dis	Caribbean	Both	50-69 yea	HIV/AIDS	Rate	1992	908.762	1384.44	593.533
DALYs (Dis	North Afric	Both	50-69 yea	Sexually tra	Number	1993	3919.57	7392.85	2096.84
DALYs (Dis	North Afric	Both	50-69 yea	Sexually tra	Rate	1993	11.6453	21.9646	6.22984
DALYs (Dis	Eastern Su	Both	50-69 yea	HIV/AIDS	Number	1994	1632083	2083358	1187942
DALYs (Dis	Eastern Su	Both	50-69 yea	HIV/AIDS	Rate	1994	11207.4	14306.3	8157.52
DALYs (Dis	Eastern Su	Both	50-69 yea	Sexually tra	Number	1994	18435.9	33160.4	8524.64
DALYs (Dis	Eastern Su	Both	50-69 yea	Sexually tra	Rate	1994	126.598	227.71	58.5382
DALYs (Dis	Western E	Both	50-69 yea	Sexually tra	Number	1990	8031.88	14429.5	4871.98
DALYs (Dis	Western E	Both	50-69 yea	Sexually tra	Rate	1990	9.7053	17.4358	5.88705
DALYs (Dis	Southeast	Both	50-69 yea	HIV/AIDS	Number	1996	159025	176700	129758
DALYs (Dis	Southeast	Both	50-69 yea	HIV/AIDS	Rate	1996	295.848	328.73	241.399
DALYs (Dis	Caribbean	Both	50-69 yea	HIV/AIDS	Number	1995	75972.9	104539	53918
DALYs (Dis	Caribbean	Both	50-69 yea	HIV/AIDS	Rate	1995	1636.3	2251.56	1161.28
DALYs (Dis	Australasia	Both	50-69 yea	HIV/AIDS	Number	1994	3658.53	3887.09	3450.83
DALYs (Dis	Australasia	Both	50-69 yea	HIV/AIDS	Rate	1994	100.782	107.078	95.0601
DALYs (Dis	Oceania	Both	50-69 yea	HIV/AIDS	Number	1996	905.985	2191.63	379.577
DALYs (Dis	Oceania	Both	50-69 yea	HIV/AIDS	Rate	1996	145.692	352.437	61.04
DALYs (Dis	Eastern Eu	Both	50-69 yea	HIV/AIDS	Number	1995	30874.5	32937.7	29549.8
DALYs (Dis	Eastern Eu	Both	50-69 yea	HIV/AIDS	Rate	1995	65.5197	69.8982	62.7085
DALYs (Dis	Oceania	Both	50-69 yea	HIV/AIDS	Number	1990	69.0979	321.855	12.2817
DALYs (Dis	Oceania	Both	50-69 yea	HIV/AIDS	Rate	1990	12.1815	56.7409	2.16518
DALYs (Dis	East Asia	Both	50-69 yea	HIV/AIDS	Number	1991	23510.4	30738.6	7190.06
DALYs (Dis	East Asia	Both	50-69 yea	HIV/AIDS	Rate	1991	14.434	18.8717	4.41428
DALYs (Dis	Central Eu	Both	50-69 yea	HIV/AIDS	Number	1996	5110.86	5501.59	4710.37
DALYs (Dis	Central Eu	Both	50-69 yea	HIV/AIDS	Rate	1996	20.8703	22.4659	19.2349
DALYs (Dis	Caribbean	Both	50-69 yea	HIV/AIDS	Number	1990	20490.2	32235.6	13831.4
DALYs (Dis	Caribbean	Both	50-69 yea	HIV/AIDS	Rate	1990	496.096	780.467	334.876
DALYs (Dis	Eastern Eu	Both	50-69 yea	Sexually tra	Number	1991	12484.4	18001.3	9047.07
DALYs (Dis	Eastern Eu	Both	50-69 yea	Sexually tra	Rate	1991	24.7476	35.6837	17.9339
DALYs (Dis	Central Eu	Both	50-69 yea	Sexually tra	Number	1990	2769.18	4855.77	1645.51
DALYs (Dis	Central Eu	Both	50-69 yea	Sexually tra	Rate	1990	11.0028	19.2935	6.53814
DALYs (Dis	Central Lat	Both	50-69 yea	HIV/AIDS	Number	1992	27750.3	28819	27031.2
DALYs (Dis	Central Lat	Both	50-69 yea	HIV/AIDS	Rate	1992	180.876	187.841	176.188
DALYs (Dis	Central Lat	Both	50-69 yea	Sexually tra	Number	1992	3922.77	7226.85	2252
DALYs (Dis	Central Lat	Both	50-69 yea	Sexually tra	Rate	1992	25.5685	47.1043	14.6784
DALYs (Dis	Central As	Both	50-69 yea	HIV/AIDS	Number	1996	1799.82	1902.02	1711.72
DALYs (Dis	Central As	Both	50-69 yea	HIV/AIDS	Rate	1996	21.8239	23.0632	20.7557
DALYs (Dis	Australasia	Both	50-69 yea	HIV/AIDS	Number	1992	3269.19	3474.54	3083.45
DALYs (Dis	Australasia	Both	50-69 yea	HIV/AIDS	Rate	1992	92.8001	98.6293	87.5277
DALYs (Dis	Andean La	Both	50-69 yea	Sexually tra	Number	1991	817.371	1512.03	457.238
DALYs (Dis	Andean La	Both	50-69 yea	Sexually tra	Rate	1991	22.6324	41.867	12.6606
DALYs (Dis	Andean La	Both	50-69 yea	HIV/AIDS	Number	1992	5089.28	13137.9	3172.91
DALYs (Dis	Andean La	Both	50-69 yea	HIV/AIDS	Rate	1992	136.801	353.149	85.2883
DALYs (Dis	Andean La	Both	50-69 yea	Sexually tra	Number	1997	949.73	1794.4	526.828
DALYs (Dis	Andean La	Both	50-69 yea	Sexually tra	Rate	1997	21.7596	41.1121	12.0703
DALYs (Dis	Andean La	Both	50-69 yea	Sexually tra	Number	1990	803.748	1489.33	460.957
DALYs (Dis	Andean La	Both	50-69 yea	Sexually tra	Rate	1990	23.0355	42.6845	13.2111
DALYs (Dis	Caribbean	Both	50-69 yea	Sexually tra	Number	1992	1319.41	2254.09	791.348
DALYs (Dis	Caribbean	Both	50-69 yea	Sexually tra	Rate	1992	30.5204	52.1413	18.3054
DALYs (Dis	Central As	Both	50-69 yea	HIV/AIDS	Number	1990	969.478	1031.24	898.098
DALYs (Dis	Central As	Both	50-69 yea	HIV/AIDS	Rate	1990	11.1163	11.8244	10.2978
DALYs (Dis	High-incor	Both	50-69 yea	Sexually tra	Number	1995	4000.86	7448.81	2173.37
DALYs (Dis	High-incor	Both	50-69 yea	Sexually tra	Rate	1995	10.5076	19.5631	5.70801
DALYs (Dis	East Asia	Both	50-69 yea	HIV/AIDS	Number	1997	49709	59768.5	44504.5
DALYs (Dis	East Asia	Both	50-69 yea	HIV/AIDS	Rate	1997	27.2617	32.7786	24.4075
DALYs (Dis	Tropical La	Both	50-69 yea	Sexually tra	Number	1992	4914.47	9096.9	2767.37
DALYs (Dis	Tropical La	Both	50-69 yea	Sexually tra	Rate	1992	28.9444	53.5772	16.2987
DALYs (Dis	South Asia	Both	50-69 yea	HIV/AIDS	Number	1990	5125.3	13404.5	2594.02
DALYs (Dis	South Asia	Both	50-69 yea	HIV/AIDS	Rate	1990	4.77077	12.4773	2.41459
DALYs (Dis	South Asia	Both	50-69 yea	Sexually tra	Number	1990	39136.6	49831.4	28282.6
DALYs (Dis	South Asia	Both	50-69 yea	Sexually tra	Rate	1990	36.4295	46.3844	26.3262
DALYs (Dis	High-incor	Both	50-69 yea	Sexually tra	Number	1996	4022.79	7522.67	2172.35
DALYs (Dis	High-incor	Both	50-69 yea	Sexually tra	Rate	1996	10.5104	19.6545	5.67571
DALYs (Dis	Oceania	Both	50-69 yea	HIV/AIDS	Number	1995	566.911	1467.74	232.994
DALYs (Dis	Oceania	Both	50-69 yea	HIV/AIDS	Rate	1995	93.118	241.084	38.2704
DALYs (Dis	Western S	Both	50-69 yea	HIV/AIDS	Number	1994	468105	650479	325096
DALYs (Dis	Western S	Both	50-69 yea	HIV/AIDS	Rate	1994	2741.19	3809.16	1903.74
DALYs (Dis	North Afric	Both	50-69 yea	Sexually tra	Number	1990	3679.03	6833.32	1969.38
DALYs (Dis	North Afric	Both	50-69 yea	Sexually tra	Rate	1990	11.7119	21.7533	6.26935
DALYs (Dis	High-incor	Both	50-69 yea	HIV/AIDS	Number	1991	150968	158773	145620
DALYs (Dis	High-incor	Both	50-69 yea	HIV/AIDS	Rate	1991	316.275	332.626	305.072
DALYs (Dis	Western E	Both	50-69 yea	Sexually tra	Number	1992	7572.29	13877.6	4393.47
DALYs (Dis	Western E	Both	50-69 yea	Sexually tra	Rate	1992	9.10668	16.6896	5.28372
DALYs (Dis	Caribbean	Both	50-69 yea	HIV/AIDS	Number	1996	85129.5	113782	62217.4
DALYs (Dis	Caribbean	Both	50-69 yea	HIV/AIDS	Rate	1996	1786.77	2388.16	1305.87
DALYs (Dis	North Afric	Both	50-69 yea	HIV/AIDS	Number	1995	9304.52	17825.7	4716.86
DALYs (Dis	North Afric	Both	50-69 yea	HIV/AIDS	Rate	1995	26.3966	50.5708	13.3816
DALYs (Dis	Southeast	Both	50-69 yea	Sexually tra	Number	1992	9311.5	16711.5	5614.12
DALYs (Dis	Southeast	Both	50-69 yea	Sexually tra	Rate	1992	18.8366	33.8063	11.357
DALYs (Dis	Tropical La	Both	50-69 yea	HIV/AIDS	Number	1997	54349.6	58321.4	51773.1
DALYs (Dis	Tropical La	Both	50-69 yea	HIV/AIDS	Rate	1997	278.704	299.071	265.491
DALYs (Dis	Eastern Eu	Both	50-69 yea	HIV/AIDS	Number	1996	30717.6	32724.6	29395
DALYs (Dis	Eastern Eu	Both	50-69 yea	HIV/AIDS	Rate	1996	66.9398	71.3133	64.0575
DALYs (Dis	Oceania	Both	50-69 yea	HIV/AIDS	Number	1993	220.021	816.819	61.0282
DALYs (Dis	Oceania	Both	50-69 yea	HIV/AIDS	Rate	1993	37.4567	139.056	10.3895
DALYs (Dis	East Asia	Both	50-69 yea	Sexually tra	Number	1991	30382.2	48568.4	20388.3
DALYs (Dis	East Asia	Both	50-69 yea	Sexually tra	Rate	1991	18.6529	29.8181	12.5172
DALYs (Dis	Eastern Su	Both	50-69 yea	HIV/AIDS	Number	1991	904772	1230320	613378
DALYs (Dis	Eastern Su	Both	50-69 yea	HIV/AIDS	Rate	1991	6499.38	8837.94	4406.17
DALYs (Dis	Eastern Su	Both	50-69 yea	Sexually tra	Number	1991	18209.2	32917.3	8395.68
DALYs (Dis	Eastern Su	Both	50-69 yea	Sexually tra	Rate	1991	130.805	236.46	60.3099
DALYs (Dis	Western S	Both	50-69 yea	Sexually tra	Number	1998	5170.92	8947.47	3128.18
DALYs (Dis	Western S	Both	50-69 yea	Sexually tra	Rate	1998	28.1395	48.6911	17.0232
DALYs (Dis	Central Eu	Both	50-69 yea	HIV/AIDS	Number	1990	4438.39	4675.74	4150
DALYs (Dis	Central Eu	Both	50-69 yea	HIV/AIDS	Rate	1990	17.6351	18.5782	16.4892
DALYs (Dis	Central Su	Both	50-69 yea	HIV/AIDS	Number	1998	315631	385992	257553
DALYs (Dis	Central Su	Both	50-69 yea	HIV/AIDS	Rate	1998	6295.5	7698.9	5137.09
DALYs (Dis	Central As	Both	50-69 yea	Sexually tra	Number	1990	2152.86	3197.34	1557.12
DALYs (Dis	Central As	Both	50-69 yea	Sexually tra	Rate	1990	24.6852	36.6614	17.8543
DALYs (Dis	South Asia	Both	50-69 yea	HIV/AIDS	Number	1995	46489.8	65404.7	34015.6
DALYs (Dis	South Asia	Both	50-69 yea	HIV/AIDS	Rate	1995	38.4524	54.0973	28.1348
DALYs (Dis	South Asia	Both	50-69 yea	Sexually tra	Number	1995	41113.1	53630.1	30892.2
DALYs (Dis	South Asia	Both	50-69 yea	Sexually tra	Rate	1995	34.0053	44.3583	25.5514
DALYs (Dis	Southern S	Both	50-69 yea	Sexually tra	Number	1997	3487.1	5291.38	2478.42
DALYs (Dis	Southern S	Both	50-69 yea	Sexually tra	Rate	1997	62.1908	94.3693	44.2015
DALYs (Dis	Central Su	Both	50-69 yea	Sexually tra	Number	1992	4097.32	7511.58	1520.1
DALYs (Dis	Central Su	Both	50-69 yea	Sexually tra	Rate	1992	89.3406	163.787	33.1452
DALYs (Dis	Central Su	Both	50-69 yea	Sexually tra	Number	1997	4157.39	7904.05	1599.07
DALYs (Dis	Central Su	Both	50-69 yea	Sexually tra	Rate	1997	84.2064	160.094	32.3886
DALYs (Dis	Caribbean	Both	50-69 yea	Sexually tra	Number	1994	1328.38	2258.61	796.567
DALYs (Dis	Caribbean	Both	50-69 yea	Sexually tra	Rate	1994	29.3248	49.8602	17.5847
DALYs (Dis	Southern L	Both	50-69 yea	HIV/AIDS	Number	1990	5740.4	5970.38	5525.72
DALYs (Dis	Southern L	Both	50-69 yea	HIV/AIDS	Rate	1990	76.6648	79.7362	73.7977
DALYs (Dis	Oceania	Both	50-69 yea	HIV/AIDS	Number	1992	159.724	647.581	37.9168
DALYs (Dis	Oceania	Both	50-69 yea	HIV/AIDS	Rate	1992	27.6068	111.929	6.5536
DALYs (Dis	Western S	Both	50-69 yea	HIV/AIDS	Number	1991	211120	328053	135861
DALYs (Dis	Western S	Both	50-69 yea	HIV/AIDS	Rate	1991	1327.5	2062.76	854.276
DALYs (Dis	Central As	Both	50-69 yea	HIV/AIDS	Number	1992	1190.13	1250.88	1119.15
DALYs (Dis	Central As	Both	50-69 yea	HIV/AIDS	Rate	1992	13.3397	14.0207	12.5441
DALYs (Dis	Tropical La	Both	50-69 yea	HIV/AIDS	Number	1991	33157.2	35091.4	31855.7
DALYs (Dis	Tropical La	Both	50-69 yea	HIV/AIDS	Rate	1991	200.687	212.394	192.81
DALYs (Dis	Southern S	Both	50-69 yea	Sexually tra	Number	1992	3056.13	4871.03	2012.06
DALYs (Dis	Southern S	Both	50-69 yea	Sexually tra	Rate	1992	61.6641	98.2837	40.5977
DALYs (Dis	Southeast	Both	50-69 yea	HIV/AIDS	Number	1990	9724.41	14800.7	6657.43
DALYs (Dis	Southeast	Both	50-69 yea	HIV/AIDS	Rate	1990	20.5858	31.3319	14.0933
DALYs (Dis	Southeast	Both	50-69 yea	Sexually tra	Number	1994	9641.42	17235.4	5762.76
DALYs (Dis	Southeast	Both	50-69 yea	Sexually tra	Rate	1994	18.7274	33.478	11.1936
DALYs (Dis	South Asia	Both	50-69 yea	HIV/AIDS	Number	1996	69186.7	92039.9	53238.6
DALYs (Dis	South Asia	Both	50-69 yea	HIV/AIDS	Rate	1996	56.0135	74.5154	43.1019
DALYs (Dis	South Asia	Both	50-69 yea	Sexually tra	Number	1996	42341.6	55666.8	31703.4
DALYs (Dis	South Asia	Both	50-69 yea	Sexually tra	Rate	1996	34.2797	45.0678	25.6671
DALYs (Dis	Tropical La	Both	50-69 yea	HIV/AIDS	Number	1993	44864.8	47352	43007
DALYs (Dis	Tropical La	Both	50-69 yea	HIV/AIDS	Rate	1993	257.234	271.495	246.583
DALYs (Dis	Central Eu	Both	50-69 yea	Sexually tra	Number	1994	2548.74	4648.88	1438.19
DALYs (Dis	Central Eu	Both	50-69 yea	Sexually tra	Rate	1994	10.296	18.7798	5.80978
DALYs (Dis	Western S	Both	50-69 yea	Sexually tra	Number	1992	4888.03	8182.58	3011.43
DALYs (Dis	Western S	Both	50-69 yea	Sexually tra	Rate	1992	30.0275	50.2661	18.4994
DALYs (Dis	Oceania	Both	50-69 yea	Sexually tra	Number	1994	154.42	280.28	83.3074
DALYs (Dis	Oceania	Both	50-69 yea	Sexually tra	Rate	1994	25.8493	46.9178	13.9453
DALYs (Dis	Western S	Both	50-69 yea	HIV/AIDS	Number	1993	368695	527680	249846
DALYs (Dis	Western S	Both	50-69 yea	HIV/AIDS	Rate	1993	2212.98	3167.24	1499.62
DALYs (Dis	Australasia	Both	50-69 yea	Sexually tra	Number	1993	345.377	662.561	179.874
DALYs (Dis	Australasia	Both	50-69 yea	Sexually tra	Rate	1993	9.68411	18.5777	5.04354
DALYs (Dis	High-incor	Both	50-69 yea	HIV/AIDS	Number	1997	120231	132089	112361
DALYs (Dis	High-incor	Both	50-69 yea	HIV/AIDS	Rate	1997	231.504	254.337	216.351
DALYs (Dis	East Asia	Both	50-69 yea	Sexually tra	Number	1996	27227.2	48498.4	16410.1
DALYs (Dis	East Asia	Both	50-69 yea	Sexually tra	Rate	1996	15.318	27.285	9.23226
DALYs (Dis	Southern L	Both	50-69 yea	Sexually tra	Number	1997	1377.45	2483.23	827.692
DALYs (Dis	Southern L	Both	50-69 yea	Sexually tra	Rate	1997	16.9067	30.479	10.159
DALYs (Dis	High-incor	Both	50-69 yea	Sexually tra	Number	2000	9106.83	17614.5	4882.79
DALYs (Dis	High-incor	Both	50-69 yea	Sexually tra	Rate	2000	16.0734	31.0893	8.61802
DALYs (Dis	High-incor	Both	50-69 yea	Sexually tra	Number	1991	8195.83	15157.9	4657.71
DALYs (Dis	High-incor	Both	50-69 yea	Sexually tra	Rate	1991	17.1701	31.7556	9.75784
DALYs (Dis	Oceania	Both	50-69 yea	Sexually tra	Number	1992	149.372	266.472	81.3482
DALYs (Dis	Oceania	Both	50-69 yea	Sexually tra	Rate	1992	25.8176	46.0574	14.0603
DALYs (Dis	Central Eu	Both	50-69 yea	HIV/AIDS	Number	1994	5021.78	5394.04	4642.01
DALYs (Dis	Central Eu	Both	50-69 yea	HIV/AIDS	Rate	1994	20.2862	21.79	18.752
DALYs (Dis	Central As	Both	50-69 yea	Sexually tra	Number	1994	2254.28	3265.75	1663.77
DALYs (Dis	Central As	Both	50-69 yea	Sexually tra	Rate	1994	26.0036	37.671	19.1919
DALYs (Dis	Eastern Eu	Both	50-69 yea	HIV/AIDS	Number	1990	23377.1	24696.4	22421.8
DALYs (Dis	Eastern Eu	Both	50-69 yea	HIV/AIDS	Rate	1990	47.1421	49.8026	45.2155
DALYs (Dis	High-incor	Both	50-69 yea	Sexually tra	Number	1990	3922.26	7148.76	2203.18
DALYs (Dis	High-incor	Both	50-69 yea	Sexually tra	Rate	1990	11.4976	20.9556	6.45832
DALYs (Dis	Central As	Both	50-69 yea	Sexually tra	Number	1999	1955.11	2830.08	1452.36
DALYs (Dis	Central As	Both	50-69 yea	Sexually tra	Rate	1999	24.7245	35.7895	18.3667
DALYs (Dis	Central Lat	Both	50-69 yea	HIV/AIDS	Number	1996	39588.6	42180.7	38029.8
DALYs (Dis	Central Lat	Both	50-69 yea	HIV/AIDS	Rate	1996	226.104	240.909	217.201
DALYs (Dis	Central Lat	Both	50-69 yea	Sexually tra	Number	1996	4203.7	7936.17	2296.56
DALYs (Dis	Central Lat	Both	50-69 yea	Sexually tra	Rate	1996	24.0088	45.3262	13.1164
DALYs (Dis	High-incor	Both	50-69 yea	HIV/AIDS	Number	1997	4450.5	5063.58	4071.42
DALYs (Dis	High-incor	Both	50-69 yea	HIV/AIDS	Rate	1997	11.3039	12.8611	10.3411
DALYs (Dis	Central Eu	Both	50-69 yea	Sexually tra	Number	1999	2432.88	4505.44	1346.22
DALYs (Dis	Central Eu	Both	50-69 yea	Sexually tra	Rate	1999	9.75746	18.0698	5.39925
DALYs (Dis	Tropical La	Both	50-69 yea	Sexually tra	Number	2000	6222.23	11738.4	3457.03
DALYs (Dis	Tropical La	Both	50-69 yea	Sexually tra	Rate	2000	28.89	54.5017	16.0511
DALYs (Dis	Australasia	Both	50-69 yea	Sexually tra	Number	1991	361.92	689.306	198.271
DALYs (Dis	Australasia	Both	50-69 yea	Sexually tra	Rate	1991	10.4047	19.8167	5.70004
DALYs (Dis	Central Lat	Both	50-69 yea	HIV/AIDS	Number	2000	50206.5	53643.2	48157.9
DALYs (Dis	Central Lat	Both	50-69 yea	HIV/AIDS	Rate	2000	249.095	266.146	238.931
DALYs (Dis	Central Lat	Both	50-69 yea	Sexually tra	Number	2000	4733.59	8960.2	2538.51
DALYs (Dis	Central Lat	Both	50-69 yea	Sexually tra	Rate	2000	23.4853	44.4552	12.5946
DALYs (Dis	Australasia	Both	50-69 yea	HIV/AIDS	Number	1999	1849.65	2036.92	1726.56
DALYs (Dis	Australasia	Both	50-69 yea	HIV/AIDS	Rate	1999	44.5634	49.0753	41.5978
DALYs (Dis	South Asia	Both	50-69 yea	HIV/AIDS	Number	1994	31909.4	48181.3	22212
DALYs (Dis	South Asia	Both	50-69 yea	HIV/AIDS	Rate	1994	26.9888	40.7516	18.7868
DALYs (Dis	South Asia	Both	50-69 yea	Sexually tra	Number	1994	40893.4	52800.1	30534.6
DALYs (Dis	South Asia	Both	50-69 yea	Sexually tra	Rate	1994	34.5875	44.6581	25.826
DALYs (Dis	East Asia	Both	50-69 yea	Sexually tra	Number	2000	27238.7	49948.7	16096.7
DALYs (Dis	East Asia	Both	50-69 yea	Sexually tra	Rate	2000	13.7238	25.1659	8.11005
DALYs (Dis	North Afric	Both	50-69 yea	HIV/AIDS	Number	1994	7856.38	15328.8	3940.34
DALYs (Dis	North Afric	Both	50-69 yea	HIV/AIDS	Rate	1994	22.814	44.513	11.4423

DALYs (Dis	Tropical La	Both	50-69 yea	Sexually tra	Rate	1993	29.0319	53.9232	16.4218
DALYs (Dis	East Asia	Both	50-69 yea	HIV/AIDS	Number	2001	81379.7	107014	67767.7
DALYs (Dis	East Asia	Both	50-69 yea	HIV/AIDS	Rate	2001	39.5379	51.9923	32.9246
DALYs (Dis	Southern S	Both	50-69 yea	Sexually tra	Number	2001	3670.86	5736.08	2563.87
DALYs (Dis	Southern S	Both	50-69 yea	Sexually tra	Rate	2001	59.7233	93.3236	41.7131
DALYs (Dis	Andean La	Both	50-69 yea	Sexually tra	Number	2001	1077.32	2011.69	587.95
DALYs (Dis	Andean La	Both	50-69 yea	Sexually tra	Rate	2001	21.6298	40.3895	11.8045
DALYs (Dis	Tropical La	Both	50-69 yea	HIV/AIDS	Number	2001	71428.7	73849.9	69444.7
DALYs (Dis	Tropical La	Both	50-69 yea	HIV/AIDS	Rate	2001	320.546	331.411	311.642
DALYs (Dis	Central Eu	Both	50-69 yea	HIV/AIDS	Number	1993	5938.69	6298.53	5563.56
DALYs (Dis	Central Eu	Both	50-69 yea	HIV/AIDS	Rate	1993	23.8452	25.2901	22.339
DALYs (Dis	High-incor	Both	50-69 yea	HIV/AIDS	Number	1992	2123.57	2421.38	1955.44
DALYs (Dis	High-incor	Both	50-69 yea	HIV/AIDS	Rate	1992	5.87779	6.70209	5.41243
DALYs (Dis	Eastern Eu	Both	50-69 yea	Sexually tra	Number	1999	13861.1	18609.3	8312.89
DALYs (Dis	Eastern Eu	Both	50-69 yea	Sexually tra	Rate	1999	31.0327	41.6632	18.6111
DALYs (Dis	Western E	Both	50-69 yea	Sexually tra	Number	1997	7022.76	13393.4	3781.78
DALYs (Dis	Western E	Both	50-69 yea	Sexually tra	Rate	1997	8.24255	15.7197	4.43865
DALYs (Dis	Andean La	Both	50-69 yea	HIV/AIDS	Number	1998	8804.91	21958.5	5996.03
DALYs (Dis	Andean La	Both	50-69 yea	HIV/AIDS	Rate	1998	195.055	486.446	132.83
DALYs (Dis	Southern L	Both	50-69 yea	Sexually tra	Number	1992	1394.19	2435.24	854.978
DALYs (Dis	Southern L	Both	50-69 yea	Sexually tra	Rate	1992	18.1704	31.7384	11.1429
DALYs (Dis	Eastern Eu	Both	50-69 yea	Sexually tra	Number	1993	15346.8	20944.9	10233.9
DALYs (Dis	Eastern Eu	Both	50-69 yea	Sexually tra	Rate	1993	30.6158	41.7835	20.4159
DALYs (Dis	North Afric	Both	50-69 yea	Sexually tra	Number	1994	4000.5	7566.23	2151.04
DALYs (Dis	North Afric	Both	50-69 yea	Sexually tra	Rate	1994	11.617	21.9715	6.24637
DALYs (Dis	Central As	Both	50-69 yea	Sexually tra	Number	1992	2267.34	3351.02	1664.47
DALYs (Dis	Central As	Both	50-69 yea	Sexually tra	Rate	1992	25.4137	37.5603	18.6564
DALYs (Dis	Oceania	Both	50-69 yea	HIV/AIDS	Number	2000	4978.11	11004.3	2009.77
DALYs (Dis	Oceania	Both	50-69 yea	HIV/AIDS	Rate	2000	726.557	1606.08	293.327
DALYs (Dis	Central Eu	Both	50-69 yea	HIV/AIDS	Number	2000	5609.21	5936.97	5256.38
DALYs (Dis	Central Eu	Both	50-69 yea	HIV/AIDS	Rate	2000	22.2426	23.5423	20.8435
DALYs (Dis	High-incor	Both	50-69 yea	HIV/AIDS	Number	1995	227398	244128	217435
DALYs (Dis	High-incor	Both	50-69 yea	HIV/AIDS	Rate	1995	456.145	489.705	436.16
DALYs (Dis	Eastern Eu	Both	50-69 yea	Sexually tra	Number	1990	10955.1	16392.7	7910.2
DALYs (Dis	Eastern Eu	Both	50-69 yea	Sexually tra	Rate	1990	22.0919	33.0573	15.9516
DALYs (Dis	Central Su	Both	50-69 yea	Sexually tra	Number	1993	4118.7	7585.92	1544.27
DALYs (Dis	Central Su	Both	50-69 yea	Sexually tra	Rate	1993	88.4215	162.857	33.1528
DALYs (Dis	Western S	Both	50-69 yea	Sexually tra	Number	1991	4795.13	7952.07	2987.32
DALYs (Dis	Western S	Both	50-69 yea	Sexually tra	Rate	1991	30.1512	50.0017	18.7839
DALYs (Dis	Southeast	Both	50-69 yea	HIV/AIDS	Number	2000	281326	309158	248992
DALYs (Dis	Southeast	Both	50-69 yea	HIV/AIDS	Rate	2000	480.324	527.844	425.118
DALYs (Dis	Central Lat	Both	50-69 yea	HIV/AIDS	Number	1991	22816.6	23471.8	22311.7
DALYs (Dis	Central Lat	Both	50-69 yea	HIV/AIDS	Rate	1991	153.498	157.906	150.101
DALYs (Dis	Central Lat	Both	50-69 yea	Sexually tra	Number	1991	3878.22	7151.19	2237.58
DALYs (Dis	Central Lat	Both	50-69 yea	Sexually tra	Rate	1991	26.0906	48.1093	15.0532
DALYs (Dis	Andean La	Both	50-69 yea	HIV/AIDS	Number	1994	6327.33	16478	3893.74
DALYs (Dis	Andean La	Both	50-69 yea	HIV/AIDS	Rate	1994	159.833	416.246	98.3585
DALYs (Dis	East Asia	Both	50-69 yea	Sexually tra	Number	1995	28176.1	49035.8	17736.2
DALYs (Dis	East Asia	Both	50-69 yea	Sexually tra	Rate	1995	16.2263	28.2391	10.214
DALYs (Dis	Caribbean	Both	50-69 yea	Sexually tra	Number	1999	1462.9	2508.48	896.109
DALYs (Dis	Caribbean	Both	50-69 yea	Sexually tra	Rate	1999	28.3068	48.5385	17.3395
DALYs (Dis	Southern L	Both	50-69 yea	Sexually tra	Number	2001	1412.9	2585.91	825.524
DALYs (Dis	Southern L	Both	50-69 yea	Sexually tra	Rate	2001	16.3112	29.8528	9.53022
DALYs (Dis	Southern L	Both	50-69 yea	HIV/AIDS	Number	1993	8193.31	8488.09	7931.74
DALYs (Dis	Southern L	Both	50-69 yea	HIV/AIDS	Rate	1993	105.545	109.342	102.175
DALYs (Dis	Western S	Both	50-69 yea	HIV/AIDS	Number	1999	1013132	1224373	817466
DALYs (Dis	Western S	Both	50-69 yea	HIV/AIDS	Rate	1999	5409.58	6537.49	4364.83
DALYs (Dis	High-incor	Both	50-69 yea	HIV/AIDS	Number	2001	5460.27	6258.72	4915.3
DALYs (Dis	High-incor	Both	50-69 yea	HIV/AIDS	Rate	2001	12.6545	14.505	11.3915
DALYs (Dis	Central Su	Both	50-69 yea	Sexually tra	Number	2001	4059.85	7894.11	1648.21
DALYs (Dis	Central Su	Both	50-69 yea	Sexually tra	Rate	2001	76.7498	149.235	31.1587
DALYs (Dis	Australasia	Both	50-69 yea	Sexually tra	Number	2002	393.105	768.039	195.23
DALYs (Dis	Australasia	Both	50-69 yea	Sexually tra	Rate	2002	8.64312	16.8867	4.29248
DALYs (Dis	Western E	Both	50-69 yea	HIV/AIDS	Number	2002	48482.2	52842.4	45636.5
DALYs (Dis	Western E	Both	50-69 yea	HIV/AIDS	Rate	2002	53.5885	58.408	50.4431
DALYs (Dis	Australasia	Both	50-69 yea	Sexually tra	Number	1995	343.659	673.777	174.564
DALYs (Dis	Australasia	Both	50-69 yea	Sexually tra	Rate	1995	9.28934	18.2127	4.71858
DALYs (Dis	Western E	Both	50-69 yea	HIV/AIDS	Number	1993	84217.4	88159.9	81226.6
DALYs (Dis	Western E	Both	50-69 yea	HIV/AIDS	Rate	1993	101.058	105.789	97.4695
DALYs (Dis	East Asia	Both	50-69 yea	HIV/AIDS	Number	1993	33205.7	38197.8	19730.2
DALYs (Dis	East Asia	Both	50-69 yea	HIV/AIDS	Rate	1993	19.7498	22.719	11.7349
DALYs (Dis	Tropical La	Both	50-69 yea	Sexually tra	Number	1991	4765.63	8789.58	2708.33
DALYs (Dis	Tropical La	Both	50-69 yea	Sexually tra	Rate	1991	28.8444	53.1997	16.3924
DALYs (Dis	Tropical La	Both	50-69 yea	HIV/AIDS	Number	1995	57813.8	61114.7	55509.3
DALYs (Dis	Tropical La	Both	50-69 yea	HIV/AIDS	Rate	1995	314.221	332.161	301.696
DALYs (Dis	Andean La	Both	50-69 yea	HIV/AIDS	Number	1990	3585.46	8853.03	2270.22
DALYs (Dis	Andean La	Both	50-69 yea	HIV/AIDS	Rate	1990	102.76	253.729	65.0649
DALYs (Dis	Central Eu	Both	50-69 yea	HIV/AIDS	Number	1992	4626.31	4934.8	4286.79
DALYs (Dis	Central Eu	Both	50-69 yea	HIV/AIDS	Rate	1992	18.4632	19.6944	17.1082
DALYs (Dis	High-incor	Both	50-69 yea	Sexually tra	Number	1993	3977.29	7392.17	2179.56
DALYs (Dis	High-incor	Both	50-69 yea	Sexually tra	Rate	1993	10.7646	20.007	5.89899
DALYs (Dis	High-incor	Both	50-69 yea	HIV/AIDS	Number	1993	2321.84	2700.37	2121.91
DALYs (Dis	High-incor	Both	50-69 yea	HIV/AIDS	Rate	1993	6.28408	7.30859	5.74297
DALYs (Dis	Central Su	Both	50-69 yea	HIV/AIDS	Number	1991	132109	189644	89121.5
DALYs (Dis	Central Su	Both	50-69 yea	HIV/AIDS	Rate	1991	2927.63	4202.63	1974.99
DALYs (Dis	North Afric	Both	50-69 yea	HIV/AIDS	Number	1992	5422.63	11317	2625.92
DALYs (Dis	North Afric	Both	50-69 yea	HIV/AIDS	Rate	1992	16.4843	34.4027	7.98254
DALYs (Dis	Southeast	Both	50-69 yea	HIV/AIDS	Number	1992	36508.6	54470.2	21894.9
DALYs (Dis	Southeast	Both	50-69 yea	HIV/AIDS	Rate	1992	73.8547	110.19	44.2919
DALYs (Dis	Australasia	Both	50-69 yea	Sexually tra	Number	1998	367.201	724.762	183.242
DALYs (Dis	Australasia	Both	50-69 yea	Sexually tra	Rate	1998	9.11042	17.9817	4.54632
DALYs (Dis	North Afric	Both	50-69 yea	HIV/AIDS	Number	1996	10864.5	20665.4	5572.23
DALYs (Dis	North Afric	Both	50-69 yea	HIV/AIDS	Rate	1996	30.1128	57.2778	15.4444
DALYs (Dis	South Asia	Both	50-69 yea	HIV/AIDS	Number	1993	20796.2	35457.3	13401.4
DALYs (Dis	South Asia	Both	50-69 yea	HIV/AIDS	Rate	1993	18.0008	30.6913	11.6
DALYs (Dis	South Asia	Both	50-69 yea	Sexually tra	Number	1993	40470.9	52151.8	29532.4
DALYs (Dis	South Asia	Both	50-69 yea	Sexually tra	Rate	1993	35.031	45.1417	25.5627
DALYs (Dis	Southern S	Both	50-69 yea	HIV/AIDS	Number	1993	216290	388262	101666
DALYs (Dis	Southern S	Both	50-69 yea	HIV/AIDS	Rate	1993	4253.72	7635.82	1999.44
DALYs (Dis	Western S	Both	50-69 yea	HIV/AIDS	Number	1995	574171	771820	413277
DALYs (Dis	Western S	Both	50-69 yea	HIV/AIDS	Rate	1995	3304.74	4442.35	2378.69
DALYs (Dis	Southern L	Both	50-69 yea	Sexually tra	Number	1994	1364.21	2424.58	816.052
DALYs (Dis	Southern L	Both	50-69 yea	Sexually tra	Rate	1994	17.369	30.8694	10.3899
DALYs (Dis	Southern S	Both	50-69 yea	HIV/AIDS	Number	1998	1003083	1381912	663814
DALYs (Dis	Southern S	Both	50-69 yea	HIV/AIDS	Rate	1998	17494.5	24101.5	11577.4
DALYs (Dis	Andean La	Both	50-69 yea	Sexually tra	Number	1994	882.879	1651.41	487.207
DALYs (Dis	Andean La	Both	50-69 yea	Sexually tra	Rate	1994	22.3021	41.7157	12.3072
DALYs (Dis	Andean La	Both	50-69 yea	HIV/AIDS	Number	2002	13351.9	24147.2	10099.6
DALYs (Dis	Andean La	Both	50-69 yea	HIV/AIDS	Rate	2002	259.059	468.514	195.957
DALYs (Dis	High-incor	Both	50-69 yea	HIV/AIDS	Number	2001	142480	154928	133343
DALYs (Dis	High-incor	Both	50-69 yea	HIV/AIDS	Rate	2001	244.173	265.504	228.513
DALYs (Dis	North Afric	Both	50-69 yea	Sexually tra	Number	1992	3839.83	7245.53	2083.68
DALYs (Dis	North Afric	Both	50-69 yea	Sexually tra	Rate	1992	11.6727	22.0257	6.33418
DALYs (Dis	Southern L	Both	50-69 yea	HIV/AIDS	Number	2002	12499.6	13383.2	11907
DALYs (Dis	Southern L	Both	50-69 yea	HIV/AIDS	Rate	2002	141.774	151.796	135.053
DALYs (Dis	Central Eu	Both	50-69 yea	Sexually tra	Number	1992	2651.03	4748.54	1553.21
DALYs (Dis	Central Eu	Both	50-69 yea	Sexually tra	Rate	1992	10.58	18.951	6.19871
DALYs (Dis	High-incor	Both	50-69 yea	HIV/AIDS	Number	1994	2469.19	2959.34	2229.37
DALYs (Dis	High-incor	Both	50-69 yea	HIV/AIDS	Rate	1994	6.54202	7.84063	5.9066
DALYs (Dis	Australasia	Both	50-69 yea	HIV/AIDS	Number	1991	2698.51	2864.38	2546.32
DALYs (Dis	Australasia	Both	50-69 yea	HIV/AIDS	Rate	1991	77.5786	82.3471	73.2034
DALYs (Dis	Southern L	Both	50-69 yea	HIV/AIDS	Number	1998	8644.89	9557.53	8249.34
DALYs (Dis	Southern L	Both	50-69 yea	HIV/AIDS	Rate	1998	104.655	115.703	99.8663
DALYs (Dis	East Asia	Both	50-69 yea	HIV/AIDS	Number	1995	41907.7	48773.8	37098.9
DALYs (Dis	East Asia	Both	50-69 yea	HIV/AIDS	Rate	1995	24.1341	28.0882	21.3648
DALYs (Dis	Southern S	Both	50-69 yea	HIV/AIDS	Number	2002	1982628	2380788	1564205
DALYs (Dis	Southern S	Both	50-69 yea	HIV/AIDS	Rate	2002	31392	37696.3	24766.9
DALYs (Dis	Western S	Both	50-69 yea	Sexually tra	Number	2002	5158.77	9452.15	2968.03
DALYs (Dis	Western S	Both	50-69 yea	Sexually tra	Rate	2002	25.4996	46.7216	14.6708
DALYs (Dis	Central Su	Both	50-69 yea	Sexually tra	Number	1995	4174.62	7800.63	1565.57
DALYs (Dis	Central Su	Both	50-69 yea	Sexually tra	Rate	1995	87.0574	162.674	32.6484
DALYs (Dis	High-incor	Both	50-69 yea	Sexually tra	Number	1994	8266.11	15563.5	4642.31
DALYs (Dis	High-incor	Both	50-69 yea	Sexually tra	Rate	1994	16.7824	31.5979	9.42509
DALYs (Dis	Southern S	Both	50-69 yea	Sexually tra	Number	1993	3059.57	4872.13	2000.17
DALYs (Dis	Southern S	Both	50-69 yea	Sexually tra	Rate	1993	60.1717	95.8187	39.3368
DALYs (Dis	Southeast	Both	50-69 yea	Sexually tra	Number	1996	9976.59	17992.1	5867.09
DALYs (Dis	Southeast	Both	50-69 yea	Sexually tra	Rate	1996	18.5603	33.4721	10.915
DALYs (Dis	Eastern Eu	Both	50-69 yea	HIV/AIDS	Number	1994	32445.5	34463	31144.9
DALYs (Dis	Eastern Eu	Both	50-69 yea	HIV/AIDS	Rate	1994	66.6181	70.7607	63.9477
DALYs (Dis	Australasia	Both	50-69 yea	HIV/AIDS	Number	1995	3920.98	4201.36	3693.23
DALYs (Dis	Australasia	Both	50-69 yea	HIV/AIDS	Rate	1995	105.987	113.566	99.8305
DALYs (Dis	Caribbean	Both	50-69 yea	Sexually tra	Number	1995	1342.66	2278.21	795.361
DALYs (Dis	Caribbean	Both	50-69 yea	Sexually tra	Rate	1995	28.9182	49.0681	17.1305
DALYs (Dis	Oceania	Both	50-69 yea	Sexually tra	Number	1999	175.013	321.645	90.6776
DALYs (Dis	Oceania	Both	50-69 yea	Sexually tra	Rate	1999	25.9777	47.7427	13.4596
DALYs (Dis	Central Su	Both	50-69 yea	Sexually tra	Number	1991	4081.67	7540.71	1496.75
DALYs (Dis	Central Su	Both	50-69 yea	Sexually tra	Rate	1991	90.4525	167.107	33.169
DALYs (Dis	Central Lat	Both	50-69 yea	HIV/AIDS	Number	1993	32693	34171.8	31686.4
DALYs (Dis	Central Lat	Both	50-69 yea	HIV/AIDS	Rate	1993	206.387	215.723	200.033
DALYs (Dis	Central Lat	Both	50-69 yea	Sexually tra	Number	1993	3977.57	7387.37	2261.5
DALYs (Dis	Central Lat	Both	50-69 yea	Sexually tra	Rate	1993	25.1099	46.6356	14.2766
DALYs (Dis	Tropical La	Both	50-69 yea	Sexually tra	Number	1996	5467.09	10245.8	3055.59
DALYs (Dis	Tropical La	Both	50-69 yea	Sexually tra	Rate	1996	28.9255	54.2091	16.1667
DALYs (Dis	High-incor	Both	50-69 yea	Sexually tra	Number	1996	8331.14	15889.9	4608.5
DALYs (Dis	High-incor	Both	50-69 yea	Sexually tra	Rate	1996	16.5231	31.5144	9.14002
DALYs (Dis	South Asia	Both	50-69 yea	HIV/AIDS	Number	1992	12758.2	24681.8	7355.67
DALYs (Dis	South Asia	Both	50-69 yea	HIV/AIDS	Rate	1992	11.3066	21.8735	6.51875
DALYs (Dis	South Asia	Both	50-69 yea	Sexually tra	Number	1992	40595.7	51937.3	29479.2
DALYs (Dis	South Asia	Both	50-69 yea	Sexually tra	Rate	1992	35.9768	46.028	26.1251
DALYs (Dis	Western E	Both	50-69 yea	HIV/AIDS	Number	1995	100123	105213	96414.4
DALYs (Dis	Western E	Both	50-69 yea	HIV/AIDS	Rate	1995	119.42	125.49	114.996
DALYs (Dis	Western E	Both	50-69 yea	Sexually tra	Number	1993	7396.6	13744.1	4193.44
DALYs (Dis	Western E	Both	50-69 yea	Sexually tra	Rate	1993	8.8757	16.4925	5.032
DALYs (Dis	Southern L	Both	50-69 yea	Sexually tra	Number	1993	1373.9	2419.67	833.847
DALYs (Dis	Southern L	Both	50-69 yea	Sexually tra	Rate	1993	17.6984	31.1698	10.7415
DALYs (Dis	Southeast	Both	50-69 yea	Sexually tra	Number	1995	9797.81	17534.2	5853.35
DALYs (Dis	Southeast	Both	50-69 yea	Sexually tra	Rate	1995	18.6441	33.3656	11.1382
DALYs (Dis	Western S	Both	50-69 yea	Sexually tra	Number	1995	5101.06	8639.33	3145.62
DALYs (Dis	Western S	Both	50-69 yea	Sexually tra	Rate	1995	29.3601	49.7252	18.1052
DALYs (Dis	Central Lat	Both	50-69 yea	HIV/AIDS	Number	1994	36058.2	37864.4	34774.9
DALYs (Dis	Central Lat	Both	50-69 yea	HIV/AIDS	Rate	1994	220.314	231.349	212.473
DALYs (Dis	Central Lat	Both	50-69 yea	Sexually tra	Number	1994	4031.58	7548.93	2267.75
DALYs (Dis	Central Lat	Both	50-69 yea	Sexually tra	Rate	1994	24.6327	46.1236	13.8559
DALYs (Dis	Western S	Both	50-69 yea	Sexually tra	Number	1994	5071.75	8504.77	3137.74
DALYs (Dis	Western S	Both	50-69 yea	Sexually tra	Rate	1994	29.6998	49.8034	18.3744
DALYs (Dis	Caribbean	Both	50-69 yea	Sexually tra	Number	2003	1608.2	2778.45	999.399
DALYs (Dis	Caribbean	Both	50-69 yea	Sexually tra	Rate	2003	28.0509	48.4628	17.4319
DALYs (Dis	Central As	Both	50-69 yea	Sexually tra	Number	1995	2260.7	3246.63	1671.95
DALYs (Dis	Central As	Both	50-69 yea	Sexually tra	Rate	1995	26.7622	38.4337	19.7927
DALYs (Dis	Australasia	Both	50-69 yea	Sexually tra	Number	1994	344.482	669.543	180.46
DALYs (Dis	Australasia	Both	50-69 yea	Sexually tra	Rate	1994	9.48946	18.4439	4.97115
DALYs (Dis	Central Su	Both	50-69 yea	Sexually tra	Number	1994	4153.02	7744.65	1547.84
DALYs (Dis	Central Su	Both	50-69 yea	Sexually tra	Rate	1994	87.8311	163.789	32.7348
DALYs (Dis	Andean La	Both	50-69 yea	HIV/AIDS	Number	1993	5644.83	14546.3	3423.64
DALYs (Dis	Andean La	Both	50-69 yea	HIV/AIDS	Rate	1993	147.077	379.007	89.2035
DALYs (Dis	Western E	Both	50-69 yea	Sexually tra	Number	2001	7033.78	13858.6	3594.25
DALYs (Dis	Western E	Both	50-69 yea	Sexually tra	Rate	2001	7.8654	15.4971	4.0192
DALYs (Dis	Oceania	Both	50-69 yea	Sexually tra	Number	2003	195.593	360.142	103.043
DALYs (Dis	Oceania	Both	50-69 yea	Sexually tra	Rate	2003	25.8753	47.6439	13.6318
DALYs (Dis	Southeast	Both	50-69 yea	Sexually tra	Number	2003	11498.7	20663.1	6640.59
DALYs (Dis	Southeast	Both	50-69 yea	Sexually tra	Rate	2003	17.9049	32.1751	10.3402
DALYs (Dis	Central As	Both	50-69 yea	Sexually tra	Number	2003	1731.82	2700.03	1229.56
DALYs (Dis	Central As	Both	50-69 yea	Sexually tra	Rate	2003	20.983	32.714	14.8976
DALYs (Dis	Australasia	Both	50-69 yea	HIV/AIDS	Number	2003	1616.18	1753.52	1496.56
DALYs (Dis	Australasia	Both	50-69 yea	HIV/AIDS	Rate	2003	34.4171	37.3417	31.8696
DALYs (Dis	Tropical La	Both	50-69 yea	HIV/AIDS	Number	1994	50381	53450.2	48351.1
DALYs (Dis	Tropical La	Both	50-69 yea	HIV/AIDS	Rate	1994	281.275	298.411	269.943
DALYs (Dis	North Afric	Both	50-69 yea	Sexually tra	Number	2003	4901.12	9337.83	2568.15
DALYs (Dis	North Afric	Both	50-69 yea	Sexually tra	Rate	2003	11.2926	21.5152	5.91725
DALYs (Dis	Eastern Eu	Both	50-69 yea	Sexually tra	Number	2003	10342.7	15575.7	7533.28
DALYs (Dis	Eastern Eu	Both	50-69 yea	Sexually tra	Rate	2003	22.582	34.0077	16.448
DALYs (Dis	Central Eu	Both	50-69 yea	Sexually tra	Number	2003	2555.65	4761.1	1388.69
DALYs (Dis	Central Eu	Both	50-69 yea	Sexually tra	Rate	2003	9.72497	18.1173	5.28436
DALYs (Dis	Southeast	Both	50-69 yea	HIV/AIDS	Number	1995	125265	144993	94413.5
DALYs (Dis	Southeast	Both	50-69 yea	HIV/AIDS	Rate	1995	238.365	275.905	179.658
DALYs (Dis	Central Su	Both	50-69 yea	HIV/AIDS	Number	1995	255835	328158	194637
DALYs (Dis	Central Su	Both	50-69 yea	HIV/AIDS	Rate	1995	5335.17	6843.4	4058.95
DALYs (Dis	Oceania	Both	50-69 yea	Sexually tra	Number	1996	160.484	299.143	84.5737
DALYs (Dis	Oceania	Both	50-69 yea	Sexually tra	Rate	1996	25.8075	48.1054	13.6004
DALYs (Dis	Eastern Eu	Both	50-69 yea	HIV/AIDS	Number	1993	28682.7	30580.8	27439.4
DALYs (Dis	Eastern Eu	Both	50-69 yea	HIV/AIDS	Rate	1993	57.2199	61.0065	54.7396
DALYs (Dis	Andean La	Both	50-69 yea	Sexually tra	Number	1993	863.329	1611.9	476.323
DALYs (Dis	Andean La	Both	50-69 yea	Sexually tra	Rate	1993	22.4942	41.9983	12.4107
DALYs (Dis	Southern S	Both	50-69 yea	Sexually tra	Number	1995	3248.78	5107.44	2156.38
DALYs (Dis	Southern S	Both	50-69 yea	Sexually tra	Rate	1995	60.9261	95.7826	40.4397
DALYs (Dis	High-incor	Both	50-69 yea	HIV/AIDS	Number	1993	188538	199967	180929
DALYs (Dis	High-incor	Both	50-69 yea	HIV/AIDS	Rate	1993	387.533	411.023	371.893
DALYs (Dis	North Afric	Both	50-69 yea	HIV/AIDS	Number	1993	6597.74	13268.1	3289.73
DALYs (Dis	North Afric	Both	50-69 yea	HIV/AIDS	Rate	1993	19.6023	39.4203	9.77398
DALYs (Dis	High-incor	Both	50-69 yea	HIV/AIDS	Number	1996	3900.18	4527.39	3552.96
DALYs (Dis	High-incor	Both	50-69 yea	HIV/AIDS	Rate	1996	10.19	11.8287	9.28286
DALYs (Dis	Central Eu	Both	50-69 yea	Sexually tra	Number	1996	2461.86	4505.58	1370.44
DALYs (Dis	Central Eu	Both	50-69 yea	Sexually tra	Rate	1996	10.0531	18.3986	5.59624
DALYs (Dis	Eastern Su	Both	50-69 yea	HIV/AIDS	Number	2003	3099626	3415311	2820016
DALYs (Dis	Eastern Su	Both	50-69 yea	HIV/AIDS	Rate	2003	17575.3	19365.3	15989.9
DALYs (Dis	Eastern Su	Both	50-69 yea	Sexually tra	Number	2003	17619.9	31067.7	8758.29
DALYs (Dis	Eastern Su	Both	50-69 yea	Sexually tra	Rate	2003	99.9076	176.159	49.6608
DALYs (Dis	Western S	Both	50-69 yea	HIV/AIDS	Number	2003	1318422	1491421	1167369
DALYs (Dis	Western S	Both	50-69 yea	HIV/AIDS	Rate	2003	6358.46	7192.8	5629.96
DALYs (Dis	East Asia	Both	50-69 yea	Sexually tra	Number	1994	29776.6	49739	19372.8
DALYs (Dis	East Asia	Both	50-69 yea	Sexually tra	Rate	1994	17.4452	29.1405	11.3499
DALYs (Dis	Central Su	Both	50-69 yea	HIV/AIDS	Number	2002	362410	428149	307246
DALYs (Dis	Central Su	Both	50-69 yea	HIV/AIDS	Rate	2002	6650.68	7857.09	5638.35
DALYs (Dis	Western E	Both	50-69 yea	HIV/AIDS	Number	1998	48055.1	53150.7	44717.1
DALYs (Dis	Western E	Both	50-69 yea	HIV/AIDS	Rate	1998	55.7107	61.618	51.8409
DALYs (Dis	Caribbean	Both	50-69 yea	Sexually tra	Number	1996	1357.23	2342.98	801.788
DALYs (Dis	Caribbean	Both	50-69 yea	Sexually tra	Rate	1996	28.4867	49.1765	16.8286
DALYs (Dis	Western E	Both	50-69 yea	HIV/AIDS	Number	1996	86561.9	91441.5	82923.1
DALYs (Dis	Western E	Both	50-69 yea	HIV/AIDS	Rate	1996	102.749	108.541	98.4296
DALYs (Dis	Central Su	Both	50-69 yea	HIV/AIDS	Number	1994	227375	298378	167134
DALYs (Dis	Central Su	Both	50-69 yea	HIV/AIDS	Rate	1994	4808.68	6310.31	3534.67
DALYs (Dis	High-incor	Both	50-69 yea	HIV/AIDS	Number	1995	3744.19	4337.61	3452.48
DALYs (Dis	High-incor	Both	50-69 yea	HIV/AIDS	Rate	1995	9.83351	11.392	9.06738
DALYs (Dis	East Asia	Both	50-69 yea	HIV/AIDS	Number	1994	36757.2	42263.2	27464.7
DALYs (Dis	East Asia	Both	50-69 yea	HIV/AIDS	Rate	1994	21.5349	24.7607	16.0907
DALYs (Dis	Oceania	Both	50-69 yea	Sexually tra	Number	1995	157.212	290.291	83.375
DALYs (Dis	Oceania	Both	50-69 yea	Sexually tra	Rate	1995	25.8229	47.6817	13.6948
DALYs (Dis	Central Eu	Both	50-69 yea	Sexually tra	Number	1995	2492.85	4589.42	1383.67
DALYs (Dis	Central Eu	Both	50-69 yea	Sexually tra	Rate	1995	10.1411	18.6701	5.62889
DALYs (Dis	Eastern Su	Both	50-69 yea	HIV/AIDS	Number	1993	1373500	1793753	976880
DALYs (Dis	Eastern Su	Both	50-69 yea	HIV/AIDS	Rate	1993	9597.87	12534.5	6826.33
DALYs (Dis	Eastern Su	Both	50-69 yea	Sexually tra	Number	1993	18320.8	33071.8	8422.5
DALYs (Dis	Eastern Su	Both	50-69 yea	Sexually tra	Rate	1993	128.024	231.102	58.8555
DALYs (Dis	High-incor	Both	50-69 yea	Sexually tra	Number	1993	8225.74	15355.6	4648.39
DALYs (Dis	High-incor	Both	50-69 yea	Sexually tra	Rate	1993	16.9077	31.5629	9.55458
DALYs (Dis	Southern L	Both	50-69 yea	Sexually tra	Number	1995	1360.04	2414.81	804.827
DALYs (Dis	Southern L	Both	50-69 yea	Sexually tra	Rate	1995	17.1073	30.3749	10.1236
DALYs (Dis	Eastern Su	Both	50-69 yea	HIV/AIDS	Number	1999	2695746	3124217	2262881
DALYs (Dis	Eastern Su	Both	50-69 yea	HIV/AIDS	Rate	1999	16730.1	19389.3	14043.7
DALYs (Dis	Eastern Su	Both	50-69 yea	Sexually tra	Number	1999	18451.5	33797.9	9019.11
DALYs (Dis	Eastern Su	Both	50-69 yea	Sexually tra	Rate	1999	114.512	209.754	55.9737
DALYs (Dis	East Asia	Both	50-69 yea	Sexually tra	Number	2004	31890.1	56342	18923.8
DALYs (Dis	East Asia	Both	50-69 yea	Sexually tra	Rate	2004	13.7807	24.3472	8.17759
DALYs (Dis	Oceania	Both	50-69 yea	HIV/AIDS	Number	2004	13012.9	27858.2	5037.88
DALYs (Dis	Oceania	Both	50-69 yea	HIV/AIDS	Rate	2004	1664	3562.32	644.211
DALYs (Dis	Eastern Eu	Both	50-69 yea	HIV/AIDS	Number	2004	51235.4	55596.4	48709.3
DALYs (Dis	Eastern Eu	Both	50-69 yea	HIV/AIDS	Rate	2004	110.228	119.611	104.794
DALYs (Dis	High-incor	Both	50-69 yea	Sexually tra	Number	2004	4432.95	8442.54	2339.96
DALYs (Dis	High-incor	Both	50-69 yea	Sexually tra	Rate	2004	9.9556	18.9604	5.25512
DALYs (Dis	Central Eu	Both	50-69 yea	HIV/AIDS	Number	2004	5322.39	5753.33	5025.83
DALYs (Dis	Central Eu	Both	50-69 yea	HIV/AIDS	Rate	2004	19.9359	21.55	18.825
DALYs (Dis	Central As	Both	50-69 yea	HIV/AIDS	Number	2004	2719.12	3037.57	2502.22
DALYs (Dis	Central As	Both	50-69 yea	HIV/AIDS	Rate	2004	32.1431	35.9076	29.5791
DALYs (Dis	Southeast	Both	50-69 yea	HIV/AIDS	Number	2004	357562	414847	309100
DALYs (Dis	Southeast	Both	50-69 yea	HIV/AIDS	Rate	2004	538.888	625.222	465.849
DALYs (Dis	Central As	Both	50-69 yea	Sexually tra	Number	1996	2223.48	3210.43	1655.55
DALYs (Dis	Central As	Both	50-69 yea	Sexually tra	Rate	1996	26.9611	38.9284	20.0745
DALYs (Dis	Southern S	Both	50-69 yea	HIV/AIDS	Number	1995	442387	710628	234463
DALYs (Dis	Southern S	Both	50-69 yea	HIV/AIDS	Rate	1995	8296.31	13326.8	4397
DALYs (Dis	High-incor	Both	50-69 yea	Sexually tra	Number	2004	10098.7	19823.3	5300.48
DALYs (Dis	High-incor	Both	50-69 yea	Sexually tra	Rate	2004	15.6648	30.7492	8.22193
DALYs (Dis	Southern L	Both	50-69 yea	HIV/AIDS	Number	1995	9779.78	10315.8	9441.71
DALYs (Dis	Southern L	Both	50-69 yea	HIV/AIDS	Rate	1995	123.016	129.758	118.763
DALYs (Dis	Western E	Both	50-69 yea	Sexually tra	Number	1995	7173.19	13556.6	4009.8
DALYs (Dis	Western E	Both	50-69 yea	Sexually tra	Rate	1995	8.55566	16.1694	4.78259
DALYs (Dis	Caribbean	Both	50-69 yea	HIV/AIDS	Number	2004	118960	140391	100954
DALYs (Dis	Caribbean	Both	50-69 yea	HIV/AIDS	Rate	2004	2024.8	2389.57	1718.32
DALYs (Dis	Western E	Both	50-69 yea	Sexually tra	Number	1996	7058.85	13474.5	3873.9
DALYs (Dis	Western E	Both	50-69 yea	Sexually tra	Rate	1996	8.37885	15.9942	4.59832
DALYs (Dis	Tropical La	Both	50-69 yea	Sexually tra	Number	1994	5190.96	9679.15	2927.27
DALYs (Dis	Tropical La	Both	50-69 yea	Sexually tra	Rate	1994	28.981	54.0384	16.3429
DALYs (Dis	Central Lat	Both	50-69 yea	HIV/AIDS	Number	2004	66089.2	69454.8	64058.4
DALYs (Dis	Central Lat	Both	50-69 yea	HIV/AIDS	Rate	2004	282.182	296.552	273.511
DALYs (Dis	Central Lat	Both	50-69 yea	Sexually tra	Number	2004	5505.09	10486.6	2974.26
DALYs (Dis	Central Lat	Both	50-69 yea	Sexually tra	Rate	2004	23.5052	44.775	12.6993
DALYs (Dis	Tropical La	Both	50-69 yea	Sexually tra	Number	2004	6967.71	13275.2	3825.56
DALYs (Dis	Tropical La	Both	50-69 yea	Sexually tra	Rate	2004	28.0462	53.4349	15.3985
DALYs (Dis	South Asia	Both	50-69 yea	HIV/AIDS	Number	2004	521211	587787	462394
DALYs (Dis	South Asia	Both	50-69 yea	HIV/AIDS	Rate	2004	345.876	390.056	306.845
DALYs (Dis	South Asia	Both	50-69 yea	Sexually tra	Number	2004	41238.2	55528.7	31154.5
DALYs (Dis	South Asia	Both	50-69 yea	Sexually tra	Rate	2004	27.3657	36.8489	20.6741
DALYs (Dis	North Afric	Both	50-69 yea	HIV/AIDS	Number	2004	27320.6	47114.2	15668.9
DALYs (Dis	North Afric	Both	50-69 yea	HIV/AIDS	Rate	2004	60.9083	105.036	34.9322
DALYs (Dis	High-incor	Both	50-69 yea	HIV/AIDS	Number	1994	216775	231015	207722
DALYs (Dis	High-incor	Both	50-69 yea	HIV/AIDS	Rate	1994	440.11	469.02	421.729
DALYs (Dis	Southern S	Both	50-69 yea	Sexually tra	Number	1994	3186.87	5053.46	2127.12
DALYs (Dis	Southern S	Both	50-69 yea	Sexually tra	Rate	1994	61.1485	96.9641	40.8145
DALYs (Dis	North Afric	Both	50-69 yea	Sexually tra	Number	1996	4144	7804	2196.74
DALYs (Dis	North Afric	Both	50-69 yea	Sexually tra	Rate	1996	11.4858	21.6301	6.08864
DALYs (Dis	Southern S	Both	50-69 yea	HIV/AIDS	Number	1994	314801	530082	157039
DALYs (Dis	Southern S	Both	50-69 yea	HIV/AIDS	Rate	1994	6040.29	10171	3013.2
DALYs (Dis	North Afric	Both	50-69 yea	Sexually tra	Number	1995	4074.39	7698.24	2164.6
DALYs (Dis	North Afric	Both	50-69 yea	Sexually tra	Rate	1995	11.5589	21.8396	6.14089
DALYs (Dis	Southern L	Both	50-69 yea	HIV/AIDS	Number	1996	11351.4	12028.2	10972.2
DALYs (Dis	Southern L	Both	50-69 yea	HIV/AIDS	Rate	1996	141.085	149.497	136.373
DALYs (Dis	Western E	Both	50-69 yea	HIV/AIDS	Number	1994	95535	99868.7	92284.2
DALYs (Dis	Western E	Both	50-69 yea	HIV/AIDS	Rate	1994	114.18	119.36	110.295
DALYs (Dis	Caribbean	Both	50-69 yea	HIV/AIDS	Number	1994	64323.8	90992	44126.3
DALYs (Dis	Caribbean	Both	50-69 yea	HIV/AIDS	Rate	1994	1419.99	2008.7	974.115
DALYs (Dis	Southern L	Both	50-69 yea	Sexually tra	Number	1996	1355.96	2432.81	810.863
DALYs (Dis	Southern L	Both	50-69 yea	Sexually tra	Rate	1996	16.8531	30.2371	10.0781
DALYs (Dis	Southern S	Both	50-69 yea	HIV/AIDS	Number	1996	601248	908788	341592
DALYs (Dis	Southern S	Both	50-69 yea	HIV/AIDS	Rate	1996	10982.2	16599.6	6239.42
DALYs (Dis	Andean La	Both	50-69 yea	HIV/AIDS	Number	1995	6543.14	17829.8	3975.44
DALYs (Dis	Andean La	Both	50-69 yea	HIV/AIDS	Rate	1995	160.077	436.204	97.2583
DALYs (Dis	East Asia	Both	50-69 yea	HIV/AIDS	Number	1996	45084.6	53703	40455.4
DALYs (Dis	East Asia	Both	50-69 yea	HIV/AIDS	Rate	1996	25.3645	30.2131	22.7601
DALYs (Dis	Andean La	Both	50-69 yea	Sexually tra	Number	1995	905.135	1688.77	501.964
DALYs (Dis	Andean La	Both	50-69 yea	Sexually tra	Rate	1995	22.144	41.3156	12.2804
DALYs (Dis	Eastern Eu	Both	50-69 yea	Sexually tra	Number	1997	14984.7	19810.4	8799.83
DALYs (Dis	Eastern Eu	Both	50-69 yea	Sexually tra	Rate	1997	33.0437	43.6853	19.4051
DALYs (Dis	Andean La	Both	50-69 yea	HIV/AIDS	Number	1997	8103.14	20834.9	5367.87
DALYs (Dis	Andean La	Both	50-69 yea	HIV/AIDS	Rate	1997	185.654	477.355	122.985
DALYs (Dis	Southern S	Both	50-69 yea	Sexually tra	Number	1996	3222.87	5000.81	2233.24
DALYs (Dis	Southern S	Both	50-69 yea	Sexually tra	Rate	1996	58.868	91.3433	40.7917
DALYs (Dis	Eastern Eu	Both	50-69 yea	HIV/AIDS	Number	1997	32250.1	34582.1	30866.4
DALYs (Dis	Eastern Eu	Both	50-69 yea	HIV/AIDS	Rate	1997	71.1169	76.2594	68.0658
DALYs (Dis	Eastern Eu	Both	50-69 yea	Sexually tra	Number	1996	15799.8	20850.4	9354.88
DALYs (Dis	Eastern Eu	Both	50-69 yea	Sexually tra	Rate	1996	34.4309	45.437	20.3861
DALYs (Dis	Australasia	Both	50-69 yea	HIV/AIDS	Number	1998	2139.28	2380.3	1975.18
DALYs (Dis	Australasia	Both	50-69 yea	HIV/AIDS	Rate	1998	53.0765	59.0563	49.0051
DALYs (Dis	Eastern Su	Both	50-69 yea	HIV/AIDS	Number	1997	2362595	2819868	1877972
DALYs (Dis	Eastern Su	Both	50-69 yea	HIV/AIDS	Rate	1997	15216.7	18161.9	12095.4
DALYs (Dis	Eastern Su	Both	50-69 yea	Sexually tra	Number	1997	18739.9	34456	8915.43
DALYs (Dis	Eastern Su	Both	50-69 yea	Sexually tra	Rate	1997	120.698	221.92	57.4214
DALYs (Dis	East Asia	Both	50-69 yea	HIV/AIDS	Number	2005	130008	157654	114847
DALYs (Dis	East Asia	Both	50-69 yea	HIV/AIDS	Rate	2005	53.9692	65.4456	47.6758
DALYs (Dis	Western S	Both	50-69 yea	Sexually tra	Number	1997	5143.43	8790	3119.74
DALYs (Dis	Western S	Both	50-69 yea	Sexually tra	Rate	1997	28.5333	48.7628	17.3069
DALYs (Dis	Western S	Both	50-69 yea	HIV/AIDS	Number	1998	908254	1120614	710784
DALYs (Dis	Western S	Both	50-69 yea	HIV/AIDS	Rate	1998	4942.61	6098.24	3868
DALYs (Dis	Eastern Eu	Both	50-69 yea	Sexually tra	Number	1995	16622.1	21940.8	10015.9
DALYs (Dis	Eastern Eu	Both	50-69 yea	Sexually tra	Rate	1995	35.2743	46.5612	21.2551
DALYs (Dis	High-incor	Both	50-69 yea	Sexually tra	Number	1997	8498.03	16285.5	4652.84
DALYs (Dis	High-incor	Both	50-69 yea	Sexually tra	Rate	1997	16.3629	31.3575	8.959
DALYs (Dis	Tropical La	Both	50-69 yea	HIV/AIDS	Number	1998	55639.9	60161.9	52848
DALYs (Dis	Tropical La	Both	50-69 yea	HIV/AIDS	Rate	1998	276.006	298.438	262.157
DALYs (Dis	Australasia	Both	50-69 yea	HIV/AIDS	Number	1996	3371.67	3655.6	3158.92
DALYs (Dis	Australasia	Both	50-69 yea	HIV/AIDS	Rate	1996	89.2082	96.7203	83.5791
DALYs (Dis	Tropical La	Both	50-69 yea	Sexually tra	Number	1998	5807.08	10899.9	3247.25
DALYs (Dis	Tropical La	Both	50-69 yea	Sexually tra	Rate	1998	28.8065	54.07	16.1082
DALYs (Dis	Southeast	Both	50-69 yea	HIV/AIDS	Number	1994	92192.1	114095	64207.9
DALYs (Dis	Southeast	Both	50-69 yea	HIV/AIDS	Rate	1994	179.073	221.618	124.717
DALYs (Dis	Central Lat	Both	50-69 yea	HIV/AIDS	Number	1998	45074.3	48207.8	43127.3
DALYs (Dis	Central Lat	Both	50-69 yea	HIV/AIDS	Rate	1998	239.31	255.947	228.973
DALYs (Dis	Central Lat	Both	50-69 yea	Sexually tra	Number	1998	4447.94	8439.99	2402.82
DALYs (Dis	Central Lat	Both	50-69 yea	Sexually tra	Rate	1998	23.6152	44.81	12.7572
DALYs (Dis	High-incor	Both	50-69 yea	HIV/AIDS	Number	2005	7132.75	8388.26	6185.51
DALYs (Dis	High-incor	Both	50-69 yea	HIV/AIDS	Rate	2005	15.8761	18.6706	13.7677
DALYs (Dis	Eastern Su	Both	50-69 yea	HIV/AIDS	Number	1995	1891428	2349768	1425004
DALYs (Dis	Eastern Su	Both	50-69 yea	HIV/AIDS	Rate	1995	12712.2	15792.6	9577.37
DALYs (Dis	Eastern Su	Both	50-69 yea	Sexually tra	Number	1995	18684.1	33406.7	8935.99
DALYs (Dis	Eastern Su	Both	50-69 yea	Sexually tra	Rate	1995	125.575	224.524	60.0582
DALYs (Dis	Central Su	Both	50-69 yea	Sexually tra	Number	1998	4156.68	7915.03	1630.39
DALYs (Dis	Central Su	Both	50-69 yea	Sexually tra	Rate	1998	82.9082	157.871	32.5195
DALYs (Dis	East Asia	Both	50-69 yea	Sexually tra	Number	1997	26530.8	47810.5	15716.9
DALYs (Dis	East Asia	Both	50-69 yea	Sexually tra	Rate	1997	14.5502	26.2206	8.61957
DALYs (Dis	Andean La	Both	50-69 yea	HIV/AIDS	Number	1996	7074.92	19362.6	4431.04
DALYs (Dis	Andean La	Both	50-69 yea	HIV/AIDS	Rate	1996	167.687	458.926	105.023
DALYs (Dis	Tropical La	Both	50-69 yea	HIV/AIDS	Number	2005	94513.1	97941.8	91348.4
DALYs (Dis	Tropical La	Both	50-69 yea	HIV/AIDS	Rate	2005	366.611	379.911	354.335
DALYs (Dis	Andean La	Both	50-69 yea	Sexually tra	Number	2005	1215.84	2316.94	668.591
DALYs (Dis	Andean La	Both	50-69 yea	Sexually tra	Rate	2005	21.3094	40.6077	11.718
DALYs (Dis	Southeast	Both	50-69 yea	HIV/AIDS	Number	1998	224879	244553	204518
DALYs (Dis	Southeast	Both	50-69 yea	HIV/AIDS	Rate	1998	401.23	436.33	364.901
DALYs (Dis	Central Su	Both	50-69 yea	Sexually tra	Number	2005	4025.77	7780.17	1718.57
DALYs (Dis	Central Su	Both	50-69 yea	Sexually tra	Rate	2005	67.273	130.011	28.7184
DALYs (Dis	Southeast	Both	50-69 yea	Sexually tra	Number	1998	10205	18355.6	6010.4
DALYs (Dis	Southeast	Both	50-69 yea	Sexually tra	Rate	1998	18.2077	32.75	10.7237
DALYs (Dis	Southern S	Both	50-69 yea	Sexually tra	Number	2005	3748.02	6035.9	2495.52
DALYs (Dis	Southern S	Both	50-69 yea	Sexually tra	Rate	2005	54.8535	88.3374	36.5229
DALYs (Dis	Southern L	Both	50-69 yea	HIV/AIDS	Number	1999	9450.32	10366	8974
DALYs (Dis	Southern L	Both	50-69 yea	HIV/AIDS	Rate	1999	112.718	123.64	107.036
DALYs (Dis	Western E	Both	50-69 yea	Sexually tra	Number	2005	7306.05	14666.8	3707.77
DALYs (Dis	Western E	Both	50-69 yea	Sexually tra	Rate	2005	7.80317	15.6647	3.96006
DALYs (Dis	High-incor	Both	50-69 yea	HIV/AIDS	Number	1996	180499	195331	171489
DALYs (Dis	High-incor	Both	50-69 yea	HIV/AIDS	Rate	1996	357.982	387.4	340.113
DALYs (Dis	Caribbean	Both	50-69 yea	HIV/AIDS	Number	1998	95733	122908	72881
DALYs (Dis	Caribbean	Both	50-69 yea	HIV/AIDS	Rate	1998	1903.82	2444.24	1449.37
DALYs (Dis	Western E	Both	50-69 yea	Sexually tra	Number	1994	7261.01	13620.4	4063.56
DALYs (Dis	Western E	Both	50-69 yea	Sexually tra	Rate	1994	8.67811	16.2786	4.85663
DALYs (Dis	Southern L	Both	50-69 yea	Sexually tra	Number	2005	1466	2733.36	834.093
DALYs (Dis	Southern L	Both	50-69 yea	Sexually tra	Rate	2005	15.6185	29.1207	8.88625
DALYs (Dis	Oceania	Both	50-69 yea	HIV/AIDS	Number	1999	3487.65	7844.23	1433.92
DALYs (Dis	Oceania	Both	50-69 yea	HIV/AIDS	Rate	1999	517.682	1164.34	212.841
DALYs (Dis	Andean La	Both	50-69 yea	Sexually tra	Number	1996	920.754	1725.68	509.309
DALYs (Dis	Andean La	Both	50-69 yea	Sexually tra	Rate	1996	21.8234	40.9015	12.0715
DALYs (Dis	Western E	Both	50-69 yea	Sexually tra	Number	1998	7022.92	13488.8	3730.28
DALYs (Dis	Western E	Both	50-69 yea	Sexually tra	Rate	1998	8.14173	15.6377	4.32454
DALYs (Dis	Australasia	Both	50-69 yea	HIV/AIDS	Number	1997	2296.24	2547.48	2115.73
DALYs (Dis	Australasia	Both	50-69 yea	HIV/AIDS	Rate	1997	58.7102	65.1339	54.0949
DALYs (Dis	Caribbean	Both	50-69 yea	Sexually tra	Number	1998	1415.74	2477.42	853.236
DALYs (Dis	Caribbean	Both	50-69 yea	Sexually tra	Rate	1998	28.1545	49.2677	16.9681
DALYs (Dis	Central As	Both	50-69 yea	HIV/AIDS	Number	1999	2069.6	2208.17	1959.66
DALYs (Dis	Central As	Both	50-69 yea	HIV/AIDS	Rate	1999	26.1724	27.9248	24.7821
DALYs (Dis	Southern L	Both	50-69 yea	Sexually tra	Number	1999	1388.42	2505.21	813.435
DALYs (Dis	Southern L	Both	50-69 yea	Sexually tra	Rate	1999	16.5602	29.8806	9.70215
DALYs (Dis	Caribbean	Both	50-69 yea	HIV/AIDS	Number	1997	90151.2	118152	67145.3
DALYs (Dis	Caribbean	Both	50-69 yea	HIV/AIDS	Rate	1997	1842.25	2414.44	1372.12
DALYs (Dis	High-incor	Both	50-69 yea	HIV/AIDS	Number	1999	4706.74	5365.1	4280.51
DALYs (Dis	High-incor	Both	50-69 yea	HIV/AIDS	Rate	1999	11.3025	12.8835	10.279
DALYs (Dis	South Asia	Both	50-69 yea	HIV/AIDS	Number	1998	138887	171061	112997
DALYs (Dis	South Asia	Both	50-69 yea	HIV/AIDS	Rate	1998	107.694	132.642	87.6188
DALYs (Dis	South Asia	Both	50-69 yea	Sexually tra	Number	1998	43485.7	56662.5	32703.4
DALYs (Dis	South Asia	Both	50-69 yea	Sexually tra	Rate	1998	33.7191	43.9365	25.3584
DALYs (Dis	High-incor	Both	50-69 yea	HIV/AIDS	Number	2005	176674	196535	162343
DALYs (Dis	High-incor	Both	50-69 yea	HIV/AIDS	Rate	2005	264.482	294.215	243.029
DALYs (Dis	Oceania	Both	50-69 yea	Sexually tra	Number	1998	171.02	314.223	87.6302
DALYs (Dis	Oceania	Both	50-69 yea	Sexually tra	Rate	1998	25.9636	47.7042	13.3037
DALYs (Dis	Central As	Both	50-69 yea	HIV/AIDS	Number	1998	1906.36	2038.76	1809.82
DALYs (Dis	Central As	Both	50-69 yea	HIV/AIDS	Rate	1998	23.7986	25.4514	22.5934
DALYs (Dis	Central Su	Both	50-69 yea	Sexually tra	Number	1996	4214.81	7903.25	1592.59
DALYs (Dis	Central Su	Both	50-69 yea	Sexually tra	Rate	1996	86.6182	162.419	32.7291
DALYs (Dis	Eastern Su	Both	50-69 yea	HIV/AIDS	Number	1996	2138377	2601588	1661139
DALYs (Dis	Eastern Su	Both	50-69 yea	HIV/AIDS	Rate	1996	14054.4	17098.8	10917.8
DALYs (Dis	Eastern Su	Both	50-69 yea	Sexually tra	Number	1996	18736.4	34005.5	8869.02
DALYs (Dis	Eastern Su	Both	50-69 yea	Sexually tra	Rate	1996	123.144	223.499	58.2912
DALYs (Dis	Australasia	Both	50-69 yea	Sexually tra	Number	1997	355.213	703.097	176.504
DALYs (Dis	Australasia	Both	50-69 yea	Sexually tra	Rate	1997	9.08208	17.9767	4.51285
DALYs (Dis	North Afric	Both	50-69 yea	Sexually tra	Number	1998	4295.03	8191.71	2284.73
DALYs (Dis	North Afric	Both	50-69 yea	Sexually tra	Rate	1998	11.3359	21.6205	6.03013
DALYs (Dis	Central Su	Both	50-69 yea	HIV/AIDS	Number	1997	299471	372029	241113
DALYs (Dis	Central Su	Both	50-69 yea	HIV/AIDS	Rate	1997	6065.67	7535.32	4883.67
DALYs (Dis	Eastern Eu	Both	50-69 yea	HIV/AIDS	Number	1999	36218.9	38941.3	34554.9
DALYs (Dis	Eastern Eu	Both	50-69 yea	HIV/AIDS	Rate	1999	81.088	87.1831	77.3624
DALYs (Dis	Central As	Both	50-69 yea	Sexually tra	Number	1998	2085	2985.6	1555.05
DALYs (Dis	Central As	Both	50-69 yea	Sexually tra	Rate	1998	26.0287	37.2715	19.4129
DALYs (Dis	Andean La	Both	50-69 yea	Sexually tra	Number	1999	1020.58	1897.76	565.5
DALYs (Dis	Andean La	Both	50-69 yea	Sexually tra	Rate	1999	21.8566	40.6423	12.1107
DALYs (Dis	Tropical La	Both	50-69 yea	Sexually tra	Number	1997	5642.79	10550.4	3156.38
DALYs (Dis	Tropical La	Both	50-69 yea	Sexually tra	Rate	1997	28.9361	54.1022	16.1858
DALYs (Dis	Central Lat	Both	50-69 yea	HIV/AIDS	Number	1997	43093.8	45991.6	41407.1
DALYs (Dis	Central Lat	Both	50-69 yea	HIV/AIDS	Rate	1997	237.274	253.229	227.987
DALYs (Dis	Central Lat	Both	50-69 yea	Sexually tra	Number	1997	4318.8	8133.39	2345.31
DALYs (Dis	Central Lat	Both	50-69 yea	Sexually tra	Rate	1997	23.7792	44.7823	12.9132
DALYs (Dis	North Afric	Both	50-69 yea	HIV/AIDS	Number	1998	14381.2	27044.1	7575.1
DALYs (Dis	North Afric	Both	50-69 yea	HIV/AIDS	Rate	1998	37.9566	71.3778	19.9931
DALYs (Dis	High-incor	Both	50-69 yea	Sexually tra	Number	1999	4290.45	8052.62	2350.88
DALYs (Dis	High-incor	Both	50-69 yea	Sexually tra	Rate	1999	10.3029	19.3372	5.64529
DALYs (Dis	Western S	Both	50-69 yea	HIV/AIDS	Number	1997	798750	1016786	616401
DALYs (Dis	Western S	Both	50-69 yea	HIV/AIDS	Rate	1997	4431.09	5640.65	3419.5
DALYs (Dis	Oceania	Both	50-69 yea	HIV/AIDS	Number	1998	2324.54	5301.11	955.067
DALYs (Dis	Oceania	Both	50-69 yea	HIV/AIDS	Rate	1998	352.903	804.794	144.995
DALYs (Dis	Southeast	Both	50-69 yea	HIV/AIDS	Number	1997	192567	210463	168258
DALYs (Dis	Southeast	Both	50-69 yea	HIV/AIDS	Rate	1997	351.122	383.753	306.797
DALYs (Dis	Tropical La	Both	50-69 yea	HIV/AIDS	Number	1996	59261.4	63086.5	56846.8
DALYs (Dis	Tropical La	Both	50-69 yea	HIV/AIDS	Rate	1996	313.543	333.781	300.768
DALYs (Dis	Andean La	Both	50-69 yea	HIV/AIDS	Number	1999	9589.98	22529	6743.12
DALYs (Dis	Andean La	Both	50-69 yea	HIV/AIDS	Rate	1999	205.378	482.478	144.41
DALYs (Dis	Central Eu	Both	50-69 yea	Sexually tra	Number	1997	2451.24	4508.6	1366.23
DALYs (Dis	Central Eu	Both	50-69 yea	Sexually tra	Rate	1997	9.97604	18.3491	5.56028
DALYs (Dis	Caribbean	Both	50-69 yea	Sexually tra	Number	1997	1389.9	2397.05	830.857
DALYs (Dis	Caribbean	Both	50-69 yea	Sexually tra	Rate	1997	28.4028	48.984	16.9787
DALYs (Dis	Southern L	Both	50-69 yea	HIV/AIDS	Number	2006	14964.6	16049.1	14225.6
DALYs (Dis	Southern L	Both	50-69 yea	HIV/AIDS	Rate	2006	155.73	167.015	148.039
DALYs (Dis	Western S	Both	50-69 yea	HIV/AIDS	Number	1996	685361	895660	511784
DALYs (Dis	Western S	Both	50-69 yea	HIV/AIDS	Rate	1996	3875.95	5065.27	2894.31
DALYs (Dis	Australasia	Both	50-69 yea	Sexually tra	Number	1996	343.258	682.715	170.36
DALYs (Dis	Australasia	Both	50-69 yea	Sexually tra	Rate	1996	9.08196	18.0634	4.50741
DALYs (Dis	Southern L	Both	50-69 yea	HIV/AIDS	Number	1997	9229.67	10017.2	8843.41
DALYs (Dis	Southern L	Both	50-69 yea	HIV/AIDS	Rate	1997	113.284	122.95	108.543
DALYs (Dis	Central Eu	Both	50-69 yea	Sexually tra	Number	1998	2430.79	4507.08	1344.81
DALYs (Dis	Central Eu	Both	50-69 yea	Sexually tra	Rate	1998	9.82887	18.2243	5.43773
DALYs (Dis	Australasia	Both	50-69 yea	Sexually tra	Number	2006	400.032	776.144	198.979
DALYs (Dis	Australasia	Both	50-69 yea	Sexually tra	Rate	2006	7.77559	15.0862	3.86765
DALYs (Dis	Andean La	Both	50-69 yea	HIV/AIDS	Number	2006	17097	25540.9	13542.4
DALYs (Dis	Andean La	Both	50-69 yea	HIV/AIDS	Rate	2006	289.646	432.696	229.427
DALYs (Dis	Central Eu	Both	50-69 yea	HIV/AIDS	Number	1998	5589.8	5986.03	5220.71
DALYs (Dis	Central Eu	Both	50-69 yea	HIV/AIDS	Rate	1998	22.6023	24.2044	21.1099
DALYs (Dis	Western S	Both	50-69 yea	Sexually tra	Number	1996	5111.36	8728.03	3124.61
DALYs (Dis	Western S	Both	50-69 yea	Sexually tra	Rate	1996	28.9065	49.36	17.6708
DALYs (Dis	High-incor	Both	50-69 yea	Sexually tra	Number	1998	4196.88	7894.73	2293.45
DALYs (Dis	High-incor	Both	50-69 yea	Sexually tra	Rate	1998	10.3543	19.4775	5.65829
DALYs (Dis	East Asia	Both	50-69 yea	Sexually tra	Number	1999	26726.4	48700.8	15486.2
DALYs (Dis	East Asia	Both	50-69 yea	Sexually tra	Rate	1999	13.8046	25.1548	7.99888
DALYs (Dis	Southern S	Both	50-69 yea	Sexually tra	Number	1998	3647.03	5480.05	2582.65
DALYs (Dis	Southern S	Both	50-69 yea	Sexually tra	Rate	1998	63.6069	95.5761	45.0433
DALYs (Dis	Western E	Both	50-69 yea	HIV/AIDS	Number	2006	53902.6	59013.5	50189.3
DALYs (Dis	Western E	Both	50-69 yea	HIV/AIDS	Rate	2006	56.9011	62.2964	52.9813
DALYs (Dis	High-incor	Both	50-69 yea	HIV/AIDS	Number	1998	4470.62	5088.1	4073
DALYs (Dis	High-incor	Both	50-69 yea	HIV/AIDS	Rate	1998	11.0297	12.5531	10.0487
DALYs (Dis	Oceania	Both	50-69 yea	HIV/AIDS	Number	1997	1470.71	3310.36	604.162
DALYs (Dis	Oceania	Both	50-69 yea	HIV/AIDS	Rate	1997	229.457	516.477	94.2603
DALYs (Dis	Central Su	Both	50-69 yea	HIV/AIDS	Number	2006	365981	437971	302386
DALYs (Dis	Central Su	Both	50-69 yea	HIV/AIDS	Rate	2006	5913.87	7077.17	4886.26
DALYs (Dis	Southern S	Both	50-69 yea	HIV/AIDS	Number	2006	2484669	2757262	2248317
DALYs (Dis	Southern S	Both	50-69 yea	HIV/AIDS	Rate	2006	35406.9	39291.4	32038.9
DALYs (Dis	Southern L	Both	50-69 yea	Sexually tra	Number	1998	1380.63	2485.89	823.443
DALYs (Dis	Southern L	Both	50-69 yea	Sexually tra	Rate	1998	16.7139	30.0941	9.96858
DALYs (Dis	Eastern Eu	Both	50-69 yea	HIV/AIDS	Number	1998	28137.8	30686.1	26638
DALYs (Dis	Eastern Eu	Both	50-69 yea	HIV/AIDS	Rate	1998	62.663	68.3381	59.3229
DALYs (Dis	Eastern Su	Both	50-69 yea	HIV/AIDS	Number	1998	2553619	3000584	2088715
DALYs (Dis	Eastern Su	Both	50-69 yea	HIV/AIDS	Rate	1998	16127.8	18950.7	13191.6
DALYs (Dis	Eastern Su	Both	50-69 yea	Sexually tra	Number	1998	18640.7	33583.9	8902.29
DALYs (Dis	Eastern Su	Both	50-69 yea	Sexually tra	Rate	1998	117.728	212.104	56.2239
DALYs (Dis	Western S	Both	50-69 yea	Sexually tra	Number	2006	5385.1	10080.5	2956.02
DALYs (Dis	Western S	Both	50-69 yea	Sexually tra	Rate	2006	23.924	44.7837	13.1325
DALYs (Dis	Eastern Eu	Both	50-69 yea	Sexually tra	Number	1998	14014.4	18701.5	8354.7
DALYs (Dis	Eastern Eu	Both	50-69 yea	Sexually tra	Rate	1998	31.2101	41.6482	18.6059
DALYs (Dis	South Asia	Both	50-69 yea	HIV/AIDS	Number	1997	99303.9	126771	78014.6
DALYs (Dis	South Asia	Both	50-69 yea	HIV/AIDS	Rate	1997	78.6126	100.357	61.7592
DALYs (Dis	South Asia	Both	50-69 yea	Sexually tra	Number	1997	43728.3	56634.8	33046.8
DALYs (Dis	South Asia	Both	50-69 yea	Sexually tra	Rate	1997	34.6169	44.8342	26.161
DALYs (Dis	Southeast	Both	50-69 yea	Sexually tra	Number	1997	10069.8	18147.8	5893.31
DALYs (Dis	Southeast	Both	50-69 yea	Sexually tra	Rate	1997	18.3611	33.0903	10.7457
DALYs (Dis	Southeast	Both	50-69 yea	Sexually tra	Number	2007	13033.2	23713.3	7447.01
DALYs (Dis	Southeast	Both	50-69 yea	Sexually tra	Rate	2007	17.6251	32.068	10.0708
DALYs (Dis	High-incor	Both	50-69 yea	Sexually tra	Number	1999	8865.64	17093.8	4781.03
DALYs (Dis	High-incor	Both	50-69 yea	Sexually tra	Rate	1999	16.1213	31.0834	8.69386
DALYs (Dis	Oceania	Both	50-69 yea	Sexually tra	Number	2007	226.308	420.483	119.177
DALYs (Dis	Oceania	Both	50-69 yea	Sexually tra	Rate	2007	25.9075	48.1364	13.6432
DALYs (Dis	Eastern Eu	Both	50-69 yea	Sexually tra	Number	2007	8333.85	14222	5485.7
DALYs (Dis	Eastern Eu	Both	50-69 yea	Sexually tra	Rate	2007	17.453	29.7843	11.4883
DALYs (Dis	Central As	Both	50-69 yea	Sexually tra	Number	2007	1842.36	2962.62	1242.67
DALYs (Dis	Central As	Both	50-69 yea	Sexually tra	Rate	2007	20.0287	32.2073	13.5093
DALYs (Dis	Australasia	Both	50-69 yea	HIV/AIDS	Number	2007	1981.02	2157.34	1826.92
DALYs (Dis	Australasia	Both	50-69 yea	HIV/AIDS	Rate	2007	37.4481	40.7811	34.535
DALYs (Dis	North Afric	Both	50-69 yea	Sexually tra	Number	1997	4214.51	8010.81	2236.02
DALYs (Dis	North Afric	Both	50-69 yea	Sexually tra	Rate	1997	11.41	21.6879	6.05364
DALYs (Dis	Central Eu	Both	50-69 yea	HIV/AIDS	Number	1997	5424.91	5800.25	5053.75
DALYs (Dis	Central Eu	Both	50-69 yea	HIV/AIDS	Rate	1997	22.0783	23.6059	20.5678
DALYs (Dis	Central Eu	Both	50-69 yea	Sexually tra	Number	2007	2720.09	5101.64	1457.74
DALYs (Dis	Central Eu	Both	50-69 yea	Sexually tra	Rate	2007	9.70414	18.2005	5.20059
DALYs (Dis	Central Su	Both	50-69 yea	HIV/AIDS	Number	1996	279852	350594	218714
DALYs (Dis	Central Su	Both	50-69 yea	HIV/AIDS	Rate	1996	5751.22	7205.02	4494.77
DALYs (Dis	East Asia	Both	50-69 yea	HIV/AIDS	Number	2000	69996.7	90405	59337.7
DALYs (Dis	East Asia	Both	50-69 yea	HIV/AIDS	Rate	2000	35.2668	45.5492	29.8964

DALYs (Dis	High-incor	Both	50-69 yea	HIV/AIDS	Rate	1998	215.559	241.182	198.803
DALYs (Dis	Oceania	Both	50-69 yea	Sexually tr	Number	1997	166.245	309.153	86.6894
DALYs (Dis	Oceania	Both	50-69 yea	Sexually tr	Rate	1997	25.9372	48.2335	13.5251
DALYs (Dis	Central Lat	Both	50-69 yea	HIV/AIDS	Number	1999	47607.8	50944.4	45623.5
DALYs (Dis	Central Lat	Both	50-69 yea	HIV/AIDS	Rate	1999	243.794	260.88	233.632
DALYs (Dis	Central Lat	Both	50-69 yea	Sexually tr	Number	1999	4595.33	8696.45	2482.1
DALYs (Dis	Central Lat	Both	50-69 yea	Sexually tr	Rate	1999	23.5321	44.5334	12.7105
DALYs (Dis	Tropical La	Both	50-69 yea	HIV/AIDS	Number	2000	64670.7	67869.6	62349.9
DALYs (Dis	Tropical La	Both	50-69 yea	HIV/AIDS	Rate	2000	300.268	315.121	289.493
DALYs (Dis	Southeast	Both	50-69 yea	HIV/AIDS	Number	1999	254959	277635	230002
DALYs (Dis	Southeast	Both	50-69 yea	HIV/AIDS	Rate	1999	444.672	484.222	401.145
DALYs (Dis	Western Eu	Both	50-69 yea	HIV/AIDS	Number	1999	46910.4	51905.4	43836.8
DALYs (Dis	Western Eu	Both	50-69 yea	HIV/AIDS	Rate	1999	53.6784	59.3941	50.1614
DALYs (Dis	Central As	Both	50-69 yea	HIV/AIDS	Number	1997	1883	2011.59	1783.65
DALYs (Dis	Central As	Both	50-69 yea	HIV/AIDS	Rate	1997	23.2019	24.7864	21.9777
DALYs (Dis	East Asia	Both	50-69 yea	Sexually tr	Number	1998	26270.8	47934.8	15256.7
DALYs (Dis	East Asia	Both	50-69 yea	Sexually tr	Rate	1998	14.0567	25.6484	8.16335
DALYs (Dis	Tropical La	Both	50-69 yea	Sexually tr	Number	1999	6022.64	11287.1	3351.4
DALYs (Dis	Tropical La	Both	50-69 yea	Sexually tr	Rate	1999	28.8657	54.0973	16.0628
DALYs (Dis	Tropical La	Both	50-69 yea	HIV/AIDS	Number	1999	60197	64820.3	57284.1
DALYs (Dis	Tropical La	Both	50-69 yea	HIV/AIDS	Rate	1999	288.516	310.675	274.555
DALYs (Dis	Oceania	Both	50-69 yea	HIV/AIDS	Number	2008	16066.4	39129.5	4896.8
DALYs (Dis	Oceania	Both	50-69 yea	HIV/AIDS	Rate	2008	1772.34	4316.51	540.183
DALYs (Dis	Southeast	Both	50-69 yea	HIV/AIDS	Number	2008	316020	404487	263621
DALYs (Dis	Southeast	Both	50-69 yea	HIV/AIDS	Rate	2008	411.062	526.136	342.904
DALYs (Dis	Western Su	Both	50-69 yea	Sexually tr	Number	2000	5206.33	9183.23	3051.11
DALYs (Dis	Western Su	Both	50-69 yea	Sexually tr	Rate	2000	26.9861	47.5997	15.8149
DALYs (Dis	Western Su	Both	50-69 yea	HIV/AIDS	Number	2000	1118300	1327094	922841
DALYs (Dis	Western Su	Both	50-69 yea	HIV/AIDS	Rate	2000	5796.51	6878.75	4783.38
DALYs (Dis	Central As	Both	50-69 yea	HIV/AIDS	Number	2008	3524.26	4088.9	3146.27
DALYs (Dis	Central As	Both	50-69 yea	HIV/AIDS	Rate	2008	37.085	43.0265	33.1074
DALYs (Dis	East Asia	Both	50-69 yea	Sexually tr	Number	2008	33668.5	59719	19656.2
DALYs (Dis	East Asia	Both	50-69 yea	Sexually tr	Rate	2008	12.7787	22.666	7.46039
DALYs (Dis	Central Su	Both	50-69 yea	HIV/AIDS	Number	1999	326886	393090	270953
DALYs (Dis	Central Su	Both	50-69 yea	HIV/AIDS	Rate	1999	6418.73	7718.72	5320.43
DALYs (Dis	Central Eu	Both	50-69 yea	HIV/AIDS	Number	2008	5757.92	6732.21	5342.11
DALYs (Dis	Central Eu	Both	50-69 yea	HIV/AIDS	Rate	2008	20.2817	23.7136	18.8171
DALYs (Dis	Australasia	Both	50-69 yea	Sexually tr	Number	2000	386.627	753.994	188.833
DALYs (Dis	Australasia	Both	50-69 yea	Sexually tr	Rate	2000	9.03723	17.6243	4.41387
DALYs (Dis	Caribbean	Both	50-69 yea	HIV/AIDS	Number	1999	103233	129556	81762.7
DALYs (Dis	Caribbean	Both	50-69 yea	HIV/AIDS	Rate	1999	1997.52	2506.87	1582.09
DALYs (Dis	High-incor	Both	50-69 yea	Sexually tr	Number	2008	4437.16	8593.43	2311.52
DALYs (Dis	High-incor	Both	50-69 yea	Sexually tr	Rate	2008	9.68123	18.7496	5.04339
DALYs (Dis	Eastern Eu	Both	50-69 yea	HIV/AIDS	Number	2008	61918.3	66883.7	59065.8
DALYs (Dis	Eastern Eu	Both	50-69 yea	HIV/AIDS	Rate	2008	128.921	139.259	122.982
DALYs (Dis	Southeast	Both	50-69 yea	Sexually tr	Number	2001	10822.9	19412.5	6256.86
DALYs (Dis	Southeast	Both	50-69 yea	Sexually tr	Rate	2001	17.9278	32.1564	10.3643
DALYs (Dis	Caribbean	Both	50-69 yea	HIV/AIDS	Number	2008	97136.4	118579	79365.9
DALYs (Dis	Caribbean	Both	50-69 yea	HIV/AIDS	Rate	2008	1508.62	1841.65	1232.62
DALYs (Dis	Australasia	Both	50-69 yea	HIV/AIDS	Number	2000	1696.97	1842.67	1593.65
DALYs (Dis	Australasia	Both	50-69 yea	HIV/AIDS	Rate	2000	39.6659	43.0715	37.2509
DALYs (Dis	Southern S	Both	50-69 yea	HIV/AIDS	Number	2001	1739304	2145086	1316923
DALYs (Dis	Southern S	Both	50-69 yea	HIV/AIDS	Rate	2001	28297.7	34899.7	21425.8
DALYs (Dis	Western Eu	Both	50-69 yea	Sexually tr	Number	1999	7041.97	13675.4	3708.38
DALYs (Dis	Western Eu	Both	50-69 yea	Sexually tr	Rate	1999	8.05796	15.6484	4.24341
DALYs (Dis	High-incor	Both	50-69 yea	Sexually tr	Number	2008	10863.3	21445.9	5589.77
DALYs (Dis	High-incor	Both	50-69 yea	Sexually tr	Rate	2008	14.6758	28.9726	7.55156
DALYs (Dis	Central Su	Both	50-69 yea	Sexually tr	Number	1999	4137.65	7965.75	1592.64
DALYs (Dis	Central Su	Both	50-69 yea	Sexually tr	Rate	1999	81.2468	156.415	31.273
DALYs (Dis	Southeast	Both	50-69 yea	HIV/AIDS	Number	2001	307353	341222	269767
DALYs (Dis	Southeast	Both	50-69 yea	HIV/AIDS	Rate	2001	509.122	565.226	446.861
DALYs (Dis	Southeast	Both	50-69 yea	Sexually tr	Number	2000	10563.2	18923	6154.27
DALYs (Dis	Southeast	Both	50-69 yea	Sexually tr	Rate	2000	18.0351	32.3083	10.5075
DALYs (Dis	North Afric	Both	50-69 yea	HIV/AIDS	Number	2008	34502.3	63795.9	19624
DALYs (Dis	North Afric	Both	50-69 yea	HIV/AIDS	Rate	2008	66.7907	123.498	37.9888
DALYs (Dis	Western Eu	Both	50-69 yea	HIV/AIDS	Number	2001	46437.6	50932.9	43517.3
DALYs (Dis	Western Eu	Both	50-69 yea	HIV/AIDS	Rate	2001	51.9281	56.9549	48.6624
DALYs (Dis	Central Lat	Both	50-69 yea	HIV/AIDS	Number	2008	81200.9	83692.1	78915.2
DALYs (Dis	Central Lat	Both	50-69 yea	HIV/AIDS	Rate	2008	297.645	306.777	289.267
DALYs (Dis	Central Lat	Both	50-69 yea	Sexually tr	Number	2008	6452.07	12308.9	3492.67
DALYs (Dis	Central Lat	Both	50-69 yea	Sexually tr	Rate	2008	23.6503	45.1186	12.8025
DALYs (Dis	Central Su	Both	50-69 yea	HIV/AIDS	Number	2000	334971	398246	279678
DALYs (Dis	Central Su	Both	50-69 yea	HIV/AIDS	Rate	2000	6520.86	7752.63	5444.47
DALYs (Dis	Tropical La	Both	50-69 yea	Sexually tr	Number	2008	7995.97	15431.4	4267.64
DALYs (Dis	Tropical La	Both	50-69 yea	Sexually tr	Rate	2008	27.7815	53.6154	14.8276
DALYs (Dis	South Asia	Both	50-69 yea	HIV/AIDS	Number	2008	601385	716732	493903
DALYs (Dis	South Asia	Both	50-69 yea	HIV/AIDS	Rate	2008	351.681	419.135	288.828
DALYs (Dis	South Asia	Both	50-69 yea	Sexually tr	Number	2008	44181.8	58935.1	33042.8
DALYs (Dis	South Asia	Both	50-69 yea	Sexually tr	Rate	2008	25.8369	34.4644	19.323
DALYs (Dis	Caribbean	Both	50-69 yea	Sexually tr	Number	2000	1499.07	2584.77	921.36
DALYs (Dis	Caribbean	Both	50-69 yea	Sexually tr	Rate	2000	28.2632	48.7329	17.3712
DALYs (Dis	Central As	Both	50-69 yea	Sexually tr	Number	2001	1803.16	2682.55	1319.35
DALYs (Dis	Central As	Both	50-69 yea	Sexually tr	Rate	2001	22.6762	33.7353	16.5919
DALYs (Dis	Western Su	Both	50-69 yea	Sexually tr	Number	1999	5186.78	9088.01	3092.5
DALYs (Dis	Western Su	Both	50-69 yea	Sexually tr	Rate	1999	27.6946	48.5251	16.5123
DALYs (Dis	South Asia	Both	50-69 yea	HIV/AIDS	Number	1999	185854	222911	155027
DALYs (Dis	South Asia	Both	50-69 yea	HIV/AIDS	Rate	1999	141.244	169.406	117.816
DALYs (Dis	South Asia	Both	50-69 yea	Sexually tr	Number	1999	42487	55366.8	32310.1
DALYs (Dis	South Asia	Both	50-69 yea	Sexually tr	Rate	1999	32.2889	42.0771	24.5547
DALYs (Dis	Southern S	Both	50-69 yea	Sexually tr	Number	1999	3691.26	5602.99	2618.84
DALYs (Dis	Southern S	Both	50-69 yea	Sexually tr	Rate	1999	62.9893	95.6119	44.6891
DALYs (Dis	Oceania	Both	50-69 yea	HIV/AIDS	Number	2001	6955.6	14661.4	2833.85
DALYs (Dis	Oceania	Both	50-69 yea	HIV/AIDS	Rate	2001	981.586	2069.05	399.917
DALYs (Dis	North Afric	Both	50-69 yea	Sexually tr	Number	2001	4603.93	8768.85	2432.67
DALYs (Dis	North Afric	Both	50-69 yea	Sexually tr	Rate	2001	11.2854	21.4946	5.96307
DALYs (Dis	Central Su	Both	50-69 yea	Sexually tr	Number	2000	4073.17	7741.16	1616.37
DALYs (Dis	Central Su	Both	50-69 yea	Sexually tr	Rate	2000	79.2921	150.697	31.4657
DALYs (Dis	Eastern Eu	Both	50-69 yea	HIV/AIDS	Number	2002	45119.9	48569.2	42986.1
DALYs (Dis	Eastern Eu	Both	50-69 yea	HIV/AIDS	Rate	2002	99.4309	107.032	94.7288
DALYs (Dis	Eastern Eu	Both	50-69 yea	Sexually tr	Number	2002	11318.7	16294.7	8194.43
DALYs (Dis	Eastern Eu	Both	50-69 yea	Sexually tr	Rate	2002	24.9432	35.9087	18.0581
DALYs (Dis	Oceania	Both	50-69 yea	Sexually tr	Number	2001	182.972	335.129	95.4123
DALYs (Dis	Oceania	Both	50-69 yea	Sexually tr	Rate	2001	25.8213	47.294	13.4648
DALYs (Dis	Central Eu	Both	50-69 yea	Sexually tr	Number	2001	2463.7	4608.65	1347.84
DALYs (Dis	Central Eu	Both	50-69 yea	Sexually tr	Rate	2001	9.64888	18.0494	5.27873
DALYs (Dis	East Asia	Both	50-69 yea	HIV/AIDS	Number	2009	138889	160524	125845
DALYs (Dis	East Asia	Both	50-69 yea	HIV/AIDS	Rate	2009	51.588	59.6242	46.7433
DALYs (Dis	Southern L	Both	50-69 yea	Sexually tr	Number	2000	1401.17	2527.94	816.751
DALYs (Dis	Southern L	Both	50-69 yea	Sexually tr	Rate	2000	16.4472	29.6735	9.58721
DALYs (Dis	Southern L	Both	50-69 yea	HIV/AIDS	Number	2001	12472.5	13284.1	11878.2
DALYs (Dis	Southern L	Both	50-69 yea	HIV/AIDS	Rate	2001	143.988	153.358	137.127
DALYs (Dis	South Asia	Both	50-69 yea	HIV/AIDS	Number	2001	309909	359060	270463
DALYs (Dis	South Asia	Both	50-69 yea	HIV/AIDS	Rate	2001	224.538	260.149	195.958
DALYs (Dis	South Asia	Both	50-69 yea	Sexually tr	Number	2001	42345.1	55833.7	32132.2
DALYs (Dis	South Asia	Both	50-69 yea	Sexually tr	Rate	2001	30.6802	40.453	23.2806
DALYs (Dis	North Afric	Both	50-69 yea	Sexually tr	Number	2000	4461.49	8457.32	2345.59
DALYs (Dis	North Afric	Both	50-69 yea	Sexually tr	Rate	2000	11.2565	21.338	5.91799
DALYs (Dis	Western Eu	Both	50-69 yea	Sexually tr	Number	2000	7003.83	13778.3	3630.23
DALYs (Dis	Western Eu	Both	50-69 yea	Sexually tr	Rate	2000	7.91681	15.5744	4.10345
DALYs (Dis	Central Eu	Both	50-69 yea	HIV/AIDS	Number	2001	5262.54	5543.14	4962.26
DALYs (Dis	Central Eu	Both	50-69 yea	HIV/AIDS	Rate	2001	20.6103	21.7093	19.4343
DALYs (Dis	Central Eu	Both	50-69 yea	Sexually tr	Number	2000	2433.72	4541.8	1334.66
DALYs (Dis	Central Eu	Both	50-69 yea	Sexually tr	Rate	2000	9.65061	18.0099	5.29244
DALYs (Dis	High-incor	Both	50-69 yea	Sexually tr	Number	2001	4403.46	8253.89	2388.12
DALYs (Dis	High-incor	Both	50-69 yea	Sexually tr	Rate	2001	10.2053	19.1289	5.53462
DALYs (Dis	High-incor	Both	50-69 yea	HIV/AIDS	Number	2000	4622.39	5310.94	4148.3
DALYs (Dis	High-incor	Both	50-69 yea	HIV/AIDS	Rate	2000	10.8719	12.4913	9.75681
DALYs (Dis	Southern L	Both	50-69 yea	HIV/AIDS	Number	2000	10882.8	11769	10323.8
DALYs (Dis	Southern L	Both	50-69 yea	HIV/AIDS	Rate	2000	127.745	138.147	121.183
DALYs (Dis	High-incor	Both	50-69 yea	HIV/AIDS	Number	2009	8429.85	10222.9	7136.24
DALYs (Dis	High-incor	Both	50-69 yea	HIV/AIDS	Rate	2009	18.173	22.0384	15.3842
DALYs (Dis	North Afric	Both	50-69 yea	HIV/AIDS	Number	2001	20386.1	37177	11298.5
DALYs (Dis	North Afric	Both	50-69 yea	HIV/AIDS	Rate	2001	49.9714	91.1301	27.6955
DALYs (Dis	Andean La	Both	50-69 yea	Sexually tr	Number	2002	1114.75	2083.07	610.989
DALYs (Dis	Andean La	Both	50-69 yea	Sexually tr	Rate	2002	21.6288	40.4166	11.8546
DALYs (Dis	Eastern Eu	Both	50-69 yea	Sexually tr	Number	2001	12206.2	17098.1	8327.56
DALYs (Dis	Eastern Eu	Both	50-69 yea	Sexually tr	Rate	2001	27.1449	38.0236	18.5193
DALYs (Dis	High-incor	Both	50-69 yea	HIV/AIDS	Number	2009	186430	215579	165647
DALYs (Dis	High-incor	Both	50-69 yea	HIV/AIDS	Rate	2009	244.031	282.186	216.827
DALYs (Dis	Western Eu	Both	50-69 yea	Sexually tr	Number	2009	7602.13	15336.3	3801.8
DALYs (Dis	Western Eu	Both	50-69 yea	Sexually tr	Rate	2009	7.73556	15.6055	3.86852
DALYs (Dis	Western Eu	Both	50-69 yea	HIV/AIDS	Number	2000	46793.9	51243.2	43799.5
DALYs (Dis	Western Eu	Both	50-69 yea	HIV/AIDS	Rate	2000	52.8937	57.923	49.5089
DALYs (Dis	Andean La	Both	50-69 yea	Sexually tr	Number	2009	1339.45	2582.89	729.942
DALYs (Dis	Andean La	Both	50-69 yea	Sexually tr	Rate	2009	20.4465	39.4272	11.1424
DALYs (Dis	Southern L	Both	50-69 yea	Sexually tr	Number	2009	1571.23	2965.38	886.189
DALYs (Dis	Southern L	Both	50-69 yea	Sexually tr	Rate	2009	15.2215	28.7274	8.58504
DALYs (Dis	Southern S	Both	50-69 yea	Sexually tr	Number	2000	3734.39	5694.01	2660.76
DALYs (Dis	Southern S	Both	50-69 yea	Sexually tr	Rate	2000	62.4823	95.2698	44.5187
DALYs (Dis	High-incor	Both	50-69 yea	Sexually tr	Number	2002	9570.3	18582.3	5052.96
DALYs (Dis	High-incor	Both	50-69 yea	Sexually tr	Rate	2002	15.889	30.8511	8.38913
DALYs (Dis	Tropical La	Both	50-69 yea	HIV/AIDS	Number	2009	118582	124225	113287
DALYs (Dis	Tropical La	Both	50-69 yea	HIV/AIDS	Rate	2009	397.258	416.162	379.518
DALYs (Dis	North Afric	Both	50-69 yea	HIV/AIDS	Number	1999	16257	30046.8	8726.34
DALYs (Dis	North Afric	Both	50-69 yea	HIV/AIDS	Rate	1999	41.8831	77.4099	22.4818
DALYs (Dis	Central Su	Both	50-69 yea	Sexually tr	Number	2009	4219.86	7822.2	1916.05
DALYs (Dis	Central Su	Both	50-69 yea	Sexually tr	Rate	2009	61.1541	113.359	27.7673
DALYs (Dis	Southern S	Both	50-69 yea	HIV/AIDS	Number	2000	1479454	1877231	1070986
DALYs (Dis	Southern S	Both	50-69 yea	HIV/AIDS	Rate	2000	24753.6	31409.1	17919.3
DALYs (Dis	Southern S	Both	50-69 yea	Sexually tr	Number	2009	4045.75	6613.8	2646.9
DALYs (Dis	Southern S	Both	50-69 yea	Sexually tr	Rate	2009	53.134	86.8609	34.7624
DALYs (Dis	Central As	Both	50-69 yea	HIV/AIDS	Number	2001	2278.14	2472.71	2146.9
DALYs (Dis	Central As	Both	50-69 yea	HIV/AIDS	Rate	2001	28.6495	31.0964	26.9991
DALYs (Dis	East Asia	Both	50-69 yea	HIV/AIDS	Number	2002	89755.6	118006	73845.2
DALYs (Dis	East Asia	Both	50-69 yea	HIV/AIDS	Rate	2002	42.043	55.276	34.5903
DALYs (Dis	Andean La	Both	50-69 yea	HIV/AIDS	Number	2001	12066.9	23587.9	9066.01
DALYs (Dis	Andean La	Both	50-69 yea	HIV/AIDS	Rate	2001	242.273	473.586	182.022
DALYs (Dis	Central As	Both	50-69 yea	Sexually tr	Number	2000	1882.99	2779.48	1394.54
DALYs (Dis	Central As	Both	50-69 yea	Sexually tr	Rate	2000	23.95	35.3526	17.7373
DALYs (Dis	Eastern Su	Both	50-69 yea	HIV/AIDS	Number	2002	3035282	3360373	2733536
DALYs (Dis	Eastern Su	Both	50-69 yea	HIV/AIDS	Rate	2002	17659	19550.4	15903.5
DALYs (Dis	Eastern Su	Both	50-69 yea	Sexually tr	Number	2002	17719	30896.5	8815.63
DALYs (Dis	Eastern Su	Both	50-69 yea	Sexually tr	Rate	2002	103.088	179.753	51.2887
DALYs (Dis	Central Eu	Both	50-69 yea	HIV/AIDS	Number	1999	5659.72	6010.2	5289.97
DALYs (Dis	Central Eu	Both	50-69 yea	HIV/AIDS	Rate	1999	22.6992	24.1049	21.2163
DALYs (Dis	Eastern Su	Both	50-69 yea	HIV/AIDS	Number	2001	2941252	3311925	2597326
DALYs (Dis	Eastern Su	Both	50-69 yea	HIV/AIDS	Rate	2001	17578.1	19793.4	15522.7
DALYs (Dis	Eastern Su	Both	50-69 yea	Sexually tr	Number	2001	17883.8	32415.7	8746.85
DALYs (Dis	Eastern Su	Both	50-69 yea	Sexually tr	Rate	2001	106.881	193.73	52.2748
DALYs (Dis	Southern S	Both	50-69 yea	HIV/AIDS	Number	1999	1234970	1624030	855266
DALYs (Dis	Southern S	Both	50-69 yea	HIV/AIDS	Rate	1999	21074.1	27713.2	14594.6
DALYs (Dis	Oceania	Both	50-69 yea	Sexually tr	Number	2000	177.65	326.544	91.9681
DALYs (Dis	Oceania	Both	50-69 yea	Sexually tr	Rate	2000	25.9281	47.6593	13.4228
DALYs (Dis	High-incor	Both	50-69 yea	HIV/AIDS	Number	2002	153025	167367	142959
DALYs (Dis	High-incor	Both	50-69 yea	HIV/AIDS	Rate	2002	254.058	277.87	237.345
DALYs (Dis	Eastern Eu	Both	50-69 yea	HIV/AIDS	Number	2001	43459	46625.8	41419.9
DALYs (Dis	Eastern Eu	Both	50-69 yea	HIV/AIDS	Rate	2001	96.6465	103.689	92.1117
DALYs (Dis	Andean La	Both	50-69 yea	HIV/AIDS	Number	2000	10658.9	22972.4	7743.45
DALYs (Dis	Andean La	Both	50-69 yea	HIV/AIDS	Rate	2000	221.067	476.452	160.601
DALYs (Dis	Eastern Eu	Both	50-69 yea	Sexually tr	Number	2000	13288.9	18228.4	8373.21
DALYs (Dis	Eastern Eu	Both	50-69 yea	Sexually tr	Rate	2000	29.6541	40.6764	18.6847
DALYs (Dis	East Asia	Both	50-69 yea	Sexually tr	Number	2002	29782.1	52680.9	17623.5
DALYs (Dis	East Asia	Both	50-69 yea	Sexually tr	Rate	2002	13.9504	24.6766	8.25512
DALYs (Dis	Andean La	Both	50-69 yea	Sexually tr	Number	2000	1054.64	1967.42	583.213
DALYs (Dis	Andean La	Both	50-69 yea	Sexually tr	Rate	2000	21.8734	40.8046	12.0959
DALYs (Dis	Tropical La	Both	50-69 yea	HIV/AIDS	Number	2002	72598.7	75008	70436.5
DALYs (Dis	Tropical La	Both	50-69 yea	HIV/AIDS	Rate	2002	314.349	324.781	304.987
DALYs (Dis	Australasia	Both	50-69 yea	HIV/AIDS	Number	2002	1659.22	1783.57	1542.49
DALYs (Dis	Australasia	Both	50-69 yea	HIV/AIDS	Rate	2002	36.481	39.215	33.9143
DALYs (Dis	Central Lat	Both	50-69 yea	HIV/AIDS	Number	2002	57516.8	60880.4	55441.7
DALYs (Dis	Central Lat	Both	50-69 yea	HIV/AIDS	Rate	2002	264.667	280.145	255.119
DALYs (Dis	Central Lat	Both	50-69 yea	Sexually tr	Number	2002	5097.04	9714	2751.05
DALYs (Dis	Central Lat	Both	50-69 yea	Sexually tr	Rate	2002	23.4544	44.6996	12.6591
DALYs (Dis	Western Su	Both	50-69 yea	HIV/AIDS	Number	2002	1268277	1461058	1100255
DALYs (Dis	Western Su	Both	50-69 yea	HIV/AIDS	Rate	2002	6269.03	7221.94	5438.51
DALYs (Dis	Caribbean	Both	50-69 yea	Sexually tr	Number	2002	1569.5	2709.03	979.267
DALYs (Dis	Caribbean	Both	50-69 yea	Sexually tr	Rate	2002	28.0905	48.4854	17.5266
DALYs (Dis	Oceania	Both	50-69 yea	HIV/AIDS	Number	2002	9082.95	19249.7	3728.63
DALYs (Dis	Oceania	Both	50-69 yea	HIV/AIDS	Rate	2002	1241.7	2631.58	509.73
DALYs (Dis	Central Lat	Both	50-69 yea	HIV/AIDS	Number	2003	63043.2	66006.3	60993.9
DALYs (Dis	Central Lat	Both	50-69 yea	HIV/AIDS	Rate	2003	279.523	292.661	270.437
DALYs (Dis	Central Lat	Both	50-69 yea	Sexually tr	Number	2003	5292.84	10075.7	2843.74
DALYs (Dis	Central Lat	Both	50-69 yea	Sexually tr	Rate	2003	23.4676	44.674	12.6087
DALYs (Dis	Caribbean	Both	50-69 yea	HIV/AIDS	Number	2002	116715	139775	97583.4
DALYs (Dis	Caribbean	Both	50-69 yea	HIV/AIDS	Rate	2002	2088.93	2501.65	1746.52
DALYs (Dis	Caribbean	Both	50-69 yea	HIV/AIDS	Number	2003	117121	139160	98766.9
DALYs (Dis	Caribbean	Both	50-69 yea	HIV/AIDS	Rate	2003	2042.86	2427.28	1722.73
DALYs (Dis	Southern L	Both	50-69 yea	HIV/AIDS	Number	2010	19358.5	20925.3	18250.7
DALYs (Dis	Southern L	Both	50-69 yea	HIV/AIDS	Rate	2010	183.227	198.056	172.741
DALYs (Dis	High-incor	Both	50-69 yea	Sexually tr	Number	2001	9343.05	18007	4971.04
DALYs (Dis	High-incor	Both	50-69 yea	Sexually tr	Rate	2001	16.0115	30.859	8.51901
DALYs (Dis	Tropical La	Both	50-69 yea	Sexually tr	Number	2002	6587.13	12474.6	3631.72
DALYs (Dis	Tropical La	Both	50-69 yea	Sexually tr	Rate	2002	28.522	54.0146	15.7252
DALYs (Dis	Australasia	Both	50-69 yea	Sexually tr	Number	2001	393.637	778.09	190.934
DALYs (Dis	Australasia	Both	50-69 yea	Sexually tr	Rate	2001	8.92815	17.648	4.33061
DALYs (Dis	Andean La	Both	50-69 yea	HIV/AIDS	Number	2010	15944.2	25035.9	12015.7
DALYs (Dis	Andean La	Both	50-69 yea	HIV/AIDS	Rate	2010	235.082	369.131	177.16
DALYs (Dis	Western Su	Both	50-69 yea	HIV/AIDS	Number	2001	1201860	1403166	1018059
DALYs (Dis	Western Su	Both	50-69 yea	HIV/AIDS	Rate	2001	6085.84	7105.19	5155.13
DALYs (Dis	East Asia	Both	50-69 yea	Sexually tr	Number	2001	28504.5	50871.6	16828.4
DALYs (Dis	East Asia	Both	50-69 yea	Sexually tr	Rate	2001	13.8488	24.7157	8.17598
DALYs (Dis	Central Su	Both	50-69 yea	HIV/AIDS	Number	2010	342384	420830	277776
DALYs (Dis	Central Su	Both	50-69 yea	HIV/AIDS	Rate	2010	4772.6	5866.08	3872.01
DALYs (Dis	Western Su	Both	50-69 yea	Sexually tr	Number	2010	5973.06	11374.5	3183.03
DALYs (Dis	Western Su	Both	50-69 yea	Sexually tr	Rate	2010	23.474	44.7014	12.5093
DALYs (Dis	Australasia	Both	50-69 yea	Sexually tr	Number	2010	485.275	990.32	228.27
DALYs (Dis	Australasia	Both	50-69 yea	Sexually tr	Rate	2010	8.47165	17.2884	3.985
DALYs (Dis	Southern S	Both	50-69 yea	HIV/AIDS	Number	2010	1929373	2336574	1582324
DALYs (Dis	Southern S	Both	50-69 yea	HIV/AIDS	Rate	2010	24659	29863.3	20223.4
DALYs (Dis	Central As	Both	50-69 yea	Sexually tr	Number	2002	1744.01	2670.95	1256.81
DALYs (Dis	Central As	Both	50-69 yea	Sexually tr	Rate	2002	21.5313	32.9751	15.5164
DALYs (Dis	Central Su	Both	50-69 yea	HIV/AIDS	Number	2001	350873	414252	295029
DALYs (Dis	Central Su	Both	50-69 yea	HIV/AIDS	Rate	2001	6633.11	7831.27	5577.41
DALYs (Dis	Southeast	Both	50-69 yea	Sexually tr	Number	2002	11143.6	19883	6398.71
DALYs (Dis	Southeast	Both	50-69 yea	Sexually tr	Rate	2002	17.9062	31.9492	10.2819
DALYs (Dis	Australasia	Both	50-69 yea	HIV/AIDS	Number	2001	1755.63	1882.93	1644.78
DALYs (Dis	Australasia	Both	50-69 yea	HIV/AIDS	Rate	2001	39.8198	42.7072	37.3055
DALYs (Dis	Tropical La	Both	50-69 yea	Sexually tr	Number	2001	6396.14	12088.7	3569.65
DALYs (Dis	Tropical La	Both	50-69 yea	Sexually tr	Rate	2001	28.7035	54.2497	16.0193
DALYs (Dis	Central Su	Both	50-69 yea	HIV/AIDS	Number	2003	371330	435687	316386
DALYs (Dis	Central Su	Both	50-69 yea	HIV/AIDS	Rate	2003	6610.78	7756.53	5632.6
DALYs (Dis	Western Su	Both	50-69 yea	Sexually tr	Number	2001	5170.33	9381.63	3008.41
DALYs (Dis	Western Su	Both	50-69 yea	Sexually tr	Rate	2001	26.1809	47.5056	15.2337
DALYs (Dis	Southeast	Both	50-69 yea	HIV/AIDS	Number	2002	329368	370177	286751
DALYs (Dis	Southeast	Both	50-69 yea	HIV/AIDS	Rate	2002	529.248	594.823	460.769
DALYs (Dis	North Afric	Both	50-69 yea	Sexually tr	Number	2002	4746.24	9131.68	2530.31
DALYs (Dis	North Afric	Both	50-69 yea	Sexually tr	Rate	2002	11.2867	21.7154	6.01713
DALYs (Dis	Southern L	Both	50-69 yea	Sexually tr	Number	2004	1455.16	2676.84	830.446
DALYs (Dis	Southern L	Both	50-69 yea	Sexually tr	Rate	2004	15.8526	29.1616	9.04689
DALYs (Dis	Southeast	Both	50-69 yea	HIV/AIDS	Number	2003	345493	395017	297717
DALYs (Dis	Southeast	Both	50-69 yea	HIV/AIDS	Rate	2003	537.975	615.091	463.582
DALYs (Dis	Western Eu	Both	50-69 yea	HIV/AIDS	Number	2010	60959.4	67686.2	55854.5
DALYs (Dis	Western Eu	Both	50-69 yea	HIV/AIDS	Rate	2010	61.206	67.96	56.0804
DALYs (Dis	South Asia	Both	50-69 yea	HIV/AIDS	Number	2002	381606	435770	339728
DALYs (Dis	South Asia	Both	50-69 yea	HIV/AIDS	Rate	2002	268.513	306.624	239.045
DALYs (Dis	South Asia	Both	50-69 yea	Sexually tr	Number	2002	42402.6	55686.9	32241.6
DALYs (Dis	South Asia	Both	50-69 yea	Sexually tr	Rate	2002	29.8361	39.1834	22.6864
DALYs (Dis	Central Su	Both	50-69 yea	Sexually tr	Number	2002	4027.74	8067.88	1640.74
DALYs (Dis	Central Su	Both	50-69 yea	Sexually tr	Rate	2002	73.9141	148.056	30.1097
DALYs (Dis	Central As	Both	50-69 yea	Sexually tr	Number	2004	1742.08	2757.03	1208.64
DALYs (Dis	Central As	Both	50-69 yea	Sexually tr	Rate	2004	20.5934	32.5913	14.2875
DALYs (Dis	Western Eu	Both	50-69 yea	Sexually tr	Number	2002	7104.12	13993.1	3633.15
DALYs (Dis	Western Eu	Both	50-69 yea	Sexually tr	Rate	2002	7.85235	15.4669	4.01581
DALYs (Dis	Central Lat	Both	50-69 yea	HIV/AIDS	Number	2001	56353.1	60059.9	54117.9
DALYs (Dis	Central Lat	Both	50-69 yea	HIV/AIDS	Rate	2001	269.166	286.871	258.49
DALYs (Dis	Central Lat	Both	50-69 yea	Sexually tr	Number	2001	4901.2	9303.45	2644.85
DALYs (Dis	Central Lat	Both	50-69 yea	Sexually tr	Rate	2001	23.4102	44.4372	12.6329
DALYs (Dis	Central Su	Both	50-69 yea	Sexually tr	Number	2003	4042.84	8071.17	1690.23
DALYs (Dis	Central Su	Both	50-69 yea	Sexually tr	Rate	2003	71.9745	143.691	30.0912
DALYs (Dis	Southern S	Both	50-69 yea	Sexually tr	Number	2002	3676.21	5762.07	2508.4
DALYs (Dis	Southern S	Both	50-69 yea	Sexually tr	Rate	2002	58.2073	91.2338	39.7168
DALYs (Dis	Eastern Su	Both	50-69 yea	HIV/AIDS	Number	2000	2800688	3201967	2411218
DALYs (Dis	Eastern Su	Both	50-69 yea	HIV/AIDS	Rate	2000	17204.5	19669.6	14812
DALYs (Dis	Eastern Su	Both	50-69 yea	Sexually tr	Number	2000	18107.8	33007	8748.72
DALYs (Dis	Eastern Su	Both	50-69 yea	Sexually tr	Rate	2000	111.235	202.761	53.743
DALYs (Dis	High-incor	Both	50-69 yea	HIV/AIDS	Number	2004	6243.77	7381.84	5426.6
DALYs (Dis	High-incor	Both	50-69 yea	HIV/AIDS	Rate	2004	14.0224	16.5783	12.1872
DALYs (Dis	Oceania	Both	50-69 yea	HIV/AIDS	Number	2003	11154.4	23514.3	4458.04
DALYs (Dis	Oceania	Both	50-69 yea	HIV/AIDS	Rate	2003	1475.64	3110.76	589.763
DALYs (Dis	Central Eu	Both	50-69 yea	HIV/AIDS	Number	2002	5424.68	5751.83	5161.1
DALYs (Dis	Central Eu	Both	50-69 yea	HIV/AIDS	Rate	2002	20.9591	22.2232	19.9408
DALYs (Dis	Caribbean	Both	50-69 yea	Sexually tr	Number	2001	1549.78	2672.62	962.588
DALYs (Dis	Caribbean	Both	50-69 yea	Sexually tr	Rate	2001	28.4691	49.0956	17.6826
DALYs (Dis	South Asia	Both	50-69 yea	HIV/AIDS	Number	2003	454664	516736	404377
DALYs (Dis	South Asia	Both	50-69 yea	HIV/AIDS	Rate	2003	310.757	353.183	276.387
DALYs (Dis	South Asia	Both	50-69 yea	Sexually tr	Number	2003	42038.5	55068.4	31827.4
DALYs (Dis	South Asia	Both	50-69 yea	Sexually tr	Rate	2003	28.7328	37.6385	21.7537
DALYs (Dis	Oceania	Both	50-69 yea	Sexually tr	Number	2002	188.908	348.345	99.0949
DALYs (Dis	Oceania	Both	50-69 yea	Sexually tr	Rate	2002	25.8251	47.6212	13.547
DALYs (Dis	Central As	Both	50-69 yea	Sexually tr	Number	2011	2057.4	3410.94	1336.99
DALYs (Dis	Central As	Both	50-69 yea	Sexually tr	Rate	2011	19.0594	31.5984	12.3857
DALYs (Dis	Oceania	Both	50-69 yea	Sexually tr	Number	2011	260.489	489.061	138.647
DALYs (Dis	Oceania	Both	50-69 yea	Sexually tr	Rate	2011	25.5778	48.0217	13.614
DALYs (Dis	Central Eu	Both	50-69 yea	Sexually tr	Number	2002	2500.42	4692.68	1361.72
DALYs (Dis	Central Eu	Both	50-69 yea	Sexually tr	Rate	2002	9.66078	18.131	5.26122
DALYs (Dis	Central Eu	Both	50-69 yea	Sexually tr	Number	2011	2742.61	5142.24	1460.29
DALYs (Dis	Central Eu	Both	50-69 yea	Sexually tr	Rate	2011	9.39355	17.6124	5.00156
DALYs (Dis	Southern L	Both	50-69 yea	Sexually tr	Number	2002	1420.21	2594.57	821.707
DALYs (Dis	Southern L	Both	50-69 yea	Sexually tr	Rate	2002	16.1085	29.4284	9.32005
DALYs (Dis	Central As	Both	50-69 yea	HIV/AIDS	Number	2002	2333.83	2567.61	2193.11
DALYs (Dis	Central As	Both	50-69 yea	HIV/AIDS	Rate	2002	28.8131	31.6993	27.0757
DALYs (Dis	Southeast	Both	50-69 yea	Sexually tr	Number	2011	14463.6	27404.5	8003.85
DALYs (Dis	Southeast	Both	50-69 yea	Sexually tr	Rate	2011	16.7166	31.6733	9.25062
DALYs (Dis	Western Eu	Both	50-69 yea	HIV/AIDS	Number	2003	51802	56346.5	48764.9
DALYs (Dis	Western Eu	Both	50-69 yea	HIV/AIDS	Rate	2003	56.5663	61.5288	53.2499
DALYs (Dis	Eastern Eu	Both	50-69 yea	Sexually tr	Number	2011	8216.89	14351.5	5154.24
DALYs (Dis	Eastern Eu	Both	50-69 yea	Sexually tr	Rate	2011	16.4197	28.6784	10.2996
DALYs (Dis	Australasia	Both	50-69 yea	HIV/AIDS	Number	2011	1955.2	2204.04	1748.68
DALYs (Dis	Australasia	Both	50-69 yea	HIV/AIDS	Rate	2011	33.2455	37.4766	29.7339
DALYs (Dis	North Afric	Both	50-69 yea	HIV/AIDS	Number	2003	25138	44724.7	14267.2
DALYs (Dis	North Afric	Both	50-69 yea	HIV/AIDS	Rate	2003	57.9203	103.05	32.8729
DALYs (Dis	High-incor	Both	50-69 yea	Sexually tr	Number	2003	4413.2	8347.88	2348.2
DALYs (Dis	High-incor	Both	50-69 yea	Sexually tr	Rate	2003	9.99785	18.9116	5.3197
DALYs (Dis	Southern L	Both	50-69 yea	Sexually tr	Number	2003	1441.37	2660.81	834.823
DALYs (Dis	Southern L	Both	50-69 yea	Sexually tr	Rate	2003	16.0335	29.5983	9.28641
DALYs (Dis	Caribbean	Both	50-69 yea	HIV/AIDS	Number	2001	114982	138960	95066.5
DALYs (Dis	Caribbean	Both	50-69 yea	HIV/AIDS	Rate	2001	2112.19	2552.68	1746.35
DALYs (Dis	Caribbean	Both	50-69 yea	Sexually tr	Number	2011	1895.46	3223.83	1155.2
DALYs (Dis	Caribbean	Both	50-69 yea	Sexually tr	Rate	2011	27.4204	46.637	16.7115
DALYs (Dis	North Afric	Both	50-69 yea	Sexually tr	Number	2011	6229.54	12107.9	3200.6
DALYs (Dis	North Afric	Both	50-69 yea	Sexually tr	Rate	2011	10.7527	20.8993	5.52452
DALYs (Dis	Andean La	Both	50-69 yea	Sexually tr	Number	2004	1176.93	2222.65	650.683
DALYs (Dis	Andean La	Both	50-69 yea	Sexually tr	Rate	2004	21.3565	40.3319	11.8072
DALYs (Dis	Central Eu	Both	50-69 yea	HIV/AIDS	Number	2003	4911.74	5290.48	4646.37
DALYs (Dis	Central Eu	Both	50-69 yea	HIV/AIDS	Rate	2003	18.6906	20.1318	17.6808
DALYs (Dis	Central As	Both	50-69 yea	HIV/AIDS	Number	2003	2430.8	2700.35	2270.87
DALYs (Dis	Central As	Both	50-69 yea	HIV/AIDS	Rate	2003	29.452	32.7179	27.5143
DALYs (Dis	Southern S	Both	50-69 yea	HIV/AIDS	Number	2003	2205248	2578670	1802474
DALYs (Dis	Southern S	Both	50-69 yea	HIV/AIDS	Rate	2003	34001.5	39759	27791.3
DALYs (Dis	Eastern Su	Both	50-69 yea	HIV/AIDS	Number	2011	2080768	2505870	1830886
DALYs (Dis	Eastern Su	Both	50-69 yea	HIV/AIDS	Rate	2011	9357.53	11269.3	8233.77
DALYs (Dis	Eastern Su	Both	50-69 yea	Sexually tr	Number	2011	16673.9	25296.2	8952.52
DALYs (Dis	Eastern Su	Both	50-69 yea	Sexually tr	Rate	2011	74.9851	113.761	40.2608
DALYs (Dis	High-incor	Both	50-69 yea	HIV/AIDS	Number	2004	169387	187971	156660
DALYs (Dis	High-incor	Both	50-69 yea	HIV/AIDS	Rate	2004	262.747	291.575	243.005
DALYs (Dis	Western Su	Both	50-69 yea	HIV/AIDS	Number	2011	1146197	1298318	1016698
DALYs (Dis	Western Su	Both	50-69 yea	HIV/AIDS	Rate	2011	4356.75	4934.97	3864.52
DALYs (Dis	Western Eu	Both	50-69 yea	Sexually tr	Number	2003	7188.31	14368.6	3680.12
DALYs (Dis	Western Eu	Both	50-69 yea	Sexually tr	Rate	2003	7.84943	15.6901	4.01859
DALYs (Dis	North Afric	Both	50-69 yea	HIV/AIDS	Number	2002	22708.3	40559.9	12677.4
DALYs (Dis	North Afric	Both	50-69 yea	HIV/AIDS	Rate	2002	54.001	96.4526	30.1472
DALYs (Dis	Southern S	Both	50-69 yea	Sexually tr	Number	2003	3701.5	5873.93	2505.09
DALYs (Dis	Southern S	Both	50-69 yea	Sexually tr	Rate	2003	57.0714	90.5667	38.6246
DALYs (Dis	High-incor	Both	50-69 yea	HIV/AIDS	Number	2002	5749.63	6595.05	5105.75
DALYs (Dis	High-incor	Both	50-69 yea	HIV/AIDS	Rate	2002	13.1451	15.078	11.6731
DALYs (Dis	High-incor	Both	50-69 yea	HIV/AIDS	Number	2003	6243.86	7218.86	5486.51
DALYs (Dis	High-incor	Both	50-69 yea	HIV/AIDS	Rate	2003	14.1451	16.3539	12.4294
DALYs (Dis	High-incor	Both	50-69 yea	Sexually tr	Number	2002	4394.11	8333.46	2330.23
DALYs (Dis	High-incor	Both	50-69 yea	Sexually tr	Rate	2002	10.0461	19.0524	5.3275
DALYs (Dis	East Asia	Both	50-69 yea	Sexually tr	Number	2012	35908.2	65540.7	20234.3
DALYs (Dis	East Asia	Both	50-69 yea	Sexually tr	Rate	2012	12.2865	22.4256	6.92345
DALYs (Dis	Southern L	Both	50-69 yea	HIV/AIDS	Number	2003	13787.5	14755.1	13143.7
DALYs (Dis	Southern L	Both	50-69 yea	HIV/AIDS	Rate	2003	153.369	164.133	146.208
DALYs (Dis	Andean La	Both	50-69 yea	HIV/AIDS	Number	2004	14361.1	23260.1	11195.7
DALYs (Dis	Andean La	Both	50-69 yea	HIV/AIDS	Rate	2004	260.595	422.076	203.156
DALYs (Dis	Oceania	Both	50-69 yea	HIV/AIDS	Number	2012	20774.8	51024.7	7338.16
DALYs (Dis	Oceania	Both	50-69 yea	HIV/AIDS	Rate	2012	1961.12	4816.68	692.715
DALYs (Dis	Southeast	Both	50-69 yea	HIV/AIDS	Number	2012	352401	469304	292460
DALYs (Dis	Southeast	Both	50-69 yea	HIV/AIDS	Rate	2012	391.502	521.375	324.91
DALYs (Dis	Central As	Both	50-69 yea	HIV/AIDS	Number	2012	5155.24	5800.96	4715.63
DALYs (Dis	Central As	Both	50-69 yea	HIV/AIDS	Rate	2012	45.5436	51.2483	41.66
DALYs (Dis	Central Eu	Both	50-69 yea	HIV/AIDS	Number	2012	5973.96	7542.8	5197.26
DALYs (Dis	Central Eu	Both	50-69 yea	HIV/AIDS	Rate	2012	20.3162	25.6515	17.6748
DALYs (Dis	Andean La	Both	50-69 yea	Sexually tr	Number	2003	1150.1	2167.06	622.568
DALYs (Dis	Andean La	Both	50-69 yea	Sexually tr	Rate	2003	21.5792	40.6603	11.6812
DALYs (Dis	Eastern Eu	Both	50-69 yea	HIV/AIDS	Number	2012	80334	85684.5	77017.7
DALYs (Dis	Eastern Eu	Both	50-69 yea	HIV/AIDS	Rate	2012	157.606	168.104	151.1
DALYs (Dis	East Asia	Both	50-69 yea	Sexually tr	Number	2005	32898.6	57972.6	19465.5
DALYs (Dis	East Asia	Both	50-69 yea	Sexually tr	Rate	2005	13.657	24.0657	8.08057
DALYs (Dis	High-incor	Both	50-69 yea	Sexually tr	Number	2012	11741.2	22888.9	6060.71
DALYs (Dis	High-incor	Both	50-69 yea	Sexually tr	Rate	2012	14.0935	27.4744	7.27492
DALYs (Dis	High-incor	Both	50-69 yea	Sexually tr	Number	2012	4513.48	8884.99	2338.47
DALYs (Dis	High-incor	Both	50-69 yea	Sexually tr	Rate	2012	9.55922	18.8178	4.95271
DALYs (Dis	Eastern Su	Both	50-69 yea	HIV/AIDS	Number	2004	3125811	3439778	2824464
DALYs (Dis	Eastern Su	Both	50-69 yea	HIV/AIDS	Rate	2004	17275.5	19010.8	15610.1
DALYs (Dis	Eastern Su	Both	50-69 yea	Sexually tr	Number	2004	17218.1	29137.2	8659.7
DALYs (Dis	Eastern Su	Both	50-69 yea	Sexually tr	Rate	2004	95.16	161.033	47.8599
DALYs (Dis	Caribbean	Both	50-69 yea	HIV/AIDS	Number	2012	94775.9	112423	80632.2
DALYs (Dis	Caribbean	Both	50-69 yea	HIV/AIDS	Rate	2012	1333.44	1581.74	1134.45
DALYs (Dis	Western Su	Both	50-69 yea	HIV/AIDS	Number	2005	1327935	1472620	1198709
DALYs (Dis	Western Su	Both	50-69 yea	HIV/AIDS	Rate	2005	6073.77	6735.54	5482.71
DALYs (Dis	High-incor	Both	50-69 yea	HIV/AIDS	Number	2003	162917	179268	151386
DALYs (Dis	High-incor	Both	50-69 yea	HIV/AIDS	Rate	2003	261.676	287.939	243.155
DALYs (Dis	Central Lat	Both	50-69 yea	HIV/AIDS	Number	2012	90606.5	94570.1	87070.9
DALYs (Dis	Central Lat	Both	50-69 yea	HIV/AIDS	Rate	2012	285.554	298.046	274.411
DALYs (Dis	Central Lat	Both	50-69 yea	Sexually tr	Number	2012	7736.69	14593.9	4224.52
DALYs (Dis	Central Lat	Both	50-69 yea	Sexually tr	Rate	2012	24.3828	45.9939	13.3139
DALYs (Dis	Tropical La	Both	50-69 yea	Sexually tr	Number	2012	9234.33	17814.2	4934.85
DALYs (Dis	Tropical La	Both	50-69 yea	Sexually tr	Rate	2012	27.7563	53.5455	14.8331
DALYs (Dis	South Asia	Both	50-69 yea	HIV/AIDS	Number	2012	481010	650155	370796
DALYs (Dis	South Asia	Both	50-69 yea	HIV/AIDS	Rate	2012	245.662	332.048	189.374
DALYs (Dis	South Asia	Both	50-69 yea	Sexually tr	Number	2012	47213.6	64455.8	35076.5
DALYs (Dis	South Asia	Both	50-69 yea	Sexually tr	Rate	2012	24.113	32.919	17.9143
DALYs (Dis	North Afric	Both	50-69 yea	HIV/AIDS	Number	2012	44701.1	86137.8	26163.4
DALYs (Dis	North Afric	Both	50-69 yea	HIV/AIDS	Rate	2012	74.0794	142.749	43.3585
DALYs (Dis	Australasia	Both	50-69 yea	HIV/AIDS	Number	2004	2071.09	2229.55	1929.59
DALYs (Dis	Australasia	Both	50-69 yea	HIV/AIDS	Rate	2004	42.7613	46.0329	39.8399
DALYs (Dis	Eastern Eu	Both	50-69 yea	HIV/AIDS	Number	2003	50768.4	54659.6	48386.4
DALYs (Dis	Eastern Eu	Both	50-69 yea	HIV/AIDS	Rate	2003	110.847	119.343	105.646
DALYs (Dis	Eastern Eu	Both	50-69 yea	Sexually tr	Number	2004	9546.92	15012.6	6812.98
DALYs (Dis	Eastern Eu	Both	50-69 yea	Sexually tr	Rate	2004	20.5394	32.2984	14.6575
DALYs (Dis	Australasia	Both	50-69 yea	HIV/AIDS	Number	2005	1777.69	1938.02	1641.52
DALYs (Dis	Australasia	Both	50-69 yea	HIV/AIDS	Rate	2005	35.5921	38.8022	32.8656
DALYs (Dis	Tropical La	Both	50-69 yea	Sexually tr	Number	2005	7160.14	13651.9	3894.59
DALYs (Dis	Tropical La	Both	50-69 yea	Sexually tr	Rate	2005	27.7738	52.9549	15.1069
DALYs (Dis	East Asia	Both	50-69 yea	HIV/AIDS	Number	2004	108792	134668	95150.5
DALYs (Dis	East Asia	Both	50-69 yea	HIV/AIDS	Rate	2004	47.0127	58.1943	41.1176
DALYs (Dis	East Asia	Both	50-69 yea	HIV/AIDS	Number	2003	94775.5	122713	79758.4
DALYs (Dis	East Asia	Both	50-69 yea	HIV/AIDS	Rate	2003	42.6669	55.2438	35.9063
DALYs (Dis	Australasia	Both	50-69 yea	Sexually tr	Number	2005	385.9	747.377	190.982
DALYs (Dis	Australasia	Both	50-69 yea	Sexually tr	Rate	2005	7.7263	14.9636	3.82374
DALYs (Dis	Central Su	Both	50-69 yea	HIV/AIDS	Number	2005	374255	444323	313101
DALYs (Dis	Central Su	Both	50-69 yea	HIV/AIDS	Rate	2005	6254.01	7424.9	5232.1
DALYs (Dis	High-incor	Both	50-69 yea	Sexually tr	Number	2003	9836.39	19233.8	5204.54
DALYs (Dis	High-incor	Both	50-69 yea	Sexually tr	Rate	2003	15.7992	30.8933	8.35951
DALYs (Dis	Western Eu	Both	50-69 yea	Sexually tr	Number	2004	7243.55	14393	3688.19
DALYs (Dis	Western Eu	Both	50-69 yea	Sexually tr	Rate	2004	7.8233	15.545	3.98339
DALYs (Dis	Australasia	Both	50-69 yea	Sexually tr	Number	2003	387.451	756.282	195.851
DALYs (Dis	Australasia	Both	50-69 yea	Sexually tr	Rate	2003	8.25088	16.1052	4.17069
DALYs (Dis	Caribbean	Both	50-69 yea	HIV/AIDS	Number	2005	115650	136521	97196.2
DALYs (Dis	Caribbean	Both	50-69 yea	HIV/AIDS	Rate	2005	1922.59	2269.55	1615.81
DALYs (Dis	Australasia	Both	50-69 yea	Sexually tr	Number	2004	382.024	731.164	189.144
DALYs (Dis	Australasia	Both	50-69 yea	Sexually tr	Rate	2004	7.88758	15.0962	3.90522
DALYs (Dis	Western Su	Both	50-69 yea	Sexually tr	Number	2005	5290.81	9822.55	2942.98
DALYs (Dis	Western Su	Both	50-69 yea	Sexually tr	Rate	2005	24.1993	44.9268	13.4608
DALYs (Dis	Tropical La	Both	50-69 yea	HIV/AIDS	Number	2003	82306.7	85219.1	79673
DALYs (Dis	Tropical La	Both	50-69 yea	HIV/AIDS	Rate	2003	343.68	355.841	332.683
DALYs (Dis	Southeast	Both	50-69 yea	Sexually tr	Number	2005	12294	22119.6	7072.83
DALYs (Dis	Southeast	Both	50-69 yea	Sexually tr	Rate	2005	17.901	32.2077	10.2986
DALYs (Dis	Central Su	Both	50-69 yea	Sexually tr	Number	2004	4032.02	8079.66	1715.25
DALYs (Dis	Central Su	Both	50-69 yea	Sexually tr	Rate	2004	69.5868	139.443	29.6028
DALYs (Dis	North Afric	Both	50-69 yea	Sexually tr	Number	2005	5209.28	10047.2	2712.72
DALYs (Dis	North Afric	Both	50-69 yea	Sexually tr	Rate	2005	11.2234	21.6467	5.84457
DALYs (Dis	Tropical La	Both	50-69 yea	HIV/AIDS	Number	2004	83926.7	87001.4	81033.9
DALYs (Dis	Tropical La	Both	50-69 yea	HIV/AIDS	Rate	2004	337.819	350.196	326.175
DALYs (Dis	Caribbean	Both	50-69 yea	Sexually tr	Number	2005	1667.69	2916.24	1035.76
DALYs (Dis	Caribbean	Both	50-69 yea	Sexually tr	Rate	2005	27.7241	48.4802	17.2187
DALYs (Dis	Western Su	Both	50-69 yea	Sexually tr	Number	2003	5184.15	9485.12	2932.28
DALYs (Dis	Western Su	Both	50-69 yea	Sexually tr	Rate	2003	25.002	45.7447	14.1417
DALYs (Dis	Central Lat	Both	50-69 yea	HIV/AIDS	Number	2005	67784.3	70897.8	65647.8
DALYs (Dis	Central Lat	Both	50-69 yea	HIV/AIDS	Rate	2005	278.671	291.471	269.887
DALYs (Dis	Central Lat	Both	50-69 yea	Sexually tr	Number	2005	5748.81	10964.7	3109.84
DALYs (Dis	Central Lat	Both	50-69 yea	Sexually tr	Rate	2005	23.6341	45.0773	12.785
DALYs (Dis	Oceania	Both	50-69 yea	HIV/AIDS	Number	2005	14578.9	32310.8	5268.94
DALYs (Dis	Oceania	Both	50-69 yea	HIV/AIDS	Rate	2005	1799.61	3988.43	650.396
DALYs (Dis	East Asia	Both	50-69 yea	Sexually tr	Number	2003	30871.3	54502.2	18442.1
DALYs (Dis	East Asia	Both	50-69 yea	Sexually tr	Rate	2003	13.8979	24.5363	8.3024
DALYs (Dis	East Asia	Both	50-69 yea	HIV/AIDS	Number	2013	163841	190492	146244
DALYs (Dis	East Asia	Both	50-69 yea	HIV/AIDS	Rate	2013	53.8571	62.6177	48.0728
DALYs (Dis	Southeast	Both	50-69 yea	HIV/AIDS	Number	2006	341708	426411	285919
DALYs (Dis	Southeast	Both	50-69 yea	HIV/AIDS	Rate	2006	480.076	599.078	401.697
DALYs (Dis	Southern S	Both	50-69 yea	HIV/AIDS	Number	2005	2479444	2754767	2183838
DALYs (Dis	Southern S	Both	50-69 yea	HIV/AIDS	Rate	2005	36287.5	40317	31961.2
DALYs (Dis	Western Eu	Both	50-69 yea	Sexually tr	Number	2013	7714.6	15498.3	3810.77
DALYs (Dis	Western Eu	Both	50-69 yea	Sexually tr	Rate	2013	7.37724	14.8205	3.64413
DALYs (Dis	Western Su	Both	50-69 yea	Sexually tr	Number	2004	5241.66	9634.16	2973.13
DALYs (Dis	Western Su	Both	50-69 yea	Sexually tr	Rate	2004	24.6422	45.2924	13.9774
DALYs (Dis	High-incor	Both	50-69 yea	HIV/AIDS	Number	2013	10082	12405.6	8462.07
DALYs (Dis	High-incor	Both	50-69 yea	HIV/AIDS	Rate	2013	21.1887	26.072	17.7841
DALYs (Dis	Southeast	Both	50-69 yea	Sexually tr	Number	2006	12700.5	23016.4	7429.82
DALYs (Dis	Southeast	Both	50-69 yea	Sexually tr	Rate	2006	17.8434	32.3364	10.4384
DALYs (Dis	Andean La	Both	50-69 yea	HIV/AIDS	Number	2003	14081.1	23755.7	10779.9
DALYs (Dis	Andean La	Both	50-69 yea	HIV/AIDS	Rate	2003	264.201	445.725	202.262
DALYs (Dis	Oceania	Both	50-69 yea	Sexually tr	Number	2005	210.829	392.936	111.261
DALYs (Dis	Oceania	Both	50-69 yea	Sexually tr	Rate	2005	26.0247	48.5039	13.734
DALYs (Dis	Central Su	Both	50-69 yea	HIV/AIDS	Number	2004	375888	442396	317629
DALYs (Dis	Central Su	Both	50-69 yea	HIV/AIDS	Rate	2004	6487.28	7635.12	5481.81
DALYs (Dis	Southern L	Both	50-69 yea	Sexually tr	Number	2013	1736.16	3278.07	988.426
DALYs (Dis	Southern L	Both	50-69 yea	Sexually tr	Rate	2013	15.3628	29.0068	8.74633
DALYs (Dis	Central Eu	Both	50-69 yea	Sexually tr	Number	2006	2690.26	5047.14	1452.94
DALYs (Dis	Central Eu	Both	50-69 yea	Sexually tr	Rate	2006	9.73962	18.2723	5.26009
DALYs (Dis	High-incor	Both	50-69 yea	HIV/AIDS	Number	2013	182811	222963	153489
DALYs (Dis	High-incor	Both	50-69 yea	HIV/AIDS	Rate	2013	214.865	262.057	180.401
DALYs (Dis	Andean La	Both	50-69 yea	Sexually tr	Number	2013	1504.32	2925.32	788.596
DALYs (Dis	Andean La	Both	50-69 yea	Sexually tr	Rate	2013	19.8643	38.6283	10.4133
DALYs (Dis	Western Su	Both	50-69 yea	HIV/AIDS	Number	2004	1340603	1490447	1208743
DALYs (Dis	Western Su	Both	50-69 yea	HIV/AIDS	Rate	2004	6302.48	7006.94	5682.58
DALYs (Dis	Central As	Both	50-69 yea	HIV/AIDS	Number	2005	3043.35	3412.09	2797.36
DALYs (Dis	Central As	Both	50-69 yea	HIV/AIDS	Rate	2005	35.0166	39.2592	32.1862
DALYs (Dis	Tropical La	Both	50-69 yea	HIV/AIDS	Number	2013	147004	155427	139527
DALYs (Dis	Tropical La	Both	50-69 yea	HIV/AIDS	Rate	2013	426.535	450.977	404.84
DALYs (Dis	Caribbean	Both	50-69 yea	Sexually tr	Number	2004	1634.46	2840.68	1012.57
DALYs (Dis	Caribbean	Both	50-69 yea	Sexually tr	Rate	2004	27.8199	48.3507	17.2347
DALYs (Dis	Central Eu	Both	50-69 yea	HIV/AIDS	Number	2006	5602.3	6169.84	5289.93
DALYs (Dis	Central Eu	Both	50-69 yea	HIV/AIDS	Rate	2006	20.2821	22.3368	19.1513
DALYs (Dis	Southeast	Both	50-69 yea	HIV/AIDS	Number	2005	359056	426381	306697
DALYs (Dis	Southeast	Both	50-69 yea	HIV/AIDS	Rate	2005	522.812	620.843	446.574
DALYs (Dis	Oceania	Both	50-69 yea	HIV/AIDS	Number	2006	15554.7	35368.3	5044.07
DALYs (Dis	Oceania	Both	50-69 yea	HIV/AIDS	Rate	2006	1847.48	4200.8	599.098
DALYs (Dis	Southern L	Both	50-69 yea	HIV/AIDS	Number	2005	13908.1	14934.8	13259.3
DALYs (Dis	Southern L	Both	50-69 yea	HIV/AIDS	Rate	2005	148.174	159.112	141.261
DALYs (Dis	Central Su	Both	50-69 yea	Sexually tr	Number	2013	4494.18	8272.03	2082.67
DALYs (Dis	Central Su	Both	50-69 yea	Sexually tr	Rate	2013	55.4106	101.989	25.678
DALYs (Dis	Western Eu	Both	50-69 yea	Sexually tr	Number	2006	7381.53	14785.2	3715.35
DALYs (Dis	Western Eu	Both	50-69 yea	Sexually tr	Rate	2006	7.79215	15.6077	3.92203
DALYs (Dis	Southern S	Both	50-69 yea	Sexually tr	Number	2013	4100.7	6853.87	2659.85
DALYs (Dis	Southern S	Both	50-69 yea	Sexually tr	Rate	2013	48.1282	80.4411	31.2176
DALYs (Dis	Southeast	Both	50-69 yea	Sexually tr	Number	2004	11880.7	21382	6875.91
DALYs (Dis	Southeast	Both	50-69 yea	Sexually tr	Rate	2004	17.9056	32.2251	10.3628
DALYs (Dis	North Afric	Both	50-69 yea	Sexually tr	Number	2006	5364.31	10403.6	2782.58
DALYs (Dis	North Afric	Both	50-69 yea	Sexually tr	Rate	2006	11.1601	21.6439	5.78897
DALYs (Dis	South Asia	Both	50-69 yea	HIV/AIDS	Number	2005	574203	648584	505323
DALYs (Dis	South Asia	Both	50-69 yea	HIV/AIDS	Rate	2005	369.633	417.513	325.292
DALYs (Dis	South Asia	Both	50-69 yea	Sexually tr	Number	2005	41879.7	55761.3	31342.8
DALYs (Dis	South Asia	Both	50-69 yea	Sexually tr	Rate	2005	26.9593	35.8953	20.1764
DALYs (Dis	Central Eu	Both	50-69 yea	HIV/AIDS	Number	2005	5712.8	6215.25	5392.65
DALYs (Dis	Central Eu	Both	50-69 yea	HIV/AIDS	Rate	2005	21.0285	22.878	19.8501
DALYs (Dis	North Afric	Both	50-69 yea	HIV/AIDS	Number	2006	31254.1	55336	18016.1
DALYs (Dis	North Afric	Both	50-69 yea	HIV/AIDS	Rate	2006	65.022	115.123	37.4813
DALYs (Dis	South Asia	Both	50-69 yea	HIV/AIDS	Number	2006	606381	696860	520222
DALYs (Dis	South Asia	Both	50-69 yea	HIV/AIDS	Rate	2006	378.397	434.857	324.631
DALYs (Dis	South Asia	Both	50-69 yea	Sexually tr	Number	2006	42913.9	56906.2	32414.3
DALYs (Dis	South Asia	Both	50-69 yea	Sexually tr	Rate	2006	26.7793	35.5108	20.2273
DALYs (Dis	Oceania	Both	50-69 yea	Sexually tr	Number	2006	219.106	407.754	115.878
DALYs (Dis	Oceania	Both	50-69 yea	Sexually tr	Rate	2006	26.0238	48.4302	13.7632
DALYs (Dis	Eastern Eu	Both	50-69 yea	HIV/AIDS	Number	2007	58668.7	63648.6	55821.6
DALYs (Dis	Eastern Eu	Both	50-69 yea	HIV/AIDS	Rate	2007	122.866	133.295	116.904
DALYs (Dis	Central As	Both	50-69 yea	Sexually tr	Number	2006	1813.89	2907.64	1233.8
DALYs (Dis	Central As	Both	50-69 yea	Sexually tr	Rate	2006	20.3005	32.5415	13.8084
DALYs (Dis	Western Eu	Both	50-69 yea	HIV/AIDS	Number	2004	52175.6	56664.1	48899.2
DALYs (Dis	Western Eu	Both	50-69 yea	HIV/AIDS	Rate	2004	56.3515	61.1994	52.813
DALYs (Dis	Eastern Eu	Both	50-69 yea	Sexually tr	Number	2006	8488.57	14417.8	5664.6
DALYs (Dis	Eastern Eu	Both	50-69 yea	Sexually tr	Rate	2006	17.8682	30.3491	11.9239
DALYs (Dis	Southern S	Both	50-69 yea	Sexually tr	Number	2006	3829.1	6176.88	2537.45
DALYs (Dis	Southern S	Both	50-69 yea	Sexually tr	Rate	2006	54.5653	88.0215	36.1591
DALYs (Dis	High-incor	Both	50-69 yea	HIV/AIDS	Number	2006	7426.94	8880.37	6446.66
DALYs (Dis	High-incor	Both	50-69 yea	HIV/AIDS	Rate	2006	16.4334	19.6494	14.2643
DALYs (Dis	Central Eu	Both	50-69 yea	Sexually tr	Number	2005	2653.32	4996.07	1432.28
DALYs (Dis	Central Eu	Both	50-69 yea	Sexually tr	Rate	2005	9.76673	18.3903	5.27216
DALYs (Dis	Australasia	Both	50-69 yea	Sexually tr	Number	2014	530.809	1083.4	249.829
DALYs (Dis	Australasia	Both	50-69 yea	Sexually tr	Rate	2014	8.40514	17.1551	3.95594
DALYs (Dis	Central As	Both	50-69 yea	HIV/AIDS	Number	2006	3276.27	3643.92	3001.31
DALYs (Dis	Central As	Both	50-69 yea	HIV/AIDS	Rate	2006	36.6671	40.7817	33.5898
DALYs (Dis	Western Eu	Both	50-69 yea	HIV/AIDS	Number	2014	64490.3	73967.1	57240.5
DALYs (Dis	Western Eu	Both	50-69 yea	HIV/AIDS	Rate	2014	60.6378	69.5485	53.8211
DALYs (Dis	Southern L	Both	50-69 yea	HIV/AIDS	Number	2014	24763.6	27369.3	23035
DALYs (Dis	Southern L	Both	50-69 yea	HIV/AIDS	Rate	2014	214.586	237.164	199.606

DALYs (Dis	Southern S	Both	50-69 yea	Sexually tra	Rate	2004	55.9889	89.7354	37.6173
DALYs (Dis	Central Eu	Both	50-69 yea	Sexually tra	Number	2004	2608.51	4881.34	1404.84
DALYs (Dis	Central Eu	Both	50-69 yea	Sexually tra	Rate	2004	9.77058	18.2838	5.26204
DALYs (Dis	High-incor	Both	50-69 yea	HIV/AIDS	Number	2007	184012	209114	166573
DALYs (Dis	High-incor	Both	50-69 yea	HIV/AIDS	Rate	2007	257.134	292.21	232.765
DALYs (Dis	Eastern Eu	Both	50-69 yea	HIV/AIDS	Number	2006	57564.9	62301.1	54672.2
DALYs (Dis	Eastern Eu	Both	50-69 yea	HIV/AIDS	Rate	2006	121.173	131.142	115.084
DALYs (Dis	Eastern Eu	Both	50-69 yea	HIV/AIDS	Number	2005	56978.2	61607.5	54257.2
DALYs (Dis	Eastern Eu	Both	50-69 yea	HIV/AIDS	Rate	2005	120.922	130.746	115.147
DALYs (Dis	East Asia	Both	50-69 yea	HIV/AIDS	Number	2007	125603	148121	112602
DALYs (Dis	East Asia	Both	50-69 yea	HIV/AIDS	Rate	2007	48.8166	57.5682	43.7636
DALYs (Dis	High-incor	Both	50-69 yea	Sexually tra	Number	2007	10765.5	21272.3	5561.36
DALYs (Dis	High-incor	Both	50-69 yea	Sexually tra	Rate	2007	15.0435	29.7253	7.77129
DALYs (Dis	High-incor	Both	50-69 yea	Sexually tra	Number	2006	10612.8	20780.6	5500.02
DALYs (Dis	High-incor	Both	50-69 yea	Sexually tra	Rate	2006	15.3516	30.0594	7.95587
DALYs (Dis	Eastern Eu	Both	50-69 yea	Sexually tra	Number	2005	8923.08	14747.3	6149.6
DALYs (Dis	Eastern Eu	Both	50-69 yea	Sexually tra	Rate	2005	18.937	31.2975	13.051
DALYs (Dis	Oceania	Both	50-69 yea	Sexually tra	Number	2004	202.378	371.681	105.394
DALYs (Dis	Oceania	Both	50-69 yea	Sexually tra	Rate	2004	25.8787	47.5281	13.4771
DALYs (Dis	East Asia	Both	50-69 yea	Sexually tra	Number	2007	33688.7	59556.7	19705
DALYs (Dis	East Asia	Both	50-69 yea	Sexually tra	Rate	2007	13.0933	23.1471	7.65847
DALYs (Dis	Andean La	Both	50-69 yea	HIV/AIDS	Number	2005	15732.2	24053	12407.8
DALYs (Dis	Andean La	Both	50-69 yea	HIV/AIDS	Rate	2005	275.729	421.564	217.465
DALYs (Dis	High-incor	Both	50-69 yea	Sexually tra	Number	2005	10401.4	20385.4	5445.17
DALYs (Dis	High-incor	Both	50-69 yea	Sexually tra	Rate	2005	15.5711	30.5172	8.15148
DALYs (Dis	Eastern Su	Both	50-69 yea	HIV/AIDS	Number	2006	2886548	3284593	2528077
DALYs (Dis	Eastern Su	Both	50-69 yea	HIV/AIDS	Rate	2006	15142.9	17231	13262.3
DALYs (Dis	Eastern Su	Both	50-69 yea	Sexually tra	Number	2006	16729.4	26808	8752.67
DALYs (Dis	Eastern Su	Both	50-69 yea	Sexually tra	Rate	2006	87.7629	140.635	45.9167
DALYs (Dis	Southeast	Both	50-69 yea	Sexually tra	Number	2015	16417.6	31475.3	8848.94
DALYs (Dis	Southeast	Both	50-69 yea	Sexually tra	Rate	2015	16.2861	31.2232	8.77806
DALYs (Dis	Central Eu	Both	50-69 yea	Sexually tra	Number	2015	2713.31	5216.24	1419.71
DALYs (Dis	Central Eu	Both	50-69 yea	Sexually tra	Rate	2015	9.07822	17.4526	4.75008
DALYs (Dis	Australasia	Both	50-69 yea	HIV/AIDS	Number	2015	1532.25	1836.34	1282.31
DALYs (Dis	Australasia	Both	50-69 yea	HIV/AIDS	Rate	2015	23.8361	28.5666	19.9479
DALYs (Dis	High-incor	Both	50-69 yea	Sexually tra	Number	2005	4460.44	8413.8	2359.54
DALYs (Dis	High-incor	Both	50-69 yea	Sexually tra	Rate	2005	9.92803	18.7274	5.25186
DALYs (Dis	Eastern Eu	Both	50-69 yea	Sexually tra	Number	2015	8485	14758.8	5245.91
DALYs (Dis	Eastern Eu	Both	50-69 yea	Sexually tra	Rate	2015	15.6086	27.1496	9.6501
DALYs (Dis	Andean La	Both	50-69 yea	Sexually tra	Number	2006	1236.54	2357.22	666.062
DALYs (Dis	Andean La	Both	50-69 yea	Sexually tra	Rate	2006	20.9485	39.9344	11.2839
DALYs (Dis	East Asia	Both	50-69 yea	HIV/AIDS	Number	2006	126408	154140	111541
DALYs (Dis	East Asia	Both	50-69 yea	HIV/AIDS	Rate	2006	50.6231	61.7289	44.6692
DALYs (Dis	Central Lat	Both	50-69 yea	HIV/AIDS	Number	2007	76452.5	78697	74541.1
DALYs (Dis	Central Lat	Both	50-69 yea	HIV/AIDS	Rate	2007	291.206	299.756	283.926
DALYs (Dis	Central Lat	Both	50-69 yea	Sexually tra	Number	2007	6182.17	11828.9	3348.27
DALYs (Dis	Central Lat	Both	50-69 yea	Sexually tra	Rate	2007	23.5478	45.0561	12.7535
DALYs (Dis	East Asia	Both	50-69 yea	Sexually tra	Number	2006	33476.7	59369.5	19379.5
DALYs (Dis	East Asia	Both	50-69 yea	Sexually tra	Rate	2006	13.4065	23.776	7.76099
DALYs (Dis	Caribbean	Both	50-69 yea	Sexually tra	Number	2015	2183.79	3715.09	1359.19
DALYs (Dis	Caribbean	Both	50-69 yea	Sexually tra	Rate	2015	28.1698	47.923	17.5328
DALYs (Dis	Tropical La	Both	50-69 yea	HIV/AIDS	Number	2007	100663	105065	96523.8
DALYs (Dis	Tropical La	Both	50-69 yea	HIV/AIDS	Rate	2007	362.755	378.619	347.839
DALYs (Dis	Central Eu	Both	50-69 yea	HIV/AIDS	Number	2007	5529.38	6273.03	5142.18
DALYs (Dis	Central Eu	Both	50-69 yea	HIV/AIDS	Rate	2007	19.7265	22.3795	18.3451
DALYs (Dis	Central Su	Both	50-69 yea	HIV/AIDS	Number	2007	359060	434780	294282
DALYs (Dis	Central Su	Both	50-69 yea	HIV/AIDS	Rate	2007	5603.12	6784.72	4592.27
DALYs (Dis	Western E	Both	50-69 yea	Sexually tra	Number	2008	7514.2	15065.5	3766.41
DALYs (Dis	Western E	Both	50-69 yea	Sexually tra	Rate	2008	7.74028	15.5188	3.87974
DALYs (Dis	Central Su	Both	50-69 yea	Sexually tra	Number	2008	4167.26	7855.04	1876.54
DALYs (Dis	Central Su	Both	50-69 yea	Sexually tra	Rate	2008	62.712	118.208	28.2395
DALYs (Dis	Central As	Both	50-69 yea	Sexually tra	Number	2015	2217.82	3833.92	1360.56
DALYs (Dis	Central As	Both	50-69 yea	Sexually tra	Rate	2015	16.938	29.2805	10.3909
DALYs (Dis	Caribbean	Both	50-69 yea	HIV/AIDS	Number	2007	101506	123557	83079
DALYs (Dis	Caribbean	Both	50-69 yea	HIV/AIDS	Rate	2007	1611.66	1961.77	1319.08
DALYs (Dis	Western S	Both	50-69 yea	Sexually tra	Number	2007	5509.59	10353.3	3036.23
DALYs (Dis	Western S	Both	50-69 yea	Sexually tra	Rate	2007	23.7648	44.6574	13.0963
DALYs (Dis	Southeast	Both	50-69 yea	Sexually tra	Number	2008	13333.5	24387.9	7538.95
DALYs (Dis	Southeast	Both	50-69 yea	Sexually tra	Rate	2008	17.3435	31.7225	9.80627
DALYs (Dis	Central Su	Both	50-69 yea	HIV/AIDS	Number	2008	353697	429846	290059
DALYs (Dis	Central Su	Both	50-69 yea	HIV/AIDS	Rate	2008	5322.69	6468.65	4365.03
DALYs (Dis	Eastern Su	Both	50-69 yea	HIV/AIDS	Number	2015	1763019	2128151	1538246
DALYs (Dis	Eastern Su	Both	50-69 yea	HIV/AIDS	Rate	2015	6915.14	8347.31	6033.51
DALYs (Dis	Eastern Su	Both	50-69 yea	Sexually tra	Number	2015	16935.6	26127.3	9510
DALYs (Dis	Eastern Su	Both	50-69 yea	Sexually tra	Rate	2015	66.4271	102.48	37.3014
DALYs (Dis	High-incor	Both	50-69 yea	HIV/AIDS	Number	2006	182097	204646	166531
DALYs (Dis	High-incor	Both	50-69 yea	HIV/AIDS	Rate	2006	263.406	296.023	240.889
DALYs (Dis	Caribbean	Both	50-69 yea	Sexually tra	Number	2008	1763.07	3024.69	1076.77
DALYs (Dis	Caribbean	Both	50-69 yea	Sexually tra	Rate	2008	27.3821	46.9761	16.7232
DALYs (Dis	Oceania	Both	50-69 yea	Sexually tra	Number	2015	298.533	550.057	157.736
DALYs (Dis	Oceania	Both	50-69 yea	Sexually tra	Rate	2015	25.0195	46.0993	13.2196
DALYs (Dis	Central Su	Both	50-69 yea	Sexually tra	Number	2007	4110.5	7911.06	1829.58
DALYs (Dis	Central Su	Both	50-69 yea	Sexually tra	Rate	2007	64.1443	123.452	28.5506
DALYs (Dis	Western S	Both	50-69 yea	HIV/AIDS	Number	2015	1245465	1403393	1119540
DALYs (Dis	Western S	Both	50-69 yea	HIV/AIDS	Rate	2015	4131.8	4655.72	3714.05
DALYs (Dis	Australasia	Both	50-69 yea	Sexually tra	Number	2007	421.262	832.597	208.595
DALYs (Dis	Australasia	Both	50-69 yea	Sexually tra	Rate	2007	7.96329	15.7389	3.94315
DALYs (Dis	Tropical La	Both	50-69 yea	Sexually tra	Number	2007	7712.64	14888.1	4142.97
DALYs (Dis	Tropical La	Both	50-69 yea	Sexually tra	Rate	2007	27.7937	53.6516	14.9299
DALYs (Dis	Southern L	Both	50-69 yea	Sexually tra	Number	2008	1534.94	2899.7	864.971
DALYs (Dis	Southern L	Both	50-69 yea	Sexually tra	Rate	2008	15.2245	28.7611	8.57934
DALYs (Dis	Western E	Both	50-69 yea	HIV/AIDS	Number	2008	56768.2	62812.6	52324.9
DALYs (Dis	Western E	Both	50-69 yea	HIV/AIDS	Rate	2008	58.4762	64.7025	53.8993
DALYs (Dis	North Afric	Both	50-69 yea	Sexually tra	Number	2015	7364.18	14204	3861.54
DALYs (Dis	North Afric	Both	50-69 yea	Sexually tra	Rate	2015	10.8004	20.8318	5.66337
DALYs (Dis	Tropical La	Both	50-69 yea	HIV/AIDS	Number	2006	97436.2	101575	93653.6
DALYs (Dis	Tropical La	Both	50-69 yea	HIV/AIDS	Rate	2006	364.217	379.687	350.077
DALYs (Dis	High-incor	Both	50-69 yea	Sexually tra	Number	2007	4441.2	8551.02	2324.46
DALYs (Dis	High-incor	Both	50-69 yea	Sexually tra	Rate	2007	9.77359	18.8179	5.11535
DALYs (Dis	Western S	Both	50-69 yea	HIV/AIDS	Number	2006	1292053	1435027	1154759
DALYs (Dis	Western S	Both	50-69 yea	HIV/AIDS	Rate	2006	5740.1	6375.29	5130.16
DALYs (Dis	Southern L	Both	50-69 yea	HIV/AIDS	Number	2007	16330.4	17506.7	15533.5
DALYs (Dis	Southern L	Both	50-69 yea	HIV/AIDS	Rate	2007	165.885	177.834	157.791
DALYs (Dis	Southern L	Both	50-69 yea	Sexually tra	Number	2007	1514.5	2851.1	853.27
DALYs (Dis	Southern L	Both	50-69 yea	Sexually tra	Rate	2007	15.3844	28.9616	8.66757
DALYs (Dis	Tropical La	Both	50-69 yea	Sexually tra	Number	2006	7409.67	14207.4	4002.09
DALYs (Dis	Tropical La	Both	50-69 yea	Sexually tra	Rate	2006	27.6974	53.1071	14.9598
DALYs (Dis	Australasia	Both	50-69 yea	HIV/AIDS	Number	2006	2008.95	2188.1	1865.42
DALYs (Dis	Australasia	Both	50-69 yea	HIV/AIDS	Rate	2006	39.0487	42.5309	36.259
DALYs (Dis	High-incor	Both	50-69 yea	HIV/AIDS	Number	2008	8207.88	9994.89	7055.86
DALYs (Dis	High-incor	Both	50-69 yea	HIV/AIDS	Rate	2008	17.9084	21.8074	15.3948
DALYs (Dis	Southeast	Both	50-69 yea	HIV/AIDS	Number	2007	321218	415312	267048
DALYs (Dis	Southeast	Both	50-69 yea	HIV/AIDS	Rate	2007	434.392	561.637	361.136
DALYs (Dis	Andean La	Both	50-69 yea	HIV/AIDS	Number	2009	15812.3	24749.2	12102.2
DALYs (Dis	Andean La	Both	50-69 yea	HIV/AIDS	Rate	2009	241.371	377.792	184.737
DALYs (Dis	High-incor	Both	50-69 yea	Sexually tra	Number	2009	4457.41	8655.18	2314.32
DALYs (Dis	High-incor	Both	50-69 yea	Sexually tra	Rate	2009	9.60923	18.6587	4.98919
DALYs (Dis	Caribbean	Both	50-69 yea	HIV/AIDS	Number	2006	110254	131229	91563.1
DALYs (Dis	Caribbean	Both	50-69 yea	HIV/AIDS	Rate	2006	1790.32	2130.92	1486.82
DALYs (Dis	Southern S	Both	50-69 yea	HIV/AIDS	Number	2008	2269474	2587581	1953717
DALYs (Dis	Southern S	Both	50-69 yea	HIV/AIDS	Rate	2008	30626.4	34919.3	26365.3
DALYs (Dis	Oceania	Both	50-69 yea	HIV/AIDS	Number	2016	25320.8	67552.8	10424
DALYs (Dis	Oceania	Both	50-69 yea	HIV/AIDS	Rate	2016	2040.14	5442.84	839.878
DALYs (Dis	Central As	Both	50-69 yea	HIV/AIDS	Number	2016	5674.11	6163.02	5249.09
DALYs (Dis	Central As	Both	50-69 yea	HIV/AIDS	Rate	2016	41.6059	45.1909	38.4894
DALYs (Dis	Central As	Both	50-69 yea	Sexually tra	Number	2008	1877.62	3003.07	1243.98
DALYs (Dis	Central As	Both	50-69 yea	Sexually tra	Rate	2008	19.7577	31.6006	13.0901
DALYs (Dis	Central Eu	Both	50-69 yea	HIV/AIDS	Number	2016	5300	6497.9	4662.92
DALYs (Dis	Central Eu	Both	50-69 yea	HIV/AIDS	Rate	2016	17.7121	21.7153	15.583
DALYs (Dis	East Asia	Both	50-69 yea	Sexually tra	Number	2016	43276.9	80129.1	23975
DALYs (Dis	East Asia	Both	50-69 yea	Sexually tra	Rate	2016	12.524	23.1888	6.93818
DALYs (Dis	Western E	Both	50-69 yea	Sexually tra	Number	2007	7440.49	14848.8	3722.4
DALYs (Dis	Western E	Both	50-69 yea	Sexually tra	Rate	2007	7.7598	15.4861	3.88214
DALYs (Dis	Oceania	Both	50-69 yea	HIV/AIDS	Number	2007	15790.1	37263.7	4715.6
DALYs (Dis	Oceania	Both	50-69 yea	HIV/AIDS	Rate	2007	1807.64	4265.91	539.836
DALYs (Dis	Central Eu	Both	50-69 yea	Sexually tra	Number	2008	2732.13	5145.04	1471.83
DALYs (Dis	Central Eu	Both	50-69 yea	Sexually tra	Rate	2008	9.62368	18.1229	5.18438
DALYs (Dis	Oceania	Both	50-69 yea	Sexually tra	Number	2008	233.246	429.705	122.601
DALYs (Dis	Oceania	Both	50-69 yea	Sexually tra	Rate	2008	25.7302	47.4023	13.5245
DALYs (Dis	High-incor	Both	50-69 yea	Sexually tra	Number	2016	4693.29	9137.89	2389.74
DALYs (Dis	High-incor	Both	50-69 yea	Sexually tra	Rate	2016	9.51986	18.5353	4.84735
DALYs (Dis	North Afric	Both	50-69 yea	Sexually tra	Number	2008	5657.57	10921.6	2903.33
DALYs (Dis	North Afric	Both	50-69 yea	Sexually tra	Rate	2008	10.9521	21.1425	5.62037
DALYs (Dis	Eastern Eu	Both	50-69 yea	HIV/AIDS	Number	2016	112780	116743	109223
DALYs (Dis	Eastern Eu	Both	50-69 yea	HIV/AIDS	Rate	2016	205.433	212.65	198.953
DALYs (Dis	Southeast	Both	50-69 yea	HIV/AIDS	Number	2016	389045	514312	321918
DALYs (Dis	Southeast	Both	50-69 yea	HIV/AIDS	Rate	2016	372.816	492.857	308.49
DALYs (Dis	Southern S	Both	50-69 yea	Sexually tra	Number	2008	3955.42	6413.86	2594.74
DALYs (Dis	Southern S	Both	50-69 yea	Sexually tra	Rate	2008	53.3781	86.5547	35.0159
DALYs (Dis	South Asia	Both	50-69 yea	HIV/AIDS	Number	2007	612288	713891	513963
DALYs (Dis	South Asia	Both	50-69 yea	HIV/AIDS	Rate	2007	370.006	431.405	310.588
DALYs (Dis	South Asia	Both	50-69 yea	Sexually tra	Number	2007	43865.9	59146.3	32863.4
DALYs (Dis	South Asia	Both	50-69 yea	Sexually tra	Rate	2007	26.5082	35.7421	19.8593
DALYs (Dis	Caribbean	Both	50-69 yea	Sexually tra	Number	2006	1691.15	2924.75	1029.6
DALYs (Dis	Caribbean	Both	50-69 yea	Sexually tra	Rate	2006	27.4612	47.4925	16.7188
DALYs (Dis	East Asia	Both	50-69 yea	Sexually tra	Number	2009	33579.5	60021.1	19359.4
DALYs (Dis	East Asia	Both	50-69 yea	Sexually tra	Rate	2009	12.4726	22.2939	7.19073
DALYs (Dis	Central Su	Both	50-69 yea	Sexually tra	Number	2006	4071.34	7818.3	1781.17
DALYs (Dis	Central Su	Both	50-69 yea	Sexually tra	Rate	2006	65.7886	126.336	28.7819
DALYs (Dis	High-incor	Both	50-69 yea	Sexually tra	Number	2016	12648.7	24427.2	6485.66
DALYs (Dis	High-incor	Both	50-69 yea	Sexually tra	Rate	2016	14.0722	27.1764	7.21559
DALYs (Dis	Caribbean	Both	50-69 yea	HIV/AIDS	Number	2016	91497.6	107736	78513.8
DALYs (Dis	Caribbean	Both	50-69 yea	HIV/AIDS	Rate	2016	1149.68	1353.72	986.54
DALYs (Dis	Tropical La	Both	50-69 yea	Sexually tra	Number	2016	10309.4	19678	5478.38
DALYs (Dis	Tropical La	Both	50-69 yea	Sexually tra	Rate	2016	27.0723	51.6743	14.3862
DALYs (Dis	North Afric	Both	50-69 yea	HIV/AIDS	Number	2016	53600.6	105175	31938.2
DALYs (Dis	North Afric	Both	50-69 yea	HIV/AIDS	Rate	2016	75.5175	148.181	44.9974
DALYs (Dis	Eastern Eu	Both	50-69 yea	HIV/AIDS	Number	2009	62465.1	67883.6	59627.2
DALYs (Dis	Eastern Eu	Both	50-69 yea	HIV/AIDS	Rate	2009	128.953	140.139	123.095
DALYs (Dis	South Asia	Both	50-69 yea	HIV/AIDS	Number	2016	364796	551841	266771
DALYs (Dis	South Asia	Both	50-69 yea	HIV/AIDS	Rate	2016	163.355	247.114	119.46
DALYs (Dis	South Asia	Both	50-69 yea	Sexually tra	Number	2016	58022.9	80343.3	42218.3
DALYs (Dis	South Asia	Both	50-69 yea	Sexually tra	Rate	2016	25.9826	35.9777	18.9054
DALYs (Dis	Eastern Eu	Both	50-69 yea	Sexually tra	Number	2009	8216.75	14216.1	5324.36
DALYs (Dis	Eastern Eu	Both	50-69 yea	Sexually tra	Rate	2009	16.9627	29.3479	10.9916
DALYs (Dis	Central Lat	Both	50-69 yea	HIV/AIDS	Number	2016	98025.1	104344	92370.6
DALYs (Dis	Central Lat	Both	50-69 yea	HIV/AIDS	Rate	2016	269.171	286.523	253.644
DALYs (Dis	Central Lat	Both	50-69 yea	Sexually tra	Number	2016	8990.37	17021.1	4930.6
DALYs (Dis	Central Lat	Both	50-69 yea	Sexually tra	Rate	2016	24.687	46.7389	13.5392
DALYs (Dis	Central As	Both	50-69 yea	HIV/AIDS	Number	2007	3424.03	3888.41	3094.98
DALYs (Dis	Central As	Both	50-69 yea	HIV/AIDS	Rate	2007	37.2235	42.2718	33.6463
DALYs (Dis	Andean La	Both	50-69 yea	Sexually tra	Number	2008	1290.65	2477.68	692.726
DALYs (Dis	Andean La	Both	50-69 yea	Sexually tra	Rate	2008	20.3902	39.1433	10.9439
DALYs (Dis	Southern L	Both	50-69 yea	HIV/AIDS	Number	2008	16998.1	18277.4	16157.4
DALYs (Dis	Southern L	Both	50-69 yea	HIV/AIDS	Rate	2008	168.598	181.287	160.26
DALYs (Dis	Southern S	Both	50-69 yea	Sexually tra	Number	2007	3871.27	6291.11	2551.91
DALYs (Dis	Southern S	Both	50-69 yea	Sexually tra	Rate	2007	53.688	87.247	35.3907
DALYs (Dis	North Afric	Both	50-69 yea	HIV/AIDS	Number	2007	33081.2	59345.2	19064.5
DALYs (Dis	North Afric	Both	50-69 yea	HIV/AIDS	Rate	2007	66.4092	119.133	38.2713
DALYs (Dis	Central Lat	Both	50-69 yea	HIV/AIDS	Number	2006	74066.1	76894.5	71780.6
DALYs (Dis	Central Lat	Both	50-69 yea	HIV/AIDS	Rate	2006	293.135	304.329	284.089
DALYs (Dis	Central Lat	Both	50-69 yea	Sexually tra	Number	2006	5937.36	11364	3226.71
DALYs (Dis	Central Lat	Both	50-69 yea	Sexually tra	Rate	2006	23.4986	44.9759	12.7705
DALYs (Dis	High-incor	Both	50-69 yea	HIV/AIDS	Number	2008	187328	213524	168085
DALYs (Dis	High-incor	Both	50-69 yea	HIV/AIDS	Rate	2008	253.072	288.462	227.075
DALYs (Dis	Western E	Both	50-69 yea	HIV/AIDS	Number	2007	56684.6	62385.7	52552.6
DALYs (Dis	Western E	Both	50-69 yea	HIV/AIDS	Rate	2007	59.1172	65.063	54.8079
DALYs (Dis	High-incor	Both	50-69 yea	Sexually tra	Number	2009	10988	21577.7	5662.19
DALYs (Dis	High-incor	Both	50-69 yea	Sexually tra	Rate	2009	14.3829	28.2446	7.41164
DALYs (Dis	East Asia	Both	50-69 yea	HIV/AIDS	Number	2017	358546	422547	297304
DALYs (Dis	East Asia	Both	50-69 yea	HIV/AIDS	Rate	2017	100.041	117.899	82.9537
DALYs (Dis	High-incor	Both	50-69 yea	HIV/AIDS	Number	2007	7958.72	9486.61	6857.2
DALYs (Dis	High-incor	Both	50-69 yea	HIV/AIDS	Rate	2007	17.5145	20.8768	15.0904
DALYs (Dis	Southern S	Both	50-69 yea	HIV/AIDS	Number	2007	2406257	2678325	2144680
DALYs (Dis	Southern S	Both	50-69 yea	HIV/AIDS	Rate	2007	33370.7	37143.8	29743
DALYs (Dis	Eastern Su	Both	50-69 yea	HIV/AIDS	Number	2009	2226287	2704404	1909314
DALYs (Dis	Eastern Su	Both	50-69 yea	HIV/AIDS	Rate	2009	10678	12971.3	9157.73
DALYs (Dis	Eastern Su	Both	50-69 yea	Sexually tra	Number	2009	16724.4	25962.4	8884.38
DALYs (Dis	Eastern Su	Both	50-69 yea	Sexually tra	Rate	2009	80.2161	124.525	42.6126
DALYs (Dis	Western E	Both	50-69 yea	Sexually tra	Number	2017	8129.38	16620.9	4005.52
DALYs (Dis	Western E	Both	50-69 yea	Sexually tra	Rate	2017	7.30645	14.9384	3.60004
DALYs (Dis	East Asia	Both	50-69 yea	Sexually tra	Number	2010	33794.7	60660.9	19483.6
DALYs (Dis	East Asia	Both	50-69 yea	Sexually tra	Rate	2010	12.2758	22.0348	7.07732
DALYs (Dis	Australasia	Both	50-69 yea	HIV/AIDS	Number	2010	1584.33	1812.35	1394.62
DALYs (Dis	Australasia	Both	50-69 yea	HIV/AIDS	Rate	2010	27.6583	31.6389	24.3464
DALYs (Dis	East Asia	Both	50-69 yea	HIV/AIDS	Number	2010	141237	162751	127201
DALYs (Dis	East Asia	Both	50-69 yea	HIV/AIDS	Rate	2010	51.3037	59.1186	46.2051
DALYs (Dis	Australasia	Both	50-69 yea	Sexually tra	Number	2009	468.784	957.203	217.957
DALYs (Dis	Australasia	Both	50-69 yea	Sexually tra	Rate	2009	8.39925	17.1503	3.90515
DALYs (Dis	High-incor	Both	50-69 yea	HIV/AIDS	Number	2017	9549.06	12216.7	7632.2
DALYs (Dis	High-incor	Both	50-69 yea	HIV/AIDS	Rate	2017	19.3371	24.7392	15.4554
DALYs (Dis	High-incor	Both	50-69 yea	HIV/AIDS	Number	2017	179862	224126	147653
DALYs (Dis	High-incor	Both	50-69 yea	HIV/AIDS	Rate	2017	199.177	248.194	163.509
DALYs (Dis	Southern L	Both	50-69 yea	Sexually tra	Number	2017	1794.36	3397.21	1004.23
DALYs (Dis	Southern L	Both	50-69 yea	Sexually tra	Rate	2017	14.7526	27.9307	8.25641
DALYs (Dis	Tropical La	Both	50-69 yea	HIV/AIDS	Number	2017	170805	182398	160216
DALYs (Dis	Tropical La	Both	50-69 yea	HIV/AIDS	Rate	2017	435.721	465.295	408.71
DALYs (Dis	Andean La	Both	50-69 yea	HIV/AIDS	Number	2008	16300.2	24928	12622.4
DALYs (Dis	Andean La	Both	50-69 yea	HIV/AIDS	Rate	2008	257.516	393.821	199.413
DALYs (Dis	Andean La	Both	50-69 yea	Sexually tra	Number	2017	1693.35	3339.29	863.409
DALYs (Dis	Andean La	Both	50-69 yea	Sexually tra	Rate	2017	19.4939	38.4419	9.93956
DALYs (Dis	Eastern Su	Both	50-69 yea	HIV/AIDS	Number	2008	2395649	2872677	2040733
DALYs (Dis	Eastern Su	Both	50-69 yea	HIV/AIDS	Rate	2008	11855.3	14216	10098.9
DALYs (Dis	Eastern Su	Both	50-69 yea	Sexually tra	Number	2008	16674	26074.1	8846.69
DALYs (Dis	Eastern Su	Both	50-69 yea	Sexually tra	Rate	2008	82.5142	129.032	43.7795
DALYs (Dis	Central Su	Both	50-69 yea	Sexually tra	Number	2017	4785.98	8988.14	2283.17
DALYs (Dis	Central Su	Both	50-69 yea	Sexually tra	Rate	2017	49.9864	93.8752	23.8462
DALYs (Dis	Western S	Both	50-69 yea	HIV/AIDS	Number	2010	1133585	1285107	1000756
DALYs (Dis	Western S	Both	50-69 yea	HIV/AIDS	Rate	2010	4454.97	5050.44	3932.95
DALYs (Dis	Southern S	Both	50-69 yea	Sexually tra	Number	2017	4323.39	7250.11	2728.44
DALYs (Dis	Southern S	Both	50-69 yea	Sexually tra	Rate	2017	45.539	76.3668	28.7391
DALYs (Dis	Central Lat	Both	50-69 yea	HIV/AIDS	Number	2009	84002.4	86688.1	81368.9
DALYs (Dis	Central Lat	Both	50-69 yea	HIV/AIDS	Rate	2009	296.277	305.749	286.988
DALYs (Dis	Central Lat	Both	50-69 yea	Sexually tra	Number	2009	6784.57	12875	3664.55
DALYs (Dis	Central Lat	Both	50-69 yea	Sexually tra	Rate	2009	23.9292	45.4103	12.9249
DALYs (Dis	Southeast	Both	50-69 yea	HIV/AIDS	Number	2009	319182	404316	265758
DALYs (Dis	Southeast	Both	50-69 yea	HIV/AIDS	Rate	2009	399.256	505.747	332.429
DALYs (Dis	Western S	Both	50-69 yea	Sexually tra	Number	2009	5792.2	10935.3	3129.87
DALYs (Dis	Western S	Both	50-69 yea	Sexually tra	Rate	2009	23.4961	44.3591	12.6964
DALYs (Dis	Southeast	Both	50-69 yea	Sexually tra	Number	2009	13667.1	25125.4	7670.88
DALYs (Dis	Southeast	Both	50-69 yea	Sexually tra	Rate	2009	17.0958	31.4286	9.59528
DALYs (Dis	Eastern Eu	Both	50-69 yea	Sexually tra	Number	2008	8328.35	14262.9	5441.52
DALYs (Dis	Eastern Eu	Both	50-69 yea	Sexually tra	Rate	2008	17.3405	29.697	11.3299
DALYs (Dis	Australasia	Both	50-69 yea	HIV/AIDS	Number	2009	1864.34	2074.05	1687.68
DALYs (Dis	Australasia	Both	50-69 yea	HIV/AIDS	Rate	2009	33.4035	37.1609	30.2383
DALYs (Dis	Tropical La	Both	50-69 yea	HIV/AIDS	Number	2010	126861	133114	121053
DALYs (Dis	Tropical La	Both	50-69 yea	HIV/AIDS	Rate	2010	409.826	430.028	391.064
DALYs (Dis	Central Lat	Both	50-69 yea	HIV/AIDS	Number	2010	87665.9	90722.4	84697.2
DALYs (Dis	Central Lat	Both	50-69 yea	HIV/AIDS	Rate	2010	297.602	307.978	287.524
DALYs (Dis	Central Lat	Both	50-69 yea	Sexually tra	Number	2010	7066.89	13369	3827.59
DALYs (Dis	Central Lat	Both	50-69 yea	Sexually tra	Rate	2010	23.9902	45.3841	12.9936
DALYs (Dis	Australasia	Both	50-69 yea	Sexually tra	Number	2008	443.532	887.746	212.841
DALYs (Dis	Australasia	Both	50-69 yea	Sexually tra	Rate	2008	8.15905	16.3306	3.91534
DALYs (Dis	East Asia	Both	50-69 yea	HIV/AIDS	Number	2008	139726	159129	128357
DALYs (Dis	East Asia	Both	50-69 yea	HIV/AIDS	Rate	2008	53.0322	60.3965	48.7173
DALYs (Dis	Western S	Both	50-69 yea	HIV/AIDS	Number	2009	1142225	1303879	1001301
DALYs (Dis	Western S	Both	50-69 yea	HIV/AIDS	Rate	2009	4633.45	5289.2	4061.79
DALYs (Dis	North Afric	Both	50-69 yea	Sexually tra	Number	2009	5819.97	11308.1	3002.03
DALYs (Dis	North Afric	Both	50-69 yea	Sexually tra	Rate	2009	10.8552	21.0915	5.59929
DALYs (Dis	Tropical La	Both	50-69 yea	Sexually tra	Number	2009	8329.17	16267.2	4426.88
DALYs (Dis	Tropical La	Both	50-69 yea	Sexually tra	Rate	2009	27.9033	54.4963	14.8304
DALYs (Dis	Central As	Both	50-69 yea	Sexually tra	Number	2010	2000.08	3293.8	1300.64
DALYs (Dis	Central As	Both	50-69 yea	Sexually tra	Rate	2010	19.412	31.9684	12.6235
DALYs (Dis	Central Su	Both	50-69 yea	Sexually tra	Number	2010	4279.13	7831.83	1995.93
DALYs (Dis	Central Su	Both	50-69 yea	Sexually tra	Rate	2010	59.6481	109.17	27.8219
DALYs (Dis	Tropical La	Both	50-69 yea	Sexually tra	Number	2010	8645.24	17029.7	4584.02
DALYs (Dis	Tropical La	Both	50-69 yea	Sexually tra	Rate	2010	27.9286	55.0147	14.8088
DALYs (Dis	Southeast	Both	50-69 yea	Sexually tra	Number	2010	14057.8	26064	7816.44
DALYs (Dis	Southeast	Both	50-69 yea	Sexually tra	Rate	2010	16.9004	31.3343	9.39699
DALYs (Dis	Caribbean	Both	50-69 yea	HIV/AIDS	Number	2010	94239.5	115065	78889.1
DALYs (Dis	Caribbean	Both	50-69 yea	HIV/AIDS	Rate	2010	1396.54	1705.15	1169.06
DALYs (Dis	Southeast	Both	50-69 yea	HIV/AIDS	Number	2010	326298	412327	271455
DALYs (Dis	Southeast	Both	50-69 yea	HIV/AIDS	Rate	2010	392.278	495.703	326.345
DALYs (Dis	Caribbean	Both	50-69 yea	HIV/AIDS	Number	2009	92456.3	114155	75699.1
DALYs (Dis	Caribbean	Both	50-69 yea	HIV/AIDS	Rate	2009	1402.18	1731.25	1148.04
DALYs (Dis	Central Eu	Both	50-69 yea	Sexually tra	Number	2010	2724.46	5132.79	1433.38
DALYs (Dis	Central Eu	Both	50-69 yea	Sexually tra	Rate	2010	9.40792	17.7242	4.94964
DALYs (Dis	Central Eu	Both	50-69 yea	HIV/AIDS	Number	2011	5796.82	7340.45	5109.15
DALYs (Dis	Central Eu	Both	50-69 yea	HIV/AIDS	Rate	2011	19.8543	25.1413	17.499
DALYs (Dis	Central Eu	Both	50-69 yea	HIV/AIDS	Number	2009	5139.97	6312.97	4671.37
DALYs (Dis	Central Eu	Both	50-69 yea	HIV/AIDS	Rate	2009	17.9074	21.994	16.2748
DALYs (Dis	Oceania	Both	50-69 yea	HIV/AIDS	Number	2011	19725.1	48200.3	6764.33
DALYs (Dis	Oceania	Both	50-69 yea	HIV/AIDS	Rate	2011	1936.84	4732.87	664.201
DALYs (Dis	Oceania	Both	50-69 yea	HIV/AIDS	Number	2010	18343.3	45176.1	6097.03
DALYs (Dis	Oceania	Both	50-69 yea	HIV/AIDS	Rate	2010	1876.8	4622.22	623.821
DALYs (Dis	Central Eu	Both	50-69 yea	HIV/AIDS	Number	2010	5692.88	7070.5	5137.92
DALYs (Dis	Central Eu	Both	50-69 yea	HIV/AIDS	Rate	2010	19.6583	24.4153	17.7419
DALYs (Dis	Oceania	Both	50-69 yea	HIV/AIDS	Number	2009	16978.4	42471	5388.8
DALYs (Dis	Oceania	Both	50-69 yea	HIV/AIDS	Rate	2009	1804.14	4512.99	572.617
DALYs (Dis	Caribbean	Both	50-69 yea	Sexually tra	Number	2010	1857.12	3213.74	1124.4
DALYs (Dis	Caribbean	Both	50-69 yea	Sexually tra	Rate	2010	27.5207	47.6244	16.6625
DALYs (Dis	Western S	Both	50-69 yea	HIV/AIDS	Number	2008	1195518	1347346	1052408
DALYs (Dis	Western S	Both	50-69 yea	HIV/AIDS	Rate	2008	5003.3	5638.7	4404.37
DALYs (Dis	Western E	Both	50-69 yea	HIV/AIDS	Number	2018	65636.1	76720.1	56924.4
DALYs (Dis	Western E	Both	50-69 yea	HIV/AIDS	Rate	2018	58.4453	68.3149	50.688
DALYs (Dis	Western S	Both	50-69 yea	Sexually tra	Number	2008	5630.98	10626	3036.77
DALYs (Dis	Western S	Both	50-69 yea	Sexually tra	Rate	2008	23.5659	44.4702	12.709
DALYs (Dis	Australasia	Both	50-69 yea	HIV/AIDS	Number	2008	1721.68	1905.97	1570.19
DALYs (Dis	Australasia	Both	50-69 yea	HIV/AIDS	Rate	2008	31.6714	35.0615	28.8846
DALYs (Dis	South Asia	Both	50-69 yea	HIV/AIDS	Number	2011	507093	667852	394548
DALYs (Dis	South Asia	Both	50-69 yea	HIV/AIDS	Rate	2011	267.922	352.859	208.459
DALYs (Dis	South Asia	Both	50-69 yea	Sexually tra	Number	2011	46144.9	62460.9	34375.4
DALYs (Dis	South Asia	Both	50-69 yea	Sexually tra	Rate	2011	24.3806	33.0012	18.1622
DALYs (Dis	Central As	Both	50-69 yea	HIV/AIDS	Number	2011	4612.84	5248.15	4195.65
DALYs (Dis	Central As	Both	50-69 yea	HIV/AIDS	Rate	2011	42.7326	48.618	38.8678
DALYs (Dis	Australasia	Both	50-69 yea	Sexually tra	Number	2018	553.279	1135.98	259.917
DALYs (Dis	Australasia	Both	50-69 yea	Sexually tra	Rate	2018	8.31509	17.0724	3.90622
DALYs (Dis	Southern L	Both	50-69 yea	HIV/AIDS	Number	2018	26821.3	30773.6	24381
DALYs (Dis	Southern L	Both	50-69 yea	HIV/AIDS	Rate	2018	217.087	249.076	197.336
DALYs (Dis	Oceania	Both	50-69 yea	Sexually tra	Number	2010	250.069	464.14	133.142
DALYs (Dis	Oceania	Both	50-69 yea	Sexually tra	Rate	2010	25.5859	47.4888	13.6225
DALYs (Dis	Western E	Both	50-69 yea	HIV/AIDS	Number	2009	57035.7	63236.1	52333.7
DALYs (Dis	Western E	Both	50-69 yea	HIV/AIDS	Rate	2009	58.0368	64.346	53.2523
DALYs (Dis	Oceania	Both	50-69 yea	Sexually tra	Number	2009	240.758	447.065	126.336
DALYs (Dis	Oceania	Both	50-69 yea	Sexually tra	Rate	2009	25.5831	47.5053	13.4245
DALYs (Dis	Western E	Both	50-69 yea	Sexually tra	Number	2010	7647.2	15489.3	3818.69
DALYs (Dis	Western E	Both	50-69 yea	Sexually tra	Rate	2010	7.67813	15.552	3.83413
DALYs (Dis	Andean La	Both	50-69 yea	HIV/AIDS	Number	2018	21701.5	38420.3	14530.5
DALYs (Dis	Andean La	Both	50-69 yea	HIV/AIDS	Rate	2018	242.197	428.784	162.165
DALYs (Dis	Tropical La	Both	50-69 yea	HIV/AIDS	Number	2008	110504	115599	105687
DALYs (Dis	Tropical La	Both	50-69 yea	HIV/AIDS	Rate	2008	383.937	401.641	367.202
DALYs (Dis	North Afric	Both	50-69 yea	Sexually tra	Number	2010	6004.98	11739.7	3057.89
DALYs (Dis	North Afric	Both	50-69 yea	Sexually tra	Rate	2010	10.7809	21.0765	5.4899
DALYs (Dis	Southern L	Both	50-69 yea	Sexually tra	Number	2011	1626.93	3082.1	920.218
DALYs (Dis	Southern L	Both	50-69 yea	Sexually tra	Rate	2011	15.0515	28.514	8.51341
DALYs (Dis	Southern L	Both	50-69 yea	HIV/AIDS	Number	2009	18228.5	19691.8	17325.5
DALYs (Dis	Southern L	Both	50-69 yea	HIV/AIDS	Rate	2009	176.591	190.767	167.842
DALYs (Dis	High-incor	Both	50-69 yea	Sexually tra	Number	2011	4499.87	8828.59	2338.03
DALYs (Dis	High-incor	Both	50-69 yea	Sexually tra	Rate	2011	9.56924	18.7745	4.97196
DALYs (Dis	Southern S	Both	50-69 yea	HIV/AIDS	Number	2018	1649211	1925955	1436351
DALYs (Dis	Southern S	Both	50-69 yea	HIV/AIDS	Rate	2018	16929.7	19770.5	14744.6
DALYs (Dis	Central Su	Both	50-69 yea	HIV/AIDS	Number	2018	280507	346854	228300
DALYs (Dis	Central Su	Both	50-69 yea	HIV/AIDS	Rate	2018	2813.89	3479.44	2290.18
DALYs (Dis	Central As	Both	50-69 yea	HIV/AIDS	Number	2010	4107.82	4760.49	3710.5
DALYs (Dis	Central As	Both	50-69 yea	HIV/AIDS	Rate	2010	39.869	46.2036	36.0127
DALYs (Dis	Central Eu	Both	50-69 yea	Sexually tra	Number	2009	2742.7	5119.2	1463.66
DALYs (Dis	Central Eu	Both	50-69 yea	Sexually tra	Rate	2009	9.55542	17.835	5.09931
DALYs (Dis	Southern S	Both	50-69 yea	Sexually tra	Number	2011	4112.51	6815.38	2684.64
DALYs (Dis	Southern S	Both	50-69 yea	Sexually tra	Rate	2011	51.1249	84.7257	33.3743
DALYs (Dis	North Afric	Both	50-69 yea	HIV/AIDS	Number	2011	41512.2	80551.4	23982.3
DALYs (Dis	North Afric	Both	50-69 yea	HIV/AIDS	Rate	2011	71.6537	139.039	41.3956
DALYs (Dis	Western S	Both	50-69 yea	Sexually tra	Number	2018	7907.72	15513.8	3976.81
DALYs (Dis	Western S	Both	50-69 yea	Sexually tra	Rate	2018	23.3924	45.8925	11.7641
DALYs (Dis	Central As	Both	50-69 yea	Sexually tra	Number	2009	1930.68	3166.15	1274.46
DALYs (Dis	Central As	Both	50-69 yea	Sexually tra	Rate	2009	19.5572	32.0721	12.9099
DALYs (Dis	Central Su	Both	50-69 yea	HIV/AIDS	Number	2009	347180	425672	282633
DALYs (Dis	Central Su	Both	50-69 yea	HIV/AIDS	Rate	2009	5031.32	6168.83	4095.91
DALYs (Dis	High-incor	Both	50-69 yea	Sexually tra	Number	2010	4478.42	8708.06	2348.59
DALYs (Dis	High-incor	Both	50-69 yea	Sexually tra	Rate	2010	9.57268	18.6136	5.02013
DALYs (Dis	East Asia	Both	50-69 yea	Sexually tra	Number	2011	34586.8	62205.2	19660.2
DALYs (Dis	East Asia	Both	50-69 yea	Sexually tra	Rate	2011	12.2309	21.9975	6.9524
DALYs (Dis	Southern S	Both	50-69 yea	HIV/AIDS	Number	2009	2103305	2467433	1761805
DALYs (Dis	Southern S	Both	50-69 yea	HIV/AIDS	Rate	2009	27623.3	32405.5	23138.3
DALYs (Dis	Caribbean	Both	50-69 yea	Sexually tra	Number	2009	1826.53	3172.09	1116.88
DALYs (Dis	Caribbean	Both	50-69 yea	Sexually tra	Rate	2009	27.7008	48.1074	16.9384
DALYs (Dis	South Asia	Both	50-69 yea	HIV/AIDS	Number	2010	539981	693141	426549
DALYs (Dis	South Asia	Both	50-69 yea	HIV/AIDS	Rate	2010	295.161	378.881	233.157
DALYs (Dis	South Asia	Both	50-69 yea	Sexually tra	Number	2010	44964.3	61047.2	33522.7
DALYs (Dis	South Asia	Both	50-69 yea	Sexually tra	Rate	2010	24.5781	33.3693	18.324
DALYs (Dis	South Asia	Both	50-69 yea	HIV/AIDS	Number	2009	575968	705491	465208
DALYs (Dis	South Asia	Both	50-69 yea	HIV/AIDS	Rate	2009	325.702	398.946	263.069
DALYs (Dis	South Asia	Both	50-69 yea	Sexually tra	Number	2009	43708.9	58731	32702.4
DALYs (Dis	South Asia	Both	50-69 yea	Sexually tra	Rate	2009	24.7168	33.2116	18.4928
DALYs (Dis	North Afric	Both	50-69 yea	HIV/AIDS	Number	2009	35769.1	68243.9	20056.5
DALYs (Dis	North Afric	Both	50-69 yea	HIV/AIDS	Rate	2009	66.7154	127.286	37.4087
DALYs (Dis	Eastern Eu	Both	50-69 yea	Sexually tra	Number	2019	7960.89	13952.8	4814.62
DALYs (Dis	Eastern Eu	Both	50-69 yea	Sexually tra	Rate	2019	14.6373	25.6542	8.8524
DALYs (Dis	Central As	Both	50-69 yea	Sexually tra	Number	2019	2357.93	4080.53	1435.18
DALYs (Dis	Central As	Both	50-69 yea	Sexually tra	Rate	2019	15.7185	27.2017	9.56722
DALYs (Dis	Oceania	Both	50-69 yea	Sexually tra	Number	2019	341.176	634.641	178.022
DALYs (Dis	Oceania	Both	50-69 yea	Sexually tra	Rate	2019	24.6343	45.8237	12.8539
DALYs (Dis	Southeast	Both	50-69 yea	Sexually tra	Number	2019	18011.8	34539.1	9629
DALYs (Dis	Southeast	Both	50-69 yea	Sexually tra	Rate	2019	15.6397	29.9904	8.36088
DALYs (Dis	Central Eu	Both	50-69 yea	Sexually tra	Number	2019	2552.97	4899.67	1308.07
DALYs (Dis	Central Eu	Both	50-69 yea	Sexually tra	Rate	2019	8.54058	16.3911	4.37596
DALYs (Dis	Southern S	Both	50-69 yea	Sexually tra	Number	2010	4122.01	6759.24	2670.45
DALYs (Dis	Southern S	Both	50-69 yea	Sexually tra	Rate	2010	52.6827	86.3886	34.1306
DALYs (Dis	Southern S	Both	50-69 yea	HIV/AIDS	Number	2011	1761975	2175703	1447829
DALYs (Dis	Southern S	Both	50-69 yea	HIV/AIDS	Rate	2011	21904.1	27047.4	17998.8
DALYs (Dis	Australasia	Both	50-69 yea	HIV/AIDS	Number	2019	1684.94	2038.01	1411.02
DALYs (Dis	Australasia	Both	50-69 yea	HIV/AIDS	Rate	2019	25.0012	30.24	20.9368
DALYs (Dis	Southern L	Both	50-69 yea	HIV/AIDS	Number	2011	19636.8	21457.2	18463.8
DALYs (Dis	Southern L	Both	50-69 yea	HIV/AIDS	Rate	2011	181.67	198.511	170.818
DALYs (Dis	East Asia	Both	50-69 yea	HIV/AIDS	Number	2012	146017	175594	127337
DALYs (Dis	East Asia	Both	50-69 yea	HIV/AIDS	Rate	2012	49.9616	60.0817	43.5699
DALYs (Dis	Caribbean	Both	50-69 yea	Sexually tra	Number	2019	2344.19	3954.87	1443.86
DALYs (Dis	Caribbean	Both	50-69 yea	Sexually tra	Rate	2019	27.6133	46.5862	17.0078
DALYs (Dis	High-incor	Both	50-69 yea	HIV/AIDS	Number	2012	181430	219303	153280
DALYs (Dis	High-incor	Both	50-69 yea	HIV/AIDS	Rate	2012	217.778	263.238	183.988
DALYs (Dis	Southern L	Both	50-69 yea	Sexually tra	Number	2010	1597.55	2998.64	896.023
DALYs (Dis	Southern L	Both	50-69 yea	Sexually tra	Rate	2010	15.1207	28.3819	8.48079
DALYs (Dis	High-incor	Both	50-69 yea	HIV/AIDS	Number	2010	9393.03	11295.6	7990.66
DALYs (Dis	High-incor	Both	50-69 yea	HIV/AIDS	Rate	2010	20.0777	24.1445	17.0801
DALYs (Dis	North Afric	Both	50-69 yea	Sexually tra	Number	2019	8487.7	16533	4373.67
DALYs (Dis	North Afric	Both	50-69 yea	Sexually tra	Rate	2019	10.6505	20.7459	5.48816
DALYs (Dis	Eastern Su	Both	50-69 yea	HIV/AIDS	Number	2019	1680090	2025823	1429214
DALYs (Dis	Eastern Su	Both	50-69 yea	HIV/AIDS	Rate	2019	5688.11	6858.62	4838.74
DALYs (Dis	Eastern Su	Both	50-69 yea	Sexually tra	Number	2019	17693.8	26961	10152.8
DALYs (Dis	Eastern Su	Both	50-69 yea	Sexually tra	Rate	2019	59.9042	91.2792	34.3734
DALYs (Dis	Central As	Both	50-69 yea	HIV/AIDS	Number	2009	3699.29	4325	3303.39
DALYs (Dis	Central As	Both	50-69 yea	HIV/AIDS	Rate	2009	37.4727	43.811	33.4623
DALYs (Dis	North Afric	Both	50-69 yea	HIV/AIDS	Number	2010	38125.6	74246.4	21771.5
DALYs (Dis	North Afric	Both	50-69 yea	HIV/AIDS	Rate	2010	68.4477	133.296	39.0869
DALYs (Dis	Western S	Both	50-69 yea	HIV/AIDS	Number	2019	1251372	1451087	1106090
DALYs (Dis	Western S	Both	50-69 yea	HIV/AIDS	Rate	2019	3559.89	4128.04	3146.59
DALYs (Dis	High-incor	Both	50-69 yea	HIV/AIDS	Number	2011	9249.85	11304.3	7833.04
DALYs (Dis	High-incor	Both	50-69 yea	HIV/AIDS	Rate	2011	19.6704	24.0392	16.6574
DALYs (Dis	Australasia	Both	50-69 yea	Sexually tra	Number	2012	506.81	1045.23	239.212
DALYs (Dis	Australasia	Both	50-69 yea	Sexually tra	Rate	2012	8.40453	17.3332	3.96689
DALYs (Dis	Eastern Eu	Both	50-69 yea	HIV/AIDS	Number	2011	76771.7	82034.5	73637.3
DALYs (Dis	Eastern Eu	Both	50-69 yea	HIV/AIDS	Rate	2011	153.411	163.928	147.148
DALYs (Dis	Eastern Eu	Both	50-69 yea	HIV/AIDS	Number	2010	69670.8	74930.6	66663.2
DALYs (Dis	Eastern Eu	Both	50-69 yea	HIV/AIDS	Rate	2010	141.74	152.441	135.622
DALYs (Dis	High-incor	Both	50-69 yea	Sexually tra	Number	2011	11478.8	22429.5	5907.45
DALYs (Dis	High-incor	Both	50-69 yea	Sexually tra	Rate	2011	14.1374	27.6245	7.27569
DALYs (Dis	Andean La	Both	50-69 yea	HIV/AIDS	Number	2011	15266.3	25436.9	11198.9
DALYs (Dis	Andean La	Both	50-69 yea	HIV/AIDS	Rate	2011	217.362	362.172	159.451
DALYs (Dis	East Asia	Both	50-69 yea	HIV/AIDS	Number	2011	149027	170626	134786
DALYs (Dis	East Asia	Both	50-69 yea	HIV/AIDS	Rate	2011	52.7	60.3383	47.6641
DALYs (Dis	Tropical La	Both	50-69 yea	HIV/AIDS	Number	2012	138025	145448	131183
DALYs (Dis	Tropical La	Both	50-69 yea	HIV/AIDS	Rate	2012	414.872	437.186	394.308
DALYs (Dis	Southeast	Both	50-69 yea	HIV/AIDS	Number	2011	334638	431086	278105
DALYs (Dis	Southeast	Both	50-69 yea	HIV/AIDS	Rate	2011	386.765	498.237	321.426
DALYs (Dis	High-incor	Both	50-69 yea	HIV/AIDS	Number	2011	180035	215557	154183
DALYs (Dis	High-incor	Both	50-69 yea	HIV/AIDS	Rate	2011	221.734	265.483	189.894
DALYs (Dis	Eastern Su	Both	50-69 yea	HIV/AIDS	Number	2010	2141280	2608623	1859389
DALYs (Dis	Eastern Su	Both	50-69 yea	HIV/AIDS	Rate	2010	9947.92	12119.1	8638.32
DALYs (Dis	Eastern Su	Both	50-69 yea	Sexually tra	Number	2010	16706.5	26038.4	8913.03
DALYs (Dis	Eastern Su	Both	50-69 yea	Sexually tra	Rate	2010	77.6146	120.969	41.408
DALYs (Dis	Western E	Both	50-69 yea	HIV/AIDS	Number	2012	64768.1	72723.4	58751.8
DALYs (Dis	Western E	Both	50-69 yea	HIV/AIDS	Rate	2012	63.0235	70.7646	57.1692
DALYs (Dis	Tropical La	Both	50-69 yea	Sexually tra	Number	2011	8995.31	17583.6	4798.59
DALYs (Dis	Tropical La	Both	50-69 yea	Sexually tra	Rate	2011	28.0239	54.7798	14.9495
DALYs (Dis	Eastern Eu	Both	50-69 yea	Sexually tra	Number	2010	8282.24	14353.5	5276.69
DALYs (Dis	Eastern Eu	Both	50-69 yea	Sexually tra	Rate	2010	16.8496	29.2012	10.7351
DALYs (Dis	Australasia	Both	50-69 yea	Sexually tra	Number	2011	494.869	1018.82	229.004
DALYs (Dis	Australasia	Both	50-69 yea	Sexually tra	Rate	2011	8.41457	17.3236	3.8939
DALYs (Dis	Eastern Su	Both	50-69 yea	HIV/AIDS	Number	2012	1992018	2392596	1759135
DALYs (Dis	Eastern Su	Both	50-69 yea	HIV/AIDS	Rate	2012	8665.11	10407.6	7652.09
DALYs (Dis	Eastern Su	Both	50-69 yea	Sexually tra	Number	2012	16704.1	25396.4	9056.05
DALYs (Dis	Eastern Su	Both	50-69 yea	Sexually tra	Rate	2012	72.6614	110.472	39.393
DALYs (Dis	Australasia	Both	50-69 yea	HIV/AIDS	Number	2012	1698.56	1960.6	1499.4
DALYs (Dis	Australasia	Both	50-69 yea	HIV/AIDS	Rate	2012	28.1675	32.5129	24.8648
DALYs (Dis	Andean La	Both	50-69 yea	Sexually tra	Number	2010	1377.94	2664.29	747.46
DALYs (Dis	Andean La	Both	50-69 yea	Sexually tra	Rate	2010	20.3164	39.2825	11.0206
DALYs (Dis	Western E	Both	50-69 yea	HIV/AIDS	Number	2013	66777.7	75624.5	60143
DALYs (Dis	Western E	Both	50-69 yea	HIV/AIDS	Rate	2013	63.8575	72.3175	57.5129
DALYs (Dis	Tropical La	Both	50-69 yea	HIV/AIDS	Number	2011	130727	137742	124035
DALYs (Dis	Tropical La	Both	50-69 yea	HIV/AIDS	Rate	2011	407.267	429.119	386.417
DALYs (Dis	Andean La	Both	50-69 yea	Sexually tra	Number	2011	1411.95	2743.83	744.526
DALYs (Dis	Andean La	Both	50-69 yea	Sexually tra	Rate	2011	20.1034	39.0669	10.6006
DALYs (Dis	Caribbean	Both	50-69 yea	Sexually tra	Number	2012	1954.14	3346.22	1194.75
DALYs (Dis	Caribbean	Both	50-69 yea	Sexually tra	Rate	2012	27.4937	47.0794	16.8095
DALYs (Dis	Western S	Both	50-69 yea	HIV/AIDS	Number	2012	1163328	1317222	1035482
DALYs (Dis	Western S	Both	50-69 yea	HIV/AIDS	Rate	2012	4280.9	4847.21	3810.44
DALYs (Dis	Central Lat	Both	50-69 yea	HIV/AIDS	Number	2011	89704.2	93133.9	86452.7
DALYs (Dis	Central Lat	Both	50-69 yea	HIV/AIDS	Rate	2011	293.263	304.475	282.633
DALYs (Dis	Central Lat	Both	50-69 yea	Sexually tra	Number	2011	7386.72	13944.5	3998.02
DALYs (Dis	Central Lat	Both	50-69 yea	Sexually tra	Rate	2011	24.1488	45.5876	13.0704
DALYs (Dis	Central Su	Both	50-69 yea	HIV/AIDS	Number	2012	346523	423075	285732
DALYs (Dis	Central Su	Both	50-69 yea	HIV/AIDS	Rate	2012	4453.22	5437	3671.99
DALYs (Dis	Western E	Both	50-69 yea	Sexually tra	Number	2012	7701.64	15537	3805.1
DALYs (Dis	Western E	Both	50-69 yea	Sexually tra	Rate	2012	7.49419	15.1185	3.70261
DALYs (Dis	Western S	Both	50-69 yea	Sexually tra	Number	2012	6355.59	12155.7	3371.86
DALYs (Dis	Western S	Both	50-69 yea	Sexually tra	Rate	2012	23.3878	44.7315	12.408
DALYs (Dis	Caribbean	Both	50-69 yea	Sexually tra	Number	2013	2037.42	3488.31	1262.41
DALYs (Dis	Caribbean	Both	50-69 yea	Sexually tra	Rate	2013	27.8427	47.6702	17.2517
DALYs (Dis	Southeast	Both	50-69 yea	Sexually tra	Number	2013	15437.8	29013.6	8479.07
DALYs (Dis	Southeast	Both	50-69 yea	Sexually tra	Rate	2013	16.497	31.0041	9.0608
DALYs (Dis	Oceania	Both	50-69 yea	HIV/AIDS	Number	2013	21571.3	55475.6	7961.94
DALYs (Dis	Oceania	Both	50-69 yea	HIV/AIDS	Rate	2013	1956.89	5032.62	722.288
DALYs (Dis	High-incor	Both	50-69 yea	HIV/AIDS	Number	2010	179083	210832	155777
DALYs (Dis	High-incor	Both	50-69 yea	HIV/AIDS	Rate	2010	227.091	267.351	197.537
DALYs (Dis	Southern S	Both	50-69 yea	Sexually tra	Number	2012	4101.56	6797.71	2653.24
DALYs (Dis	Southern S	Both	50-69 yea	Sexually tra	Rate	2012	49.5561	82.1317	32.0572
DALYs (Dis	Central Su	Both	50-69 yea	Sexually tra	Number	2012	4412.35	8179.52	2092.09
DALYs (Dis	Central Su	Both	50-69 yea	Sexually tra	Rate	2012	56.7038	105.116	26.8857
DALYs (Dis	Southeast	Both	50-69 yea	HIV/AIDS	Number	2013	383962	523958	310848
DALYs (Dis	Southeast	Both	50-69 yea	HIV/AIDS	Rate	2013	410.304	559.906	332.174
DALYs (Dis	Caribbean	Both	50-69 yea	HIV/AIDS	Number	2011	95146.5	113579	80898
DALYs (Dis	Caribbean	Both	50-69 yea	HIV/AIDS	Rate	2011	1376.42	1643.08	1170.3
DALYs (Dis	High-incor	Both	50-69 yea	Sexually tra	Number	2010	11183.4	21929.6	5739.82
DALYs (Dis	High-incor	Both	50-69 yea	Sexually tra	Rate	2010	14.1814	27.8083	7.27852
DALYs (Dis	Western E	Both	50-69 yea	HIV/AIDS	Number	2011	62649.4	70034.7	57022.8
DALYs (Dis	Western E	Both	50-69 yea	HIV/AIDS	Rate	2011	62.0544	69.3695	56.4812
DALYs (Dis	Central Su	Both	50-69 yea	Sexually tra	Number	2011	4328.64	8003.64	2039.72
DALYs (Dis	Central Su	Both	50-69 yea	Sexually tra	Rate	2011	57.9535	107.156	27.3085
DALYs (Dis	High-incor	Both	50-69 yea	Sexually tra	Number	2014	4595.95	8861.06	2335.29
DALYs (Dis	High-incor	Both	50-69 yea	Sexually tra	Rate	2014	9.58932	18.4883	4.87251
DALYs (Dis	Caribbean	Both	50-69 yea	HIV/AIDS	Number	2013	95105.7	111987	81540.1
DALYs (Dis	Caribbean	Both	50-69 yea	HIV/AIDS	Rate	2013	1299.68	1530.37	1114.3
DALYs (Dis	Oceania	Both	50-69 yea	Sexually tra	Number	2012	269.596	501.344	142.057
DALYs (Dis	Oceania	Both	50-69 yea	Sexually tra	Rate	2012	25.4496	47.3264	13.41
DALYs (Dis	North Afric	Both	50-69 yea	Sexually tra	Number	2012	6479.3	12554.5	3359.98
DALYs (Dis	North Afric	Both	50-69 yea	Sexually tra	Rate	2012	10.7376	20.8056	5.56822
DALYs (Dis	Eastern Eu	Both	50-69 yea	HIV/AIDS	Number	2014	91558.7	95706.3	88325.4
DALYs (Dis	Eastern Eu	Both	50-69 yea	HIV/AIDS	Rate	2014	171.413	179.178	165.36
DALYs (Dis	High-incor	Both	50-69 yea	HIV/AIDS	Number	2012	9934.7	12081.3	8373.33
DALYs (Dis	High-incor	Both	50-69 yea	HIV/AIDS	Rate	2012	21.041	25.5873	17.7341
DALYs (Dis	Central As	Both	50-69 yea	Sexually tra	Number	2013	2120.51	3593.49	1334.77
DALYs (Dis	Central As	Both	50-69 yea	Sexually tra	Rate	2013	17.8268	30.2099	11.2212
DALYs (Dis	Central Eu	Both	50-69 yea	Sexually tra	Number	2013	2726.94	5147.18	1427.12
DALYs (Dis	Central Eu	Both	50-69 yea	Sexually tra	Rate	2013	9.21464	17.3929	4.82241
DALYs (Dis	Southeast	Both	50-69 yea	Sexually tra	Number	2012	14964.9	28176.1	8285.01
DALYs (Dis	Southeast	Both	50-69 yea	Sexually tra	Rate	2012	16.6254	31.3024	9.20428
DALYs (Dis	Eastern Eu	Both	50-69 yea	Sexually tra	Number	2014	8334.04	14652.2	5097.59
DALYs (Dis	Eastern Eu	Both	50-69 yea	Sexually tra	Rate	2014	15.6027	27.4314	9.54356
DALYs (Dis	Southern S	Both	50-69 yea	HIV/AIDS	Number	2013	1537649	1902792	1288632
DALYs (Dis	Southern S	Both	50-69 yea	HIV/AIDS	Rate	2013	18046.8	22332.3	15124.2
DALYs (Dis	North Afric	Both	50-69 yea	Sexually tra	Number	2013	6758.32	13208.1	3573.27
DALYs (Dis	North Afric	Both	50-69 yea	Sexually tra	Rate	2013	10.7512	21.0116	5.6844
DALYs (Dis	Oceania	Both	50-69 yea	Sexually tra	Number	2013	278.76	516.69	147.203
DALYs (Dis	Oceania	Both	50-69 yea	Sexually tra	Rate	2013	25.2884	46.873	13.3539
DALYs (Dis	Southern L	Both	50-69 yea	Sexually tra	Number	2012	1692.95	3167.09	959.064
DALYs (Dis	Southern L	Both	50-69 yea	Sexually tra	Rate	2012	15.314	28.6488	8.67547
DALYs (Dis	High-incor	Both	50-69 yea	Sexually tra	Number	2013	4559.07	8984.16	2357.22
DALYs (Dis	High-incor	Both	50-69 yea	Sexually tra	Rate	2013	9.58147	18.8814	4.954
DALYs (Dis	Central Su	Both	50-69 yea	HIV/AIDS	Number	2011	344855	422030	282333
DALYs (Dis	Central Su	Both	50-69 yea	HIV/AIDS	Rate	2011	4617.06	5650.31	3779.99
DALYs (Dis	Andean La	Both	50-69 yea	HIV/AIDS	Number	2013	19482.7	33223.9	12973.4
DALYs (Dis	Andean La	Both	50-69 yea	HIV/AIDS	Rate	2013	257.265	438.715	171.312
DALYs (Dis	Southern L	Both	50-69 yea	HIV/AIDS	Number	2012	21703.1	23792.2	20356.5
DALYs (Dis	Southern L	Both	50-69 yea	HIV/AIDS	Rate	2012	196.321	215.219	184.14
DALYs (Dis	Southern S	Both	50-69 yea	HIV/AIDS	Number	2012	1621913	2016710	1345823
DALYs (Dis	Southern S	Both	50-69 yea	HIV/AIDS	Rate	2012	19596.4	24366.4	16260.6
DALYs (Dis	Southern L	Both	50-69 yea	HIV/AIDS	Number	2013	23706	26044	22148.5
DALYs (Dis	Southern L	Both	50-69 yea	HIV/AIDS	Rate	2013	209.768	230.457	195.987
DALYs (Dis	Western E	Both	50-69 yea	Sexually tra	Number	2011	7681.88	15562	3819.13
DALYs (Dis	Western E	Both	50-69 yea	Sexually tra	Rate	2011	7.60891	15.4142	3.78285
DALYs (Dis	Central Eu	Both	50-69 yea	Sexually tra	Number	2012	2740.57	5169.3	1454.84
DALYs (Dis	Central Eu	Both	50-69 yea	Sexually tra	Rate	2012	9.32011	17.5797	4.94762
DALYs (Dis	Central As	Both	50-69 yea	HIV/AIDS	Number	2013	4972.79	5628.9	4540.66
DALYs (Dis	Central As	Both	50-69 yea	HIV/AIDS	Rate	2013	41.8055	47.3213	38.1726
DALYs (Dis	East Asia	Both	50-69 yea	HIV/AIDS	Number	2014	214901	237535	196946
DALYs (Dis	East Asia	Both	50-69 yea	HIV/AIDS	Rate	2014	67.6019	74.7219	61.9537
DALYs (Dis	Andean La	Both	50-69 yea	HIV/AIDS	Number	2012	16416.5	27742.3	11474.7
DALYs (Dis	Andean La	Both	50-69 yea	HIV/AIDS	Rate	2012	225.211	380.586	157.416

DALYs (Dis	North Afric	Both	50-69 yea	HIV/AIDS	Rate	2013	75.1106	145.457	44.3492
DALYs (Dis	Australasia	Both	50-69 yea	HIV/AIDS	Number	2014	1611.66	1915.66	1378.22
DALYs (Dis	Australasia	Both	50-69 yea	HIV/AIDS	Rate	2014	25.52	30.3337	21.8236
DALYs (Dis	Tropical La	Both	50-69 yea	HIV/AIDS	Number	2015	163237	173153	154120
DALYs (Dis	Tropical La	Both	50-69 yea	HIV/AIDS	Rate	2015	442.561	469.445	417.842
DALYs (Dis	Western Su	Both	50-69 yea	Sexually tra	Number	2015	6995.81	13551.3	3625.21
DALYs (Dis	Western Su	Both	50-69 yea	Sexually tra	Rate	2015	23.2084	44.956	12.0265
DALYs (Dis	Caribbean	Both	50-69 yea	HIV/AIDS	Number	2015	95868.9	111623	82914.3
DALYs (Dis	Caribbean	Both	50-69 yea	HIV/AIDS	Rate	2015	1236.66	1439.88	1069.55
DALYs (Dis	Australasia	Both	50-69 yea	Sexually tra	Number	2015	538.554	1104.34	257.486
DALYs (Dis	Australasia	Both	50-69 yea	Sexually tra	Rate	2015	8.37789	17.1794	4.00553
DALYs (Dis	Caribbean	Both	50-69 yea	Sexually tra	Number	2014	2113.11	3607.36	1309.16
DALYs (Dis	Caribbean	Both	50-69 yea	Sexually tra	Rate	2014	28.046	47.8782	17.3757
DALYs (Dis	Central Lat	Both	50-69 yea	HIV/AIDS	Number	2014	96583	101817	91718.3
DALYs (Dis	Central Lat	Both	50-69 yea	HIV/AIDS	Rate	2014	283.576	298.945	269.293
DALYs (Dis	Central Lat	Both	50-69 yea	Sexually tra	Number	2014	8434.26	16077.4	4579.01
DALYs (Dis	Central Lat	Both	50-69 yea	Sexually tra	Rate	2014	24.7638	47.2046	13.4444
DALYs (Dis	Eastern Eu	Both	50-69 yea	Sexually tra	Number	2012	8200.27	14400.6	5059.59
DALYs (Dis	Eastern Eu	Both	50-69 yea	Sexually tra	Rate	2012	16.088	28.2524	9.92636
DALYs (Dis	Western Eu	Both	50-69 yea	Sexually tra	Number	2015	7845.61	15856.6	3861.22
DALYs (Dis	Western Eu	Both	50-69 yea	Sexually tra	Rate	2015	7.24317	14.639	3.56473
DALYs (Dis	Eastern Eu	Both	50-69 yea	HIV/AIDS	Number	2013	81475.8	86062.2	78228.8
DALYs (Dis	Eastern Eu	Both	50-69 yea	HIV/AIDS	Rate	2013	156.009	164.791	149.792
DALYs (Dis	High-incor	Both	50-69 yea	Sexually tra	Number	2013	12007.5	23384.7	6211.56
DALYs (Dis	High-incor	Both	50-69 yea	Sexually tra	Rate	2013	14.1129	27.4849	7.3007
DALYs (Dis	Australasia	Both	50-69 yea	HIV/AIDS	Number	2013	1555.75	1831.12	1330.54
DALYs (Dis	Australasia	Both	50-69 yea	HIV/AIDS	Rate	2013	25.1574	29.6104	21.5157
DALYs (Dis	Western Eu	Both	50-69 yea	Sexually tra	Number	2014	7749.07	15670	3810.12
DALYs (Dis	Western Eu	Both	50-69 yea	Sexually tra	Rate	2014	7.28616	14.7339	3.58252
DALYs (Dis	Western Su	Both	50-69 yea	HIV/AIDS	Number	2014	1223413	1375254	1105109
DALYs (Dis	Western Su	Both	50-69 yea	HIV/AIDS	Rate	2014	4211.24	4733.91	3804.01
DALYs (Dis	Tropical La	Both	50-69 yea	Sexually tra	Number	2014	9716.27	18542.4	5188.12
DALYs (Dis	Tropical La	Both	50-69 yea	Sexually tra	Rate	2014	27.2361	51.9769	14.5431
DALYs (Dis	Tropical La	Both	50-69 yea	HIV/AIDS	Number	2014	157038	166213	148810
DALYs (Dis	Tropical La	Both	50-69 yea	HIV/AIDS	Rate	2014	440.2	465.92	417.136
DALYs (Dis	Central Su	Both	50-69 yea	HIV/AIDS	Number	2015	318567	394615	258901
DALYs (Dis	Central Su	Both	50-69 yea	HIV/AIDS	Rate	2015	3612.39	4474.73	2935.81
DALYs (Dis	Southern L	Both	50-69 yea	Sexually tra	Number	2014	1774.78	3314.77	1014.96
DALYs (Dis	Southern L	Both	50-69 yea	Sexually tra	Rate	2014	15.3791	28.7237	8.79502
DALYs (Dis	Southeast	Both	50-69 yea	HIV/AIDS	Number	2014	401561	544365	323494
DALYs (Dis	Southeast	Both	50-69 yea	HIV/AIDS	Rate	2014	413.17	560.104	332.847
DALYs (Dis	Caribbean	Both	50-69 yea	HIV/AIDS	Number	2014	96252.4	112793	82936.4
DALYs (Dis	Caribbean	Both	50-69 yea	HIV/AIDS	Rate	2014	1277.5	1497.03	1100.76
DALYs (Dis	Oceania	Both	50-69 yea	HIV/AIDS	Number	2014	22315.7	59027.7	8703
DALYs (Dis	Oceania	Both	50-69 yea	HIV/AIDS	Rate	2014	1945.52	5146.13	758.742
DALYs (Dis	Central Su	Both	50-69 yea	Sexually tra	Number	2015	4638.2	8880.55	2170.18
DALYs (Dis	Central Su	Both	50-69 yea	Sexually tra	Rate	2015	52.5948	100.701	24.6087
DALYs (Dis	Southeast	Both	50-69 yea	Sexually tra	Number	2014	15939.7	30072.6	8656.84
DALYs (Dis	Southeast	Both	50-69 yea	Sexually tra	Rate	2014	16.4006	30.942	8.90712
DALYs (Dis	Central Su	Both	50-69 yea	HIV/AIDS	Number	2013	338920	416302	276173
DALYs (Dis	Central Su	Both	50-69 yea	HIV/AIDS	Rate	2013	4178.68	5132.76	3405.05
DALYs (Dis	North Afric	Both	50-69 yea	Sexually tra	Number	2014	7044.95	13607.2	3721.86
DALYs (Dis	North Afric	Both	50-69 yea	Sexually tra	Rate	2014	10.7583	20.7795	5.68362
DALYs (Dis	Central Su	Both	50-69 yea	Sexually tra	Number	2014	4568.46	8472.62	2119.21
DALYs (Dis	Central Su	Both	50-69 yea	Sexually tra	Rate	2014	54.023	100.19	25.0601
DALYs (Dis	North Afric	Both	50-69 yea	HIV/AIDS	Number	2015	51482	99910.6	30602.8
DALYs (Dis	North Afric	Both	50-69 yea	HIV/AIDS	Rate	2015	75.5041	146.53	44.8824
DALYs (Dis	Southeast	Both	50-69 yea	HIV/AIDS	Number	2015	402939	531997	332285
DALYs (Dis	Southeast	Both	50-69 yea	HIV/AIDS	Rate	2015	399.712	527.736	329.623
DALYs (Dis	Southern L	Both	50-69 yea	Sexually tra	Number	2015	1799.59	3378.39	1036.41
DALYs (Dis	Southern L	Both	50-69 yea	Sexually tra	Rate	2015	15.295	28.7133	8.80861
DALYs (Dis	East Asia	Both	50-69 yea	HIV/AIDS	Number	2015	275522	307165	252947
DALYs (Dis	East Asia	Both	50-69 yea	HIV/AIDS	Rate	2015	82.9666	92.4951	76.1689
DALYs (Dis	Oceania	Both	50-69 yea	HIV/AIDS	Number	2015	23576.3	63017.7	9496.48
DALYs (Dis	Oceania	Both	50-69 yea	HIV/AIDS	Rate	2015	1975.89	5281.41	795.884
DALYs (Dis	South Asia	Both	50-69 yea	HIV/AIDS	Number	2014	442992	616439	338928
DALYs (Dis	South Asia	Both	50-69 yea	HIV/AIDS	Rate	2014	211.515	294.33	161.827
DALYs (Dis	South Asia	Both	50-69 yea	Sexually tra	Number	2014	52349.7	71526.7	37997.8
DALYs (Dis	South Asia	Both	50-69 yea	Sexually tra	Rate	2014	24.9953	34.1517	18.1428
DALYs (Dis	Southern L	Both	50-69 yea	HIV/AIDS	Number	2015	26398.7	29299.7	24509.6
DALYs (Dis	Southern L	Both	50-69 yea	HIV/AIDS	Rate	2015	224.366	249.022	208.31
DALYs (Dis	High-incor	Both	50-69 yea	HIV/AIDS	Number	2015	9362.73	11819.5	7588.67
DALYs (Dis	High-incor	Both	50-69 yea	HIV/AIDS	Rate	2015	19.2112	24.2521	15.571
DALYs (Dis	Central Lat	Both	50-69 yea	HIV/AIDS	Number	2015	98206.8	103808	93276.4
DALYs (Dis	Central Lat	Both	50-69 yea	HIV/AIDS	Rate	2015	278.569	294.458	264.584
DALYs (Dis	Central Lat	Both	50-69 yea	Sexually tra	Number	2015	8745.86	16587.4	4761.21
DALYs (Dis	Central Lat	Both	50-69 yea	Sexually tra	Rate	2015	24.8081	47.0509	13.5054
DALYs (Dis	Central Eu	Both	50-69 yea	Sexually tra	Number	2014	2705.42	5142.47	1402.47
DALYs (Dis	Central Eu	Both	50-69 yea	Sexually tra	Rate	2014	9.09091	17.28	4.71265
DALYs (Dis	Southern L	Both	50-69 yea	HIV/AIDS	Number	2016	26699.9	29991.4	24467.9
DALYs (Dis	Southern L	Both	50-69 yea	HIV/AIDS	Rate	2016	222.936	250.418	204.299
DALYs (Dis	Central Eu	Both	50-69 yea	HIV/AIDS	Number	2014	5976.95	7335.35	5249.49
DALYs (Dis	Central Eu	Both	50-69 yea	HIV/AIDS	Rate	2014	20.0841	24.6487	17.6396
DALYs (Dis	North Afric	Both	50-69 yea	HIV/AIDS	Number	2014	49354	94842.8	29195.5
DALYs (Dis	North Afric	Both	50-69 yea	HIV/AIDS	Rate	2014	75.368	144.834	44.5841
DALYs (Dis	High-incor	Both	50-69 yea	Sexually tra	Number	2015	12483.2	24392.2	6433.1
DALYs (Dis	High-incor	Both	50-69 yea	Sexually tra	Rate	2015	14.1032	27.5577	7.26797
DALYs (Dis	Australasia	Both	50-69 yea	Sexually tra	Number	2013	521.963	1050.56	247.201
DALYs (Dis	Australasia	Both	50-69 yea	Sexually tra	Rate	2013	8.44047	16.9883	3.99739
DALYs (Dis	Western Su	Both	50-69 yea	Sexually tra	Number	2013	6561.81	12634	3452.11
DALYs (Dis	Western Su	Both	50-69 yea	Sexually tra	Rate	2013	23.3593	44.9754	12.2891
DALYs (Dis	High-incor	Both	50-69 yea	HIV/AIDS	Number	2016	9596.11	12093.2	7750.31
DALYs (Dis	High-incor	Both	50-69 yea	HIV/AIDS	Rate	2016	19.4647	24.5299	15.7207
DALYs (Dis	Central As	Both	50-69 yea	HIV/AIDS	Number	2014	6225.75	6815.72	5768.51
DALYs (Dis	Central As	Both	50-69 yea	HIV/AIDS	Rate	2014	49.7646	54.4805	46.1098
DALYs (Dis	Southern S	Both	50-69 yea	Sexually tra	Number	2015	4175.89	7001.93	2677.74
DALYs (Dis	Southern S	Both	50-69 yea	Sexually tra	Rate	2015	46.2793	77.5989	29.676
DALYs (Dis	Western Su	Both	50-69 yea	HIV/AIDS	Number	2013	1192466	1341928	1071781
DALYs (Dis	Western Su	Both	50-69 yea	HIV/AIDS	Rate	2013	4245.04	4777.11	3815.41
DALYs (Dis	Central As	Both	50-69 yea	HIV/AIDS	Number	2015	6007.94	6595.6	5564.65
DALYs (Dis	Central As	Both	50-69 yea	HIV/AIDS	Rate	2015	45.884	50.372	42.4985
DALYs (Dis	Southern L	Both	50-69 yea	Sexually tra	Number	2016	1814.4	3421.23	1032.23
DALYs (Dis	Southern L	Both	50-69 yea	Sexually tra	Rate	2016	15.1497	28.5662	8.61878
DALYs (Dis	Southern S	Both	50-69 yea	HIV/AIDS	Number	2015	1578824	1890181	1360820
DALYs (Dis	Southern S	Both	50-69 yea	HIV/AIDS	Rate	2015	17497.3	20947.9	15081.3
DALYs (Dis	Andean La	Both	50-69 yea	Sexually tra	Number	2015	1601.92	3129.02	819.84
DALYs (Dis	Andean La	Both	50-69 yea	Sexually tra	Rate	2015	19.6695	38.4202	10.0666
DALYs (Dis	Andean La	Both	50-69 yea	HIV/AIDS	Number	2016	23668.7	39991.7	15669.7
DALYs (Dis	Andean La	Both	50-69 yea	HIV/AIDS	Rate	2016	281.332	475.351	186.254
DALYs (Dis	Central As	Both	50-69 yea	Sexually tra	Number	2014	2156.73	3713.92	1331.16
DALYs (Dis	Central As	Both	50-69 yea	Sexually tra	Rate	2014	17.2395	29.6867	10.6405
DALYs (Dis	High-incor	Both	50-69 yea	HIV/AIDS	Number	2015	183663	227557	152012
DALYs (Dis	High-incor	Both	50-69 yea	HIV/AIDS	Rate	2015	207.498	257.088	171.74
DALYs (Dis	Central Eu	Both	50-69 yea	HIV/AIDS	Number	2015	5541.42	6782.25	4844.72
DALYs (Dis	Central Eu	Both	50-69 yea	HIV/AIDS	Rate	2015	18.5405	22.6921	16.2095
DALYs (Dis	Western Eu	Both	50-69 yea	HIV/AIDS	Number	2015	68515.9	78344.5	60783.4
DALYs (Dis	Western Eu	Both	50-69 yea	HIV/AIDS	Rate	2015	63.2549	72.3287	56.1161
DALYs (Dis	South Asia	Both	50-69 yea	HIV/AIDS	Number	2015	404319	582051	301920
DALYs (Dis	South Asia	Both	50-69 yea	HIV/AIDS	Rate	2015	186.877	269.025	139.548
DALYs (Dis	South Asia	Both	50-69 yea	Sexually tra	Number	2015	56590	76815.2	41268.6
DALYs (Dis	South Asia	Both	50-69 yea	Sexually tra	Rate	2015	26.156	35.5041	19.0744
DALYs (Dis	Central Eu	Both	50-69 yea	Sexually tra	Number	2016	2666.77	5127.75	1396.67
DALYs (Dis	Central Eu	Both	50-69 yea	Sexually tra	Rate	2016	8.91209	17.1364	4.66753
DALYs (Dis	Oceania	Both	50-69 yea	Sexually tra	Number	2016	308.078	563.7	162.221
DALYs (Dis	Oceania	Both	50-69 yea	Sexually tra	Rate	2016	24.8223	45.4182	13.0704
DALYs (Dis	Western Su	Both	50-69 yea	HIV/AIDS	Number	2017	1255910	1431655	1124186
DALYs (Dis	Western Su	Both	50-69 yea	HIV/AIDS	Rate	2017	3861.68	4402.07	3456.66
DALYs (Dis	Oceania	Both	50-69 yea	Sexually tra	Number	2014	288.496	533.614	152.099
DALYs (Dis	Oceania	Both	50-69 yea	Sexually tra	Rate	2014	25.1515	46.5213	13.2603
DALYs (Dis	Western Su	Both	50-69 yea	Sexually tra	Number	2017	7648.66	14823.4	3901.78
DALYs (Dis	Western Su	Both	50-69 yea	Sexually tra	Rate	2017	23.5182	45.5793	11.9972
DALYs (Dis	Central As	Both	50-69 yea	Sexually tra	Number	2016	2261.6	3903.47	1383.33
DALYs (Dis	Central As	Both	50-69 yea	Sexually tra	Rate	2016	16.5834	28.6225	10.1434
DALYs (Dis	High-incor	Both	50-69 yea	Sexually tra	Number	2015	4661.48	9061.75	2379.43
DALYs (Dis	High-incor	Both	50-69 yea	Sexually tra	Rate	2015	9.56478	18.5936	4.8823
DALYs (Dis	Australasia	Both	50-69 yea	HIV/AIDS	Number	2017	1671.19	1993.95	1413.53
DALYs (Dis	Australasia	Both	50-69 yea	HIV/AIDS	Rate	2017	25.3922	30.2963	21.4773
DALYs (Dis	Caribbean	Both	50-69 yea	HIV/AIDS	Number	2018	84491.9	99287.6	71900.6
DALYs (Dis	Caribbean	Both	50-69 yea	HIV/AIDS	Rate	2018	1016.1	1194.03	864.674
DALYs (Dis	East Asia	Both	50-69 yea	Sexually tra	Number	2015	41507.1	77307.7	23155.3
DALYs (Dis	East Asia	Both	50-69 yea	Sexually tra	Rate	2015	12.4988	23.2793	6.97265
DALYs (Dis	Southern S	Both	50-69 yea	Sexually tra	Number	2014	4135.67	6911.7	2691.06
DALYs (Dis	Southern S	Both	50-69 yea	Sexually tra	Rate	2014	47.1561	78.8093	30.6843
DALYs (Dis	Southeast	Both	50-69 yea	HIV/AIDS	Number	2018	351841	469696	284905
DALYs (Dis	Southeast	Both	50-69 yea	HIV/AIDS	Rate	2018	315.331	420.957	255.341
DALYs (Dis	Tropical La	Both	50-69 yea	Sexually tra	Number	2013	9487.64	18215.7	5080.7
DALYs (Dis	Tropical La	Both	50-69 yea	Sexually tra	Rate	2013	27.5286	52.8533	14.7418
DALYs (Dis	Central Lat	Both	50-69 yea	HIV/AIDS	Number	2013	95382.5	100093	91324.8
DALYs (Dis	Central Lat	Both	50-69 yea	HIV/AIDS	Rate	2013	290.043	304.366	277.704
DALYs (Dis	Central Lat	Both	50-69 yea	Sexually tra	Number	2013	8106.03	15339.2	4397.1
DALYs (Dis	Central Lat	Both	50-69 yea	Sexually tra	Rate	2013	24.6491	46.6442	13.3709
DALYs (Dis	Andean La	Both	50-69 yea	Sexually tra	Number	2016	1645.74	3216.21	843.677
DALYs (Dis	Andean La	Both	50-69 yea	Sexually tra	Rate	2016	19.5616	38.2287	10.0281
DALYs (Dis	Caribbean	Both	50-69 yea	Sexually tra	Number	2017	2281.62	3916.69	1413.38
DALYs (Dis	Caribbean	Both	50-69 yea	Sexually tra	Rate	2017	28.0175	48.0955	17.3558
DALYs (Dis	Western Su	Both	50-69 yea	Sexually tra	Number	2016	7319.71	14156.8	3824.8
DALYs (Dis	Western Su	Both	50-69 yea	Sexually tra	Rate	2016	23.3825	45.2234	12.2182
DALYs (Dis	Central Su	Both	50-69 yea	HIV/AIDS	Number	2016	310391	385060	253838
DALYs (Dis	Central Su	Both	50-69 yea	HIV/AIDS	Rate	2016	3377.24	4189.68	2761.91
DALYs (Dis	North Afric	Both	50-69 yea	Sexually tra	Number	2016	7655.82	14792.7	3973.3
DALYs (Dis	North Afric	Both	50-69 yea	Sexually tra	Rate	2016	10.7862	20.8413	5.59795
DALYs (Dis	Tropical La	Both	50-69 yea	Sexually tra	Number	2017	10539.1	20184.5	5588.01
DALYs (Dis	Tropical La	Both	50-69 yea	Sexually tra	Rate	2017	26.8851	51.4905	14.2549
DALYs (Dis	High-incor	Both	50-69 yea	HIV/AIDS	Number	2014	9752.94	12129.2	8058.09
DALYs (Dis	High-incor	Both	50-69 yea	HIV/AIDS	Rate	2014	20.3492	25.3072	16.813
DALYs (Dis	Central Eu	Both	50-69 yea	HIV/AIDS	Number	2018	5013.44	6030.34	4513.63
DALYs (Dis	Central Eu	Both	50-69 yea	HIV/AIDS	Rate	2018	16.7529	20.151	15.0828
DALYs (Dis	Australasia	Both	50-69 yea	Sexually tra	Number	2016	544.064	1113.14	254.67
DALYs (Dis	Australasia	Both	50-69 yea	Sexually tra	Rate	2016	8.33583	17.0549	3.90191
DALYs (Dis	Central Su	Both	50-69 yea	HIV/AIDS	Number	2017	296931	369829	243136
DALYs (Dis	Central Su	Both	50-69 yea	HIV/AIDS	Rate	2017	3101.25	3862.62	2539.39
DALYs (Dis	Eastern Eu	Both	50-69 yea	HIV/AIDS	Number	2015	102998	106532	99551.6
DALYs (Dis	Eastern Eu	Both	50-69 yea	HIV/AIDS	Rate	2015	189.47	195.972	183.13
DALYs (Dis	Eastern Eu	Both	50-69 yea	Sexually tra	Number	2016	8435.16	14688.8	5191.29
DALYs (Dis	Eastern Eu	Both	50-69 yea	Sexually tra	Rate	2016	15.3649	26.7561	9.45608
DALYs (Dis	South Asia	Both	50-69 yea	HIV/AIDS	Number	2018	324389	521297	231453
DALYs (Dis	South Asia	Both	50-69 yea	HIV/AIDS	Rate	2018	136.538	219.418	97.4204
DALYs (Dis	South Asia	Both	50-69 yea	Sexually tra	Number	2018	59242.7	80861.1	42041.7
DALYs (Dis	South Asia	Both	50-69 yea	Sexually tra	Rate	2018	24.9358	34.0351	17.6957
DALYs (Dis	Oceania	Both	50-69 yea	HIV/AIDS	Number	2018	28560.2	75234	11946.5
DALYs (Dis	Oceania	Both	50-69 yea	HIV/AIDS	Rate	2018	2140.35	5638.16	895.292
DALYs (Dis	High-incor	Both	50-69 yea	Sexually tra	Number	2017	12657.8	24414.3	6523.49
DALYs (Dis	High-incor	Both	50-69 yea	Sexually tra	Rate	2017	14.0171	27.036	7.22402
DALYs (Dis	East Asia	Both	50-69 yea	HIV/AIDS	Number	2016	328834	373325	291443
DALYs (Dis	East Asia	Both	50-69 yea	HIV/AIDS	Rate	2016	95.1623	108.038	84.3415
DALYs (Dis	Eastern Eu	Both	50-69 yea	HIV/AIDS	Number	2019	97186.2	103131	92109
DALYs (Dis	Eastern Eu	Both	50-69 yea	HIV/AIDS	Rate	2019	178.692	189.622	169.356
DALYs (Dis	High-incor	Both	50-69 yea	Sexually tra	Number	2018	4727.97	9198.31	2417.34
DALYs (Dis	High-incor	Both	50-69 yea	Sexually tra	Rate	2018	9.56055	18.6001	4.88816
DALYs (Dis	Central As	Both	50-69 yea	HIV/AIDS	Number	2018	5639.6	6174.31	5154.39
DALYs (Dis	Central As	Both	50-69 yea	HIV/AIDS	Rate	2018	38.7434	42.4168	35.4101
DALYs (Dis	Central As	Both	50-69 yea	Sexually tra	Number	2018	2324.57	4032.74	1424.64
DALYs (Dis	Central As	Both	50-69 yea	Sexually tra	Rate	2018	15.9695	27.7044	9.7871
DALYs (Dis	North Afric	Both	50-69 yea	HIV/AIDS	Number	2018	57802.1	114003	33902.9
DALYs (Dis	North Afric	Both	50-69 yea	HIV/AIDS	Rate	2018	75.3641	148.64	44.2036
DALYs (Dis	Southeast	Both	50-69 yea	Sexually tra	Number	2017	17019.3	32686.2	9253.41
DALYs (Dis	Southeast	Both	50-69 yea	Sexually tra	Rate	2017	15.762	30.2715	8.56981
DALYs (Dis	Eastern Eu	Both	50-69 yea	HIV/AIDS	Number	2018	100106	105484	95450.6
DALYs (Dis	Eastern Eu	Both	50-69 yea	HIV/AIDS	Rate	2018	182.985	192.816	174.476
DALYs (Dis	Australasia	Both	50-69 yea	Sexually tra	Number	2017	548.074	1141.94	255.479
DALYs (Dis	Australasia	Both	50-69 yea	Sexually tra	Rate	2017	8.32749	17.3508	3.88177
DALYs (Dis	High-incor	Both	50-69 yea	Sexually tra	Number	2019	12774.6	24373.7	6676.35
DALYs (Dis	High-incor	Both	50-69 yea	Sexually tra	Rate	2019	14.0258	26.7611	7.33029
DALYs (Dis	East Asia	Both	50-69 yea	Sexually tra	Number	2017	44762.8	83123.6	24895.4
DALYs (Dis	East Asia	Both	50-69 yea	Sexually tra	Rate	2017	12.4897	23.1931	6.94629
DALYs (Dis	Andean La	Both	50-69 yea	HIV/AIDS	Number	2015	23621	39738.8	15265.5
DALYs (Dis	Andean La	Both	50-69 yea	HIV/AIDS	Rate	2015	290.035	487.94	187.441
DALYs (Dis	Andean La	Both	50-69 yea	HIV/AIDS	Number	2019	20589.9	37529.6	14417.2
DALYs (Dis	Andean La	Both	50-69 yea	HIV/AIDS	Rate	2019	222.947	406.369	156.109
DALYs (Dis	East Asia	Both	50-69 yea	Sexually tra	Number	2019	47890.4	88916.3	26292.2
DALYs (Dis	East Asia	Both	50-69 yea	Sexually tra	Rate	2019	12.5646	23.3283	6.89807
DALYs (Dis	Caribbean	Both	50-69 yea	Sexually tra	Number	2018	2315.65	3915.9	1414.04
DALYs (Dis	Caribbean	Both	50-69 yea	Sexually tra	Rate	2018	27.8479	47.0924	17.0052
DALYs (Dis	North Afric	Both	50-69 yea	Sexually tra	Number	2017	7906.44	15263.1	4160.33
DALYs (Dis	North Afric	Both	50-69 yea	Sexually tra	Rate	2017	10.7096	20.6745	5.63536
DALYs (Dis	Oceania	Both	50-69 yea	Sexually tra	Number	2017	317.129	587.021	166.694
DALYs (Dis	Oceania	Both	50-69 yea	Sexually tra	Rate	2017	24.6431	45.6155	12.9533
DALYs (Dis	Central As	Both	50-69 yea	Sexually tra	Number	2017	2287.19	3958.71	1400.07
DALYs (Dis	Central As	Both	50-69 yea	Sexually tra	Rate	2017	16.2064	28.0504	9.9205
DALYs (Dis	Tropical La	Both	50-69 yea	HIV/AIDS	Number	2016	169058	179804	159281
DALYs (Dis	Tropical La	Both	50-69 yea	HIV/AIDS	Rate	2016	443.944	472.164	418.271
DALYs (Dis	Western Eu	Both	50-69 yea	HIV/AIDS	Number	2016	66683.4	76912	58473.4
DALYs (Dis	Western Eu	Both	50-69 yea	HIV/AIDS	Rate	2016	60.5754	69.8671	53.1174
DALYs (Dis	Southern L	Both	50-69 yea	Sexually tra	Number	2018	1809.62	3471.61	1009.52
DALYs (Dis	Southern L	Both	50-69 yea	Sexually tra	Rate	2018	14.6468	28.0987	8.1709
DALYs (Dis	Eastern Eu	Both	50-69 yea	Sexually tra	Number	2018	8068.62	14131.8	4861.94
DALYs (Dis	Eastern Eu	Both	50-69 yea	Sexually tra	Rate	2018	14.7488	25.8317	8.88723
DALYs (Dis	Central Su	Both	50-69 yea	Sexually tra	Number	2016	4710.08	9023.33	2237.1
DALYs (Dis	Central Su	Both	50-69 yea	Sexually tra	Rate	2016	51.2485	98.1793	24.341
DALYs (Dis	East Asia	Both	50-69 yea	HIV/AIDS	Number	2019	376610	484666	288615
DALYs (Dis	East Asia	Both	50-69 yea	HIV/AIDS	Rate	2019	98.8082	127.158	75.7216
DALYs (Dis	Eastern Eu	Both	50-69 yea	Sexually tra	Number	2017	8172.74	14505.7	4977.82
DALYs (Dis	Eastern Eu	Both	50-69 yea	Sexually tra	Rate	2017	14.9081	26.4602	9.08017
DALYs (Dis	Andean La	Both	50-69 yea	Sexually tra	Number	2019	1770.26	3480.97	910.133
DALYs (Dis	Andean La	Both	50-69 yea	Sexually tra	Rate	2019	19.1683	37.6918	9.85487
DALYs (Dis	High-incor	Both	50-69 yea	HIV/AIDS	Number	2016	182940	227203	151165
DALYs (Dis	High-incor	Both	50-69 yea	HIV/AIDS	Rate	2016	203.529	252.774	168.178
DALYs (Dis	Oceania	Both	50-69 yea	HIV/AIDS	Number	2017	27076.1	72000.5	11187
DALYs (Dis	Oceania	Both	50-69 yea	HIV/AIDS	Rate	2017	2104	5594.93	869.304
DALYs (Dis	Southeast	Both	50-69 yea	HIV/AIDS	Number	2019	338930	452738	269680
DALYs (Dis	Southeast	Both	50-69 yea	HIV/AIDS	Rate	2019	294.294	393.113	234.164
DALYs (Dis	High-incor	Both	50-69 yea	HIV/AIDS	Number	2019	175665	221715	142239
DALYs (Dis	High-incor	Both	50-69 yea	HIV/AIDS	Rate	2019	192.871	243.432	156.171
DALYs (Dis	Western Su	Both	50-69 yea	HIV/AIDS	Number	2016	1257162	1422484	1131182
DALYs (Dis	Western Su	Both	50-69 yea	HIV/AIDS	Rate	2016	4015.96	4544.08	3613.52
DALYs (Dis	Oceania	Both	50-69 yea	Sexually tra	Number	2018	327.952	603.863	173.672
DALYs (Dis	Oceania	Both	50-69 yea	Sexually tra	Rate	2018	24.5772	45.2545	13.0153
DALYs (Dis	Eastern Su	Both	50-69 yea	HIV/AIDS	Number	2018	1674660	2029831	1436627
DALYs (Dis	Eastern Su	Both	50-69 yea	HIV/AIDS	Rate	2018	5892.72	7142.48	5055.14
DALYs (Dis	Eastern Su	Both	50-69 yea	Sexually tra	Number	2018	17453.6	26856	9894.26
DALYs (Dis	Eastern Su	Both	50-69 yea	Sexually tra	Rate	2018	61.4149	94.4996	34.8155
DALYs (Dis	Eastern Su	Both	50-69 yea	HIV/AIDS	Number	2017	1695468	2050748	1461840
DALYs (Dis	Eastern Su	Both	50-69 yea	HIV/AIDS	Rate	2017	6189.43	7486.4	5336.55
DALYs (Dis	Eastern Su	Both	50-69 yea	Sexually tra	Number	2017	17264.9	26094.4	9773.55
DALYs (Dis	Eastern Su	Both	50-69 yea	Sexually tra	Rate	2017	63.0266	95.2595	35.679
DALYs (Dis	North Afric	Both	50-69 yea	HIV/AIDS	Number	2017	55489.6	109761	33171.9
DALYs (Dis	North Afric	Both	50-69 yea	HIV/AIDS	Rate	2017	75.1631	148.676	44.9328
DALYs (Dis	Western Eu	Both	50-69 yea	Sexually tra	Number	2018	8186.6	16645.3	4014.02
DALYs (Dis	Western Eu	Both	50-69 yea	Sexually tra	Rate	2018	7.28971	14.8217	3.57426
DALYs (Dis	Caribbean	Both	50-69 yea	HIV/AIDS	Number	2019	84699.2	98898.5	72783.1
DALYs (Dis	Caribbean	Both	50-69 yea	HIV/AIDS	Rate	2019	997.709	1164.97	857.344
DALYs (Dis	Southern S	Both	50-69 yea	HIV/AIDS	Number	2017	1704616	1977596	1483073
DALYs (Dis	Southern S	Both	50-69 yea	HIV/AIDS	Rate	2017	17955	20830.4	15621.5
DALYs (Dis	Southern S	Both	50-69 yea	HIV/AIDS	Number	2016	1665403	1952822	1443898
DALYs (Dis	Southern S	Both	50-69 yea	HIV/AIDS	Rate	2016	17994.3	21099.8	15601
DALYs (Dis	Central Eu	Both	50-69 yea	Sexually tra	Number	2018	2592.88	5016.98	1333.92
DALYs (Dis	Central Eu	Both	50-69 yea	Sexually tra	Rate	2018	8.66437	16.7648	4.45743
DALYs (Dis	Central Eu	Both	50-69 yea	HIV/AIDS	Number	2017	5173.19	6265.29	4580.75
DALYs (Dis	Central Eu	Both	50-69 yea	HIV/AIDS	Rate	2017	17.308	20.9619	15.3259
DALYs (Dis	Australasia	Both	50-69 yea	HIV/AIDS	Number	2016	1658.12	1965.49	1396.6
DALYs (Dis	Australasia	Both	50-69 yea	HIV/AIDS	Rate	2016	25.4047	30.1141	21.3978
DALYs (Dis	Central Eu	Both	50-69 yea	Sexually tra	Number	2017	2623.75	5070.75	1342.93
DALYs (Dis	Central Eu	Both	50-69 yea	Sexually tra	Rate	2017	8.77831	16.9653	4.49305
DALYs (Dis	High-incor	Both	50-69 yea	Sexually tra	Number	2017	4705.43	9243.5	2398.4
DALYs (Dis	High-incor	Both	50-69 yea	Sexually tra	Rate	2017	9.52861	18.7183	4.85682
DALYs (Dis	Southeast	Both	50-69 yea	Sexually tra	Number	2018	17466.6	33275.1	9300.52
DALYs (Dis	Southeast	Both	50-69 yea	Sexually tra	Rate	2018	15.6541	29.8222	8.33542
DALYs (Dis	Southern L	Both	50-69 yea	HIV/AIDS	Number	2017	26741.2	30168.9	24470.1
DALYs (Dis	Southern L	Both	50-69 yea	HIV/AIDS	Rate	2017	219.857	248.038	201.185
DALYs (Dis	High-incor	Both	50-69 yea	Sexually tra	Number	2019	4770.49	9344.38	2426.64
DALYs (Dis	High-incor	Both	50-69 yea	Sexually tra	Rate	2019	9.6264	18.8561	4.89673
DALYs (Dis	High-incor	Both	50-69 yea	HIV/AIDS	Number	2018	177584	221813	144662
DALYs (Dis	High-incor	Both	50-69 yea	HIV/AIDS	Rate	2018	196.031	244.855	159.689
DALYs (Dis	Tropical La	Both	50-69 yea	HIV/AIDS	Number	2019	172046	185016	161088
DALYs (Dis	Tropical La	Both	50-69 yea	HIV/AIDS	Rate	2019	416.605	448.011	390.069
DALYs (Dis	Tropical La	Both	50-69 yea	Sexually tra	Number	2018	10671.2	20556.9	5602.24
DALYs (Dis	Tropical La	Both	50-69 yea	Sexually tra	Rate	2018	26.4984	51.0464	13.9113
DALYs (Dis	High-incor	Both	50-69 yea	Sexually tra	Number	2018	12692.8	24342.7	6587.79
DALYs (Dis	High-incor	Both	50-69 yea	Sexually tra	Rate	2018	14.0113	26.8715	7.27214
DALYs (Dis	East Asia	Both	50-69 yea	Sexually tra	Number	2018	46344.4	86214.3	25420.6
DALYs (Dis	East Asia	Both	50-69 yea	Sexually tra	Rate	2018	12.4962	23.2467	6.85438
DALYs (Dis	Southern S	Both	50-69 yea	Sexually tra	Number	2016	4239.68	7090.61	2699.23
DALYs (Dis	Southern S	Both	50-69 yea	Sexually tra	Rate	2016	45.8088	76.6125	29.1646
DALYs (Dis	Southeast	Both	50-69 yea	Sexually tra	Number	2016	16718.5	31884	9049.79
DALYs (Dis	Southeast	Both	50-69 yea	Sexually tra	Rate	2016	16.0211	30.554	8.67228
DALYs (Dis	Western Eu	Both	50-69 yea	Sexually tra	Number	2016	8017.13	16288.4	3943.56
DALYs (Dis	Western Eu	Both	50-69 yea	Sexually tra	Rate	2016	7.28278	14.7964	3.58234
DALYs (Dis	South Asia	Both	50-69 yea	HIV/AIDS	Number	2017	333851	529921	237937
DALYs (Dis	South Asia	Both	50-69 yea	HIV/AIDS	Rate	2017	144.972	230.114	103.322
DALYs (Dis	South Asia	Both	50-69 yea	Sexually tra	Number	2017	58922.8	81339.8	42144.7
DALYs (Dis	South Asia	Both	50-69 yea	Sexually tra	Rate	2017	25.5867	35.3211	18.301
DALYs (Dis	Central Lat	Both	50-69 yea	HIV/AIDS	Number	2018	98357.5	104777	92237.8
DALYs (Dis	Central Lat	Both	50-69 yea	HIV/AIDS	Rate	2018	253.987	270.564	238.184
DALYs (Dis	Central Lat	Both	50-69 yea	Sexually tra	Number	2018	9329.66	17648.3	5117.69
DALYs (Dis	Central Lat	Both	50-69 yea	Sexually tra	Rate	2018	24.0918	45.5728	13.2153
DALYs (Dis	Central As	Both	50-69 yea	HIV/AIDS	Number	2017	5617.24	6193.72	5155.98
DALYs (Dis	Central As	Both	50-69 yea	HIV/AIDS	Rate	2017	39.8023	43.8871	36.5339
DALYs (Dis	Central Su	Both	50-69 yea	Sexually tra	Number	2019	4956.17	9168.67	2408.82
DALYs (Dis	Central Su	Both	50-69 yea	Sexually tra	Rate	2019	47.7739	88.3793	23.2193
DALYs (Dis	Western Eu	Both	50-69 yea	Sexually tra	Number	2019	8224.75	16820.7	4013.5
DALYs (Dis	Western Eu	Both	50-69 yea	Sexually tra	Rate	2019	7.26832	14.8646	3.54679
DALYs (Dis	Southern S	Both	50-69 yea	Sexually tra	Number	2019	4266.84	7331.8	2613.35
DALYs (Dis	Southern S	Both	50-69 yea	Sexually tra	Rate	2019	42.6485	73.2837	26.1212
DALYs (Dis	Southern L	Both	50-69 yea	Sexually tra	Number	2019	1832.25	3543.99	1016.37
DALYs (Dis	Southern L	Both	50-69 yea	Sexually tra	Rate	2019	14.5661	28.1742	8.07998
DALYs (Dis	Southern S	Both	50-69 yea	Sexually tra	Number	2018	4261.05	7262.12	2611.66
DALYs (Dis	Southern S	Both	50-69 yea	Sexually tra	Rate	2018	43.741	74.5478	26.8095
DALYs (Dis	Caribbean	Both	50-69 yea	Sexually tra	Number	2016	2235.85	3850.43	1398.26
DALYs (Dis	Caribbean	Both	50-69 yea	Sexually tra	Rate	2016	28.0938	48.3814	17.5694
DALYs (Dis	High-incor	Both	50-69 yea	HIV/AIDS	Number	2019	9295.96	11860.7	7410.47
DALYs (Dis	High-incor	Both	50-69 yea	HIV/AIDS	Rate	2019	18.7584	23.9338	14.9536
DALYs (Dis	North Afric	Both	50-69 yea	Sexually tra	Number	2018	8181.63	15743.7	4226.68
DALYs (Dis	North Afric	Both	50-69 yea	Sexually tra	Rate	2018	10.6675	20.5271	5.51086
DALYs (Dis	East Asia	Both	50-69 yea	HIV/AIDS	Number	2018	373848	462308	295075
DALYs (Dis	East Asia	Both	50-69 yea	HIV/AIDS	Rate	2018	100.804	124.656	79.5637
DALYs (Dis	Eastern Su	Both	50-69 yea	HIV/AIDS	Number	2016	1727413	2090630	1503057
DALYs (Dis	Eastern Su	Both	50-69 yea	HIV/AIDS	Rate	2016	6539.3	7914.3	5689.98
DALYs (Dis	Eastern Su	Both	50-69 yea	Sexually tra	Number	2016	17081.8	25901.1	9646.33
DALYs (Dis	Eastern Su	Both	50-69 yea	Sexually tra	Rate	2016	64.6648	98.0513	36.5172
DALYs (Dis	High-incor	Both	50-69 yea	HIV/AIDS	Number	2018	9417.24	12053.1	7500.2
DALYs (Dis	High-incor	Both	50-69 yea	HIV/AIDS	Rate	2018	19.0428	24.3729	15.1663
DALYs (Dis	Tropical La	Both	50-69 yea	Sexually tra	Number	2019	10783.8	20945.9	5697.55
DALYs (Dis	Tropical La	Both	50-69 yea	Sexually tra	Rate	2019	26.1127	50.7198	13.7965
DALYs (Dis	Andean La	Both	50-69 yea	HIV/AIDS	Number	2017	22734.3	39269.7	15603.4
DALYs (Dis	Andean La	Both	50-69 yea	HIV/AIDS	Rate	2017	261.718	452.073	179.626
DALYs (Dis	Caribbean	Both	50-69 yea	HIV/AIDS	Number	2017	86252.4	102389	73348.6
DALYs (Dis	Caribbean	Both	50-69 yea	HIV/AIDS	Rate	2017	1059.15	1257.3	900.694
DALYs (Dis	Oceania	Both	50-69 yea	HIV/AIDS	Number	2019	29527	77705.1	12569.8
DALYs (Dis	Oceania	Both	50-69 yea	HIV/AIDS	Rate	2019	2131.97	5610.62	907.593
DALYs (Dis	Central Lat	Both	50-69 yea	HIV/AIDS	Number	2017	98181.8	104356	92386.1
DALYs (Dis	Central Lat	Both	50-69 yea	HIV/AIDS	Rate	2017	261.373	277.809	245.945
DALYs (Dis	Central Lat	Both	50-69 yea	Sexually tra	Number	2017	9154.13	17505.4	5036.02
DALYs (Dis	Central Lat	Both	50-69 yea	Sexually tra	Rate	2017	24.3696	46.6019	13.4066
DALYs (Dis	Central As	Both	50-69 yea	HIV/AIDS	Number	2019	5697.55	6345.53	5155.05
DALYs (Dis	Central As	Both	50-69 yea	HIV/AIDS	Rate	2019	37.9812	42.3008	34.3647
DALYs (Dis	Australasia	Both	50-69 yea	Sexually tra	Number	2019	559.349	1174.9	260.153
DALYs (Dis	Australasia	Both	50-69 yea	Sexually tra	Rate	2019	8.29964	17.4332	3.86016
DALYs (Dis	South Asia	Both	50-69 yea	HIV/AIDS	Number	2019	333390	527041	244567
DALYs (Dis	South Asia	Both	50-69 yea	HIV/AIDS	Rate	2019	135.983	214.969	99.7537
DALYs (Dis	South Asia	Both	50-69 yea	Sexually tra	Number	2019	59790.6	83527.4	42249.1
DALYs (Dis	South Asia	Both	50-69 yea	Sexually tra	Rate	2019	24.3874	34.0691	17.2325
DALYs (Dis	Western Eu	Both	50-69 yea	HIV/AIDS	Number	2017	66016.7	77205	57463.5
DALYs (Dis	Western Eu	Both	50-69 yea	HIV/AIDS	Rate	2017	59.3339	69.3896	51.6465
DALYs (Dis	Western Su	Both	50-69 yea	HIV/AIDS	Number	2018	1250931	1441113	1102025
DALYs (Dis	Western Su	Both	50-69 yea	HIV/AIDS	Rate	2018	3700.47	4263.06	3259.98
DALYs (Dis	Andean La	Both	50-69 yea	Sexually tra	Number	2018	1733.94	3427.23	896.328
DALYs (Dis	Andean La	Both	50-69 yea	Sexually tra	Rate	2018	19.3514	38.2491	10.0033
DALYs (Dis	Australasia	Both	50-69 yea	HIV/AIDS	Number	2018	1680.04	2020.17	1408.24
DALYs (Dis	Australasia	Both	50-69 yea	HIV/AIDS	Rate	2018	25.249	30.3606	21.1641
DALYs (Dis	Eastern Eu	Both	50-69 yea	HIV/AIDS	Number	2017	103934	108090	99548.3
DALYs (Dis	Eastern Eu	Both	50-69 yea	HIV/AIDS	Rate	2017	189.589	197.169	181.589
DALYs (Dis	Central Eu	Both	50-69 yea	HIV/AIDS	Number	2019	4874.72	5761.39	4414.68
DALYs (Dis	Central Eu	Both	50-69 yea	HIV/AIDS	Rate	2019	16.3076	19.2738	14.7686
DALYs (Dis	Southeast	Both	50-69 yea	HIV/AIDS	Number	2017	369250	491721	303005
DALYs (Dis	Southeast	Both	50-69 yea	HIV/AIDS	Rate	2017	341.972	455.395	280.621
DALYs (Dis	Western Su	Both	50-69 yea	Sexually tra	Number	2019	8147.49	15944.7	4115.28
DALYs (Dis	Western Su	Both	50-69 yea	Sexually tra	Rate	2019	23.1779	45.3595	11.7071
DALYs (Dis	North Afric	Both	50-69 yea	HIV/AIDS	Number	2019	60120.9	118512	35370
DALYs (Dis	North Afric	Both	50-69 yea	HIV/AIDS	Rate	2019	75.4408	148.711	44.383
DALYs (Dis	Central Su	Both	50-69 yea	HIV/AIDS	Number	2019	273590	337663	221301
DALYs (Dis	Central Su	Both	50-69 yea	HIV/AIDS	Rate	2019	2637.21	3254.83	2133.18
DALYs (Dis	Central Su	Both	50-69 yea	Sexually tra	Number	2018	4868.81	9042.46	2387.95
DALYs (Dis	Central Su	Both	50-69 yea	Sexually tra	Rate	2018	48.8411	90.7088	23.9546
DALYs (Dis	Central Lat	Both	50-69 yea	HIV/AIDS	Number	2019	98648.3	105611	92335.3
DALYs (Dis	Central Lat	Both	50-69 yea	HIV/AIDS	Rate	2019	247.254	264.706	231.431
DALYs (Dis	Central Lat	Both	50-69 yea	Sexually tra	Number	2019	9517.22	18135.8	5211.95
DALYs (Dis	Central Lat	Both	50-69 yea	Sexually tra	Rate	2019	23.8542	45.4559	13.0634
DALYs (Dis	Tropical La	Both	50-69 yea	HIV/AIDS	Number	2018	171493	184172	160169
DALYs (Dis	Tropical La	Both	50-69 yea	HIV/AIDS	Rate	2018	425.846	457.33	397.726
DALYs (Dis	Southern L	Both	50-69 yea	HIV/AIDS	Number	2019	27050.3	31206.2	24376.6
DALYs (Dis	Southern L	Both	50-69 yea	HIV/AIDS	Rate	2019	215.046	248.085	193.79
DALYs (Dis	Southern S	Both	50-69 yea	HIV/AIDS	Number	2019	1621763	1902030	1417256
DALYs (Dis	Southern S	Both	50-69 yea	HIV/AIDS	Rate	2019	16210	19011.4	14165.9
DALYs (Dis	Western Eu	Both	50-69 yea	HIV/AIDS	Number	2019	65212.7	76561.8	56302.8
DALYs (Dis	Western Eu	Both	50-69 yea	HIV/AIDS	Rate	2019	57.6293	67.6586	49.7555
Prevalence	East Asia	Both	50-69 yea	Sexually tra	Number	1990	3.5E+07	4E+07	3E+07
Prevalence	East Asia	Both	50-69 yea	Sexually tra	Rate	1990	21731.8	25105	18701.2
Prevalence	East Asia	Both	50-69 yea	Sexually tra	Number	1991	3.7E+07	4.2E+07	3.2E+07
Prevalence	East Asia	Both	50-69 yea	Sexually tra	Rate	1991	22525	26053.2	19417.7
Prevalence	East Asia	Both	50-69 yea	Sexually tra	Number	1992	3.8E+07	4.4E+07	3.3E+07
Prevalence	East Asia	Both	50-69 yea	Sexually tra	Rate	1992	23130.9	26817.5	19944.1
Prevalence	East Asia	Both	50-69 yea	Sexually tra	Number	1993	4E+07	4.6E+07	3.4E+07
Prevalence	East Asia	Both	50-69 yea	Sexually tra	Rate	1993	23547.3	27239.6	20279
Prevalence	East Asia	Both	50-69 yea	Sexually tra	Number	1994	4.1E+07	4.7E+07	3.5E+07
Prevalence	East Asia	Both	50-69 yea	Sexually tra	Rate	1994	23791.7	27457	20445.8
Prevalence	East Asia	Both	50-69 yea	Sexually tra	Number	1995	4.1E+07	4.8E+07	3.6E+07
Prevalence	East Asia	Both	50-69 yea	Sexually tra	Rate	1995	23880.1	27498.6	20517
Prevalence	East Asia	Both	50-69 yea	Sexually tra	Number	1996	4.2E+07	4.8E+07	3.6E+07
Prevalence	East Asia	Both	50-69 yea	Sexually tra	Rate	1996	23653.2	27271.8	20344.3
Prevalence	East Asia	Both	50-69 yea	Sexually tra	Number	1997	4.2E+07	4.9E+07	3.6E+07
Prevalence	East Asia	Both	50-69 yea	Sexually tra	Rate	1997	23081.7	26689.1	19850.8
Prevalence	East Asia	Both	50-69 yea	Sexually tra	Number	1998	4.2E+07	4.8E+07	3.6E+07
Prevalence	East Asia	Both	50-69 yea	Sexually tra	Rate	1998	22406.6	25838.6	19334.8
Prevalence	East Asia	Both	50-69 yea	Sexually tra	Number	1999	4.2E+07	4.9E+07	3.7E+07
Prevalence	East Asia	Both	50-69 yea	Sexually tra	Rate	1999	21849.9	25157.6	18858.6
Prevalence	East Asia	Both	50-69 yea	Sexually tra	Number	2000	4.3E+07	4.9E+07	3.7E+07
Prevalence	East Asia	Both	50-69 yea	Sexually tra	Rate	2000	21615	24893.7	18616.3
Prevalence	East Asia	Both	50-69 yea	Sexually tra	Number	2001	4.5E+07	5.1E+07	3.8E+07
Prevalence	East Asia	Both	50-69 yea	Sexually tra	Rate	2001	21643.7	24824.5	18653
Prevalence	East Asia	Both	50-69 yea	Sexually tra	Number	2002	4.6E+07	5.3E+07	4E+07
Prevalence	East Asia	Both	50-69 yea	Sexually tra	Rate	2002	21684.7	24938.2	18697.6
Prevalence	East Asia	Both	50-69 yea	Sexually tra	Number	2003	4.8E+07	5.6E+07	4.2E+07
Prevalence	East Asia	Both	50-69 yea	Sexually tra	Rate	2003	21744.4	25019.9	18745.9
Prevalence	East Asia	Both	50-69 yea	Sexually tra	Number	2004	5E+07	5.8E+07	4.4E+07
Prevalence	East Asia	Both	50-69 yea	Sexually tra	Rate	2004	21788.7	25053.7	18851
Prevalence	East Asia	Both	50-69 yea	Sexually tra	Number	2005	5.3E+07	6.1E+07	4.5E+07
Prevalence	East Asia	Both	50-69 yea	Sexually tra	Rate	2005	21828.8	25131	18858.3
Prevalence	East Asia	Both	50-69 yea	Sexually tra	Number	2006	5.4E+07	6.2E+07	4.7E+07
Prevalence	East Asia	Both	50-69 yea	Sexually tra	Rate	2006	21590.7	24808.5	18646
Prevalence	East Asia	Both	50-69 yea	Sexually tra	Number	2007	5.4E+07	6.2E+07	4.7E+07
Prevalence	East Asia	Both	50-69 yea	Sexually tra	Rate	2007	21015.4	24197.5	18115.3
Prevalence	East Asia	Both	50-69 yea	Sexually tra	Number	2008	5.4E+07	6.2E+07	4.6E+07
Prevalence	East Asia	Both	50-69 yea	Sexually tra	Rate	2008	20319.1	23398.2	17497.7
Prevalence	East Asia	Both	50-69 yea	Sexually tra	Number	2009	5.3E+07	6.1E+07	4.6E+07
Prevalence	East Asia	Both	50-69 yea	Sexually tra	Rate	2009	19721.2	22761.5	16946.2
Prevalence	East Asia	Both	50-69 yea	Sexually tra	Number	2010	5.4E+07	6.2E+07	4.6E+07
Prevalence	East Asia	Both	50-69 yea	Sexually tra	Rate	2010	19463.7	22445.8	16728.1
Prevalence	East Asia	Both	50-69 yea	Sexually tra	Number	2011	5.6E+07	6.4E+07	4.8E+07
Prevalence	East Asia	Both	50-69 yea	Sexually tra	Rate	2011	19669	22684	16908.3
Prevalence	East Asia	Both	50-69 yea	Sexually tra	Number	2012	5.9E+07	6.8E+07	5.1E+07
Prevalence	East Asia	Both	50-69 yea	Sexually tra	Rate	2012	20205.7	23282	17396
Prevalence	East Asia	Both	50-69 yea	Sexually tra	Number	2013	6.3E+07	7.3E+07	5.5E+07
Prevalence	East Asia	Both	50-69 yea	Sexually tra	Rate	2013	20865.3	24061.4	17931.7
Prevalence	East Asia	Both	50-69 yea	Sexually tra	Number	2014	6.8E+07	7.9E+07	5.8E+07
Prevalence	East Asia	Both	50-69 yea	Sexually tra	Rate	2014	21423.9	24713.3	18365.5
Prevalence	East Asia	Both	50-69 yea	Sexually tra	Number	2015	7.2E+07	8.3E+07	6.2E+07
Prevalence	East Asia	Both	50-69 yea	Sexually tra	Rate	2015	21681.3	25037	18619.4
Prevalence	East Asia	Both	50-69 yea	Sexually tra	Number	2016	7.5E+07	8.6E+07	6.4E+07
Prevalence	East Asia	Both	50-69 yea	Sexually tra	Rate	2016	21675.9	25008.3	18618.7
Prevalence	East Asia	Both	50-69 yea	Sexually tra	Number	2017	7.8E+07	9E+07	6.7E+07
Prevalence	East Asia	Both	50-69 yea	Sexually tra	Rate	2017	21666.1	24990.3	18598.3
Prevalence	East Asia	Both	50-69 yea	Sexually tra	Number	2018	8.1E+07	9.3E+07	6.9E+07
Prevalence	East Asia	Both	50-69 yea	Sexually tra	Rate	2018	21721.2	25076	18656.3
Prevalence	East Asia	Both	50-69 yea	Sexually tra	Number	2019	8.3E+07	9.6E+07	7.1E+07
Prevalence	East Asia	Both	50-69 yea	Sexually tra	Rate	2019	21842.3	25239.8	18729.8
Prevalence	Southeast	Both	50-69 yea	Sexually tra	Number	1990	1.4E+07	1.6E+07	1.2E+07
Prevalence	Southeast	Both	50-69 yea	Sexually tra	Rate	1990	29337.8	33245.6	25828.8
Prevalence	Southeast	Both	50-69 yea	Sexually tra	Number	1991	1.4E+07	1.6E+07	1.3E+07
Prevalence	Southeast	Both	50-69 yea	Sexually tra	Rate	1991	29463.4	33385.8	25957.8
Prevalence	Southeast	Both	50-69 yea	Sexually tra	Number	1992	1.5E+07	1.7E+07	1.3E+07
Prevalence	Southeast	Both	50-69 yea	Sexually tra	Rate	1992	29568.8	33547.7	26046
Prevalence	Southeast	Both	50-69 yea	Sexually tra	Number	1993	1.5E+07	1.7E+07	1.3E+07
Prevalence	Southeast	Both	50-69 yea	Sexually tra	Rate	1993	29648.3	33576.4	26088
Prevalence	Southeast	Both	50-69 yea	Sexually tra	Number	1994	1.5E+07	1.7E+07	1.3E+07
Prevalence	Southeast	Both	50-69 yea	Sexually tra	Rate	1994	29696.2	33672.4	26133.6
Prevalence	Southeast	Both	50-69 yea	Sexually tra	Number	1995	1.6E+07	1.8E+07	1.4E+07
Prevalence	Southeast	Both	50-69 yea	Sexually tra	Rate	1995	29709.8	33686.4	26154.3
Prevalence	Southeast	Both	50-69 yea	Sexually tra	Number	1996	1.6E+07	1.8E+07	1.4E+07
Prevalence	Southeast	Both	50-69 yea	Sexually tra	Rate	1996	29685	33650.1	26089.6
Prevalence	Southeast	Both	50-69 yea	Sexually tra	Number	1997	1.6E+07	1.8E+07	1.4E+07
Prevalence	Southeast	Both	50-69 yea	Sexually tra	Rate	1997	29635.5	33598.2	26107.8
Prevalence	Southeast	Both	50-69 yea	Sexually tra	Number	1998	1.7E+07	1.9E+07	1.5E+07
Prevalence	Southeast	Both	50-69 yea	Sexually tra	Rate	1998	29574	33545.2	26045.7
Prevalence	Southeast	Both	50-69 yea	Sexually tra	Number	1999	1.7E+07	1.9E+07	1.5E+07
Prevalence	Southeast	Both	50-69 yea	Sexually tra	Rate	1999	29522.6	33511.9	25983.1
Prevalence	Southeast	Both	50-69 yea	Sexually tra	Number	2000	1.7E+07	2E+07	1.5E+07
Prevalence	Southeast	Both	50-69 yea	Sexually tra	Rate	2000	29491.6	33471.4	25982.3
Prevalence	Southeast	Both	50-69 yea	Sexually tra	Number	2001	1.8E+07	2E+07	1.6E+07
Prevalence	Southeast	Both	50-69 yea	Sexually tra	Rate	2001	29501.1	33480.9	25992.5
Prevalence	Southeast	Both	50-69 yea	Sexually tra	Number	2002	1.8E+07	2.1E+07	1.6E+07
Prevalence	Southeast	Both	50-69 yea	Sexually tra	Rate	2002	29527.7	33478.6	25994.3
Prevalence	Southeast	Both	50-69 yea	Sexually tra	Number	2003	1.9E+07	2.2E+07	1.7E+07
Prevalence	Southeast	Both	50-69 yea	Sexually tra	Rate	2003	29557	33506.5	26004
Prevalence	Southeast	Both	50-69 yea	Sexually tra	Number	2004	2E+07	2.2E+07	1.7E+07
Prevalence	Southeast	Both	50-69 yea	Sexually tra	Rate	2004	29582.8	33538.2	26036.4
Prevalence	Southeast	Both	50-69 yea	Sexually tra	Number	2005	2E+07	2.3E+07	1.8E+07
Prevalence	Southeast	Both	50-69 yea	Sexually tra	Rate	2005	29605.9	33563.8	26007.1
Prevalence	Southeast	Both	50-69 yea	Sexually tra	Number	2006	2.1E+07	2.4E+07	1.8E+07
Prevalence	Southeast	Both	50-69 yea	Sexually tra	Rate	2006	29567.9	33563.9	25973.2
Prevalence	Southeast	Both	50-69 yea	Sexually tra	Number	2007	2.2E+07	2.5E+07	1.9E+07
Prevalence	Southeast	Both	50-69 yea	Sexually tra	Rate	2007	29475.8	33494.8	25807.6
Prevalence	Southeast	Both	50-69 yea	Sexually tra	Number	2008	2.3E+07	2.6E+07	2E+07
Prevalence	Southeast	Both	50-69 yea	Sexually tra	Rate	2008	29358.3	33524.1	25613.3
Prevalence	Southeast	Both	50-69 yea	Sexually tra	Number	2009	2.3E+07	2.7E+07	2E+07
Prevalence	Southeast	Both	50-69 yea	Sexually tra	Rate	2009	29256.8	33540.8	25461.8
Prevalence	Southeast	Both	50-69 yea	Sexually tra	Number	2010	2.4E+07	2.8E+07	2.1E+07
Prevalence	Southeast	Both	50-69 yea	Sexually tra	Rate	2010	29223.4	33568.4	25390.8
Prevalence	Southeast	Both	50-69 yea	Sexually tra	Number	2011	2.5E+07	2.9E+07	2.2E+07
Prevalence	Southeast	Both	50-69 yea	Sexually tra	Rate	2011	29227.1	33555.4	25380.6
Prevalence	Southeast	Both	50-69 yea	Sexually tra	Number	2012	2.6E+07	3E+07	2.3E+07
Prevalence	Southeast	Both	50-69 yea	Sexually tra	Rate	2012	29236.6	33499.5	25404.3
Prevalence	Southeast	Both	50-69 yea	Sexually tra	Number	2013	2.7E+07	3.1E+07	2.4E+07
Prevalence	Southeast	Both	50-69 yea	Sexually tra	Rate	2013	29238.7	33496	25417.7
Prevalence	Southeast	Both	50-69 yea	Sexually tra	Number	2014	2.8E+07	3.3E+07	2.5E+07
Prevalence	Southeast	Both	50-69 yea	Sexually tra	Rate	2014	29233.7	33499	25414.9
Prevalence	Southeast	Both	50-69 yea	Sexually tra	Number	2015	2.9E+07	3.4E+07	2.6E+07
Prevalence	Southeast	Both	50-69 yea	Sexually tra	Rate	2015	29220.2	33477.2	25395.3
Prevalence	Southeast	Both	50-69 yea	Sexually tra	Number	2016	3E+07	3.5E+07	2.6E+07
Prevalence	Southeast	Both	50-69 yea	Sexually tra	Rate	2016	29112.4	33428.8	25269.3
Prevalence	Southeast	Both	50-69 yea	Sexually tra	Number	2017	3.1E+07	3.6E+07	2.7E+07
Prevalence	Southeast	Both	50-69 yea	Sexually tra	Rate	2017	28999.5	33327.8	25221.2
Prevalence	Southeast	Both	50-69 yea	Sexually tra	Number	2018	3.2E+07	3.7E+07	2.8E+07
Prevalence	Southeast	Both	50-69 yea	Sexually tra	Rate	2018	29011.5	33319.6	25207.3
Prevalence	Southeast	Both	50-69 yea	Sexually tra	Number	2019	3.4E+07	3.9E+07	2.9E+07
Prevalence	Southeast	Both	50-69 yea	Sexually tra	Rate	2019	29097.9	33467.1	25285.2
Prevalence	Eastern Eu	Both	50-69 yea	HIV/AIDS	Number	1990	7066.3	9007.51	5273.04
Prevalence	Eastern Eu	Both	50-69 yea	HIV/AIDS	Rate	1990	14.2498	18.1644	10.6336
Prevalence	Eastern Eu	Both	50-69 yea	HIV/AIDS	Number	1991	7774.76	9947.19	5888.53
Prevalence	Eastern Eu	Both	50-69 yea	HIV/AIDS	Rate	1991	15.4118	19.7182	11.6727
Prevalence	Eastern Eu	Both	50-69 yea	HIV/AIDS	Number	1992	8226.22	10500.4	6347.59
Prevalence	Eastern Eu	Both	50-69 yea	HIV/AIDS	Rate	1992	16.16	20.6276	12.4695
Prevalence	Eastern Eu	Both	50-69 yea	HIV/AIDS	Number	1993	8270.53	10662.7	6363.43
Prevalence	Eastern Eu	Both	50-69 yea	HIV/AIDS	Rate	1993	16.4991	21.2714	12.6946
Prevalence	Eastern Eu	Both	50-69 yea	HIV/AIDS	Number	1994	8142.54	10649	6230.04
Prevalence	Eastern Eu	Both	50-69 yea	HIV/AIDS	Rate	1994	16.7185	21.8649	12.7917
Prevalence	Eastern Eu	Both	50-69 yea	HIV/AIDS	Number	1995	7946.61	10545.5	6073.29
Prevalence	Eastern Eu	Both	50-69 yea	HIV/AIDS	Rate	1995	16.8637	22.3789	12.8883
Prevalence	Eastern Eu	Both	50-69 yea	HIV/AIDS	Number	1996	7995.83	10722.8	6029.6
Prevalence	Eastern Eu	Both	50-69 yea	HIV/AIDS	Rate	1996	17.4245	23.3671	13.1397
Prevalence	Eastern Eu	Both	50-69 yea	HIV/AIDS	Number	1997	8498.81	11445.5	6399.19
Prevalence	Eastern Eu	Both	50-69 yea	HIV/AIDS	Rate	1997	18.7413	25.2393	14.1113
Prevalence	Eastern Eu	Both	50-69 yea	HIV/AIDS	Number	1998	9238.47	12346.1	6993.39
Prevalence	Eastern Eu	Both	50-69 yea	HIV/AIDS	Rate	1998	20.5741	27.4948	15.5743
Prevalence	Eastern Eu	Both	50-69 yea	HIV/AIDS	Number	1999	10209.2	13653.4	7747.24
Prevalence	Eastern Eu	Both	50-69 yea	HIV/AIDS	Rate	1999	22.8567	30.5677	17.3448
Prevalence	Eastern Eu	Both	50-69 yea	HIV/AIDS	Number	2000	11299.5	15128.4	8459.72
Prevalence	Eastern Eu	Both	50-69 yea	HIV/AIDS	Rate	2000	25.2147	33.759	18.8778
Prevalence	Eastern Eu	Both	50-69 yea	HIV/AIDS	Number	2001	12303.7	16563.6	9063.64
Prevalence	Eastern Eu	Both	50-69 yea	HIV/AIDS	Rate	2001	27.3616	36.8351	20.1562
Prevalence	Eastern Eu	Both	50-69 yea	HIV/AIDS	Number	2002	13253.2	18015.2	9688.02
Prevalence	Eastern Eu	Both	50-69 yea	HIV/AIDS	Rate	2002	29.2062	39.7001	21.3495
Prevalence	Eastern Eu	Both	50-69 yea	HIV/AIDS	Number	2003	14290.8	19533.9	10374.4
Prevalence	Eastern Eu	Both	50-69 yea	HIV/AIDS	Rate	2003	31.2023	42.6501	22.6513
Prevalence	Eastern Eu	Both	50-69 yea	HIV/AIDS	Number	2004	15319.8	20970.3	11111.2
Prevalence	Eastern Eu	Both	50-69 yea	HIV/AIDS	Rate	2004	32.9593	45.1159	23.9049
Prevalence	Eastern Eu	Both	50-69 yea	HIV/AIDS	Number	2005	16366.9	22354.9	11984.9
Prevalence	Eastern Eu	Both	50-69 yea	HIV/AIDS	Rate	2005	34.7346	47.4426	25.4349
Prevalence	Eastern Eu	Both	50-69 yea	HIV/AIDS	Number	2006	17389.8	23711.2	13020.8
Prevalence	Eastern Eu	Both	50-69 yea	HIV/AIDS	Rate	2006	36.6052	49.9114	27.4085
Prevalence	Eastern Eu	Both	50-69 yea	HIV/AIDS	Number	2007	18506.9	25069.8	14184.7
Prevalence	Eastern Eu	Both	50-69 yea	HIV/AIDS	Rate	2007	38.7578	52.502	29.7061
Prevalence	Eastern Eu	Both	50-69 yea	HIV/AIDS	Number	2008	20121	26833.5	15723.4
Prevalence	Eastern Eu	Both	50-69 yea	HIV/AIDS	Rate	2008	41.8942	55.8703	32.7379
Prevalence	Eastern Eu	Both	50-69 yea	HIV/AIDS	Number	2009	22274	28942.9	17782.5
Prevalence	Eastern Eu	Both	50-69 yea	HIV/AIDS	Rate	2009	45.9825	59.7498	36.7102
Prevalence	Eastern Eu	Both	50-69 yea	HIV/AIDS	Number	2010	25241.7	31698.4	20584.5
Prevalence	Eastern Eu	Both	50-69 yea	HIV/AIDS	Rate	2010	51.3525	64.4882	41.8778

Prevalence	Oceania	Both	50-69 yea	Sexually tr	Rate	1998	38692.3	42298.5	35099
Prevalence	Oceania	Both	50-69 yea	Sexually tr	Number	1999	262281	285107	238980
Prevalence	Oceania	Both	50-69 yea	Sexually tr	Rate	1999	38931.2	42319.3	35472.6
Prevalence	Oceania	Both	50-69 yea	Sexually tr	Number	2000	267163	289957	243950
Prevalence	Oceania	Both	50-69 yea	Sexually tr	Rate	2000	38992.6	42319.3	35604.7
Prevalence	Oceania	Both	50-69 yea	Sexually tr	Number	2001	277121	299912	253802
Prevalence	Oceania	Both	50-69 yea	Sexually tr	Rate	2001	39107.7	42324.1	35817
Prevalence	Oceania	Both	50-69 yea	Sexually tr	Number	2002	286483	309270	262371
Prevalence	Oceania	Both	50-69 yea	Sexually tr	Rate	2002	39164.3	42279.5	35868
Prevalence	Oceania	Both	50-69 yea	Sexually tr	Number	2003	296429	319715	271778
Prevalence	Oceania	Both	50-69 yea	Sexually tr	Rate	2003	39215.1	42295.8	35954
Prevalence	Oceania	Both	50-69 yea	Sexually tr	Number	2004	307016	330449	281944
Prevalence	Oceania	Both	50-69 yea	Sexually tr	Rate	2004	39259.2	42255.6	36053.1
Prevalence	Oceania	Both	50-69 yea	Sexually tr	Number	2005	318325	342211	293262
Prevalence	Oceania	Both	50-69 yea	Sexually tr	Rate	2005	39293.9	42242.4	36200.1
Prevalence	Oceania	Both	50-69 yea	Sexually tr	Number	2006	329463	356237	301774
Prevalence	Oceania	Both	50-69 yea	Sexually tr	Rate	2006	39131.2	42311.3	35842.5
Prevalence	Oceania	Both	50-69 yea	Sexually tr	Number	2007	338226	369114	307260
Prevalence	Oceania	Both	50-69 yea	Sexually tr	Rate	2007	38719.7	42255.7	35174.7
Prevalence	Oceania	Both	50-69 yea	Sexually tr	Number	2008	346520	384357	310905
Prevalence	Oceania	Both	50-69 yea	Sexually tr	Rate	2008	38225.9	42399.8	34297
Prevalence	Oceania	Both	50-69 yea	Sexually tr	Number	2009	356026	401292	315470
Prevalence	Oceania	Both	50-69 yea	Sexually tr	Rate	2009	37831.5	42641.6	33522
Prevalence	Oceania	Both	50-69 yea	Sexually tr	Number	2010	368334	418733	321978
Prevalence	Oceania	Both	50-69 yea	Sexually tr	Rate	2010	37686.3	42842.8	32943.4
Prevalence	Oceania	Both	50-69 yea	Sexually tr	Number	2011	383768	436350	335985
Prevalence	Oceania	Both	50-69 yea	Sexually tr	Rate	2011	37682.9	42845.9	32991
Prevalence	Oceania	Both	50-69 yea	Sexually tr	Number	2012	399373	454869	349439
Prevalence	Oceania	Both	50-69 yea	Sexually tr	Rate	2012	37700.5	42939.2	32986.7
Prevalence	Oceania	Both	50-69 yea	Sexually tr	Number	2013	415868	473434	364835
Prevalence	Oceania	Both	50-69 yea	Sexually tr	Rate	2013	37726.5	42948.8	33097
Prevalence	Oceania	Both	50-69 yea	Sexually tr	Number	2014	433138	491755	380076
Prevalence	Oceania	Both	50-69 yea	Sexually tr	Rate	2014	37761.7	42872	33135.6
Prevalence	Oceania	Both	50-69 yea	Sexually tr	Number	2015	451010	513358	395662
Prevalence	Oceania	Both	50-69 yea	Sexually tr	Rate	2015	37798.4	43023.7	33159.8
Prevalence	Oceania	Both	50-69 yea	Sexually tr	Number	2016	468055	531210	409943
Prevalence	Oceania	Both	50-69 yea	Sexually tr	Rate	2016	37712	42800.4	33029.7
Prevalence	Oceania	Both	50-69 yea	Sexually tr	Number	2017	484204	549185	424885
Prevalence	Oceania	Both	50-69 yea	Sexually tr	Rate	2017	37626	42675.4	33016.5
Prevalence	Oceania	Both	50-69 yea	Sexually tr	Number	2018	502845	569614	441624
Prevalence	Oceania	Both	50-69 yea	Sexually tr	Rate	2018	37684.1	42687.8	33096
Prevalence	Oceania	Both	50-69 yea	Sexually tr	Number	2019	523991	592707	461836
Prevalence	Oceania	Both	50-69 yea	Sexually tr	Rate	2019	37834.3	42795.9	33346.5
Prevalence	East Asia	Both	50-69 yea	HIV/AIDS	Number	1990	10033.8	12948.5	7597.46
Prevalence	East Asia	Both	50-69 yea	HIV/AIDS	Rate	1990	6.26387	8.08341	4.74291
Prevalence	East Asia	Both	50-69 yea	HIV/AIDS	Number	1991	10964.8	14137.5	8832.28
Prevalence	East Asia	Both	50-69 yea	HIV/AIDS	Rate	1991	6.73175	8.67962	5.4225
Prevalence	East Asia	Both	50-69 yea	HIV/AIDS	Number	1992	11953.2	15683.9	9771.88
Prevalence	East Asia	Both	50-69 yea	HIV/AIDS	Rate	1992	7.22164	9.47558	5.90376
Prevalence	East Asia	Both	50-69 yea	HIV/AIDS	Number	1993	12985.6	17708.1	10455.9
Prevalence	East Asia	Both	50-69 yea	HIV/AIDS	Rate	1993	7.72348	10.5323	6.21885
Prevalence	East Asia	Both	50-69 yea	HIV/AIDS	Number	1994	14177.6	20120	11236.1
Prevalence	East Asia	Both	50-69 yea	HIV/AIDS	Rate	1994	8.3062	11.7877	6.58286
Prevalence	East Asia	Both	50-69 yea	HIV/AIDS	Number	1995	15514.6	22799.2	11940.5
Prevalence	East Asia	Both	50-69 yea	HIV/AIDS	Rate	1995	8.93469	13.1298	6.87638
Prevalence	East Asia	Both	50-69 yea	HIV/AIDS	Number	1996	16872.8	25922.7	12580.6
Prevalence	East Asia	Both	50-69 yea	HIV/AIDS	Rate	1996	9.4926	14.584	7.07782
Prevalence	East Asia	Both	50-69 yea	HIV/AIDS	Number	1997	18514.8	29586.3	13370.3
Prevalence	East Asia	Both	50-69 yea	HIV/AIDS	Rate	1997	10.154	16.2259	7.33261
Prevalence	East Asia	Both	50-69 yea	HIV/AIDS	Number	1998	20726.7	34595.4	14433.1
Prevalence	East Asia	Both	50-69 yea	HIV/AIDS	Rate	1998	11.0902	18.5109	7.7227
Prevalence	East Asia	Both	50-69 yea	HIV/AIDS	Number	1999	24084.4	41223.8	15910.9
Prevalence	East Asia	Both	50-69 yea	HIV/AIDS	Rate	1999	12.44	21.2928	8.21826
Prevalence	East Asia	Both	50-69 yea	HIV/AIDS	Number	2000	28513.4	51114.4	17894.6
Prevalence	East Asia	Both	50-69 yea	HIV/AIDS	Rate	2000	14.3661	25.7532	9.01592
Prevalence	East Asia	Both	50-69 yea	HIV/AIDS	Number	2001	34885.9	63283.3	20935.5
Prevalence	East Asia	Both	50-69 yea	HIV/AIDS	Rate	2001	16.9491	30.7458	10.1714
Prevalence	East Asia	Both	50-69 yea	HIV/AIDS	Number	2002	43112.9	77451.7	24875.5
Prevalence	East Asia	Both	50-69 yea	HIV/AIDS	Rate	2002	20.1948	36.2796	11.6521
Prevalence	East Asia	Both	50-69 yea	HIV/AIDS	Number	2003	53540.6	96554.6	29872.2
Prevalence	East Asia	Both	50-69 yea	HIV/AIDS	Rate	2003	24.1033	43.4678	13.4481
Prevalence	East Asia	Both	50-69 yea	HIV/AIDS	Number	2004	66345.8	117793	35946.9
Prevalence	East Asia	Both	50-69 yea	HIV/AIDS	Rate	2004	28.6701	50.9022	15.5338
Prevalence	East Asia	Both	50-69 yea	HIV/AIDS	Number	2005	80416.9	140641	42354.1
Prevalence	East Asia	Both	50-69 yea	HIV/AIDS	Rate	2005	33.3829	58.3831	17.5821
Prevalence	East Asia	Both	50-69 yea	HIV/AIDS	Number	2006	94692.5	164288	47395.7
Prevalence	East Asia	Both	50-69 yea	HIV/AIDS	Rate	2006	37.9219	65.7931	18.9807
Prevalence	East Asia	Both	50-69 yea	HIV/AIDS	Number	2007	108517	186498	54594.3
Prevalence	East Asia	Both	50-69 yea	HIV/AIDS	Rate	2007	42.1757	72.4838	21.2184
Prevalence	East Asia	Both	50-69 yea	HIV/AIDS	Number	2008	120629	205684	62021.2
Prevalence	East Asia	Both	50-69 yea	HIV/AIDS	Rate	2008	45.784	78.0661	23.5398
Prevalence	East Asia	Both	50-69 yea	HIV/AIDS	Number	2009	131155	222014	68649.3
Prevalence	East Asia	Both	50-69 yea	HIV/AIDS	Rate	2009	48.7154	82.4637	25.4987
Prevalence	East Asia	Both	50-69 yea	HIV/AIDS	Number	2010	140377	236676	74432.8
Prevalence	East Asia	Both	50-69 yea	HIV/AIDS	Rate	2010	50.9915	85.9715	27.0374
Prevalence	East Asia	Both	50-69 yea	HIV/AIDS	Number	2011	148796	249940	80244
Prevalence	East Asia	Both	50-69 yea	HIV/AIDS	Rate	2011	52.6184	88.3859	28.3766
Prevalence	East Asia	Both	50-69 yea	HIV/AIDS	Number	2012	156742	260460	85481.5
Prevalence	East Asia	Both	50-69 yea	HIV/AIDS	Rate	2012	53.6314	89.1199	29.2486
Prevalence	East Asia	Both	50-69 yea	HIV/AIDS	Number	2013	164554	272025	91061.7
Prevalence	East Asia	Both	50-69 yea	HIV/AIDS	Rate	2013	54.0917	89.419	29.9335
Prevalence	East Asia	Both	50-69 yea	HIV/AIDS	Number	2014	171617	281274	94767.2
Prevalence	East Asia	Both	50-69 yea	HIV/AIDS	Rate	2014	53.9859	88.481	29.8111
Prevalence	East Asia	Both	50-69 yea	HIV/AIDS	Number	2015	176788	290409	96341.1
Prevalence	East Asia	Both	50-69 yea	HIV/AIDS	Rate	2015	53.2353	87.4494	29.0107
Prevalence	East Asia	Both	50-69 yea	HIV/AIDS	Number	2016	179620	299700	96448.2
Prevalence	East Asia	Both	50-69 yea	HIV/AIDS	Rate	2016	51.9807	86.731	27.9114
Prevalence	East Asia	Both	50-69 yea	HIV/AIDS	Number	2017	180562	307087	96180.2
Prevalence	East Asia	Both	50-69 yea	HIV/AIDS	Rate	2017	50.3802	85.6831	26.8361
Prevalence	East Asia	Both	50-69 yea	HIV/AIDS	Number	2018	180263	309750	94893.1
Prevalence	East Asia	Both	50-69 yea	HIV/AIDS	Rate	2018	48.606	83.5204	25.5868
Prevalence	East Asia	Both	50-69 yea	HIV/AIDS	Number	2019	178363	311366	93633.7
Prevalence	East Asia	Both	50-69 yea	HIV/AIDS	Rate	2019	46.7957	81.6907	24.5659
Prevalence	Central Eu	Both	50-69 yea	HIV/AIDS	Number	1990	1084.6	1319.76	873.613
Prevalence	Central Eu	Both	50-69 yea	HIV/AIDS	Rate	1990	4.30946	5.24382	3.47114
Prevalence	Central Eu	Both	50-69 yea	HIV/AIDS	Number	1991	1118.37	1418.49	869.978
Prevalence	Central Eu	Both	50-69 yea	HIV/AIDS	Rate	1991	4.44433	5.637	3.45725
Prevalence	Central Eu	Both	50-69 yea	HIV/AIDS	Number	1992	1152.64	1502.14	863.609
Prevalence	Central Eu	Both	50-69 yea	HIV/AIDS	Rate	1992	4.60007	5.9949	3.44659
Prevalence	Central Eu	Both	50-69 yea	HIV/AIDS	Number	1993	1198.45	1582.92	870.36
Prevalence	Central Eu	Both	50-69 yea	HIV/AIDS	Rate	1993	4.81206	6.35581	3.4947
Prevalence	Central Eu	Both	50-69 yea	HIV/AIDS	Number	1994	1256	1663.06	895.808
Prevalence	Central Eu	Both	50-69 yea	HIV/AIDS	Rate	1994	5.07377	6.71815	3.61874
Prevalence	Central Eu	Both	50-69 yea	HIV/AIDS	Number	1995	1333.74	1747.2	955.924
Prevalence	Central Eu	Both	50-69 yea	HIV/AIDS	Rate	1995	5.42575	7.10777	3.88878
Prevalence	Central Eu	Both	50-69 yea	HIV/AIDS	Number	1996	1439.25	1853.55	1046.55
Prevalence	Central Eu	Both	50-69 yea	HIV/AIDS	Rate	1996	5.87723	7.569	4.2736
Prevalence	Central Eu	Both	50-69 yea	HIV/AIDS	Number	1997	1572.38	1965.62	1147.17
Prevalence	Central Eu	Both	50-69 yea	HIV/AIDS	Rate	1997	6.39929	7.99967	4.66876
Prevalence	Central Eu	Both	50-69 yea	HIV/AIDS	Number	1998	1735.64	2118.34	1296.56
Prevalence	Central Eu	Both	50-69 yea	HIV/AIDS	Rate	1998	7.01802	8.56549	5.24264
Prevalence	Central Eu	Both	50-69 yea	HIV/AIDS	Number	1999	1952.03	2327.96	1476.15
Prevalence	Central Eu	Both	50-69 yea	HIV/AIDS	Rate	1999	7.82895	9.33664	5.92036
Prevalence	Central Eu	Both	50-69 yea	HIV/AIDS	Number	2000	2211.73	2643.2	1755.97
Prevalence	Central Eu	Both	50-69 yea	HIV/AIDS	Rate	2000	8.77033	10.4813	6.96308
Prevalence	Central Eu	Both	50-69 yea	HIV/AIDS	Number	2001	2511.96	2992.57	2009.58
Prevalence	Central Eu	Both	50-69 yea	HIV/AIDS	Rate	2001	9.83789	11.7202	7.87038
Prevalence	Central Eu	Both	50-69 yea	HIV/AIDS	Number	2002	2849.64	3466.31	2249.76
Prevalence	Central Eu	Both	50-69 yea	HIV/AIDS	Rate	2002	11.0101	13.3927	8.69234
Prevalence	Central Eu	Both	50-69 yea	HIV/AIDS	Number	2003	3202.23	3934.39	2513.12
Prevalence	Central Eu	Both	50-69 yea	HIV/AIDS	Rate	2003	12.1854	14.9715	9.56313
Prevalence	Central Eu	Both	50-69 yea	HIV/AIDS	Number	2004	3558.24	4414.61	2770.3
Prevalence	Central Eu	Both	50-69 yea	HIV/AIDS	Rate	2004	13.3279	16.5356	10.3766
Prevalence	Central Eu	Both	50-69 yea	HIV/AIDS	Number	2005	3942.14	4922	3055.43
Prevalence	Central Eu	Both	50-69 yea	HIV/AIDS	Rate	2005	14.5108	18.1176	11.2469
Prevalence	Central Eu	Both	50-69 yea	HIV/AIDS	Number	2006	4335.22	5413.55	3362.42
Prevalence	Central Eu	Both	50-69 yea	HIV/AIDS	Rate	2006	15.6949	19.5988	12.1731
Prevalence	Central Eu	Both	50-69 yea	HIV/AIDS	Number	2007	4724.81	5883.25	3667.99
Prevalence	Central Eu	Both	50-69 yea	HIV/AIDS	Rate	2007	16.8561	20.989	13.0859
Prevalence	Central Eu	Both	50-69 yea	HIV/AIDS	Number	2008	5125.27	6384	3978.42
Prevalence	Central Eu	Both	50-69 yea	HIV/AIDS	Rate	2008	18.0533	22.487	14.0136
Prevalence	Central Eu	Both	50-69 yea	HIV/AIDS	Number	2009	5541.3	6880.74	4298.96
Prevalence	Central Eu	Both	50-69 yea	HIV/AIDS	Rate	2009	19.3056	23.9721	14.9773
Prevalence	Central Eu	Both	50-69 yea	HIV/AIDS	Number	2010	5956.98	7379.43	4632.87
Prevalence	Central Eu	Both	50-69 yea	HIV/AIDS	Rate	2010	20.5702	25.4821	15.9979
Prevalence	Central Eu	Both	50-69 yea	HIV/AIDS	Number	2011	6364.7	7876.71	4968.05
Prevalence	Central Eu	Both	50-69 yea	HIV/AIDS	Rate	2011	21.7994	26.9781	17.0158
Prevalence	Central Eu	Both	50-69 yea	HIV/AIDS	Number	2012	6756.16	8337.05	5285.61
Prevalence	Central Eu	Both	50-69 yea	HIV/AIDS	Rate	2012	22.9763	28.3526	17.9753
Prevalence	Central Eu	Both	50-69 yea	HIV/AIDS	Number	2013	7143.69	8815.03	5592.54
Prevalence	Central Eu	Both	50-69 yea	HIV/AIDS	Rate	2013	24.1393	29.787	18.8978
Prevalence	Central Eu	Both	50-69 yea	HIV/AIDS	Number	2014	7528.86	9253.16	5918.01
Prevalence	Central Eu	Both	50-69 yea	HIV/AIDS	Rate	2014	25.2989	31.093	19.8861
Prevalence	Central Eu	Both	50-69 yea	HIV/AIDS	Number	2015	7922.88	9709.15	6246.71
Prevalence	Central Eu	Both	50-69 yea	HIV/AIDS	Rate	2015	26.5084	32.4849	20.9003
Prevalence	Central Eu	Both	50-69 yea	HIV/AIDS	Number	2016	8355.01	10179.4	6595.44
Prevalence	Central Eu	Both	50-69 yea	HIV/AIDS	Rate	2016	27.9216	34.0185	22.0413
Prevalence	Central Eu	Both	50-69 yea	HIV/AIDS	Number	2017	8842.74	10704.5	7011.54
Prevalence	Central Eu	Both	50-69 yea	HIV/AIDS	Rate	2017	29.5853	35.8143	23.4586
Prevalence	Central Eu	Both	50-69 yea	HIV/AIDS	Number	2018	9444.23	11367	7502.86
Prevalence	Central Eu	Both	50-69 yea	HIV/AIDS	Rate	2018	31.5589	37.9839	25.0716
Prevalence	Central Eu	Both	50-69 yea	HIV/AIDS	Number	2019	10060.8	12014.7	8022.12
Prevalence	Central Eu	Both	50-69 yea	HIV/AIDS	Rate	2019	33.6569	40.1933	26.8368
Prevalence	Central As	Both	50-69 yea	Sexually tr	Number	1990	1857359	2127125	1600509
Prevalence	Central As	Both	50-69 yea	Sexually tr	Rate	1990	21296.9	24390.1	18351.8
Prevalence	Central As	Both	50-69 yea	Sexually tr	Number	1991	1883105	2159643	1623145
Prevalence	Central As	Both	50-69 yea	Sexually tr	Rate	1991	21240.8	24360	18308.5
Prevalence	Central As	Both	50-69 yea	Sexually tr	Number	1992	1888389	2165769	1630953
Prevalence	Central As	Both	50-69 yea	Sexually tr	Rate	1992	21166.2	24275.2	18280.7
Prevalence	Central As	Both	50-69 yea	Sexually tr	Number	1993	1866274	2139387	1615746
Prevalence	Central As	Both	50-69 yea	Sexually tr	Rate	1993	21074.3	24158.3	18245.2
Prevalence	Central As	Both	50-69 yea	Sexually tr	Number	1994	1816898	2089393	1570161
Prevalence	Central As	Both	50-69 yea	Sexually tr	Rate	1994	20958.2	24101.5	18112.1
Prevalence	Central As	Both	50-69 yea	Sexually tr	Number	1995	1762788	2027015	1522160
Prevalence	Central As	Both	50-69 yea	Sexually tr	Rate	1995	20867.9	23995.9	18019.4
Prevalence	Central As	Both	50-69 yea	Sexually tr	Number	1996	1714317	1968711	1480308
Prevalence	Central As	Both	50-69 yea	Sexually tr	Rate	1996	20787.1	23871.8	17949.6
Prevalence	Central As	Both	50-69 yea	Sexually tr	Number	1997	1684333	1939304	1455104
Prevalence	Central As	Both	50-69 yea	Sexually tr	Rate	1997	20754	23895.7	17929.5
Prevalence	Central As	Both	50-69 yea	Sexually tr	Number	1998	1661769	1914262	1435948
Prevalence	Central As	Both	50-69 yea	Sexually tr	Rate	1998	20745.2	23897.2	17926.1
Prevalence	Central As	Both	50-69 yea	Sexually tr	Number	1999	1642701	1891934	1419512
Prevalence	Central As	Both	50-69 yea	Sexually tr	Rate	1999	20773.8	23925.6	17951.3
Prevalence	Central As	Both	50-69 yea	Sexually tr	Number	2000	1636803	1881765	1411370
Prevalence	Central As	Both	50-69 yea	Sexually tr	Rate	2000	20818.7	23934.4	17951.4
Prevalence	Central As	Both	50-69 yea	Sexually tr	Number	2001	1662180	1912189	1436581
Prevalence	Central As	Both	50-69 yea	Sexually tr	Rate	2001	20903.3	24047.4	18066.2
Prevalence	Central As	Both	50-69 yea	Sexually tr	Number	2002	1697591	1951225	1464108
Prevalence	Central As	Both	50-69 yea	Sexually tr	Rate	2002	20958.2	24089.5	18075.6
Prevalence	Central As	Both	50-69 yea	Sexually tr	Number	2003	1735272	1995973	1500613
Prevalence	Central As	Both	50-69 yea	Sexually tr	Rate	2003	21024.8	24183.5	18181.7
Prevalence	Central As	Both	50-69 yea	Sexually tr	Number	2004	1784504	2050052	1541542
Prevalence	Central As	Both	50-69 yea	Sexually tr	Rate	2004	21094.9	24234	18222.8
Prevalence	Central As	Both	50-69 yea	Sexually tr	Number	2005	1840950	2108221	1593325
Prevalence	Central As	Both	50-69 yea	Sexually tr	Rate	2005	21181.8	24257	18332.7
Prevalence	Central As	Both	50-69 yea	Sexually tr	Number	2006	1898929	2177572	1639570
Prevalence	Central As	Both	50-69 yea	Sexually tr	Rate	2006	21252.3	24370.8	18349.6
Prevalence	Central As	Both	50-69 yea	Sexually tr	Number	2007	1961965	2246180	1696525
Prevalence	Central As	Both	50-69 yea	Sexually tr	Rate	2007	21329	24418.8	18443.3
Prevalence	Central As	Both	50-69 yea	Sexually tr	Number	2008	2035041	2333570	1754232
Prevalence	Central As	Both	50-69 yea	Sexually tr	Rate	2008	21414.2	24555.6	18459.4
Prevalence	Central As	Both	50-69 yea	Sexually tr	Number	2009	2122135	2436199	1834260
Prevalence	Central As	Both	50-69 yea	Sexually tr	Rate	2009	21496.6	24678	18580.5
Prevalence	Central As	Both	50-69 yea	Sexually tr	Number	2010	2220820	2549052	1923541
Prevalence	Central As	Both	50-69 yea	Sexually tr	Rate	2010	21554.5	24740.2	18669.2
Prevalence	Central As	Both	50-69 yea	Sexually tr	Number	2011	2329669	2673271	2013029
Prevalence	Central As	Both	50-69 yea	Sexually tr	Rate	2011	21581.7	24764.7	18648.4
Prevalence	Central As	Both	50-69 yea	Sexually tr	Number	2012	2443450	2810521	2107635
Prevalence	Central As	Both	50-69 yea	Sexually tr	Rate	2012	21586.5	24829.4	18619.8
Prevalence	Central As	Both	50-69 yea	Sexually tr	Number	2013	2565610	2951987	2214165
Prevalence	Central As	Both	50-69 yea	Sexually tr	Rate	2013	21568.7	24816.9	18614.1
Prevalence	Central As	Both	50-69 yea	Sexually tr	Number	2014	2693362	3100227	2318094
Prevalence	Central As	Both	50-69 yea	Sexually tr	Rate	2014	21529	24781.2	18529.3
Prevalence	Central As	Both	50-69 yea	Sexually tr	Number	2015	2812129	3236707	2424726
Prevalence	Central As	Both	50-69 yea	Sexually tr	Rate	2015	21476.8	24719.4	18518.2
Prevalence	Central As	Both	50-69 yea	Sexually tr	Number	2016	2911446	3349033	2513604
Prevalence	Central As	Both	50-69 yea	Sexually tr	Rate	2016	21348.4	24557.1	18431.2
Prevalence	Central As	Both	50-69 yea	Sexually tr	Number	2017	2992875	3441113	2585368
Prevalence	Central As	Both	50-69 yea	Sexually tr	Rate	2017	21206.7	24382.8	18319.3
Prevalence	Central As	Both	50-69 yea	Sexually tr	Number	2018	3071421	3532968	2651255
Prevalence	Central As	Both	50-69 yea	Sexually tr	Rate	2018	21100.3	24271.1	18213.8
Prevalence	Central As	Both	50-69 yea	Sexually tr	Number	2019	3150825	3630378	2733132
Prevalence	Central As	Both	50-69 yea	Sexually tr	Rate	2019	21004.1	24200.9	18219.7
Prevalence	Western E	Both	50-69 yea	HIV/AIDS	Number	1990	24628.8	30974.2	18204.7
Prevalence	Western E	Both	50-69 yea	HIV/AIDS	Rate	1990	29.7601	37.4276	21.9975
Prevalence	Western E	Both	50-69 yea	HIV/AIDS	Number	1991	27454.4	34752.3	20039.9
Prevalence	Western E	Both	50-69 yea	HIV/AIDS	Rate	1991	33.0163	41.7926	24.0997
Prevalence	Western E	Both	50-69 yea	HIV/AIDS	Number	1992	29928.5	38105.9	21697.8
Prevalence	Western E	Both	50-69 yea	HIV/AIDS	Rate	1992	35.9929	45.8274	26.0945
Prevalence	Western E	Both	50-69 yea	HIV/AIDS	Number	1993	32217.2	41420.3	23093.9
Prevalence	Western E	Both	50-69 yea	HIV/AIDS	Rate	1993	38.6597	49.7031	27.7119
Prevalence	Western E	Both	50-69 yea	HIV/AIDS	Number	1994	34440.5	44683.6	24374.5
Prevalence	Western E	Both	50-69 yea	HIV/AIDS	Rate	1994	41.1621	53.4043	29.1316
Prevalence	Western E	Both	50-69 yea	HIV/AIDS	Number	1995	36562.5	48027	25541.9
Prevalence	Western E	Both	50-69 yea	HIV/AIDS	Rate	1995	43.6091	57.2831	30.4645
Prevalence	Western E	Both	50-69 yea	HIV/AIDS	Number	1996	38997	51501.2	27292.3
Prevalence	Western E	Both	50-69 yea	HIV/AIDS	Rate	1996	46.2894	61.1318	32.3959
Prevalence	Western E	Both	50-69 yea	HIV/AIDS	Number	1997	42210.7	55986.1	29560.5
Prevalence	Western E	Both	50-69 yea	HIV/AIDS	Rate	1997	49.5424	65.7105	34.6949
Prevalence	Western E	Both	50-69 yea	HIV/AIDS	Number	1998	45999.4	61075.3	32329
Prevalence	Western E	Both	50-69 yea	HIV/AIDS	Rate	1998	53.3274	70.8051	37.4793
Prevalence	Western E	Both	50-69 yea	HIV/AIDS	Number	1999	50338.4	66515.2	35334.7
Prevalence	Western E	Both	50-69 yea	HIV/AIDS	Rate	1999	57.601	76.1118	40.4326
Prevalence	Western E	Both	50-69 yea	HIV/AIDS	Number	2000	55242.1	72603.7	38829.9
Prevalence	Western E	Both	50-69 yea	HIV/AIDS	Rate	2000	62.4431	82.0679	43.8915
Prevalence	Western E	Both	50-69 yea	HIV/AIDS	Number	2001	60704.4	78943.5	42790.7
Prevalence	Western E	Both	50-69 yea	HIV/AIDS	Rate	2001	67.8816	88.2772	47.85
Prevalence	Western E	Both	50-69 yea	HIV/AIDS	Number	2002	67054.9	85869.2	47587.5
Prevalence	Western E	Both	50-69 yea	HIV/AIDS	Rate	2002	74.1173	94.9132	52.5996
Prevalence	Western E	Both	50-69 yea	HIV/AIDS	Number	2003	74164.5	94208.6	53004.7
Prevalence	Western E	Both	50-69 yea	HIV/AIDS	Rate	2003	80.9856	102.873	57.8796
Prevalence	Western E	Both	50-69 yea	HIV/AIDS	Number	2004	81720.1	102936	58579.1
Prevalence	Western E	Both	50-69 yea	HIV/AIDS	Rate	2004	88.2607	111.174	63.2676
Prevalence	Western E	Both	50-69 yea	HIV/AIDS	Number	2005	89933.3	112429	65315.4
Prevalence	Western E	Both	50-69 yea	HIV/AIDS	Rate	2005	96.0525	120.079	69.7596
Prevalence	Western E	Both	50-69 yea	HIV/AIDS	Number	2006	99057.3	123350	72480.8
Prevalence	Western E	Both	50-69 yea	HIV/AIDS	Rate	2006	104.568	130.211	76.5129
Prevalence	Western E	Both	50-69 yea	HIV/AIDS	Number	2007	109284	135542	80538.2
Prevalence	Western E	Both	50-69 yea	HIV/AIDS	Rate	2007	113.974	141.359	83.9946
Prevalence	Western E	Both	50-69 yea	HIV/AIDS	Number	2008	120600	149144	89425.2
Prevalence	Western E	Both	50-69 yea	HIV/AIDS	Rate	2008	124.229	153.632	92.1158
Prevalence	Western E	Both	50-69 yea	HIV/AIDS	Number	2009	132982	164418	99151.6
Prevalence	Western E	Both	50-69 yea	HIV/AIDS	Rate	2009	135.316	167.303	100.892
Prevalence	Western E	Both	50-69 yea	HIV/AIDS	Number	2010	146669	180992	109767
Prevalence	Western E	Both	50-69 yea	HIV/AIDS	Rate	2010	147.262	181.724	110.211
Prevalence	Western E	Both	50-69 yea	HIV/AIDS	Number	2011	161710	199272	121512
Prevalence	Western E	Both	50-69 yea	HIV/AIDS	Rate	2011	160.174	197.38	120.358
Prevalence	Western E	Both	50-69 yea	HIV/AIDS	Number	2012	178134	219059	134830
Prevalence	Western E	Both	50-69 yea	HIV/AIDS	Rate	2012	173.335	213.158	131.198
Prevalence	Western E	Both	50-69 yea	HIV/AIDS	Number	2013	195303	240336	148325
Prevalence	Western E	Both	50-69 yea	HIV/AIDS	Rate	2013	186.762	229.826	141.839
Prevalence	Western E	Both	50-69 yea	HIV/AIDS	Number	2014	212777	262053	162009
Prevalence	Western E	Both	50-69 yea	HIV/AIDS	Rate	2014	200.066	246.399	152.331
Prevalence	Western E	Both	50-69 yea	HIV/AIDS	Number	2015	230295	283850	175746
Prevalence	Western E	Both	50-69 yea	HIV/AIDS	Rate	2015	212.611	262.054	162.251
Prevalence	Western E	Both	50-69 yea	HIV/AIDS	Number	2016	246635	303921	188514
Prevalence	Western E	Both	50-69 yea	HIV/AIDS	Rate	2016	224.044	276.083	171.246
Prevalence	Western E	Both	50-69 yea	HIV/AIDS	Number	2017	261161	321893	199372
Prevalence	Western E	Both	50-69 yea	HIV/AIDS	Rate	2017	234.724	289.308	179.19
Prevalence	Western E	Both	50-69 yea	HIV/AIDS	Number	2018	274521	338464	209697
Prevalence	Western E	Both	50-69 yea	HIV/AIDS	Rate	2018	244.446	301.383	186.723
Prevalence	Western E	Both	50-69 yea	HIV/AIDS	Number	2019	286869	353904	219464
Prevalence	Western E	Both	50-69 yea	HIV/AIDS	Rate	2019	253.51	312.75	193.943
Prevalence	Central Eu	Both	50-69 yea	Sexually tr	Number	1990	3830891	4414378	3313942
Prevalence	Central Eu	Both	50-69 yea	Sexually tr	Rate	1990	15221.3	17539.7	13167.3
Prevalence	Central Eu	Both	50-69 yea	Sexually tr	Number	1991	3839820	4414928	3318942
Prevalence	Central Eu	Both	50-69 yea	Sexually tr	Rate	1991	15259.2	17544.7	13189.3
Prevalence	Central Eu	Both	50-69 yea	Sexually tr	Number	1992	3830960	4404035	3302881
Prevalence	Central Eu	Both	50-69 yea	Sexually tr	Rate	1992	15289	17576.1	13181.5
Prevalence	Central Eu	Both	50-69 yea	Sexually tr	Number	1993	3811405	4382267	3277453
Prevalence	Central Eu	Both	50-69 yea	Sexually tr	Rate	1993	15303.7	17595.8	13159.7
Prevalence	Central Eu	Both	50-69 yea	Sexually tr	Number	1994	3788845	4366256	3252244
Prevalence	Central Eu	Both	50-69 yea	Sexually tr	Rate	1994	15305.6	17638.1	13137.9
Prevalence	Central Eu	Both	50-69 yea	Sexually tr	Number	1995	3758664	4335608	3239591
Prevalence	Central Eu	Both	50-69 yea	Sexually tr	Rate	1995	15290.6	17637.6	13178.9
Prevalence	Central Eu	Both	50-69 yea	Sexually tr	Number	1996	3735066	4313072	3217132
Prevalence	Central Eu	Both	50-69 yea	Sexually tr	Rate	1996	15252.2	17612.5	13137.2
Prevalence	Central Eu	Both	50-69 yea	Sexually tr	Number	1997	3731468	4296263	3206958
Prevalence	Central Eu	Both	50-69 yea	Sexually tr	Rate	1997	15186.3	17484.9	13051.7
Prevalence	Central Eu	Both	50-69 yea	Sexually tr	Number	1998	3738176	4299069	3215087
Prevalence	Central Eu	Both	50-69 yea	Sexually tr	Rate	1998	15115.3	17383.2	13000.2
Prevalence	Central Eu	Both	50-69 yea	Sexually tr	Number	1999	3755085	4323548	3244427
Prevalence	Central Eu	Both	50-69 yea	Sexually tr	Rate	1999	15060.4	17340.3	13012.3
Prevalence	Central Eu	Both	50-69 yea	Sexually tr	Number	2000	3795092	4365372	3273061
Prevalence	Central Eu	Both	50-69 yea	Sexually tr	Rate	2000	15049	17310.3	12978.9
Prevalence	Central Eu	Both	50-69 yea	Sexually tr	Number	2001	3850423	4433934	3319266
Prevalence	Central Eu	Both	50-69 yea	Sexually tr	Rate	2001	15079.9	17365.2	12999.7
Prevalence	Central Eu	Both	50-69 yea	Sexually tr	Number	2002	3916821	4515934	3371400
Prevalence	Central Eu	Both	50-69 yea	Sexually tr	Rate	2002	15133.3	17448.1	13026
Prevalence	Central Eu	Both	50-69 yea	Sexually tr	Number	2003	3992915	4612266	3433918
Prevalence	Central Eu	Both	50-69 yea	Sexually tr	Rate	2003	15194.2	17551	13067
Prevalence	Central Eu	Both	50-69 yea	Sexually tr	Number	2004	4070059	4695591	3493955
Prevalence	Central Eu	Both	50-69 yea	Sexually tr	Rate	2004	15245	17588.1	13087.2
Prevalence	Central Eu	Both	50-69 yea	Sexually tr	Number	2005	4146716	4788789	3563065
Prevalence	Central Eu	Both	50-69 yea	Sexually tr	Rate	2005	15263.8	17627.3	13115.5
Prevalence	Central Eu	Both	50-69 yea	Sexually tr	Number	2006	4217263	4879113	3623263
Prevalence	Central Eu	Both	50-69 yea	Sexually tr	Rate	2006	15267.8	17664	13117.4
Prevalence	Central Eu	Both	50-69 yea	Sexually tr	Number	2007	4282061	4945598	3672190
Prevalence	Central Eu	Both	50-69 yea	Sexually tr	Rate	2007	15276.6	17643.8	13100.8
Prevalence	Central Eu	Both	50-69 yea	Sexually tr	Number	2008	4338795	5020893	3724553
Prevalence	Central Eu	Both	50-69 yea	Sexually tr	Rate	2008	15283	17685.6	13119.4
Prevalence	Central Eu	Both	50-69 yea	Sexually tr	Number	2009	4387388	5075575	3762036
Prevalence	Central Eu	Both	50-69 yea	Sexually tr	Rate	2009	15285.4	17683	13106.7
Prevalence	Central Eu	Both	50-69 yea	Sexually tr	Number	2010	4421885	5127642	3793178
Prevalence	Central Eu	Both	50-69 yea	Sexually tr	Rate	2010	15269.3	17706.4	13098.3
Prevalence	Central Eu	Both	50-69 yea	Sexually tr	Number	2011	4452550	5155968	3824896
Prevalence	Central Eu	Both	50-69 yea	Sexually tr	Rate	2011	15250.2	17659.4	13100.4
Prevalence	Central Eu	Both	50-69 yea	Sexually tr	Number	2012	4479199	5196003	3850729
Prevalence	Central Eu	Both	50-69 yea	Sexually tr	Rate	2012	15232.8	17670.5	13095.5
Prevalence	Central Eu	Both	50-69 yea	Sexually tr	Number	2013	4502713	5222982	3872609
Prevalence	Central Eu	Both	50-69 yea	Sexually tr	Rate	2013	15215.2	17649.1	13086
Prevalence	Central Eu	Both	50-69 yea	Sexually tr	Number	2014	4521699	5243795	3896197
Prevalence	Central Eu	Both	50-69 yea	Sexually tr	Rate	2014	15194.1	17620.5	13092.2
Prevalence	Central Eu	Both	50-69 yea	Sexually tr	Number	2015	4529769	5251752	3902506
Prevalence	Central Eu	Both	50-69 yea	Sexually tr	Rate	2015	15155.7	17571.4	13057
Prevalence	Central Eu	Both	50-69 yea	Sexually tr	Number	2016	4505584	5240347	3884333
Prevalence	Central Eu	Both	50-69 yea	Sexually tr	Rate	2016	15057.2	17512.7	12981
Prevalence	Central Eu	Both	50-69 yea	Sexually tr	Number	2017	4470860	5193168	3853916
Prevalence	Central Eu	Both	50-69 yea	Sexually tr	Rate	2017	14958.2	17374.9	12894.1
Prevalence	Central Eu	Both	50-69 yea	Sexually tr	Number	2018	4466704	5195465	3841254
Prevalence	Central Eu	Both	50-69 yea	Sexually tr	Rate	2018	14926	17361.2	12836
Prevalence	Central Eu	Both	50-69 yea	Sexually tr	Number	2019	4454948	5173616	3831870
Prevalence	Central Eu	Both	50-69 yea	Sexually tr	Rate	2019	14903.3	17307.5	12818.9
Prevalence	Australasi	Both	50-69 yea	Sexually tr	Number	1990	772096	902162	652211
Prevalence	Australasi	Both	50-69 yea	Sexually tr	Rate	1990	22433.7	26212.8	18950.3
Prevalence	Australasi	Both	50-69 yea	Sexually tr	Number	1991	749222	860481	642785
Prevalence	Australasi	Both	50-69 yea	Sexually tr	Rate	1991	21539.2	24737.7	18479.2
Prevalence	Australasi	Both	50-69 yea	Sexually tr	Number	1992	730389	823251	638522
Prevalence	Australasi	Both	50-69 yea	Sexually tr	Rate	1992	20733	23369	18125.3
Prevalence	Australasi	Both	50-69 yea	Sexually tr	Number	1993	715821	800609	636067
Prevalence	Australasi	Both	50-69 yea	Sexually tr	Rate	1993	20071	22448.4	17834.8
Prevalence	Australasi	Both	50-69 yea	Sexually tr	Number	1994	712628	791588	636963
Prevalence	Australasi	Both	50-69 yea	Sexually tr	Rate	1994	19630.8	21805.9	17546.4
Prevalence	Australasi	Both	50-69 yea	Sexually tr	Number	1995	720650	802721	641383
Prevalence	Australasi	Both	50-69 yea	Sexually tr	Rate	1995	19479.7	21698.1	17337
Prevalence	Australasi	Both	50-69 yea	Sexually tr	Number	1996	736984	819720	656396
Prevalence	Australasi	Both	50-69 yea	Sexually tr	Rate	1996	19499.2	21688.3	17367
Prevalence	Australasi	Both	50-69 yea	Sexually tr	Number	1997	764593	850477	680669
Prevalence	Australasi	Both	50-69 yea	Sexually tr	Rate	1997	19549.1	21745	17403.3
Prevalence	Australasi	Both	50-69 yea	Sexually tr	Number	1998	789799	877934	705991
Prevalence	Australasi	Both	50-69 yea	Sexually tr	Rate	1998	19595.3	21782	17516
Prevalence	Australasi	Both	50-69 yea	Sexually tr	Number	1999	814271	906923	725574
Prevalence	Australasi	Both	50-69 yea	Sexually tr	Rate	1999	19618.1	21850.4	17481.2
Prevalence	Australasi	Both	50-69 yea	Sexually tr	Number	2000	840051	936974	748634
Prevalence	Australasi	Both	50-69 yea	Sexually tr	Rate	2000	19635.8	21901.3	17499
Prevalence	Australasi	Both	50-69 yea	Sexually tr	Number	2001	845964	929392	763842
Prevalence	Australasi	Both	50-69 yea	Sexually tr	Rate	2001	19187.5	21079.7	17324.8
Prevalence	Australasi	Both	50-69 yea	Sexually tr	Number	2002	822064	890316	754185
Prevalence	Australasi	Both	50-69 yea	Sexually tr	Rate	2002	18074.6	19575.2	16582.1
Prevalence	Australasi	Both	50-69 yea	Sexually tr	Number	2003	786390	840778	728932
Prevalence	Australasi	Both	50-69 yea	Sexually tr	Rate	2003	16746.4	17904.6	15522.8
Prevalence	Australasi	Both	50-69 yea	Sexually tr	Number	2004	757224	801058	709313
Prevalence	Australasi	Both	50-69 yea	Sexually tr	Rate	2004	15634.2	16539.3	14645
Prevalence	Australasi	Both	50-69 yea	Sexually tr	Number	2005	757381	798189	714611
Prevalence	Australasi	Both	50-69 yea	Sexually tr	Rate	2005	15163.9	15980.9	14307.6
Prevalence	Australasi	Both	50-69 yea	Sexually tr	Number	2006	802524	851385	750752
Prevalence	Australasi	Both	50-69 yea	Sexually tr	Rate	2006	15599	16548.7	14592.7
Prevalence	Australasi	Both	50-69 yea	Sexually tr	Number	2007	879740	949414	807996
Prevalence	Australasi	Both	50-69 yea	Sexually tr	Rate	2007	16630.1	17947.2	15273.9
Prevalence	Australasi	Both	50-69 yea	Sexually tr	Number	2008	971844	1072469	872684
Prevalence	Australasi	Both	50-69 yea	Sexually tr	Rate	2008	17877.7	19728.7	16053.6
Prevalence	Australasi	Both	50-69 yea	Sexually tr	Number	2009	1055765	1194058	926700
Prevalence	Australasi	Both	50-69 yea	Sexually tr	Rate	2009	18916.3	21394.1	16603.8
Prevalence	Australasi	Both	50-69 yea	Sexually tr	Number	2010	1108795	1279190	953208
Prevalence	Australasi	Both	50-69 yea	Sexually tr	Rate	2010	19356.7	22331.3	16640.5
Prevalence	Australasi	Both	50-69 yea	Sexually tr	Number	2011	1138429	1316712	980673
Prevalence	Australasi	Both	50-69 yea	Sexually tr	Rate	2011	19357.4	22388.9	16675
Prevalence	Australasi	Both	50-69 yea	Sexually tr	Number	2012	1166992	1353171	1003494
Prevalence	Australasi	Both	50-69 yea	Sexually tr	Rate	2012	19352.4	22439.9	16641.1
Prevalence	Australasi	Both	50-69 yea	Sexually tr	Number	2013	1197073	1389421	1027128
Prevalence	Australasi	Both	50-69 yea	Sexually tr	Rate	2013	19357.4	22467.8	16609.3
Prevalence	Australasi	Both	50-69 yea	Sexually tr	Number	2014	1222176	1416957	1050215
Prevalence	Australasi	Both	50-69 yea	Sexually tr	Rate	2014	19352.7	22436.9	16629.7
Prevalence	Australasi	Both	50-69 yea	Sexually tr	Number	2015	1243463	1444963	1063103
Prevalence	Australasi	Both	50-69 yea	Sexually tr	Rate	2015	19343.6	22478.2	16537.9
Prevalence	Australasi	Both	50-69 yea	Sexually tr	Number	2016	1263588	1461563	1084850
Prevalence	Australasi	Both	50-69 yea	Sexually tr	Rate	2016	19360	22393.2	16621.4
Prevalence	Australasi	Both	50-69 yea	Sexually tr	Number	2017	1275476	1482188	1098536
Prevalence	Australasi	Both	50-69 yea	Sexually tr	Rate	2017	19379.7	22520.5	16691.3
Prevalence	Australasi	Both	50-69 yea	Sexually tr	Number	2018	1288798	1496513	1107518
Prevalence	Australasi	Both	50-69 yea	Sexually tr	Rate	2018	19369	22490.7	16644.6
Prevalence	Australasi	Both	50-69 yea	Sexually tr	Number	2019	1303704	1515053	1120127
Prevalence	Australasi	Both	50-69 yea	Sexually tr	Rate	2019	19344.4	22480.4	16620.5
Prevalence	Southeast	Both	50-69 yea	HIV/AIDS	Number	1990	13309.2	17075.9	10157.4
Prevalence	Southeast	Both	50-69 yea	HIV/AIDS	Rate	1990	28.1745	36.1484	21.5025
Prevalence	Southeast	Both	50-69 yea	HIV/AIDS	Number	1991	21962	27593.8	16969.1
Prevalence	Southeast	Both	50-69 yea	HIV/AIDS	Rate	1991	45.3794	57.0161	35.0627
Prevalence	Southeast	Both	50-69 yea	HIV/AIDS	Number	1992	30332.4	38156.8	23722.5
Prevalence	Southeast	Both	50-69 yea	HIV/AIDS	Rate	1992	61.3606	77.1888	47.9891
Prevalence	Southeast	Both	50-69 yea	HIV/AIDS	Number	1993	38689.4	49473.6	29590.9
Prevalence	Southeast	Both	50-69 yea	HIV/AIDS	Rate	1993	76.689	98.0651	58.6542
Prevalence	Southeast	Both	50-69 yea	HIV/AIDS	Number	1994	46520.7	59107.9	35014.3
Prevalence	Southeast	Both	50-69 yea	HIV/AIDS	Rate	1994	90.3615	114.811	68.0116
Prevalence	Southeast	Both	50-69 yea	HIV/AIDS	Number	1995	53345.4	67742.9	40116.2
Prevalence	Southeast	Both	50-69 yea	HIV/AIDS	Rate	1995	101.51	128.907	76.3364
Prevalence	Southeast	Both	50-69 yea	HIV/AIDS	Number	1996	59596.6	75851.7	45242.5
Prevalence	Southeast	Both	50-69 yea	HIV/AIDS	Rate	1996	110.872	141.113	84.1684
Prevalence	Southeast	Both	50-69 yea	HIV/AIDS	Number	1997	65286.6	83358	49744.6
Prevalence	Southeast	Both	50-69 yea	HIV/AIDS	Rate	1997	119.042	151.993	90.7033
Prevalence	Southeast	Both	50-69 yea	HIV/AIDS	Number	1998	69925	89338.2	53716.4
Prevalence	Southeast	Both	50-69 yea	HIV/AIDS	Rate	1998	124.76	159.397	95.8408
Prevalence	Southeast	Both	50-69 yea	HIV/AIDS	Number	1999	73508.4	94363.9	56153.9
Prevalence	Southeast	Both	50-69 yea	HIV/AIDS	Rate	1999	128.205	164.579	97.9377
Prevalence	Southeast	Both	50-69 yea	HIV/AIDS	Number	2000	76522.6	99552.5	59038.1
Prevalence	Southeast	Both	50-69 yea	HIV/AIDS	Rate	2000	130.652	169.972	100.799
Prevalence	Southeast	Both	50-69 yea	HIV/AIDS	Number	2001	80441.2	105670	62762.3
Prevalence	Southeast	Both	50-69 yea	HIV/AIDS	Rate	2001	133.249	175.039	103.964
Prevalence	Southeast	Both	50-69 yea	HIV/AIDS	Number	2002	85365.6	111488	67989.7
Prevalence	Southeast	Both	50-69 yea	HIV/AIDS	Rate	2002	137.171	179.145	109.25
Prevalence	Southeast	Both	50-69 yea	HIV/AIDS	Number	2003	92187.6	117563	75588.1
Prevalence	Southeast	Both	50-69 yea	HIV/AIDS	Rate	2003	143.547	183.06	117.7
Prevalence	Southeast	Both	50-69 yea	HIV/AIDS	Number	2004	100575	123217	83587.3
Prevalence	Southeast	Both	50-69 yea	HIV/AIDS	Rate	2004	151.579	185.703	125.976
Prevalence	Southeast	Both	50-69 yea	HIV/AIDS	Number	2005	110299	134262	91779.7
Prevalence	Southeast	Both	50-69 yea	HIV/AIDS	Rate	2005	160.603	195.496	133.638
Prevalence	Southeast	Both	50-69 yea	HIV/AIDS	Number	2006	121959	148887	100885
Prevalence	Southeast	Both	50-69 yea	HIV/AIDS	Rate	2006	171.344	209.176	141.737
Prevalence	Southeast	Both	50-69 yea	HIV/AIDS	Number	2007	135360	167462	110119
Prevalence	South Asia	Both	50-69 yea	HIV/AIDS	Number	1990	7353.95	10599.9	5952.21
Prevalence	South Asia	Both	50-69 yea	HIV/AIDS	Rate	1990	6.84526	9.86668	5.54048
Prevalence	South Asia	Both	50-69 yea	HIV/AIDS	Number	1991	9206.48	12927.4	7542.3
Prevalence	South Asia	Both	50-69 yea	HIV/AIDS	Rate	1991	8.35731	11.7351	6.84663
Prevalence	South Asia	Both	50-69 yea	HIV/AIDS	Number	1992	11412.6	15329.8	9395.35
Prevalence	South Asia	Both	50-69 yea	HIV/AIDS	Rate	1992	10.1141	13.5856	8.32636
Prevalence	South Asia	Both	50-69 yea	HIV/AIDS	Number	1993	14150.7	18482.7	11715.4
Prevalence	South Asia	Both	50-69 yea	HIV/AIDS	Rate	1993	12.2486	15.9983	10.1407
Prevalence	South Asia	Both	50-69 yea	HIV/AIDS	Number	1994	17629.8	22786.2	14572.3
Prevalence	South Asia	Both	50-69 yea	HIV/AIDS	Rate	1994	14.9112	19.2725	12.3252
Prevalence	South Asia	Both	50-69 yea	HIV/AIDS	Number	1995	22056.6	28139.2	18063.2
Prevalence	South Asia	Both	50-69 yea	HIV/AIDS	Rate	1995	18.2434	23.2744	14.9404
Prevalence	South Asia	Both	50-69 yea	HIV/AIDS	Number	1996	27770.9	35666.8	22578.2
Prevalence	South Asia	Both	50-69 yea	HIV/AIDS	Rate	1996	22.4833	28.8758	18.2793
Prevalence	South Asia	Both	50-69 yea	HIV/AIDS	Number	1997	35104.8	44764.1	28362.2
Prevalence	South Asia	Both	50-69 yea	HIV/AIDS	Rate	1997	27.7903	35.4369	22.4526
Prevalence	South Asia	Both	50-69 yea	HIV/AIDS	Number	1998	43773.4	55365.5	35629.5
Prevalence	South Asia	Both	50-69 yea	HIV/AIDS	Rate	1998	33.9421	42.9308	27.6274
Prevalence	South Asia	Both	50-69 yea	HIV/AIDS	Number	1999	53473.9	67777	43398.7
Prevalence	South Asia	Both	50-69 yea	HIV/AIDS	Rate	1999	40.6386	51.5085	32.9817
Prevalence	South Asia	Both	50-69 yea	HIV/AIDS	Number	2000	63497.1	80554.4	51068.2
Prevalence	South Asia	Both	50-69 yea	HIV/AIDS	Rate	2000	47.3795	60.1071	38.1054
Prevalence	South Asia	Both	50-69 yea	HIV/AIDS	Number	2001	75015.9	94119.9	59849.1
Prevalence	South Asia	Both	50-69 yea	HIV/AIDS	Rate	2001	54.351	68.1925	43.3623
Prevalence	South Asia	Both	50-69 yea	HIV/AIDS	Number	2002	85942.9	109051	68143
Prevalence	South Asia	Both	50-69 yea	HIV/AIDS	Rate	2002	60.4727	76.7325	47.948
Prevalence	South Asia	Both	50-69 yea	HIV/AIDS	Number	2003	95924.3	122651	75491.4
Prevalence	South Asia	Both	50-69 yea	HIV/AIDS	Rate	2003	65.5631	83.8304	51.5974
Prevalence	South Asia	Both	50-69 yea	HIV/AIDS	Number	2004	104885	135402	81889.9
Prevalence	South Asia	Both	50-69 yea	HIV/AIDS	Rate	2004	69.6019	89.853	54.3422
Prevalence	South Asia	Both	50-69 yea	HIV/AIDS	Number	2005	112670	146173	87478
Prevalence	South Asia	Both	50-69 yea	HIV/AIDS	Rate	2005	72.5292	94.096	56.3123
Prevalence	South Asia	Both	50-69 yea	HIV/AIDS	Number	2006	118716	157670	90796.2
Prevalence	South Asia	Both	50-69 yea	HIV/AIDS	Rate	2006	74.0814	98.3896	56.659
Prevalence	South Asia	Both	50-69 yea	HIV/AIDS	Number	2007	124960	168070	94643.7
Prevalence	South Asia	Both	50-69 yea	HIV/AIDS	Rate	2007	75.5135	101.565	57.1932
Prevalence	South Asia	Both	50-69 yea	HIV/AIDS	Number	2008	132199	179777	101112
Prevalence	South Asia	Both	50-69 yea	HIV/AIDS	Rate	2008	77.3083	105.131	59.1289
Prevalence	South Asia	Both	50-69 yea	HIV/AIDS	Number	2009	141114	192340	108271
Prevalence	South Asia	Both	50-69 yea	HIV/AIDS	Rate	2009	79.798	108.766	61.2257
Prevalence	South Asia	Both	50-69 yea	HIV/AIDS	Number	2010	152110	205053	118726
Prevalence	South Asia	Both	50-69 yea	HIV/AIDS	Rate	2010	83.1452	112.085	64.897
Prevalence	South Asia	Both	50-69 yea	HIV/AIDS	Number	2011	165008	219187	129337
Prevalence	South Asia	Both	50-69 yea	HIV/AIDS	Rate	2011	87.1818	115.807	68.3352
Prevalence	South Asia	Both	50-69 yea	HIV/AIDS	Number	2012	180435	236591	141471
Prevalence	South Asia	Both	50-69 yea	HIV/AIDS	Rate	2012	92.1518	120.832	72.2524
Prevalence	South Asia	Both	50-69 yea	HIV/AIDS	Number	2013	198188	252547	156830
Prevalence	South Asia	Both	50-69 yea	HIV/AIDS	Rate	2013	97.8524	124.692	77.4325
Prevalence	South Asia	Both	50-69 yea	HIV/AIDS	Number	2014	218555	275685	173829
Prevalence	South Asia	Both	50-69 yea	HIV/AIDS	Rate	2014	104.353	131.631	82.9979
Prevalence	South Asia	Both	50-69 yea	HIV/AIDS	Number	2015	241695	303252	193296
Prevalence	South Asia	Both	50-69 yea	HIV/AIDS	Rate	2015	111.712	140.164	89.3417
Prevalence	South Asia	Both	50-69 yea	HIV/AIDS	Number	2016	266942	331831	214572
Prevalence	South Asia	Both	50-69 yea	HIV/AIDS	Rate	2016	119.537	148.594	96.0853
Prevalence	South Asia	Both	50-69 yea	HIV/AIDS	Number	2017	294640	366647	236508
Prevalence	South Asia	Both	50-69 yea	HIV/AIDS	Rate	2017	127.945	159.213	102.701
Prevalence	South Asia	Both	50-69 yea	HIV/AIDS	Number	2018	323795	401873	259221
Prevalence	South Asia	Both	50-69 yea	HIV/AIDS	Rate	2018	136.288	169.152	109.109
Prevalence	South Asia	Both	50-69 yea	HIV/AIDS	Number	2019	353233	440806	282285
Prevalence	South Asia	Both	50-69 yea	HIV/AIDS	Rate	2019	144.076	179.796	115.138
Prevalence	Central As	Both	50-69 yea	HIV/AIDS	Number	1990	317.259	411.935	245.872
Prevalence	Central As	Both	50-69 yea	HIV/AIDS	Rate	1990	3.63777	4.72334	2.81923
Prevalence	Central As	Both	50-69 yea	HIV/AIDS	Number	1991	370.035	476.267	291.327
Prevalence	Central As	Both	50-69 yea	HIV/AIDS	Rate	1991	4.17386	5.37212	3.28607
Prevalence	Central As	Both	50-69 yea	HIV/AIDS	Number	1992	414.02	540.438	331.373
Prevalence	Central As	Both	50-69 yea	HIV/AIDS	Rate	1992	4.64058	6.05756	3.71423
Prevalence	Central As	Both	50-69 yea	HIV/AIDS	Number	1993	445.346	603.211	357.452
Prevalence	Central As	Both	50-69 yea	HIV/AIDS	Rate	1993	5.02891	6.81155	4.0364
Prevalence	Central As	Both	50-69 yea	HIV/AIDS	Number	1994	464.851	634.774	369.838
Prevalence	Central As	Both	50-69 yea	HIV/AIDS	Rate	1994	5.36214	7.32224	4.26615
Prevalence	Central As	Both	50-69 yea	HIV/AIDS	Number	1995	476.078	656.85	375.359
Prevalence	Central As	Both	50-69 yea	HIV/AIDS	Rate	1995	5.63583	7.77581	4.44351
Prevalence	Central As	Both	50-69 yea	HIV/AIDS	Number	1996	487.563	677.524	384.098
Prevalence	Central As	Both	50-69 yea	HIV/AIDS	Rate	1996	5.91199	8.21539	4.65742
Prevalence	Central As	Both	50-69 yea	HIV/AIDS	Number	1997	503.884	705.161	395.228
Prevalence	Central As	Both	50-69 yea	HIV/AIDS	Rate	1997	6.20876	8.68886	4.86993
Prevalence	Central As	Both	50-69 yea	HIV/AIDS	Number	1998	524.296	744.141	411.2
Prevalence	Central As	Both	50-69 yea	HIV/AIDS	Rate	1998	6.5452	9.28969	5.13333
Prevalence	Central As	Both	50-69 yea	HIV/AIDS	Number	1999	549.527	782.934	429.186
Prevalence	Central As	Both	50-69 yea	HIV/AIDS	Rate	1999	6.94939	9.90108	5.42754
Prevalence	Central As	Both	50-69 yea	HIV/AIDS	Number	2000	580.681	835.259	448.718
Prevalence	Central As	Both	50-69 yea	HIV/AIDS	Rate	2000	7.38576	10.6238	5.7073
Prevalence	Central As	Both	50-69 yea	HIV/AIDS	Number	2001	633.465	922.834	478.65
Prevalence	Central As	Both	50-69 yea	HIV/AIDS	Rate	2001	7.96636	11.6054	6.01942
Prevalence	Central As	Both	50-69 yea	HIV/AIDS	Number	2002	691.661	1022.49	510.331
Prevalence	Central As	Both	50-69 yea	HIV/AIDS	Rate	2002	8.53912	12.6234	6.30045
Prevalence	Central As	Both	50-69 yea	HIV/AIDS	Number	2003	761.487	1135.14	546.289
Prevalence	Central As	Both	50-69 yea	HIV/AIDS	Rate	2003	9.2263	13.7535	6.61892
Prevalence	Central As	Both	50-69 yea	HIV/AIDS	Number	2004	847.137	1259.44	590.773
Prevalence	Central As	Both	50-69 yea	HIV/AIDS	Rate	2004	10.0141	14.888	6.98362
Prevalence	Central As	Both	50-69 yea	HIV/AIDS	Number	2005	954.6	1402.42	653.505
Prevalence	Central As	Both	50-69 yea	HIV/AIDS	Rate	2005	10.9836	16.1362	7.51918
Prevalence	Central As	Both	50-69 yea	HIV/AIDS	Number	2006	1074.92	1553.25	726.71
Prevalence	Central As	Both	50-69 yea	HIV/AIDS	Rate	2006	12.0302	17.3836	8.13313
Prevalence	Central As	Both	50-69 yea	HIV/AIDS	Number	2007	1217.89	1710.79	813.327
Prevalence	Central As	Both	50-69 yea	HIV/AIDS	Rate	2007	13.2399	18.5984	8.84187
Prevalence	Central As	Both	50-69 yea	HIV/AIDS	Number	2008	1401.04	1903.91	944.989
Prevalence	Central As	Both	50-69 yea	HIV/AIDS	Rate	2008	14.7428	20.0344	9.94389
Prevalence	Central As	Both	50-69 yea	HIV/AIDS	Number	2009	1640.97	2169.21	1163.76
Prevalence	Central As	Both	50-69 yea	HIV/AIDS	Rate	2009	16.6226	21.9734	11.7885
Prevalence	Central As	Both	50-69 yea	HIV/AIDS	Number	2010	1946.11	2513.29	1438.53
Prevalence	Central As	Both	50-69 yea	HIV/AIDS	Rate	2010	18.8882	24.3931	13.9619
Prevalence	Central As	Both	50-69 yea	HIV/AIDS	Number	2011	2332.42	2892.68	1821.61
Prevalence	Central As	Both	50-69 yea	HIV/AIDS	Rate	2011	21.6071	26.7973	16.8751
Prevalence	Central As	Both	50-69 yea	HIV/AIDS	Number	2012	2798.14	3379.96	2257.61
Prevalence	Central As	Both	50-69 yea	HIV/AIDS	Rate	2012	24.72	29.86	19.9447
Prevalence	Central As	Both	50-69 yea	HIV/AIDS	Number	2013	3372.83	4054.13	2764.23
Prevalence	Central As	Both	50-69 yea	HIV/AIDS	Rate	2013	28.3548	34.0824	23.2384
Prevalence	Central As	Both	50-69 yea	HIV/AIDS	Number	2014	4089.72	5076.98	3194.54

Prevalence	Southeast	Both	50-69 yea	HIV/AIDS	Rate	2019	262.533	322.623	222.969
Prevalence	High-incor	Both	50-69 yea	HIV/AIDS	Number	1990	61660.5	89770.3	40511.5
Prevalence	High-incor	Both	50-69 yea	HIV/AIDS	Rate	1990	129.759	188.913	85.2525
Prevalence	High-incor	Both	50-69 yea	HIV/AIDS	Number	1991	68422.2	100676	43959.5
Prevalence	High-incor	Both	50-69 yea	HIV/AIDS	Rate	1991	143.343	210.914	92.0945
Prevalence	High-incor	Both	50-69 yea	HIV/AIDS	Number	1992	75149.8	111244	46976.6
Prevalence	High-incor	Both	50-69 yea	HIV/AIDS	Rate	1992	156.589	231.799	97.8848
Prevalence	High-incor	Both	50-69 yea	HIV/AIDS	Number	1993	82879.3	124947	50709.6
Prevalence	High-incor	Both	50-69 yea	HIV/AIDS	Rate	1993	170.355	256.824	104.231
Prevalence	High-incor	Both	50-69 yea	HIV/AIDS	Number	1994	90033.3	138295	53907.8
Prevalence	High-incor	Both	50-69 yea	HIV/AIDS	Rate	1994	182.791	280.774	109.447
Prevalence	High-incor	Both	50-69 yea	HIV/AIDS	Number	1995	96705.7	150923	56456.3
Prevalence	High-incor	Both	50-69 yea	HIV/AIDS	Rate	1995	193.985	302.741	113.248
Prevalence	High-incor	Both	50-69 yea	HIV/AIDS	Number	1996	104677	165344	59630.6
Prevalence	High-incor	Both	50-69 yea	HIV/AIDS	Rate	1996	207.605	327.925	118.265
Prevalence	High-incor	Both	50-69 yea	HIV/AIDS	Number	1997	119142	189006	65941.5
Prevalence	High-incor	Both	50-69 yea	HIV/AIDS	Rate	1997	229.408	363.93	126.97
Prevalence	High-incor	Both	50-69 yea	HIV/AIDS	Number	1998	136403	216625	74600.6
Prevalence	High-incor	Both	50-69 yea	HIV/AIDS	Rate	1998	254.973	404.929	139.448
Prevalence	High-incor	Both	50-69 yea	HIV/AIDS	Number	1999	155118	244647	84006.7
Prevalence	High-incor	Both	50-69 yea	HIV/AIDS	Rate	1999	282.068	444.867	152.758
Prevalence	High-incor	Both	50-69 yea	HIV/AIDS	Number	2000	177412	277904	95918.6
Prevalence	High-incor	Both	50-69 yea	HIV/AIDS	Rate	2000	313.129	490.494	169.294
Prevalence	High-incor	Both	50-69 yea	HIV/AIDS	Number	2001	202951	316755	109683
Prevalence	High-incor	Both	50-69 yea	HIV/AIDS	Rate	2001	347.802	542.832	187.966
Prevalence	High-incor	Both	50-69 yea	HIV/AIDS	Number	2002	230302	357618	124319
Prevalence	High-incor	Both	50-69 yea	HIV/AIDS	Rate	2002	382.356	593.731	206.399
Prevalence	High-incor	Both	50-69 yea	HIV/AIDS	Number	2003	260492	402338	140278
Prevalence	High-incor	Both	50-69 yea	HIV/AIDS	Rate	2003	418.401	646.234	225.314
Prevalence	High-incor	Both	50-69 yea	HIV/AIDS	Number	2004	294382	452969	158111
Prevalence	High-incor	Both	50-69 yea	HIV/AIDS	Rate	2004	456.636	702.631	245.256
Prevalence	High-incor	Both	50-69 yea	HIV/AIDS	Number	2005	331294	512588	177015
Prevalence	High-incor	Both	50-69 yea	HIV/AIDS	Rate	2005	495.951	767.35	264.994
Prevalence	High-incor	Both	50-69 yea	HIV/AIDS	Number	2006	370361	575561	196721
Prevalence	High-incor	Both	50-69 yea	HIV/AIDS	Rate	2006	535.734	832.559	284.56
Prevalence	High-incor	Both	50-69 yea	HIV/AIDS	Number	2007	409886	634699	216446
Prevalence	High-incor	Both	50-69 yea	HIV/AIDS	Rate	2007	572.763	886.91	302.456
Prevalence	High-incor	Both	50-69 yea	HIV/AIDS	Number	2008	450402	693902	235713
Prevalence	High-incor	Both	50-69 yea	HIV/AIDS	Rate	2008	608.475	937.433	318.438
Prevalence	High-incor	Both	50-69 yea	HIV/AIDS	Number	2009	491661	754975	255465
Prevalence	High-incor	Both	50-69 yea	HIV/AIDS	Rate	2009	643.571	988.24	334.397
Prevalence	High-incor	Both	50-69 yea	HIV/AIDS	Number	2010	535009	822688	276198
Prevalence	High-incor	Both	50-69 yea	HIV/AIDS	Rate	2010	678.431	1043.23	350.24
Prevalence	High-incor	Both	50-69 yea	HIV/AIDS	Number	2011	578546	889087	297174
Prevalence	High-incor	Both	50-69 yea	HIV/AIDS	Rate	2011	712.545	1095.01	366.003
Prevalence	High-incor	Both	50-69 yea	HIV/AIDS	Number	2012	619799	956481	317359
Prevalence	High-incor	Both	50-69 yea	HIV/AIDS	Rate	2012	743.97	1148.1	380.939
Prevalence	High-incor	Both	50-69 yea	HIV/AIDS	Number	2013	659834	1018537	338526
Prevalence	High-incor	Both	50-69 yea	HIV/AIDS	Rate	2013	775.53	1197.13	397.883
Prevalence	High-incor	Both	50-69 yea	HIV/AIDS	Number	2014	700741	1077579	360840
Prevalence	High-incor	Both	50-69 yea	HIV/AIDS	Rate	2014	806.806	1240.68	415.457
Prevalence	High-incor	Both	50-69 yea	HIV/AIDS	Number	2015	739507	1128168	382114
Prevalence	High-incor	Both	50-69 yea	HIV/AIDS	Rate	2015	835.478	1274.58	431.703
Prevalence	High-incor	Both	50-69 yea	HIV/AIDS	Number	2016	774519	1179726	403104
Prevalence	High-incor	Both	50-69 yea	HIV/AIDS	Rate	2016	861.688	1312.5	448.472
Prevalence	High-incor	Both	50-69 yea	HIV/AIDS	Number	2017	802077	1211199	419735
Prevalence	High-incor	Both	50-69 yea	HIV/AIDS	Rate	2017	888.21	1341.27	464.809
Prevalence	High-incor	Both	50-69 yea	HIV/AIDS	Number	2018	826106	1243785	434638
Prevalence	High-incor	Both	50-69 yea	HIV/AIDS	Rate	2018	911.923	1372.99	479.788
Prevalence	High-incor	Both	50-69 yea	HIV/AIDS	Number	2019	849670	1279468	448969
Prevalence	High-incor	Both	50-69 yea	HIV/AIDS	Rate	2019	932.895	1404.79	492.945
Prevalence	High-incor	Both	50-69 yea	Sexually tr	Number	1990	6687246	7513427	5946694
Prevalence	High-incor	Both	50-69 yea	Sexually tr	Rate	1990	19602.7	22024.6	17431.9
Prevalence	High-incor	Both	50-69 yea	Sexually tr	Number	1991	6779066	7672523	5988439
Prevalence	High-incor	Both	50-69 yea	Sexually tr	Rate	1991	19300.5	21844.2	17049.5
Prevalence	High-incor	Both	50-69 yea	Sexually tr	Number	1992	6866698	7837548	6019238
Prevalence	High-incor	Both	50-69 yea	Sexually tr	Rate	1992	19006.2	21693.4	16660.5
Prevalence	High-incor	Both	50-69 yea	Sexually tr	Number	1993	6921498	7945283	6010998
Prevalence	High-incor	Both	50-69 yea	Sexually tr	Rate	1993	18733.1	21504	16268.8
Prevalence	High-incor	Both	50-69 yea	Sexually tr	Number	1994	6995967	8069812	6029847
Prevalence	High-incor	Both	50-69 yea	Sexually tr	Rate	1994	18535.5	21380.6	15975.8
Prevalence	High-incor	Both	50-69 yea	Sexually tr	Number	1995	7020286	8140604	6017100
Prevalence	High-incor	Both	50-69 yea	Sexually tr	Rate	1995	18437.6	21380	15802.9
Prevalence	High-incor	Both	50-69 yea	Sexually tr	Number	1996	7030503	8143273	6034117
Prevalence	High-incor	Both	50-69 yea	Sexually tr	Rate	1996	18368.7	21276	15765.4
Prevalence	High-incor	Both	50-69 yea	Sexually tr	Number	1997	7187359	8311058	6181064
Prevalence	High-incor	Both	50-69 yea	Sexually tr	Rate	1997	18255.3	21109.4	15699.4
Prevalence	High-incor	Both	50-69 yea	Sexually tr	Number	1998	7348468	8493456	6329257
Prevalence	High-incor	Both	50-69 yea	Sexually tr	Rate	1998	18129.8	20954.7	15615.2
Prevalence	High-incor	Both	50-69 yea	Sexually tr	Number	1999	7505096	8653274	6463940
Prevalence	High-incor	Both	50-69 yea	Sexually tr	Rate	1999	18022.4	20779.5	15522.2
Prevalence	High-incor	Both	50-69 yea	Sexually tr	Number	2000	7642543	8810895	6590891
Prevalence	High-incor	Both	50-69 yea	Sexually tr	Rate	2000	17975.3	20723.2	15501.8
Prevalence	High-incor	Both	50-69 yea	Sexually tr	Number	2001	7752996	8936116	6688320
Prevalence	High-incor	Both	50-69 yea	Sexually tr	Rate	2001	17968	20710	15500.6
Prevalence	High-incor	Both	50-69 yea	Sexually tr	Number	2002	7865774	9056717	6789483
Prevalence	High-incor	Both	50-69 yea	Sexually tr	Rate	2002	17983.2	20706	15522.5
Prevalence	High-incor	Both	50-69 yea	Sexually tr	Number	2003	7946605	9161436	6861390
Prevalence	High-incor	Both	50-69 yea	Sexually tr	Rate	2003	18002.6	20754.7	15544.1
Prevalence	High-incor	Both	50-69 yea	Sexually tr	Number	2004	8029790	9282836	6932773
Prevalence	High-incor	Both	50-69 yea	Sexually tr	Rate	2004	18033.5	20847.6	15569.8
Prevalence	High-incor	Both	50-69 yea	Sexually tr	Number	2005	8129335	9412537	7002104
Prevalence	High-incor	Both	50-69 yea	Sexually tr	Rate	2005	18094.2	20950.4	15585.3
Prevalence	High-incor	Both	50-69 yea	Sexually tr	Number	2006	8203424	9499385	7065233
Prevalence	High-incor	Both	50-69 yea	Sexually tr	Rate	2006	18151.5	21019	15633
Prevalence	High-incor	Both	50-69 yea	Sexually tr	Number	2007	8265323	9582970	7105614
Prevalence	High-incor	Both	50-69 yea	Sexually tr	Rate	2007	18189.2	21088.9	15637.1
Prevalence	High-incor	Both	50-69 yea	Sexually tr	Number	2008	8354389	9689546	7199481
Prevalence	High-incor	Both	50-69 yea	Sexually tr	Rate	2008	18228	21141.2	15708.2
Prevalence	High-incor	Both	50-69 yea	Sexually tr	Number	2009	8468436	9841790	7293068
Prevalence	High-incor	Both	50-69 yea	Sexually tr	Rate	2009	18256.2	21216.8	15722.3
Prevalence	High-incor	Both	50-69 yea	Sexually tr	Number	2010	8567686	9957822	7379305
Prevalence	High-incor	Both	50-69 yea	Sexually tr	Rate	2010	18313.5	21285	15773.4
Prevalence	High-incor	Both	50-69 yea	Sexually tr	Number	2011	8647361	1E+07	7430888
Prevalence	High-incor	Both	50-69 yea	Sexually tr	Rate	2011	18389.1	21380	15802.2
Prevalence	High-incor	Both	50-69 yea	Sexually tr	Number	2012	8709509	1E+07	7494384
Prevalence	High-incor	Both	50-69 yea	Sexually tr	Rate	2012	18446.1	21454.3	15872.6
Prevalence	High-incor	Both	50-69 yea	Sexually tr	Number	2013	8803942	1E+07	7591174
Prevalence	High-incor	Both	50-69 yea	Sexually tr	Rate	2013	18502.6	21496.9	15953.8
Prevalence	High-incor	Both	50-69 yea	Sexually tr	Number	2014	8891475	1E+07	7679775
Prevalence	High-incor	Both	50-69 yea	Sexually tr	Rate	2014	18551.8	21573.9	16023.6
Prevalence	High-incor	Both	50-69 yea	Sexually tr	Number	2015	9055518	1.1E+07	7805459
Prevalence	High-incor	Both	50-69 yea	Sexually tr	Rate	2015	18580.8	21584.6	16015.8
Prevalence	High-incor	Both	50-69 yea	Sexually tr	Number	2016	9165017	1.1E+07	7885598
Prevalence	High-incor	Both	50-69 yea	Sexually tr	Rate	2016	18590.3	21596.8	15995.1
Prevalence	High-incor	Both	50-69 yea	Sexually tr	Number	2017	9204206	1.1E+07	7946073
Prevalence	High-incor	Both	50-69 yea	Sexually tr	Rate	2017	18638.8	21637.4	16091
Prevalence	High-incor	Both	50-69 yea	Sexually tr	Number	2018	9256712	1.1E+07	7967912
Prevalence	High-incor	Both	50-69 yea	Sexually tr	Rate	2018	18718.2	21755.3	16112.1
Prevalence	High-incor	Both	50-69 yea	Sexually tr	Number	2019	9317871	1.1E+07	8018260
Prevalence	High-incor	Both	50-69 yea	Sexually tr	Rate	2019	18802.6	21861.9	16180.1
Prevalence	Southern L	Both	50-69 yea	Sexually tr	Number	1990	2383990	2557579	2223012
Prevalence	Southern L	Both	50-69 yea	Sexually tr	Rate	1990	31838.9	34157.3	29689
Prevalence	Southern L	Both	50-69 yea	Sexually tr	Number	1991	2414233	2590535	2250247
Prevalence	Southern L	Both	50-69 yea	Sexually tr	Rate	1991	31842.4	34167.7	29679.5
Prevalence	Southern L	Both	50-69 yea	Sexually tr	Number	1992	2443519	2624339	2279574
Prevalence	Southern L	Both	50-69 yea	Sexually tr	Rate	1992	31846.4	34203	29709.7
Prevalence	Southern L	Both	50-69 yea	Sexually tr	Number	1993	2472861	2660994	2306314
Prevalence	Southern L	Both	50-69 yea	Sexually tr	Rate	1993	31855	34278.5	29709.6
Prevalence	Southern L	Both	50-69 yea	Sexually tr	Number	1994	2502367	2687775	2334167
Prevalence	Southern L	Both	50-69 yea	Sexually tr	Rate	1994	31859.8	34220.4	29718.3
Prevalence	Southern L	Both	50-69 yea	Sexually tr	Number	1995	2532698	2724836	2355607
Prevalence	Southern L	Both	50-69 yea	Sexually tr	Rate	1995	31857.7	34274.5	29630.2
Prevalence	Southern L	Both	50-69 yea	Sexually tr	Number	1996	2563355	2755433	2388569
Prevalence	Southern L	Both	50-69 yea	Sexually tr	Rate	1996	31859.6	34246.9	29687.2
Prevalence	Southern L	Both	50-69 yea	Sexually tr	Number	1997	2595815	2788150	2412786
Prevalence	Southern L	Both	50-69 yea	Sexually tr	Rate	1997	31860.8	34221.5	29614.3
Prevalence	Southern L	Both	50-69 yea	Sexually tr	Number	1998	2632133	2828777	2451071
Prevalence	Southern L	Both	50-69 yea	Sexually tr	Rate	1998	31864.5	34245.1	29672.6
Prevalence	Southern L	Both	50-69 yea	Sexually tr	Number	1999	2672642	2870510	2487861
Prevalence	Southern L	Both	50-69 yea	Sexually tr	Rate	1999	31877.6	34237.7	29673.7
Prevalence	Southern L	Both	50-69 yea	Sexually tr	Number	2000	2715589	2919500	2526456
Prevalence	Southern L	Both	50-69 yea	Sexually tr	Rate	2000	31876.2	34269.8	29656.1
Prevalence	Southern L	Both	50-69 yea	Sexually tr	Number	2001	2761247	2973415	2570731
Prevalence	Southern L	Both	50-69 yea	Sexually tr	Rate	2001	31877.1	34326.4	29677.7
Prevalence	Southern L	Both	50-69 yea	Sexually tr	Number	2002	2811237	3023308	2611006
Prevalence	Southern L	Both	50-69 yea	Sexually tr	Rate	2002	31885.9	34291.2	29614.8
Prevalence	Southern L	Both	50-69 yea	Sexually tr	Number	2003	2866740	3077505	2661650
Prevalence	Southern L	Both	50-69 yea	Sexually tr	Rate	2003	31889.1	34233.6	29607.7
Prevalence	Southern L	Both	50-69 yea	Sexually tr	Number	2004	2927738	3143548	2720939
Prevalence	Southern L	Both	50-69 yea	Sexually tr	Rate	2004	31894.8	34245.9	29641.9
Prevalence	Southern L	Both	50-69 yea	Sexually tr	Number	2005	2992875	3217418	2782315
Prevalence	Southern L	Both	50-69 yea	Sexually tr	Rate	2005	31885.5	34277.7	29642.2
Prevalence	Southern L	Both	50-69 yea	Sexually tr	Number	2006	3057249	3298958	2823640
Prevalence	Southern L	Both	50-69 yea	Sexually tr	Rate	2006	31815.3	34330.7	29384.3
Prevalence	Southern L	Both	50-69 yea	Sexually tr	Number	2007	3118916	3405331	2856035
Prevalence	Southern L	Both	50-69 yea	Sexually tr	Rate	2007	31682.1	34591.6	29011.8
Prevalence	Southern L	Both	50-69 yea	Sexually tr	Number	2008	3176967	3535231	2863324
Prevalence	Southern L	Both	50-69 yea	Sexually tr	Rate	2008	31511.2	35064.7	28400.3
Prevalence	Southern L	Both	50-69 yea	Sexually tr	Number	2009	3237873	3660236	2869471
Prevalence	Southern L	Both	50-69 yea	Sexually tr	Rate	2009	31367.2	35458.9	27798.3
Prevalence	Southern L	Both	50-69 yea	Sexually tr	Number	2010	3307091	3804079	2890650
Prevalence	Southern L	Both	50-69 yea	Sexually tr	Rate	2010	31301.4	36005.3	27359.8
Prevalence	Southern L	Both	50-69 yea	Sexually tr	Number	2011	3383280	3887412	2948717
Prevalence	Southern L	Both	50-69 yea	Sexually tr	Rate	2011	31300.4	35964.4	27280.1
Prevalence	Southern L	Both	50-69 yea	Sexually tr	Number	2012	3462405	3970263	3026073
Prevalence	Southern L	Both	50-69 yea	Sexually tr	Rate	2012	31320.1	35914.1	27373.1
Prevalence	Southern L	Both	50-69 yea	Sexually tr	Number	2013	3541793	4063021	3083589
Prevalence	Southern L	Both	50-69 yea	Sexually tr	Rate	2013	31340.5	35952.7	27285.9
Prevalence	Southern L	Both	50-69 yea	Sexually tr	Number	2014	3618256	4131878	3163505
Prevalence	Southern L	Both	50-69 yea	Sexually tr	Rate	2014	31353.5	35804.2	27412.9
Prevalence	Southern L	Both	50-69 yea	Sexually tr	Number	2015	3688667	4223114	3225701
Prevalence	Southern L	Both	50-69 yea	Sexually tr	Rate	2015	31350.4	35892.8	27415.6
Prevalence	Southern L	Both	50-69 yea	Sexually tr	Number	2016	3748026	4285964	3270019
Prevalence	Southern L	Both	50-69 yea	Sexually tr	Rate	2016	31294.8	35786.5	27303.6
Prevalence	Southern L	Both	50-69 yea	Sexually tr	Number	2017	3799367	4354382	3299503
Prevalence	Southern L	Both	50-69 yea	Sexually tr	Rate	2017	31237.1	35800.2	27127.4
Prevalence	Southern L	Both	50-69 yea	Sexually tr	Number	2018	3859362	4407401	3363327
Prevalence	Southern L	Both	50-69 yea	Sexually tr	Rate	2018	31237.1	35672.8	27222.3
Prevalence	Southern L	Both	50-69 yea	Sexually tr	Number	2019	3933325	4498206	3436409
Prevalence	Southern L	Both	50-69 yea	Sexually tr	Rate	2019	31269.4	35760.1	27318.9
Prevalence	Australasia	Both	50-69 yea	HIV/AIDS	Number	1990	1006.64	1218.42	776.348
Prevalence	Australasia	Both	50-69 yea	HIV/AIDS	Rate	1990	29.2484	35.4019	22.5572
Prevalence	Australasia	Both	50-69 yea	HIV/AIDS	Number	1991	1081.94	1321.92	824.63
Prevalence	Australasia	Both	50-69 yea	HIV/AIDS	Rate	1991	31.1045	38.0036	23.707
Prevalence	Australasia	Both	50-69 yea	HIV/AIDS	Number	1992	1156.29	1420.49	870.785
Prevalence	Australasia	Both	50-69 yea	HIV/AIDS	Rate	1992	32.8227	40.3223	24.7183
Prevalence	Australasia	Both	50-69 yea	HIV/AIDS	Number	1993	1226.83	1525.18	914.893
Prevalence	Australasia	Both	50-69 yea	HIV/AIDS	Rate	1993	34.3994	42.7647	25.6529
Prevalence	Australasia	Both	50-69 yea	HIV/AIDS	Number	1994	1305.04	1639.14	958.959
Prevalence	Australasia	Both	50-69 yea	HIV/AIDS	Rate	1994	35.9499	45.1534	26.4165
Prevalence	Australasia	Both	50-69 yea	HIV/AIDS	Number	1995	1383.51	1752.66	995.634
Prevalence	Australasia	Both	50-69 yea	HIV/AIDS	Rate	1995	37.3972	47.3756	26.9127
Prevalence	Australasia	Both	50-69 yea	HIV/AIDS	Number	1996	1473.39	1867.15	1048.07
Prevalence	Australasia	Both	50-69 yea	HIV/AIDS	Rate	1996	38.9833	49.4014	27.7299
Prevalence	Australasia	Both	50-69 yea	HIV/AIDS	Number	1997	1617.52	2049.37	1155.74
Prevalence	Australasia	Both	50-69 yea	HIV/AIDS	Rate	1997	41.3568	52.3981	29.5499
Prevalence	Australasia	Both	50-69 yea	HIV/AIDS	Number	1998	1796	2255.47	1298
Prevalence	Australasia	Both	50-69 yea	HIV/AIDS	Rate	1998	44.5597	55.9592	32.2041
Prevalence	Australasia	Both	50-69 yea	HIV/AIDS	Number	1999	2017.05	2483.11	1464.24
Prevalence	Australasia	Both	50-69 yea	HIV/AIDS	Rate	1999	48.5965	59.8253	35.2777
Prevalence	Australasia	Both	50-69 yea	HIV/AIDS	Number	2000	2278.55	2776.77	1653.42
Prevalence	Australasia	Both	50-69 yea	HIV/AIDS	Rate	2000	53.2599	64.9058	38.648
Prevalence	Australasia	Both	50-69 yea	HIV/AIDS	Number	2001	2554.41	3109.93	1850.74
Prevalence	Australasia	Both	50-69 yea	HIV/AIDS	Rate	2001	57.9371	70.5369	41.9769
Prevalence	Australasia	Both	50-69 yea	HIV/AIDS	Number	2002	2834.09	3446.43	2054.13
Prevalence	Australasia	Both	50-69 yea	HIV/AIDS	Rate	2002	62.3127	75.7761	45.1638
Prevalence	Australasia	Both	50-69 yea	HIV/AIDS	Number	2003	3136.63	3817.2	2299.65
Prevalence	Australasia	Both	50-69 yea	HIV/AIDS	Rate	2003	66.7954	81.2884	48.9717
Prevalence	Australasia	Both	50-69 yea	HIV/AIDS	Number	2004	3453.52	4202.67	2560.68
Prevalence	Australasia	Both	50-69 yea	HIV/AIDS	Rate	2004	71.304	86.7715	52.8699
Prevalence	Australasia	Both	50-69 yea	HIV/AIDS	Number	2005	3792.34	4618.78	2845.83
Prevalence	Australasia	Both	50-69 yea	HIV/AIDS	Rate	2005	75.9283	92.475	56.9778
Prevalence	Australasia	Both	50-69 yea	HIV/AIDS	Number	2006	4142.76	5043.95	3115.54
Prevalence	Australasia	Both	50-69 yea	HIV/AIDS	Rate	2006	80.5245	98.0412	60.5579
Prevalence	Australasia	Both	50-69 yea	HIV/AIDS	Number	2007	4486.65	5452.14	3375.08
Prevalence	Australasia	Both	50-69 yea	HIV/AIDS	Rate	2007	84.813	103.064	63.8006
Prevalence	Australasia	Both	50-69 yea	HIV/AIDS	Number	2008	4846.94	5920.15	3630.32
Prevalence	Australasia	Both	50-69 yea	HIV/AIDS	Rate	2008	89.1625	108.905	66.782
Prevalence	Australasia	Both	50-69 yea	HIV/AIDS	Number	2009	5224.96	6389	3900.28
Prevalence	Australasia	Both	50-69 yea	HIV/AIDS	Rate	2009	93.6161	114.472	69.8818
Prevalence	Australasia	Both	50-69 yea	HIV/AIDS	Number	2010	5613.14	6910.45	4182.65
Prevalence	Australasia	Both	50-69 yea	HIV/AIDS	Rate	2010	97.9908	120.639	73.0183
Prevalence	Australasia	Both	50-69 yea	HIV/AIDS	Number	2011	6013.96	7431.8	4456.52
Prevalence	Australasia	Both	50-69 yea	HIV/AIDS	Rate	2011	102.259	126.367	75.7769
Prevalence	Australasia	Both	50-69 yea	HIV/AIDS	Number	2012	6414	7945.21	4728.65
Prevalence	Australasia	Both	50-69 yea	HIV/AIDS	Rate	2012	106.365	131.757	78.416
Prevalence	Australasia	Both	50-69 yea	HIV/AIDS	Number	2013	6819.34	8459.97	5010.59
Prevalence	Australasia	Both	50-69 yea	HIV/AIDS	Rate	2013	110.273	136.803	81.0244
Prevalence	Australasia	Both	50-69 yea	HIV/AIDS	Number	2014	7194.57	8936.08	5264.98
Prevalence	Australasia	Both	50-69 yea	HIV/AIDS	Rate	2014	113.923	141.499	83.3688
Prevalence	Australasia	Both	50-69 yea	HIV/AIDS	Number	2015	7535.63	9361.52	5500.67
Prevalence	Australasia	Both	50-69 yea	HIV/AIDS	Rate	2015	117.226	145.63	85.5699
Prevalence	Australasia	Both	50-69 yea	HIV/AIDS	Number	2016	7847.38	9758.57	5697.31
Prevalence	Australasia	Both	50-69 yea	HIV/AIDS	Rate	2016	120.233	149.515	87.2909
Prevalence	Australasia	Both	50-69 yea	HIV/AIDS	Number	2017	8097.38	10102.3	5846.42
Prevalence	Australasia	Both	50-69 yea	HIV/AIDS	Rate	2017	123.032	153.495	88.8311
Prevalence	Australasia	Both	50-69 yea	HIV/AIDS	Number	2018	8341.39	10445.1	6006.99
Prevalence	Australasia	Both	50-69 yea	HIV/AIDS	Rate	2018	125.361	156.976	90.2775
Prevalence	Australasia	Both	50-69 yea	HIV/AIDS	Number	2019	8581.65	10853.4	6153.35
Prevalence	Australasia	Both	50-69 yea	HIV/AIDS	Rate	2019	127.335	161.042	91.3036
Prevalence	Eastern Eu	Both	50-69 yea	Sexually tr	Number	1990	1.3E+07	1.4E+07	1.1E+07
Prevalence	Eastern Eu	Both	50-69 yea	Sexually tr	Rate	1990	25208.9	29091.7	21783.5
Prevalence	Eastern Eu	Both	50-69 yea	Sexually tr	Number	1991	1.3E+07	1.5E+07	1.1E+07
Prevalence	Eastern Eu	Both	50-69 yea	Sexually tr	Rate	1991	25145.6	28938.1	21745.7
Prevalence	Eastern Eu	Both	50-69 yea	Sexually tr	Number	1992	1.3E+07	1.5E+07	1.1E+07
Prevalence	Eastern Eu	Both	50-69 yea	Sexually tr	Rate	1992	25065.1	28775.2	21666.9
Prevalence	Eastern Eu	Both	50-69 yea	Sexually tr	Number	1993	1.3E+07	1.4E+07	1.1E+07
Prevalence	Eastern Eu	Both	50-69 yea	Sexually tr	Rate	1993	24956.5	28684.2	21647.6
Prevalence	Eastern Eu	Both	50-69 yea	Sexually tr	Number	1994	1.2E+07	1.4E+07	1E+07
Prevalence	Eastern Eu	Both	50-69 yea	Sexually tr	Rate	1994	24849.2	28496.9	21473.2
Prevalence	Eastern Eu	Both	50-69 yea	Sexually tr	Number	1995	1.2E+07	1.3E+07	1E+07
Prevalence	Eastern Eu	Both	50-69 yea	Sexually tr	Rate	1995	24759.5	28393.9	21465.2
Prevalence	Eastern Eu	Both	50-69 yea	Sexually tr	Number	1996	1.1E+07	1.3E+07	9831568
Prevalence	Eastern Eu	Both	50-69 yea	Sexually tr	Rate	1996	24684.8	28316.1	21424.9
Prevalence	Eastern Eu	Both	50-69 yea	Sexually tr	Number	1997	1.1E+07	1.3E+07	9704901
Prevalence	Eastern Eu	Both	50-69 yea	Sexually tr	Rate	1997	24657.3	28314.2	21401
Prevalence	Eastern Eu	Both	50-69 yea	Sexually tr	Number	1998	1.1E+07	1.3E+07	9615571
Prevalence	Eastern Eu	Both	50-69 yea	Sexually tr	Rate	1998	24666.9	28339.9	21413.9
Prevalence	Eastern Eu	Both	50-69 yea	Sexually tr	Number	1999	1.1E+07	1.3E+07	9579703
Prevalence	Eastern Eu	Both	50-69 yea	Sexually tr	Rate	1999	24721.2	28406.8	21447.3
Prevalence	Eastern Eu	Both	50-69 yea	Sexually tr	Number	2000	1.1E+07	1.3E+07	9659981
Prevalence	Eastern Eu	Both	50-69 yea	Sexually tr	Rate	2000	24802.5	28526.8	21556.1
Prevalence	Eastern Eu	Both	50-69 yea	Sexually tr	Number	2001	1.1E+07	1.3E+07	9718649
Prevalence	Eastern Eu	Both	50-69 yea	Sexually tr	Rate	2001	24879.4	28591	21612.8
Prevalence	Eastern Eu	Both	50-69 yea	Sexually tr	Number	2002	1.1E+07	1.3E+07	9823459
Prevalence	Eastern Eu	Both	50-69 yea	Sexually tr	Rate	2002	24919.5	28592.8	21648
Prevalence	Eastern Eu	Both	50-69 yea	Sexually tr	Number	2003	1.1E+07	1.3E+07	9939960
Prevalence	Eastern Eu	Both	50-69 yea	Sexually tr	Rate	2003	24986.5	28704.5	21702.8
Prevalence	Eastern Eu	Both	50-69 yea	Sexually tr	Number	2004	1.2E+07	1.3E+07	1E+07
Prevalence	Eastern Eu	Both	50-69 yea	Sexually tr	Rate	2004	25045	28795.5	21810.3
Prevalence	Eastern Eu	Both	50-69 yea	Sexually tr	Number	2005	1.2E+07	1.4E+07	1E+07
Prevalence	Eastern Eu	Both	50-69 yea	Sexually tr	Rate	2005	25100.2	28851.8	21818.9
Prevalence	Eastern Eu	Both	50-69 yea	Sexually tr	Number	2006	1.2E+07	1.4E+07	1E+07
Prevalence	Eastern Eu	Both	50-69 yea	Sexually tr	Rate	2006	25160.3	28920.5	21835.1
Prevalence	Eastern Eu	Both	50-69 yea	Sexually tr	Number	2007	1.2E+07	1.4E+07	1E+07
Prevalence	Eastern Eu	Both	50-69 yea	Sexually tr	Rate	2007	25203.5	28976.1	21882.3
Prevalence	Eastern Eu	Both	50-69 yea	Sexually tr	Number	2008	1.2E+07	1.4E+07	1.1E+07
Prevalence	Eastern Eu	Both	50-69 yea	Sexually tr	Rate	2008	25268.3	29079	21903.9
Prevalence	Eastern Eu	Both	50-69 yea	Sexually tr	Number	2009	1.2E+07	1.4E+07	1.1E+07
Prevalence	Eastern Eu	Both	50-69 yea	Sexually tr	Rate	2009	25316.8	29167.5	21949.7
Prevalence	Eastern Eu	Both	50-69 yea	Sexually tr	Number	2010	1.2E+07	1.4E+07	1.1E+07
Prevalence	Eastern Eu	Both	50-69 yea	Sexually tr	Rate	2010	25336.6	29199.8	21936.2
Prevalence	Eastern Eu	Both	50-69 yea	Sexually tr	Number	2011	1.3E+07	1.5E+07	1.1E+07
Prevalence	Eastern Eu	Both	50-69 yea	Sexually tr	Rate	2011	25331	29163.9	21964.2
Prevalence	Eastern Eu	Both	50-69 yea	Sexually tr	Number	2012	1.3E+07	1.5E+07	1.1E+07
Prevalence	Eastern Eu	Both	50-69 yea	Sexually tr	Rate	2012	25284.8	29117.5	21944.6
Prevalence	Eastern Eu	Both	50-69 yea	Sexually tr	Number	2013	1.3E+07	1.5E+07	1.1E+07
Prevalence	Eastern Eu	Both	50-69 yea	Sexually tr	Rate	2013	25211	29054.6	21861.2
Prevalence	Eastern Eu	Both	50-69 yea	Sexually tr	Number	2014	1.3E+07	1.5E+07	1.2E+07
Prevalence	Eastern Eu	Both	50-69 yea	Sexually tr	Rate	2014	25119.8	28939.9	21792.6
Prevalence	Eastern Eu	Both	50-69 yea	Sexually tr	Number	2015	1.4E+07	1.6E+07	1.2E+07
Prevalence	Eastern Eu	Both	50-69 yea	Sexually tr	Rate	2015	25019.5	28788.8	21672.5
Prevalence	Eastern Eu	Both	50-69 yea	Sexually tr	Number	2016	1.4E+07	1.6E+07	1.2E+07
Prevalence	Eastern Eu	Both	50-69 yea	Sexually tr	Rate	2016	24930.5	28693.1	21581.2
Prevalence	Eastern Eu	Both	50-69 yea	Sexually tr	Number	2017	1.4E+07	1.6E+07	1.2E+07
Prevalence	Eastern Eu	Both	50-69 yea	Sexually tr	Rate	2017	24864.3	28612.3	21589.4
Prevalence	Eastern Eu	Both	50-69 yea	Sexually tr	Number	2018	1.4E+07	1.6E+07	1.2E+07
Prevalence	Eastern Eu	Both	50-69 yea	Sexually tr	Rate	2018	24817	28568.5	21554.9
Prevalence	Eastern Eu	Both	50-69 yea	Sexually tr	Number	2019	1.3E+07	1.6E+07	1.2E+07
Prevalence	Eastern Eu	Both	50-69 yea	Sexually tr	Rate	2019	24793.4	28595.8	21441.5
Prevalence	Central Lat	Both	50-69 yea	Sexually tr	Number	1990	5785786	6467213	5180436
Prevalence	Central Lat	Both	50-69 yea	Sexually tr	Rate	1990	40144.3	44872.3	35944.1
Prevalence	Central Lat	Both	50-69 yea	Sexually tr	Number	1991	5921257	6605982	5308523
Prevalence	Central Lat	Both	50-69 yea	Sexually tr	Rate	1991	39835	44441.5	35712.9
Prevalence	Central Lat	Both	50-69 yea	Sexually tr	Number	1992	6058413	6752080	5434842
Prevalence	Central Lat	Both	50-69 yea	Sexually tr	Rate	1992	39488.5	44009.8	35424.1
Prevalence	Central Lat	Both	50-69 yea	Sexually tr	Number	1993	6203722	6910826	5566700
Prevalence	Central Lat	Both	50-69 yea	Sexually tr	Rate	1993	39163.4	43627.3	35142
Prevalence	Central Lat	Both	50-69 yea	Sexually tr	Number	1994	6369723	7085827	5711945
Prevalence	Central Lat	Both	50-69 yea	Sexually tr	Rate	1994	38918.7	43294	34899.7
Prevalence	Central Lat	Both	50-69 yea	Sexually tr	Number	1995	6560815	7294083	5890829
Prevalence	Central Lat	Both	50-69 yea	Sexually tr	Rate	1995	38807.2	43144.5	34844.2
Prevalence	Central Lat	Both	50-69 yea	Sexually tr	Number	1996	6771092	7530805	6073573
Prevalence	Central Lat	Both	50-69 yea	Sexually tr	Rate	1996	38672.1	43011.1	34688.3
Prevalence	Central Lat	Both	50-69 yea	Sexually tr	Number	1997	6965725	7759425	6239591
Prevalence	Central Lat	Both	50-69 yea	Sexually tr	Rate	1997	38353.2	42723.3	34355.1
Prevalence	Central Lat	Both	50-69 yea	Sexually tr	Number	1998	7148493	7975752	6389349
Prevalence	Central Lat	Both	50-69 yea	Sexually tr	Rate	1998	37953.1	42345.3	33922.6
Prevalence	Central Lat	Both	50-69 yea	Sexually tr	Number	1999	7347138	8208558	6548329
Prevalence	Central Lat	Both	50-69 yea	Sexually tr	Rate	1999	37623.8	42035	33533.2
Prevalence	Central Lat	Both	50-69 yea	Sexually tr	Number	2000	7549967	8457860	6719781
Prevalence	Central Lat	Both	50-69 yea	Sexually tr	Rate	2000	37458.5	41962.9	33339.6
Prevalence	Central Lat	Both	50-69 yea	Sexually tr	Number	2001	7845459	8801134	6978355
Prevalence	Central Lat	Both	50-69 yea	Sexually tr	Rate	2001	37473.2	42037.9	33331.5
Prevalence	Central Lat	Both	50-69 yea	Sexually tr	Number	2002	8154287	9135235	7254578
Prevalence	Central Lat	Both	50-69 yea	Sexually tr	Rate	2002	37522.5	42036.4	33382.4
Prevalence	Central Lat	Both	50-69 yea	Sexually tr	Number	2003	8477644	9498225	7529746
Prevalence	Central Lat	Both	50-69 yea	Sexually tr	Rate	2003	37588.4	42113.5	33385.6
Prevalence	Central Lat	Both	50-69 yea	Sexually tr	Number	2004	8817697	9896317	7837962
Prevalence	Central Lat	Both	50-69 yea	Sexually tr	Rate	2004	37649.1	42254.5	33465.9
Prevalence	Central Lat	Both	50-69 yea	Sexually tr	Number	2005	9166216	1E+07	8135418
Prevalence	Central Lat	Both	50-69 yea	Sexually tr	Rate	2005	37683.6	42345.3	33445.8
Prevalence	Central Lat	Both	50-69 yea	Sexually tr	Number	2006	9558585	1.1E+07	8474401
Prevalence	Central Lat	Both	50-69 yea	Sexually tr	Rate	2006	37830.4	42520.9	33539.5
Prevalence	Central Lat	Both	50-69 yea	Sexually tr	Number	2007	1E+07	1.1E+07	8889903
Prevalence	Central Lat	Both	50-69 yea	Sexually tr	Rate	2007	38204.2	43024.8	33861.5
Prevalence	Central Lat	Both	50-69 yea	Sexually tr	Number	2008	1.1E+07	1.2E+07	9330030
Prevalence	Central Lat	Both	50-69 yea	Sexually tr	Rate	2008	38666.1	43596.9	34199.6
Prevalence	Central Lat	Both	50-69 yea	Sexually tr	Number	2009	1.1E+07	1.2E+07	9782942
Prevalence	Central Lat	Both	50-69 yea	Sexually tr	Rate	2009	39069.1	44066.1	34504.5
Prevalence	Central Lat	Both	50-69 yea	Sexually tr	Number	2010	1.2E+07	1.3E+07	1E+07
Prevalence	Central Lat	Both	50-69 yea	Sexually tr	Rate	2010	39276.8	44351.8	34678.3
Prevalence	Central Lat	Both	50-69 yea	Sexually tr	Number	2011	1.2E+07	1.4E+07	1.1E+07
Prevalence	Central Lat	Both	50-69 yea	Sexually tr	Rate	2011	39364.8	44391.9	34725.8
Prevalence	Central Lat	Both	50-69 yea	Sexually tr	Number	2012	1.3E+07	1.4E+07	1.1E+07
Prevalence	Central Lat	Both	50-69 yea	Sexually tr	Rate	2012	39520.2	44574	34813.5
Prevalence	Central Lat	Both	50-69 yea	Sexually tr	Number	2013	1.3E+07	1.5E+07	1.1E+07
Prevalence	Central Lat	Both	50-69 yea	Sexually tr	Rate	2013	39693.3	44753.3	34956.6
Prevalence	Central Lat	Both	50-69 yea	Sexually tr	Number	2014	1.4E+07	1.5E+07	1.2E+07
Prevalence	Central Lat	Both	50-69 yea	Sexually tr	Rate	2014	39831.8	45020.2	35048.9
Prevalence	Central Lat	Both	50-69 yea	Sexually tr	Number	2015	1.4E+07	1.6E+07	1.2E+07
Prevalence	Central Lat	Both	50-69 yea	Sexually tr	Rate	2015	39898.2	45116.7	35104
Prevalence	Central Lat	Both	50-69 yea	Sexually tr	Number	2016	1.5E+07	1.6E+07	1.3E+07
Prevalence	Central Lat	Both	50-69 yea	Sexually tr	Rate	2016	39896.8	45036	35110.7
Prevalence	Central Lat	Both	50-69 yea	Sexually tr	Number	2017	1.5E+07	1.7E+07	1.3E+07
Prevalence	Central Lat	Both	50-69 yea	Sexually tr	Rate	2017	39875.5	45091.2	35140.1
Prevalence	Central Lat	Both	50-69 yea	Sexually tr	Number	2018	1.5E+07	1.7E+07	1.4E+07
Prevalence	Central Lat	Both	50-69 yea	Sexually tr	Rate	2018	39810.9	45006.2	35098
Prevalence	Central Lat	Both	50-69 yea	Sexually tr	Number	2019	1.6E+07	1.8E+07	1.4E+07
Prevalence	Central Lat	Both	50-69 yea	Sexually tr	Rate	2019	39697.9	44922.9	34977.4
Prevalence	Andean La	Both	50-69 yea	Sexually tr	Number	1990	1450486	1611386	1299154
Prevalence	Andean La	Both	50-69 yea	Sexually tr	Rate	1990	41571.1	46182.5	37233.9
Prevalence	Andean La	Both	50-69 yea	Sexually tr	Number	1991	1518391	1685235	1363389
Prevalence	Andean La	Both	50-69 yea	Sexually tr	Rate	1991	42043.2	46663	37751.3
Prevalence	Andean La	Both	50-69 yea	Sexually tr	Number	1992	1580409	1753642	1422796
Prevalence	Andean La	Both	50-69 yea	Sexually tr	Rate	1992	42481.6	47138.1	38244.9
Prevalence	Andean La	Both	50-69 yea	Sexually tr	Number	1993	1643220	1821153	1484560
Prevalence	Andean La	Both	50-69 yea	Sexually tr	Rate	1993	42814.3	47450.4	38680.4
Prevalence	Andean La	Both	50-69 yea	Sexually tr	Number	1994	1703850	1886472	1536747
Prevalence	Andean La	Both	50-69 yea	Sexually tr	Rate	1994	43040.4	47653.6	38819.3
Prevalence	Andean La	Both	50-69 yea	Sexually tr	Number	1995	1762557	1949537	1587521
Prevalence	Andean La	Both	50-69 yea	Sexually tr	Rate	1995	43120.6	47695.1	38838.4
Prevalence	Andean La	Both	50-69 yea	Sexually tr	Number	1996	1819494	2012874	1643210
Prevalence	Andean La	Both	50-69 yea	Sexually tr	Rate	1996	43125	47708.4	38946.8
Prevalence	Andean La	Both	50-69 yea	Sexually tr	Number	1997	1883397	2084775	1701865
Prevalence	Andean La	Both	50-69 yea	Sexually tr	Rate	1997	43151.1	47765	38992
Prevalence	Andean La	Both	50-69 yea	Sexually tr	Number	1998	1947722	2152638	1760216
Prevalence	Andean La	Both	50-69 yea	Sexually tr	Rate	1998	43147.8	47687.3	38994
Prevalence	Andean La	Both	50-69 yea	Sexually tr	Number	1999	2015356	2225730	1822840
Prevalence	Andean La	Both	50-69 yea	Sexually tr	Rate	1999	43160.6	47666	39037.7
Prevalence	Andean La	Both	50-69 yea	Sexually tr	Number	2000	2080946	2297692	1880651
Prevalence	Andean La	Both	50-69 yea	Sexually tr	Rate	2000	43159.2	47654.5	39005
Prevalence	Andean La	Both	50-69 yea	Sexually tr	Number	2001	2154714	2380713	1947331
Prevalence	Andean La	Both	50-69 yea	Sexually tr	Rate	2001	43261.2	47798.7	39097.4
Prevalence	Andean La	Both	50-69 yea	Sexually tr	Number	2002	2241703	2475967	2024413
Prevalence	Andean La	Both	50-69 yea	Sexually tr	Rate	2002	43494.4	48039.7	39278.4
Prevalence	Andean La	Both	50-69 yea	Sexually tr	Number	2003	2333088	2579496	2105351
Prevalence	Andean La	Both	50-69 yea	Sexually tr	Rate	2003	43775.5	48398.8	39502.5
Prevalence	Andean La	Both	50-69 yea	Sexually tr	Number	2004	2425268	2688412	2183740
Prevalence	Andean La	Both	50-69 yea	Sexually tr	Rate	2004	44008.6	48783.6	39625.9
Prevalence	Andean La	Both	50-69 yea	Sexually tr	Number	2005	2516254	2793736	2258463
Prevalence	Andean La	Both	50-69 yea	Sexually tr	Rate	2005	44101.1	48964.3	39582.9
Prevalence	Andean La	Both	50-69 yea	Sexually tr	Number	2006	2593890	2885202	2325438
Prevalence	Andean La	Both	50-69 yea	Sexually tr	Rate	2006	43943.8	48879	39395.9
Prevalence	Andean La	Both	50-69 yea	Sexually tr	Number	2007	2664062	2973945	2380857
Prevalence	Andean La	Both	50-69 yea	Sexually tr	Rate	2007	43566.5	48634.1	38935.1
Prevalence	Andean La	Both	50-69 yea	Sexually tr	Number	2008	2728692	3067338	2417326
Prevalence	Andean La	Both	50-69 yea	Sexually tr	Rate	2008	43108.9	48458.9	38189.8
Prevalence	Andean La	Both	50-69 yea	Sexually tr	Number	2009	2798979	3157323	2467488
Prevalence	Andean La	Both	50-69 yea	Sexually tr	Rate	2009	42725.8	48195.9	37665.7
Prevalence	Andean La	Both	50-69 yea	Sexually tr	Number	2010	2887544	3266845	2527210
Prevalence	Andean La	Both	50-69 yea	Sexually tr	Rate	2010	42574.2	48166.6	37261.4
Prevalence	Andean La	Both	50-69 yea	Sexually tr	Number	2011	2990924	3373859	2626027
Prevalence	Andean La	Both	50-69 yea	Sexually tr	Rate	2011	42585	48037.3	37389.6
Prevalence	Andean La	Both	50-69 yea	Sexually tr	Number	2012	3106093	3500103	2726208
Prevalence	Andean La	Both	50-69 yea	Sexually tr	Rate	2012	42611.2	48016.5	37399.8
Prevalence	Andean La	Both	50-69 yea	Sexually tr	Number	2013	3228966	3640207	2836110
Prevalence	Andean La	Both	50-69 yea	Sexually tr	Rate	2013	42637.9	48068.3	37450.3
Prevalence	Andean La	Both	50-69 yea	Sexually tr	Number	2014	3353918	3781324	2945189
[truncated: 9,626,158 more chars]
